# Mapping temperature‐sensitive mutations at a genome scale to engineer growth switches in *Escherichia coli*

**DOI:** 10.15252/msb.202311596

**Published:** 2023-08-29

**Authors:** Thorben Schramm, Paul Lubrano, Vanessa Pahl, Amelie Stadelmann, Andreas Verhülsdonk, Hannes Link

**Affiliations:** ^1^ Interfaculty Institute of Microbiology and Infection Medicine University of Tübingen Tübingen Germany; ^2^ Cluster of Excellence “Controlling Microbes to Fight Infections” University of Tübingen Tübingen Germany; ^3^ Present address: Department of Biology, Institute of Molecular Systems Biology ETH Zurich Zürich Switzerland

**Keywords:** CRISPR‐Cas9 genome editing, growth‐switches, metabolic valves, metabolomics, temperature‐sensitive mutations, Biotechnology & Synthetic Biology, Metabolism, Microbiology, Virology & Host Pathogen Interaction

## Abstract

Temperature‐sensitive (TS) mutants are a unique tool to perturb and engineer cellular systems. Here, we constructed a CRISPR library with 15,120 *Escherichia coli* mutants, each with a single amino acid change in one of 346 essential proteins. 1,269 of these mutants showed temperature‐sensitive growth in a time‐resolved competition assay. We reconstructed 94 TS mutants and measured their metabolism under growth arrest at 42°C using metabolomics. Metabolome changes were strong and mutant‐specific, showing that metabolism of nongrowing *E. coli* is perturbation‐dependent. For example, 24 TS mutants of metabolic enzymes overproduced the direct substrate metabolite due to a bottleneck in their associated pathway. A strain with TS homoserine kinase (ThrB^F267D^) produced homoserine for 24 h, and production was tunable by temperature. Finally, we used a TS subunit of DNA polymerase III (DnaX^L289Q^) to decouple growth from arginine overproduction in engineered *E. coli*. These results provide a strategy to identify TS mutants *en masse* and demonstrate their large potential to produce bacterial metabolites with nongrowing cells.

## Graphical abstract

A CRISPR library with 15,120 *E. coli* mutants is constructed, 1,269 of which are temperature sensitive (TS). Follow‐up analyses reveal specific metabolic perturbations in TS mutants and show that TS DNA polymerase decouples growth from arginine overproduction.

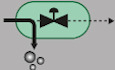

## Introduction

Targeted perturbations of cellular networks are key to understand and engineer their function. Gene deletions, for instance, can improve microbial production strains (Burgard *et al*, 2003), and knockout mutant libraries enabled systematic analyses of gene‐gene networks (Tong *et al*, 2004), gene‐metabolite networks (Mülleder *et al*, 2016; Fuhrer *et al*, 2017), and gene regulatory networks (Kemmeren *et al*, 2014). However, gene deletions are static and irreversible perturbations, and they are not feasible if the gene of interest is essential for cell growth. RNA interference (Na *et al*, 2013) or CRISPR interference (Qi *et al*, 2013) allow inducible knockdowns of essential genes, and these methods were used to construct synthetic regulatory circuits (Qi *et al*, 2013; Santos‐Moreno *et al*, 2020) and dynamic growth switches (Li *et al*, 2016).

Temperature‐sensitive (TS) mutations are an alternative method to perturb essential genes. At low (permissive) temperatures, genes with a TS mutation encode a functional product, while at higher (nonpermissive) temperatures, the gene product is not functional. Several molecular mechanisms can lead to thermal sensitivity, the most important of which induce changes in protein stability or changes in protein folding. Thermolabile mutant proteins, for instance, unfold at higher temperatures, which reduces their activity or inactivates the protein completely. In contrast, proteins with temperature‐sensitive folding are not correctly folded at higher temperatures (Sadler & Novick, 1965; Haase‐Pettingell & King, 1997). Other TS mechanisms alter interactions of the TS protein with other molecules, such as a TS mutant of the Drosophila muscle regulator Mef2, which changes DNA binding upon temperature changes (Lovato *et al*, 2009).

TS mutations provide several unique advantages compared with other perturbation methods like RNA or CRISPR interference. First, a single mutation is often sufficient for a TS phenotype and, thus, auxiliary components like dCas9 (Qi *et al*, 2013) or small regulatory RNAs (Na *et al*, 2013) are not required. Second, many TS mutations enable fast perturbations, especially if the mutant protein is thermolabile and unfolds within seconds or minutes (Plaza del Pino *et al*, 2000). Third, TS mutations have almost no polar effects since mutations are small and will mainly affect a single gene product. Finally, temperature shifts are extremely versatile, well‐controllable, and reversible. Focused ultrasound, for instance, can control TS proteins *in vivo* (Piraner *et al*, 2017), and almost all bioreactors are equipped with temperature control.

TS mutants were used to engineer diverse cellular systems with applications in medical and industrial biotechnology (Weber, 2003; Cho *et al*, 2012; Lynch *et al*, 2016, 2019; Piraner *et al*, 2017; Harder *et al*, 2018; Schramm *et al*, 2020; Wang *et al*, 2021; Kasari *et al*, 2022). Thermal control of growth is especially important to realize two‐stage bioprocesses, which separate a growth phase from a production phase with nongrowing cells (Burg *et al*, 2016). Unlike processes with growing cells that require regulation of growth‐related parameters, two‐stage bioprocesses eliminate the need for controlling biomass levels via the supply of growth‐limiting nutrients. This allows for continuous processes without the additional complexity of growth control. However, most applications are based on known temperature‐sensitive mutations, such as the transcriptional repressor CI857 from *Escherichia virus Lambda* (Harder *et al*, 2018; Wang *et al*, 2021; Kasari *et al*, 2022). The search for new TS mutants was mainly driven by large‐scale genetic screens in yeast (Costanzo *et al*, 2016). These efforts required a comprehensive collection of TS mutants of *Saccharomyces cerevisiae*, which, for the most part, were constructed with random mutagenesis approaches (Ben‐Aroya *et al*, 2008; Li *et al*, 2011; Kofoed *et al*, 2015).

Currently, there is no comprehensive collection of TS *Escherichia coli* strains. The *E*. *coli* Genetic Stock Center Database (Berlyn, 1999) lists 178 TS mutations in 116 protein‐coding genes. We found an additional 41 temperature‐sensitive *E. coli* strains in the literature that cover another 15 genes (Dataset EV1). To our knowledge, 87 TS *E. coli* strains with mutations in 32 genes were sequenced. All the 87 strains had at least one amino acid substitution, and 29 had more than one. These temperature‐sensitive *E. coli* strains were relevant to study the function of individual genes and contributed to important findings like how DNA is replicated (Blinkova *et al*, 1993; Saluja & Godson, 1995; Vandewiele *et al*, 2002; Georgescu *et al*, 2008; Hansen & Atlung, 2018).

Here, we used a high‐throughput approach to construct and identify temperature‐sensitive mutants. We used a CRISPR method to construct 15,120 *E. coli* strains, each with a single amino acid change in one of 346 essential proteins, and we measured their growth at two temperatures. Based on these results, we constructed a panel of 94 TS *E. coli* strains with single mutations and analyzed their growth and metabolism by metabolomics. Many TS mutants of enzymes accumulated the direct substrate metabolite. For example, TS variants of homoserine O‐succinyltransferase (MetA^F285W^) and homoserine kinase (ThrB^F267D^) overproduced homoserine, and the production was tunable by temperature. Finally, we used a TS subunit of DNA polymerase (DnaX^L289Q^) to control the growth of an arginine overproducing *E. coli*.

## Results

### 15,120 *E. coli* mutants with single amino acid changes in 346 essential proteins

We used a modified version of a CRISPR‐Cas9 method (Garst *et al*, 2017) to create a library of 15,120 *E. coli* strains, each with a different amino acid change in an essential gene. As a starting point, we selected 352 proteins that are essential for the growth of *E. coli* on minimal glucose medium (Patrick *et al*, 2007; Goodall *et al*, 2018). We then designed single amino acid changes that may cause temperature sensitivity of the respective protein using the TSpred algorithm (Varadarajan *et al*, 1996; Tan *et al*, 2014) (Dataset EV1). To reduce the design space, we included only amino acid changes to alanine, aspartate, glutamine, proline, and tryptophan (Varadarajan *et al*, 1996; Tan *et al*, 2014). Per protein, we designed up to 50 amino acid changes (10 sites each with 5 substitutions) considering the following design rules: (i) a minimal distance between the protospacer adjacent motif (PAM) site and the mutation site to maximize editing efficiency, (ii) a maximal number of possible amino acid changes at a given site, and (iii) minimal CRISPR off‐targets of the single guide RNA (sgRNA).

For 154 genes, we found less than 50 amino acid changes either because the number of predicted sites was limited or due to constraints by the design rules. For example, TSpred predicted no temperature‐sensitive mutations in *rpmA* and *rpmH*, which encode small ribosomal proteins with 85 and 45 amino acids, respectively. Another set of genes (*leuL*, *rplU*, *rpmC*, and *rpsI*) had no substitutions that fulfilled our design rules. In total, we designed 16,038 single amino acid changes and inserted the respective mutations into 346 essential genes from various functional categories (Dataset EV2).

**Figure 1:**
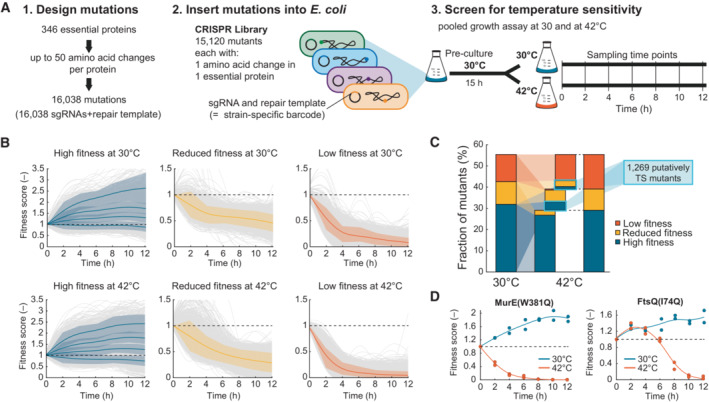
CRISPR screen with 15,120 *E. coli* mutants identifies temperature‐sensitive mutations Schematic of the CRISPR screen. 16,038 sgRNAs plus repair templates (barcodes) were designed to introduce amino acid changes in 346 essential proteins (step 1). 15,120 of the barcodes were present in the final CRISPR library (step 2). The CRISPR library was cultured at 30 and at 42°C (*n* = 2 replicates). Strain‐specific barcodes (sgRNA and repair template) were sequenced every 2 h to determine the composition of the library (step 3).K‐means clustering of fitness scores of 8,884 strains in the CRISPR library. Time‐course data were clustered into k = 6 clusters per temperature. The fitness scores were calculated by normalizing the read counts of the barcode of each mutant to the total number of reads per sample and to the first time point. Gray curves are the moving average of the mean of two replicates. Colored lines are cluster means and shaded areas their standard deviation (blue: 4 clusters with high fitness, yellow: 1 cluster with reduced fitness, red: 1 cluster with low fitness). Dashed lines indicate a fitness score of 1.Relative composition of the CRISPR library at 30 and 42°C. Blue indicates high fitness, yellow a reduced fitness, and red low fitness. The bar graph in the middle connects the 30 and 42°C data. The light blue box indicates putative TS mutants.Examples of fitness score dynamics of two strains that show temperature sensitivity (MurE^W381Q^ and FtsQ^I74Q^). Dots show data from two replicates per temperature. The lines are the moving average through the means. Blue: 30°C culture. Red: 42°C culture. Dashed lines indicate a fitness score of 1. Source data are available online for this figure.

The CRISPR‐based genome editing method uses homologous recombination by *Escherichia virus Lambda* Red (Garst *et al*, 2017). Templates for homologous recombination were 85‐bp‐long DNA sequences that had the desired mutation to introduce single amino acid changes and an additional silent mutation at the protospacer adjacent site. The homologous DNA sequence was encoded on a plasmid next to the respective sgRNA and functioned as a strain‐specific barcode. We constructed the plasmids in a pooled approach using 200 bp array‐synthesized oligonucleotides and measured the library composition directly after construction by deep sequencing (Fig 1A and Appendix Fig S1). Out of all 16,038 designed amino acid substitutions, 15,582 (97%) were present in the plasmid library.

Next, we used the plasmid library for the transformation of an *E. coli* strain, which carried a second plasmid with Cas9 and the *Lambda* Red system (Appendix Fig S2). In these strains, we induced Cas9 expression and *Lambda* Red‐mediated recombination to obtain the final CRISPR library. This library contained 15,120 of all designed 16,038 single amino acid substitutions (94%) and targeted all 346 genes that we included in the initial library design.

### Time‐resolved competition assays identify putative TS mutants

After constructing a CRISPR library with 15,120 mutants, we sought to identify mutants that are temperature‐sensitive. For this purpose, we used a time‐resolved competition assay, in which we cultivated the pooled CRISPR library at 30 and 42°C (Fig 1A). First, we grew the CRISPR library for 15 h on minimal glucose medium at 30°C and expected that strains with a strong growth defect would disappear from the library during this preculture phase. After 15 h, the preculture was then used to inoculate two main cultures: a 30°C culture and a 42°C culture. These two main cultures were incubated for 12 h. Every 3 h, the cultures were back‐diluted into fresh medium to avoid limitations of oxygen and nutrients. Every 2 h, we determined the composition of the library by deep sequencing of the strain‐specific barcodes, which was reproducible between two independent experiments (Appendix Fig S3). Fitness scores of single mutants were determined by normalizing the read counts of their barcodes to the total number of reads and the first time point.

Out of all 15,120 strains, 6,236 dropped out from the library after the 15‐h preculture phase (strains with average reads < 15). For the remaining 8,884 strains, we explored dynamic patterns in the main cultures with k‐means clustering (Fig 1B). This analysis revealed that 5,118 strains had a high fitness at 30°C, 1,712 strains had mild fitness defects at 30°C, and 2,054 strains had strong fitness defects at 30°C. Most strains were not affected by temperature. This means that 96% of the strains with a fitness defect at 30°C also had a fitness defect at 42°C (Fig 1C). Similarly, 84% of the mutants with a high fitness at 30°C also had a high fitness at 42°C. However, 1,269 mutants (8.4% of the library) had a higher fitness at 30°C than at 42°C, thus indicating that these strains are TS mutants (blue boxes in Fig 1C).

**Figure EV1:**
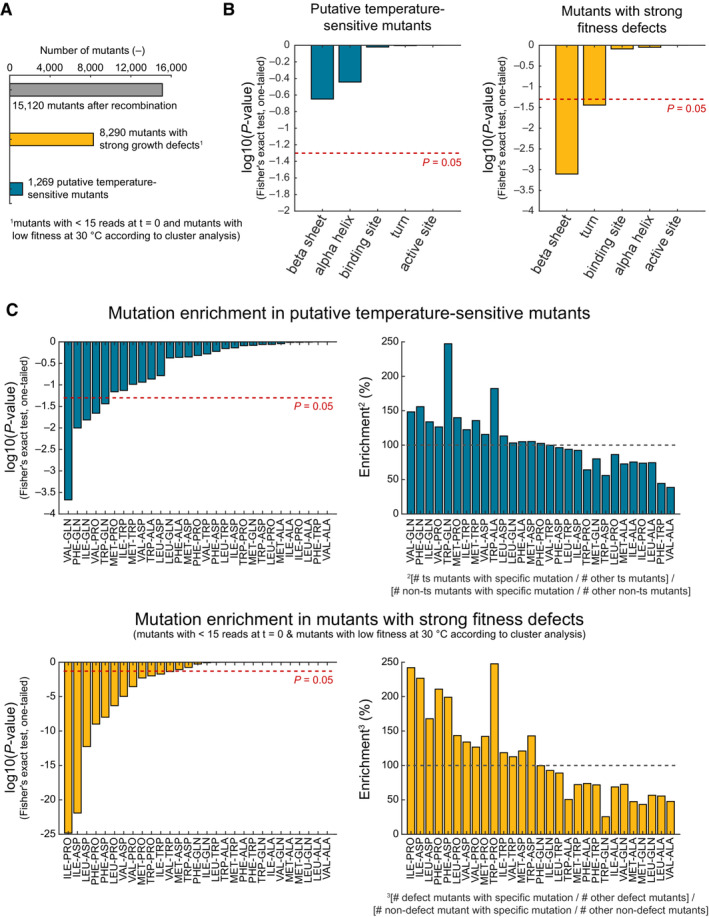
Mutation enrichment in TS alleles and mutants with strong fitness defects The bar plot shows the total number of mutants in our CRISPR library after recombination (gray), the number of putative temperature‐sensitive (TS) mutants (blue), and the number of mutants with strong fitness defects (yellow), which are strains with less than 15 reads at the start of the pooled fitness assay (*t* = 0 h) and strains that have a low fitness at 30°C based on cluster analysis.The bar plots show the *P*‐values for testing an enrichment of mutations in alpha helices, beta sheets, turns, binding, and active sites among the putative TS mutants (blue bars) and strong fitness defect mutants (yellow bars). We used a one‐tailed Fisher's exact test (also see Dataset EV3) and considered conditions with *P*‐values < 0.05 as enriched.The left bar plots show *P*‐values of the indicated mutations that were tested for an enrichment in the putative TS mutants (upper chart) and fitness defect mutants (lower chart) by a one‐tailed Fisher's exact test. We considered conditions with *P*‐values < 0.05 as enriched. The right bar plots show the %‐enrichment between the putative TS and all other mutants (upper chart) and the growth defect mutants and all other mutants (lower chart).

In summary, we constructed a CRISPR library with 15,120 strains and identified two groups of mutations (Fig EV1A): 1,269 putative TS mutations and 8,290 “low‐fitness” mutations (6,236 dropout mutants and 2,054 mutants with low fitness at 30°C). Next, we inspected whether these two groups of mutations are linked to structural properties of the protein or to specific amino acid changes.

### Distinct amino acid changes are enriched in TS mutants and low‐fitness mutants

Having identified 8,290 mutations that strongly reduce fitness (low‐fitness mutations) as well as 1,269 putative TS mutations (Fig EV1A), we tested whether the mutations preferentially occur in alpha helices, beta sheets, active and binding sites, or turns (Fig EV1B). Furthermore, we tested whether certain amino acid changes are enriched in the two groups (Fig EV1C). Low‐fitness mutants showed an enrichment for mutations in beta sheets (*P* = 7.8e−04, Fisher's exact test, one‐tailed, Fig EV1B and Dataset EV3) and turns (*P* = 0.036). Moreover, low‐fitness mutations were enriched in mutations that changed hydrophobic amino acids (Ile, Leu, Phe, Val, Met, and Trp) into proline or aspartate (Fig EV1C).

The putative TS mutations were not enriched in structure‐related features (Fig EV1B). However, putative TS mutations showed an enrichment of five mutations: Val‐Gln, Phe‐Gln, Iso‐Gln, Val‐Pro, and Trp‐Gln (*P*‐values < 0.05, Fisher's exact test, one‐tailed, Fig EV1C). Thus, although we had a limited search space of maximal 50 mutations per gene, each predicted with TSpred, we could identify simple design rules that may help to improve predictions of TS mutations.

### An allelic series of cysteine‐tRNA ligase (CysS) confirms function of TS mutations

To classify the strength of TS mutations, we clustered the fitness dynamics of the 1,269 TS mutants into four groups using k‐means clustering (Appendix Fig S4). A group of 64 TS mutants had strong TS phenotypes, because these strains grew well at 30°C and disappeared fast from the library at 42°C (first cluster in Appendix Fig S4). The MurE^W381Q^ mutant is an example of a strain with such a strong TS phenotype (Fig 1D). Another group of 284 mutants disappeared with a time delay from the 42°C cultures, and the FtsQ^I74Q^ strain is an example for the delayed TS phenotypes (Fig 1D). The other two clusters captured 459 strains with mild TS phenotypes and 462 strains with weak TS phenotypes. These strains showed a growth defect at 30°C and a more severe growth defect at 42°C. Thus, the growth phenotypes of the 1,269 putative TS mutants exhibit distinct characteristics, and it seems that they are primarily determined by the specific nature of each mutation rather than the overall gene function (Dataset EV11).

**Figure EV2:**
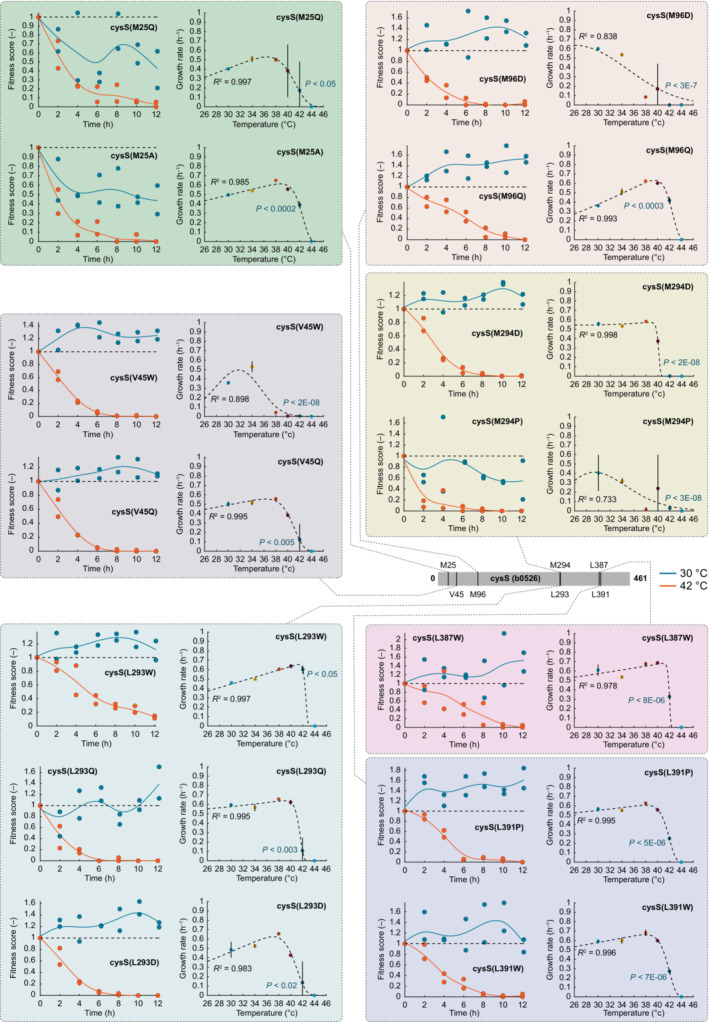
Fitness score dynamics and growth kinetics of an allelic series of cysS TS mutants We reconstructed a panel of 14 *cysS* alleles from the CRISPR library that were putative TS mutants. Each box shows a single mutation site in *cysS*, and each site had more than one allele available, except for the site *cysS* ^L387^. In each box, the left charts show the fitness score dynamics of the indicated mutant from the pooled fitness assay (Fig 1A). The dots in the left figures show data from two replicates per temperature, and the lines are the moving average through the means (blue: 30°C culture, red: 42°C culture). Dashed lines indicate a fitness score of 1. The right figures in each box show the maximum specific growth rates during plate reader growth at six different temperatures (30, 34, 38, 40, 42, and 44°C). The dots in the right figures are the mean of three replicates, and the vertical lines are the standard deviation. The dashed lines were calculated by fitting an Arrhenius‐type function to the data (*R* ^2^ is the coefficient of determination, also see Appendix Fig S9). Indicated *P*‐values were calculated with two‐sample *t*‐tests (two‐tailed) comparing each the mutant strain against the unedited control strain at 42°C (Dataset EV4).

The 1,269 putative TS alleles occurred in 267 of the 346 genes. On average, the CRISPR screen identified 3.7 TS alleles per gene, and five genes had more than 16 TS alleles: *panM* (30), *panB* (25), *panC* (17), *cysN* (17), and *cysS* (17) (Appendix Fig S5). To validate some of the putative TS mutations, we focused on *cysS*, which encodes cysteine‐tRNA ligase. The CRISPR screen identified 17 TS alleles for *cysS*, and we reconstructed 14 of these *cysS* mutants (Fig EV2). All the 14 *cysS* mutants that we tested showed significant TS phenotypes when we cultured them one‐by‐one in 96‐well plates at six different temperatures (two‐sample *t*‐test, two‐tailed, α = 0.05, *n* = 3, mutant vs. control at 42°C, Fig EV2 and Dataset EV4). These results provided first evidence that the pooled CRISPR screen identified authentic TS mutants.

### A panel of 94 TS mutants shows distinct growth–temperature relationships

**Figure 2:**
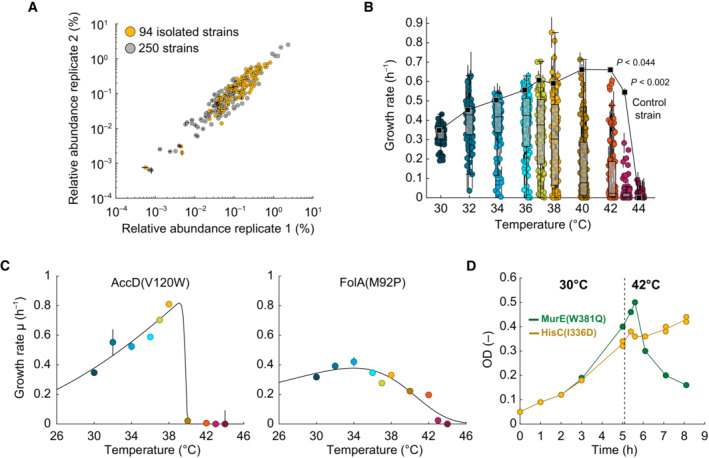
Reconstruction of 94 TS mutants and characterization of growth–temperature relationships Relative abundance of strains in the pooled sublibrary of 250 putative TS mutants. The strain library was constructed twice (replicate 1 and 2), and dots are the mean of two technical sequencing replicates. The black lines indicate the difference between the two technical replicates. The read counts of single strains were normalized to the total number of reads. Yellow dots indicate 94 TS mutants that were isolated from the pooled sublibrary.Maximal specific growth rates of 94 TS mutants and a control strain at 10 different temperatures between 30 and 44°C. Dots are the mean (*n* = 3 biological replicates for each temperature and each of the 95 strains). Vertical lines indicate standard deviations. Black squares and the line indicate the control strain. The box‐whisker plots show the median and 25^th^/75^th^ percentiles. Two‐sample *t*‐tests (two‐tailed) were performed for each mutant against the control strain (also see Dataset EV7). At 42 and 43°C, all *P*‐values were below the respective indicated values.Examples of maximal specific growth rates of the TS mutants AccD^V120W^ and FolA^M92P^ at different temperatures (Fig EV2 shows all 94 TS mutants). Dots are the mean, and vertical black lines indicate the standard deviation (*n* = 3). The black line was calculated by fitting an Arrhenius‐type function to the data (also see Appendix Fig S9).Growth dynamics of the MurE^W381Q^ strain (green) and the HisC^I336D^ strain (yellow) during a temperature shift from 30 to 42°C. Dots show data from two replicates, and the lines connect the mean. Source data are available online for this figure.

To reconstruct the best TS mutants, we scored temperature sensitivity of all strains in our library by a set of criteria (Appendix Fig S6). With this approach, we obtained high‐scoring TS mutations for 250 essential genes in the CRISPR library (Dataset EV5) and constructed a sublibrary with these 250 strains using the same CRISPR‐Cas9 method (Garst *et al*, 2017). Sequencing of the sublibrary showed that all 250 strains were present in the library, but their relative abundance varied markedly between 0.0007% and 2.4% (Fig 2A). If these differences in the abundance of single strains were caused by experimental variation, we expected that cloning the sublibrary a second time would reduce the variation. However, the relative abundance of the 250 strains remained remarkably constant between the two cloning rounds, thus indicating that mutant‐specific parameters (e.g., editing efficiency) influenced the abundance of single strains in the library and not experimental variation during cloning.

**Figure EV3:**
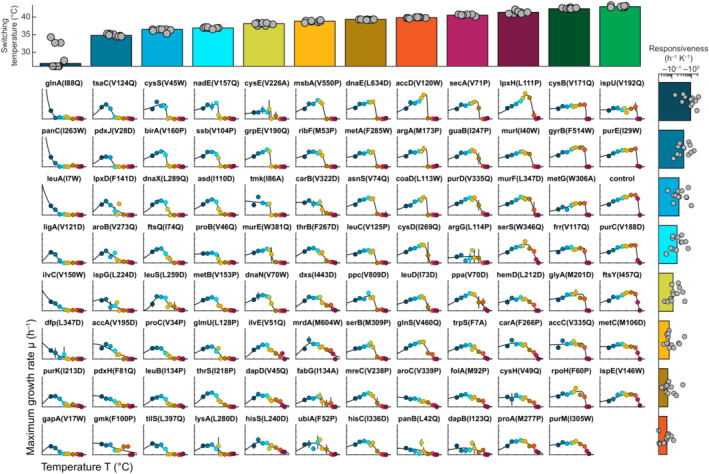
Maximum specific growth rates of 94 TS mutants at different temperatures The charts show the maximum specific growth rates μ (h^−1^) of 94 TS mutants (and a control strain) at 10 different temperatures ranging from 30 to 44°C. The growth rates were determined from growth curves in 96‐well microtiter plate cultivations (Dataset EV7). Dots show the mean from three replicates, and black vertical lines show the standard deviation. An empirical Arrhenius‐type function was fitted to the data (black lines, also see Appendix Fig S9). The strains were sorted according to their responsiveness and switching temperature, which are parameters based on the Arrhenius‐type functions. The upper dot plot shows the switching temperatures (°C) of the strains in the columns below. The dot plot on the right side shows the responsiveness (h^−1^ K^−1^) of the strains in the rows. The bars indicate the medians of the responsiveness values/switching temperatures.

We then randomly isolated 2016 clones from the sublibrary and tested them for temperature sensitivity in 96‐well plates. In total, 456 clones (22%) showed temperature‐sensitive growth and sequencing of the single clones identified 123 unique strains (Dataset EV6). Out of the 123 strains, we selected 94 strains for further analysis. Notably, the group of 94 strains was biased toward high abundant strains in the pool of 250 strains (*P* = 1.2e‐06, Wilcoxon rank‐sum test, two‐tailed, Appendix Fig S7A), which could explain why we picked them over others. The selected 94 TS mutants covered genes from all functional categories, except ribosomal subunits (Appendix Fig S7B). To characterize growth–temperature relationships of the 94 TS mutants, we cultured them together with a nonedited control strain at 10 different temperatures between 30 and 44°C (Figs 2B and EV3). All 94 strains showed a significant TS phenotype based on a *t*‐test against the nonedited control strain at 42°C (two‐sample *t*‐test, two‐tailed, *n* = 3, α = 0.05, Dataset EV7). We also used two mock CRISPR‐edited controls to confirm that the editing itself has no effect on growth. In these mock edit controls, we reintroduced a wild‐type sequence of *thrA* and *metA* together with a silent PAM mutation. Both the *thrA*‐control and the *metA*‐control grew like the nonedited control strain (Appendix Fig S8 and Dataset EV4).

At 30°C, most TS mutants had similar specific growth rates as the control strain (0.35 h^−1^), whereas specific growth rates varied markedly at higher temperatures. At 38°C, for example, specific growth rates of the 94 mutants varied between no growth and 0.85 h^−1^, which is close to the maximal specific growth rate that is possible for *E. coli* on minimal glucose medium (Monk *et al*, 2016). The nonedited control strain had a higher specific growth rate at 42°C (0.66 h^−1^) than at 37°C (0.61 h^−1^), which is consistent with previous studies (Schmidt *et al*, 2016). At 43°C, the specific growth rate of the control strain decreased slightly to 0.54 h^−1^, whereas all TS mutants showed a strong growth defect at this temperature.

The growth–temperature relationships of all TS mutants and the control strain followed an empirical Arrhenius‐type function with an activating term and an inactivating term (Appendix Fig S9, median *R* ^2^ value = 0.96). These two terms may reflect that higher temperatures activate overall metabolism, but, at the same time, they inactivate the TS protein. Although all growth–temperature relationships qualitatively matched the Arrhenius‐type function, they differed quantitatively across the 94 TS mutants, resulting in distinct parameters (Fig EV3).

K‐means clustering of the temperature–growth relationship identified 52 TS mutants with a switch‐like response and 42 TS mutants with a gradual response (Appendix Fig S10). The AccD^V120W^ mutant is an example for a switch‐like response (Fig 2C), because a small change in temperature (38–40°C) had a large effect on growth rates (0.81–0.02 h^−1^). In contrast, the FolA^M29P^ mutant is an example for a gradual temperature–growth relationship (Fig 2C).

Next, we wondered how fast a TS mutant switches from growth to no growth upon temperature increases. Therefore, we selected two TS mutants from our panel of 94 strains that showed a fast response in the pooled competition assay: the MurE^W381Q^ mutant (Fig 1D) and the HisC^I336D^ mutant (Appendix Fig S11). At 30°C, the two strains grew normally, and, after 5 h, we transferred them to 42°C (Fig 2D). After this temperature shift, the MurE^W381Q^ mutant was able to grow for another 30 min, but then the optical density of the culture decreased, indicating cell lysis. Perturbation of the MurE reaction may have caused a limitation in peptidoglycan biosynthesis, and it is thus possible that cell lysis of MurE^W381Q^ at 42°C resembles the bactericidal effect of antibiotics that target peptidoglycan metabolism (Williams & Bardsley, 1999; Bush & Bradford, 2016). The HisC^I336D^ strain also showed a reduction of growth after 30 min at 42°C, but did not lyse like the MurE^W381Q^ strain. Since the histidinol‐phosphate aminotransferase (HisC) catalyzes the seventh step in histidine synthesis (Grisolia *et al*, 1985), the growth arrest of the HisC^I336D^ mutant at 42°C was probably due to a histidine auxotrophy.

To test which TS mutants are conditionally auxotrophic, we cultured the TS mutants in rich medium (LB). Forty‐nine out of the 94 TS mutants indeed grew like the control strain at 42°C in LB medium (two‐sample *t*‐test, two‐tailed, *n* = 3, α = 0.05, Appendix Fig S12 and Dataset EV8). This is in sharp contrast to the significant growth defect of these 49 strains in minimal medium at 42°C (Fig 2B and Dataset EV7). These data indicate that the 49 TS mutants are conditionally auxotrophic and that supplementation of the missing nutrient restores growth. For example, 28 out of 31 TS mutants of genes in amino acid biosynthesis pathways lost their TS phenotype in rich LB medium, because *de novo* amino acid synthesis is not active in the presence of external amino acids.

In conclusion, we constructed a panel of 94 TS mutants that had growth defects at higher temperatures but grew similar to an unedited control strain at 30°C. Although the growth–temperature relationships differed across the 94 mutants, they all followed an empirical Arrhenius‐type function. Dynamic temperature shifts demonstrated that growth of the TS mutants MurE^W381Q^ and HisC^I336D^ responded within 30 min after a shift from 30 to 42°C, either with cell lysis (MurE^W381Q^) or with a growth arrest (HisC^I336D^). Thus, different TS mutants have distinct growth phenotypes, and we wondered whether these differences occur also at the level of metabolism.

### TS mutants show distinct metabolomes and increases in substrate metabolites

**Figure 3:**
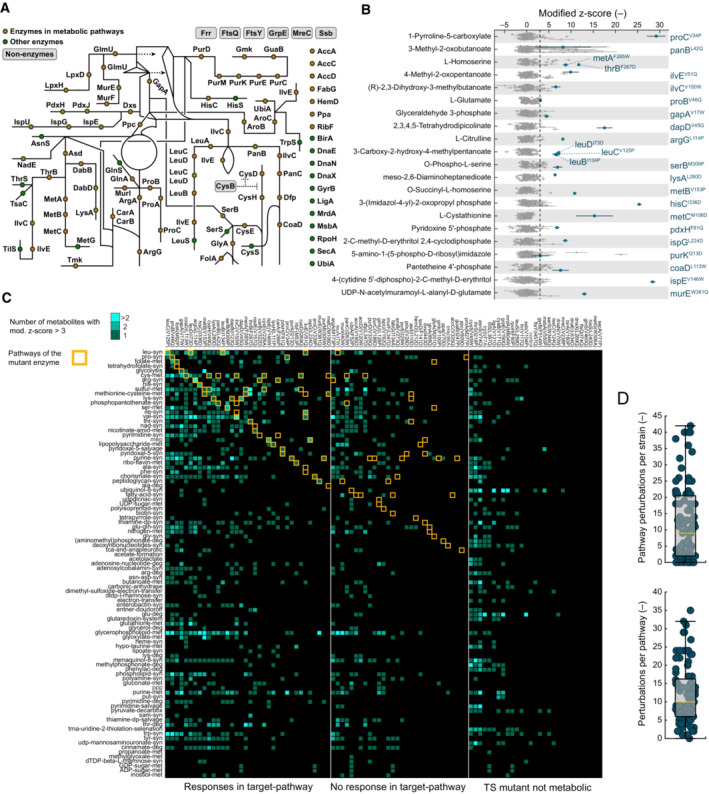
Metabolome responses of 94 TS mutants are strong, mutant‐specific, and metabolism‐wide Location of TS mutants in the metabolic network. Sixty‐six TS mutations affect metabolic enzymes (yellow dots). Twenty‐two TS mutations affect nonmetabolic enzymes (green dots). Seven TS mutations affect proteins without enzymatic function (gray boxes).Subset of the metabolome data. Shown are substrate metabolites that increase in 24 TS mutants (blue dots). Each strip of the dot plot shows one metabolite in all 94 TS mutants (mod. z‐score normalized). Dots are means (*n* = 3). Lines indicate standard deviations (calculated with error propagation). Each metabolite is the substrate for at least one TS mutant enzyme, which is indicated next to each strip of the dot plot.Pathway‐focused analysis of the metabolome data. Pathways with metabolite increases (mod. z‐score > 3) are highlighted in the heatmap. The color code indicates if one, two, or more than two metabolites increase in a particular pathway. Target pathways (yellow boxes) are defined as pathways that involve a TS mutant enzyme.Number of pathways that respond per strain (each dot is one strain, shown are 95 strains), and number of TS mutants that led to responses in a pathway (each dot is one pathway, shown are 93 pathways). A “response” means that at least one metabolite increases in a pathway (mod. z‐score > 3). The box‐whisker plot indicates the median (yellow line), and the 25^th^/75^th^ percentiles (gray box). Analysis is based on mean values of the metabolome data from three biological replicates. Source data are available online for this figure.

To probe how the TS mutations affect cellular metabolism, we analyzed the metabolomes of the 94 TS mutants (Fig 3A). For this purpose, we cultured the TS mutants and an unedited control strain at 42°C and measured metabolite level after 16 h by flow‐injection time‐of‐flight mass spectrometry (FI‐MS; Fuhrer *et al*, 2011; Farke *et al*, 2023). FI‐MS detected 325 metabolites, out of which 219 showed strong increases in at least one TS mutant (mod. z‐score > 3). We chose this mod. z‐score value of 3 considering the distributions of standard deviations between replicates (*n* = 3) and mod. z‐score values (Appendix Fig S13). We analyzed whether these strong metabolome changes were a global response to the growth reduction at 42°C or whether the metabolome responses were specific to the TS mutants. Most metabolite increases were specific, because 53 metabolites increased in a single TS mutant and only 14 metabolites increased in more than 10 mutants (increases with mod. z‐score > 3).

**Figure EV4:**
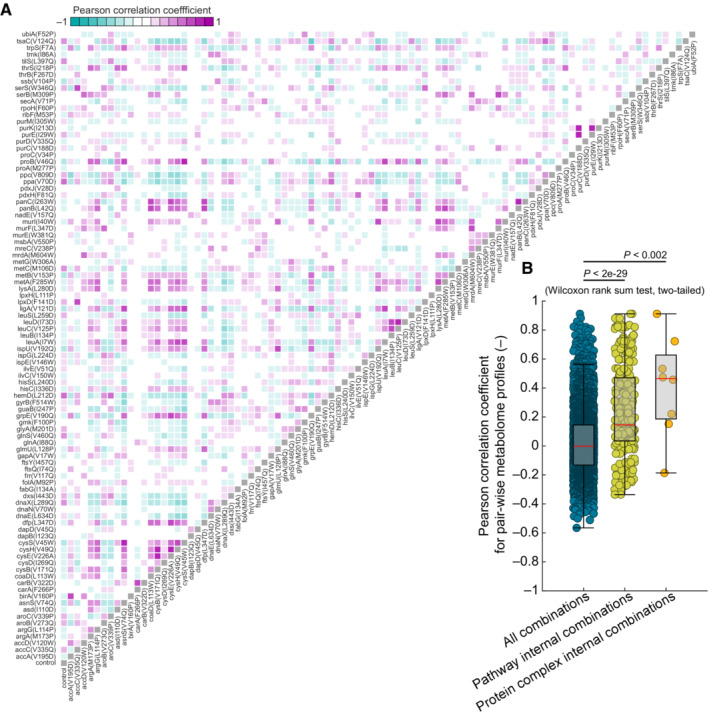
Correlation analysis of metabolomes of 94 TS mutants The heatmap shows the Pearson correlation coefficients (PCC) between metabolite data of all pair‐wise combinations of 94 TS mutants and a control strain. The metabolite levels were measured by FI‐MS after cultivation of the strains in 96‐well microtiter plates at 42°C for 16 h (*n* = 3). Data of 325 metabolites (mod. z‐scores) were used to calculate the PCC values.The dot plot shows all PCC values from (A) (given under “all combinations”), the PCC values from pairs of genes that are in the same metabolic pathway (given under “pathway internal combinations”), and from genes, whose proteins form a complex (given under “protein complex internal combinations”). The box‐whisker plot indicates the median (red line), and the 25^th^ and 75^th^ percentiles (each dot is a combination, shown are all 4,465 combinations, 272 pathway internal combinations, and 8 protein complex internal combinations). We tested for differences between the PCC values in the three groups using a Wilcoxon rank‐sum test (two‐tailed) and indicate the respective *P*‐values. Analysis is based on mean values of the metabolome data from three biological replicates.

Overall metabolome profiles were also TS mutant‐specific because there was almost no correlation between pair‐wise metabolome profiles of two TS mutants (or the control strain). This is illustrated by the median Pearson correlation coefficient (PCC) across all pairs of the 95 metabolomes, which was 0 (Fig EV4). However, the similarity was significantly higher between functionally related genes, which were genes in the same metabolic pathway and genes that belong to the same protein complex (*P* < 0.002, Wilcoxon rank‐sum test, two‐tailed, Fig EV4B and Dataset EV9). For example, subunits of the same enzyme complex had a high PCC, such as carbamoyl‐phosphate synthetase subunits CarA^F266P^ and CarB^V322D^, (PCC = 0.72) and the subunits of 3‐isopropylmalate dehydratase LeuC^V125P^ and LeuD^I73D^ (PCC = 0.91). Moreover, the TS mutant of the transcriptional regulator of the cysteine pathway CysB^V171Q^ had a similar metabolome as the TS mutants CysH^V49Q^ (PCC = 0.81) and CysE^V226A^ (PCC = 0.80), which are two enzymes in the cysteine pathway. Thus, metabolome responses in the TS mutants were strong and specific, and TS mutants with similar functions had similar metabolomes.

Sixty‐six out of 94 TS mutations affected enzymes that catalyze reactions in 44 metabolic pathways (Fig 3A). If these TS enzymes are less active at 42°C, they may create a metabolic bottleneck that limits flux through the associated pathway. Previous studies already showed that metabolic bottlenecks and perturbations in enzyme capacity can increase the concentration of upstream metabolites, especially the levels of substrate metabolites (Fendt *et al*, 2010; Fuhrer *et al*, 2017; Donati *et al*, 2021). Consistent with perturbations in enzyme capacity, the MurE^W381Q^ strain had a strong increase in the MurE‐substrate (UDP‐N‐acetyl‐α‐d‐muramoyl‐l‐alanyl‐d‐glutamate) and the HisC^I336D^ strain had an increase in the HisC‐substrate (3‐(imidazol‐4‐yl)‐2‐oxopropyl phosphate; Fig 3B). Thus, the metabolome data confirmed that the proximate cause of growth defects of the MurE^W381Q^ and HisC^I336D^ strains (Fig 2C) are bottlenecks in peptidoglycan and histidine biosynthesis, respectively. In total, substrate metabolites increased in 24 strains with TS enzymes (Fig 3B). The remaining 42 strains with TS enzymes showed no increases in the direct substrates, either because substrate metabolites were not covered by the metabolome data or because the substrate metabolite is unstable, like in the case of ProA^M277P^ (Smith *et al*, 1984), PurD^V335Q^ (Cheng *et al*, 1990), and PurE^I29W^ (Mueller *et al*, 1994). Another explanation for the absence of increasing substrates is that they are used by branching or competing metabolic pathways.

Next, we examined global metabolome changes and counted the number of metabolites that increased per metabolic pathway and per TS mutant (Fig 3C and Dataset EV10). Thirty‐six TS mutants showed a response in their “target pathway,” which is the metabolic pathway that involves the TS enzyme. These 36 TS mutants with local responses in the target pathway included the 24 strains with increases in direct substrates (Fig 3B) and another 12 mutants, in which other metabolites of the target pathway increased. Apart from local responses in the target pathways, we observed responses in distal pathways: A single TS mutation perturbed on average 12 metabolic pathways (Fig 3D), demonstrating that a single perturbation has global effects on metabolism.

Global metabolome changes can originate from regulatory interactions or from metabolites that participate in multiple pathways. For example, phosphoenolpyruvate (PEP) participates not only in glycolysis but also in aromatic amino acid biosynthesis. Therefore, the GapA^V17W^ strain, which has a temperature‐sensitive glycolysis enzyme (glyceraldehyde‐3‐phosphate dehydrogenase, GapA), had a primary bottleneck in glycolysis and a secondary bottleneck in aromatic amino acid biosynthesis. The glycolysis bottleneck was evidenced by increases in the substrate metabolite glyceraldehyde‐phosphate (Appendix Fig S14), and the bottleneck in aromatic amino acid biosynthesis was evidenced by increases in shikimate‐phosphate (Appendix Fig S14). Shikimate‐phosphate and PEP are both substrates of the 6^th^ step of aromatic amino acid biosynthesis catalyzed by 3‐phosphoshikimate 1‐carboxyvinyltransferase (AroA), and therefore, the low PEP levels in the GapA^V17W^ strain (Appendix Fig S14) seem to perturb the AroA capacity.

Regulatory interactions are another source of global metabolome changes in the TS mutants. For example, increases in substrate metabolites can allosterically inhibit enzymes in another metabolic pathway. An example of this regulatory crosstalk is the PanB^L42Q^ strain, in which local metabolome changes in the phosphopantothenate pathway propagate into the tyrosine biosynthesis pathway via an allosteric interaction. PanB catalyzes the first step of phosphopantothenate biosynthesis (Jones *et al*, 1993), and the PanB‐substrate is 3‐methyl‐2‐oxobutanoate, which increased in the TS mutant PanB^L42Q^ (Fig 3B). 3‐methyl‐2‐oxobutanoate is a known inhibitor of TyrB, which explains increases in the TryB‐substrate (hydroxy‐phenylpyruvate) in the PanB^L42Q^ strain (Appendix Fig S14).

In summary, most TS mutants showed strong and specific metabolome changes, demonstrating that TS mutants have diverse metabolic states. Despite the gene‐specific response of the metabolome, we also noticed a growth rate effect in nongrowing strains (growth rate < 0.1 h^−1^), which had more increased metabolites than growing strains (growth rate ≥ 0.1 h^−1^, Appendix Fig S15). Moreover, a random forest model predicted nongrowing strains based on their metabolome data with an accuracy of up to 70% (Appendix Fig S15). Together, these results suggest that the metabolome changes are influenced at two levels: (i) by global growth effects and (ii) by local effects in the perturbed metabolic pathways. Next, we inspected the accumulation of substrate metabolites.

### Production of substrate metabolites is long‐lasting and tunable by temperature

**Figure 4:**
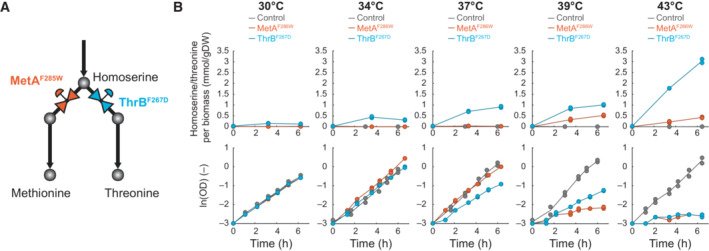
MetA^F285W^ and ThrB^F267D^ function as metabolic valves and overproduce homoserine Two TS mutants, MetA^F285W^ and ThrB^F267D^, at the homoserine branchpoint. MetA catalyzes the first step in the methionine biosynthesis pathway in *E. coli*, and ThrB catalyzes the first step in the threonine biosynthesis pathway.Biomass‐specific concentration of the pool of homoserine and threonine (LC–MS/MS could not separate homoserine and threonine). Shown are cultures of the MetA^F285W^ strain (red), the ThrB^F267D^ strain (blue), and the control strain (gray). The strains were grown in shaking flasks at the indicated temperatures. Dots show samples from two replicates (*n* = 2). Lines connect mean values. Lower charts show the optical density (OD) in the same cultures. Source data are available online for this figure.

In 24 TS mutants, the direct substrate metabolite increased (Fig 3B), presumably, because the TS mutation reduced enzyme capacity at 42°C, which in turn leads to a bottleneck in the target pathway. To further analyze increases in substrate metabolites, we examined whether a TS mutant produces substrate metabolites for a longer period of time and whether substrate increases are tunable by temperature. Therefore, we focused on homoserine, which increased in the MetA^F285W^ strain and in the ThrB^F267D^ strain (Fig 3B). MetA (homoserine O‐succinyltransferase) catalyzes the first step in methionine biosynthesis, ThrB (homoserine kinase) catalyzes the first step in threonine biosynthesis, and homoserine is the common substrate of MetA and ThrB (Fig 4A). We cultured the MetA^F285W^ strain and the ThrB^F267D^ strain at different temperatures and measured homoserine in the whole culture broth using LC–MS/MS (Fig 4B). Our LC–MS/MS method could not separate homoserine and threonine, and therefore, we measured the total pool of homoserine and threonine. In the following, we assume that homoserine is responsible for the increases in the total pool of homoserine and threonine because threonine is downstream of MetA and ThrB. At 30°C, homoserine did not accumulate in both mutants (MetA^F285W^ and ThrB^F267D^, Fig 4B), demonstrating that the two TS enzymes are functional at 30°C. This is consistent with their normal growth phenotype at 30°C, which was similar to the control strain (Fig 4B). Higher temperatures induced homoserine production: Gradual temperature increases (34, 37, 39, and 43°C) led to gradual increases in homoserine production in the ThrB^F267D^ strain (Fig 4B). At the same time, growth of the ThrB^F267D^ strain decreased at higher temperatures. At 43°C, the ThrB^F267D^ strain did not grow and stably produced homoserine for at least 6 h. The MetA^F285W^ mutant showed a similar behavior as the ThrB^F267D^ strain but produced less homoserine.

**Figure EV5:**
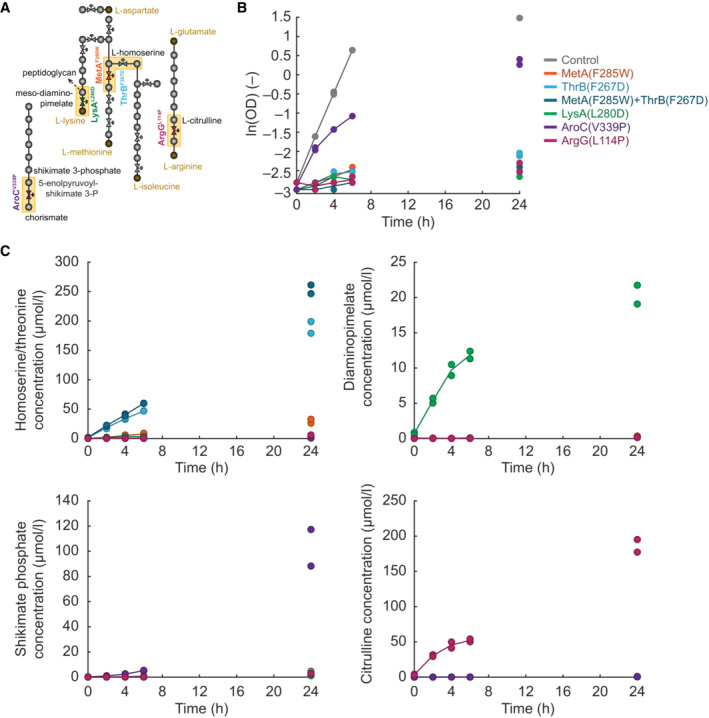
Substrate production in TS mutants of enzymes Schematic of biosynthesis pathways of chorismate, lysine, methionine, isoleucine, and arginine. Dots represent metabolites. Valve symbols indicate TS mutant enzymes.The chart shows the natural logarithm of biomass data (OD) from shaking flask cultivations of TS mutants and a control strain at 42°C. Dots are data from two replicates, and the lines connect the means.The charts show the concentrations (μmol/l) of indicated metabolites during the cultivations from (B). The concentrations were quantified in samples of the whole culture broth by LC–MS/MS. Dots are data from two replicates, and the lines connect the means.

Thus, the TS mutants MetA^F285W^ and ThrB^F267D^ enable tight control of homoserine overproduction: At 30°C, they do not overproduce homoserine, whereas higher temperatures gradually increase homoserine production. Both strains remain metabolically active for at least 6 h, even if growth is fully arrested (at 43°C). In additional experiments, we found that homoserine production continued for up to 24 h, with specific production rates of 48 μmol g_DW_^−1^ h^−1^ for the MetA^F285W^ strain and 270 μmol g_DW_^−1^ h^−1^ for the ThrB^F267D^ strain (Fig EV5). Combining the MetA^F285W^ and the ThrB^F267D^ mutations into a double TS mutant had an additive effect, because it increased the homoserine production even further to 477 μmol g_DW_^−1^ h^−1^. Other three TS mutants (LysA^L280D^, AroC^V339P^, and ArgG^L114P^) showed a similar overproduction of substrate metabolites during a phase of 24‐h growth arrest (Fig EV5), demonstrating that TS mutants are generally applicable to produce a wide range of bacterial metabolites.

### Temperature‐sensitive DNA polymerase DnaX^L289Q^ decouples microbial growth from overproduction of arginine

**Figure 5:**
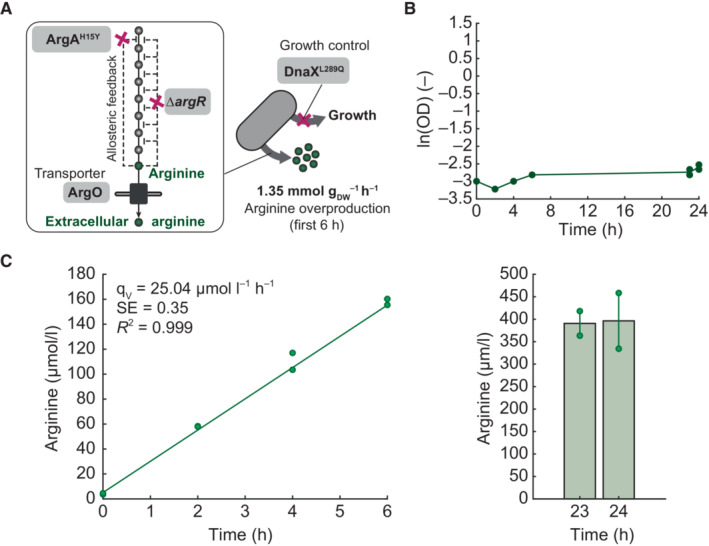
Decoupling growth from arginine overproduction by DnaX^L289Q^ Arginine overproduction strain with the TS mutation DnaX^L289Q^ for growth control. Dysregulation of the arginine pathway was achieved by deleting *argR* (removes transcriptional feedback) and inserting the ArgA^H15Y^ mutation (removes allosteric feedback). The arginine exporter ArgO was overexpressed from a plasmid.OD of the engineered arginine overproduction strain during cultivations at 42°C in shaking flasks. Dots are samples from two replicates (*n* = 2), and the line connects the means.Arginine concentration (μmol/l) during the same cultivation (shown in B). Arginine levels were quantified in the whole culture broth by LC–MS/MS and calibrated with an authentic arginine standard. Dots show samples from two replicates (*n* = 2). The volumetric arginine production rate q_V_ in the initial 6 h of cultivation was determined using a linear regression model (*R* ^2^ = 0.99, SE: standard error of the slope q_V_). The bar plot shows final arginine concentrations that were determined after 23 and 24 h. Source data are available online for this figure.

Finally, we tested whether we could use a TS mutant to control the growth of an engineered overproduction strain. Therefore, we inserted the TS mutation DnaX^L289Q^ into an arginine overproducing *E. coli* strain (Sander *et al*, 2019) to control its growth by temperature (Fig 5A). We selected the DnaX^L289Q^ mutation, because the DnaX^L289Q^ mutant grew well between 30 and 34°C and stopped growing at temperatures above 38°C (Fig EV3). Moreover, the DnaX^L289Q^ mutant showed no strong metabolome changes at 42°C (Fig 3C), and we assumed that this reduces interferences with arginine production. The arginine overproduction strain was constructed by removing transcriptional feedback of the arginine repressor (Δ*argR*), which results in overexpression of arginine enzymes (Sander *et al*, 2019). Additionally, a point mutation was inserted into the first enzyme of the arginine pathway (ArgA^H15Y^) to remove allosteric feedback inhibition by arginine. To facilitate transport of arginine, the arginine exporter ArgO was overexpressed from a plasmid (Fig 5A). This resulted in a strain with four modifications: (i) overexpression of ArgO, (ii) a point mutation ArgA^H15Y^, (iii) a gene deletion Δ*argR*, and (iv) a point mutation DnaX^L289Q^. In the following, we refer to this strain as the TS arginine production strain.

The TS arginine production strain was cultivated for 24 h at 42°C in minimal glucose medium. As expected, the strain did not grow at 42°C due to the TS mutation DnaX^L289Q^ (Fig 5B). Arginine was quantified in the culture broth by LC–MS/MS to estimate the biomass‐specific arginine production rate (Fig 5C). During the initial 6 h of the growth arrest, the arginine production rate was constant at 1.35 mmol g_DW_^−1^ h^−1^, which is 59% of the production rate (2.3 mmol g_DW_^−1^ h^−1^) that can be achieved with the growing arginine production strain without the DnaX^L289Q^ mutation (Sander *et al*, 2019). Despite the lower arginine production rate compared with growing cells (59%), producing arginine with nongrowing *E. coli* has several advantages for process performance. The main advantage is a higher yield, because nongrowing bacteria convert the feedstock mainly toward product synthesis. Another advantage is that nongrowing bacteria are more stable and less likely to undergo mutations that lead to a decline in product formation. Finally, two‐stage processes with nongrowing cells simplify process control, because there is no need to control growth‐related parameters like feeding rates. Thus, the DnaX^L289Q^ TS allele can stop the growth of arginine‐producing *E. coli*, and this opens up new possibilities for a temperature‐controlled production of arginine in two‐stage bioprocesses.

## Discussion

CRISPR‐based genome editing methods have become highly efficient and versatile in their application. They enabled the construction of thousands of targeted genomic edits in large mutant libraries, which were then screened for various phenotypes such as responses to antibiotics (Garst *et al*, 2017; Meier *et al*, 2022; Dewachter *et al*, 2023) or improved production of biochemicals (Liang *et al*, 2017). Here, we used CRISPR‐based genome editing to screen for single amino acid changes that cause temperature sensitivity. So far, TS mutants were mostly constructed by random mutagenesis approaches and subsequent screening for TS growth of single colonies (Ben‐Aroya *et al*, 2008; Li *et al*, 2011; Kofoed *et al*, 2015). Here, we designed and introduced 15,120 mutations that resulted in up to 50 single amino acid changes in 346 essential proteins (out of all 352 essential proteins). Amino acid changes were designed with the TSpred algorithm (Tan *et al*, 2014), such that they have a high chance to decrease thermal stability of the target protein. Our data indicate that many of these predictions decreased protein stability already at 30°C, because a large fraction of the CRISPR library (55%) had strong fitness defects at 30°C. Nevertheless, 1,269 strains in the CRISPR library grew well at 30°C and showed fitness defects at 42°C, thus indicating that they are temperature‐sensitive. Despite the limited search space (max. 50 mutations per gene), we found that distinct amino acid changes were enriched in TS mutants. Thus, future studies could scale up the approach presented here and search all TS alleles of essential *E. coli* proteins. Such a dataset may have the potential to train machine learning models that predict TS mutations in *E. coli* proteins and then transfer this knowledge to other organisms.

To validate that the CRISPR screen identified genuine TS mutants, we focused on the allelic series of *cysS*. To this end, we reconstructed 14 TS mutants of *cysS*, all of which exhibited temperature‐dependent growth in plate reader cultivations. This confirmed that the CRISPR screen effectively identified authentic TS mutants. Additionally, we reconstructed a pool of 250 putative TS mutants and successfully confirmed a TS phenotype in 94 of them. However, for the remaining 156 strains, it has proven to be a challenge to determine whether they possess the TS phenotype or not. A conservative estimate is that only 94 out of the initial 250 strains are true TS mutants. However, it is important to note that the abundance of the 250 strains varied significantly, and we observed a bias toward highly abundant mutants during isolation of the strains. Therefore, the 2016 random isolates have likely missed a significant portion of the 250 strains. Furthermore, measurements of TS phenotypes in plate readers were limited to a ca. 6‐h exponential phase. In contrast, the CRISPR screen allowed us to monitor TS phenotypes for a duration of 14 h, because we back‐diluted the cultures. Therefore, it is likely that the plate reader screening method missed some delayed TS phenotypes that have been captured in the extended time frame of the CRISPR screen. These factors suggest that the number of true TS mutants in the sublibrary of 250 strains could potentially be higher than our conservative estimate of 94.

We used the panel of 94 TS mutants to show that they function as growth switches, which arrest cellular growth at temperatures between 32 and 44°C. A unique advantage is that some of these growth switches operate at very fast time scales, as exemplified for the MurE^W381Q^ and HisC^I336D^ mutants that switched from growth to no growth within 30 min. Future studies should explore if all mutants respond at such fast time scales and if the growth switches are reversible (i.e., if cells regrow upon temperature decreases). Others constructed growth switches by expressing essential genes under control of inducible promoters (Izard *et al*, 2015) or by repressing transcription of essential genes by CRISPR interference (Li *et al*, 2016, 2020). These growth switches operate at slower time scales than TS mutants, because they interfere with *de novo* synthesis of essential proteins and do not alter the activity of proteins that are already expressed. The consequence is that knockdowns with CRISPR interference, for instance, show significant time delays between induction and the emergence of a phenotype (Donati *et al*, 2021; Anglada‐Girotto *et al*, 2022).

A further challenge for all growth switches is that bacteria can escape the growth arrest by mutations and other compensatory mechanisms. For example, cells can escape a CRISPRi‐mediated growth arrest by loss‐of‐function mutations in the dCas9 protein or in sgRNA sequences that alleviate the transcriptional repression. We expect that TS mutants are less prone to escaping their growth‐arrested state, because this would require unique mutations that restore the protein function at high temperatures. Moreover, combining multiple TS mutations (e.g., our double TS mutant MetA^F285W^ ThrB^F267D^) should further decrease the risk of escape mutations, since cells have to restore the function of two essential proteins.

The TS mutants allowed us to arrest growth in various ways, for example, by blocking replication (DnaX^L289Q^) or metabolic functions like histidine biosynthesis (HisC^I336D^). The way we arrested growth had strong effects on metabolism under growth arrest: Some TS mutants had a wild‐type‐like metabolome, whereas other TS mutants had strong metabolome changes in many metabolic pathways. This gene‐specific response of metabolism of the TS mutants might be relevant for their application in bioprocesses, where TS mutants could function as metabolic valves (Venayak *et al*, 2018) or growth switches in two‐stage bioprocesses (Burg *et al*, 2016). So far, it remains unclear whether some TS mutants are better suited for bioprocesses than others. It is likely that the choice of TS mutant has a significant impact on the longevity of (nongrowing) producer cells and thus on the productivity of a bioprocess. However, more work is required to understand which TS mutants can improve the production of a particular bio‐based product (Jang *et al*, 2023). Nevertheless, we expect that TS‐based growth control creates new possibilities to increase robustness and productivity of two‐stage bioprocesses.

In conclusion, there is a growing interest in nongrowing bacteria, and we have shown that TS mutants are a robust and versatile tool to control bacterial growth. Our data indicate that the metabolic state of nongrowing bacteria is diverse, which means that nongrowing bacteria do not enter a universal standby mode. Instead, the metabolic state of nongrowing bacteria depends on the cellular process that causes growth arrest. Our ability to control bacterial growth, with TS mutants or other synthetic circuits, will open up novel applications in metabolic engineering and industrial biotechnology.

## Materials and Methods

### Reagents and Tools table

**Table:** 

Reagent/Resource	Reference or source	Identifier or Catalog number
**Experimental models**		
*Escherichia coli* MegaX DH10B T1^R^	Invitrogen, Thermo Fisher Scientific Inc.	#C640003
*Escherichia coli* BW25113	Datsenko and Wanner (2000)	–
*Escherichia coli* BW25113//pTS041	this study	–
*Escherichia coli* BW25113//pTS041//pTS040(…)	this study, also see Expanded View Datasets EV2 and EV5	–
*Escherichia coli* BW25113 *metA*(F285W) *thrB*(F267D) //pTS041//pTS055(ThrB^F267D^)	This study	–
*Escherichia coli* MG1655 Δ*argR argA*(H15Y)	Sander *et al* (2019)	–
*Escherichia coli* MG1655 Δ*argR argA*(H15Y)//pTS041	This study	–
*Escherichia coli* MG1655 Δ*argR argA*(H15Y) *dnaX*(L289Q) //pTS041//pTS040(DnaX^L289Q^)	This study	–
*Escherichia coli* MG1655 Δ*argR argA*(H15Y) *dnaX*(L289Q) //pTS041//pTS056	This study	–
**Recombinant DNA**		
pTS040	This study, p15A ori, *cmR*, homology arms and sgRNA for CRISPR	https://benchling.com/s/seq‐0ijrtTWuspbAebVXD6bM?m=slm‐cpPflbh0BtEj4KsblUhN
pTS041	This study, pSC101 ori, *kanR*, *tetR*, *Streptococcus pyogenes cas9*, *araC*, *Escherichia virus Lambda red*	https://benchling.com/s/seq‐EaE3OVG44GyhJG0acsVY?m=slm‐Klx34pqqKkyC4iKaZvEH
pTS055	This study, p15A ori, *speI*, homology arms and sgRNA for CRISPR	https://benchling.com/s/seq‐lThcIt5a7cbYoQkn4kO2?m=slm‐aSvHclqLE2GxUq0jPKjo
pargO	Sander *et al* (2019), used to construct pTS056	‐
pTS056	This study, p15A ori, *ampR*, *tetR*, *E. coli argO*	https://benchling.com/s/seq‐aJ3x3DQ9S3ciPy5ClPbi?m=slm‐vlL6DP9BXQfaiM8CcTZD
**Oligonucleotides**		
CRISPR library	Design by T.S., oligonucleotide pool produced by Twist Bioscience	Expanded View Dataset EV2
CRISPR library of 250 putatively TS mutants	Design by T.S., oligonucleotide pool produced by Twist Bioscience	Expanded View Dataset EV5
cgatgccattgggatatatcaacggtggta	seq_F, sequencing primer for pTS040 this study	–
CTGCAGTCTAGACTCGAGTAAGGATCCAGTTC	seq_R, sequencing primer for pTS040 and pTS055, this study	
tctgcctcgtgatacgcctatctagtagacgtcgatatctggcgaaaatg	pTS040‐R, for pTS040 cloning, this study	–
tcgacgtctactagataggcgtatcacgaggcagaTCCTCTGGCGGAAAGCCT	library_F, for pTS040 cloning, this study	–
GTTTTAGAGCTAGAAATAGCAAGTTAAAATAAGGC	Ec‐F‐control, for pTS040 and pTS055 cloning, Larson *et al* (2013)	–
ACTTTTTCAAGTTGATAACGGACTAGCCTTATTTTAACTTGCTATTTCTAGCTCTAAAAC	CREATE_insertR (general), for pTS040 and pTS055 cloning, Garst *et al* (2017)	–
gtatcacgaggcagaTCCTCTG	NGS_F, for amplicon deep sequencing of pTS040, this study	–
ACTCGGTGCCACTTTTTCAAGTT	BC1, for amplicon deep sequencing of pTS040, Garst *et al* (2017)	–
gaaattctgcctcgtgatacgcctagtgcgcggaacccctatttgtttatttttctaaatacattca	pTS055_R, for pTS055 cloning, this study	–
cgcactaggcgtatcacgaggcagaatttcTCCTCTGGCGGAAAGCCT	library_spec_F, for pTS055 cloning, this study	–
TCGTCGGCAGCGTCAGATGTGTATAAGAGACAGGTATCACGAGGCAGATCCTCTG	index_F, indexing primer for NGS, this study	–
GTCTCGTGGGCTCGGAGATGTGTATAAGAGACAGACTCGGTGCCACTTTTTCAAGTT	index_R, indexing primer for NGS, this study	–
**Chemicals, enzymes and other reagents**		
Q5 High‐fidelity DNA polymerase	New England Biolabs, Inc.	#M0491L
NEBuilder HiFi DNA Assembly Master Mix	New England Biolabs, Inc.	#E2621X
AMPure XP PCR beads	Beckman Coulter	#A63881
l‐arabinose	Sigma‐Aldrich (Merck KGaA)	#A3256‐500G
anhydrotetracycline (aTc)	Sigma‐Aldrich (Merck KGaA)	#37919‐100MG‐R
Carbenicillin	Sigma‐Aldrich (Merck KGaA)	#C1389‐1G
Chloramphenicol	Sigma‐Aldrich (Merck KGaA)	#C0378‐25G
Kanamycin	Carl Roth GmbH + Co. KG	#T832.3
Spectinomycin	Sigma‐Aldrich (Merck KGaA)	#S4014‐5G
l‐glycerol	Sigma‐Aldrich (Merck KGaA)	#15523‐1L‐M
l‐arginine	Sigma‐Aldrich (Merck KGaA)	#W381918‐1KG
l‐homoserine	Sigma‐Aldrich (Merck KGaA)	#H6515‐1G
Shikimate 3‐phosphate	Sigma‐Aldrich (Merck KGaA)	#S0702‐1MG
l‐citrulline	Sigma‐Aldrich (Merck KGaA)	#C7629‐10MG
Na_2_HPO_4_	Carl Roth GmbH + Co. KG	#P030.2
KH_2_PO_4_	Carl Roth GmbH + Co. KG	#3904.1
NaCl	Carl Roth GmbH + Co. KG	#9265.1
(NH_4_)_2_SO_4_	Sigma‐Aldrich (Merck KGaA)	#A3920
ZnSO_4_ 7× H_2_O	Sigma‐Aldrich (Merck KGaA)	#Z0251‐100G
CuCl_2_ 2× H_2_O	Sigma‐Aldrich (Merck KGaA)	#307483‐100G
MnSO_4_ × H_2_O	Sigma‐Aldrich (Merck KGaA)	#M8179‐100G
CoCl_2_ 6× H_2_O	Sigma‐Aldrich (Merck KGaA)	#C8661‐25G
Thiamine‐HCl	Sigma‐Aldrich (Merck KGaA)	#T4625‐25G
MgSO_4_ 7× H_2_O	Sigma‐Aldrich (Merck KGaA)	#63138‐250G
CaCl_2_ 2× H_2_O	Sigma‐Aldrich (Merck KGaA)	#C8106‐500G
FeCl_3_ 6× H_2_O	Sigma‐Aldrich (Merck KGaA)	#31232‐250G‐D
D‐glucose	Carl Roth GmbH + Co. KG	#X997.4
**Software**		
Matlab (Version: R2022a)	The MathWorks, Inc.	https://ch.mathworks.com/de/products/matlab.html
MSConvert	Chambers *et al* (2012)	–
Adobe Illustrator 2023	Adobe	–
Cas‐OFFinder	Bae *et al* (2014)	http://www.rgenome.net/cas‐offinder/
TSpred	Tan *et al* (2014) and Varadarajan *et al* (1996)	http://cospi.iiserpune.ac.in/TSpred/Predict.html
R (Version: 4.2.2)	The R Foundation for Statistical Computing	https://www.r‐project.org
RStudio Desktop	Posit Software, PBC formerly RStudio, PBC	
R package “Peptides”	Osorio *et al* (2015)	
**Other**		
Epoch 2 plate reader	Biotek (now: Agilent Technologies)	–
Infinite 200 PRO plate reader	TECAN	–
NextSeq 500 Mid Output Kit v2.5 (300 cycles)	Illumina	#20024908
6546 QTOF mass spectrometer	Agilent Technologies	–
6495 triple QQQ mass spectrometer	Agilent Technologies	–
1290 Infinity II UHPLC system	Agilent Technologies	–
DNA Clean & Concentrator‐5	Zymo Research	#D4004
NucleoSpin Gel and PCR Clean‑up Kit	Macherey‐Nagel	#740609.250
0.1 cm Gene Pulser Cuvette	BioRad	#165‐2089
Micropulser Electroporator	BioRad	#1652100
Breathe‐Easy	Diversified Biotech	#BEM‐1
96‐well plates	Greiner Bio‐One GmbH	#655185

### Methods and Protocols

#### Construction of plasmids

The CRISPR‐Cas9 genome editing method was a modified version of the CREATE method (Garst *et al*, 2017). Two plasmids (pTS040 and pTS041) were constructed using Gibson assembly. pTS040 had the p15A origin of replication and carried a chloramphenicol resistance gene, a cassette with the homology arm for recombination, and the guide RNA of the CRISPR system under control of a constitutive promoter (P_J23119_). pTS041 had the pSC101 origin of replication and carried a kanamycin resistance gene, a gene for the anhydrotetracycline (aTc)‐sensitive repressor *tetR*, *cas9* under control of the aTc controlled P_LtetO1_ promoter, the arabinose‐sensitive repressor *araC*, and the *Escherichia virus Lambda* genes *red* under control of the arabinose‐controlled promoter P_araBAD_. pTS055 was pTS040 with a spectinomycin resistance gene instead of a chloramphenicol resistance gene. pTS056 had a p15A origin of replication, an ampicillin resistance gene, the anhydrotetracycline(aTc)‐sensitive repressor *tetR*, and *argO* encoding for an arginine exporter under the aTc controlled P_LtetO1_ promoter. pTS056 was based on a plasmid from (Sander *et al*, 2019). Plasmids were constructed with Q5 High‐fidelity DNA polymerase (New England BioLabs Inc., NEB) and the Gibson Assembly Master Mix (NEB). We used the DNA Clean & Concentrator Kit (Zymo Research) to purify DNA after PCRs.

#### Design of the temperature‐sensitive *E. coli* library

The TSpred tool (Varadarajan *et al*, 1996; Tan *et al*, 2014) was used to predict TS mutations for all 352 genes that are essential for the growth of *E. coli* on minimal glucose medium (Patrick *et al*, 2007; Goodall *et al*, 2018). It is predicted, which amino acid of a protein, upon substitution by one of the five amino acids alanine, tryptophan, glutamine, aspartate, or proline, is likely to introduce temperature sensitivity. If possible, crystal structures of the target proteins were given as an input for the algorithm. Otherwise, amino acid sequences were given as input. The predictions are listed in Expanded View Dataset EV1.

Because the quality of a protospacer for CRISPR‐Cas9 genome editing is strongly affected by the distance of the PAM to the target site (Garst *et al*, 2017) and its off‐targets, we considered every PAM within 30 bp distance for every predicted site and tested whether a silent PAM mutation was possible. In some cases, the PAM and the target site overlapped, and, therefore, not every amino acid substitution was possible to remove the PAM. The Cas‐OFFinder (Bae *et al*, 2014) was used to identify off‐targets with up to 4 mismatches. We also tested whether the protospacers have a 11 PAM‐proximal perfect match to multiple PAMs (Rousset *et al*, 2018). Based on these results, we then ranked each available design for every site with a custom scoring system and chose 10 predicted sites for each gene that had the highest‐ranking designs (Table 1). We excluded designs that did not reach a minimum score of 3 such that some target genes yielded no or less than 10 designs. The final library contained 16,038 members covering 346 genes.

**Table 1:** Custom scoring system of cassette designs. The minimum score was 3. Score larger than 3 are indicated with green, and scores smaller than 3 with gray.

Distance	0 nt	3 nt	6 nt	9 nt	12 nt	15 nt	18 nt	21 nt	24 nt	27 nt	30 nt
Class 1	5	4.8	4.6	4.4	4.2	4.0	3.8	3.6	3.4	3.2	3.0
Class 2	4.41	4.21	4.01	3.81	3.61	3.41	3.21	3.01	2.81	2.61	2.41
Class 3	3.9	3.7	3.5	3.3	3.1	2.9	2.7	2.5	2.3	2.1	1.9
Class 4	2	1.8	1.6	1.4	1.2	1.0	0.8	0.6	0.4	0.2	0

The distance between the silent PAM mutation and the target site is considered in steps of three nucleotides (nt). Cassette designs in “class 1” do not have another 11 nt PAM‐proximal perfect match and no off‐target with up to four mismatches. Cassette designs in “class 2” do not have another 11 nt PAM‐proximal perfect match. Cassette designs in “class 3” do not have an off‐target with up to four mismatches. Cassette designs in “class 4” have other 11 nt PAM‐proximal perfect matches and off‐targets with up to 4 mismatches. For each site, the 10 highest‐ranking cassette designs were chosen. The minimum score was 3. If only four amino acid substitutions were possible at a given site, a penalty of −0.95 was applied to the score (3 substitutions: −1.25, 2 substitutions: −3).

Oligonucleotides to construct the library were 200 bp long and contained in the following order: a spacer sequence (“TCCTCTGGCGGAAAGCC”), a homology sequence with the desired mutation and a silent PAM mutation, another spacer (“GATC”), the J23119 promoter (“TTGACAGCTAGCTCAGTCCTAGGTATAATACTAGT”), a protospacer, and a part of the sgRNA‐Cas9 handle (“GTTTTAGAGCTAGAAATAGCAAGTTAAAATAAGGCTAG”). To calculate hydrophobicities over a 11 amino acid window (Kyte & Doolittle, 1982), we used R (version 4.2.2), RStudio Desktop (version 2022.07.2‐576), and the R package “Peptides” (Version 2.4.5) (Osorio *et al*, 2015). Protein structural information was obtained from UniProt (The UniProt Consortium, 2023).

#### Strain construction

##### Cloning the CRISPR libraries and single mutants

The oligonucleotide pools were manufactured by Twist Bioscience (South San Francisco, United States). The oligonucleotides were used as template for PCR amplification (oligonucleotide concentration: 0.1 μM; 15 cycles). The PCR products of correct size were purified by agarose gel electrophoresis (NucleoSpin Gel and PCR Clean‐up Kit, Macherey‐Nagel). The purified linear DNA was used for cloning of pTS040 by Gibson assembly (NEBuilder HiFi DNA Assembly Reaction, NEB) and electroporation of *E. coli* MegaX DH10B T1^R^ cells (Invitrogen, Thermo Fisher Scientific Inc.). *E. coli* BW25113 carrying pTS041 was cultured in LB medium at 37°C under shaking of 220 rpm until exponential growth. Expression of the *Lambda red* genes was induced with l‐arabinose (7.5 g/l). After 30 min, the culture was harvested for electroporation with the pooled pTS041 plasmid library (0.1 cm Gene Pulser Cuvette #165‐2089 and Micropulser, BioRad). Cells were recovered in SOC medium with kanamycin and 1 μM aTc for Cas9 induction at 30°C for 2 h and streaked out onto LB agar plates with kanamycin, chloramphenicol, and 1 μM aTc. After incubation overnight at 30°C, colonies were pooled by flushing the agar plates with LB medium, glycerol added (final concentration: 22 vol.‐%), the OD was determined, and the strain library stored as cryo stocks. Plasmids to reconstruct single mutants (*cysS* allelic series and mock edit controls) were cloned using an *in vivo* assembly cloning approach (García‐Nafría *et al*, 2016) and 300‐bp‐long double‐strand DNA fragments with the same design as the oligonucleotides from the large CRISPR libraries plus additional 50 bp overhangs at each site into the template plasmid. The DNA fragments were purchased from Twist Bioscience (South San Francisco, United States).

##### Construction of the sublibrary of 250 mutants

The sublibrary of 250 mutants (Dataset EV5) was constructed following the same protocol as for the large CRISPR library. After plating the pooled library onto LB agar plates with kanamycin and chloramphenicol, 2016 colonies were picked, cultivated in LB with kanamycin and chloramphenicol in 96‐well plates at 30°C overnight, and cryo stocks prepared. Subsequently, growth was measured in plate reader cultivations at 30 and 42°C. Four hundred and fifty‐six isolates were selected for sequencing, out of which 123 were unique (Dataset EV6) and 94 selected for further work.

##### Construction of the double TS mutant MetA^F285W^ + ThrB^F267D^ by sequential CRISPR‐Cas9 genome editing

A 50 ml LB culture without chloramphenicol of the TS mutant MetA^F285W^ (*E. coli* BW25113 *metA*(F285W)// pTS041// pTS040(MetAF285W)) was started from cryo stock and incubated for 22 h at 30°C under 220 rpm of shaking. After diluting the culture 1:10,000 in fresh LB without chloramphenicol, the cells were further incubated overnight at 30°C under shaking of 220 rpm. A fresh culture was started in the morning by 1:50 dilution in the same medium and conditions. After ca. 2 h, *Lambda Red* was induced for 30 min by addition of l‐arabinose (7.5 g/l). Cells were subsequently transformed by electroporation with pTS055(ThrB^F267D^) (0.1 cm Gene Pulser Cuvette #165–2089 and Micropulser, BioRad). Cells were recovered in SOC medium with kanamycin and 1 μM aTc for Cas9 induction at 30°C for 2 h and streaked out onto LB agar plates with kanamycin, spectinomycin, and 1 μM aTc. After incubation at room temperature, single isolates were stored as cryo stocks and checked for correct mutations by Sanger sequencing.

##### Construction of the DnaX^L289Q^ arginine overproduction strain

The strain *E. coli* MG1655 Δ*argR* ArgA‐H15Y//pTS041 was used for transformation with pTS040(DnaX^L289Q^). The genomic edit was confirmed by Sanger sequencing. Subsequently, the strain was transformed with pTS056 that was used to overexpress the arginine exporter ArgO. It was previously described that basal expression of ArgO was sufficient (Sander *et al*, 2019) such that aTc was not added to subsequent cultures.

#### Cultivations

If not stated otherwise, minimal medium (M9) was used for the experiments and contained 42.2 mM Na_2_HPO_4_, 11.3 mM (NH_4_)_2_SO_4,_ 22 mM KH_2_PO_4_, 8.56 mM NaCl, 1 mM MgSO_4_ × 7 H_2_O, 100 μM CaCl_2_ × 2 H_2_O, 60 μM FeCl_3_, 6.3 μM ZnSO_4_ × 7 H_2_O, 7 μM CuCl_2_ × 2 H_2_O, 7.1 μM, MnSO_4_ × 2 H_2_O, 7.6 μM CoCl_2_ × 6 H_2_O, and 2.8 μM thiamine‐HCL. Glucose was used as carbon source (final concentration 5 g/l). M9 and LB agar plates contained 1.5% agar. Chloramphenicol (30 μg/ml final concentration), kanamycin (50 μg/ml final concentration), carbenicillin (100 μg/ml final concentration), and spectinomycin (50 μg/ml final concentration) were added to the media when required.

##### Competition experiment and sampling for amplicon sequencing

The TS plasmid library (before electroporation of *E. coli* BW25113//pTS041) was used as a sample for amplicon sequencing (“sample before recombination”). Plasmids were extracted from the cryo stock of the TS strain library (*E. coli* BW25113//pTS041//pTS040(TS‐library), “sample after recombination”). Seventy‐five milliliter M9 medium was inoculated with 200 μl of the TS strain library from cryo stock and incubated in a 500 ml shake flask for 15 h at 30°C under shaking of 220 rpm. Ten milliliter of the exponentially growing culture were used for plasmid extraction (“sample time point zero”). Three hundred milliliter of M9 medium was inoculated with the previous culture to a start OD of 0.1. The 300 ml culture was split up to each 150 ml for cultivation in 1 l shake flasks at 30 and 42°C under shaking of 220 rpm. Every 3 h, the 150 ml cultures were back‐diluted to an OD of 0.1. Every 2 h, a sample for plasmid extraction was taken (sample volume × OD ≥ 5).

##### Plate reader cultivations

Five hundred microliter of LB in 2 ml deep‐well plates (96‐well) was inoculated from cryo stocks, covered with Breathe‐Easy (Diversified Biotech BEM‐1) adhesive membrane, and incubated for 6 h at 30°C under shaking at 220 rpm. Five hundred microliter M9 (5 g/l glucose) was inoculated with 1 μl of the LB precultures and incubated overnight at 30°C in 2 ml deep‐well plates under shaking of 220 rpm. A 297 μl of M9 (5 g/l glucose) was inoculated with 3 μl of the overnight cultures in 96‐well Greiner plates (flat‐bottomed). One hundred and fifty microliter were transferred to a second 96‐well Greiner plate. Each plate was incubated at each two different temperatures. Epoch 2 (BioTek, now: Agilent Technologies) or Infinite 200 Pro (TECAN trading AG) plate readers were used for incubation and measurements of OD at 600 nm every 10 min. Maximum specific growth rates were calculated in exponential growth phases if applicable. Negative values were considered as no growth (= 0 h^−1^). Statistical testing was performed on raw values.

##### Dynamic switch from 30 to 42°C with the TS mutants MurE^W381Q^ and HisC^I74Q^

Five milliliter LB cultures were started from cryo stock. After ca. 6 h at 30°C under shaking at 220 rpm, 5 ml M9 (5 g/l glucose) overnight cultures (30°C, 220 rpm) were started using 25 μl of the LB culture for inoculation. Overnight M9 cultures were washed: The cultures were pelletized by 5 min of centrifugation at, 3,200 *g* and 30°C. After removing the supernatant, 5 ml of fresh M9 glucose medium was added for resuspending cells. This step was repeated further two times. Final 15 ml cultures were started in 100 ml shaking flasks at an OD of 0.05 and incubated for 5 h under shaking of 220 rpm and 30°C. Then, cultures were transferred to 42°C for further incubation. The OD_600_ was measured regularly.

##### 96‐well cultivation and sampling for metabolomics by flow‐injection mass spectrometry

Five hundred microliter of LB in 2 ml deep‐well plates (96‐well) was inoculated from cryo stocks, covered with Breathe‐Easy (Diversified Biotech BEM‐1) adhesive membrane, and incubated for 6 h at 30°C under shaking at 220 rpm. A 495 μl M9 (5 g/l glucose) was inoculated with 5 μl of the LB precultures and incubated for 24 h at 30°C in 2 ml deep‐well plates, under shaking of 220 rpm. One hundred microliter M9 precultures were transferred to 900 μl of fresh M9 (5 g/l glucose) in 2 ml deep‐well plates and incubated for 16 h at 42°C, under shaking of 220 rpm. A 850 μl of the liquid culture was centrifuged in 2 ml deep‐well plates for 15 min at 3,200 *g* at 4°C. The supernatant was removed, and the cell pellets were stored at −80°C. One hundred microliter of −20°C cold 40:40:20 acetonitrile:methanol:water was added to the frozen cell pellets and incubated for 4 h at −20°C. The plate was vortexed and 80 μl of the cell extract transferred to v‐bottomed 96‐well storage plates. The cell extracts were stored at −80°C until further analysis by FI‐MS.

##### Metabolic valve experiments with MetA^F285W^ and ThrB^F267D^

Five milliliter LB cultures were started from cryo stock. After ca. 6 h at 30°C under shaking at 220 rpm, 40 ml M9 (5 g/l glucose) overnight cultures (30°C, 220 rpm) were started using 200 μl of the LB culture for inoculation. Overnight M9 cultures were washed: The cultures were pelletized by 5‐min centrifugation at 3,200 *g* and 40°C. After removing the supernatant, 30 ml of fresh M9 glucose medium was added for resuspending cells. This step was repeated further two times. Final 80 ml cultures were started at an OD of 0.05 and split up to five 15 ml cultures in 100 ml shaking flasks. Each of the five 15 ml cultures was incubated at a different temperature (30/34/37/39/43°C) for ca. 6 h under shaking of 220 rpm. At the start of the cultivation and hourly throughout the cultivation, the OD_600_ was measured. At the start of the cultivation and after 3 and 6 h, whole culture broth samples for LC–MS/MS analysis were taken.

##### Two‐stage production experiments (24 h)

Five milliliter LB cultures were started from cryo stock. After ca. 6 h at 30°C under shaking at 220 rpm, 5 ml M9 (5 g/l glucose) overnight cultures (30°C, 220 rpm) were started using 25 μl of the LB culture for inoculation. Overnight M9 cultures were washed: The cultures were pelletized by 5 min of centrifugation at 3,200 *g* and 40°C. After removing the supernatant, 5 ml of fresh M9 glucose medium was added for resuspending cells. This step was repeated further two times. Final 15 ml cultures were started in 100 ml shaking flasks at an OD of 0.05 and incubated for 24 h under shaking of 220 rpm and 42°C. At the start of the cultivation and after 2‐, 4‐, 6‐, and 24‐h incubation, the OD_600_ was measured, and whole culture broth samples for LC–MS/MS analysis were taken.

##### Arginine overproduction experiment with a DnaX^L289Q^ mutant

Ten milliliter LB cultures were started from cryo stock. After ca. 6 h at 30°C under shaking at 220 rpm, 10 ml M9 (5 g/l glucose) overnight cultures (30°C, 220 rpm) were started using 50 μl of the LB culture for inoculation. Overnight M9 cultures were washed: the cultures were pelletized by 5‐min centrifugation at 3,200 *g* and 40°C. After removing the supernatant, 10 ml of fresh M9 glucose medium was added for resuspending cells. This step was repeated an additional two times. The final 50 ml cultures were started in 500 ml shaking flasks at an OD of 0.05 and incubated for 24 h under shaking of 220 rpm and 42°C. At the start of the cultivation and after 2‐, 4‐, 6‐, 23‐, and 24‐h incubation, the OD_600_ was measured, and whole culture broth samples for LC–MS/MS analysis were taken.

#### Sample processing for NGS

Using 3 ng total plasmid DNA, a plasmid part covering the homology sequences and protospacers was amplified (15 cycles) using two primers suited for further indexing PCRs (forward: “TCGTCGGCAGCGTCAGATGTGTATAAGAGACAGGTATCACGAGGCAGATCCTCTG,” reverse: “GTCTCGTGGGCTCGGAGATGTGTATAAGAGACAGACTCGGTGCCACTTTTTCAAGTT”). Amplicons were purified by AMPure XP PCR beads (Beckman Coulter, #A63881). Using standard Illumina indexing primers, amplicons were indexed in a second PCR and again purified by bead clean‐up. Amplicons were pooled and sequenced on an Illumina NextSeq500 (paired‐end, NextSeq™ 500 Mid Output Kit v2.5, #20024908, 300 cycles). Two cartridges were required to yield the desired sequencing depth of around 4 million reads per sample.

#### NGS data analysis

Demultiplexed paired‐end reads were aligned, merged (based on overlapping sequences), and trimmed to the region of interest using a custom Matlab script. The resulting processed reads were mapped against the designed sequences of the library. For each library member, the number of matching reads was counted. Only reads that shared a 100% identity with a designed sequence were counted since mutations could indicate a malfunction of the CRISPR‐Cas9 genome editing system with no genomic edit. Fitness scores were calculated by normalizing the read count of an individual mutant to the total number of reads of a sample and by subsequently normalizing the data to the first sample of the experiment (*t* = 0 h). Using the fitness scores, the area under the curve (AUC) was determined for the 30 and 42°C time series of each mutant (i). An error e was estimated using the fitness scores $\bar{n}$ for each replicate (A and B) and time point t normalized to the mean fitness scores:

$$e_{i}=\sum_{t}\frac{\left|{\bar{n}}_{i,A,t,30{}^{\circ}C}-{\bar{n}}_{i,B,t,30{}^{\circ}C}\right|}{\frac{{\bar{n}}_{i,A,t,30{}^{\circ}C}+{\bar{n}}_{i,B,t,30{}^{\circ}C}}{2}}+\frac{\left|{\bar{n}}_{i,A,t,42{}^{\circ}C}-{\bar{n}}_{i,B,t,42{}^{\circ}C}\right|}{\frac{{\bar{n}}_{i,A,t,42{}^{\circ}C}+{\bar{n}}_{i,B,t,42{}^{\circ}C}}{2}}$$

#### Scoring temperature sensitivity

Only mutants were considered that met the following criteria:

- They had at least an average of 15 reads at time point zero (${\bar{r}}_{t=0h}$).
- The mean fitness score of the last sample at 30°C (${\bar{n}}_{i,t=12h,30{}^{\circ}C}$) was greater than 0.3.
- The mean fitness score of the last sample at 42°C (${\bar{n}}_{i,t=12h,42{}^{\circ}C}$) was lower than 0.4.
- The error $e_{i}$ was lower than 15.
- The area under the curve for the 30°C time series (${\mathrm{AUC}}_{i,30{}^{\circ}C}$) was greater than 5.
- The mean fitness score of the last samples for the different temperatures fulfilled the following criterion:
$${\bar{n}}_{i,t=12h,30{}^{\circ}C}>\frac{1}{\left(1+{\left(\frac{0.25}{{\bar{n}}_{i,t=12h,42{}^{\circ}C}}\right)}^{3}\right)}+0.3$$

For each gene, the putative temperature‐sensitive mutants were sorted by the number of reads at time point zero$\;{\bar{r}}_{t=0h}$, the relative area under the curve difference ${\text{diff}}_{\mathrm{AUC}}=\frac{{\mathrm{AUC}}_{30{}^{\circ}C}-{\mathrm{AUC}}_{42{}^{\circ}C}}{{\mathrm{AUC}}_{42{}^{\circ}C}}$, the difference between the fitness scores at 30 and 42°C at the last time point (${\text{diff}}_{t=12}$), and the error e. Based on placement in the sortings (rank), a score was calculated for each candidate i:

$${\text{score}}_{i}={\mathrm{rank}}_{i,{\bar{n}}_{t=0h}}\cdot 1.25+{\mathrm{rank}}_{i,{\text{diff}}_{\mathrm{AUC}}}\cdot 2+{\mathrm{rank}}_{i,{\text{diff}}_{t=12h}}\cdot 1.5+{\mathrm{rank}}_{i,e}\cdot 0.75$$

For each gene, the putative temperature‐sensitive mutant with the lowest score was selected for a new library with a total of 250 mutants.

#### Metabolomics

##### Flow‐injection mass spectrometry (FI‐MS)

Flow‐injection mass spectrometry (FI‐MS) was performed as described before (Fuhrer *et al*, 2011; Farke *et al*, 2023). An Agilent 6546 QTOF mass spectrometer (Agilent Technologies, Santa Clara, USA) was used to analyze metabolite levels in metabolite extracts. The source parameters were as follows: source gas 225°C, flow rate of the drying gas 11 l/min, nebulizer pressure 20 psi, sheath gas temperature 350°C, sheath gas flow 10 l/min, and nozzle voltage 2,000 V. Spectra in a 50–1,100 m/z range were acquired in 10 Ghz mode with an acquisition rate of 1.4 spectra/s. The mobile phase was 10 mM (NH_4_)_2_CO_3_, 0.04% NH_4_OH, 60:40 Isopropanol:H_2_O. The reference masses for online mass calibration in negative mode were 59.050 Da (C_3_H_8_O, Isopropanol) and 1033.988 Da (C_18_H_18_F_24_N_3_O_6_P_3_, HP‐921); in positive mode, 121.050 Da (C_5_H_4_N_4_, Purine) and 922.009 Da (C_18_H_18_F_24_N_3_O_6_P_3_, HP‐921).

##### FI‐MS data analysis

Raw data files were converted into “.mzXML” files by MSConvert (Chambers *et al*, 2012). Following data analysis was performed by custom MATLAB scripts that utilized MATLAB functions (The MathWorks, Inc., Massachusetts, USA). The 10 spectra with the highest signal in the total ion count (TIC) were summed. Peaks with a minimum peak height of 1,000 units and a peak prominence of 500 units were selected, and annotated with a 3 mDa tolerance by matching monoisotopic masses of metabolites with a single proton loss for negative mode and single proton gain in positive mode. Double annotations (positive and negative mode) were manually cured based on peak shape and height. For each metabolite, the maximum height of the annotated peak was taken for further analysis. Mean values (*x*) and standard deviations (*σ*_*x*_) were calculated for each sample (*i*) with the maximum peak heights. The data were then normalized to the control strain and converted into log_2_ space:

$$x_{i,\mathrm{norm}}=\log_{2}\left(\frac{x_{i}}{x_{\text{control}}}\right)$$

Subsequently, modified z‐scores were calculated as follows:

$$\text{modified}\;z-{\text{score}}_{i}=\frac{0.6745\cdot \left(x_{i,\mathrm{norm}}-\text{median}\left(x_{i,\mathrm{norm}}\right)\right)}{\text{median}\left(\left\|x_{i,\mathrm{norm}}-\text{median}\left(x_{i,\mathrm{norm}}\right)\right\|\right)}$$

The standard deviations of the modified z‐score values were calculated by error propagation using *σ*_*x*_.

##### Targeted metabolomics by LC–MS/MS

Whole culture broth samples were taken by transferring 100 μl of the culture broth to −20°C cold 50:50 acetonitrile:methanol in 1.5 ml reaction tubes. The samples were stored at −80°C until further processing. The samples were centrifugated for 15 min at 17,000 *g* and −9°C. Metabolite concentrations in the supernatant were analyzed by an isotope‐ratio‐based LC–MS/MS method (Guder *et al*, 2017). Changes to the LC parameters were: in the initial 0.3 min, the analyte was discarded into the waste. Between 0.3 and 2.0 min the analyte was injected to the ESI. 2.0 to 2.3 min the analyte was discarded. An internal, fully ^13^C‐labeled standard was calibrated with authentic ^12^C‐metabolite standards. Based on the calibrated ^13^C‐standard and isotope ratios, absolute metabolite concentrations in the samples were calculated. Homoserine and threonine could not be distinguished. We used an authentic homoserine standard to calculate absolute concentrations.

## Author contributions

**Thorben Schramm:** Conceptualization; resources; data curation; software; formal analysis; investigation; visualization; methodology; writing – original draft; writing – review and editing. **Paul Lubrano:** Investigation. **Vanessa Pahl:** Investigation. **Amelie Stadelmann:** Investigation. **Andreas Verhuelsdonk:** Investigation. **Hannes Link:** Conceptualization; supervision; funding acquisition; investigation; visualization; methodology; writing – original draft; project administration; writing – review and editing.

## Disclosure and competing interests statement

The authors declare that they have no conflict of interest. Open Access funding enabled and organized by Projekt DEAL.

## Supporting information



Appendix S1

Expanded View Figures PDF

Dataset EV1

Dataset EV2

Dataset EV3

Dataset EV4

Dataset EV5

Dataset EV6

Dataset EV7

Dataset EV8

Dataset EV9

Dataset EV10

Dataset EV11

PDF+

Source Data for Figure 1

Source Data for Figure 2

Source Data for Figure 3

Source Data for Figure 4

Source Data for Figure 5

## Data Availability

Illumina sequencing data are provided on the EMBL‐EBI European Nucleotide Archive (ENA) online repository: PRJEB64015 (https://www.ebi.ac.uk/ena/browser/view/PRJEB64015). Metabolomics data are provided on the MassIVE repository: MassIVE MSV000092437 (https://massive.ucsd.edu/ProteoSAFe/FindDatasets?query=MSV000092437).

## References

[msb202311596-bib-0068] Anglada-Girotto M , Handschin G , Ortmayr K , Campos AI , Gillet L , Manfredi P , Mulholland CV , Berney M , Jenal U , Picotti P *et al* (2022) Combining CRISPRi and metabolomics for functional annotation of compound libraries. Nat Chem Biol 18: 482–491 35194207 10.1038/s41589-022-00970-3 PMC7612681

[msb202311596-bib-0001] Bae S , Park J , Kim J‐S (2014) Cas‐OFFinder: a fast and versatile algorithm that searches for potential off‐target sites of Cas9 RNA‐guided endonucleases. Bioinformatics 30: 1473–1475 24463181 10.1093/bioinformatics/btu048 PMC4016707

[msb202311596-bib-0002] Ben‐Aroya S , Coombes C , Kwok T , O'Donnell KA , Boeke JD , Hieter P (2008) Toward a comprehensive temperature‐sensitive mutant repository of the essential genes of *Saccharomyces cerevisiae* . Mol Cell 30: 248–258 18439903 10.1016/j.molcel.2008.02.021 PMC4130347

[msb202311596-bib-0003] Berlyn MKB (1999) CGSC: the *E.coli* genetic stock center database. In Bioinformatics: databases and systems, Letovsky S (ed), pp 175–183. Boston, MA: Springer US

[msb202311596-bib-0004] Blinkova A , Hervas C , Stukenberg PT , Onrust R , O'Donnell ME , Walker JR (1993) The *Escherichia coli* DNA polymerase III holoenzyme contains both products of the dnaX gene, tau and gamma, but only tau is essential. J Bacteriol 175: 6018–6027 8376347 10.1128/jb.175.18.6018-6027.1993 PMC206684

[msb202311596-bib-0005] Burg JM , Cooper CB , Ye Z , Reed BR , Moreb EA , Lynch MD (2016) Large‐scale bioprocess competitiveness: the potential of dynamic metabolic control in two‐stage fermentations. Curr Opin Chem Eng 14: 121–136

[msb202311596-bib-0006] Burgard AP , Pharkya P , Maranas CD (2003) Optknock: a bilevel programming framework for identifying gene knockout strategies for microbial strain optimization. Biotechnol Bioeng 84: 647–657 14595777 10.1002/bit.10803

[msb202311596-bib-0007] Bush K , Bradford PA (2016) β‐lactams and β‐lactamase inhibitors: an overview. Cold Spring Harb Perspect Med 6: a025247 27329032 10.1101/cshperspect.a025247 PMC4968164

[msb202311596-bib-0008] Chambers MC , Maclean B , Burke R , Amodei D , Ruderman DL , Neumann S , Gatto L , Fischer B , Pratt B , Egertson J *et al* (2012) A cross‐platform toolkit for mass spectrometry and proteomics. Nat Biotechnol 30: 918–920 23051804 10.1038/nbt.2377 PMC3471674

[msb202311596-bib-0009] Cheng YS , Rudolph J , Stern M , Stubbe J , Flannigan KA , Smith JM (1990) Glycinamide ribonucleotide synthetase from *Escherichia coli*: cloning, overproduction, sequencing, isolation, and characterization. Biochemistry 29: 218–227 2182115 10.1021/bi00453a030

[msb202311596-bib-0010] Cho H‐S , Seo SW , Kim YM , Jung GY , Park JM (2012) Engineering glyceraldehyde‐3‐phosphate dehydrogenase for switching control of glycolysis in *Escherichia coli* . Biotechnol Bioeng 109: 2612–2619 22528318 10.1002/bit.24532

[msb202311596-bib-0011] Costanzo M , VanderSluis B , Koch EN , Baryshnikova A , Pons C , Tan G , Wang W , Usaj M , Hanchard J , Lee SD *et al* (2016) A global genetic interaction network maps a wiring diagram of cellular function. Science 353: aaf1420 27708008 10.1126/science.aaf1420 PMC5661885

[msb202311596-bib-0069] Datsenko KA , Wanner BL (2000) One-step inactivation of chromosomal genes in *Escherichia coli* K-12 using PCR products. Proc Natl Acad Sci 97: 6640–6645 10829079 10.1073/pnas.120163297 PMC18686

[msb202311596-bib-0012] Dewachter L , Brooks AN , Noon K , Cialek C , Clark‐ElSayed A , Schalck T , Krishnamurthy N , Versées W , Vranken W , Michiels J (2023) Deep mutational scanning of essential bacterial proteins can guide antibiotic development. Nat Commun 14: 241 36646716 10.1038/s41467-023-35940-3 PMC9842644

[msb202311596-bib-0013] Donati S , Kuntz M , Pahl V , Farke N , Beuter D , Glatter T , Gomes‐Filho JV , Randau L , Wang C‐Y , Link H (2021) Multi‐omics analysis of CRISPRi‐knockdowns identifies mechanisms that buffer decreases of enzymes in *E. coli* metabolism. Cell Syst 12: 56–67.e6 33238135 10.1016/j.cels.2020.10.011

[msb202311596-bib-0014] Farke N , Schramm T , Verhülsdonk A , Rapp J , Link H (2023) Systematic analysis of in‐source modifications of primary metabolites during flow‐injection time‐of‐flight mass spectrometry. Anal Biochem 664: 115036 36627043 10.1016/j.ab.2023.115036 PMC9902335

[msb202311596-bib-0015] Fendt S‐M , Buescher JM , Rudroff F , Picotti P , Zamboni N , Sauer U (2010) Tradeoff between enzyme and metabolite efficiency maintains metabolic homeostasis upon perturbations in enzyme capacity. Mol Syst Biol 6: 356 20393576 10.1038/msb.2010.11 PMC2872607

[msb202311596-bib-0016] Fuhrer T , Heer D , Begemann B , Zamboni N (2011) High‐throughput, accurate mass metabolome profiling of cellular extracts by flow injection–time‐of‐flight mass spectrometry. Anal Chem 83: 7074–7080 21830798 10.1021/ac201267k

[msb202311596-bib-0017] Fuhrer T , Zampieri M , Sévin DC , Sauer U , Zamboni N (2017) Genomewide landscape of gene–metabolome associations in *Escherichia coli* . Mol Syst Biol 13: 907 28093455 10.15252/msb.20167150 PMC5293155

[msb202311596-bib-0018] García‐Nafría J , Watson JF , Greger IH (2016) IVA cloning: a single‐tube universal cloning system exploiting bacterial *In Vivo* Assembly. Sci Rep 6: 27459 27264908 10.1038/srep27459 PMC4893743

[msb202311596-bib-0019] Garst AD , Bassalo MC , Pines G , Lynch SA , Halweg‐Edwards AL , Liu R , Liang L , Wang Z , Zeitoun R , Alexander WG *et al* (2017) Genome‐wide mapping of mutations at single‐nucleotide resolution for protein, metabolic and genome engineering. Nat Biotechnol 35: 48–55 27941803 10.1038/nbt.3718

[msb202311596-bib-0020] Georgescu RE , Kim S‐S , Yurieva O , Kuriyan J , Kong X‐P , O'Donnell M (2008) Structure of a sliding clamp on DNA. Cell 132: 43–54 18191219 10.1016/j.cell.2007.11.045 PMC2443641

[msb202311596-bib-0021] Goodall ECA , Robinson A , Johnston IG , Jabbari S , Turner KA , Cunningham AF , Lund PA , Cole JA , Henderson IR (2018) The essential genome of *Escherichia coli* K‐12. mBio 9: e02096‐17 29463657 10.1128/mBio.02096-17 PMC5821084

[msb202311596-bib-0022] Grisolia V , Carlomagno MS , Nappo AG , Bruni CB (1985) Cloning, structure, and expression of the Escherichia coli K‐12 hisC gene. J Bacteriol 164: 1317–1323 2999081 10.1128/jb.164.3.1317-1323.1985 PMC219332

[msb202311596-bib-0023] Guder JC , Schramm T , Sander T , Link H (2017) Time‐optimized isotope ratio LC–MS/MS for high‐throughput quantification of primary metabolites. Anal Chem 89: 1624–1631 28050903 10.1021/acs.analchem.6b03731

[msb202311596-bib-0024] Haase‐Pettingell C , King J (1997) Prevalence of temperature sensitive folding mutations in the parallel beta coil domain of the phage P22 tailspike endorhamnosidase. J Mol Biol 267: 88–102 9096209 10.1006/jmbi.1996.0841

[msb202311596-bib-0025] Hansen FG , Atlung T (2018) The DnaA tale. Front Microbiol 9: 319 29541066 10.3389/fmicb.2018.00319 PMC5835720

[msb202311596-bib-0026] Harder B‐J , Bettenbrock K , Klamt S (2018) Temperature‐dependent dynamic control of the TCA cycle increases volumetric productivity of itaconic acid production by *Escherichia coli* . Biotechnol Bioeng 115: 156–164 28865130 10.1002/bit.26446 PMC5725713

[msb202311596-bib-0027] Izard J , Gomez Balderas CD , Ropers D , Lacour S , Song X , Yang Y , Lindner AB , Geiselmann J , de Jong H (2015) A synthetic growth switch based on controlled expression of RNA polymerase. Mol Syst Biol 11: 840 26596932 10.15252/msb.20156382 PMC4670729

[msb202311596-bib-0028] Jang WD , Kim GB , Lee SY (2023) An interactive metabolic map of bio‐based chemicals. Trends Biotechnol 41: 10–14 35961799 10.1016/j.tibtech.2022.07.013

[msb202311596-bib-0029] Jones CE , Brook JM , Buck D , Abell C , Smith AG (1993) Cloning and sequencing of the *Escherichia coli* panB gene, which encodes ketopantoate hydroxymethyltransferase, and overexpression of the enzyme. J Bacteriol 175: 2125–2130 8096212 10.1128/jb.175.7.2125-2130.1993 PMC204323

[msb202311596-bib-0030] Kasari M , Kasari V , Kärmas M , Jõers A (2022) Decoupling growth and production by removing the origin of replication from a bacterial chromosome. ACS Synth Biol 11: 2610–2622 35798328 10.1021/acssynbio.1c00618 PMC9397407

[msb202311596-bib-0031] Kemmeren P , Sameith K , van de Pasch LAL , Benschop JJ , Lenstra TL , Margaritis T , O'Duibhir E , Apweiler E , van Wageningen S , Ko CW *et al* (2014) Large‐scale genetic perturbations reveal regulatory networks and an abundance of gene‐specific repressors. Cell 157: 740–752 24766815 10.1016/j.cell.2014.02.054

[msb202311596-bib-0032] Kofoed M , Milbury KL , Chiang JH , Sinha S , Ben‐Aroya S , Giaever G , Nislow C , Hieter P , Stirling PC (2015) An updated collection of sequence barcoded temperature‐sensitive alleles of yeast essential genes. G3 5: 1879–1887 26175450 10.1534/g3.115.019174 PMC4555224

[msb202311596-bib-0033] Kyte J , Doolittle RF (1982) A simple method for displaying the hydropathic character of a protein. J Mol Biol 157: 105–132 7108955 10.1016/0022-2836(82)90515-0

[msb202311596-bib-0070] Larson MH , Gilbert LA , Wang X , Lim WA , Weissman JS , Qi LS (2013) CRISPR interference (CRISPRi) for sequence-specific control of gene expression. Nat Protoc 8: 2180–2196 24136345 10.1038/nprot.2013.132 PMC3922765

[msb202311596-bib-0034] Li Z , Vizeacoumar FJ , Bahr S , Li J , Warringer J , Vizeacoumar FS , Min R , VanderSluis B , Bellay J , DeVit M *et al* (2011) Systematic exploration of essential yeast gene function with temperature‐sensitive mutants. Nat Biotechnol 29: 361–367 21441928 10.1038/nbt.1832 PMC3286520

[msb202311596-bib-0035] Li S , Jendresen CB , Grünberger A , Ronda C , Jensen SI , Noack S , Nielsen AT (2016) Enhanced protein and biochemical production using CRISPRi‐based growth switches. Metab Eng 38: 274–284 27647432 10.1016/j.ymben.2016.09.003

[msb202311596-bib-0036] Li S , Jendresen CB , Landberg J , Pedersen LE , Sonnenschein N , Jensen SI , Nielsen AT (2020) Genome‐wide CRISPRi‐based identification of targets for decoupling growth from production. ACS Synth Biol 9: 1030–1040 32268068 10.1021/acssynbio.9b00143

[msb202311596-bib-0037] Liang L , Liu R , Garst AD , Lee T , Nogué VSI , Beckham GT , Gill RT (2017) CRISPR EnAbled Trackable genome engineering for isopropanol production in *Escherichia coli* . Metab Eng 41: 1–10 28216108 10.1016/j.ymben.2017.02.009

[msb202311596-bib-0038] Lovato TL , Adams MM , Baker PW , Cripps RM (2009) A molecular mechanism of temperature sensitivity for mutations affecting the *Drosophila* muscle regulator myocyte enhancer factor‐2. Genetics 183: 107–117 19564485 10.1534/genetics.109.105056 PMC2746136

[msb202311596-bib-0039] Lynch MD , Gill RT , Lipscomb TEW (2016) Method for producing 3‐hydroxypropionic acid and other products. (https://www.osti.gov/servlets/purl/1261625)

[msb202311596-bib-0040] Lynch M , Louie M , Copley S , Spindler E , Prather B , Lipscomb M , Lipscomb T , Liao H , Hogsett D , Evans R (2019) Microorganisms and methods for the production of fatty acids and fatty acid derived products. (https://www.osti.gov/servlets/purl/1600243)

[msb202311596-bib-0041] Meier G , Thavarasah S , Ehrenbolger K , Hutter CAJ , Hürlimann LM , Barandun J , Seeger MA (2023) Deep mutational scan of a drug efflux pump reveals its structure–function landscape. Nat Chem Biol 19: 440–450 36443574 10.1038/s41589-022-01205-1 PMC7615509

[msb202311596-bib-0042] Monk JM , Koza A , Campodonico MA , Machado D , Seoane JM , Palsson BO , Herrgård MJ , Feist AM (2016) Multi‐omics quantification of species variation of *Escherichia coli* links molecular features with strain phenotypes. Cell Syst 3: 238–251.e12 27667363 10.1016/j.cels.2016.08.013 PMC5058344

[msb202311596-bib-0043] Mueller EJ , Meyer E , Rudolph J , Davisson VJ , Stubbe J (1994) N5‐carboxyaminoimidazole ribonucleotide: evidence for a new intermediate and two new enzymic activities in the de novo purine biosynthetic pathway of *Escherichia coli* . Biochemistry 33: 2269–2278 8117684 10.1021/bi00174a038

[msb202311596-bib-0044] Mülleder M , Calvani E , Alam MT , Wang RK , Eckerstorfer F , Zelezniak A , Ralser M (2016) Functional metabolomics describes the yeast biosynthetic regulome. Cell 167: 553–565.e12 27693354 10.1016/j.cell.2016.09.007 PMC5055083

[msb202311596-bib-0045] Na D , Yoo SM , Chung H , Park H , Park JH , Lee SY (2013) Metabolic engineering of *Escherichia coli* using synthetic small regulatory RNAs. Nat Biotechnol 31: 170–174 23334451 10.1038/nbt.2461

[msb202311596-bib-0046] Osorio D , Rondón‐Villarreal P , Torres R (2015) Peptides: a package for data mining of antimicrobial peptides. R J 7: 4

[msb202311596-bib-0047] Patrick WM , Quandt EM , Swartzlander DB , Matsumura I (2007) Multicopy suppression underpins metabolic evolvability. Mol Biol Evol 24: 2716–2722 17884825 10.1093/molbev/msm204 PMC2678898

[msb202311596-bib-0048] Piraner DI , Abedi MH , Moser BA , Lee‐Gosselin A , Shapiro MG (2017) Tunable thermal bioswitches for *in vivo* control of microbial therapeutics. Nat Chem Biol 13: 75–80 27842069 10.1038/nchembio.2233

[msb202311596-bib-0049] Plaza del Pino IM , Ibarra‐Molero B , Sanchez‐Ruiz JM (2000) Lower kinetic limit to protein thermal stability: a proposal regarding protein stability *in vivo* and its relation with misfolding diseases. Proteins 40: 58–70 10813831 10.1002/(sici)1097-0134(20000701)40:1<58::aid-prot80>3.0.co;2-m

[msb202311596-bib-0050] Qi LS , Larson MH , Gilbert LA , Doudna JA , Weissman JS , Arkin AP , Lim WA (2013) Repurposing CRISPR as an RNA‐guided platform for sequence‐specific control of gene expression. Cell 152: 1173–1183 23452860 10.1016/j.cell.2013.02.022 PMC3664290

[msb202311596-bib-0051] Rousset F , Cui L , Siouve E , Becavin C , Depardieu F , Bikard D (2018) Genome‐wide CRISPR‐dCas9 screens in *E. coli* identify essential genes and phage host factors. PLoS Genet 14: e1007749 30403660 10.1371/journal.pgen.1007749 PMC6242692

[msb202311596-bib-0052] Sadler JR , Novick A (1965) The properties of repressor and the kinetics of its action. J Mol Biol 12: 305–327 14337495 10.1016/s0022-2836(65)80255-8

[msb202311596-bib-0053] Saluja D , Godson GN (1995) Biochemical characterization of *Escherichia coli* temperature‐sensitive dnaB mutants dnaB8, dnaB252, dnaB70, dnaB43, and dnaB454. J Bacteriol 177: 1104–1111 7532169 10.1128/jb.177.4.1104-1111.1995 PMC176710

[msb202311596-bib-0054] Sander T , Wang CY , Glatter T , Link H (2019) CRISPRi‐based downregulation of transcriptional feedback improves growth and metabolism of arginine overproducing *E. coli* . ACS Synth Biol 8: 1983–1990 31429546 10.1021/acssynbio.9b00183

[msb202311596-bib-0055] Santos‐Moreno J , Tasiudi E , Stelling J , Schaerli Y (2020) Multistable and dynamic CRISPRi‐based synthetic circuits. Nat Commun 11: 2746 32488086 10.1038/s41467-020-16574-1 PMC7265303

[msb202311596-bib-0056] Schmidt A , Kochanowski K , Vedelaar S , Ahrné E , Volkmer B , Callipo L , Knoops K , Bauer M , Aebersold R , Heinemann M (2016) The quantitative and condition‐dependent *Escherichia coli* proteome. Nat Biotechnol 34: 104–110 26641532 10.1038/nbt.3418 PMC4888949

[msb202311596-bib-0057] Schramm T , Lempp M , Beuter D , Sierra SG , Glatter T , Link H (2020) High‐throughput enrichment of temperature‐sensitive argininosuccinate synthetase for two‐stage citrulline production in *E. coli* . Metab Eng 60: 14–24 32179161 10.1016/j.ymben.2020.03.004 PMC7225747

[msb202311596-bib-0058] Smith CJ , Deutch AH , Rushlow KE (1984) Purification and characteristics of a gamma‐glutamyl kinase involved in *Escherichia coli* proline biosynthesis. J Bacteriol 157: 545–551 6319365 10.1128/jb.157.2.545-551.1984 PMC215281

[msb202311596-bib-0059] Tan KP , Khare S , Varadarajan R , Madhusudhan MS (2014) TSpred: a web server for the rational design of temperature‐sensitive mutants. Nucleic Acids Res 42: W277–W284 24782523 10.1093/nar/gku319 PMC4086094

[msb202311596-bib-0060] The UniProt Consortium (2023) UniProt: the Universal Protein Knowledgebase in 2023. Nucleic Acids Res 51: D523–D531 36408920 10.1093/nar/gkac1052 PMC9825514

[msb202311596-bib-0061] Tong AHY , Lesage G , Bader GD , Ding H , Xu H , Xin X , Young J , Berriz GF , Brost RL , Chang M *et al* (2004) Global mapping of the yeast genetic interaction network. Science 303: 808–813 14764870 10.1126/science.1091317

[msb202311596-bib-0062] Vandewiele D , Fernández de Henestrosa AR , Timms AR , Bridges BA , Woodgate R (2002) Sequence analysis and phenotypes of five temperature sensitive mutator alleles of dnaE, encoding modified α‐catalytic subunits of *Escherichia coli* DNA polymerase III holoenzyme. Mutat Res 499: 85–95 11804607 10.1016/s0027-5107(01)00268-8

[msb202311596-bib-0063] Varadarajan R , Nagarajaram HA , Ramakrishnan C (1996) A procedure for the prediction of temperature‐sensitive mutants of a globular protein based solely on the amino acid sequence. Proc Natl Acad Sci USA 93: 13908–13913 8943034 10.1073/pnas.93.24.13908 PMC19465

[msb202311596-bib-0064] Venayak N , von Kamp A , Klamt S , Mahadevan R (2018) MoVE identifies metabolic valves to switch between phenotypic states. Nat Commun 9: 5332 30552335 10.1038/s41467-018-07719-4 PMC6294006

[msb202311596-bib-0065] Wang X , Han J‐N , Zhang X , Ma Y‐Y , Lin Y , Wang H , Li D‐J , Zheng T‐R , Wu F‐Q , Ye J‐W *et al* (2021) Reversible thermal regulation for bifunctional dynamic control of gene expression in *Escherichia coli* . Nat Commun 12: 1411 33658500 10.1038/s41467-021-21654-x PMC7930084

[msb202311596-bib-0066] Weber W (2003) Conditional human VEGF‐mediated vascularization in chicken embryos using a novel temperature‐inducible gene regulation (TIGR) system. Nucleic Acids Res 31: 69e–669e 10.1093/nar/gng069 PMC162344 12799458

[msb202311596-bib-0067] Williams DH , Bardsley B (1999) The vancomycin group of antibiotics and the fight against resistant bacteria. Angew Chem Int Ed 38: 1172–1193 10.1002/(SICI)1521-3773(19990503)38:9<1172::AID-ANIE1172>3.0.CO;2-C 29711719

