# Supplementary material for: Mapping temperature‐sensitive mutations at a genome scale to engineer growth switches in Escherichia coli
Source: Mol Syst Biol. 2023 Aug 29;19(10):e11596. doi: 10.15252/msb.202311596 (PMC10568205; doi:10.15252/msb.202311596)
Supplement: Supplementary file 1 — Appendix S1 [file MSB-19-e11596-s018.pdf]

Appendix Figures

Mapping temperature-sensitive mutations at a genome-scale to engineer growth-switches in *E. coli*

Thorben Schramm[1,2], Paul Lubrano[1], Vanessa Pahl[1], Amelie Stadelmann[1], Andreas Verhülsdonk[1], Hannes Link[1,*]

[1]Interfaculty Institute of Microbiology and Infection Medicine, University of Tübingen, Auf der Morgenstelle 24, 72076 Tübingen, Germany

[2]Present address: Institute of Molecular Systems Biology, Department of Biology, ETH Zurich, Otto-Stern-Weg 3, 8093 Zürich, Switzerland

*Correspondence: hannes.link@uni-tuebingen.de

Content

Appendix Figure S1 – The CRISPR library composition at different steps of construction.
Appendix Figure S2 – Construction steps of pooled CRISPR strain libraries.
Appendix Figure S3 – Deep sequencing of CRISPR barcodes is reproducible.
Appendix Figure S4 – Cluster analysis of putative temperature-sensitive mutants.
Appendix Figure S5 – Number of alleles per gene*:* all mutants in the library *vs.* putative TS mutants.
Appendix Figure S6 – A scoring system for TS mutations.
Appendix Figure S7 – Abundance of the 94 selected TS mutants in the sub-library and their coverage of functional categories.
Appendix Figure S8 – Growth rates of the unedited and two mock edit control strains.
Appendix Figure S9 – An empirical Arrhenius-type function describes the growth rate/temperature relationship of TS mutants.
Appendix Figure S10 – Categorization of TS mutants based on their growth rate/temperature dependencies.
Appendix Figure S11 – Fitness score dynamics of the TS mutant HisCI336D during the growth competition experiment.
Appendix Figure S12 – Growth in rich medium reveals conditionally auxotrophic TS mutants.
Appendix Figure S13 – The distributions of mod. z-score values and mod. z-score standard deviations.
Appendix Figure S14 – Metabolite increases in the TS mutants GapA^{V17W}, AroC^{V339P}, and PanB^{L42Q}.
Appendix Figure S15– Predicting non-growing strains from their metabolic profile

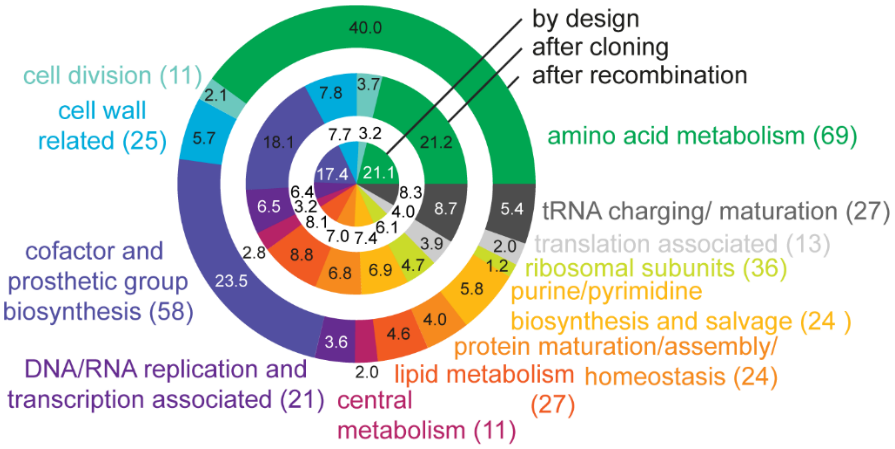

Appendix Figure S1 – The CRISPR library composition at different steps of construction.

The CRISPR library covered 346 essential genes in 12 functional categories. The chart shows the relative share of the 12 categories in the library at different steps in the construction. The inner circle indicates the composition in the original design, the middle circle the composition after cloning of the pooled plasmid library, and the outer circle the composition after inserting the mutations to the genome (recombination step).

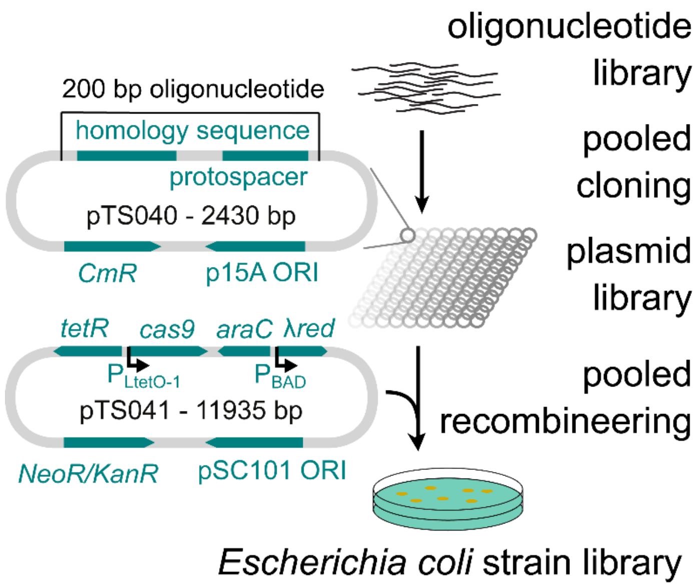

Appendix Figure S2 – Construction steps of pooled CRISPR strain libraries.

Array-synthesized oligonucleotide pools were used to clone pooled plasmid libraries based on the pTS040 plasmid. pTS040 had a p15A origin of replication (ORI) and carried a chloramphenicol resistance gene as well as the homology and guide RNA sequences. In a second step, the pooled plasmid libraries were used for transformation of an *Escherichia coli* strain (BW25113) that already carried the pTS041 plasmid. pTS041 had a pSC101 ORI and carried genes for a kanamycin resistance, the transcriptional repressors *araC* and *tetR*, the *Escherichia virus Lambda red* system, and *Streptococcus pyogenes cas9*. *cas9* was under control of the $P_{LtetO\text{-}1}$ promoter. The *Lambda red* system was under control of the P_{BAD} promoter. After transformation, cells were plated to agar plates. Colonies were collected from the plates and pooled yielding the final CRISPR libraries.

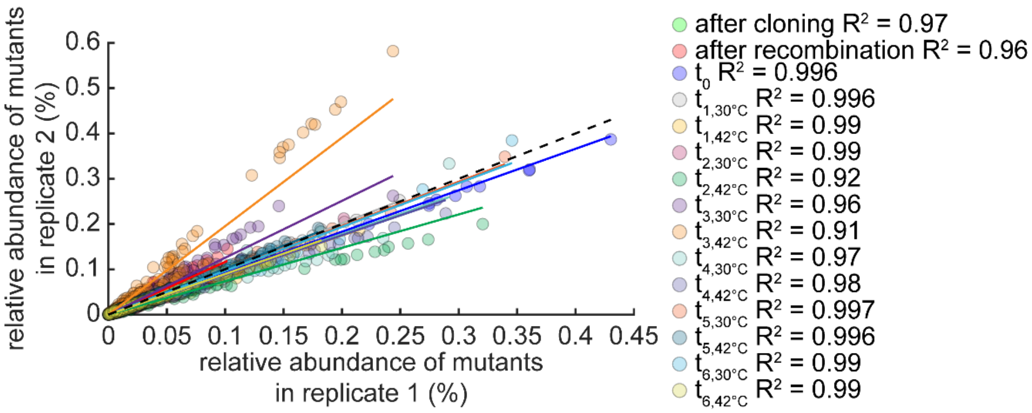

Appendix Figure S3 – Deep sequencing of CRISPR barcodes is reproducible.

The parity plot shows the relative abundance of single strains in the CRISPR library at different steps during construction and during the competitive growth experiment (also see Fig 1). The abundance was measured by next generation amplicon deep sequencing (n = 2). Read counts of single mutants with 100% sequence identity were normalized to the total number of reads to calculate the relative abundance. Lines indicate linear regressions. R^2 is the coefficient of determination.

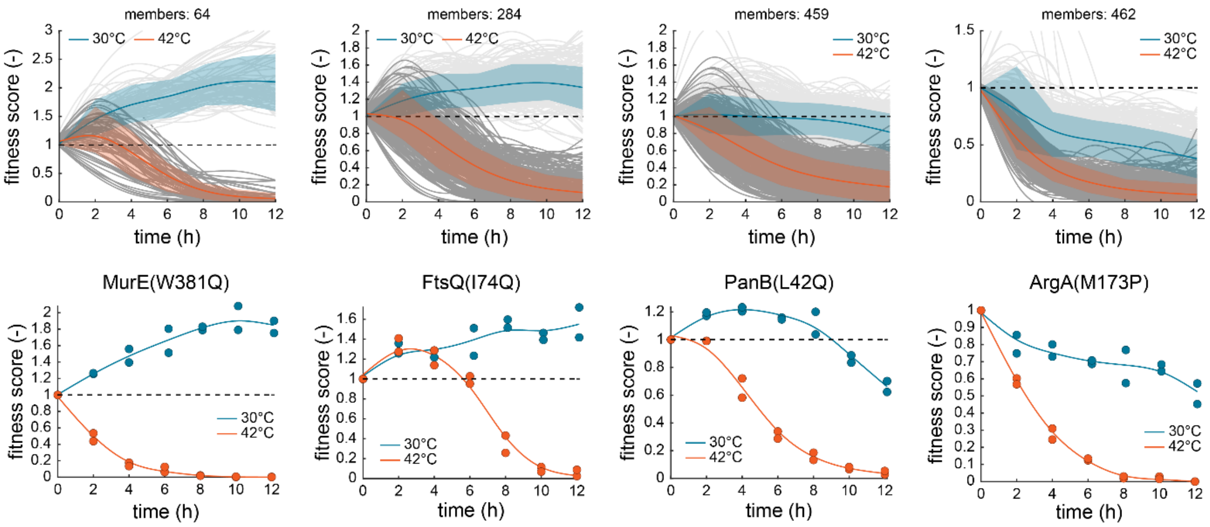

Appendix Figure S4 – Cluster analysis of putative temperature-sensitive mutants.

The upper charts show the fitness scores dynamics at 30 °C and 42 °C for 1,269 putative TS mutants, which are separated into four different clusters. The clusters were identified by k-means clustering. Grey curves are the moving average of the mean of n=2 replicates (light grey: 30°C culture, dark grey: 42°C culture). Colored lines are cluster means and shaded areas their standard deviation (blue: 30 °C, red: 42 °C). The lower charts show the fitness score dynamics of four individual TS mutants, each from the cluster above. Dots show data from n = 2 replicates per temperature. The lines are the moving average through the means (blue: 30°C, red: 42°C).

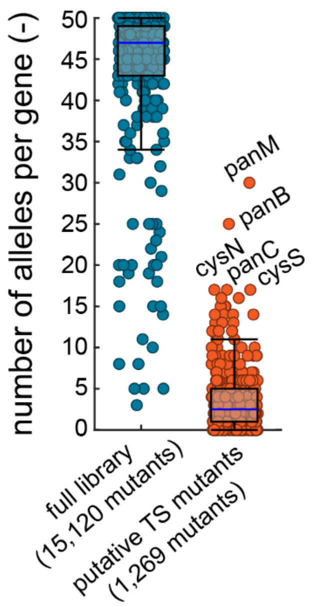

Appendix Figure S5 – Number of alleles per gene: all mutants in the library vs. putative TS mutants.

The dot plot shows the number of alleles per gene for all mutants in the CRISPR library (blue dots) and for the 1,269 putative temperature-sensitive mutants (red dots). The box whisker plots show the median (blue line) and 25th/75th percentiles.

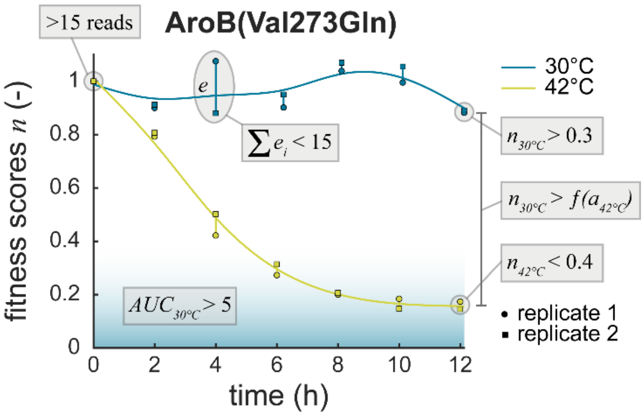

Appendix Figure S6 – A scoring system for TS mutations.

Due to complex dynamics of fitness scores but also to reduce the number of putative TS mutants, we scored temperature-sensitivity of all mutants in the CRISPR library. The chart illustrates six measures important for the scoring (also see M.7). The fitness scores of the $AroB^{Val273Gln}$ during the competitive growth experiment (Fig 1A) are shown as an example. Squares and dots indicate individual replicates (n = 2). Lines are the moving average through means. Fitness scores were calculated as described in M.6. During scoring, mutants were discarded when they failed a set of minimum requirements, as indicated in the figure.

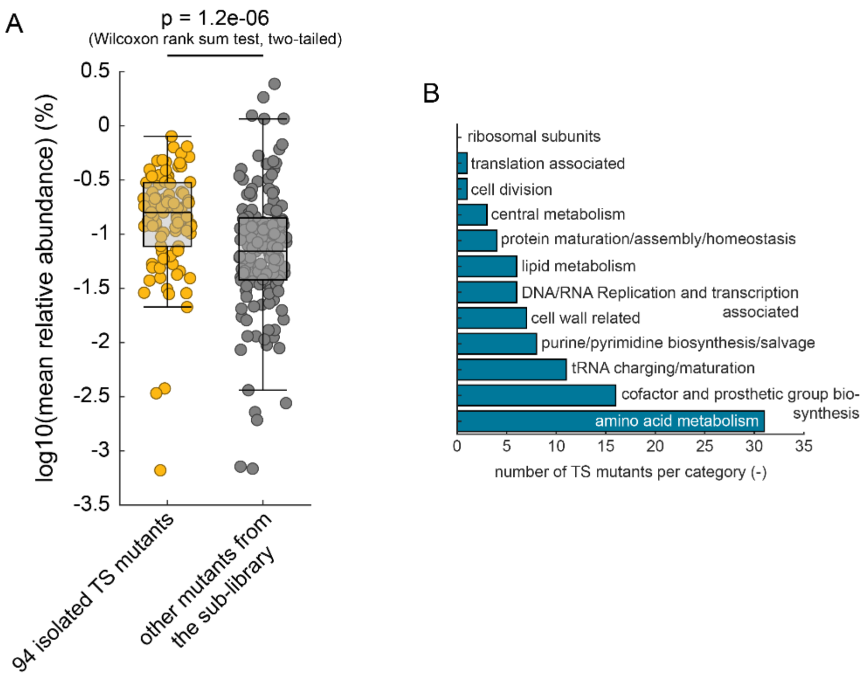

Appendix Figure S7 – Abundance of the 94 selected TS mutants in the sub-library and their coverage of functional categories.

A, The dot plot shows the log10 mean relative abundance of mutants in the sub-library of 250 strains. The data was obtained from deep sequencing (Fig 2A). Yellow dots show the abundance of the selected 94 temperature-sensitive mutants, grey dots show the other 156 mutants in the sub-library. The box whisker plots show the median and 25th/75th percentiles. We used a Wilcoxon rank sum test (two-tailed) to test for differences between the 94 TS and the other 156 mutants (p-value is shown above).

B, The bar plot shows how many of the 94 selected TS mutants belong to each of the functional categories as defined by Dataset EV10.

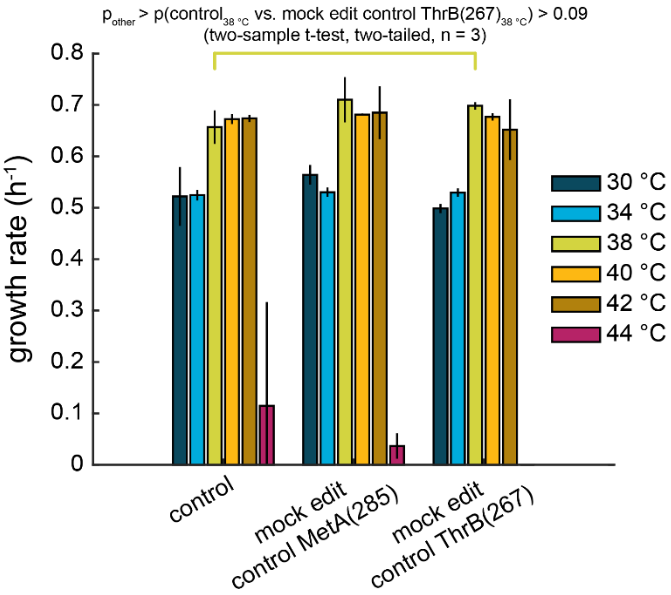

Appendix Figure S8 – Growth rates of the unedited and two mock edit control strains.

The bar plot shows the mean maximum specific growth rates of the unedited control strain and the mock edit controls MetA(285) and ThrB(267) during plate reader cultivations (n = 3) at 6 different temperatures (30°C, 34°C, 38°C, 40°C, 42°C, and 44°C, indicated by different colors). The vertical lines show the standard deviation. The mock edit control strains were constructed by inserting the wildtype sequence and a silent PAM mutation into the genome. A two-sample t-test (two-tailed) was used to compare the growth rates of the mock edit controls with the unedited control strain. The lowest p-value (= 0.098) was observed for the comparison between the mock edit control ThrB(267) and the control strain at 38°C, all other comparisons had higher p-values (also see Dataset EV4 for all p-values).

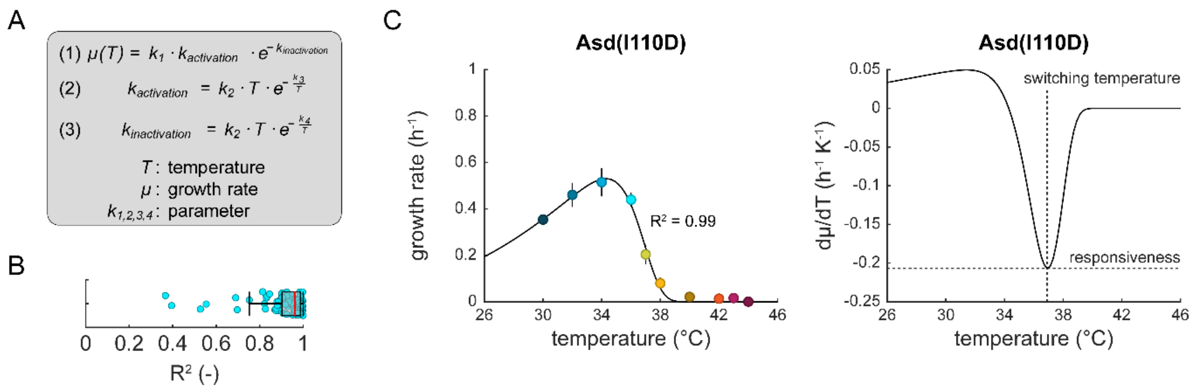

Appendix Figure S9 – An empirical Arrhenius-type function describes the growth rate/temperature relationship of TS mutants.

A, The empirical Arrhenius-type function (1) that was used to describe the maximum specific growth rate μ of *E. coli* strains as a function of the temperature T had an activating term $k_{activation}$ (2) and an inactivating term $k_{inactivation}$ (3). The Arrhenius-type function further had four parameters $k_{1,2,3,4}$ that were fitted to experimental data.

B, The dot plot shows the coefficient of determination R^2 of the Arrhenius-type function in **A** fitted to experimental data of 94 TS mutants and a control strain without mutation. Dots are data of individual strains. The box-whiskers plot indicates the median (red line) and the 25th and 75th percentiles.

C, The left chart shows the maximum growth rate (h^{-1}) of the TS mutant Asd^{I110D} at different temperatures (°C). The data was measured in microtiter plate cultivations (data also shown in Fig EV 2). Dots are the mean, vertical black lines the standard deviation (n = 3). The black line is the fitted Arrhenius-type function of **A**. R^2 is the coefficient of determination. The right chart shows the first derivative (dμ/dT) of the Arrhenius-type function (fitted to Asd^{I110D}). The minimum dμ/dT is the "responsiveness" value that also determines the "switching temperature".

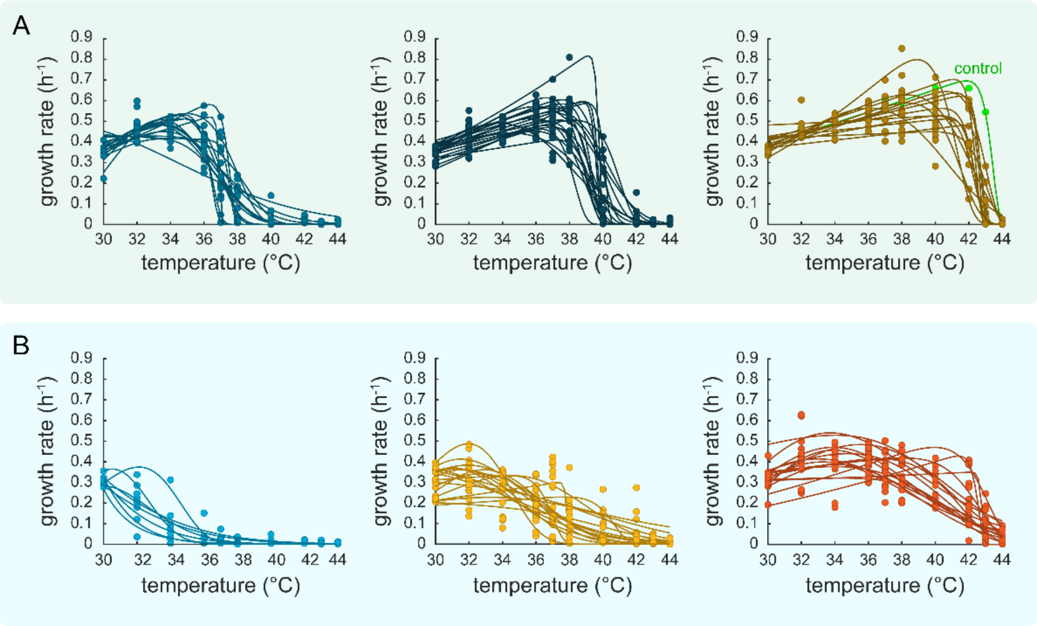

Appendix Figure S10 – Categorization of TS mutants based on their growth rate/temperature dependencies.

The charts in **A** and **B** show the maximum specific growth rates (h^{-1}) of 94 TS mutants and a control strain without mutation at ten different temperatures (°C). Dots are the mean of n = 3 replicates, and the lines were calculated by fitting an Arrhenius-type function to the data (also see Appendix Fig S9). The data is also shown Fig EV3, but here with k-means clustering. The six charts show the individual clusters from this analysis. From left to right, the clusters were centered around different temperatures (low to high).

A, The charts show strains that were categorized as "switch-like", which means that they typically switched from fast growth to no growth within a small temperature range.

B, The charts show TS mutants categorized as "gradually" switching.

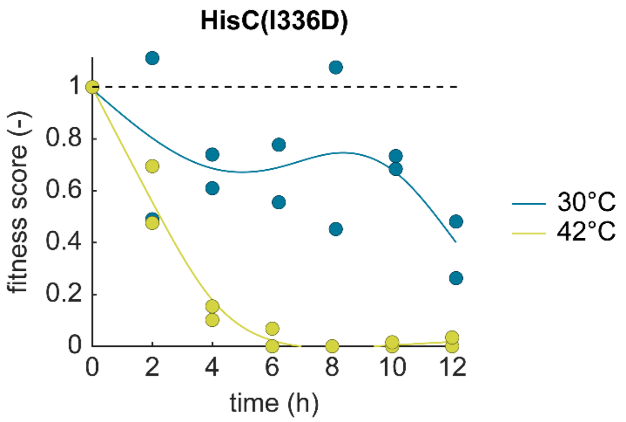

Appendix Figure S11 – Fitness score dynamics of the TS mutant HisC^{I336D} during the growth competition experiment.

The charts show the fitness score dynamics of the HisC^{I336D} during the competitive growth assay (Fig 1A). Dots show data from n = 2 replicates per temperature. The lines are the moving average through the means (blue: 30°C, red: 42°C). Blue color indicates data from the 30°C culture, and yellow shows data from the 42°C culture.

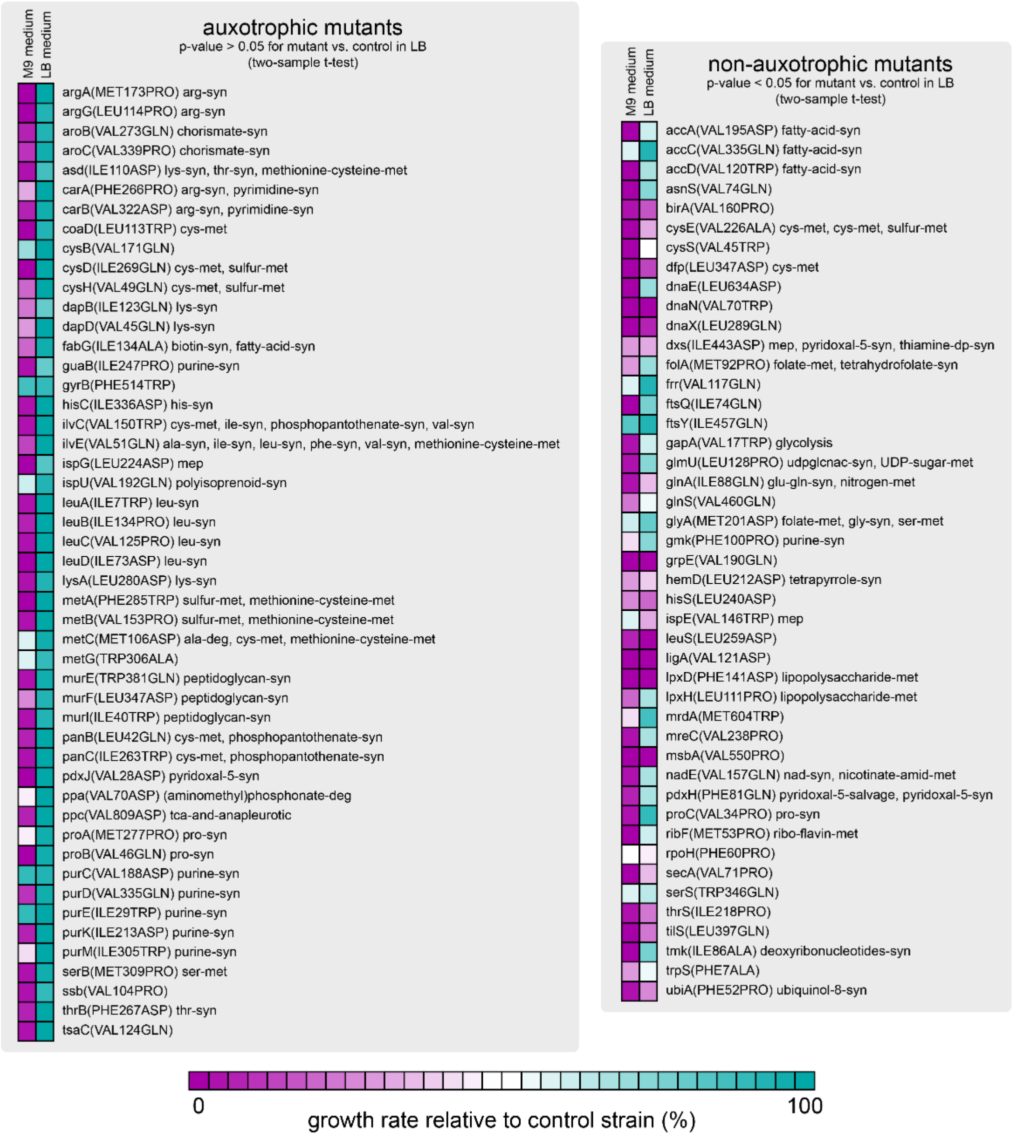

Appendix Figure S12 – Growth in rich medium reveals conditionally auxotrophic TS mutants.

The heatmap shows the mean maximum specific growth rates of the 94 selected TS mutants during plate reader cultivations at 42°C relative to the unedited control strain (n = 3 replicates). The left column in each box shows the relative growth rates for cultivations in minimal M9 medium, and the right column cultivation in rich LB medium. We used two-sample t-tests (two-tailed) to compare each mutant with the unedited control strain (M9 medium: Dataset EV7, LB medium: Dataset EV8) and to determine, which mutant was conditionally auxotrophic: mutants with p-values lower than 0.05 were considered non-auxotrophic (right box, 45 mutants), and the other mutants as conditionally auxotrophic (left box, 49 mutants).

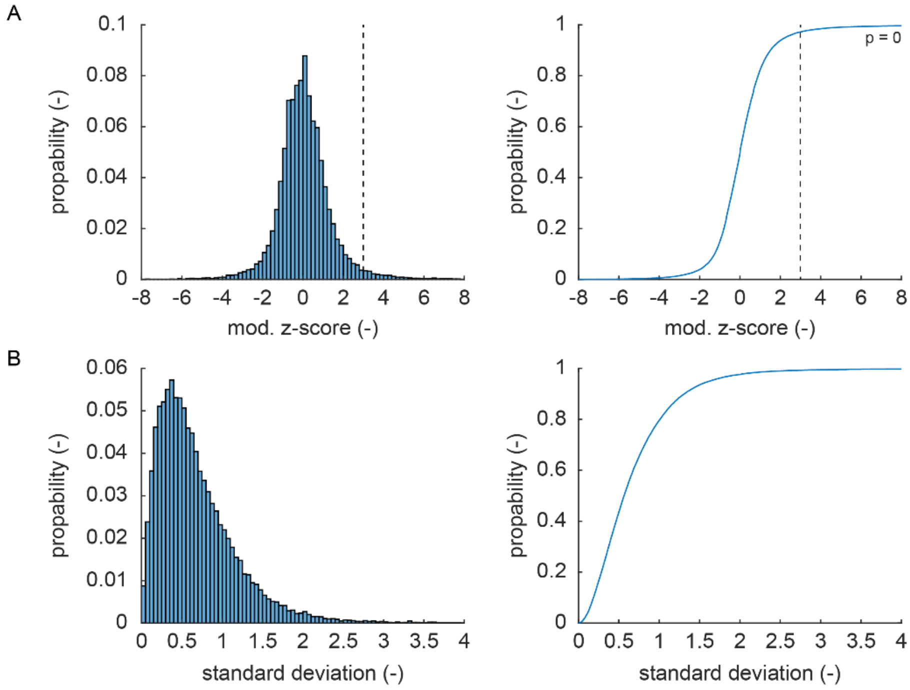

Appendix Figure S13 – The distributions of mod. z-score values and mod. z-score standard deviations.

A, The left histogram shows the empirical probabilities for the mod. z-score values based on the flow-injection mass spectrometry measurement of the 94 TS mutants and the control strain. The vertical dashed lines indicate the mod. z-score cutoff of 3. The right chart shows the empirical cumulative distribution function for all measured mod. z-scores. A one-sample Kolmogorov-Smirnov test confirmed normal distribution (p = 0, implementation by Matlab function “kstest”).

B, The left histogram shows the empirical probabilities for all standard deviations of the mod. z-scores (calculated by error propagation). The right chart shows the empirical cumulative distribution function for all standard deviations.

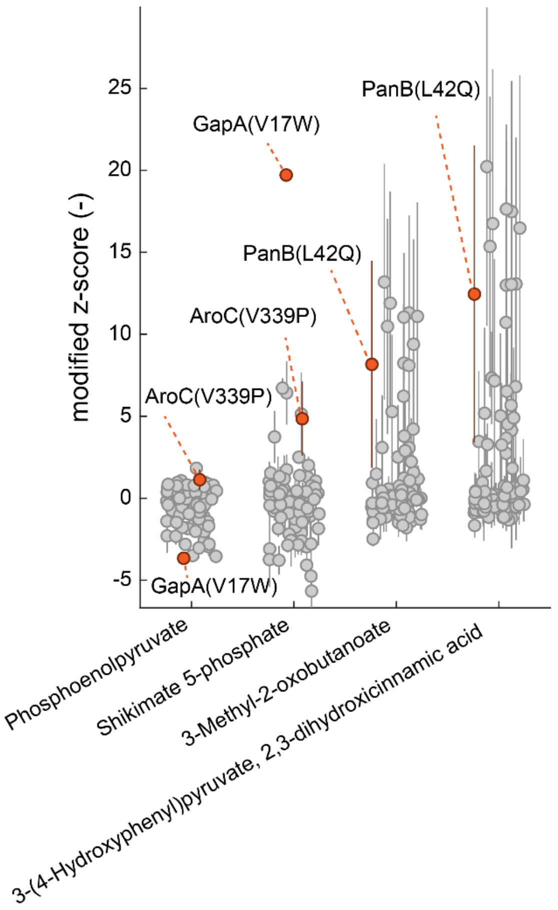

Appendix Figure S14 – Metabolite increases in the TS mutants GapA^{V17W}, AroC^{V339P}, and PanB^{L42Q}.

The chart shows the metabolite level (mod. z-score) in the 94 TS mutants and a control strain. Grey dots are the mean and vertical lines the standard deviation (n = 3). Data of the three TS mutants GapA^{V17W}, AroC^{V339P}, and PanB^{L42Q} is highlighted in red.

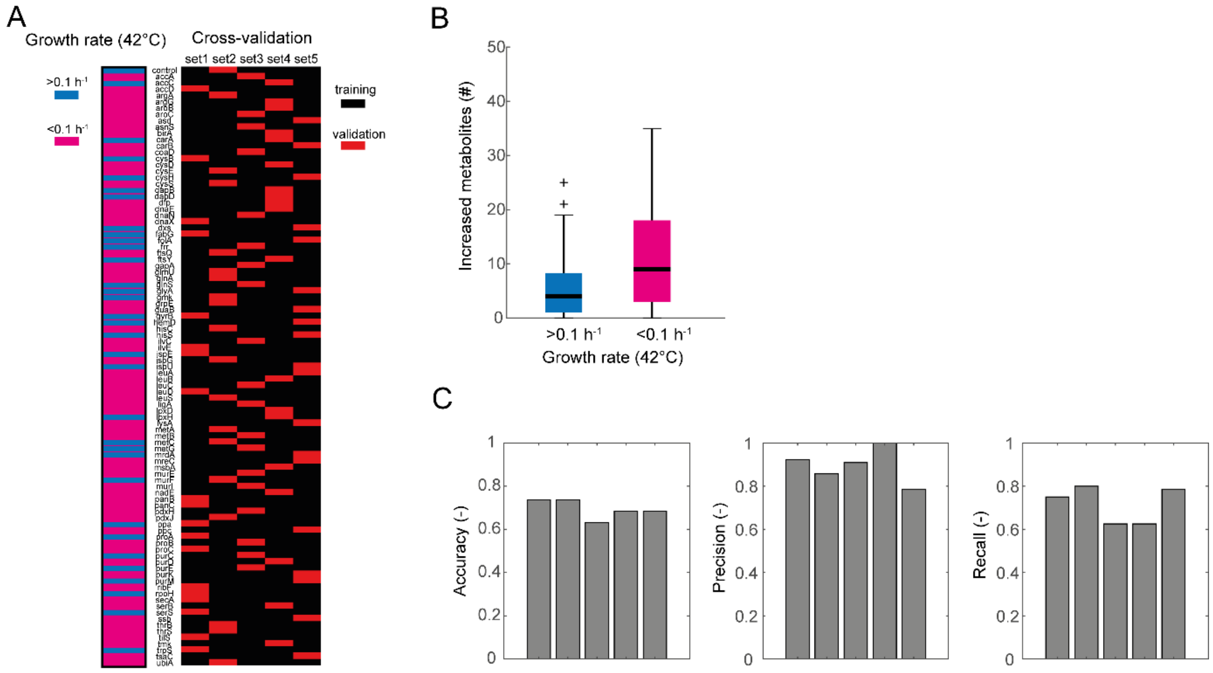

Appendix Figure S15 – Predicting non-growing strains from their metabolic profile

A, The left heatmap shows the classification of the 94 selected TS mutants into growing strains (mutants with a mean maximum specific growth rate ≥ 0.1 h^{-1} during plate reader cultivation in minimal M9 medium at 42 °C, also see Dataset EV7) and non-growing strains (growth rate < 0.1 h^{-1}). The mutants and their corresponding metabolomic profile (also see Fig 3) were then five times randomly distributed into training and validation datasets (set1 to set5) as indicated in the right heatmap. These datasets were then individually used to train a random forest (RF) model to predict non-growing mutants based on their metabolomic profile. The Matlab TreeBagger function was used to train random forest (RF) regression models. Predictor data was the z-scored metabolome data and class labels were based on growth at 42°C. 5-fold cross-validation was used for training and testing, resulting in 5 RF models trained on 76 strains. Predictions of growth of the remaining 19 strains was used to evaluate performance of the 5 models (accuracy, precision, and recall given in **C**).

B, The chart shows the number of metabolites with a mod. z-score > 3 per TS mutant in the group of growing TS mutants (blue) and non-growing TS mutants (magenta). The box whiskers indicate the median and $25^{th}/75^{th}$ percentiles.

C, The bar plots show the three performance parameters accuracy, precision, and recall of the five random forest models, which were trained on different datasets, as described in **A**. Accuracy, precision and recall were calculated based on true positive (TP), false positive (FP), true negative (TN) and false negative (FN) predictions of the 19 strains that were not used for training.
