# Supplementary material for: Mapping temperature‐sensitive mutations at a genome scale to engineer growth switches in Escherichia coli
Source: Mol Syst Biol. 2023 Aug 29;19(10):e11596. doi: 10.15252/msb.202311596 (PMC10568205; doi:10.15252/msb.202311596)
Supplement: Supplementary file 2 — Expanded View Figures PDF [file MSB-19-e11596-s017.pdf]

Expanded View Figures

Figure EV1. Mutation enrichment in TS alleles and mutants with strong fitness defects.

A The bar plot shows the total number of mutants in our CRISPR library after recombination (gray), the number of putative temperature-sensitive (TS) mutants (blue), and the number of mutants with strong fitness defects (yellow), which are strains with less than 15 reads at the start of the pooled fitness assay ($t = 0$ h) and strains that have a low fitness at 30°C based on cluster analysis.

B The bar plots show the *P*-values for testing an enrichment of mutations in alpha helices, beta sheets, turns, binding, and active sites among the putative TS mutants (blue bars) and strong fitness defect mutants (yellow bars). We used a one-tailed Fisher's exact test (also see Dataset EV3) and considered conditions with *P*-values < 0.05 as enriched.

C The left bar plots show *P*-values of the indicated mutations that were tested for an enrichment in the putative TS mutants (upper chart) and fitness defect mutants (lower chart) by a one-tailed Fisher's exact test. We considered conditions with *P*-values < 0.05 as enriched. The right bar plots show the %-enrichment between the putative TS and all other mutants (upper chart) and the growth defect mutants and all other mutants (lower chart).

© 2023 The Authors

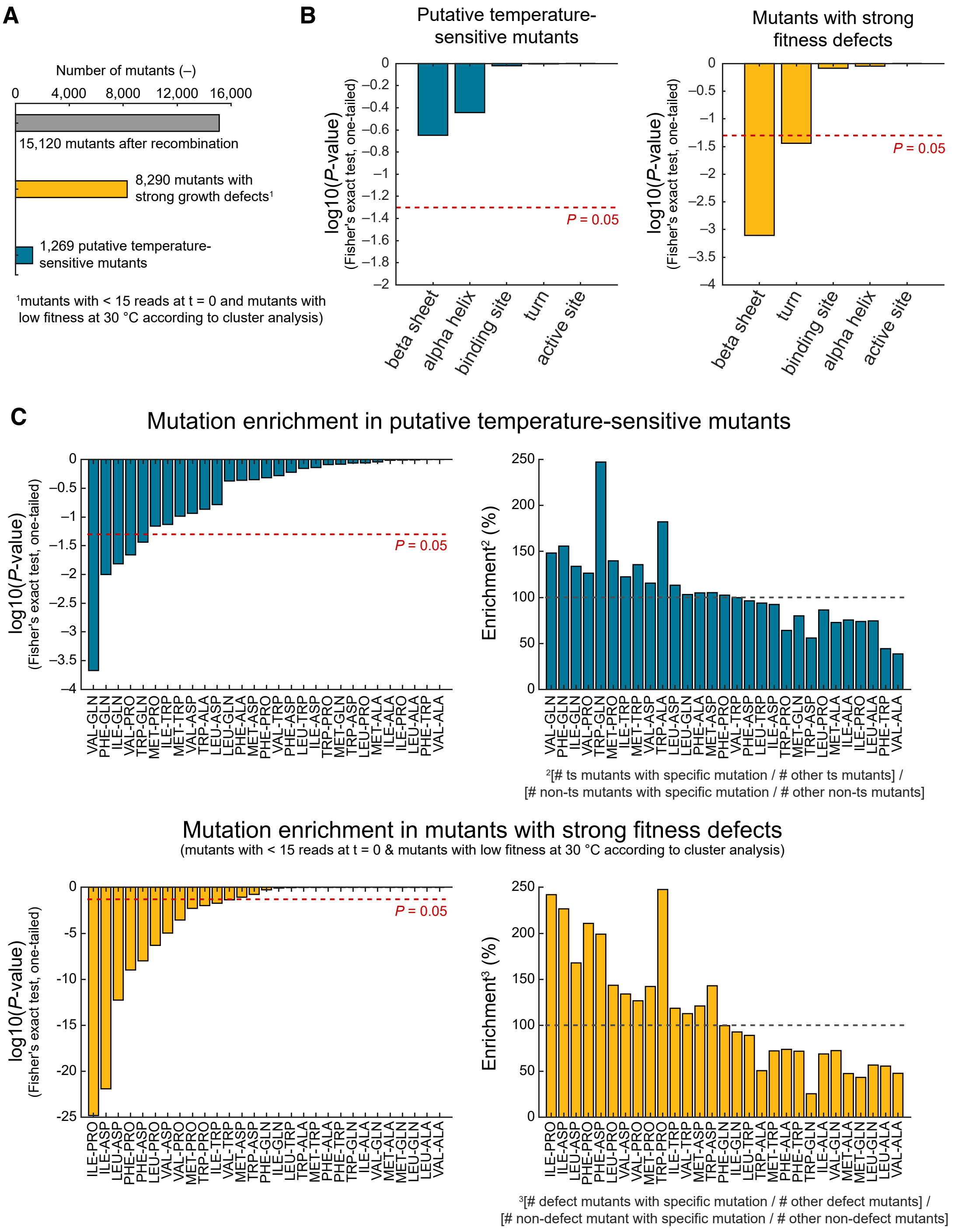
A
Number of mutants (–)
0 4,000 8,000 12,000 16,000
15,120 mutants after recombination
8,290 mutants with strong growth defects[1]
1,269 putative temperature-sensitive mutants
[1]mutants with < 15 reads at t = 0 and mutants with low fitness at 30 °C according to cluster analysis)
B
Putative temperature-sensitive mutants
Mutants with strong fitness defects
log10(P-value) (Fisher's exact test, one-tailed)
P = 0.05
beta sheet
alpha helix
binding site
turn
active site
C
Mutation enrichment in putative temperature-sensitive mutants
Enrichment[2] (%)
[2][# ts mutants with specific mutation / # other ts mutants] / [# non-ts mutants with specific mutation / # other non-ts mutants]
Mutation enrichment in mutants with strong fitness defects
(mutants with < 15 reads at t = 0 & mutants with low fitness at 30 °C according to cluster analysis)
Enrichment[3] (%)
[3][# defect mutants with specific mutation / # other defect mutants] / [# non-defect mutant with specific mutation / # other non-defect mutants]

Figure EV1.

Figure EV2. Fitness score dynamics and growth kinetics of an allelic series of cysS TS mutants.

We reconstructed a panel of 14 *cysS* alleles from the CRISPR library that were putative TS mutants. Each box shows a single mutation site in *cysS*, and each site had more than one allele available, except for the site *cysS*^{L387}. In each box, the left charts show the fitness score dynamics of the indicated mutant from the pooled fitness assay (Fig 1A). The dots in the left figures show data from two replicates per temperature, and the lines are the moving average through the means (blue: 30°C culture, red: 42°C culture). Dashed lines indicate a fitness score of 1. The right figures in each box show the maximum specific growth rates during plate reader growth at six different temperatures (30, 34, 38, 40, 42, and 44°C). The dots in the right figures are the mean of three replicates, and the vertical lines are the standard deviation. The dashed lines were calculated by fitting an Arrhenius-type function to the data (R^2 is the coefficient of determination, also see Appendix Fig S9). Indicated P-values were calculated with two-sample t-tests (two-tailed) comparing each the mutant strain against the unedited control strain at 42°C (Dataset EV4).

 © 2023 The Authors

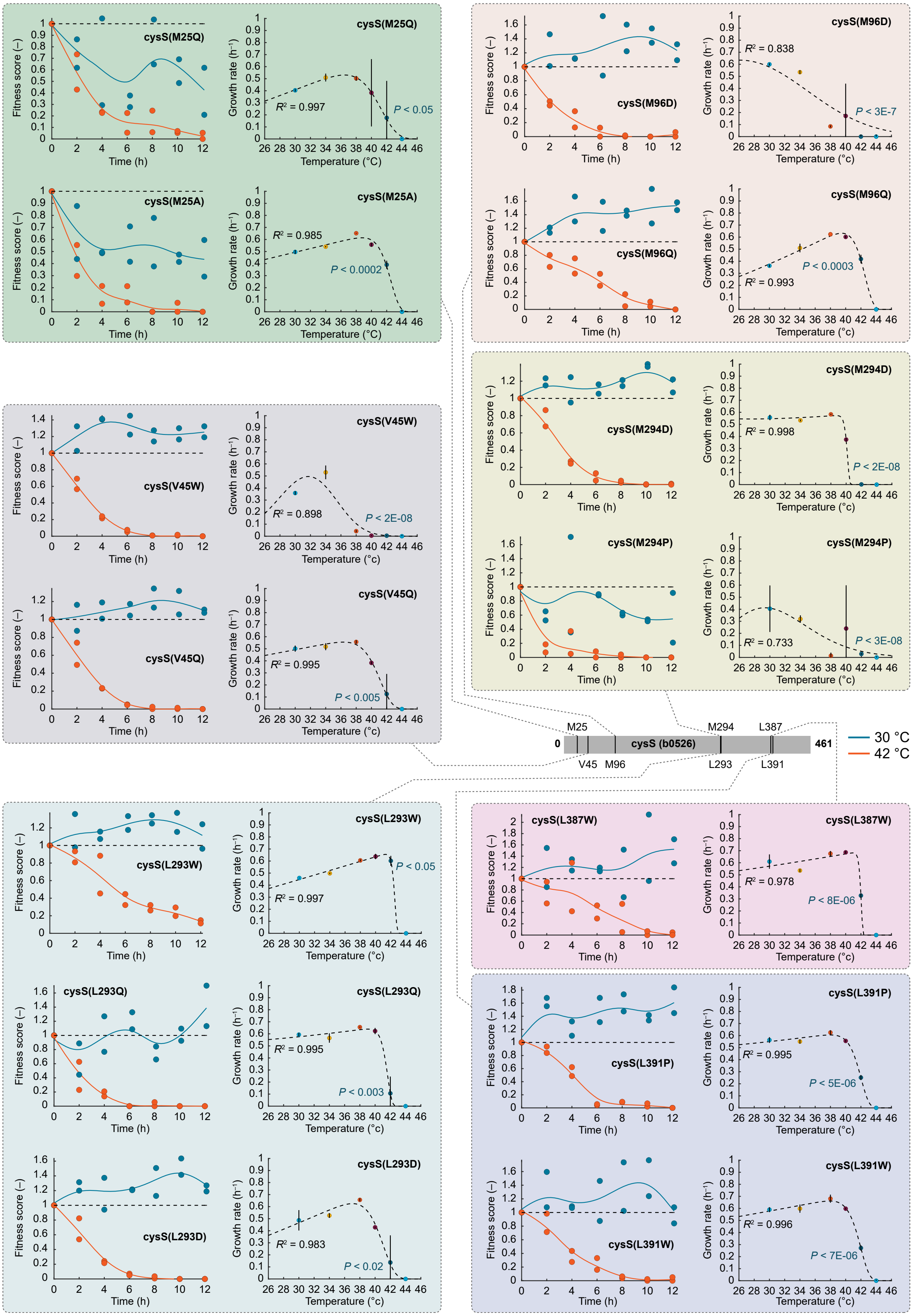
Fitness score (–)
Time (h)
Growth rate (h⁻¹)
Temperature (°C)
cysS(M25Q)
R² = 0.997
P < 0.05
cysS(M25A)
R² = 0.985
P < 0.0002
cysS(V45W)
R² = 0.898
P < 2E-08
cysS(V45Q)
R² = 0.995
P < 0.005
cysS(M96D)
R² = 0.838
P < 3E-7
cysS(M96Q)
R² = 0.993
P < 0.0003
cysS(M294D)
R² = 0.998
P < 2E-08
cysS(M294P)
R² = 0.733
P < 3E-08
0
M25
V45
M96
cysS (b0526)
M294
L293
L387
L391
461
30 °C
42 °C
cysS(L293W)
R² = 0.997
P < 0.05
cysS(L293Q)
R² = 0.995
P < 0.003
cysS(L293D)
R² = 0.983
P < 0.02
cysS(L387W)
R² = 0.978
P < 8E-06
cysS(L391P)
R² = 0.995
P < 5E-06
cysS(L391W)
R² = 0.996
P < 7E-06

Figure EV2.

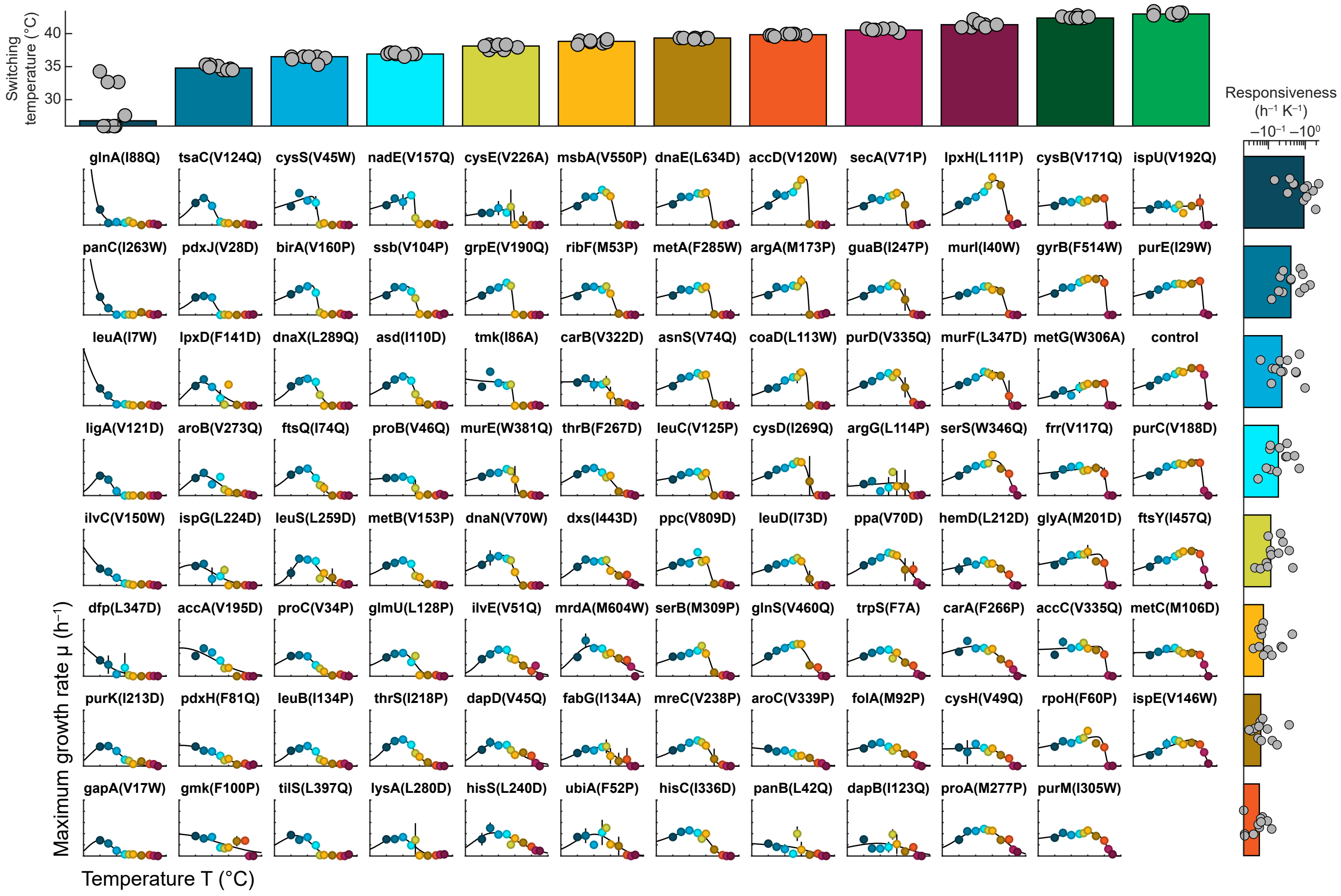

Figure EV3. Maximum specific growth rates of 94 TS mutants at different temperatures.

The charts show the maximum specific growth rates μ (h^{-1}) of 94 TS mutants (and a control strain) at 10 different temperatures ranging from 30 to 44°C. The growth rates were determined from growth curves in 96-well microtiter plate cultivations (Dataset EV7). Dots show the mean from three replicates, and black vertical lines show the standard deviation. An empirical Arrhenius-type function was fitted to the data (black lines, also see Appendix Fig S9). The strains were sorted according to their responsiveness and switching temperature, which are parameters based on the Arrhenius-type functions. The upper dot plot shows the switching temperatures (°C) of the strains in the columns below. The dot plot on the right side shows the responsiveness (h^{-1} K^{-1}) of the strains in the rows. The bars indicate the medians of the responsiveness values/switching temperatures.

© 2023 The Authors

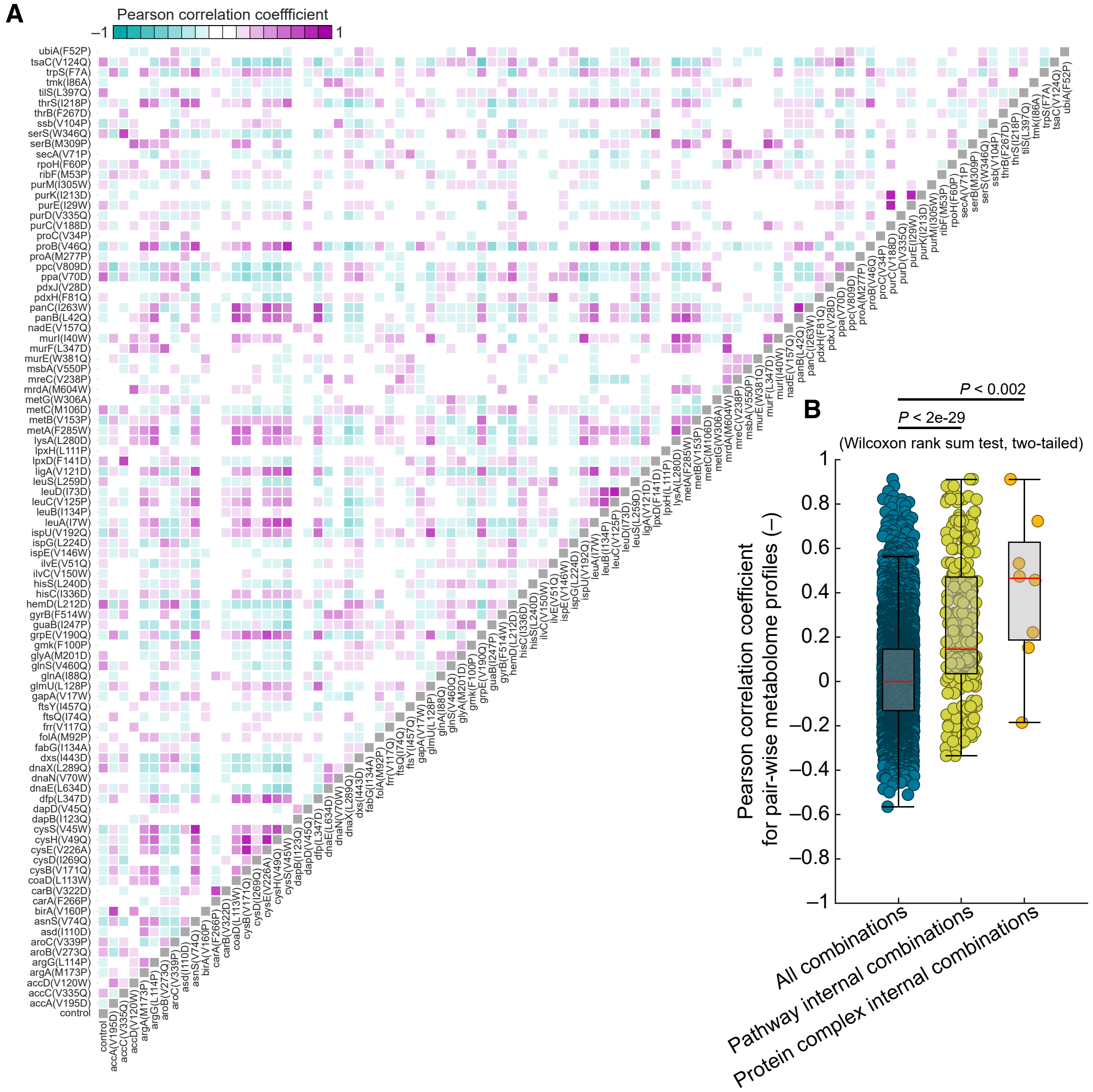

Figure EV4. Correlation analysis of metabolomes of 94 TS mutants.

A The heatmap shows the Pearson correlation coefficients (PCC) between metabolite data of all pair-wise combinations of 94 TS mutants and a control strain. The metabolite levels were measured by FI-MS after cultivation of the strains in 96-well microtiter plates at 42°C for 16 h (n = 3). Data of 325 metabolites (mod. z-scores) were used to calculate the PCC values.

B The dot plot shows all PCC values from (A) (given under "all combinations"), the PCC values from pairs of genes that are in the same metabolic pathway (given under "pathway internal combinations"), and from genes, whose proteins form a complex (given under "protein complex internal combinations"). The box-whisker plot indicates the median (red line), and the 25th and 75th percentiles (each dot is a combination, shown are all 4,465 combinations, 272 pathway internal combinations, and 8 protein complex internal combinations). We tested for differences between the PCC values in the three groups using a Wilcoxon rank-sum test (two-tailed) and indicate the respective P-values. Analysis is based on mean values of the metabolome data from three biological replicates.

© 2023 The Authors

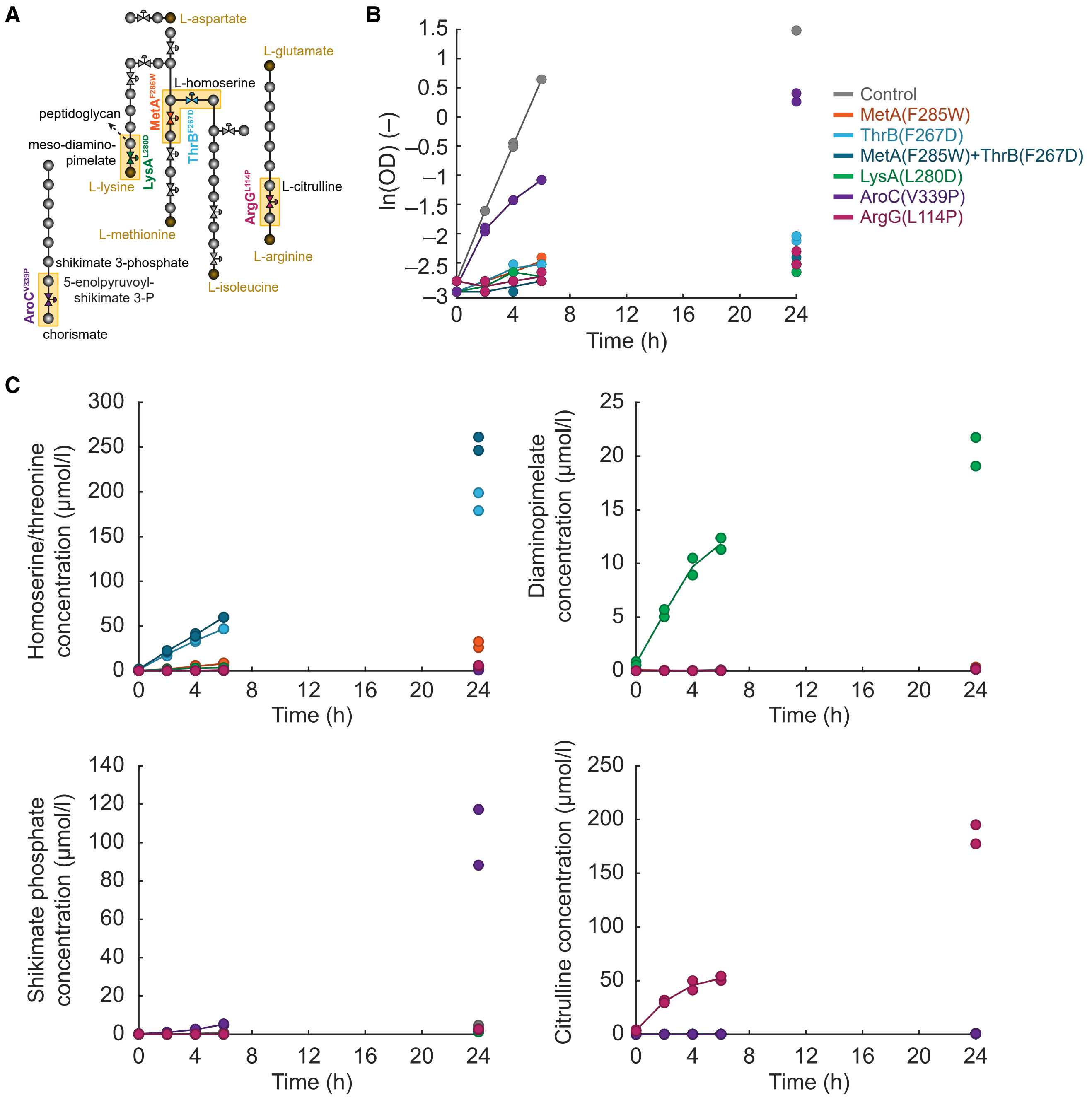

Figure EV5. Substrate production in TS mutants of enzymes.

A Schematic of biosynthesis pathways of chorismate, lysine, methionine, isoleucine, and arginine. Dots represent metabolites. Valve symbols indicate TS mutant enzymes.

B The chart shows the natural logarithm of biomass data (OD) from shaking flask cultivations of TS mutants and a control strain at 42°C. Dots are data from two replicates, and the lines connect the means.

C The charts show the concentrations (µmol/l) of indicated metabolites during the cultivations from (B). The concentrations were quantified in samples of the whole culture broth by LC–MS/MS. Dots are data from two replicates, and the lines connect the means.

© 2023 The Authors
