# Supplementary material for: Mapping temperature‐sensitive mutations at a genome scale to engineer growth switches in Escherichia coli
Source: Mol Syst Biol. 2023 Aug 29;19(10):e11596. doi: 10.15252/msb.202311596 (PMC10568205; doi:10.15252/msb.202311596)
Supplement: Supplementary file 13 — Dataset EV11 [file MSB-19-e11596-s015.pdf]

Expanded View Dataset

Dataset EV11

Mapping temperature-sensitive mutations at a genome-scale to engineer growth-switches in *E. coli*

Thorben Schramm[1,2], Paul Lubrano[1], Vanessa Pahl[1], Amelie Stadelmann[1], Andreas Verhülsdonk[1], Hannes Link[1,*]

[1]Interfaculty Institute of Microbiology and Infection Medicine, University of Tübingen, Auf der Morgenstelle 24, 72076 Tübingen, Germany

[2]Present address: Institute of Molecular Systems Biology, Department of Biology, ETH Zurich, Otto-Stern-Weg 3, 8093 Zürich, Switzerland

*Correspondence: hannes.link@uni-tuebingen.de

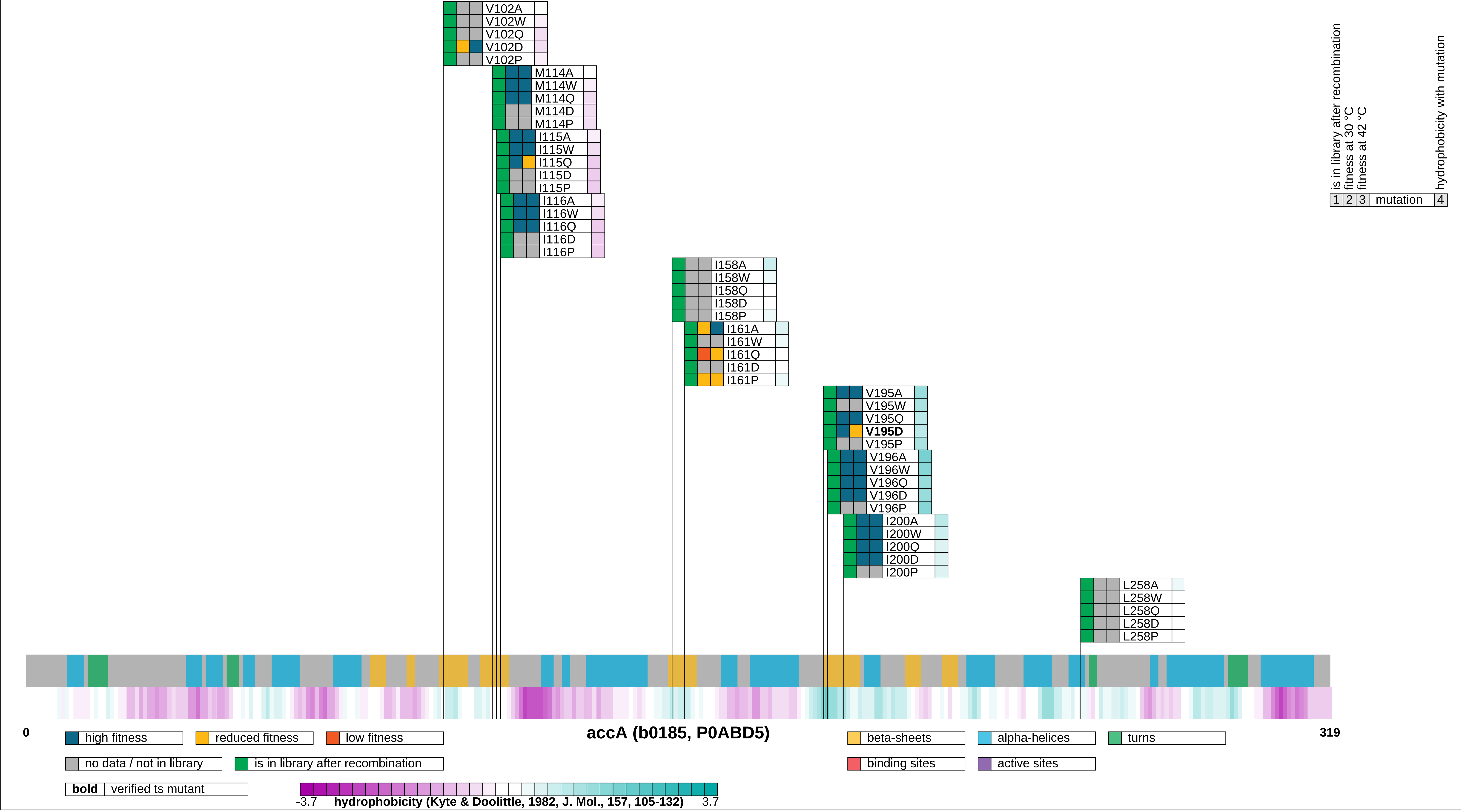

V102A
V102W
V102Q
V102D
V102P
M114A
M114W
M114Q
M114D
M114P
I115A
I115W
I115Q
I115D
I115P
I116A
I116W
I116Q
I116D
I116P
I158A
I158W
I158Q
I158D
I158P
I161A
I161W
I161Q
I161D
I161P
V195A
V195W
V195Q
V195D
V195P
V196A
V196W
V196Q
V196D
V196P
I200A
I200W
I200Q
I200D
I200P
L258A
L258W
L258Q
L258D
L258P
is in library after recombination
fitness at 30 °C
fitness at 42 °C
hydrophobicity with mutation
1 2 3 mutation 4
0
accA (b0185, P0ABD5)
319
high fitness
reduced fitness
low fitness
beta-sheets
alpha-helices
turns
no data / not in library
is in library after recombination
binding sites
active sites
bold verified ts mutant
-3.7
hydrophobicity (Kyte & Doolittle, 1982, J. Mol., 157, 105-132)
3.7

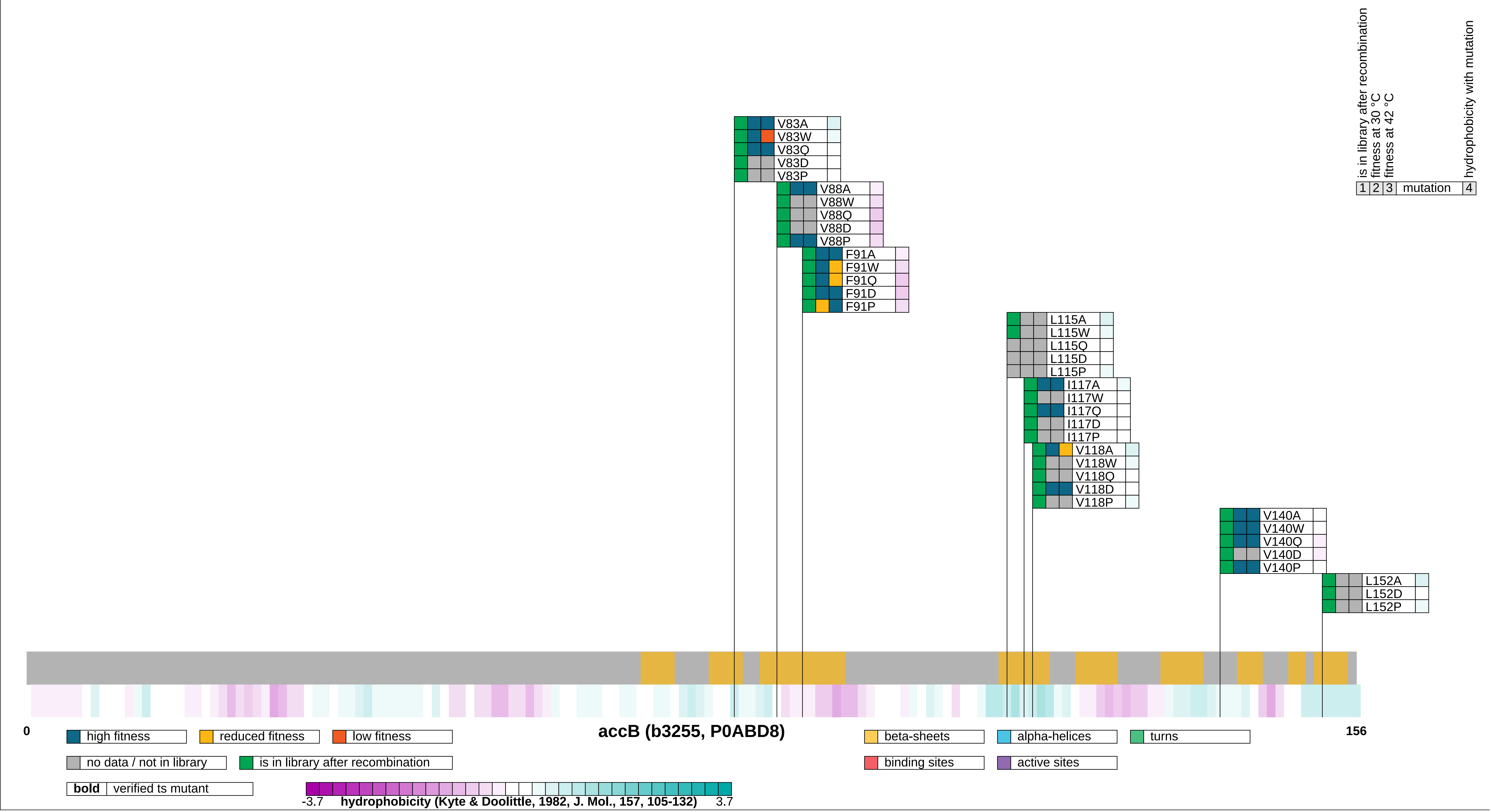

is in library after recombination
fitness at 30 °C
fitness at 42 °C
hydrophobicity with mutation
1 2 3 mutation 4
V83A
V83W
V83Q
V83D
V83P
V88A
V88W
V88Q
V88D
V88P
F91A
F91W
F91Q
F91D
F91P
L115A
L115W
L115Q
L115D
L115P
I117A
I117W
I117Q
I117D
I117P
V118A
V118W
V118Q
V118D
V118P
V140A
V140W
V140Q
V140D
V140P
L152A
L152D
L152P
0
156
accB (b3255, P0ABD8)
high fitness
reduced fitness
low fitness
no data / not in library
is in library after recombination
bold verified ts mutant
beta-sheets
alpha-helices
turns
binding sites
active sites
-3.7
hydrophobicity (Kyte & Doolittle, 1982, J. Mol., 157, 105-132)
3.7

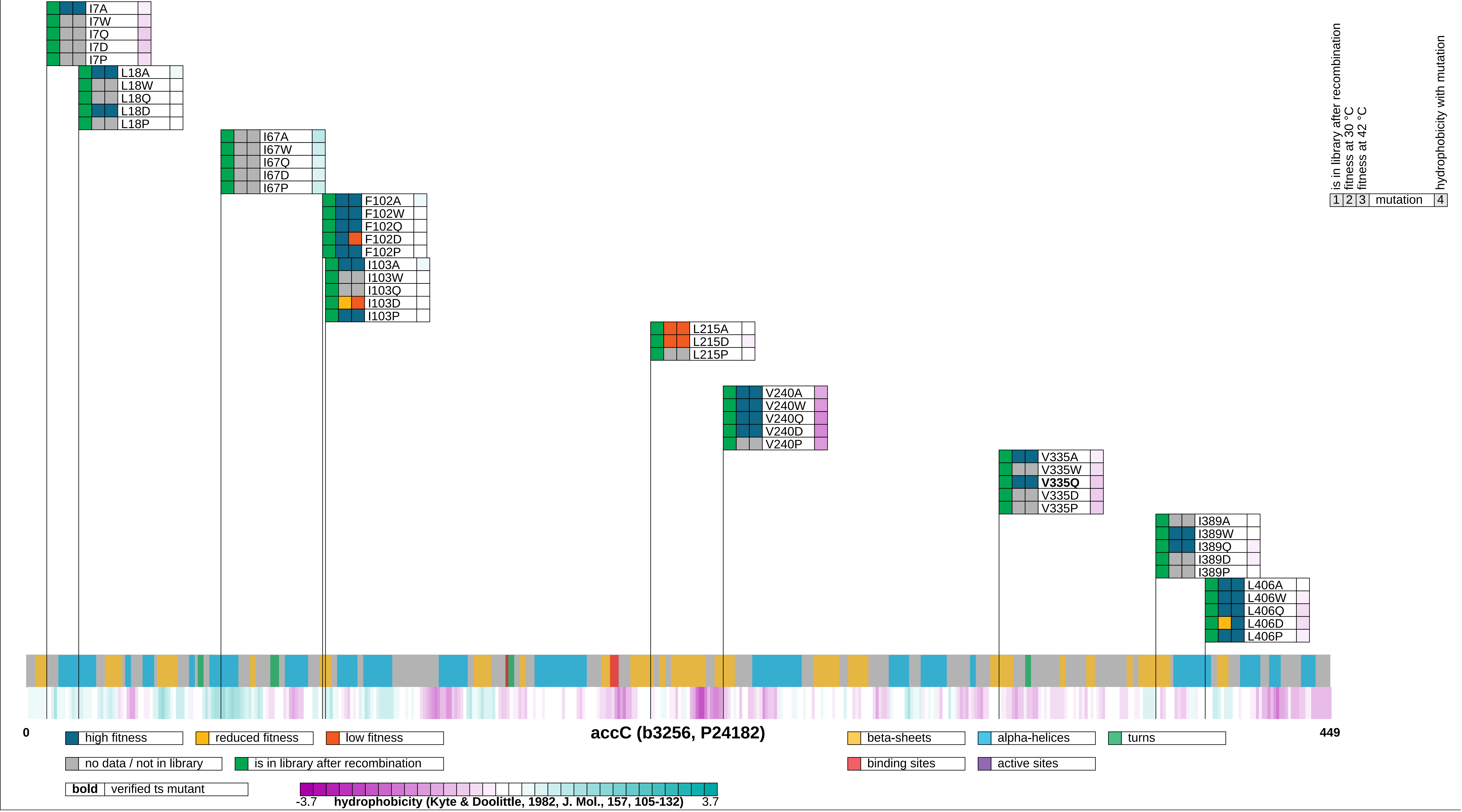

I7A
I7W
I7Q
I7D
I7P
L18A
L18W
L18Q
L18D
L18P
I67A
I67W
I67Q
I67D
I67P
F102A
F102W
F102Q
F102D
F102P
I103A
I103W
I103Q
I103D
I103P
L215A
L215D
L215P
V240A
V240W
V240Q
V240D
V240P
V335A
V335W
V335Q
V335D
V335P
I389A
I389W
I389Q
I389D
I389P
L406A
L406W
L406Q
L406D
L406P
is in library after recombination
fitness at 30 °C
fitness at 42 °C
hydrophobicity with mutation
1 2 3 mutation 4
0
accC (b3256, P24182)
449
high fitness
reduced fitness
low fitness
beta-sheets
alpha-helices
turns
no data / not in library
is in library after recombination
binding sites
active sites
bold verified ts mutant
-3.7
hydrophobicity (Kyte & Doolittle, 1982, J. Mol., 157, 105-132)
3.7

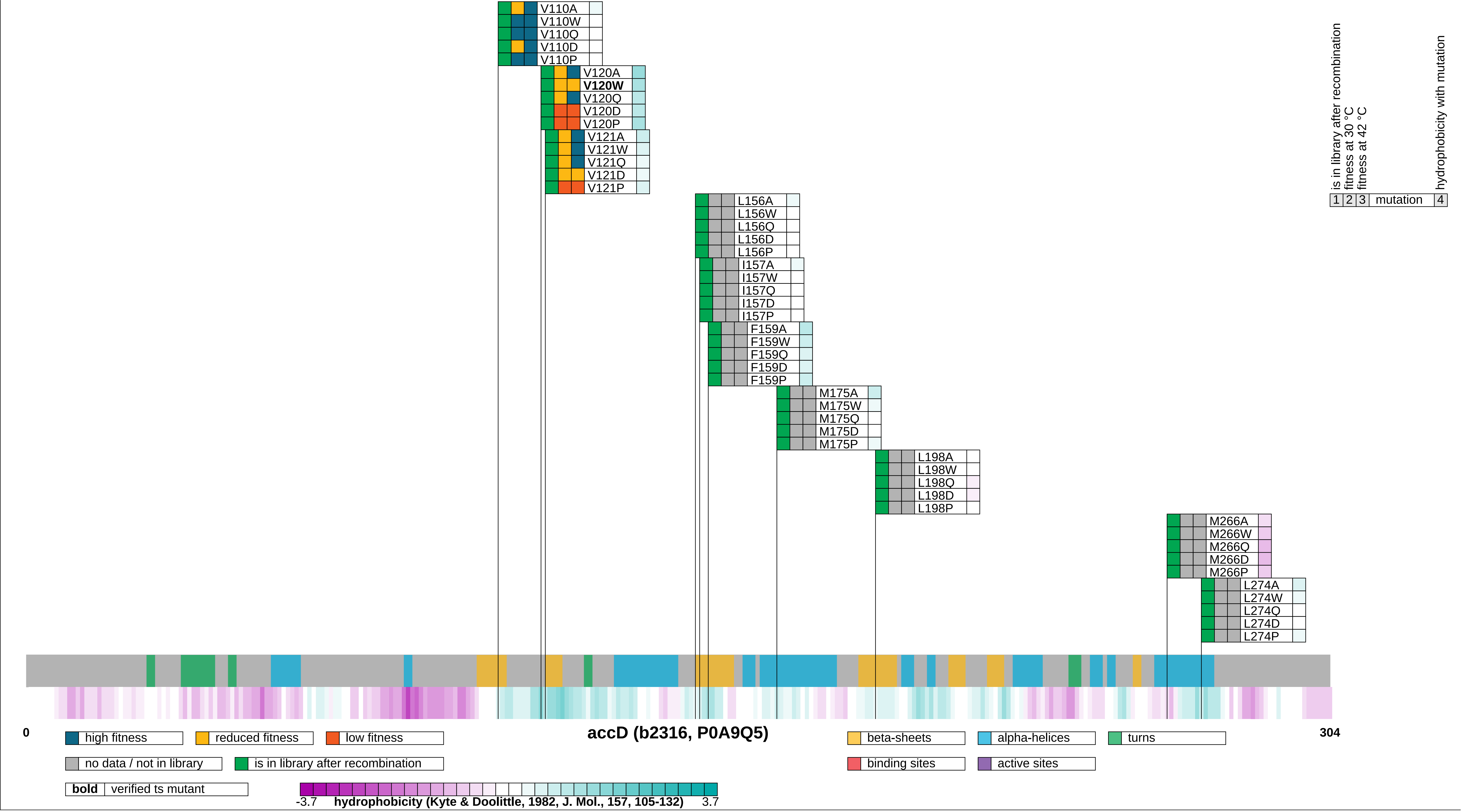
V110A
V110W
V110Q
V110D
V110P
V120A
V120W
V120Q
V120D
V120P
V121A
V121W
V121Q
V121D
V121P
L156A
L156W
L156Q
L156D
L156P
I157A
I157W
I157Q
I157D
I157P
F159A
F159W
F159Q
F159D
F159P
M175A
M175W
M175Q
M175D
M175P
L198A
L198W
L198Q
L198D
L198P
M266A
M266W
M266Q
M266D
M266P
L274A
L274W
L274Q
L274D
L274P
is in library after recombination
fitness at 30 °C
fitness at 42 °C
hydrophobicity with mutation
1 2 3 mutation 4
0
accD (b2316, P0A9Q5)
304
high fitness
reduced fitness
low fitness
no data / not in library
is in library after recombination
bold verified ts mutant
beta-sheets
alpha-helices
turns
binding sites
active sites
-3.7 hydrophobicity (Kyte & Doolittle, 1982, J. Mol., 157, 105-132) 3.7

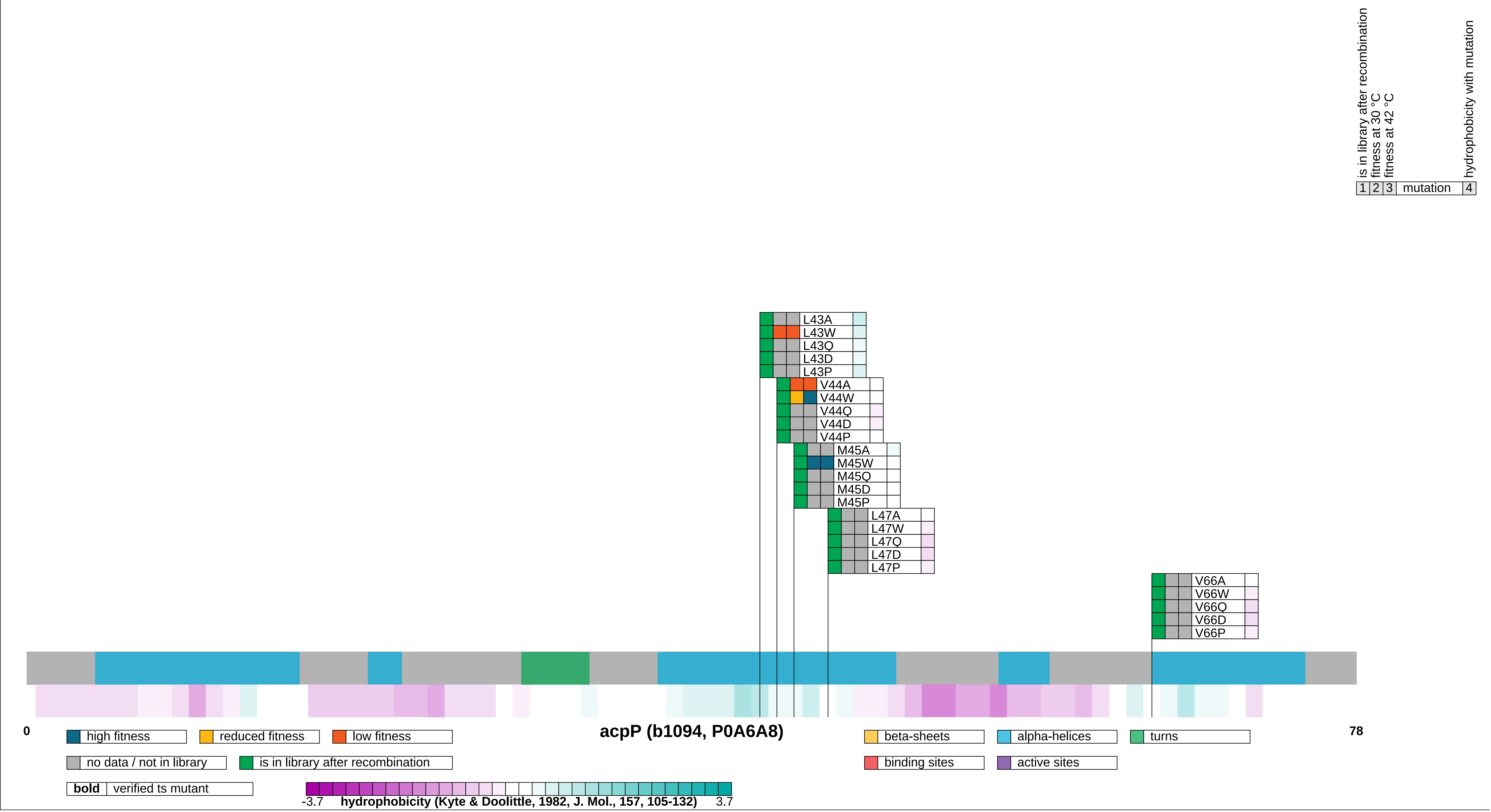
is in library after recombination
fitness at 30 °C
fitness at 42 °C
hydrophobicity with mutation
1 2 3 mutation 4
L43A
L43W
L43Q
L43D
L43P
V44A
V44W
V44Q
V44D
V44P
M45A
M45W
M45Q
M45D
M45P
L47A
L47W
L47Q
L47D
L47P
V66A
V66W
V66Q
V66D
V66P
0
acpP (b1094, P0A6A8)
78
high fitness
reduced fitness
low fitness
beta-sheets
alpha-helices
turns
no data / not in library
is in library after recombination
binding sites
active sites
bold verified ts mutant
-3.7
hydrophobicity (Kyte & Doolittle, 1982, J. Mol., 157, 105-132)
3.7

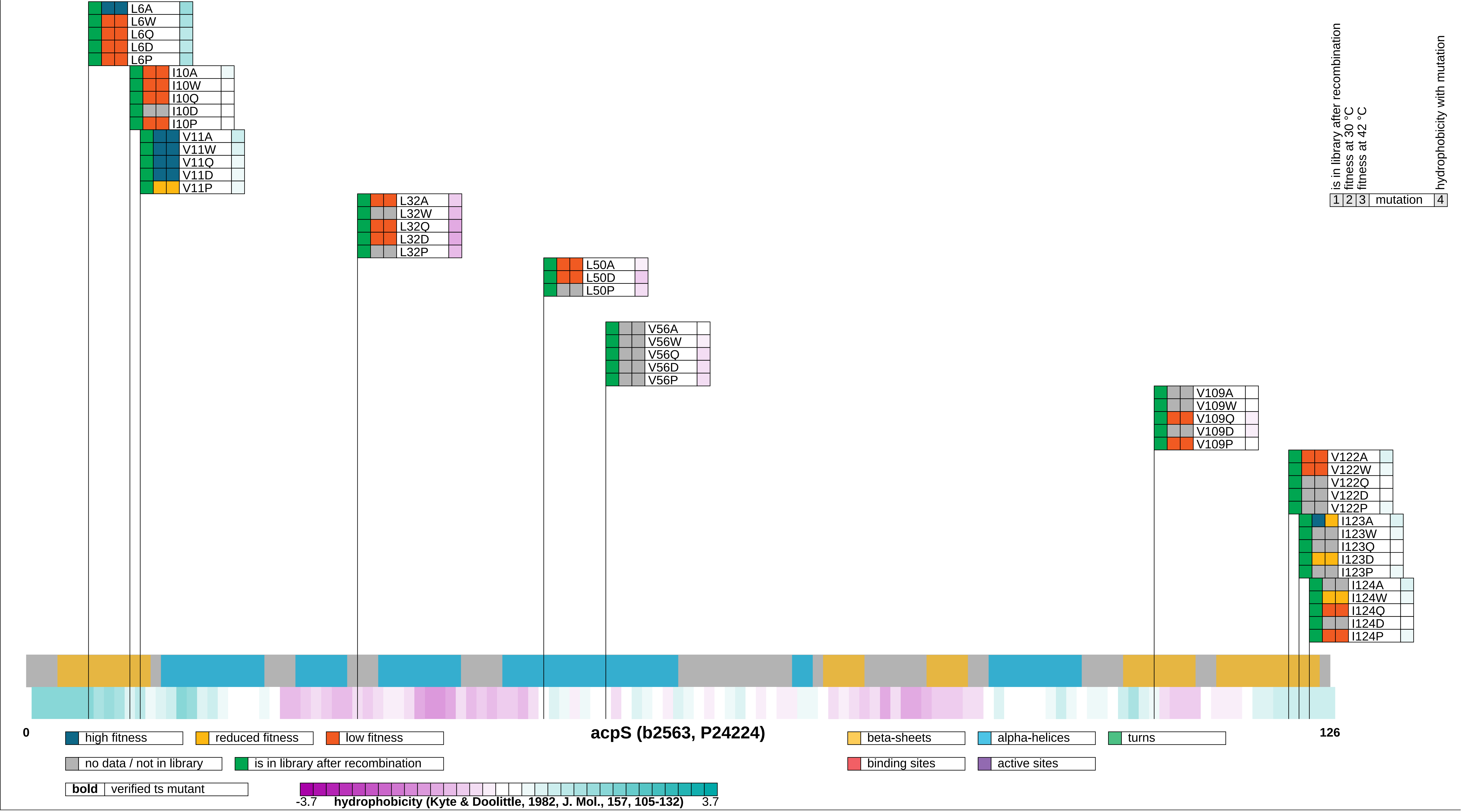

L6A
L6W
L6Q
L6D
L6P
I10A
I10W
I10Q
I10D
I10P
V11A
V11W
V11Q
V11D
V11P
L32A
L32W
L32Q
L32D
L32P
L50A
L50D
L50P
V56A
V56W
V56Q
V56D
V56P
V109A
V109W
V109Q
V109D
V109P
V122A
V122W
V122Q
V122D
V122P
I123A
I123W
I123Q
I123D
I123P
I124A
I124W
I124Q
I124D
I124P
is in library after recombination
fitness at 30 °C
fitness at 42 °C
hydrophobicity with mutation
1
2
3
mutation
4
0
acpS (b2563, P24224)
126
high fitness
reduced fitness
low fitness
beta-sheets
alpha-helices
turns
no data / not in library
is in library after recombination
binding sites
active sites
bold
verified ts mutant
-3.7
hydrophobicity (Kyte & Doolittle, 1982, J. Mol., 157, 105-132)
3.7

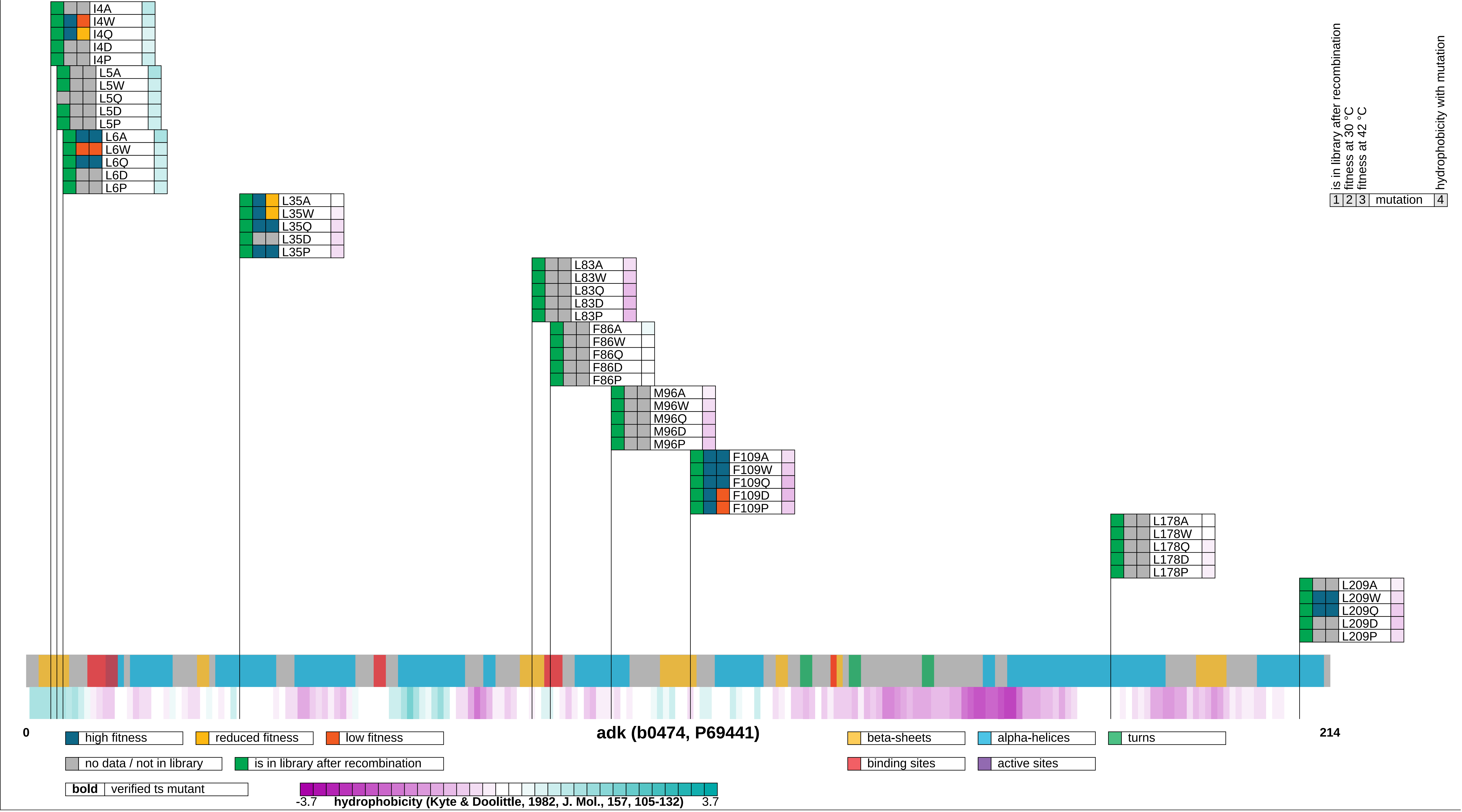

I4A
I4W
I4Q
I4D
I4P
L5A
L5W
L5Q
L5D
L5P
L6A
L6W
L6Q
L6D
L6P
L35A
L35W
L35Q
L35D
L35P
L83A
L83W
L83Q
L83D
L83P
F86A
F86W
F86Q
F86D
F86P
M96A
M96W
M96Q
M96D
M96P
F109A
F109W
F109Q
F109D
F109P
L178A
L178W
L178Q
L178D
L178P
L209A
L209W
L209Q
L209D
L209P
is in library after recombination
fitness at 30 °C
fitness at 42 °C
hydrophobicity with mutation
1 2 3 mutation 4
0
adk (b0474, P69441)
214
high fitness
reduced fitness
low fitness
no data / not in library
is in library after recombination
bold verified ts mutant
beta-sheets
alpha-helices
turns
binding sites
active sites
-3.7
hydrophobicity (Kyte & Doolittle, 1982, J. Mol., 157, 105-132)
3.7

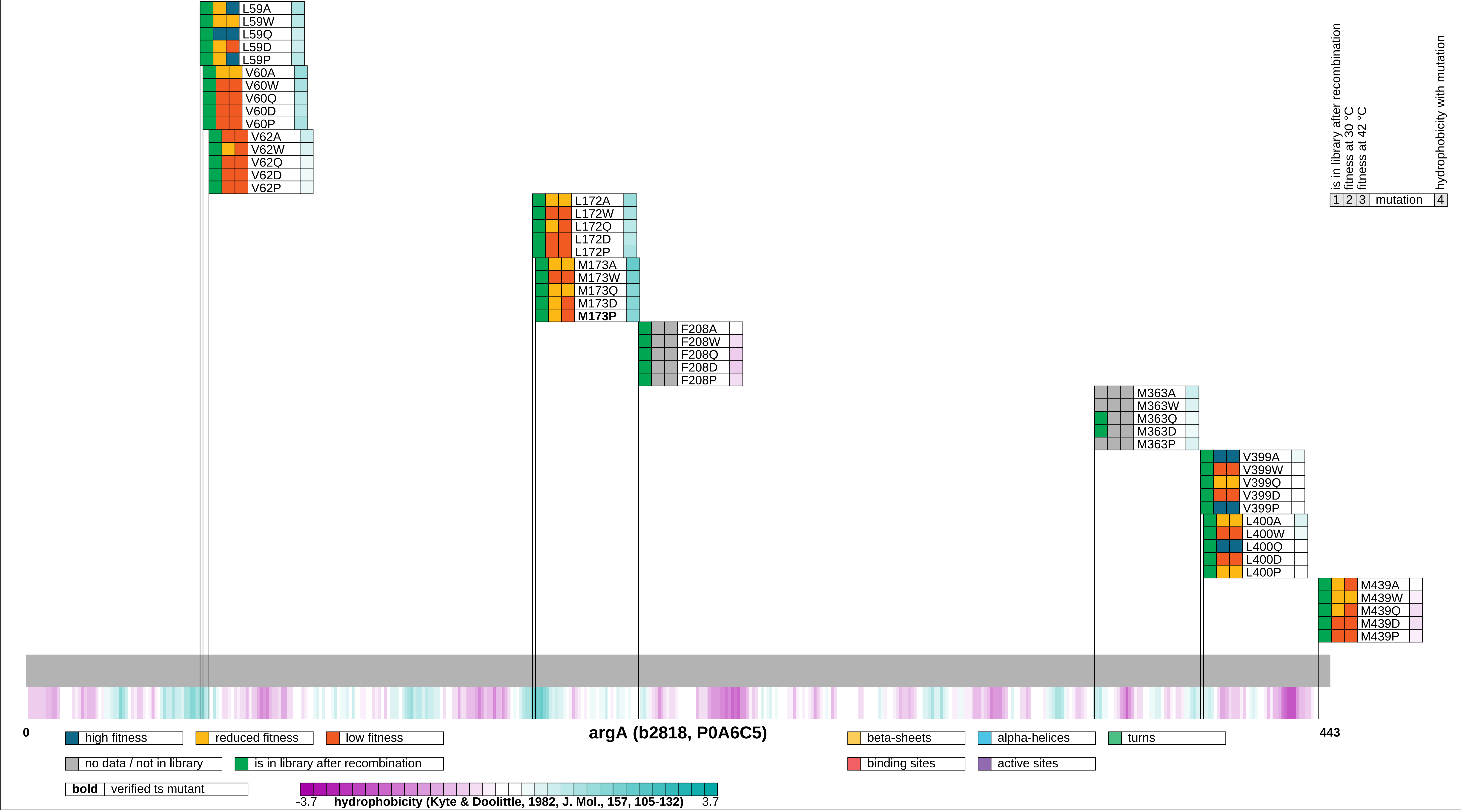

L59A
L59W
L59Q
L59D
L59P
V60A
V60W
V60Q
V60D
V60P
V62A
V62W
V62Q
V62D
V62P
L172A
L172W
L172Q
L172D
L172P
M173A
M173W
M173Q
M173D
M173P
F208A
F208W
F208Q
F208D
F208P
M363A
M363W
M363Q
M363D
M363P
V399A
V399W
V399Q
V399D
V399P
L400A
L400W
L400Q
L400D
L400P
M439A
M439W
M439Q
M439D
M439P
is in library after recombination
fitness at 30 °C
fitness at 42 °C
hydrophobicity with mutation
1 2 3 mutation 4
0
443
argA (b2818, P0A6C5)
high fitness
reduced fitness
low fitness
no data / not in library
is in library after recombination
bold verified ts mutant
beta-sheets
alpha-helices
turns
binding sites
active sites
-3.7 **hydrophobicity (Kyte & Doolittle, 1982, J. Mol., 157, 105-132)** 3.7

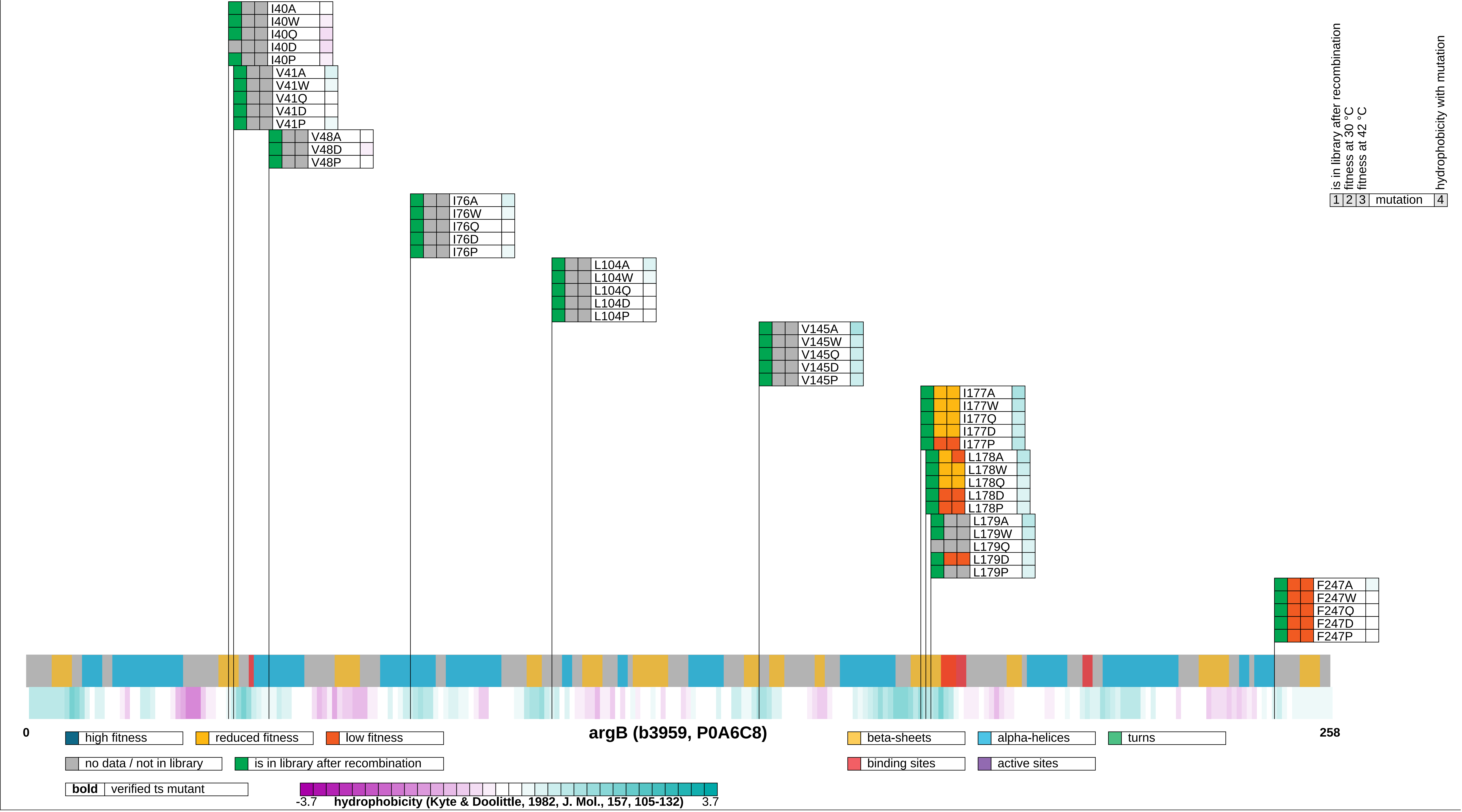

I40A
I40W
I40Q
I40D
I40P
V41A
V41W
V41Q
V41D
V41P
V48A
V48D
V48P
I76A
I76W
I76Q
I76D
I76P
L104A
L104W
L104Q
L104D
L104P
V145A
V145W
V145Q
V145D
V145P
I177A
I177W
I177Q
I177D
I177P
L178A
L178W
L178Q
L178D
L178P
L179A
L179W
L179Q
L179D
L179P
F247A
F247W
F247Q
F247D
F247P
is in library after recombination
fitness at 30 °C
fitness at 42 °C
hydrophobicity with mutation
1 2 3 mutation 4
0
258
argB (b3959, P0A6C8)
high fitness
reduced fitness
low fitness
no data / not in library
is in library after recombination
bold verified ts mutant
beta-sheets
alpha-helices
turns
binding sites
active sites
-3.7
hydrophobicity (Kyte & Doolittle, 1982, J. Mol., 157, 105-132)
3.7

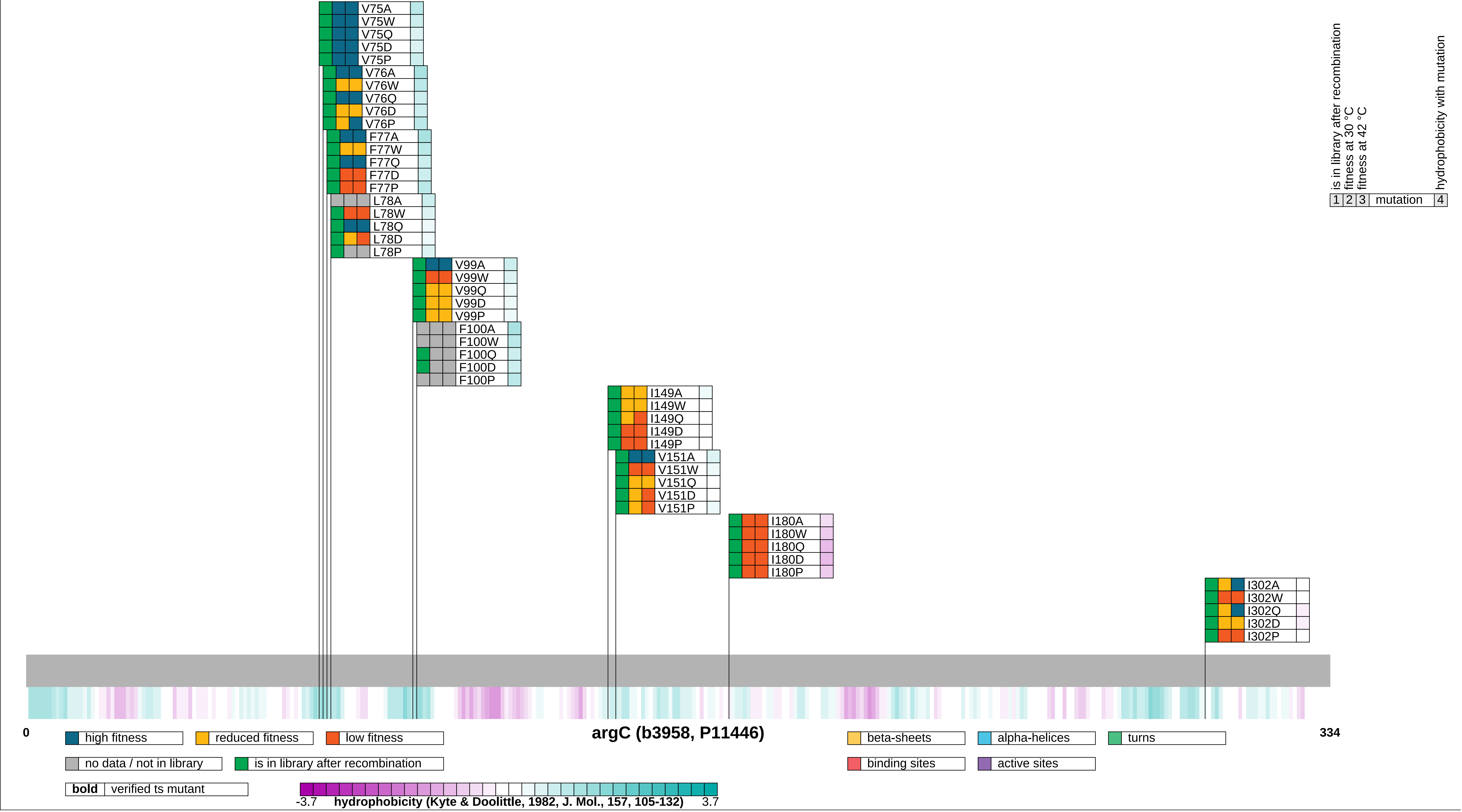

V75A
V75W
V75Q
V75D
V75P
V76A
V76W
V76Q
V76D
V76P
F77A
F77W
F77Q
F77D
F77P
L78A
L78W
L78Q
L78D
L78P
V99A
V99W
V99Q
V99D
V99P
F100A
F100W
F100Q
F100D
F100P
I149A
I149W
I149Q
I149D
I149P
V151A
V151W
V151Q
V151D
V151P
I180A
I180W
I180Q
I180D
I180P
I302A
I302W
I302Q
I302D
I302P
is in library after recombination
fitness at 30 °C
fitness at 42 °C
hydrophobicity with mutation
1 2 3 mutation 4
0
argC (b3958, P11446)
334
high fitness
reduced fitness
low fitness
no data / not in library
is in library after recombination
bold verified ts mutant
beta-sheets
alpha-helices
turns
binding sites
active sites
-3.7
hydrophobicity (Kyte & Doolittle, 1982, J. Mol., 157, 105-132)
3.7

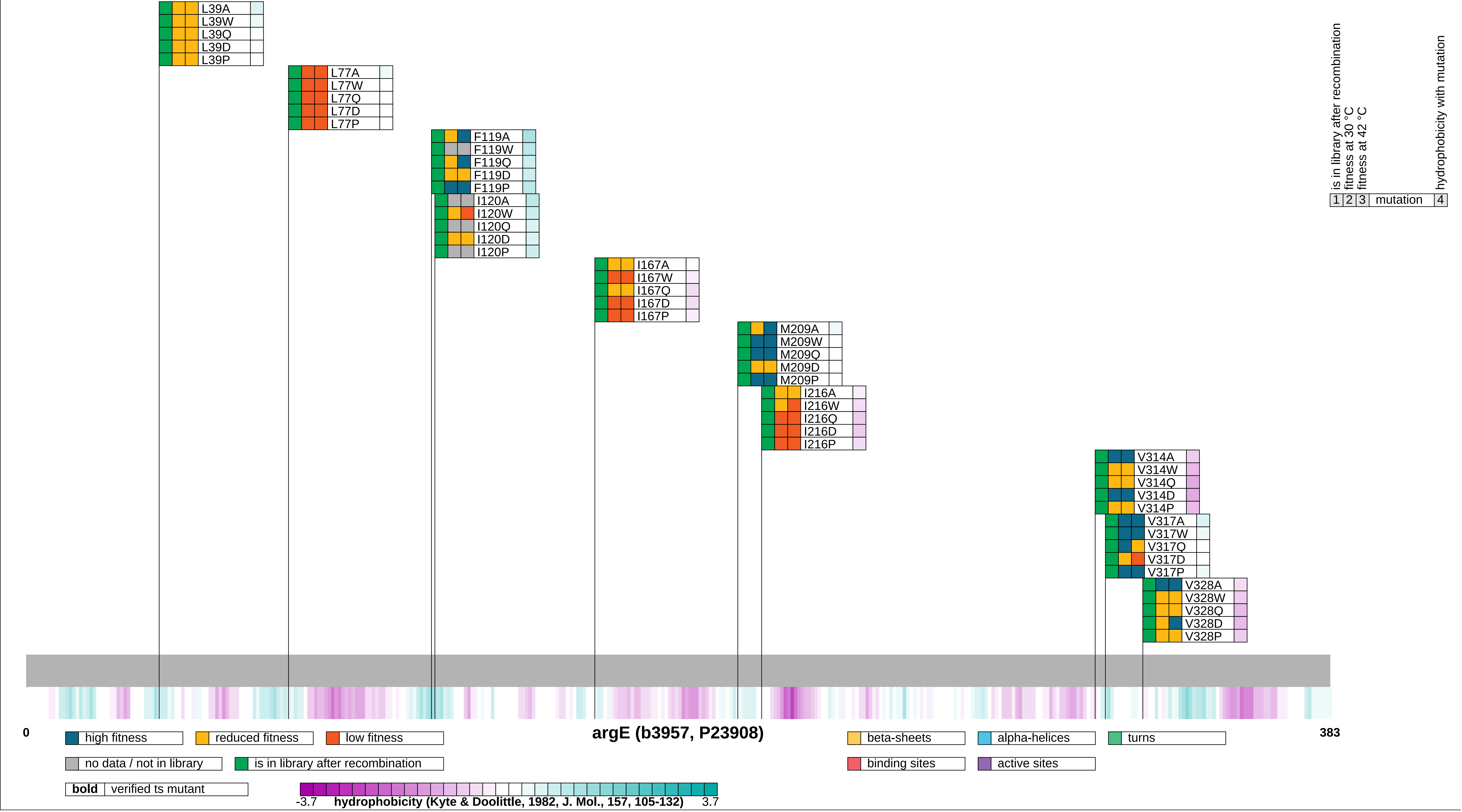

L39A
L39W
L39Q
L39D
L39P
L77A
L77W
L77Q
L77D
L77P
F119A
F119W
F119Q
F119D
F119P
I120A
I120W
I120Q
I120D
I120P
I167A
I167W
I167Q
I167D
I167P
M209A
M209W
M209Q
M209D
M209P
I216A
I216W
I216Q
I216D
I216P
V314A
V314W
V314Q
V314D
V314P
V317A
V317W
V317Q
V317D
V317P
V328A
V328W
V328Q
V328D
V328P
is in library after recombination
fitness at 30 °C
fitness at 42 °C
hydrophobicity with mutation
1 2 3 mutation 4
0
argE (b3957, P23908)
383
high fitness
reduced fitness
low fitness
no data / not in library
is in library after recombination
bold verified ts mutant
beta-sheets
alpha-helices
turns
binding sites
active sites
-3.7
hydrophobicity (Kyte & Doolittle, 1982, J. Mol., 157, 105-132)
3.7

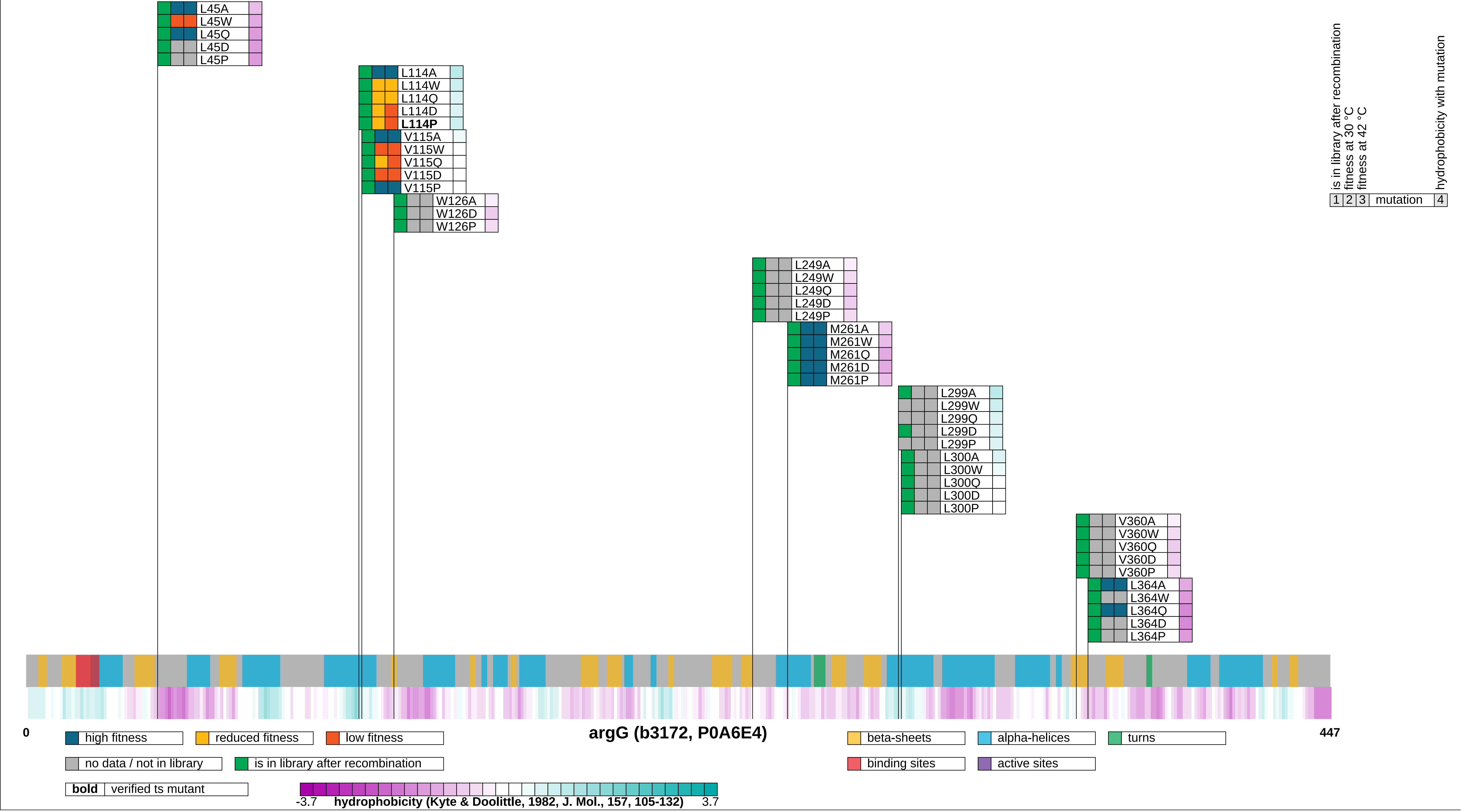

L45A
L45W
L45Q
L45D
L45P
L114A
L114W
L114Q
L114D
L114P
V115A
V115W
V115Q
V115D
V115P
W126A
W126D
W126P
L249A
L249W
L249Q
L249D
L249P
M261A
M261W
M261Q
M261D
M261P
L299A
L299W
L299Q
L299D
L299P
L300A
L300W
L300Q
L300D
L300P
V360A
V360W
V360Q
V360D
V360P
L364A
L364W
L364Q
L364D
L364P
is in library after recombination
fitness at 30 °C
fitness at 42 °C
hydrophobicity with mutation
1 2 3 mutation 4
0
argG (b3172, P0A6E4)
447
high fitness
reduced fitness
low fitness
no data / not in library
is in library after recombination
bold verified ts mutant
beta-sheets
alpha-helices
turns
binding sites
active sites
-3.7
hydrophobicity (Kyte & Doolittle, 1982, J. Mol., 157, 105-132)
3.7

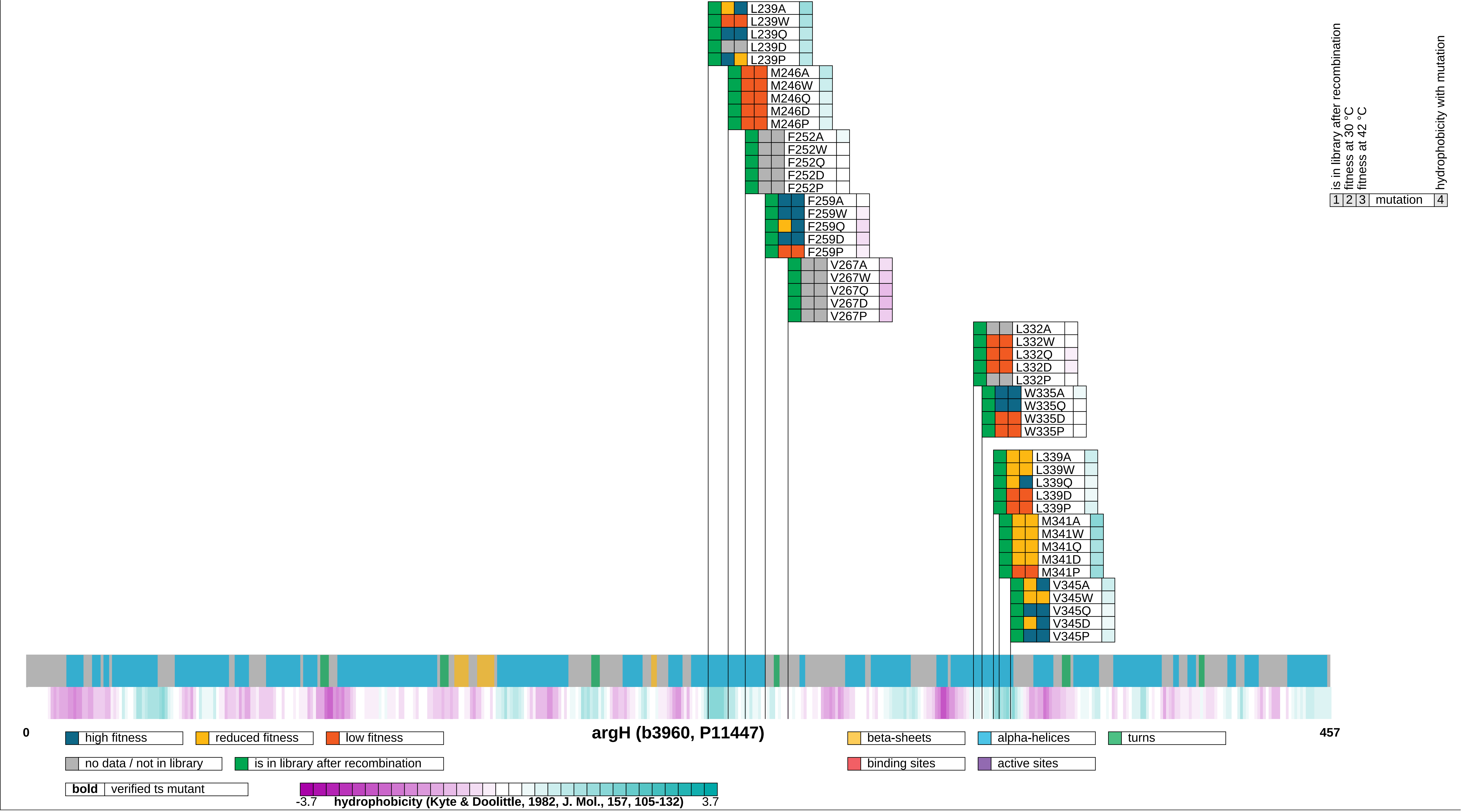

L239A
L239W
L239Q
L239D
L239P
M246A
M246W
M246Q
M246D
M246P
F252A
F252W
F252Q
F252D
F252P
F259A
F259W
F259Q
F259D
F259P
V267A
V267W
V267Q
V267D
V267P
L332A
L332W
L332Q
L332D
L332P
W335A
W335Q
W335D
W335P
L339A
L339W
L339Q
L339D
L339P
M341A
M341W
M341Q
M341D
M341P
V345A
V345W
V345Q
V345D
V345P
is in library after recombination
fitness at 30 °C
fitness at 42 °C
hydrophobicity with mutation
1 2 3 mutation 4
0
argH (b3960, P11447)
457
high fitness
reduced fitness
low fitness
beta-sheets
alpha-helices
turns
no data / not in library
is in library after recombination
binding sites
active sites
bold verified ts mutant
-3.7
hydrophobicity (Kyte & Doolittle, 1982, J. Mol., 157, 105-132)
3.7

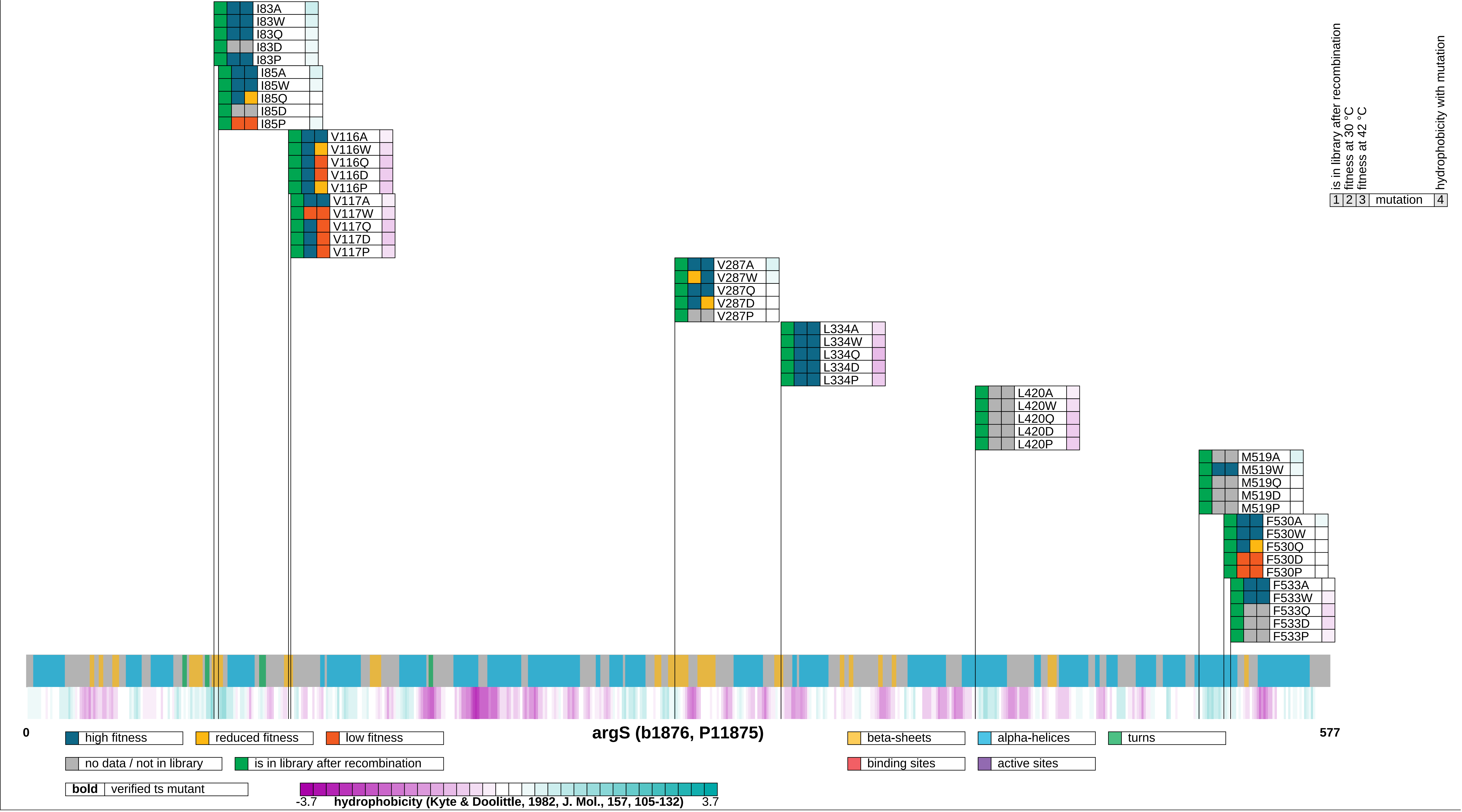
I83A
I83W
I83Q
I83D
I83P
I85A
I85W
I85Q
I85D
I85P
V116A
V116W
V116Q
V116D
V116P
V117A
V117W
V117Q
V117D
V117P
V287A
V287W
V287Q
V287D
V287P
L334A
L334W
L334Q
L334D
L334P
L420A
L420W
L420Q
L420D
L420P
M519A
M519W
M519Q
M519D
M519P
F530A
F530W
F530Q
F530D
F530P
F533A
F533W
F533Q
F533D
F533P
is in library after recombination
fitness at 30 °C
fitness at 42 °C
hydrophobicity with mutation
1 2 3 mutation 4
0
argS (b1876, P11875)
577
high fitness
reduced fitness
low fitness
beta-sheets
alpha-helices
turns
no data / not in library
is in library after recombination
binding sites
active sites
bold verified ts mutant
-3.7
hydrophobicity (Kyte & Doolittle, 1982, J. Mol., 157, 105-132)
3.7

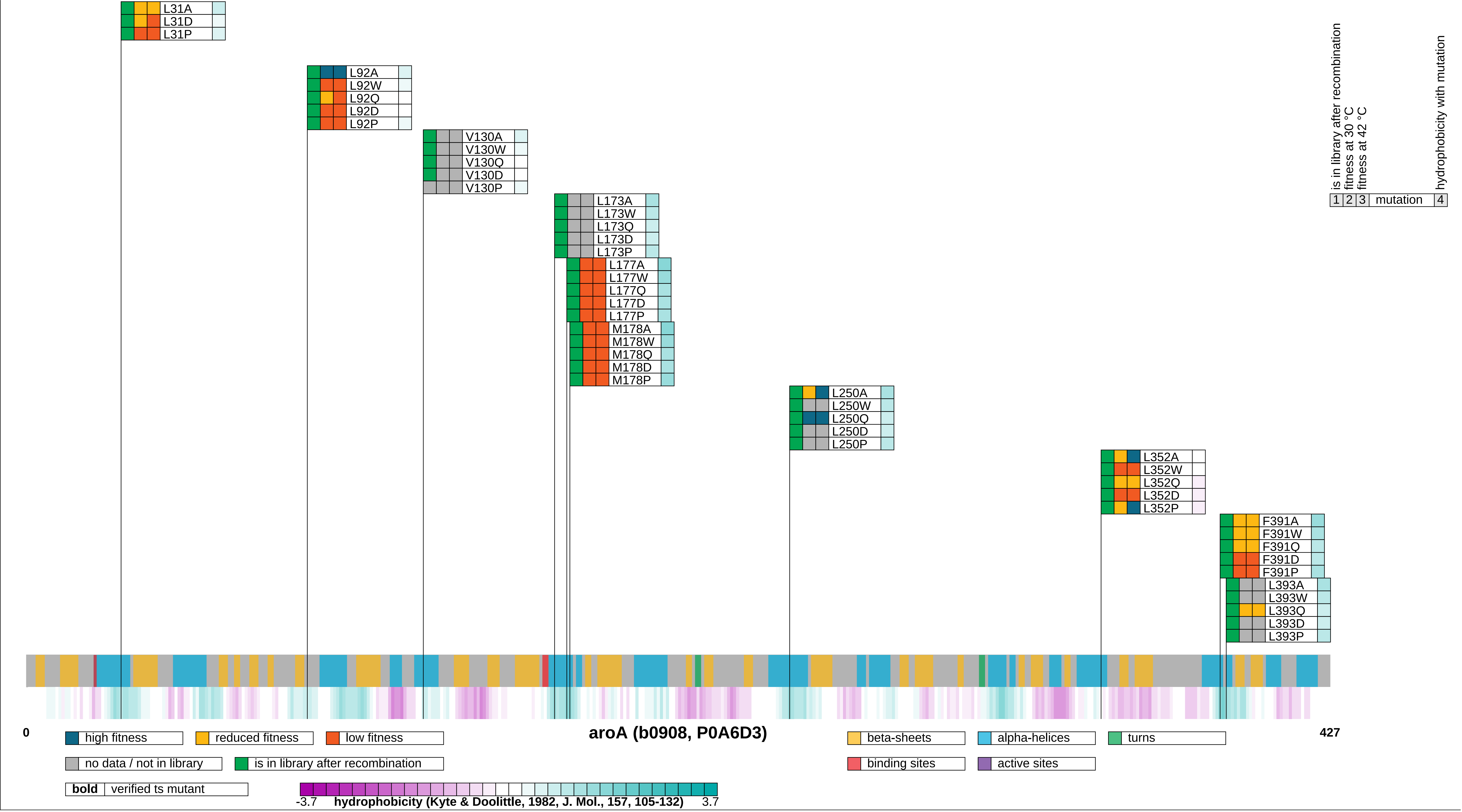

L31A
L31D
L31P
L92A
L92W
L92Q
L92D
L92P
V130A
V130W
V130Q
V130D
V130P
L173A
L173W
L173Q
L173D
L173P
L177A
L177W
L177Q
L177D
L177P
M178A
M178W
M178Q
M178D
M178P
L250A
L250W
L250Q
L250D
L250P
L352A
L352W
L352Q
L352D
L352P
F391A
F391W
F391Q
F391D
F391P
L393A
L393W
L393Q
L393D
L393P
is in library after recombination
fitness at 30 °C
fitness at 42 °C
hydrophobicity with mutation
1 2 3 mutation 4
0
aroA (b0908, P0A6D3)
427
high fitness
reduced fitness
low fitness
beta-sheets
alpha-helices
turns
no data / not in library
is in library after recombination
binding sites
active sites
bold verified ts mutant
-3.7 hydrophobicity (Kyte & Doolittle, 1982, J. Mol., 157, 105-132) 3.7

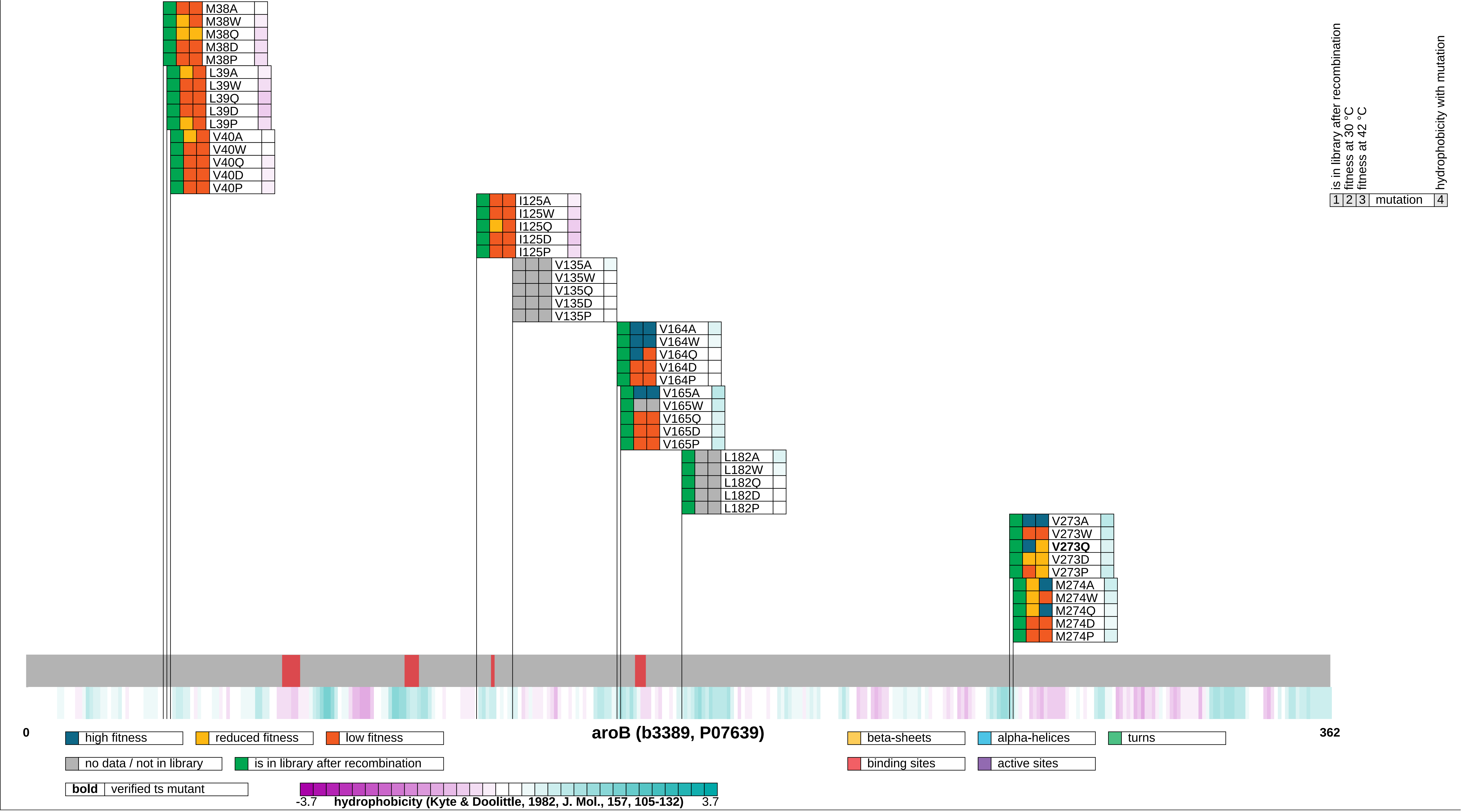
is in library after recombination
fitness at 30 °C
fitness at 42 °C
hydrophobicity with mutation
1 2 3 mutation 4
M38A
M38W
M38Q
M38D
M38P
L39A
L39W
L39Q
L39D
L39P
V40A
V40W
V40Q
V40D
V40P
I125A
I125W
I125Q
I125D
I125P
V135A
V135W
V135Q
V135D
V135P
V164A
V164W
V164Q
V164D
V164P
V165A
V165W
V165Q
V165D
V165P
L182A
L182W
L182Q
L182D
L182P
V273A
V273W
V273Q
V273D
V273P
M274A
M274W
M274Q
M274D
M274P
0
aroB (b3389, P07639)
362
high fitness
reduced fitness
low fitness
beta-sheets
alpha-helices
turns
no data / not in library
is in library after recombination
binding sites
active sites
bold verified ts mutant
-3.7
hydrophobicity (Kyte & Doolittle, 1982, J. Mol., 157, 105-132)
3.7

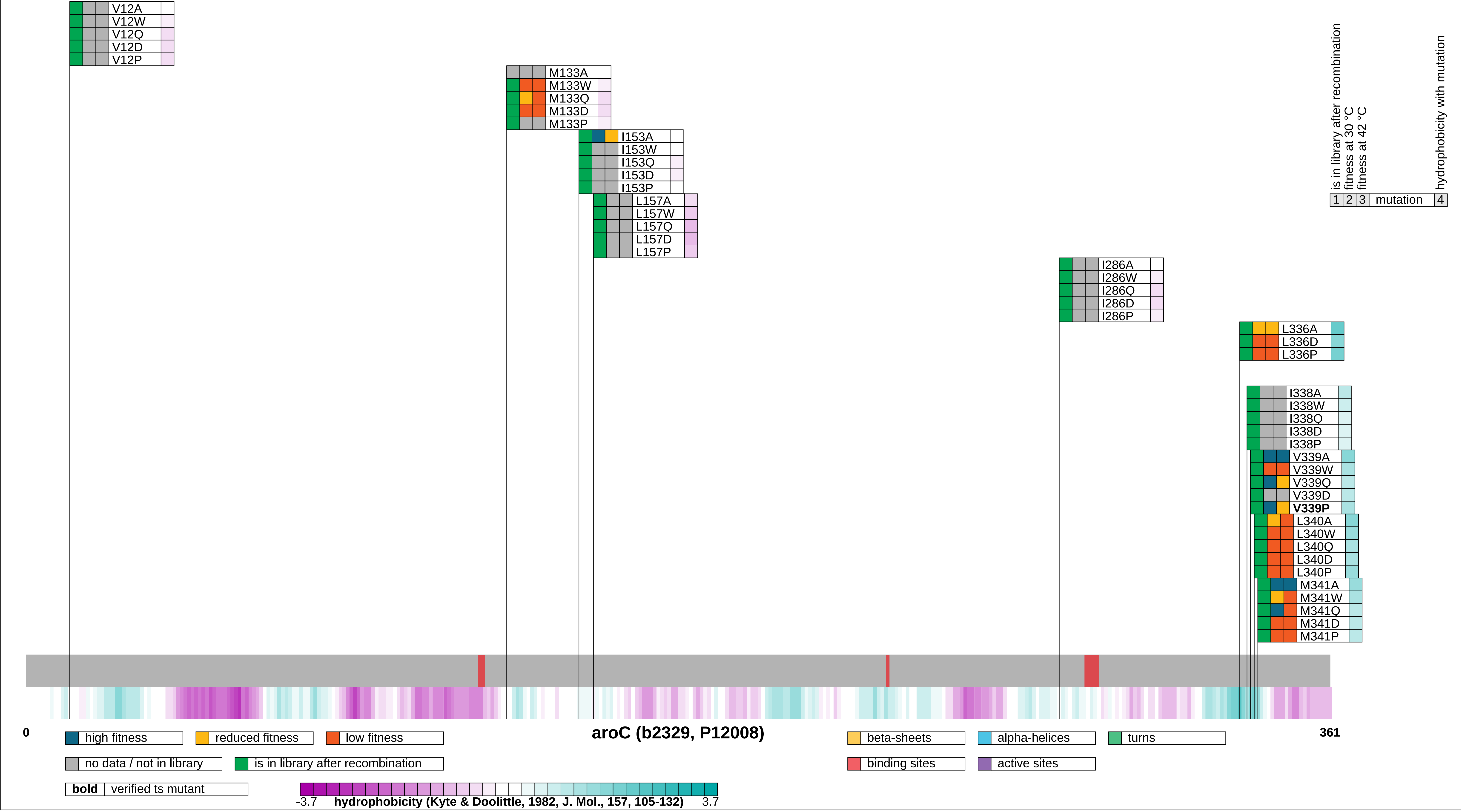

V12A
V12W
V12Q
V12D
V12P
M133A
M133W
M133Q
M133D
M133P
I153A
I153W
I153Q
I153D
I153P
L157A
L157W
L157Q
L157D
L157P
I286A
I286W
I286Q
I286D
I286P
L336A
L336D
L336P
I338A
I338W
I338Q
I338D
I338P
V339A
V339W
V339Q
V339D
V339P
L340A
L340W
L340Q
L340D
L340P
M341A
M341W
M341Q
M341D
M341P
is in library after recombination
fitness at 30 °C
fitness at 42 °C
hydrophobicity with mutation
1 2 3 mutation 4
0
aroC (b2329, P12008)
361
high fitness
reduced fitness
low fitness
beta-sheets
alpha-helices
turns
no data / not in library
is in library after recombination
binding sites
active sites
bold verified ts mutant
-3.7
hydrophobicity (Kyte & Doolittle, 1982, J. Mol., 157, 105-132)
3.7

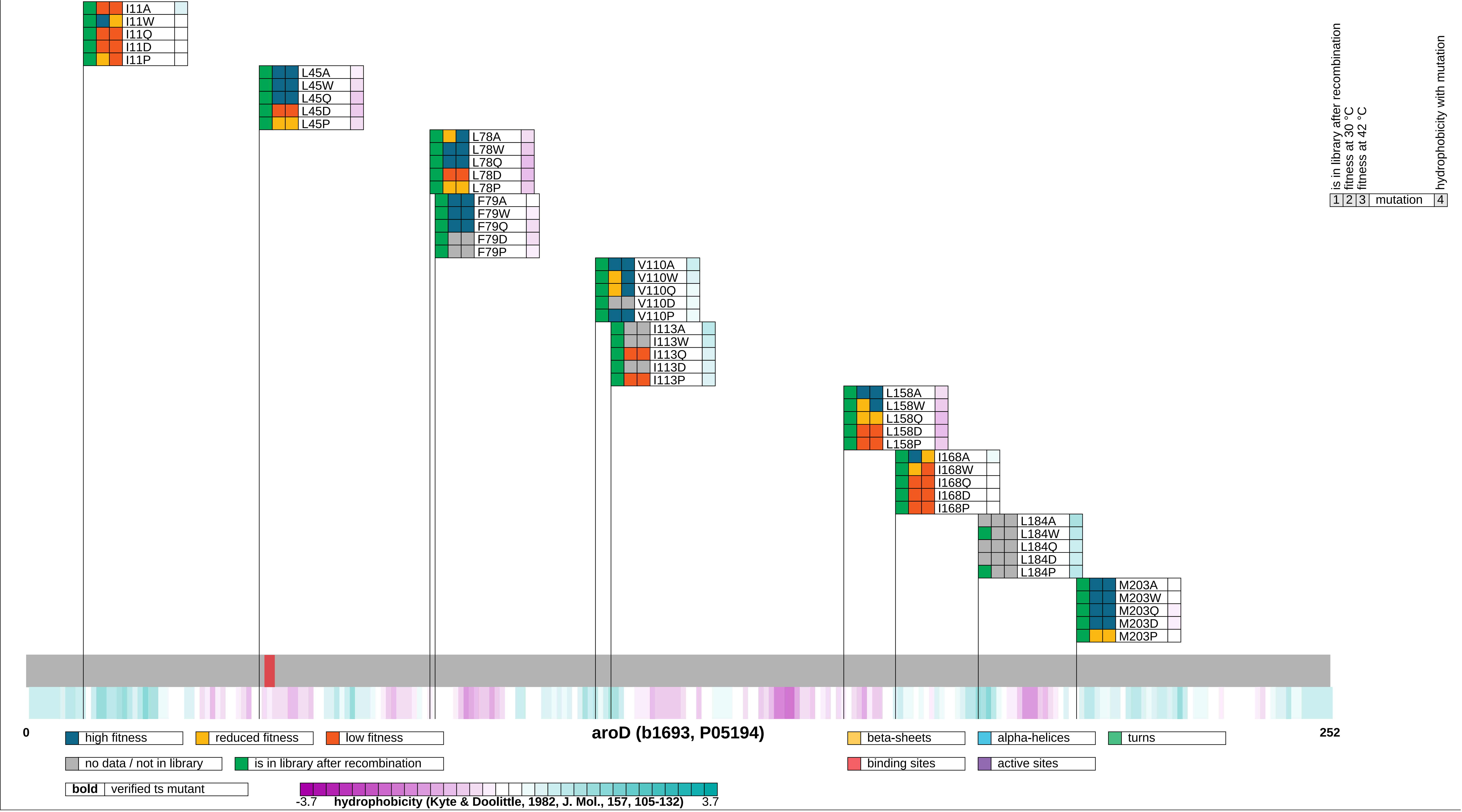

I11A
I11W
I11Q
I11D
I11P
L45A
L45W
L45Q
L45D
L45P
L78A
L78W
L78Q
L78D
L78P
F79A
F79W
F79Q
F79D
F79P
V110A
V110W
V110Q
V110D
V110P
I113A
I113W
I113Q
I113D
I113P
L158A
L158W
L158Q
L158D
L158P
I168A
I168W
I168Q
I168D
I168P
L184A
L184W
L184Q
L184D
L184P
M203A
M203W
M203Q
M203D
M203P
is in library after recombination
fitness at 30 °C
fitness at 42 °C
hydrophobicity with mutation
1 2 3 mutation 4
0
aroD (b1693, P05194)
252
high fitness
reduced fitness
low fitness
no data / not in library
is in library after recombination
bold verified ts mutant
beta-sheets
alpha-helices
turns
binding sites
active sites
-3.7
hydrophobicity (Kyte & Doolittle, 1982, J. Mol., 157, 105-132)
3.7

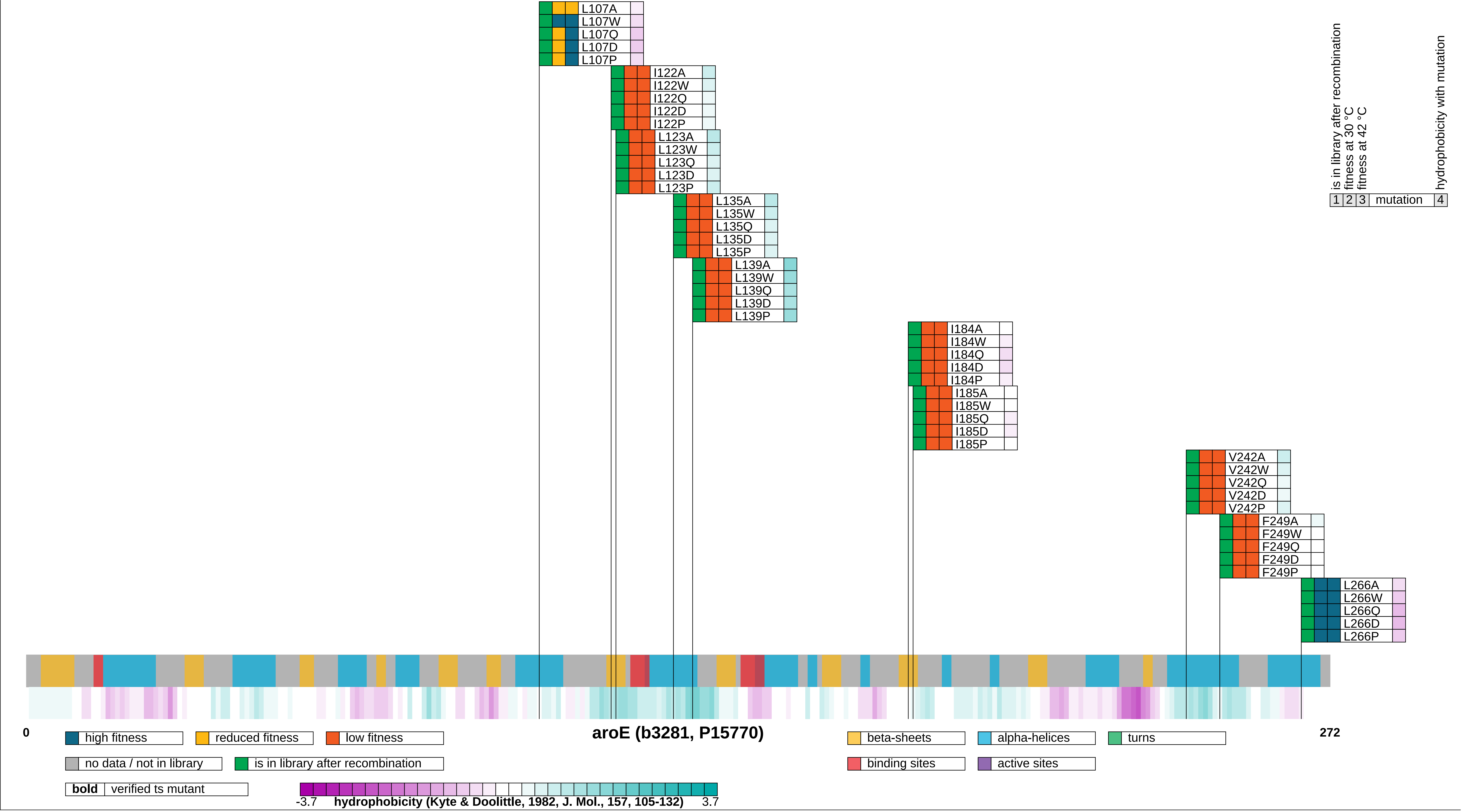

L107A
L107W
L107Q
L107D
L107P
I122A
I122W
I122Q
I122D
I122P
L123A
L123W
L123Q
L123D
L123P
L135A
L135W
L135Q
L135D
L135P
L139A
L139W
L139Q
L139D
L139P
I184A
I184W
I184Q
I184D
I184P
I185A
I185W
I185Q
I185D
I185P
V242A
V242W
V242Q
V242D
V242P
F249A
F249W
F249Q
F249D
F249P
L266A
L266W
L266Q
L266D
L266P
1 is in library after recombination
2 fitness at 30 °C
3 fitness at 42 °C
mutation
4 hydrophobicity with mutation
0
aroE (b3281, P15770)
272
high fitness
reduced fitness
low fitness
beta-sheets
alpha-helices
turns
no data / not in library
is in library after recombination
binding sites
active sites
bold verified ts mutant
-3.7
hydrophobicity (Kyte & Doolittle, 1982, J. Mol., 157, 105-132)
3.7

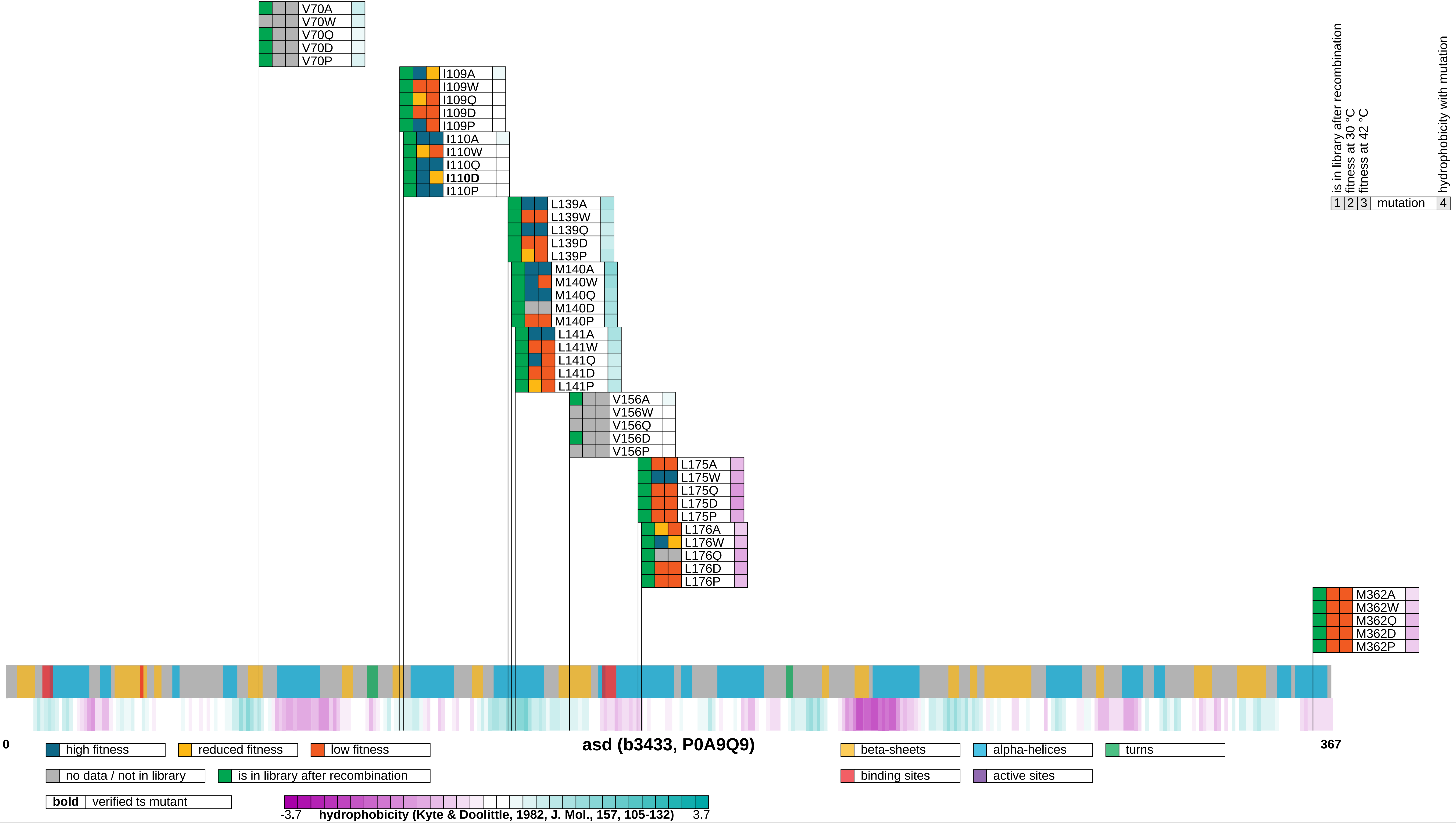
V70A
V70W
V70Q
V70D
V70P
I109A
I109W
I109Q
I109D
I109P
I110A
I110W
I110Q
I110D
I110P
L139A
L139W
L139Q
L139D
L139P
M140A
M140W
M140Q
M140D
M140P
L141A
L141W
L141Q
L141D
L141P
V156A
V156W
V156Q
V156D
V156P
L175A
L175W
L175Q
L175D
L175P
L176A
L176W
L176Q
L176D
L176P
M362A
M362W
M362Q
M362D
M362P
is in library after recombination
fitness at 30 °C
fitness at 42 °C
hydrophobicity with mutation
1 2 3 mutation 4
0
asd (b3433, P0A9Q9)
367
high fitness
reduced fitness
low fitness
no data / not in library
is in library after recombination
bold verified ts mutant
beta-sheets
alpha-helices
turns
binding sites
active sites
-3.7
hydrophobicity (Kyte & Doolittle, 1982, J. Mol., 157, 105-132)
3.7

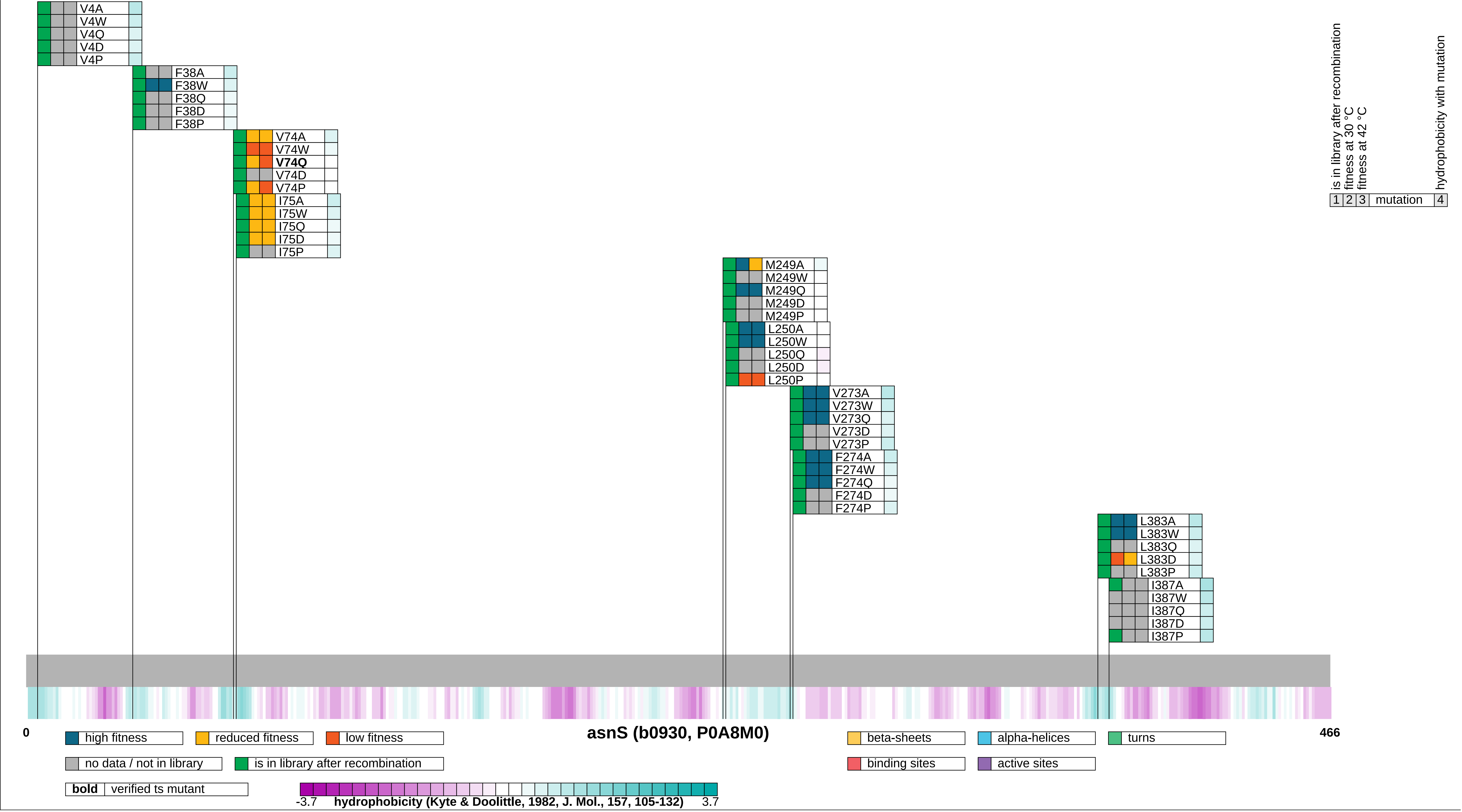

V4A
V4W
V4Q
V4D
V4P
F38A
F38W
F38Q
F38D
F38P
V74A
V74W
V74Q
V74D
V74P
I75A
I75W
I75Q
I75D
I75P
M249A
M249W
M249Q
M249D
M249P
L250A
L250W
L250Q
L250D
L250P
V273A
V273W
V273Q
V273D
V273P
F274A
F274W
F274Q
F274D
F274P
L383A
L383W
L383Q
L383D
L383P
I387A
I387W
I387Q
I387D
I387P
is in library after recombination
fitness at 30 °C
fitness at 42 °C
hydrophobicity with mutation
1 2 3 mutation 4
0
asnS (b0930, P0A8M0)
466
high fitness
reduced fitness
low fitness
beta-sheets
alpha-helices
turns
no data / not in library
is in library after recombination
binding sites
active sites
bold verified ts mutant
-3.7 hydrophobicity (Kyte & Doolittle, 1982, J. Mol., 157, 105-132) 3.7

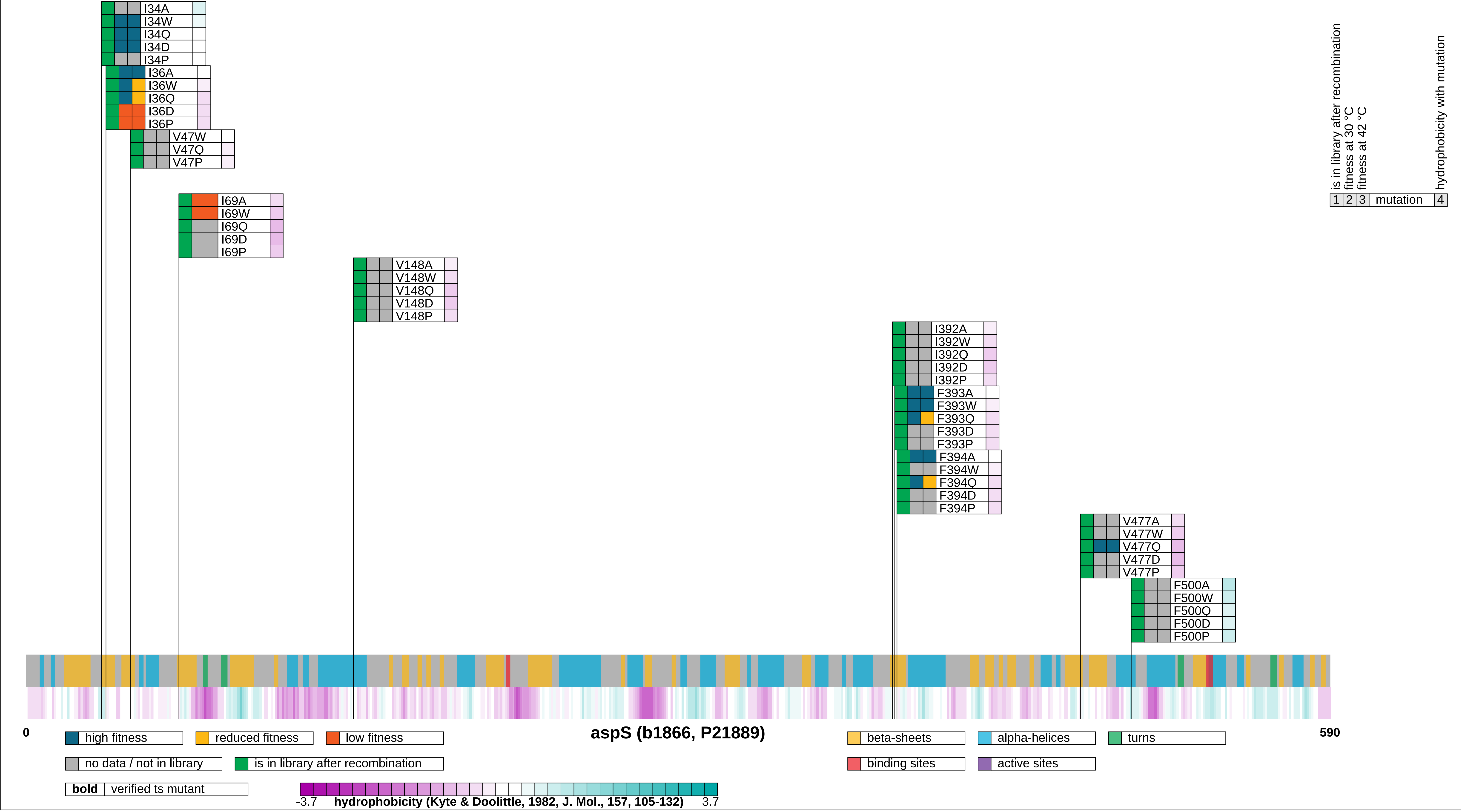

I34A
I34W
I34Q
I34D
I34P
I36A
I36W
I36Q
I36D
I36P
V47W
V47Q
V47P
I69A
I69W
I69Q
I69D
I69P
V148A
V148W
V148Q
V148D
V148P
I392A
I392W
I392Q
I392D
I392P
F393A
F393W
F393Q
F393D
F393P
F394A
F394W
F394Q
F394D
F394P
V477A
V477W
V477Q
V477D
V477P
F500A
F500W
F500Q
F500D
F500P
is in library after recombination
fitness at 30 °C
fitness at 42 °C
hydrophobicity with mutation
1 2 3 mutation 4
0
aspS (b1866, P21889)
590
high fitness
reduced fitness
low fitness
no data / not in library
is in library after recombination
bold verified ts mutant
beta-sheets
alpha-helices
turns
binding sites
active sites
-3.7 hydrophobicity (Kyte & Doolittle, 1982, J. Mol., 157, 105-132) 3.7

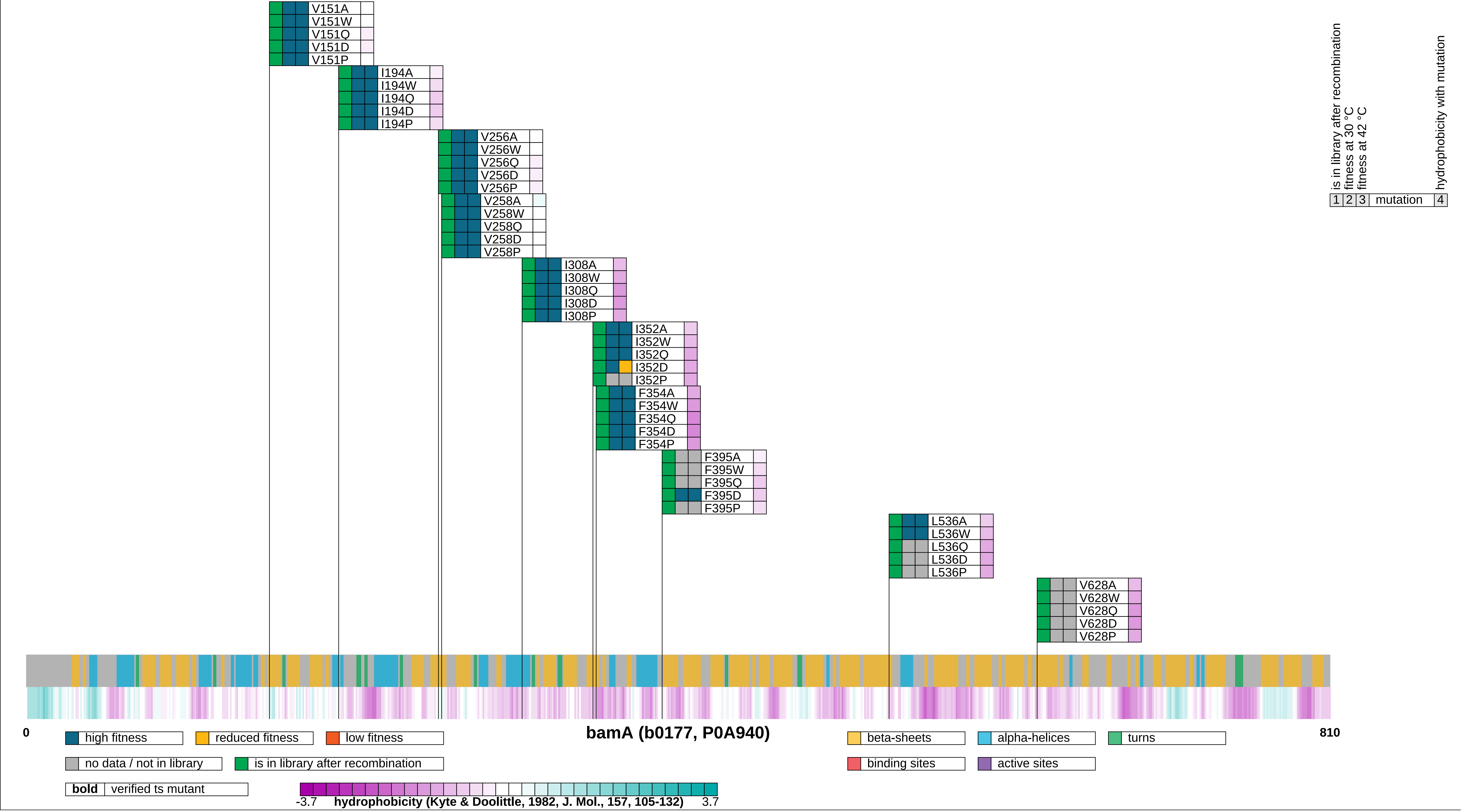
V151A
V151W
V151Q
V151D
V151P
I194A
I194W
I194Q
I194D
I194P
V256A
V256W
V256Q
V256D
V256P
V258A
V258W
V258Q
V258D
V258P
I308A
I308W
I308Q
I308D
I308P
I352A
I352W
I352Q
I352D
I352P
F354A
F354W
F354Q
F354D
F354P
F395A
F395W
F395Q
F395D
F395P
L536A
L536W
L536Q
L536D
L536P
V628A
V628W
V628Q
V628D
V628P
is in library after recombination
fitness at 30 °C
fitness at 42 °C
hydrophobicity with mutation
1 2 3 mutation 4
0
bamA (b0177, P0A940)
810
high fitness
reduced fitness
low fitness
beta-sheets
alpha-helices
turns
no data / not in library
is in library after recombination
binding sites
active sites
bold verified ts mutant
-3.7 hydrophobicity (Kyte & Doolittle, 1982, J. Mol., 157, 105-132) 3.7

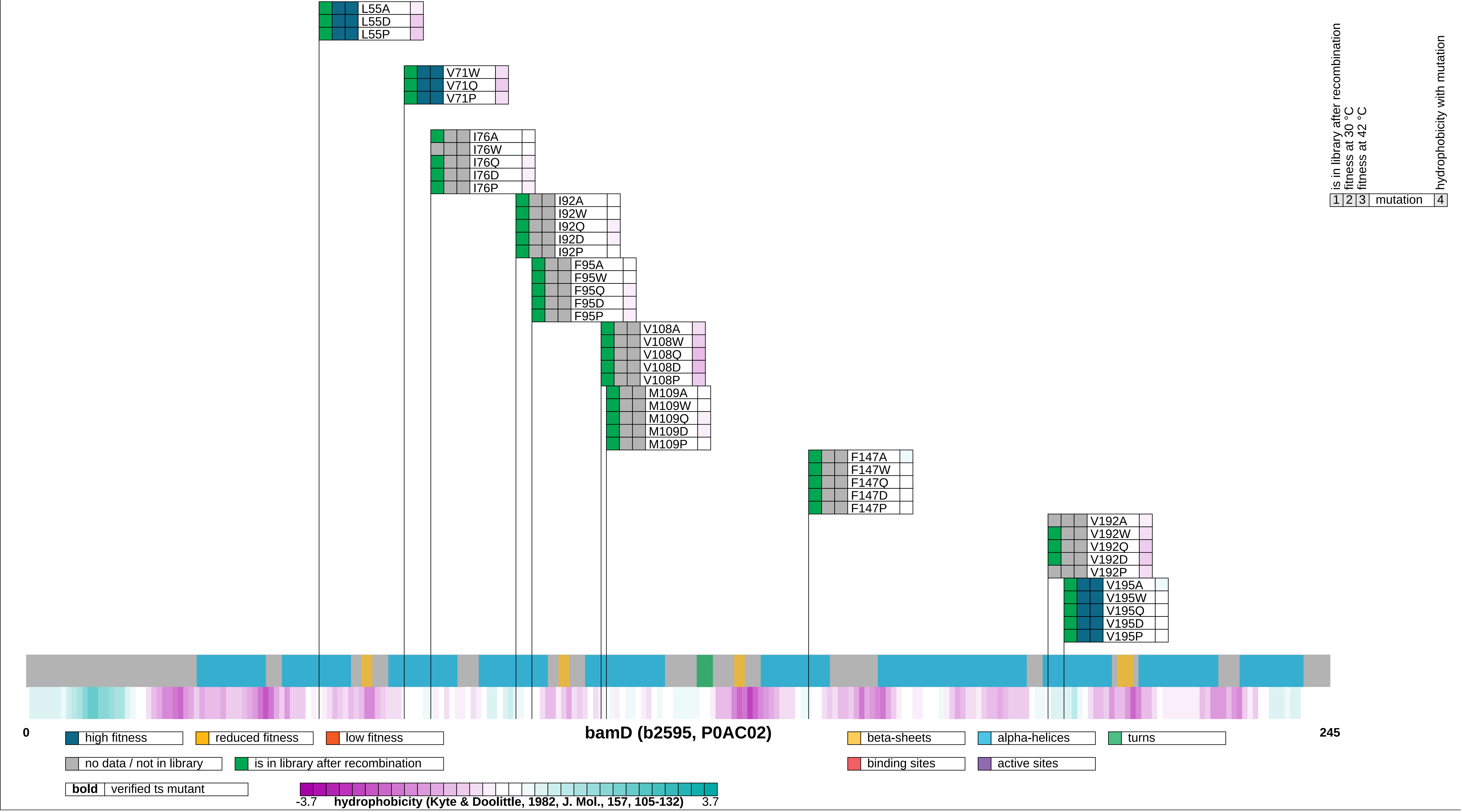

L55A
L55D
L55P
V71W
V71Q
V71P
I76A
I76W
I76Q
I76D
I76P
I92A
I92W
I92Q
I92D
I92P
F95A
F95W
F95Q
F95D
F95P
V108A
V108W
V108Q
V108D
V108P
M109A
M109W
M109Q
M109D
M109P
F147A
F147W
F147Q
F147D
F147P
V192A
V192W
V192Q
V192D
V192P
V195A
V195W
V195Q
V195D
V195P
is in library after recombination
fitness at 30 °C
fitness at 42 °C
hydrophobicity with mutation
1 2 3 mutation 4
0
bamD (b2595, P0AC02)
245
high fitness
reduced fitness
low fitness
beta-sheets
alpha-helices
turns
no data / not in library
is in library after recombination
binding sites
active sites
bold verified ts mutant
-3.7 hydrophobicity (Kyte & Doolittle, 1982, J. Mol., 157, 105-132) 3.7

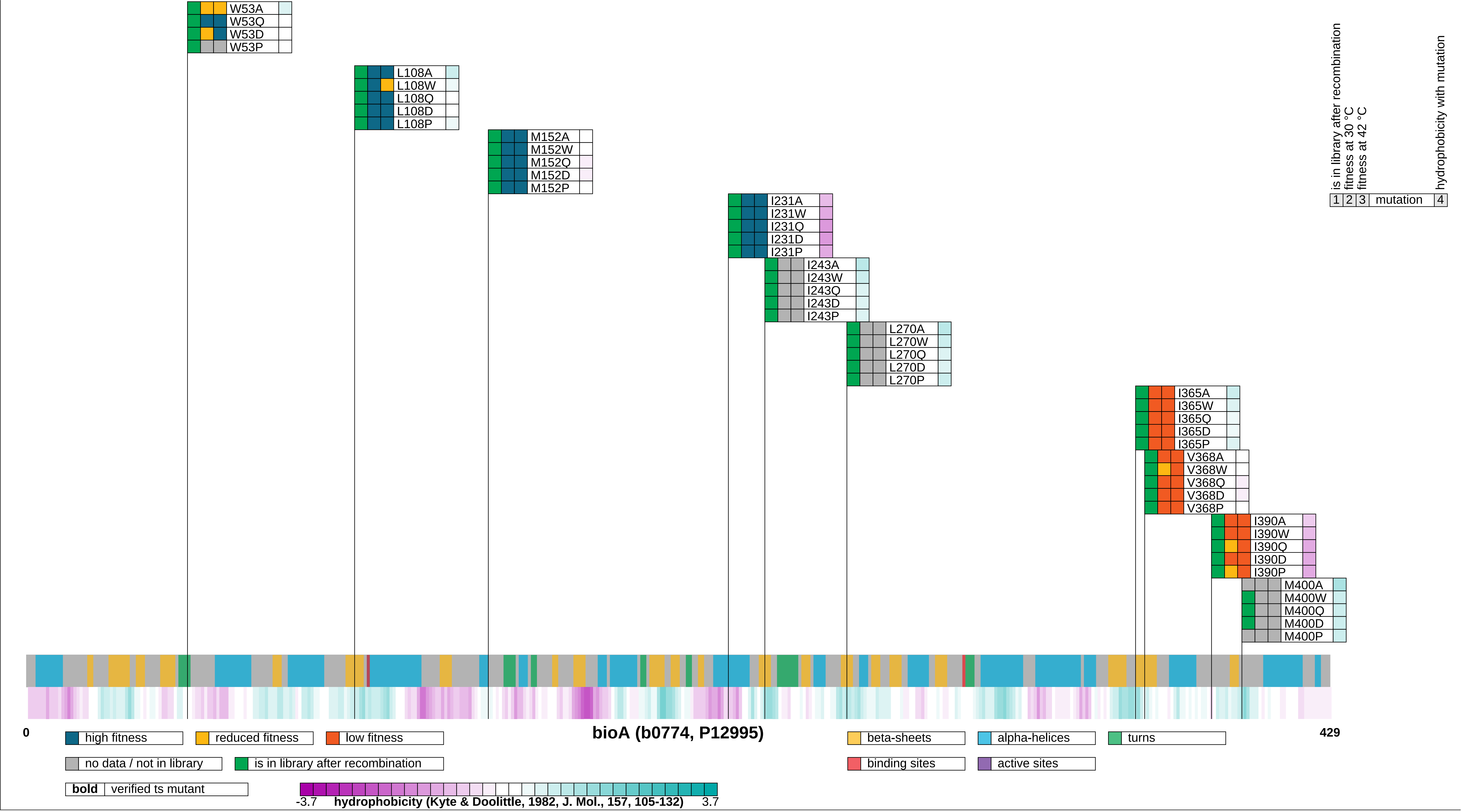

W53A
W53Q
W53D
W53P
L108A
L108W
L108Q
L108D
L108P
M152A
M152W
M152Q
M152D
M152P
I231A
I231W
I231Q
I231D
I231P
I243A
I243W
I243Q
I243D
I243P
L270A
L270W
L270Q
L270D
L270P
I365A
I365W
I365Q
I365D
I365P
V368A
V368W
V368Q
V368D
V368P
I390A
I390W
I390Q
I390D
I390P
M400A
M400W
M400Q
M400D
M400P
is in library after recombination
fitness at 30 °C
fitness at 42 °C
hydrophobicity with mutation
1 2 3 mutation 4
0
bioA (b0774, P12995)
429
high fitness
reduced fitness
low fitness
beta-sheets
alpha-helices
turns
no data / not in library
is in library after recombination
binding sites
active sites
bold verified ts mutant
-3.7
hydrophobicity (Kyte & Doolittle, 1982, J. Mol., 157, 105-132)
3.7

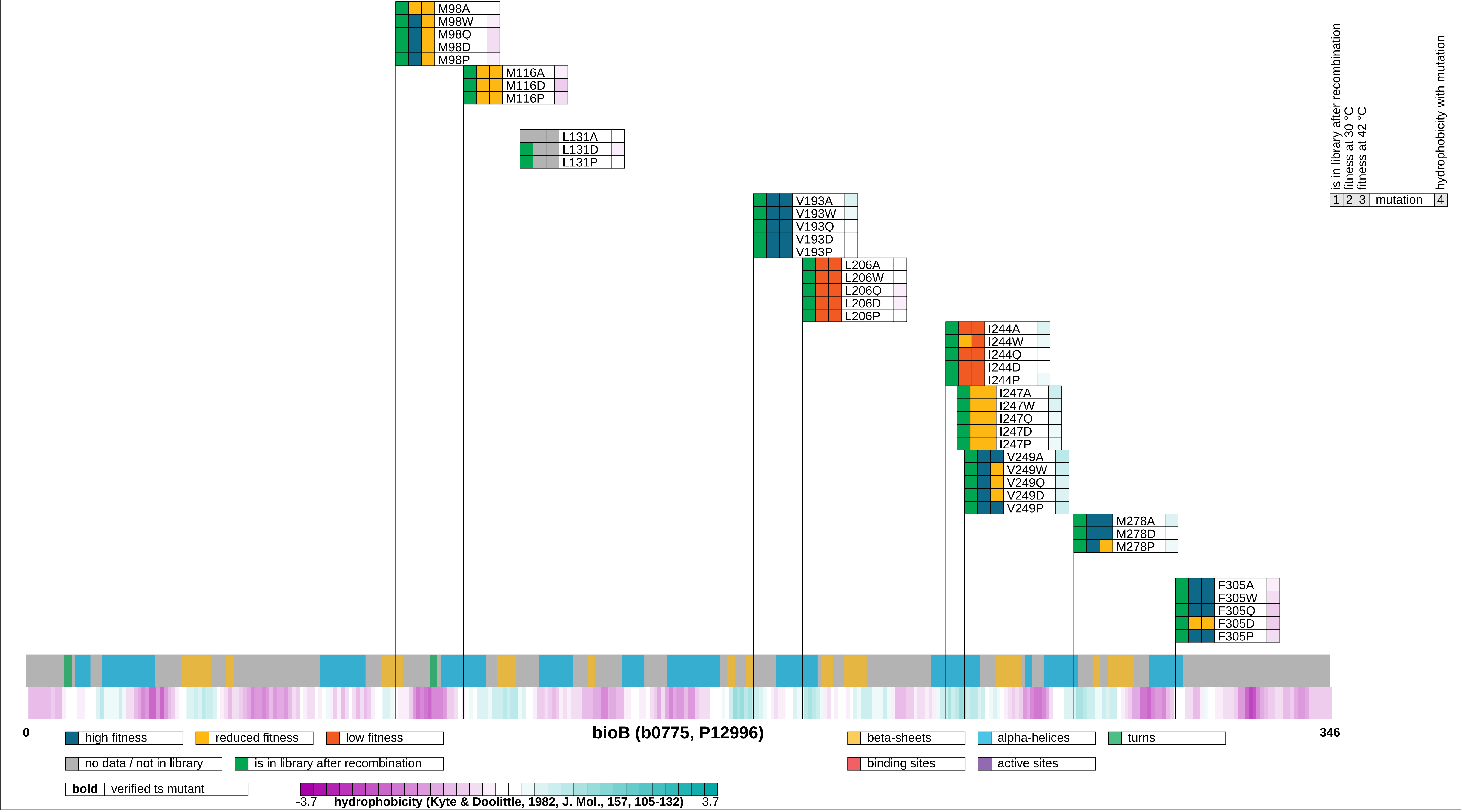

M98A
M98W
M98Q
M98D
M98P
M116A
M116D
M116P
L131A
L131D
L131P
V193A
V193W
V193Q
V193D
V193P
L206A
L206W
L206Q
L206D
L206P
I244A
I244W
I244Q
I244D
I244P
I247A
I247W
I247Q
I247D
I247P
V249A
V249W
V249Q
V249D
V249P
M278A
M278D
M278P
F305A
F305W
F305Q
F305D
F305P
is in library after recombination
fitness at 30 °C
fitness at 42 °C
hydrophobicity with mutation
1 2 3 mutation 4
0
bioB (b0775, P12996)
346
high fitness
reduced fitness
low fitness
no data / not in library
is in library after recombination
beta-sheets
alpha-helices
turns
binding sites
active sites
bold verified ts mutant
-3.7
hydrophobicity (Kyte & Doolittle, 1982, J. Mol., 157, 105-132)
3.7

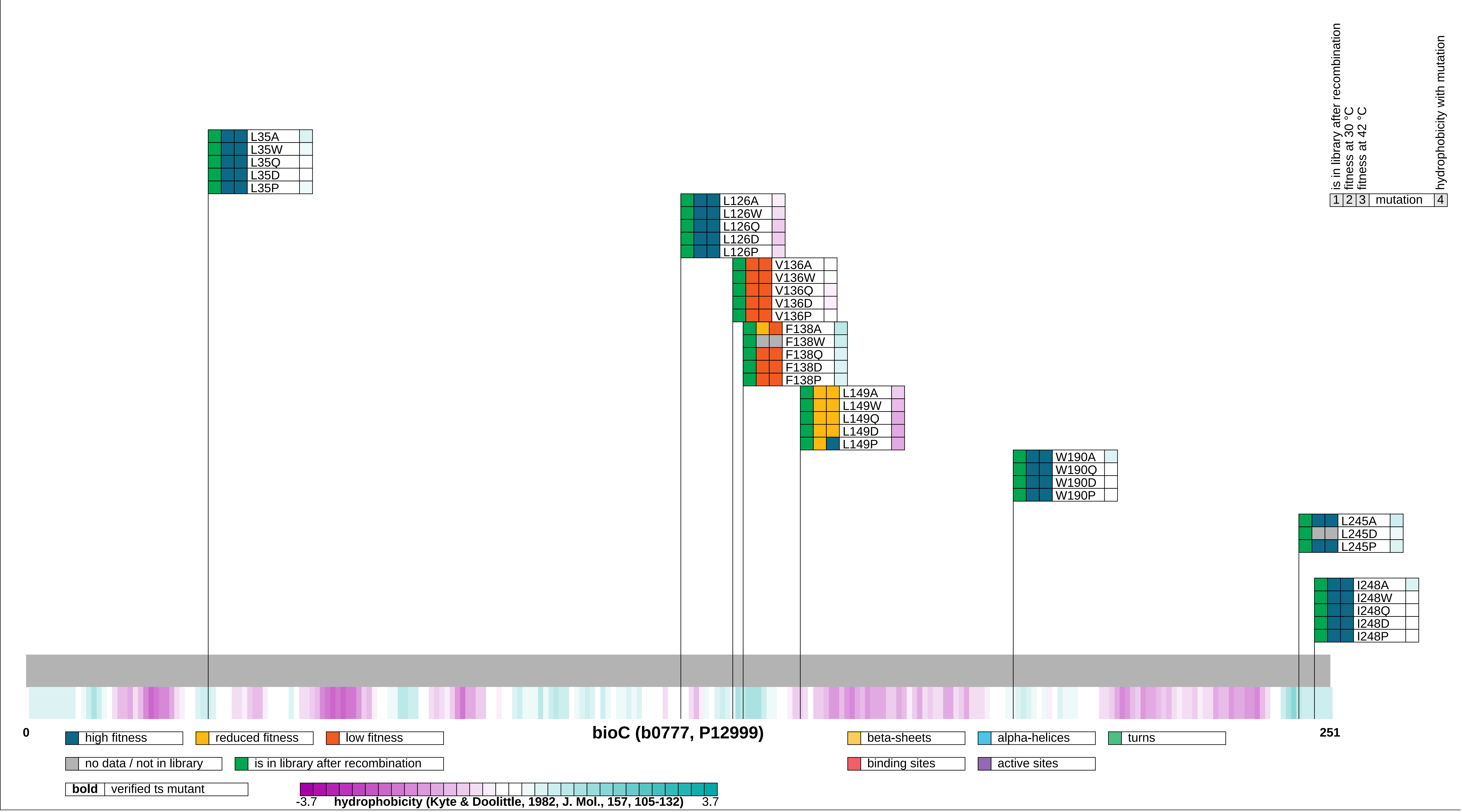
is in library after recombination
fitness at 30 °C
fitness at 42 °C
hydrophobicity with mutation
1 2 3 mutation 4
L35A
L35W
L35Q
L35D
L35P
L126A
L126W
L126Q
L126D
L126P
V136A
V136W
V136Q
V136D
V136P
F138A
F138W
F138Q
F138D
F138P
L149A
L149W
L149Q
L149D
L149P
W190A
W190Q
W190D
W190P
L245A
L245D
L245P
I248A
I248W
I248Q
I248D
I248P
0
bioC (b0777, P12999)
251
high fitness
reduced fitness
low fitness
beta-sheets
alpha-helices
turns
no data / not in library
is in library after recombination
binding sites
active sites
bold verified ts mutant
-3.7
hydrophobicity (Kyte & Doolittle, 1982, J. Mol., 157, 105-132)
3.7

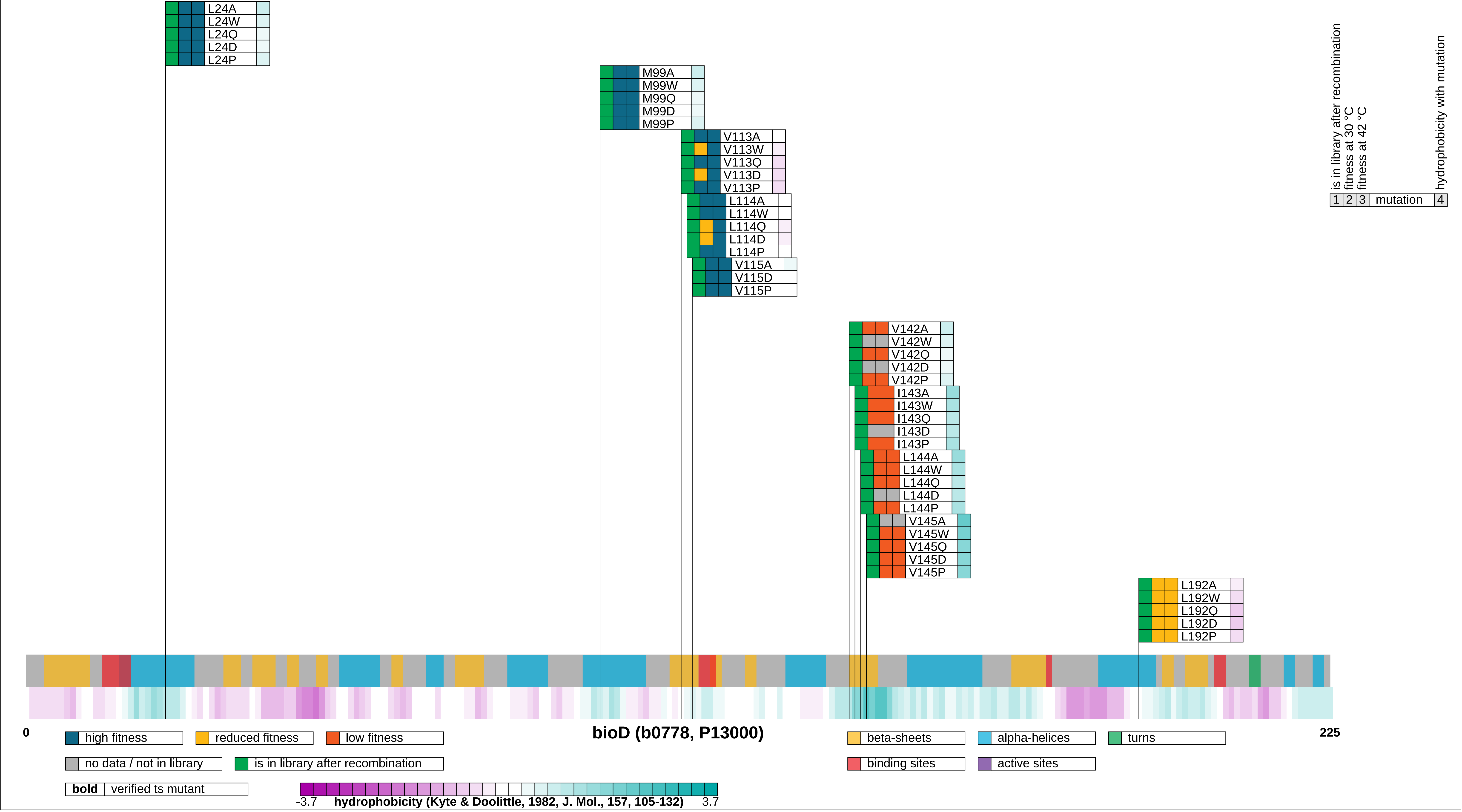

L24A
L24W
L24Q
L24D
L24P
M99A
M99W
M99Q
M99D
M99P
V113A
V113W
V113Q
V113D
V113P
L114A
L114W
L114Q
L114D
L114P
V115A
V115D
V115P
V142A
V142W
V142Q
V142D
V142P
I143A
I143W
I143Q
I143D
I143P
L144A
L144W
L144Q
L144D
L144P
V145A
V145W
V145Q
V145D
V145P
L192A
L192W
L192Q
L192D
L192P
is in library after recombination
fitness at 30 °C
fitness at 42 °C
hydrophobicity with mutation
1
2
3
mutation
4
0
bioD (b0778, P13000)
225
high fitness
reduced fitness
low fitness
no data / not in library
is in library after recombination
bold
verified ts mutant
beta-sheets
alpha-helices
turns
binding sites
active sites
-3.7
hydrophobicity (Kyte & Doolittle, 1982, J. Mol., 157, 105-132)
3.7

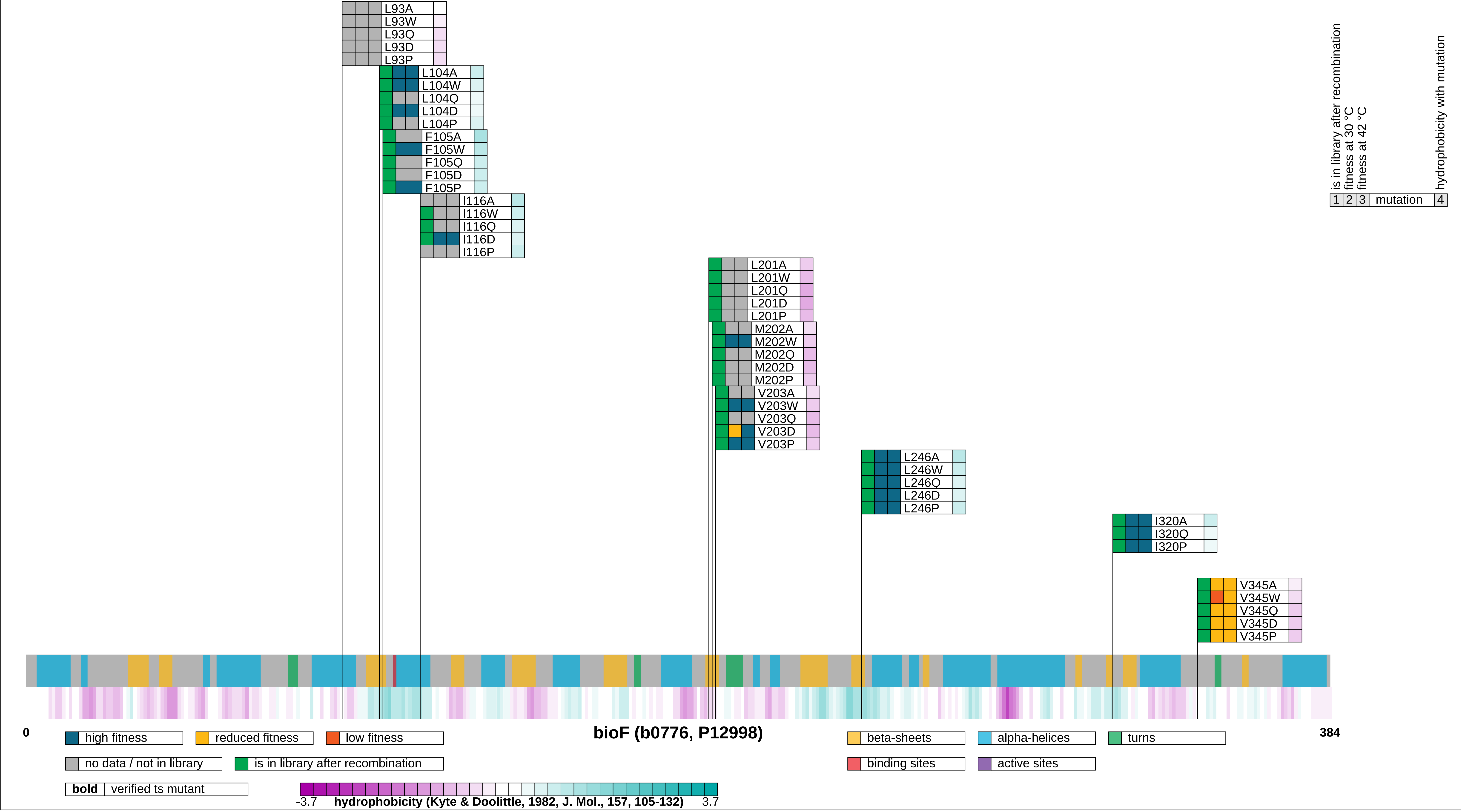
L93A
L93W
L93Q
L93D
L93P
L104A
L104W
L104Q
L104D
L104P
F105A
F105W
F105Q
F105D
F105P
I116A
I116W
I116Q
I116D
I116P
L201A
L201W
L201Q
L201D
L201P
M202A
M202W
M202Q
M202D
M202P
V203A
V203W
V203Q
V203D
V203P
L246A
L246W
L246Q
L246D
L246P
I320A
I320Q
I320P
V345A
V345W
V345Q
V345D
V345P
is in library after recombination
fitness at 30 °C
fitness at 42 °C
hydrophobicity with mutation
1 2 3 mutation 4
0
bioF (b0776, P12998)
384
high fitness
reduced fitness
low fitness
no data / not in library
is in library after recombination
bold verified ts mutant
beta-sheets
alpha-helices
turns
binding sites
active sites
-3.7 hydrophobicity (Kyte & Doolittle, 1982, J. Mol., 157, 105-132) 3.7

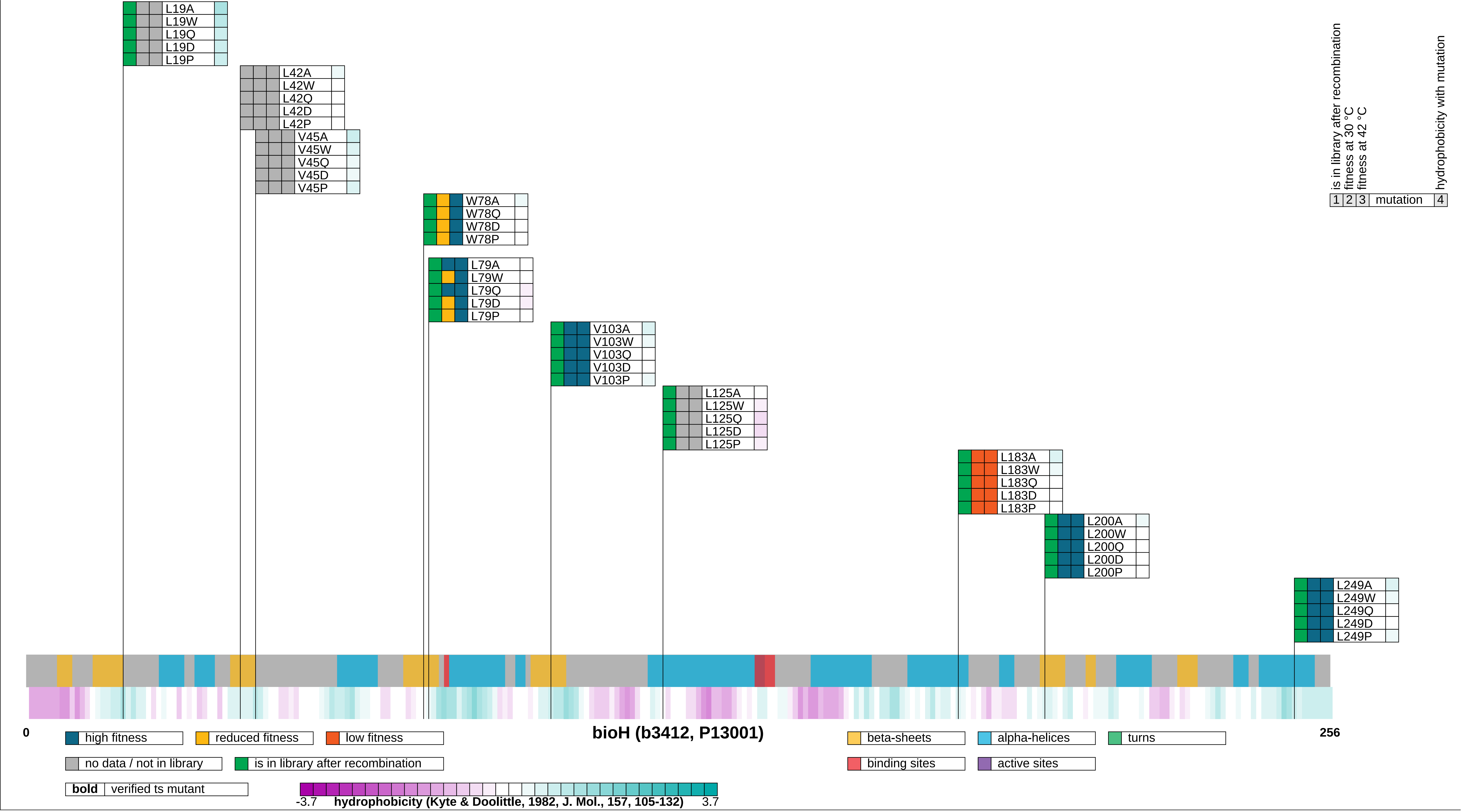

L19A
L19W
L19Q
L19D
L19P
L42A
L42W
L42Q
L42D
L42P
V45A
V45W
V45Q
V45D
V45P
W78A
W78Q
W78D
W78P
L79A
L79W
L79Q
L79D
L79P
V103A
V103W
V103Q
V103D
V103P
L125A
L125W
L125Q
L125D
L125P
L183A
L183W
L183Q
L183D
L183P
L200A
L200W
L200Q
L200D
L200P
L249A
L249W
L249Q
L249D
L249P
is in library after recombination
fitness at 30 °C
fitness at 42 °C
hydrophobicity with mutation
1
2
3
mutation
4
0
bioH (b3412, P13001)
256
high fitness
reduced fitness
low fitness
no data / not in library
is in library after recombination
bold
verified ts mutant
beta-sheets
alpha-helices
turns
binding sites
active sites
-3.7
hydrophobicity (Kyte & Doolittle, 1982, J. Mol., 157, 105-132)
3.7

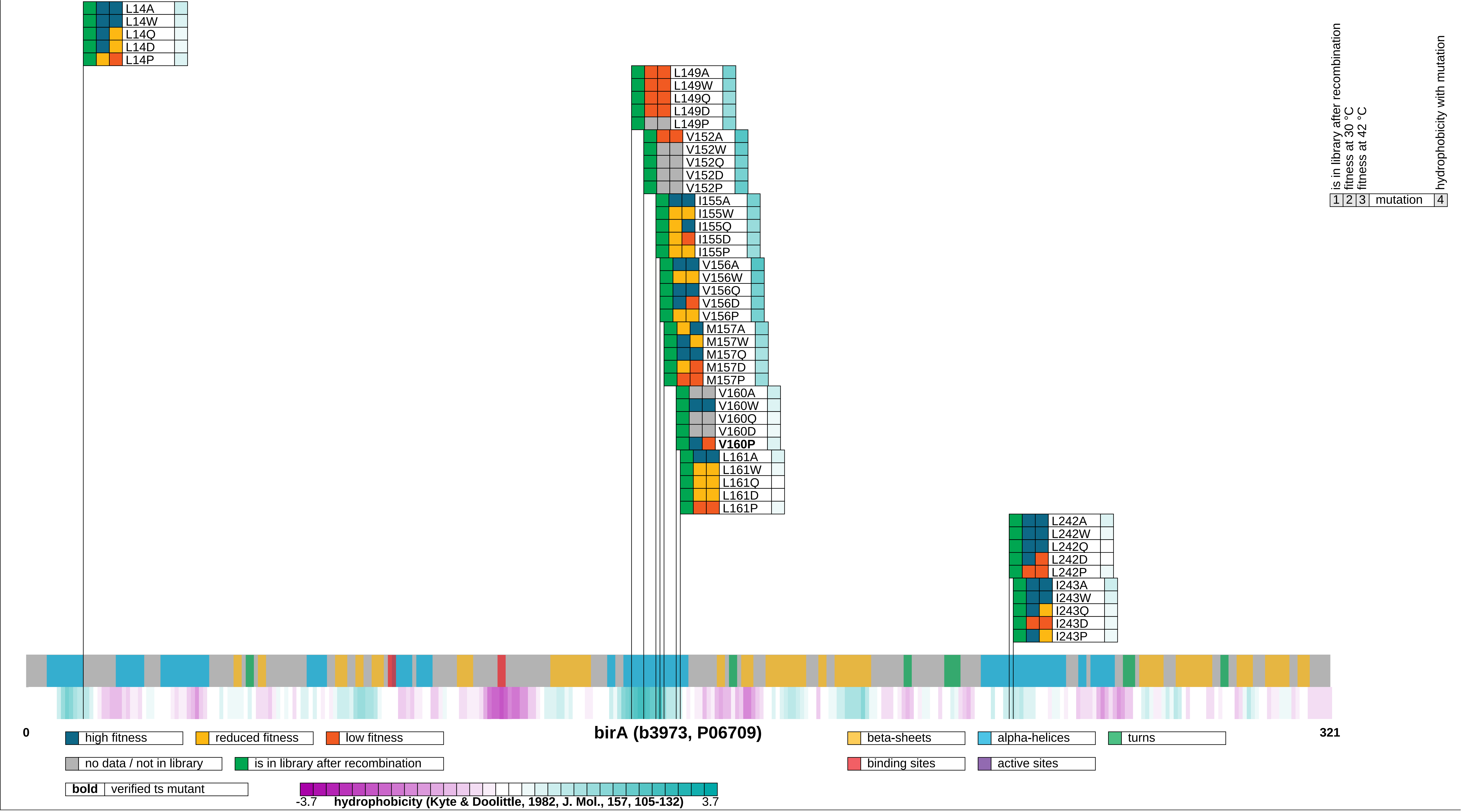
L14A
L14W
L14Q
L14D
L14P
L149A
L149W
L149Q
L149D
L149P
V152A
V152W
V152Q
V152D
V152P
I155A
I155W
I155Q
I155D
I155P
V156A
V156W
V156Q
V156D
V156P
M157A
M157W
M157Q
M157D
M157P
V160A
V160W
V160Q
V160D
V160P
L161A
L161W
L161Q
L161D
L161P
L242A
L242W
L242Q
L242D
L242P
I243A
I243W
I243Q
I243D
I243P
is in library after recombination
fitness at 30 °C
fitness at 42 °C
hydrophobicity with mutation
1 2 3 mutation 4
0
birA (b3973, P06709)
321
high fitness
reduced fitness
low fitness
beta-sheets
alpha-helices
turns
no data / not in library
is in library after recombination
binding sites
active sites
bold verified ts mutant
-3.7 hydrophobicity (Kyte & Doolittle, 1982, J. Mol., 157, 105-132) 3.7

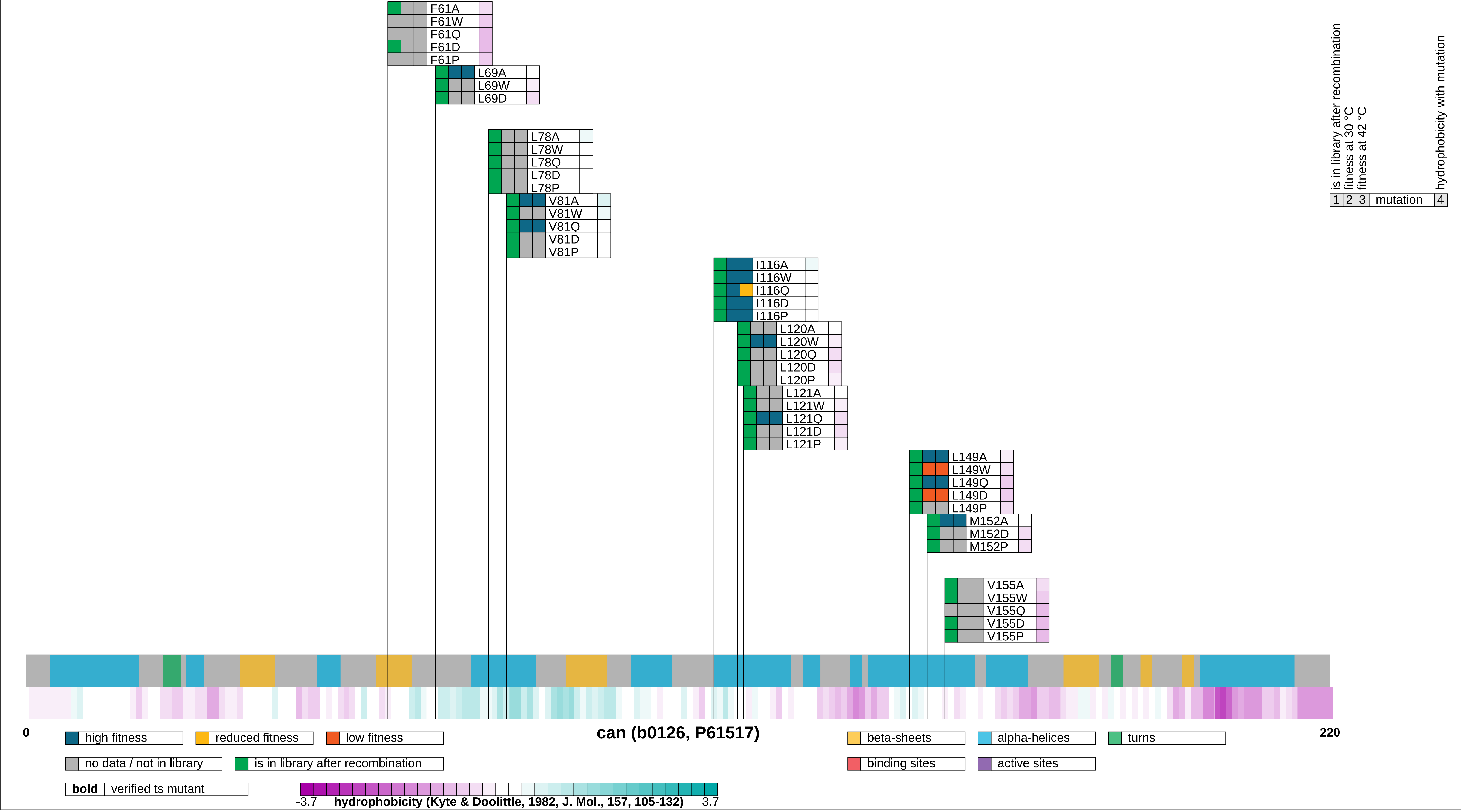

F61A
F61W
F61Q
F61D
F61P
L69A
L69W
L69D
L78A
L78W
L78Q
L78D
L78P
V81A
V81W
V81Q
V81D
V81P
I116A
I116W
I116Q
I116D
I116P
L120A
L120W
L120Q
L120D
L120P
L121A
L121W
L121Q
L121D
L121P
L149A
L149W
L149Q
L149D
L149P
M152A
M152D
M152P
V155A
V155W
V155Q
V155D
V155P
is in library after recombination
fitness at 30 °C
fitness at 42 °C
hydrophobicity with mutation
1 2 3 mutation 4
0
can (b0126, P61517)
220
high fitness
reduced fitness
low fitness
beta-sheets
alpha-helices
turns
no data / not in library
is in library after recombination
binding sites
active sites
bold verified ts mutant
-3.7
hydrophobicity (Kyte & Doolittle, 1982, J. Mol., 157, 105-132)
3.7

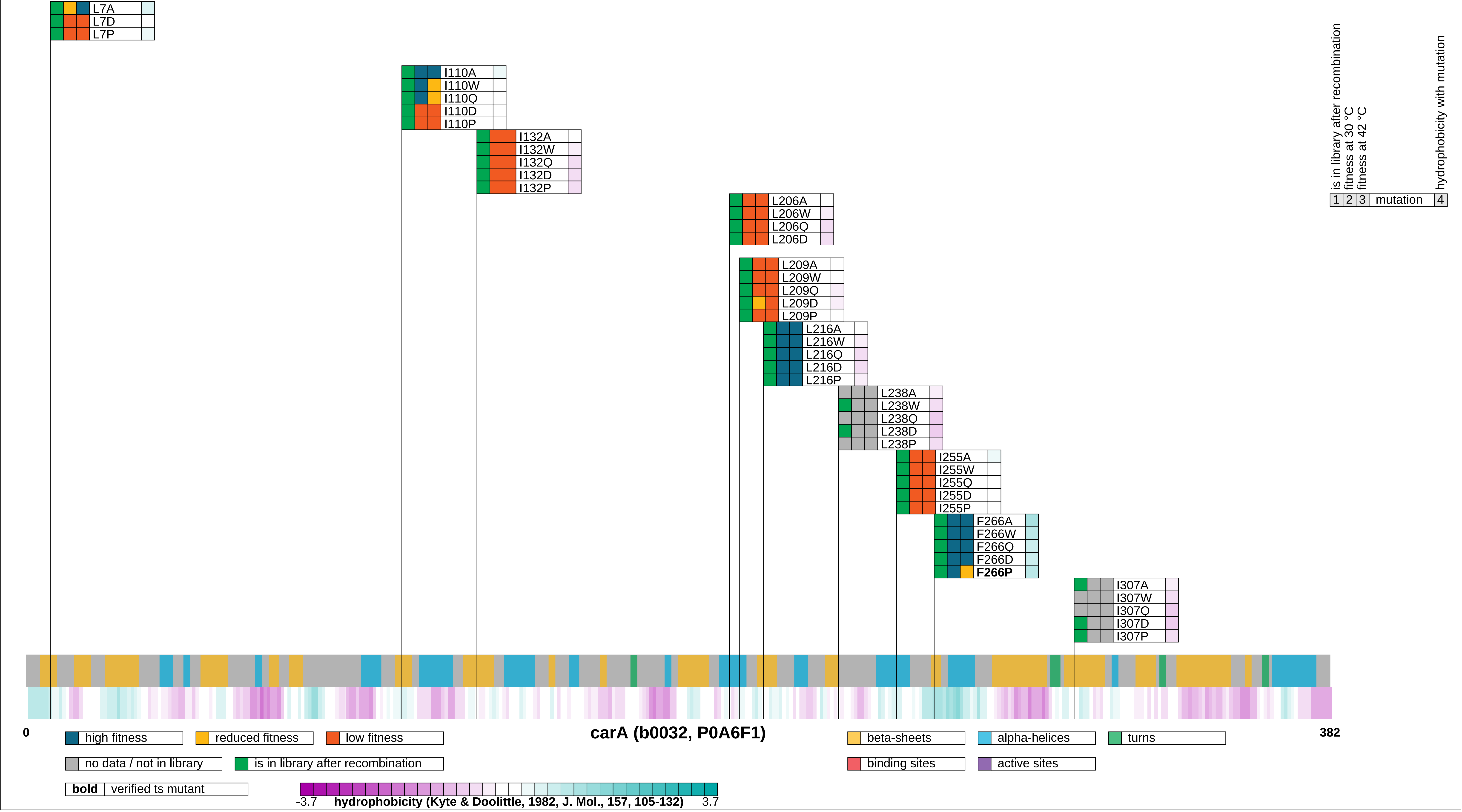

is in library after recombination
fitness at 30 °C
fitness at 42 °C
hydrophobicity with mutation
1 2 3 mutation 4
L7A
L7D
L7P
I110A
I110W
I110Q
I110D
I110P
I132A
I132W
I132Q
I132D
I132P
L206A
L206W
L206Q
L206D
L209A
L209W
L209Q
L209D
L209P
L216A
L216W
L216Q
L216D
L216P
L238A
L238W
L238Q
L238D
L238P
I255A
I255W
I255Q
I255D
I255P
F266A
F266W
F266Q
F266D
F266P
I307A
I307W
I307Q
I307D
I307P
0
carA (b0032, P0A6F1)
382
high fitness
reduced fitness
low fitness
beta-sheets
alpha-helices
turns
no data / not in library
is in library after recombination
binding sites
active sites
bold verified ts mutant
-3.7
hydrophobicity (Kyte & Doolittle, 1982, J. Mol., 157, 105-132)
3.7

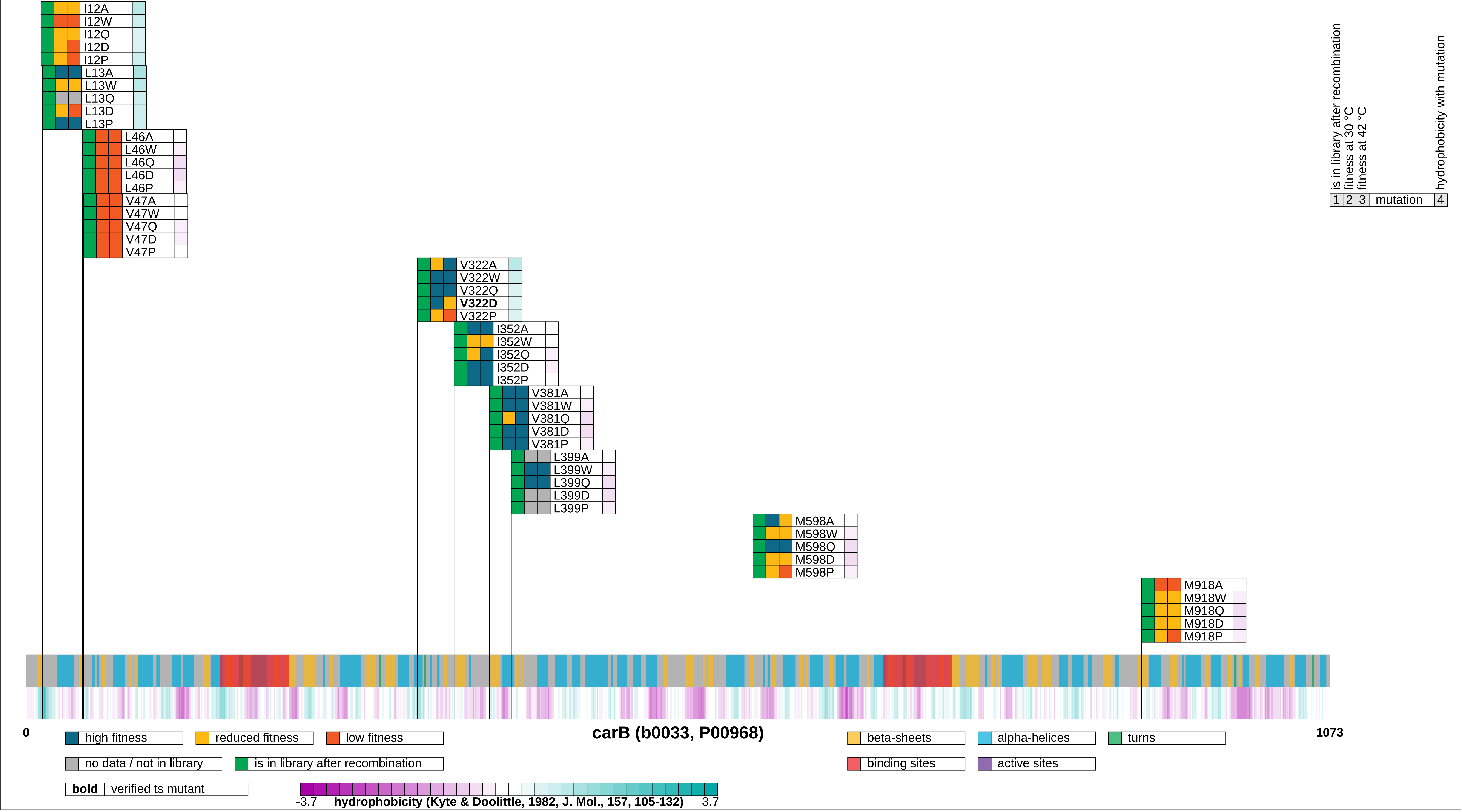

I12A
I12W
I12Q
I12D
I12P
L13A
L13W
L13Q
L13D
L13P
L46A
L46W
L46Q
L46D
L46P
V47A
V47W
V47Q
V47D
V47P
V322A
V322W
V322Q
V322D
V322P
I352A
I352W
I352Q
I352D
I352P
V381A
V381W
V381Q
V381D
V381P
L399A
L399W
L399Q
L399D
L399P
M598A
M598W
M598Q
M598D
M598P
M918A
M918W
M918Q
M918D
M918P
is in library after recombination
fitness at 30 °C
fitness at 42 °C
hydrophobicity with mutation
1 2 3 mutation 4
0
carB (b0033, P00968)
1073
high fitness
reduced fitness
low fitness
beta-sheets
alpha-helices
turns
no data / not in library
is in library after recombination
binding sites
active sites
bold verified ts mutant
-3.7
hydrophobicity (Kyte & Doolittle, 1982, J. Mol., 157, 105-132)
3.7

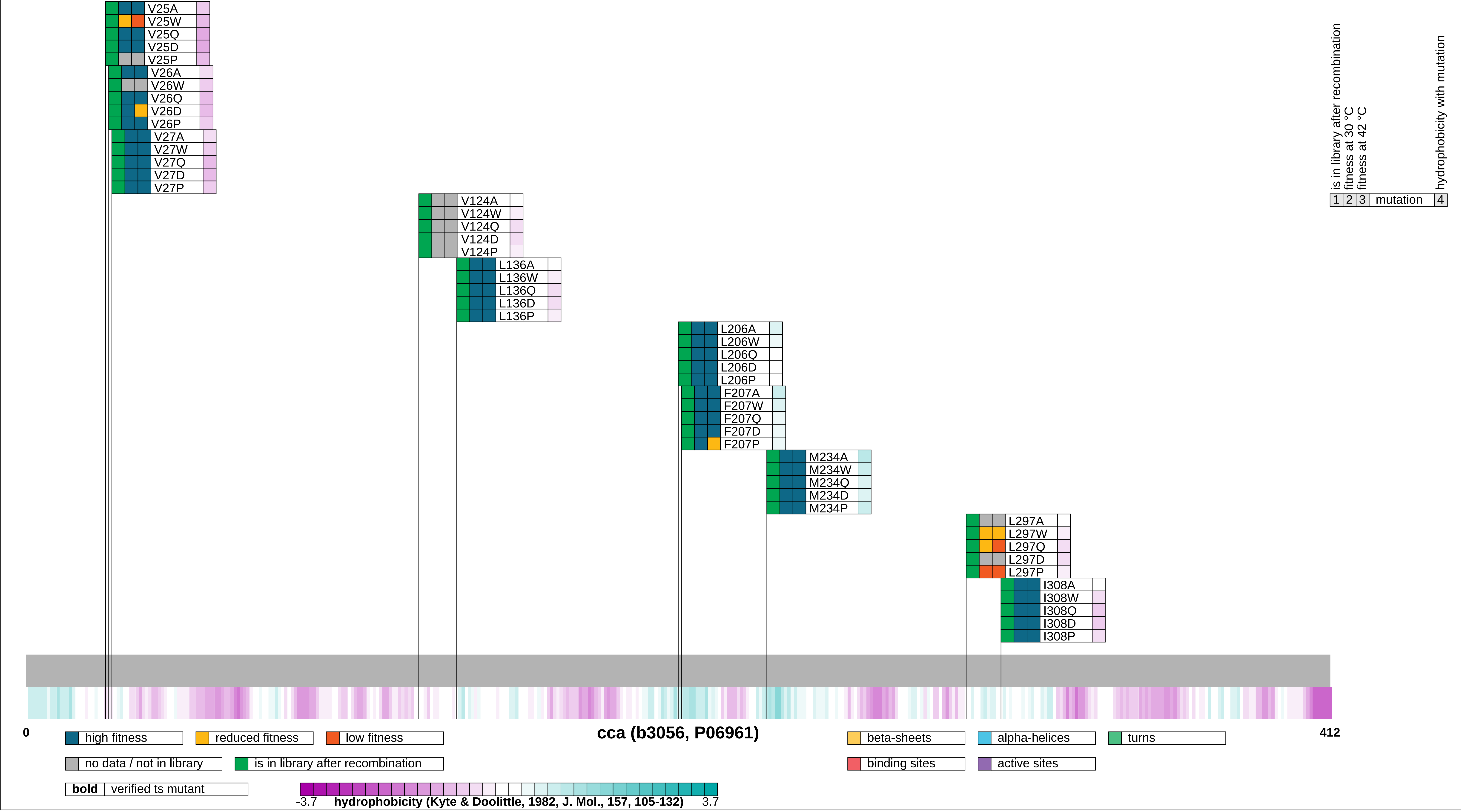

V25A
V25W
V25Q
V25D
V25P
V26A
V26W
V26Q
V26D
V26P
V27A
V27W
V27Q
V27D
V27P
V124A
V124W
V124Q
V124D
V124P
L136A
L136W
L136Q
L136D
L136P
L206A
L206W
L206Q
L206D
L206P
F207A
F207W
F207Q
F207D
F207P
M234A
M234W
M234Q
M234D
M234P
L297A
L297W
L297Q
L297D
L297P
I308A
I308W
I308Q
I308D
I308P
is in library after recombination
fitness at 30 °C
fitness at 42 °C
hydrophobicity with mutation
1 2 3 mutation 4
0
cca (b3056, P06961)
412
high fitness
reduced fitness
low fitness
beta-sheets
alpha-helices
turns
no data / not in library
is in library after recombination
binding sites
active sites
bold verified ts mutant
-3.7
hydrophobicity (Kyte & Doolittle, 1982, J. Mol., 157, 105-132)
3.7

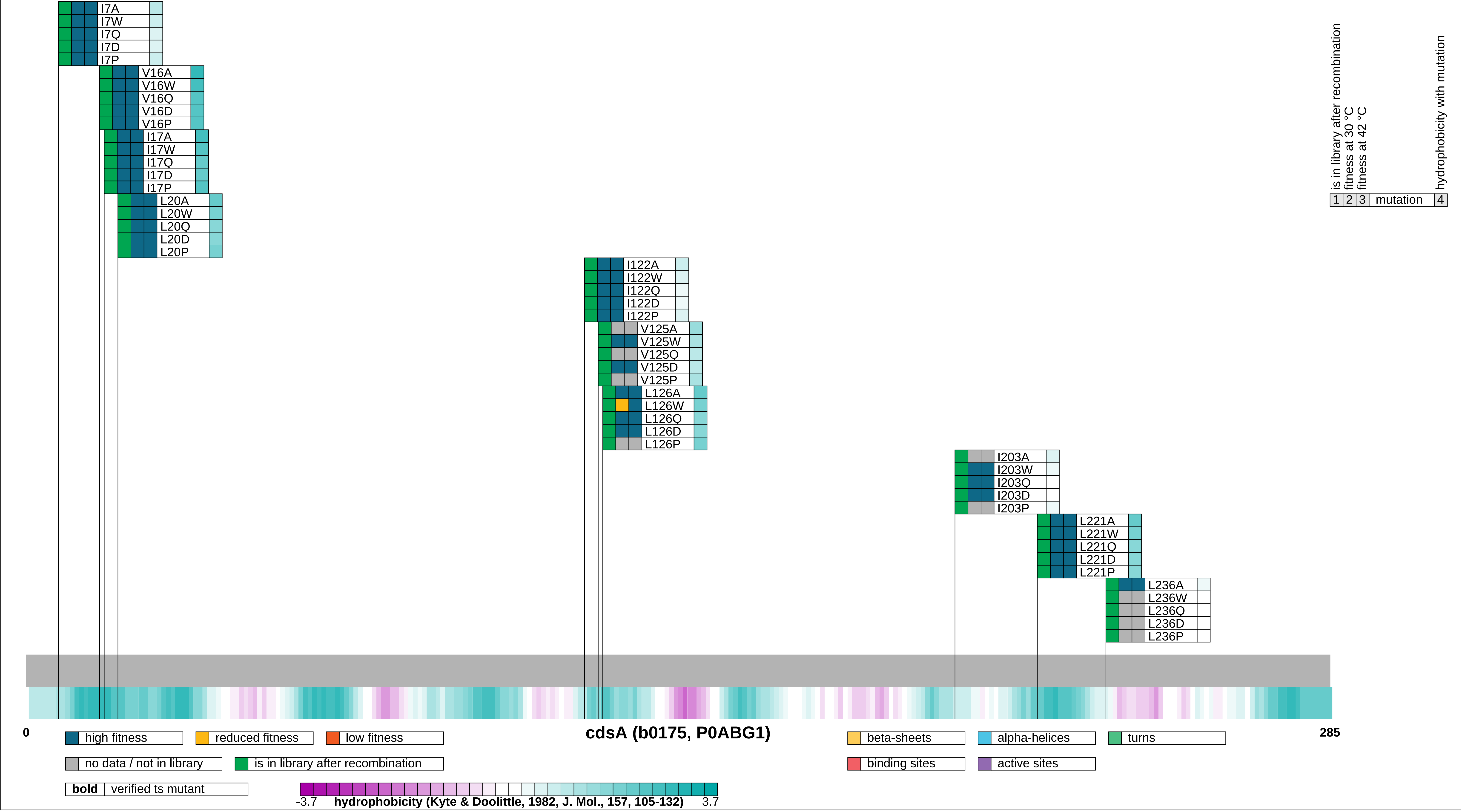

I7A
I7W
I7Q
I7D
I7P
V16A
V16W
V16Q
V16D
V16P
I17A
I17W
I17Q
I17D
I17P
L20A
L20W
L20Q
L20D
L20P
I122A
I122W
I122Q
I122D
I122P
V125A
V125W
V125Q
V125D
V125P
L126A
L126W
L126Q
L126D
L126P
I203A
I203W
I203Q
I203D
I203P
L221A
L221W
L221Q
L221D
L221P
L236A
L236W
L236Q
L236D
L236P
is in library after recombination
fitness at 30 °C
fitness at 42 °C
hydrophobicity with mutation
1 2 3 mutation 4
0
cdsA (b0175, P0ABG1)
285
high fitness
reduced fitness
low fitness
beta-sheets
alpha-helices
turns
no data / not in library
is in library after recombination
binding sites
active sites
bold verified ts mutant
-3.7
hydrophobicity (Kyte & Doolittle, 1982, J. Mol., 157, 105-132)
3.7

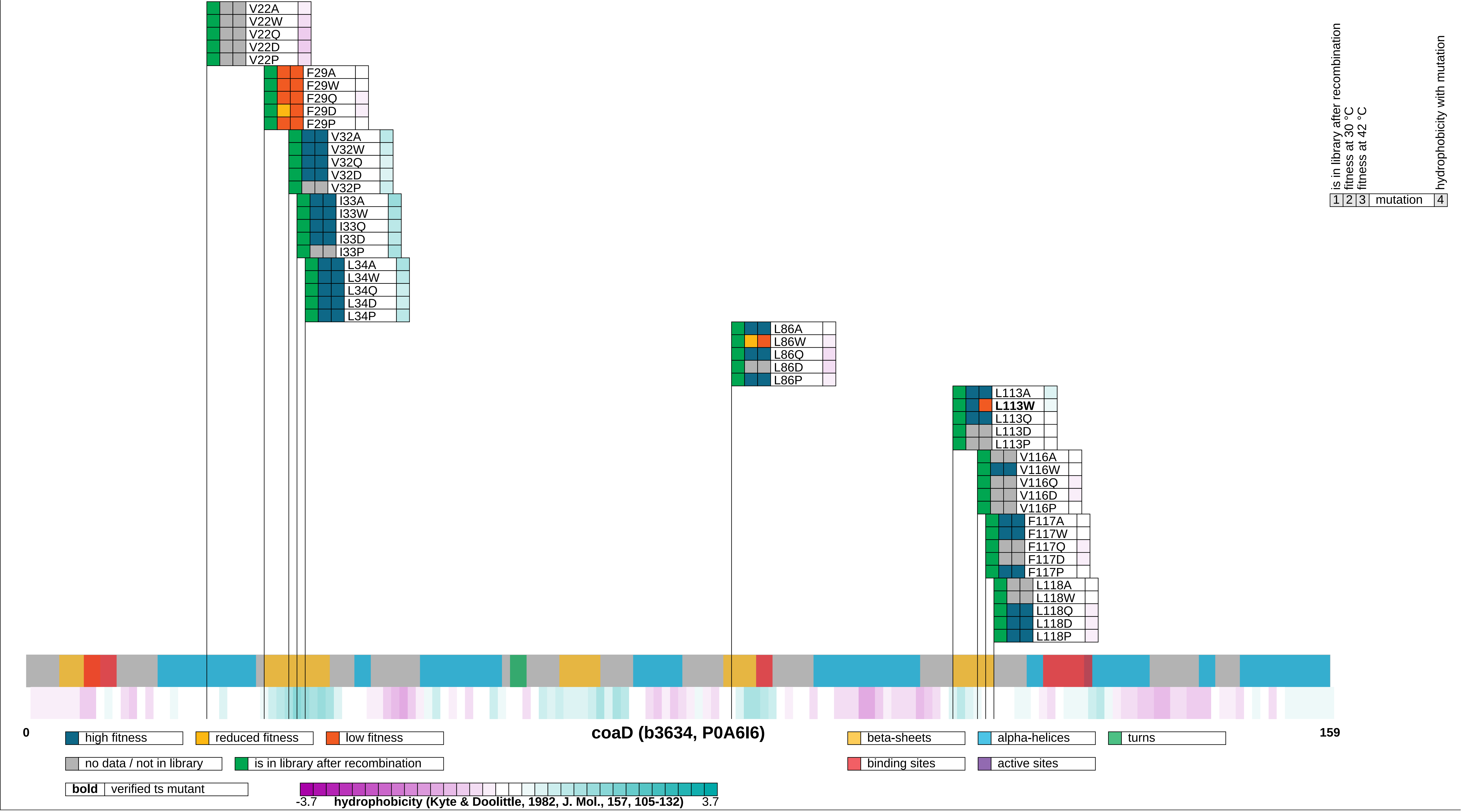
V22A
V22W
V22Q
V22D
V22P
F29A
F29W
F29Q
F29D
F29P
V32A
V32W
V32Q
V32D
V32P
I33A
I33W
I33Q
I33D
I33P
L34A
L34W
L34Q
L34D
L34P
L86A
L86W
L86Q
L86D
L86P
L113A
L113W
L113Q
L113D
L113P
V116A
V116W
V116Q
V116D
V116P
F117A
F117W
F117Q
F117D
F117P
L118A
L118W
L118Q
L118D
L118P
is in library after recombination
fitness at 30 °C
fitness at 42 °C
hydrophobicity with mutation
1 2 3 mutation 4
0
coaD (b3634, P0A6I6)
159
high fitness
reduced fitness
low fitness
beta-sheets
alpha-helices
turns
no data / not in library
is in library after recombination
binding sites
active sites
bold verified ts mutant
-3.7 hydrophobicity (Kyte & Doolittle, 1982, J. Mol., 157, 105-132) 3.7

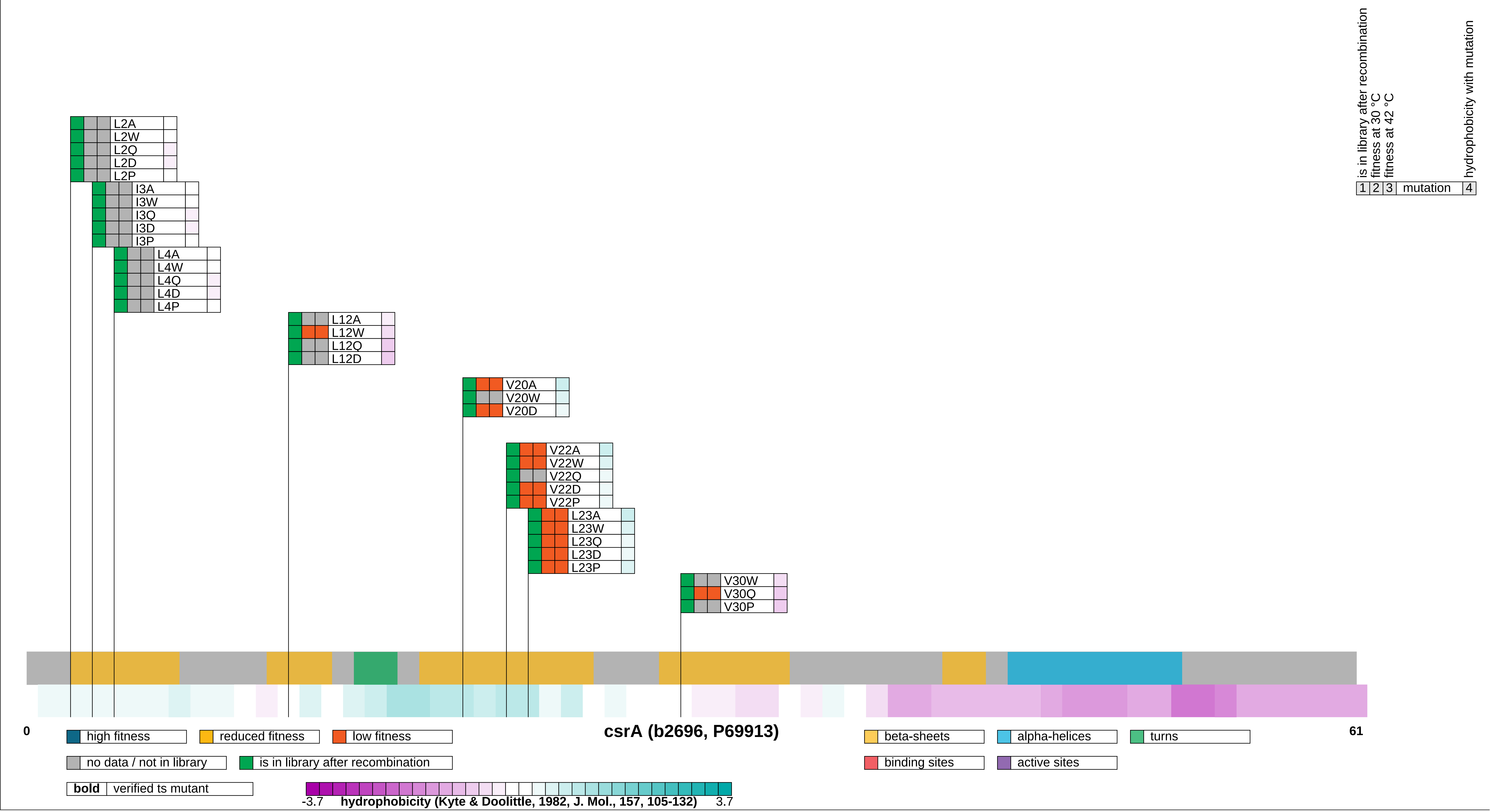

is in library after recombination
fitness at 30 °C
fitness at 42 °C
hydrophobicity with mutation
1 2 3 mutation 4
L2A
L2W
L2Q
L2D
L2P
I3A
I3W
I3Q
I3D
I3P
L4A
L4W
L4Q
L4D
L4P
L12A
L12W
L12Q
L12D
V20A
V20W
V20D
V22A
V22W
V22Q
V22D
V22P
L23A
L23W
L23Q
L23D
L23P
V30W
V30Q
V30P
0
csrA (b2696, P69913)
61
high fitness
reduced fitness
low fitness
beta-sheets
alpha-helices
turns
no data / not in library
is in library after recombination
binding sites
active sites
bold verified ts mutant
-3.7 hydrophobicity (Kyte & Doolittle, 1982, J. Mol., 157, 105-132) 3.7

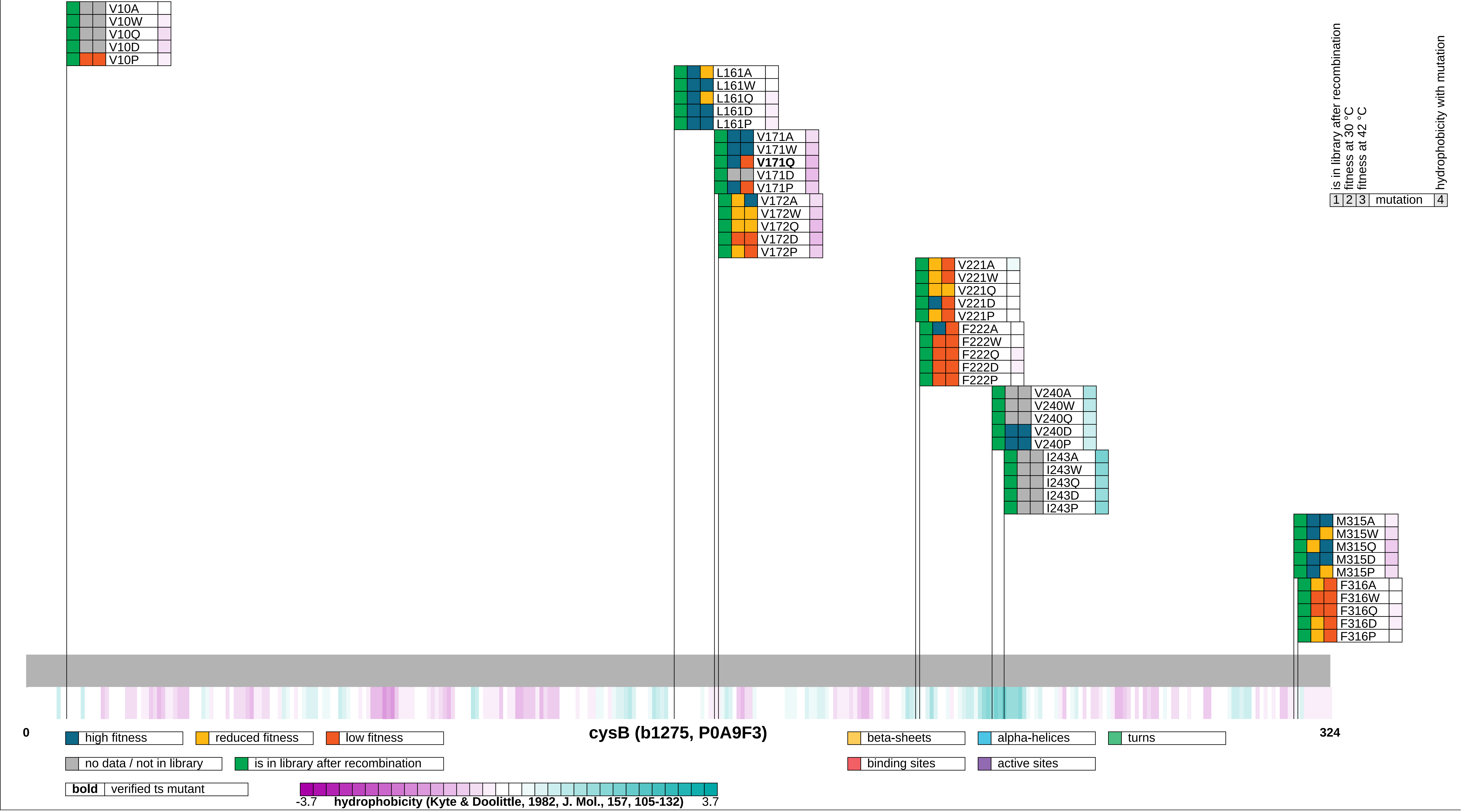
V10A
V10W
V10Q
V10D
V10P
L161A
L161W
L161Q
L161D
L161P
V171A
V171W
V171Q
V171D
V171P
V172A
V172W
V172Q
V172D
V172P
V221A
V221W
V221Q
V221D
V221P
F222A
F222W
F222Q
F222D
F222P
V240A
V240W
V240Q
V240D
V240P
I243A
I243W
I243Q
I243D
I243P
M315A
M315W
M315Q
M315D
M315P
F316A
F316W
F316Q
F316D
F316P
is in library after recombination
fitness at 30 °C
fitness at 42 °C
hydrophobicity with mutation
1 2 3 mutation 4
0
cysB (b1275, P0A9F3)
324
high fitness
reduced fitness
low fitness
no data / not in library
is in library after recombination
bold verified ts mutant
beta-sheets
alpha-helices
turns
binding sites
active sites
-3.7
hydrophobicity (Kyte & Doolittle, 1982, J. Mol., 157, 105-132)
3.7

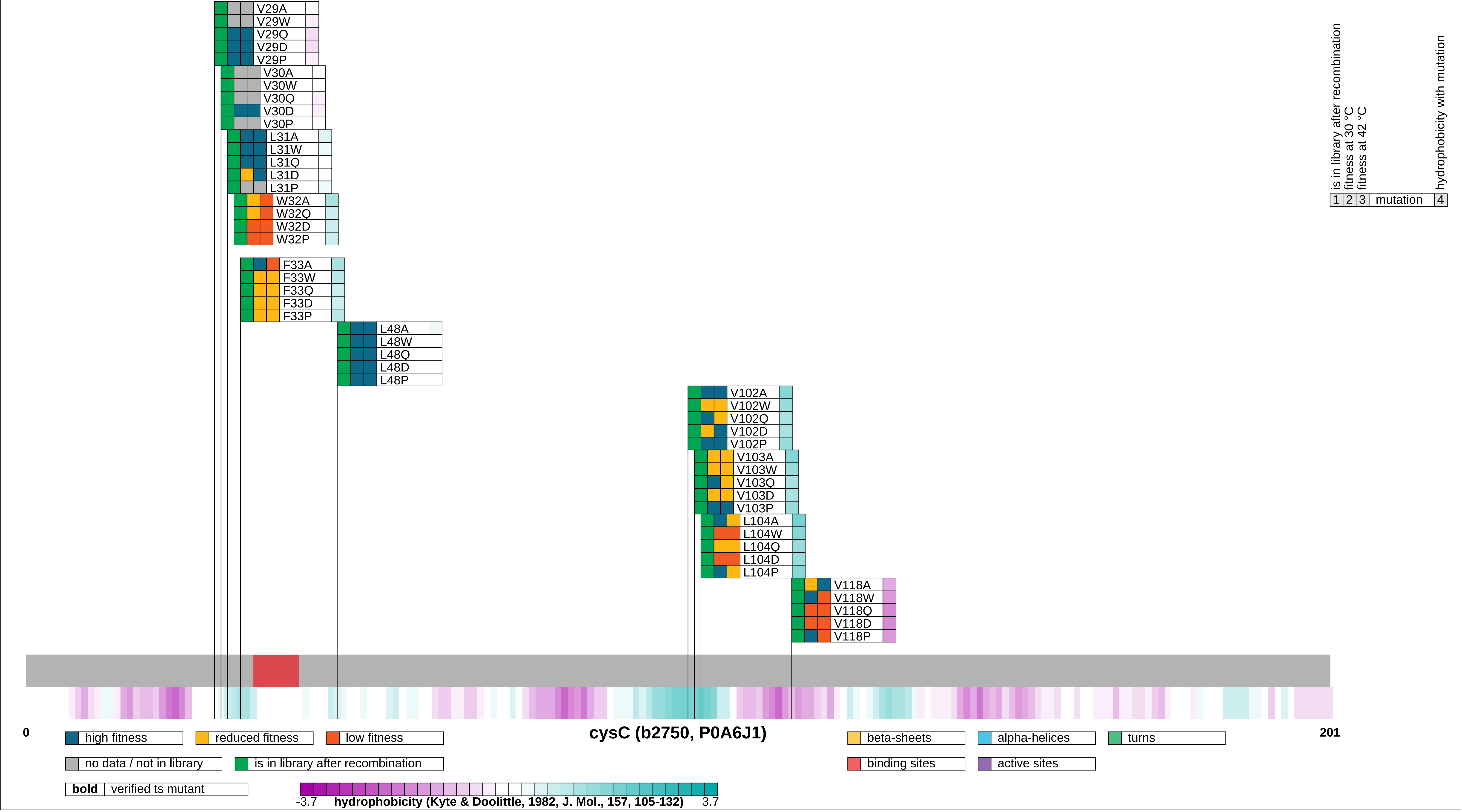
V29A
V29W
V29Q
V29D
V29P
V30A
V30W
V30Q
V30D
V30P
L31A
L31W
L31Q
L31D
L31P
W32A
W32Q
W32D
W32P
F33A
F33W
F33Q
F33D
F33P
L48A
L48W
L48Q
L48D
L48P
V102A
V102W
V102Q
V102D
V102P
V103A
V103W
V103Q
V103D
V103P
L104A
L104W
L104Q
L104D
L104P
V118A
V118W
V118Q
V118D
V118P
is in library after recombination
fitness at 30 °C
fitness at 42 °C
hydrophobicity with mutation
1 2 3 mutation 4
0
cysC (b2750, P0A6J1)
201
high fitness
reduced fitness
low fitness
beta-sheets
alpha-helices
turns
no data / not in library
is in library after recombination
binding sites
active sites
bold verified ts mutant
-3.7
hydrophobicity (Kyte & Doolittle, 1982, J. Mol., 157, 105-132)
3.7

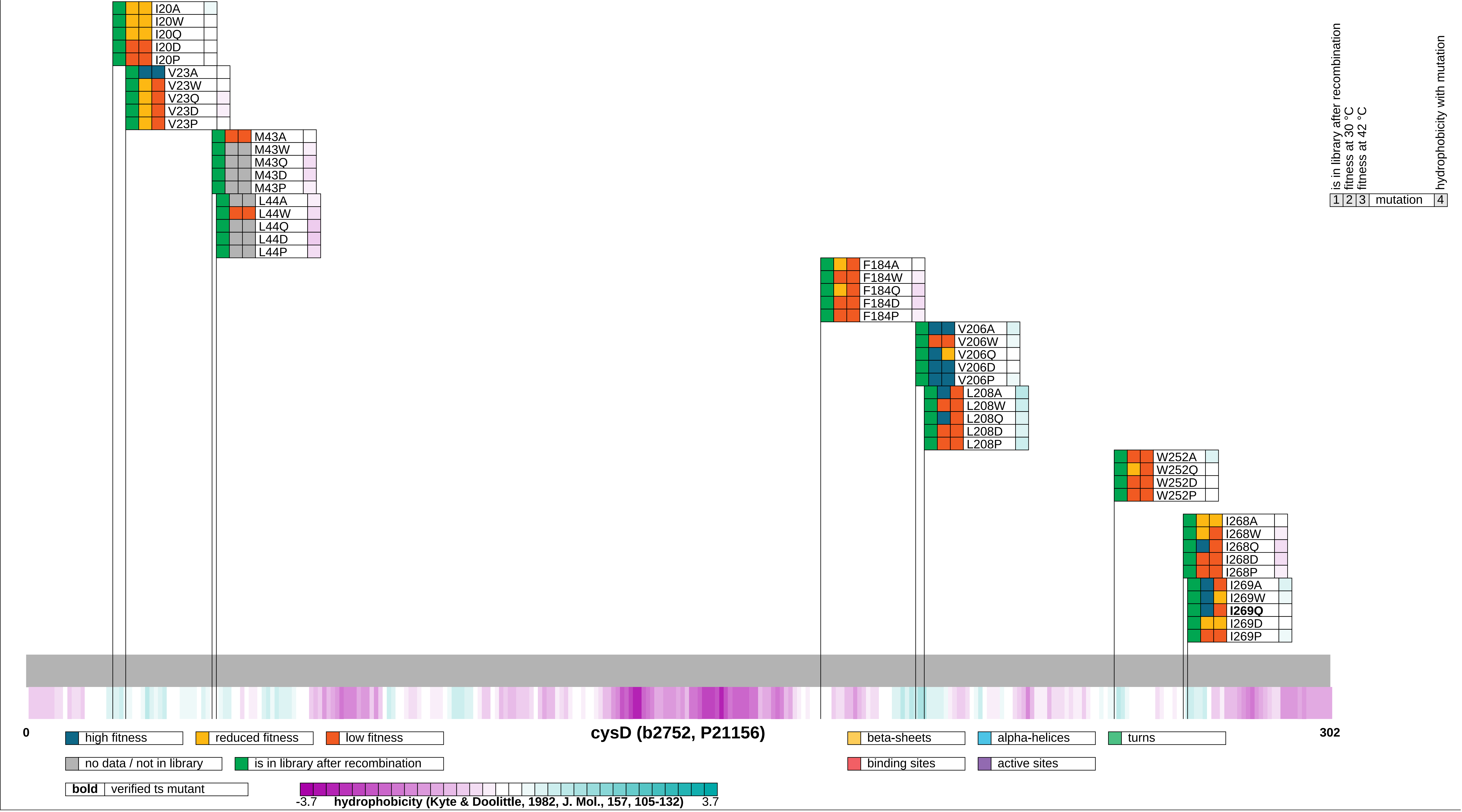
I20A
I20W
I20Q
I20D
I20P
V23A
V23W
V23Q
V23D
V23P
M43A
M43W
M43Q
M43D
M43P
L44A
L44W
L44Q
L44D
L44P
F184A
F184W
F184Q
F184D
F184P
V206A
V206W
V206Q
V206D
V206P
L208A
L208W
L208Q
L208D
L208P
W252A
W252Q
W252D
W252P
I268A
I268W
I268Q
I268D
I268P
I269A
I269W
I269Q
I269D
I269P
is in library after recombination
fitness at 30 °C
fitness at 42 °C
hydrophobicity with mutation
1 2 3 mutation 4
0
cysD (b2752, P21156)
302
high fitness
reduced fitness
low fitness
no data / not in library
is in library after recombination
bold verified ts mutant
beta-sheets
alpha-helices
turns
binding sites
active sites
-3.7 hydrophobicity (Kyte & Doolittle, 1982, J. Mol., 157, 105-132) 3.7

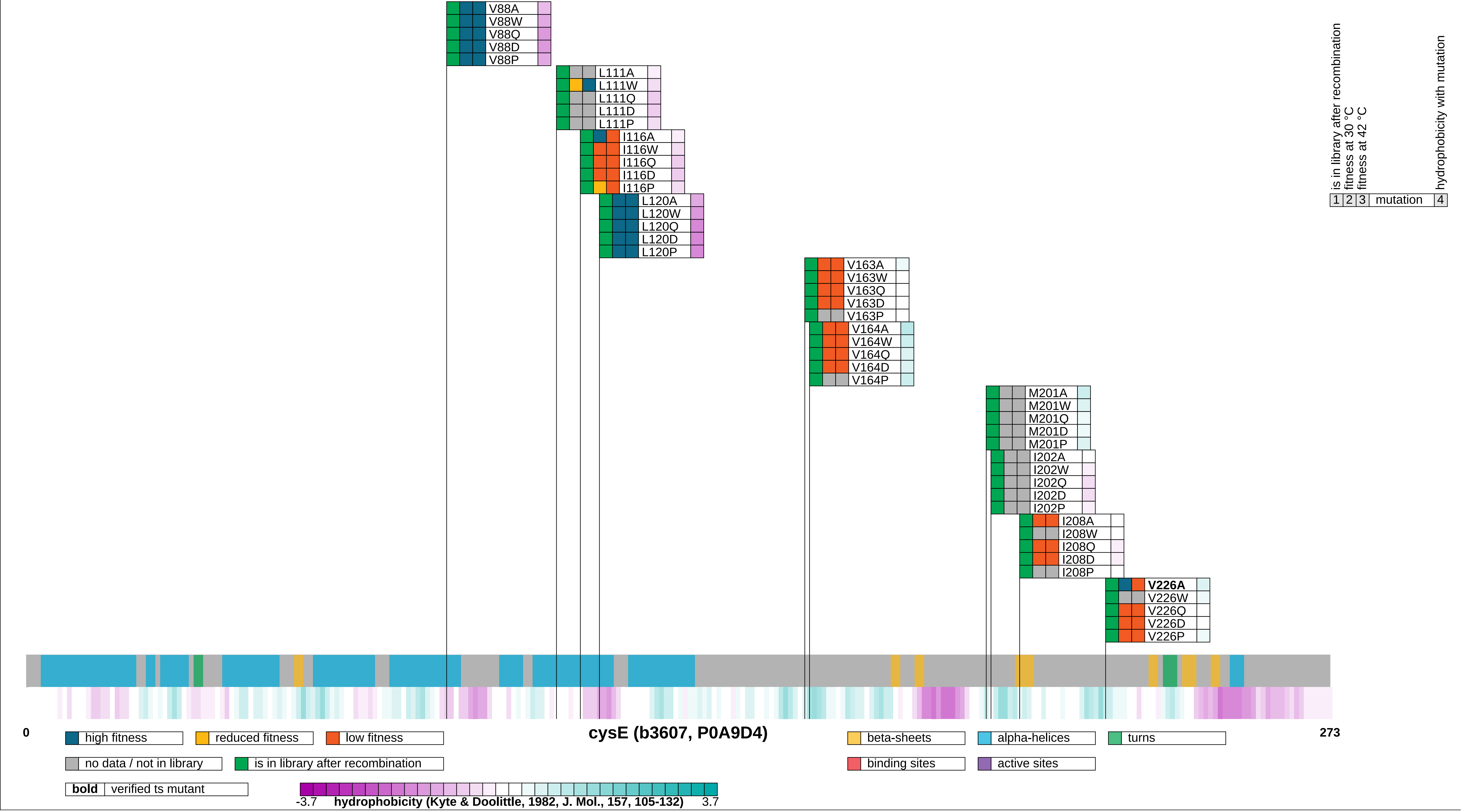

V88A
V88W
V88Q
V88D
V88P
L111A
L111W
L111Q
L111D
L111P
I116A
I116W
I116Q
I116D
I116P
L120A
L120W
L120Q
L120D
L120P
V163A
V163W
V163Q
V163D
V163P
V164A
V164W
V164Q
V164D
V164P
M201A
M201W
M201Q
M201D
M201P
I202A
I202W
I202Q
I202D
I202P
I208A
I208W
I208Q
I208D
I208P
V226A
V226W
V226Q
V226D
V226P
is in library after recombination
fitness at 30 °C
fitness at 42 °C
hydrophobicity with mutation
1
2
3
mutation
4
0
cysE (b3607, P0A9D4)
273
high fitness
reduced fitness
low fitness
no data / not in library
is in library after recombination
bold
verified ts mutant
beta-sheets
alpha-helices
turns
binding sites
active sites
-3.7
hydrophobicity (Kyte & Doolittle, 1982, J. Mol., 157, 105-132)
3.7

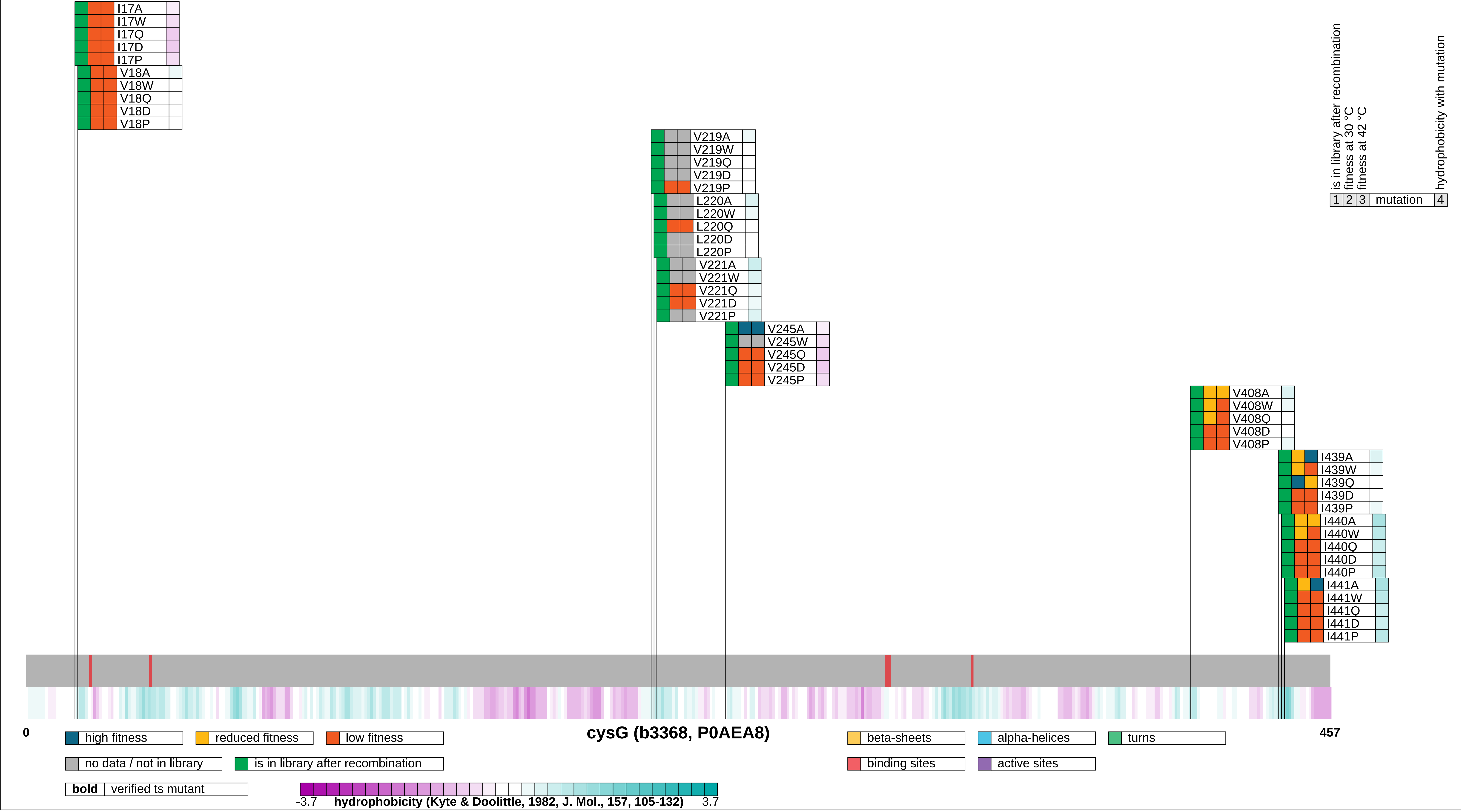
I17A
I17W
I17Q
I17D
I17P
V18A
V18W
V18Q
V18D
V18P
V219A
V219W
V219Q
V219D
V219P
L220A
L220W
L220Q
L220D
L220P
V221A
V221W
V221Q
V221D
V221P
V245A
V245W
V245Q
V245D
V245P
V408A
V408W
V408Q
V408D
V408P
I439A
I439W
I439Q
I439D
I439P
I440A
I440W
I440Q
I440D
I440P
I441A
I441W
I441Q
I441D
I441P
is in library after recombination
fitness at 30 °C
fitness at 42 °C
hydrophobicity with mutation
1 2 3 mutation 4
0
cysG (b3368, P0AEA8)
457
high fitness
reduced fitness
low fitness
beta-sheets
alpha-helices
turns
no data / not in library
is in library after recombination
binding sites
active sites
bold verified ts mutant
-3.7
hydrophobicity (Kyte & Doolittle, 1982, J. Mol., 157, 105-132)
3.7

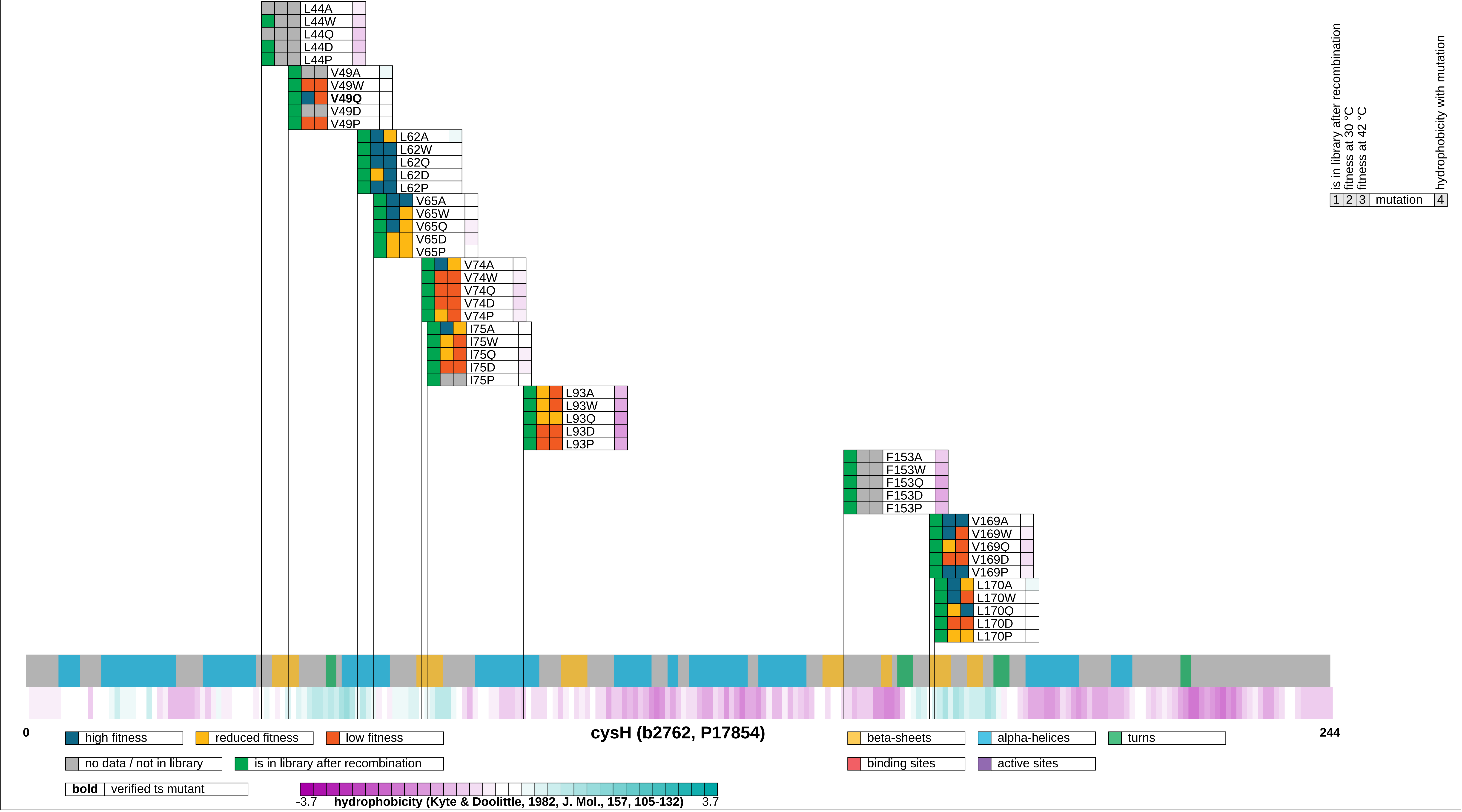

L44A
L44W
L44Q
L44D
L44P
V49A
V49W
V49Q
V49D
V49P
L62A
L62W
L62Q
L62D
L62P
V65A
V65W
V65Q
V65D
V65P
V74A
V74W
V74Q
V74D
V74P
I75A
I75W
I75Q
I75D
I75P
L93A
L93W
L93Q
L93D
L93P
F153A
F153W
F153Q
F153D
F153P
V169A
V169W
V169Q
V169D
V169P
L170A
L170W
L170Q
L170D
L170P
is in library after recombination
fitness at 30 °C
fitness at 42 °C
hydrophobicity with mutation
1 2 3 mutation 4
0
cysH (b2762, P17854)
244
high fitness
reduced fitness
low fitness
beta-sheets
alpha-helices
turns
no data / not in library
is in library after recombination
binding sites
active sites
bold verified ts mutant
-3.7 hydrophobicity (Kyte & Doolittle, 1982, J. Mol., 157, 105-132) 3.7

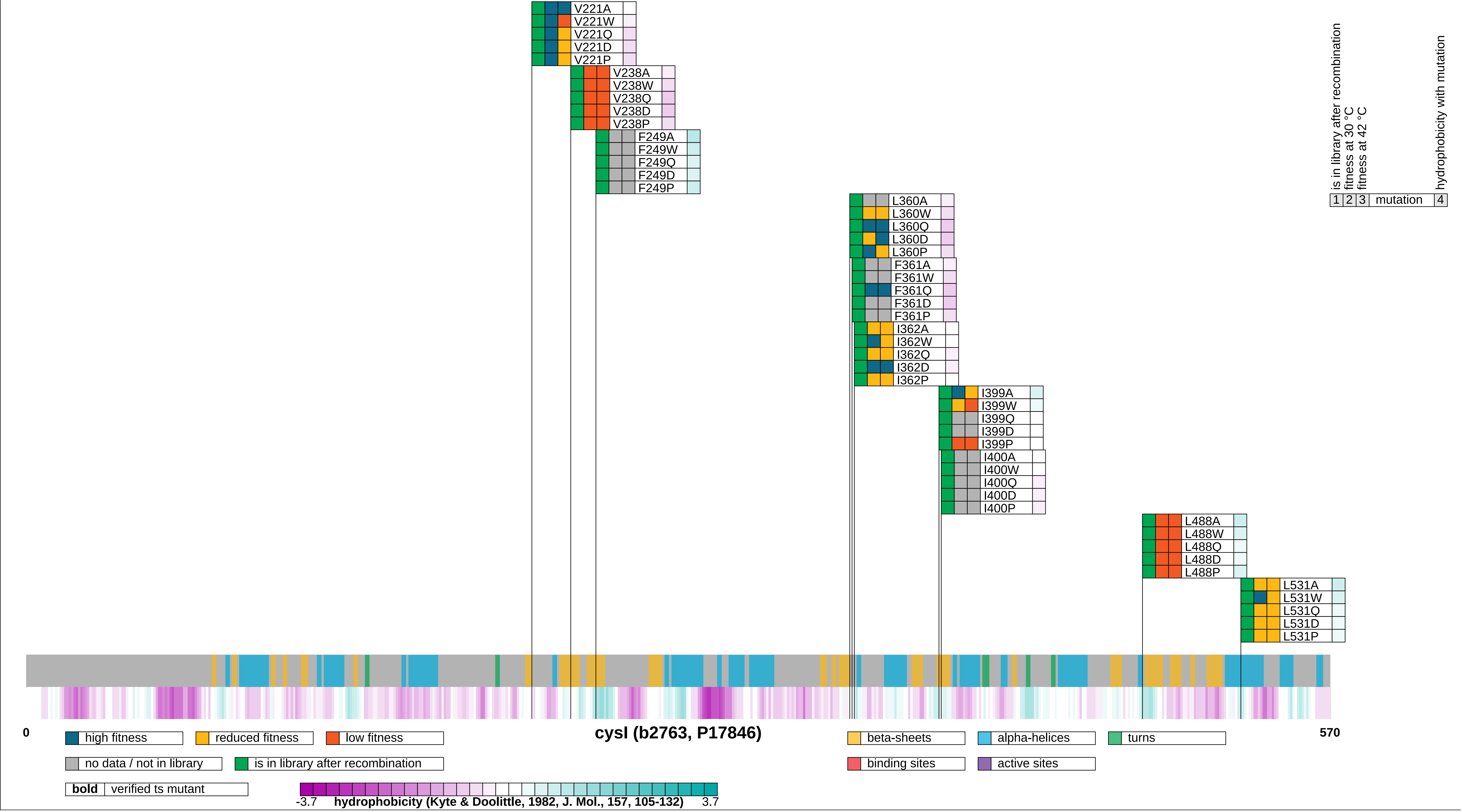

V221A
V221W
V221Q
V221D
V221P
V238A
V238W
V238Q
V238D
V238P
F249A
F249W
F249Q
F249D
F249P
L360A
L360W
L360Q
L360D
L360P
F361A
F361W
F361Q
F361D
F361P
I362A
I362W
I362Q
I362D
I362P
I399A
I399W
I399Q
I399D
I399P
I400A
I400W
I400Q
I400D
I400P
L488A
L488W
L488Q
L488D
L488P
L531A
L531W
L531Q
L531D
L531P
is in library after recombination
fitness at 30 °C
fitness at 42 °C
hydrophobicity with mutation
1 2 3 mutation 4
0
cysI (b2763, P17846)
570
high fitness
reduced fitness
low fitness
no data / not in library
is in library after recombination
bold verified ts mutant
beta-sheets
alpha-helices
turns
binding sites
active sites
-3.7
hydrophobicity (Kyte & Doolittle, 1982, J. Mol., 157, 105-132)
3.7

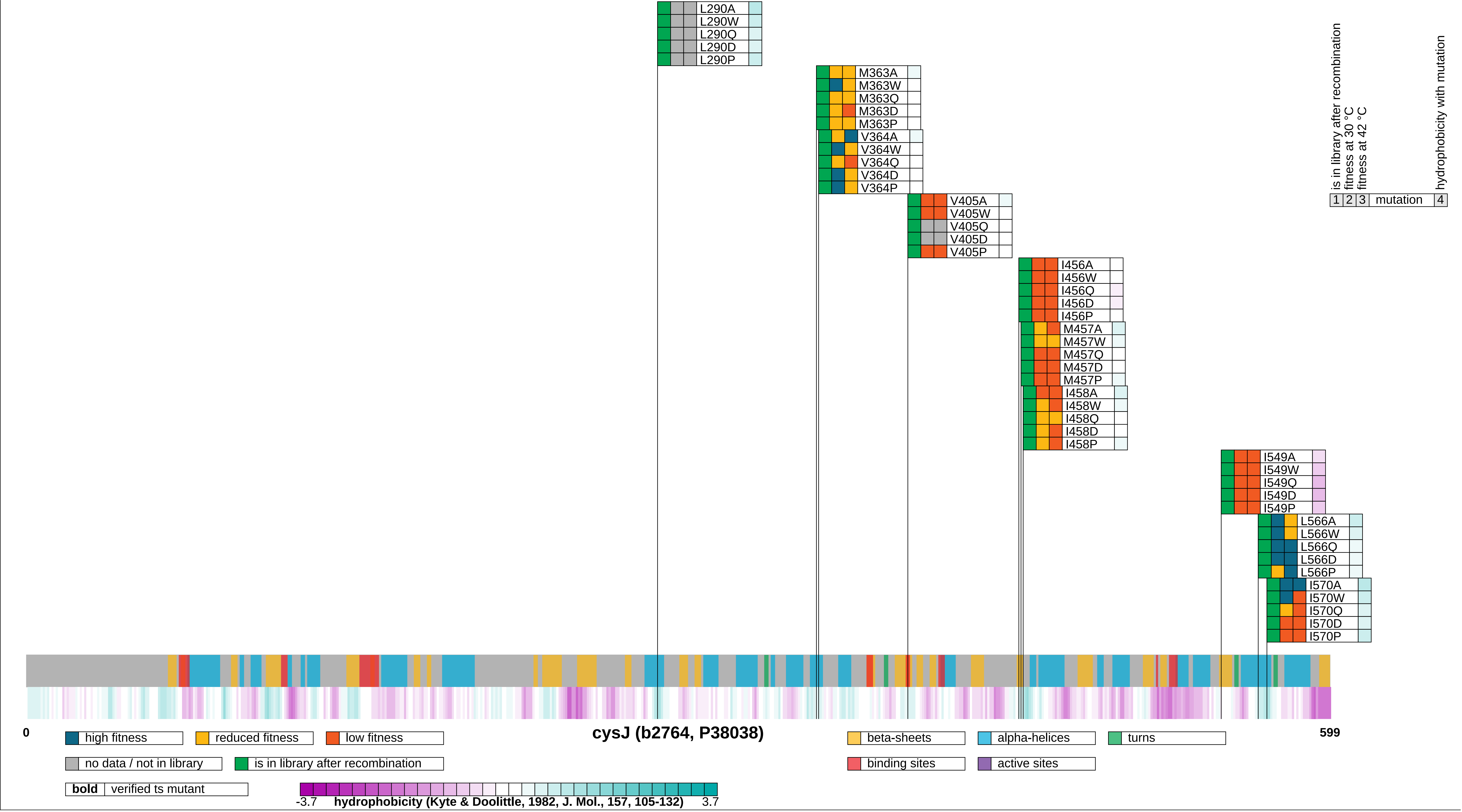
L290A
L290W
L290Q
L290D
L290P
M363A
M363W
M363Q
M363D
M363P
V364A
V364W
V364Q
V364D
V364P
V405A
V405W
V405Q
V405D
V405P
I456A
I456W
I456Q
I456D
I456P
M457A
M457W
M457Q
M457D
M457P
I458A
I458W
I458Q
I458D
I458P
I549A
I549W
I549Q
I549D
I549P
L566A
L566W
L566Q
L566D
L566P
I570A
I570W
I570Q
I570D
I570P
is in library after recombination
fitness at 30 °C
fitness at 42 °C
hydrophobicity with mutation
1 2 3 mutation 4
0
cysJ (b2764, P38038)
599
high fitness
reduced fitness
low fitness
no data / not in library
is in library after recombination
bold verified ts mutant
beta-sheets
alpha-helices
turns
binding sites
active sites
-3.7 hydrophobicity (Kyte & Doolittle, 1982, J. Mol., 157, 105-132) 3.7

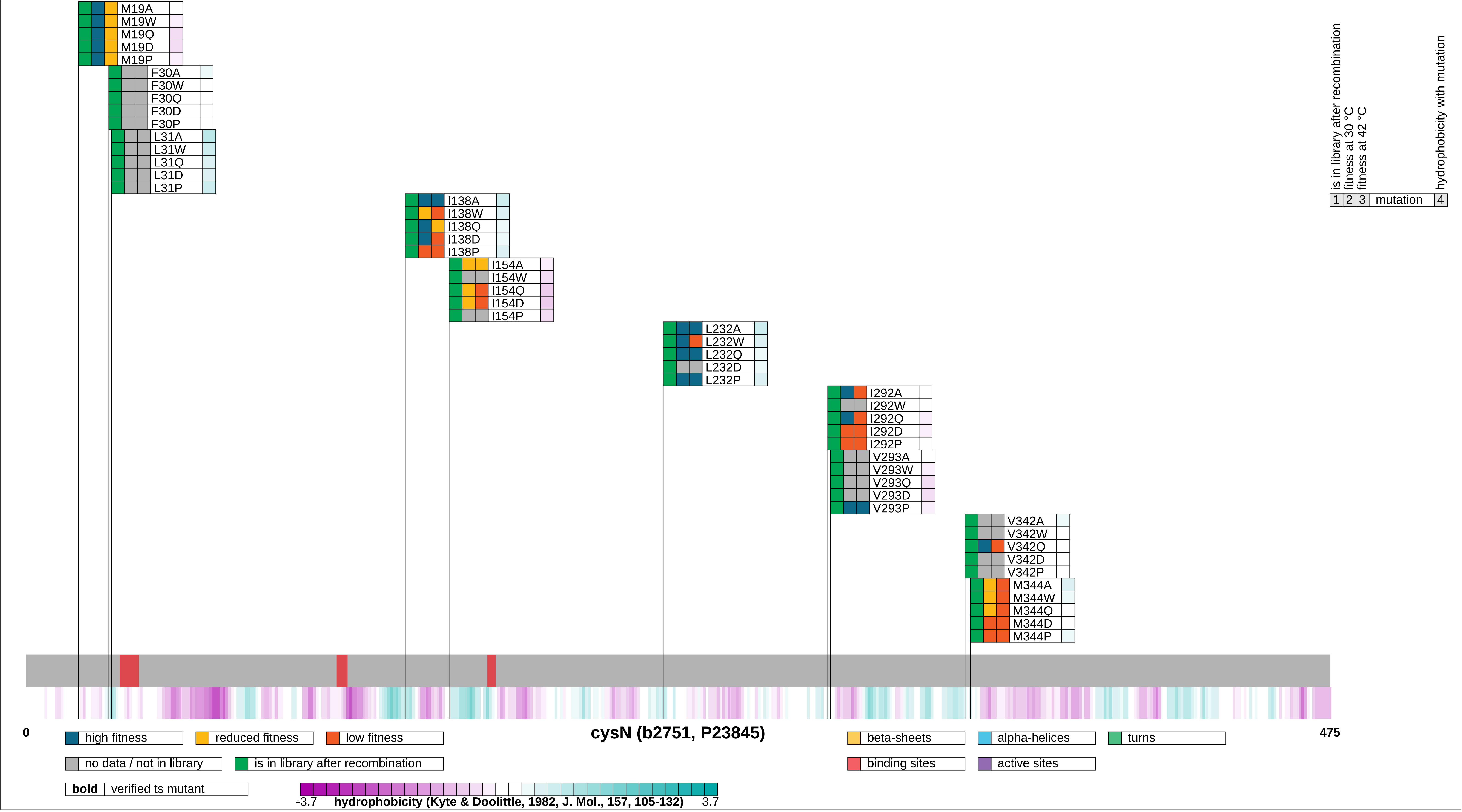

M19A
M19W
M19Q
M19D
M19P
F30A
F30W
F30Q
F30D
F30P
L31A
L31W
L31Q
L31D
L31P
I138A
I138W
I138Q
I138D
I138P
I154A
I154W
I154Q
I154D
I154P
L232A
L232W
L232Q
L232D
L232P
I292A
I292W
I292Q
I292D
I292P
V293A
V293W
V293Q
V293D
V293P
V342A
V342W
V342Q
V342D
V342P
M344A
M344W
M344Q
M344D
M344P
is in library after recombination
fitness at 30 °C
fitness at 42 °C
hydrophobicity with mutation
1 2 3 mutation 4
0
cysN (b2751, P23845)
475
high fitness
reduced fitness
low fitness
no data / not in library
is in library after recombination
bold verified ts mutant
beta-sheets
alpha-helices
turns
binding sites
active sites
-3.7 hydrophobicity (Kyte & Doolittle, 1982, J. Mol., 157, 105-132) 3.7

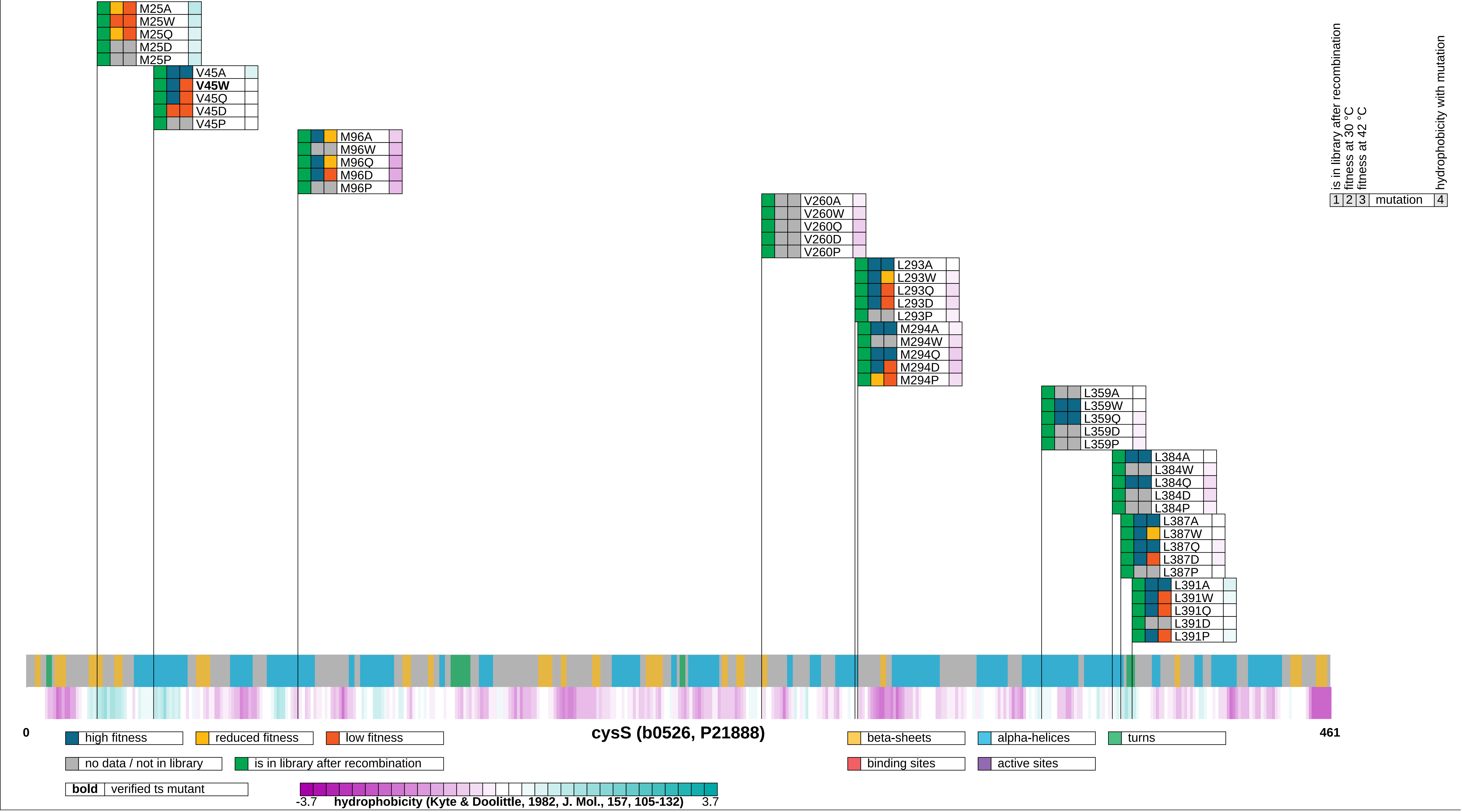
M25A
M25W
M25Q
M25D
M25P
V45A
V45W
V45Q
V45D
V45P
M96A
M96W
M96Q
M96D
M96P
V260A
V260W
V260Q
V260D
V260P
L293A
L293W
L293Q
L293D
L293P
M294A
M294W
M294Q
M294D
M294P
L359A
L359W
L359Q
L359D
L359P
L384A
L384W
L384Q
L384D
L384P
L387A
L387W
L387Q
L387D
L387P
L391A
L391W
L391Q
L391D
L391P
is in library after recombination
fitness at 30 °C
fitness at 42 °C
hydrophobicity with mutation
1 2 3 mutation 4
0
cysS (b0526, P21888)
461
high fitness
reduced fitness
low fitness
beta-sheets
alpha-helices
turns
no data / not in library
is in library after recombination
binding sites
active sites
bold verified ts mutant
-3.7
hydrophobicity (Kyte & Doolittle, 1982, J. Mol., 157, 105-132)
3.7

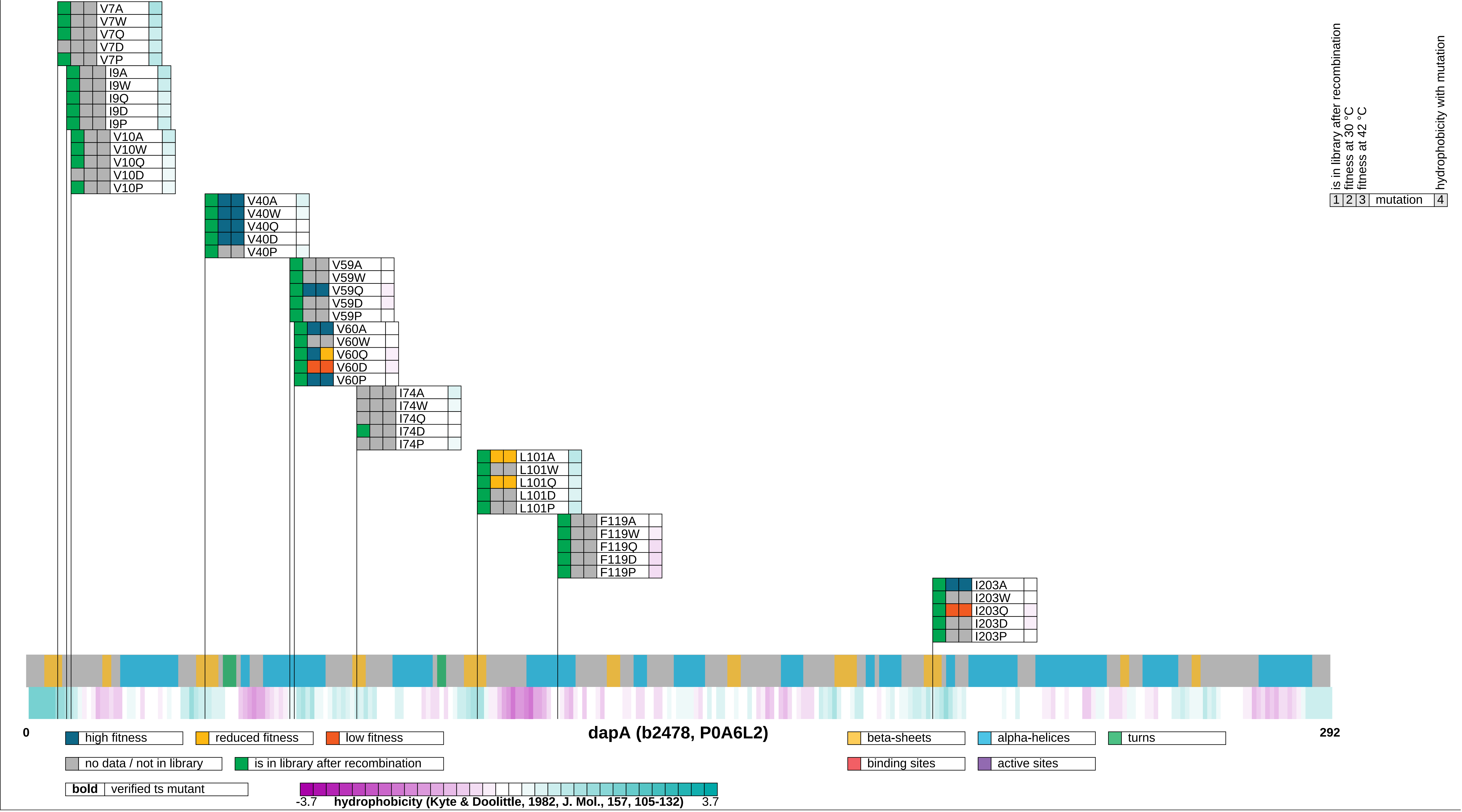
V7A
V7W
V7Q
V7D
V7P
I9A
I9W
I9Q
I9D
I9P
V10A
V10W
V10Q
V10D
V10P
V40A
V40W
V40Q
V40D
V40P
V59A
V59W
V59Q
V59D
V59P
V60A
V60W
V60Q
V60D
V60P
I74A
I74W
I74Q
I74D
I74P
L101A
L101W
L101Q
L101D
L101P
F119A
F119W
F119Q
F119D
F119P
I203A
I203W
I203Q
I203D
I203P
is in library after recombination
fitness at 30 °C
fitness at 42 °C
hydrophobicity with mutation
1 2 3 mutation 4
0
292
dapA (b2478, P0A6L2)
high fitness
reduced fitness
low fitness
no data / not in library
is in library after recombination
bold verified ts mutant
beta-sheets
alpha-helices
turns
binding sites
active sites
-3.7
hydrophobicity (Kyte & Doolittle, 1982, J. Mol., 157, 105-132)
3.7

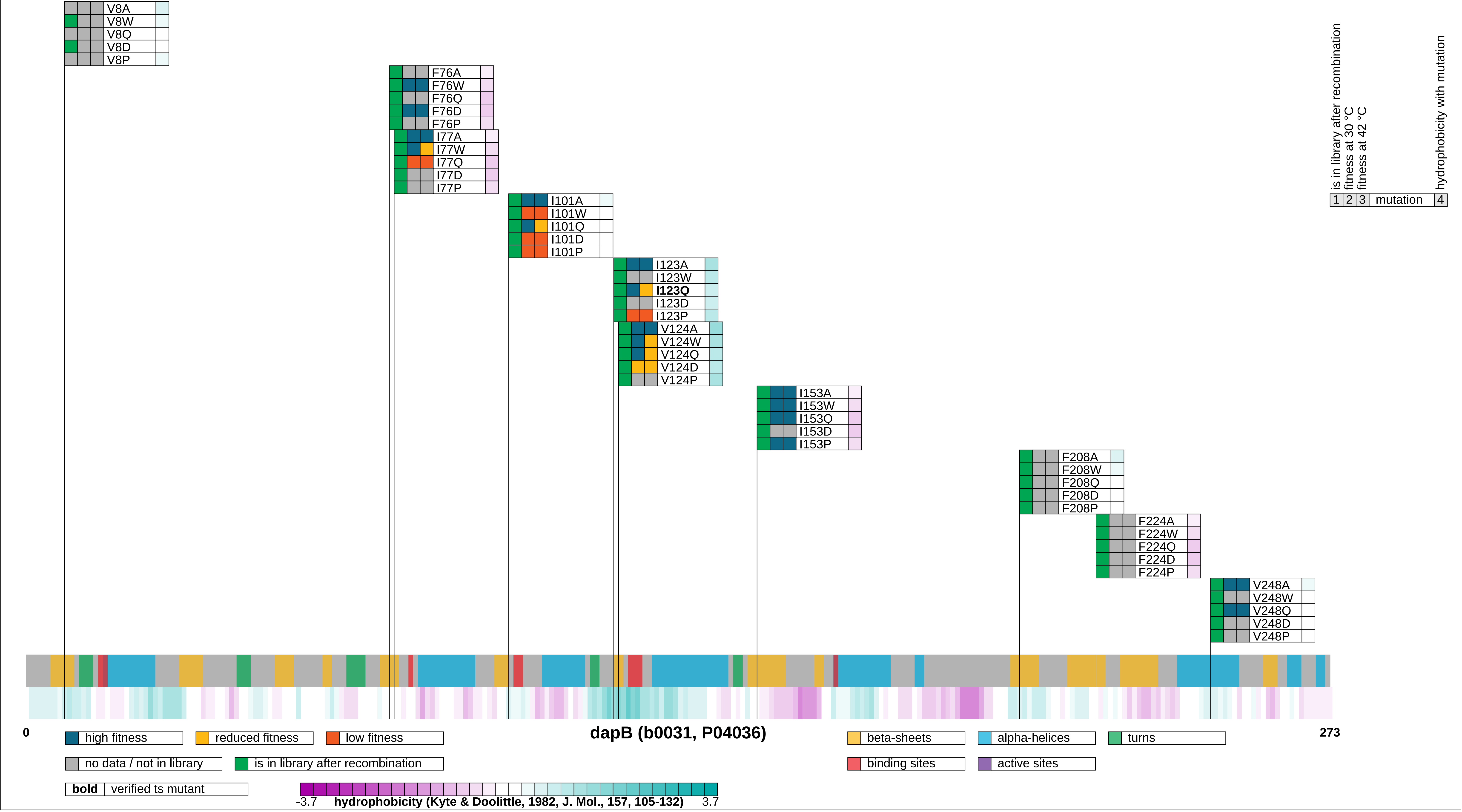
V8A
V8W
V8Q
V8D
V8P
F76A
F76W
F76Q
F76D
F76P
I77A
I77W
I77Q
I77D
I77P
I101A
I101W
I101Q
I101D
I101P
I123A
I123W
I123Q
I123D
I123P
V124A
V124W
V124Q
V124D
V124P
I153A
I153W
I153Q
I153D
I153P
F208A
F208W
F208Q
F208D
F208P
F224A
F224W
F224Q
F224D
F224P
V248A
V248W
V248Q
V248D
V248P
is in library after recombination
fitness at 30 °C
fitness at 42 °C
hydrophobicity with mutation
1 2 3 mutation 4
0
dapB (b0031, P04036)
273
high fitness
reduced fitness
low fitness
no data / not in library
is in library after recombination
bold verified ts mutant
beta-sheets
alpha-helices
turns
binding sites
active sites
-3.7 hydrophobicity (Kyte & Doolittle, 1982, J. Mol., 157, 105-132) 3.7

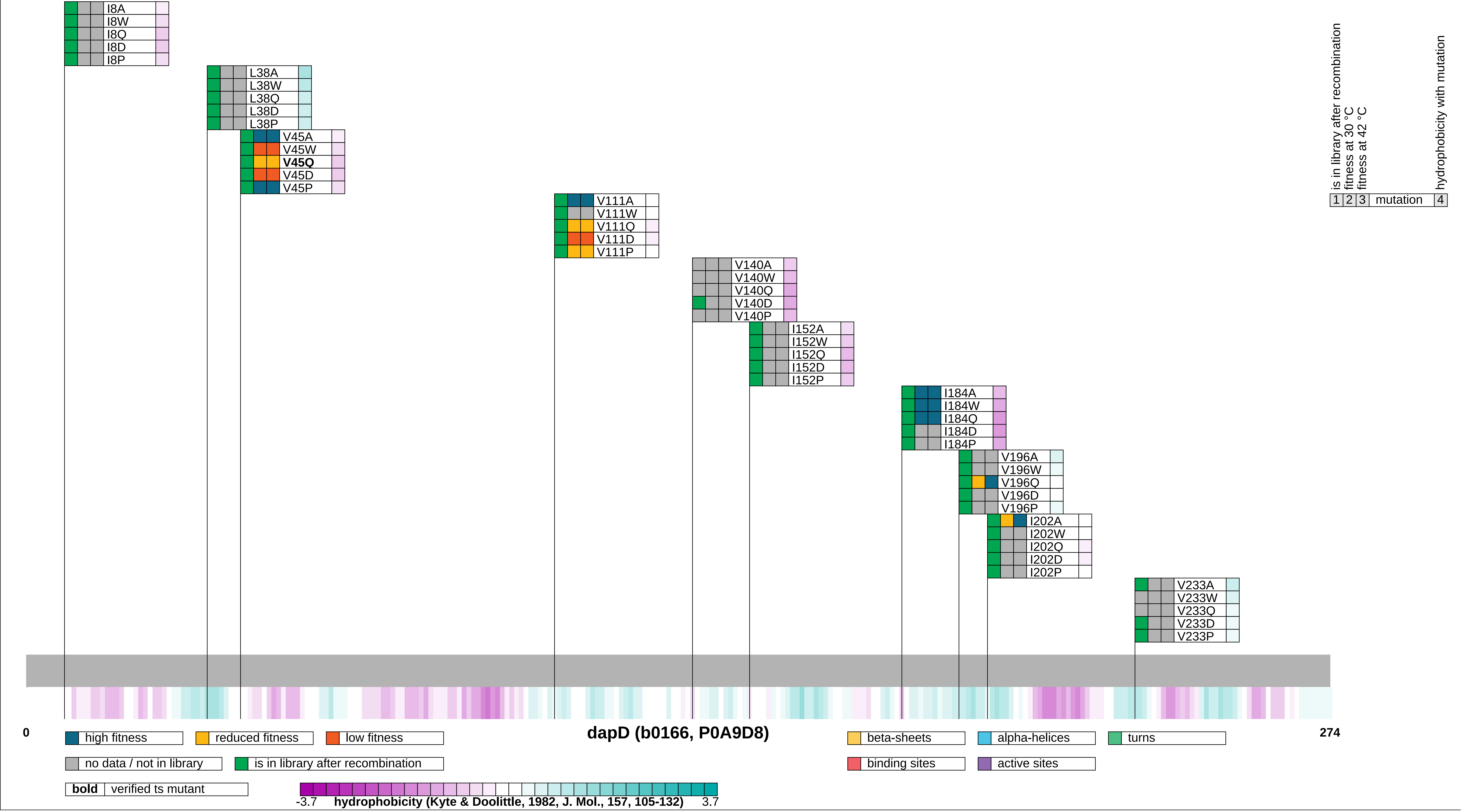

I8A
I8W
I8Q
I8D
I8P
L38A
L38W
L38Q
L38D
L38P
V45A
V45W
V45Q
V45D
V45P
V111A
V111W
V111Q
V111D
V111P
V140A
V140W
V140Q
V140D
V140P
I152A
I152W
I152Q
I152D
I152P
I184A
I184W
I184Q
I184D
I184P
V196A
V196W
V196Q
V196D
V196P
I202A
I202W
I202Q
I202D
I202P
V233A
V233W
V233Q
V233D
V233P
is in library after recombination
fitness at 30 °C
fitness at 42 °C
hydrophobicity with mutation
1 2 3 mutation 4
0
dapD (b0166, P0A9D8)
274
high fitness
reduced fitness
low fitness
no data / not in library
is in library after recombination
bold verified ts mutant
beta-sheets
alpha-helices
turns
binding sites
active sites
-3.7
hydrophobicity (Kyte & Doolittle, 1982, J. Mol., 157, 105-132)
3.7

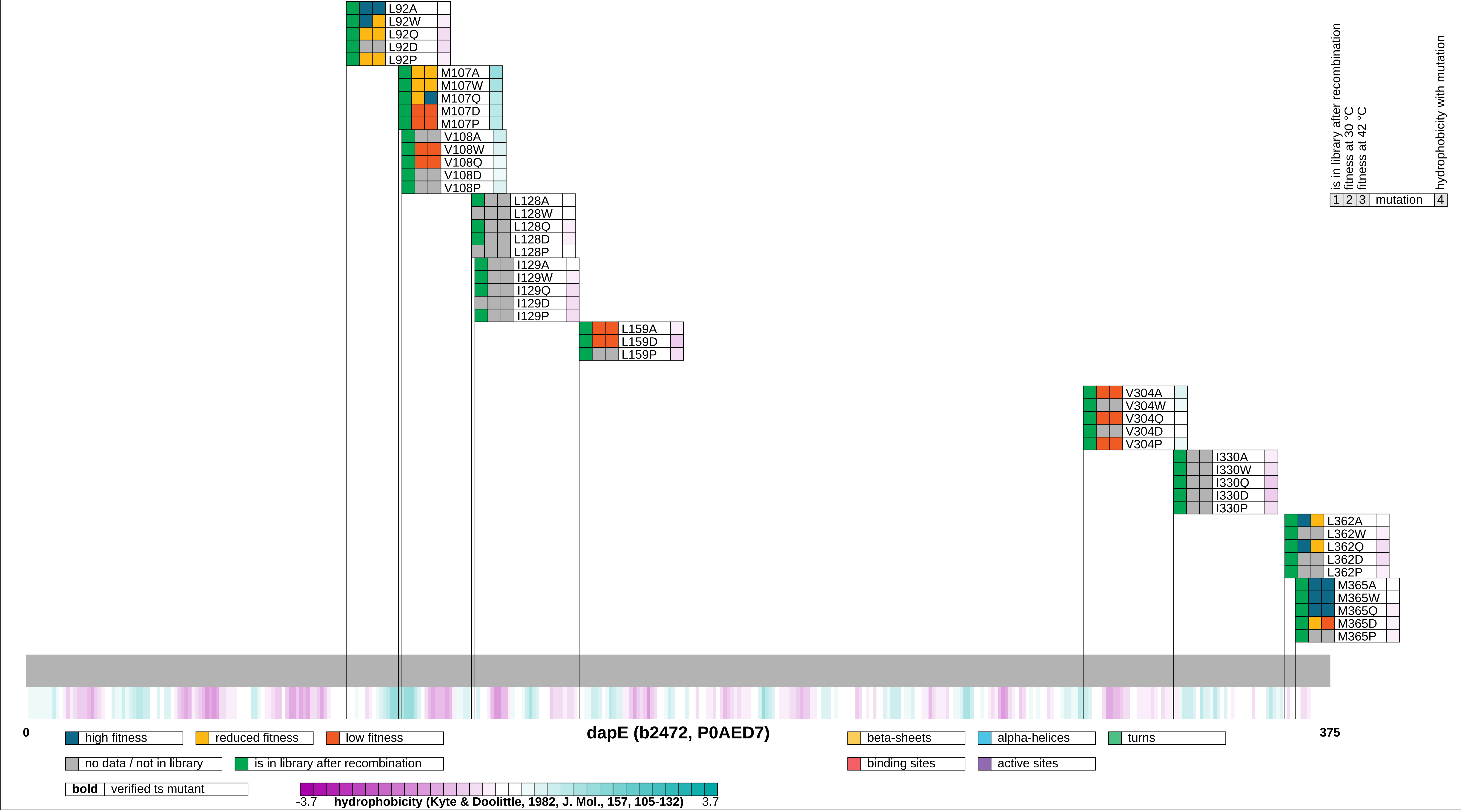

L92A
L92W
L92Q
L92D
L92P
M107A
M107W
M107Q
M107D
M107P
V108A
V108W
V108Q
V108D
V108P
L128A
L128W
L128Q
L128D
L128P
I129A
I129W
I129Q
I129D
I129P
L159A
L159D
L159P
V304A
V304W
V304Q
V304D
V304P
I330A
I330W
I330Q
I330D
I330P
L362A
L362W
L362Q
L362D
L362P
M365A
M365W
M365Q
M365D
M365P
is in library after recombination
fitness at 30 °C
fitness at 42 °C
hydrophobicity with mutation
1 2 3 mutation 4
0
375
dapE (b2472, P0AED7)
high fitness
reduced fitness
low fitness
beta-sheets
alpha-helices
turns
no data / not in library
is in library after recombination
binding sites
active sites
bold verified ts mutant
-3.7
hydrophobicity (Kyte & Doolittle, 1982, J. Mol., 157, 105-132)
3.7

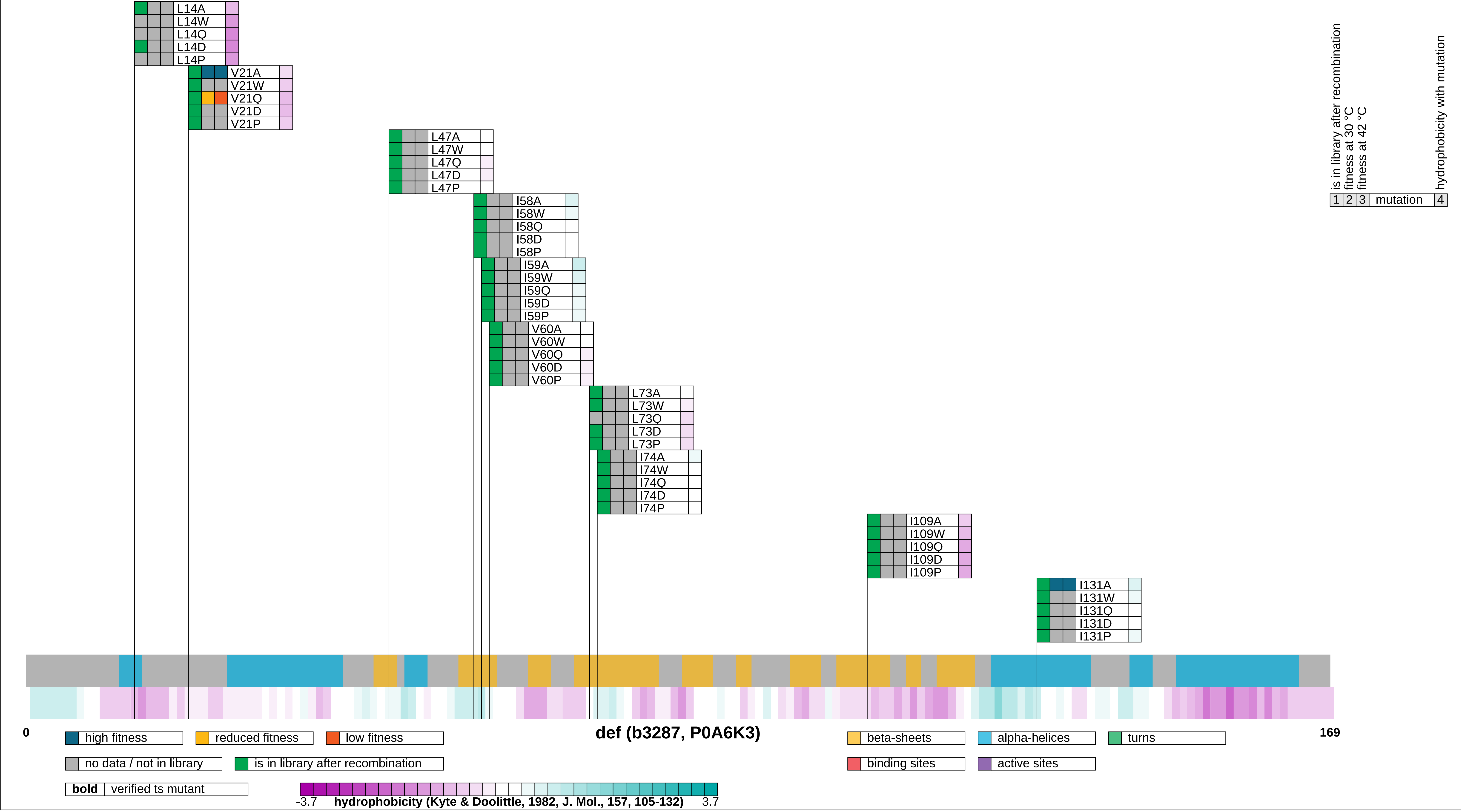

L14A
L14W
L14Q
L14D
L14P
V21A
V21W
V21Q
V21D
V21P
L47A
L47W
L47Q
L47D
L47P
I58A
I58W
I58Q
I58D
I58P
I59A
I59W
I59Q
I59D
I59P
V60A
V60W
V60Q
V60D
V60P
L73A
L73W
L73Q
L73D
L73P
I74A
I74W
I74Q
I74D
I74P
I109A
I109W
I109Q
I109D
I109P
I131A
I131W
I131Q
I131D
I131P
is in library after recombination
fitness at 30 °C
fitness at 42 °C
hydrophobicity with mutation
1 2 3 mutation 4
0
def (b3287, P0A6K3)
169
high fitness
reduced fitness
low fitness
no data / not in library
is in library after recombination
bold verified ts mutant
beta-sheets
alpha-helices
turns
binding sites
active sites
-3.7 hydrophobicity (Kyte & Doolittle, 1982, J. Mol., 157, 105-132) 3.7

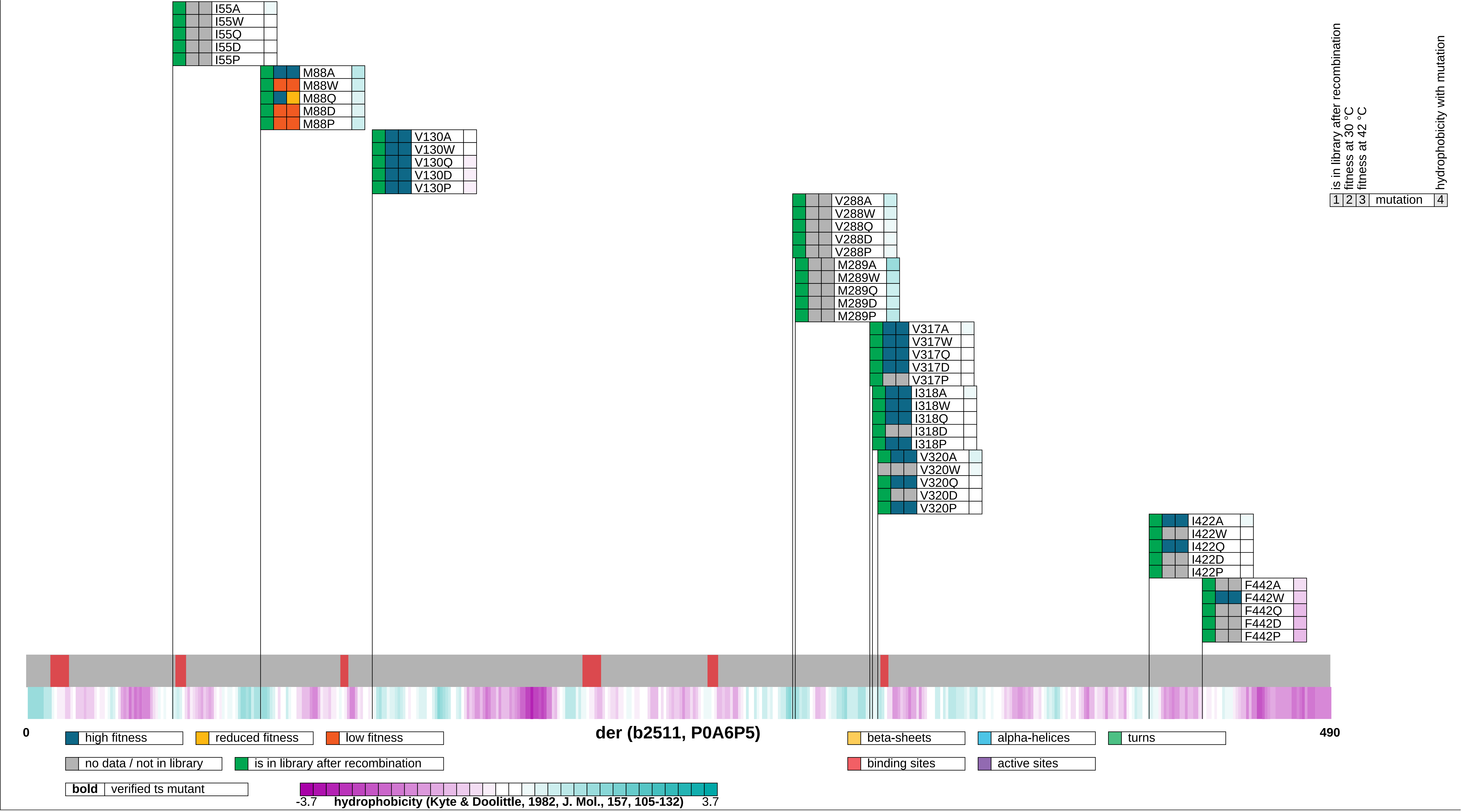

I55A
I55W
I55Q
I55D
I55P
M88A
M88W
M88Q
M88D
M88P
V130A
V130W
V130Q
V130D
V130P
V288A
V288W
V288Q
V288D
V288P
M289A
M289W
M289Q
M289D
M289P
V317A
V317W
V317Q
V317D
V317P
I318A
I318W
I318Q
I318D
I318P
V320A
V320W
V320Q
V320D
V320P
I422A
I422W
I422Q
I422D
I422P
F442A
F442W
F442Q
F442D
F442P
is in library after recombination
fitness at 30 °C
fitness at 42 °C
hydrophobicity with mutation
1 2 3 mutation 4
0
der (b2511, P0A6P5)
490
high fitness
reduced fitness
low fitness
beta-sheets
alpha-helices
turns
no data / not in library
is in library after recombination
binding sites
active sites
bold verified ts mutant
-3.7 hydrophobicity (Kyte & Doolittle, 1982, J. Mol., 157, 105-132) 3.7

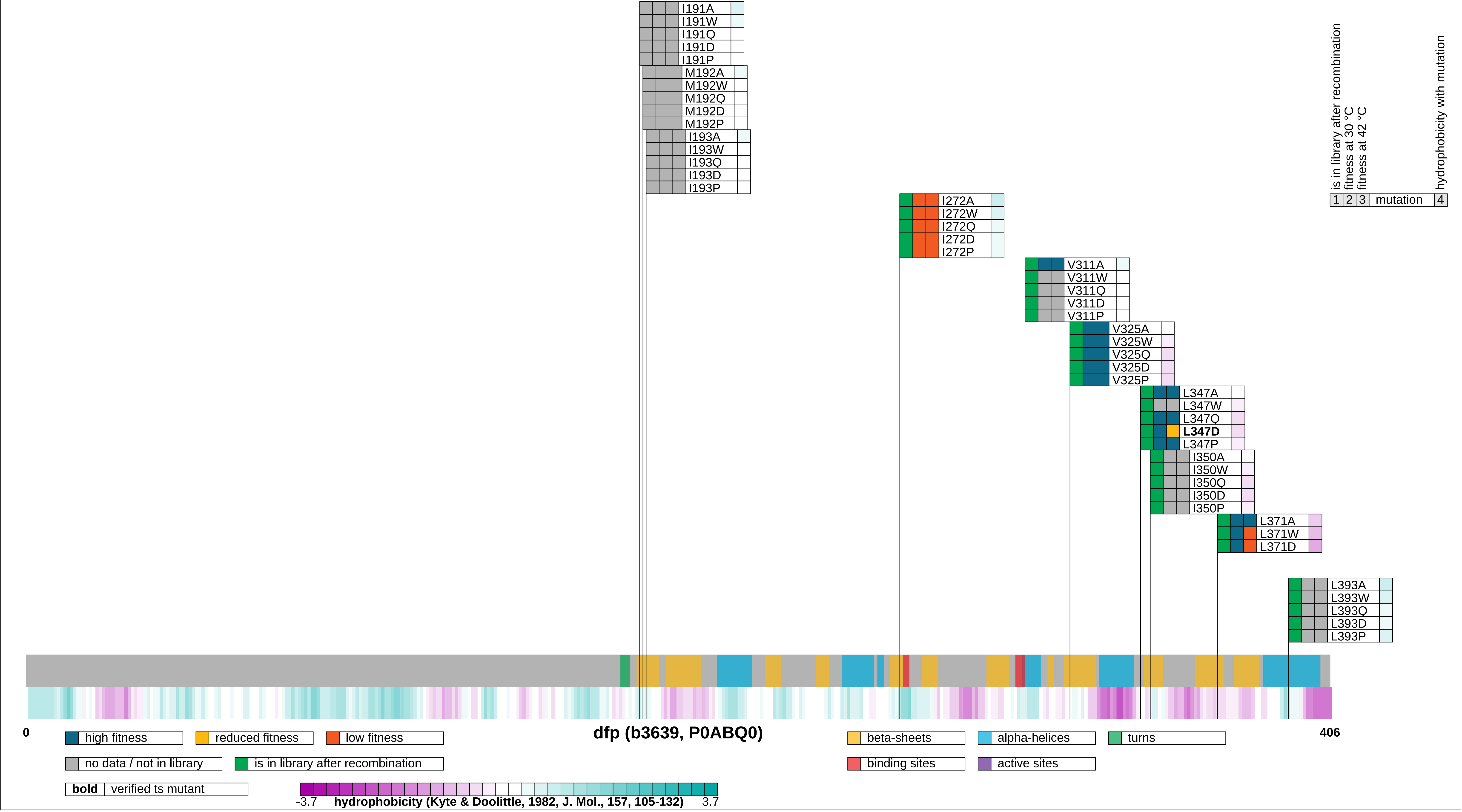

I191A
I191W
I191Q
I191D
I191P
M192A
M192W
M192Q
M192D
M192P
I193A
I193W
I193Q
I193D
I193P
I272A
I272W
I272Q
I272D
I272P
V311A
V311W
V311Q
V311D
V311P
V325A
V325W
V325Q
V325D
V325P
L347A
L347W
L347Q
L347D
L347P
I350A
I350W
I350Q
I350D
I350P
L371A
L371W
L371D
L393A
L393W
L393Q
L393D
L393P
is in library after recombination
fitness at 30 °C
fitness at 42 °C
hydrophobicity with mutation
1 2 3 mutation 4
0
dfp (b3639, P0ABQ0)
406
high fitness
reduced fitness
low fitness
no data / not in library
is in library after recombination
bold verified ts mutant
beta-sheets
alpha-helices
turns
binding sites
active sites
-3.7
hydrophobicity (Kyte & Doolittle, 1982, J. Mol., 157, 105-132)
3.7

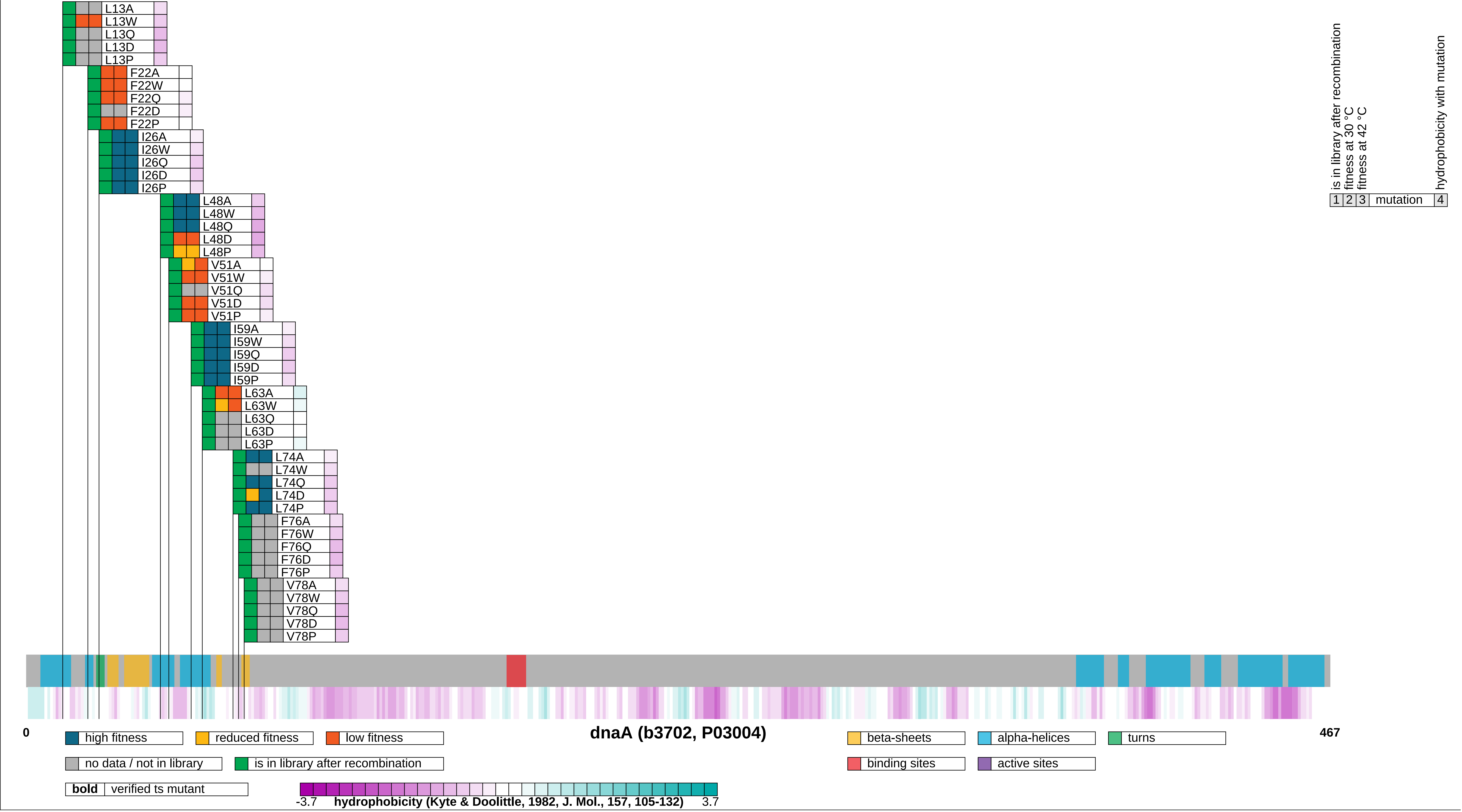
L13A
L13W
L13Q
L13D
L13P
F22A
F22W
F22Q
F22D
F22P
I26A
I26W
I26Q
I26D
I26P
L48A
L48W
L48Q
L48D
L48P
V51A
V51W
V51Q
V51D
V51P
I59A
I59W
I59Q
I59D
I59P
L63A
L63W
L63Q
L63D
L63P
L74A
L74W
L74Q
L74D
L74P
F76A
F76W
F76Q
F76D
F76P
V78A
V78W
V78Q
V78D
V78P
is in library after recombination
fitness at 30 °C
fitness at 42 °C
hydrophobicity with mutation
1 2 3 mutation 4
0
dnaA (b3702, P03004)
467
high fitness
reduced fitness
low fitness
no data / not in library
is in library after recombination
bold verified ts mutant
beta-sheets
alpha-helices
turns
binding sites
active sites
-3.7
hydrophobicity (Kyte & Doolittle, 1982, J. Mol., 157, 105-132)
3.7

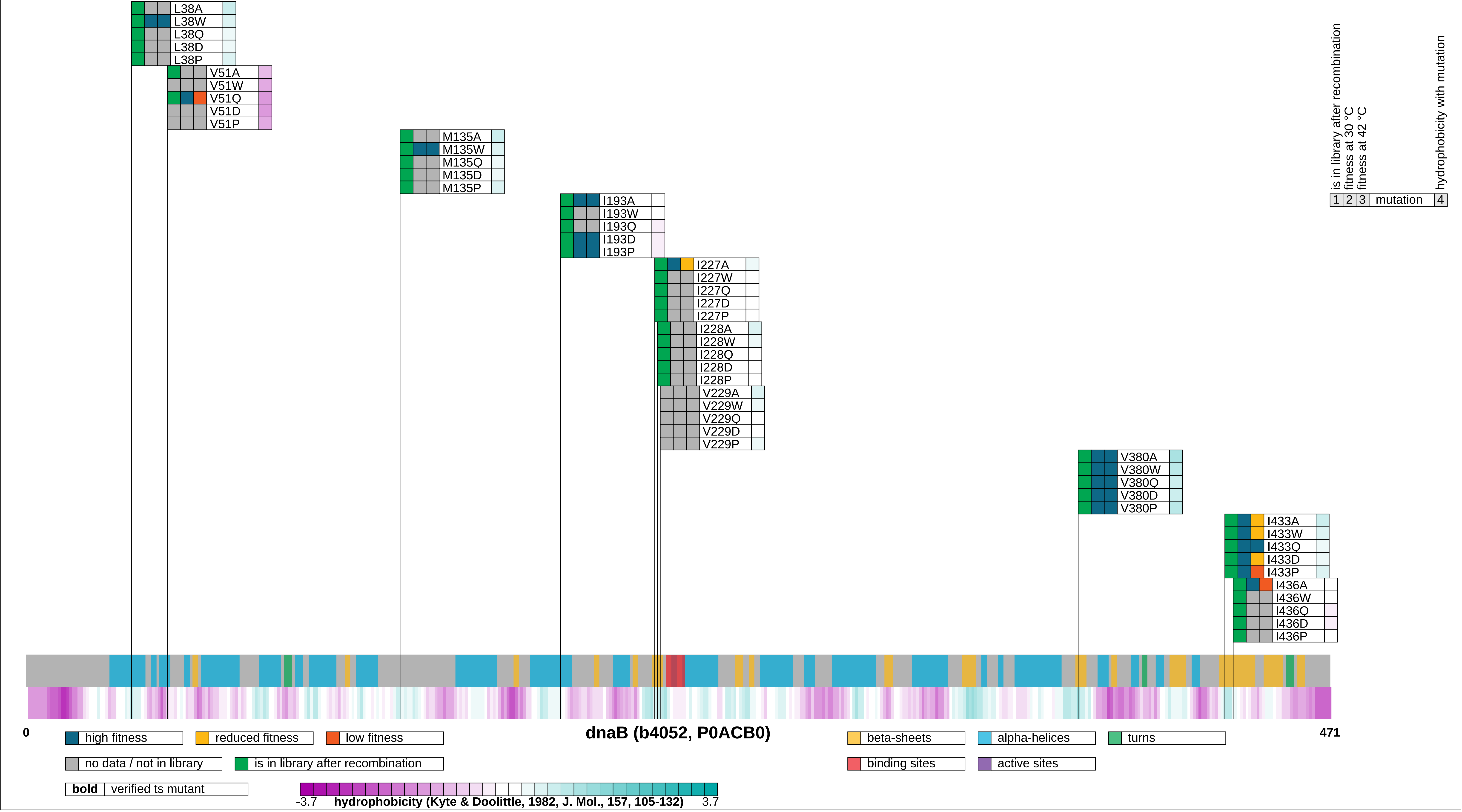
L38A
L38W
L38Q
L38D
L38P
V51A
V51W
V51Q
V51D
V51P
M135A
M135W
M135Q
M135D
M135P
I193A
I193W
I193Q
I193D
I193P
I227A
I227W
I227Q
I227D
I227P
I228A
I228W
I228Q
I228D
I228P
V229A
V229W
V229Q
V229D
V229P
V380A
V380W
V380Q
V380D
V380P
I433A
I433W
I433Q
I433D
I433P
I436A
I436W
I436Q
I436D
I436P
is in library after recombination
fitness at 30 °C
fitness at 42 °C
hydrophobicity with mutation
1 2 3 mutation 4
0
dnaB (b4052, P0ACB0)
471
high fitness
reduced fitness
low fitness
no data / not in library
is in library after recombination
bold verified ts mutant
beta-sheets
alpha-helices
turns
binding sites
active sites
-3.7 hydrophobicity (Kyte & Doolittle, 1982, J. Mol., 157, 105-132) 3.7

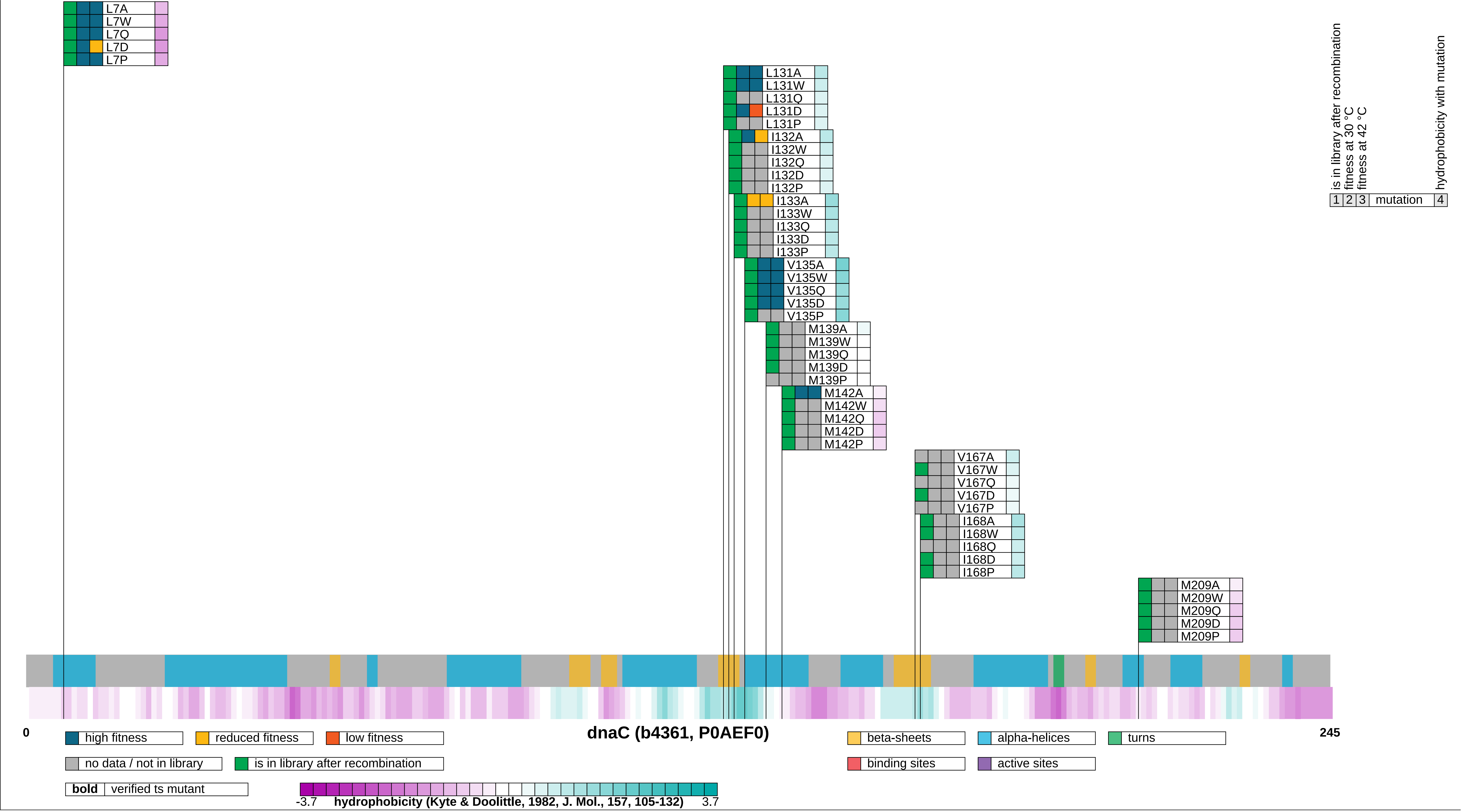

L7A
L7W
L7Q
L7D
L7P
L131A
L131W
L131Q
L131D
L131P
I132A
I132W
I132Q
I132D
I132P
I133A
I133W
I133Q
I133D
I133P
V135A
V135W
V135Q
V135D
V135P
M139A
M139W
M139Q
M139D
M139P
M142A
M142W
M142Q
M142D
M142P
V167A
V167W
V167Q
V167D
V167P
I168A
I168W
I168Q
I168D
I168P
M209A
M209W
M209Q
M209D
M209P
is in library after recombination
fitness at 30 °C
fitness at 42 °C
hydrophobicity with mutation
1 2 3 mutation 4
0
dnaC (b4361, P0AEF0)
245
high fitness
reduced fitness
low fitness
beta-sheets
alpha-helices
turns
no data / not in library
is in library after recombination
binding sites
active sites
bold verified ts mutant
-3.7 hydrophobicity (Kyte & Doolittle, 1982, J. Mol., 157, 105-132) 3.7

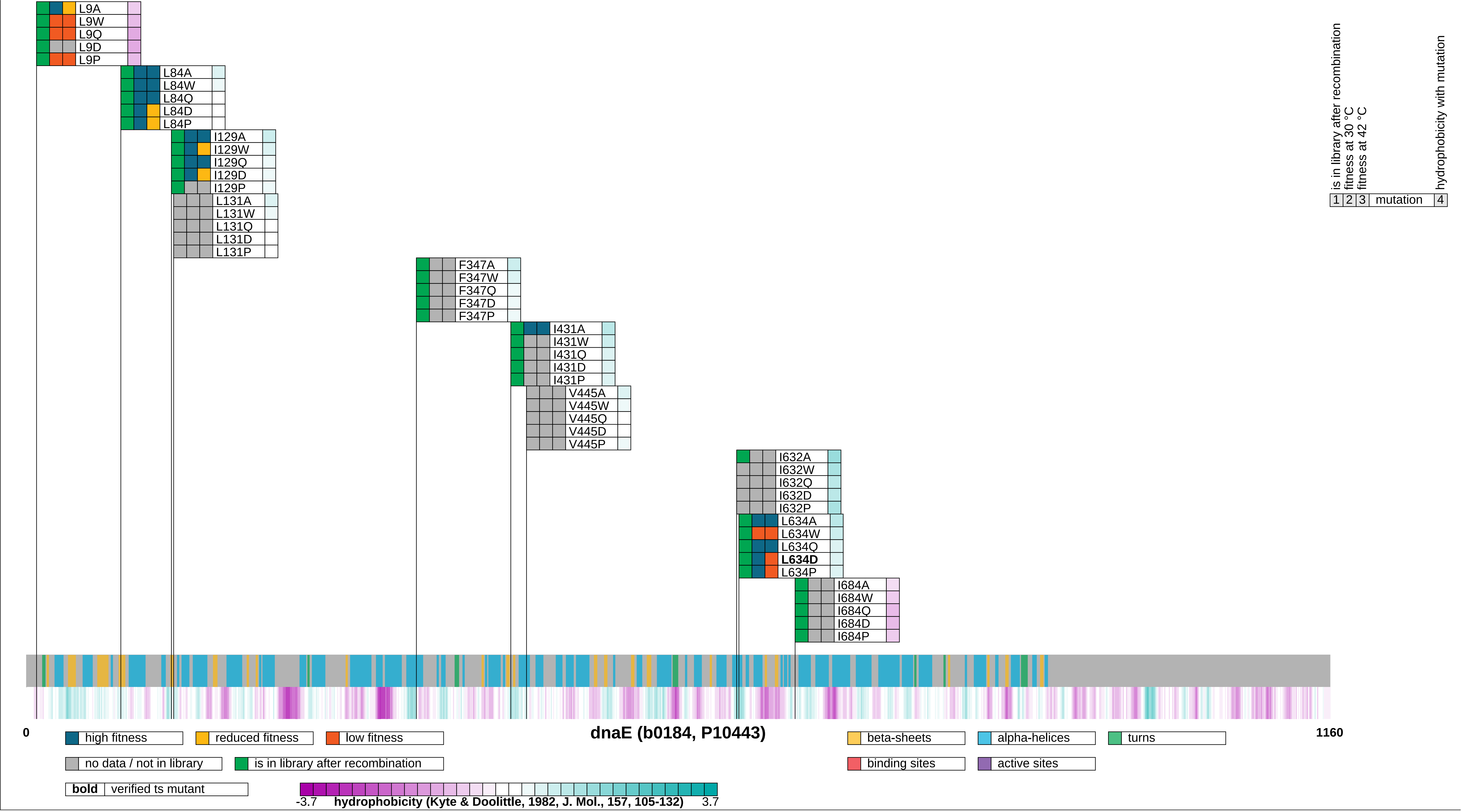
L9A
L9W
L9Q
L9D
L9P
L84A
L84W
L84Q
L84D
L84P
I129A
I129W
I129Q
I129D
I129P
L131A
L131W
L131Q
L131D
L131P
F347A
F347W
F347Q
F347D
F347P
I431A
I431W
I431Q
I431D
I431P
V445A
V445W
V445Q
V445D
V445P
I632A
I632W
I632Q
I632D
I632P
L634A
L634W
L634Q
L634D
L634P
I684A
I684W
I684Q
I684D
I684P
is in library after recombination
fitness at 30 °C
fitness at 42 °C
hydrophobicity with mutation
1 2 3 mutation 4
0
dnaE (b0184, P10443)
1160
high fitness
reduced fitness
low fitness
no data / not in library
is in library after recombination
bold verified ts mutant
beta-sheets
alpha-helices
turns
binding sites
active sites
-3.7 hydrophobicity (Kyte & Doolittle, 1982, J. Mol., 157, 105-132) 3.7

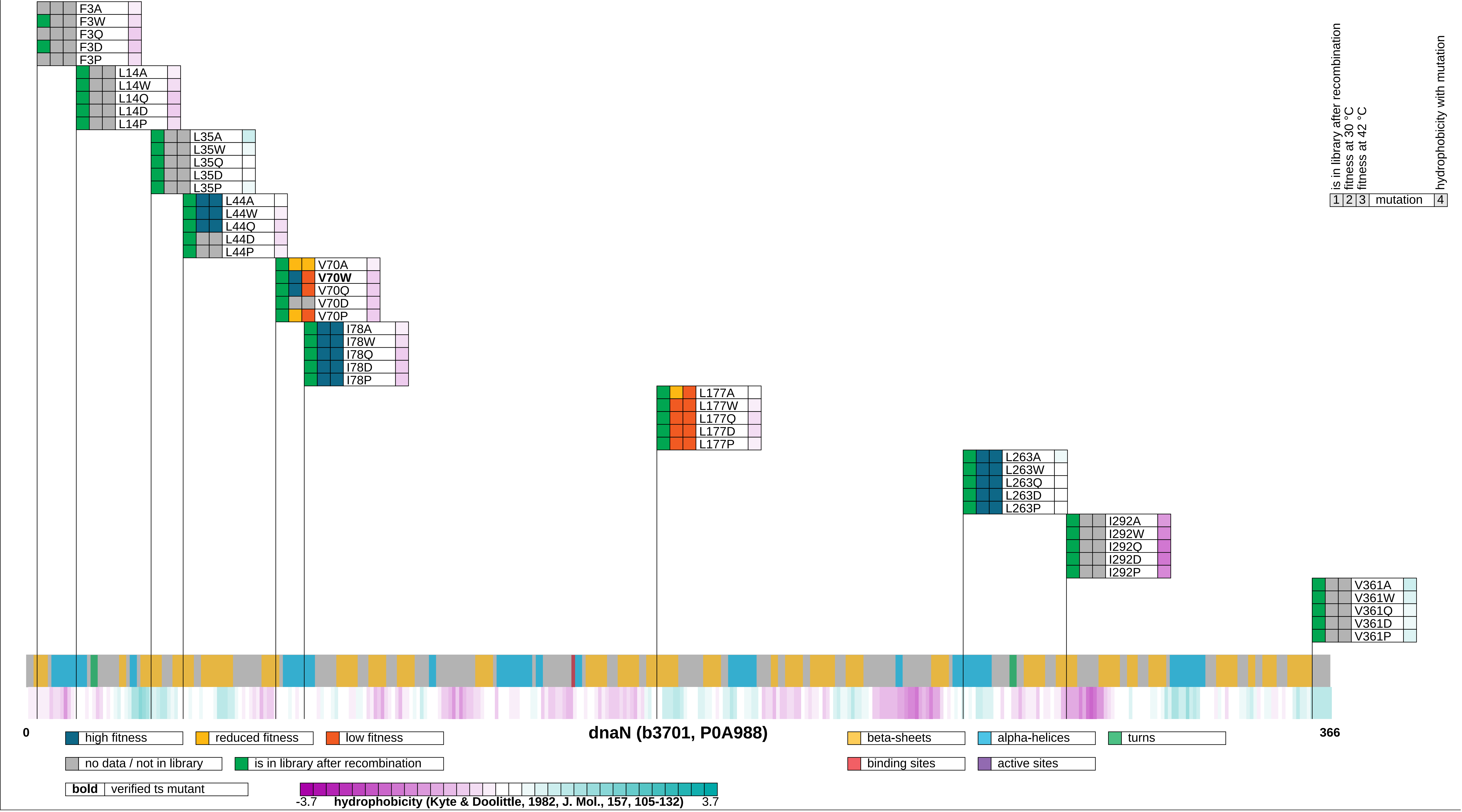
F3A
F3W
F3Q
F3D
F3P
L14A
L14W
L14Q
L14D
L14P
L35A
L35W
L35Q
L35D
L35P
L44A
L44W
L44Q
L44D
L44P
V70A
V70W
V70Q
V70D
V70P
I78A
I78W
I78Q
I78D
I78P
L177A
L177W
L177Q
L177D
L177P
L263A
L263W
L263Q
L263D
L263P
I292A
I292W
I292Q
I292D
I292P
V361A
V361W
V361Q
V361D
V361P
is in library after recombination
fitness at 30 °C
fitness at 42 °C
hydrophobicity with mutation
1 2 3 mutation 4
0
366
dnaN (b3701, P0A988)
high fitness
reduced fitness
low fitness
no data / not in library
is in library after recombination
bold verified ts mutant
beta-sheets
alpha-helices
turns
binding sites
active sites
-3.7
hydrophobicity (Kyte & Doolittle, 1982, J. Mol., 157, 105-132)
3.7

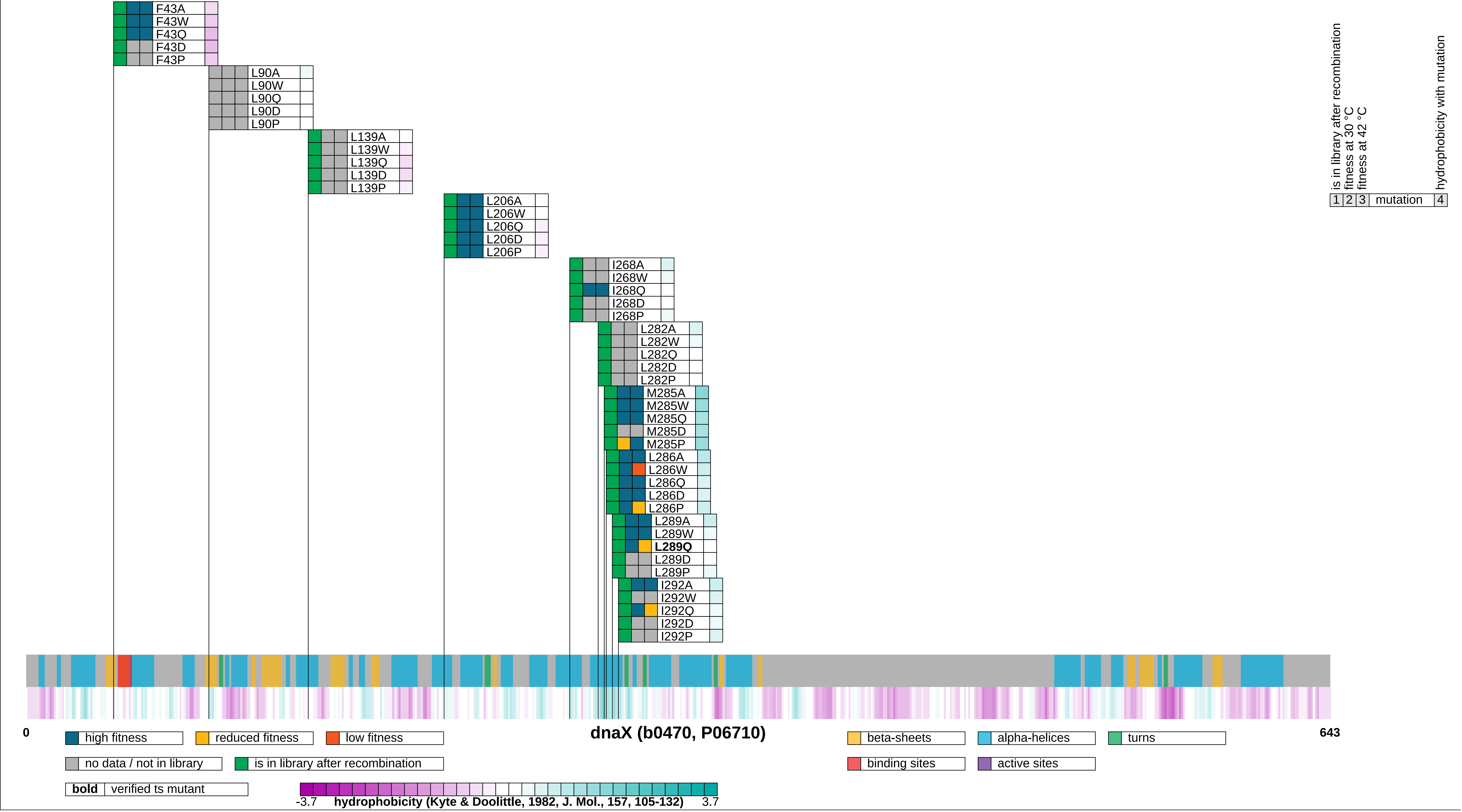

F43A
F43W
F43Q
F43D
F43P
L90A
L90W
L90Q
L90D
L90P
L139A
L139W
L139Q
L139D
L139P
L206A
L206W
L206Q
L206D
L206P
I268A
I268W
I268Q
I268D
I268P
L282A
L282W
L282Q
L282D
L282P
M285A
M285W
M285Q
M285D
M285P
L286A
L286W
L286Q
L286D
L286P
L289A
L289W
L289Q
L289D
L289P
I292A
I292W
I292Q
I292D
I292P
is in library after recombination
fitness at 30 °C
fitness at 42 °C
hydrophobicity with mutation
1 2 3 mutation 4
0
dnaX (b0470, P06710)
643
high fitness
reduced fitness
low fitness
beta-sheets
alpha-helices
turns
no data / not in library
is in library after recombination
binding sites
active sites
bold verified ts mutant
-3.7 hydrophobicity (Kyte & Doolittle, 1982, J. Mol., 157, 105-132) 3.7

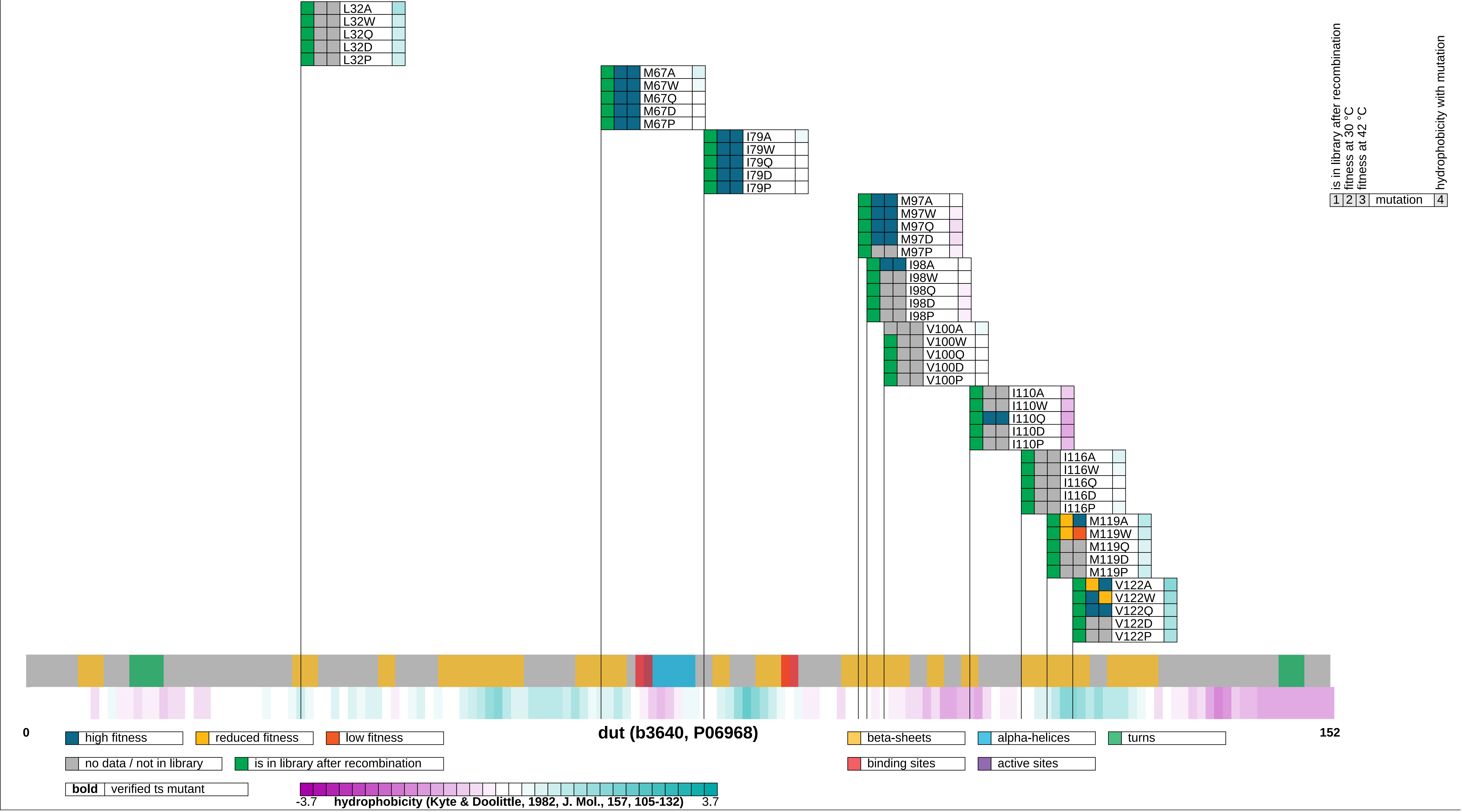

L32A
L32W
L32Q
L32D
L32P
M67A
M67W
M67Q
M67D
M67P
I79A
I79W
I79Q
I79D
I79P
M97A
M97W
M97Q
M97D
M97P
I98A
I98W
I98Q
I98D
I98P
V100A
V100W
V100Q
V100D
V100P
I110A
I110W
I110Q
I110D
I110P
I116A
I116W
I116Q
I116D
I116P
M119A
M119W
M119Q
M119D
M119P
V122A
V122W
V122Q
V122D
V122P
is in library after recombination
fitness at 30 °C
fitness at 42 °C
hydrophobicity with mutation
1 2 3 mutation 4
0
152
dut (b3640, P06968)
high fitness
reduced fitness
low fitness
no data / not in library
is in library after recombination
bold verified ts mutant
beta-sheets
alpha-helices
turns
binding sites
active sites
-3.7
hydrophobicity (Kyte & Doolittle, 1982, J. Mol., 157, 105-132)
3.7

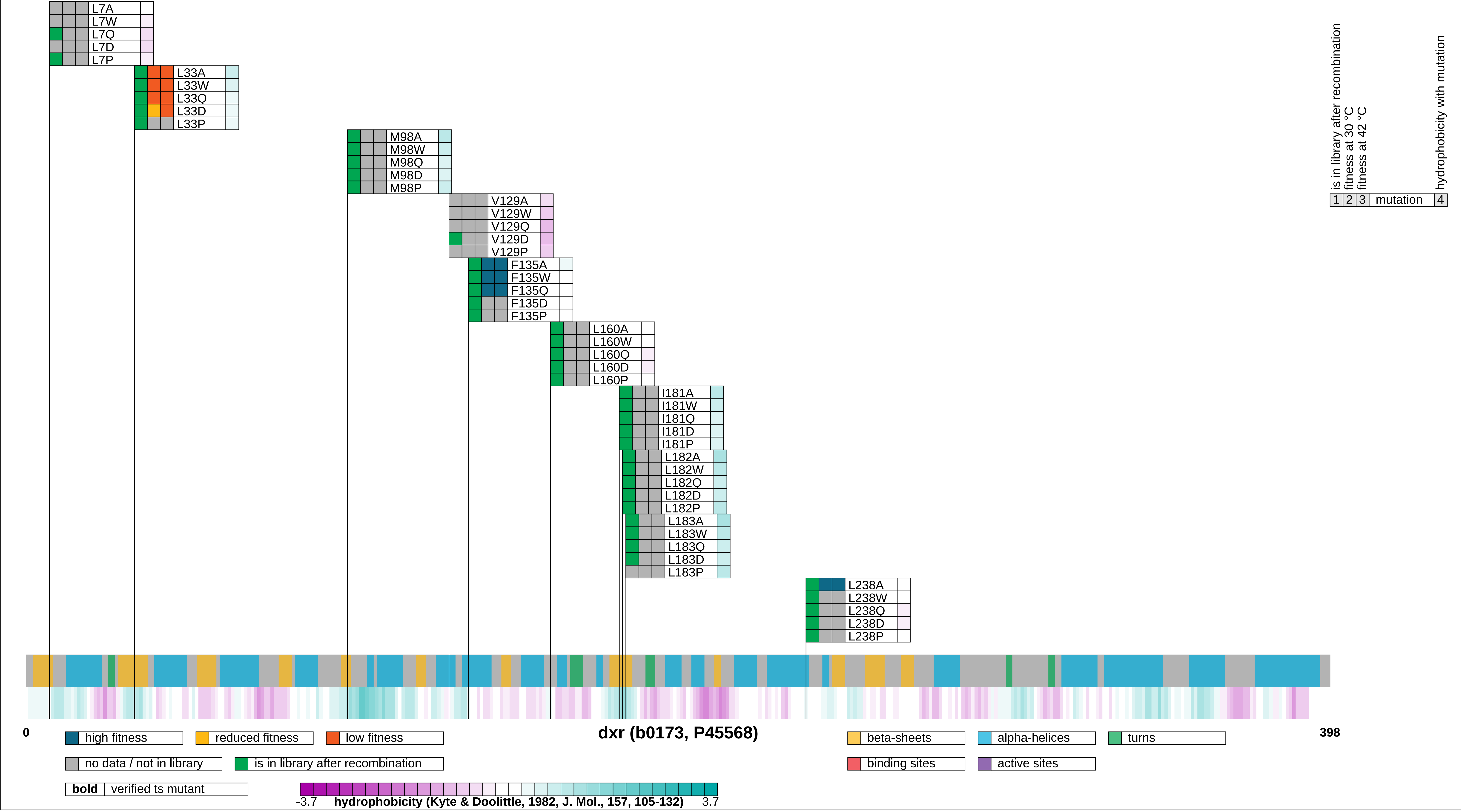
L7A
L7W
L7Q
L7D
L7P
L33A
L33W
L33Q
L33D
L33P
M98A
M98W
M98Q
M98D
M98P
V129A
V129W
V129Q
V129D
V129P
F135A
F135W
F135Q
F135D
F135P
L160A
L160W
L160Q
L160D
L160P
I181A
I181W
I181Q
I181D
I181P
L182A
L182W
L182Q
L182D
L182P
L183A
L183W
L183Q
L183D
L183P
L238A
L238W
L238Q
L238D
L238P
is in library after recombination
fitness at 30 °C
fitness at 42 °C
hydrophobicity with mutation
1 2 3 mutation 4
0
398
dxr (b0173, P45568)
high fitness
reduced fitness
low fitness
no data / not in library
is in library after recombination
bold verified ts mutant
beta-sheets
alpha-helices
turns
binding sites
active sites
-3.7
hydrophobicity (Kyte & Doolittle, 1982, J. Mol., 157, 105-132)
3.7

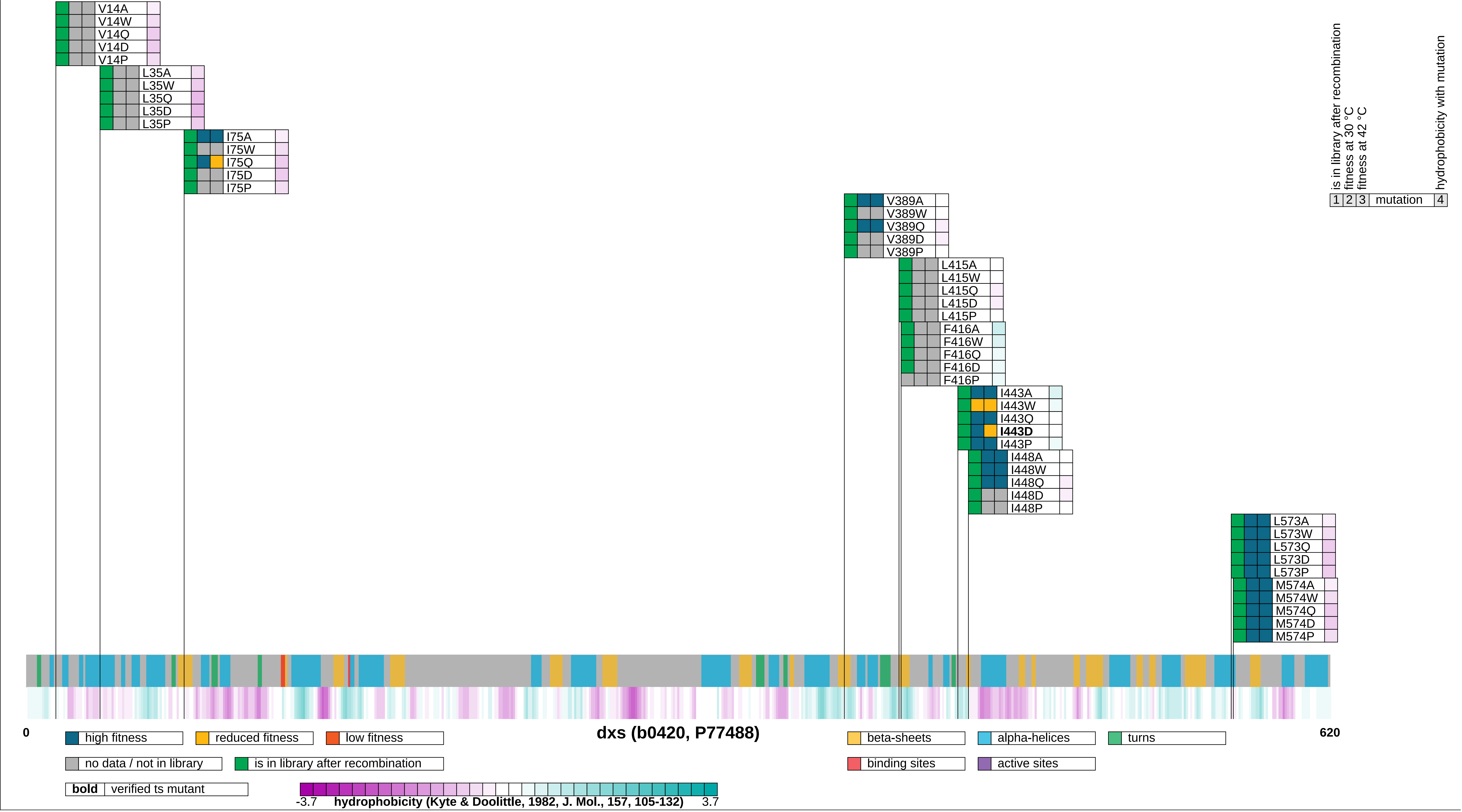

V14A
V14W
V14Q
V14D
V14P
L35A
L35W
L35Q
L35D
L35P
I75A
I75W
I75Q
I75D
I75P
V389A
V389W
V389Q
V389D
V389P
L415A
L415W
L415Q
L415D
L415P
F416A
F416W
F416Q
F416D
F416P
I443A
I443W
I443Q
I443D
I443P
I448A
I448W
I448Q
I448D
I448P
L573A
L573W
L573Q
L573D
L573P
M574A
M574W
M574Q
M574D
M574P
is in library after recombination
fitness at 30 °C
fitness at 42 °C
hydrophobicity with mutation
1 2 3 mutation 4
0
dxs (b0420, P77488)
620
high fitness
reduced fitness
low fitness
beta-sheets
alpha-helices
turns
no data / not in library
is in library after recombination
binding sites
active sites
bold verified ts mutant
-3.7 hydrophobicity (Kyte & Doolittle, 1982, J. Mol., 157, 105-132) 3.7

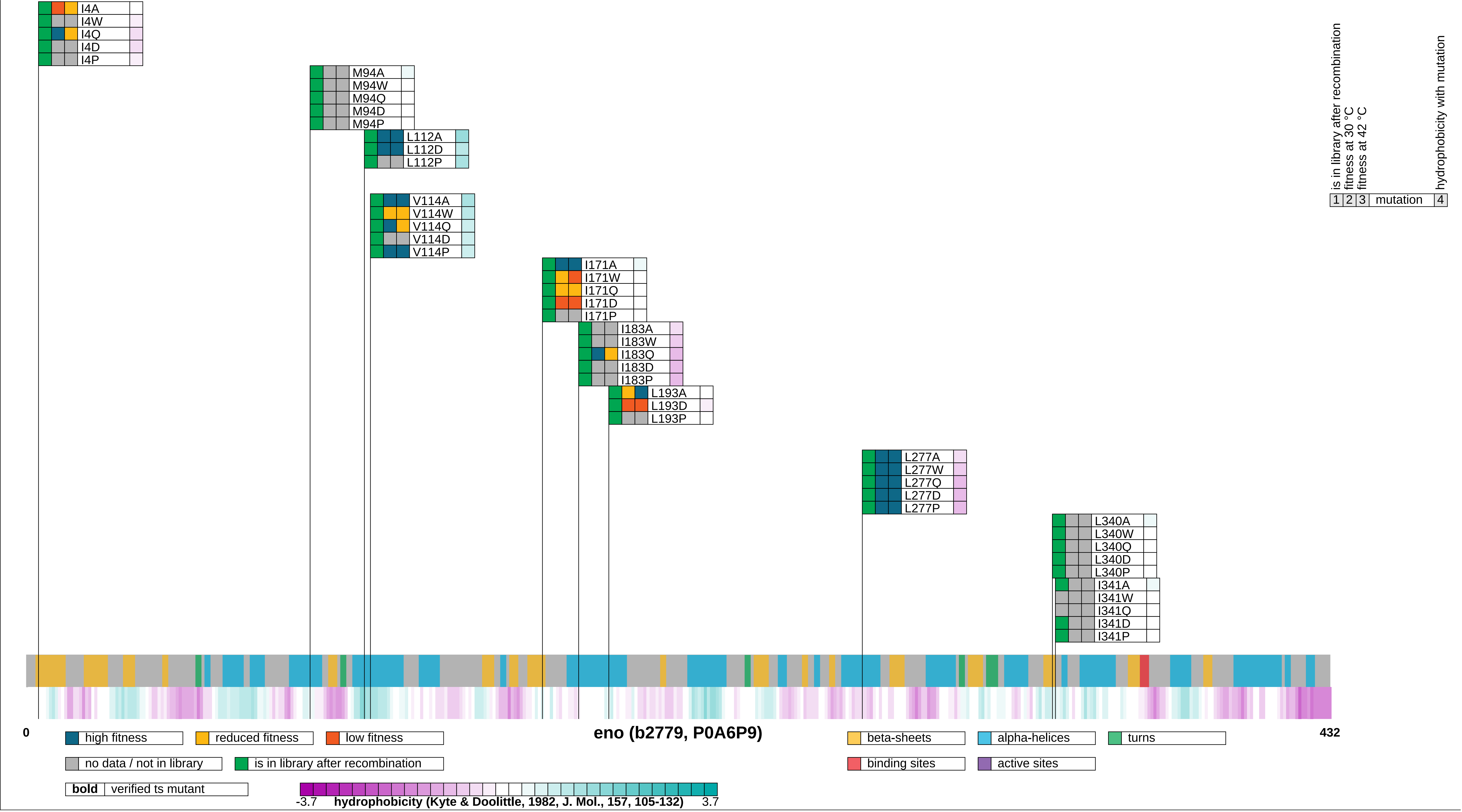
I4A
I4W
I4Q
I4D
I4P
M94A
M94W
M94Q
M94D
M94P
L112A
L112D
L112P
V114A
V114W
V114Q
V114D
V114P
I171A
I171W
I171Q
I171D
I171P
I183A
I183W
I183Q
I183D
I183P
L193A
L193D
L193P
L277A
L277W
L277Q
L277D
L277P
L340A
L340W
L340Q
L340D
L340P
I341A
I341W
I341Q
I341D
I341P
is in library after recombination
fitness at 30 °C
fitness at 42 °C
hydrophobicity with mutation
1 2 3 mutation 4
0
eno (b2779, P0A6P9)
432
high fitness
reduced fitness
low fitness
beta-sheets
alpha-helices
turns
no data / not in library
is in library after recombination
binding sites
active sites
bold verified ts mutant
-3.7 hydrophobicity (Kyte & Doolittle, 1982, J. Mol., 157, 105-132) 3.7

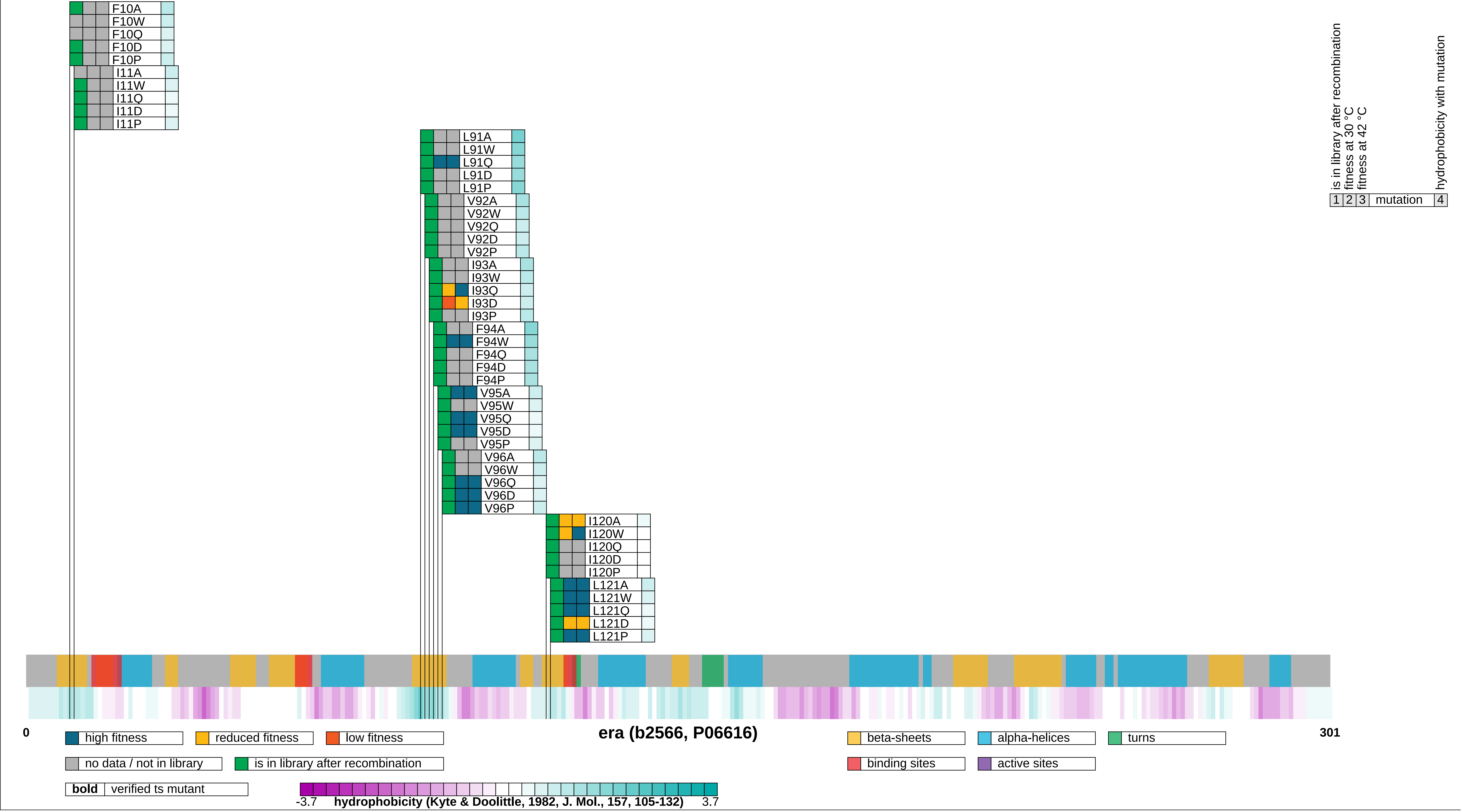
F10A
F10W
F10Q
F10D
F10P
I11A
I11W
I11Q
I11D
I11P
L91A
L91W
L91Q
L91D
L91P
V92A
V92W
V92Q
V92D
V92P
I93A
I93W
I93Q
I93D
I93P
F94A
F94W
F94Q
F94D
F94P
V95A
V95W
V95Q
V95D
V95P
V96A
V96W
V96Q
V96D
V96P
I120A
I120W
I120Q
I120D
I120P
L121A
L121W
L121Q
L121D
L121P
is in library after recombination
fitness at 30 °C
fitness at 42 °C
hydrophobicity with mutation
1 2 3 mutation 4
0
era (b2566, P06616)
301
high fitness
reduced fitness
low fitness
no data / not in library
is in library after recombination
beta-sheets
alpha-helices
turns
binding sites
active sites
bold verified ts mutant
-3.7 hydrophobicity (Kyte & Doolittle, 1982, J. Mol., 157, 105-132) 3.7

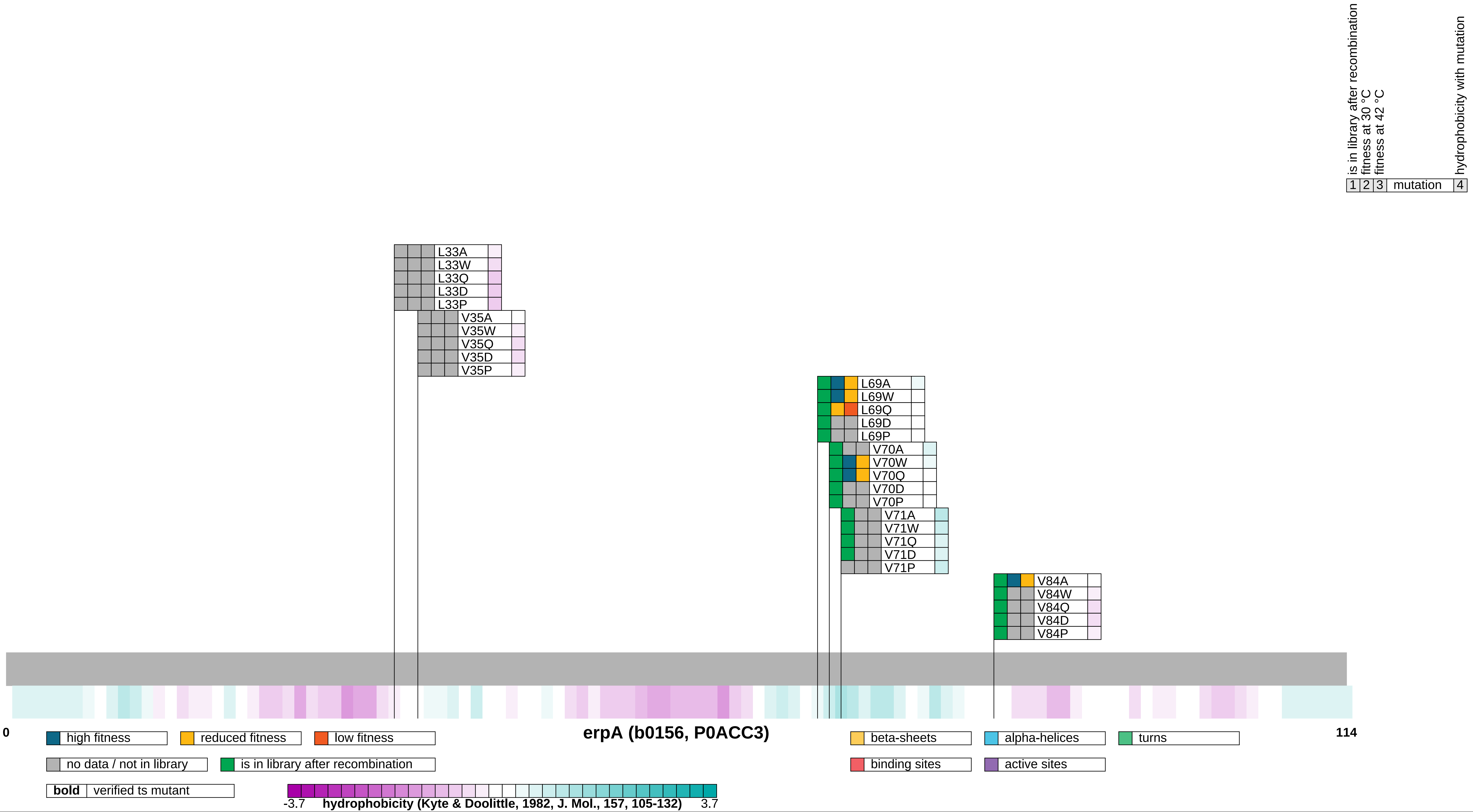
is in library after recombination
fitness at 30 °C
fitness at 42 °C
hydrophobicity with mutation
1 2 3 mutation 4
L33A
L33W
L33Q
L33D
L33P
V35A
V35W
V35Q
V35D
V35P
L69A
L69W
L69Q
L69D
L69P
V70A
V70W
V70Q
V70D
V70P
V71A
V71W
V71Q
V71D
V71P
V84A
V84W
V84Q
V84D
V84P
0
erpA (b0156, P0ACC3)
114
high fitness
reduced fitness
low fitness
beta-sheets
alpha-helices
turns
no data / not in library
is in library after recombination
binding sites
active sites
bold verified ts mutant
-3.7
hydrophobicity (Kyte & Doolittle, 1982, J. Mol., 157, 105-132)
3.7

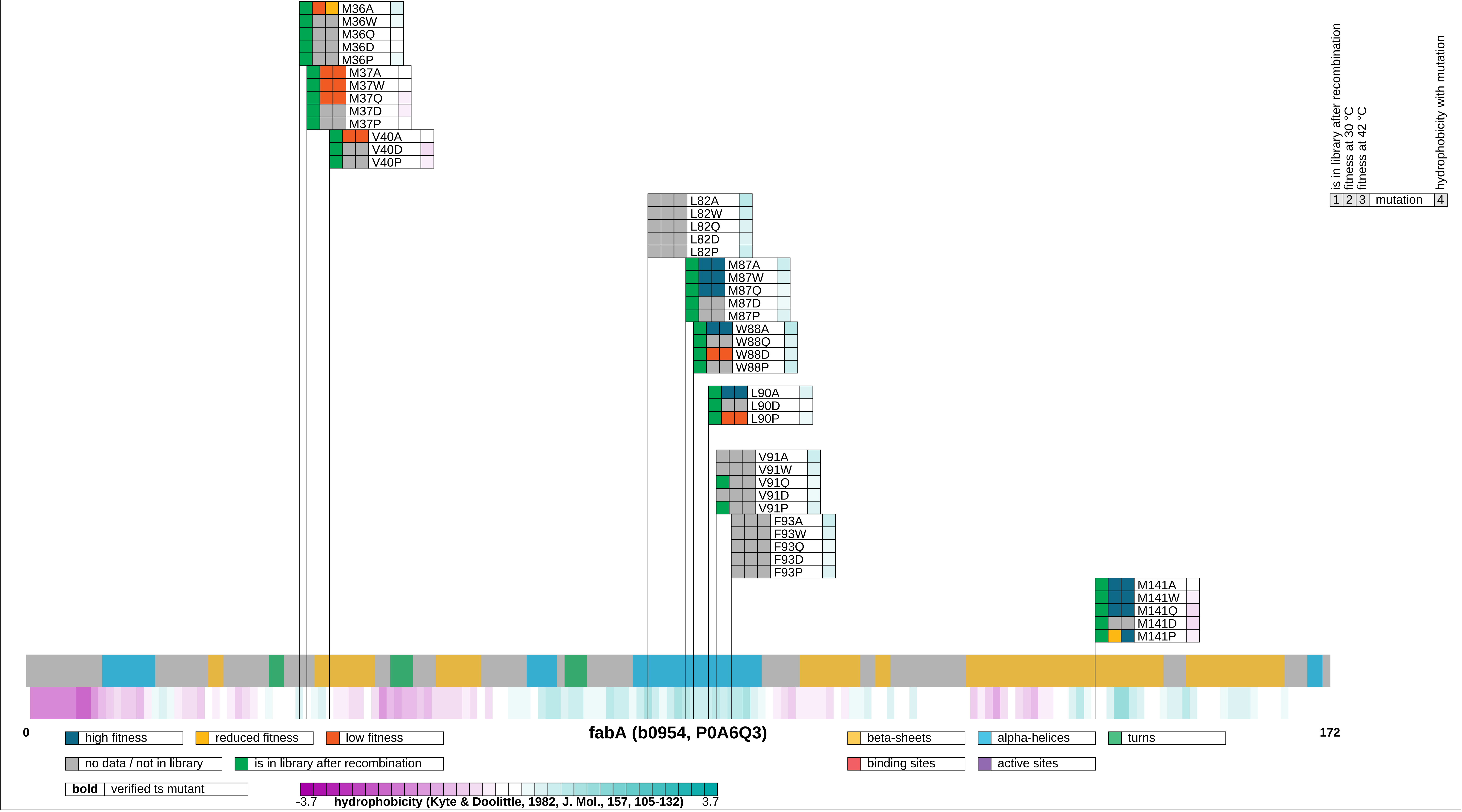

M36A
M36W
M36Q
M36D
M36P
M37A
M37W
M37Q
M37D
M37P
V40A
V40D
V40P
L82A
L82W
L82Q
L82D
L82P
M87A
M87W
M87Q
M87D
M87P
W88A
W88Q
W88D
W88P
L90A
L90D
L90P
V91A
V91W
V91Q
V91D
V91P
F93A
F93W
F93Q
F93D
F93P
M141A
M141W
M141Q
M141D
M141P
is in library after recombination
fitness at 30 °C
fitness at 42 °C
hydrophobicity with mutation
1 2 3 mutation 4
0
fabA (b0954, P0A6Q3)
172
high fitness
reduced fitness
low fitness
beta-sheets
alpha-helices
turns
no data / not in library
is in library after recombination
binding sites
active sites
bold verified ts mutant
-3.7
hydrophobicity (Kyte & Doolittle, 1982, J. Mol., 157, 105-132)
3.7

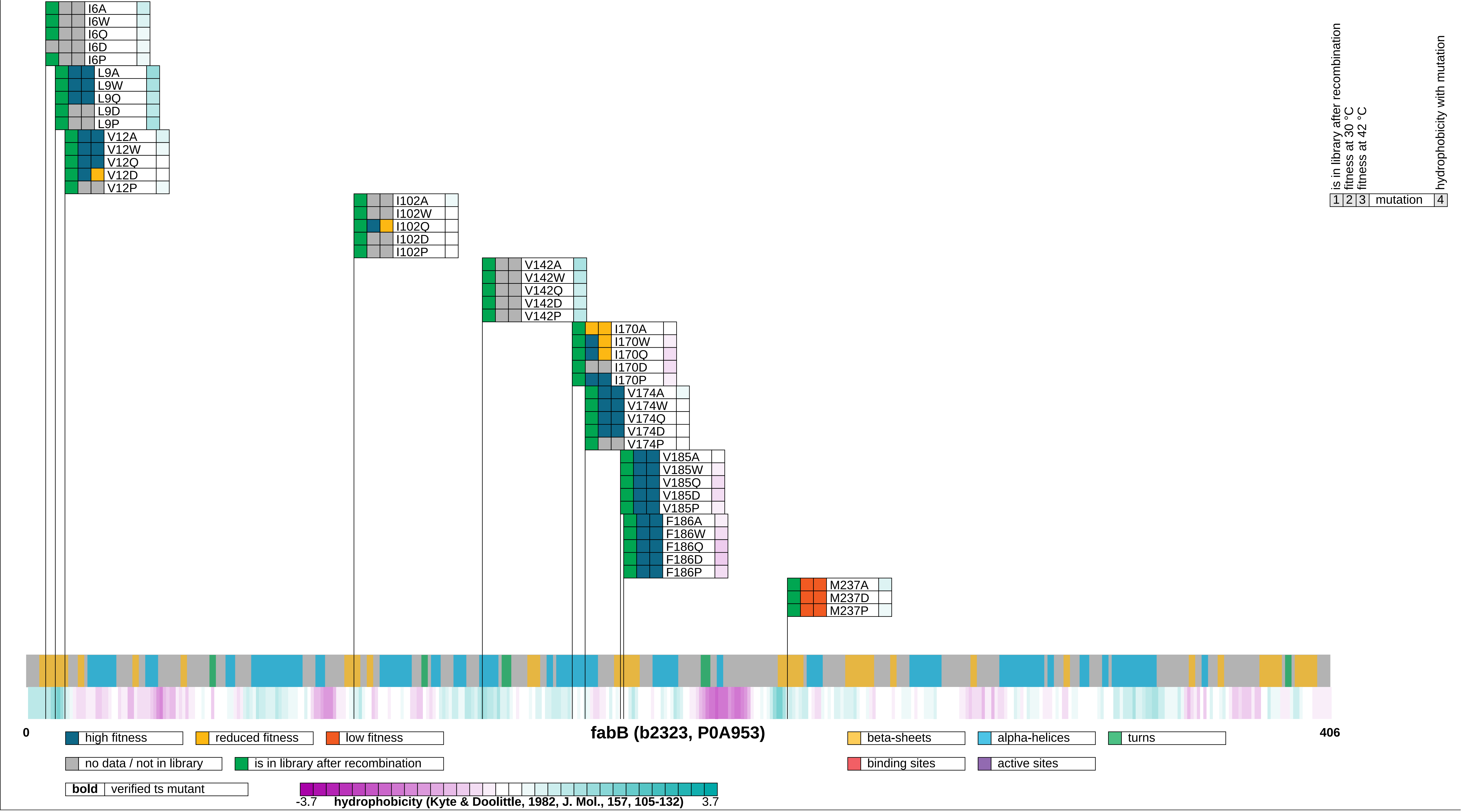

I6A
I6W
I6Q
I6D
I6P
L9A
L9W
L9Q
L9D
L9P
V12A
V12W
V12Q
V12D
V12P
I102A
I102W
I102Q
I102D
I102P
V142A
V142W
V142Q
V142D
V142P
I170A
I170W
I170Q
I170D
I170P
V174A
V174W
V174Q
V174D
V174P
V185A
V185W
V185Q
V185D
V185P
F186A
F186W
F186Q
F186D
F186P
M237A
M237D
M237P
is in library after recombination
fitness at 30 °C
fitness at 42 °C
hydrophobicity with mutation
1 2 3 mutation 4
0
fabB (b2323, P0A953)
406
high fitness
reduced fitness
low fitness
beta-sheets
alpha-helices
turns
no data / not in library
is in library after recombination
binding sites
active sites
bold verified ts mutant
-3.7
hydrophobicity (Kyte & Doolittle, 1982, J. Mol., 157, 105-132)
3.7

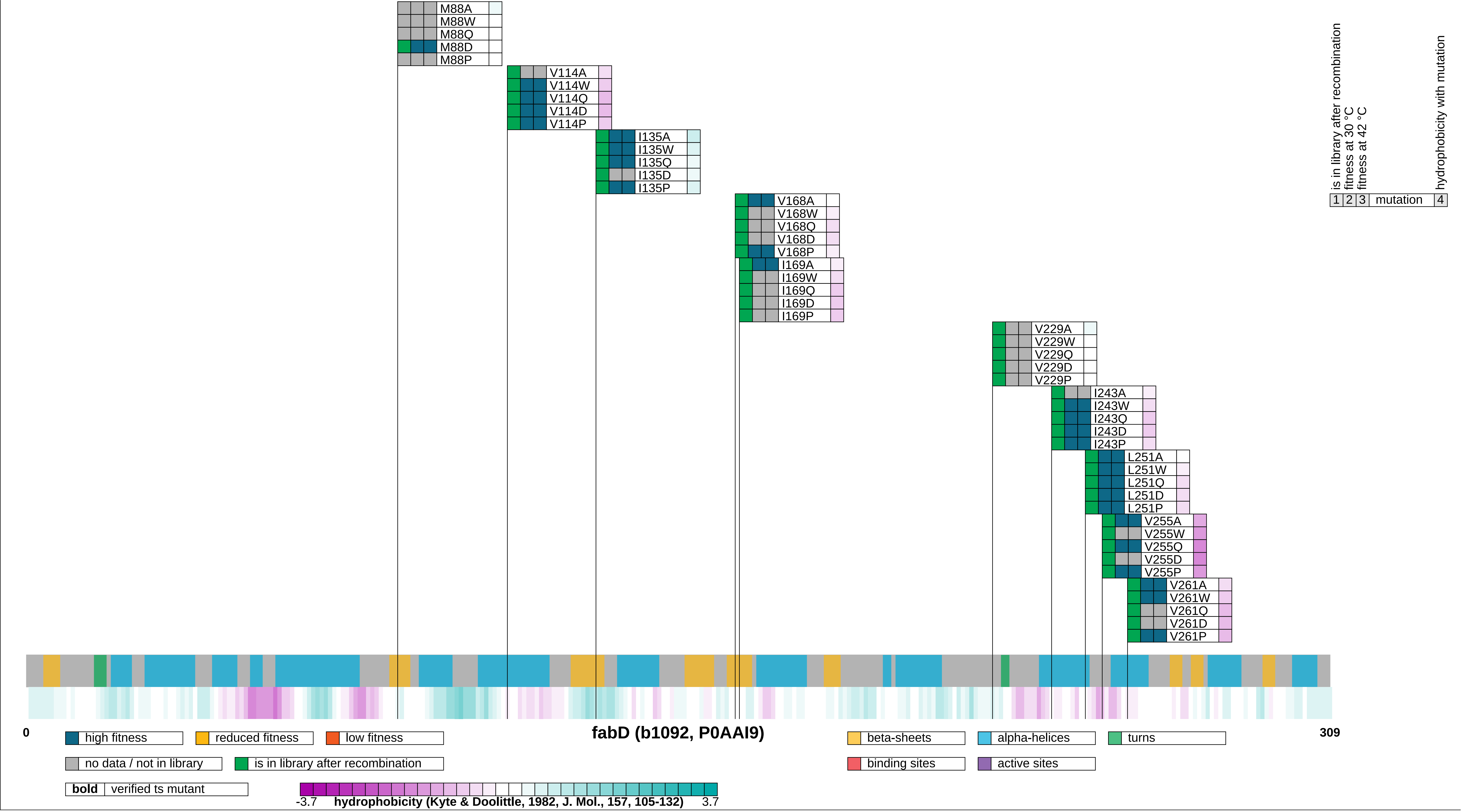

M88A
M88W
M88Q
M88D
M88P
V114A
V114W
V114Q
V114D
V114P
I135A
I135W
I135Q
I135D
I135P
V168A
V168W
V168Q
V168D
V168P
I169A
I169W
I169Q
I169D
I169P
V229A
V229W
V229Q
V229D
V229P
I243A
I243W
I243Q
I243D
I243P
L251A
L251W
L251Q
L251D
L251P
V255A
V255W
V255Q
V255D
V255P
V261A
V261W
V261Q
V261D
V261P
is in library after recombination
fitness at 30 °C
fitness at 42 °C
hydrophobicity with mutation
1 2 3 mutation 4
0
fabD (b1092, P0AAI9)
309
high fitness
reduced fitness
low fitness
beta-sheets
alpha-helices
turns
no data / not in library
is in library after recombination
binding sites
active sites
bold verified ts mutant
-3.7 hydrophobicity (Kyte & Doolittle, 1982, J. Mol., 157, 105-132) 3.7

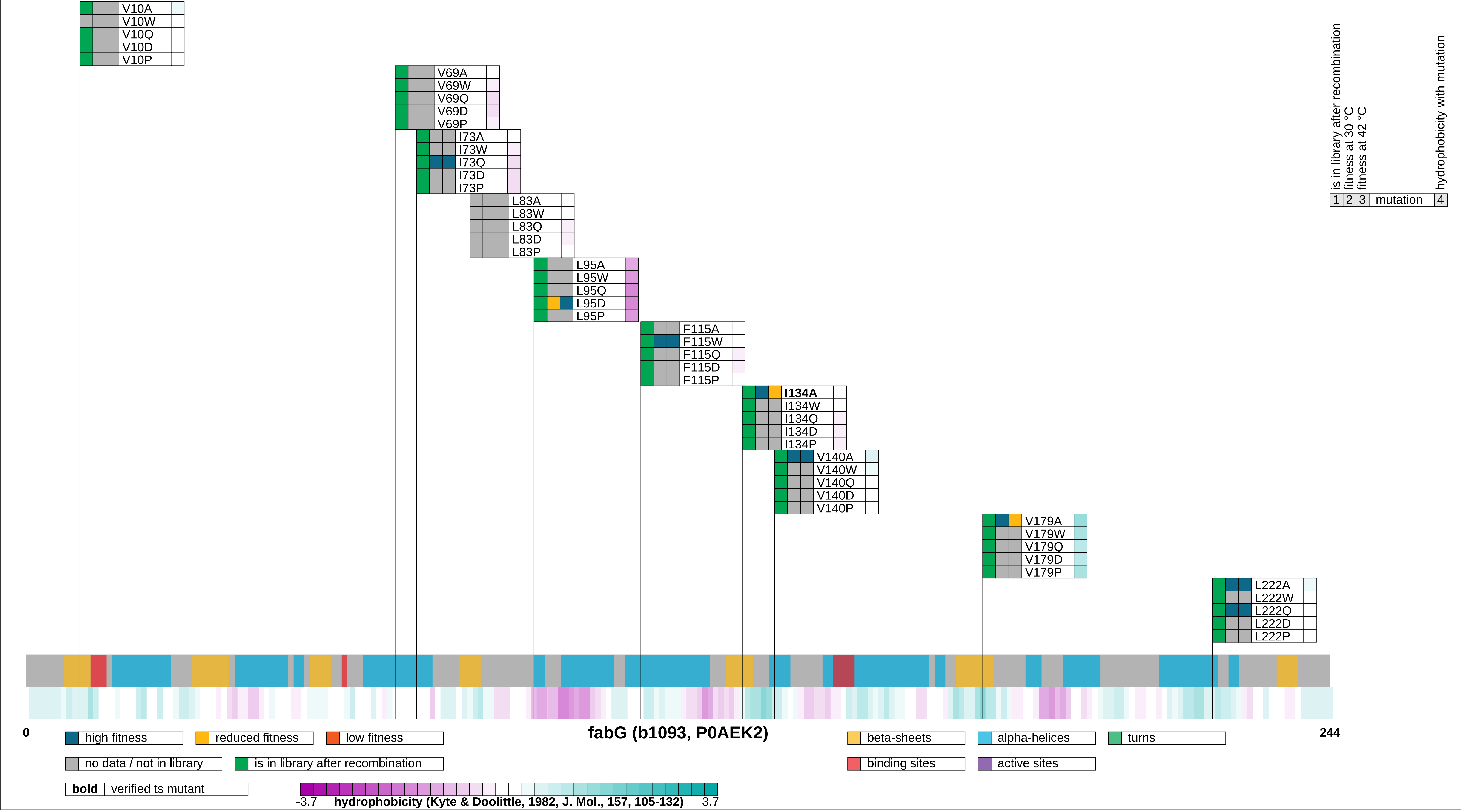

V10A
V10W
V10Q
V10D
V10P
V69A
V69W
V69Q
V69D
V69P
I73A
I73W
I73Q
I73D
I73P
L83A
L83W
L83Q
L83D
L83P
L95A
L95W
L95Q
L95D
L95P
F115A
F115W
F115Q
F115D
F115P
I134A
I134W
I134Q
I134D
I134P
V140A
V140W
V140Q
V140D
V140P
V179A
V179W
V179Q
V179D
V179P
L222A
L222W
L222Q
L222D
L222P
is in library after recombination
fitness at 30 °C
fitness at 42 °C
hydrophobicity with mutation
1 2 3 mutation 4
0
fabG (b1093, P0AEK2)
244
high fitness
reduced fitness
low fitness
beta-sheets
alpha-helices
turns
no data / not in library
is in library after recombination
binding sites
active sites
bold verified ts mutant
-3.7
hydrophobicity (Kyte & Doolittle, 1982, J. Mol., 157, 105-132)
3.7

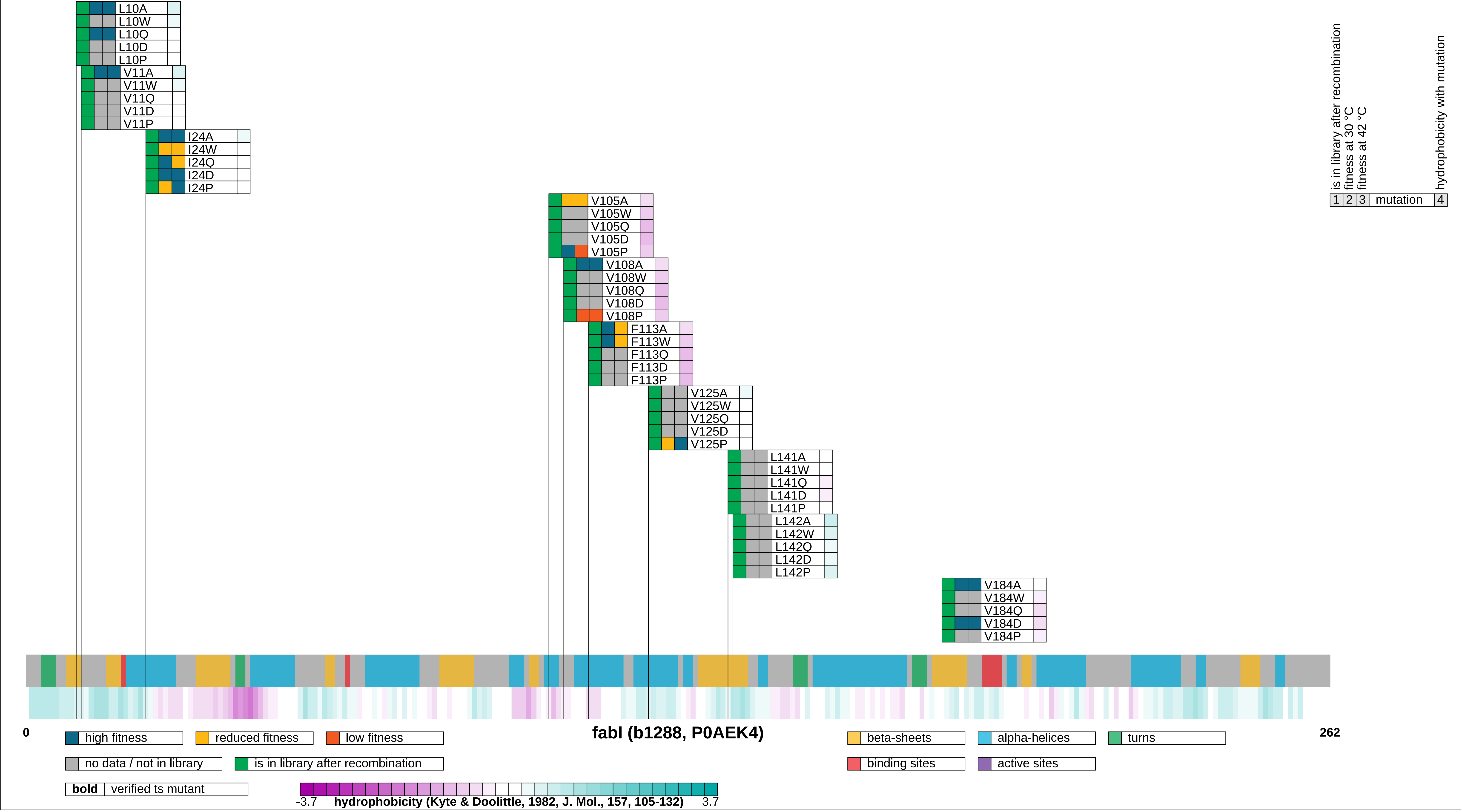

L10A
L10W
L10Q
L10D
L10P
V11A
V11W
V11Q
V11D
V11P
I24A
I24W
I24Q
I24D
I24P
V105A
V105W
V105Q
V105D
V105P
V108A
V108W
V108Q
V108D
V108P
F113A
F113W
F113Q
F113D
F113P
V125A
V125W
V125Q
V125D
V125P
L141A
L141W
L141Q
L141D
L141P
L142A
L142W
L142Q
L142D
L142P
V184A
V184W
V184Q
V184D
V184P
is in library after recombination
fitness at 30 °C
fitness at 42 °C
hydrophobicity with mutation
1 2 3 mutation 4
0
262
fabI (b1288, P0AEK4)
high fitness
reduced fitness
low fitness
no data / not in library
is in library after recombination
bold verified ts mutant
beta-sheets
alpha-helices
turns
binding sites
active sites
-3.7 hydrophobicity (Kyte & Doolittle, 1982, J. Mol., 157, 105-132) 3.7

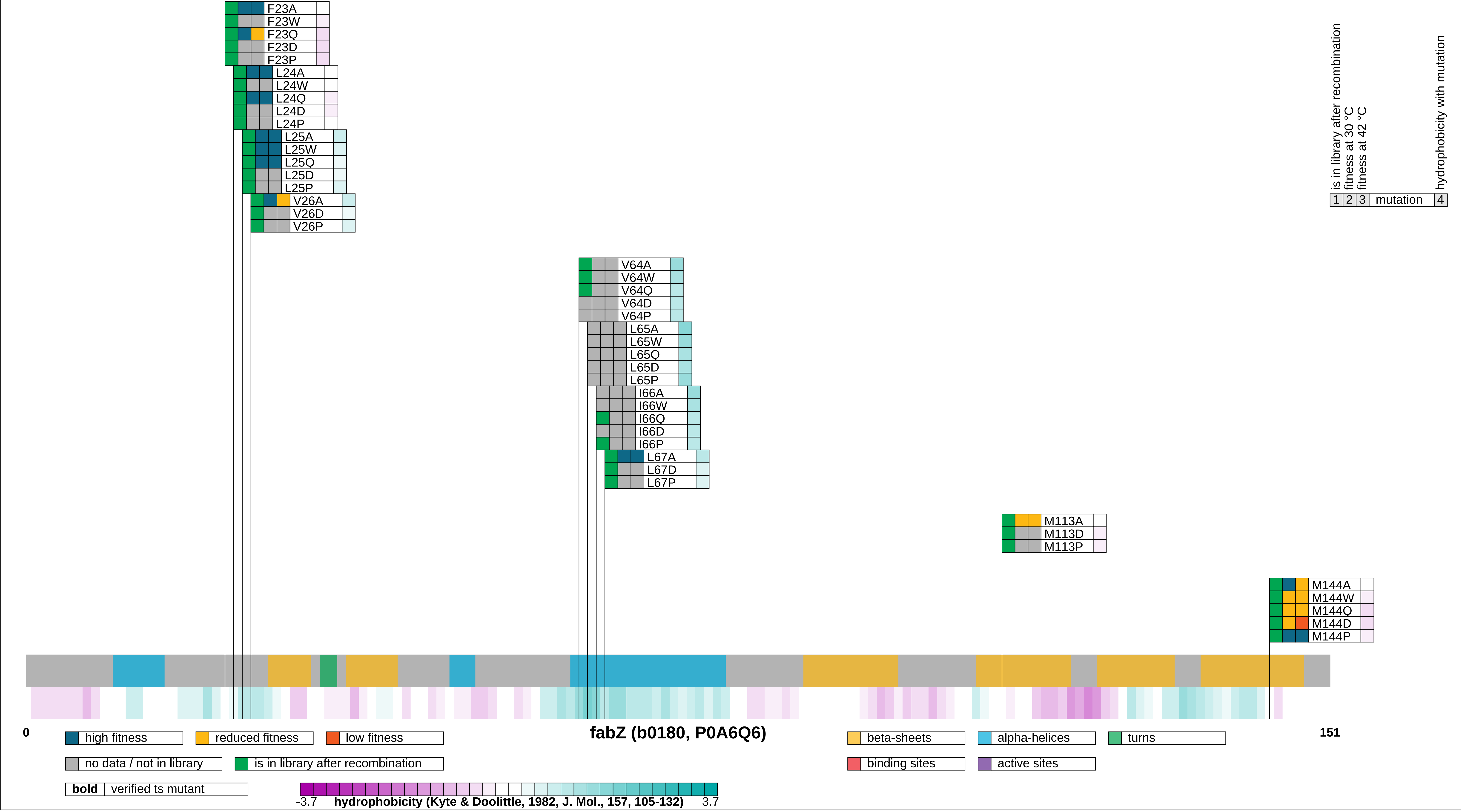
is in library after recombination
fitness at 30 °C
fitness at 42 °C
hydrophobicity with mutation
1 2 3 mutation 4
F23A
F23W
F23Q
F23D
F23P
L24A
L24W
L24Q
L24D
L24P
L25A
L25W
L25Q
L25D
L25P
V26A
V26D
V26P
V64A
V64W
V64Q
V64D
V64P
L65A
L65W
L65Q
L65D
L65P
I66A
I66W
I66Q
I66D
I66P
L67A
L67D
L67P
M113A
M113D
M113P
M144A
M144W
M144Q
M144D
M144P
0
fabZ (b0180, P0A6Q6)
151
high fitness
reduced fitness
low fitness
beta-sheets
alpha-helices
turns
no data / not in library
is in library after recombination
binding sites
active sites
bold verified ts mutant
-3.7 hydrophobicity (Kyte & Doolittle, 1982, J. Mol., 157, 105-132) 3.7

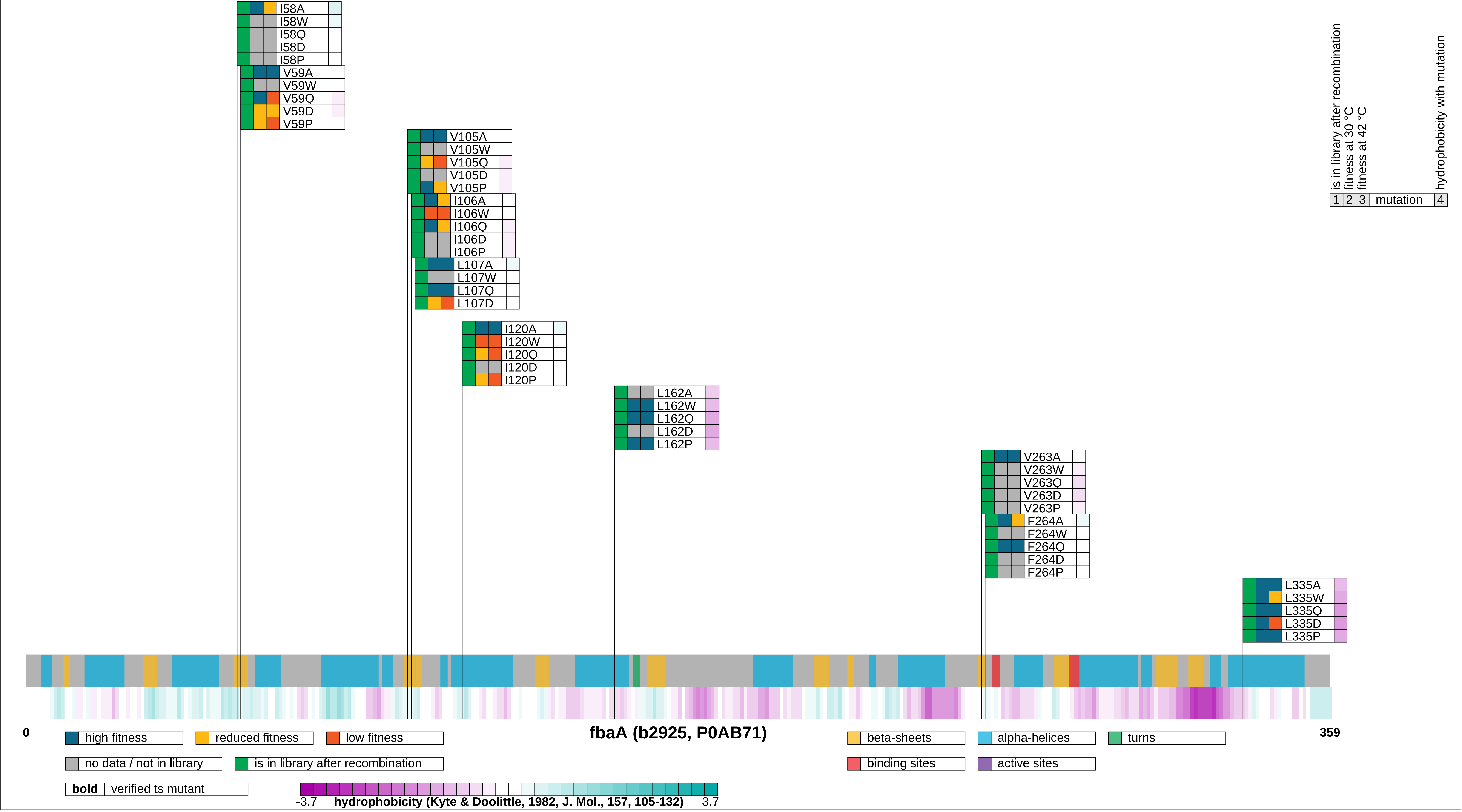

is in library after recombination
fitness at 30 °C
fitness at 42 °C
hydrophobicity with mutation
1 2 3 mutation 4
I58A
I58W
I58Q
I58D
I58P
V59A
V59W
V59Q
V59D
V59P
V105A
V105W
V105Q
V105D
V105P
I106A
I106W
I106Q
I106D
I106P
L107A
L107W
L107Q
L107D
I120A
I120W
I120Q
I120D
I120P
L162A
L162W
L162Q
L162D
L162P
V263A
V263W
V263Q
V263D
V263P
F264A
F264W
F264Q
F264D
F264P
L335A
L335W
L335Q
L335D
L335P
0
fbaA (b2925, P0AB71)
359
high fitness
reduced fitness
low fitness
beta-sheets
alpha-helices
turns
no data / not in library
is in library after recombination
binding sites
active sites
bold verified ts mutant
-3.7 hydrophobicity (Kyte & Doolittle, 1982, J. Mol., 157, 105-132) 3.7

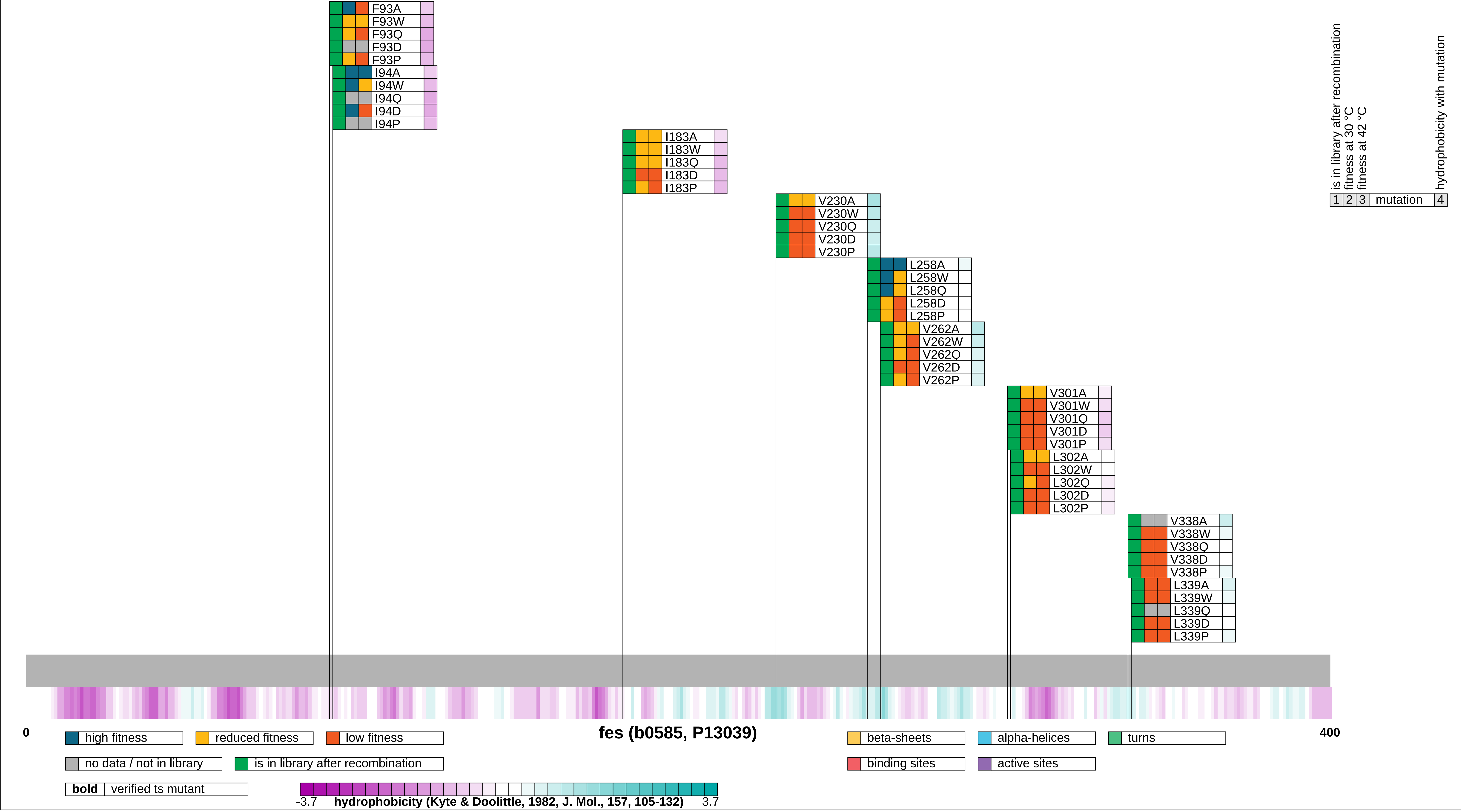
F93A
F93W
F93Q
F93D
F93P
I94A
I94W
I94Q
I94D
I94P
I183A
I183W
I183Q
I183D
I183P
V230A
V230W
V230Q
V230D
V230P
L258A
L258W
L258Q
L258D
L258P
V262A
V262W
V262Q
V262D
V262P
V301A
V301W
V301Q
V301D
V301P
L302A
L302W
L302Q
L302D
L302P
V338A
V338W
V338Q
V338D
V338P
L339A
L339W
L339Q
L339D
L339P
is in library after recombination
fitness at 30 °C
fitness at 42 °C
hydrophobicity with mutation
1 2 3 mutation 4
0
400
fes (b0585, P13039)
high fitness
reduced fitness
low fitness
no data / not in library
is in library after recombination
bold verified ts mutant
beta-sheets
alpha-helices
turns
binding sites
active sites
-3.7
hydrophobicity (Kyte & Doolittle, 1982, J. Mol., 157, 105-132)
3.7

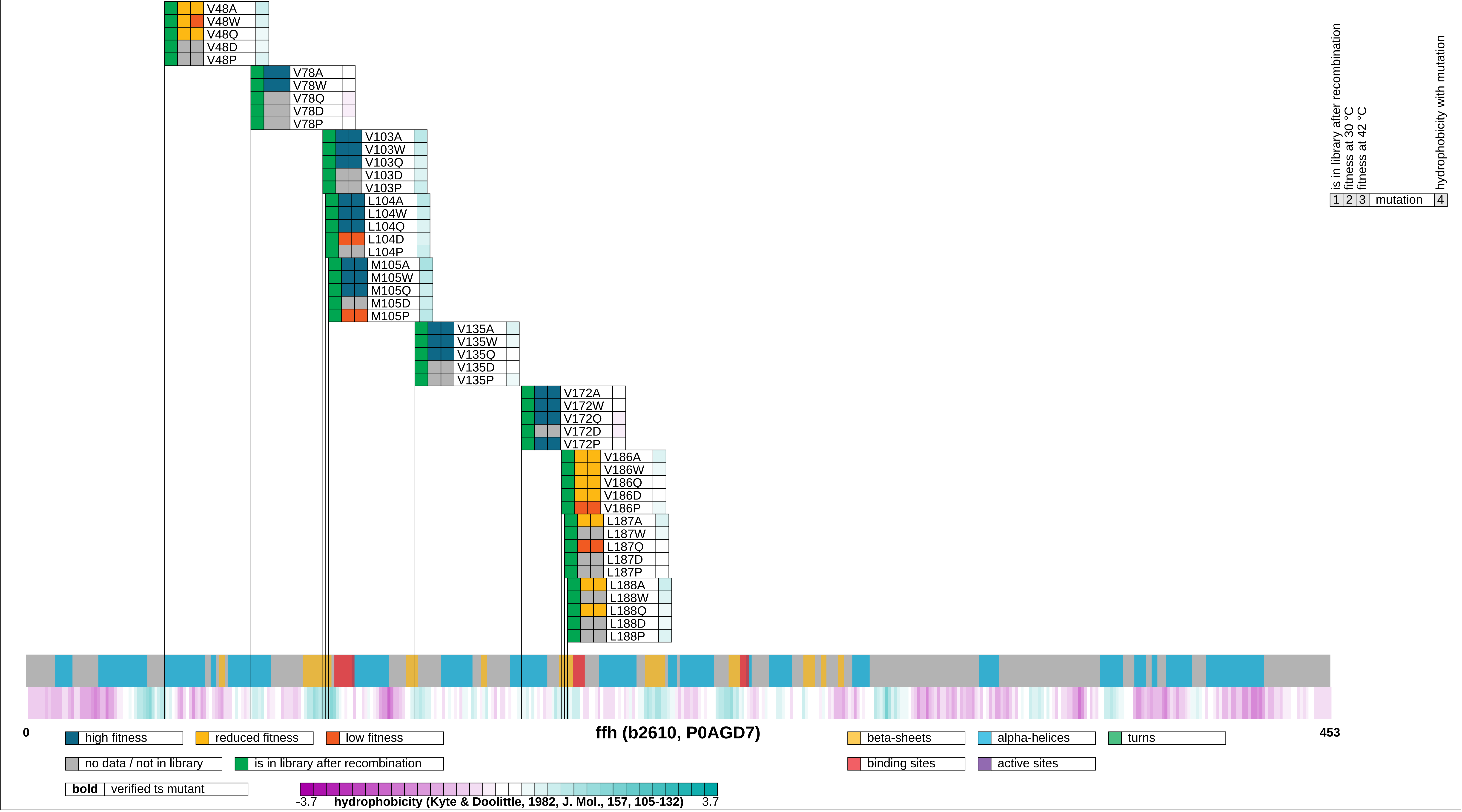
V48A
V48W
V48Q
V48D
V48P
V78A
V78W
V78Q
V78D
V78P
V103A
V103W
V103Q
V103D
V103P
L104A
L104W
L104Q
L104D
L104P
M105A
M105W
M105Q
M105D
M105P
V135A
V135W
V135Q
V135D
V135P
V172A
V172W
V172Q
V172D
V172P
V186A
V186W
V186Q
V186D
V186P
L187A
L187W
L187Q
L187D
L187P
L188A
L188W
L188Q
L188D
L188P
is in library after recombination
fitness at 30 °C
fitness at 42 °C
hydrophobicity with mutation
1 2 3 mutation 4
0
453
ffh (b2610, P0AGD7)
high fitness
reduced fitness
low fitness
no data / not in library
is in library after recombination
bold verified ts mutant
beta-sheets
alpha-helices
turns
binding sites
active sites
-3.7 hydrophobicity (Kyte & Doolittle, 1982, J. Mol., 157, 105-132) 3.7

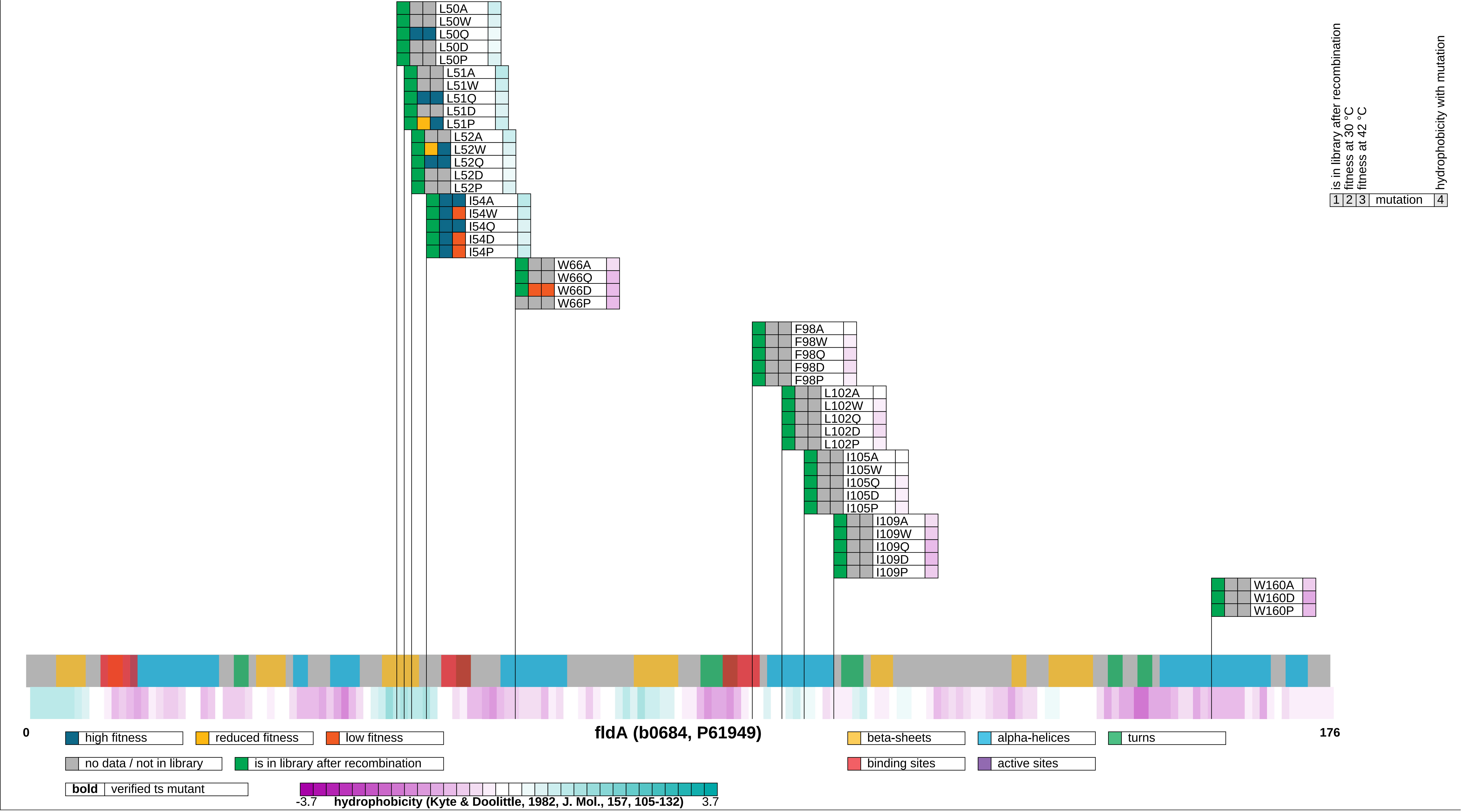

L50A
L50W
L50Q
L50D
L50P
L51A
L51W
L51Q
L51D
L51P
L52A
L52W
L52Q
L52D
L52P
I54A
I54W
I54Q
I54D
I54P
W66A
W66Q
W66D
W66P
F98A
F98W
F98Q
F98D
F98P
L102A
L102W
L102Q
L102D
L102P
I105A
I105W
I105Q
I105D
I105P
I109A
I109W
I109Q
I109D
I109P
W160A
W160D
W160P
is in library after recombination
fitness at 30 °C
fitness at 42 °C
hydrophobicity with mutation
1 2 3 mutation 4
0
176
fldA (b0684, P61949)
high fitness
reduced fitness
low fitness
no data / not in library
is in library after recombination
bold verified ts mutant
beta-sheets
alpha-helices
turns
binding sites
active sites
-3.7
hydrophobicity (Kyte & Doolittle, 1982, J. Mol., 157, 105-132)
3.7

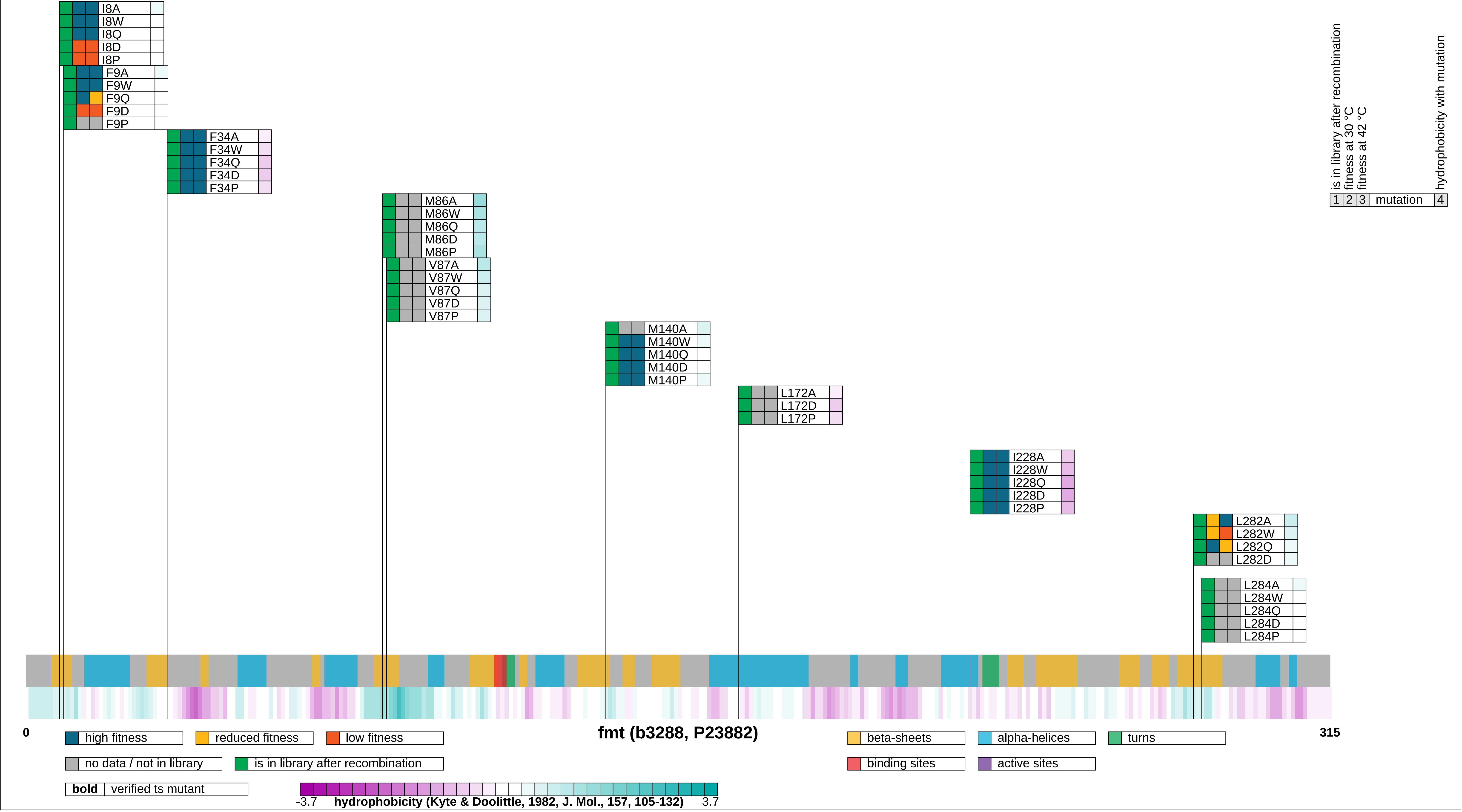
I8A
I8W
I8Q
I8D
I8P
F9A
F9W
F9Q
F9D
F9P
F34A
F34W
F34Q
F34D
F34P
M86A
M86W
M86Q
M86D
M86P
V87A
V87W
V87Q
V87D
V87P
M140A
M140W
M140Q
M140D
M140P
L172A
L172D
L172P
I228A
I228W
I228Q
I228D
I228P
L282A
L282W
L282Q
L282D
L284A
L284W
L284Q
L284D
L284P
is in library after recombination
fitness at 30 °C
fitness at 42 °C
hydrophobicity with mutation
1 2 3 mutation 4
0
fmt (b3288, P23882)
315
high fitness
reduced fitness
low fitness
beta-sheets
alpha-helices
turns
no data / not in library
is in library after recombination
binding sites
active sites
bold verified ts mutant
-3.7
hydrophobicity (Kyte & Doolittle, 1982, J. Mol., 157, 105-132)
3.7

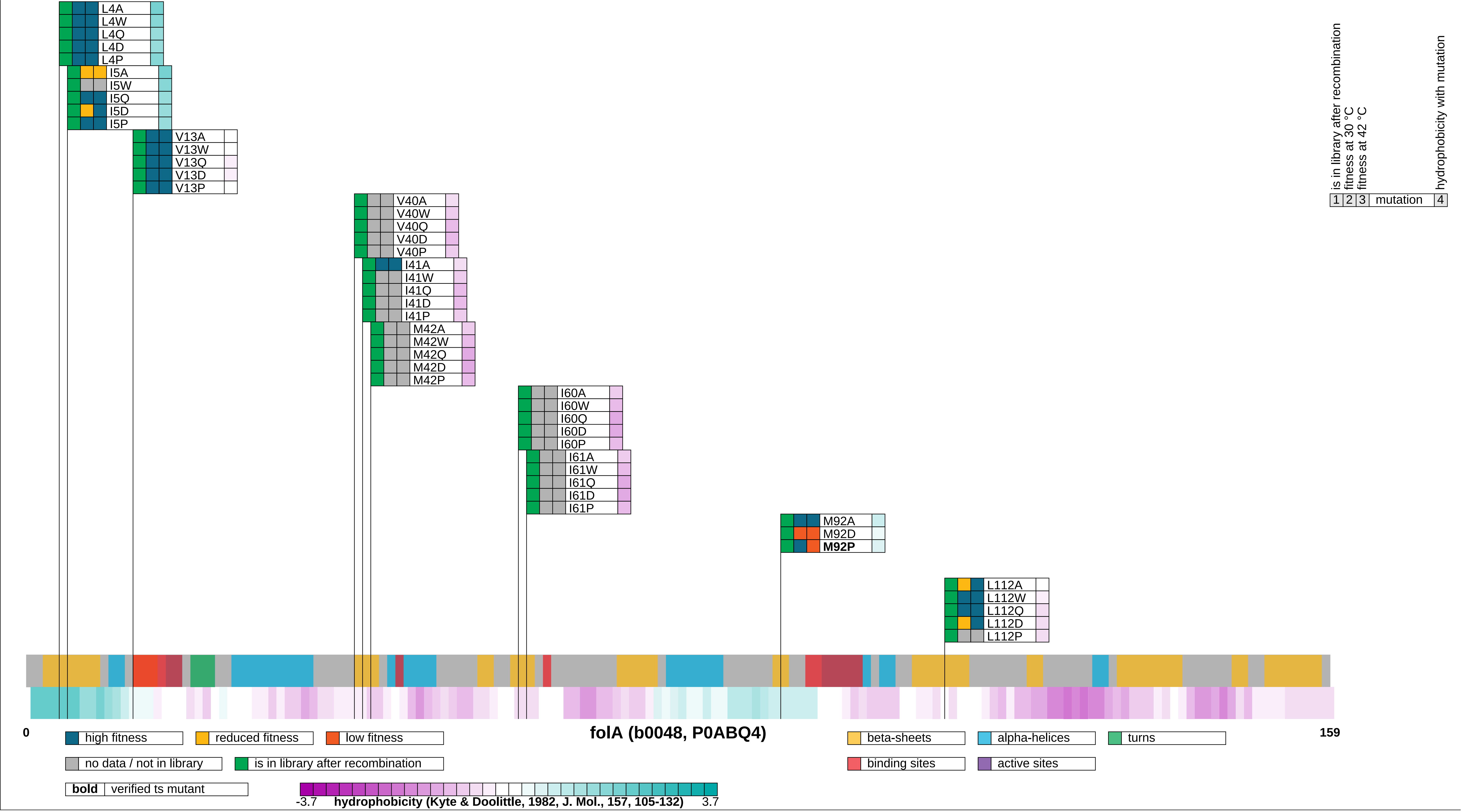

L4A
L4W
L4Q
L4D
L4P
I5A
I5W
I5Q
I5D
I5P
V13A
V13W
V13Q
V13D
V13P
V40A
V40W
V40Q
V40D
V40P
I41A
I41W
I41Q
I41D
I41P
M42A
M42W
M42Q
M42D
M42P
I60A
I60W
I60Q
I60D
I60P
I61A
I61W
I61Q
I61D
I61P
M92A
M92D
M92P
L112A
L112W
L112Q
L112D
L112P
is in library after recombination
fitness at 30 °C
fitness at 42 °C
hydrophobicity with mutation
1 2 3 mutation 4
0
folA (b0048, P0ABQ4)
159
high fitness
reduced fitness
low fitness
no data / not in library
is in library after recombination
bold verified ts mutant
beta-sheets
alpha-helices
turns
binding sites
active sites
-3.7
hydrophobicity (Kyte & Doolittle, 1982, J. Mol., 157, 105-132)
3.7

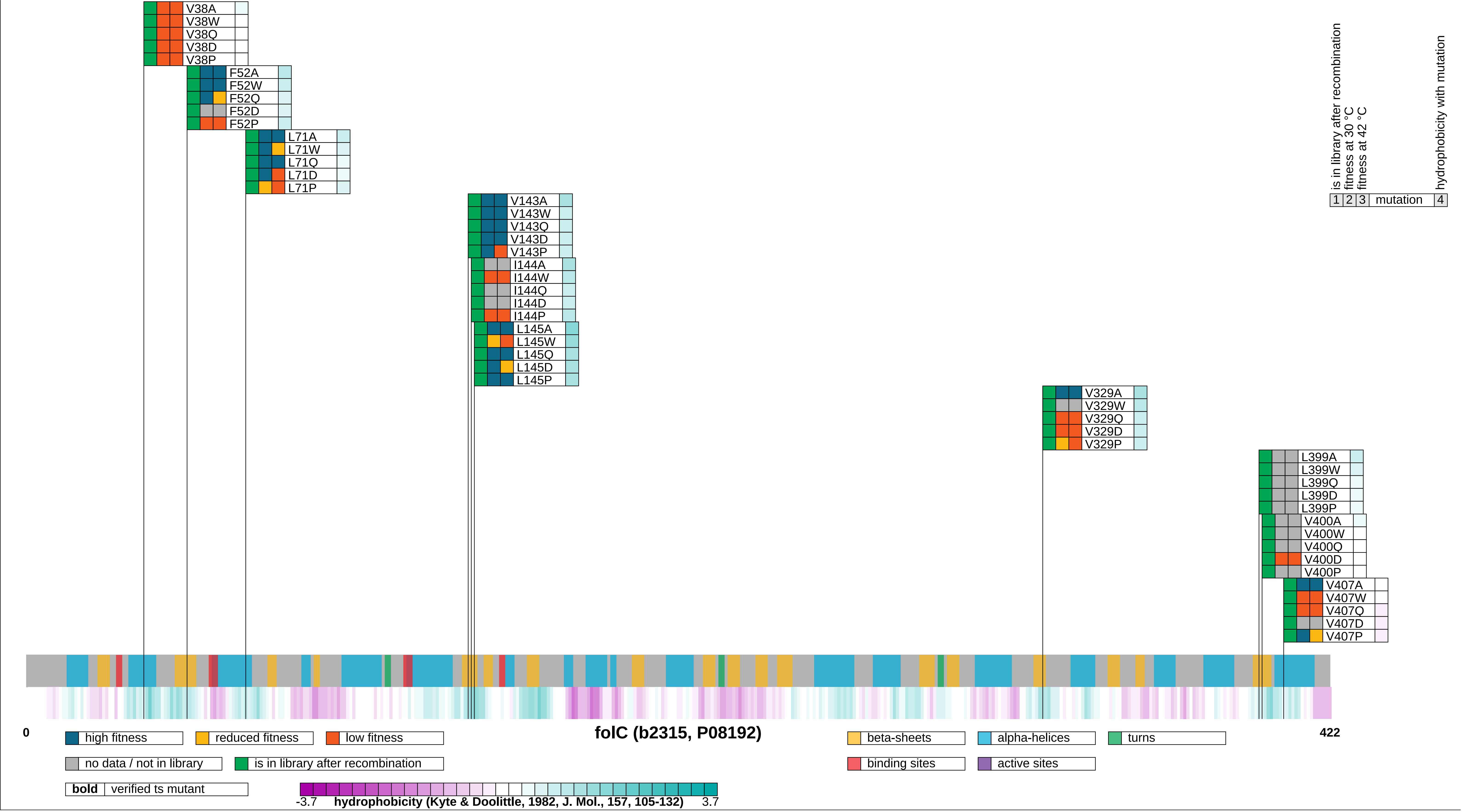

V38A
V38W
V38Q
V38D
V38P
F52A
F52W
F52Q
F52D
F52P
L71A
L71W
L71Q
L71D
L71P
V143A
V143W
V143Q
V143D
V143P
I144A
I144W
I144Q
I144D
I144P
L145A
L145W
L145Q
L145D
L145P
V329A
V329W
V329Q
V329D
V329P
L399A
L399W
L399Q
L399D
L399P
V400A
V400W
V400Q
V400D
V400P
V407A
V407W
V407Q
V407D
V407P
is in library after recombination
fitness at 30 °C
fitness at 42 °C
hydrophobicity with mutation
1 2 3 mutation 4
0
422
folC (b2315, P08192)
high fitness
reduced fitness
low fitness
no data / not in library
is in library after recombination
bold verified ts mutant
beta-sheets
alpha-helices
turns
binding sites
active sites
-3.7
hydrophobicity (Kyte & Doolittle, 1982, J. Mol., 157, 105-132)
3.7

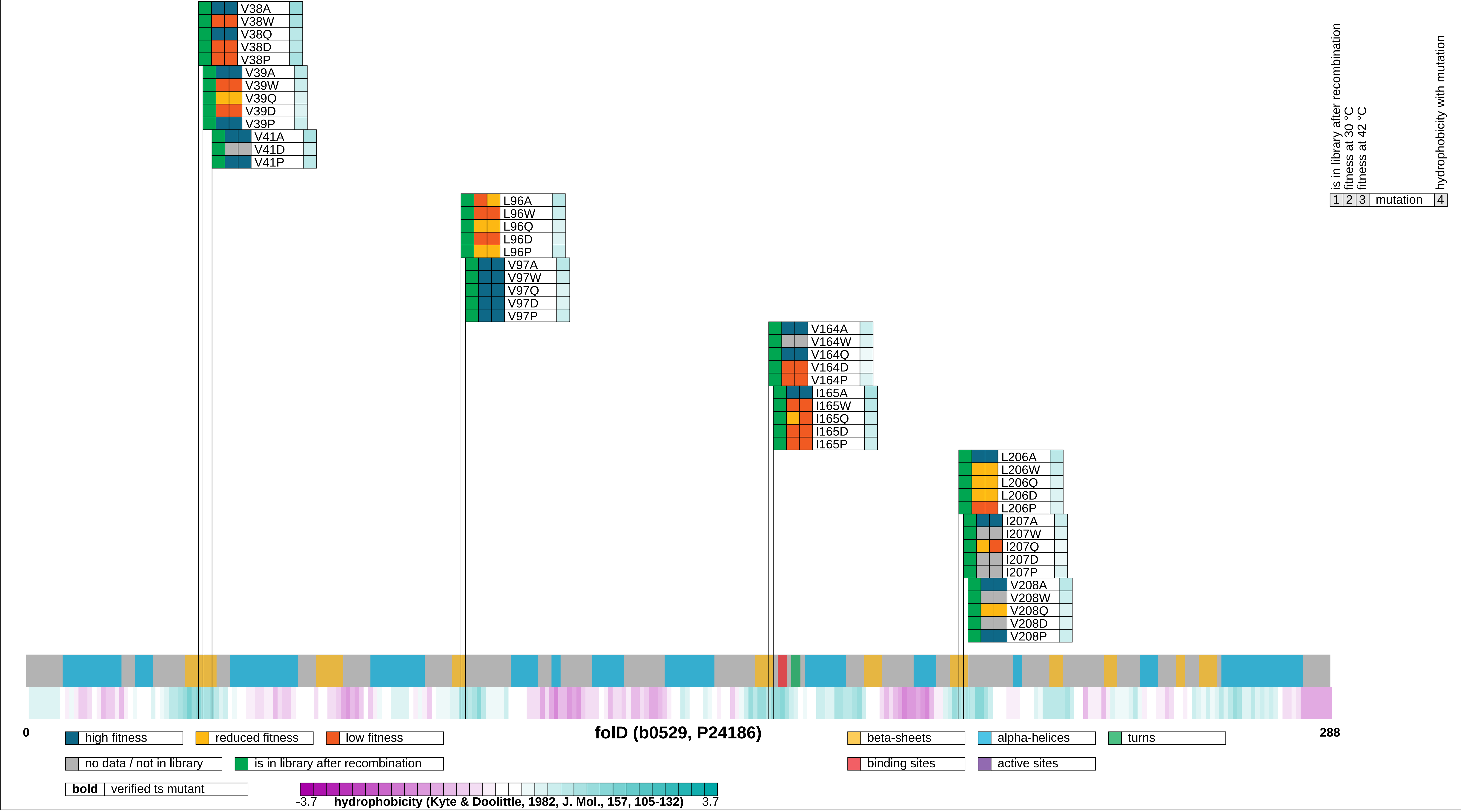
V38A
V38W
V38Q
V38D
V38P
V39A
V39W
V39Q
V39D
V39P
V41A
V41D
V41P
L96A
L96W
L96Q
L96D
L96P
V97A
V97W
V97Q
V97D
V97P
V164A
V164W
V164Q
V164D
V164P
I165A
I165W
I165Q
I165D
I165P
L206A
L206W
L206Q
L206D
L206P
I207A
I207W
I207Q
I207D
I207P
V208A
V208W
V208Q
V208D
V208P
is in library after recombination
fitness at 30 °C
fitness at 42 °C
hydrophobicity with mutation
1 2 3 mutation 4
0
folD (b0529, P24186)
288
high fitness
reduced fitness
low fitness
beta-sheets
alpha-helices
turns
no data / not in library
is in library after recombination
binding sites
active sites
bold verified ts mutant
-3.7
hydrophobicity (Kyte & Doolittle, 1982, J. Mol., 157, 105-132)
3.7

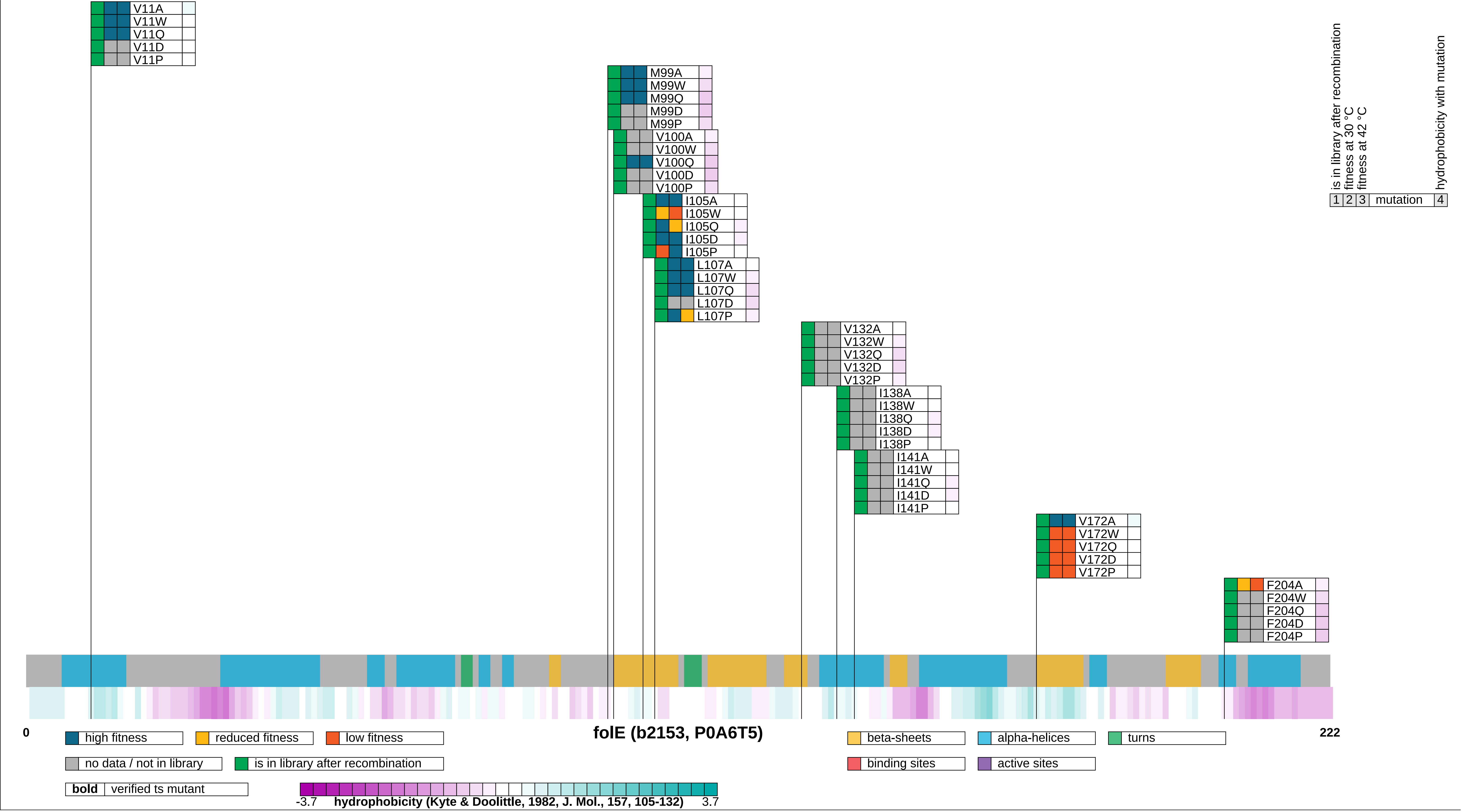

V11A
V11W
V11Q
V11D
V11P
M99A
M99W
M99Q
M99D
M99P
V100A
V100W
V100Q
V100D
V100P
I105A
I105W
I105Q
I105D
I105P
L107A
L107W
L107Q
L107D
L107P
V132A
V132W
V132Q
V132D
V132P
I138A
I138W
I138Q
I138D
I138P
I141A
I141W
I141Q
I141D
I141P
V172A
V172W
V172Q
V172D
V172P
F204A
F204W
F204Q
F204D
F204P
is in library after recombination
fitness at 30 °C
fitness at 42 °C
hydrophobicity with mutation
1
2
3
mutation
4
0
folE (b2153, P0A6T5)
222
high fitness
reduced fitness
low fitness
beta-sheets
alpha-helices
turns
no data / not in library
is in library after recombination
binding sites
active sites
bold
verified ts mutant
-3.7
hydrophobicity (Kyte & Doolittle, 1982, J. Mol., 157, 105-132)
3.7

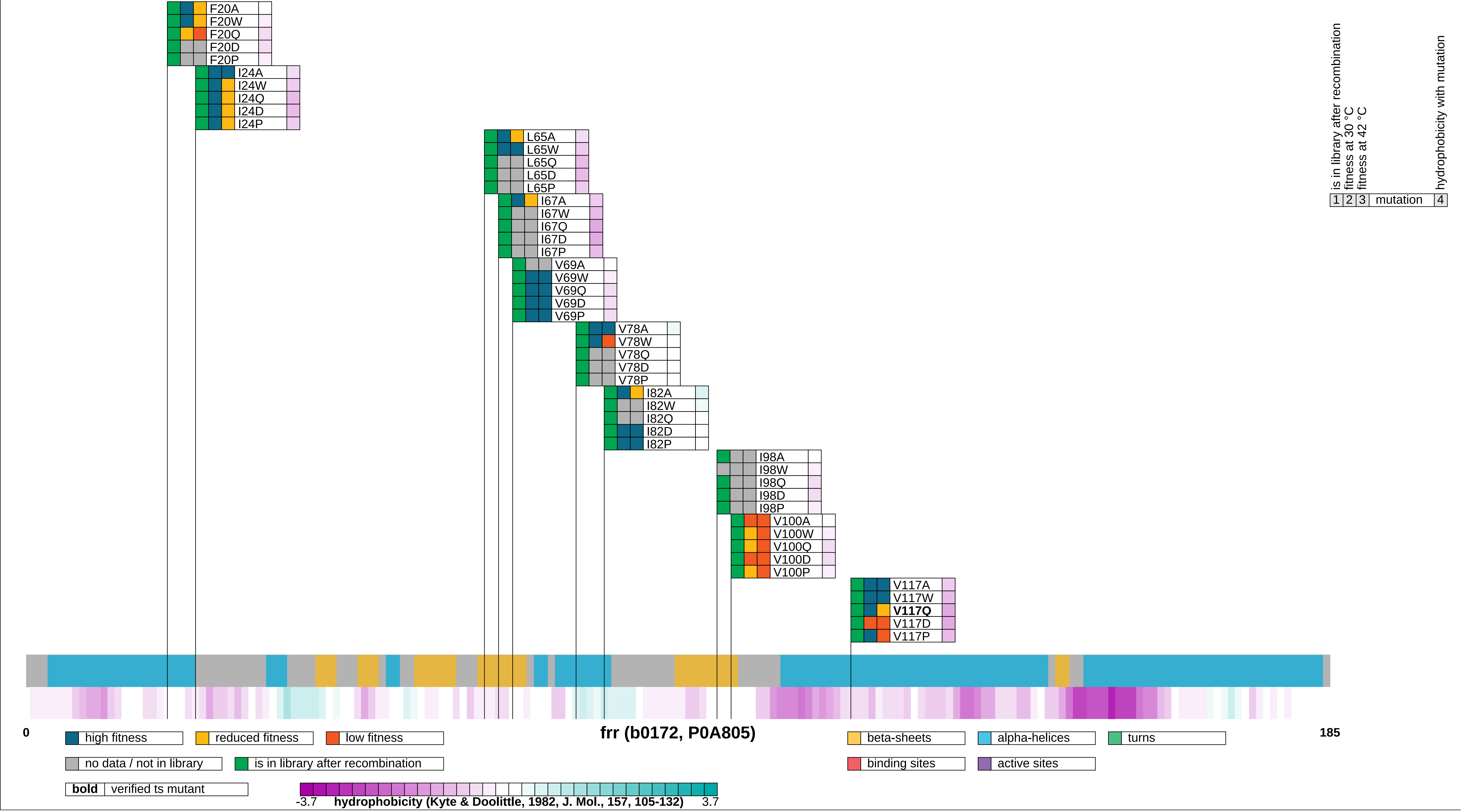
F20A
F20W
F20Q
F20D
F20P
I24A
I24W
I24Q
I24D
I24P
L65A
L65W
L65Q
L65D
L65P
I67A
I67W
I67Q
I67D
I67P
V69A
V69W
V69Q
V69D
V69P
V78A
V78W
V78Q
V78D
V78P
I82A
I82W
I82Q
I82D
I82P
I98A
I98W
I98Q
I98D
I98P
V100A
V100W
V100Q
V100D
V100P
V117A
V117W
V117Q
V117D
V117P
is in library after recombination
fitness at 30 °C
fitness at 42 °C
hydrophobicity with mutation
1 2 3 mutation 4
0
frr (b0172, P0A805)
185
high fitness
reduced fitness
low fitness
no data / not in library
is in library after recombination
bold verified ts mutant
beta-sheets
alpha-helices
turns
binding sites
active sites
-3.7
hydrophobicity (Kyte & Doolittle, 1982, J. Mol., 157, 105-132)
3.7

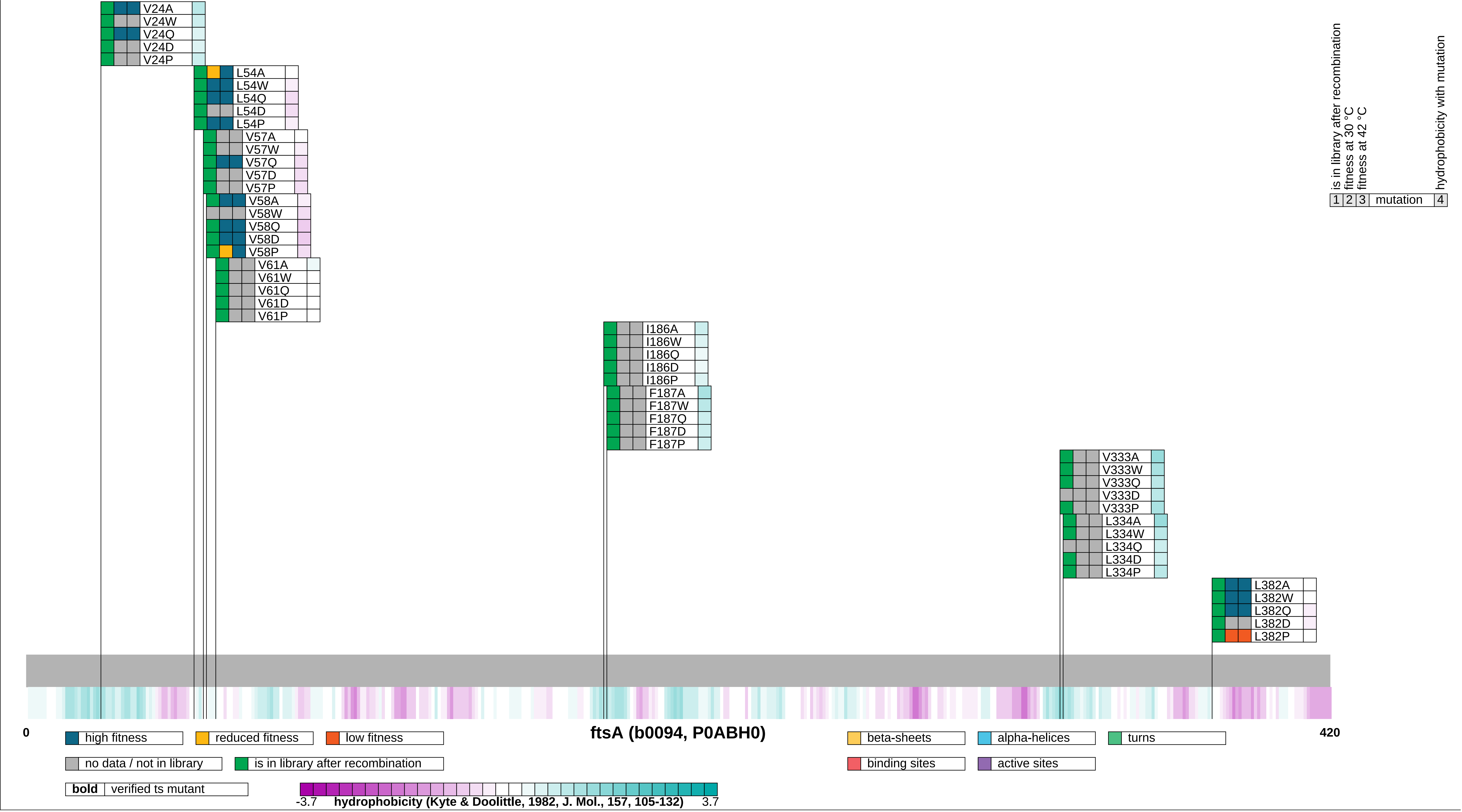

V24A
V24W
V24Q
V24D
V24P
L54A
L54W
L54Q
L54D
L54P
V57A
V57W
V57Q
V57D
V57P
V58A
V58W
V58Q
V58D
V58P
V61A
V61W
V61Q
V61D
V61P
I186A
I186W
I186Q
I186D
I186P
F187A
F187W
F187Q
F187D
F187P
V333A
V333W
V333Q
V333D
V333P
L334A
L334W
L334Q
L334D
L334P
L382A
L382W
L382Q
L382D
L382P
1 is in library after recombination
2 fitness at 30 °C
3 fitness at 42 °C
mutation
4 hydrophobicity with mutation
0
420
ftsA (b0094, P0ABH0)
high fitness
reduced fitness
low fitness
no data / not in library
is in library after recombination
bold verified ts mutant
beta-sheets
alpha-helices
turns
binding sites
active sites
-3.7
hydrophobicity (Kyte & Doolittle, 1982, J. Mol., 157, 105-132)
3.7

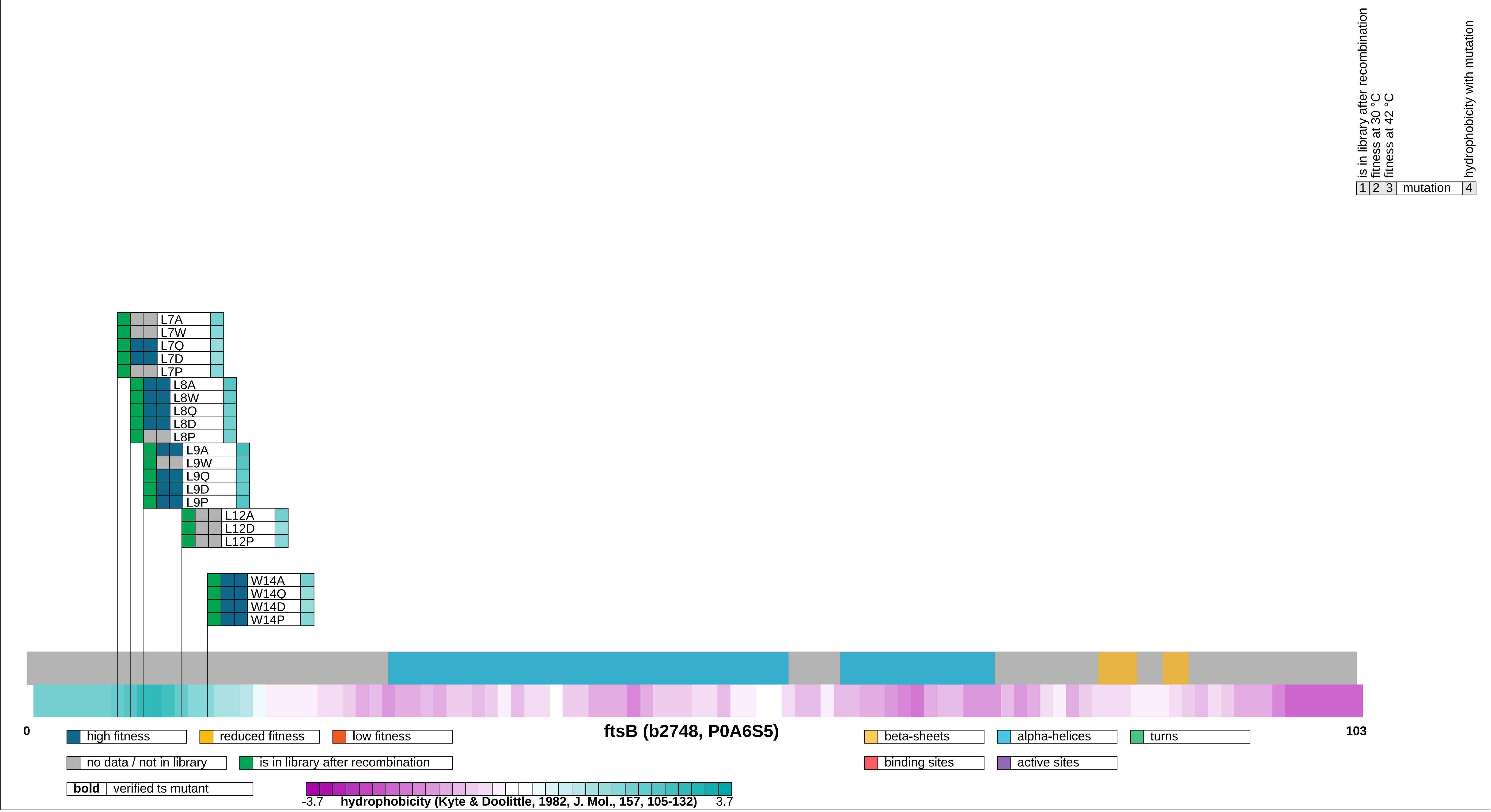

is in library after recombination
fitness at 30 °C
fitness at 42 °C
hydrophobicity with mutation
1 2 3 mutation 4
L7A
L7W
L7Q
L7D
L7P
L8A
L8W
L8Q
L8D
L8P
L9A
L9W
L9Q
L9D
L9P
L12A
L12D
L12P
W14A
W14Q
W14D
W14P
0
ftsB (b2748, P0A6S5)
103
high fitness
reduced fitness
low fitness
beta-sheets
alpha-helices
turns
no data / not in library
is in library after recombination
binding sites
active sites
bold verified ts mutant
-3.7
hydrophobicity (Kyte & Doolittle, 1982, J. Mol., 157, 105-132)
3.7

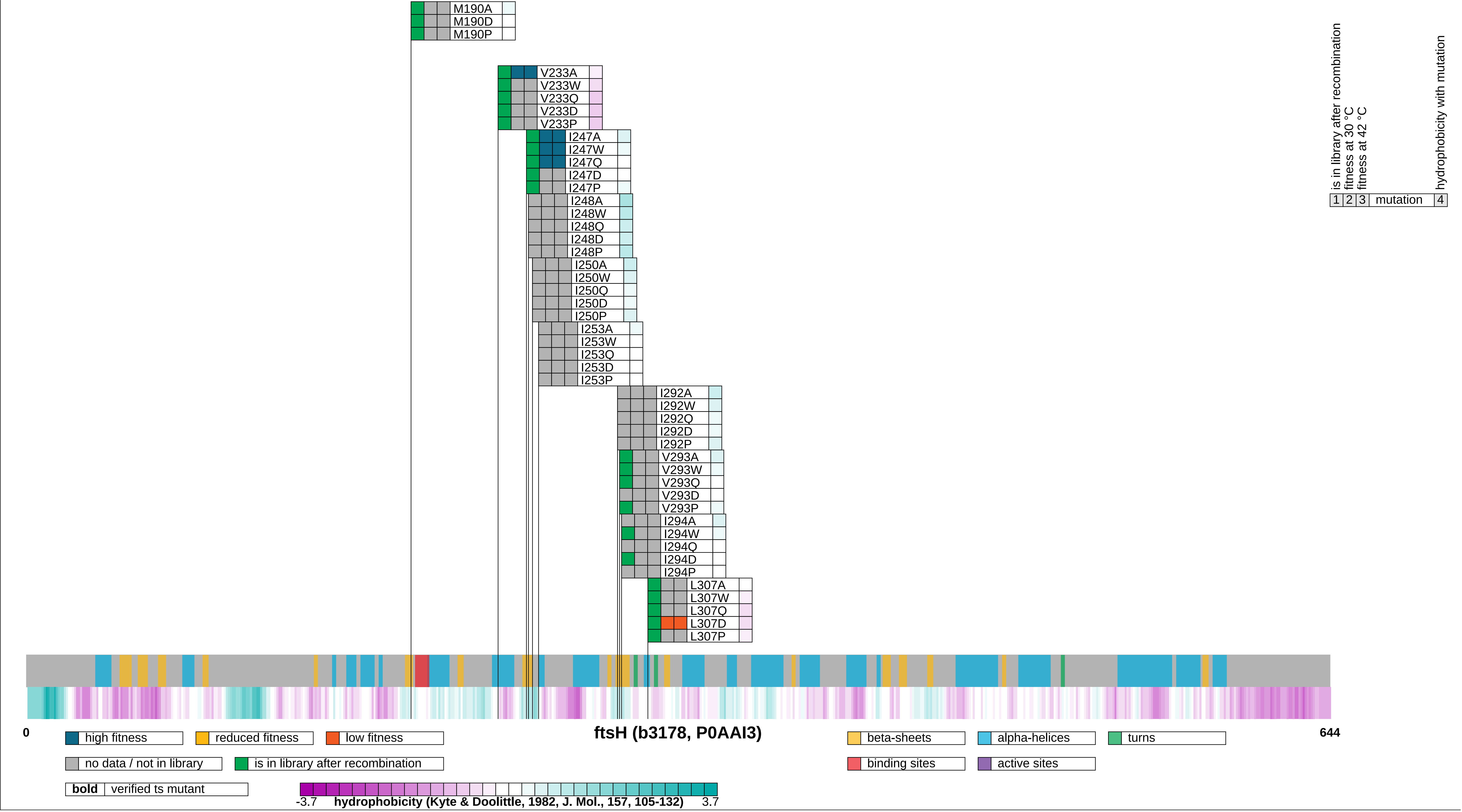

M190A
M190D
M190P
V233A
V233W
V233Q
V233D
V233P
I247A
I247W
I247Q
I247D
I247P
I248A
I248W
I248Q
I248D
I248P
I250A
I250W
I250Q
I250D
I250P
I253A
I253W
I253Q
I253D
I253P
I292A
I292W
I292Q
I292D
I292P
V293A
V293W
V293Q
V293D
V293P
I294A
I294W
I294Q
I294D
I294P
L307A
L307W
L307Q
L307D
L307P
is in library after recombination
fitness at 30 °C
fitness at 42 °C
hydrophobicity with mutation
1 2 3 mutation 4
0
ftsH (b3178, P0AAI3)
644
high fitness
reduced fitness
low fitness
beta-sheets
alpha-helices
turns
no data / not in library
is in library after recombination
binding sites
active sites
bold verified ts mutant
-3.7 hydrophobicity (Kyte & Doolittle, 1982, J. Mol., 157, 105-132) 3.7

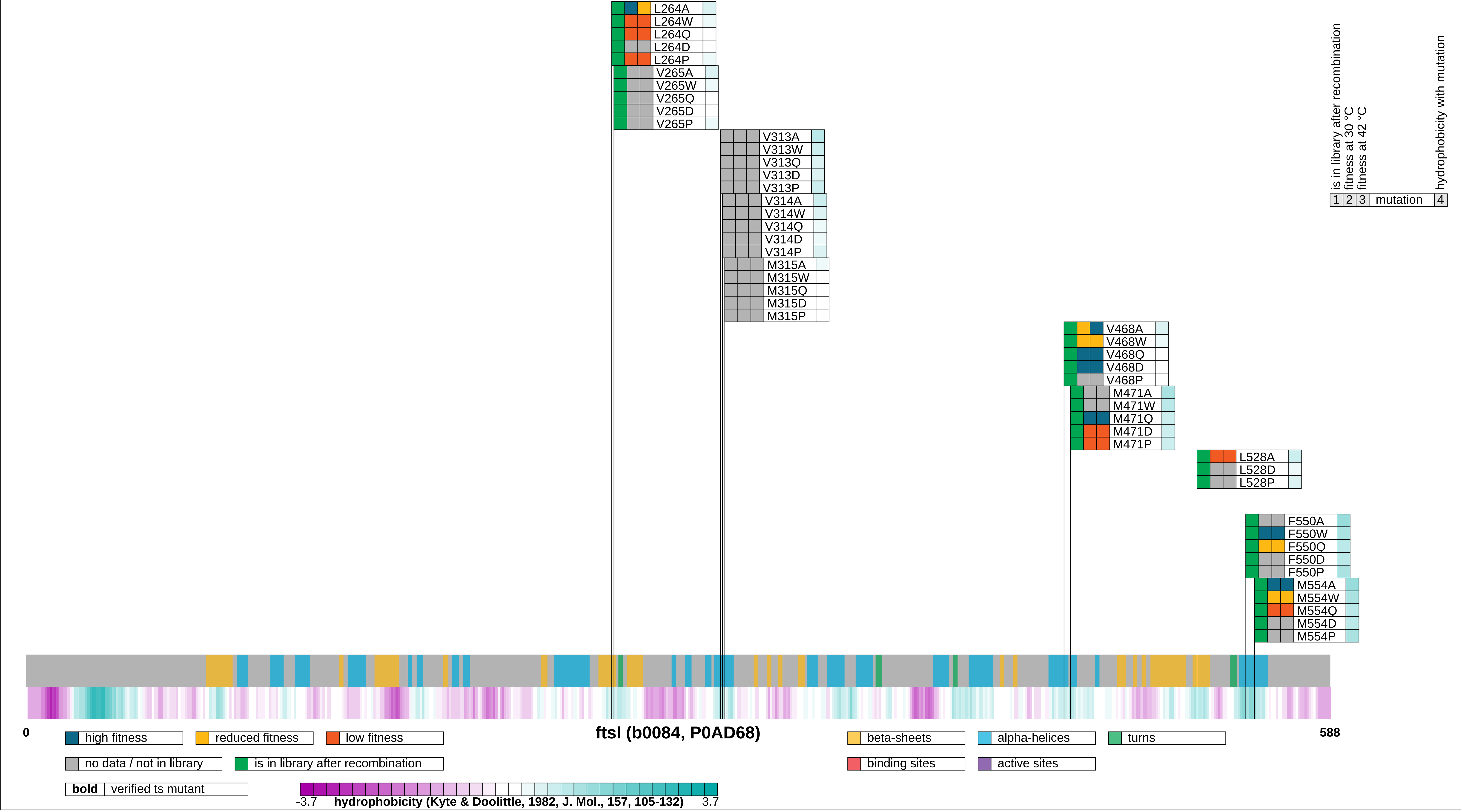

L264A
L264W
L264Q
L264D
L264P
V265A
V265W
V265Q
V265D
V265P
V313A
V313W
V313Q
V313D
V313P
V314A
V314W
V314Q
V314D
V314P
M315A
M315W
M315Q
M315D
M315P
V468A
V468W
V468Q
V468D
V468P
M471A
M471W
M471Q
M471D
M471P
L528A
L528D
L528P
F550A
F550W
F550Q
F550D
F550P
M554A
M554W
M554Q
M554D
M554P
is in library after recombination
fitness at 30 °C
fitness at 42 °C
hydrophobicity with mutation
1 2 3 mutation 4
0
ftsI (b0084, P0AD68)
588
high fitness
reduced fitness
low fitness
no data / not in library
is in library after recombination
bold verified ts mutant
beta-sheets
alpha-helices
turns
binding sites
active sites
-3.7 hydrophobicity (Kyte & Doolittle, 1982, J. Mol., 157, 105-132) 3.7

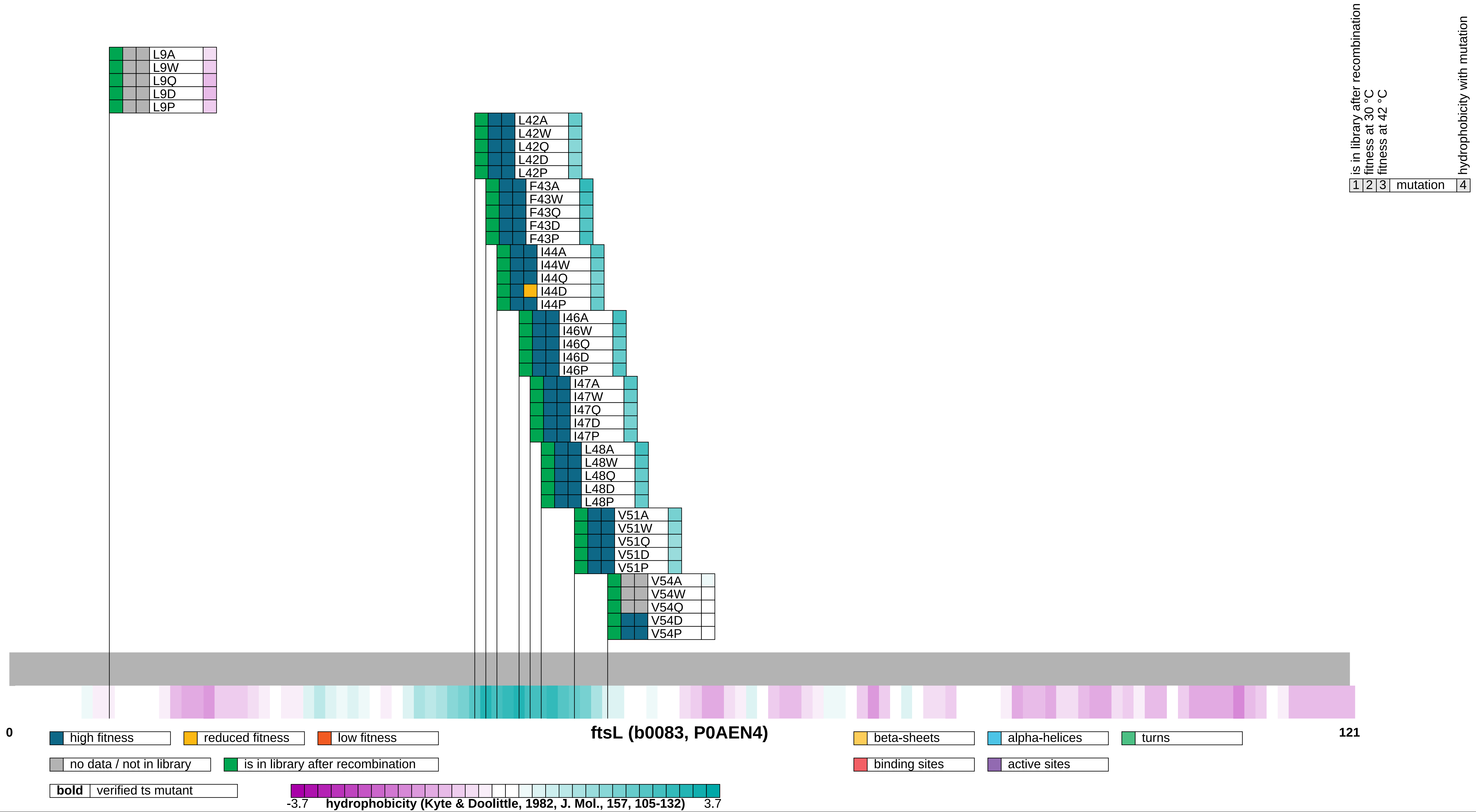

is in library after recombination
fitness at 30 °C
fitness at 42 °C
hydrophobicity with mutation
1
2
3
mutation
4
L9A
L9W
L9Q
L9D
L9P
L42A
L42W
L42Q
L42D
L42P
F43A
F43W
F43Q
F43D
F43P
I44A
I44W
I44Q
I44D
I44P
I46A
I46W
I46Q
I46D
I46P
I47A
I47W
I47Q
I47D
I47P
L48A
L48W
L48Q
L48D
L48P
V51A
V51W
V51Q
V51D
V51P
V54A
V54W
V54Q
V54D
V54P
0
ftsL (b0083, P0AEN4)
121
high fitness
reduced fitness
low fitness
beta-sheets
alpha-helices
turns
no data / not in library
is in library after recombination
binding sites
active sites
bold
verified ts mutant
-3.7
hydrophobicity (Kyte & Doolittle, 1982, J. Mol., 157, 105-132)
3.7

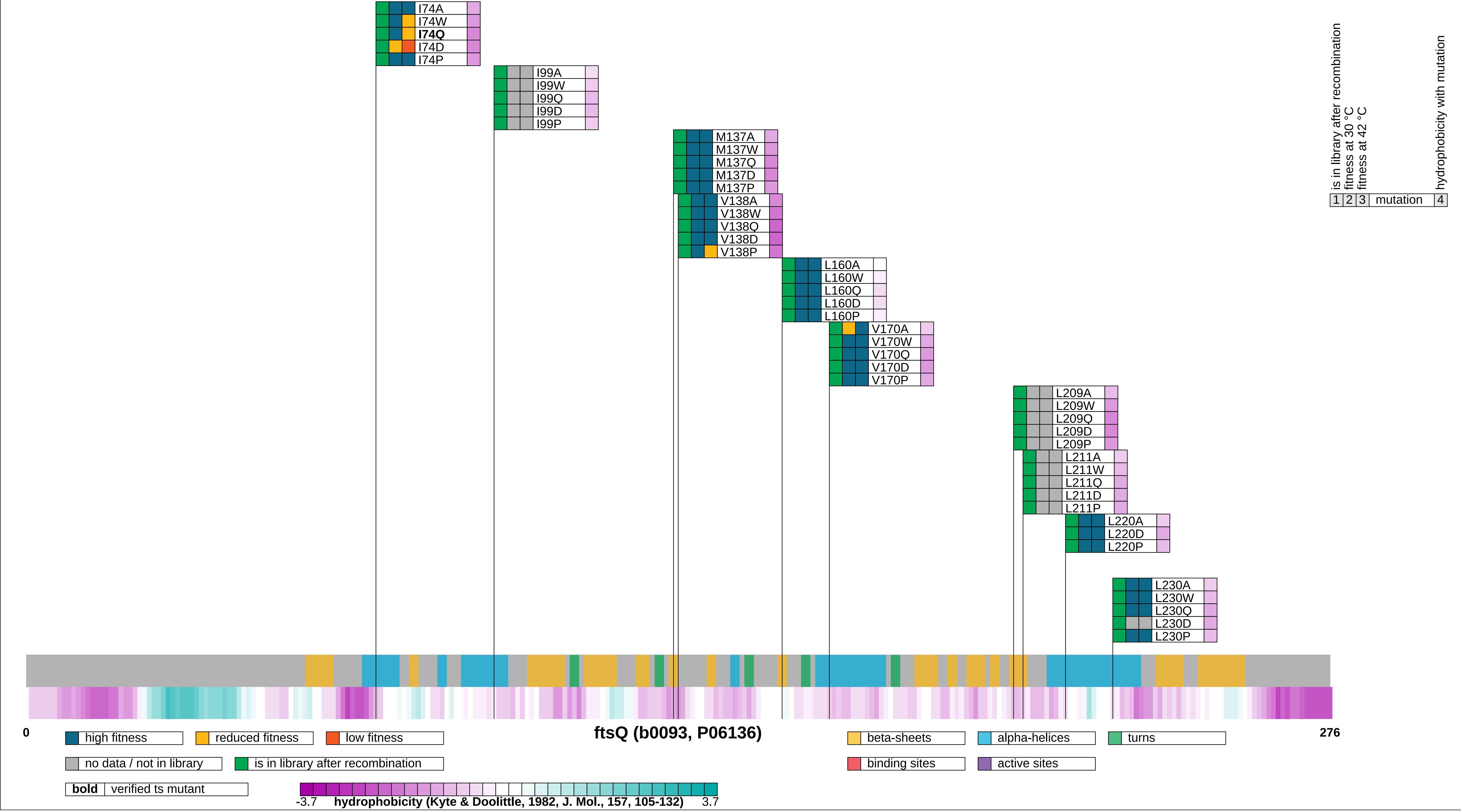
I74A
I74W
I74Q
I74D
I74P
I99A
I99W
I99Q
I99D
I99P
M137A
M137W
M137Q
M137D
M137P
V138A
V138W
V138Q
V138D
V138P
L160A
L160W
L160Q
L160D
L160P
V170A
V170W
V170Q
V170D
V170P
L209A
L209W
L209Q
L209D
L209P
L211A
L211W
L211Q
L211D
L211P
L220A
L220D
L220P
L230A
L230W
L230Q
L230D
L230P
is in library after recombination
fitness at 30 °C
fitness at 42 °C
hydrophobicity with mutation
1 2 3 mutation 4
0
ftsQ (b0093, P06136)
276
high fitness
reduced fitness
low fitness
beta-sheets
alpha-helices
turns
no data / not in library
is in library after recombination
binding sites
active sites
bold verified ts mutant
-3.7
hydrophobicity (Kyte & Doolittle, 1982, J. Mol., 157, 105-132)
3.7

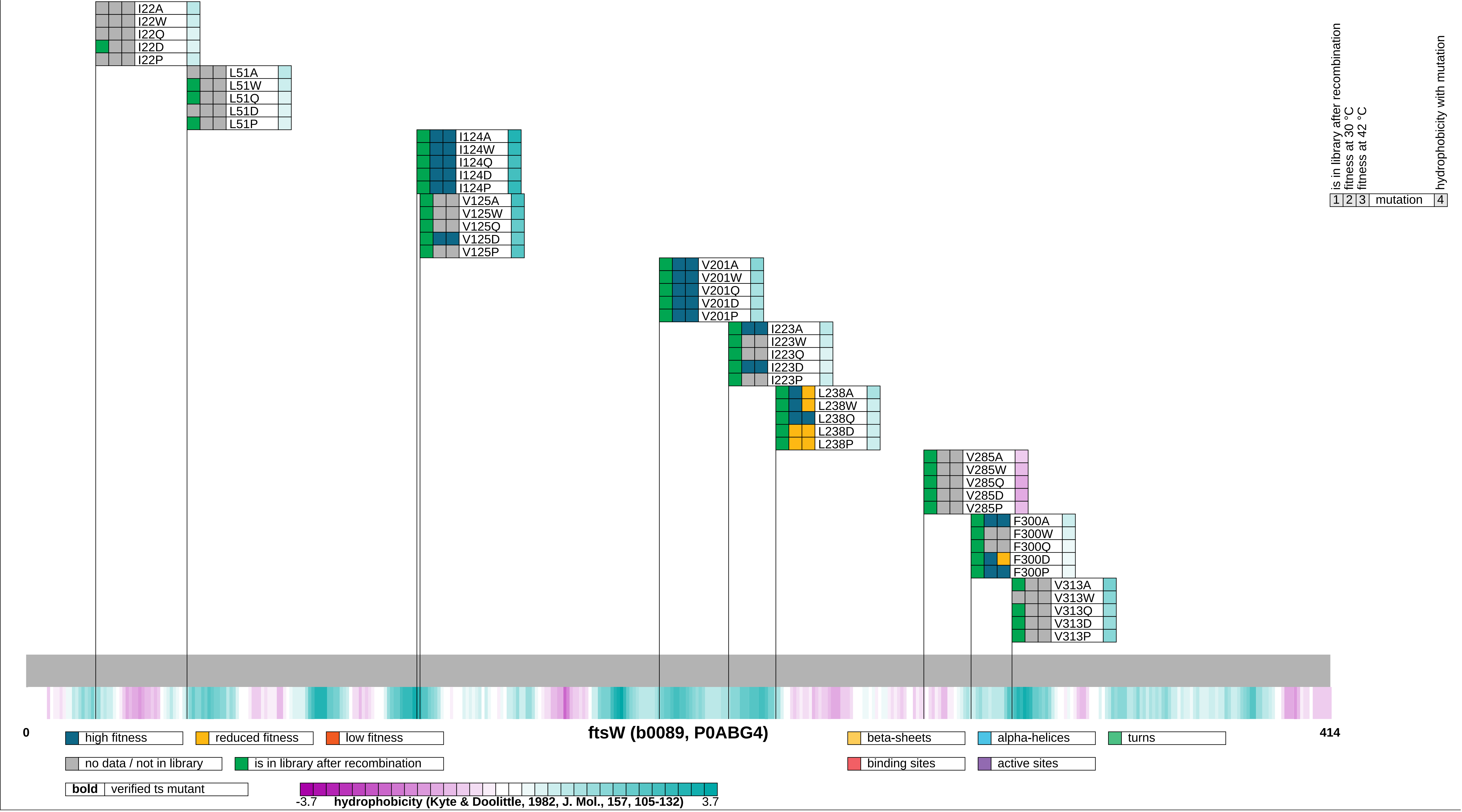
I22A
I22W
I22Q
I22D
I22P
L51A
L51W
L51Q
L51D
L51P
I124A
I124W
I124Q
I124D
I124P
V125A
V125W
V125Q
V125D
V125P
V201A
V201W
V201Q
V201D
V201P
I223A
I223W
I223Q
I223D
I223P
L238A
L238W
L238Q
L238D
L238P
V285A
V285W
V285Q
V285D
V285P
F300A
F300W
F300Q
F300D
F300P
V313A
V313W
V313Q
V313D
V313P
is in library after recombination
fitness at 30 °C
fitness at 42 °C
hydrophobicity with mutation
1 2 3 mutation 4
0
ftsW (b0089, P0ABG4)
414
high fitness
reduced fitness
low fitness
beta-sheets
alpha-helices
turns
no data / not in library
is in library after recombination
binding sites
active sites
bold verified ts mutant
-3.7 hydrophobicity (Kyte & Doolittle, 1982, J. Mol., 157, 105-132) 3.7

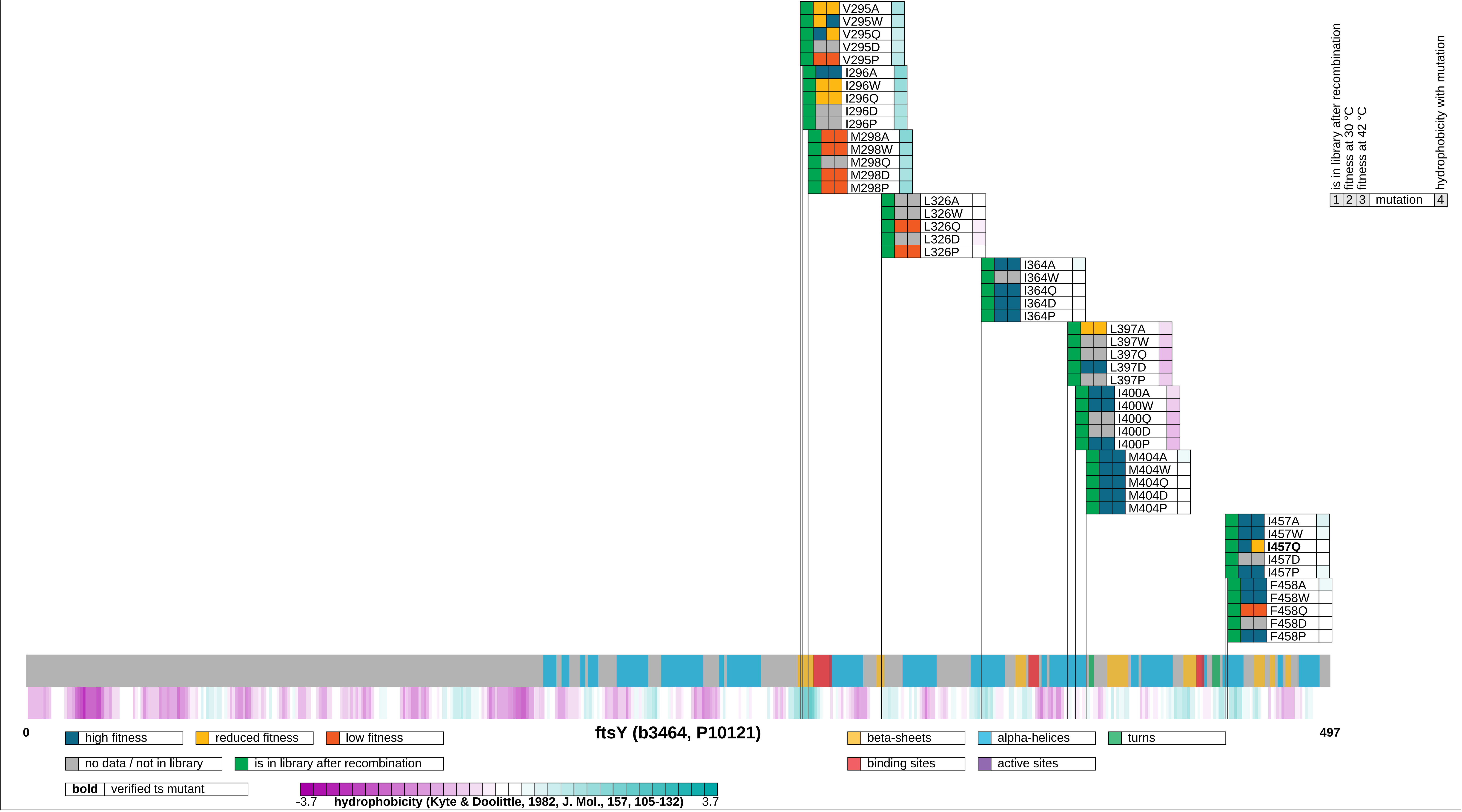

is in library after recombination
fitness at 30 °C
fitness at 42 °C
hydrophobicity with mutation
1 2 3 mutation 4
V295A
V295W
V295Q
V295D
V295P
I296A
I296W
I296Q
I296D
I296P
M298A
M298W
M298Q
M298D
M298P
L326A
L326W
L326Q
L326D
L326P
I364A
I364W
I364Q
I364D
I364P
L397A
L397W
L397Q
L397D
L397P
I400A
I400W
I400Q
I400D
I400P
M404A
M404W
M404Q
M404D
M404P
I457A
I457W
I457Q
I457D
I457P
F458A
F458W
F458Q
F458D
F458P
0
ftsY (b3464, P10121)
497
high fitness
reduced fitness
low fitness
beta-sheets
alpha-helices
turns
no data / not in library
is in library after recombination
binding sites
active sites
bold verified ts mutant
-3.7
hydrophobicity (Kyte & Doolittle, 1982, J. Mol., 157, 105-132)
3.7

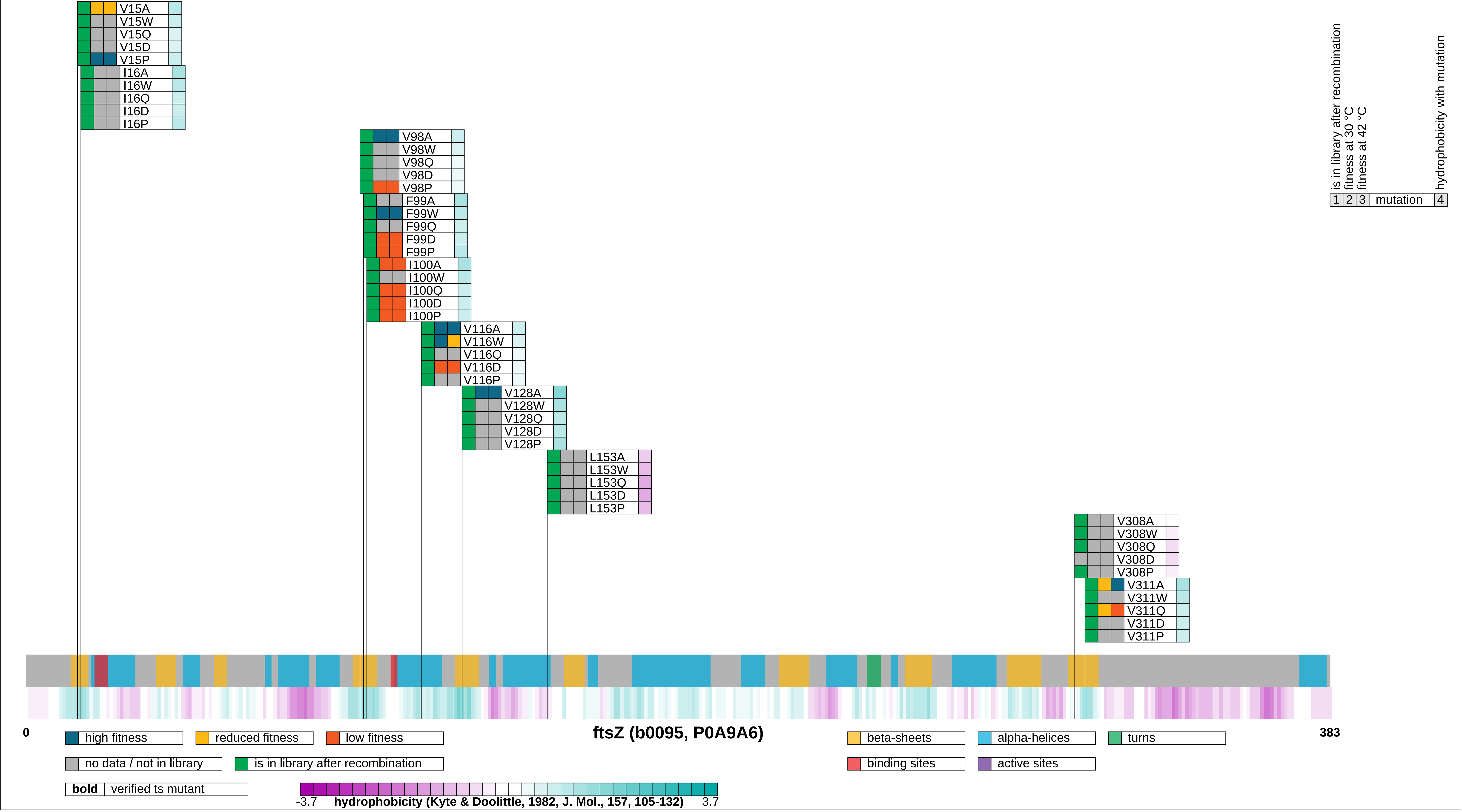

V15A
V15W
V15Q
V15D
V15P
I16A
I16W
I16Q
I16D
I16P
V98A
V98W
V98Q
V98D
V98P
F99A
F99W
F99Q
F99D
F99P
I100A
I100W
I100Q
I100D
I100P
V116A
V116W
V116Q
V116D
V116P
V128A
V128W
V128Q
V128D
V128P
L153A
L153W
L153Q
L153D
L153P
V308A
V308W
V308Q
V308D
V308P
V311A
V311W
V311Q
V311D
V311P
is in library after recombination
fitness at 30 °C
fitness at 42 °C
hydrophobicity with mutation
1 2 3 mutation 4
0
ftsZ (b0095, P0A9A6)
383
high fitness
reduced fitness
low fitness
no data / not in library
is in library after recombination
bold verified ts mutant
beta-sheets
alpha-helices
turns
binding sites
active sites
-3.7 hydrophobicity (Kyte & Doolittle, 1982, J. Mol., 157, 105-132) 3.7

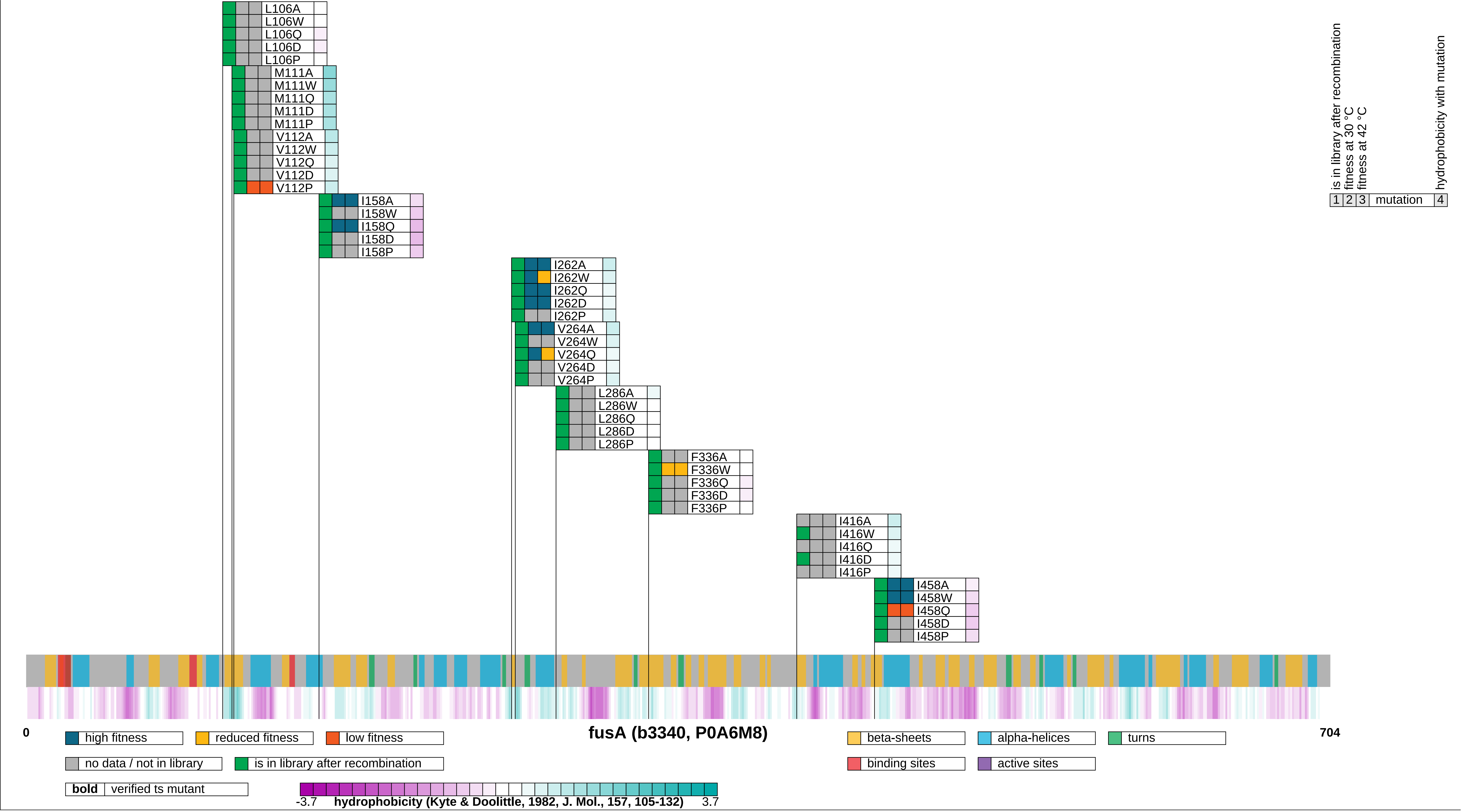

is in library after recombination
fitness at 30 °C
fitness at 42 °C
hydrophobicity with mutation
1 2 3 mutation 4
L106A
L106W
L106Q
L106D
L106P
M111A
M111W
M111Q
M111D
M111P
V112A
V112W
V112Q
V112D
V112P
I158A
I158W
I158Q
I158D
I158P
I262A
I262W
I262Q
I262D
I262P
V264A
V264W
V264Q
V264D
V264P
L286A
L286W
L286Q
L286D
L286P
F336A
F336W
F336Q
F336D
F336P
I416A
I416W
I416Q
I416D
I416P
I458A
I458W
I458Q
I458D
I458P
0
fusA (b3340, P0A6M8)
704
high fitness
reduced fitness
low fitness
no data / not in library
is in library after recombination
bold verified ts mutant
beta-sheets
alpha-helices
turns
binding sites
active sites
-3.7 hydrophobicity (Kyte & Doolittle, 1982, J. Mol., 157, 105-132) 3.7

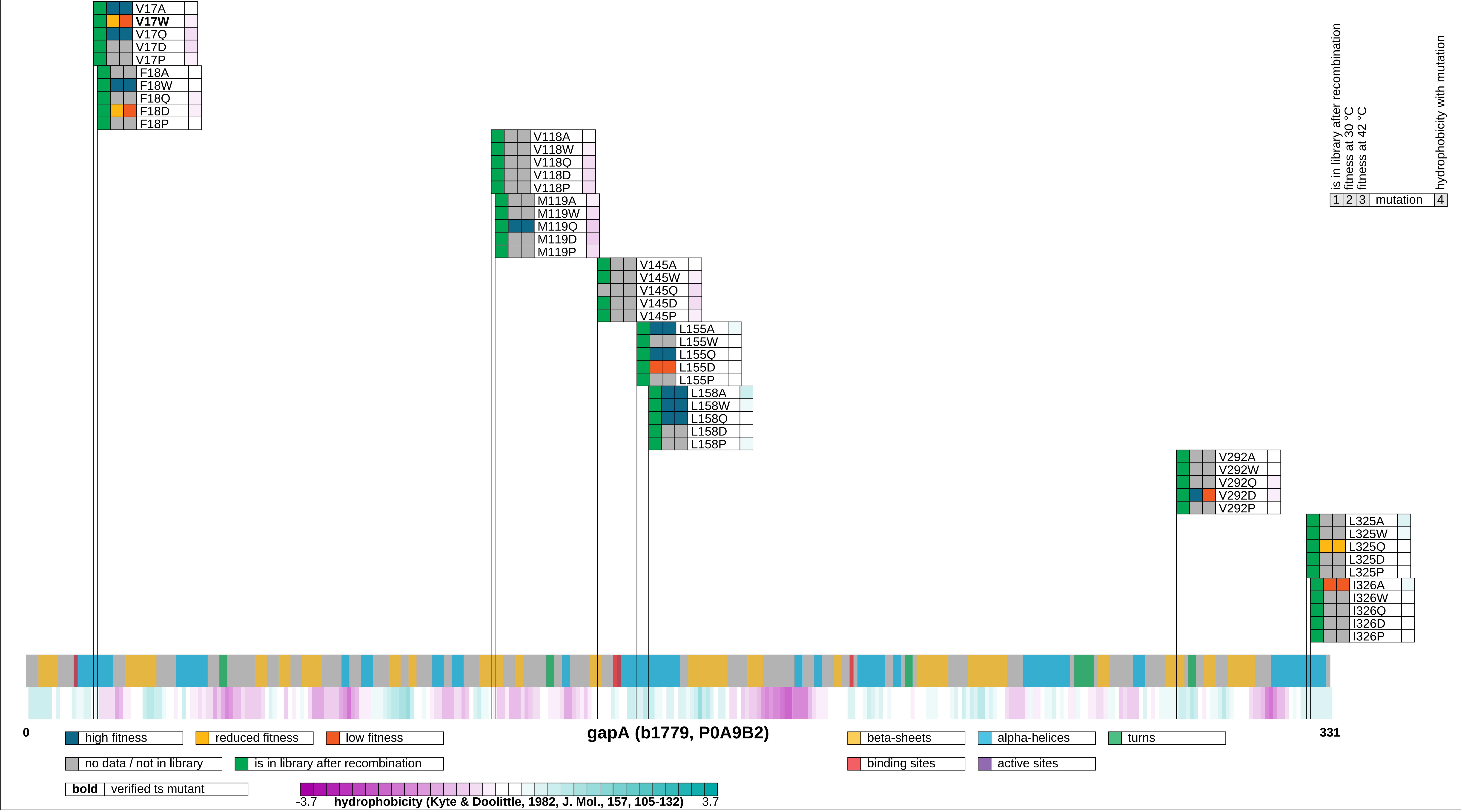

V17A
V17W
V17Q
V17D
V17P
F18A
F18W
F18Q
F18D
F18P
V118A
V118W
V118Q
V118D
V118P
M119A
M119W
M119Q
M119D
M119P
V145A
V145W
V145Q
V145D
V145P
L155A
L155W
L155Q
L155D
L155P
L158A
L158W
L158Q
L158D
L158P
V292A
V292W
V292Q
V292D
V292P
L325A
L325W
L325Q
L325D
L325P
I326A
I326W
I326Q
I326D
I326P
is in library after recombination
fitness at 30 °C
fitness at 42 °C
hydrophobicity with mutation
1 2 3 mutation 4
0
331
gapA (b1779, P0A9B2)
high fitness
reduced fitness
low fitness
no data / not in library
is in library after recombination
bold verified ts mutant
beta-sheets
alpha-helices
turns
binding sites
active sites
-3.7
hydrophobicity (Kyte & Doolittle, 1982, J. Mol., 157, 105-132)
3.7

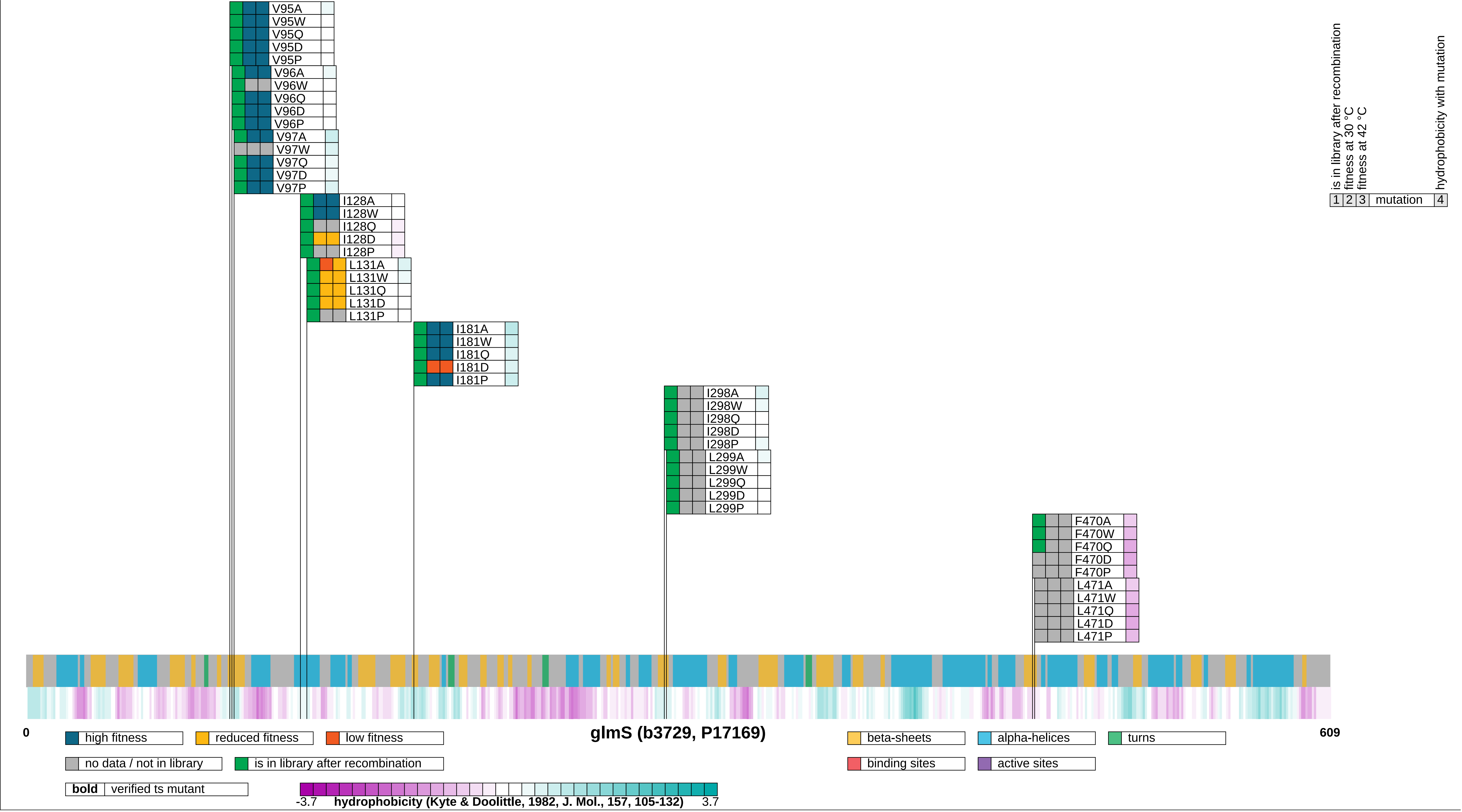

V95A
V95W
V95Q
V95D
V95P
V96A
V96W
V96Q
V96D
V96P
V97A
V97W
V97Q
V97D
V97P
I128A
I128W
I128Q
I128D
I128P
L131A
L131W
L131Q
L131D
L131P
I181A
I181W
I181Q
I181D
I181P
I298A
I298W
I298Q
I298D
I298P
L299A
L299W
L299Q
L299D
L299P
F470A
F470W
F470Q
F470D
F470P
L471A
L471W
L471Q
L471D
L471P
is in library after recombination
fitness at 30 °C
fitness at 42 °C
hydrophobicity with mutation
1 2 3 mutation 4
0
glmS (b3729, P17169)
609
high fitness
reduced fitness
low fitness
no data / not in library
is in library after recombination
bold verified ts mutant
beta-sheets
alpha-helices
turns
binding sites
active sites
-3.7 hydrophobicity (Kyte & Doolittle, 1982, J. Mol., 157, 105-132) 3.7

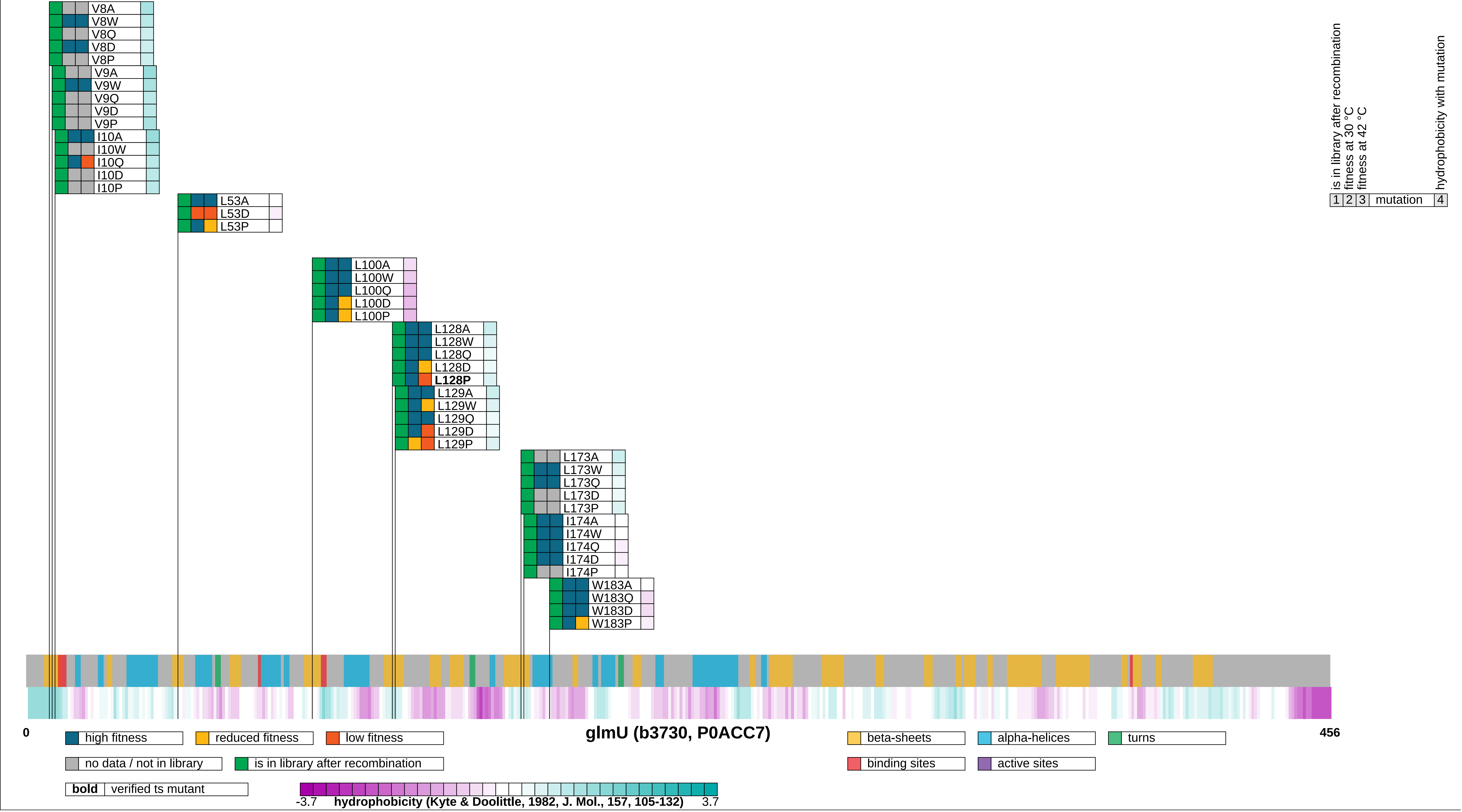
V8A
V8W
V8Q
V8D
V8P
V9A
V9W
V9Q
V9D
V9P
I10A
I10W
I10Q
I10D
I10P
L53A
L53D
L53P
L100A
L100W
L100Q
L100D
L100P
L128A
L128W
L128Q
L128D
L128P
L129A
L129W
L129Q
L129D
L129P
L173A
L173W
L173Q
L173D
L173P
I174A
I174W
I174Q
I174D
I174P
W183A
W183Q
W183D
W183P
is in library after recombination
fitness at 30 °C
fitness at 42 °C
hydrophobicity with mutation
1 2 3 mutation 4
0
glmU (b3730, P0ACC7)
456
high fitness
reduced fitness
low fitness
beta-sheets
alpha-helices
turns
no data / not in library
is in library after recombination
binding sites
active sites
bold verified ts mutant
-3.7
hydrophobicity (Kyte & Doolittle, 1982, J. Mol., 157, 105-132)
3.7

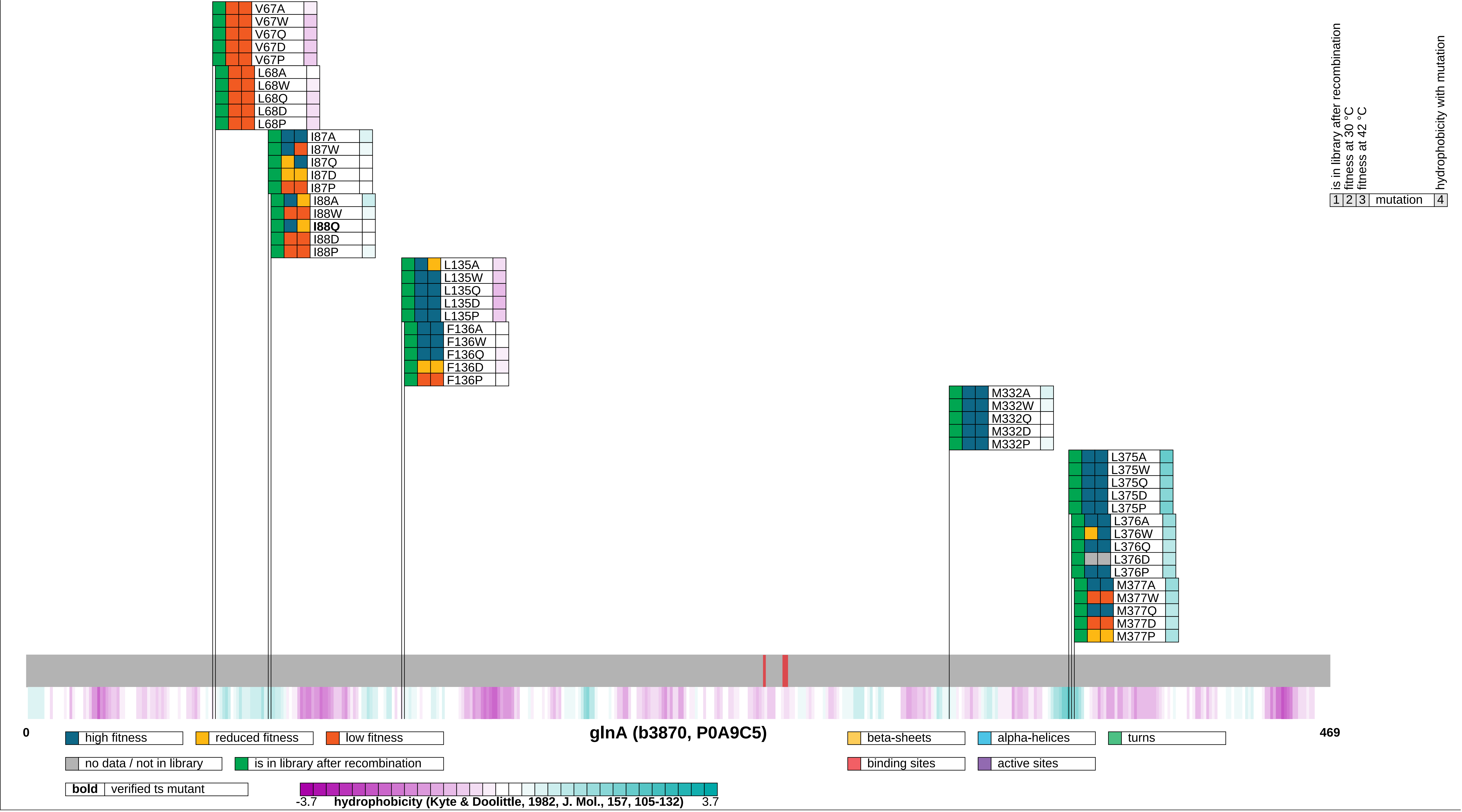

V67A
V67W
V67Q
V67D
V67P
L68A
L68W
L68Q
L68D
L68P
I87A
I87W
I87Q
I87D
I87P
I88A
I88W
I88Q
I88D
I88P
L135A
L135W
L135Q
L135D
L135P
F136A
F136W
F136Q
F136D
F136P
M332A
M332W
M332Q
M332D
M332P
L375A
L375W
L375Q
L375D
L375P
L376A
L376W
L376Q
L376D
L376P
M377A
M377W
M377Q
M377D
M377P
is in library after recombination
fitness at 30 °C
fitness at 42 °C
hydrophobicity with mutation
1 2 3 mutation 4
0
469
glnA (b3870, P0A9C5)
high fitness
reduced fitness
low fitness
beta-sheets
alpha-helices
turns
no data / not in library
is in library after recombination
binding sites
active sites
bold verified ts mutant
-3.7
hydrophobicity (Kyte & Doolittle, 1982, J. Mol., 157, 105-132)
3.7

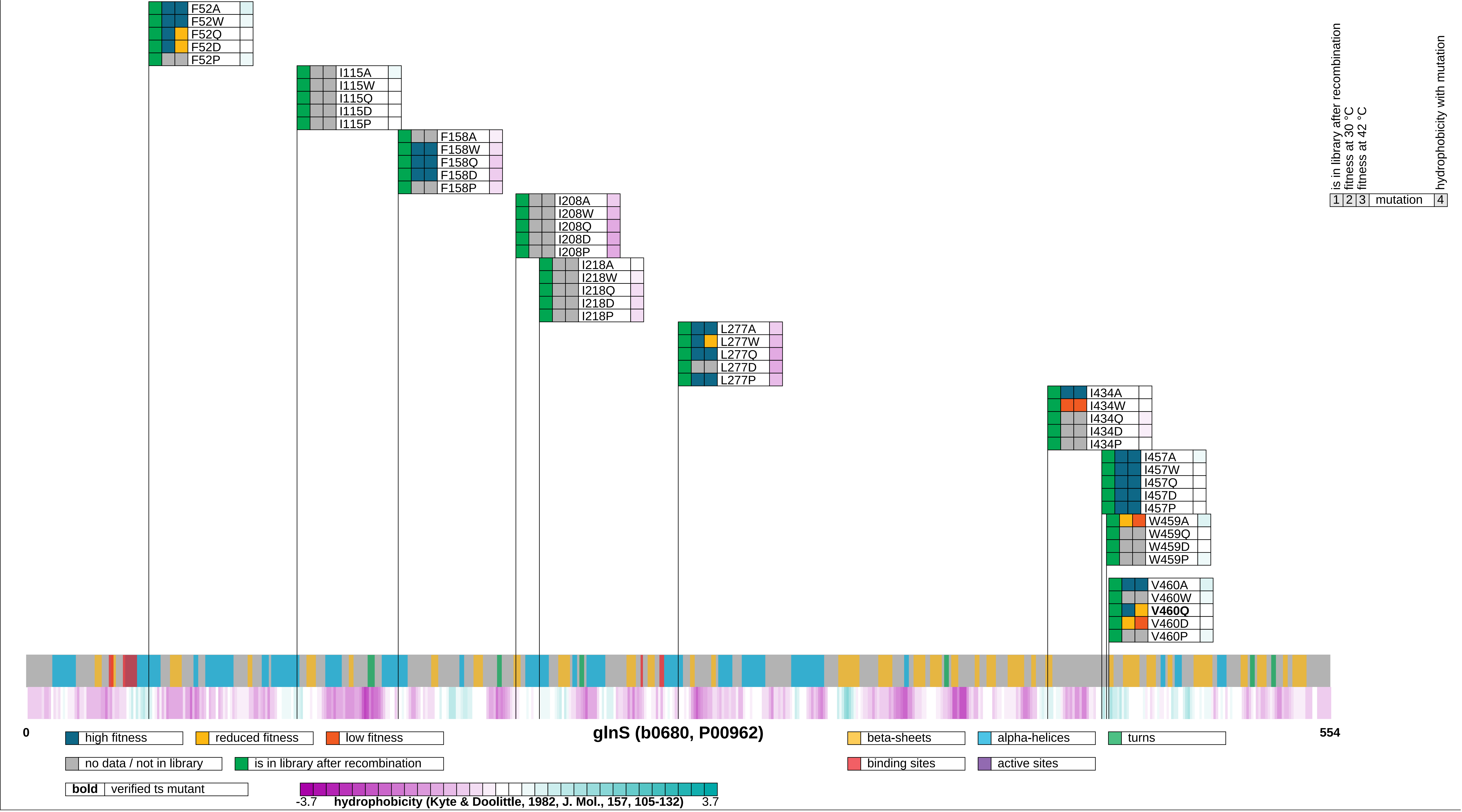

F52A
F52W
F52Q
F52D
F52P
I115A
I115W
I115Q
I115D
I115P
F158A
F158W
F158Q
F158D
F158P
I208A
I208W
I208Q
I208D
I208P
I218A
I218W
I218Q
I218D
I218P
L277A
L277W
L277Q
L277D
L277P
I434A
I434W
I434Q
I434D
I434P
I457A
I457W
I457Q
I457D
I457P
W459A
W459Q
W459D
W459P
V460A
V460W
V460Q
V460D
V460P
is in library after recombination
fitness at 30 °C
fitness at 42 °C
hydrophobicity with mutation
1 2 3 mutation 4
0
glnS (b0680, P00962)
554
high fitness
reduced fitness
low fitness
no data / not in library
is in library after recombination
bold verified ts mutant
beta-sheets
alpha-helices
turns
binding sites
active sites
-3.7
hydrophobicity (Kyte & Doolittle, 1982, J. Mol., 157, 105-132)
3.7

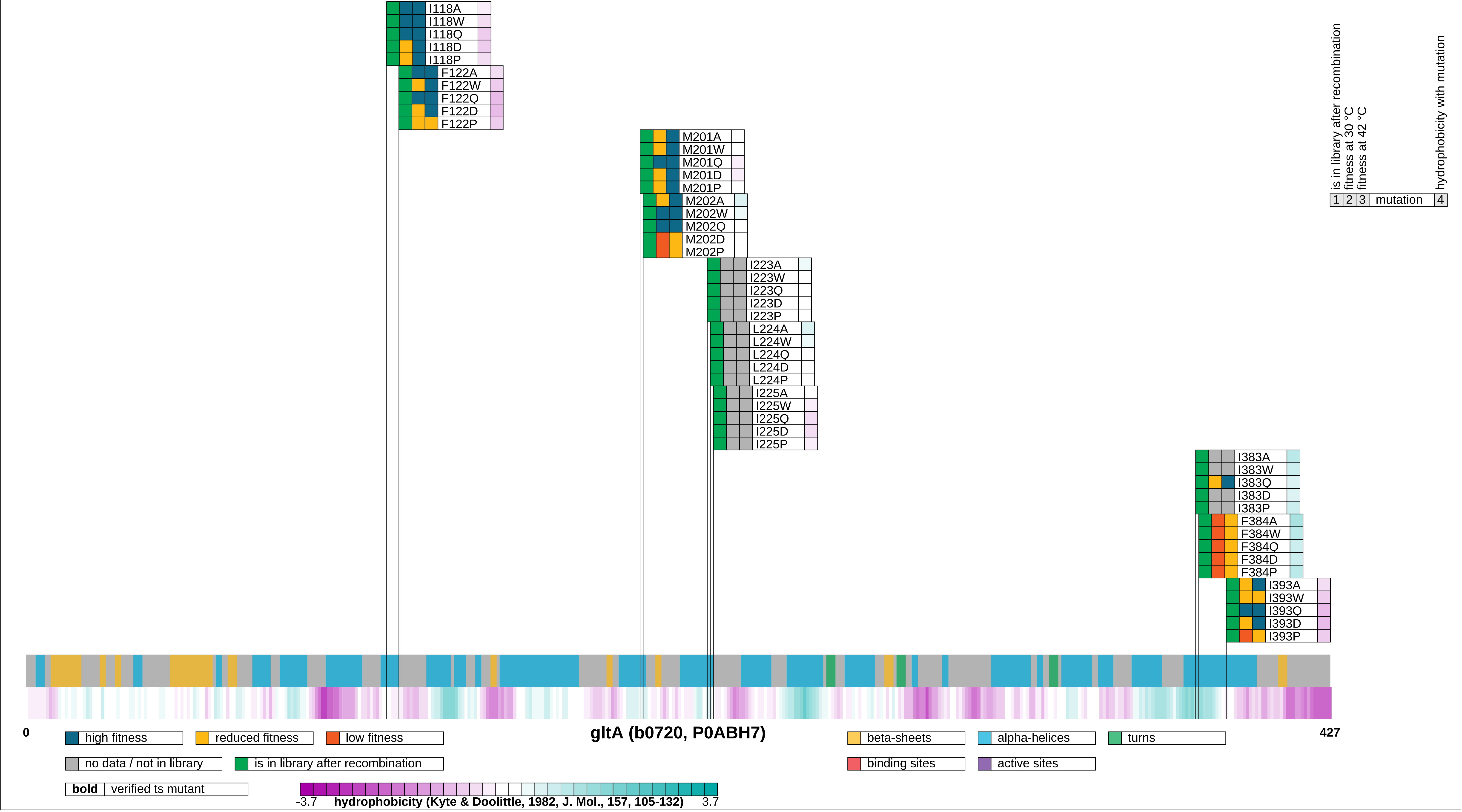

I118A
I118W
I118Q
I118D
I118P
F122A
F122W
F122Q
F122D
F122P
M201A
M201W
M201Q
M201D
M201P
M202A
M202W
M202Q
M202D
M202P
I223A
I223W
I223Q
I223D
I223P
L224A
L224W
L224Q
L224D
L224P
I225A
I225W
I225Q
I225D
I225P
I383A
I383W
I383Q
I383D
I383P
F384A
F384W
F384Q
F384D
F384P
I393A
I393W
I393Q
I393D
I393P
is in library after recombination
fitness at 30 °C
fitness at 42 °C
hydrophobicity with mutation
1 2 3 mutation 4
0
gltA (b0720, P0ABH7)
427
high fitness
reduced fitness
low fitness
beta-sheets
alpha-helices
turns
no data / not in library
is in library after recombination
binding sites
active sites
bold verified ts mutant
-3.7 hydrophobicity (Kyte & Doolittle, 1982, J. Mol., 157, 105-132) 3.7

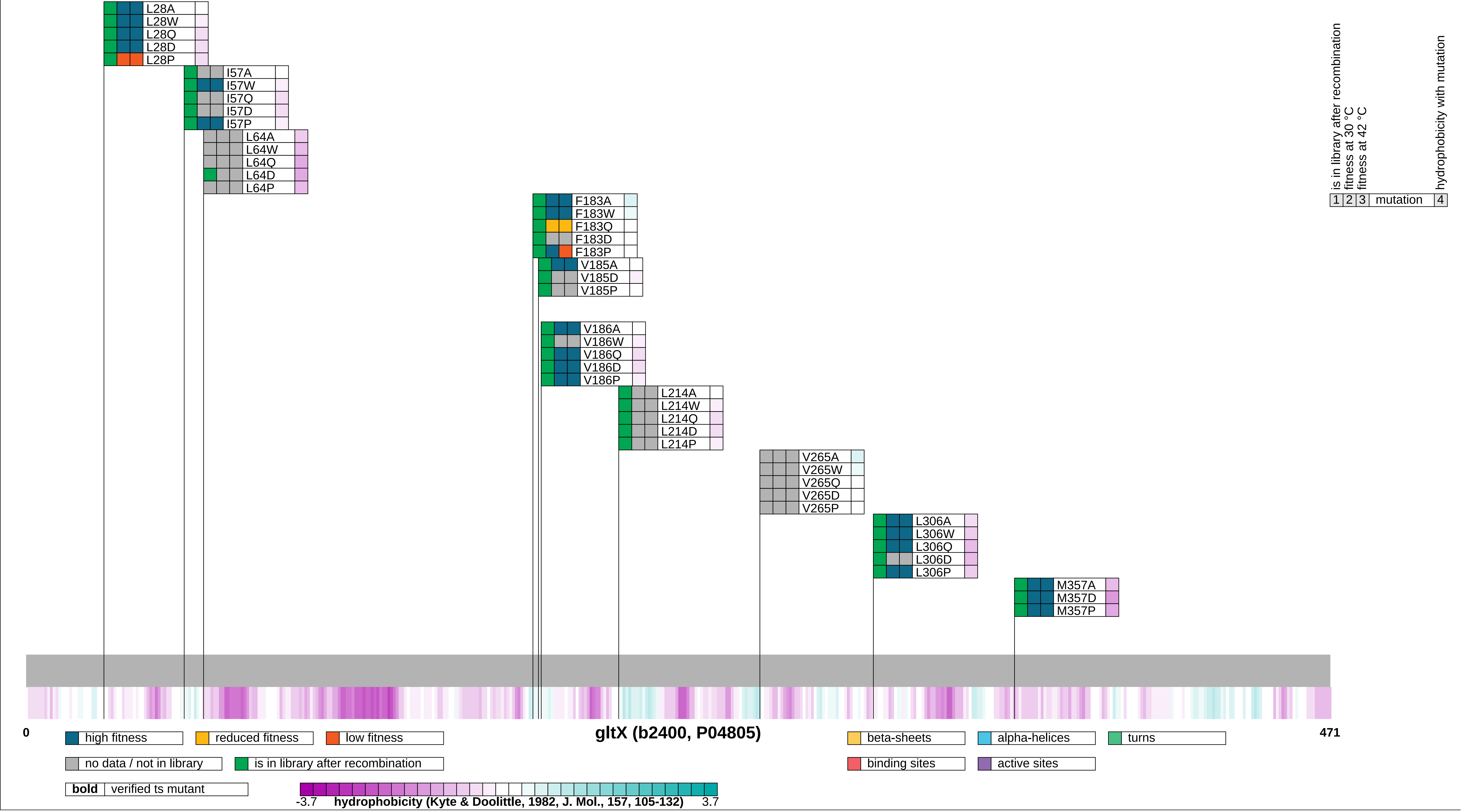

L28A
L28W
L28Q
L28D
L28P
I57A
I57W
I57Q
I57D
I57P
L64A
L64W
L64Q
L64D
L64P
F183A
F183W
F183Q
F183D
F183P
V185A
V185D
V185P
V186A
V186W
V186Q
V186D
V186P
L214A
L214W
L214Q
L214D
L214P
V265A
V265W
V265Q
V265D
V265P
L306A
L306W
L306Q
L306D
L306P
M357A
M357D
M357P
is in library after recombination
fitness at 30 °C
fitness at 42 °C
hydrophobicity with mutation
1 2 3 mutation 4
0
gltX (b2400, P04805)
471
high fitness
reduced fitness
low fitness
no data / not in library
is in library after recombination
bold verified ts mutant
beta-sheets
alpha-helices
turns
binding sites
active sites
-3.7
hydrophobicity (Kyte & Doolittle, 1982, J. Mol., 157, 105-132)
3.7

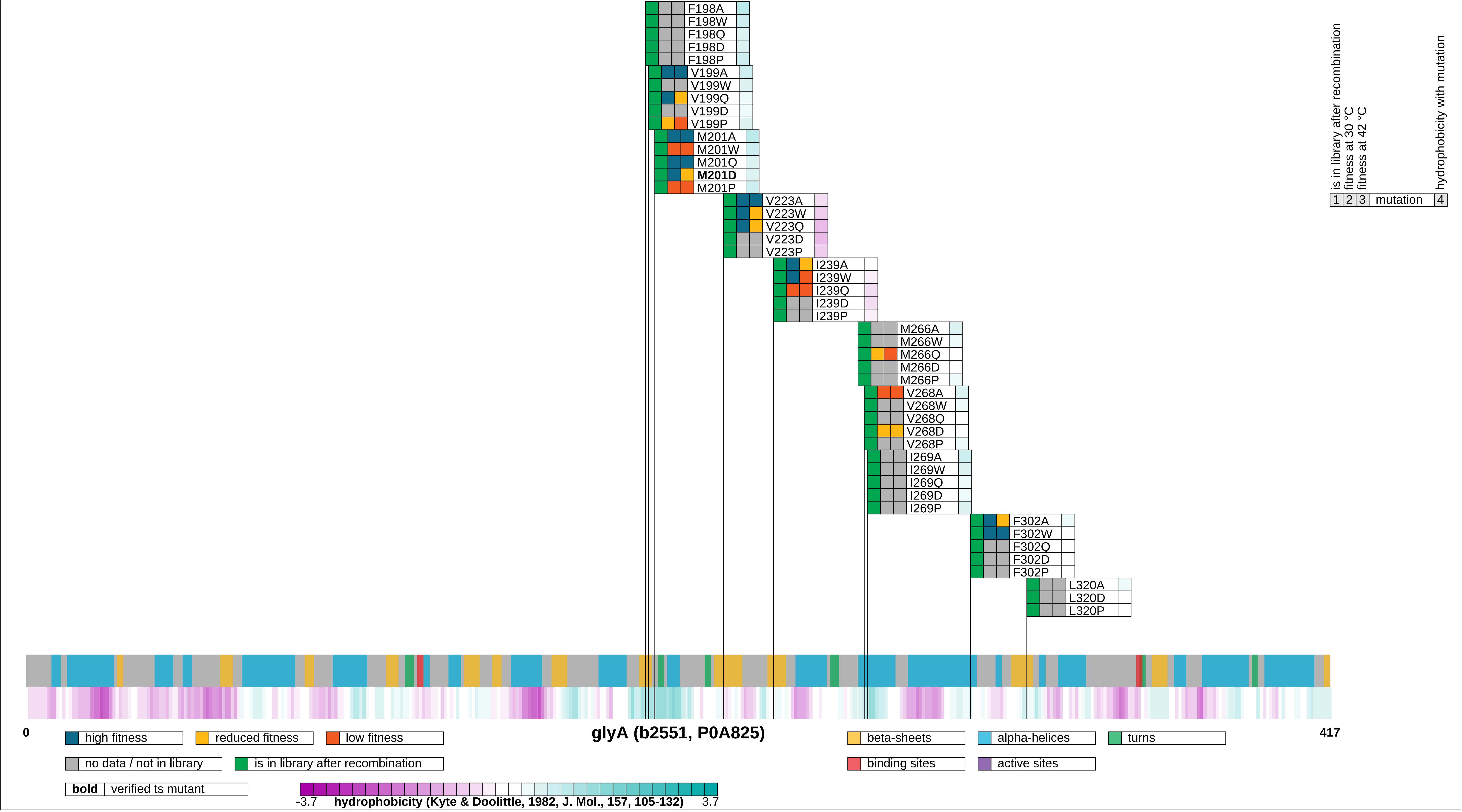

F198A
F198W
F198Q
F198D
F198P
V199A
V199W
V199Q
V199D
V199P
M201A
M201W
M201Q
M201D
M201P
V223A
V223W
V223Q
V223D
V223P
I239A
I239W
I239Q
I239D
I239P
M266A
M266W
M266Q
M266D
M266P
V268A
V268W
V268Q
V268D
V268P
I269A
I269W
I269Q
I269D
I269P
F302A
F302W
F302Q
F302D
F302P
L320A
L320D
L320P
is in library after recombination
fitness at 30 °C
fitness at 42 °C
hydrophobicity with mutation
1 2 3 mutation 4
0
glyA (b2551, P0A825)
417
high fitness
reduced fitness
low fitness
beta-sheets
alpha-helices
turns
no data / not in library
is in library after recombination
binding sites
active sites
bold verified ts mutant
-3.7 hydrophobicity (Kyte & Doolittle, 1982, J. Mol., 157, 105-132) 3.7

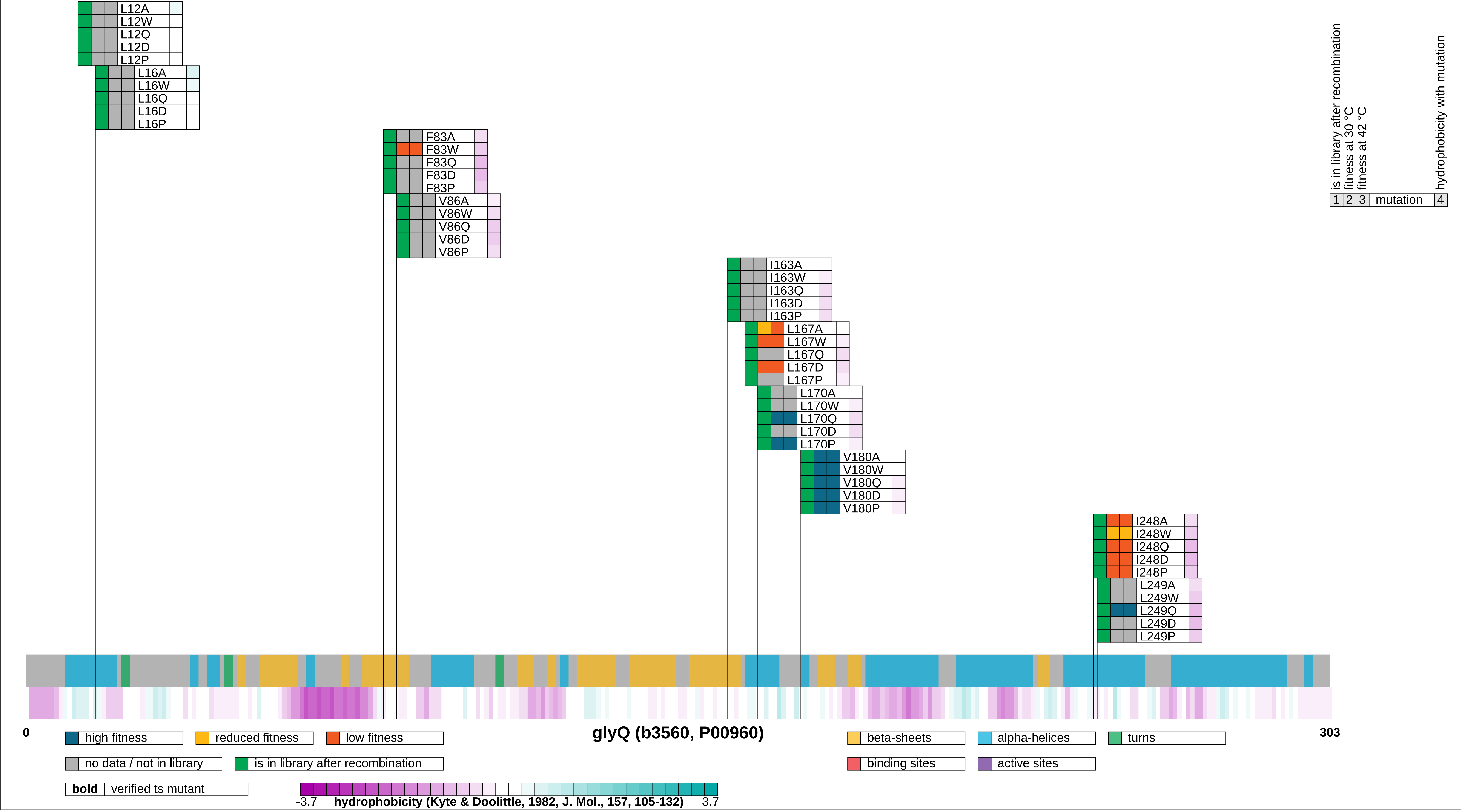
L12A
L12W
L12Q
L12D
L12P
L16A
L16W
L16Q
L16D
L16P
F83A
F83W
F83Q
F83D
F83P
V86A
V86W
V86Q
V86D
V86P
I163A
I163W
I163Q
I163D
I163P
L167A
L167W
L167Q
L167D
L167P
L170A
L170W
L170Q
L170D
L170P
V180A
V180W
V180Q
V180D
V180P
I248A
I248W
I248Q
I248D
I248P
L249A
L249W
L249Q
L249D
L249P
is in library after recombination
fitness at 30 °C
fitness at 42 °C
hydrophobicity with mutation
1 2 3 mutation 4
0
glyQ (b3560, P00960)
303
high fitness
reduced fitness
low fitness
no data / not in library
is in library after recombination
bold verified ts mutant
beta-sheets
alpha-helices
turns
binding sites
active sites
-3.7 hydrophobicity (Kyte & Doolittle, 1982, J. Mol., 157, 105-132) 3.7

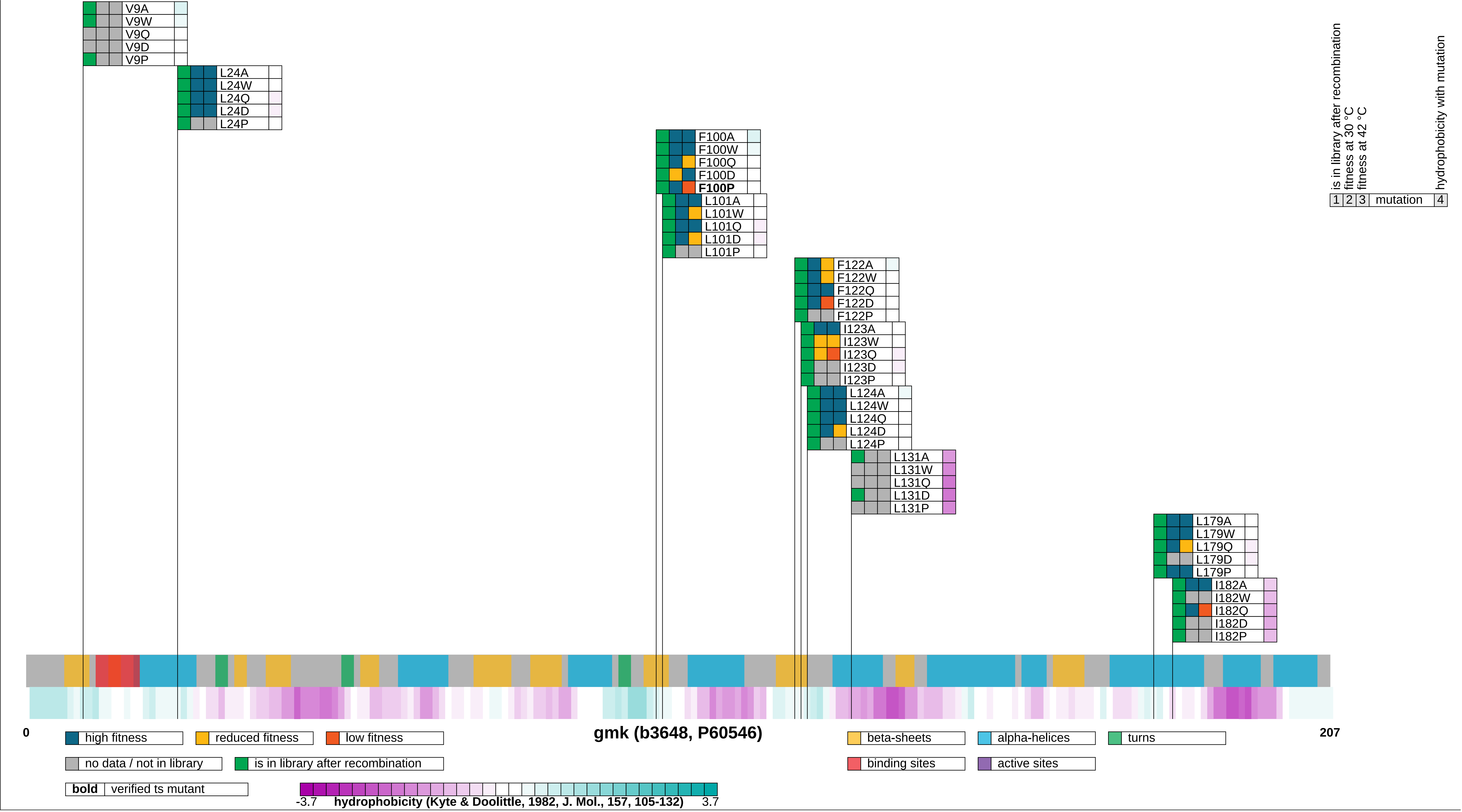

V9A
V9W
V9Q
V9D
V9P
L24A
L24W
L24Q
L24D
L24P
F100A
F100W
F100Q
F100D
F100P
L101A
L101W
L101Q
L101D
L101P
F122A
F122W
F122Q
F122D
F122P
I123A
I123W
I123Q
I123D
I123P
L124A
L124W
L124Q
L124D
L124P
L131A
L131W
L131Q
L131D
L131P
L179A
L179W
L179Q
L179D
L179P
I182A
I182W
I182Q
I182D
I182P
is in library after recombination
fitness at 30 °C
fitness at 42 °C
hydrophobicity with mutation
1 2 3 mutation 4
0
gmk (b3648, P60546)
207
high fitness
reduced fitness
low fitness
beta-sheets
alpha-helices
turns
no data / not in library
is in library after recombination
binding sites
active sites
bold verified ts mutant
-3.7
hydrophobicity (Kyte & Doolittle, 1982, J. Mol., 157, 105-132)
3.7

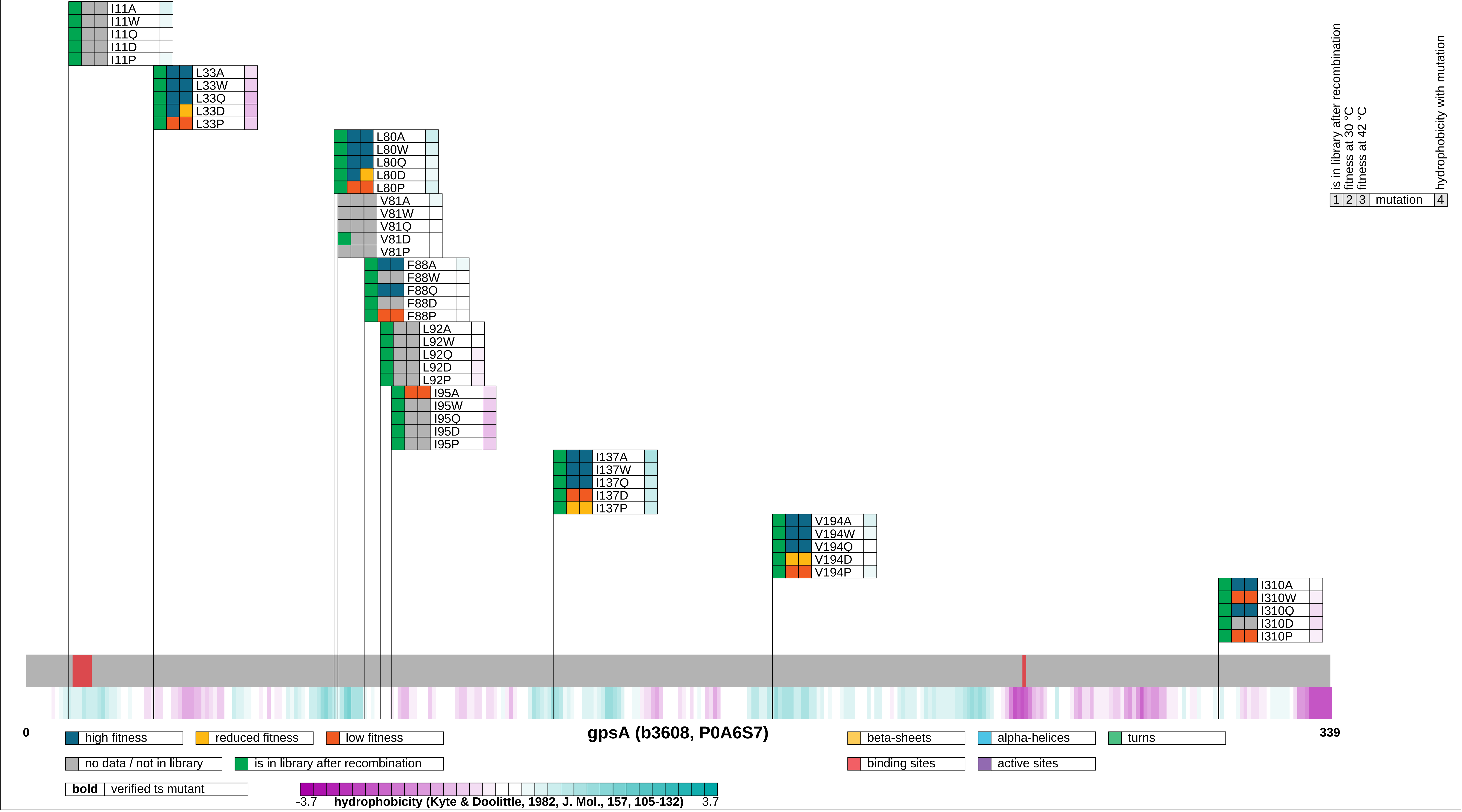

I11A
I11W
I11Q
I11D
I11P
L33A
L33W
L33Q
L33D
L33P
L80A
L80W
L80Q
L80D
L80P
V81A
V81W
V81Q
V81D
V81P
F88A
F88W
F88Q
F88D
F88P
L92A
L92W
L92Q
L92D
L92P
I95A
I95W
I95Q
I95D
I95P
I137A
I137W
I137Q
I137D
I137P
V194A
V194W
V194Q
V194D
V194P
I310A
I310W
I310Q
I310D
I310P
is in library after recombination
fitness at 30 °C
fitness at 42 °C
hydrophobicity with mutation
1 2 3 mutation 4
0
gpsA (b3608, P0A6S7)
339
high fitness
reduced fitness
low fitness
beta-sheets
alpha-helices
turns
no data / not in library
is in library after recombination
binding sites
active sites
bold verified ts mutant
-3.7
hydrophobicity (Kyte & Doolittle, 1982, J. Mol., 157, 105-132)
3.7

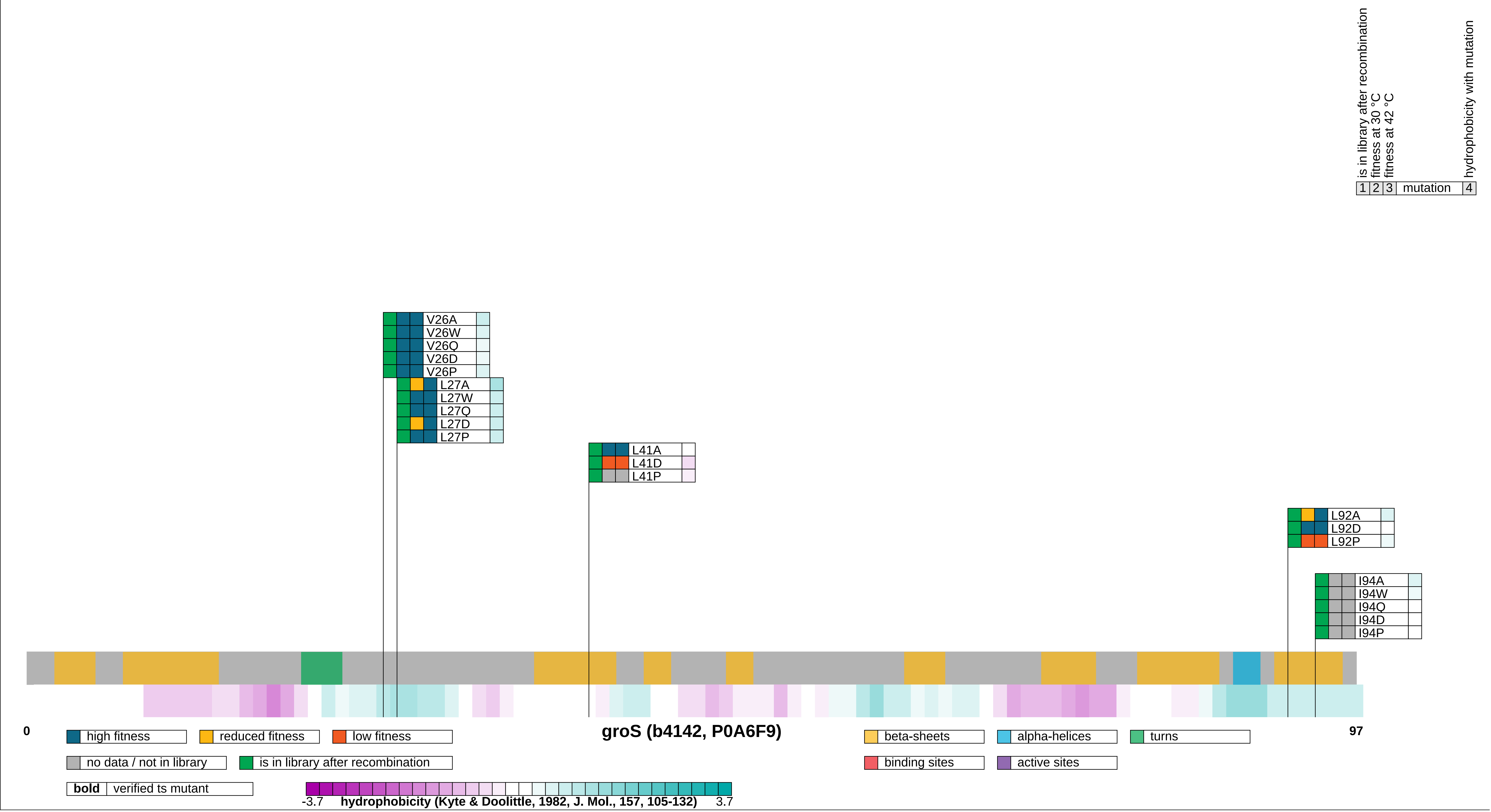

is in library after recombination
fitness at 30 °C
fitness at 42 °C
hydrophobicity with mutation
1 2 3 mutation 4
V26A
V26W
V26Q
V26D
V26P
L27A
L27W
L27Q
L27D
L27P
L41A
L41D
L41P
L92A
L92D
L92P
I94A
I94W
I94Q
I94D
I94P
0
groS (b4142, P0A6F9)
97
high fitness
reduced fitness
low fitness
beta-sheets
alpha-helices
turns
no data / not in library
is in library after recombination
binding sites
active sites
bold verified ts mutant
-3.7
hydrophobicity (Kyte & Doolittle, 1982, J. Mol., 157, 105-132)
3.7

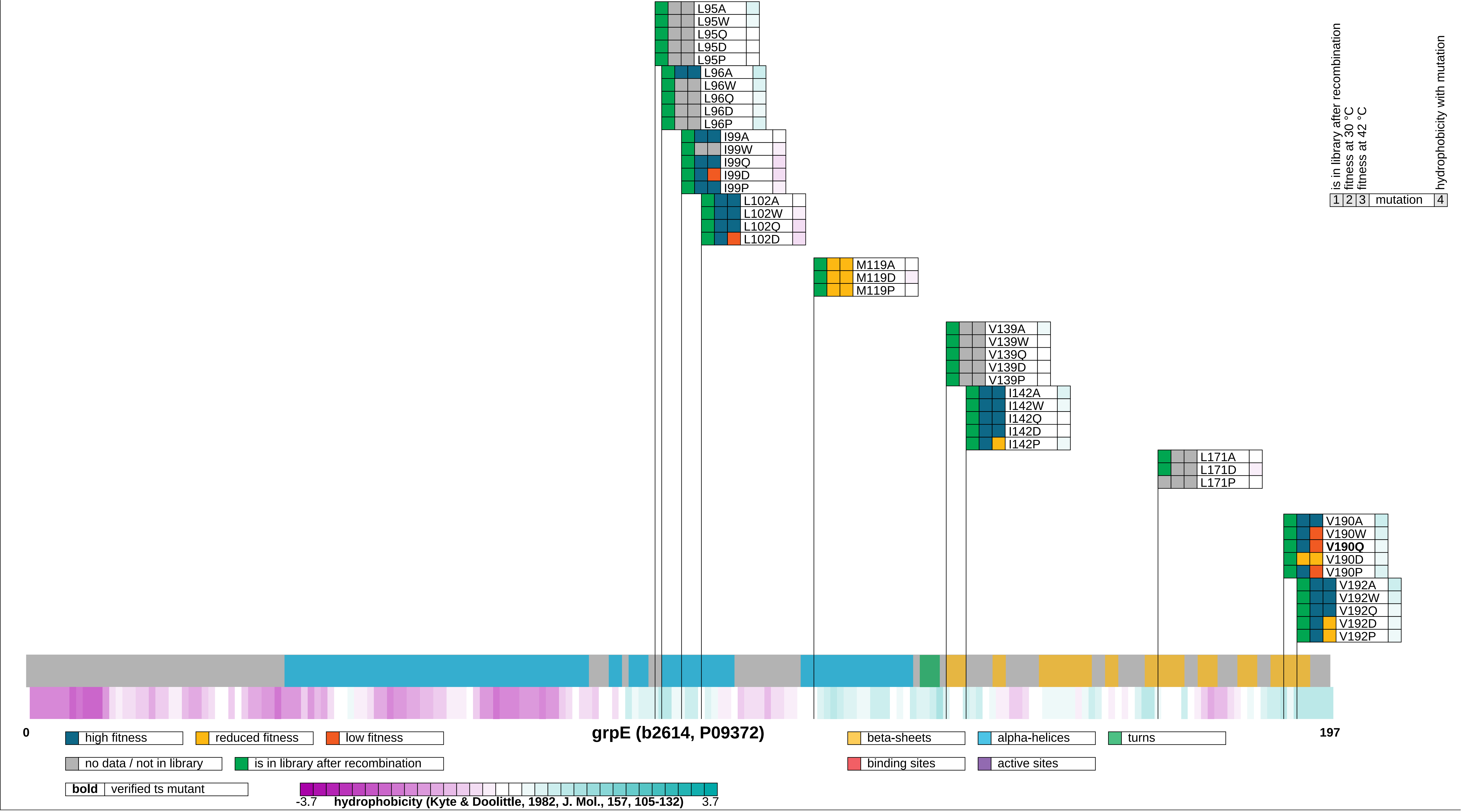

L95A
L95W
L95Q
L95D
L95P
L96A
L96W
L96Q
L96D
L96P
I99A
I99W
I99Q
I99D
I99P
L102A
L102W
L102Q
L102D
M119A
M119D
M119P
V139A
V139W
V139Q
V139D
V139P
I142A
I142W
I142Q
I142D
I142P
L171A
L171D
L171P
V190A
V190W
V190Q
V190D
V190P
V192A
V192W
V192Q
V192D
V192P
is in library after recombination
fitness at 30 °C
fitness at 42 °C
hydrophobicity with mutation
1
2
3
mutation
4
0
197
grpE (b2614, P09372)
high fitness
reduced fitness
low fitness
no data / not in library
is in library after recombination
bold verified ts mutant
beta-sheets
alpha-helices
turns
binding sites
active sites
-3.7
hydrophobicity (Kyte & Doolittle, 1982, J. Mol., 157, 105-132)
3.7

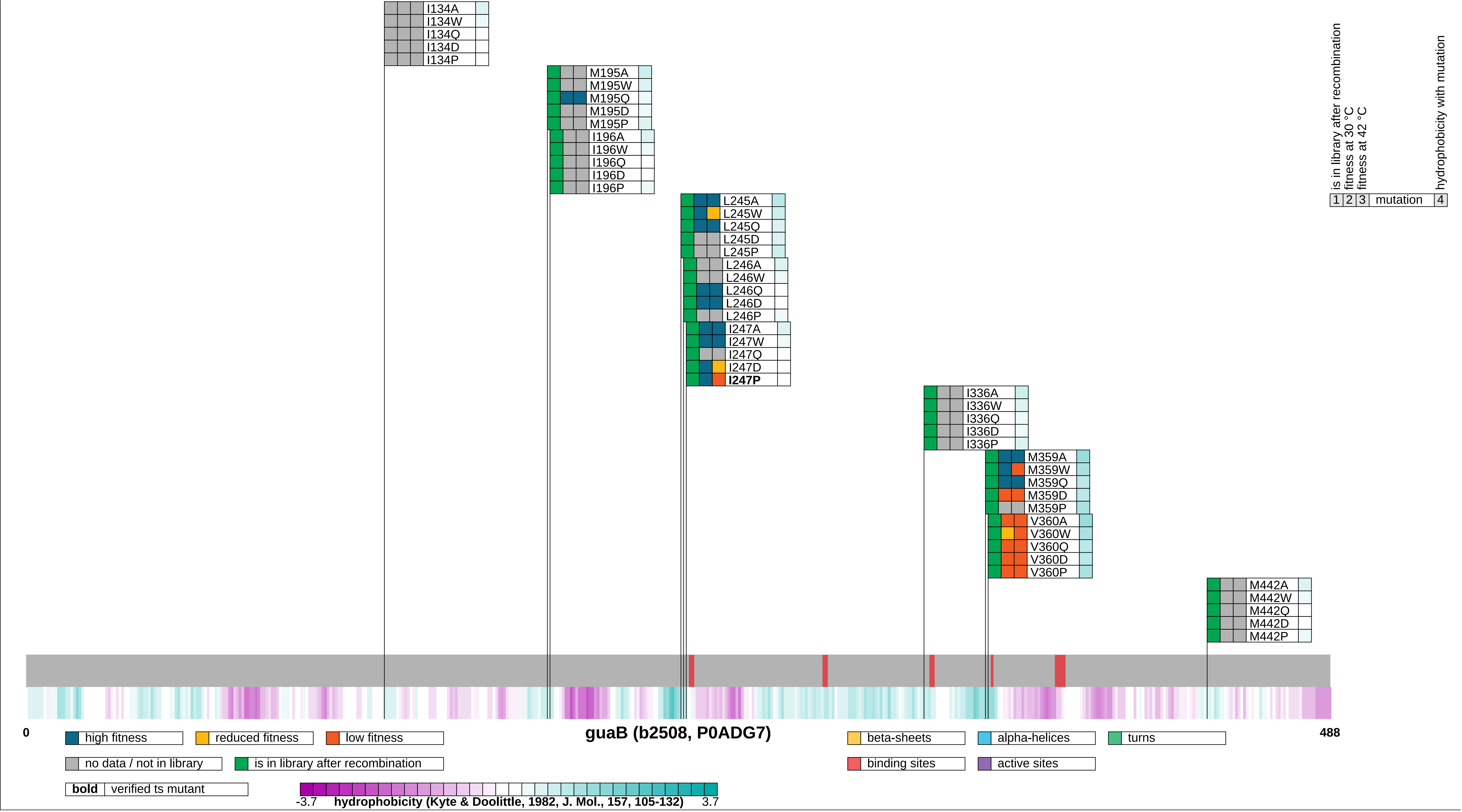

I134A
I134W
I134Q
I134D
I134P
M195A
M195W
M195Q
M195D
M195P
I196A
I196W
I196Q
I196D
I196P
L245A
L245W
L245Q
L245D
L245P
L246A
L246W
L246Q
L246D
L246P
I247A
I247W
I247Q
I247D
I247P
I336A
I336W
I336Q
I336D
I336P
M359A
M359W
M359Q
M359D
M359P
V360A
V360W
V360Q
V360D
V360P
M442A
M442W
M442Q
M442D
M442P
is in library after recombination
fitness at 30 °C
fitness at 42 °C
hydrophobicity with mutation
1 2 3 mutation 4
0
guaB (b2508, P0ADG7)
488
high fitness
reduced fitness
low fitness
no data / not in library
is in library after recombination
bold verified ts mutant
beta-sheets
alpha-helices
turns
binding sites
active sites
-3.7
hydrophobicity (Kyte & Doolittle, 1982, J. Mol., 157, 105-132)
3.7

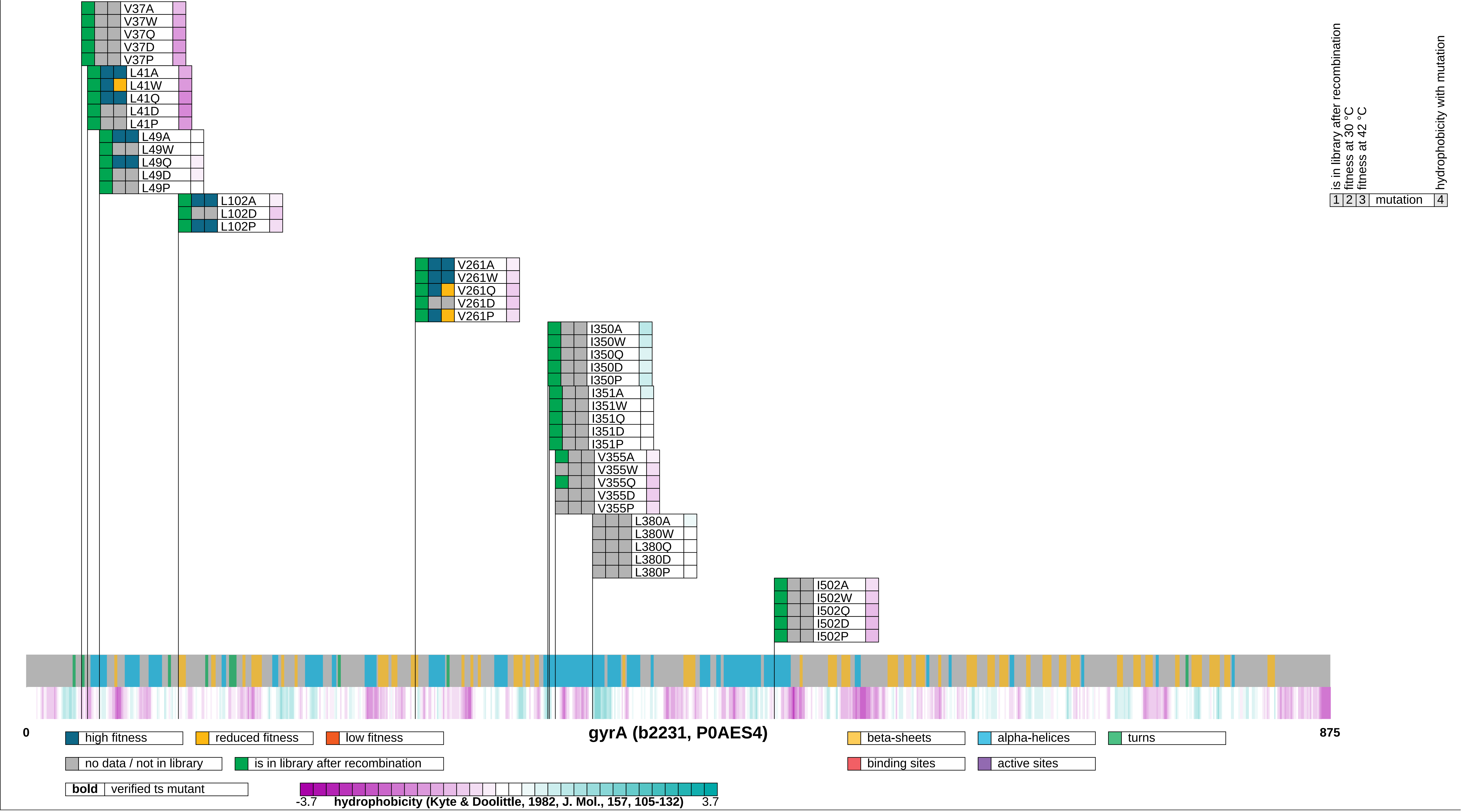

V37A
V37W
V37Q
V37D
V37P
L41A
L41W
L41Q
L41D
L41P
L49A
L49W
L49Q
L49D
L49P
L102A
L102D
L102P
V261A
V261W
V261Q
V261D
V261P
I350A
I350W
I350Q
I350D
I350P
I351A
I351W
I351Q
I351D
I351P
V355A
V355W
V355Q
V355D
V355P
L380A
L380W
L380Q
L380D
L380P
I502A
I502W
I502Q
I502D
I502P
is in library after recombination
fitness at 30 °C
fitness at 42 °C
hydrophobicity with mutation
1 2 3 mutation 4
0
gyrA (b2231, P0AES4)
875
high fitness
reduced fitness
low fitness
no data / not in library
is in library after recombination
bold verified ts mutant
beta-sheets
alpha-helices
turns
binding sites
active sites
-3.7
hydrophobicity (Kyte & Doolittle, 1982, J. Mol., 157, 105-132)
3.7

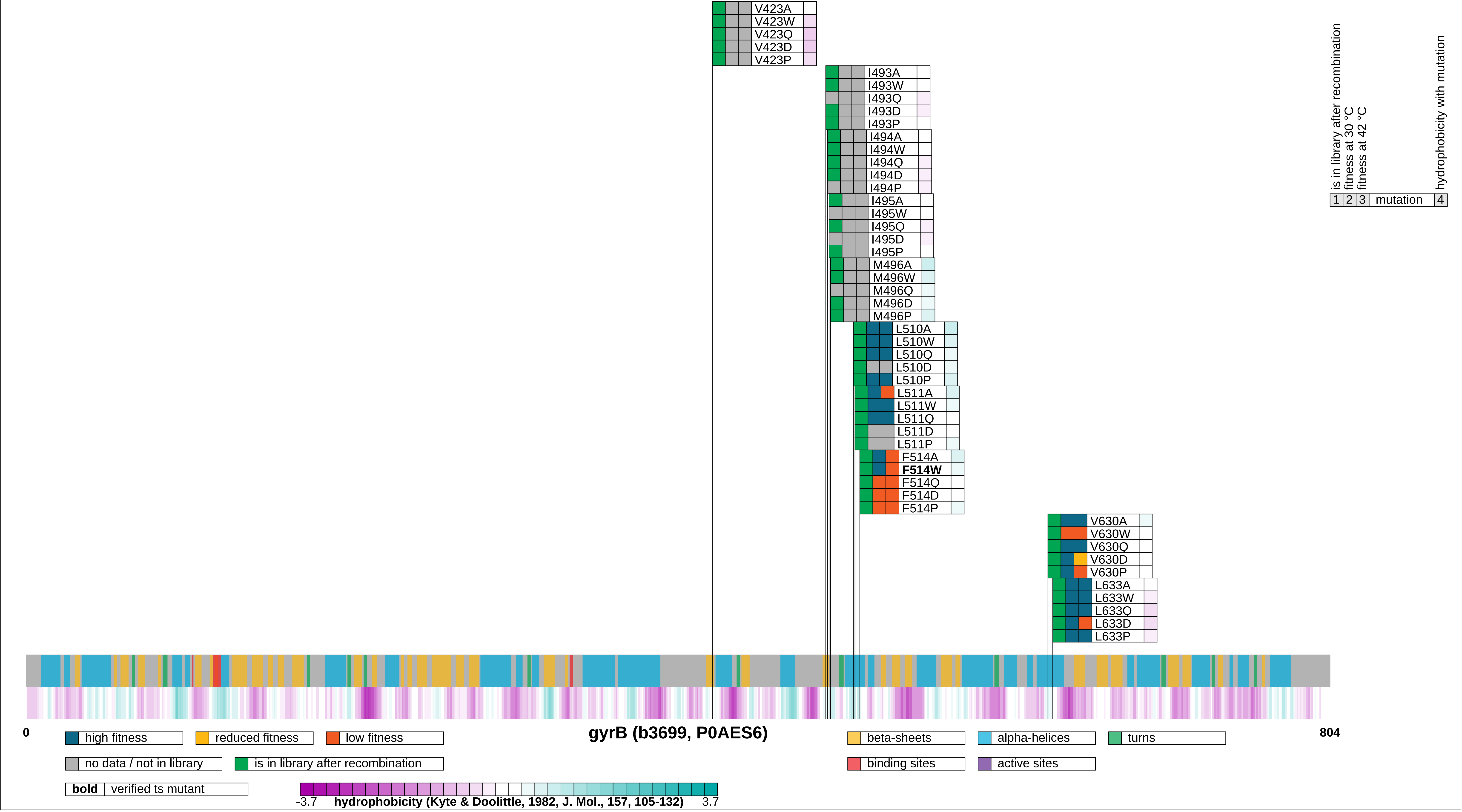

V423A
V423W
V423Q
V423D
V423P
I493A
I493W
I493Q
I493D
I493P
I494A
I494W
I494Q
I494D
I494P
I495A
I495W
I495Q
I495D
I495P
M496A
M496W
M496Q
M496D
M496P
L510A
L510W
L510Q
L510D
L510P
L511A
L511W
L511Q
L511D
L511P
F514A
F514W
F514Q
F514D
F514P
V630A
V630W
V630Q
V630D
V630P
L633A
L633W
L633Q
L633D
L633P
is in library after recombination
fitness at 30 °C
fitness at 42 °C
hydrophobicity with mutation
1 2 3 mutation 4
0
gyrB (b3699, P0AES6)
804
high fitness
reduced fitness
low fitness
no data / not in library
is in library after recombination
bold verified ts mutant
beta-sheets
alpha-helices
turns
binding sites
active sites
-3.7
hydrophobicity (Kyte & Doolittle, 1982, J. Mol., 157, 105-132)
3.7

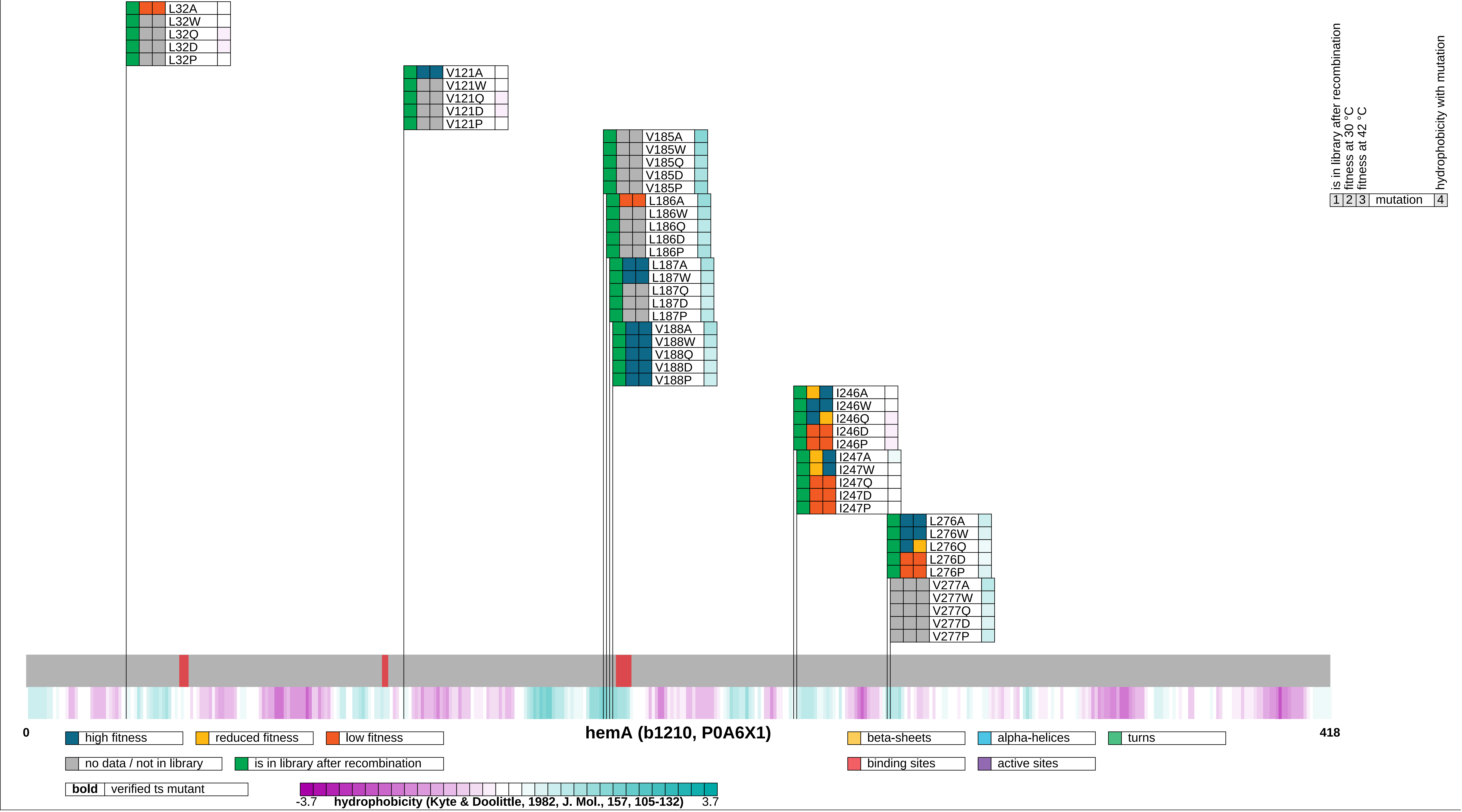

L32A
L32W
L32Q
L32D
L32P
V121A
V121W
V121Q
V121D
V121P
V185A
V185W
V185Q
V185D
V185P
L186A
L186W
L186Q
L186D
L186P
L187A
L187W
L187Q
L187D
L187P
V188A
V188W
V188Q
V188D
V188P
I246A
I246W
I246Q
I246D
I246P
I247A
I247W
I247Q
I247D
I247P
L276A
L276W
L276Q
L276D
L276P
V277A
V277W
V277Q
V277D
V277P
is in library after recombination
fitness at 30 °C
fitness at 42 °C
hydrophobicity with mutation
1 2 3 mutation 4
0
hemA (b1210, P0A6X1)
418
high fitness
reduced fitness
low fitness
no data / not in library
is in library after recombination
bold verified ts mutant
beta-sheets
alpha-helices
turns
binding sites
active sites
-3.7 hydrophobicity (Kyte & Doolittle, 1982, J. Mol., 157, 105-132) 3.7

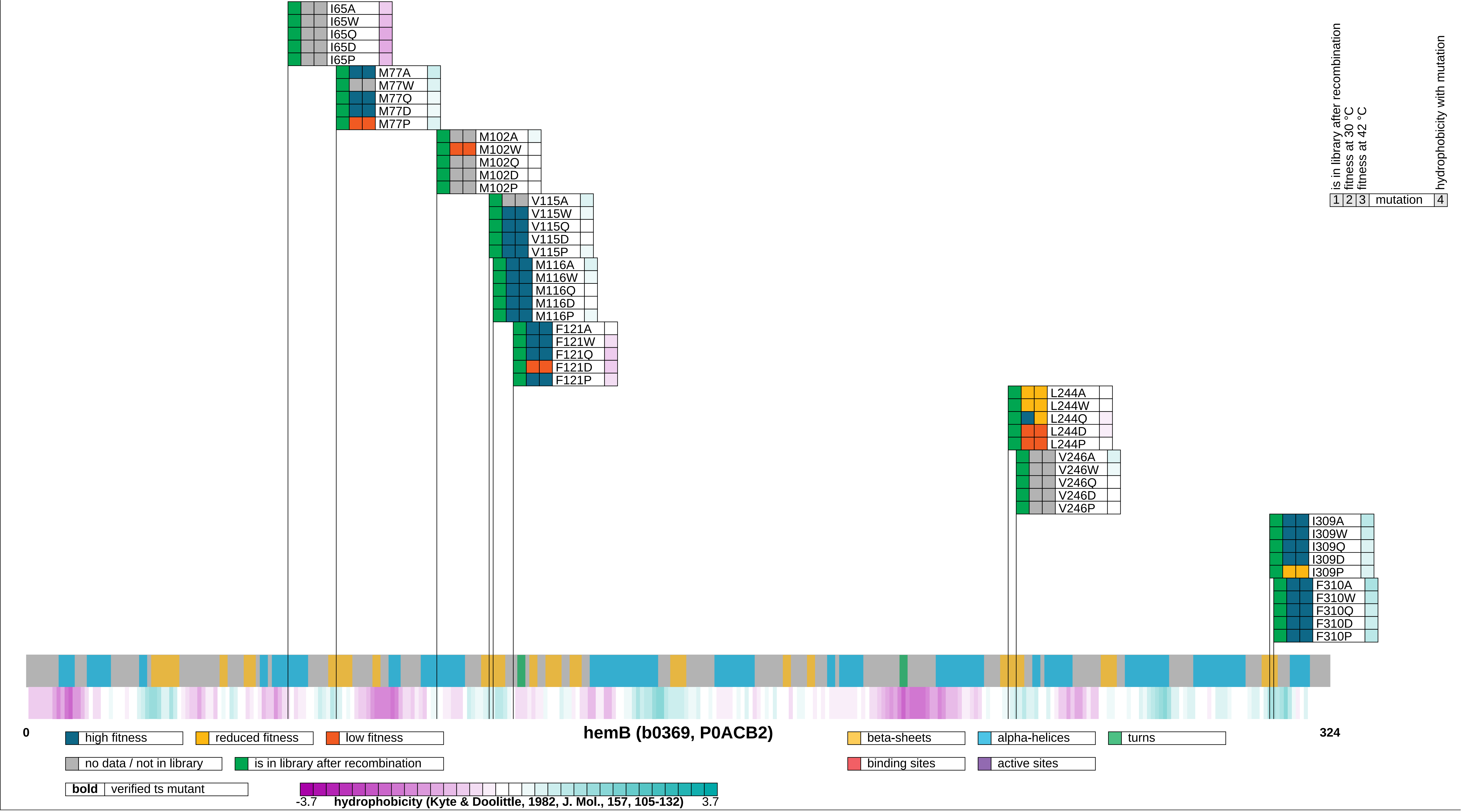

I65A
I65W
I65Q
I65D
I65P
M77A
M77W
M77Q
M77D
M77P
M102A
M102W
M102Q
M102D
M102P
V115A
V115W
V115Q
V115D
V115P
M116A
M116W
M116Q
M116D
M116P
F121A
F121W
F121Q
F121D
F121P
L244A
L244W
L244Q
L244D
L244P
V246A
V246W
V246Q
V246D
V246P
I309A
I309W
I309Q
I309D
I309P
F310A
F310W
F310Q
F310D
F310P
1 is in library after recombination
2 fitness at 30 °C
3 fitness at 42 °C
mutation
4 hydrophobicity with mutation
0
324
hemB (b0369, P0ACB2)
high fitness
reduced fitness
low fitness
no data / not in library
is in library after recombination
bold verified ts mutant
beta-sheets
alpha-helices
turns
binding sites
active sites
-3.7
hydrophobicity (Kyte & Doolittle, 1982, J. Mol., 157, 105-132)
3.7

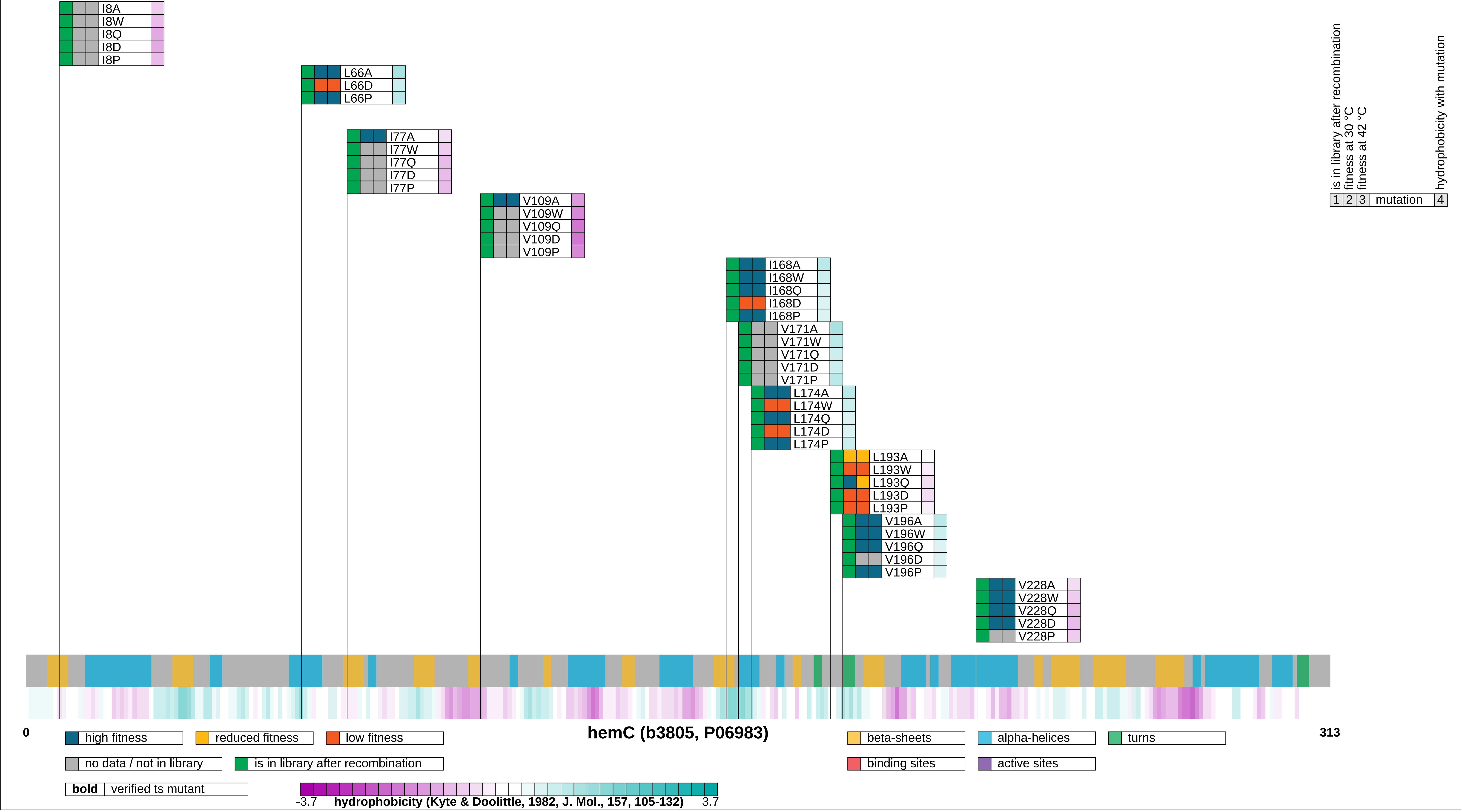

I8A
I8W
I8Q
I8D
I8P
L66A
L66D
L66P
I77A
I77W
I77Q
I77D
I77P
V109A
V109W
V109Q
V109D
V109P
I168A
I168W
I168Q
I168D
I168P
V171A
V171W
V171Q
V171D
V171P
L174A
L174W
L174Q
L174D
L174P
L193A
L193W
L193Q
L193D
L193P
V196A
V196W
V196Q
V196D
V196P
V228A
V228W
V228Q
V228D
V228P
is in library after recombination
fitness at 30 °C
fitness at 42 °C
hydrophobicity with mutation
1 2 3 mutation 4
0
hemC (b3805, P06983)
313
high fitness
reduced fitness
low fitness
beta-sheets
alpha-helices
turns
no data / not in library
is in library after recombination
binding sites
active sites
bold verified ts mutant
-3.7
hydrophobicity (Kyte & Doolittle, 1982, J. Mol., 157, 105-132)
3.7

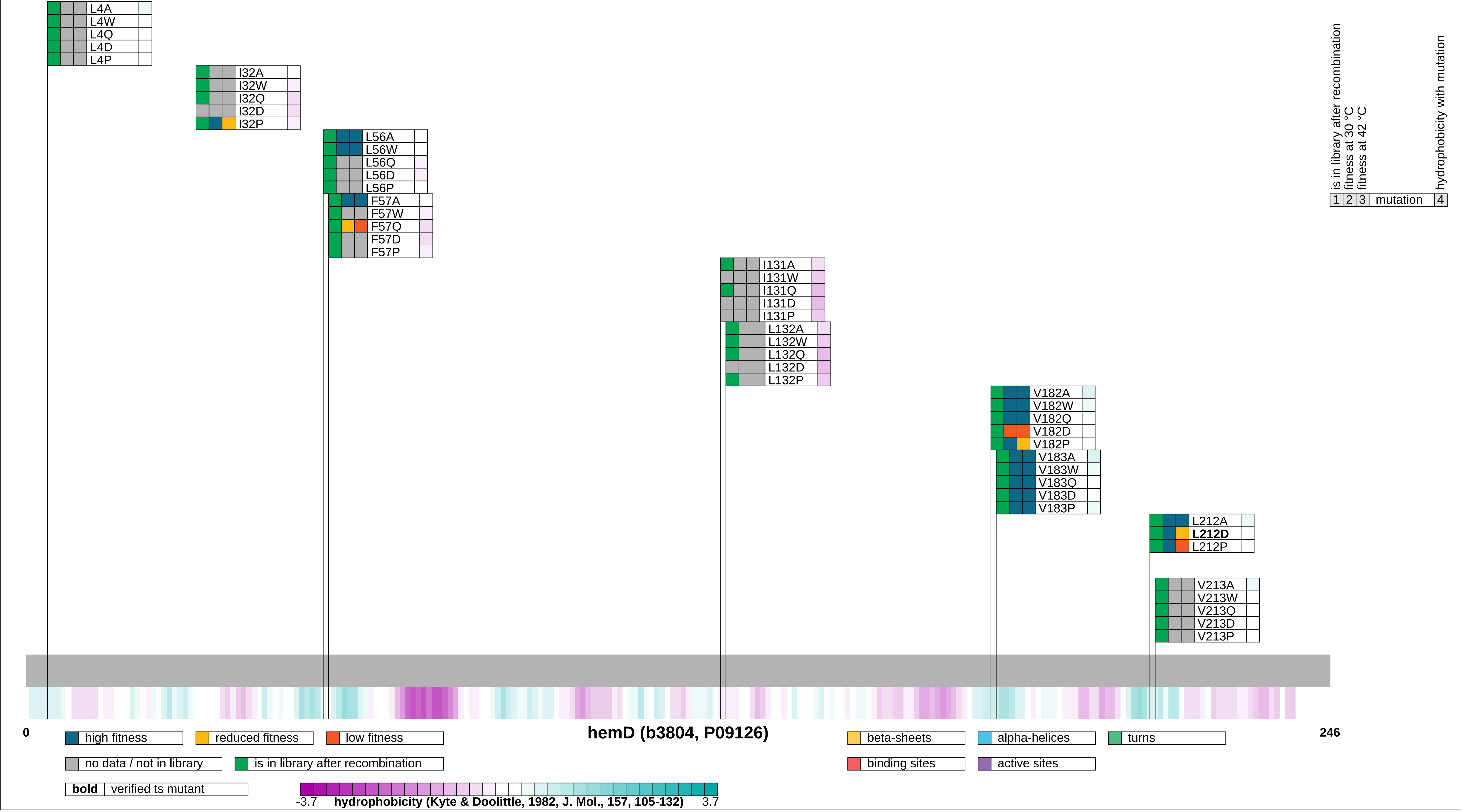

L4A
L4W
L4Q
L4D
L4P
I32A
I32W
I32Q
I32D
I32P
L56A
L56W
L56Q
L56D
L56P
F57A
F57W
F57Q
F57D
F57P
I131A
I131W
I131Q
I131D
I131P
L132A
L132W
L132Q
L132D
L132P
V182A
V182W
V182Q
V182D
V182P
V183A
V183W
V183Q
V183D
V183P
L212A
L212D
L212P
V213A
V213W
V213Q
V213D
V213P
is in library after recombination
fitness at 30 °C
fitness at 42 °C
hydrophobicity with mutation
1 2 3 mutation 4
0
hemD (b3804, P09126)
246
high fitness
reduced fitness
low fitness
beta-sheets
alpha-helices
turns
no data / not in library
is in library after recombination
binding sites
active sites
bold verified ts mutant
-3.7 hydrophobicity (Kyte & Doolittle, 1982, J. Mol., 157, 105-132) 3.7

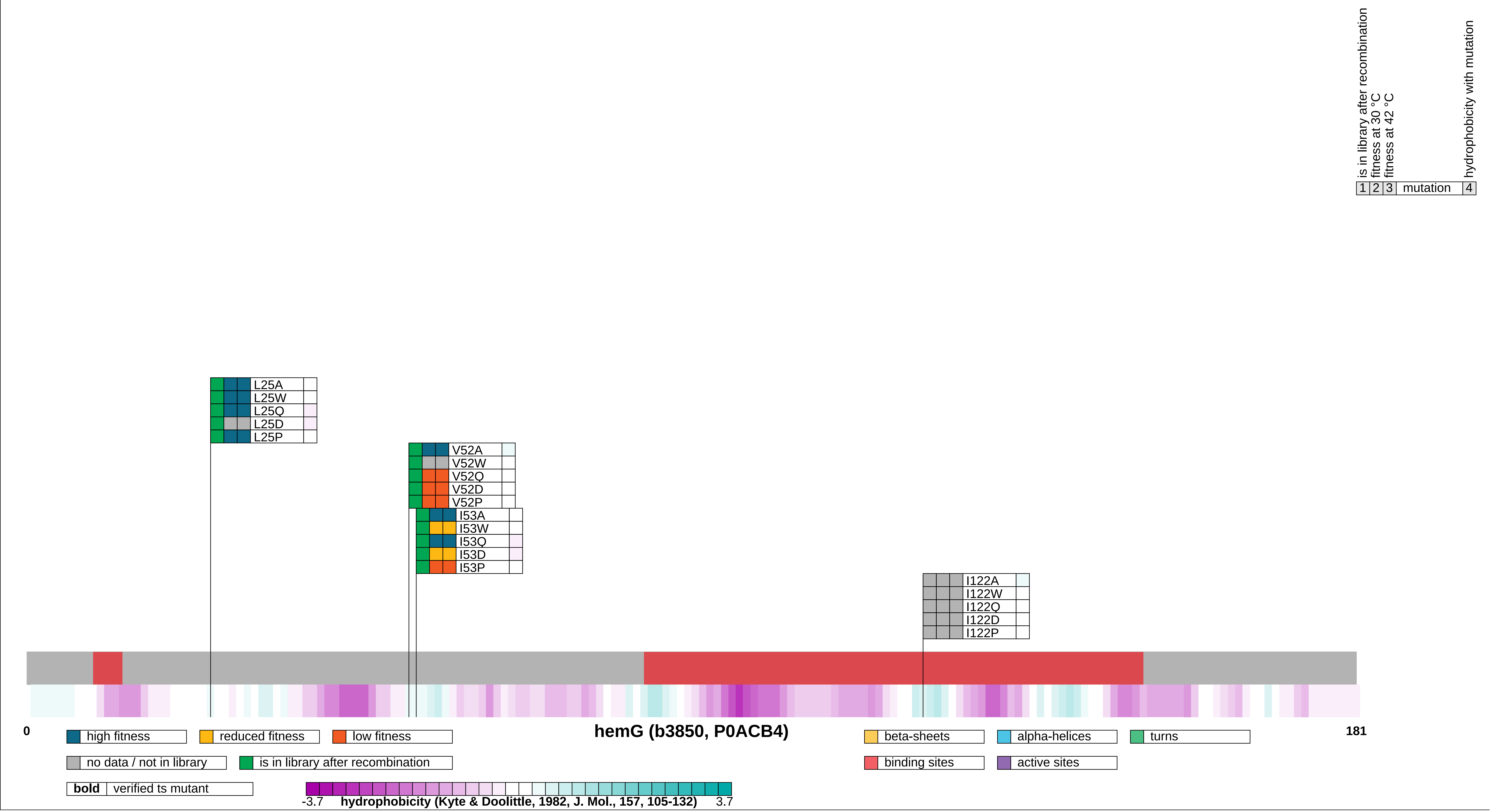

is in library after recombination
fitness at 30 °C
fitness at 42 °C
hydrophobicity with mutation
1
2
3
mutation
4
L25A
L25W
L25Q
L25D
L25P
V52A
V52W
V52Q
V52D
V52P
I53A
I53W
I53Q
I53D
I53P
I122A
I122W
I122Q
I122D
I122P
0
hemG (b3850, P0ACB4)
181
high fitness
reduced fitness
low fitness
beta-sheets
alpha-helices
turns
no data / not in library
is in library after recombination
binding sites
active sites
bold
verified ts mutant
-3.7
hydrophobicity (Kyte & Doolittle, 1982, J. Mol., 157, 105-132)
3.7

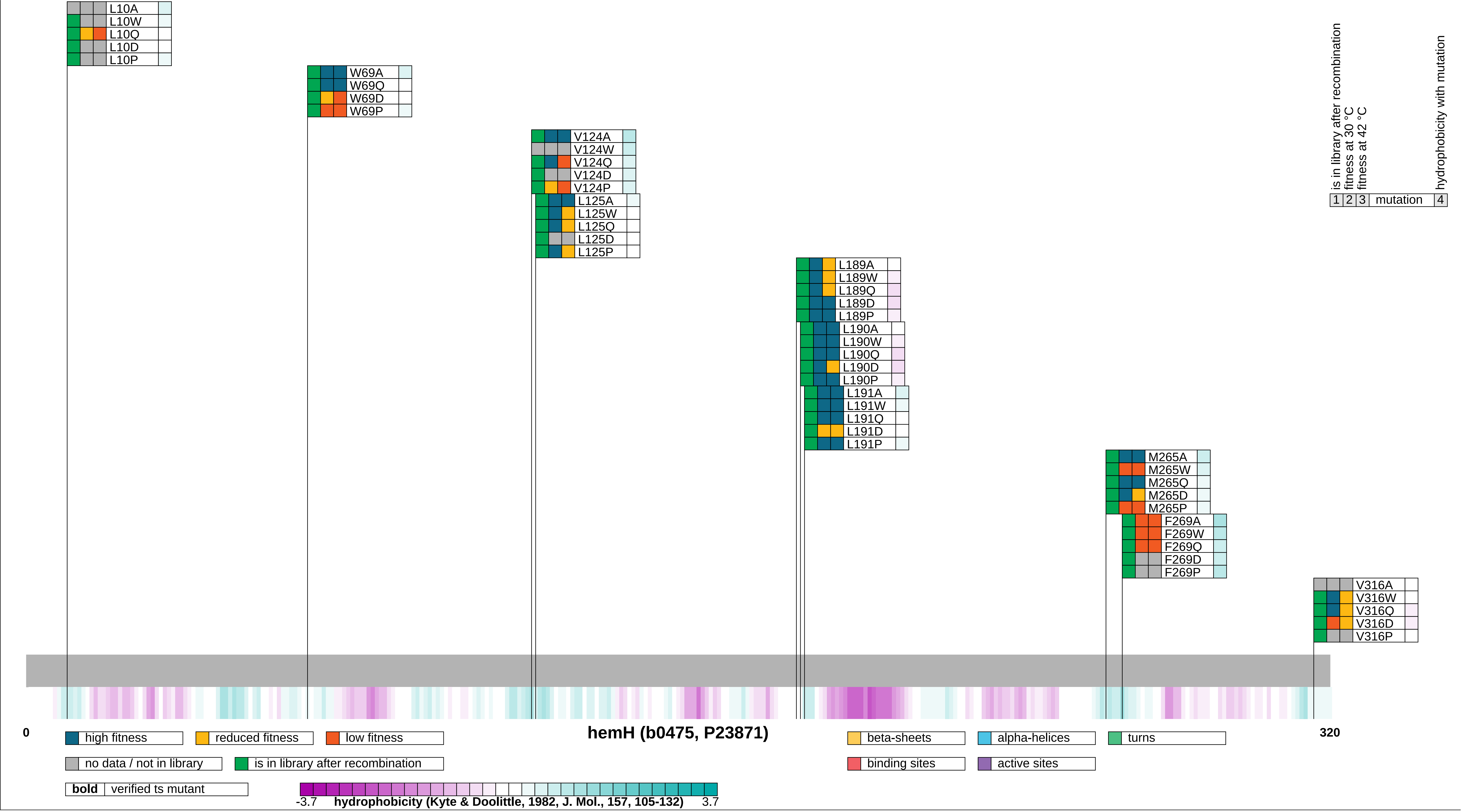

L10A
L10W
L10Q
L10D
L10P
W69A
W69Q
W69D
W69P
V124A
V124W
V124Q
V124D
V124P
L125A
L125W
L125Q
L125D
L125P
L189A
L189W
L189Q
L189D
L189P
L190A
L190W
L190Q
L190D
L190P
L191A
L191W
L191Q
L191D
L191P
M265A
M265W
M265Q
M265D
M265P
F269A
F269W
F269Q
F269D
F269P
V316A
V316W
V316Q
V316D
V316P
is in library after recombination
fitness at 30 °C
fitness at 42 °C
hydrophobicity with mutation
1 2 3 mutation 4
0
320
hemH (b0475, P23871)
high fitness
reduced fitness
low fitness
no data / not in library
is in library after recombination
bold verified ts mutant
beta-sheets
alpha-helices
turns
binding sites
active sites
-3.7
hydrophobicity (Kyte & Doolittle, 1982, J. Mol., 157, 105-132)
3.7

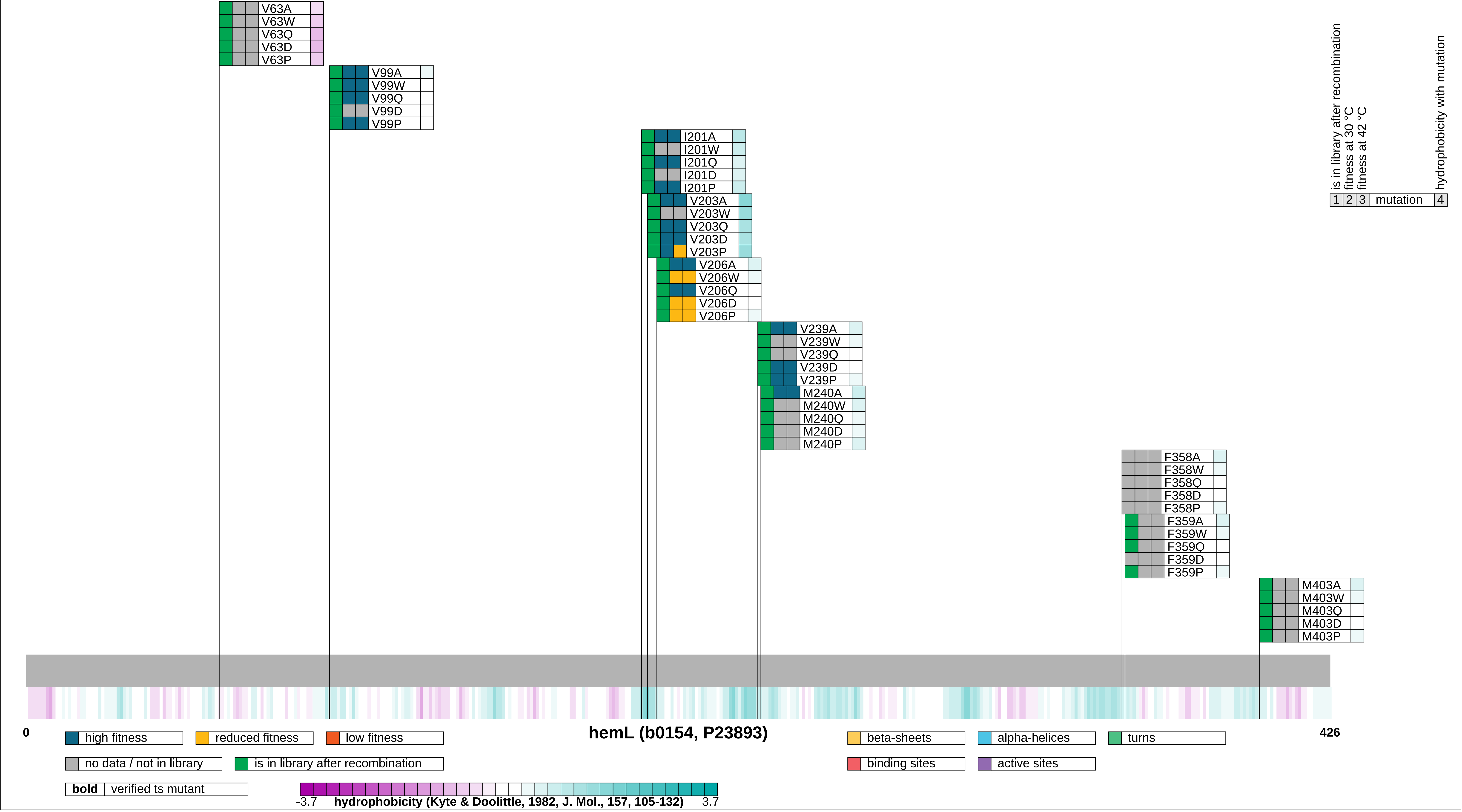

V63A
V63W
V63Q
V63D
V63P
V99A
V99W
V99Q
V99D
V99P
I201A
I201W
I201Q
I201D
I201P
V203A
V203W
V203Q
V203D
V203P
V206A
V206W
V206Q
V206D
V206P
V239A
V239W
V239Q
V239D
V239P
M240A
M240W
M240Q
M240D
M240P
F358A
F358W
F358Q
F358D
F358P
F359A
F359W
F359Q
F359D
F359P
M403A
M403W
M403Q
M403D
M403P
is in library after recombination
fitness at 30 °C
fitness at 42 °C
hydrophobicity with mutation
1
2
3
mutation
4
0
426
hemL (b0154, P23893)
high fitness
reduced fitness
low fitness
beta-sheets
alpha-helices
turns
no data / not in library
is in library after recombination
binding sites
active sites
bold
verified ts mutant
-3.7
hydrophobicity (Kyte & Doolittle, 1982, J. Mol., 157, 105-132)
3.7

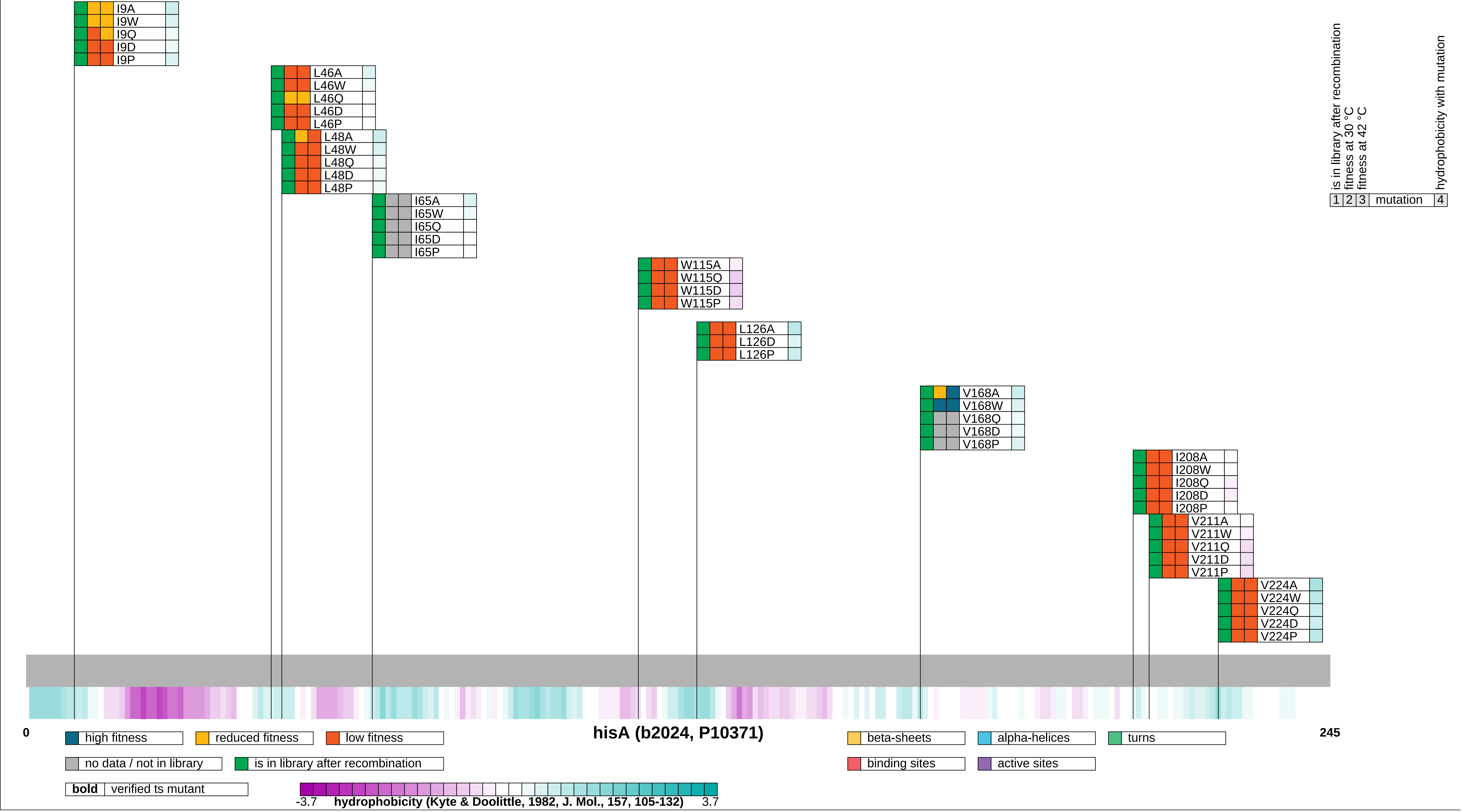

I9A
I9W
I9Q
I9D
I9P
L46A
L46W
L46Q
L46D
L46P
L48A
L48W
L48Q
L48D
L48P
I65A
I65W
I65Q
I65D
I65P
W115A
W115Q
W115D
W115P
L126A
L126D
L126P
V168A
V168W
V168Q
V168D
V168P
I208A
I208W
I208Q
I208D
I208P
V211A
V211W
V211Q
V211D
V211P
V224A
V224W
V224Q
V224D
V224P
is in library after recombination
fitness at 30 °C
fitness at 42 °C
hydrophobicity with mutation
1 2 3 mutation 4
0
hisA (b2024, P10371)
245
high fitness
reduced fitness
low fitness
beta-sheets
alpha-helices
turns
no data / not in library
is in library after recombination
binding sites
active sites
bold verified ts mutant
-3.7 hydrophobicity (Kyte & Doolittle, 1982, J. Mol., 157, 105-132) 3.7

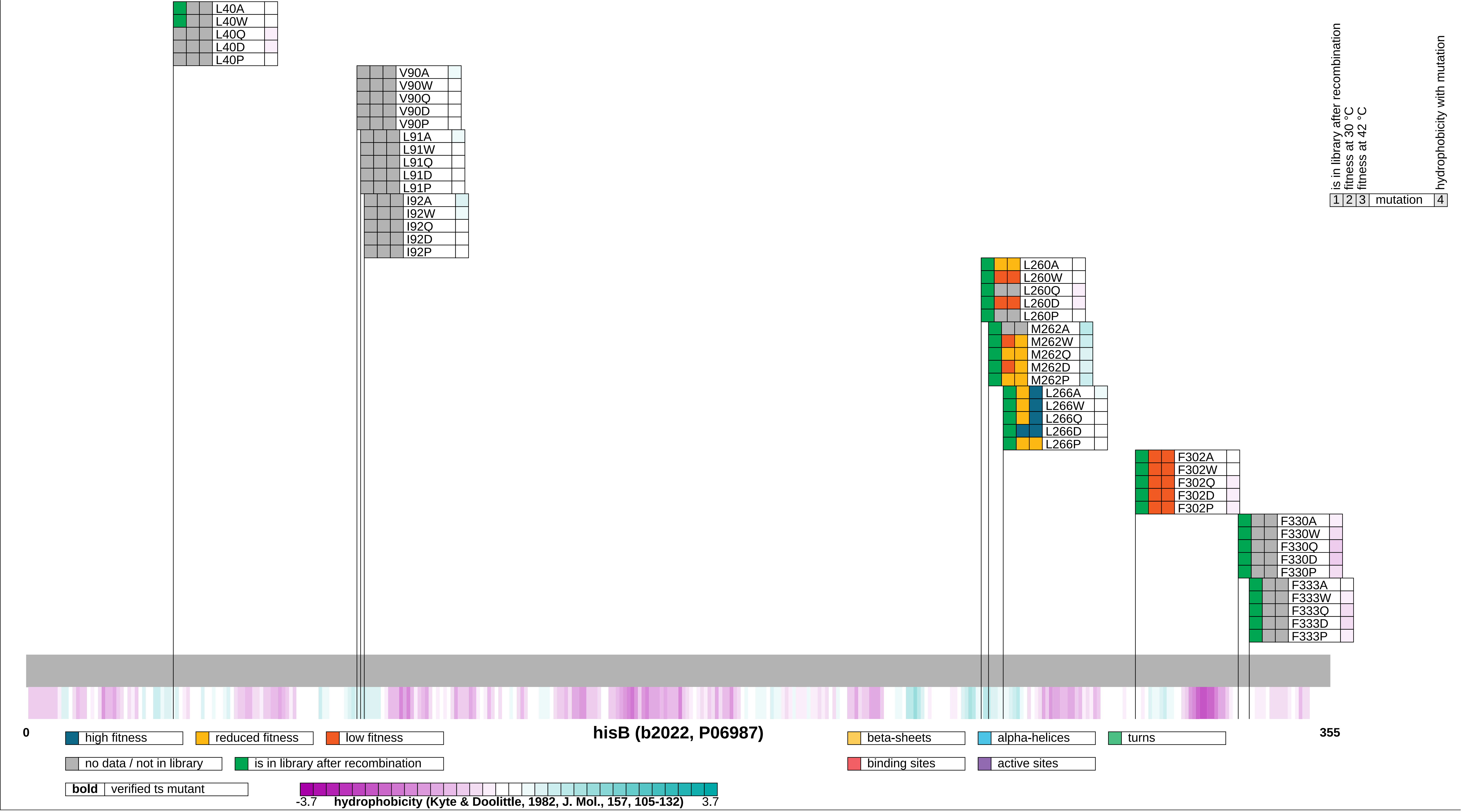

L40A
L40W
L40Q
L40D
L40P
V90A
V90W
V90Q
V90D
V90P
L91A
L91W
L91Q
L91D
L91P
I92A
I92W
I92Q
I92D
I92P
L260A
L260W
L260Q
L260D
L260P
M262A
M262W
M262Q
M262D
M262P
L266A
L266W
L266Q
L266D
L266P
F302A
F302W
F302Q
F302D
F302P
F330A
F330W
F330Q
F330D
F330P
F333A
F333W
F333Q
F333D
F333P
is in library after recombination
fitness at 30 °C
fitness at 42 °C
hydrophobicity with mutation
1 2 3 mutation 4
0
hisB (b2022, P06987)
355
high fitness
reduced fitness
low fitness
no data / not in library
is in library after recombination
bold verified ts mutant
beta-sheets
alpha-helices
turns
binding sites
active sites
-3.7 hydrophobicity (Kyte & Doolittle, 1982, J. Mol., 157, 105-132) 3.7

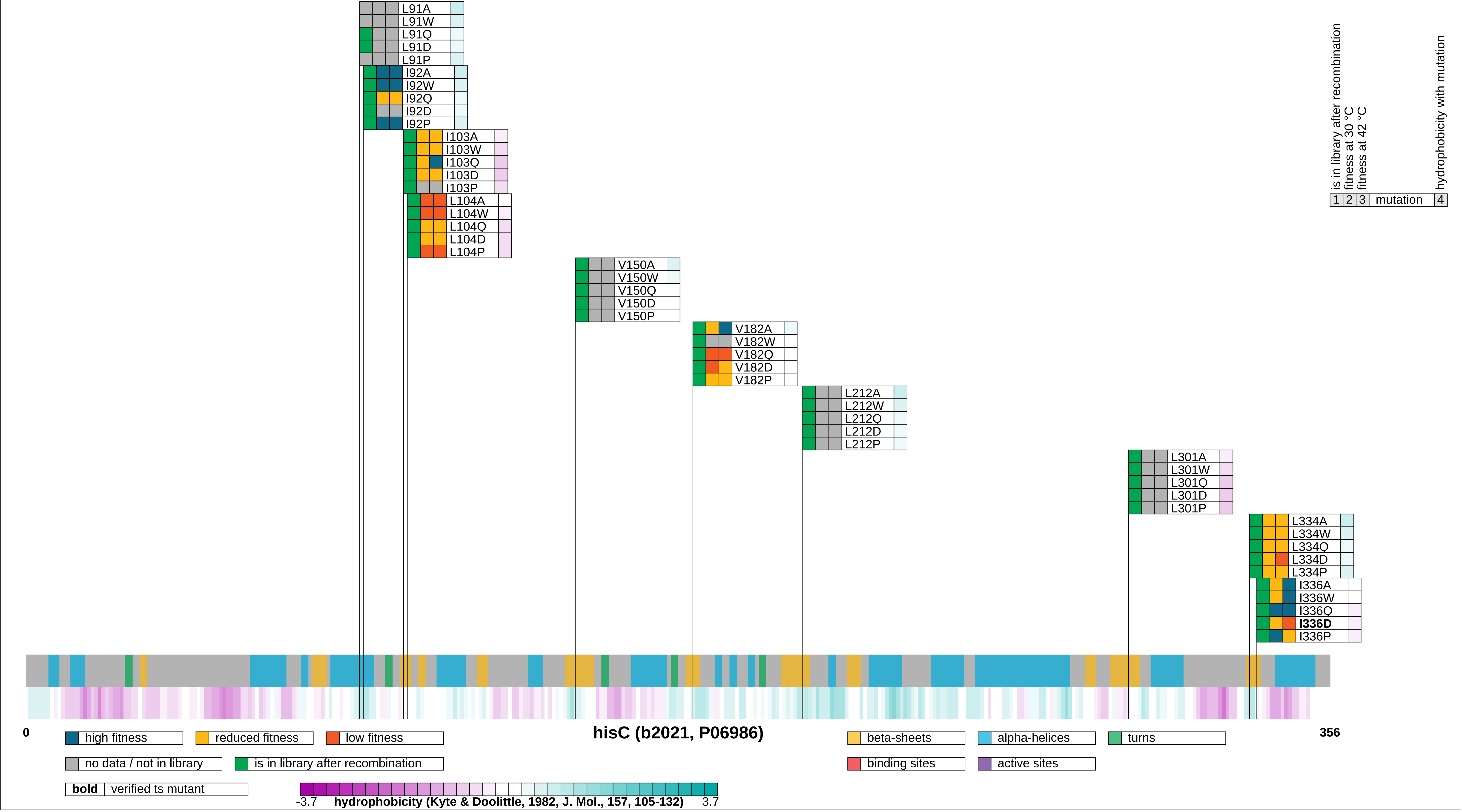

L91A
L91W
L91Q
L91D
L91P
I92A
I92W
I92Q
I92D
I92P
I103A
I103W
I103Q
I103D
I103P
L104A
L104W
L104Q
L104D
L104P
V150A
V150W
V150Q
V150D
V150P
V182A
V182W
V182Q
V182D
V182P
L212A
L212W
L212Q
L212D
L212P
L301A
L301W
L301Q
L301D
L301P
L334A
L334W
L334Q
L334D
L334P
I336A
I336W
I336Q
I336D
I336P
1 is in library after recombination
2 fitness at 30 °C
3 fitness at 42 °C
mutation
4 hydrophobicity with mutation
0
356
hisC (b2021, P06986)
high fitness
reduced fitness
low fitness
no data / not in library
is in library after recombination
bold verified ts mutant
beta-sheets
alpha-helices
turns
binding sites
active sites
-3.7
hydrophobicity (Kyte & Doolittle, 1982, J. Mol., 157, 105-132)
3.7

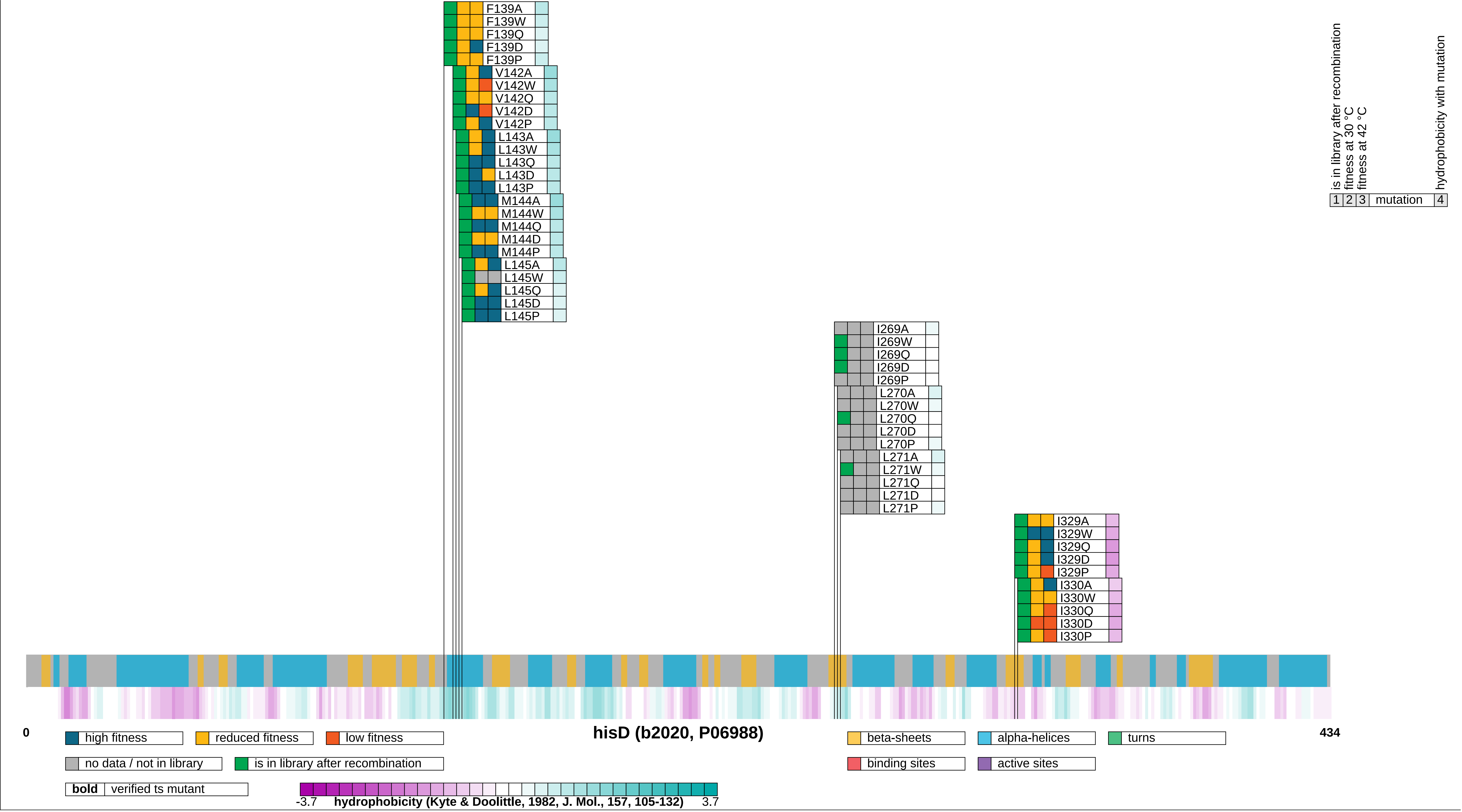

F139A
F139W
F139Q
F139D
F139P
V142A
V142W
V142Q
V142D
V142P
L143A
L143W
L143Q
L143D
L143P
M144A
M144W
M144Q
M144D
M144P
L145A
L145W
L145Q
L145D
L145P
I269A
I269W
I269Q
I269D
I269P
L270A
L270W
L270Q
L270D
L270P
L271A
L271W
L271Q
L271D
L271P
I329A
I329W
I329Q
I329D
I329P
I330A
I330W
I330Q
I330D
I330P
is in library after recombination
fitness at 30 °C
fitness at 42 °C
hydrophobicity with mutation
1 2 3 mutation 4
0
hisD (b2020, P06988)
434
high fitness
reduced fitness
low fitness
beta-sheets
alpha-helices
turns
no data / not in library
is in library after recombination
binding sites
active sites
bold verified ts mutant
-3.7
hydrophobicity (Kyte & Doolittle, 1982, J. Mol., 157, 105-132)
3.7

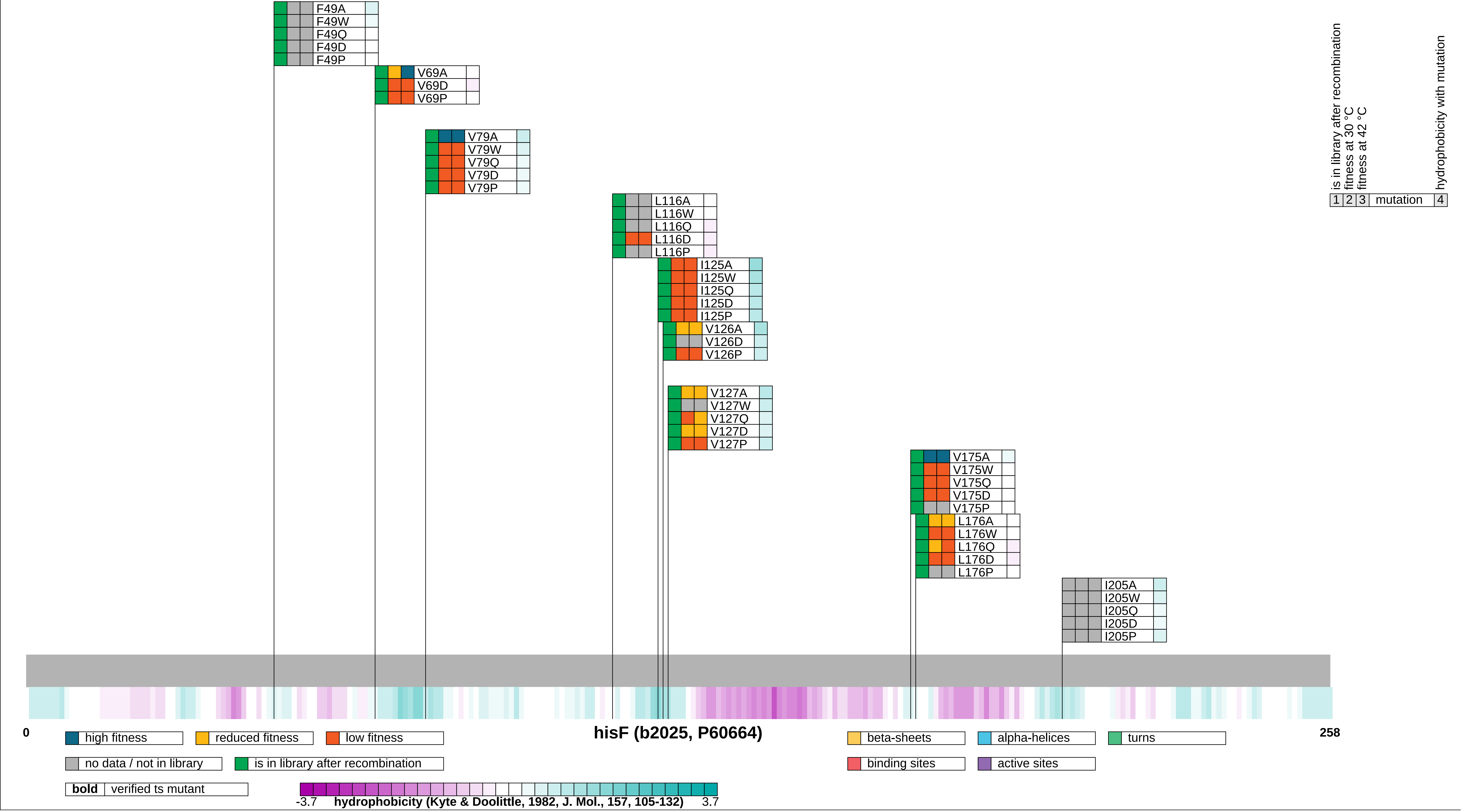

F49A
F49W
F49Q
F49D
F49P
V69A
V69D
V69P
V79A
V79W
V79Q
V79D
V79P
L116A
L116W
L116Q
L116D
L116P
I125A
I125W
I125Q
I125D
I125P
V126A
V126D
V126P
V127A
V127W
V127Q
V127D
V127P
V175A
V175W
V175Q
V175D
V175P
L176A
L176W
L176Q
L176D
L176P
I205A
I205W
I205Q
I205D
I205P
is in library after recombination
fitness at 30 °C
fitness at 42 °C
hydrophobicity with mutation
1 2 3 mutation 4
0
hisF (b2025, P60664)
258
high fitness
reduced fitness
low fitness
no data / not in library
is in library after recombination
bold verified ts mutant
beta-sheets
alpha-helices
turns
binding sites
active sites
-3.7
hydrophobicity (Kyte & Doolittle, 1982, J. Mol., 157, 105-132)
3.7

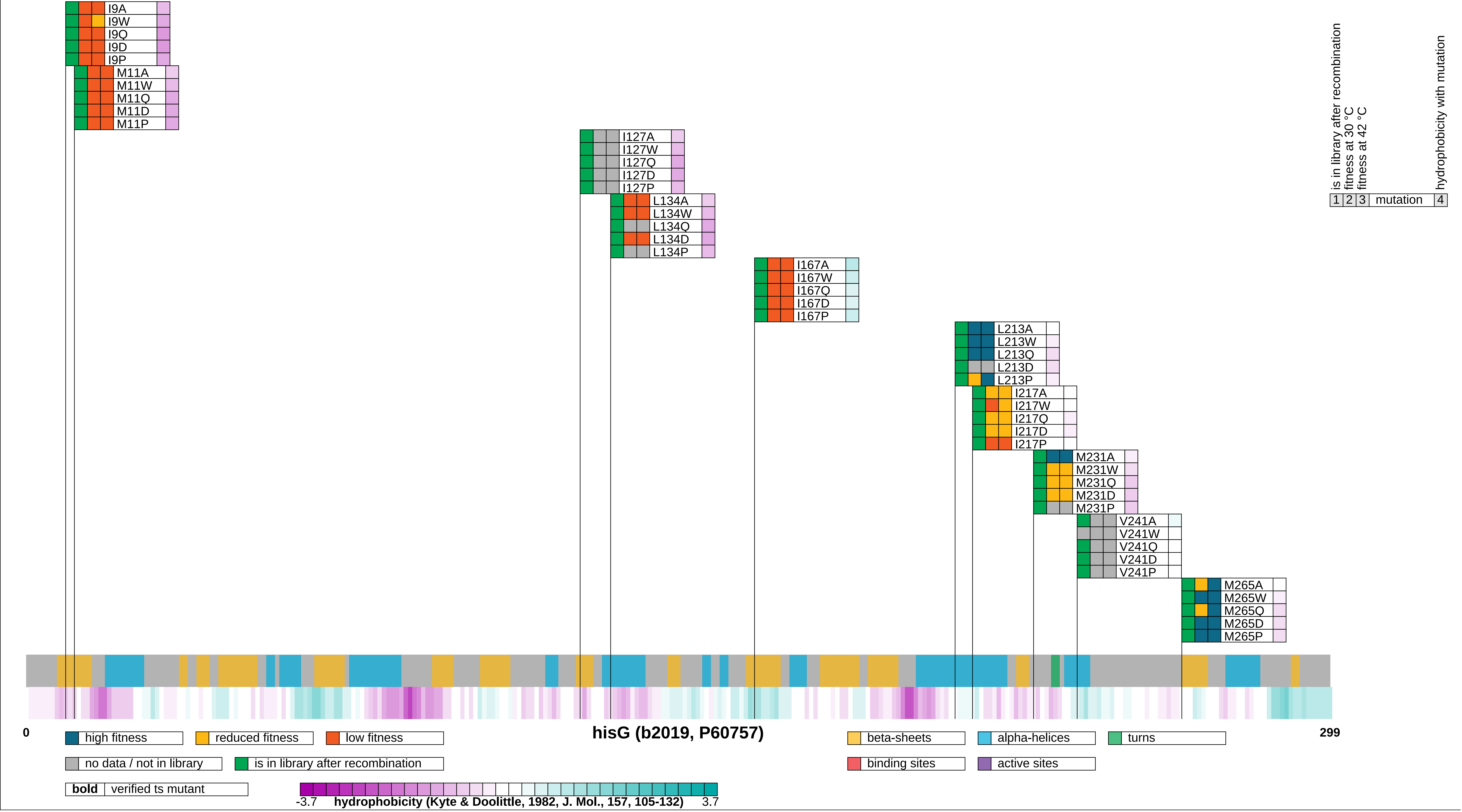
is in library after recombination
fitness at 30 °C
fitness at 42 °C
hydrophobicity with mutation
1 2 3 mutation 4
I9A
I9W
I9Q
I9D
I9P
M11A
M11W
M11Q
M11D
M11P
I127A
I127W
I127Q
I127D
I127P
L134A
L134W
L134Q
L134D
L134P
I167A
I167W
I167Q
I167D
I167P
L213A
L213W
L213Q
L213D
L213P
I217A
I217W
I217Q
I217D
I217P
M231A
M231W
M231Q
M231D
M231P
V241A
V241W
V241Q
V241D
V241P
M265A
M265W
M265Q
M265D
M265P
0
hisG (b2019, P60757)
299
high fitness
reduced fitness
low fitness
beta-sheets
alpha-helices
turns
no data / not in library
is in library after recombination
binding sites
active sites
bold verified ts mutant
-3.7 hydrophobicity (Kyte & Doolittle, 1982, J. Mol., 157, 105-132) 3.7

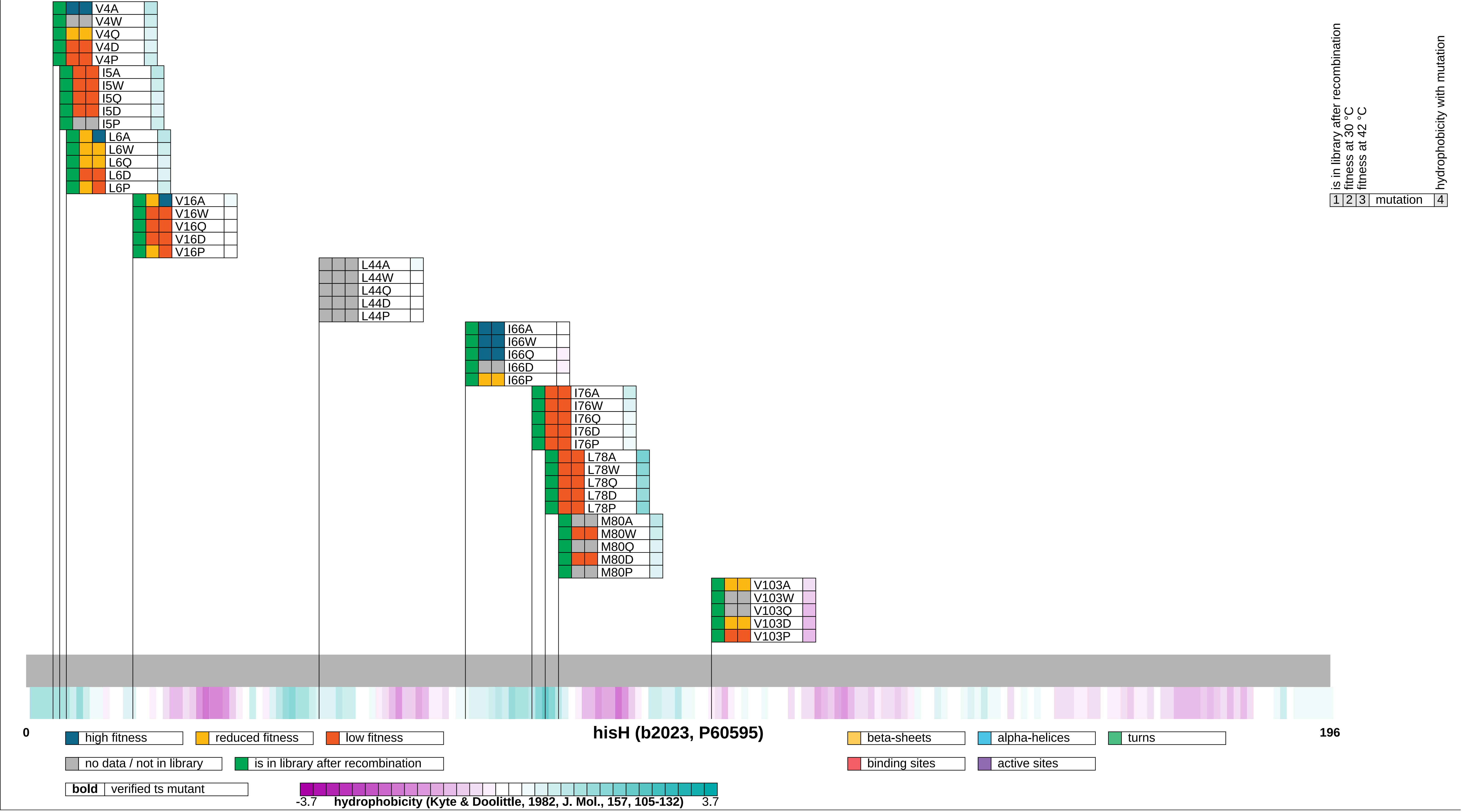

V4A
V4W
V4Q
V4D
V4P
I5A
I5W
I5Q
I5D
I5P
L6A
L6W
L6Q
L6D
L6P
V16A
V16W
V16Q
V16D
V16P
L44A
L44W
L44Q
L44D
L44P
I66A
I66W
I66Q
I66D
I66P
I76A
I76W
I76Q
I76D
I76P
L78A
L78W
L78Q
L78D
L78P
M80A
M80W
M80Q
M80D
M80P
V103A
V103W
V103Q
V103D
V103P
is in library after recombination
fitness at 30 °C
fitness at 42 °C
hydrophobicity with mutation
1
2
3
mutation
4
0
hisH (b2023, P60595)
196
high fitness
reduced fitness
low fitness
beta-sheets
alpha-helices
turns
no data / not in library
is in library after recombination
binding sites
active sites
bold
verified ts mutant
-3.7
hydrophobicity (Kyte & Doolittle, 1982, J. Mol., 157, 105-132)
3.7

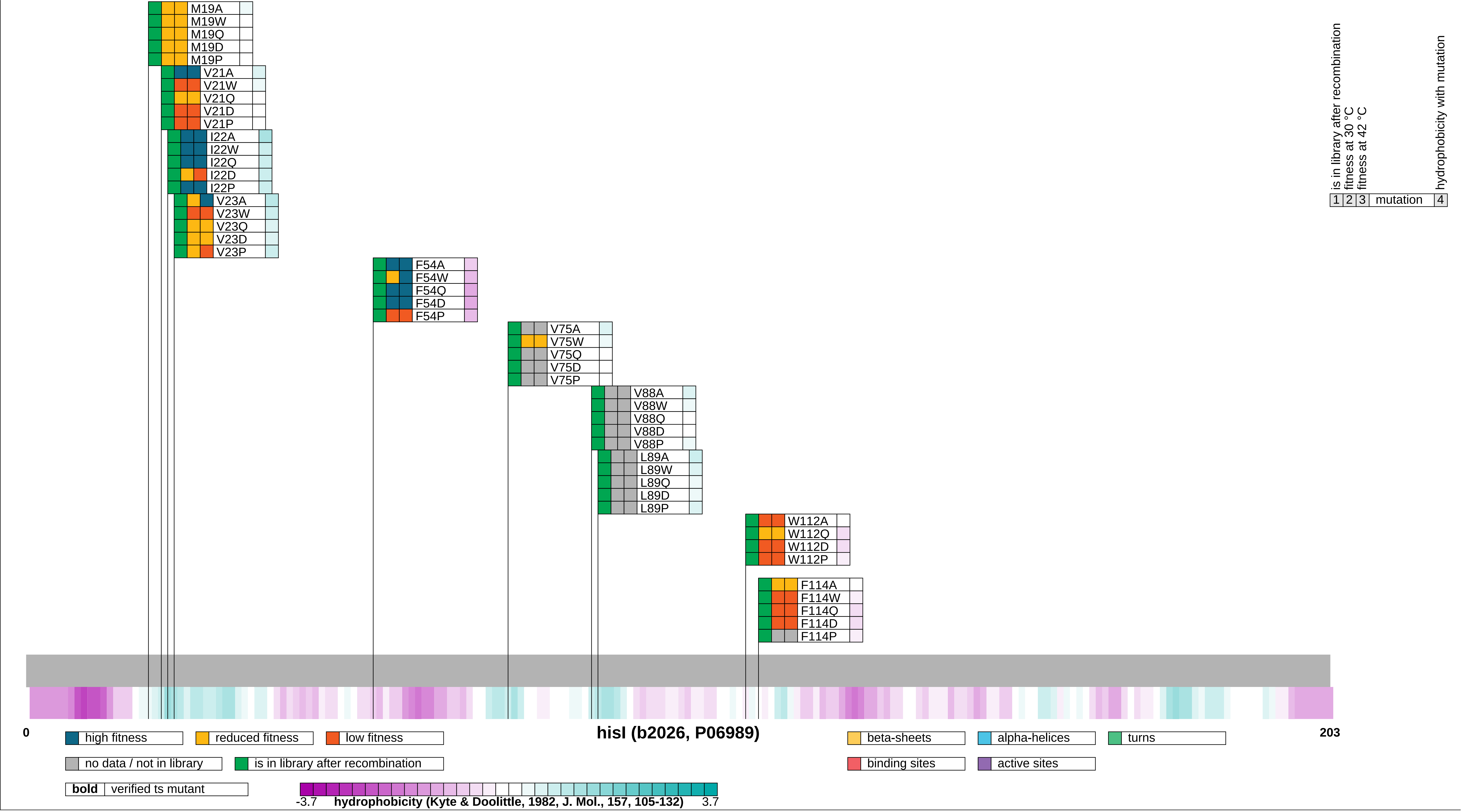
M19A
M19W
M19Q
M19D
M19P
V21A
V21W
V21Q
V21D
V21P
I22A
I22W
I22Q
I22D
I22P
V23A
V23W
V23Q
V23D
V23P
F54A
F54W
F54Q
F54D
F54P
V75A
V75W
V75Q
V75D
V75P
V88A
V88W
V88Q
V88D
V88P
L89A
L89W
L89Q
L89D
L89P
W112A
W112Q
W112D
W112P
F114A
F114W
F114Q
F114D
F114P
is in library after recombination
fitness at 30 °C
fitness at 42 °C
hydrophobicity with mutation
1 2 3 mutation 4
0
203
hisI (b2026, P06989)
high fitness
reduced fitness
low fitness
no data / not in library
is in library after recombination
bold verified ts mutant
beta-sheets
alpha-helices
turns
binding sites
active sites
-3.7 hydrophobicity (Kyte & Doolittle, 1982, J. Mol., 157, 105-132) 3.7

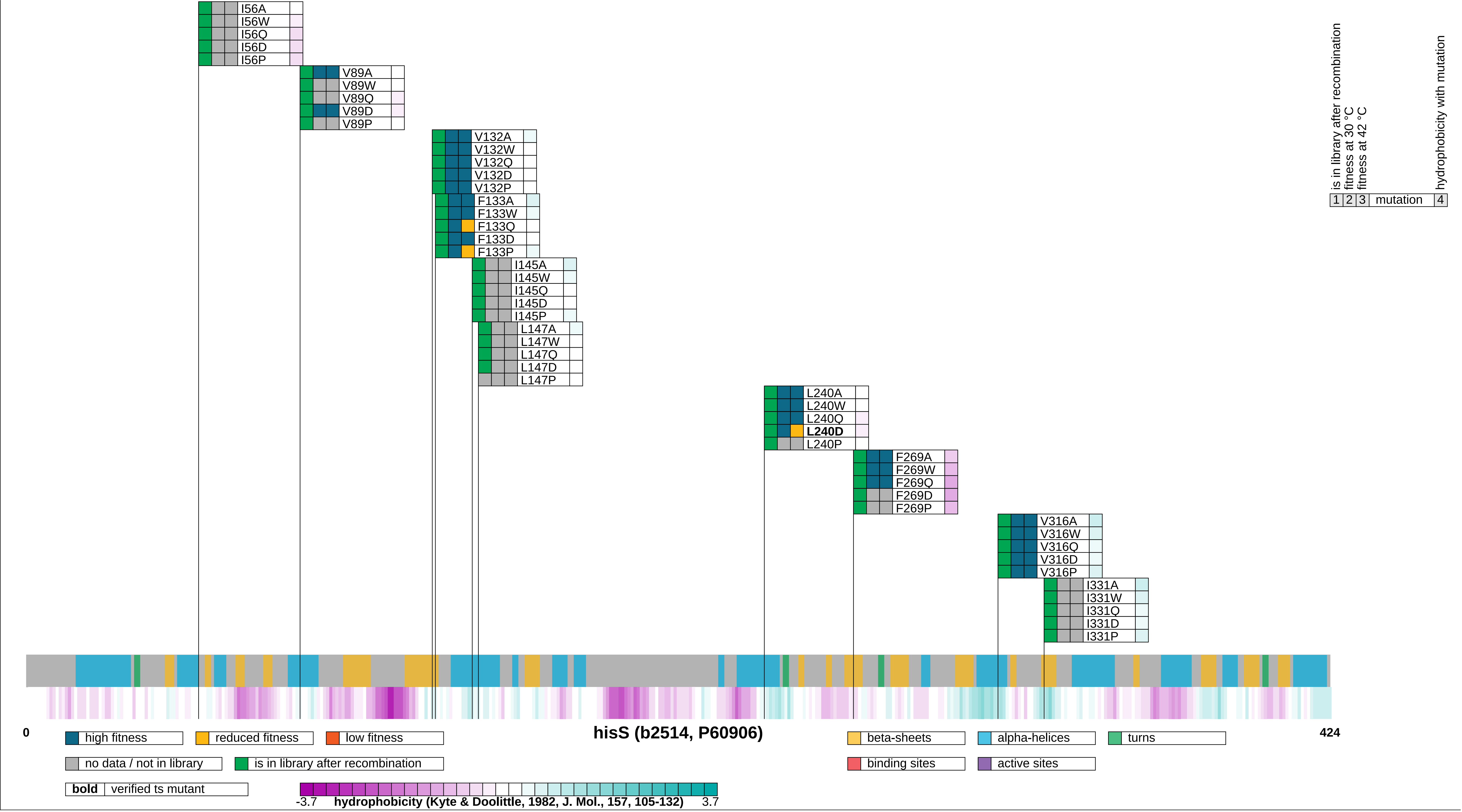
I56A
I56W
I56Q
I56D
I56P
V89A
V89W
V89Q
V89D
V89P
V132A
V132W
V132Q
V132D
V132P
F133A
F133W
F133Q
F133D
F133P
I145A
I145W
I145Q
I145D
I145P
L147A
L147W
L147Q
L147D
L147P
L240A
L240W
L240Q
L240D
L240P
F269A
F269W
F269Q
F269D
F269P
V316A
V316W
V316Q
V316D
V316P
I331A
I331W
I331Q
I331D
I331P
is in library after recombination
fitness at 30 °C
fitness at 42 °C
hydrophobicity with mutation
1
2
3
mutation
4
0
hisS (b2514, P60906)
424
high fitness
reduced fitness
low fitness
beta-sheets
alpha-helices
turns
no data / not in library
is in library after recombination
binding sites
active sites
bold
verified ts mutant
-3.7
hydrophobicity (Kyte & Doolittle, 1982, J. Mol., 157, 105-132)
3.7

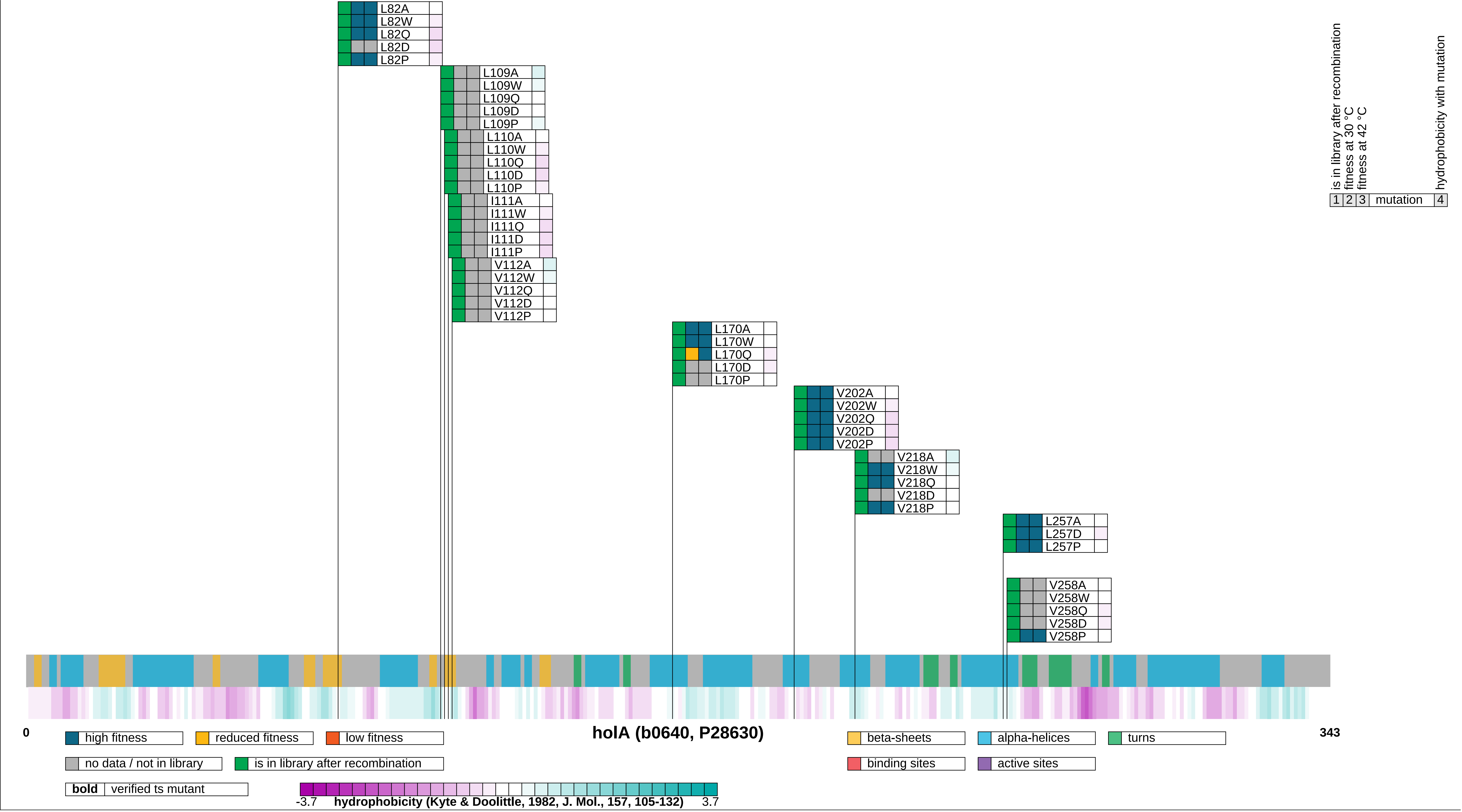

is in library after recombination
fitness at 30 °C
fitness at 42 °C
hydrophobicity with mutation
1
2
3
mutation
4
L82A
L82W
L82Q
L82D
L82P
L109A
L109W
L109Q
L109D
L109P
L110A
L110W
L110Q
L110D
L110P
I111A
I111W
I111Q
I111D
I111P
V112A
V112W
V112Q
V112D
V112P
L170A
L170W
L170Q
L170D
L170P
V202A
V202W
V202Q
V202D
V202P
V218A
V218W
V218Q
V218D
V218P
L257A
L257D
L257P
V258A
V258W
V258Q
V258D
V258P
0
holA (b0640, P28630)
343
high fitness
reduced fitness
low fitness
beta-sheets
alpha-helices
turns
no data / not in library
is in library after recombination
binding sites
active sites
bold
verified ts mutant
-3.7
hydrophobicity (Kyte & Doolittle, 1982, J. Mol., 157, 105-132)
3.7

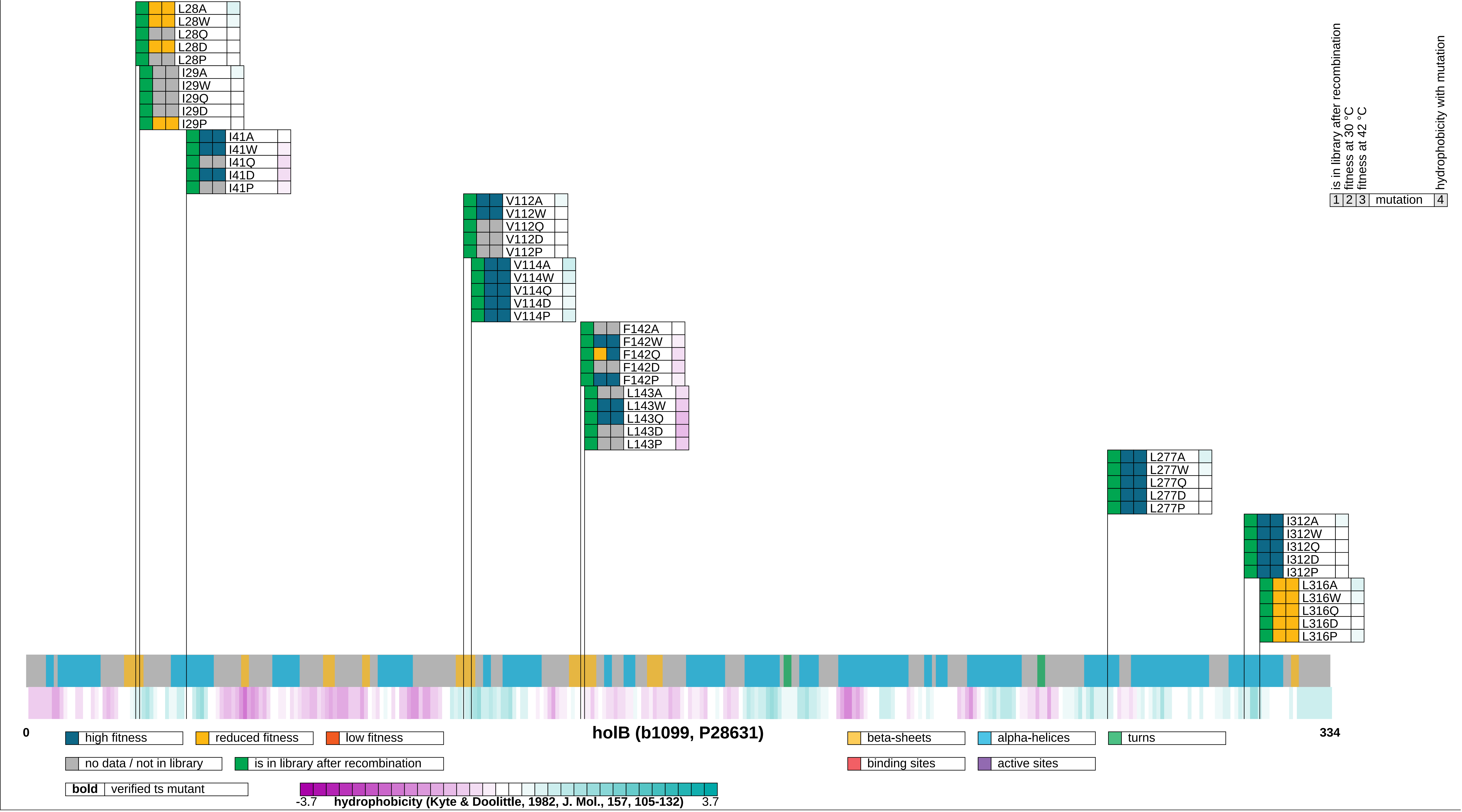
L28A
L28W
L28Q
L28D
L28P
I29A
I29W
I29Q
I29D
I29P
I41A
I41W
I41Q
I41D
I41P
V112A
V112W
V112Q
V112D
V112P
V114A
V114W
V114Q
V114D
V114P
F142A
F142W
F142Q
F142D
F142P
L143A
L143W
L143Q
L143D
L143P
L277A
L277W
L277Q
L277D
L277P
I312A
I312W
I312Q
I312D
I312P
L316A
L316W
L316Q
L316D
L316P
is in library after recombination
fitness at 30 °C
fitness at 42 °C
hydrophobicity with mutation
1 2 3 mutation 4
0
holB (b1099, P28631)
334
high fitness
reduced fitness
low fitness
beta-sheets
alpha-helices
turns
no data / not in library
is in library after recombination
binding sites
active sites
bold verified ts mutant
-3.7 hydrophobicity (Kyte & Doolittle, 1982, J. Mol., 157, 105-132) 3.7

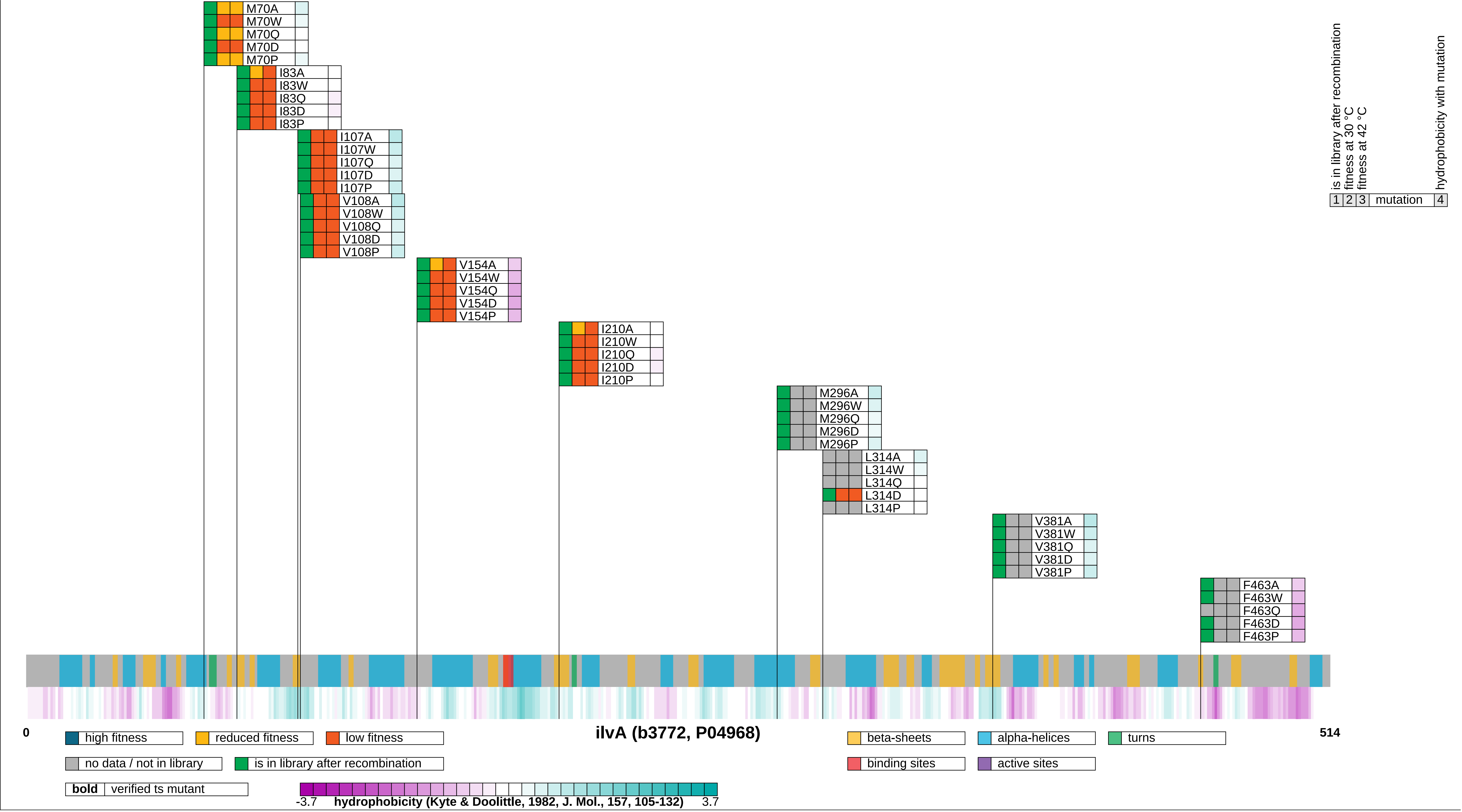

is in library after recombination
fitness at 30 °C
fitness at 42 °C
hydrophobicity with mutation
1 2 3 mutation 4
M70A
M70W
M70Q
M70D
M70P
I83A
I83W
I83Q
I83D
I83P
I107A
I107W
I107Q
I107D
I107P
V108A
V108W
V108Q
V108D
V108P
V154A
V154W
V154Q
V154D
V154P
I210A
I210W
I210Q
I210D
I210P
M296A
M296W
M296Q
M296D
M296P
L314A
L314W
L314Q
L314D
L314P
V381A
V381W
V381Q
V381D
V381P
F463A
F463W
F463Q
F463D
F463P
0
ilvA (b3772, P04968)
514
high fitness
reduced fitness
low fitness
beta-sheets
alpha-helices
turns
no data / not in library
is in library after recombination
binding sites
active sites
bold verified ts mutant
-3.7 hydrophobicity (Kyte & Doolittle, 1982, J. Mol., 157, 105-132) 3.7

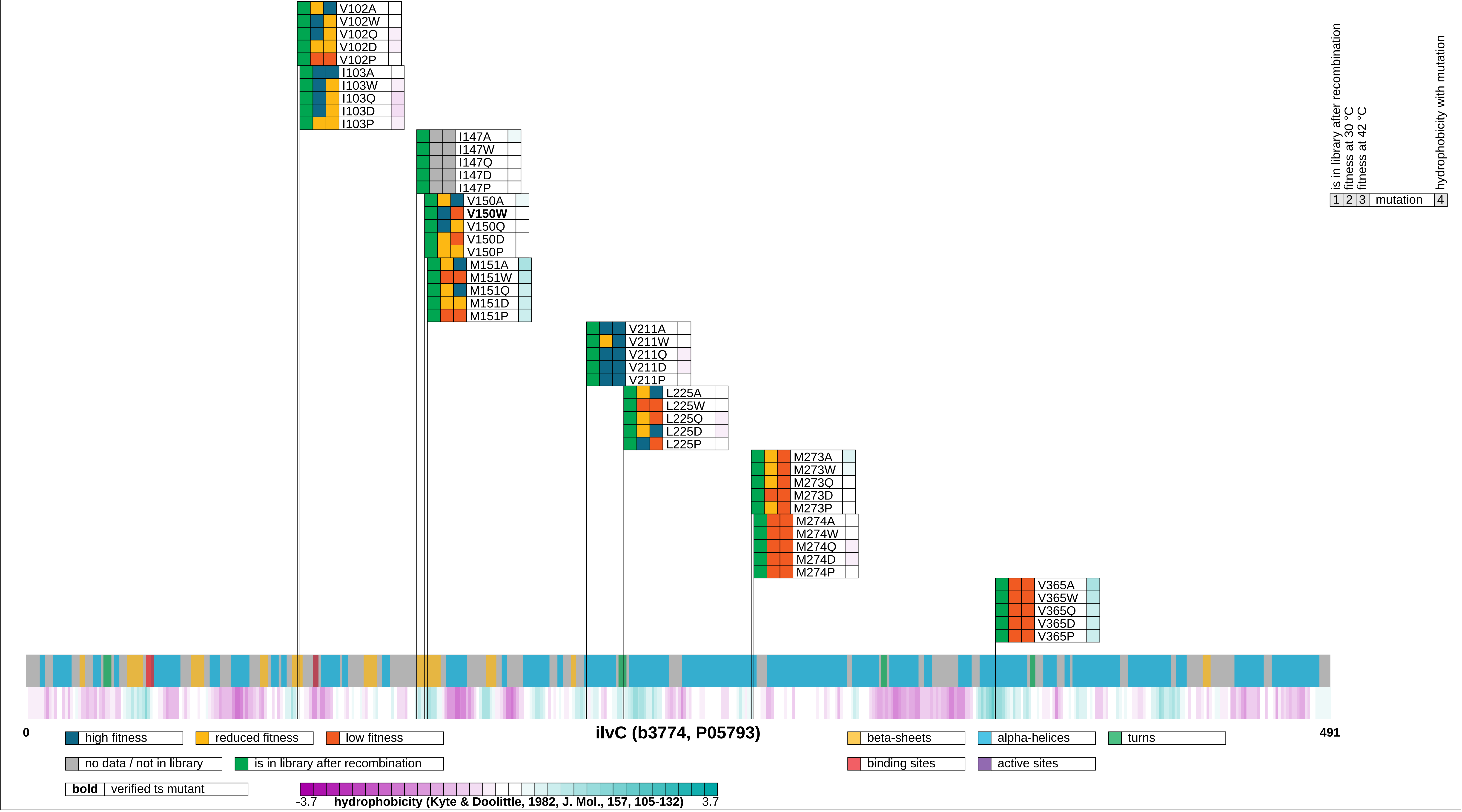

V102A
V102W
V102Q
V102D
V102P
I103A
I103W
I103Q
I103D
I103P
I147A
I147W
I147Q
I147D
I147P
V150A
V150W
V150Q
V150D
V150P
M151A
M151W
M151Q
M151D
M151P
V211A
V211W
V211Q
V211D
V211P
L225A
L225W
L225Q
L225D
L225P
M273A
M273W
M273Q
M273D
M273P
M274A
M274W
M274Q
M274D
M274P
V365A
V365W
V365Q
V365D
V365P
is in library after recombination
fitness at 30 °C
fitness at 42 °C
hydrophobicity with mutation
1 2 3 mutation 4
0
ilvC (b3774, P05793)
491
high fitness
reduced fitness
low fitness
beta-sheets
alpha-helices
turns
no data / not in library
is in library after recombination
binding sites
active sites
bold verified ts mutant
-3.7
hydrophobicity (Kyte & Doolittle, 1982, J. Mol., 157, 105-132)
3.7

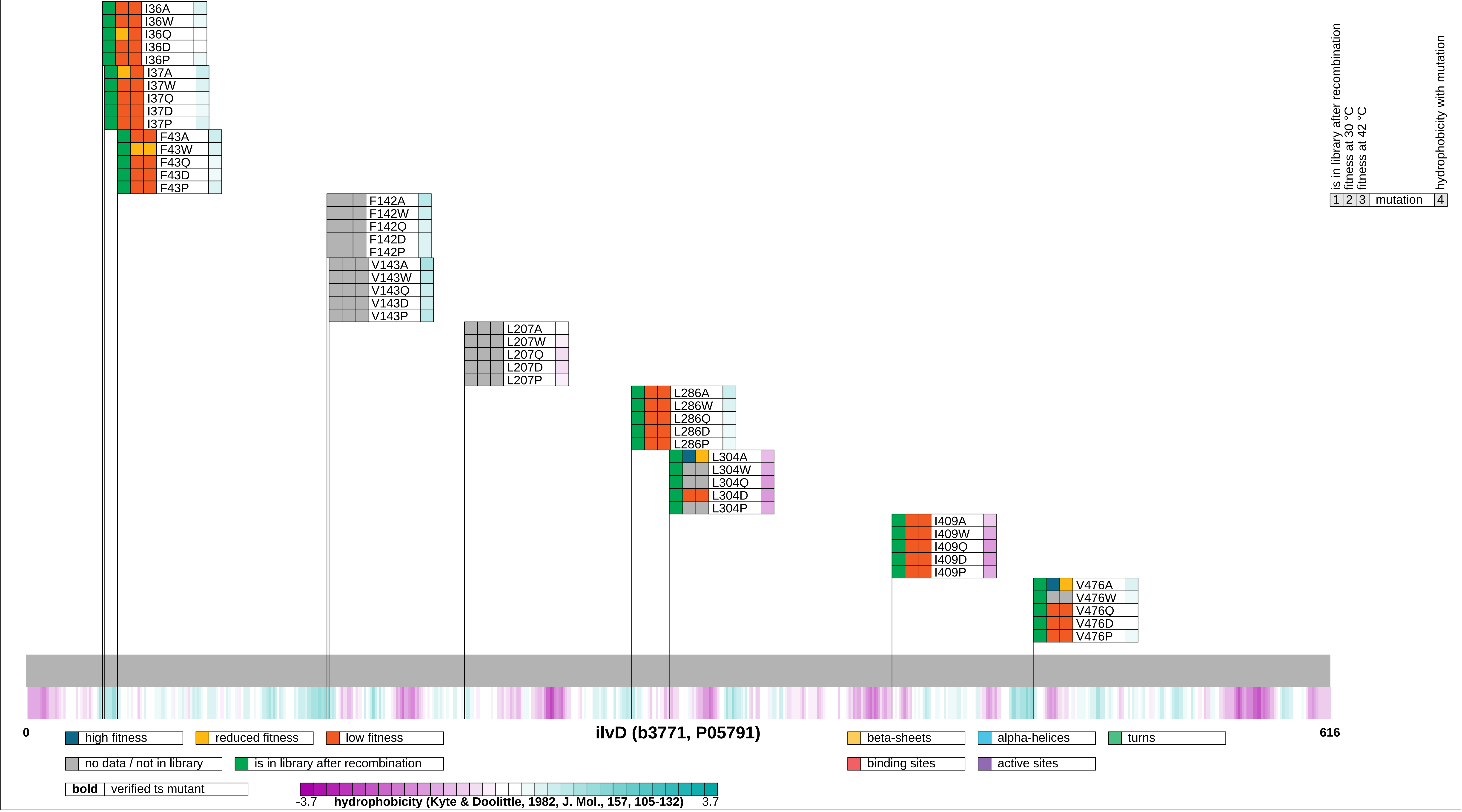

I36A
I36W
I36Q
I36D
I36P
I37A
I37W
I37Q
I37D
I37P
F43A
F43W
F43Q
F43D
F43P
F142A
F142W
F142Q
F142D
F142P
V143A
V143W
V143Q
V143D
V143P
L207A
L207W
L207Q
L207D
L207P
L286A
L286W
L286Q
L286D
L286P
L304A
L304W
L304Q
L304D
L304P
I409A
I409W
I409Q
I409D
I409P
V476A
V476W
V476Q
V476D
V476P
is in library after recombination
fitness at 30 °C
fitness at 42 °C
hydrophobicity with mutation
1
2
3
mutation
4
0
ilvD (b3771, P05791)
616
high fitness
reduced fitness
low fitness
no data / not in library
is in library after recombination
bold
verified ts mutant
beta-sheets
alpha-helices
turns
binding sites
active sites
-3.7
hydrophobicity (Kyte & Doolittle, 1982, J. Mol., 157, 105-132)
3.7

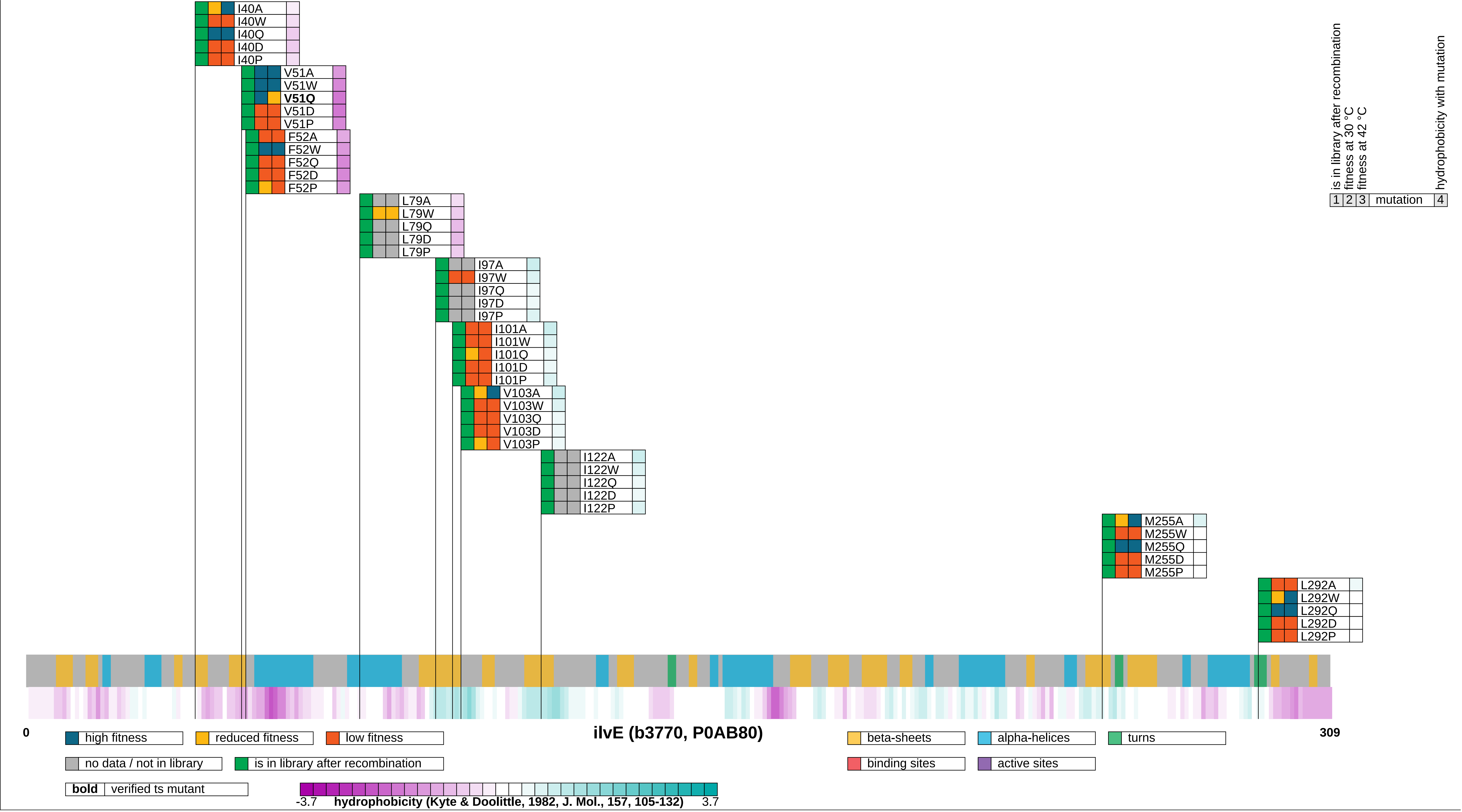

I40A
I40W
I40Q
I40D
I40P
V51A
V51W
V51Q
V51D
V51P
F52A
F52W
F52Q
F52D
F52P
L79A
L79W
L79Q
L79D
L79P
I97A
I97W
I97Q
I97D
I97P
I101A
I101W
I101Q
I101D
I101P
V103A
V103W
V103Q
V103D
V103P
I122A
I122W
I122Q
I122D
I122P
M255A
M255W
M255Q
M255D
M255P
L292A
L292W
L292Q
L292D
L292P
is in library after recombination
fitness at 30 °C
fitness at 42 °C
hydrophobicity with mutation
1 2 3 mutation 4
0
ilvE (b3770, P0AB80)
309
high fitness
reduced fitness
low fitness
beta-sheets
alpha-helices
turns
no data / not in library
is in library after recombination
binding sites
active sites
bold verified ts mutant
-3.7
hydrophobicity (Kyte & Doolittle, 1982, J. Mol., 157, 105-132)
3.7

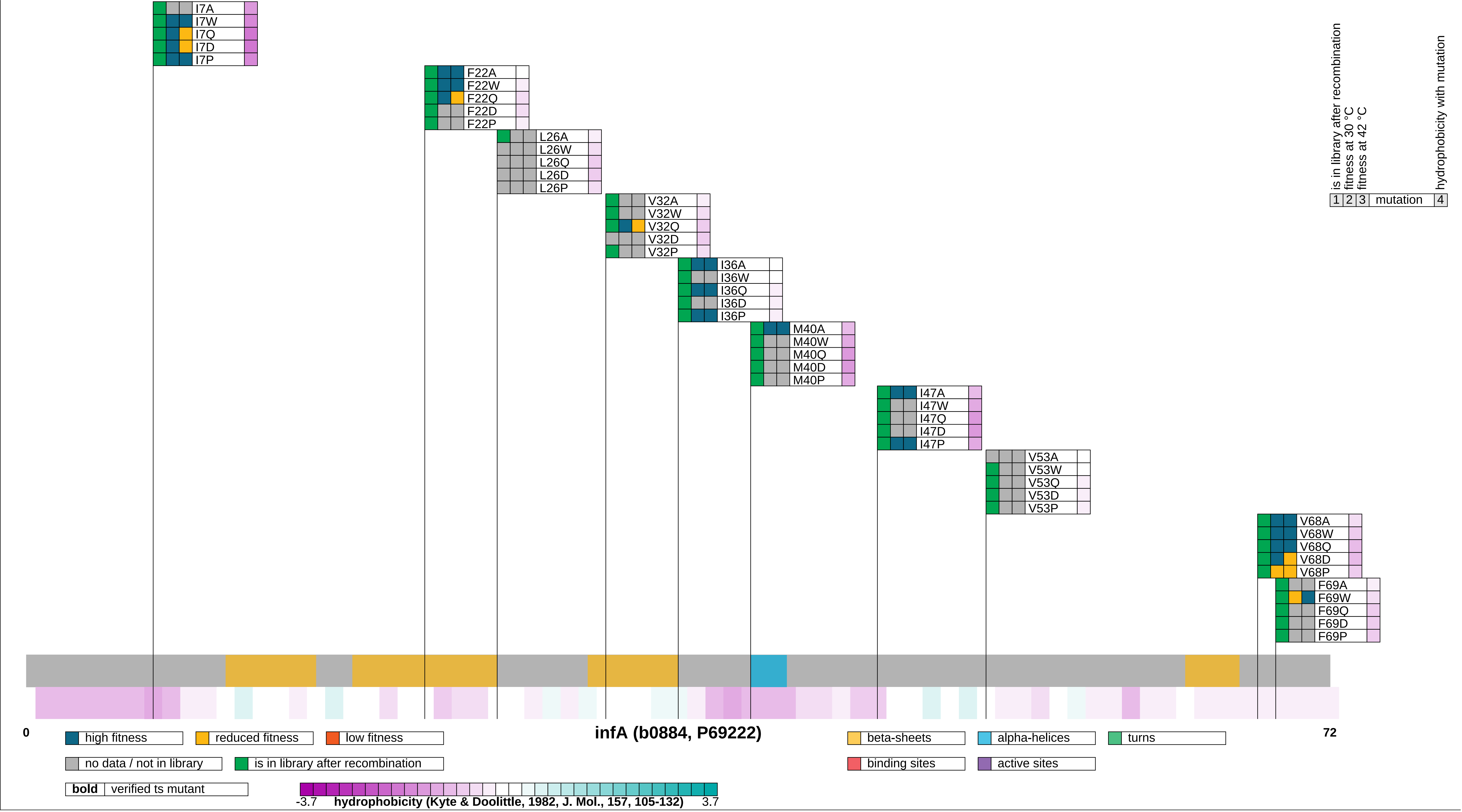

I7A
I7W
I7Q
I7D
I7P
F22A
F22W
F22Q
F22D
F22P
L26A
L26W
L26Q
L26D
L26P
V32A
V32W
V32Q
V32D
V32P
I36A
I36W
I36Q
I36D
I36P
M40A
M40W
M40Q
M40D
M40P
I47A
I47W
I47Q
I47D
I47P
V53A
V53W
V53Q
V53D
V53P
V68A
V68W
V68Q
V68D
V68P
F69A
F69W
F69Q
F69D
F69P
is in library after recombination
fitness at 30 °C
fitness at 42 °C
hydrophobicity with mutation
1
2
3
mutation
4
0
infA (b0884, P69222)
72
high fitness
reduced fitness
low fitness
beta-sheets
alpha-helices
turns
no data / not in library
is in library after recombination
binding sites
active sites
bold
verified ts mutant
-3.7
hydrophobicity (Kyte & Doolittle, 1982, J. Mol., 157, 105-132)
3.7

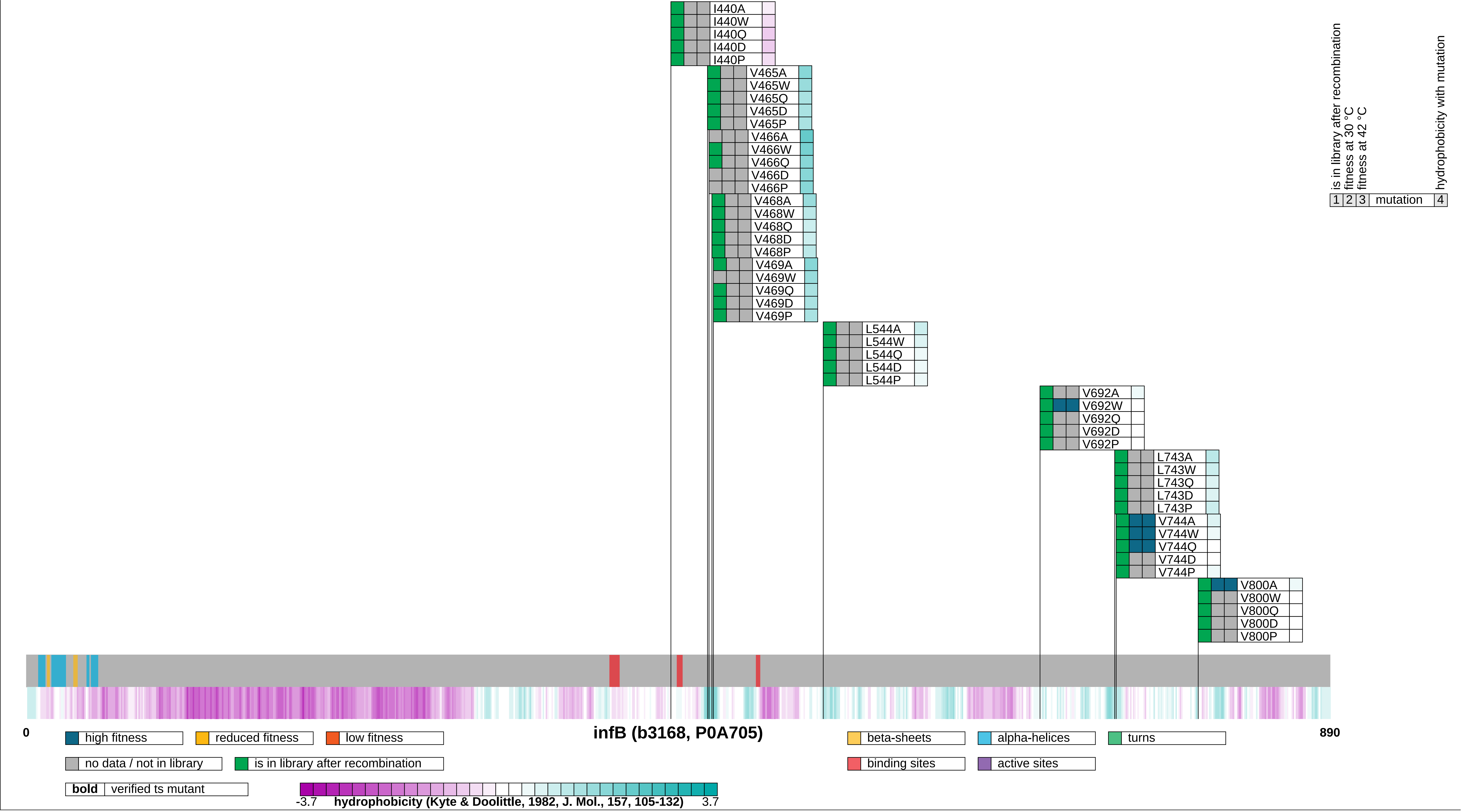

I440A
I440W
I440Q
I440D
I440P
V465A
V465W
V465Q
V465D
V465P
V466A
V466W
V466Q
V466D
V466P
V468A
V468W
V468Q
V468D
V468P
V469A
V469W
V469Q
V469D
V469P
L544A
L544W
L544Q
L544D
L544P
V692A
V692W
V692Q
V692D
V692P
L743A
L743W
L743Q
L743D
L743P
V744A
V744W
V744Q
V744D
V744P
V800A
V800W
V800Q
V800D
V800P
is in library after recombination
fitness at 30 °C
fitness at 42 °C
hydrophobicity with mutation
1 2 3 mutation 4
0
infB (b3168, P0A705)
890
high fitness
reduced fitness
low fitness
no data / not in library
is in library after recombination
bold verified ts mutant
beta-sheets
alpha-helices
turns
binding sites
active sites
-3.7 hydrophobicity (Kyte & Doolittle, 1982, J. Mol., 157, 105-132) 3.7

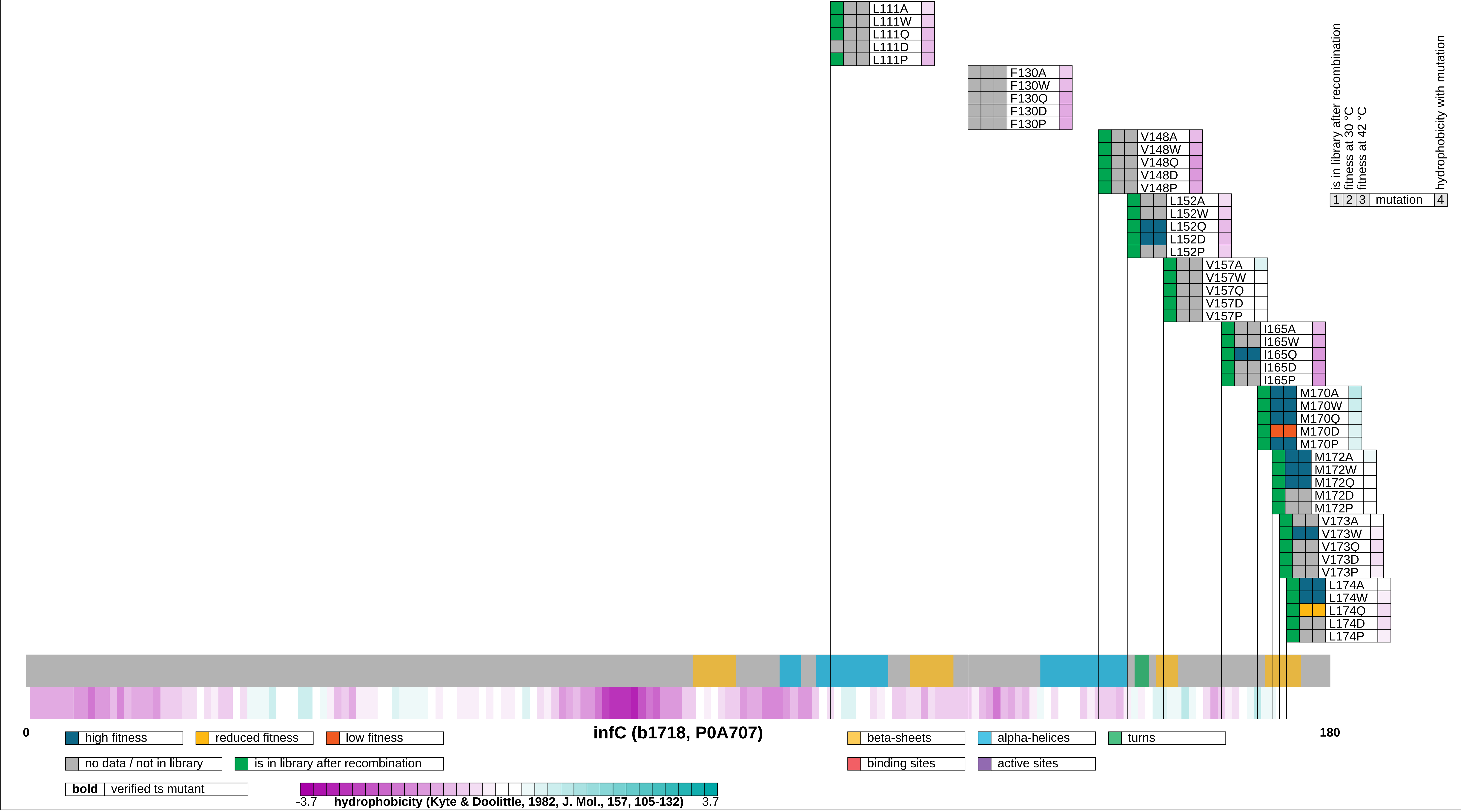

L111A
L111W
L111Q
L111D
L111P
F130A
F130W
F130Q
F130D
F130P
V148A
V148W
V148Q
V148D
V148P
L152A
L152W
L152Q
L152D
L152P
V157A
V157W
V157Q
V157D
V157P
I165A
I165W
I165Q
I165D
I165P
M170A
M170W
M170Q
M170D
M170P
M172A
M172W
M172Q
M172D
M172P
V173A
V173W
V173Q
V173D
V173P
L174A
L174W
L174Q
L174D
L174P
is in library after recombination
fitness at 30 °C
fitness at 42 °C
hydrophobicity with mutation
1 2 3 mutation 4
0
infC (b1718, P0A707)
180
high fitness
reduced fitness
low fitness
no data / not in library
is in library after recombination
bold verified ts mutant
beta-sheets
alpha-helices
turns
binding sites
active sites
-3.7 hydrophobicity (Kyte & Doolittle, 1982, J. Mol., 157, 105-132) 3.7

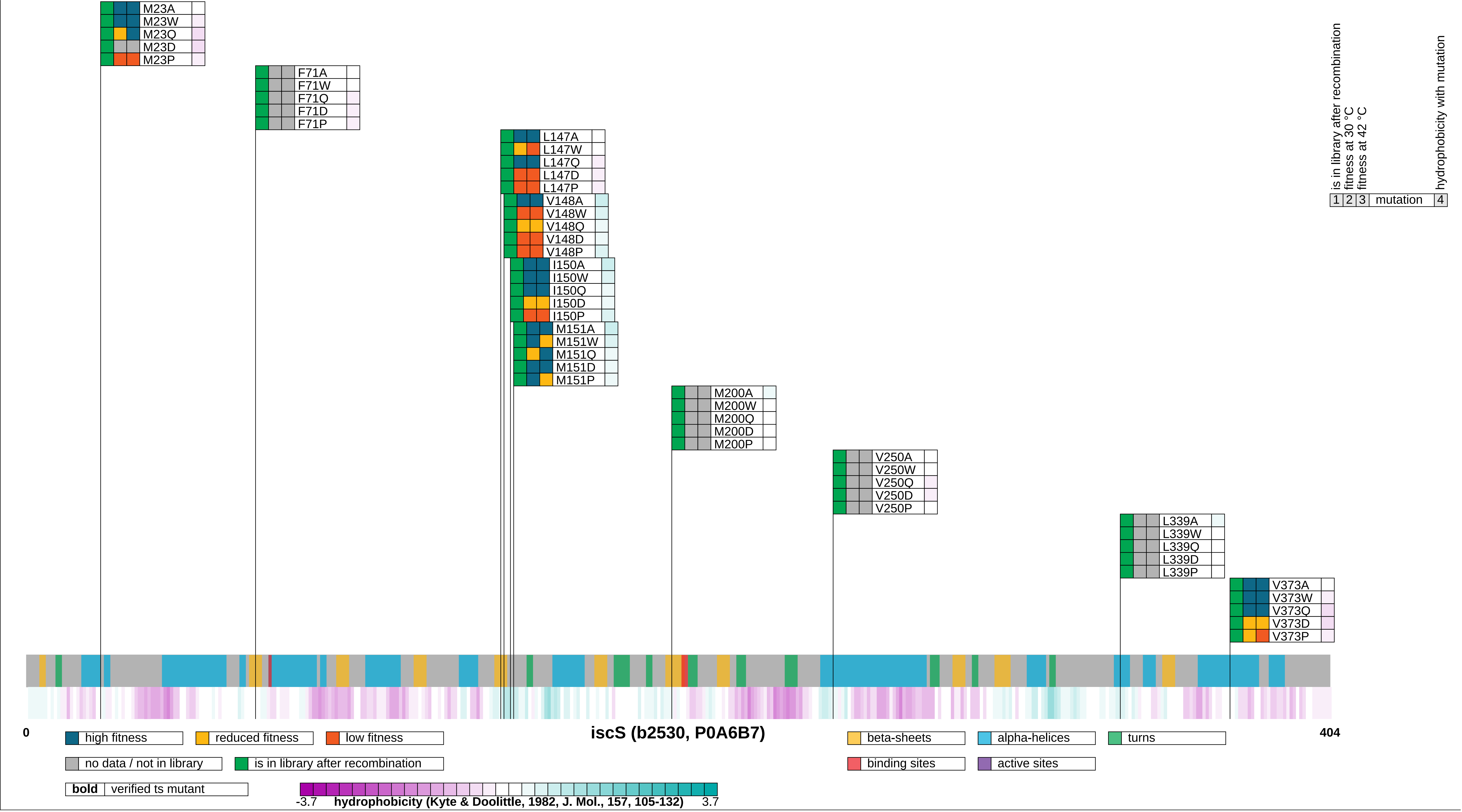

M23A
M23W
M23Q
M23D
M23P
F71A
F71W
F71Q
F71D
F71P
L147A
L147W
L147Q
L147D
L147P
V148A
V148W
V148Q
V148D
V148P
I150A
I150W
I150Q
I150D
I150P
M151A
M151W
M151Q
M151D
M151P
M200A
M200W
M200Q
M200D
M200P
V250A
V250W
V250Q
V250D
V250P
L339A
L339W
L339Q
L339D
L339P
V373A
V373W
V373Q
V373D
V373P
is in library after recombination
fitness at 30 °C
fitness at 42 °C
hydrophobicity with mutation
1 2 3 mutation 4
0
iscS (b2530, P0A6B7)
404
high fitness
reduced fitness
low fitness
beta-sheets
alpha-helices
turns
no data / not in library
is in library after recombination
binding sites
active sites
bold verified ts mutant
-3.7
hydrophobicity (Kyte & Doolittle, 1982, J. Mol., 157, 105-132)
3.7

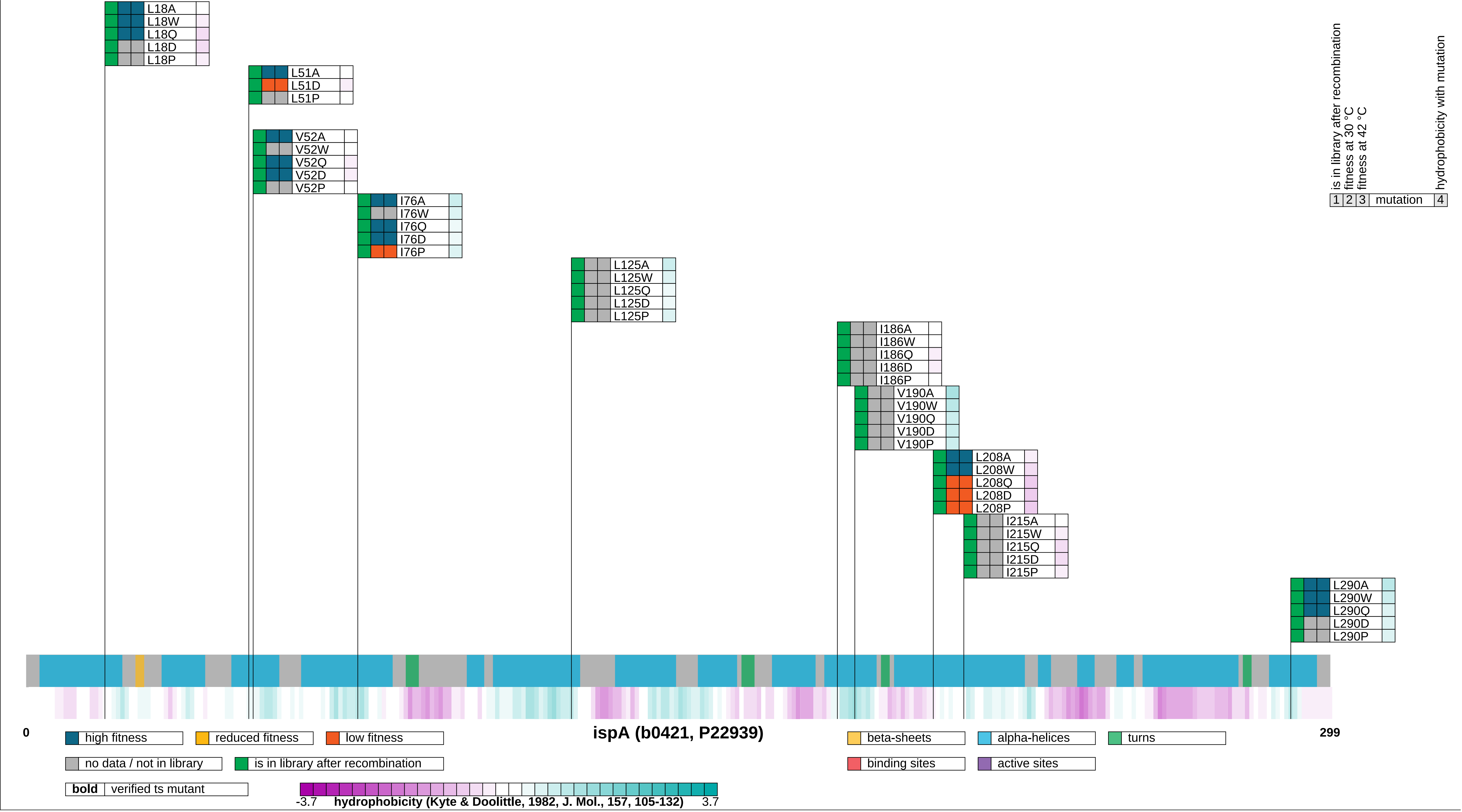

L18A
L18W
L18Q
L18D
L18P
L51A
L51D
L51P
V52A
V52W
V52Q
V52D
V52P
I76A
I76W
I76Q
I76D
I76P
L125A
L125W
L125Q
L125D
L125P
I186A
I186W
I186Q
I186D
I186P
V190A
V190W
V190Q
V190D
V190P
L208A
L208W
L208Q
L208D
L208P
I215A
I215W
I215Q
I215D
I215P
L290A
L290W
L290Q
L290D
L290P
is in library after recombination
fitness at 30 °C
fitness at 42 °C
hydrophobicity with mutation
1 2 3 mutation 4
0
ispA (b0421, P22939)
299
high fitness
reduced fitness
low fitness
beta-sheets
alpha-helices
turns
no data / not in library
is in library after recombination
binding sites
active sites
bold verified ts mutant
-3.7 hydrophobicity (Kyte & Doolittle, 1982, J. Mol., 157, 105-132) 3.7

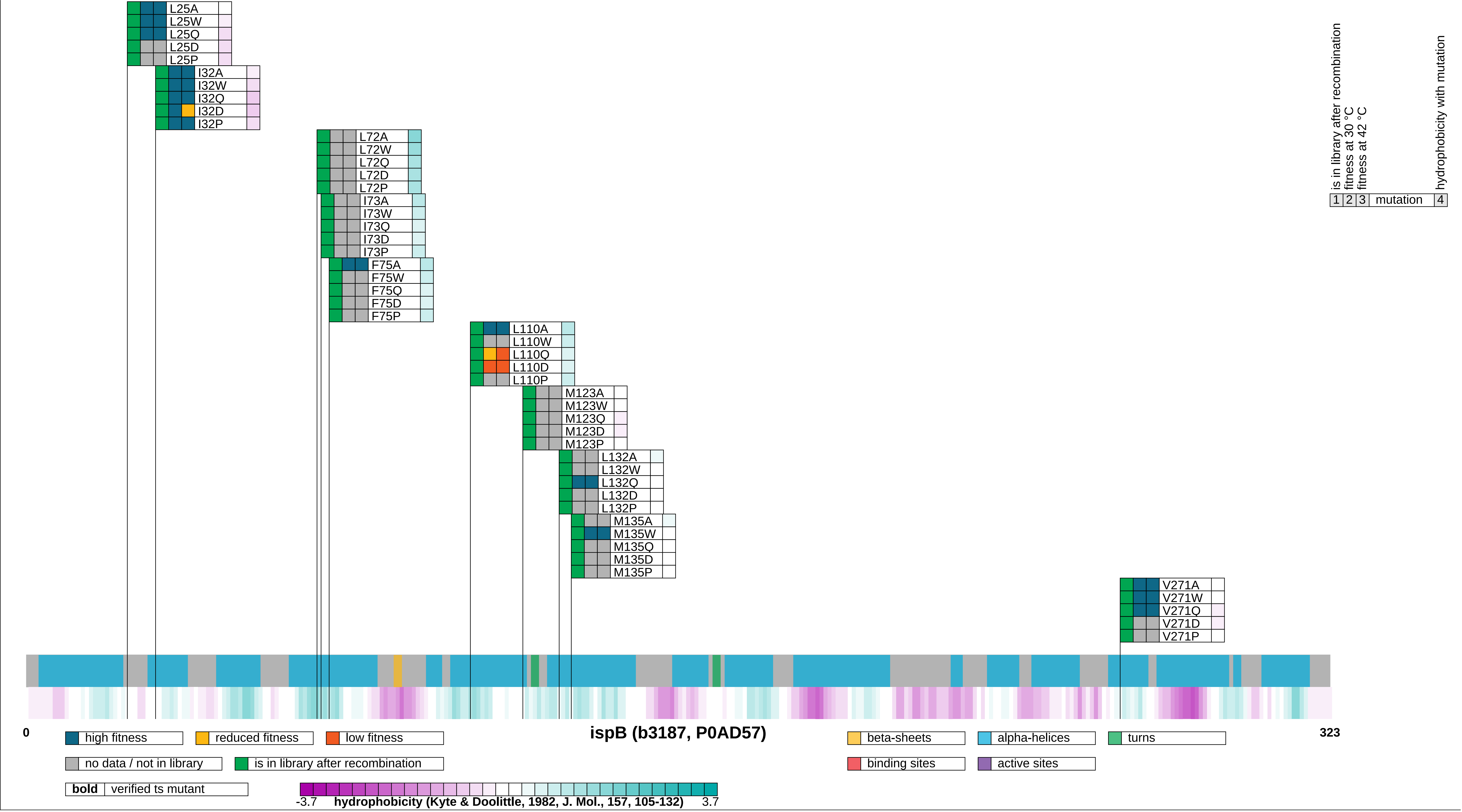
L25A
L25W
L25Q
L25D
L25P
I32A
I32W
I32Q
I32D
I32P
L72A
L72W
L72Q
L72D
L72P
I73A
I73W
I73Q
I73D
I73P
F75A
F75W
F75Q
F75D
F75P
L110A
L110W
L110Q
L110D
L110P
M123A
M123W
M123Q
M123D
M123P
L132A
L132W
L132Q
L132D
L132P
M135A
M135W
M135Q
M135D
M135P
V271A
V271W
V271Q
V271D
V271P
is in library after recombination
fitness at 30 °C
fitness at 42 °C
hydrophobicity with mutation
1 2 3 mutation 4
0
ispB (b3187, P0AD57)
323
high fitness
reduced fitness
low fitness
no data / not in library
is in library after recombination
bold verified ts mutant
beta-sheets
alpha-helices
turns
binding sites
active sites
-3.7 hydrophobicity (Kyte & Doolittle, 1982, J. Mol., 157, 105-132) 3.7

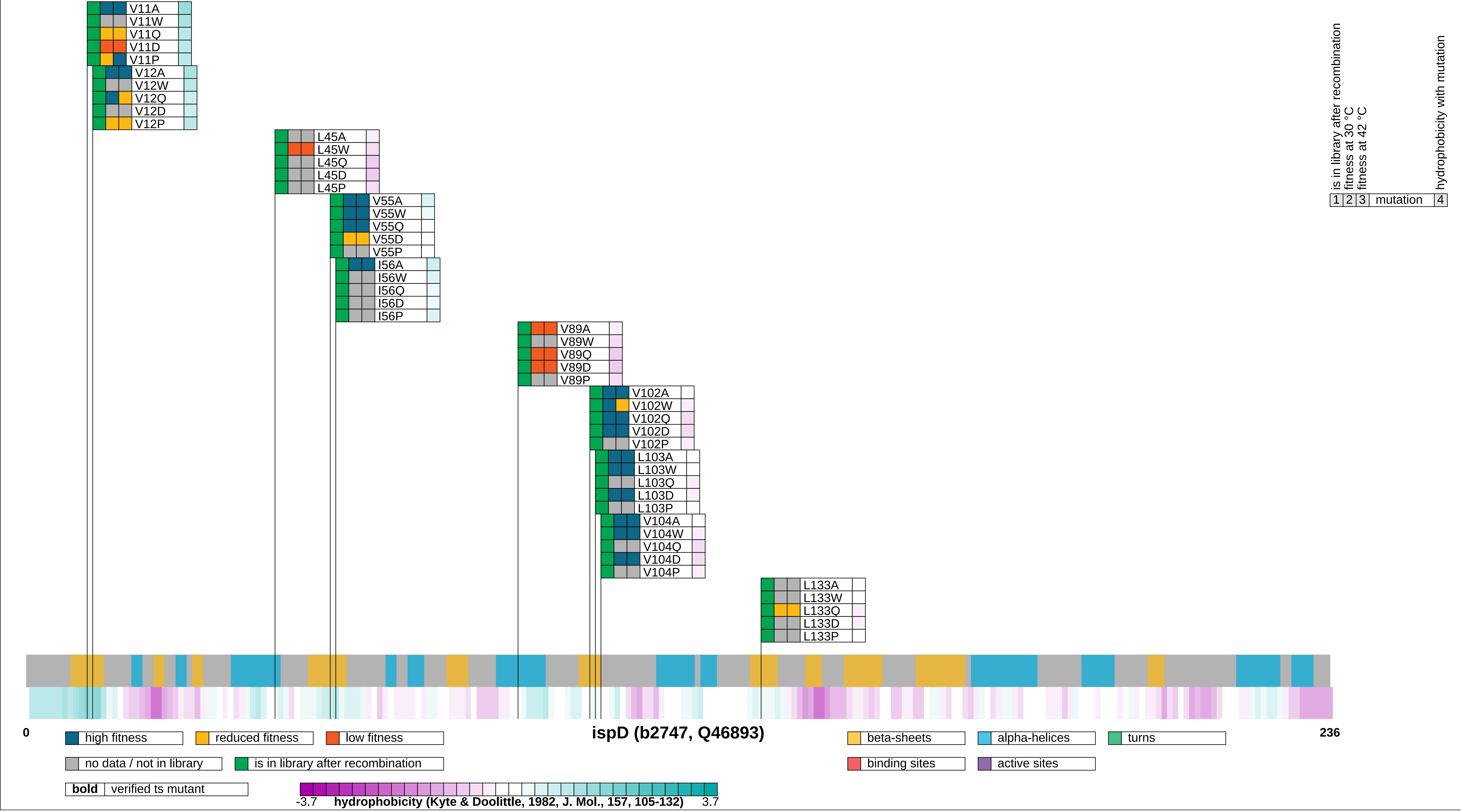
V11A
V11W
V11Q
V11D
V11P
V12A
V12W
V12Q
V12D
V12P
L45A
L45W
L45Q
L45D
L45P
V55A
V55W
V55Q
V55D
V55P
I56A
I56W
I56Q
I56D
I56P
V89A
V89W
V89Q
V89D
V89P
V102A
V102W
V102Q
V102D
V102P
L103A
L103W
L103Q
L103D
L103P
V104A
V104W
V104Q
V104D
V104P
L133A
L133W
L133Q
L133D
L133P
is in library after recombination
fitness at 30 °C
fitness at 42 °C
hydrophobicity with mutation
1 2 3 mutation 4
0
ispD (b2747, Q46893)
236
high fitness
reduced fitness
low fitness
beta-sheets
alpha-helices
turns
no data / not in library
is in library after recombination
binding sites
active sites
bold verified ts mutant
-3.7 hydrophobicity (Kyte & Doolittle, 1982, J. Mol., 157, 105-132) 3.7

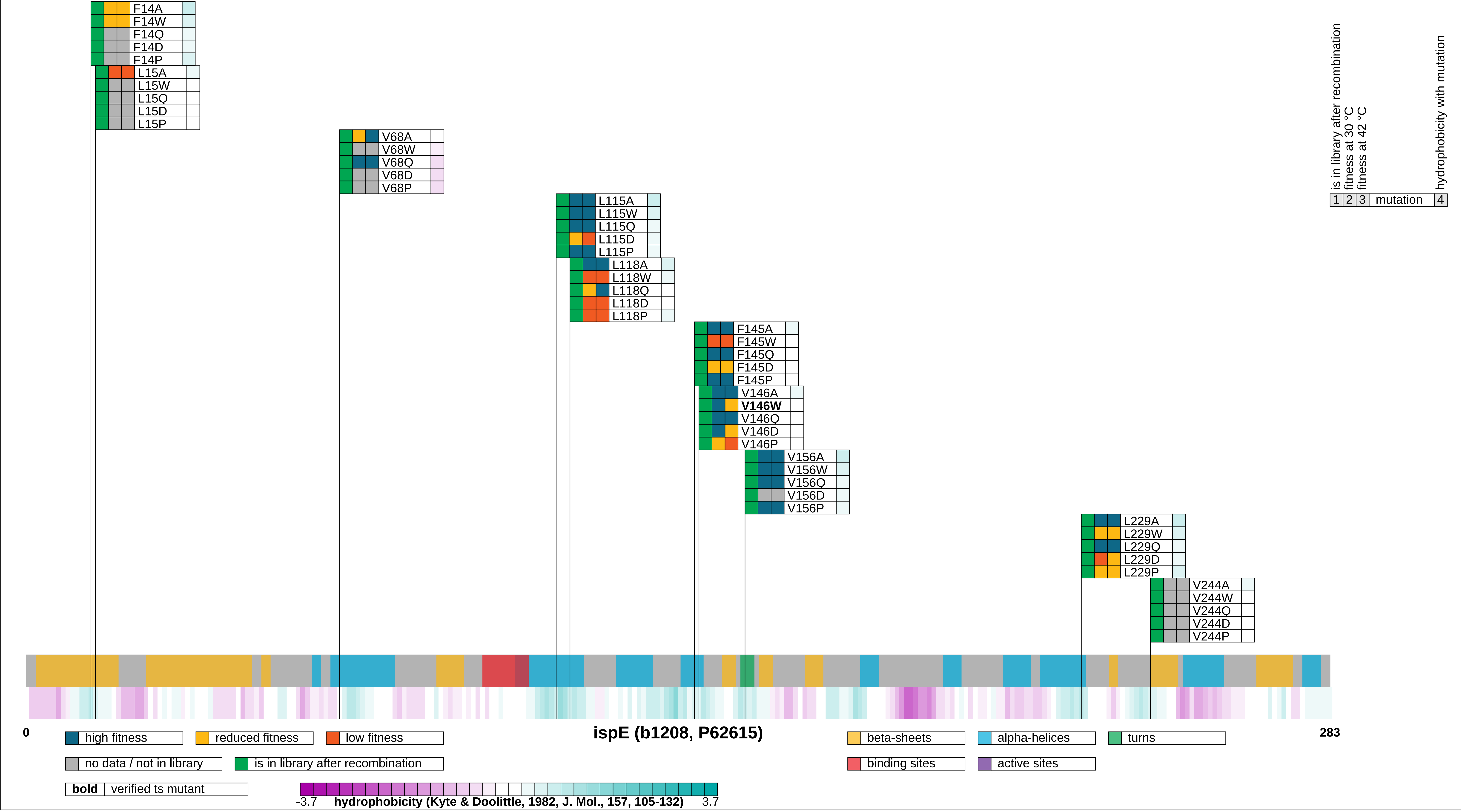
F14A
F14W
F14Q
F14D
F14P
L15A
L15W
L15Q
L15D
L15P
V68A
V68W
V68Q
V68D
V68P
L115A
L115W
L115Q
L115D
L115P
L118A
L118W
L118Q
L118D
L118P
F145A
F145W
F145Q
F145D
F145P
V146A
V146W
V146Q
V146D
V146P
V156A
V156W
V156Q
V156D
V156P
L229A
L229W
L229Q
L229D
L229P
V244A
V244W
V244Q
V244D
V244P
is in library after recombination
fitness at 30 °C
fitness at 42 °C
hydrophobicity with mutation
1 2 3 mutation 4
0
ispE (b1208, P62615)
283
high fitness
reduced fitness
low fitness
beta-sheets
alpha-helices
turns
no data / not in library
is in library after recombination
binding sites
active sites
bold verified ts mutant
-3.7 hydrophobicity (Kyte & Doolittle, 1982, J. Mol., 157, 105-132) 3.7

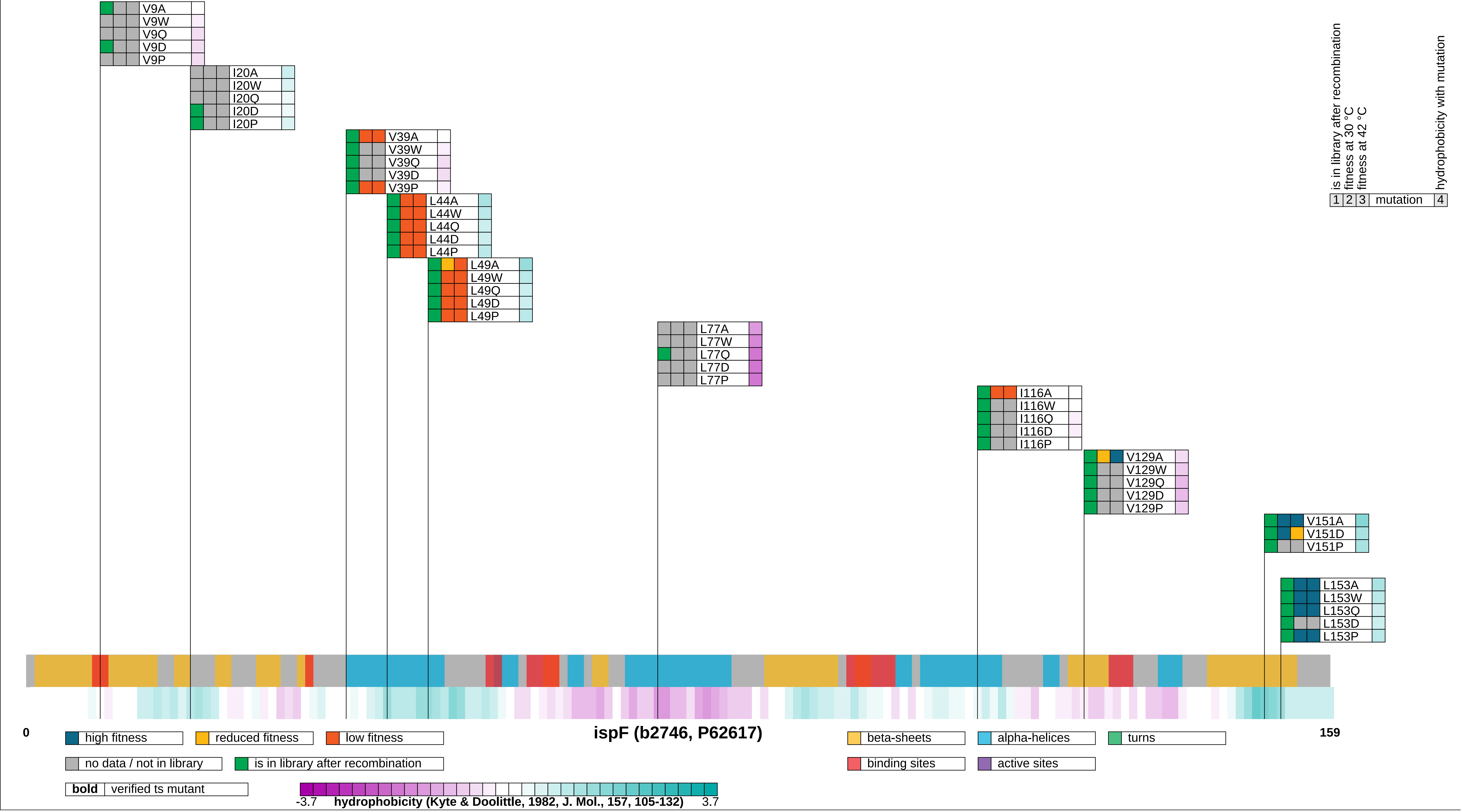

V9A
V9W
V9Q
V9D
V9P
I20A
I20W
I20Q
I20D
I20P
V39A
V39W
V39Q
V39D
V39P
L44A
L44W
L44Q
L44D
L44P
L49A
L49W
L49Q
L49D
L49P
L77A
L77W
L77Q
L77D
L77P
I116A
I116W
I116Q
I116D
I116P
V129A
V129W
V129Q
V129D
V129P
V151A
V151D
V151P
L153A
L153W
L153Q
L153D
L153P
is in library after recombination
fitness at 30 °C
fitness at 42 °C
hydrophobicity with mutation
1 2 3 mutation 4
0
ispF (b2746, P62617)
159
high fitness
reduced fitness
low fitness
beta-sheets
alpha-helices
turns
no data / not in library
is in library after recombination
binding sites
active sites
bold verified ts mutant
-3.7 hydrophobicity (Kyte & Doolittle, 1982, J. Mol., 157, 105-132) 3.7

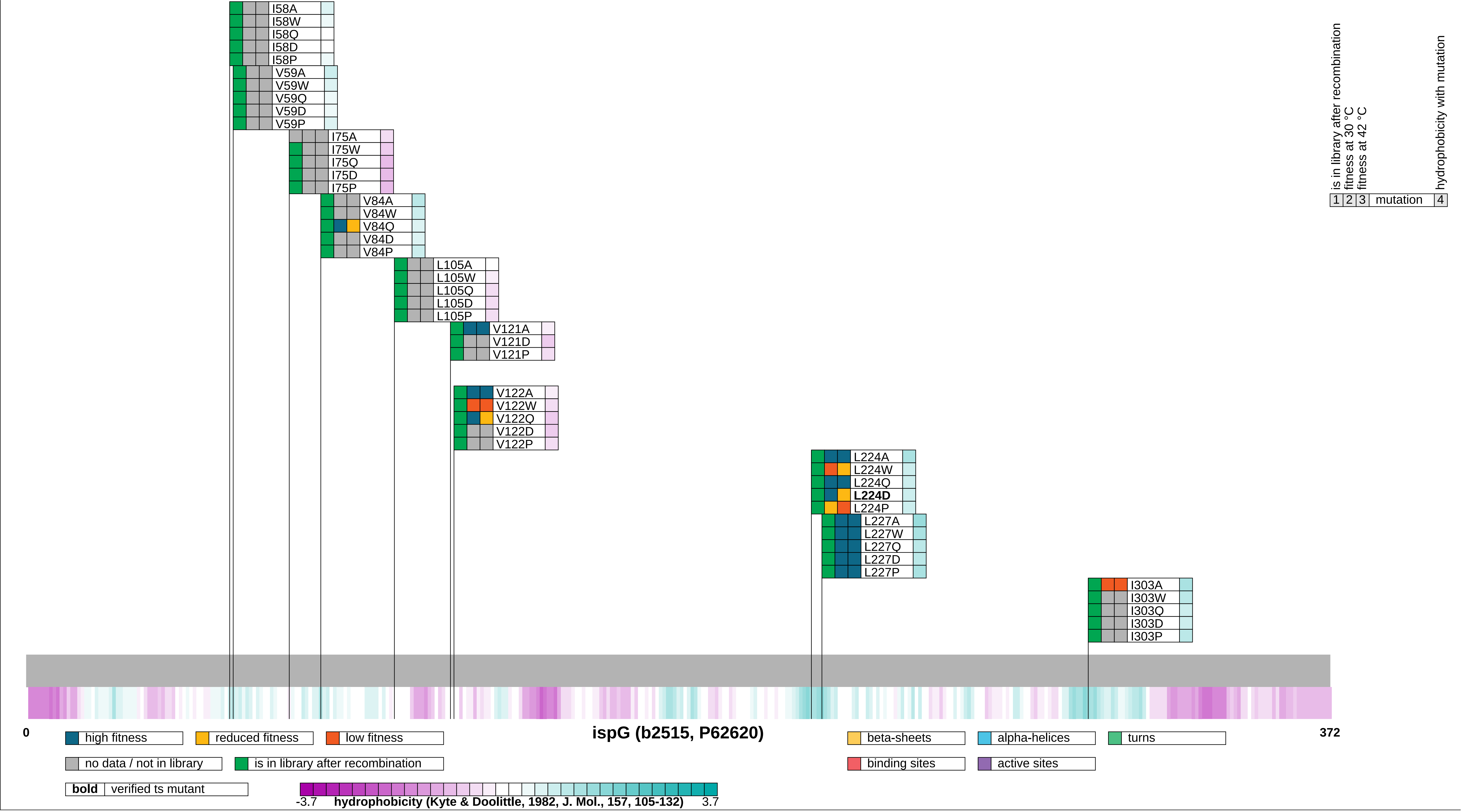

I58A
I58W
I58Q
I58D
I58P
V59A
V59W
V59Q
V59D
V59P
I75A
I75W
I75Q
I75D
I75P
V84A
V84W
V84Q
V84D
V84P
L105A
L105W
L105Q
L105D
L105P
V121A
V121D
V121P
V122A
V122W
V122Q
V122D
V122P
L224A
L224W
L224Q
L224D
L224P
L227A
L227W
L227Q
L227D
L227P
I303A
I303W
I303Q
I303D
I303P
is in library after recombination
fitness at 30 °C
fitness at 42 °C
hydrophobicity with mutation
1 2 3 mutation 4
0
ispG (b2515, P62620)
372
high fitness
reduced fitness
low fitness
beta-sheets
alpha-helices
turns
no data / not in library
is in library after recombination
binding sites
active sites
bold verified ts mutant
-3.7 hydrophobicity (Kyte & Doolittle, 1982, J. Mol., 157, 105-132) 3.7

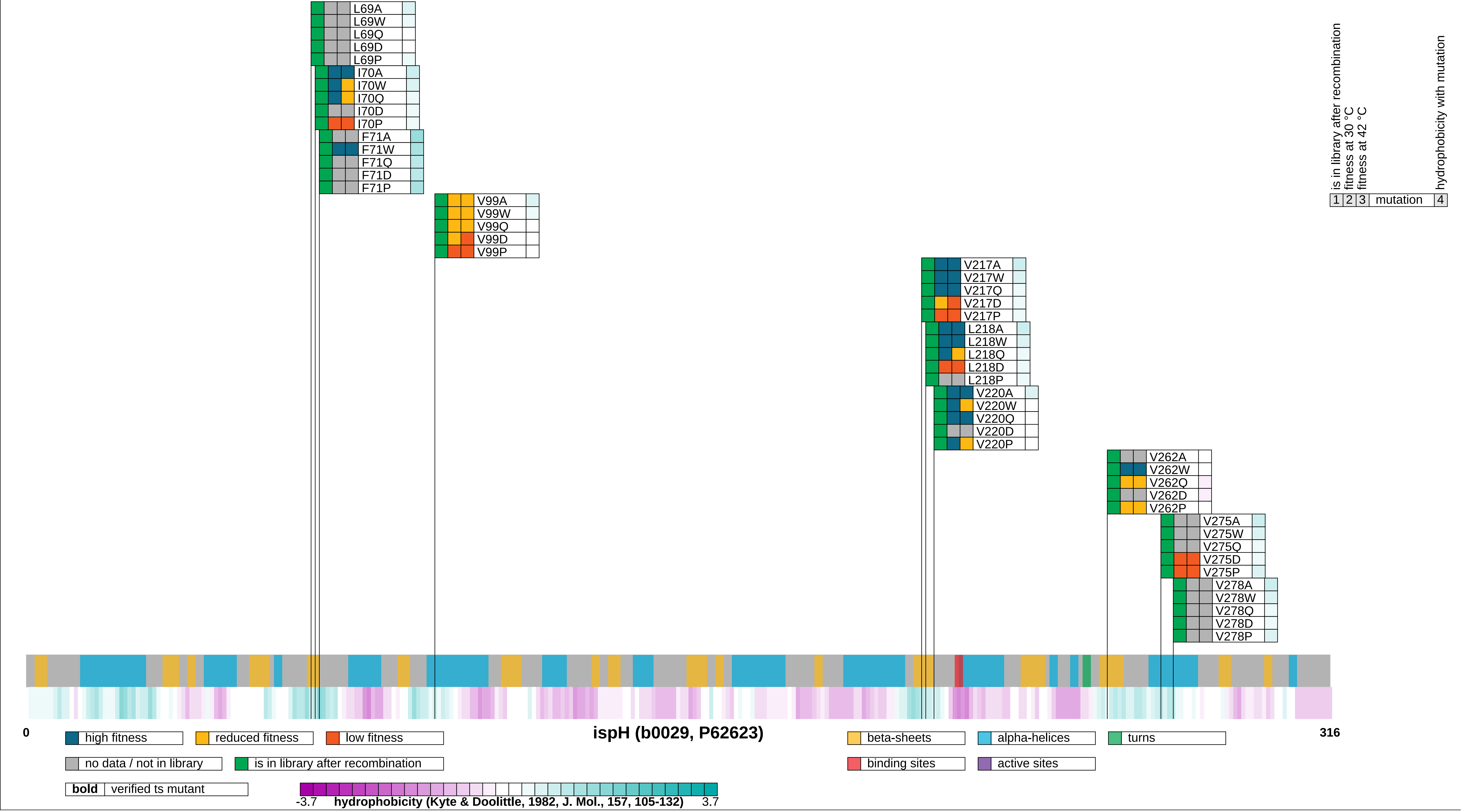
L69A
L69W
L69Q
L69D
L69P
I70A
I70W
I70Q
I70D
I70P
F71A
F71W
F71Q
F71D
F71P
V99A
V99W
V99Q
V99D
V99P
V217A
V217W
V217Q
V217D
V217P
L218A
L218W
L218Q
L218D
L218P
V220A
V220W
V220Q
V220D
V220P
V262A
V262W
V262Q
V262D
V262P
V275A
V275W
V275Q
V275D
V275P
V278A
V278W
V278Q
V278D
V278P
is in library after recombination
fitness at 30 °C
fitness at 42 °C
hydrophobicity with mutation
1 2 3 mutation 4
0
ispH (b0029, P62623)
316
high fitness
reduced fitness
low fitness
no data / not in library
is in library after recombination
bold verified ts mutant
beta-sheets
alpha-helices
turns
binding sites
active sites
-3.7
hydrophobicity (Kyte & Doolittle, 1982, J. Mol., 157, 105-132)
3.7

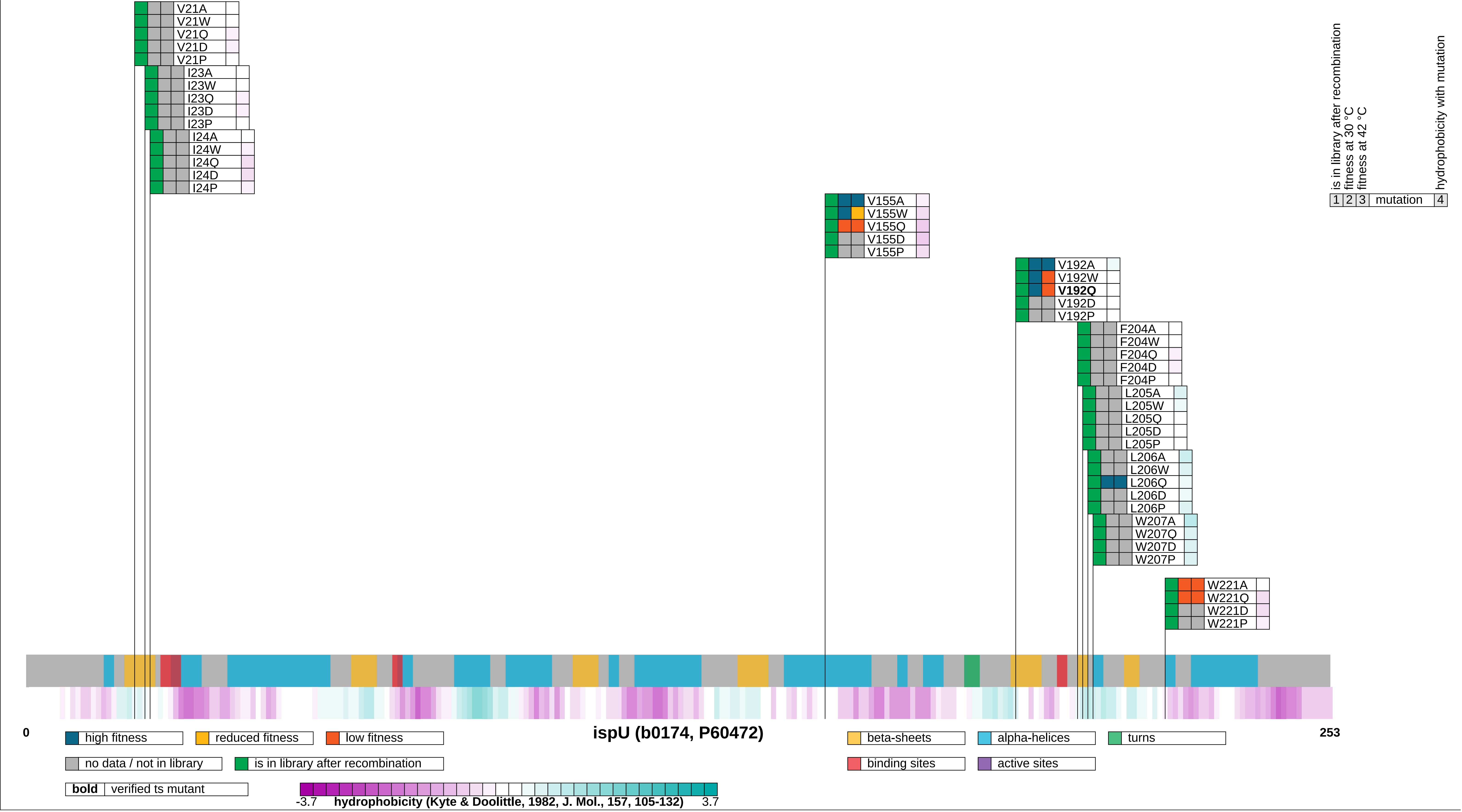

V21A
V21W
V21Q
V21D
V21P
I23A
I23W
I23Q
I23D
I23P
I24A
I24W
I24Q
I24D
I24P
V155A
V155W
V155Q
V155D
V155P
V192A
V192W
V192Q
V192D
V192P
F204A
F204W
F204Q
F204D
F204P
L205A
L205W
L205Q
L205D
L205P
L206A
L206W
L206Q
L206D
L206P
W207A
W207Q
W207D
W207P
W221A
W221Q
W221D
W221P
is in library after recombination
fitness at 30 °C
fitness at 42 °C
hydrophobicity with mutation
1 2 3 mutation 4
0
ispU (b0174, P60472)
253
high fitness
reduced fitness
low fitness
no data / not in library
is in library after recombination
bold verified ts mutant
beta-sheets
alpha-helices
turns
binding sites
active sites
-3.7
hydrophobicity (Kyte & Doolittle, 1982, J. Mol., 157, 105-132)
3.7

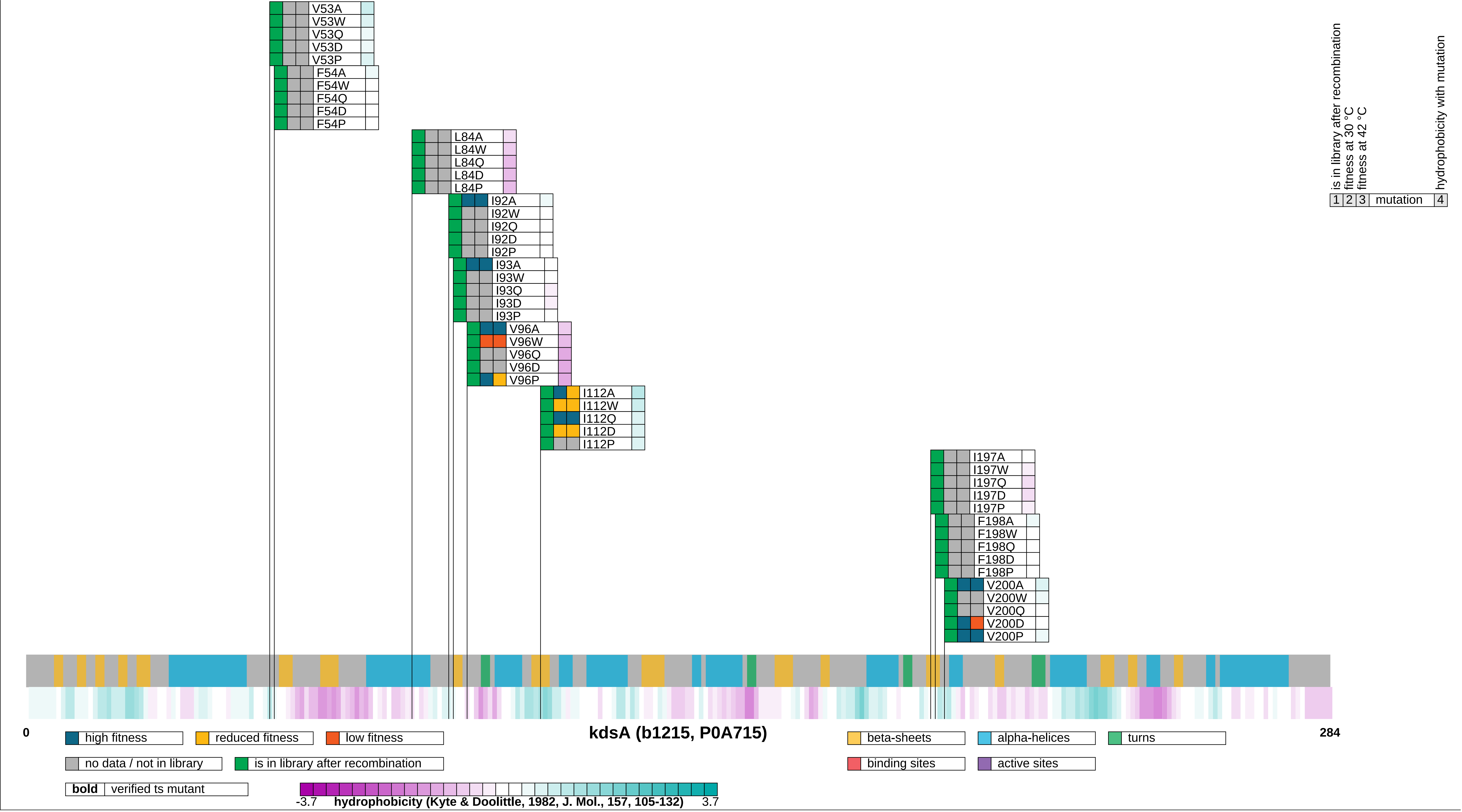

V53A
V53W
V53Q
V53D
V53P
F54A
F54W
F54Q
F54D
F54P
L84A
L84W
L84Q
L84D
L84P
I92A
I92W
I92Q
I92D
I92P
I93A
I93W
I93Q
I93D
I93P
V96A
V96W
V96Q
V96D
V96P
I112A
I112W
I112Q
I112D
I112P
I197A
I197W
I197Q
I197D
I197P
F198A
F198W
F198Q
F198D
F198P
V200A
V200W
V200Q
V200D
V200P
is in library after recombination
fitness at 30 °C
fitness at 42 °C
hydrophobicity with mutation
1 2 3 mutation 4
0
kdsA (b1215, P0A715)
284
high fitness
reduced fitness
low fitness
no data / not in library
is in library after recombination
bold verified ts mutant
beta-sheets
alpha-helices
turns
binding sites
active sites
-3.7 hydrophobicity (Kyte & Doolittle, 1982, J. Mol., 157, 105-132) 3.7

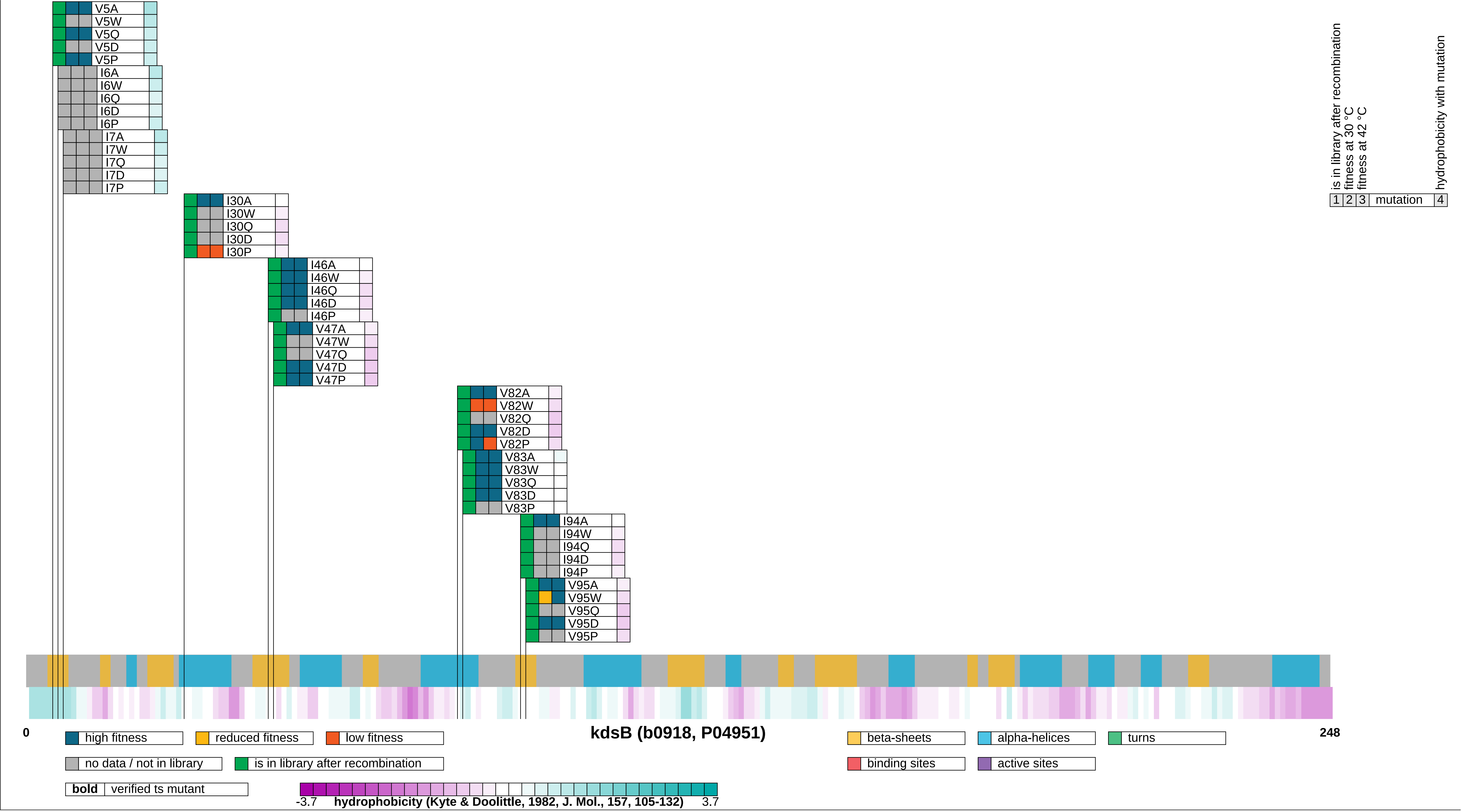

V5A
V5W
V5Q
V5D
V5P
I6A
I6W
I6Q
I6D
I6P
I7A
I7W
I7Q
I7D
I7P
I30A
I30W
I30Q
I30D
I30P
I46A
I46W
I46Q
I46D
I46P
V47A
V47W
V47Q
V47D
V47P
V82A
V82W
V82Q
V82D
V82P
V83A
V83W
V83Q
V83D
V83P
I94A
I94W
I94Q
I94D
I94P
V95A
V95W
V95Q
V95D
V95P
is in library after recombination
fitness at 30 °C
fitness at 42 °C
hydrophobicity with mutation
1 2 3 mutation 4
0
kdsB (b0918, P04951)
248
high fitness
reduced fitness
low fitness
beta-sheets
alpha-helices
turns
no data / not in library
is in library after recombination
binding sites
active sites
bold verified ts mutant
-3.7 hydrophobicity (Kyte & Doolittle, 1982, J. Mol., 157, 105-132) 3.7

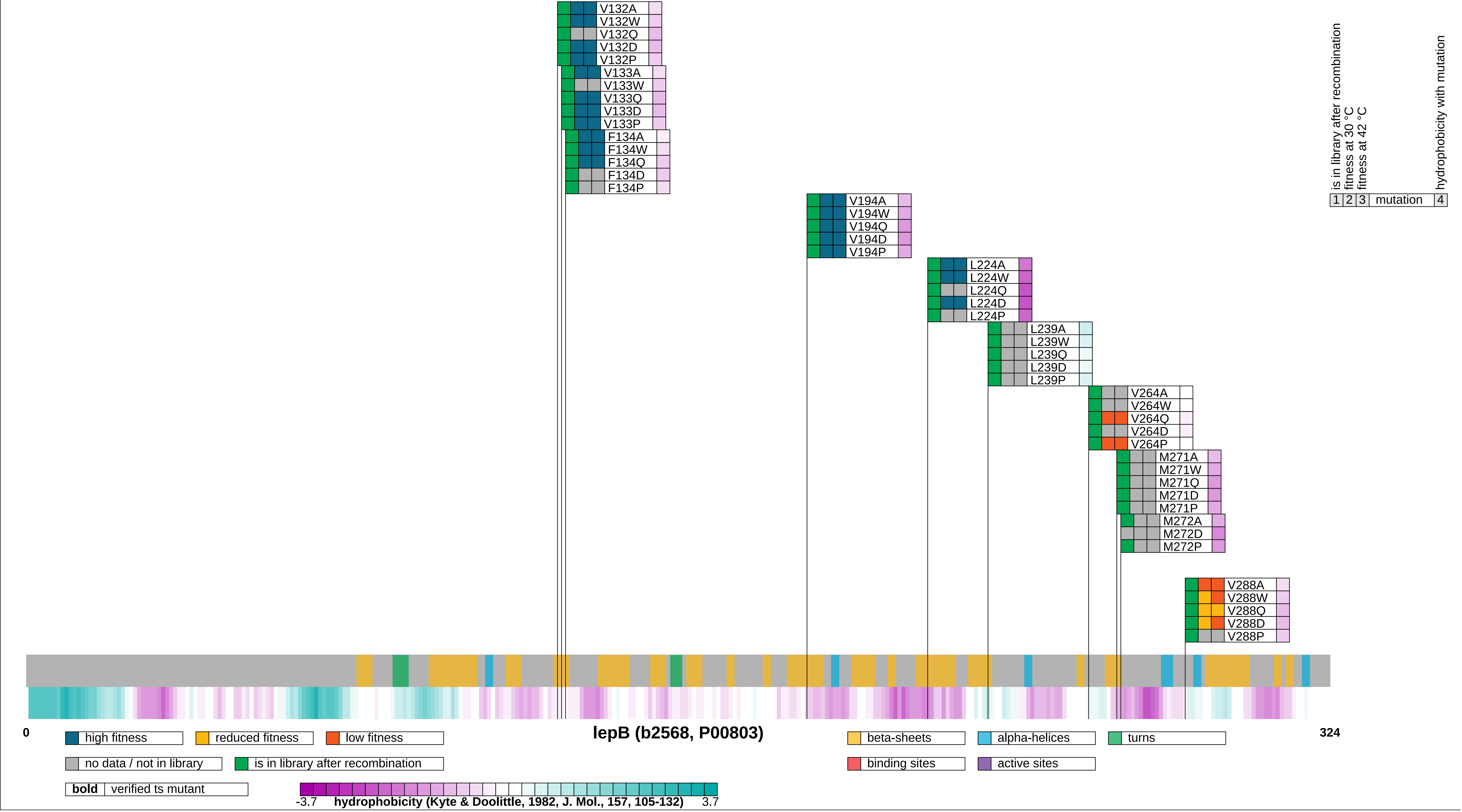

V132A
V132W
V132Q
V132D
V132P
V133A
V133W
V133Q
V133D
V133P
F134A
F134W
F134Q
F134D
F134P
V194A
V194W
V194Q
V194D
V194P
L224A
L224W
L224Q
L224D
L224P
L239A
L239W
L239Q
L239D
L239P
V264A
V264W
V264Q
V264D
V264P
M271A
M271W
M271Q
M271D
M271P
M272A
M272D
M272P
V288A
V288W
V288Q
V288D
V288P
is in library after recombination
fitness at 30 °C
fitness at 42 °C
hydrophobicity with mutation
1 2 3 mutation 4
0
lepB (b2568, P00803)
324
high fitness
reduced fitness
low fitness
no data / not in library
is in library after recombination
bold verified ts mutant
beta-sheets
alpha-helices
turns
binding sites
active sites
-3.7 hydrophobicity (Kyte & Doolittle, 1982, J. Mol., 157, 105-132) 3.7

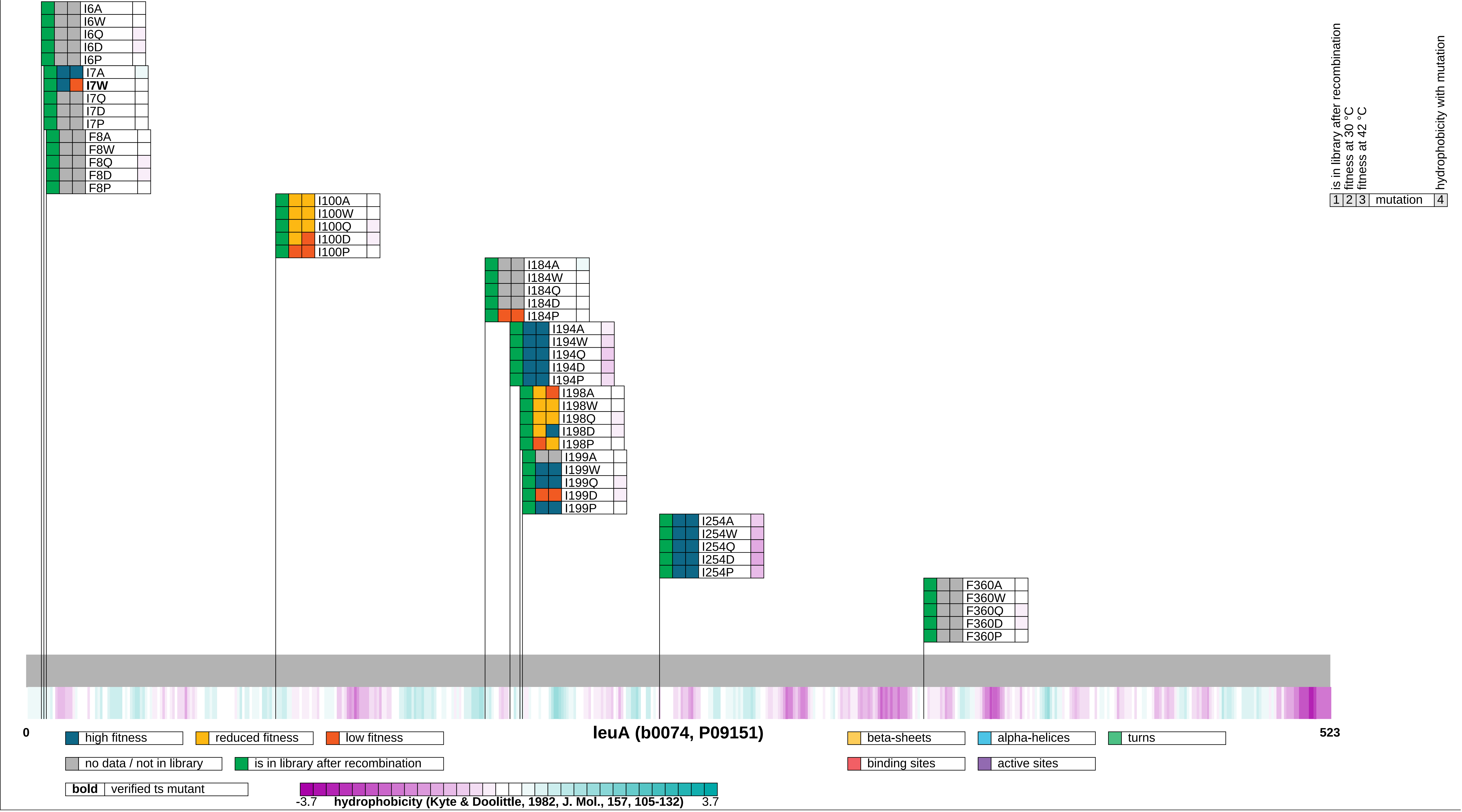

I6A
I6W
I6Q
I6D
I6P
I7A
I7W
I7Q
I7D
I7P
F8A
F8W
F8Q
F8D
F8P
I100A
I100W
I100Q
I100D
I100P
I184A
I184W
I184Q
I184D
I184P
I194A
I194W
I194Q
I194D
I194P
I198A
I198W
I198Q
I198D
I198P
I199A
I199W
I199Q
I199D
I199P
I254A
I254W
I254Q
I254D
I254P
F360A
F360W
F360Q
F360D
F360P
is in library after recombination
fitness at 30 °C
fitness at 42 °C
hydrophobicity with mutation
1 2 3 mutation 4
0
leuA (b0074, P09151)
523
high fitness
reduced fitness
low fitness
beta-sheets
alpha-helices
turns
no data / not in library
is in library after recombination
binding sites
active sites
bold verified ts mutant
-3.7
hydrophobicity (Kyte & Doolittle, 1982, J. Mol., 157, 105-132)
3.7

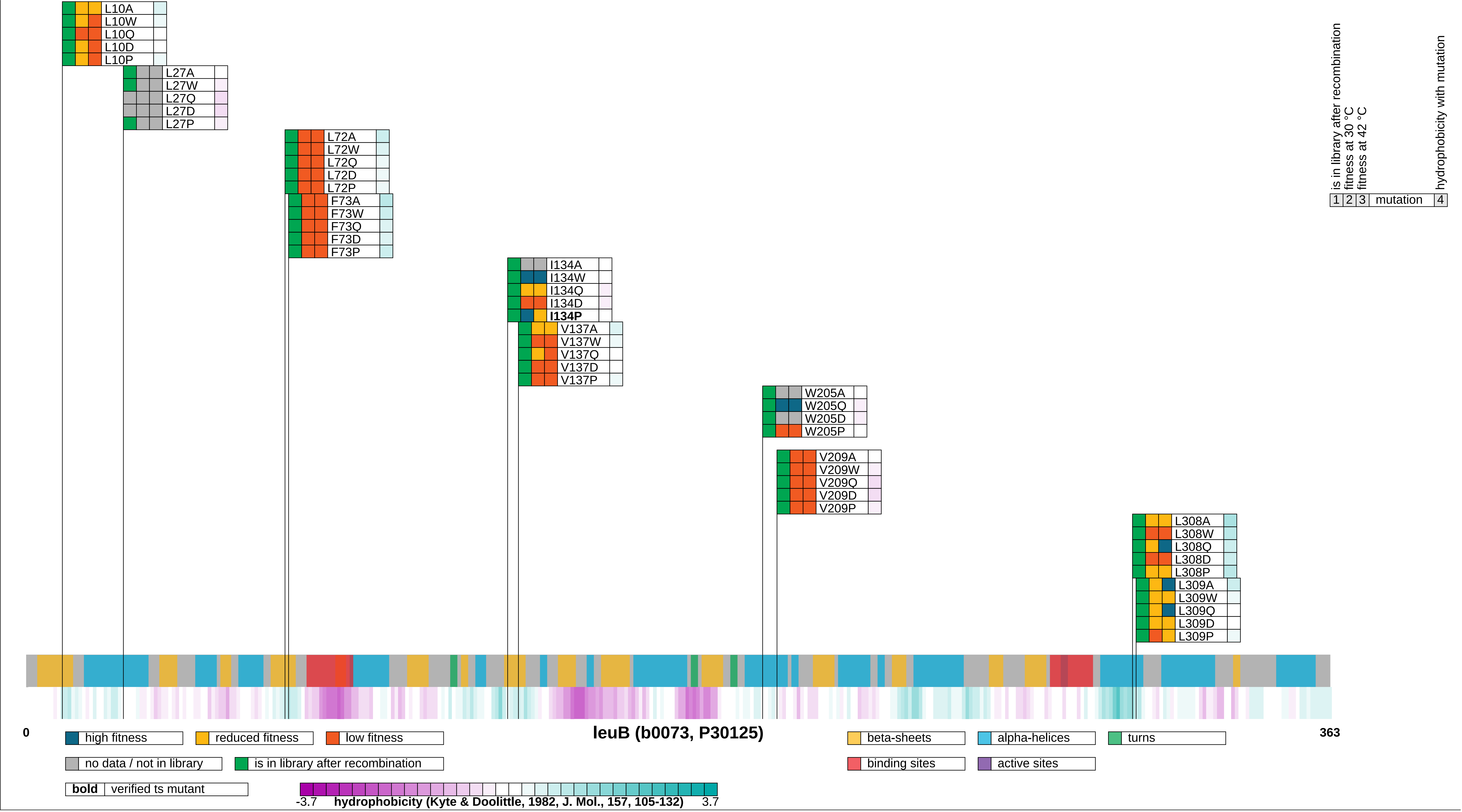
L10A
L10W
L10Q
L10D
L10P
L27A
L27W
L27Q
L27D
L27P
L72A
L72W
L72Q
L72D
L72P
F73A
F73W
F73Q
F73D
F73P
I134A
I134W
I134Q
I134D
I134P
V137A
V137W
V137Q
V137D
V137P
W205A
W205Q
W205D
W205P
V209A
V209W
V209Q
V209D
V209P
L308A
L308W
L308Q
L308D
L308P
L309A
L309W
L309Q
L309D
L309P
is in library after recombination
fitness at 30 °C
fitness at 42 °C
hydrophobicity with mutation
1 2 3 mutation 4
0
leuB (b0073, P30125)
363
high fitness
reduced fitness
low fitness
no data / not in library
is in library after recombination
bold verified ts mutant
beta-sheets
alpha-helices
turns
binding sites
active sites
-3.7 hydrophobicity (Kyte & Doolittle, 1982, J. Mol., 157, 105-132) 3.7

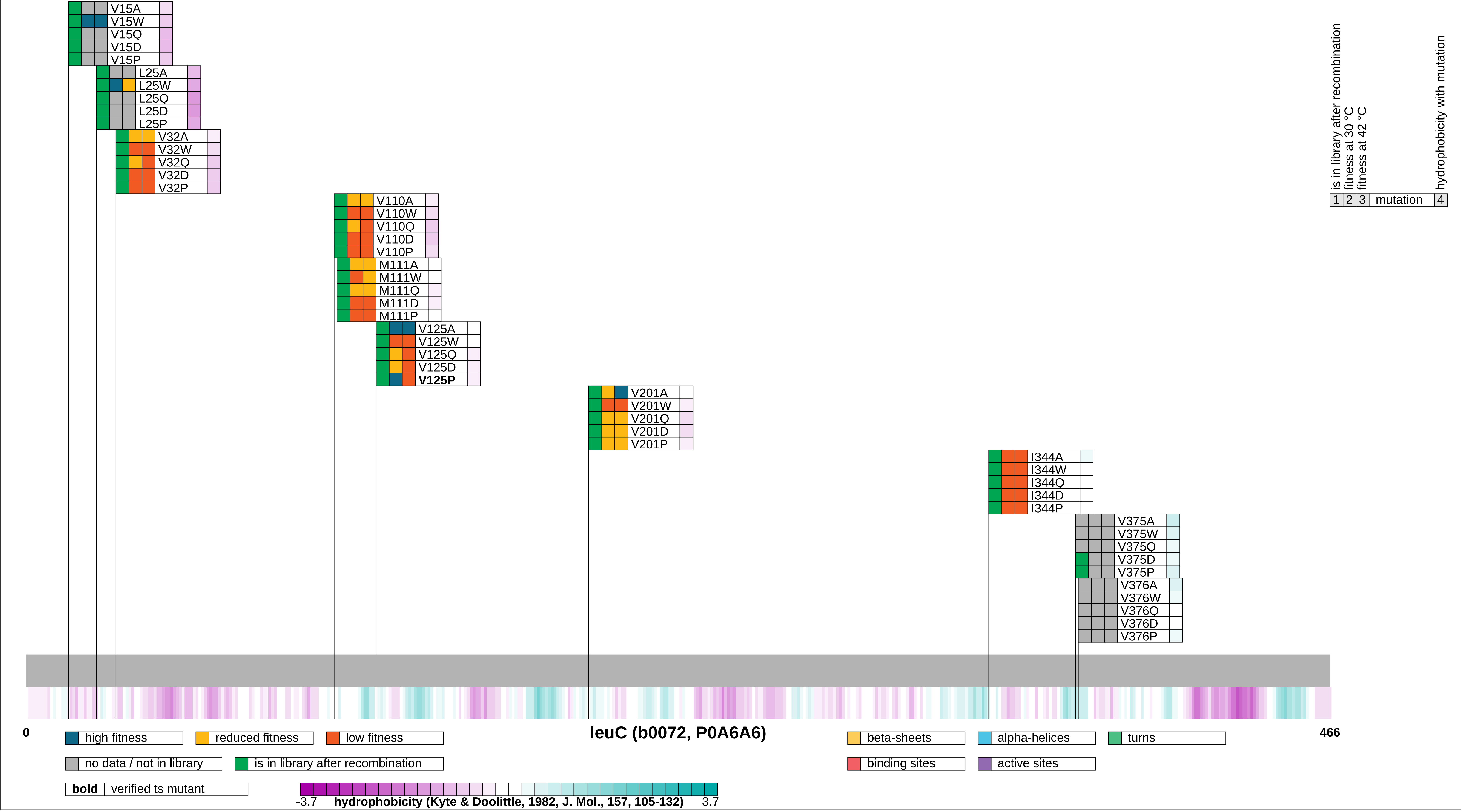

V15A
V15W
V15Q
V15D
V15P
L25A
L25W
L25Q
L25D
L25P
V32A
V32W
V32Q
V32D
V32P
V110A
V110W
V110Q
V110D
V110P
M111A
M111W
M111Q
M111D
M111P
V125A
V125W
V125Q
V125D
V125P
V201A
V201W
V201Q
V201D
V201P
I344A
I344W
I344Q
I344D
I344P
V375A
V375W
V375Q
V375D
V375P
V376A
V376W
V376Q
V376D
V376P
is in library after recombination
fitness at 30 °C
fitness at 42 °C
hydrophobicity with mutation
1 2 3 mutation 4
0
leuC (b0072, P0A6A6)
466
high fitness
reduced fitness
low fitness
beta-sheets
alpha-helices
turns
no data / not in library
is in library after recombination
binding sites
active sites
bold verified ts mutant
-3.7 hydrophobicity (Kyte & Doolittle, 1982, J. Mol., 157, 105-132) 3.7

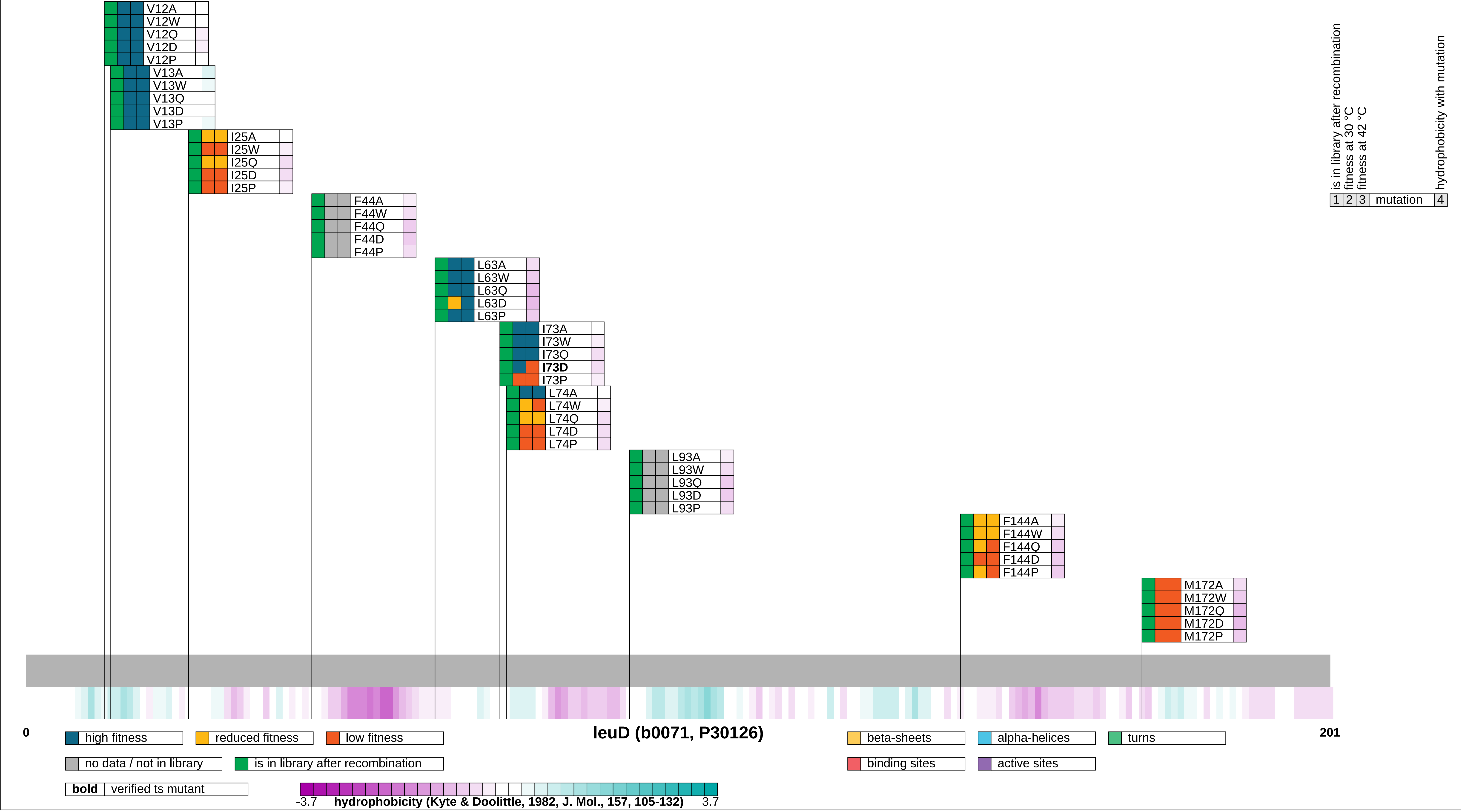

V12A
V12W
V12Q
V12D
V12P
V13A
V13W
V13Q
V13D
V13P
I25A
I25W
I25Q
I25D
I25P
F44A
F44W
F44Q
F44D
F44P
L63A
L63W
L63Q
L63D
L63P
I73A
I73W
I73Q
I73D
I73P
L74A
L74W
L74Q
L74D
L74P
L93A
L93W
L93Q
L93D
L93P
F144A
F144W
F144Q
F144D
F144P
M172A
M172W
M172Q
M172D
M172P
is in library after recombination
fitness at 30 °C
fitness at 42 °C
hydrophobicity with mutation
1
2
3
mutation
4
0
leuD (b0071, P30126)
201
high fitness
reduced fitness
low fitness
no data / not in library
is in library after recombination
bold
verified ts mutant
beta-sheets
alpha-helices
turns
binding sites
active sites
-3.7
hydrophobicity (Kyte & Doolittle, 1982, J. Mol., 157, 105-132)
3.7

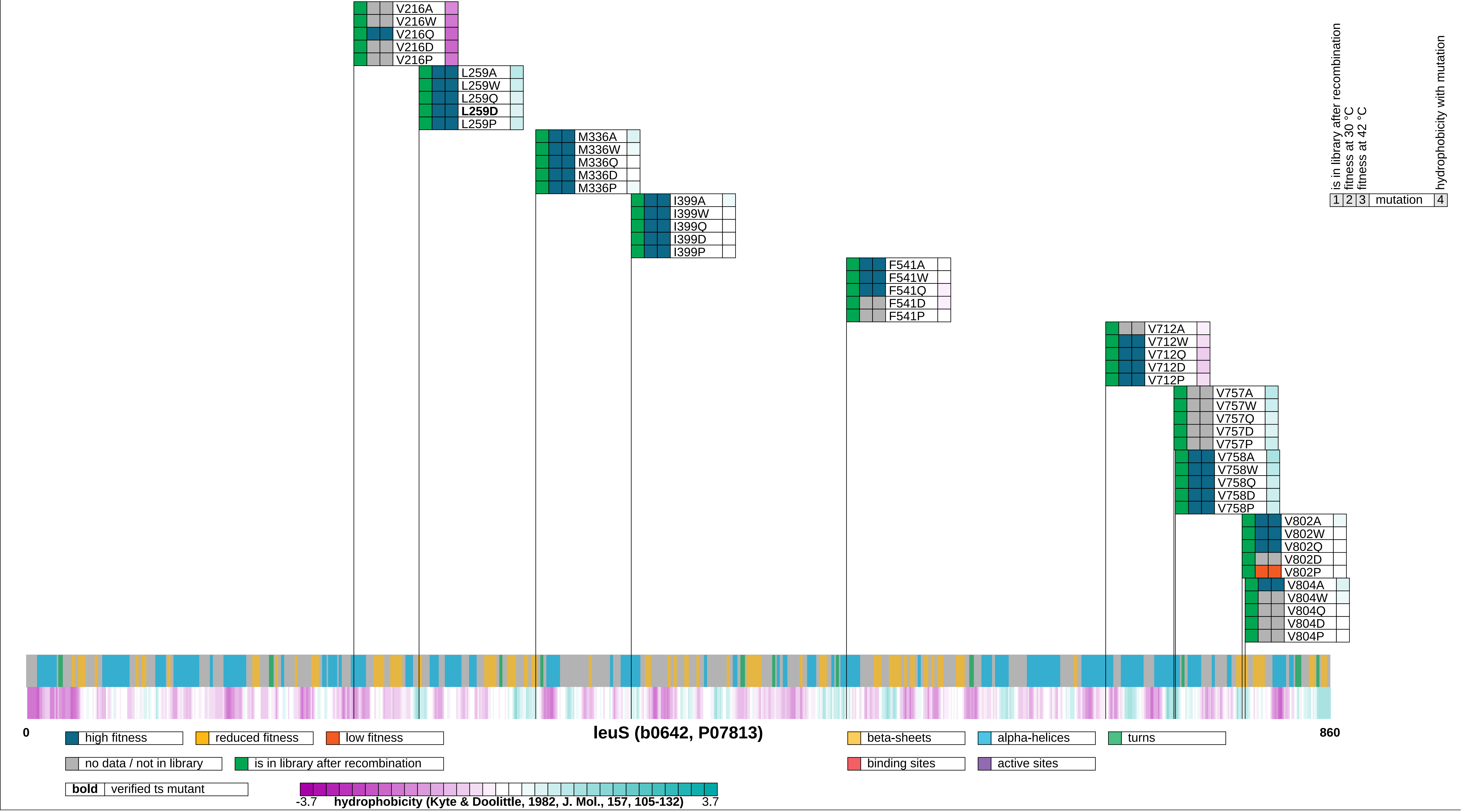

V216A
V216W
V216Q
V216D
V216P
L259A
L259W
L259Q
L259D
L259P
M336A
M336W
M336Q
M336D
M336P
I399A
I399W
I399Q
I399D
I399P
F541A
F541W
F541Q
F541D
F541P
V712A
V712W
V712Q
V712D
V712P
V757A
V757W
V757Q
V757D
V757P
V758A
V758W
V758Q
V758D
V758P
V802A
V802W
V802Q
V802D
V802P
V804A
V804W
V804Q
V804D
V804P
is in library after recombination
fitness at 30 °C
fitness at 42 °C
hydrophobicity with mutation
1 2 3 mutation 4
0
leuS (b0642, P07813)
860
high fitness
reduced fitness
low fitness
no data / not in library
is in library after recombination
beta-sheets
alpha-helices
turns
binding sites
active sites
bold verified ts mutant
-3.7
hydrophobicity (Kyte & Doolittle, 1982, J. Mol., 157, 105-132)
3.7

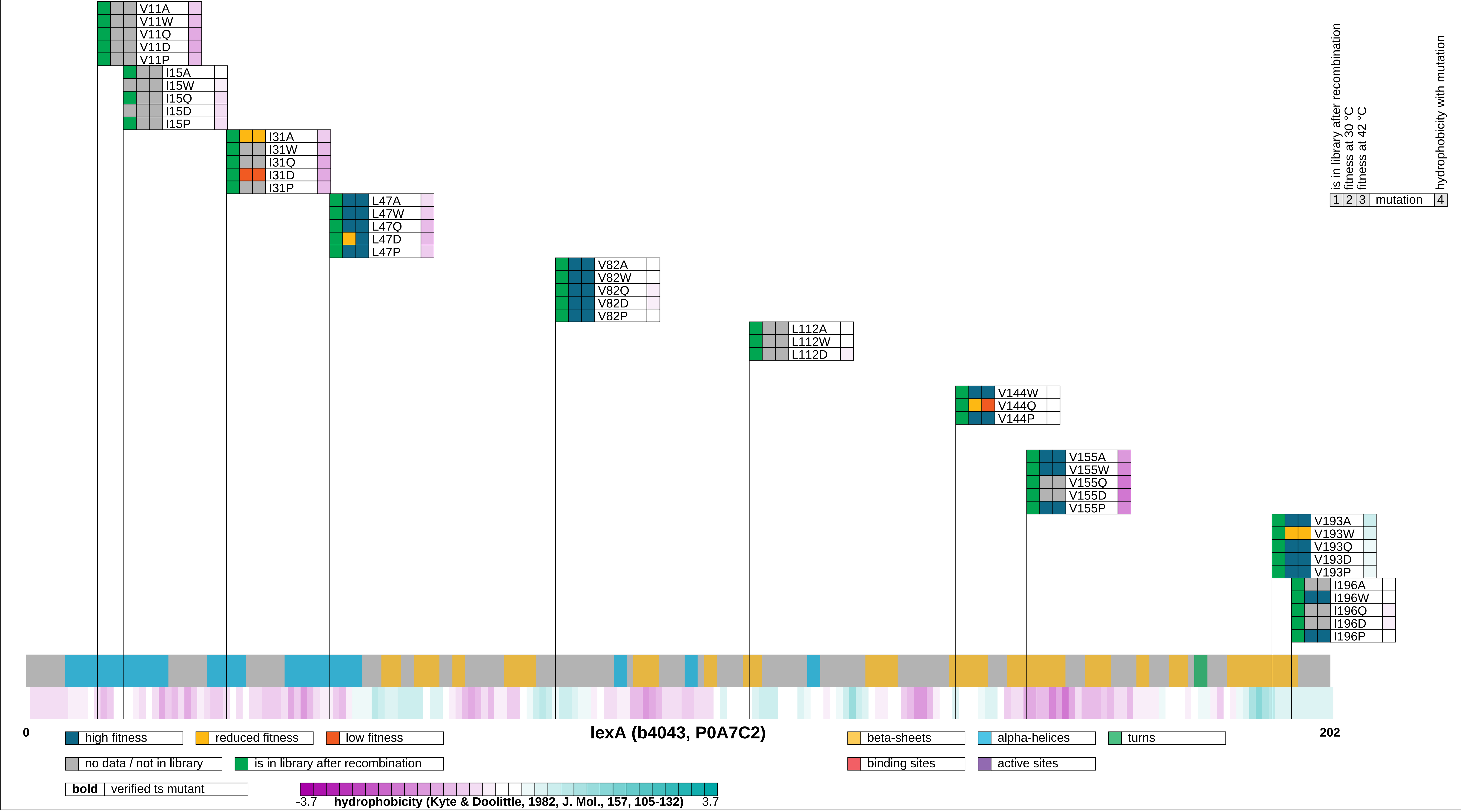

V11A
V11W
V11Q
V11D
V11P
I15A
I15W
I15Q
I15D
I15P
I31A
I31W
I31Q
I31D
I31P
L47A
L47W
L47Q
L47D
L47P
V82A
V82W
V82Q
V82D
V82P
L112A
L112W
L112D
V144W
V144Q
V144P
V155A
V155W
V155Q
V155D
V155P
V193A
V193W
V193Q
V193D
V193P
I196A
I196W
I196Q
I196D
I196P
is in library after recombination
fitness at 30 °C
fitness at 42 °C
hydrophobicity with mutation
1 2 3 mutation 4
0
lexA (b4043, P0A7C2)
202
high fitness
reduced fitness
low fitness
no data / not in library
is in library after recombination
bold verified ts mutant
beta-sheets
alpha-helices
turns
binding sites
active sites
-3.7 hydrophobicity (Kyte & Doolittle, 1982, J. Mol., 157, 105-132) 3.7

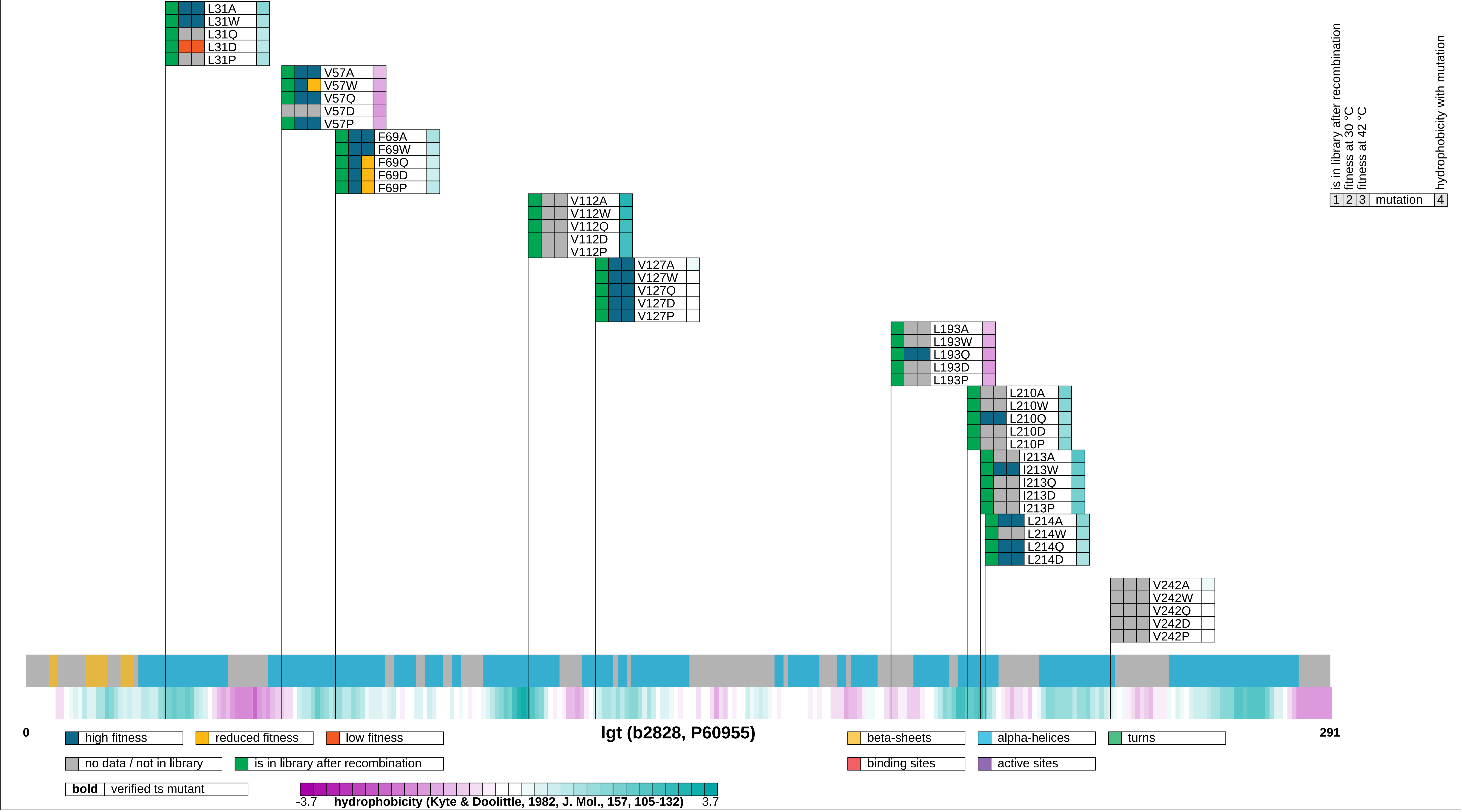

L31A
L31W
L31Q
L31D
L31P
V57A
V57W
V57Q
V57D
V57P
F69A
F69W
F69Q
F69D
F69P
V112A
V112W
V112Q
V112D
V112P
V127A
V127W
V127Q
V127D
V127P
L193A
L193W
L193Q
L193D
L193P
L210A
L210W
L210Q
L210D
L210P
I213A
I213W
I213Q
I213D
I213P
L214A
L214W
L214Q
L214D
V242A
V242W
V242Q
V242D
V242P
is in library after recombination
fitness at 30 °C
fitness at 42 °C
hydrophobicity with mutation
1 2 3 mutation 4
0
lgt (b2828, P60955)
291
high fitness
reduced fitness
low fitness
no data / not in library
is in library after recombination
bold verified ts mutant
beta-sheets
alpha-helices
turns
binding sites
active sites
-3.7
hydrophobicity (Kyte & Doolittle, 1982, J. Mol., 157, 105-132)
3.7

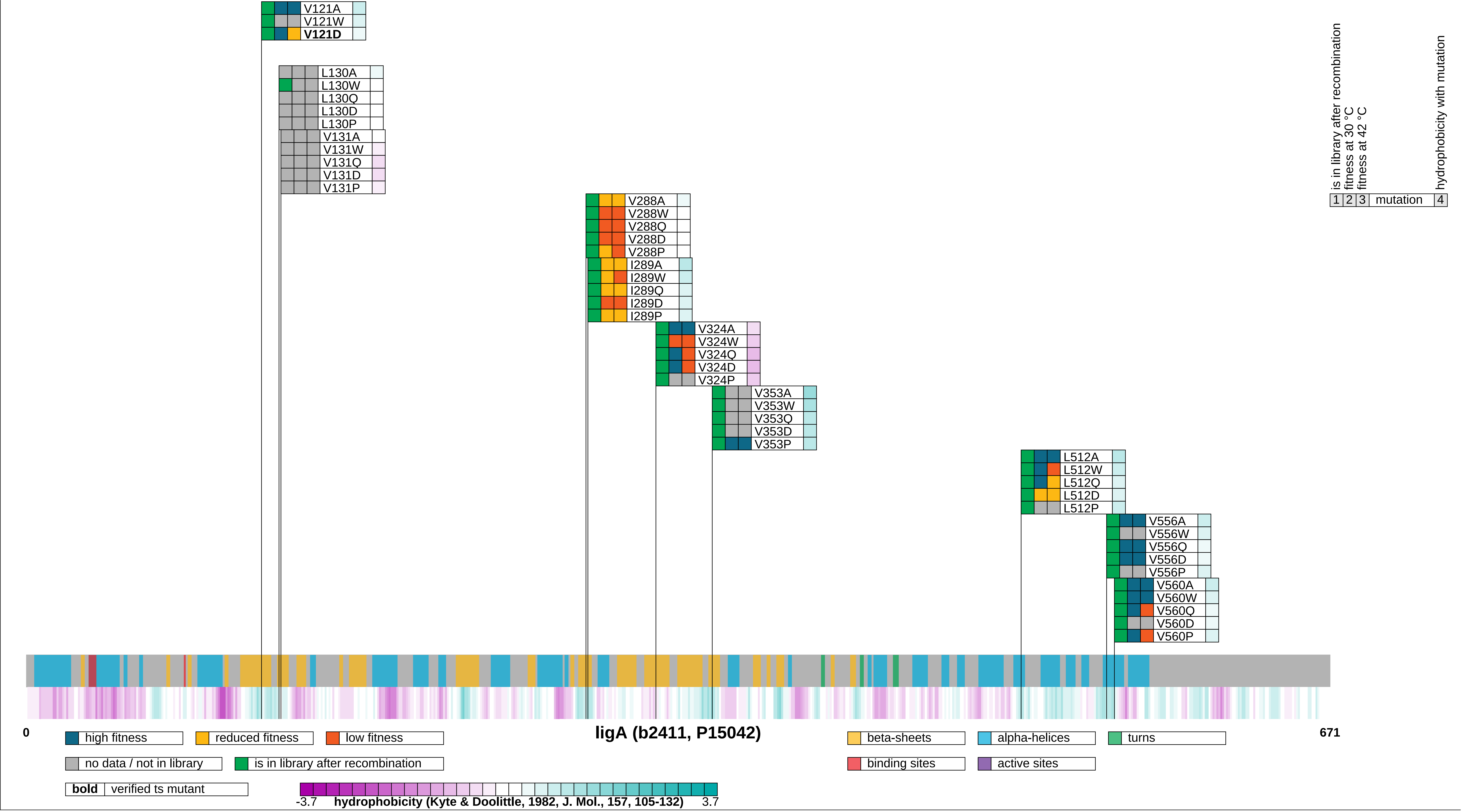

V121A
V121W
V121D
L130A
L130W
L130Q
L130D
L130P
V131A
V131W
V131Q
V131D
V131P
V288A
V288W
V288Q
V288D
V288P
I289A
I289W
I289Q
I289D
I289P
V324A
V324W
V324Q
V324D
V324P
V353A
V353W
V353Q
V353D
V353P
L512A
L512W
L512Q
L512D
L512P
V556A
V556W
V556Q
V556D
V556P
V560A
V560W
V560Q
V560D
V560P
is in library after recombination
fitness at 30 °C
fitness at 42 °C
hydrophobicity with mutation
1 2 3 mutation 4
0
ligA (b2411, P15042)
671
high fitness
reduced fitness
low fitness
beta-sheets
alpha-helices
turns
no data / not in library
is in library after recombination
binding sites
active sites
bold verified ts mutant
-3.7 hydrophobicity (Kyte & Doolittle, 1982, J. Mol., 157, 105-132) 3.7

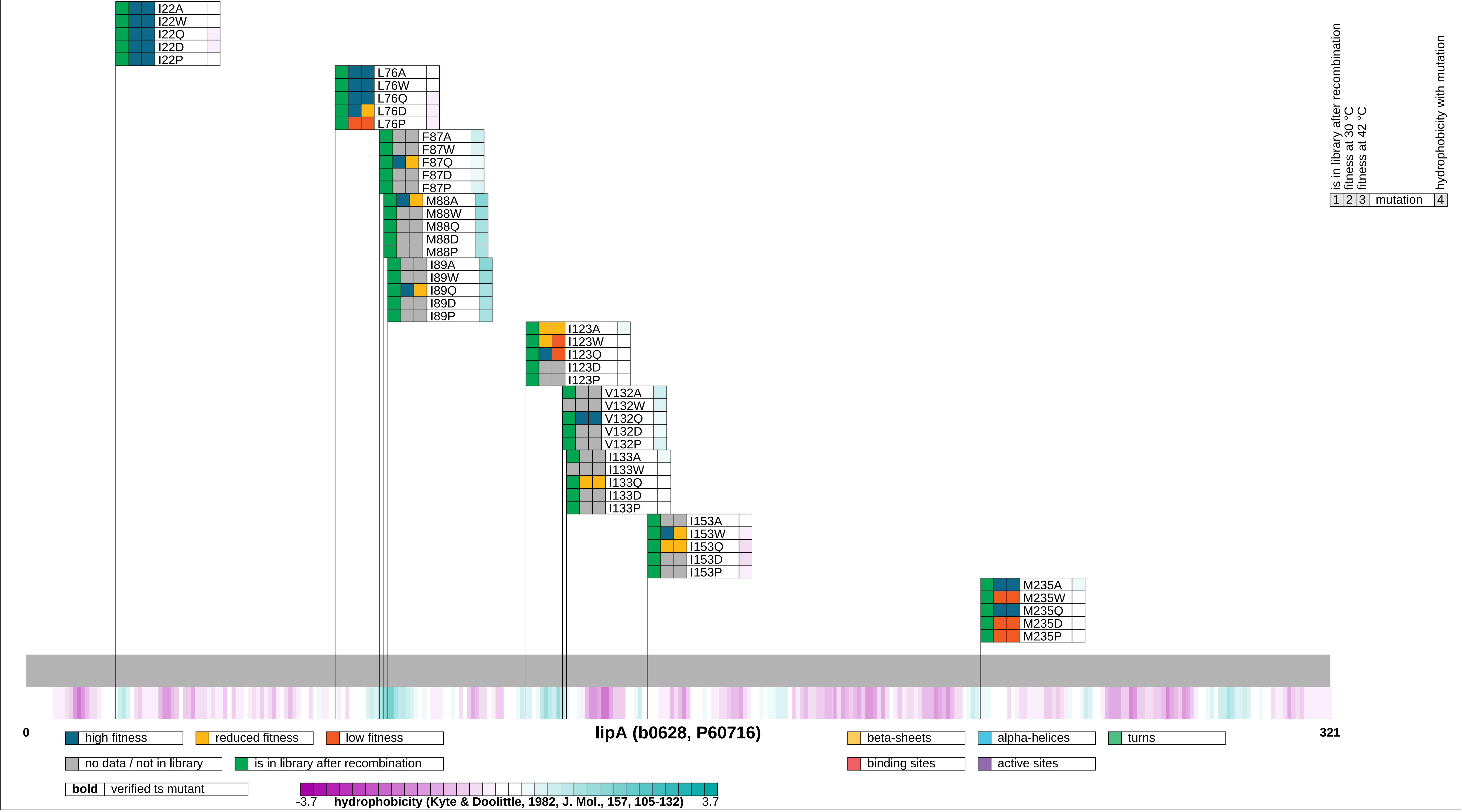

I22A
I22W
I22Q
I22D
I22P
L76A
L76W
L76Q
L76D
L76P
F87A
F87W
F87Q
F87D
F87P
M88A
M88W
M88Q
M88D
M88P
I89A
I89W
I89Q
I89D
I89P
I123A
I123W
I123Q
I123D
I123P
V132A
V132W
V132Q
V132D
V132P
I133A
I133W
I133Q
I133D
I133P
I153A
I153W
I153Q
I153D
I153P
M235A
M235W
M235Q
M235D
M235P
is in library after recombination
fitness at 30 °C
fitness at 42 °C
hydrophobicity with mutation
1
2
3
mutation
4
0
lipA (b0628, P60716)
321
high fitness
reduced fitness
low fitness
no data / not in library
is in library after recombination
bold
verified ts mutant
beta-sheets
alpha-helices
turns
binding sites
active sites
-3.7
hydrophobicity (Kyte & Doolittle, 1982, J. Mol., 157, 105-132)
3.7

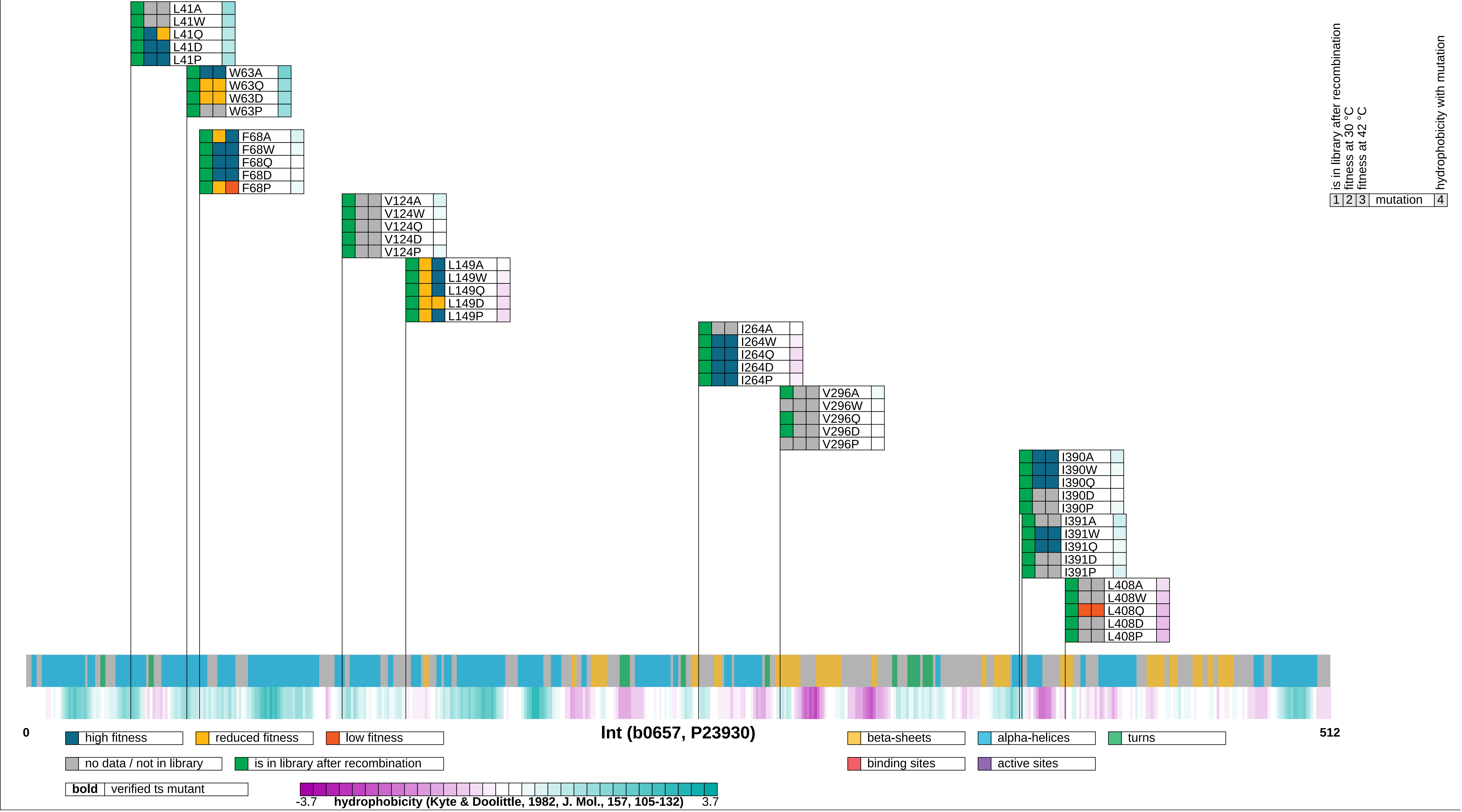

L41A
L41W
L41Q
L41D
L41P
W63A
W63Q
W63D
W63P
F68A
F68W
F68Q
F68D
F68P
V124A
V124W
V124Q
V124D
V124P
L149A
L149W
L149Q
L149D
L149P
I264A
I264W
I264Q
I264D
I264P
V296A
V296W
V296Q
V296D
V296P
I390A
I390W
I390Q
I390D
I390P
I391A
I391W
I391Q
I391D
I391P
L408A
L408W
L408Q
L408D
L408P
is in library after recombination
fitness at 30 °C
fitness at 42 °C
hydrophobicity with mutation
1 2 3 mutation 4
0
Int (b0657, P23930)
512
high fitness
reduced fitness
low fitness
beta-sheets
alpha-helices
turns
no data / not in library
is in library after recombination
binding sites
active sites
bold verified ts mutant
-3.7 hydrophobicity (Kyte & Doolittle, 1982, J. Mol., 157, 105-132) 3.7

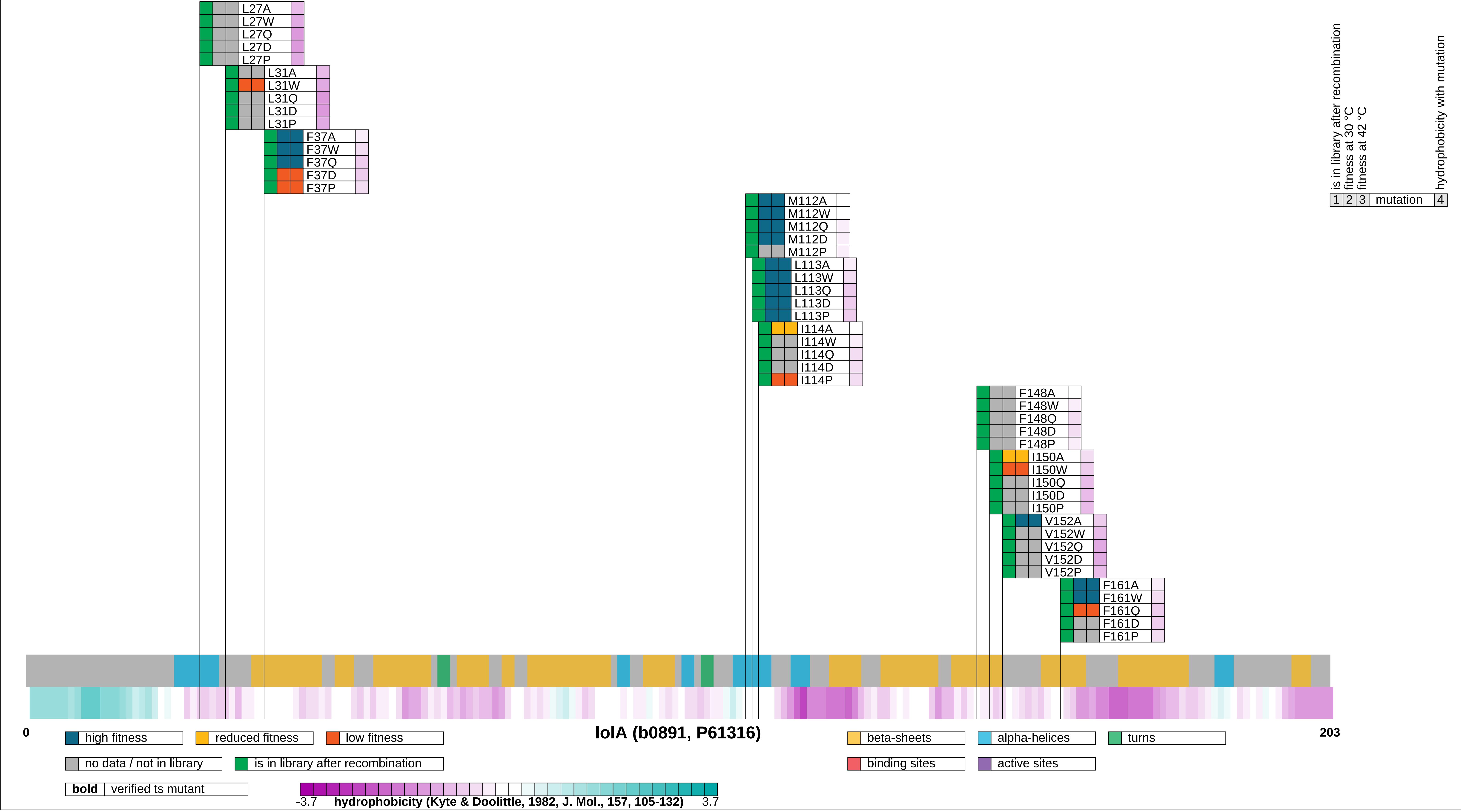

L27A
L27W
L27Q
L27D
L27P
L31A
L31W
L31Q
L31D
L31P
F37A
F37W
F37Q
F37D
F37P
M112A
M112W
M112Q
M112D
M112P
L113A
L113W
L113Q
L113D
L113P
I114A
I114W
I114Q
I114D
I114P
F148A
F148W
F148Q
F148D
F148P
I150A
I150W
I150Q
I150D
I150P
V152A
V152W
V152Q
V152D
V152P
F161A
F161W
F161Q
F161D
F161P
is in library after recombination
fitness at 30 °C
fitness at 42 °C
hydrophobicity with mutation
1 2 3 mutation 4
0
203
lolA (b0891, P61316)
high fitness
reduced fitness
low fitness
no data / not in library
is in library after recombination
bold verified ts mutant
beta-sheets
alpha-helices
turns
binding sites
active sites
-3.7
hydrophobicity (Kyte & Doolittle, 1982, J. Mol., 157, 105-132)
3.7

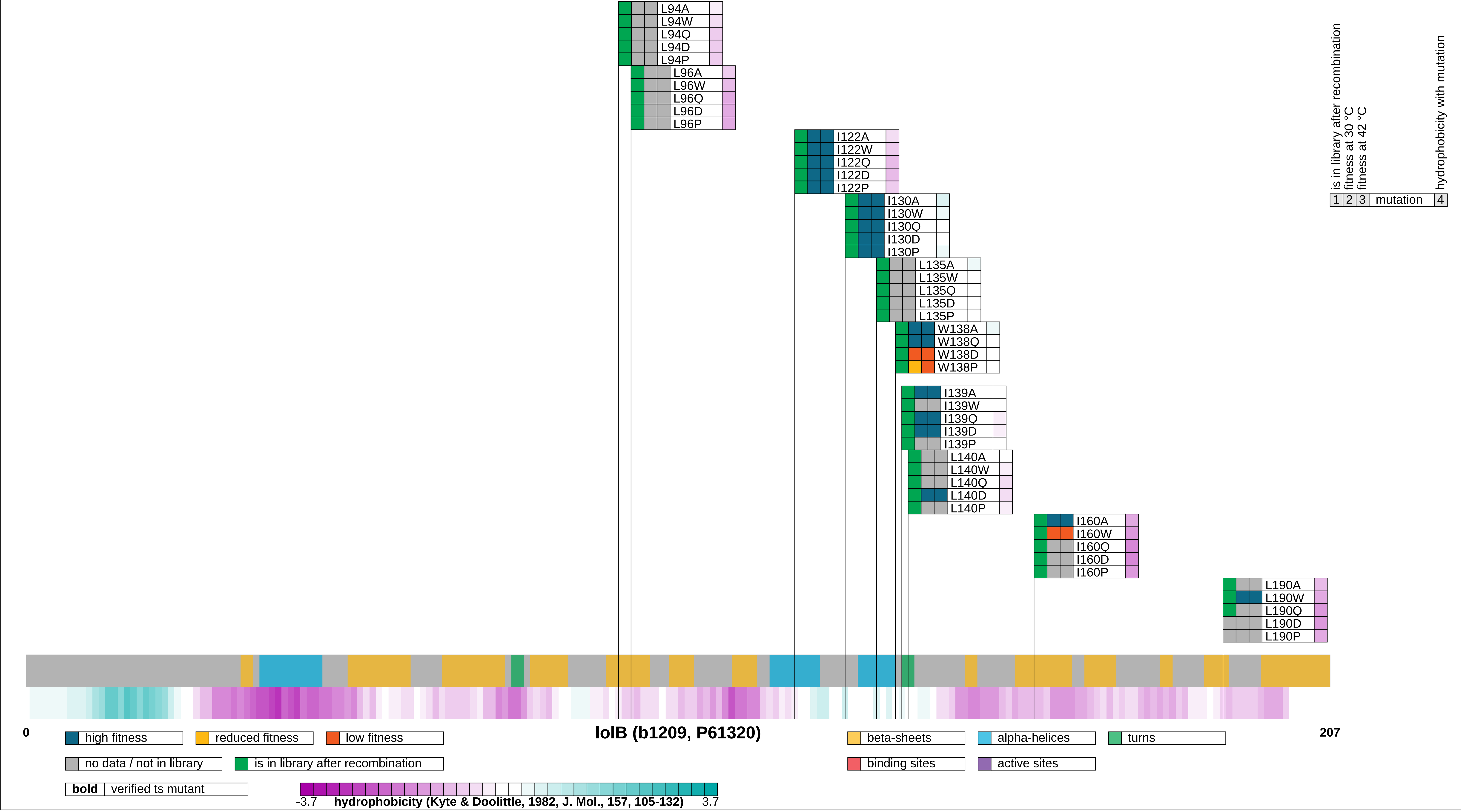

is in library after recombination
fitness at 30 °C
fitness at 42 °C
hydrophobicity with mutation
1 2 3 mutation 4
L94A
L94W
L94Q
L94D
L94P
L96A
L96W
L96Q
L96D
L96P
I122A
I122W
I122Q
I122D
I122P
I130A
I130W
I130Q
I130D
I130P
L135A
L135W
L135Q
L135D
L135P
W138A
W138Q
W138D
W138P
I139A
I139W
I139Q
I139D
I139P
L140A
L140W
L140Q
L140D
L140P
I160A
I160W
I160Q
I160D
I160P
L190A
L190W
L190Q
L190D
L190P
0
lolB (b1209, P61320)
207
high fitness
reduced fitness
low fitness
no data / not in library
is in library after recombination
bold verified ts mutant
beta-sheets
alpha-helices
turns
binding sites
active sites
-3.7
hydrophobicity (Kyte & Doolittle, 1982, J. Mol., 157, 105-132)
3.7

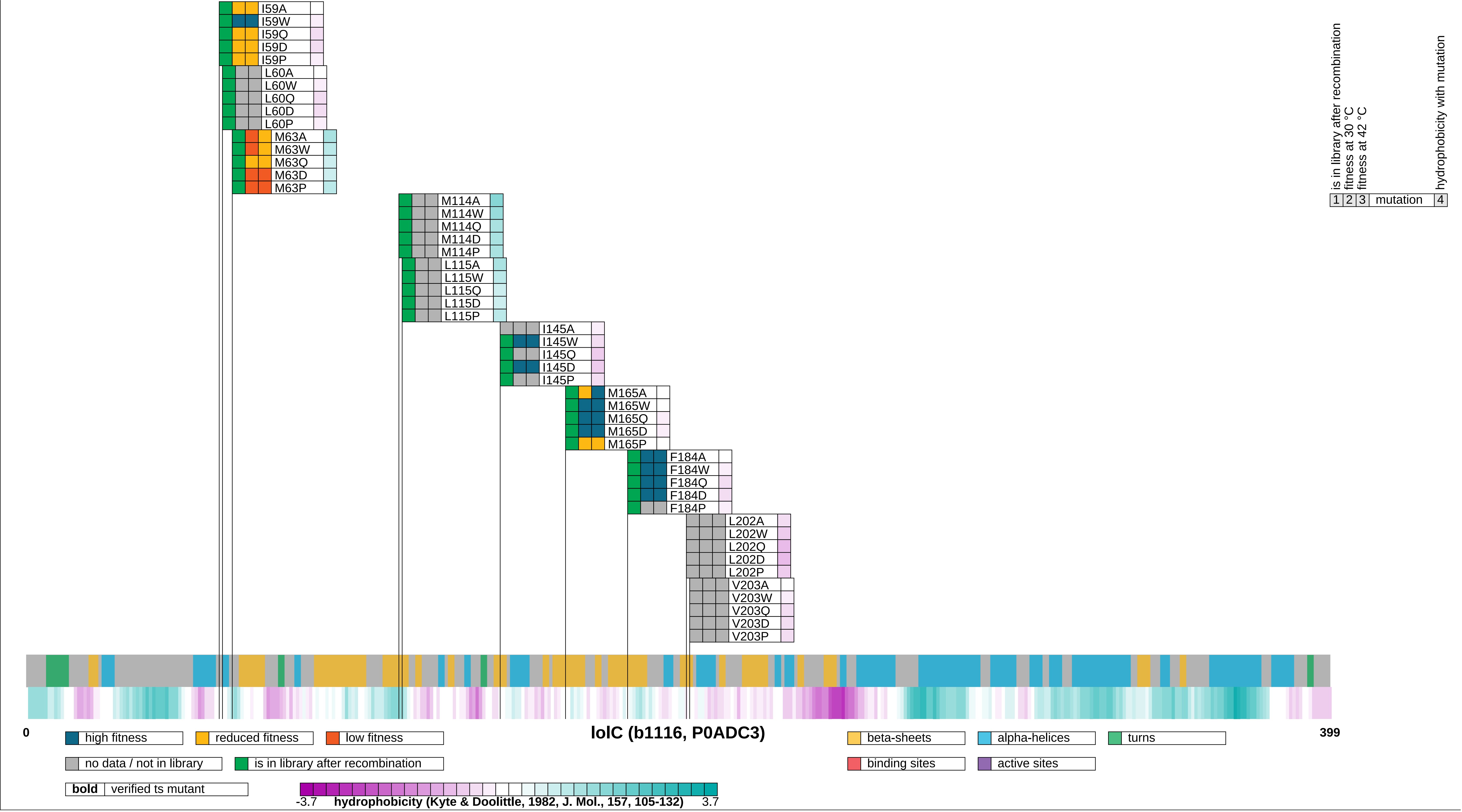

I59A
I59W
I59Q
I59D
I59P
L60A
L60W
L60Q
L60D
L60P
M63A
M63W
M63Q
M63D
M63P
M114A
M114W
M114Q
M114D
M114P
L115A
L115W
L115Q
L115D
L115P
I145A
I145W
I145Q
I145D
I145P
M165A
M165W
M165Q
M165D
M165P
F184A
F184W
F184Q
F184D
F184P
L202A
L202W
L202Q
L202D
L202P
V203A
V203W
V203Q
V203D
V203P
is in library after recombination
fitness at 30 °C
fitness at 42 °C
hydrophobicity with mutation
1 2 3 mutation 4
0
lolC (b1116, P0ADC3)
399
high fitness
reduced fitness
low fitness
no data / not in library
is in library after recombination
bold verified ts mutant
beta-sheets
alpha-helices
turns
binding sites
active sites
-3.7 hydrophobicity (Kyte & Doolittle, 1982, J. Mol., 157, 105-132) 3.7

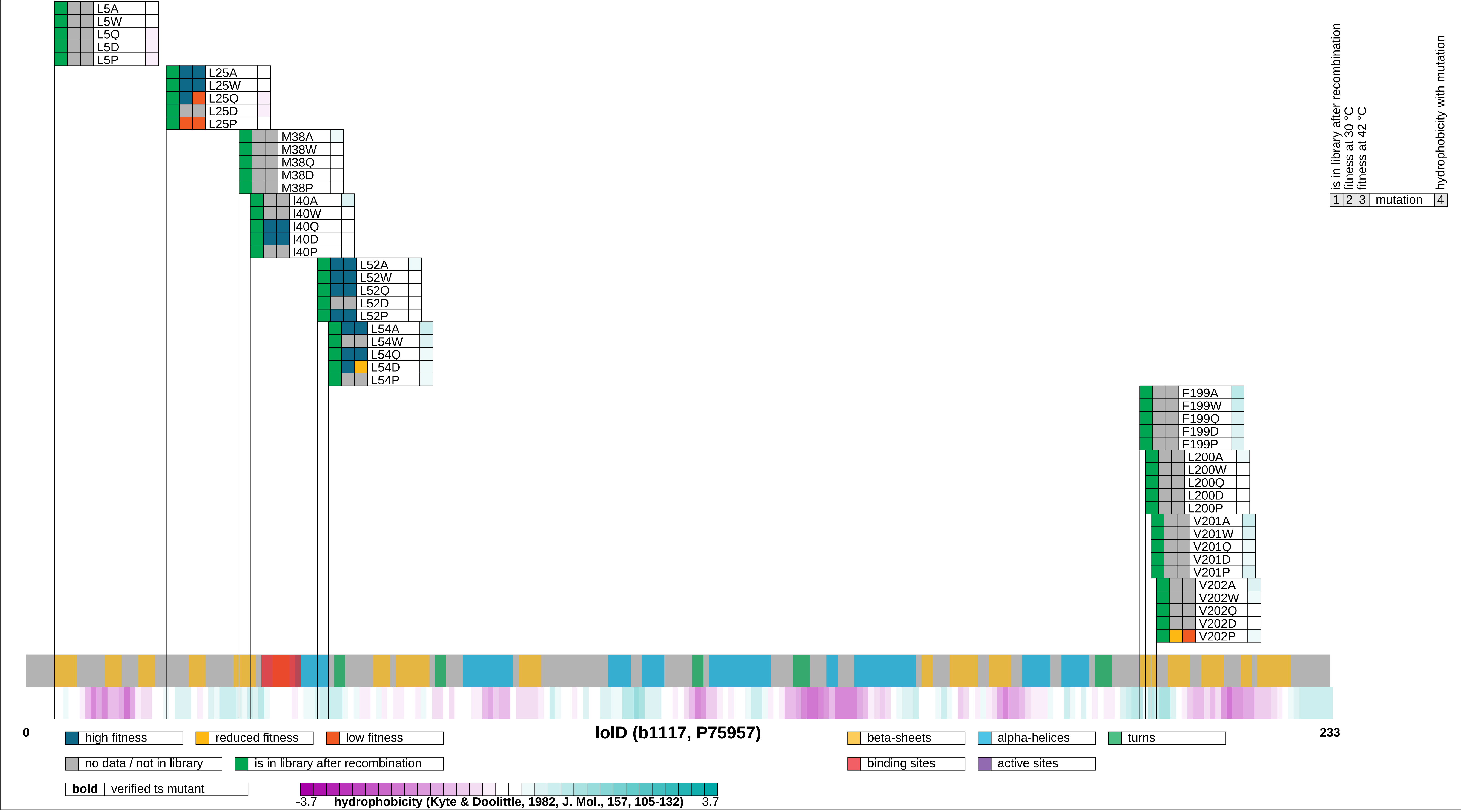

L5A
L5W
L5Q
L5D
L5P
L25A
L25W
L25Q
L25D
L25P
M38A
M38W
M38Q
M38D
M38P
I40A
I40W
I40Q
I40D
I40P
L52A
L52W
L52Q
L52D
L52P
L54A
L54W
L54Q
L54D
L54P
F199A
F199W
F199Q
F199D
F199P
L200A
L200W
L200Q
L200D
L200P
V201A
V201W
V201Q
V201D
V201P
V202A
V202W
V202Q
V202D
V202P
is in library after recombination
fitness at 30 °C
fitness at 42 °C
hydrophobicity with mutation
1 2 3 mutation 4
0
lolD (b1117, P75957)
233
high fitness
reduced fitness
low fitness
no data / not in library
is in library after recombination
bold verified ts mutant
beta-sheets
alpha-helices
turns
binding sites
active sites
-3.7
hydrophobicity (Kyte & Doolittle, 1982, J. Mol., 157, 105-132)
3.7

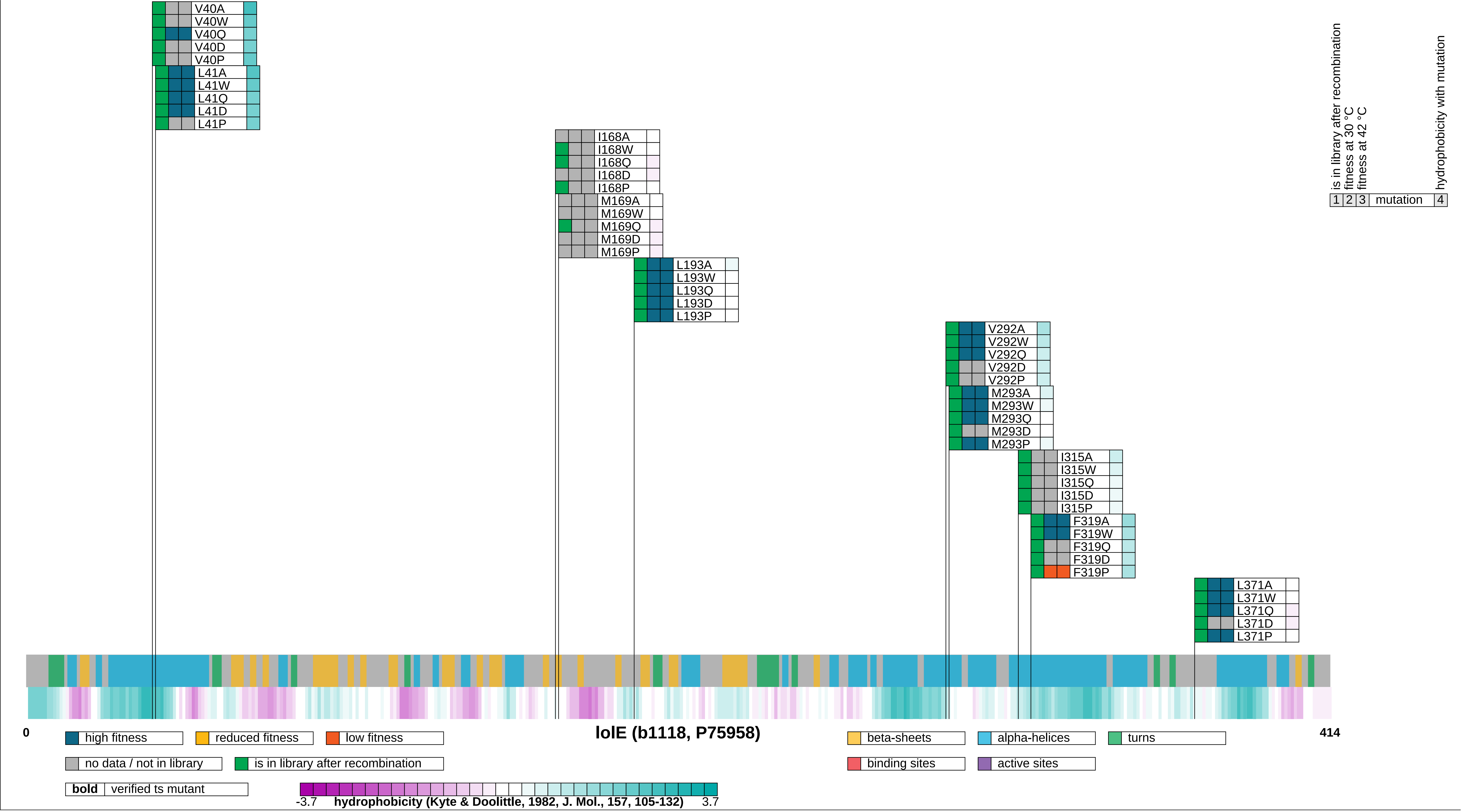
V40A
V40W
V40Q
V40D
V40P
L41A
L41W
L41Q
L41D
L41P
I168A
I168W
I168Q
I168D
I168P
M169A
M169W
M169Q
M169D
M169P
L193A
L193W
L193Q
L193D
L193P
V292A
V292W
V292Q
V292D
V292P
M293A
M293W
M293Q
M293D
M293P
I315A
I315W
I315Q
I315D
I315P
F319A
F319W
F319Q
F319D
F319P
L371A
L371W
L371Q
L371D
L371P
is in library after recombination
fitness at 30 °C
fitness at 42 °C
hydrophobicity with mutation
1 2 3 mutation 4
0
lolE (b1118, P75958)
414
high fitness
reduced fitness
low fitness
beta-sheets
alpha-helices
turns
no data / not in library
is in library after recombination
binding sites
active sites
bold verified ts mutant
-3.7 hydrophobicity (Kyte & Doolittle, 1982, J. Mol., 157, 105-132) 3.7

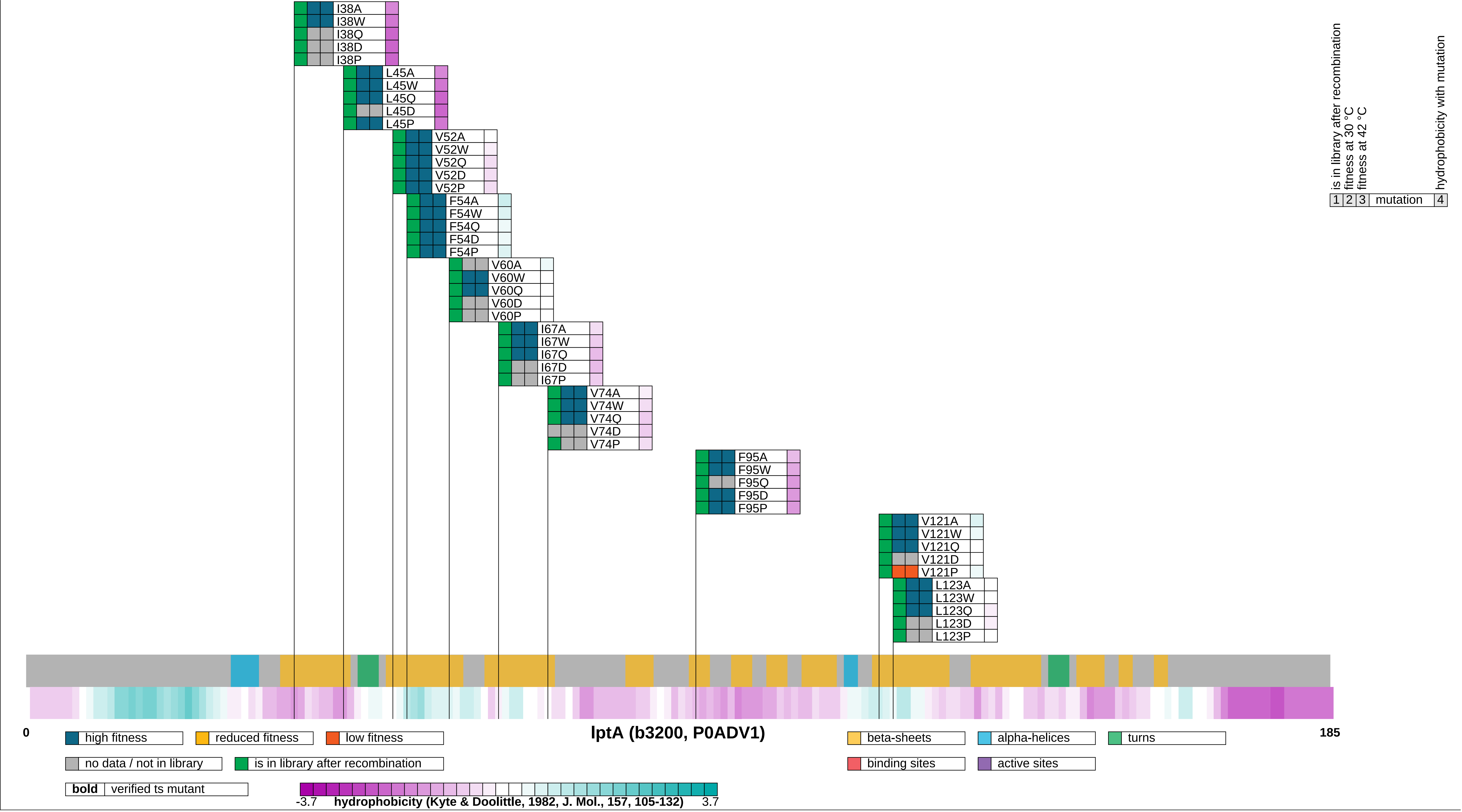

is in library after recombination
fitness at 30 °C
fitness at 42 °C
hydrophobicity with mutation
1 2 3 mutation 4
I38A
I38W
I38Q
I38D
I38P
L45A
L45W
L45Q
L45D
L45P
V52A
V52W
V52Q
V52D
V52P
F54A
F54W
F54Q
F54D
F54P
V60A
V60W
V60Q
V60D
V60P
I67A
I67W
I67Q
I67D
I67P
V74A
V74W
V74Q
V74D
V74P
F95A
F95W
F95Q
F95D
F95P
V121A
V121W
V121Q
V121D
V121P
L123A
L123W
L123Q
L123D
L123P
0
lptA (b3200, P0ADV1)
185
high fitness
reduced fitness
low fitness
beta-sheets
alpha-helices
turns
no data / not in library
is in library after recombination
binding sites
active sites
bold verified ts mutant
-3.7
hydrophobicity (Kyte & Doolittle, 1982, J. Mol., 157, 105-132)
3.7

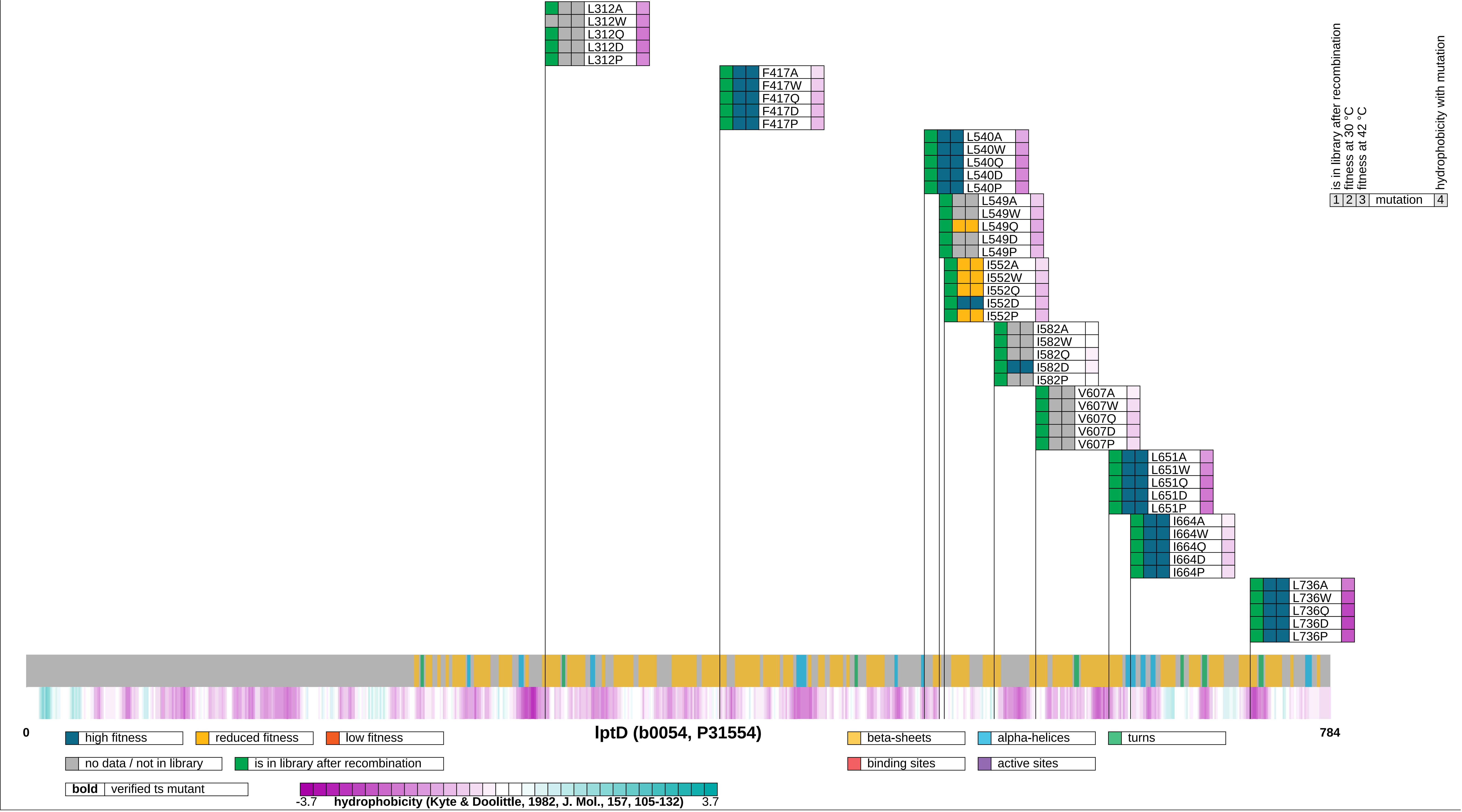

is in library after recombination
fitness at 30 °C
fitness at 42 °C
hydrophobicity with mutation
1 2 3 mutation 4
L312A
L312W
L312Q
L312D
L312P
F417A
F417W
F417Q
F417D
F417P
L540A
L540W
L540Q
L540D
L540P
L549A
L549W
L549Q
L549D
L549P
I552A
I552W
I552Q
I552D
I552P
I582A
I582W
I582Q
I582D
I582P
V607A
V607W
V607Q
V607D
V607P
L651A
L651W
L651Q
L651D
L651P
I664A
I664W
I664Q
I664D
I664P
L736A
L736W
L736Q
L736D
L736P
0
lptD (b0054, P31554)
784
high fitness
reduced fitness
low fitness
beta-sheets
alpha-helices
turns
no data / not in library
is in library after recombination
binding sites
active sites
bold verified ts mutant
-3.7
hydrophobicity (Kyte & Doolittle, 1982, J. Mol., 157, 105-132)
3.7

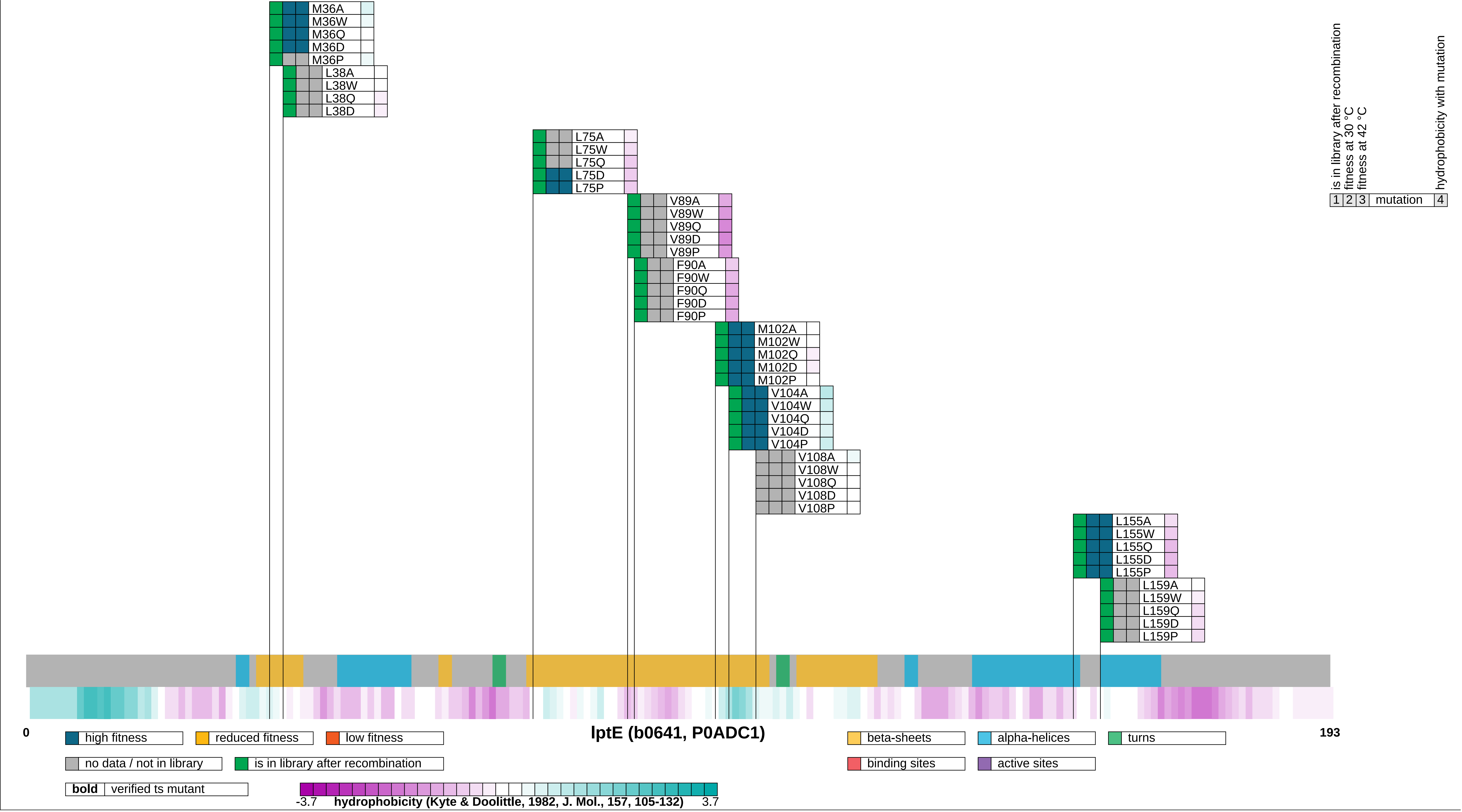

M36A
M36W
M36Q
M36D
M36P
L38A
L38W
L38Q
L38D
L75A
L75W
L75Q
L75D
L75P
V89A
V89W
V89Q
V89D
V89P
F90A
F90W
F90Q
F90D
F90P
M102A
M102W
M102Q
M102D
M102P
V104A
V104W
V104Q
V104D
V104P
V108A
V108W
V108Q
V108D
V108P
L155A
L155W
L155Q
L155D
L155P
L159A
L159W
L159Q
L159D
L159P
is in library after recombination
fitness at 30 °C
fitness at 42 °C
hydrophobicity with mutation
1
2
3
mutation
4
0
lptE (b0641, P0ADC1)
193
high fitness
reduced fitness
low fitness
no data / not in library
is in library after recombination
bold
verified ts mutant
beta-sheets
alpha-helices
turns
binding sites
active sites
-3.7
hydrophobicity (Kyte & Doolittle, 1982, J. Mol., 157, 105-132)
3.7

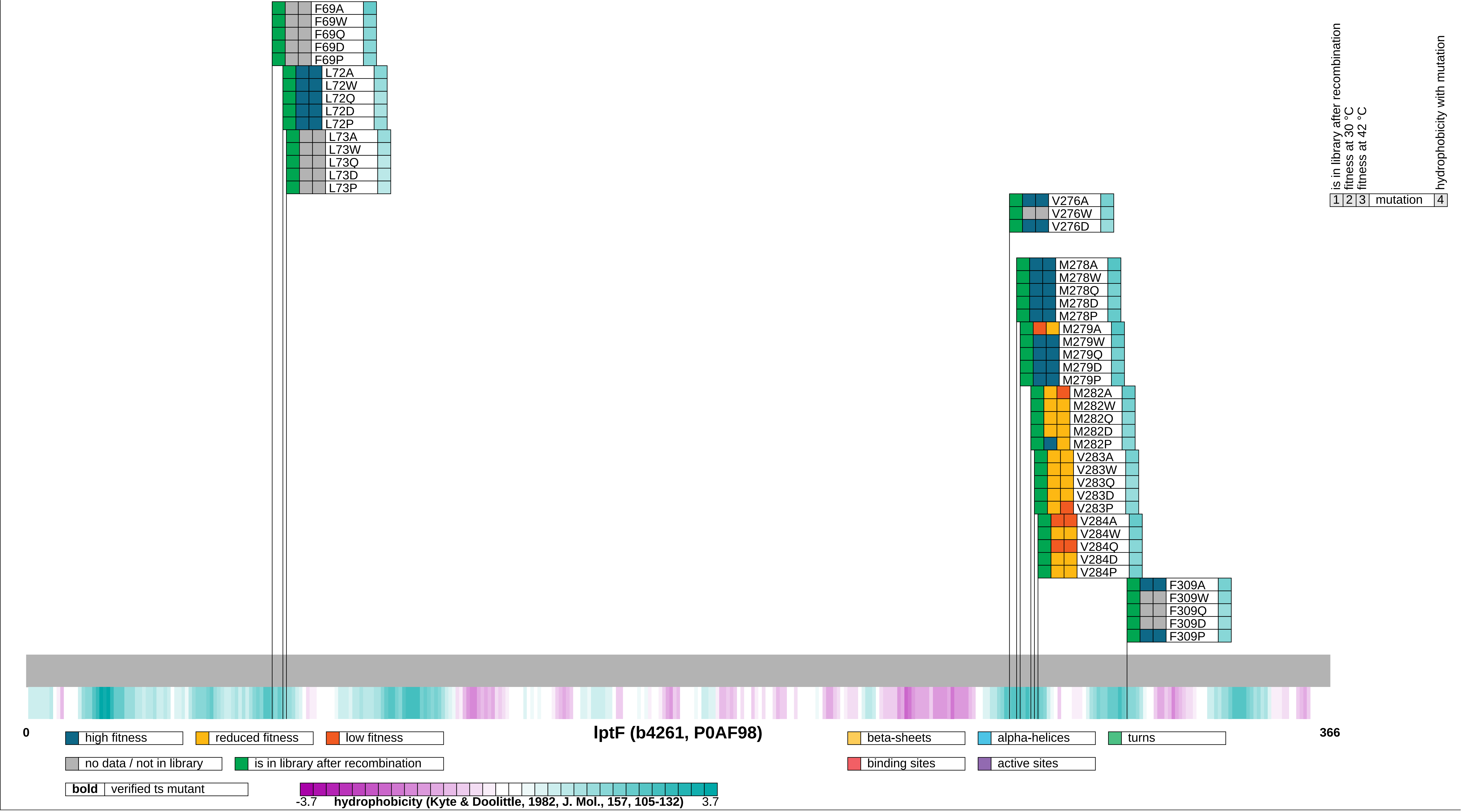
is in library after recombination
fitness at 30 °C
fitness at 42 °C
hydrophobicity with mutation
1 2 3 mutation 4
F69A
F69W
F69Q
F69D
F69P
L72A
L72W
L72Q
L72D
L72P
L73A
L73W
L73Q
L73D
L73P
V276A
V276W
V276D
M278A
M278W
M278Q
M278D
M278P
M279A
M279W
M279Q
M279D
M279P
M282A
M282W
M282Q
M282D
M282P
V283A
V283W
V283Q
V283D
V283P
V284A
V284W
V284Q
V284D
V284P
F309A
F309W
F309Q
F309D
F309P
0
lptF (b4261, P0AF98)
366
high fitness
reduced fitness
low fitness
beta-sheets
alpha-helices
turns
no data / not in library
is in library after recombination
binding sites
active sites
bold verified ts mutant
-3.7
hydrophobicity (Kyte & Doolittle, 1982, J. Mol., 157, 105-132)
3.7

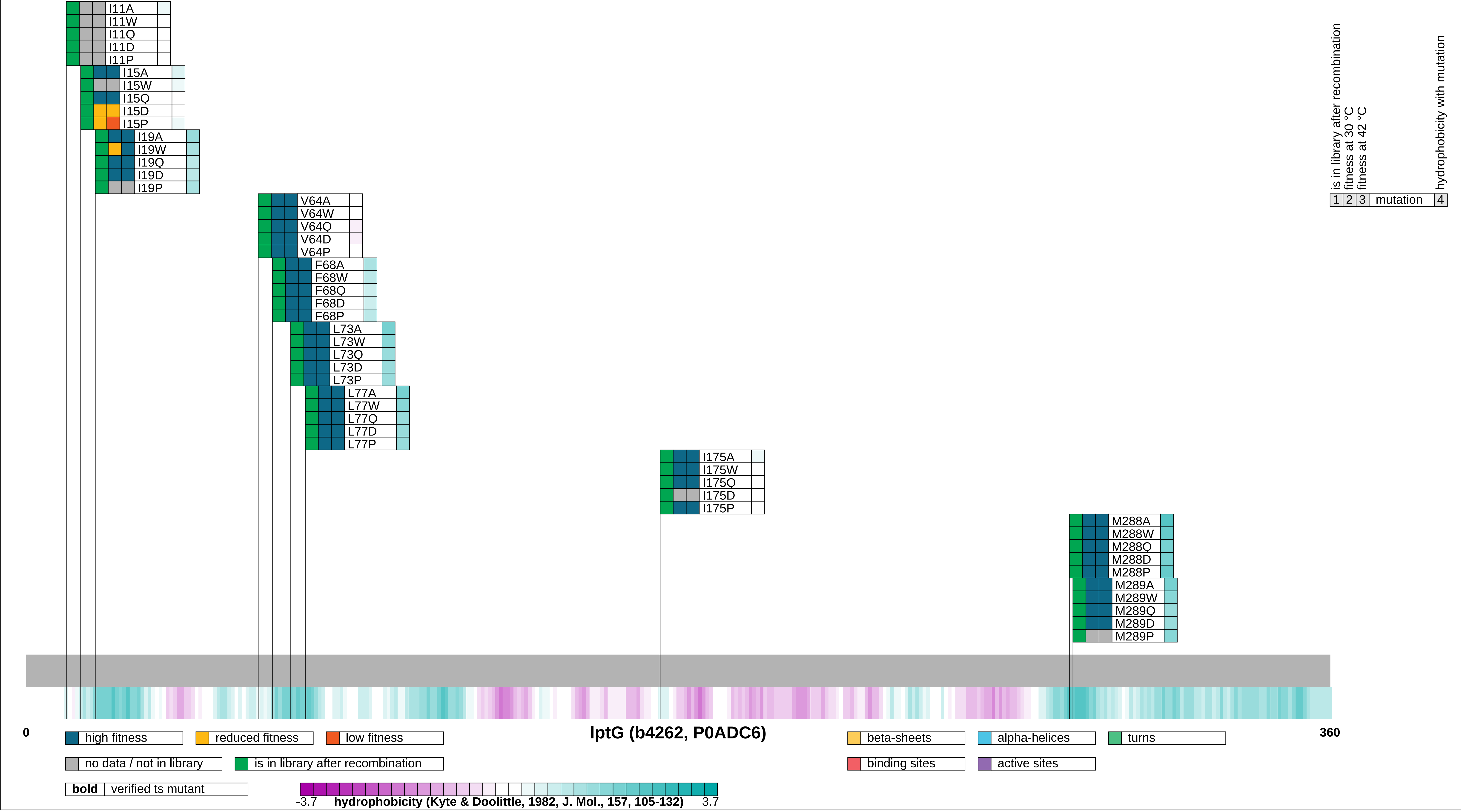

I11A
I11W
I11Q
I11D
I11P
I15A
I15W
I15Q
I15D
I15P
I19A
I19W
I19Q
I19D
I19P
V64A
V64W
V64Q
V64D
V64P
F68A
F68W
F68Q
F68D
F68P
L73A
L73W
L73Q
L73D
L73P
L77A
L77W
L77Q
L77D
L77P
I175A
I175W
I175Q
I175D
I175P
M288A
M288W
M288Q
M288D
M288P
M289A
M289W
M289Q
M289D
M289P
is in library after recombination
fitness at 30 °C
fitness at 42 °C
hydrophobicity with mutation
1 2 3 mutation 4
0
360
lptG (b4262, P0ADC6)
high fitness
reduced fitness
low fitness
beta-sheets
alpha-helices
turns
no data / not in library
is in library after recombination
binding sites
active sites
bold verified ts mutant
-3.7
hydrophobicity (Kyte & Doolittle, 1982, J. Mol., 157, 105-132)
3.7

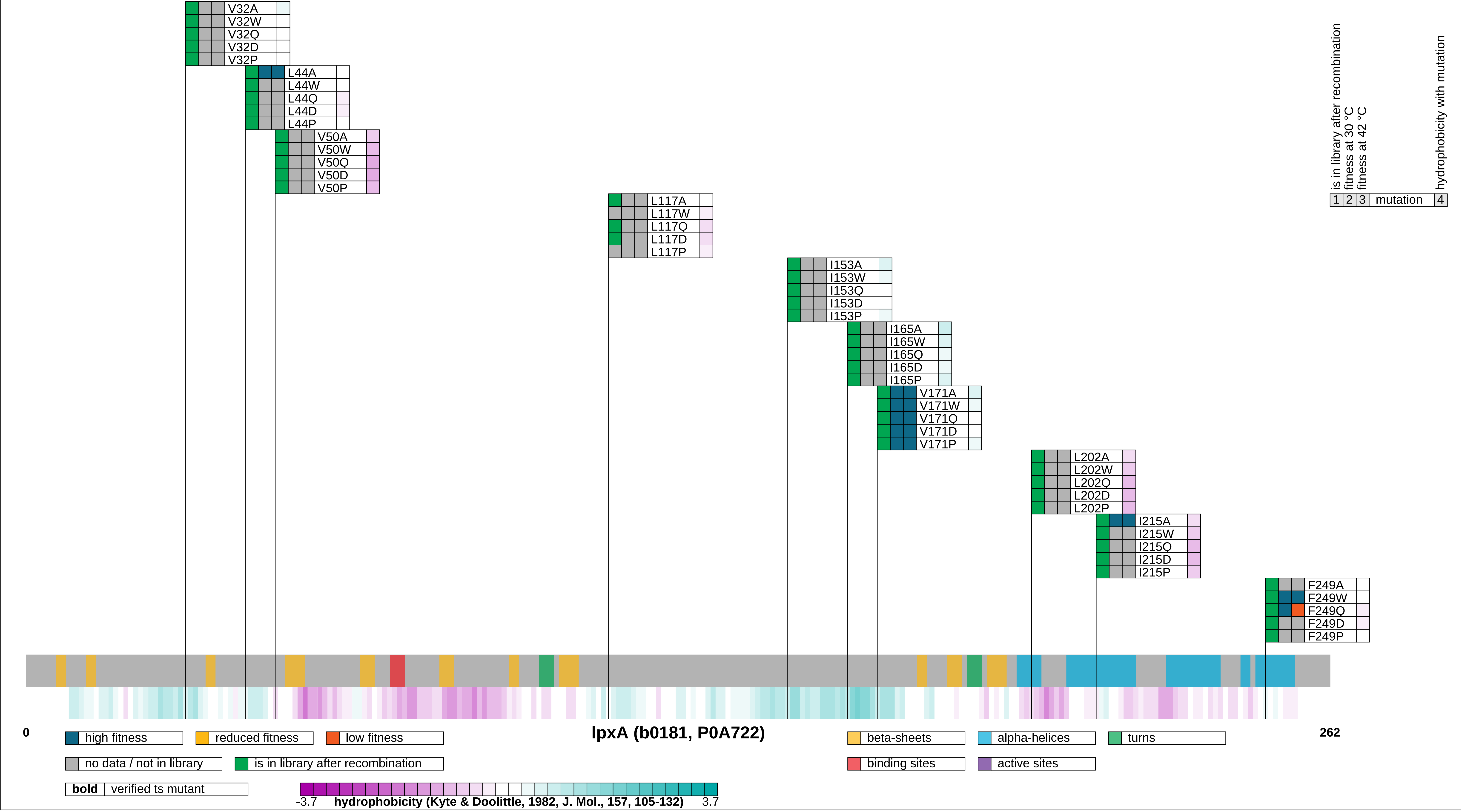

V32A
V32W
V32Q
V32D
V32P
L44A
L44W
L44Q
L44D
L44P
V50A
V50W
V50Q
V50D
V50P
L117A
L117W
L117Q
L117D
L117P
I153A
I153W
I153Q
I153D
I153P
I165A
I165W
I165Q
I165D
I165P
V171A
V171W
V171Q
V171D
V171P
L202A
L202W
L202Q
L202D
L202P
I215A
I215W
I215Q
I215D
I215P
F249A
F249W
F249Q
F249D
F249P
is in library after recombination
fitness at 30 °C
fitness at 42 °C
hydrophobicity with mutation
1 2 3 mutation 4
0
lpxA (b0181, P0A722)
262
high fitness
reduced fitness
low fitness
no data / not in library
is in library after recombination
bold verified ts mutant
beta-sheets
alpha-helices
turns
binding sites
active sites
-3.7
hydrophobicity (Kyte & Doolittle, 1982, J. Mol., 157, 105-132)
3.7

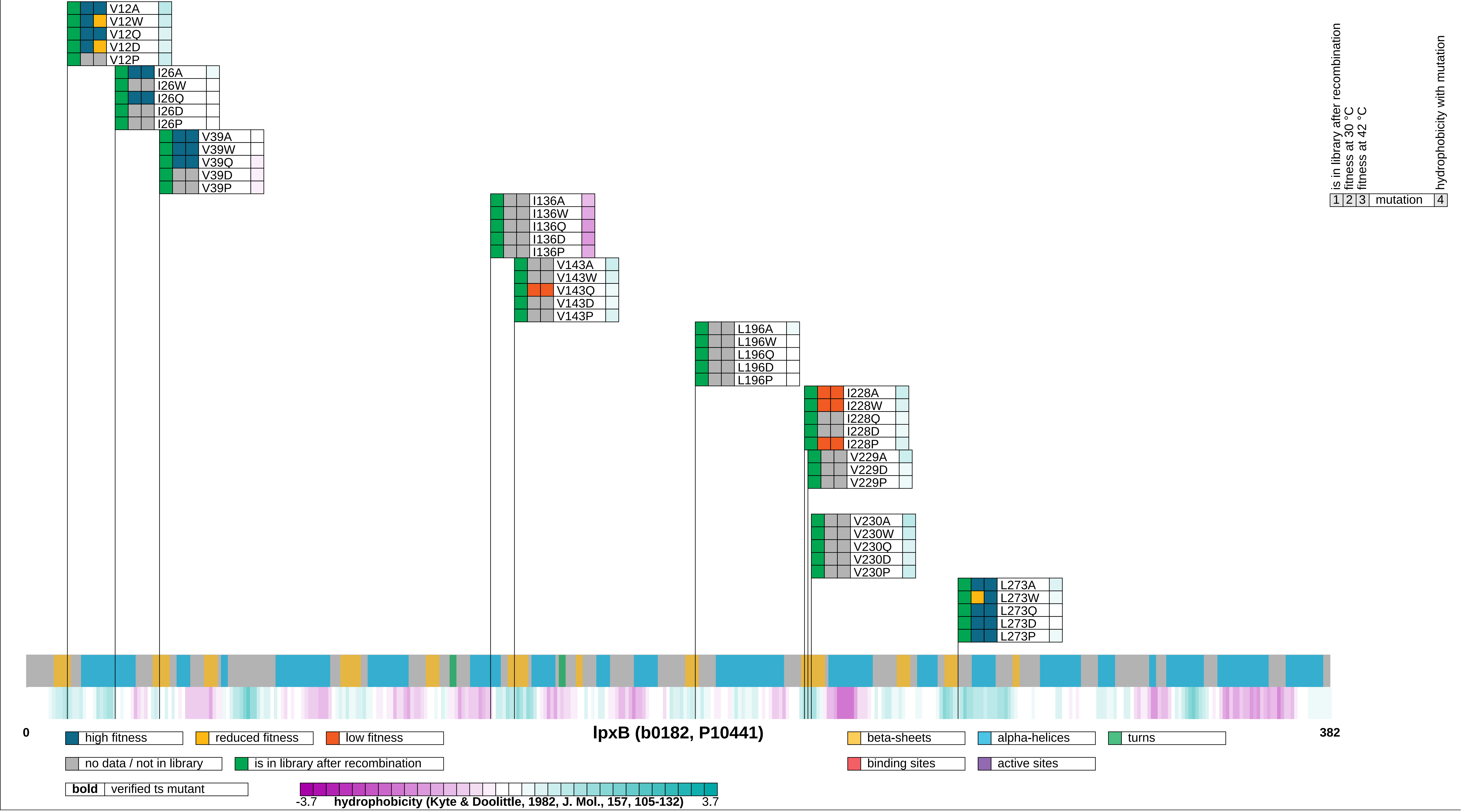

V12A
V12W
V12Q
V12D
V12P
I26A
I26W
I26Q
I26D
I26P
V39A
V39W
V39Q
V39D
V39P
I136A
I136W
I136Q
I136D
I136P
V143A
V143W
V143Q
V143D
V143P
L196A
L196W
L196Q
L196D
L196P
I228A
I228W
I228Q
I228D
I228P
V229A
V229D
V229P
V230A
V230W
V230Q
V230D
V230P
L273A
L273W
L273Q
L273D
L273P
is in library after recombination
fitness at 30 °C
fitness at 42 °C
hydrophobicity with mutation
1 2 3 mutation 4
0
lpxB (b0182, P10441)
382
high fitness
reduced fitness
low fitness
no data / not in library
is in library after recombination
bold verified ts mutant
beta-sheets
alpha-helices
turns
binding sites
active sites
-3.7 hydrophobicity (Kyte & Doolittle, 1982, J. Mol., 157, 105-132) 3.7

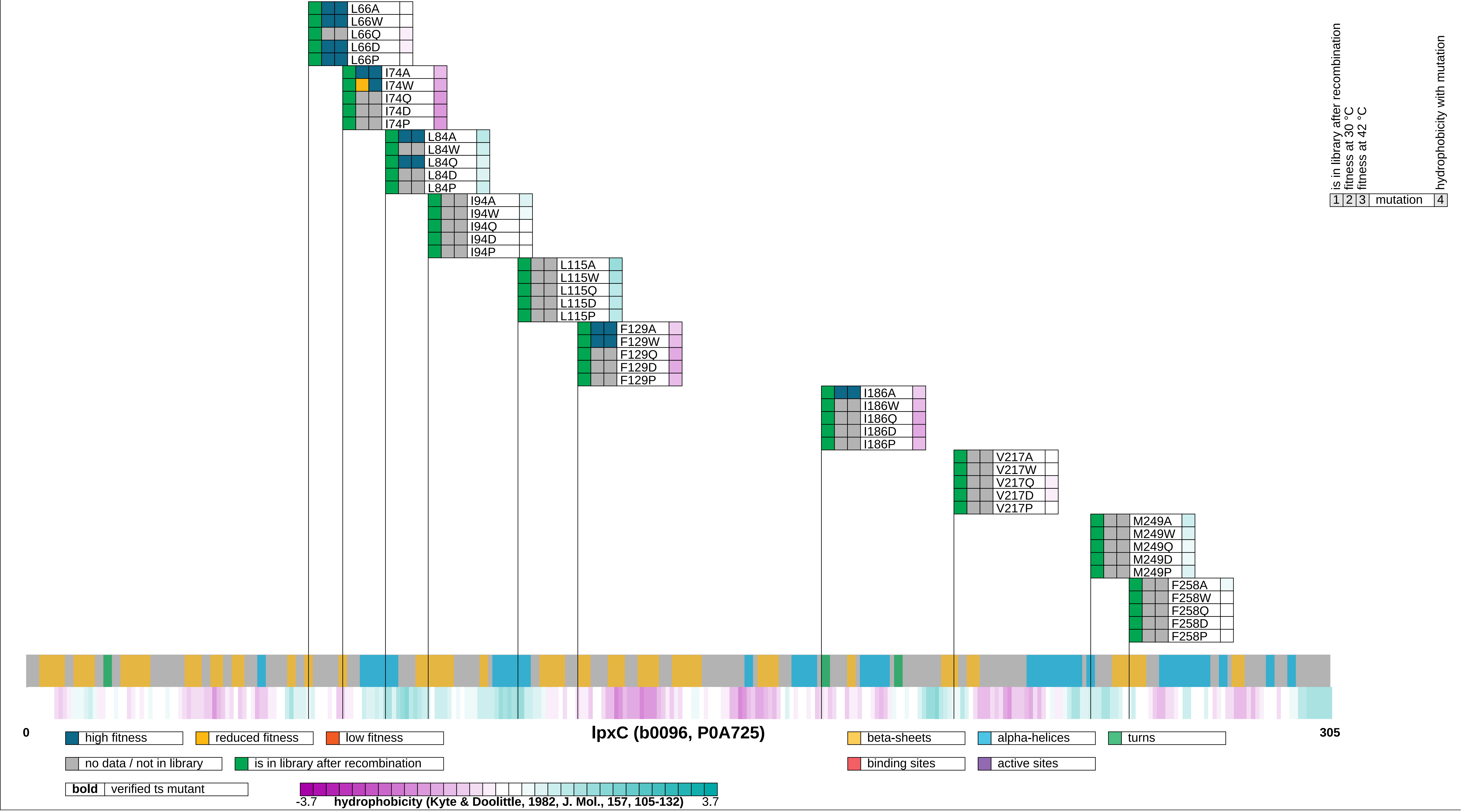
L66A
L66W
L66Q
L66D
L66P
I74A
I74W
I74Q
I74D
I74P
L84A
L84W
L84Q
L84D
L84P
I94A
I94W
I94Q
I94D
I94P
L115A
L115W
L115Q
L115D
L115P
F129A
F129W
F129Q
F129D
F129P
I186A
I186W
I186Q
I186D
I186P
V217A
V217W
V217Q
V217D
V217P
M249A
M249W
M249Q
M249D
M249P
F258A
F258W
F258Q
F258D
F258P
is in library after recombination
fitness at 30 °C
fitness at 42 °C
hydrophobicity with mutation
1 2 3 mutation 4
0
lpxC (b0096, P0A725)
305
high fitness
reduced fitness
low fitness
beta-sheets
alpha-helices
turns
no data / not in library
is in library after recombination
binding sites
active sites
bold verified ts mutant
-3.7
hydrophobicity (Kyte & Doolittle, 1982, J. Mol., 157, 105-132)
3.7

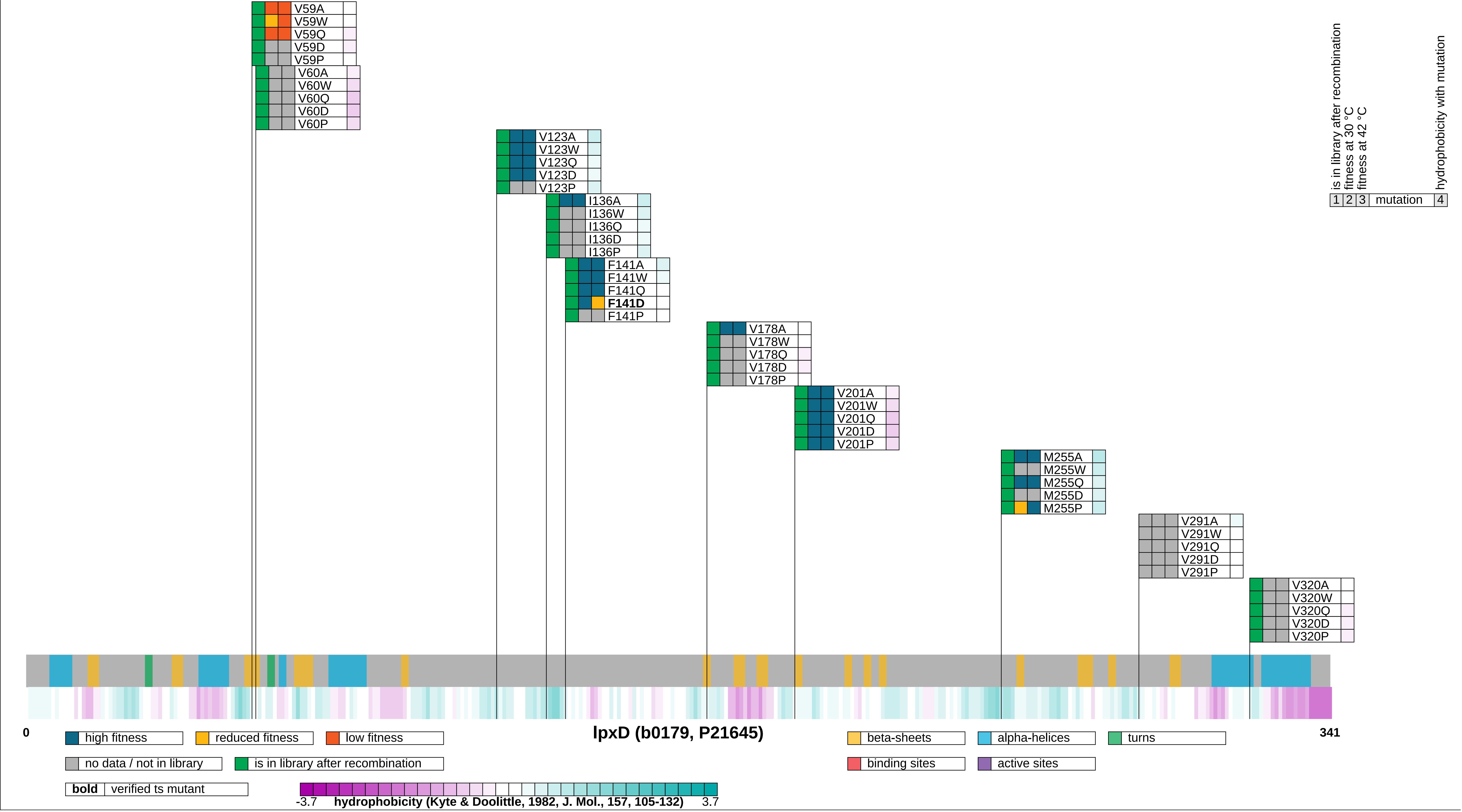
V59A
V59W
V59Q
V59D
V59P
V60A
V60W
V60Q
V60D
V60P
V123A
V123W
V123Q
V123D
V123P
I136A
I136W
I136Q
I136D
I136P
F141A
F141W
F141Q
F141D
F141P
V178A
V178W
V178Q
V178D
V178P
V201A
V201W
V201Q
V201D
V201P
M255A
M255W
M255Q
M255D
M255P
V291A
V291W
V291Q
V291D
V291P
V320A
V320W
V320Q
V320D
V320P
is in library after recombination
fitness at 30 °C
fitness at 42 °C
hydrophobicity with mutation
1 2 3 mutation 4
0
lpxD (b0179, P21645)
341
high fitness
reduced fitness
low fitness
beta-sheets
alpha-helices
turns
no data / not in library
is in library after recombination
binding sites
active sites
bold verified ts mutant
-3.7 hydrophobicity (Kyte & Doolittle, 1982, J. Mol., 157, 105-132) 3.7

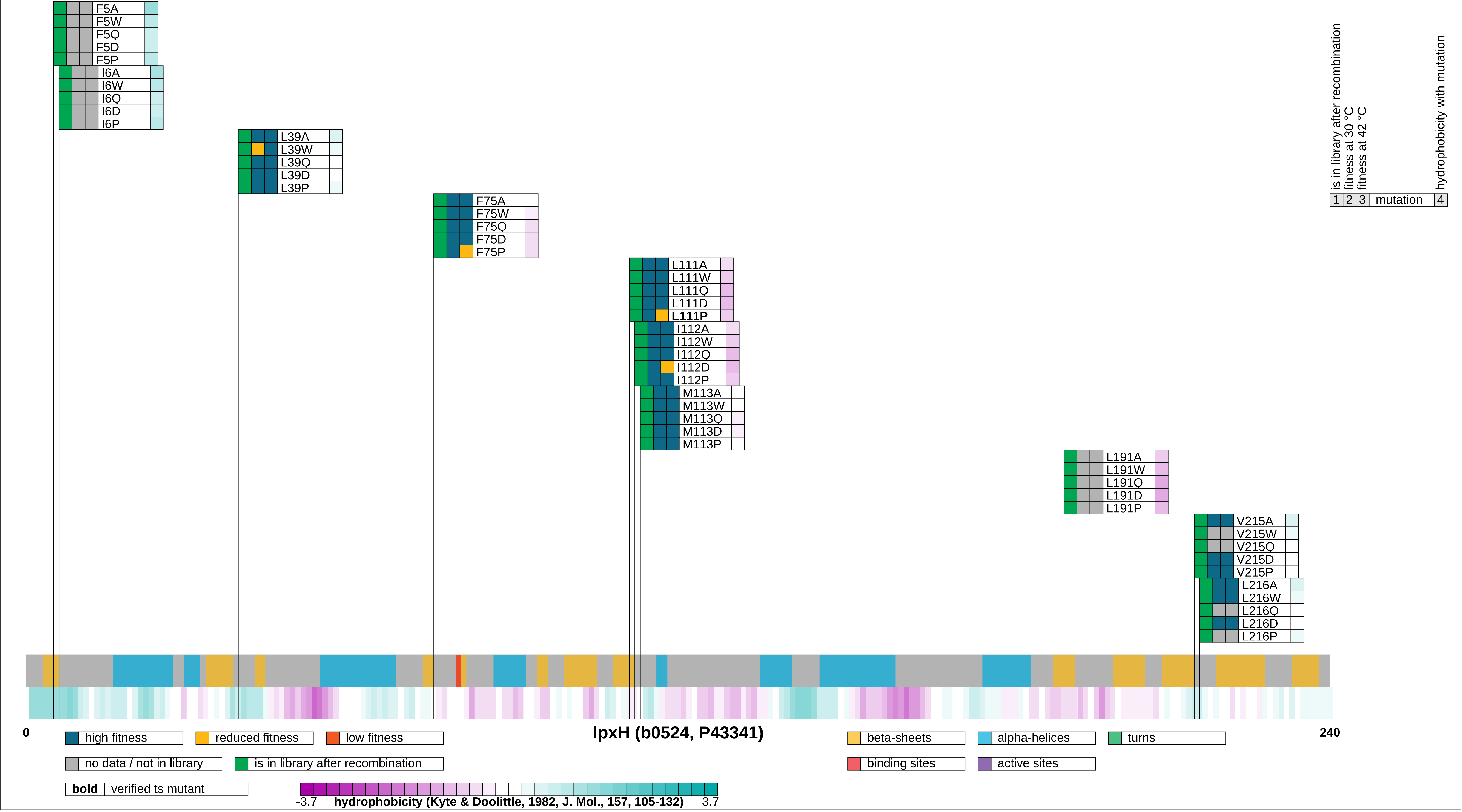

F5A
F5W
F5Q
F5D
F5P
I6A
I6W
I6Q
I6D
I6P
L39A
L39W
L39Q
L39D
L39P
F75A
F75W
F75Q
F75D
F75P
L111A
L111W
L111Q
L111D
L111P
I112A
I112W
I112Q
I112D
I112P
M113A
M113W
M113Q
M113D
M113P
L191A
L191W
L191Q
L191D
L191P
V215A
V215W
V215Q
V215D
V215P
L216A
L216W
L216Q
L216D
L216P
is in library after recombination
fitness at 30 °C
fitness at 42 °C
hydrophobicity with mutation
1 2 3 mutation 4
0
lpxH (b0524, P43341)
240
high fitness
reduced fitness
low fitness
no data / not in library
is in library after recombination
bold verified ts mutant
beta-sheets
alpha-helices
turns
binding sites
active sites
-3.7
hydrophobicity (Kyte & Doolittle, 1982, J. Mol., 157, 105-132)
3.7

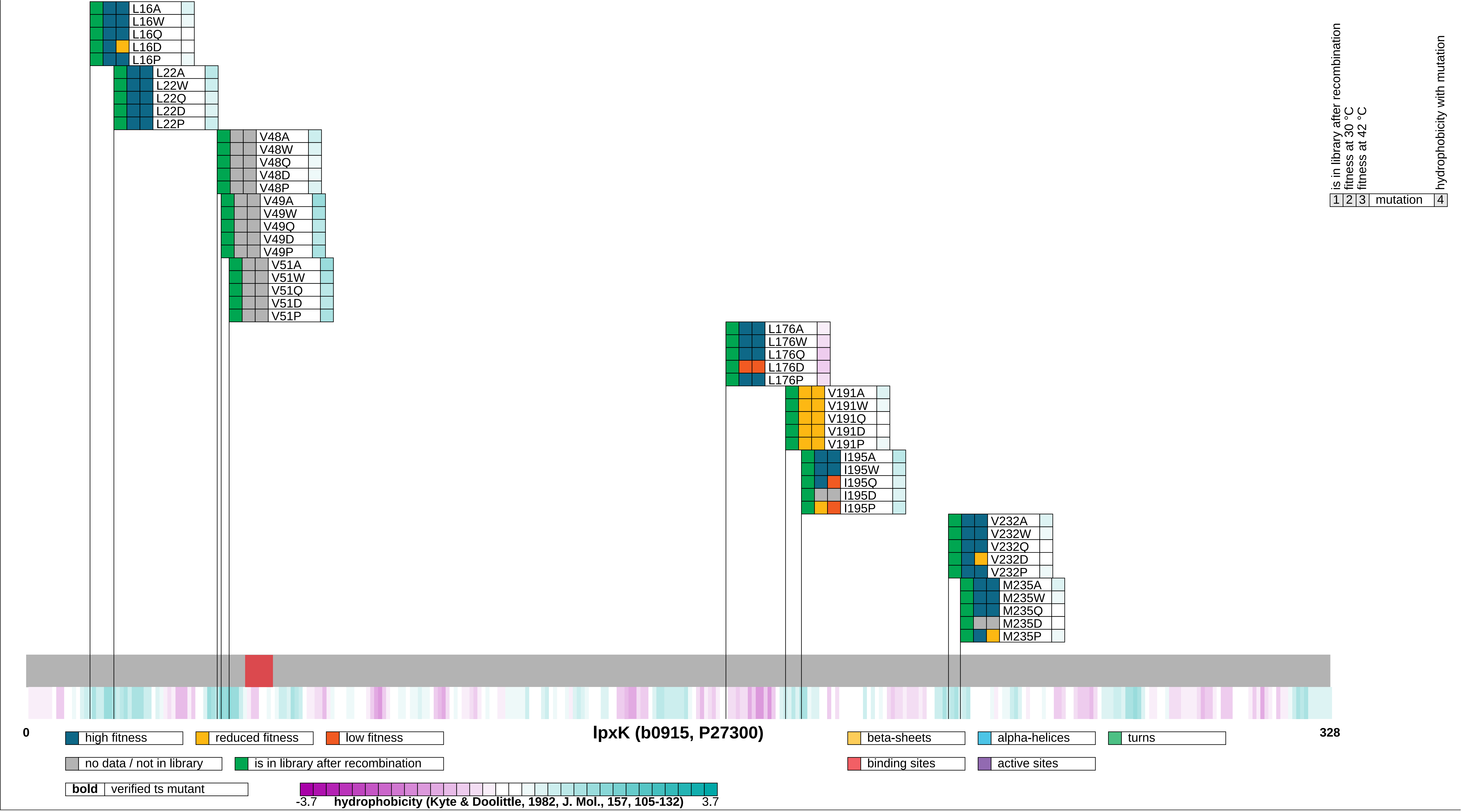
L16A
L16W
L16Q
L16D
L16P
L22A
L22W
L22Q
L22D
L22P
V48A
V48W
V48Q
V48D
V48P
V49A
V49W
V49Q
V49D
V49P
V51A
V51W
V51Q
V51D
V51P
L176A
L176W
L176Q
L176D
L176P
V191A
V191W
V191Q
V191D
V191P
I195A
I195W
I195Q
I195D
I195P
V232A
V232W
V232Q
V232D
V232P
M235A
M235W
M235Q
M235D
M235P
is in library after recombination
fitness at 30 °C
fitness at 42 °C
hydrophobicity with mutation
1 2 3 mutation 4
0
lpxK (b0915, P27300)
328
high fitness
reduced fitness
low fitness
no data / not in library
is in library after recombination
bold verified ts mutant
beta-sheets
alpha-helices
turns
binding sites
active sites
-3.7 hydrophobicity (Kyte & Doolittle, 1982, J. Mol., 157, 105-132) 3.7

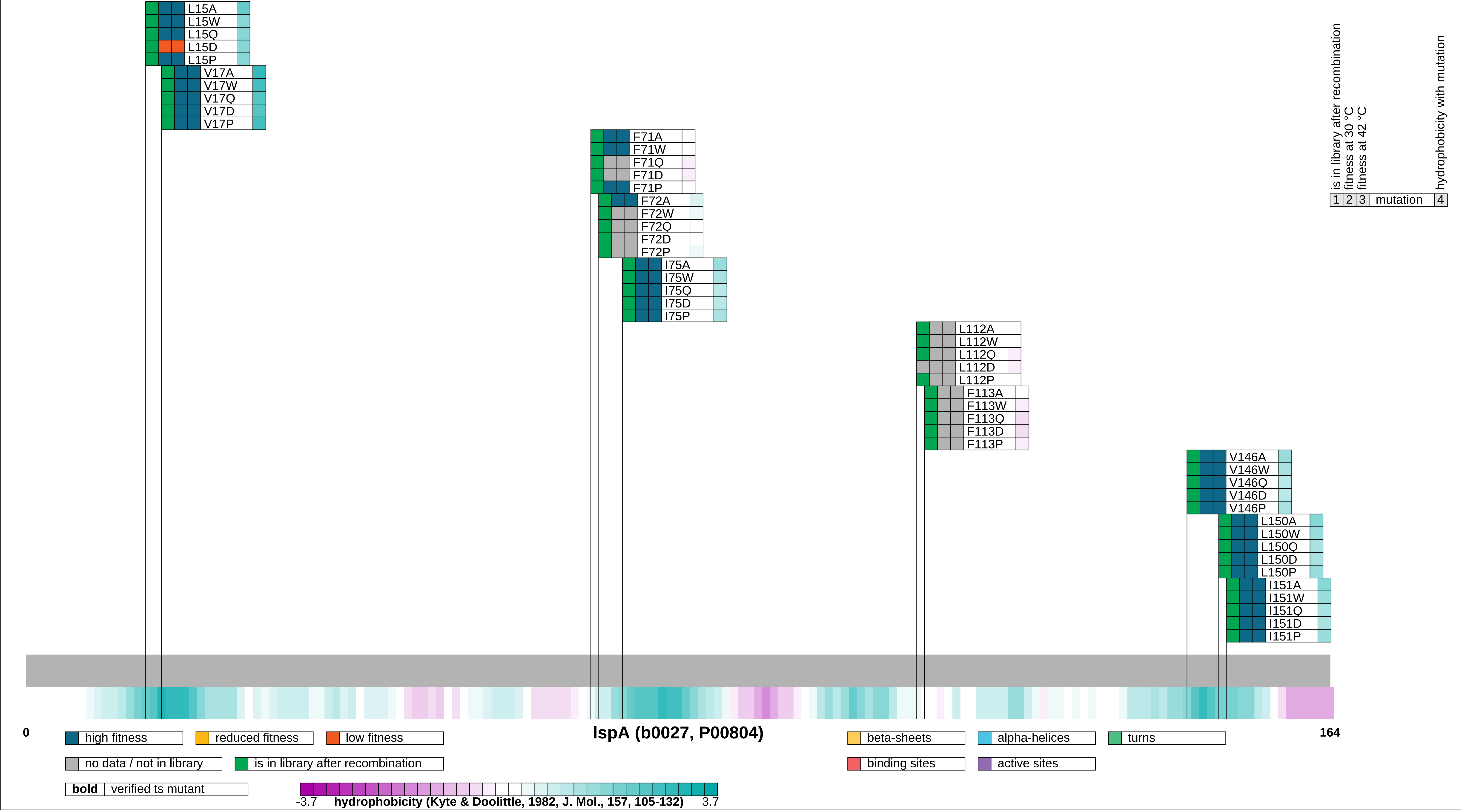

L15A
L15W
L15Q
L15D
L15P
V17A
V17W
V17Q
V17D
V17P
F71A
F71W
F71Q
F71D
F71P
F72A
F72W
F72Q
F72D
F72P
I75A
I75W
I75Q
I75D
I75P
L112A
L112W
L112Q
L112D
L112P
F113A
F113W
F113Q
F113D
F113P
V146A
V146W
V146Q
V146D
V146P
L150A
L150W
L150Q
L150D
L150P
I151A
I151W
I151Q
I151D
I151P
is in library after recombination
fitness at 30 °C
fitness at 42 °C
hydrophobicity with mutation
1 2 3 mutation 4
0
IspA (b0027, P00804)
164
high fitness
reduced fitness
low fitness
beta-sheets
alpha-helices
turns
no data / not in library
is in library after recombination
binding sites
active sites
bold verified ts mutant
-3.7 hydrophobicity (Kyte & Doolittle, 1982, J. Mol., 157, 105-132) 3.7

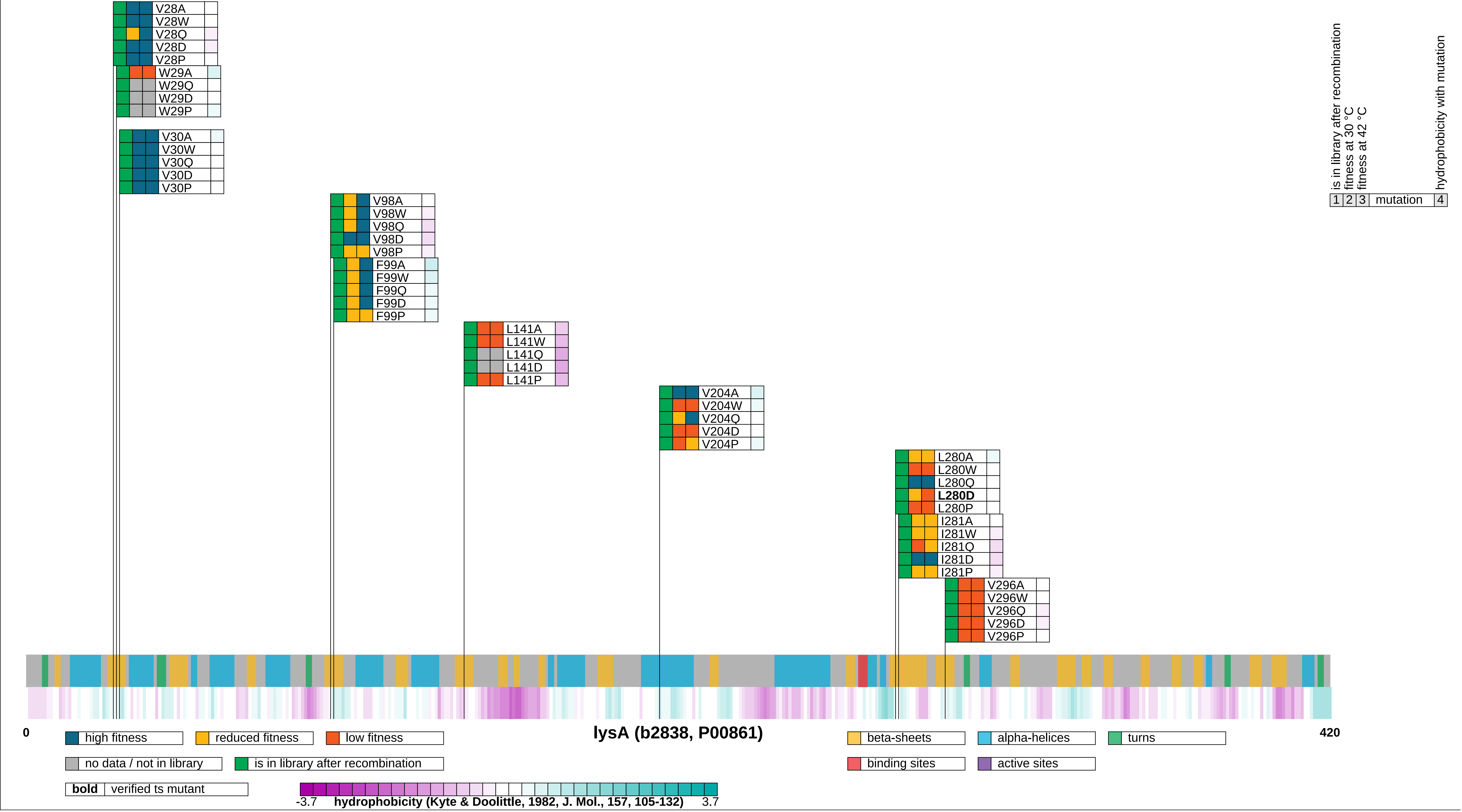

is in library after recombination
fitness at 30 °C
fitness at 42 °C
hydrophobicity with mutation
1 2 3 mutation 4
V28A
V28W
V28Q
V28D
V28P
W29A
W29Q
W29D
W29P
V30A
V30W
V30Q
V30D
V30P
V98A
V98W
V98Q
V98D
V98P
F99A
F99W
F99Q
F99D
F99P
L141A
L141W
L141Q
L141D
L141P
V204A
V204W
V204Q
V204D
V204P
L280A
L280W
L280Q
L280D
L280P
I281A
I281W
I281Q
I281D
I281P
V296A
V296W
V296Q
V296D
V296P
0
lysA (b2838, P00861)
420
high fitness
reduced fitness
low fitness
beta-sheets
alpha-helices
turns
no data / not in library
is in library after recombination
binding sites
active sites
bold verified ts mutant
-3.7
hydrophobicity (Kyte & Doolittle, 1982, J. Mol., 157, 105-132)
3.7

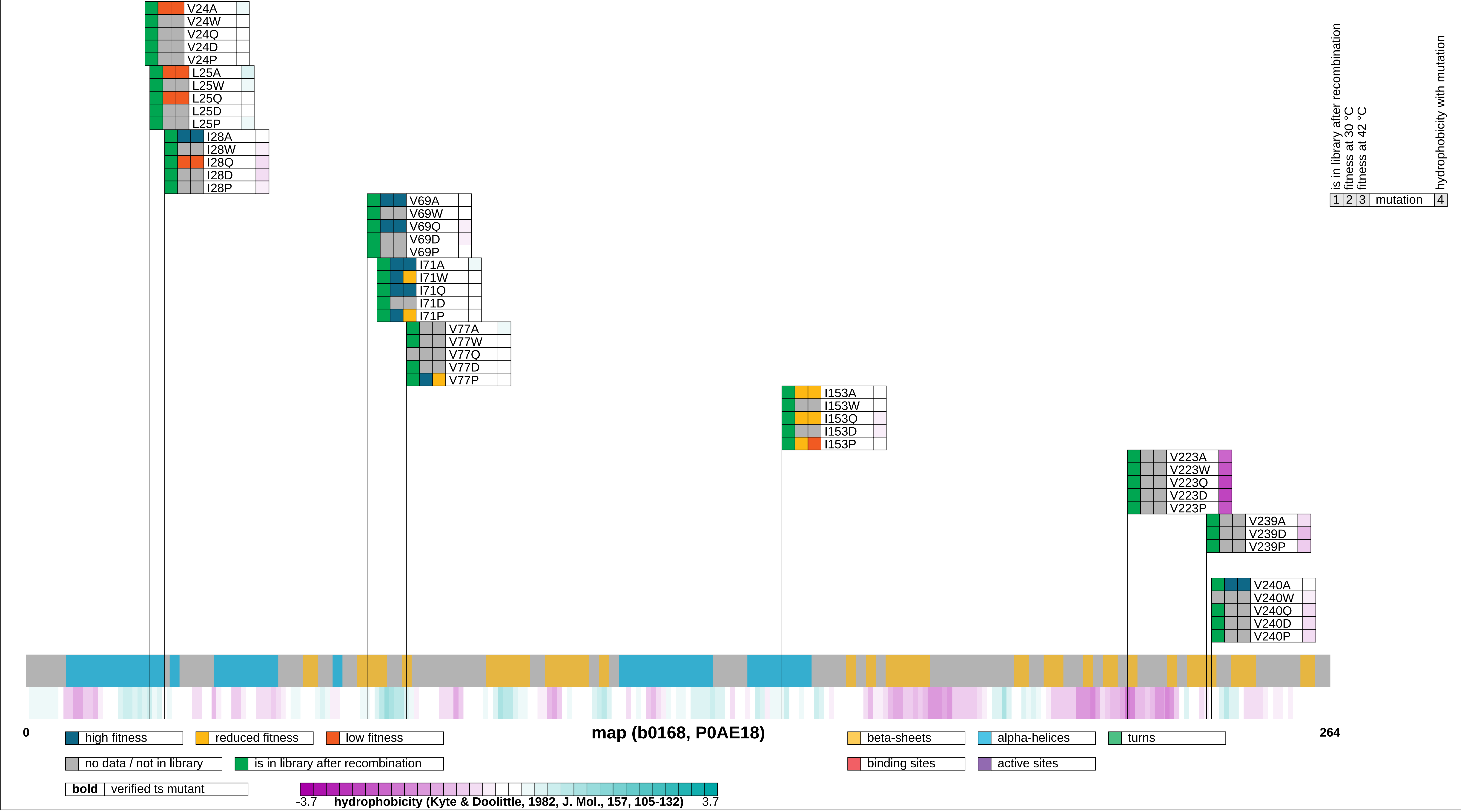
V24A
V24W
V24Q
V24D
V24P
L25A
L25W
L25Q
L25D
L25P
I28A
I28W
I28Q
I28D
I28P
V69A
V69W
V69Q
V69D
V69P
I71A
I71W
I71Q
I71D
I71P
V77A
V77W
V77Q
V77D
V77P
I153A
I153W
I153Q
I153D
I153P
V223A
V223W
V223Q
V223D
V223P
V239A
V239D
V239P
V240A
V240W
V240Q
V240D
V240P
is in library after recombination
fitness at 30 °C
fitness at 42 °C
hydrophobicity with mutation
1
2
3
mutation
4
0
map (b0168, P0AE18)
264
high fitness
reduced fitness
low fitness
no data / not in library
is in library after recombination
bold
verified ts mutant
beta-sheets
alpha-helices
turns
binding sites
active sites
-3.7
hydrophobicity (Kyte & Doolittle, 1982, J. Mol., 157, 105-132)
3.7

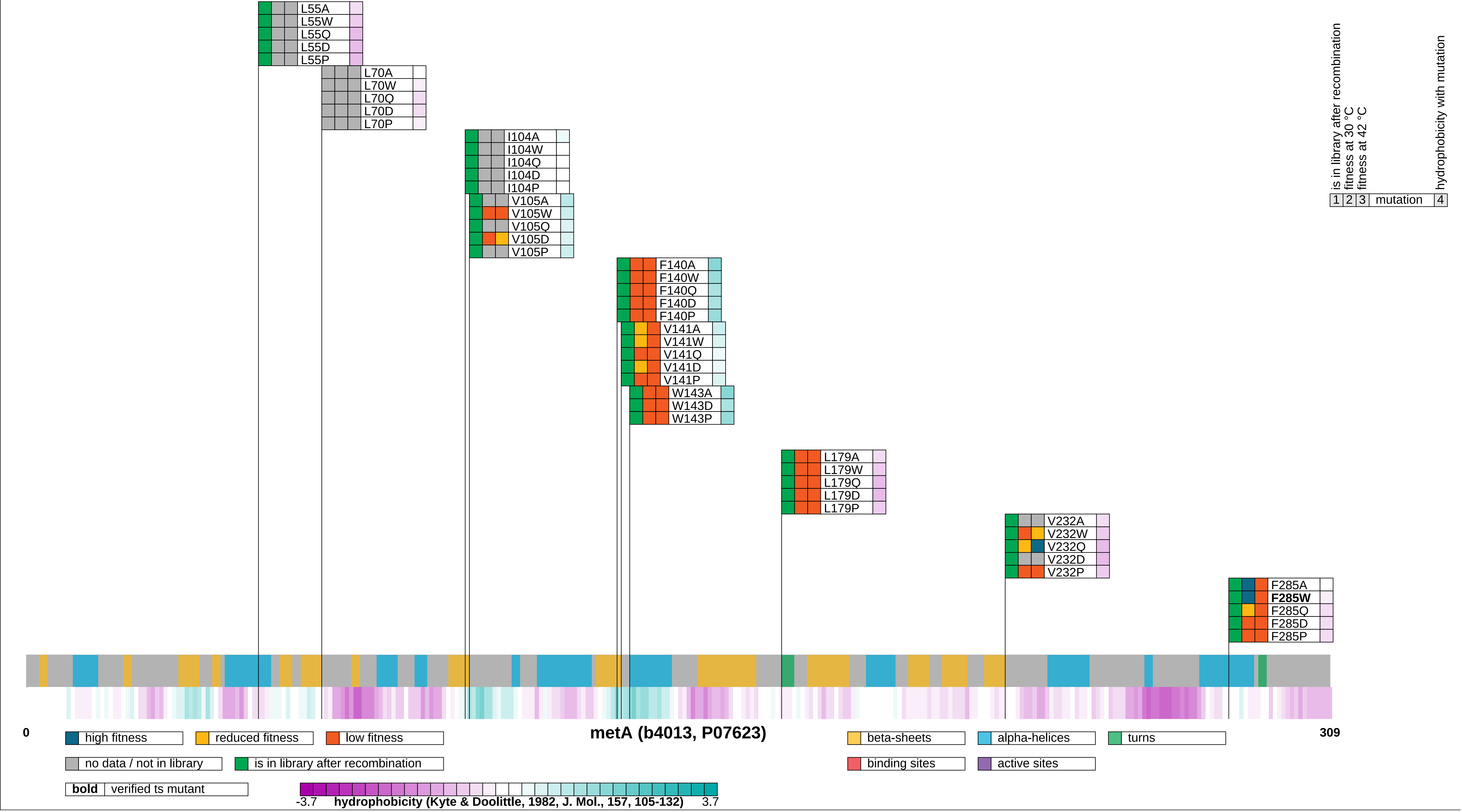

L55A
L55W
L55Q
L55D
L55P
L70A
L70W
L70Q
L70D
L70P
I104A
I104W
I104Q
I104D
I104P
V105A
V105W
V105Q
V105D
V105P
F140A
F140W
F140Q
F140D
F140P
V141A
V141W
V141Q
V141D
V141P
W143A
W143D
W143P
L179A
L179W
L179Q
L179D
L179P
V232A
V232W
V232Q
V232D
V232P
F285A
F285W
F285Q
F285D
F285P
is in library after recombination
fitness at 30 °C
fitness at 42 °C
hydrophobicity with mutation
1 2 3 mutation 4
0
metA (b4013, P07623)
309
high fitness
reduced fitness
low fitness
no data / not in library
is in library after recombination
bold verified ts mutant
beta-sheets
alpha-helices
turns
binding sites
active sites
-3.7
hydrophobicity (Kyte & Doolittle, 1982, J. Mol., 157, 105-132)
3.7

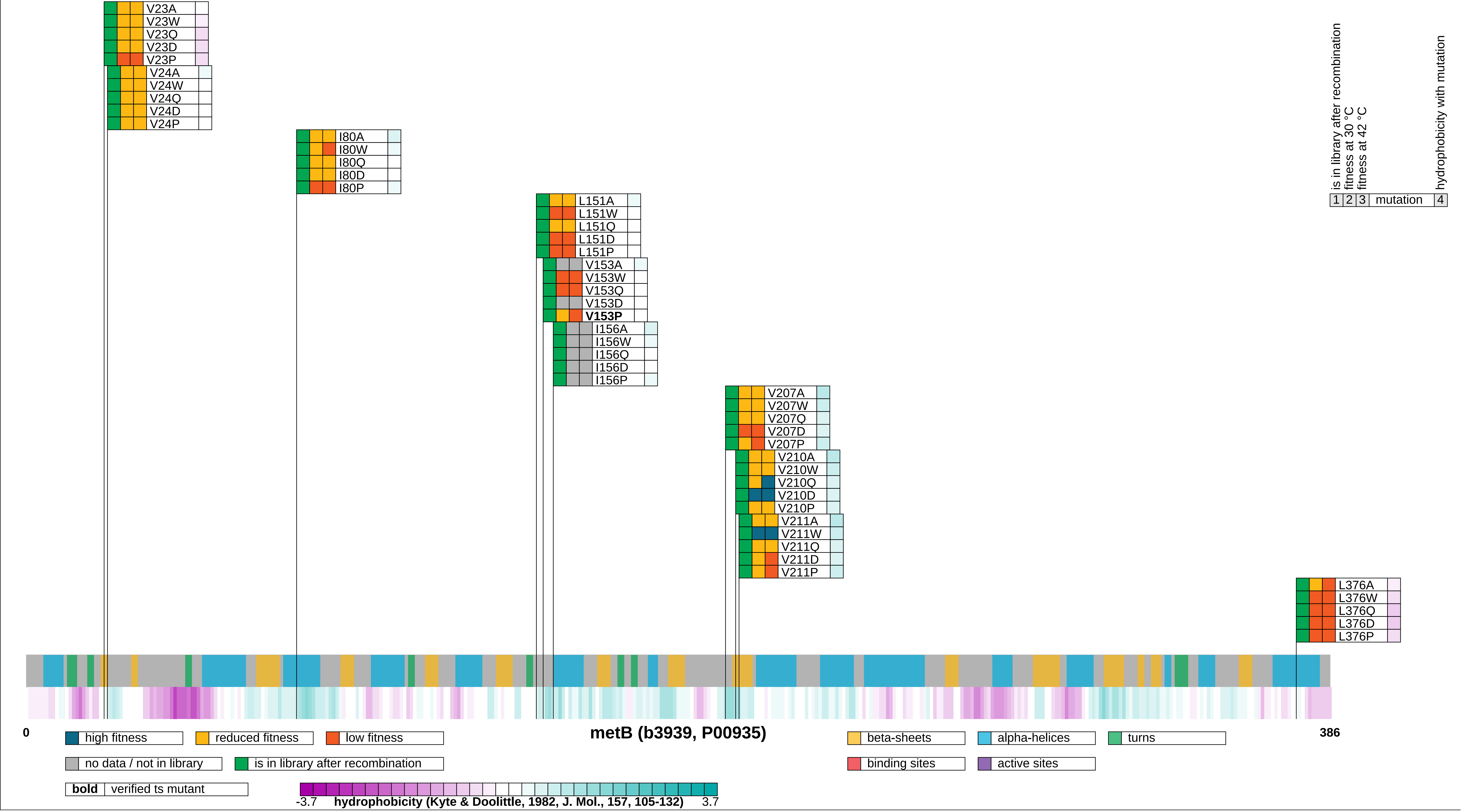

V23A
V23W
V23Q
V23D
V23P
V24A
V24W
V24Q
V24D
V24P
I80A
I80W
I80Q
I80D
I80P
L151A
L151W
L151Q
L151D
L151P
V153A
V153W
V153Q
V153D
V153P
I156A
I156W
I156Q
I156D
I156P
V207A
V207W
V207Q
V207D
V207P
V210A
V210W
V210Q
V210D
V210P
V211A
V211W
V211Q
V211D
V211P
L376A
L376W
L376Q
L376D
L376P
is in library after recombination
fitness at 30 °C
fitness at 42 °C
hydrophobicity with mutation
1 2 3 mutation 4
0
386
metB (b3939, P00935)
high fitness
reduced fitness
low fitness
beta-sheets
alpha-helices
turns
no data / not in library
is in library after recombination
binding sites
active sites
bold verified ts mutant
-3.7
hydrophobicity (Kyte & Doolittle, 1982, J. Mol., 157, 105-132)
3.7

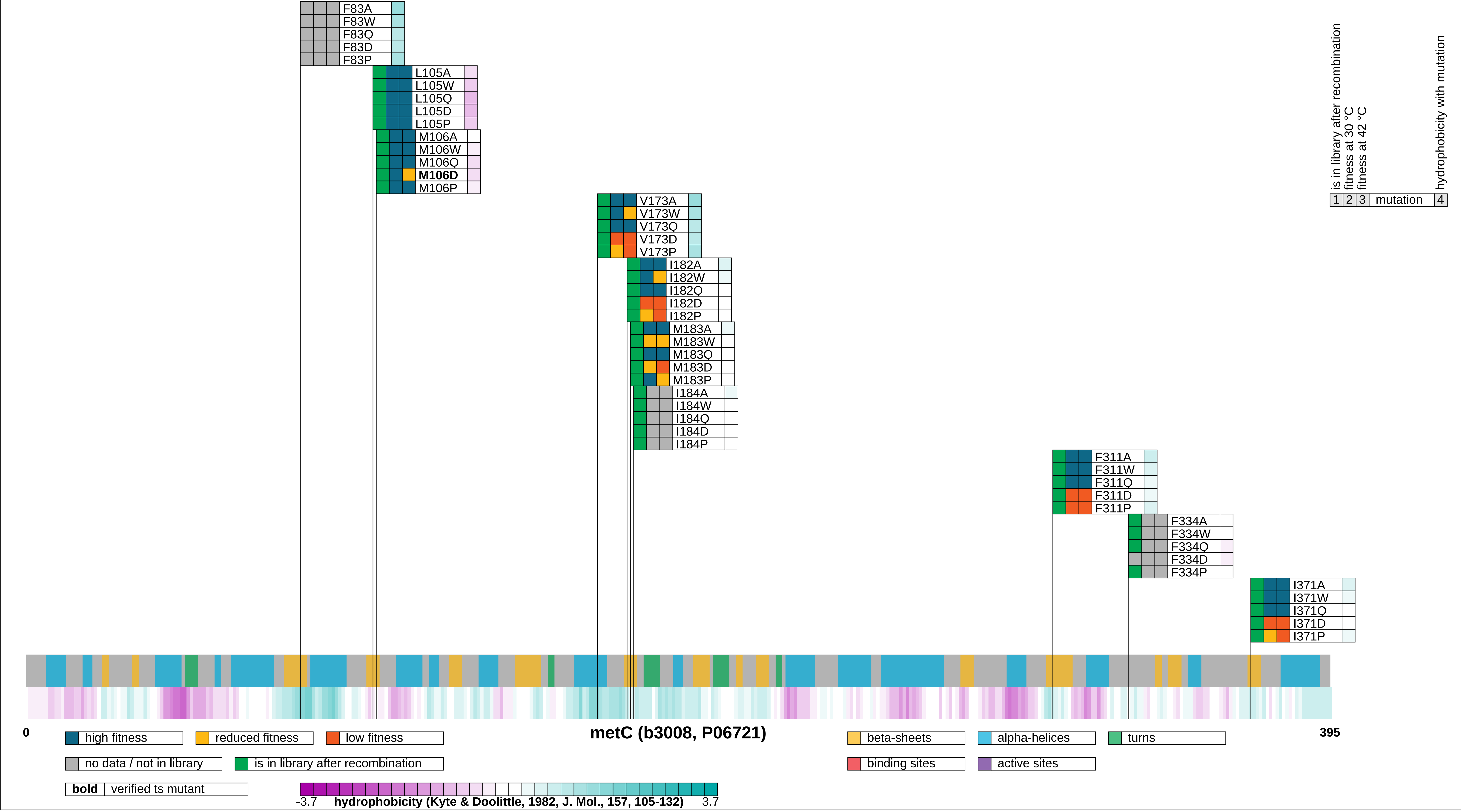

F83A
F83W
F83Q
F83D
F83P
L105A
L105W
L105Q
L105D
L105P
M106A
M106W
M106Q
M106D
M106P
V173A
V173W
V173Q
V173D
V173P
I182A
I182W
I182Q
I182D
I182P
M183A
M183W
M183Q
M183D
M183P
I184A
I184W
I184Q
I184D
I184P
F311A
F311W
F311Q
F311D
F311P
F334A
F334W
F334Q
F334D
F334P
I371A
I371W
I371Q
I371D
I371P
is in library after recombination
fitness at 30 °C
fitness at 42 °C
hydrophobicity with mutation
1 2 3 mutation 4
0
metC (b3008, P06721)
395
high fitness
reduced fitness
low fitness
beta-sheets
alpha-helices
turns
no data / not in library
is in library after recombination
binding sites
active sites
bold verified ts mutant
-3.7
hydrophobicity (Kyte & Doolittle, 1982, J. Mol., 157, 105-132)
3.7

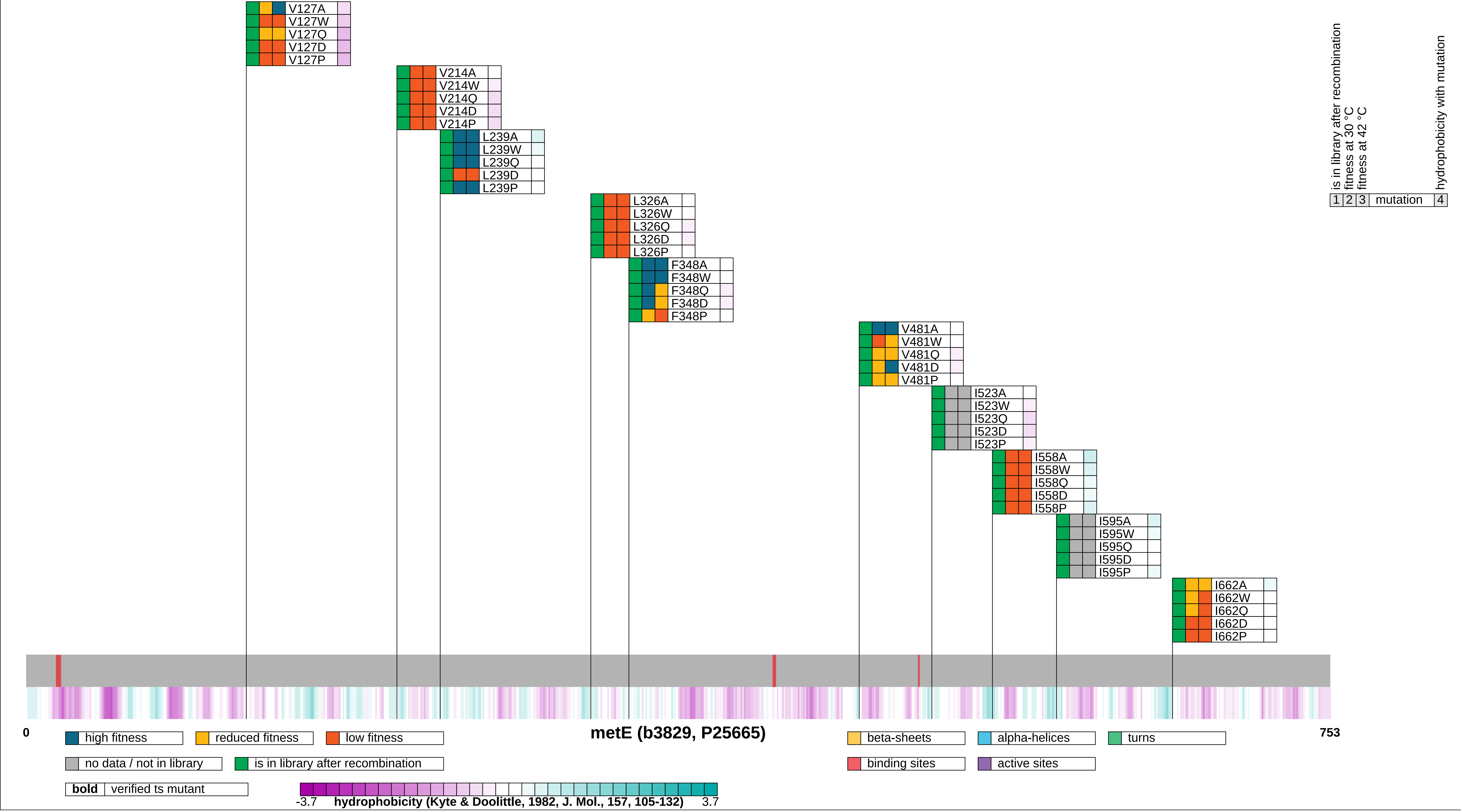

V127A
V127W
V127Q
V127D
V127P
V214A
V214W
V214Q
V214D
V214P
L239A
L239W
L239Q
L239D
L239P
L326A
L326W
L326Q
L326D
L326P
F348A
F348W
F348Q
F348D
F348P
V481A
V481W
V481Q
V481D
V481P
I523A
I523W
I523Q
I523D
I523P
I558A
I558W
I558Q
I558D
I558P
I595A
I595W
I595Q
I595D
I595P
I662A
I662W
I662Q
I662D
I662P
1 is in library after recombination
2 fitness at 30 °C
3 fitness at 42 °C
mutation
4 hydrophobicity with mutation
0
metE (b3829, P25665)
753
high fitness
reduced fitness
low fitness
no data / not in library
is in library after recombination
bold verified ts mutant
beta-sheets
alpha-helices
turns
binding sites
active sites
-3.7
hydrophobicity (Kyte & Doolittle, 1982, J. Mol., 157, 105-132)
3.7

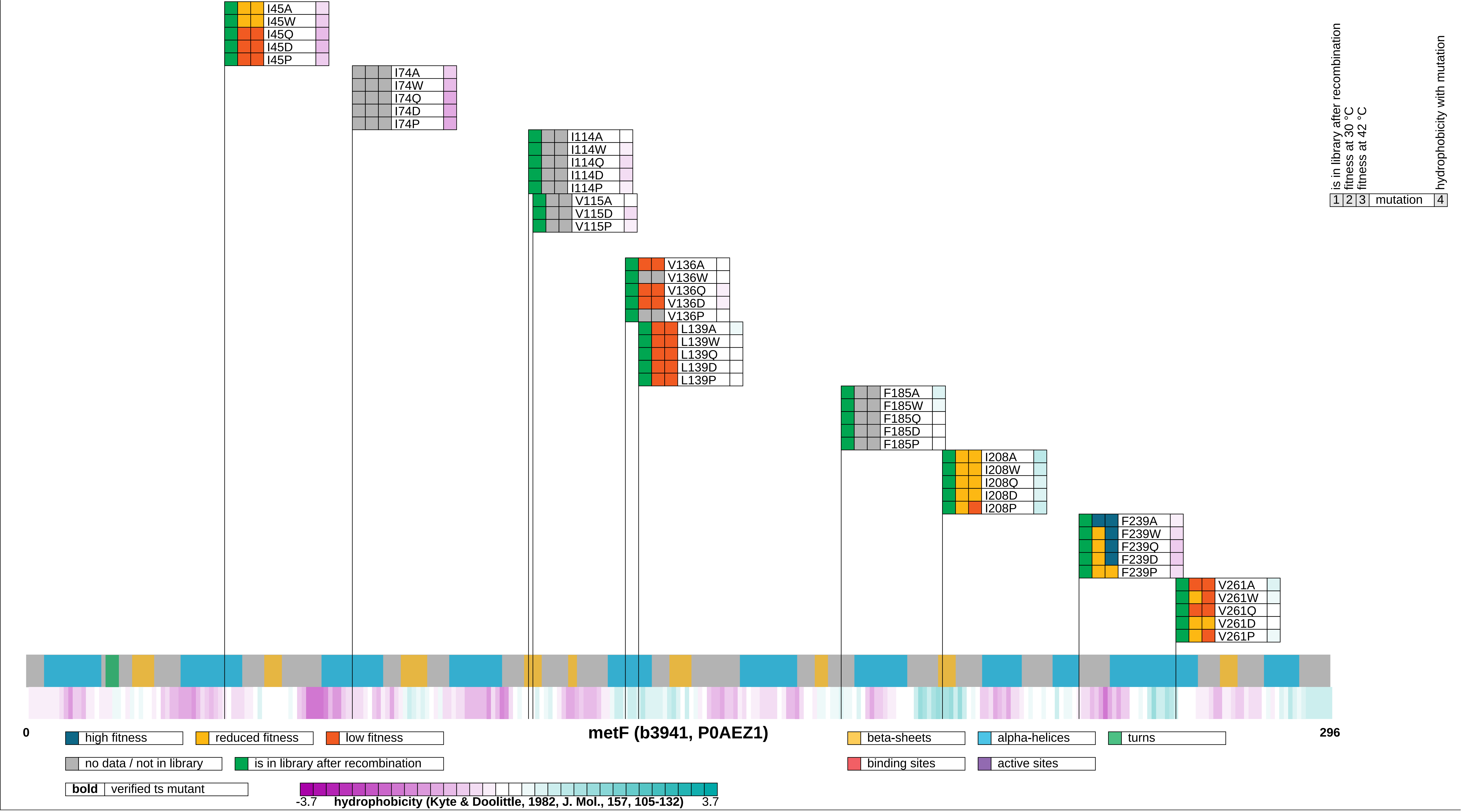
is in library after recombination
fitness at 30 °C
fitness at 42 °C
hydrophobicity with mutation
1 2 3 mutation 4
I45A
I45W
I45Q
I45D
I45P
I74A
I74W
I74Q
I74D
I74P
I114A
I114W
I114Q
I114D
I114P
V115A
V115D
V115P
V136A
V136W
V136Q
V136D
V136P
L139A
L139W
L139Q
L139D
L139P
F185A
F185W
F185Q
F185D
F185P
I208A
I208W
I208Q
I208D
I208P
F239A
F239W
F239Q
F239D
F239P
V261A
V261W
V261Q
V261D
V261P
0
metF (b3941, P0AEZ1)
296
high fitness
reduced fitness
low fitness
beta-sheets
alpha-helices
turns
no data / not in library
is in library after recombination
binding sites
active sites
bold verified ts mutant
-3.7
hydrophobicity (Kyte & Doolittle, 1982, J. Mol., 157, 105-132)
3.7

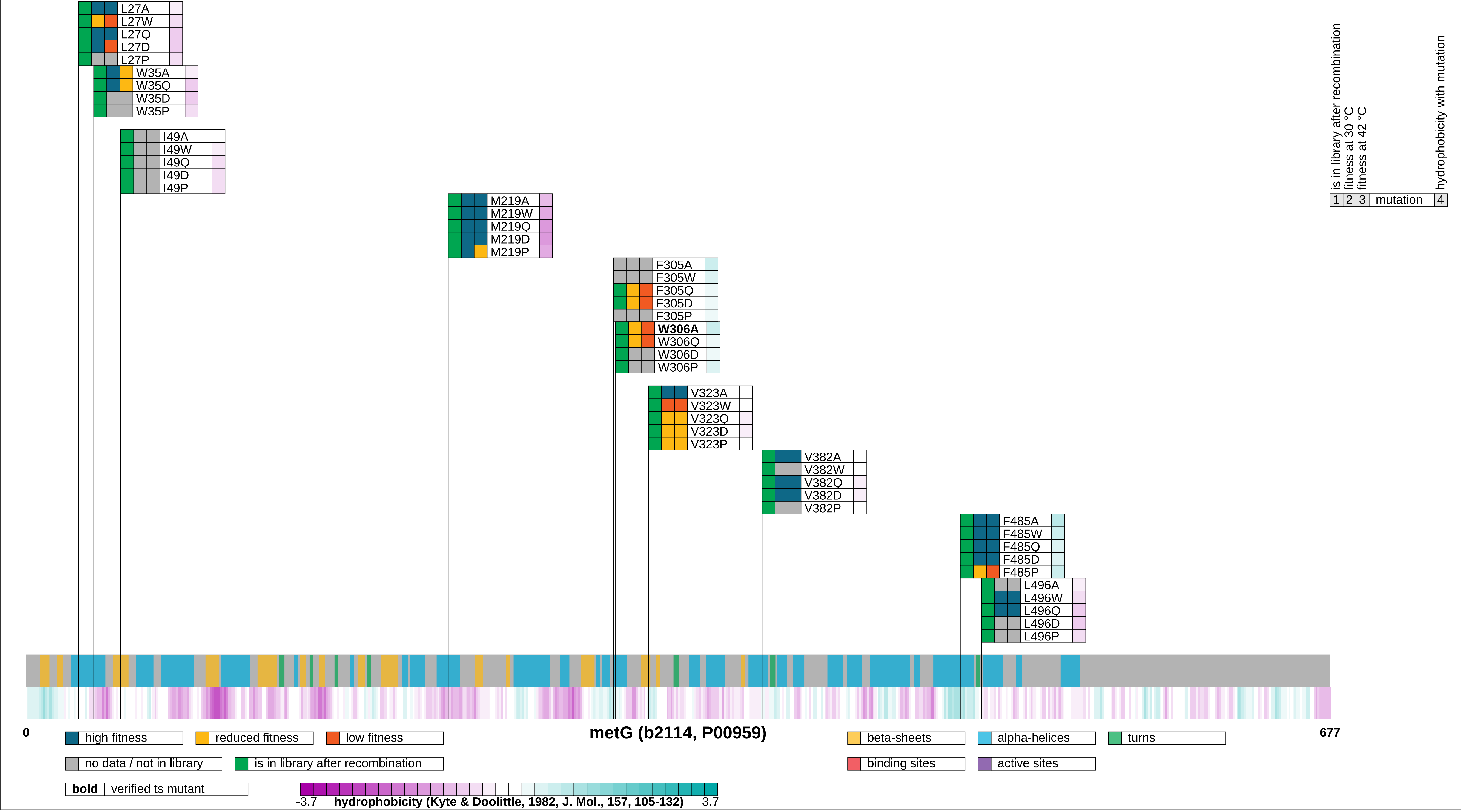

L27A
L27W
L27Q
L27D
L27P
W35A
W35Q
W35D
W35P
I49A
I49W
I49Q
I49D
I49P
M219A
M219W
M219Q
M219D
M219P
F305A
F305W
F305Q
F305D
F305P
W306A
W306Q
W306D
W306P
V323A
V323W
V323Q
V323D
V323P
V382A
V382W
V382Q
V382D
V382P
F485A
F485W
F485Q
F485D
F485P
L496A
L496W
L496Q
L496D
L496P
is in library after recombination
fitness at 30 °C
fitness at 42 °C
hydrophobicity with mutation
1 2 3 mutation 4
0
metG (b2114, P00959)
677
high fitness
reduced fitness
low fitness
beta-sheets
alpha-helices
turns
no data / not in library
is in library after recombination
binding sites
active sites
bold verified ts mutant
-3.7 hydrophobicity (Kyte & Doolittle, 1982, J. Mol., 157, 105-132) 3.7

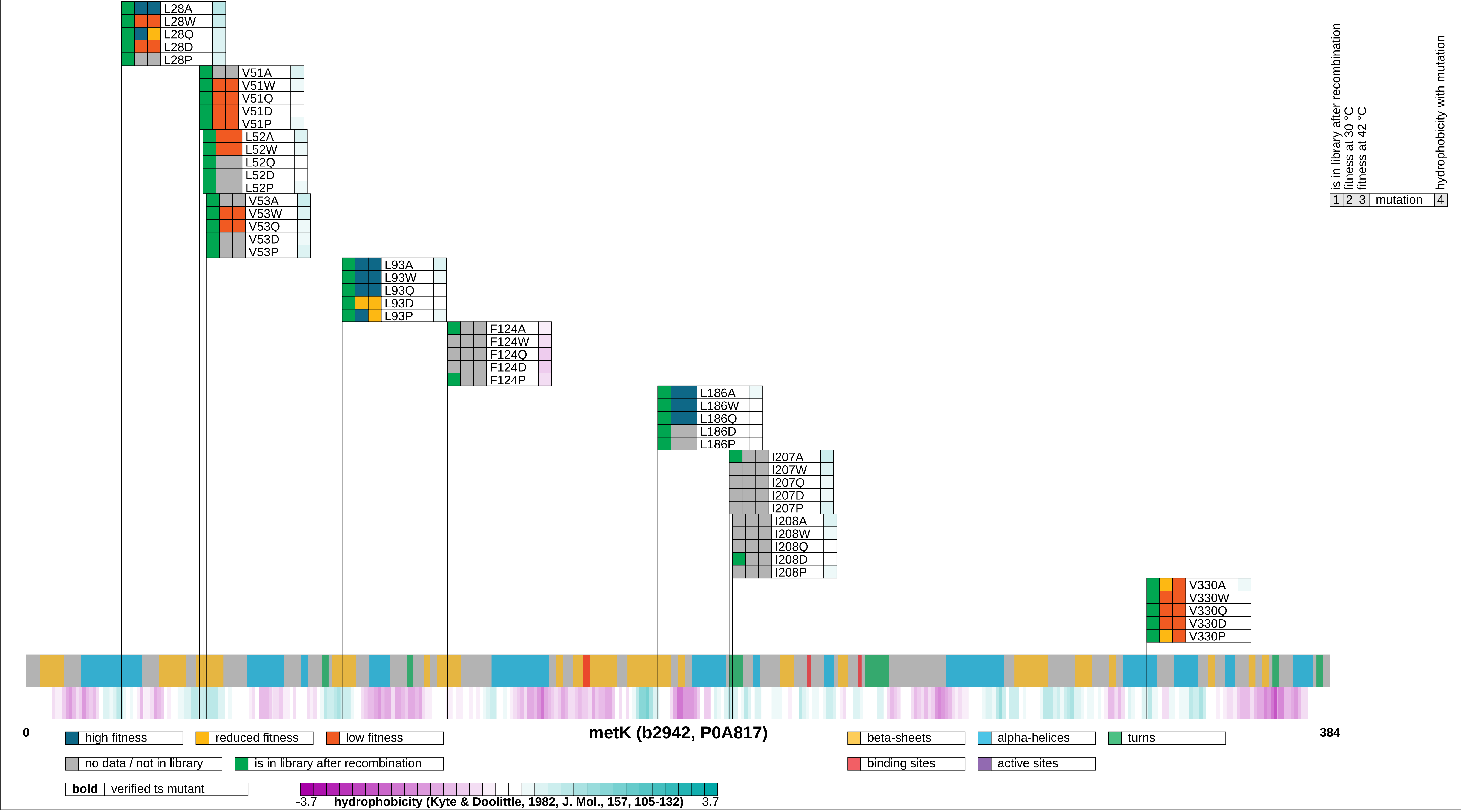

L28A
L28W
L28Q
L28D
L28P
V51A
V51W
V51Q
V51D
V51P
L52A
L52W
L52Q
L52D
L52P
V53A
V53W
V53Q
V53D
V53P
L93A
L93W
L93Q
L93D
L93P
F124A
F124W
F124Q
F124D
F124P
L186A
L186W
L186Q
L186D
L186P
I207A
I207W
I207Q
I207D
I207P
I208A
I208W
I208Q
I208D
I208P
V330A
V330W
V330Q
V330D
V330P
is in library after recombination
fitness at 30 °C
fitness at 42 °C
hydrophobicity with mutation
1 2 3 mutation 4
0
metK (b2942, P0A817)
384
high fitness
reduced fitness
low fitness
beta-sheets
alpha-helices
turns
no data / not in library
is in library after recombination
binding sites
active sites
bold verified ts mutant
-3.7 hydrophobicity (Kyte & Doolittle, 1982, J. Mol., 157, 105-132) 3.7

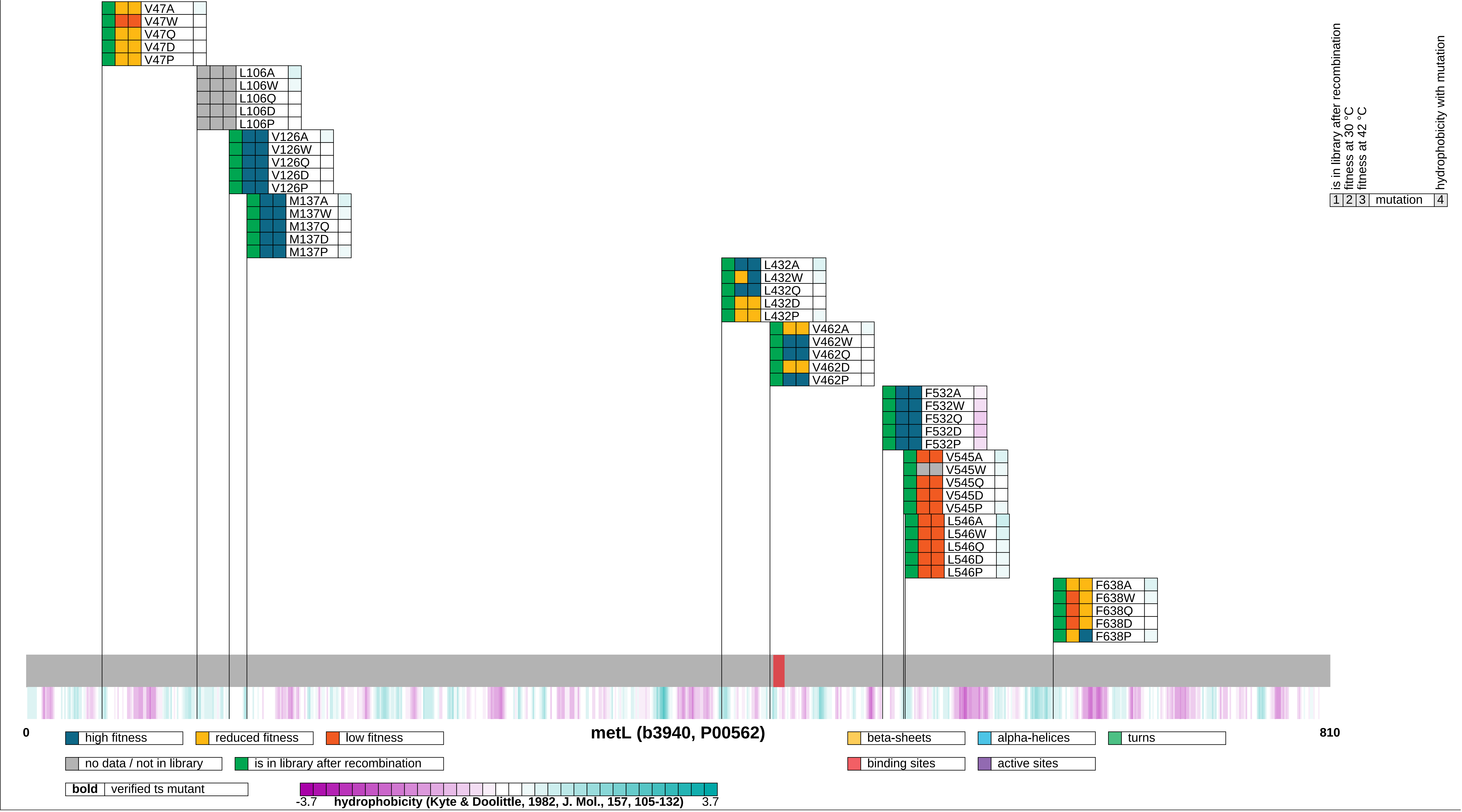

V47A
V47W
V47Q
V47D
V47P
L106A
L106W
L106Q
L106D
L106P
V126A
V126W
V126Q
V126D
V126P
M137A
M137W
M137Q
M137D
M137P
L432A
L432W
L432Q
L432D
L432P
V462A
V462W
V462Q
V462D
V462P
F532A
F532W
F532Q
F532D
F532P
V545A
V545W
V545Q
V545D
V545P
L546A
L546W
L546Q
L546D
L546P
F638A
F638W
F638Q
F638D
F638P
is in library after recombination
fitness at 30 °C
fitness at 42 °C
hydrophobicity with mutation
1 2 3 mutation 4
0
metL (b3940, P00562)
810
high fitness
reduced fitness
low fitness
beta-sheets
alpha-helices
turns
no data / not in library
is in library after recombination
binding sites
active sites
bold verified ts mutant
-3.7 hydrophobicity (Kyte & Doolittle, 1982, J. Mol., 157, 105-132) 3.7

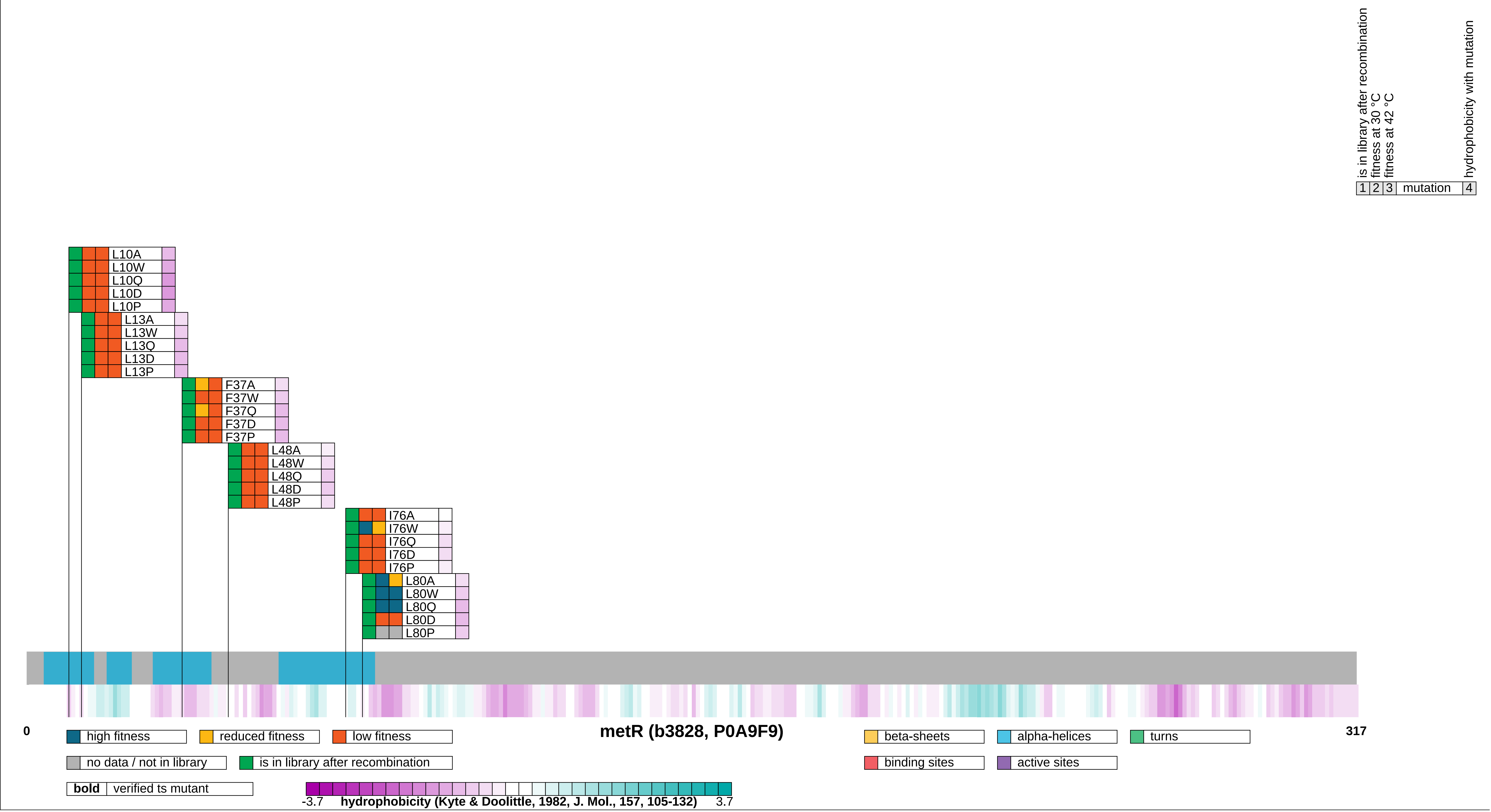

is in library after recombination
fitness at 30 °C
fitness at 42 °C
hydrophobicity with mutation
1 2 3 mutation 4
L10A
L10W
L10Q
L10D
L10P
L13A
L13W
L13Q
L13D
L13P
F37A
F37W
F37Q
F37D
F37P
L48A
L48W
L48Q
L48D
L48P
I76A
I76W
I76Q
I76D
I76P
L80A
L80W
L80Q
L80D
L80P
0
metR (b3828, P0A9F9)
317
high fitness
reduced fitness
low fitness
beta-sheets
alpha-helices
turns
no data / not in library
is in library after recombination
binding sites
active sites
bold verified ts mutant
-3.7
hydrophobicity (Kyte & Doolittle, 1982, J. Mol., 157, 105-132)
3.7

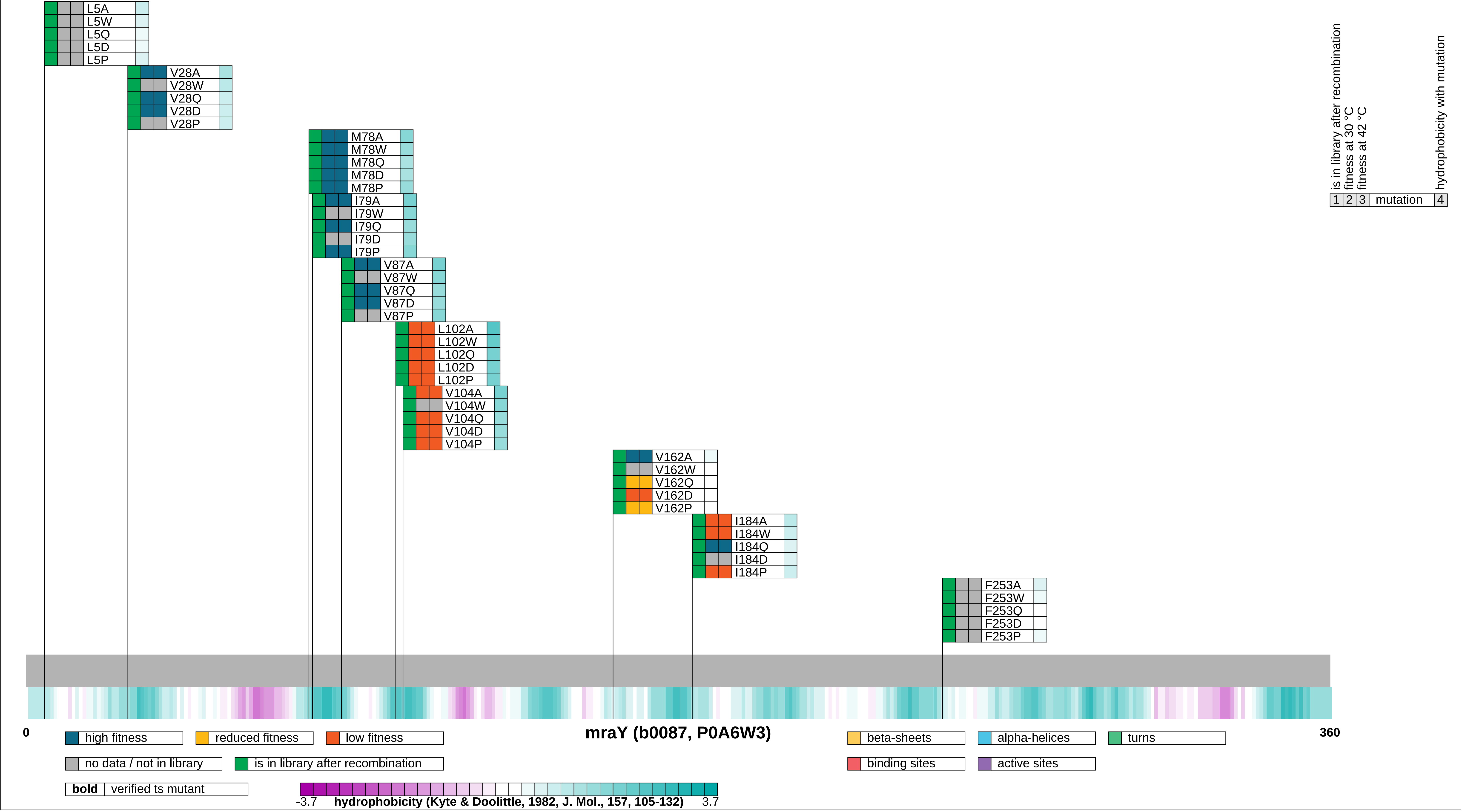
L5A
L5W
L5Q
L5D
L5P
V28A
V28W
V28Q
V28D
V28P
M78A
M78W
M78Q
M78D
M78P
I79A
I79W
I79Q
I79D
I79P
V87A
V87W
V87Q
V87D
V87P
L102A
L102W
L102Q
L102D
L102P
V104A
V104W
V104Q
V104D
V104P
V162A
V162W
V162Q
V162D
V162P
I184A
I184W
I184Q
I184D
I184P
F253A
F253W
F253Q
F253D
F253P
is in library after recombination
fitness at 30 °C
fitness at 42 °C
hydrophobicity with mutation
1
2
3
mutation
4
0
360
mraY (b0087, P0A6W3)
high fitness
reduced fitness
low fitness
no data / not in library
is in library after recombination
bold
verified ts mutant
beta-sheets
alpha-helices
turns
binding sites
active sites
-3.7
hydrophobicity (Kyte & Doolittle, 1982, J. Mol., 157, 105-132)
3.7

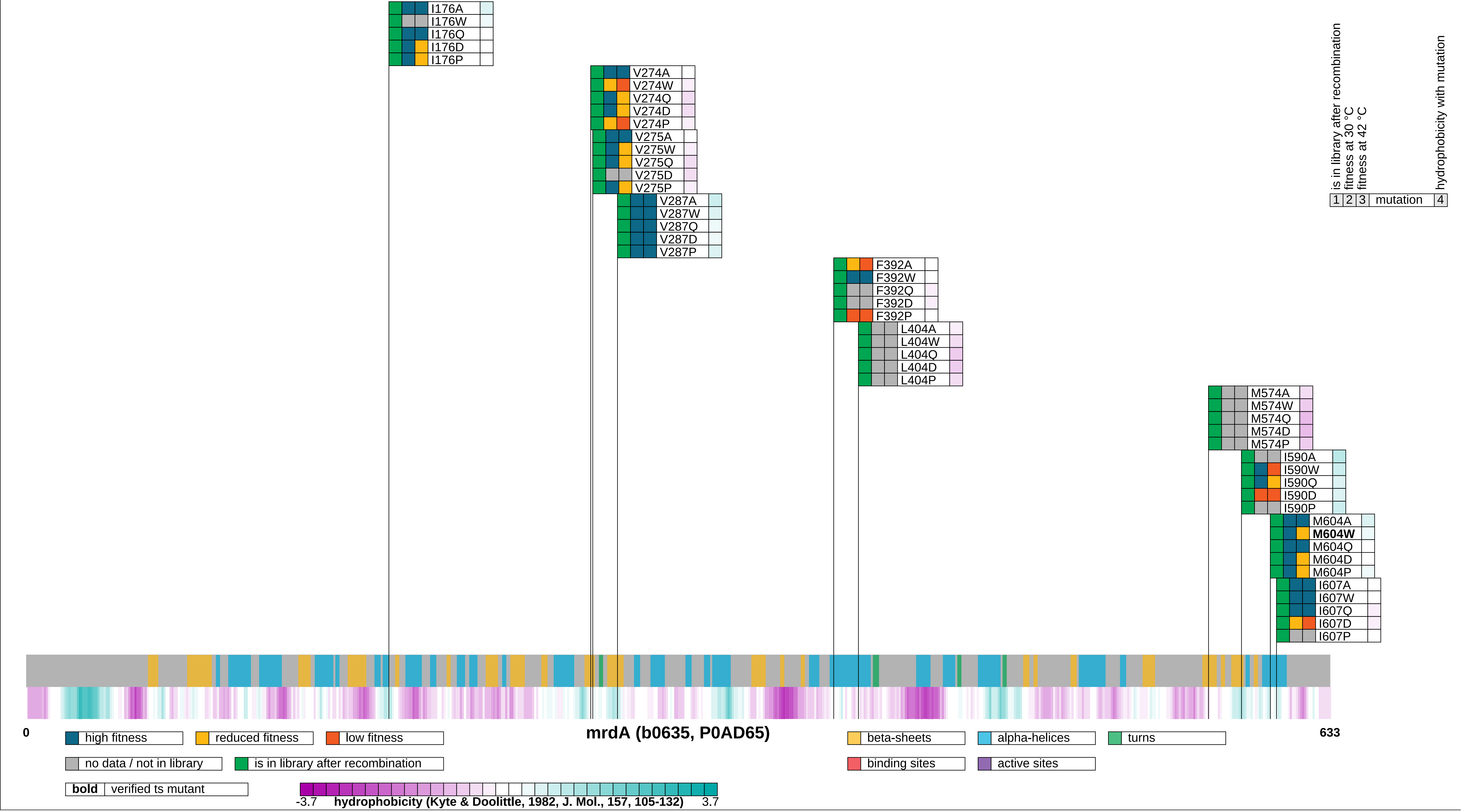
I176A
I176W
I176Q
I176D
I176P
V274A
V274W
V274Q
V274D
V274P
V275A
V275W
V275Q
V275D
V275P
V287A
V287W
V287Q
V287D
V287P
F392A
F392W
F392Q
F392D
F392P
L404A
L404W
L404Q
L404D
L404P
M574A
M574W
M574Q
M574D
M574P
I590A
I590W
I590Q
I590D
I590P
M604A
M604W
M604Q
M604D
M604P
I607A
I607W
I607Q
I607D
I607P
is in library after recombination
fitness at 30 °C
fitness at 42 °C
hydrophobicity with mutation
1 2 3 mutation 4
0
mrdA (b0635, P0AD65)
633
high fitness
reduced fitness
low fitness
no data / not in library
is in library after recombination
beta-sheets
alpha-helices
turns
binding sites
active sites
bold verified ts mutant
-3.7 hydrophobicity (Kyte & Doolittle, 1982, J. Mol., 157, 105-132) 3.7

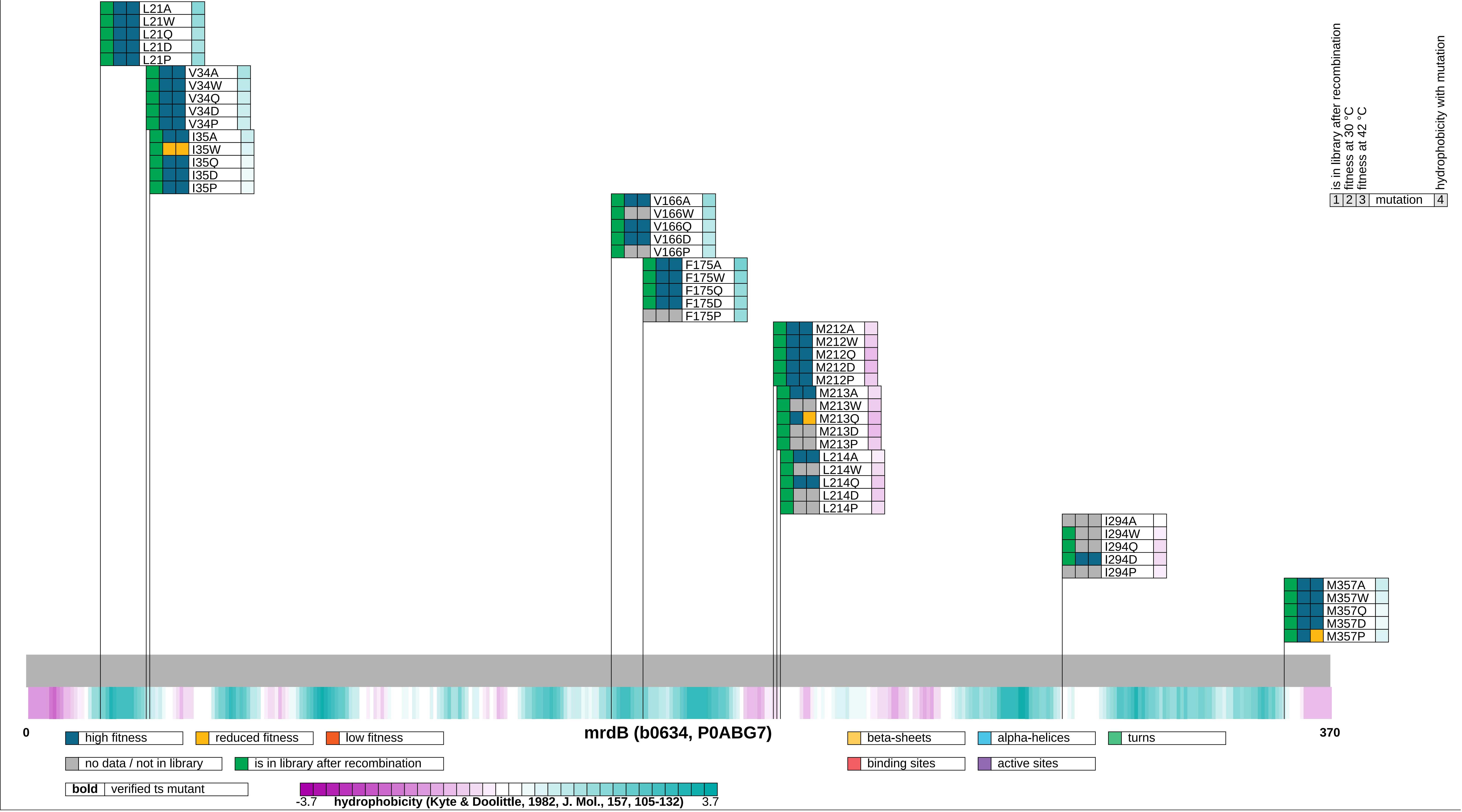

L21A
L21W
L21Q
L21D
L21P
V34A
V34W
V34Q
V34D
V34P
I35A
I35W
I35Q
I35D
I35P
V166A
V166W
V166Q
V166D
V166P
F175A
F175W
F175Q
F175D
F175P
M212A
M212W
M212Q
M212D
M212P
M213A
M213W
M213Q
M213D
M213P
L214A
L214W
L214Q
L214D
L214P
I294A
I294W
I294Q
I294D
I294P
M357A
M357W
M357Q
M357D
M357P
is in library after recombination
fitness at 30 °C
fitness at 42 °C
hydrophobicity with mutation
1
2
3
mutation
4
0
mrdB (b0634, P0ABG7)
370
high fitness
reduced fitness
low fitness
beta-sheets
alpha-helices
turns
no data / not in library
is in library after recombination
binding sites
active sites
bold
verified ts mutant
-3.7
hydrophobicity (Kyte & Doolittle, 1982, J. Mol., 157, 105-132)
3.7

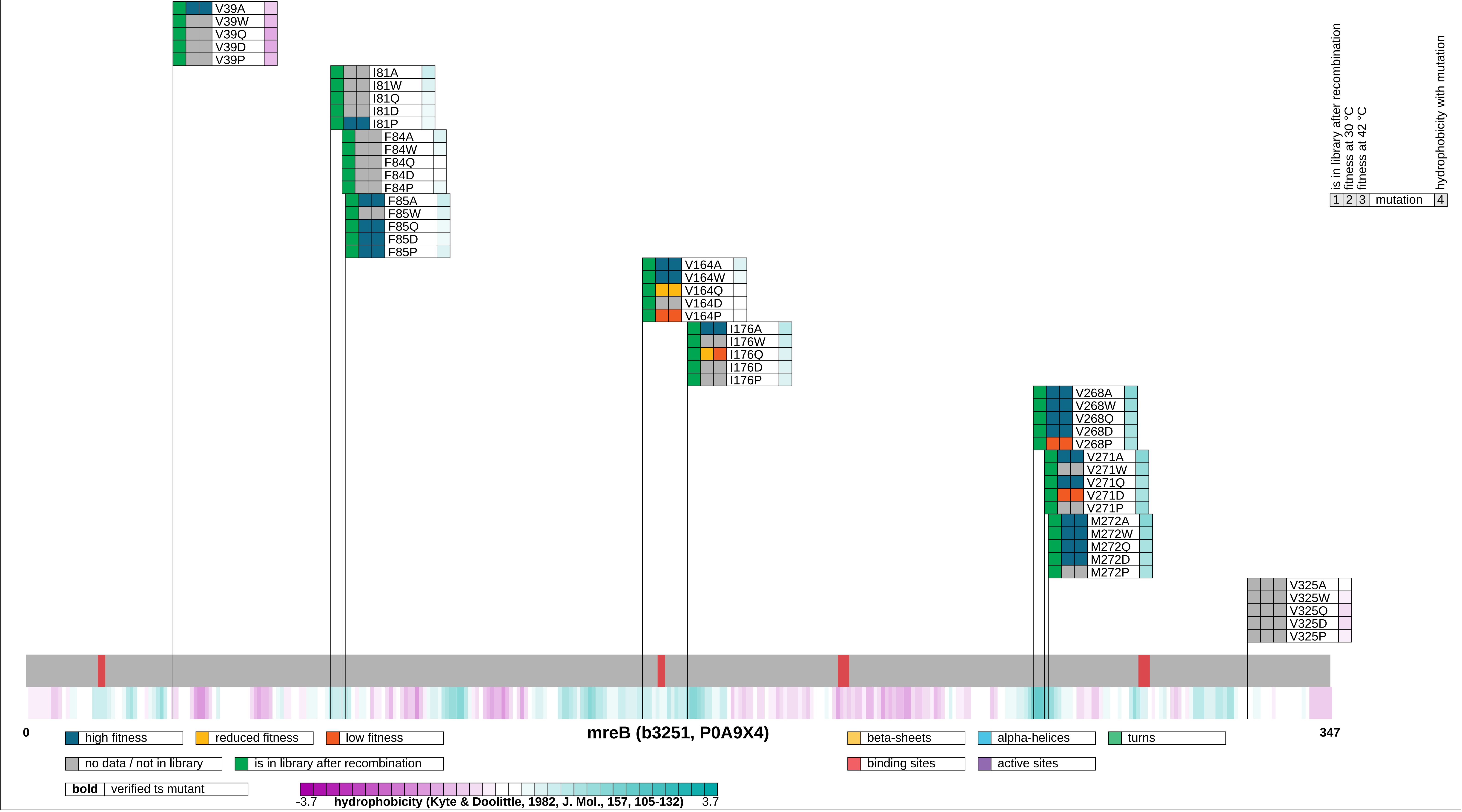

V39A
V39W
V39Q
V39D
V39P
I81A
I81W
I81Q
I81D
I81P
F84A
F84W
F84Q
F84D
F84P
F85A
F85W
F85Q
F85D
F85P
V164A
V164W
V164Q
V164D
V164P
I176A
I176W
I176Q
I176D
I176P
V268A
V268W
V268Q
V268D
V268P
V271A
V271W
V271Q
V271D
V271P
M272A
M272W
M272Q
M272D
M272P
V325A
V325W
V325Q
V325D
V325P
is in library after recombination
fitness at 30 °C
fitness at 42 °C
hydrophobicity with mutation
1
2
3
mutation
4
0
mreB (b3251, P0A9X4)
347
high fitness
reduced fitness
low fitness
beta-sheets
alpha-helices
turns
no data / not in library
is in library after recombination
binding sites
active sites
bold
verified ts mutant
-3.7
hydrophobicity (Kyte & Doolittle, 1982, J. Mol., 157, 105-132)
3.7

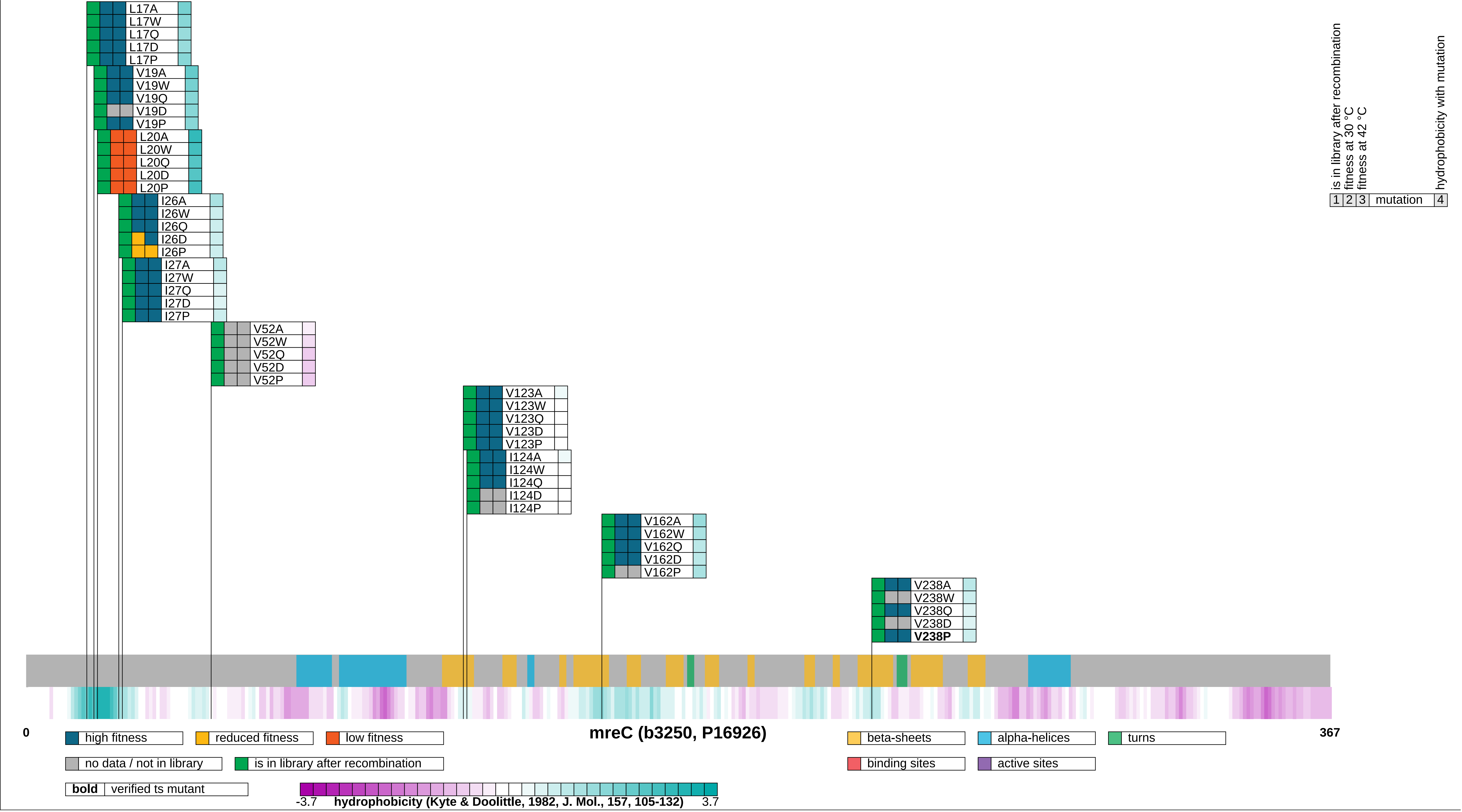

L17A
L17W
L17Q
L17D
L17P
V19A
V19W
V19Q
V19D
V19P
L20A
L20W
L20Q
L20D
L20P
I26A
I26W
I26Q
I26D
I26P
I27A
I27W
I27Q
I27D
I27P
V52A
V52W
V52Q
V52D
V52P
V123A
V123W
V123Q
V123D
V123P
I124A
I124W
I124Q
I124D
I124P
V162A
V162W
V162Q
V162D
V162P
V238A
V238W
V238Q
V238D
V238P
is in library after recombination
fitness at 30 °C
fitness at 42 °C
hydrophobicity with mutation
1 2 3 mutation 4
0
mreC (b3250, P16926)
367
high fitness
reduced fitness
low fitness
beta-sheets
alpha-helices
turns
no data / not in library
is in library after recombination
binding sites
active sites
bold verified ts mutant
-3.7 hydrophobicity (Kyte & Doolittle, 1982, J. Mol., 157, 105-132) 3.7

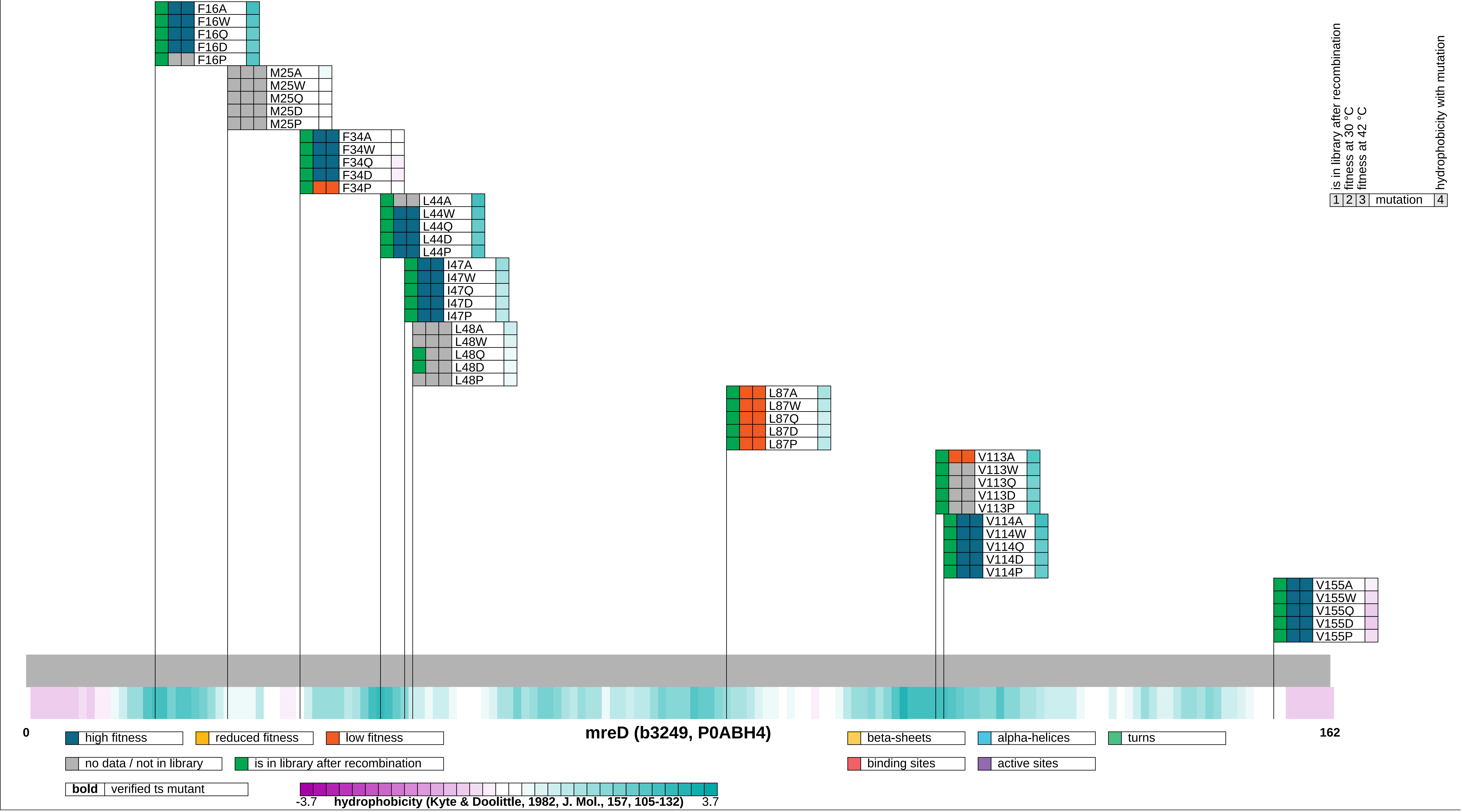

F16A
F16W
F16Q
F16D
F16P
M25A
M25W
M25Q
M25D
M25P
F34A
F34W
F34Q
F34D
F34P
L44A
L44W
L44Q
L44D
L44P
I47A
I47W
I47Q
I47D
I47P
L48A
L48W
L48Q
L48D
L48P
L87A
L87W
L87Q
L87D
L87P
V113A
V113W
V113Q
V113D
V113P
V114A
V114W
V114Q
V114D
V114P
V155A
V155W
V155Q
V155D
V155P
is in library after recombination
fitness at 30 °C
fitness at 42 °C
hydrophobicity with mutation
1 2 3 mutation 4
0
mreD (b3249, P0ABH4)
162
high fitness
reduced fitness
low fitness
beta-sheets
alpha-helices
turns
no data / not in library
is in library after recombination
binding sites
active sites
bold verified ts mutant
-3.7 hydrophobicity (Kyte & Doolittle, 1982, J. Mol., 157, 105-132) 3.7

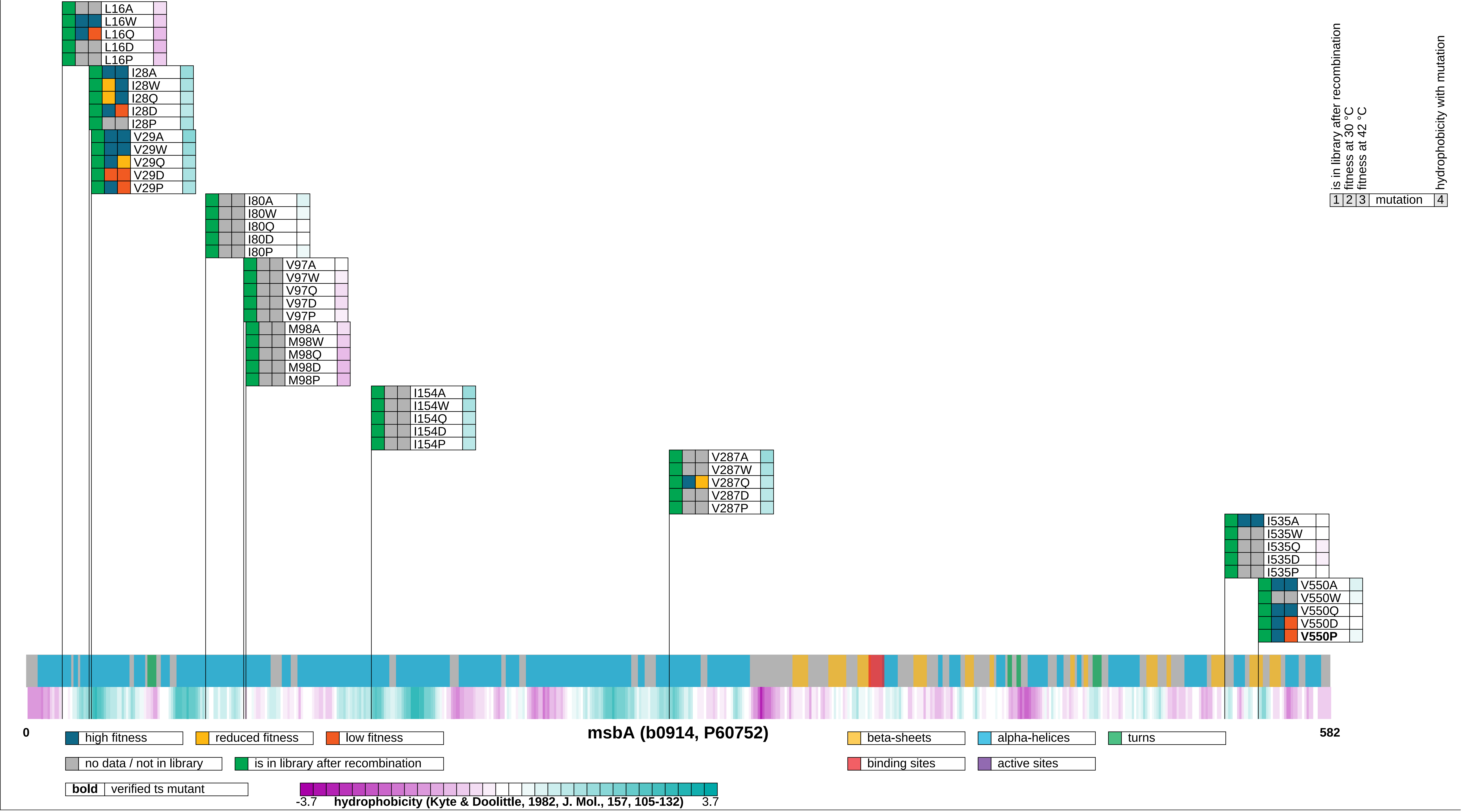

L16A
L16W
L16Q
L16D
L16P
I28A
I28W
I28Q
I28D
I28P
V29A
V29W
V29Q
V29D
V29P
I80A
I80W
I80Q
I80D
I80P
V97A
V97W
V97Q
V97D
V97P
M98A
M98W
M98Q
M98D
M98P
I154A
I154W
I154Q
I154D
I154P
V287A
V287W
V287Q
V287D
V287P
I535A
I535W
I535Q
I535D
I535P
V550A
V550W
V550Q
V550D
V550P
is in library after recombination
fitness at 30 °C
fitness at 42 °C
hydrophobicity with mutation
1 2 3 mutation 4
0
msbA (b0914, P60752)
582
high fitness
reduced fitness
low fitness
beta-sheets
alpha-helices
turns
no data / not in library
is in library after recombination
binding sites
active sites
bold verified ts mutant
-3.7
hydrophobicity (Kyte & Doolittle, 1982, J. Mol., 157, 105-132)
3.7

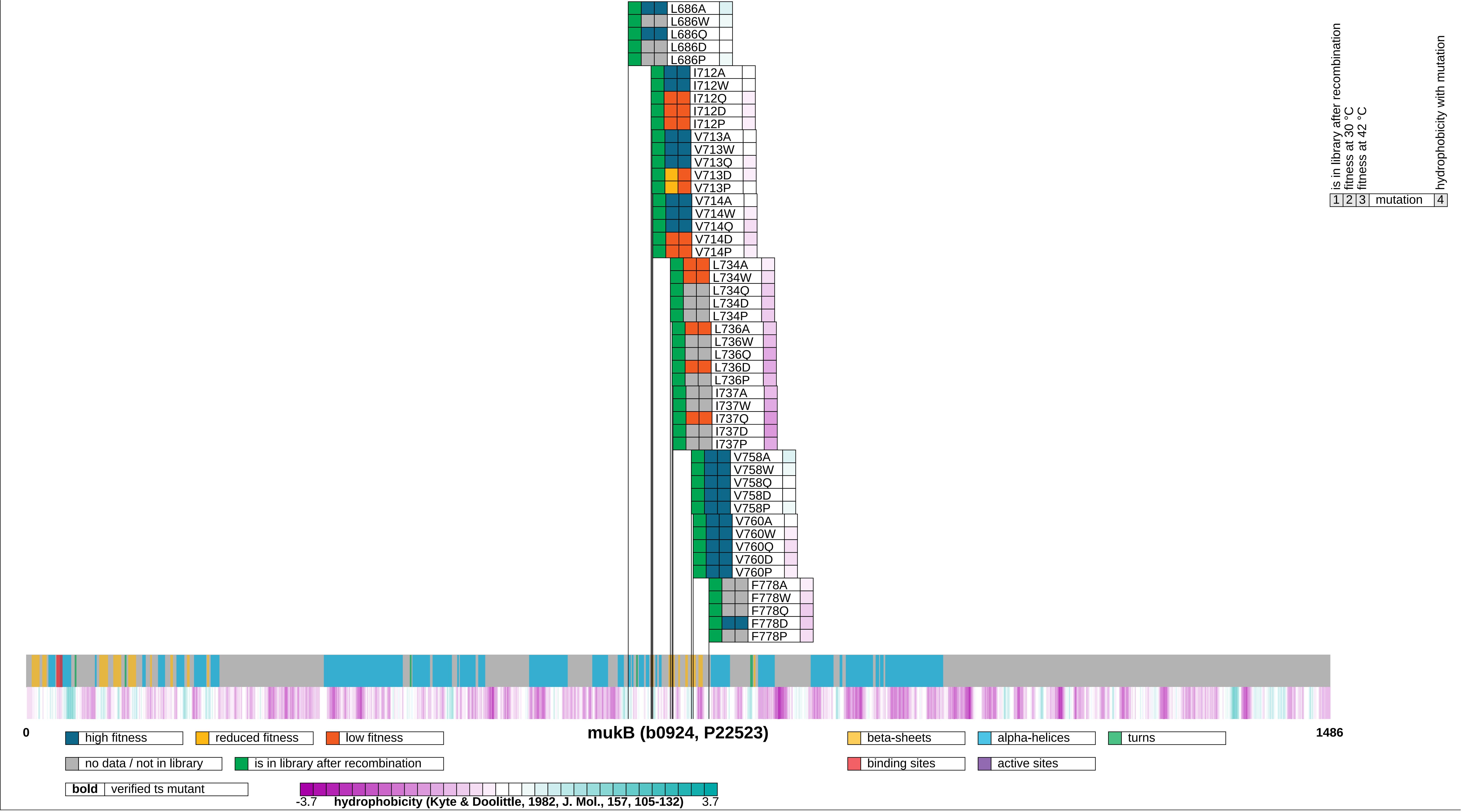
L686A
L686W
L686Q
L686D
L686P
I712A
I712W
I712Q
I712D
I712P
V713A
V713W
V713Q
V713D
V713P
V714A
V714W
V714Q
V714D
V714P
L734A
L734W
L734Q
L734D
L734P
L736A
L736W
L736Q
L736D
L736P
I737A
I737W
I737Q
I737D
I737P
V758A
V758W
V758Q
V758D
V758P
V760A
V760W
V760Q
V760D
V760P
F778A
F778W
F778Q
F778D
F778P
is in library after recombination
fitness at 30 °C
fitness at 42 °C
hydrophobicity with mutation
1 2 3 mutation 4
0
mukB (b0924, P22523)
1486
high fitness
reduced fitness
low fitness
no data / not in library
is in library after recombination
bold verified ts mutant
beta-sheets
alpha-helices
turns
binding sites
active sites
-3.7
hydrophobicity (Kyte & Doolittle, 1982, J. Mol., 157, 105-132)
3.7

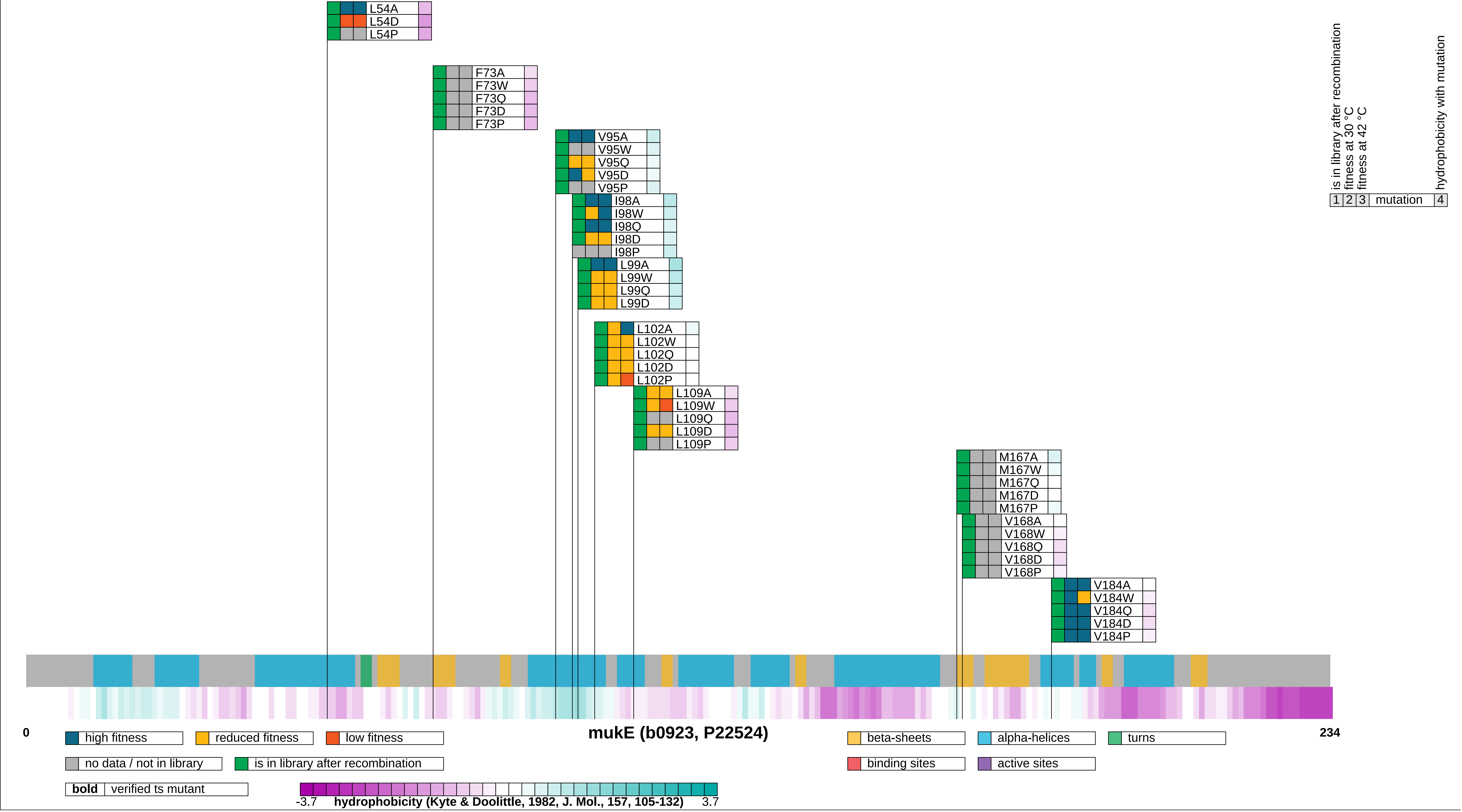

L54A
L54D
L54P
F73A
F73W
F73Q
F73D
F73P
V95A
V95W
V95Q
V95D
V95P
I98A
I98W
I98Q
I98D
I98P
L99A
L99W
L99Q
L99D
L102A
L102W
L102Q
L102D
L102P
L109A
L109W
L109Q
L109D
L109P
M167A
M167W
M167Q
M167D
M167P
V168A
V168W
V168Q
V168D
V168P
V184A
V184W
V184Q
V184D
V184P
is in library after recombination
fitness at 30 °C
fitness at 42 °C
hydrophobicity with mutation
1 2 3 mutation 4
0
mukE (b0923, P22524)
234
high fitness
reduced fitness
low fitness
beta-sheets
alpha-helices
turns
no data / not in library
is in library after recombination
binding sites
active sites
bold verified ts mutant
-3.7 hydrophobicity (Kyte & Doolittle, 1982, J. Mol., 157, 105-132) 3.7

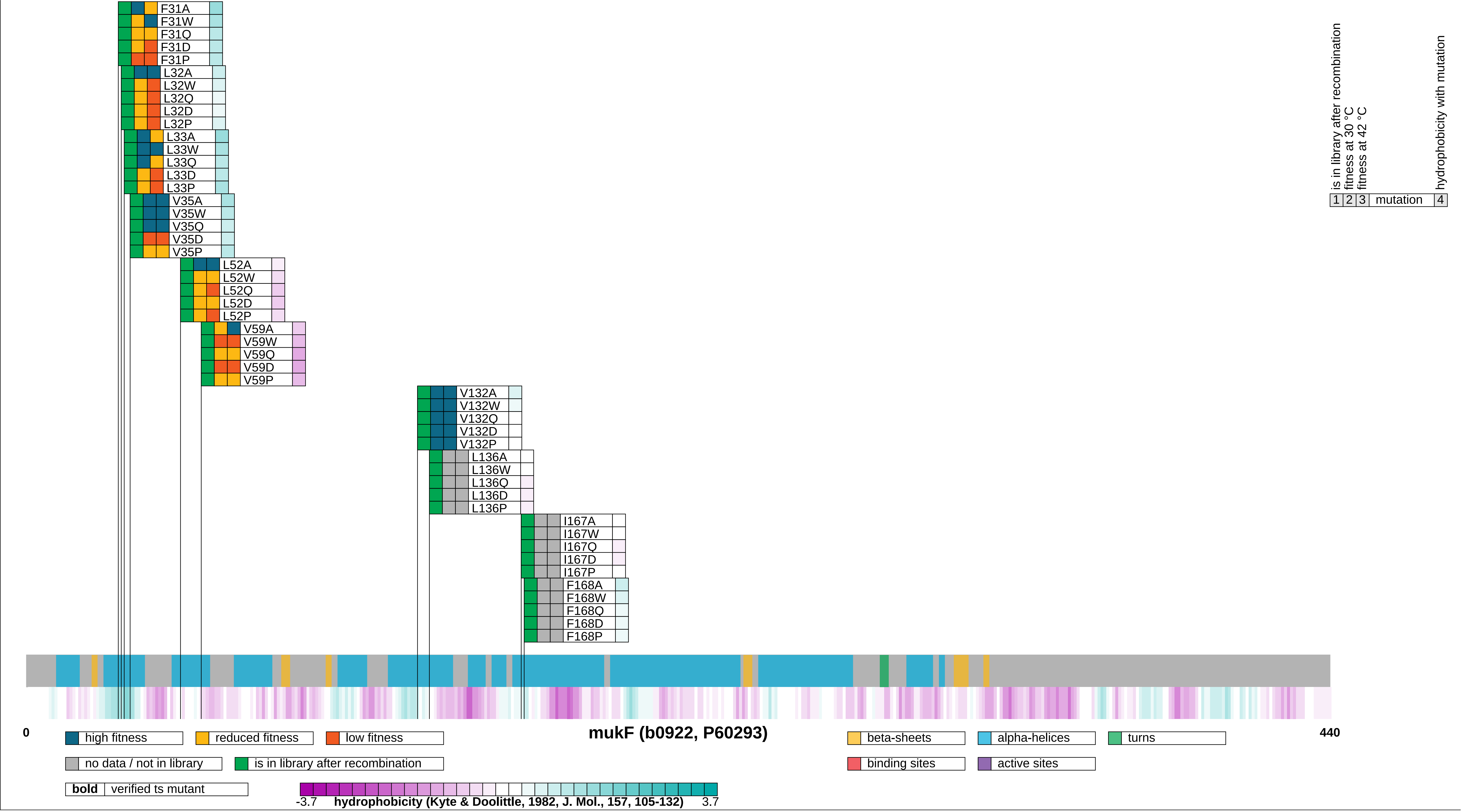

F31A
F31W
F31Q
F31D
F31P
L32A
L32W
L32Q
L32D
L32P
L33A
L33W
L33Q
L33D
L33P
V35A
V35W
V35Q
V35D
V35P
L52A
L52W
L52Q
L52D
L52P
V59A
V59W
V59Q
V59D
V59P
V132A
V132W
V132Q
V132D
V132P
L136A
L136W
L136Q
L136D
L136P
I167A
I167W
I167Q
I167D
I167P
F168A
F168W
F168Q
F168D
F168P
is in library after recombination
fitness at 30 °C
fitness at 42 °C
hydrophobicity with mutation
1
2
3
mutation
4
0
mukF (b0922, P60293)
440
high fitness
reduced fitness
low fitness
beta-sheets
alpha-helices
turns
no data / not in library
is in library after recombination
binding sites
active sites
bold
verified ts mutant
-3.7
hydrophobicity (Kyte & Doolittle, 1982, J. Mol., 157, 105-132)
3.7

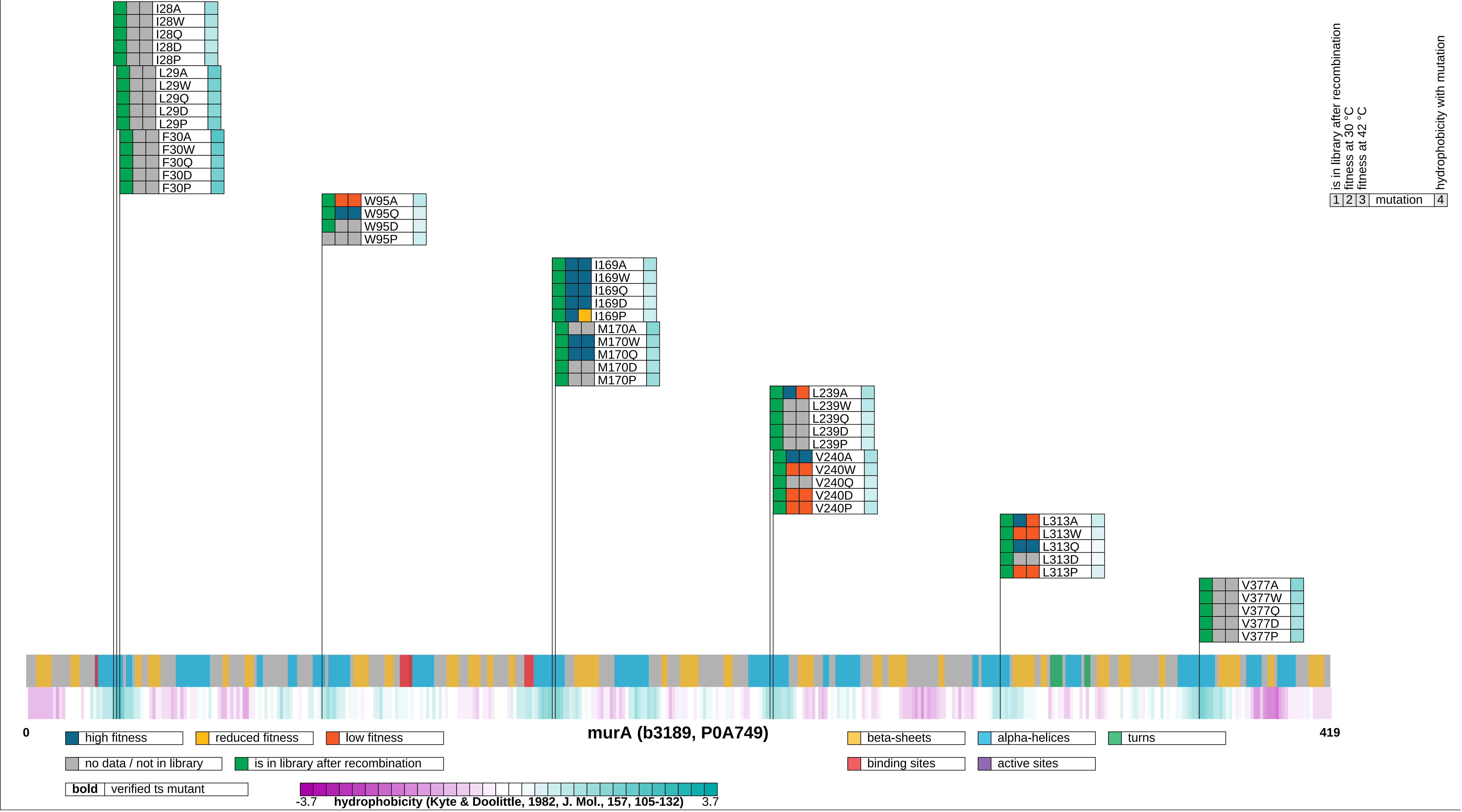
I28A
I28W
I28Q
I28D
I28P
L29A
L29W
L29Q
L29D
L29P
F30A
F30W
F30Q
F30D
F30P
W95A
W95Q
W95D
W95P
I169A
I169W
I169Q
I169D
I169P
M170A
M170W
M170Q
M170D
M170P
L239A
L239W
L239Q
L239D
L239P
V240A
V240W
V240Q
V240D
V240P
L313A
L313W
L313Q
L313D
L313P
V377A
V377W
V377Q
V377D
V377P
is in library after recombination
fitness at 30 °C
fitness at 42 °C
hydrophobicity with mutation
1 2 3 mutation 4
0
murA (b3189, P0A749)
419
high fitness
reduced fitness
low fitness
beta-sheets
alpha-helices
turns
no data / not in library
is in library after recombination
binding sites
active sites
bold verified ts mutant
-3.7 hydrophobicity (Kyte & Doolittle, 1982, J. Mol., 157, 105-132) 3.7

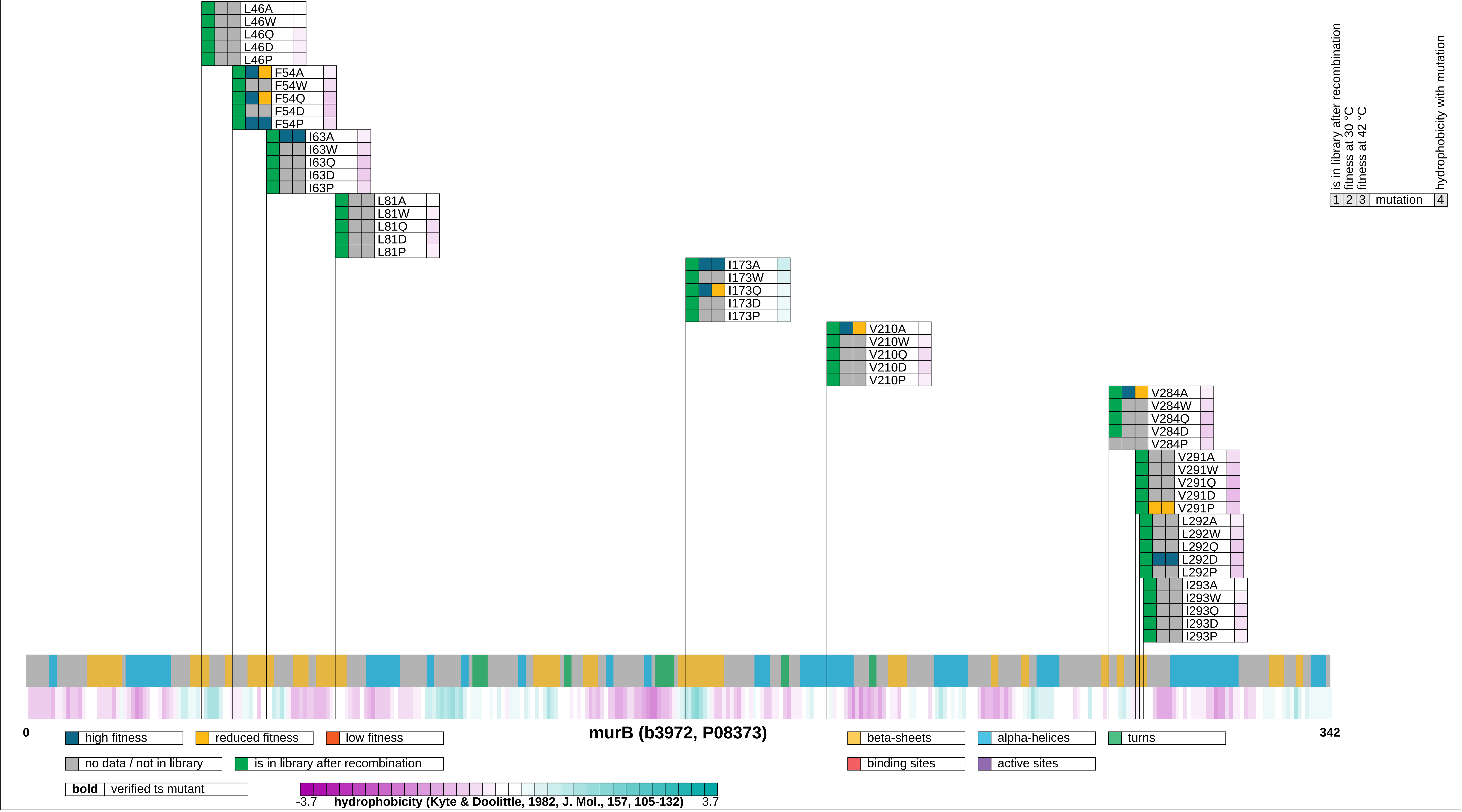

is in library after recombination
fitness at 30 °C
fitness at 42 °C
hydrophobicity with mutation
1 2 3 mutation 4
L46A
L46W
L46Q
L46D
L46P
F54A
F54W
F54Q
F54D
F54P
I63A
I63W
I63Q
I63D
I63P
L81A
L81W
L81Q
L81D
L81P
I173A
I173W
I173Q
I173D
I173P
V210A
V210W
V210Q
V210D
V210P
V284A
V284W
V284Q
V284D
V284P
V291A
V291W
V291Q
V291D
V291P
L292A
L292W
L292Q
L292D
L292P
I293A
I293W
I293Q
I293D
I293P
0
murB (b3972, P08373)
342
high fitness
reduced fitness
low fitness
beta-sheets
alpha-helices
turns
no data / not in library
is in library after recombination
binding sites
active sites
bold verified ts mutant
-3.7 hydrophobicity (Kyte & Doolittle, 1982, J. Mol., 157, 105-132) 3.7

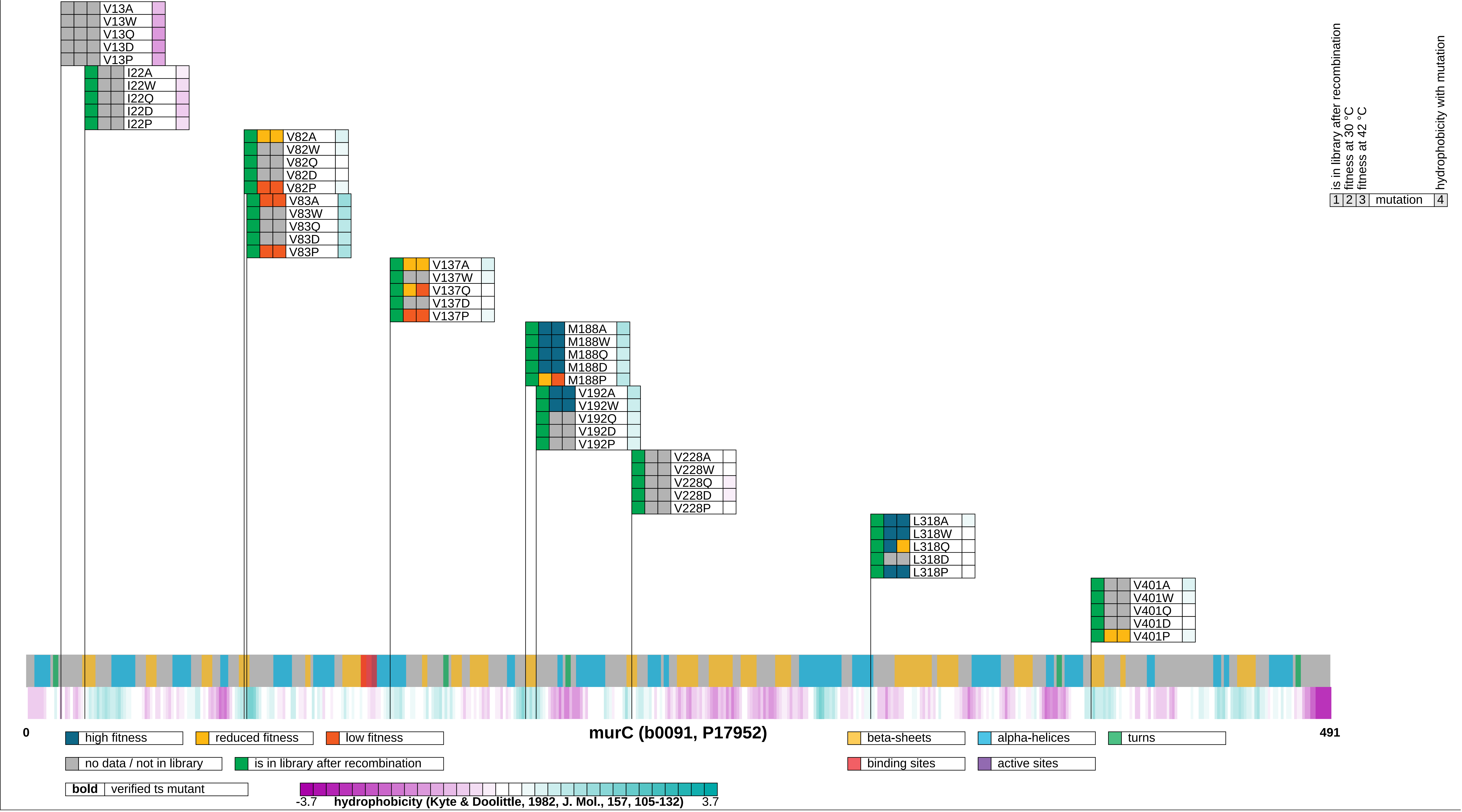

V13A
V13W
V13Q
V13D
V13P
I22A
I22W
I22Q
I22D
I22P
V82A
V82W
V82Q
V82D
V82P
V83A
V83W
V83Q
V83D
V83P
V137A
V137W
V137Q
V137D
V137P
M188A
M188W
M188Q
M188D
M188P
V192A
V192W
V192Q
V192D
V192P
V228A
V228W
V228Q
V228D
V228P
L318A
L318W
L318Q
L318D
L318P
V401A
V401W
V401Q
V401D
V401P
is in library after recombination
fitness at 30 °C
fitness at 42 °C
hydrophobicity with mutation
1 2 3 mutation 4
0
murC (b0091, P17952)
491
high fitness
reduced fitness
low fitness
no data / not in library
is in library after recombination
bold verified ts mutant
beta-sheets
alpha-helices
turns
binding sites
active sites
-3.7 hydrophobicity (Kyte & Doolittle, 1982, J. Mol., 157, 105-132) 3.7

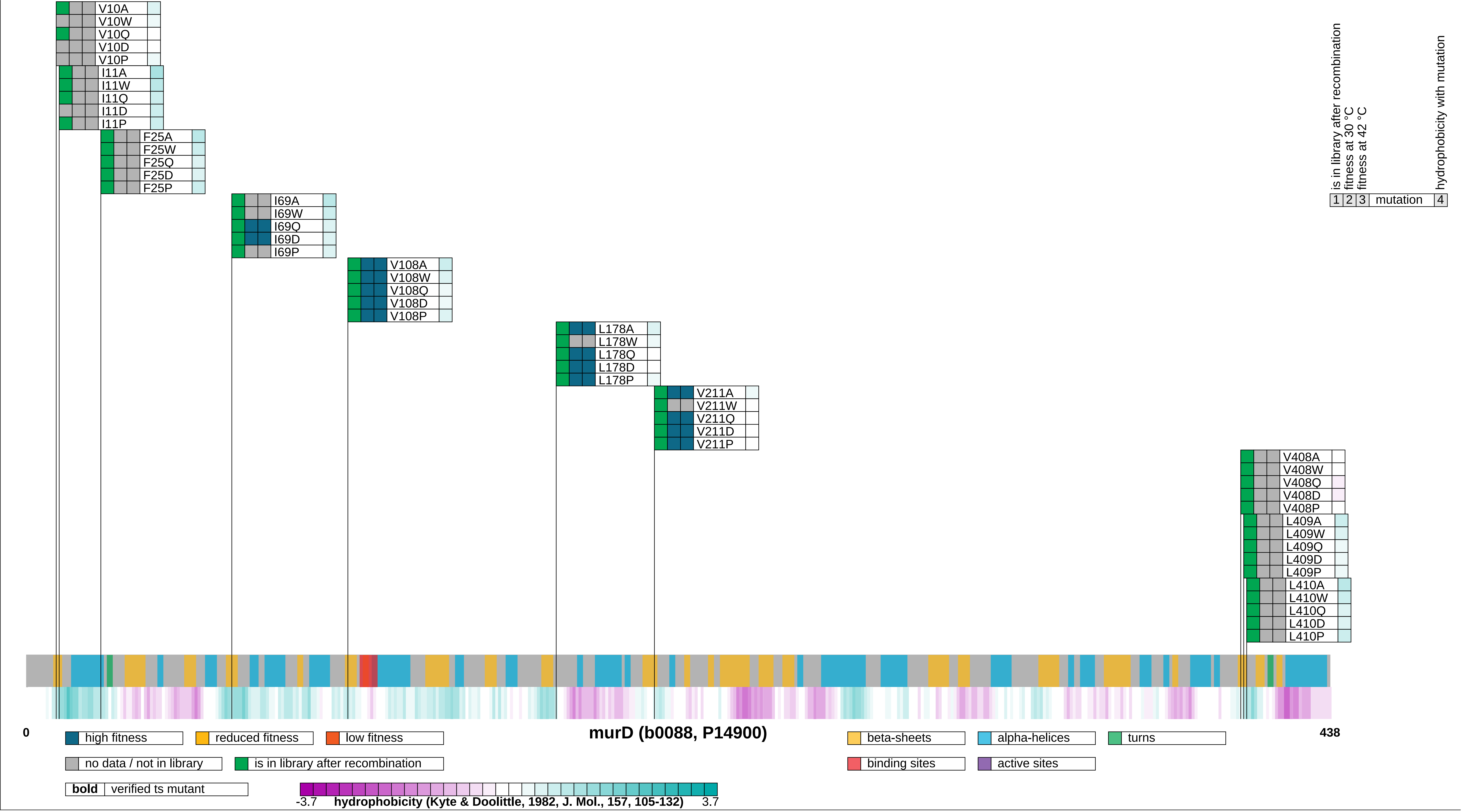

V10A
V10W
V10Q
V10D
V10P
I11A
I11W
I11Q
I11D
I11P
F25A
F25W
F25Q
F25D
F25P
I69A
I69W
I69Q
I69D
I69P
V108A
V108W
V108Q
V108D
V108P
L178A
L178W
L178Q
L178D
L178P
V211A
V211W
V211Q
V211D
V211P
V408A
V408W
V408Q
V408D
V408P
L409A
L409W
L409Q
L409D
L409P
L410A
L410W
L410Q
L410D
L410P
is in library after recombination
fitness at 30 °C
fitness at 42 °C
hydrophobicity with mutation
1 2 3 mutation 4
0
murD (b0088, P14900)
438
high fitness
reduced fitness
low fitness
beta-sheets
alpha-helices
turns
no data / not in library
is in library after recombination
binding sites
active sites
bold verified ts mutant
-3.7 hydrophobicity (Kyte & Doolittle, 1982, J. Mol., 157, 105-132) 3.7

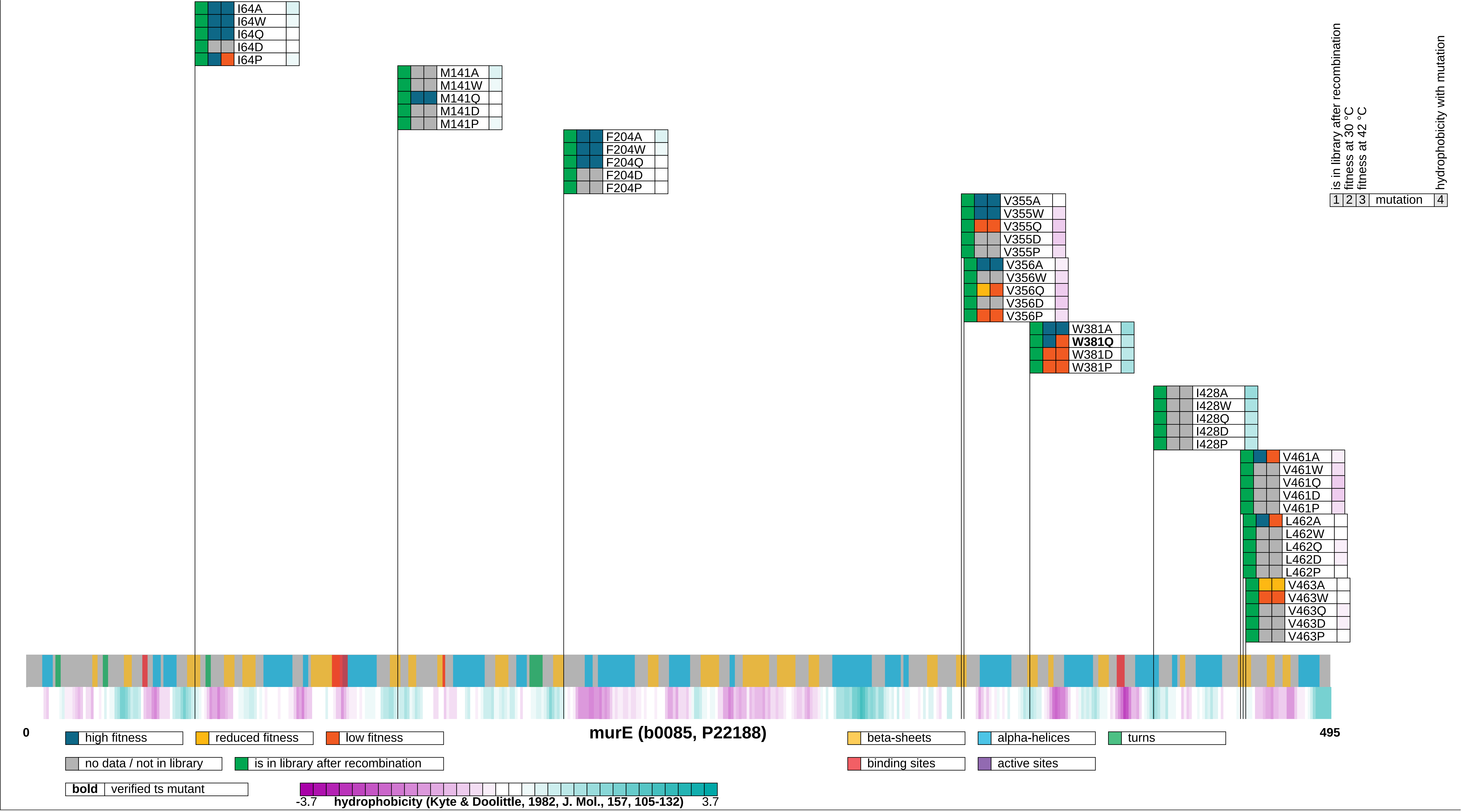

I64A
I64W
I64Q
I64D
I64P
M141A
M141W
M141Q
M141D
M141P
F204A
F204W
F204Q
F204D
F204P
V355A
V355W
V355Q
V355D
V355P
V356A
V356W
V356Q
V356D
V356P
W381A
W381Q
W381D
W381P
I428A
I428W
I428Q
I428D
I428P
V461A
V461W
V461Q
V461D
V461P
L462A
L462W
L462Q
L462D
L462P
V463A
V463W
V463Q
V463D
V463P
is in library after recombination
fitness at 30 °C
fitness at 42 °C
hydrophobicity with mutation
1 2 3 mutation 4
0
murE (b0085, P22188)
495
high fitness
reduced fitness
low fitness
beta-sheets
alpha-helices
turns
no data / not in library
is in library after recombination
binding sites
active sites
bold verified ts mutant
-3.7
hydrophobicity (Kyte & Doolittle, 1982, J. Mol., 157, 105-132)
3.7

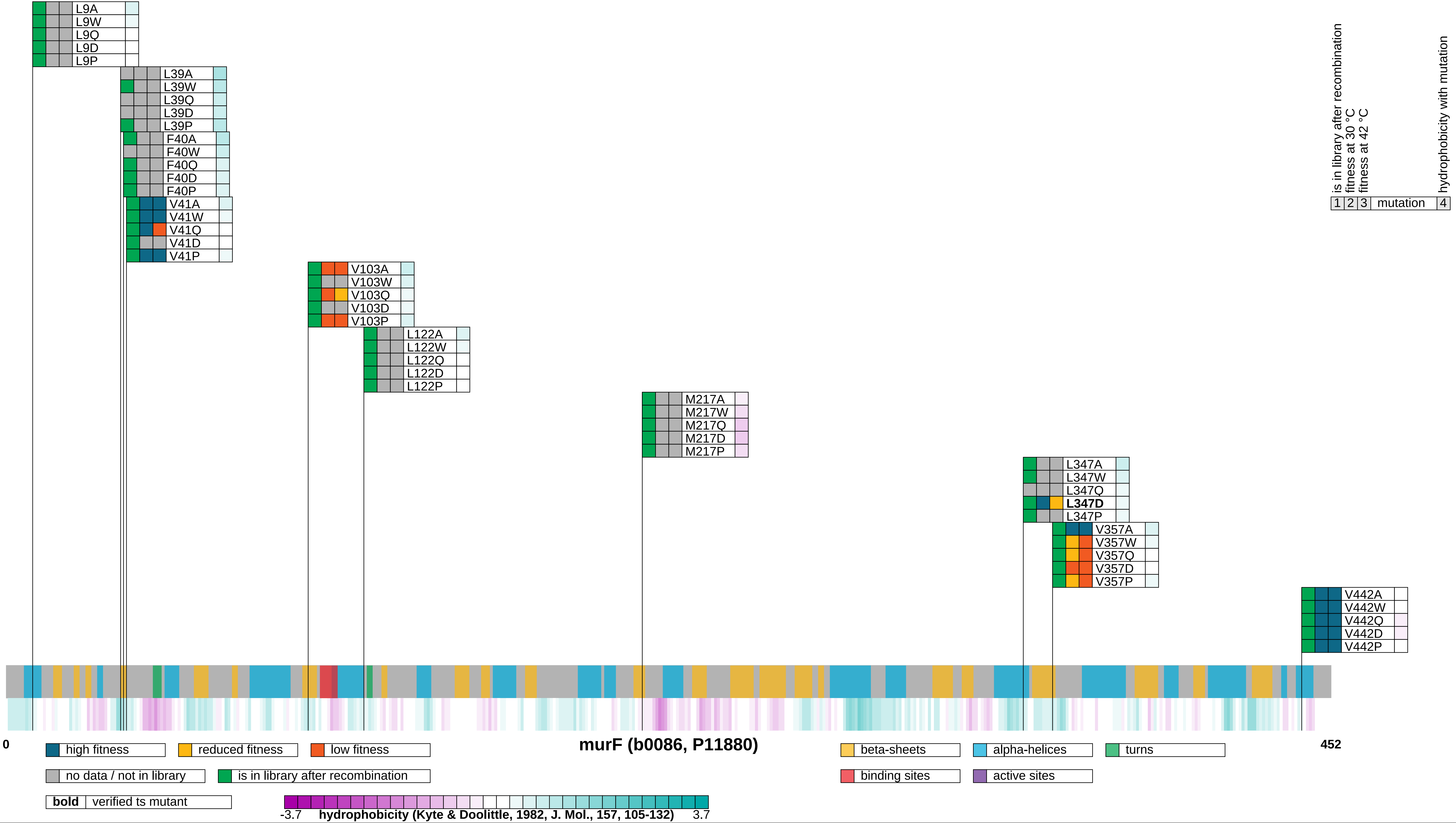
L9A
L9W
L9Q
L9D
L9P
L39A
L39W
L39Q
L39D
L39P
F40A
F40W
F40Q
F40D
F40P
V41A
V41W
V41Q
V41D
V41P
V103A
V103W
V103Q
V103D
V103P
L122A
L122W
L122Q
L122D
L122P
M217A
M217W
M217Q
M217D
M217P
L347A
L347W
L347Q
L347D
L347P
V357A
V357W
V357Q
V357D
V357P
V442A
V442W
V442Q
V442D
V442P
is in library after recombination
fitness at 30 °C
fitness at 42 °C
hydrophobicity with mutation
1 2 3 mutation 4
0
murF (b0086, P11880)
452
high fitness
reduced fitness
low fitness
beta-sheets
alpha-helices
turns
no data / not in library
is in library after recombination
binding sites
active sites
bold verified ts mutant
-3.7
hydrophobicity (Kyte & Doolittle, 1982, J. Mol., 157, 105-132)
3.7

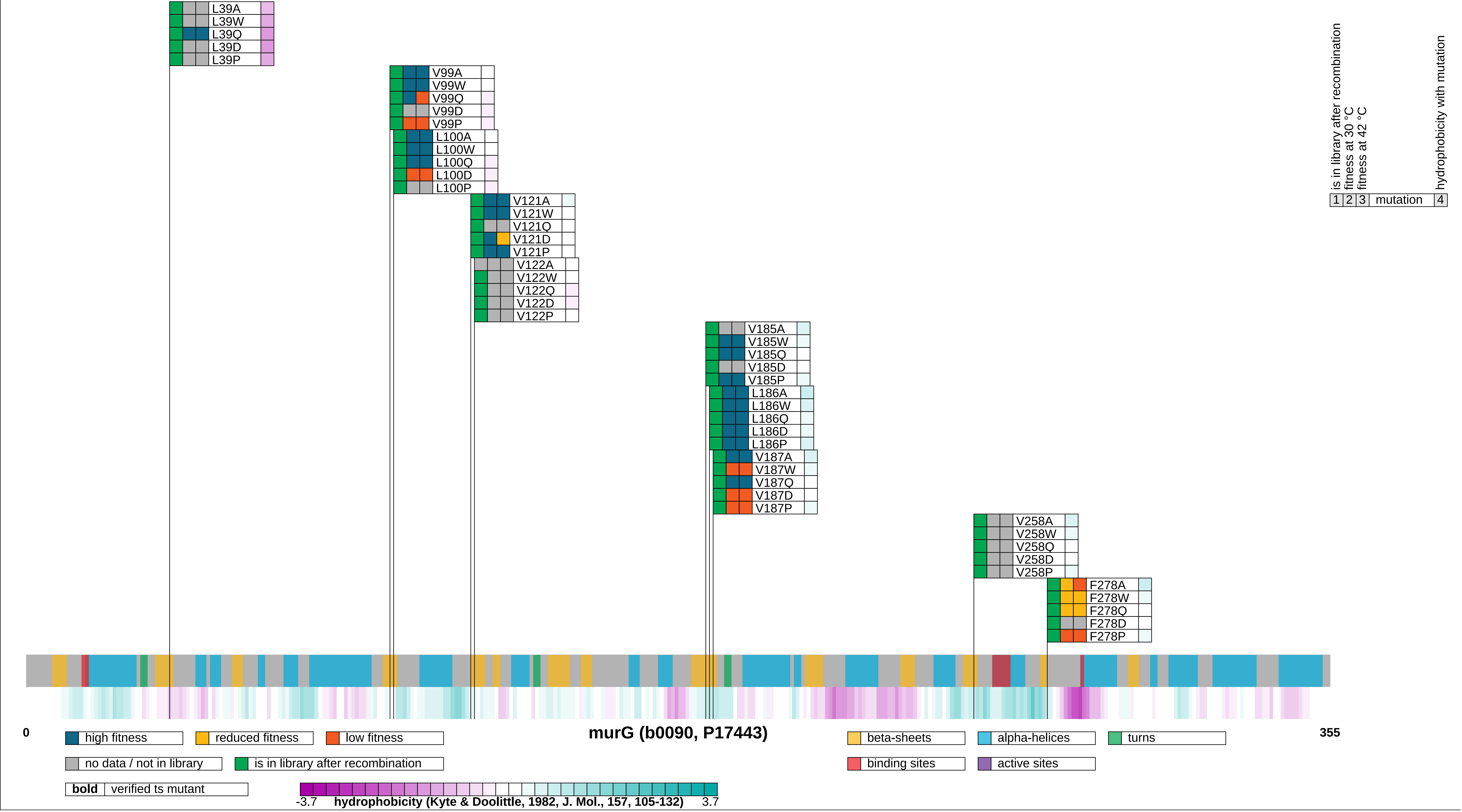
L39A
L39W
L39Q
L39D
L39P
V99A
V99W
V99Q
V99D
V99P
L100A
L100W
L100Q
L100D
L100P
V121A
V121W
V121Q
V121D
V121P
V122A
V122W
V122Q
V122D
V122P
V185A
V185W
V185Q
V185D
V185P
L186A
L186W
L186Q
L186D
L186P
V187A
V187W
V187Q
V187D
V187P
V258A
V258W
V258Q
V258D
V258P
F278A
F278W
F278Q
F278D
F278P
is in library after recombination
fitness at 30 °C
fitness at 42 °C
hydrophobicity with mutation
1 2 3 mutation 4
0
murG (b0090, P17443)
355
high fitness
reduced fitness
low fitness
no data / not in library
is in library after recombination
bold verified ts mutant
beta-sheets
alpha-helices
turns
binding sites
active sites
-3.7 hydrophobicity (Kyte & Doolittle, 1982, J. Mol., 157, 105-132) 3.7

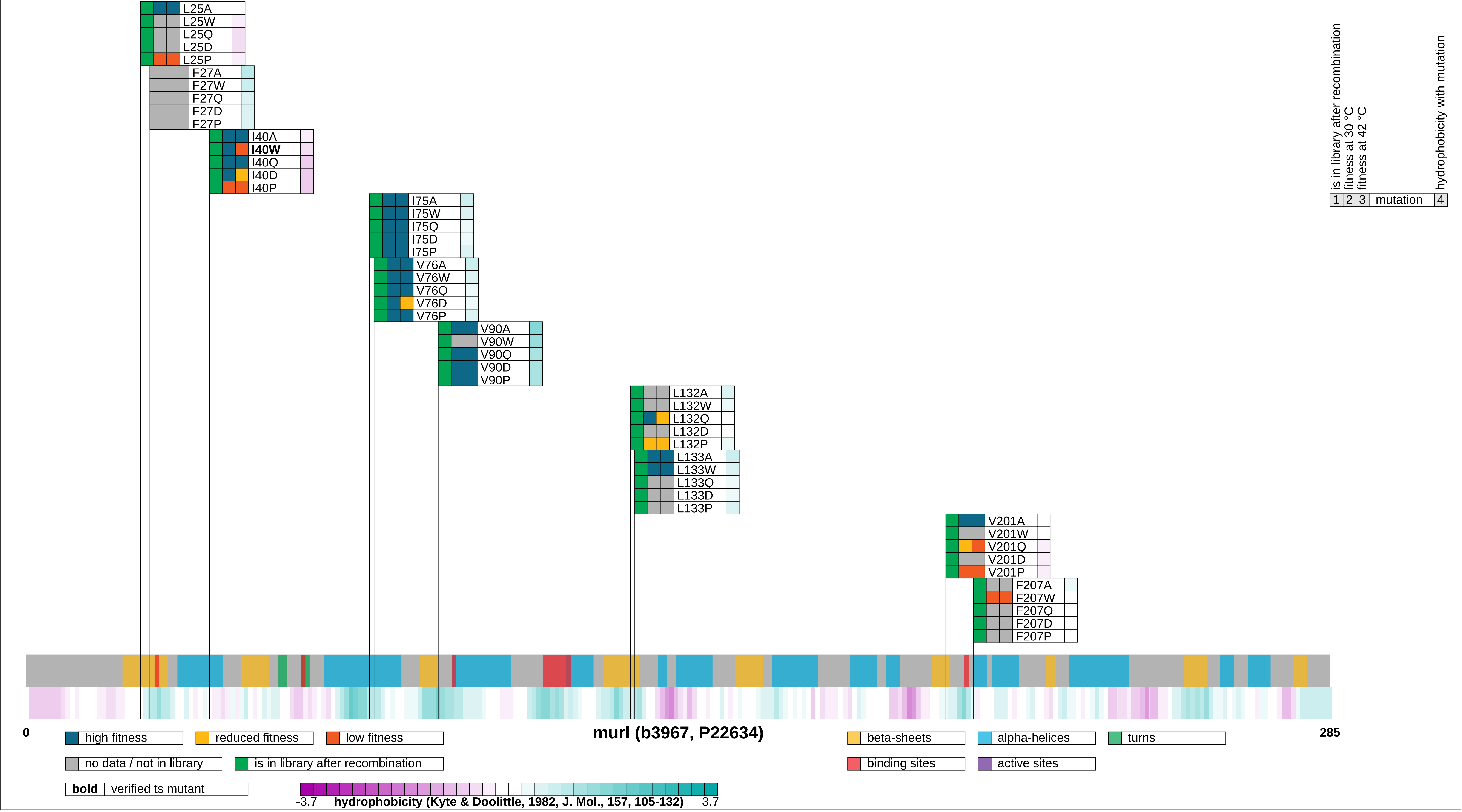

L25A
L25W
L25Q
L25D
L25P
F27A
F27W
F27Q
F27D
F27P
I40A
I40W
I40Q
I40D
I40P
I75A
I75W
I75Q
I75D
I75P
V76A
V76W
V76Q
V76D
V76P
V90A
V90W
V90Q
V90D
V90P
L132A
L132W
L132Q
L132D
L132P
L133A
L133W
L133Q
L133D
L133P
V201A
V201W
V201Q
V201D
V201P
F207A
F207W
F207Q
F207D
F207P
is in library after recombination
fitness at 30 °C
fitness at 42 °C
hydrophobicity with mutation
1 2 3 mutation 4
0
murI (b3967, P22634)
285
high fitness
reduced fitness
low fitness
no data / not in library
is in library after recombination
bold verified ts mutant
beta-sheets
alpha-helices
turns
binding sites
active sites
-3.7
hydrophobicity (Kyte & Doolittle, 1982, J. Mol., 157, 105-132)
3.7

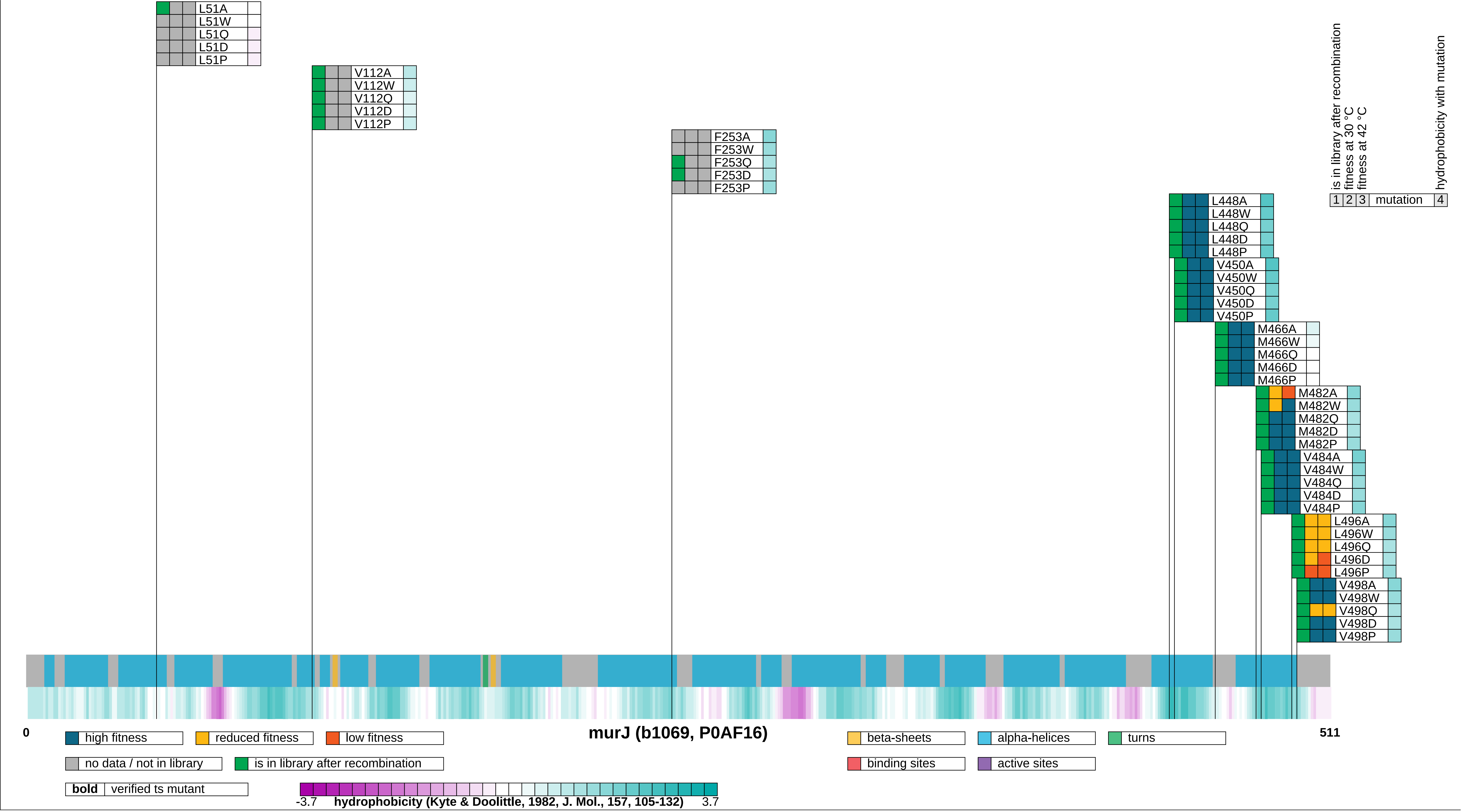

L51A
L51W
L51Q
L51D
L51P
V112A
V112W
V112Q
V112D
V112P
F253A
F253W
F253Q
F253D
F253P
L448A
L448W
L448Q
L448D
L448P
V450A
V450W
V450Q
V450D
V450P
M466A
M466W
M466Q
M466D
M466P
M482A
M482W
M482Q
M482D
M482P
V484A
V484W
V484Q
V484D
V484P
L496A
L496W
L496Q
L496D
L496P
V498A
V498W
V498Q
V498D
V498P
is in library after recombination
fitness at 30 °C
fitness at 42 °C
hydrophobicity with mutation
1 2 3 mutation 4
0
murJ (b1069, P0AF16)
511
high fitness
reduced fitness
low fitness
beta-sheets
alpha-helices
turns
no data / not in library
is in library after recombination
binding sites
active sites
bold verified ts mutant
-3.7 hydrophobicity (Kyte & Doolittle, 1982, J. Mol., 157, 105-132) 3.7

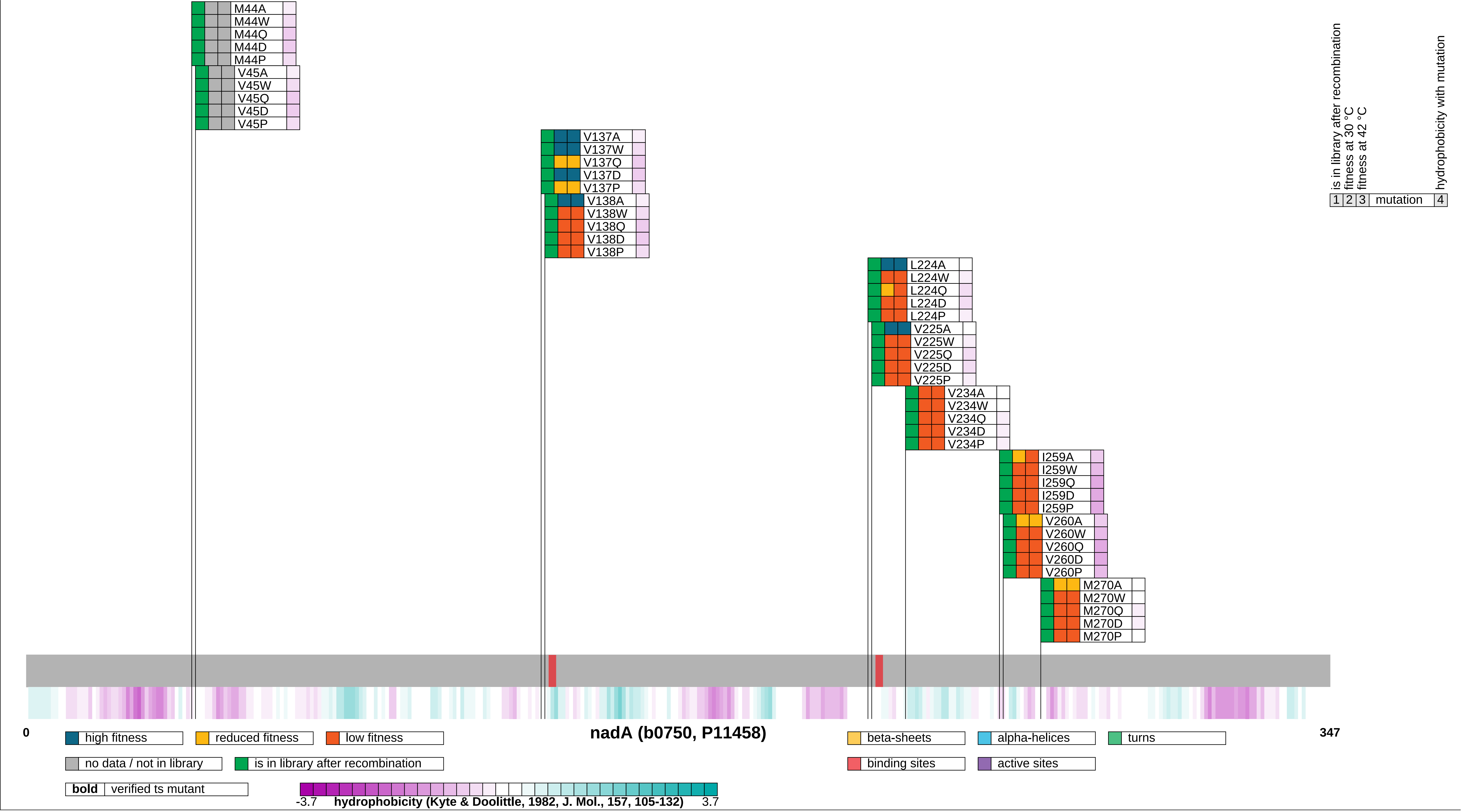

M44A
M44W
M44Q
M44D
M44P
V45A
V45W
V45Q
V45D
V45P
V137A
V137W
V137Q
V137D
V137P
V138A
V138W
V138Q
V138D
V138P
L224A
L224W
L224Q
L224D
L224P
V225A
V225W
V225Q
V225D
V225P
V234A
V234W
V234Q
V234D
V234P
I259A
I259W
I259Q
I259D
I259P
V260A
V260W
V260Q
V260D
V260P
M270A
M270W
M270Q
M270D
M270P
is in library after recombination
fitness at 30 °C
fitness at 42 °C
hydrophobicity with mutation
1 2 3 mutation 4
0
nadA (b0750, P11458)
347
high fitness
reduced fitness
low fitness
no data / not in library
is in library after recombination
bold verified ts mutant
beta-sheets
alpha-helices
turns
binding sites
active sites
-3.7 hydrophobicity (Kyte & Doolittle, 1982, J. Mol., 157, 105-132) 3.7

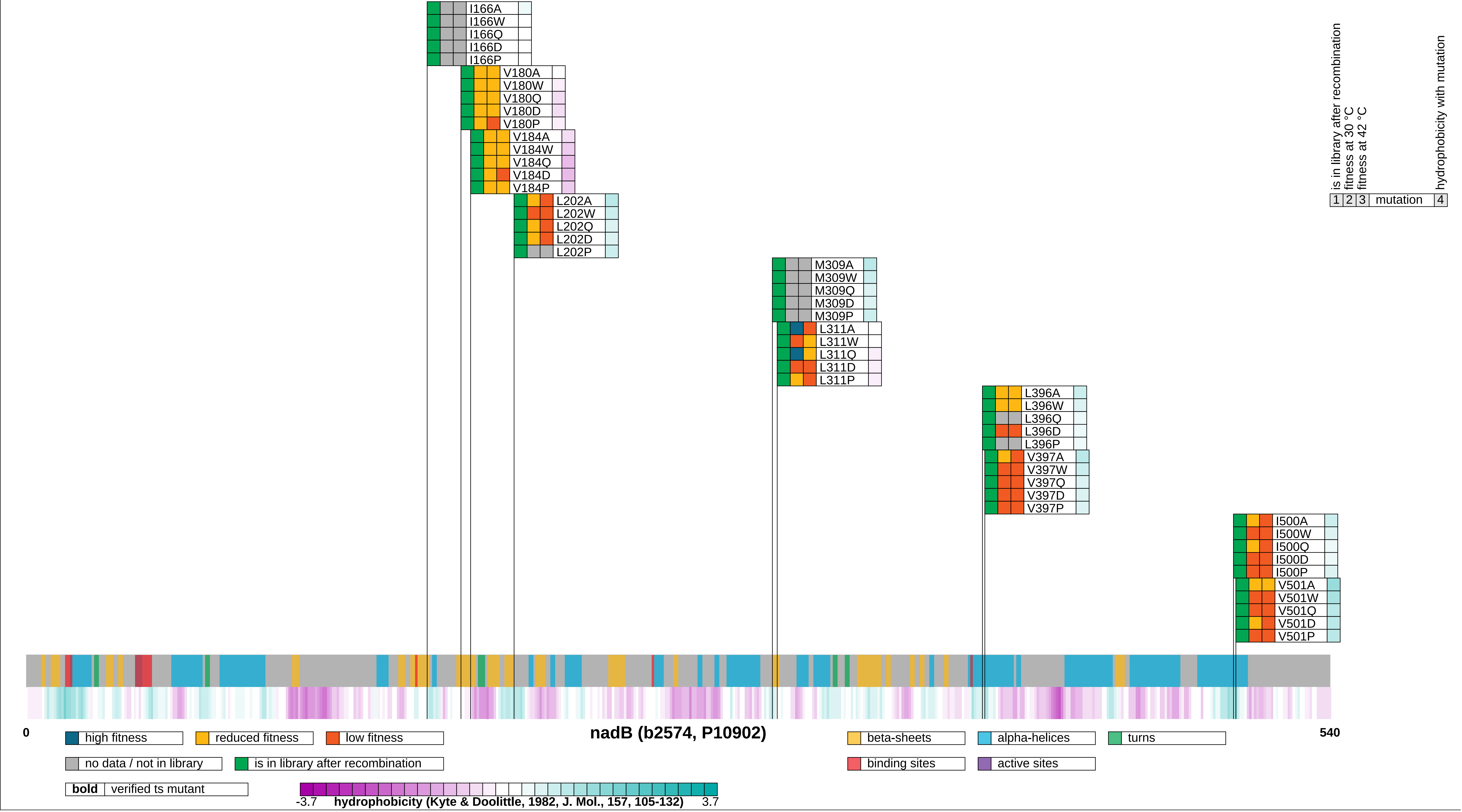

I166A
I166W
I166Q
I166D
I166P
V180A
V180W
V180Q
V180D
V180P
V184A
V184W
V184Q
V184D
V184P
L202A
L202W
L202Q
L202D
L202P
M309A
M309W
M309Q
M309D
M309P
L311A
L311W
L311Q
L311D
L311P
L396A
L396W
L396Q
L396D
L396P
V397A
V397W
V397Q
V397D
V397P
I500A
I500W
I500Q
I500D
I500P
V501A
V501W
V501Q
V501D
V501P
is in library after recombination
fitness at 30 °C
fitness at 42 °C
hydrophobicity with mutation
1 2 3 mutation 4
0
540
nadB (b2574, P10902)
high fitness
reduced fitness
low fitness
no data / not in library
is in library after recombination
bold verified ts mutant
beta-sheets
alpha-helices
turns
binding sites
active sites
-3.7
hydrophobicity (Kyte & Doolittle, 1982, J. Mol., 157, 105-132)
3.7

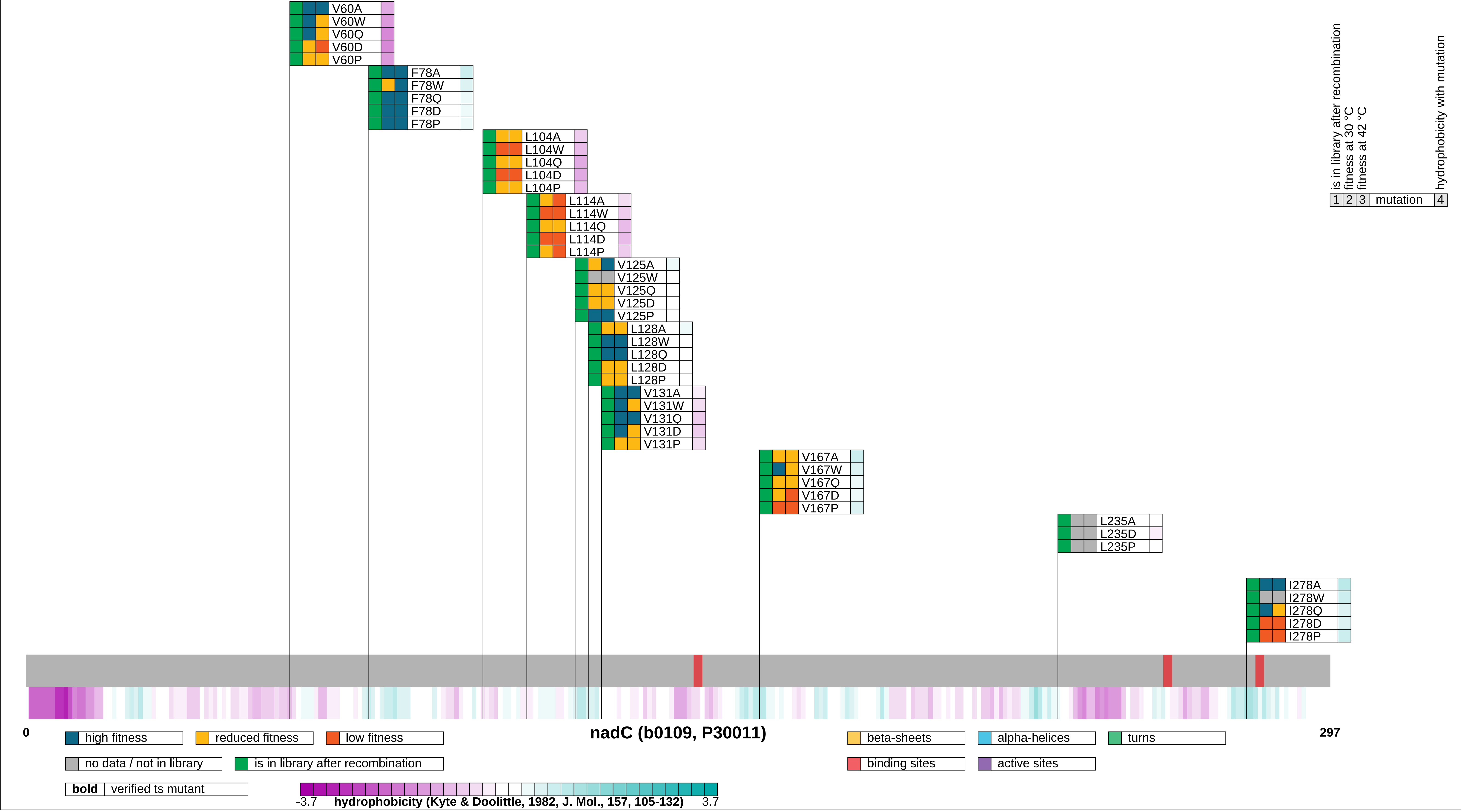

V60A
V60W
V60Q
V60D
V60P
F78A
F78W
F78Q
F78D
F78P
L104A
L104W
L104Q
L104D
L104P
L114A
L114W
L114Q
L114D
L114P
V125A
V125W
V125Q
V125D
V125P
L128A
L128W
L128Q
L128D
L128P
V131A
V131W
V131Q
V131D
V131P
V167A
V167W
V167Q
V167D
V167P
L235A
L235D
L235P
I278A
I278W
I278Q
I278D
I278P
is in library after recombination
fitness at 30 °C
fitness at 42 °C
hydrophobicity with mutation
1 2 3 mutation 4
0
nadC (b0109, P30011)
297
high fitness
reduced fitness
low fitness
no data / not in library
is in library after recombination
bold verified ts mutant
beta-sheets
alpha-helices
turns
binding sites
active sites
-3.7 hydrophobicity (Kyte & Doolittle, 1982, J. Mol., 157, 105-132) 3.7

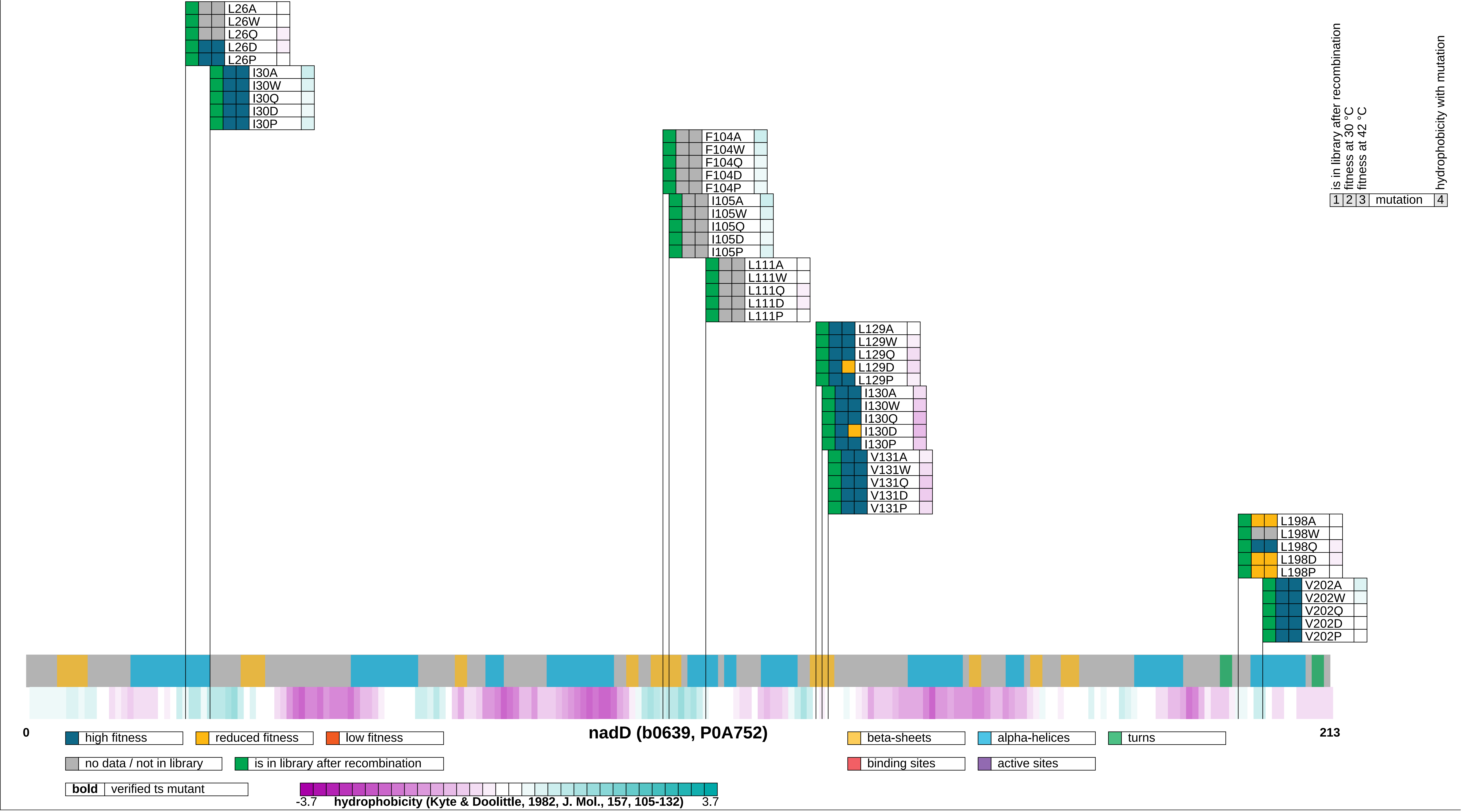

L26A
L26W
L26Q
L26D
L26P
I30A
I30W
I30Q
I30D
I30P
F104A
F104W
F104Q
F104D
F104P
I105A
I105W
I105Q
I105D
I105P
L111A
L111W
L111Q
L111D
L111P
L129A
L129W
L129Q
L129D
L129P
I130A
I130W
I130Q
I130D
I130P
V131A
V131W
V131Q
V131D
V131P
L198A
L198W
L198Q
L198D
L198P
V202A
V202W
V202Q
V202D
V202P
is in library after recombination
fitness at 30 °C
fitness at 42 °C
hydrophobicity with mutation
1 2 3 mutation 4
0
nadD (b0639, P0A752)
213
high fitness
reduced fitness
low fitness
beta-sheets
alpha-helices
turns
no data / not in library
is in library after recombination
binding sites
active sites
bold verified ts mutant
-3.7 hydrophobicity (Kyte & Doolittle, 1982, J. Mol., 157, 105-132) 3.7

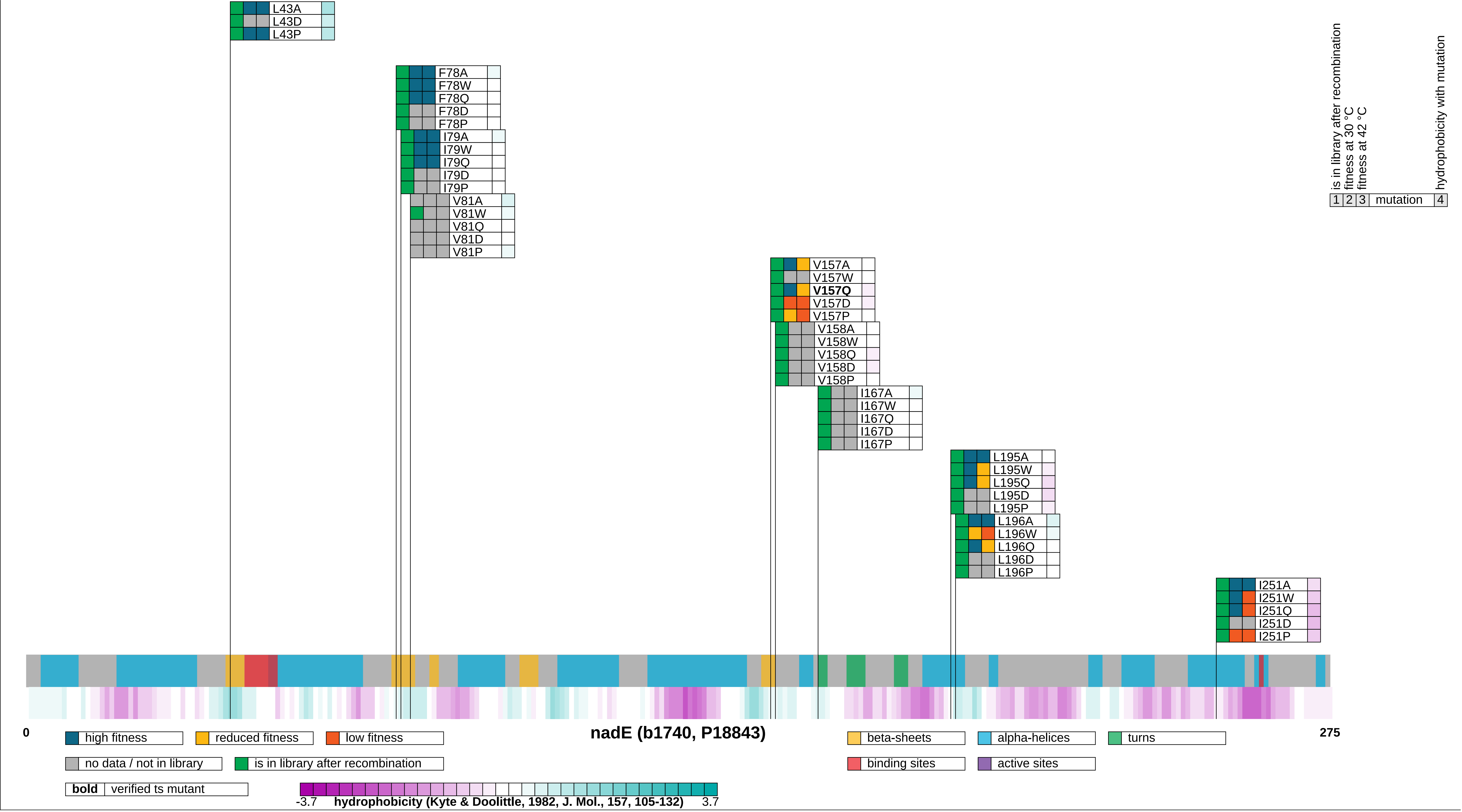

L43A
L43D
L43P
F78A
F78W
F78Q
F78D
F78P
I79A
I79W
I79Q
I79D
I79P
V81A
V81W
V81Q
V81D
V81P
V157A
V157W
V157Q
V157D
V157P
V158A
V158W
V158Q
V158D
V158P
I167A
I167W
I167Q
I167D
I167P
L195A
L195W
L195Q
L195D
L195P
L196A
L196W
L196Q
L196D
L196P
I251A
I251W
I251Q
I251D
I251P
is in library after recombination
fitness at 30 °C
fitness at 42 °C
hydrophobicity with mutation
1 2 3 mutation 4
0
nadE (b1740, P18843)
275
high fitness
reduced fitness
low fitness
no data / not in library
is in library after recombination
bold verified ts mutant
beta-sheets
alpha-helices
turns
binding sites
active sites
-3.7
hydrophobicity (Kyte & Doolittle, 1982, J. Mol., 157, 105-132)
3.7

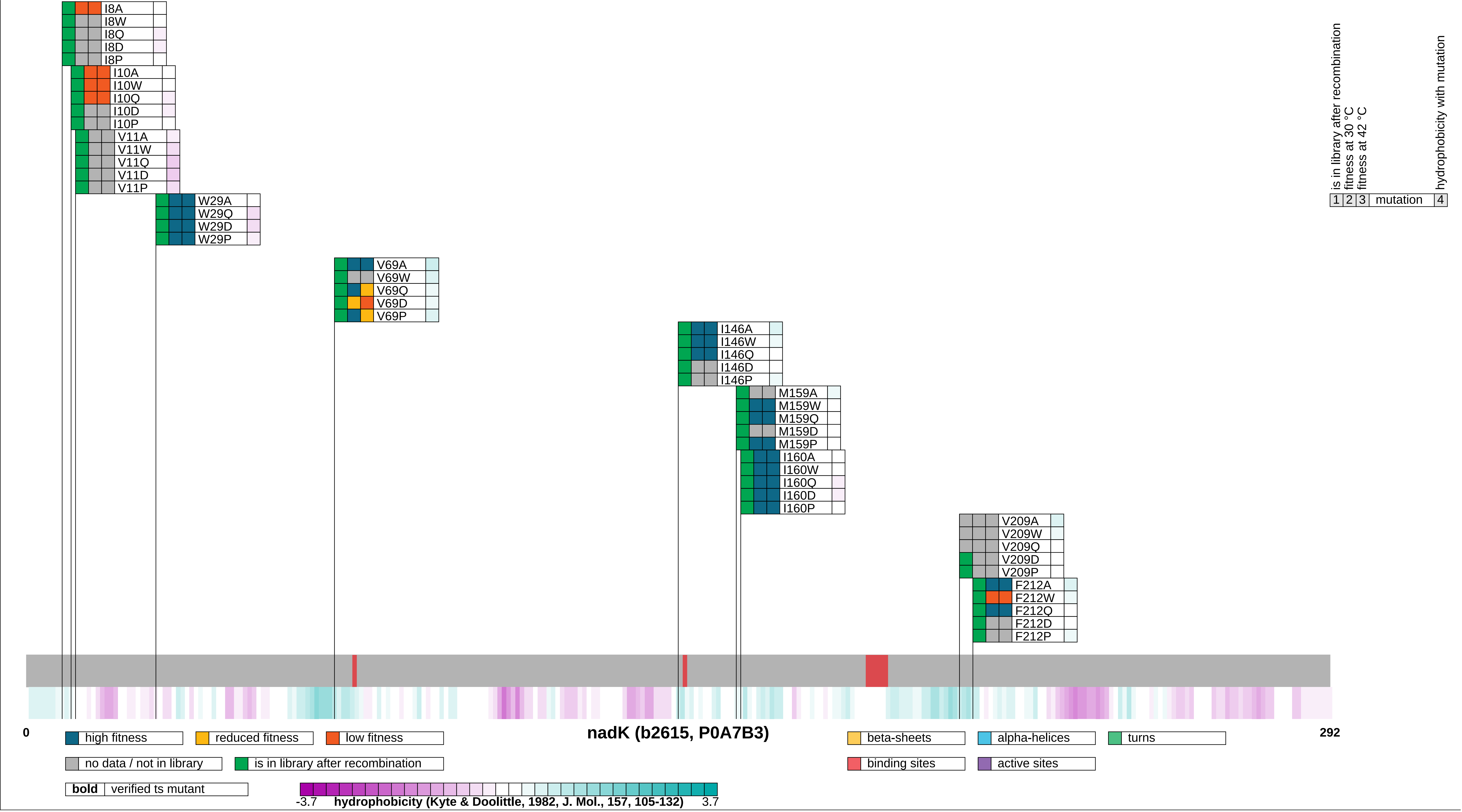

I8A
I8W
I8Q
I8D
I8P
I10A
I10W
I10Q
I10D
I10P
V11A
V11W
V11Q
V11D
V11P
W29A
W29Q
W29D
W29P
V69A
V69W
V69Q
V69D
V69P
I146A
I146W
I146Q
I146D
I146P
M159A
M159W
M159Q
M159D
M159P
I160A
I160W
I160Q
I160D
I160P
V209A
V209W
V209Q
V209D
V209P
F212A
F212W
F212Q
F212D
F212P
is in library after recombination
fitness at 30 °C
fitness at 42 °C
hydrophobicity with mutation
1 2 3 mutation 4
0
292
nadK (b2615, P0A7B3)
high fitness
reduced fitness
low fitness
no data / not in library
is in library after recombination
bold verified ts mutant
beta-sheets
alpha-helices
turns
binding sites
active sites
-3.7
hydrophobicity (Kyte & Doolittle, 1982, J. Mol., 157, 105-132)
3.7

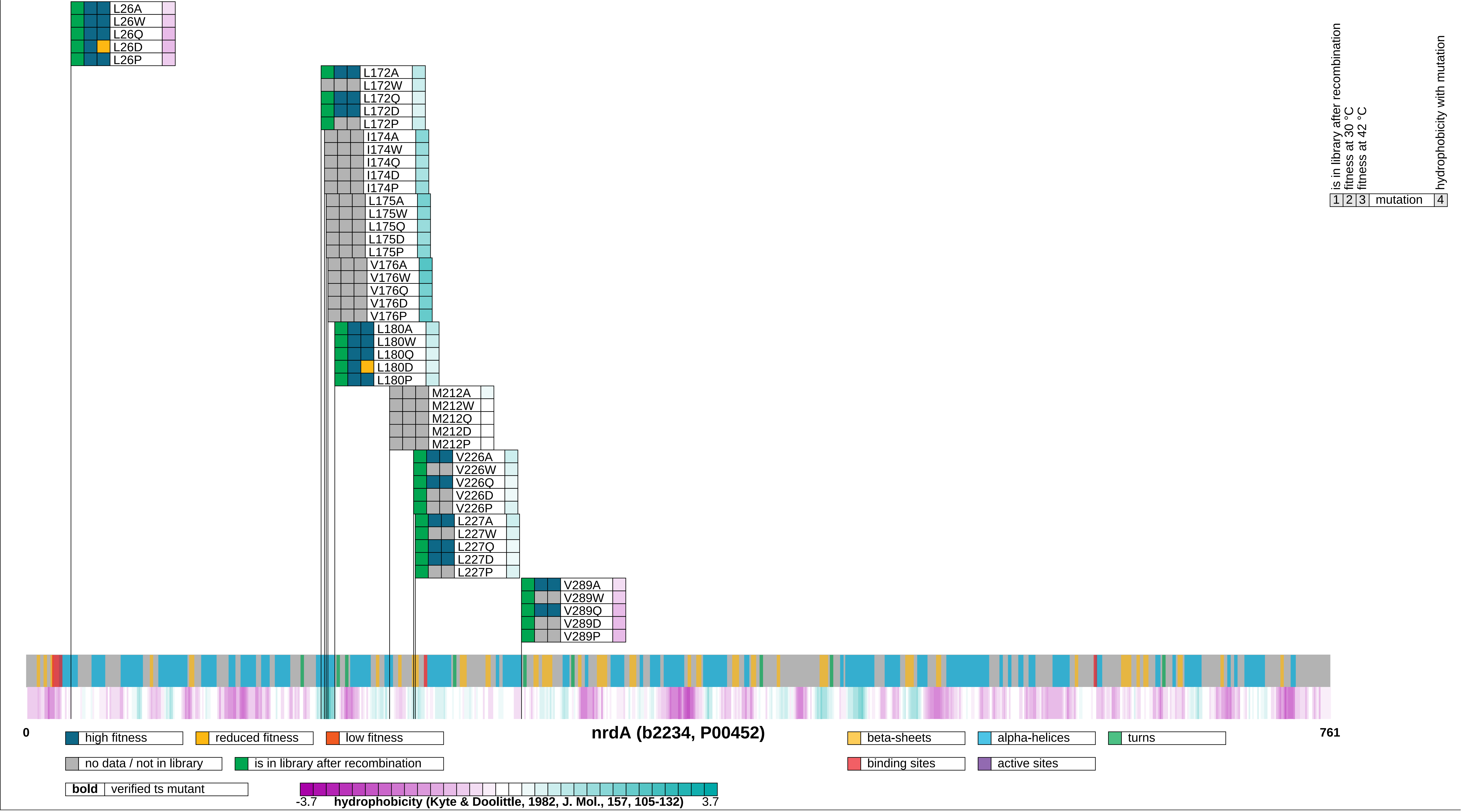

L26A
L26W
L26Q
L26D
L26P
L172A
L172W
L172Q
L172D
L172P
I174A
I174W
I174Q
I174D
I174P
L175A
L175W
L175Q
L175D
L175P
V176A
V176W
V176Q
V176D
V176P
L180A
L180W
L180Q
L180D
L180P
M212A
M212W
M212Q
M212D
M212P
V226A
V226W
V226Q
V226D
V226P
L227A
L227W
L227Q
L227D
L227P
V289A
V289W
V289Q
V289D
V289P
is in library after recombination
fitness at 30 °C
fitness at 42 °C
hydrophobicity with mutation
1 2 3 mutation 4
0
nrdA (b2234, P00452)
761
high fitness
reduced fitness
low fitness
beta-sheets
alpha-helices
turns
no data / not in library
is in library after recombination
binding sites
active sites
bold verified ts mutant
-3.7
hydrophobicity (Kyte & Doolittle, 1982, J. Mol., 157, 105-132)
3.7

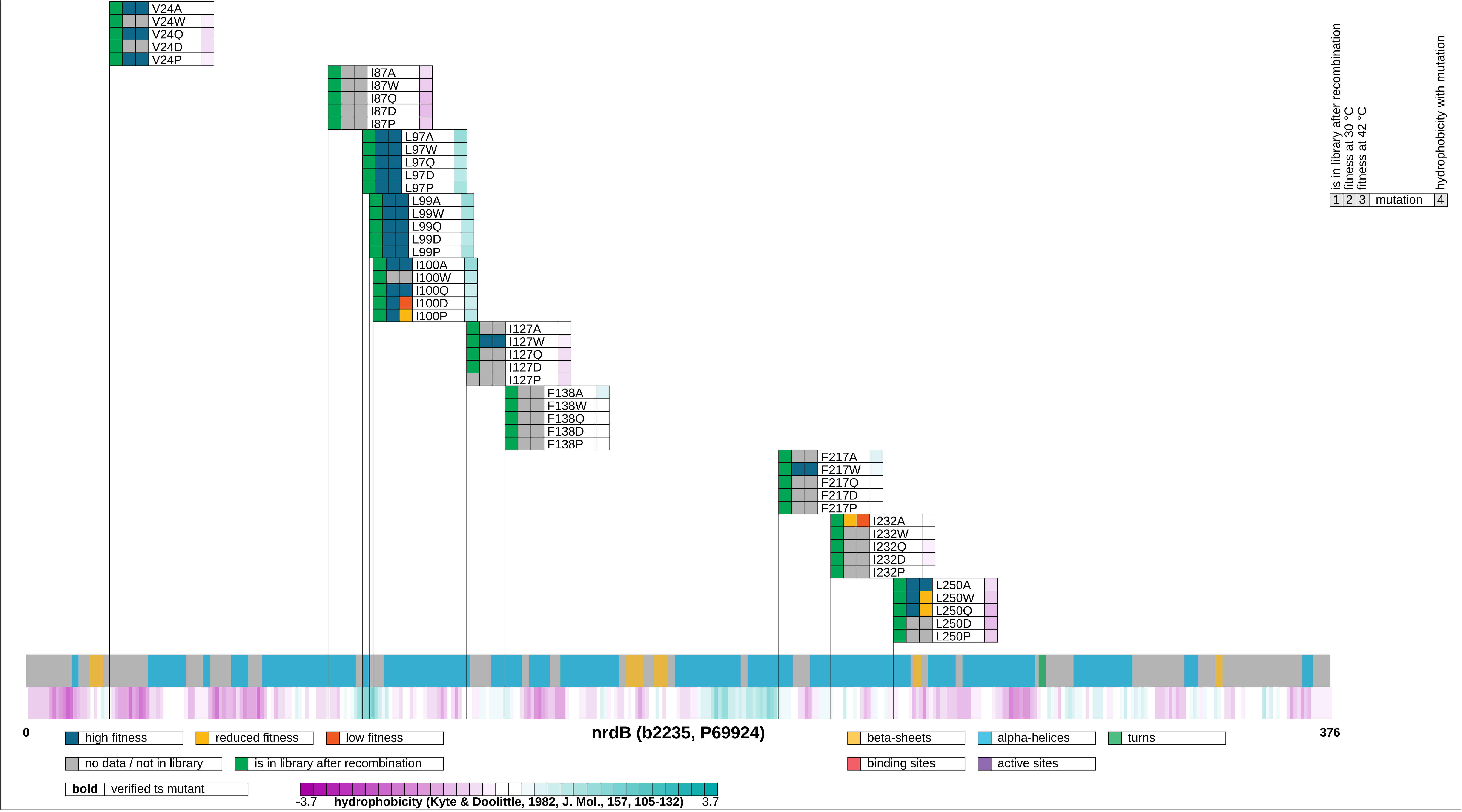
V24A
V24W
V24Q
V24D
V24P
I87A
I87W
I87Q
I87D
I87P
L97A
L97W
L97Q
L97D
L97P
L99A
L99W
L99Q
L99D
L99P
I100A
I100W
I100Q
I100D
I100P
I127A
I127W
I127Q
I127D
I127P
F138A
F138W
F138Q
F138D
F138P
F217A
F217W
F217Q
F217D
F217P
I232A
I232W
I232Q
I232D
I232P
L250A
L250W
L250Q
L250D
L250P
is in library after recombination
fitness at 30 °C
fitness at 42 °C
hydrophobicity with mutation
1
2
3
mutation
4
0
nrdB (b2235, P69924)
376
high fitness
reduced fitness
low fitness
beta-sheets
alpha-helices
turns
no data / not in library
is in library after recombination
binding sites
active sites
bold
verified ts mutant
-3.7
hydrophobicity (Kyte & Doolittle, 1982, J. Mol., 157, 105-132)
3.7

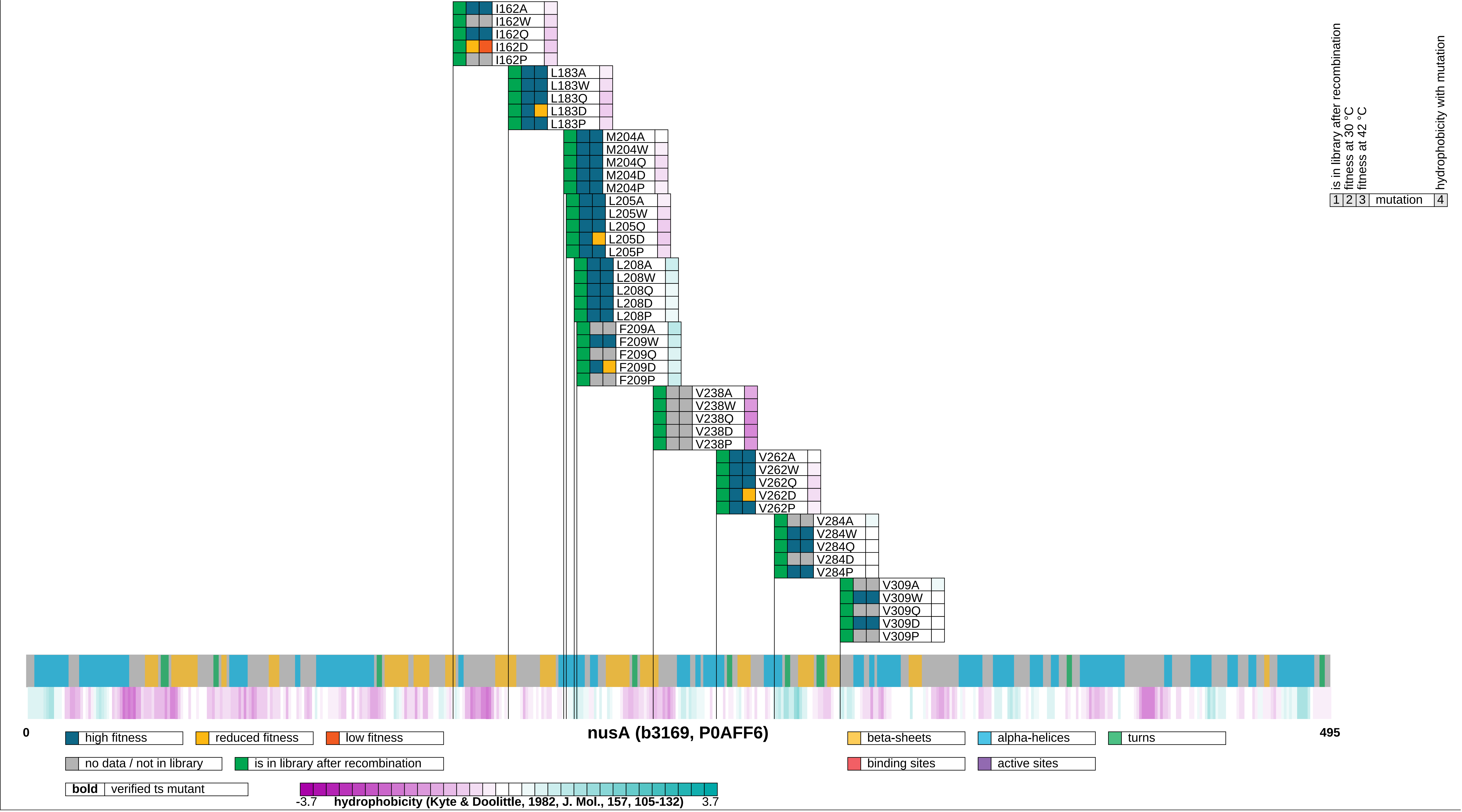
I162A
I162W
I162Q
I162D
I162P
L183A
L183W
L183Q
L183D
L183P
M204A
M204W
M204Q
M204D
M204P
L205A
L205W
L205Q
L205D
L205P
L208A
L208W
L208Q
L208D
L208P
F209A
F209W
F209Q
F209D
F209P
V238A
V238W
V238Q
V238D
V238P
V262A
V262W
V262Q
V262D
V262P
V284A
V284W
V284Q
V284D
V284P
V309A
V309W
V309Q
V309D
V309P
is in library after recombination
fitness at 30 °C
fitness at 42 °C
hydrophobicity with mutation
1 2 3 mutation 4
0
495
nusA (b3169, P0AFF6)
high fitness
reduced fitness
low fitness
no data / not in library
is in library after recombination
bold verified ts mutant
beta-sheets
alpha-helices
turns
binding sites
active sites
-3.7
hydrophobicity (Kyte & Doolittle, 1982, J. Mol., 157, 105-132)
3.7

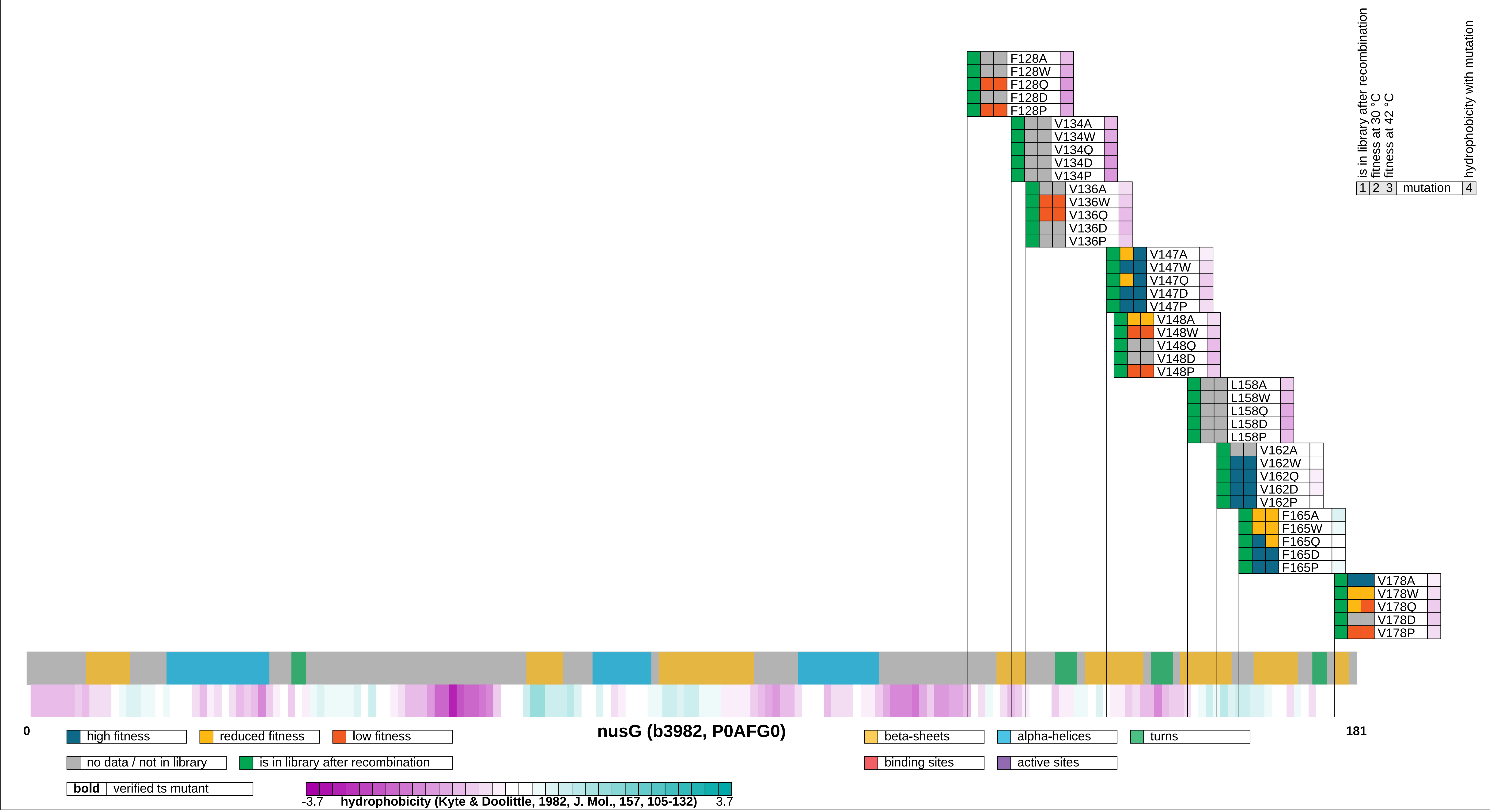

is in library after recombination
fitness at 30 °C
fitness at 42 °C
hydrophobicity with mutation
1 2 3 mutation 4
F128A
F128W
F128Q
F128D
F128P
V134A
V134W
V134Q
V134D
V134P
V136A
V136W
V136Q
V136D
V136P
V147A
V147W
V147Q
V147D
V147P
V148A
V148W
V148Q
V148D
V148P
L158A
L158W
L158Q
L158D
L158P
V162A
V162W
V162Q
V162D
V162P
F165A
F165W
F165Q
F165D
F165P
V178A
V178W
V178Q
V178D
V178P
0
nusG (b3982, P0AFG0)
181
high fitness
reduced fitness
low fitness
beta-sheets
alpha-helices
turns
no data / not in library
is in library after recombination
binding sites
active sites
bold verified ts mutant
-3.7 hydrophobicity (Kyte & Doolittle, 1982, J. Mol., 157, 105-132) 3.7

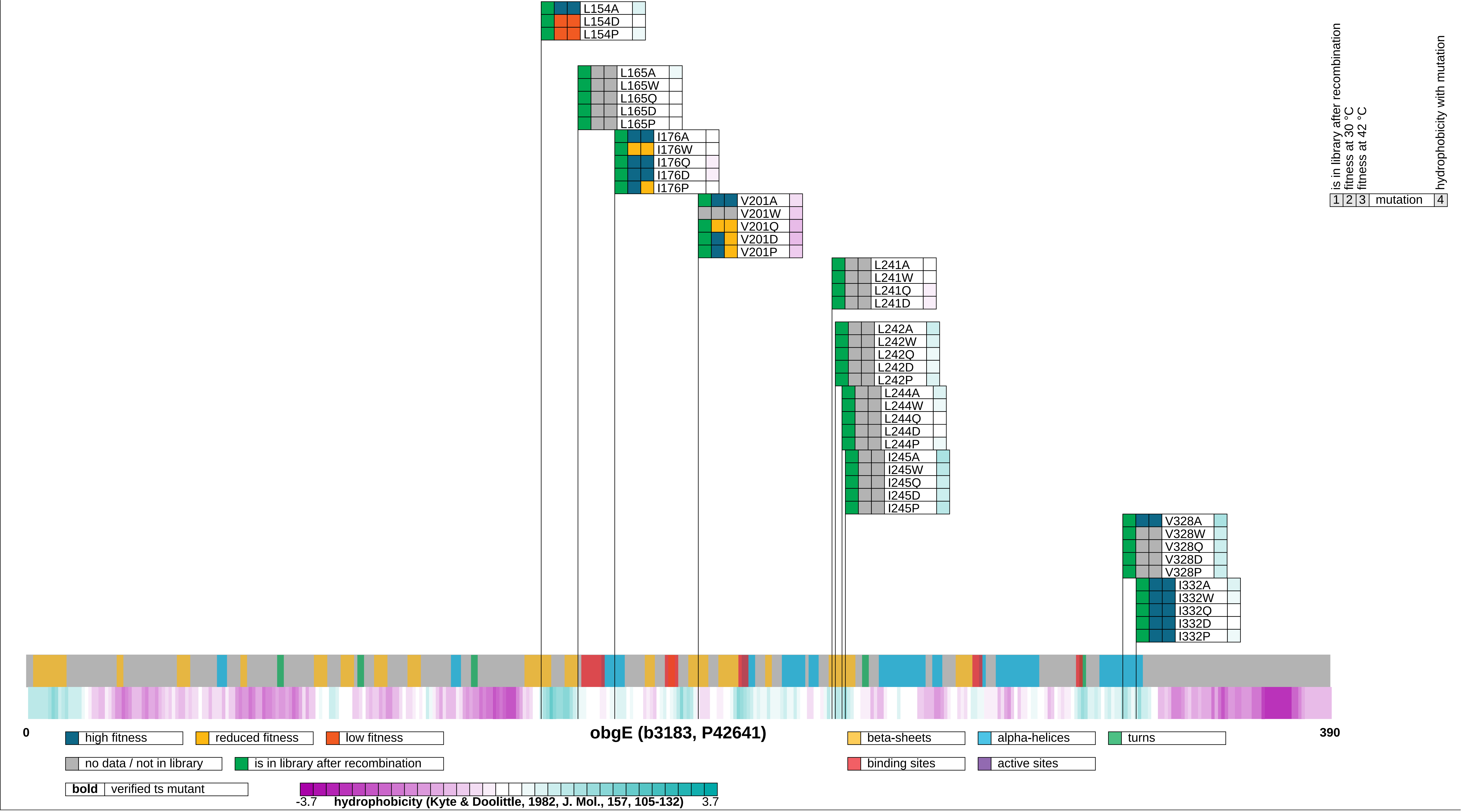

L154A
L154D
L154P
L165A
L165W
L165Q
L165D
L165P
I176A
I176W
I176Q
I176D
I176P
V201A
V201W
V201Q
V201D
V201P
L241A
L241W
L241Q
L241D
L242A
L242W
L242Q
L242D
L242P
L244A
L244W
L244Q
L244D
L244P
I245A
I245W
I245Q
I245D
I245P
V328A
V328W
V328Q
V328D
V328P
I332A
I332W
I332Q
I332D
I332P
is in library after recombination
fitness at 30 °C
fitness at 42 °C
hydrophobicity with mutation
1 2 3 mutation 4
0
obgE (b3183, P42641)
390
high fitness
reduced fitness
low fitness
no data / not in library
is in library after recombination
bold verified ts mutant
beta-sheets
alpha-helices
turns
binding sites
active sites
-3.7
hydrophobicity (Kyte & Doolittle, 1982, J. Mol., 157, 105-132)
3.7

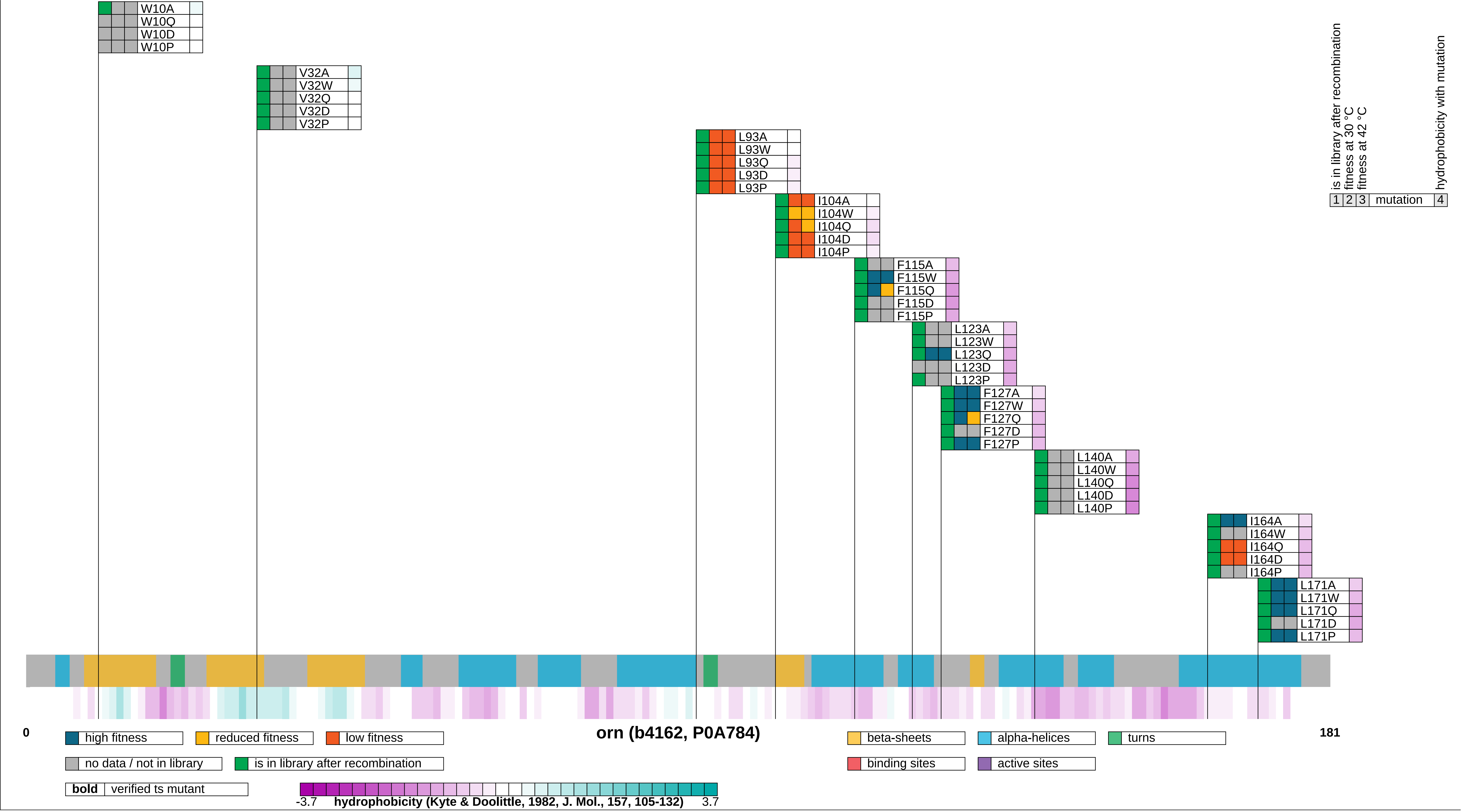

W10A
W10Q
W10D
W10P
V32A
V32W
V32Q
V32D
V32P
L93A
L93W
L93Q
L93D
L93P
I104A
I104W
I104Q
I104D
I104P
F115A
F115W
F115Q
F115D
F115P
L123A
L123W
L123Q
L123D
L123P
F127A
F127W
F127Q
F127D
F127P
L140A
L140W
L140Q
L140D
L140P
I164A
I164W
I164Q
I164D
I164P
L171A
L171W
L171Q
L171D
L171P
is in library after recombination
fitness at 30 °C
fitness at 42 °C
hydrophobicity with mutation
1 2 3 mutation 4
0
orn (b4162, P0A784)
181
high fitness
reduced fitness
low fitness
no data / not in library
is in library after recombination
bold verified ts mutant
beta-sheets
alpha-helices
turns
binding sites
active sites
-3.7 hydrophobicity (Kyte & Doolittle, 1982, J. Mol., 157, 105-132) 3.7

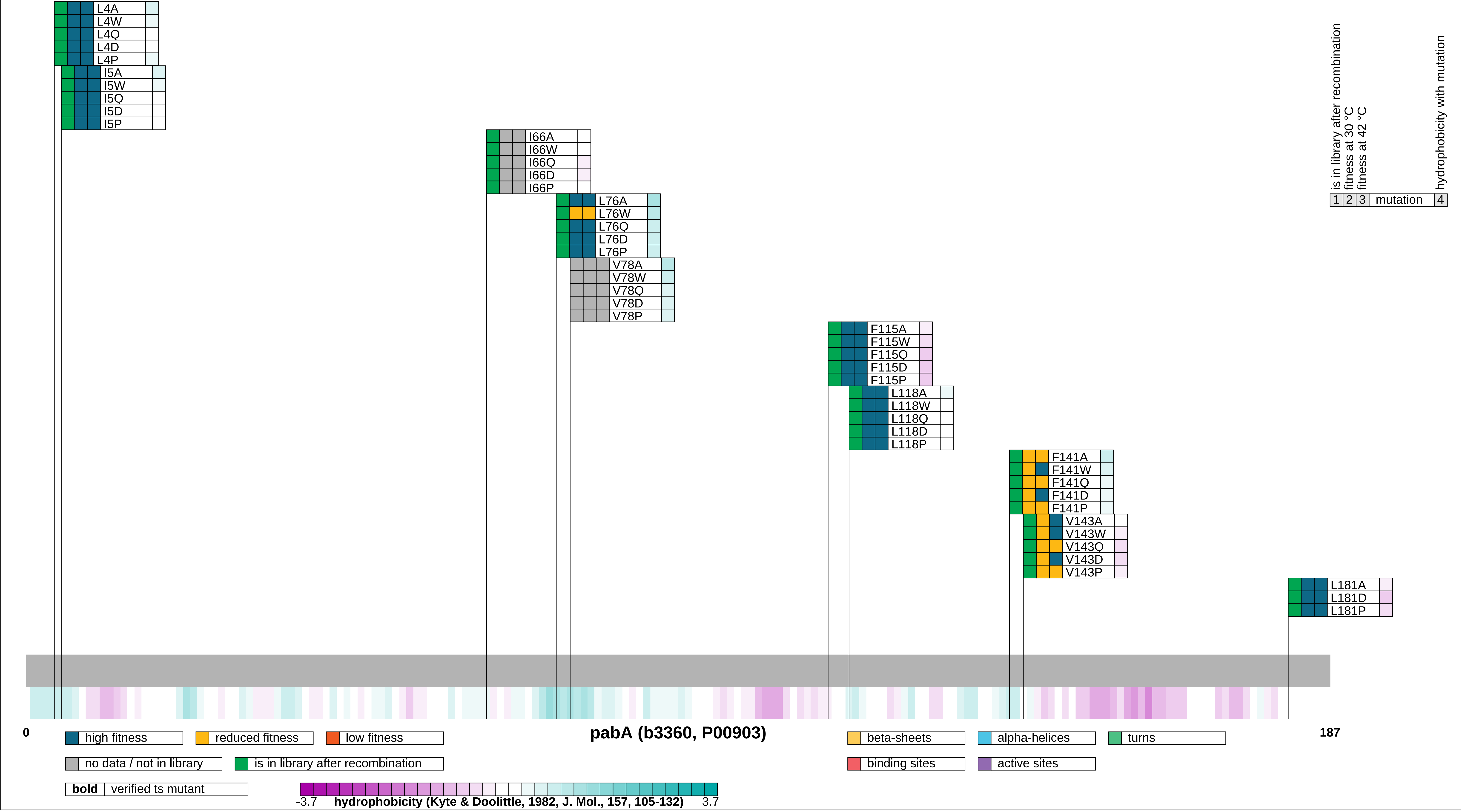

L4A
L4W
L4Q
L4D
L4P
I5A
I5W
I5Q
I5D
I5P
I66A
I66W
I66Q
I66D
I66P
L76A
L76W
L76Q
L76D
L76P
V78A
V78W
V78Q
V78D
V78P
F115A
F115W
F115Q
F115D
F115P
L118A
L118W
L118Q
L118D
L118P
F141A
F141W
F141Q
F141D
F141P
V143A
V143W
V143Q
V143D
V143P
L181A
L181D
L181P
is in library after recombination
fitness at 30 °C
fitness at 42 °C
hydrophobicity with mutation
1
2
3
mutation
4
0
pabA (b3360, P00903)
187
high fitness
reduced fitness
low fitness
beta-sheets
alpha-helices
turns
no data / not in library
is in library after recombination
binding sites
active sites
bold
verified ts mutant
-3.7
hydrophobicity (Kyte & Doolittle, 1982, J. Mol., 157, 105-132)
3.7

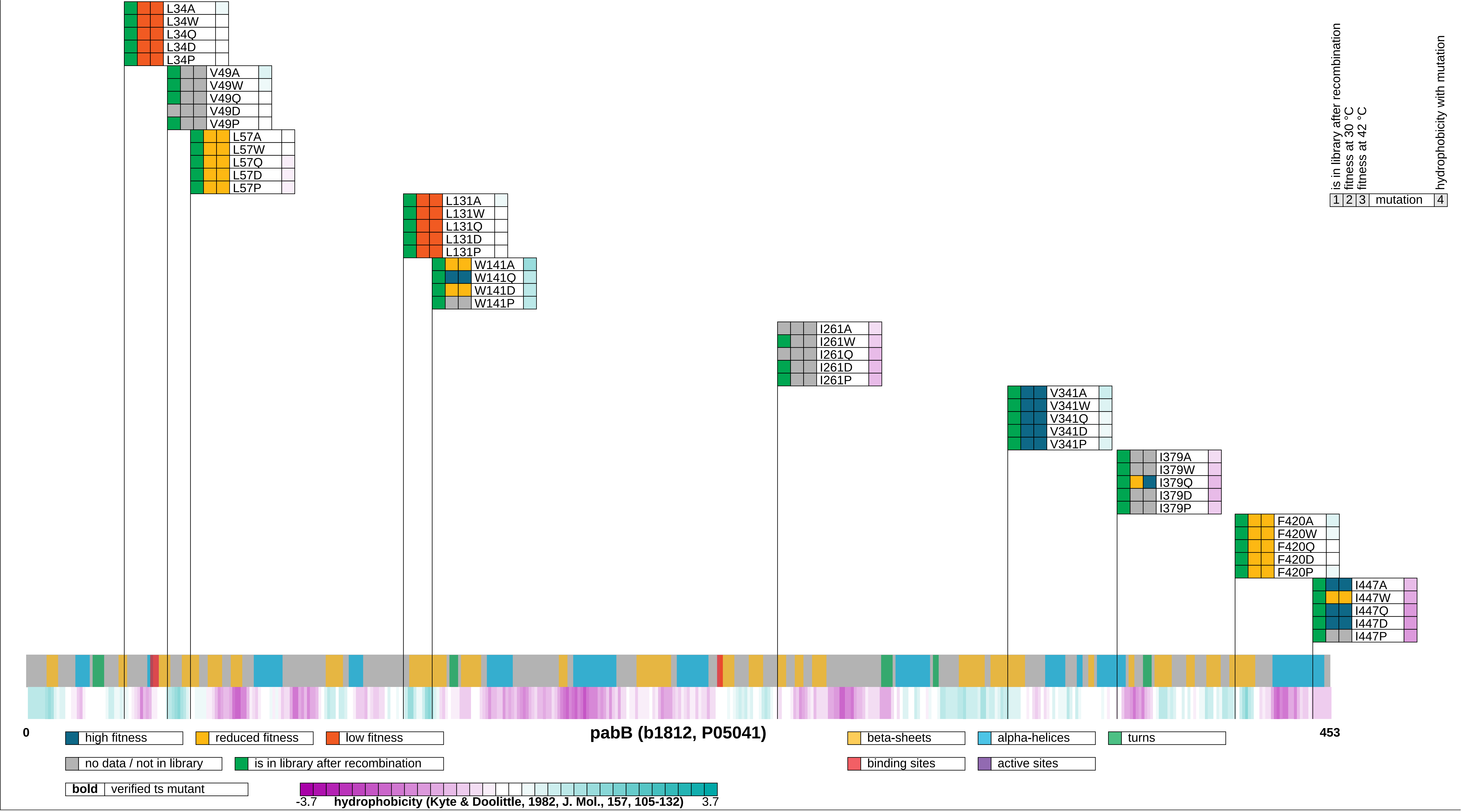
L34A
L34W
L34Q
L34D
L34P
V49A
V49W
V49Q
V49D
V49P
L57A
L57W
L57Q
L57D
L57P
L131A
L131W
L131Q
L131D
L131P
W141A
W141Q
W141D
W141P
I261A
I261W
I261Q
I261D
I261P
V341A
V341W
V341Q
V341D
V341P
I379A
I379W
I379Q
I379D
I379P
F420A
F420W
F420Q
F420D
F420P
I447A
I447W
I447Q
I447D
I447P
is in library after recombination
fitness at 30 °C
fitness at 42 °C
hydrophobicity with mutation
1 2 3 mutation 4
0
pabB (b1812, P05041)
453
high fitness
reduced fitness
low fitness
no data / not in library
is in library after recombination
bold verified ts mutant
beta-sheets
alpha-helices
turns
binding sites
active sites
-3.7
hydrophobicity (Kyte & Doolittle, 1982, J. Mol., 157, 105-132)
3.7

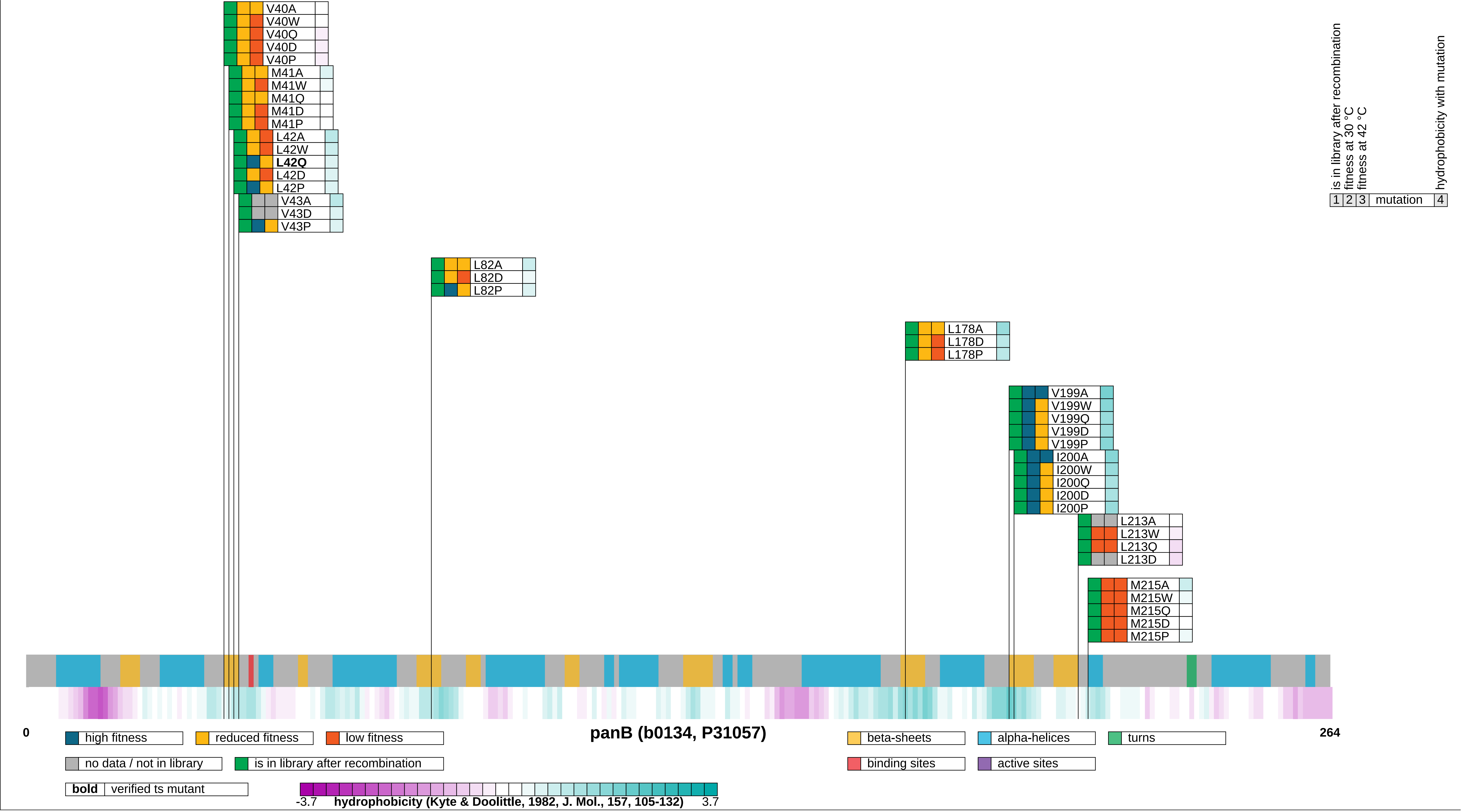

V40A
V40W
V40Q
V40D
V40P
M41A
M41W
M41Q
M41D
M41P
L42A
L42W
L42Q
L42D
L42P
V43A
V43D
V43P
L82A
L82D
L82P
L178A
L178D
L178P
V199A
V199W
V199Q
V199D
V199P
I200A
I200W
I200Q
I200D
I200P
L213A
L213W
L213Q
L213D
M215A
M215W
M215Q
M215D
M215P
is in library after recombination
fitness at 30 °C
fitness at 42 °C
hydrophobicity with mutation
1 2 3 mutation 4
0
264
panB (b0134, P31057)
high fitness
reduced fitness
low fitness
no data / not in library
is in library after recombination
bold verified ts mutant
beta-sheets
alpha-helices
turns
binding sites
active sites
-3.7 hydrophobicity (Kyte & Doolittle, 1982, J. Mol., 157, 105-132) 3.7

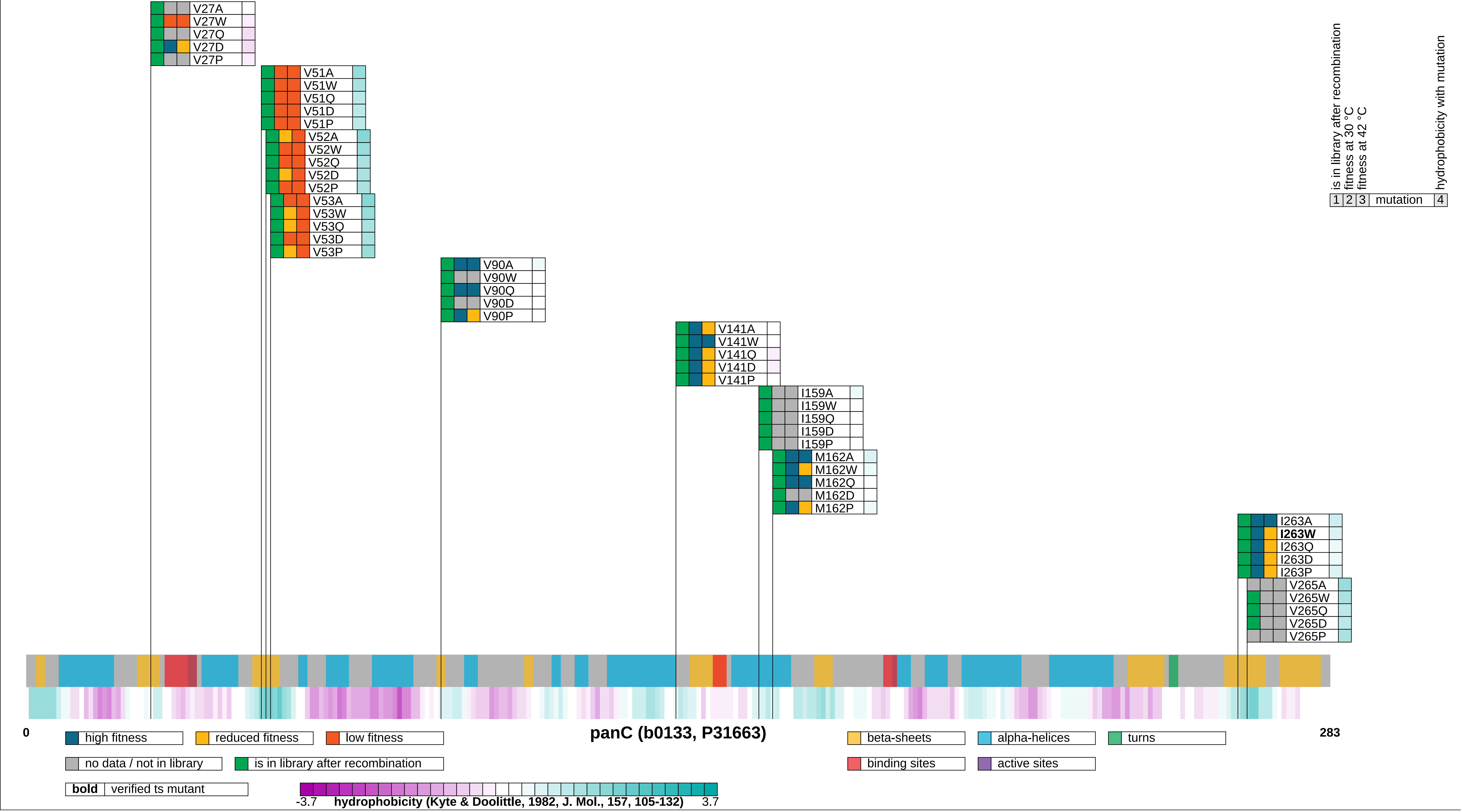

V27A
V27W
V27Q
V27D
V27P
V51A
V51W
V51Q
V51D
V51P
V52A
V52W
V52Q
V52D
V52P
V53A
V53W
V53Q
V53D
V53P
V90A
V90W
V90Q
V90D
V90P
V141A
V141W
V141Q
V141D
V141P
I159A
I159W
I159Q
I159D
I159P
M162A
M162W
M162Q
M162D
M162P
I263A
I263W
I263Q
I263D
I263P
V265A
V265W
V265Q
V265D
V265P
is in library after recombination
fitness at 30 °C
fitness at 42 °C
hydrophobicity with mutation
1 2 3 mutation 4
0
panC (b0133, P31663)
283
high fitness
reduced fitness
low fitness
no data / not in library
is in library after recombination
bold verified ts mutant
beta-sheets
alpha-helices
turns
binding sites
active sites
-3.7 hydrophobicity (Kyte & Doolittle, 1982, J. Mol., 157, 105-132) 3.7

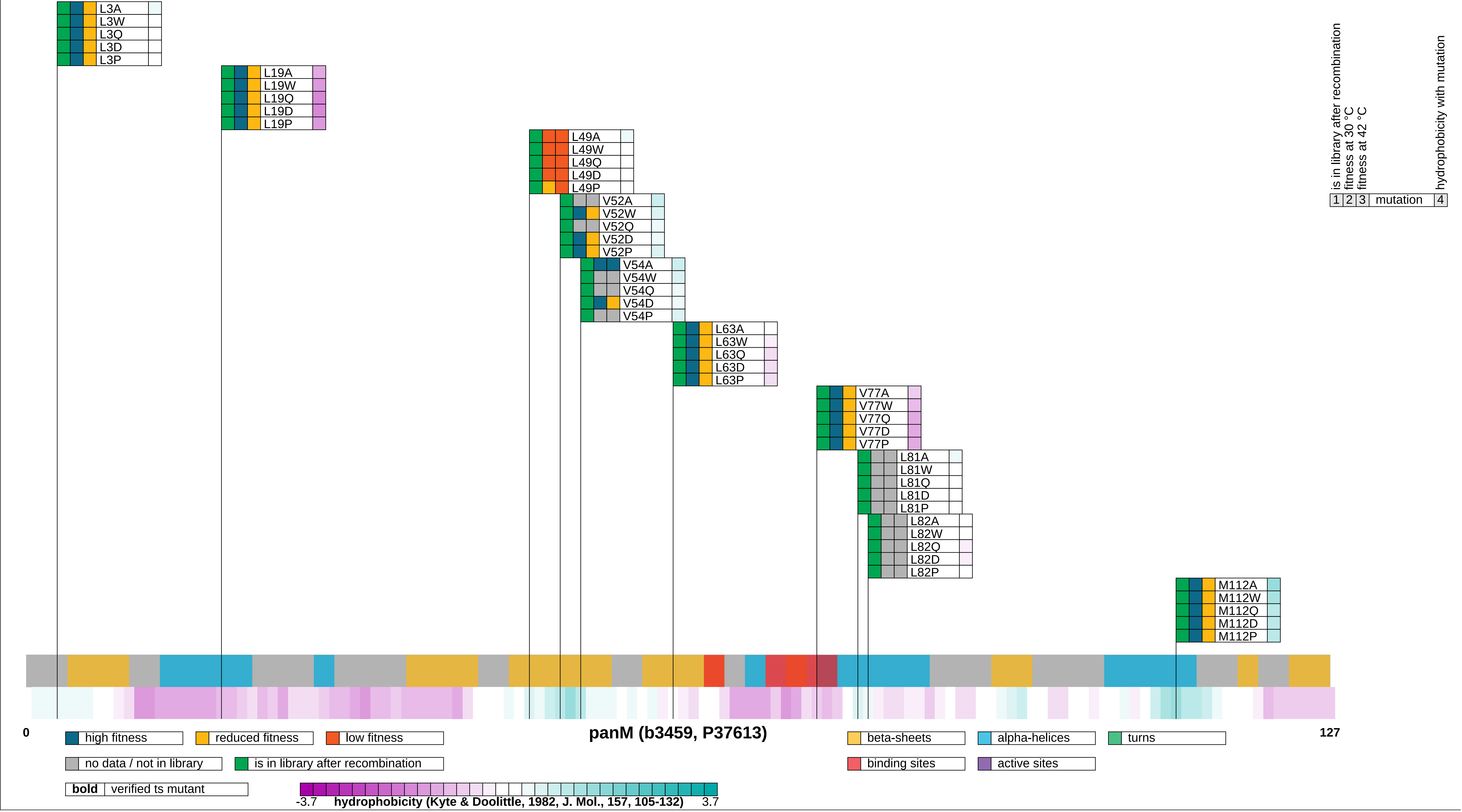

L3A
L3W
L3Q
L3D
L3P
L19A
L19W
L19Q
L19D
L19P
L49A
L49W
L49Q
L49D
L49P
V52A
V52W
V52Q
V52D
V52P
V54A
V54W
V54Q
V54D
V54P
L63A
L63W
L63Q
L63D
L63P
V77A
V77W
V77Q
V77D
V77P
L81A
L81W
L81Q
L81D
L81P
L82A
L82W
L82Q
L82D
L82P
M112A
M112W
M112Q
M112D
M112P
is in library after recombination
fitness at 30 °C
fitness at 42 °C
hydrophobicity with mutation
1 2 3 mutation 4
0
panM (b3459, P37613)
127
high fitness
reduced fitness
low fitness
no data / not in library
is in library after recombination
bold verified ts mutant
beta-sheets
alpha-helices
turns
binding sites
active sites
-3.7
hydrophobicity (Kyte & Doolittle, 1982, J. Mol., 157, 105-132)
3.7

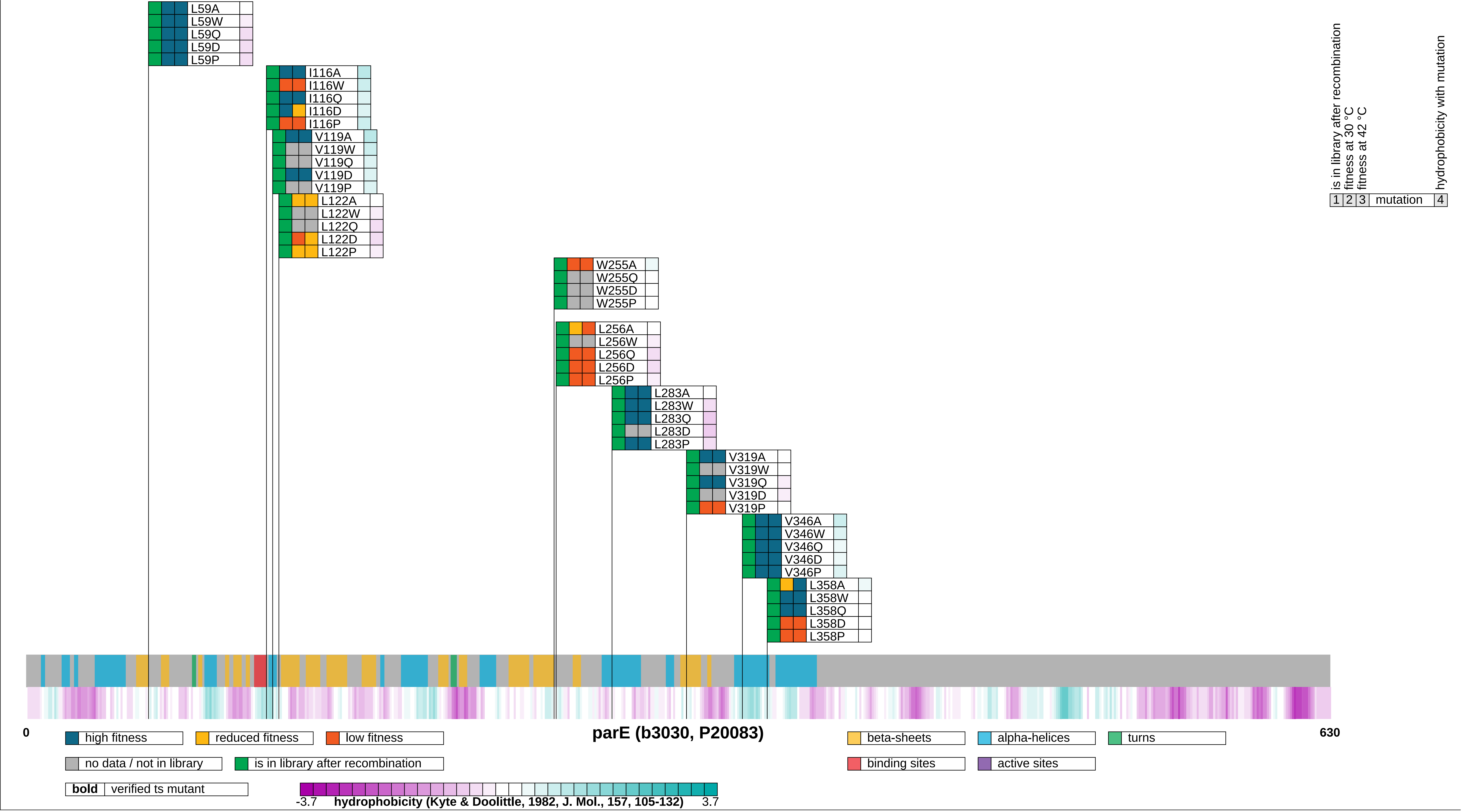
is in library after recombination
fitness at 30 °C
fitness at 42 °C
hydrophobicity with mutation
1 2 3 mutation 4
L59A
L59W
L59Q
L59D
L59P
I116A
I116W
I116Q
I116D
I116P
V119A
V119W
V119Q
V119D
V119P
L122A
L122W
L122Q
L122D
L122P
W255A
W255Q
W255D
W255P
L256A
L256W
L256Q
L256D
L256P
L283A
L283W
L283Q
L283D
L283P
V319A
V319W
V319Q
V319D
V319P
V346A
V346W
V346Q
V346D
V346P
L358A
L358W
L358Q
L358D
L358P
0
parE (b3030, P20083)
630
high fitness
reduced fitness
low fitness
beta-sheets
alpha-helices
turns
no data / not in library
is in library after recombination
binding sites
active sites
bold verified ts mutant
-3.7
hydrophobicity (Kyte & Doolittle, 1982, J. Mol., 157, 105-132)
3.7

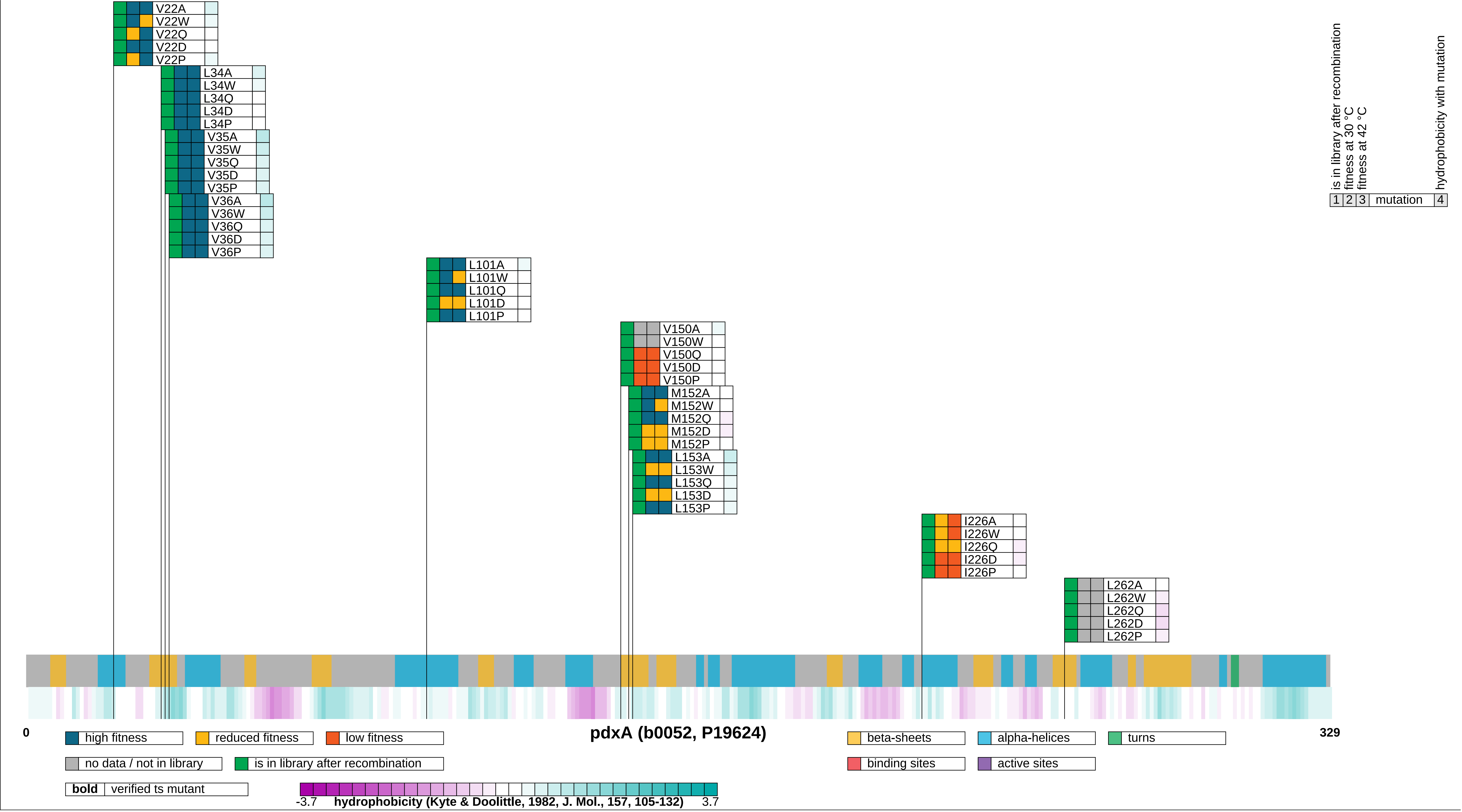

V22A
V22W
V22Q
V22D
V22P
L34A
L34W
L34Q
L34D
L34P
V35A
V35W
V35Q
V35D
V35P
V36A
V36W
V36Q
V36D
V36P
L101A
L101W
L101Q
L101D
L101P
V150A
V150W
V150Q
V150D
V150P
M152A
M152W
M152Q
M152D
M152P
L153A
L153W
L153Q
L153D
L153P
I226A
I226W
I226Q
I226D
I226P
L262A
L262W
L262Q
L262D
L262P
is in library after recombination
fitness at 30 °C
fitness at 42 °C
hydrophobicity with mutation
1 2 3 mutation 4
0
pdxA (b0052, P19624)
329
high fitness
reduced fitness
low fitness
no data / not in library
is in library after recombination
bold verified ts mutant
beta-sheets
alpha-helices
turns
binding sites
active sites
-3.7
hydrophobicity (Kyte & Doolittle, 1982, J. Mol., 157, 105-132)
3.7

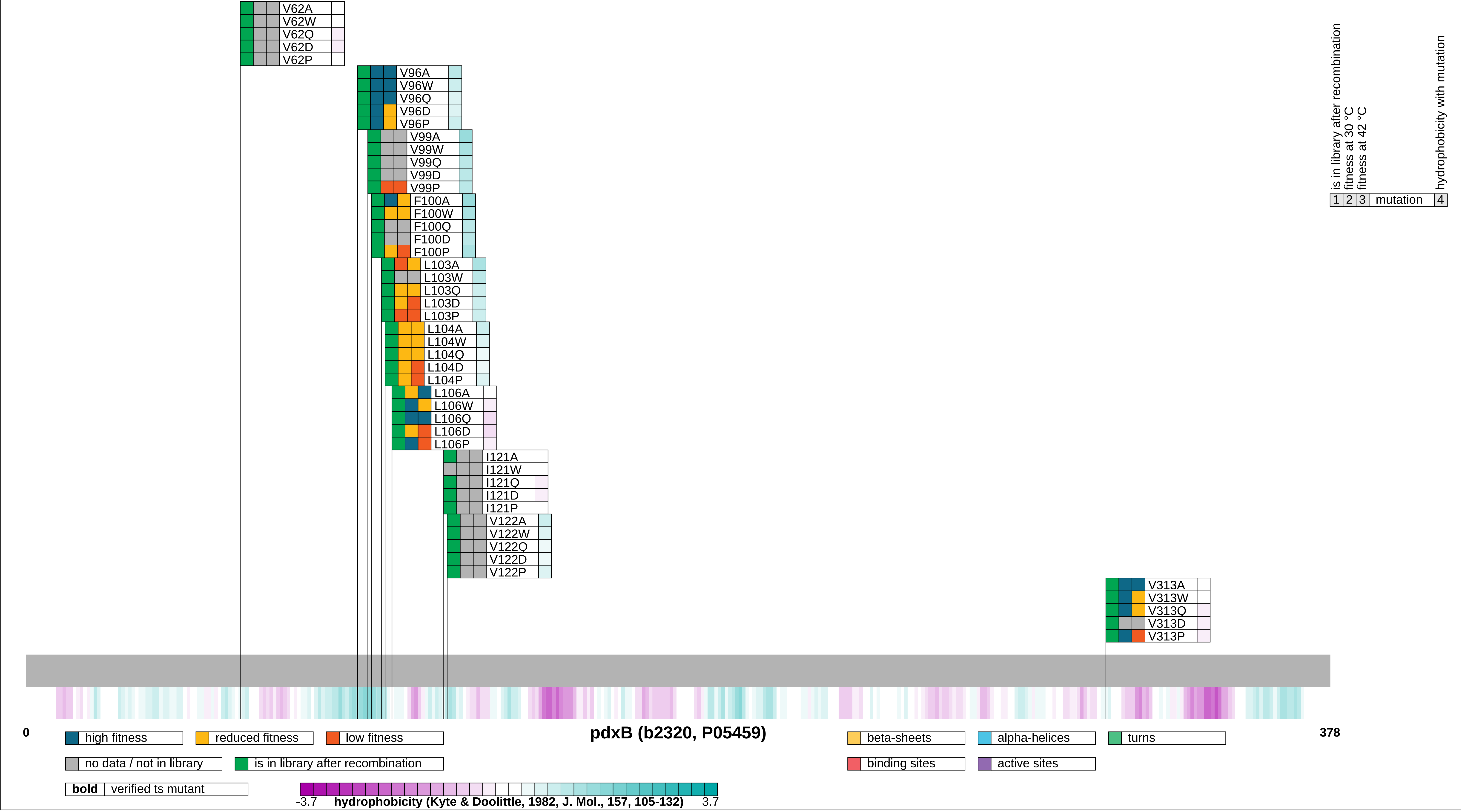

is in library after recombination
fitness at 30 °C
fitness at 42 °C
hydrophobicity with mutation
1
2
3
mutation
4
V62A
V62W
V62Q
V62D
V62P
V96A
V96W
V96Q
V96D
V96P
V99A
V99W
V99Q
V99D
V99P
F100A
F100W
F100Q
F100D
F100P
L103A
L103W
L103Q
L103D
L103P
L104A
L104W
L104Q
L104D
L104P
L106A
L106W
L106Q
L106D
L106P
I121A
I121W
I121Q
I121D
I121P
V122A
V122W
V122Q
V122D
V122P
V313A
V313W
V313Q
V313D
V313P
0
pdxB (b2320, P05459)
378
high fitness
reduced fitness
low fitness
beta-sheets
alpha-helices
turns
no data / not in library
is in library after recombination
binding sites
active sites
bold
verified ts mutant
-3.7
hydrophobicity (Kyte & Doolittle, 1982, J. Mol., 157, 105-132)
3.7

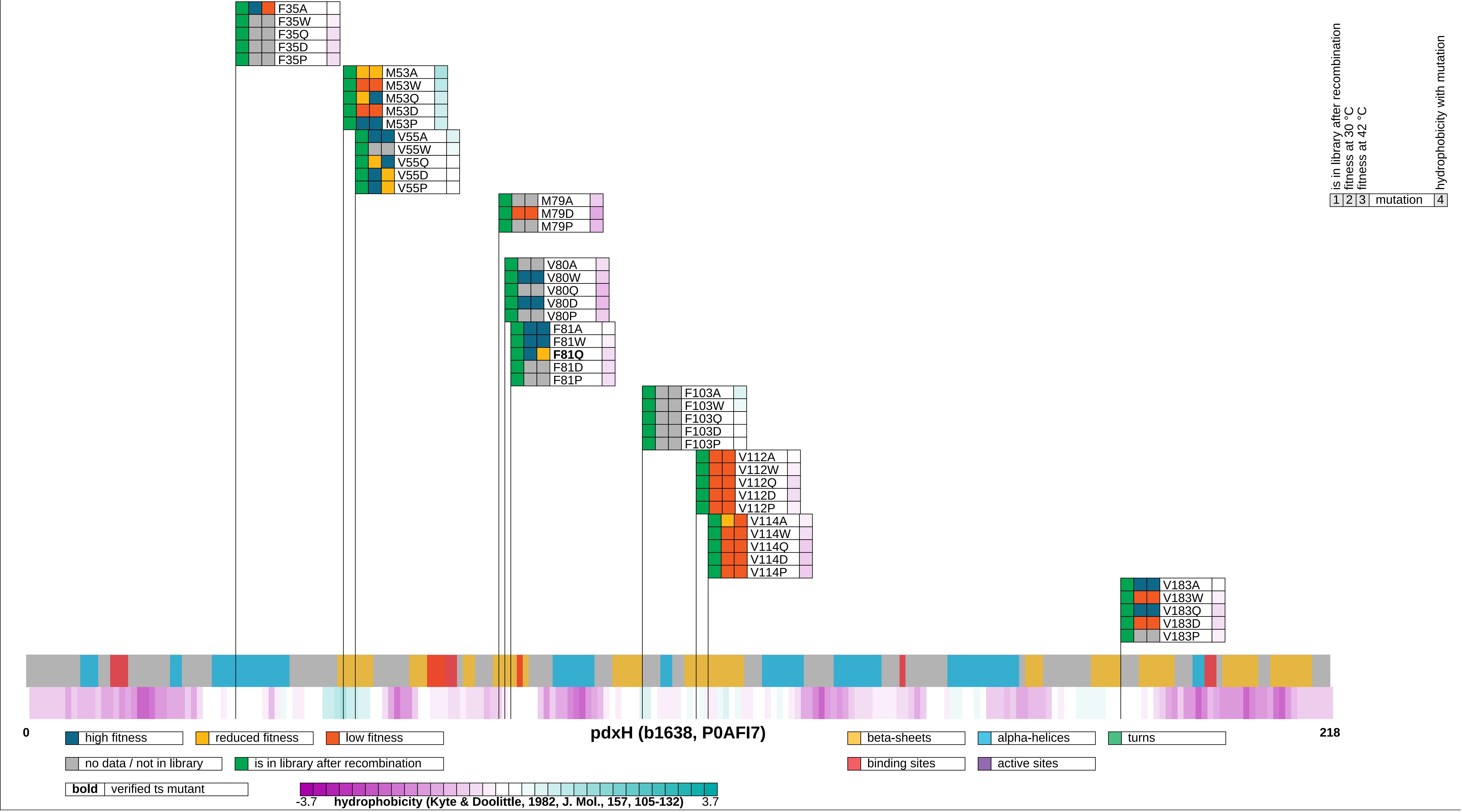

is in library after recombination
fitness at 30 °C
fitness at 42 °C
hydrophobicity with mutation
1 2 3 mutation 4
F35A
F35W
F35Q
F35D
F35P
M53A
M53W
M53Q
M53D
M53P
V55A
V55W
V55Q
V55D
V55P
M79A
M79D
M79P
V80A
V80W
V80Q
V80D
V80P
F81A
F81W
F81Q
F81D
F81P
F103A
F103W
F103Q
F103D
F103P
V112A
V112W
V112Q
V112D
V112P
V114A
V114W
V114Q
V114D
V114P
V183A
V183W
V183Q
V183D
V183P
0
pdxH (b1638, P0AFI7)
218
high fitness
reduced fitness
low fitness
beta-sheets
alpha-helices
turns
no data / not in library
is in library after recombination
binding sites
active sites
bold verified ts mutant
-3.7
hydrophobicity (Kyte & Doolittle, 1982, J. Mol., 157, 105-132)
3.7

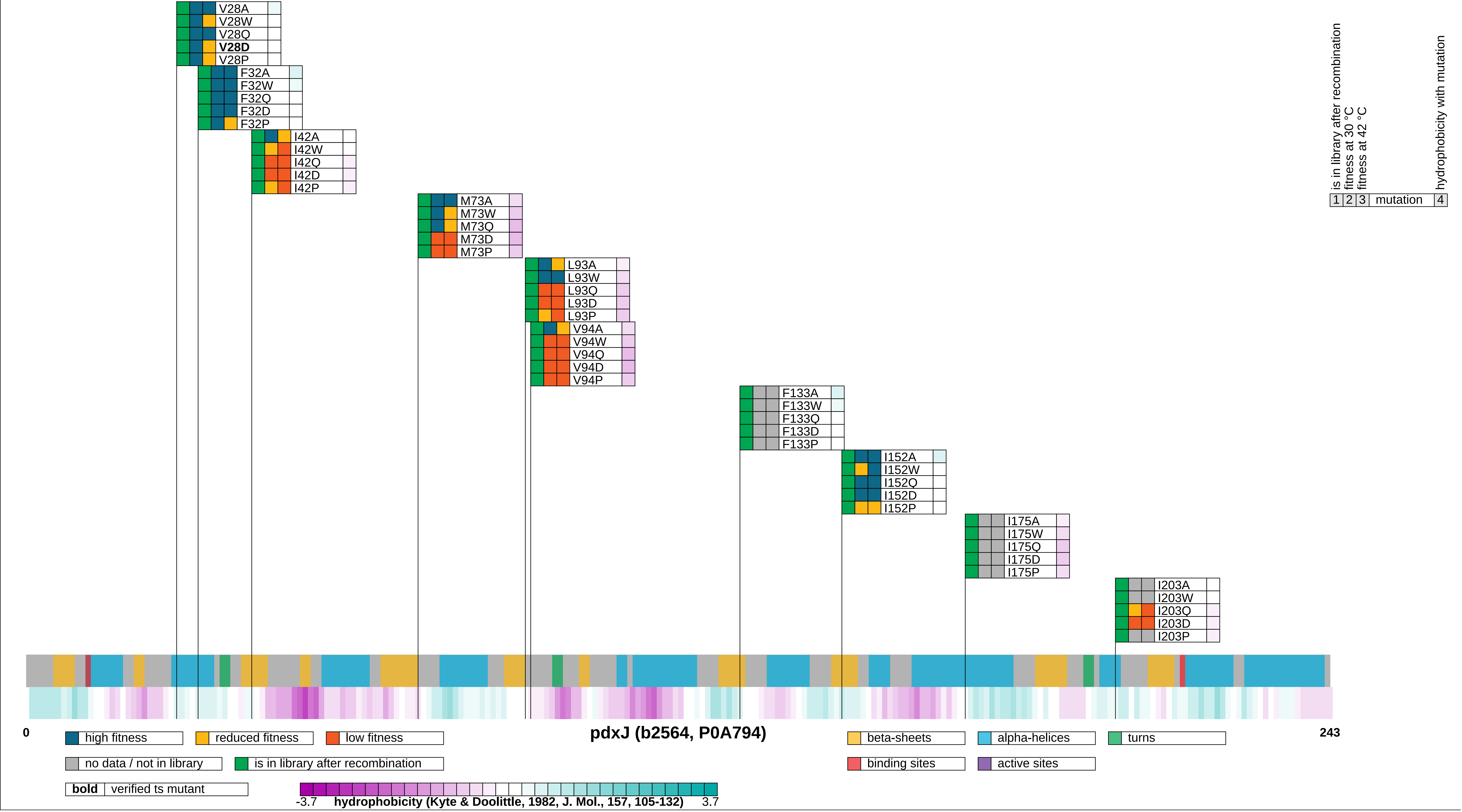

V28A
V28W
V28Q
V28D
V28P
F32A
F32W
F32Q
F32D
F32P
I42A
I42W
I42Q
I42D
I42P
M73A
M73W
M73Q
M73D
M73P
L93A
L93W
L93Q
L93D
L93P
V94A
V94W
V94Q
V94D
V94P
F133A
F133W
F133Q
F133D
F133P
I152A
I152W
I152Q
I152D
I152P
I175A
I175W
I175Q
I175D
I175P
I203A
I203W
I203Q
I203D
I203P
is in library after recombination
fitness at 30 °C
fitness at 42 °C
hydrophobicity with mutation
1 2 3 mutation 4
0
243
pdxJ (b2564, P0A794)
high fitness
reduced fitness
low fitness
beta-sheets
alpha-helices
turns
no data / not in library
is in library after recombination
binding sites
active sites
bold verified ts mutant
-3.7
hydrophobicity (Kyte & Doolittle, 1982, J. Mol., 157, 105-132)
3.7

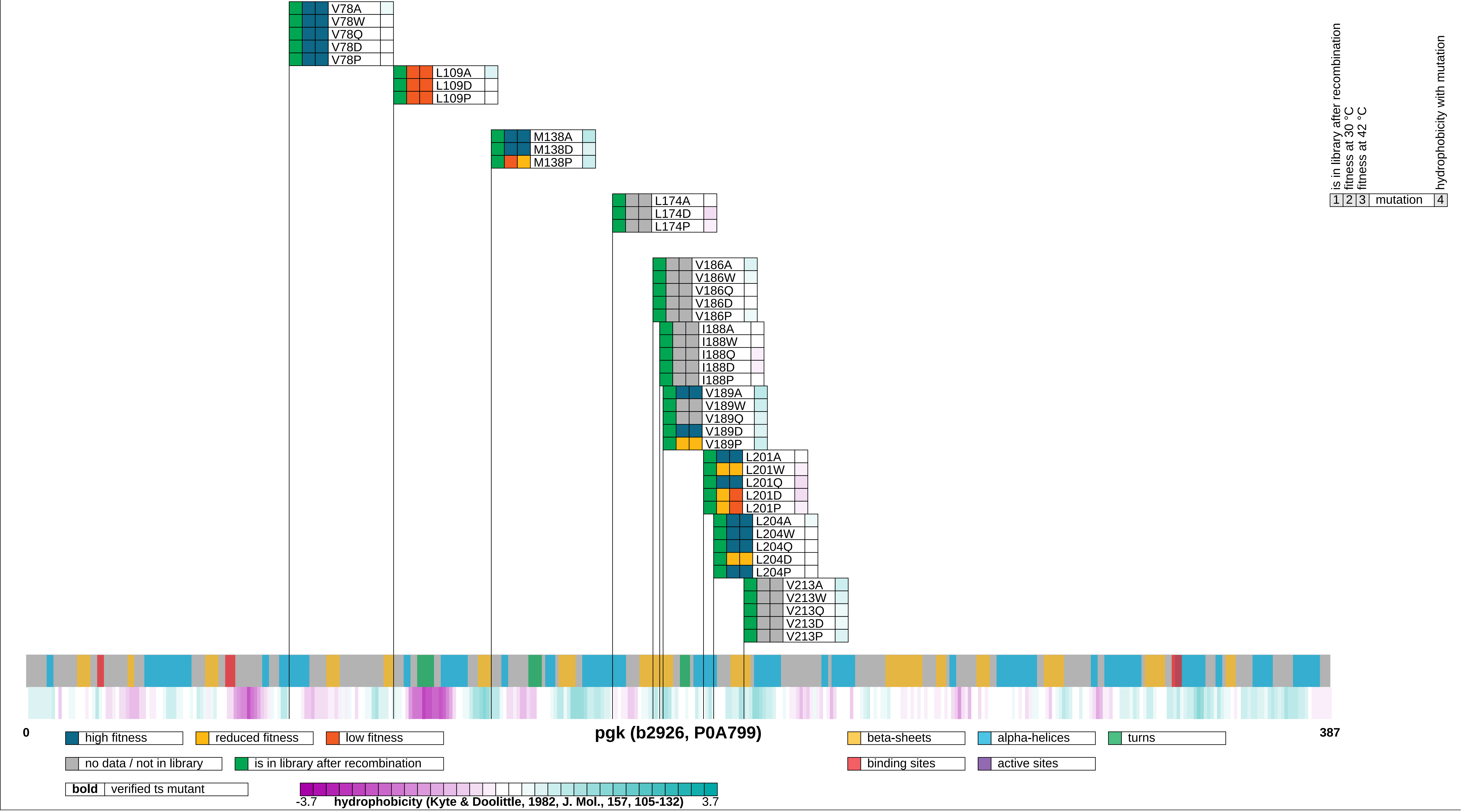
V78A
V78W
V78Q
V78D
V78P
L109A
L109D
L109P
M138A
M138D
M138P
L174A
L174D
L174P
V186A
V186W
V186Q
V186D
V186P
I188A
I188W
I188Q
I188D
I188P
V189A
V189W
V189Q
V189D
V189P
L201A
L201W
L201Q
L201D
L201P
L204A
L204W
L204Q
L204D
L204P
V213A
V213W
V213Q
V213D
V213P
is in library after recombination
fitness at 30 °C
fitness at 42 °C
hydrophobicity with mutation
1 2 3 mutation 4
0
pgk (b2926, P0A799)
387
high fitness
reduced fitness
low fitness
beta-sheets
alpha-helices
turns
no data / not in library
is in library after recombination
binding sites
active sites
bold verified ts mutant
-3.7 hydrophobicity (Kyte & Doolittle, 1982, J. Mol., 157, 105-132) 3.7

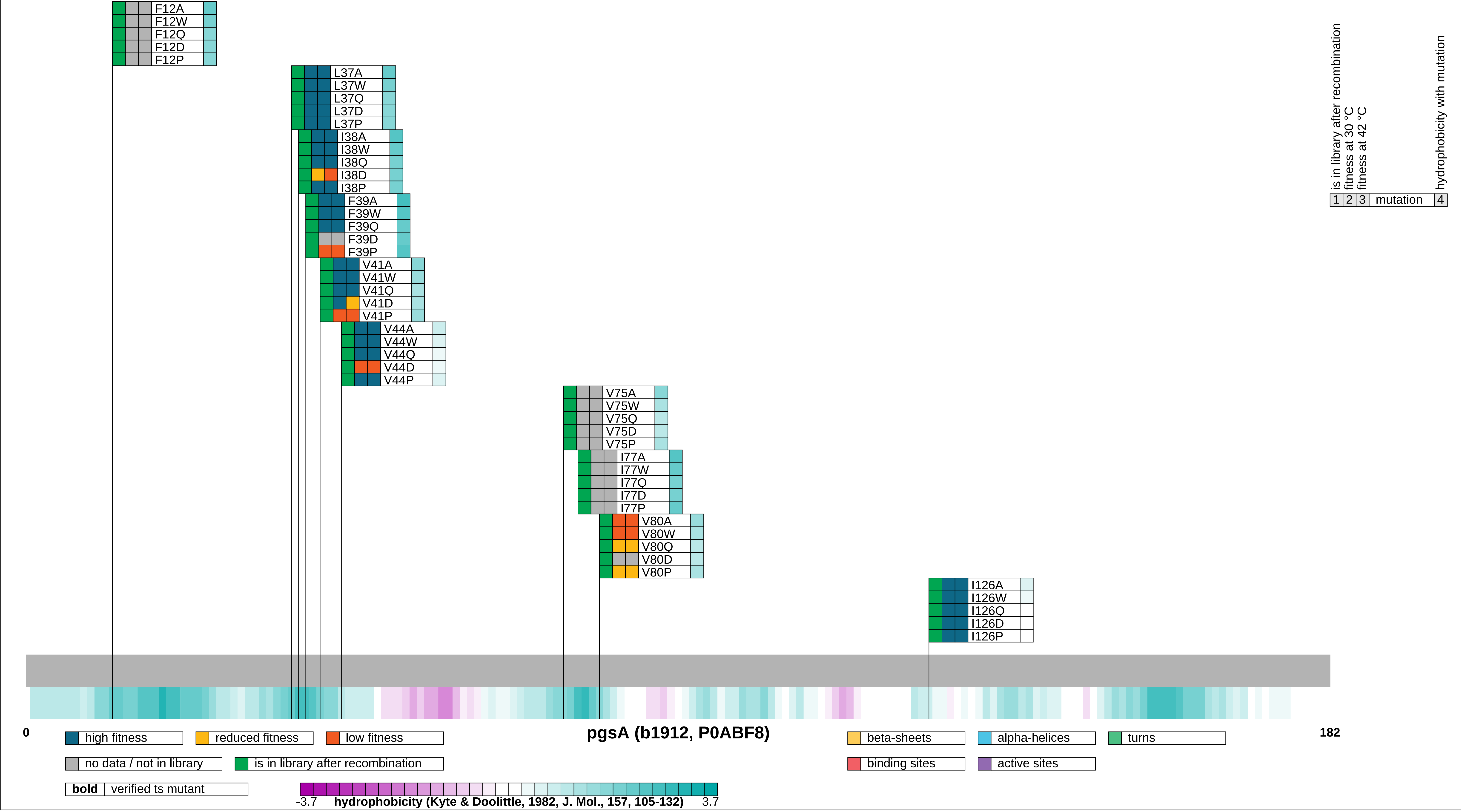

is in library after recombination
fitness at 30 °C
fitness at 42 °C
hydrophobicity with mutation
1 2 3 mutation 4
F12A
F12W
F12Q
F12D
F12P
L37A
L37W
L37Q
L37D
L37P
I38A
I38W
I38Q
I38D
I38P
F39A
F39W
F39Q
F39D
F39P
V41A
V41W
V41Q
V41D
V41P
V44A
V44W
V44Q
V44D
V44P
V75A
V75W
V75Q
V75D
V75P
I77A
I77W
I77Q
I77D
I77P
V80A
V80W
V80Q
V80D
V80P
I126A
I126W
I126Q
I126D
I126P
0
pgsA (b1912, P0ABF8)
182
high fitness
reduced fitness
low fitness
beta-sheets
alpha-helices
turns
no data / not in library
is in library after recombination
binding sites
active sites
bold verified ts mutant
-3.7
hydrophobicity (Kyte & Doolittle, 1982, J. Mol., 157, 105-132)
3.7

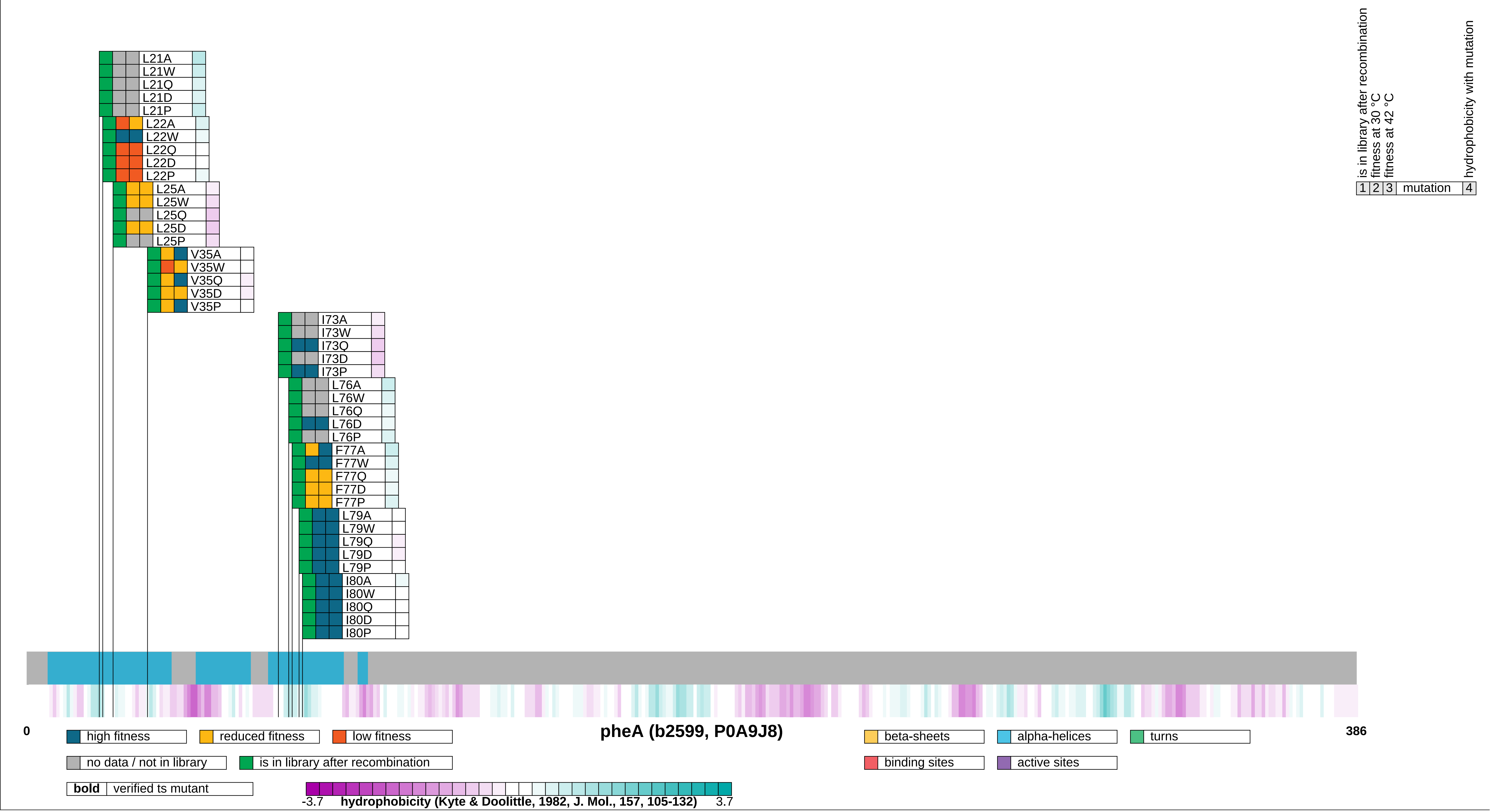
is in library after recombination
fitness at 30 °C
fitness at 42 °C
hydrophobicity with mutation
1 2 3 mutation 4
L21A
L21W
L21Q
L21D
L21P
L22A
L22W
L22Q
L22D
L22P
L25A
L25W
L25Q
L25D
L25P
V35A
V35W
V35Q
V35D
V35P
I73A
I73W
I73Q
I73D
I73P
L76A
L76W
L76Q
L76D
L76P
F77A
F77W
F77Q
F77D
F77P
L79A
L79W
L79Q
L79D
L79P
I80A
I80W
I80Q
I80D
I80P
0
pheA (b2599, P0A9J8)
386
high fitness
reduced fitness
low fitness
beta-sheets
alpha-helices
turns
no data / not in library
is in library after recombination
binding sites
active sites
bold verified ts mutant
-3.7
hydrophobicity (Kyte & Doolittle, 1982, J. Mol., 157, 105-132)
3.7

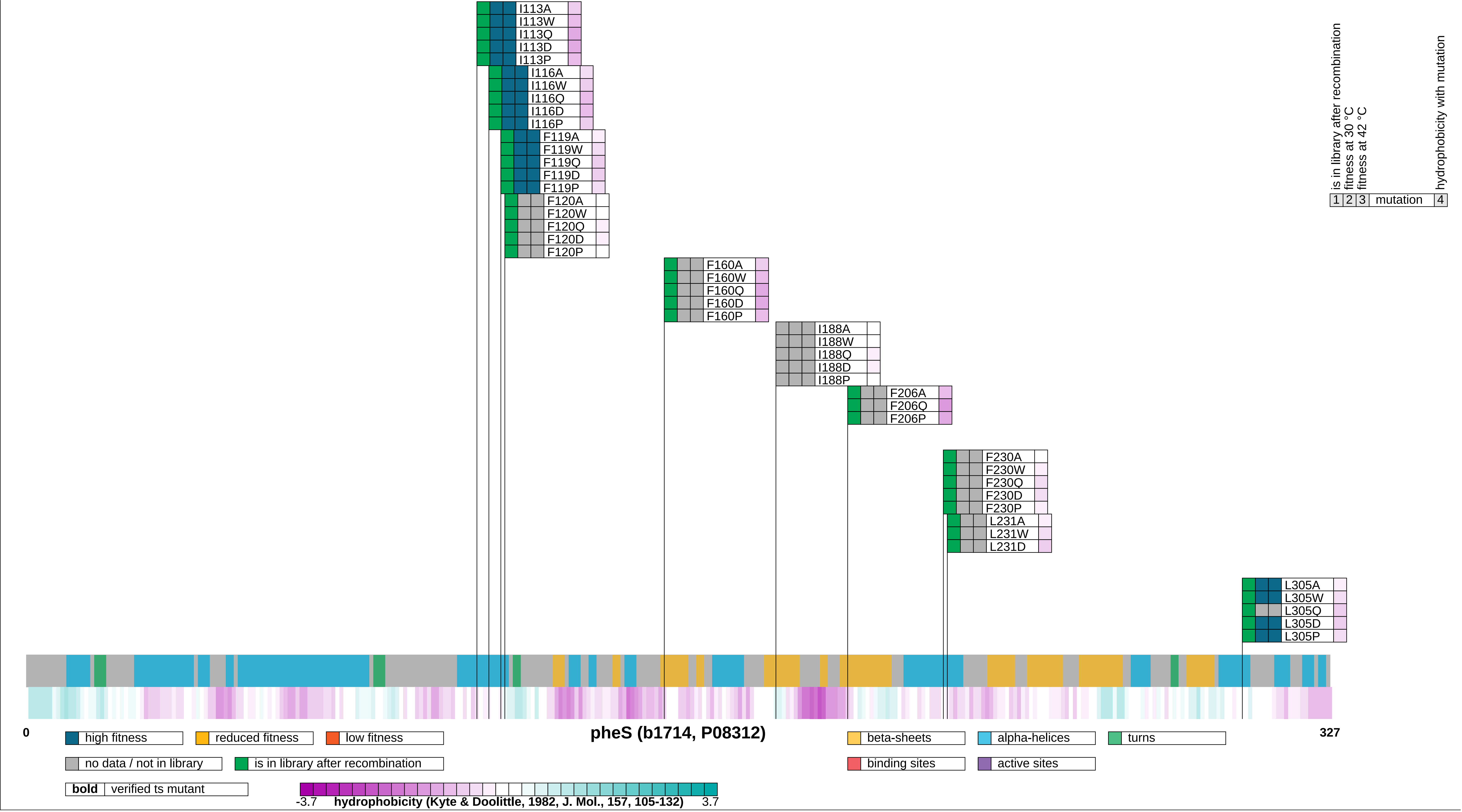
is in library after recombination
fitness at 30 °C
fitness at 42 °C
hydrophobicity with mutation
1 2 3 mutation 4
I113A
I113W
I113Q
I113D
I113P
I116A
I116W
I116Q
I116D
I116P
F119A
F119W
F119Q
F119D
F119P
F120A
F120W
F120Q
F120D
F120P
F160A
F160W
F160Q
F160D
F160P
I188A
I188W
I188Q
I188D
I188P
F206A
F206Q
F206P
F230A
F230W
F230Q
F230D
F230P
L231A
L231W
L231D
L305A
L305W
L305Q
L305D
L305P
0
pheS (b1714, P08312)
327
high fitness
reduced fitness
low fitness
beta-sheets
alpha-helices
turns
no data / not in library
is in library after recombination
binding sites
active sites
bold verified ts mutant
-3.7 hydrophobicity (Kyte & Doolittle, 1982, J. Mol., 157, 105-132) 3.7

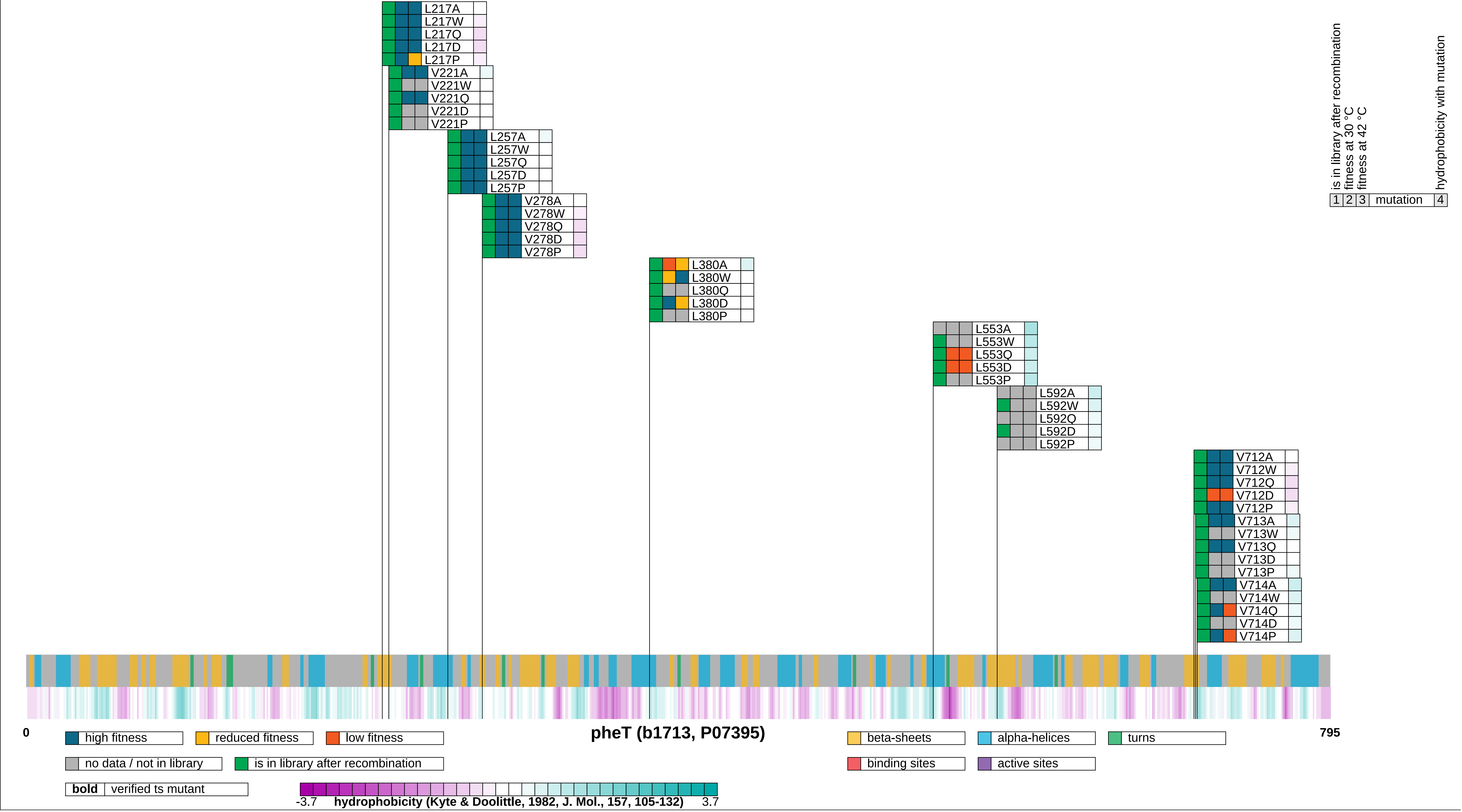

L217A
L217W
L217Q
L217D
L217P
V221A
V221W
V221Q
V221D
V221P
L257A
L257W
L257Q
L257D
L257P
V278A
V278W
V278Q
V278D
V278P
L380A
L380W
L380Q
L380D
L380P
L553A
L553W
L553Q
L553D
L553P
L592A
L592W
L592Q
L592D
L592P
V712A
V712W
V712Q
V712D
V712P
V713A
V713W
V713Q
V713D
V713P
V714A
V714W
V714Q
V714D
V714P
is in library after recombination
fitness at 30 °C
fitness at 42 °C
hydrophobicity with mutation
1 2 3 mutation 4
0
pheT (b1713, P07395)
795
high fitness
reduced fitness
low fitness
no data / not in library
is in library after recombination
bold verified ts mutant
beta-sheets
alpha-helices
turns
binding sites
active sites
-3.7
hydrophobicity (Kyte & Doolittle, 1982, J. Mol., 157, 105-132)
3.7

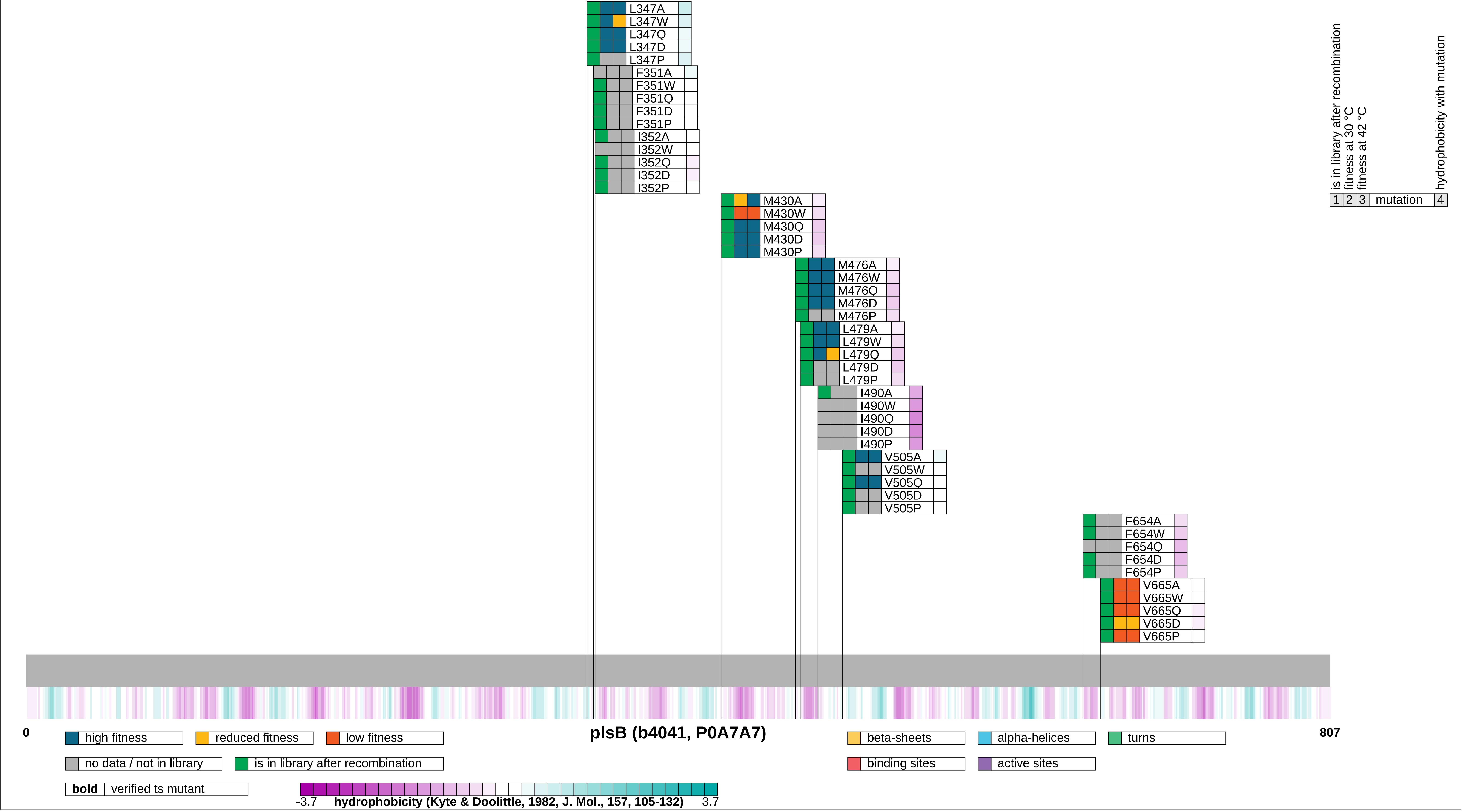
L347A
L347W
L347Q
L347D
L347P
F351A
F351W
F351Q
F351D
F351P
I352A
I352W
I352Q
I352D
I352P
M430A
M430W
M430Q
M430D
M430P
M476A
M476W
M476Q
M476D
M476P
L479A
L479W
L479Q
L479D
L479P
I490A
I490W
I490Q
I490D
I490P
V505A
V505W
V505Q
V505D
V505P
F654A
F654W
F654Q
F654D
F654P
V665A
V665W
V665Q
V665D
V665P
is in library after recombination
fitness at 30 °C
fitness at 42 °C
hydrophobicity with mutation
1 2 3 mutation 4
0
807
plsB (b4041, P0A7A7)
high fitness
reduced fitness
low fitness
no data / not in library
is in library after recombination
bold verified ts mutant
beta-sheets
alpha-helices
turns
binding sites
active sites
-3.7 hydrophobicity (Kyte & Doolittle, 1982, J. Mol., 157, 105-132) 3.7

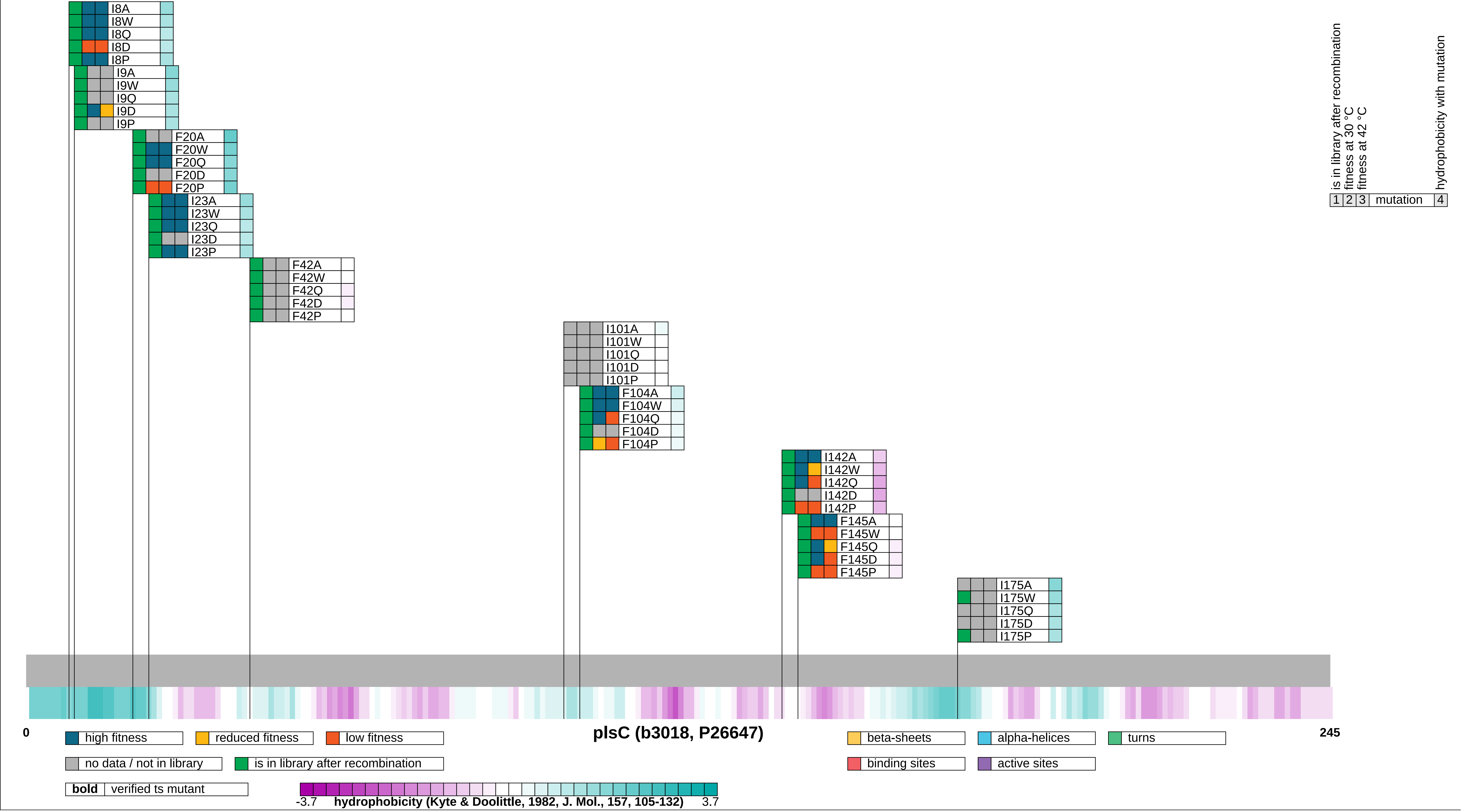
I8A
I8W
I8Q
I8D
I8P
I9A
I9W
I9Q
I9D
I9P
F20A
F20W
F20Q
F20D
F20P
I23A
I23W
I23Q
I23D
I23P
F42A
F42W
F42Q
F42D
F42P
I101A
I101W
I101Q
I101D
I101P
F104A
F104W
F104Q
F104D
F104P
I142A
I142W
I142Q
I142D
I142P
F145A
F145W
F145Q
F145D
F145P
I175A
I175W
I175Q
I175D
I175P
is in library after recombination
fitness at 30 °C
fitness at 42 °C
hydrophobicity with mutation
1 2 3 mutation 4
0
245
plsC (b3018, P26647)
high fitness
reduced fitness
low fitness
no data / not in library
is in library after recombination
bold verified ts mutant
beta-sheets
alpha-helices
turns
binding sites
active sites
-3.7 hydrophobicity (Kyte & Doolittle, 1982, J. Mol., 157, 105-132) 3.7

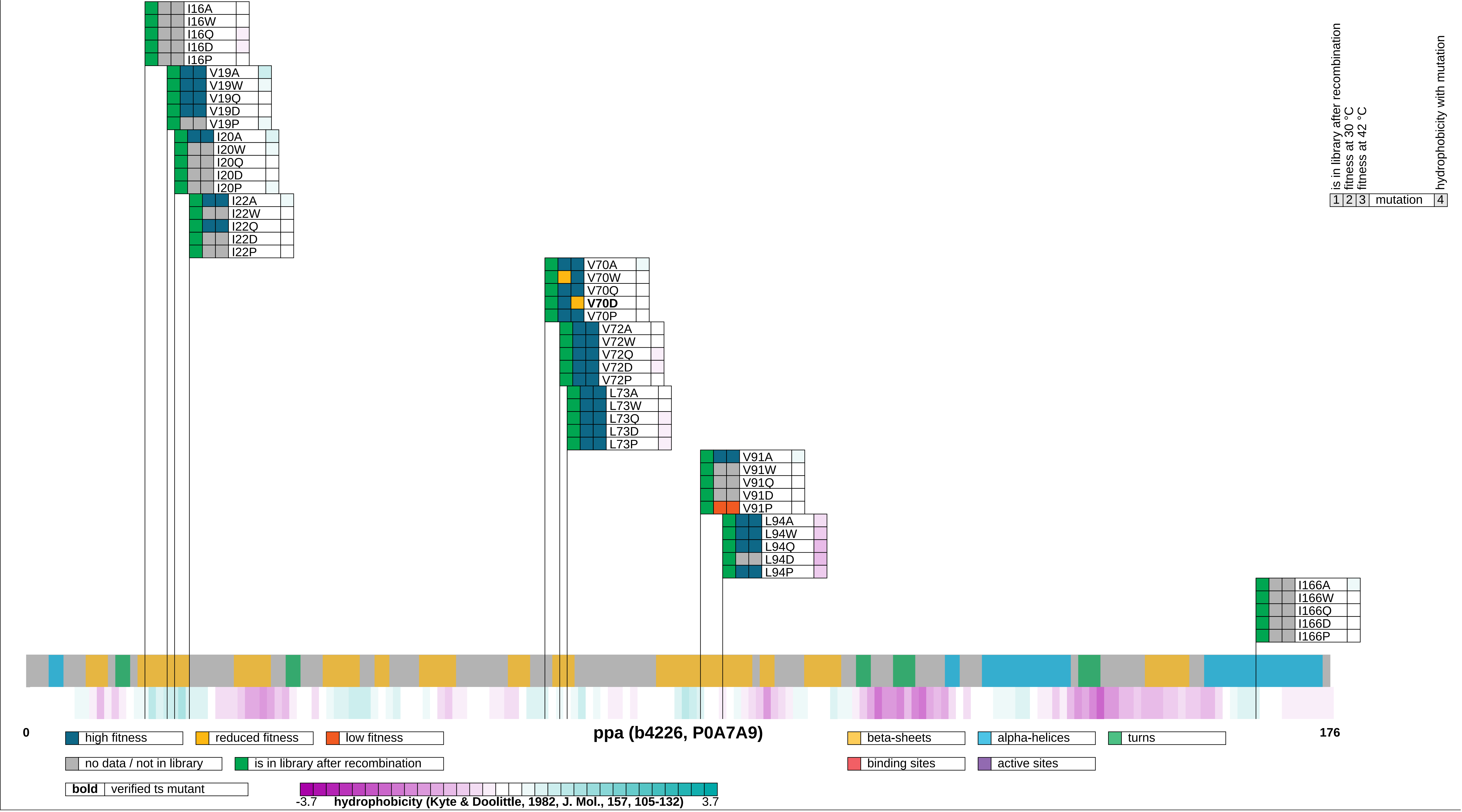
I16A
I16W
I16Q
I16D
I16P
V19A
V19W
V19Q
V19D
V19P
I20A
I20W
I20Q
I20D
I20P
I22A
I22W
I22Q
I22D
I22P
V70A
V70W
V70Q
V70D
V70P
V72A
V72W
V72Q
V72D
V72P
L73A
L73W
L73Q
L73D
L73P
V91A
V91W
V91Q
V91D
V91P
L94A
L94W
L94Q
L94D
L94P
I166A
I166W
I166Q
I166D
I166P
is in library after recombination
fitness at 30 °C
fitness at 42 °C
hydrophobicity with mutation
1 2 3 mutation 4
0
ppa (b4226, P0A7A9)
176
high fitness
reduced fitness
low fitness
beta-sheets
alpha-helices
turns
no data / not in library
is in library after recombination
binding sites
active sites
bold verified ts mutant
-3.7
hydrophobicity (Kyte & Doolittle, 1982, J. Mol., 157, 105-132)
3.7

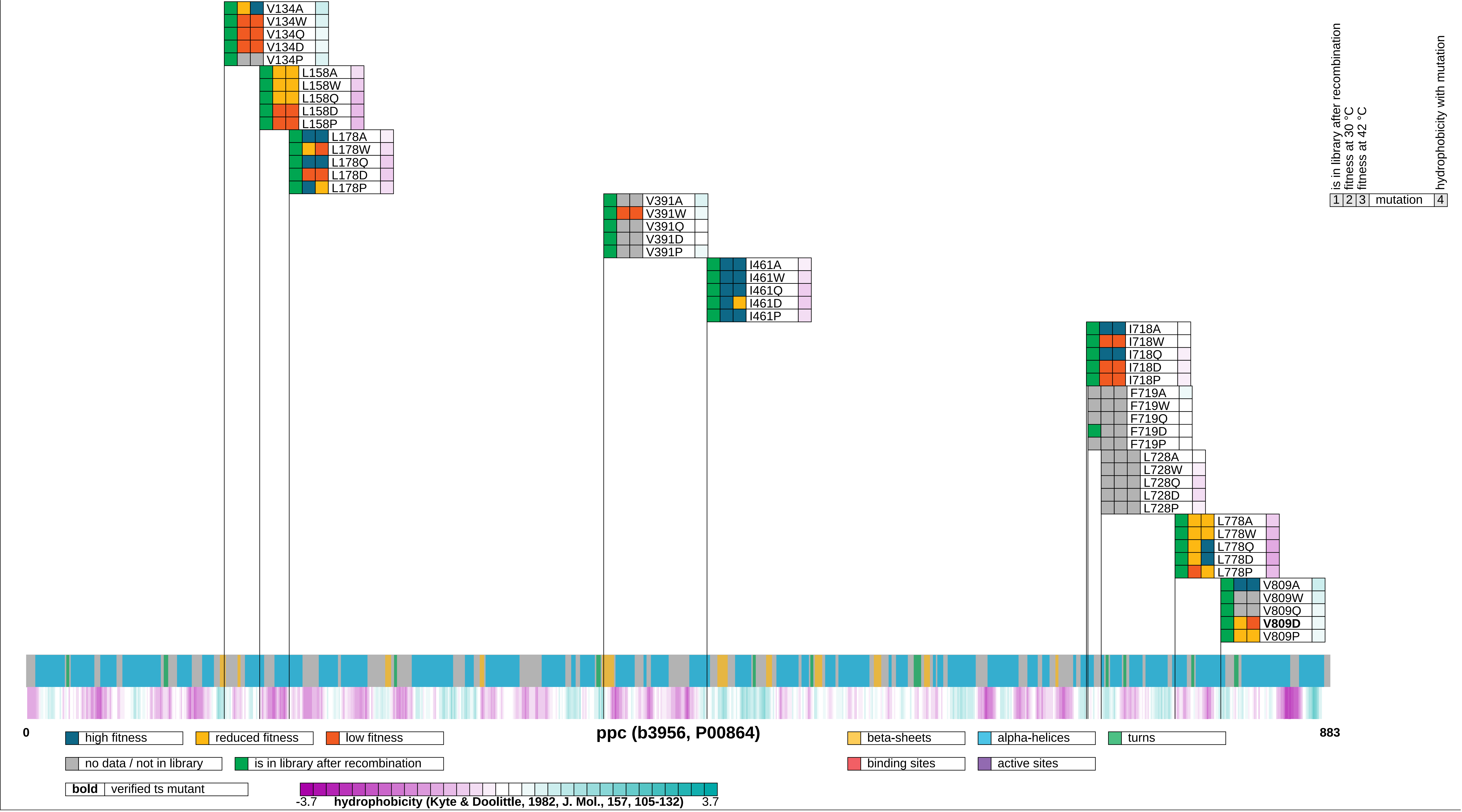

V134A
V134W
V134Q
V134D
V134P
L158A
L158W
L158Q
L158D
L158P
L178A
L178W
L178Q
L178D
L178P
V391A
V391W
V391Q
V391D
V391P
I461A
I461W
I461Q
I461D
I461P
I718A
I718W
I718Q
I718D
I718P
F719A
F719W
F719Q
F719D
F719P
L728A
L728W
L728Q
L728D
L728P
L778A
L778W
L778Q
L778D
L778P
V809A
V809W
V809Q
V809D
V809P
is in library after recombination
fitness at 30 °C
fitness at 42 °C
hydrophobicity with mutation
1 2 3 mutation 4
0
ppc (b3956, P00864)
883
high fitness
reduced fitness
low fitness
no data / not in library
is in library after recombination
bold verified ts mutant
beta-sheets
alpha-helices
turns
binding sites
active sites
-3.7
hydrophobicity (Kyte & Doolittle, 1982, J. Mol., 157, 105-132)
3.7

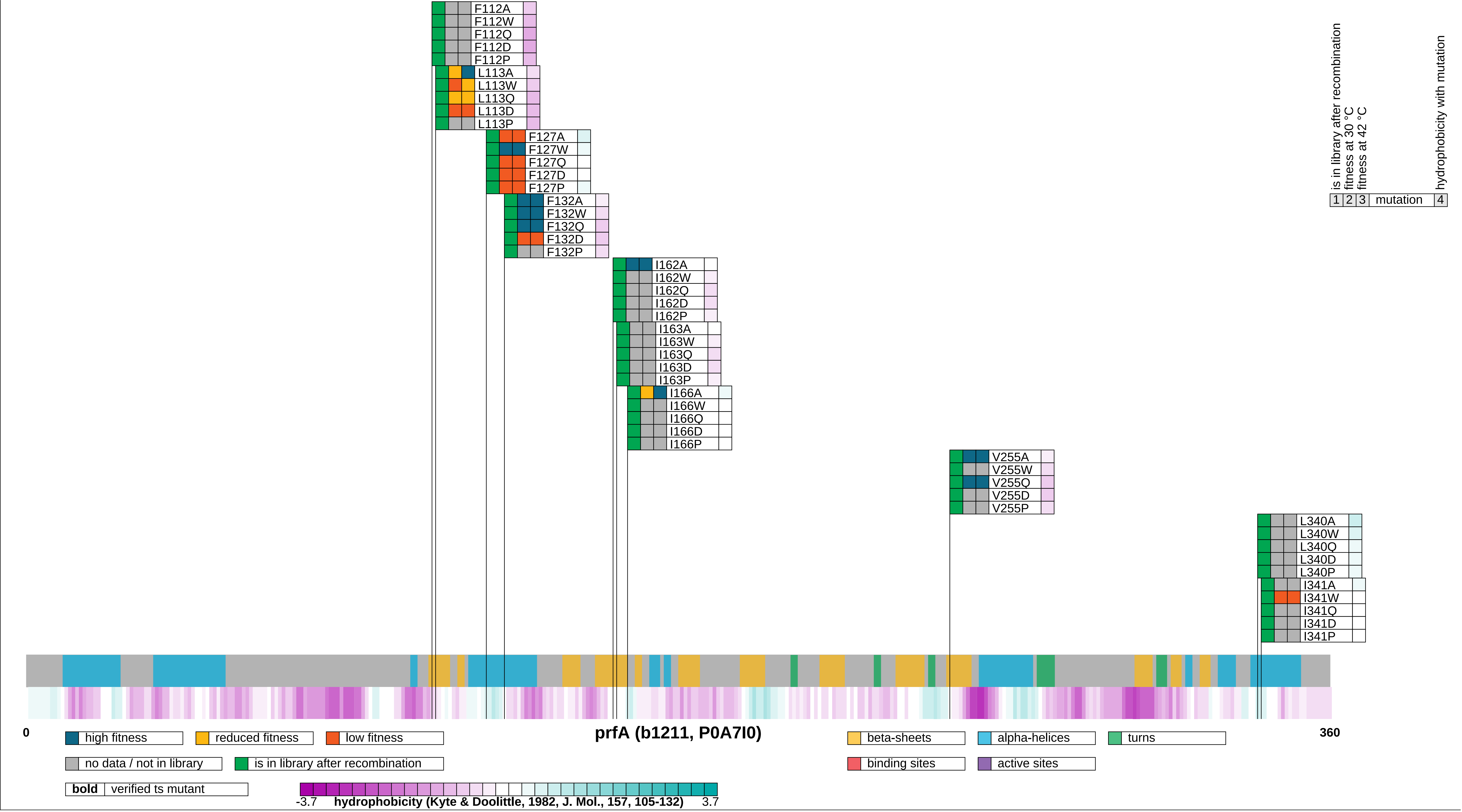

F112A
F112W
F112Q
F112D
F112P
L113A
L113W
L113Q
L113D
L113P
F127A
F127W
F127Q
F127D
F127P
F132A
F132W
F132Q
F132D
F132P
I162A
I162W
I162Q
I162D
I162P
I163A
I163W
I163Q
I163D
I163P
I166A
I166W
I166Q
I166D
I166P
V255A
V255W
V255Q
V255D
V255P
L340A
L340W
L340Q
L340D
L340P
I341A
I341W
I341Q
I341D
I341P
is in library after recombination
fitness at 30 °C
fitness at 42 °C
hydrophobicity with mutation
1 2 3 mutation 4
0
prfA (b1211, P0A7I0)
360
high fitness
reduced fitness
low fitness
no data / not in library
is in library after recombination
bold verified ts mutant
beta-sheets
alpha-helices
turns
binding sites
active sites
-3.7
hydrophobicity (Kyte & Doolittle, 1982, J. Mol., 157, 105-132)
3.7

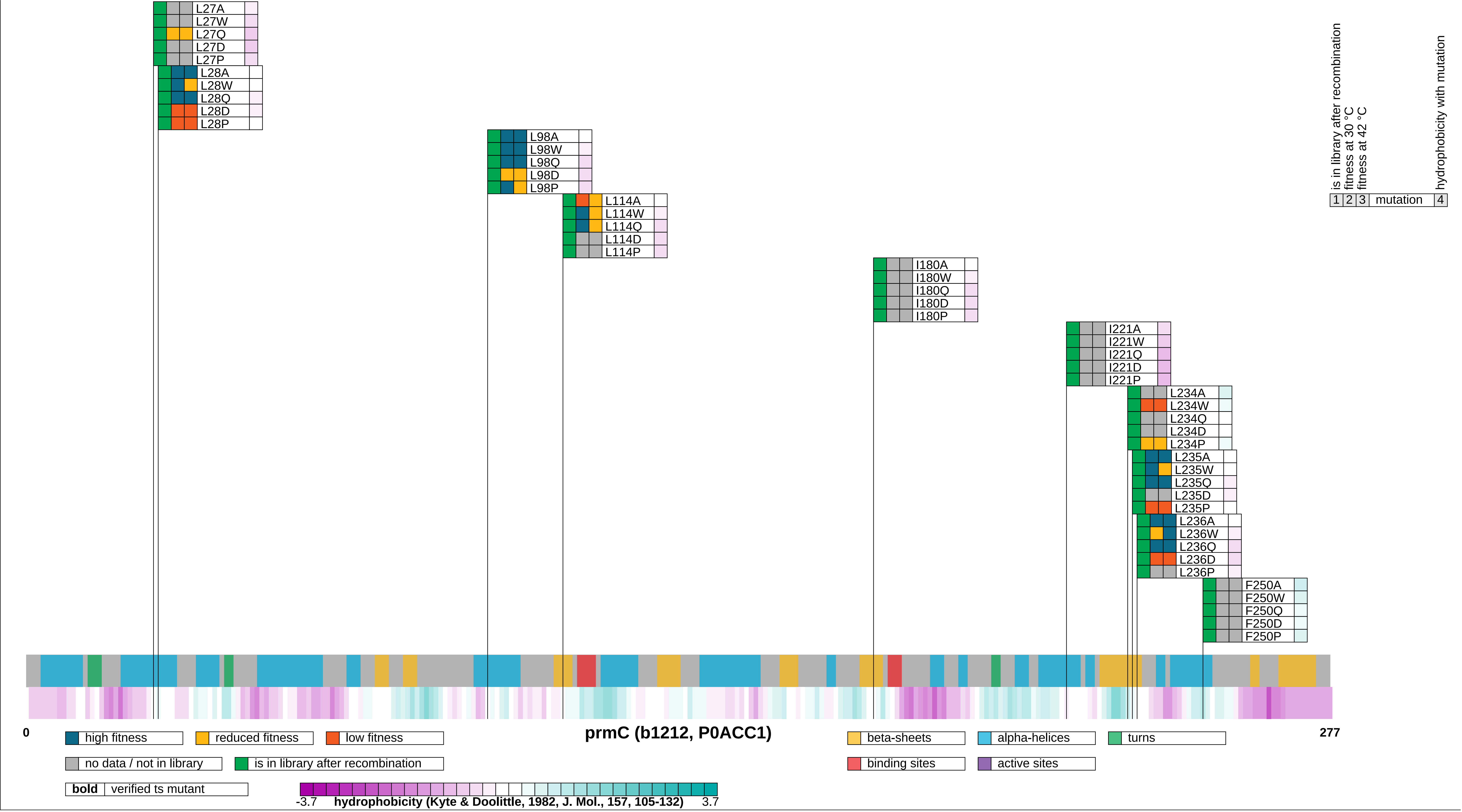

L27A
L27W
L27Q
L27D
L27P
L28A
L28W
L28Q
L28D
L28P
L98A
L98W
L98Q
L98D
L98P
L114A
L114W
L114Q
L114D
L114P
I180A
I180W
I180Q
I180D
I180P
I221A
I221W
I221Q
I221D
I221P
L234A
L234W
L234Q
L234D
L234P
L235A
L235W
L235Q
L235D
L235P
L236A
L236W
L236Q
L236D
L236P
F250A
F250W
F250Q
F250D
F250P
is in library after recombination
fitness at 30 °C
fitness at 42 °C
hydrophobicity with mutation
1
2
3
mutation
4
0
prmC (b1212, P0ACC1)
277
high fitness
reduced fitness
low fitness
no data / not in library
is in library after recombination
bold
verified ts mutant
beta-sheets
alpha-helices
turns
binding sites
active sites
-3.7
hydrophobicity (Kyte & Doolittle, 1982, J. Mol., 157, 105-132)
3.7

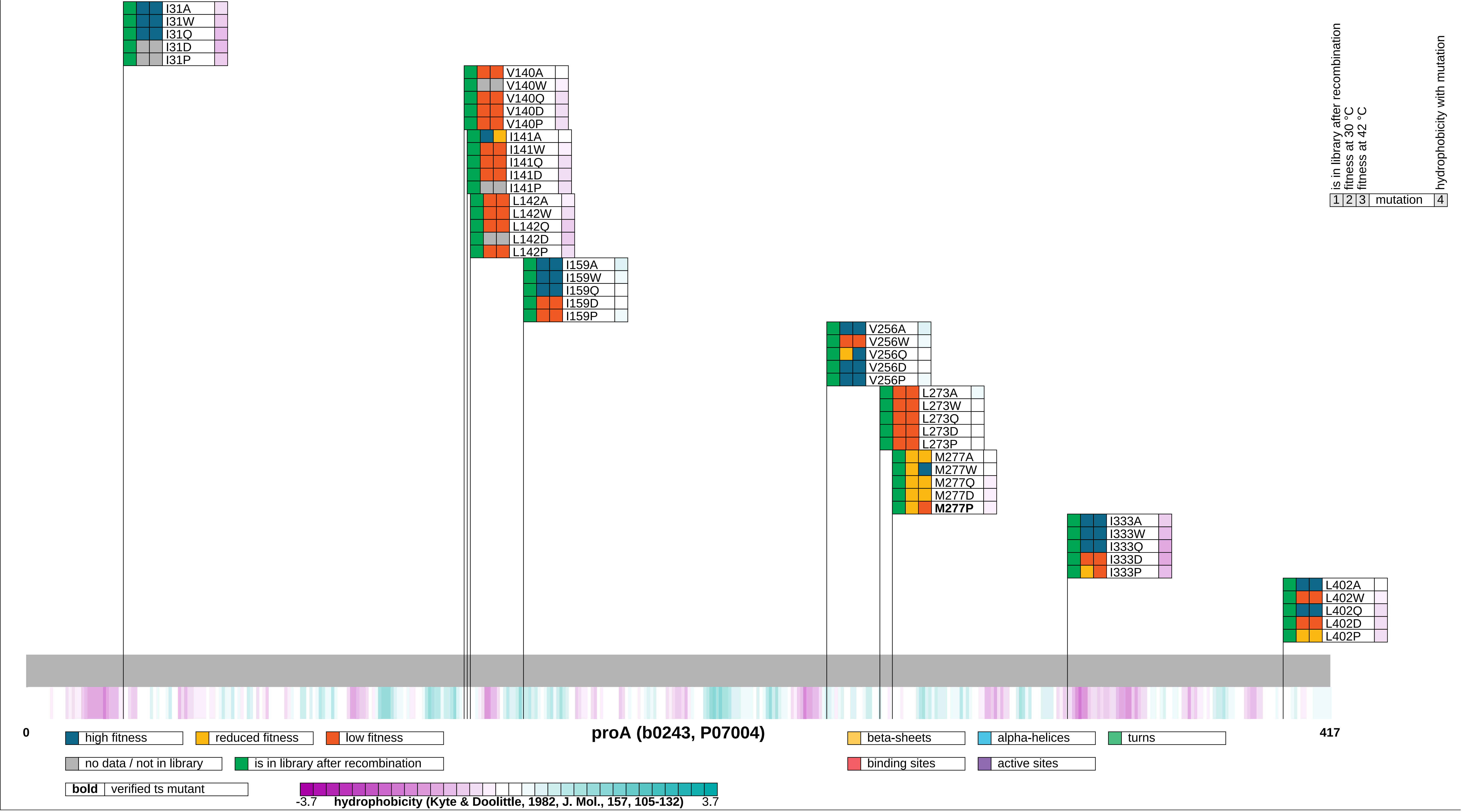
I31A
I31W
I31Q
I31D
I31P
V140A
V140W
V140Q
V140D
V140P
I141A
I141W
I141Q
I141D
I141P
L142A
L142W
L142Q
L142D
L142P
I159A
I159W
I159Q
I159D
I159P
V256A
V256W
V256Q
V256D
V256P
L273A
L273W
L273Q
L273D
L273P
M277A
M277W
M277Q
M277D
M277P
I333A
I333W
I333Q
I333D
I333P
L402A
L402W
L402Q
L402D
L402P
is in library after recombination
fitness at 30 °C
fitness at 42 °C
hydrophobicity with mutation
1 2 3 mutation 4
0
proA (b0243, P07004)
417
high fitness
reduced fitness
low fitness
beta-sheets
alpha-helices
turns
no data / not in library
is in library after recombination
binding sites
active sites
bold verified ts mutant
-3.7
hydrophobicity (Kyte & Doolittle, 1982, J. Mol., 157, 105-132)
3.7

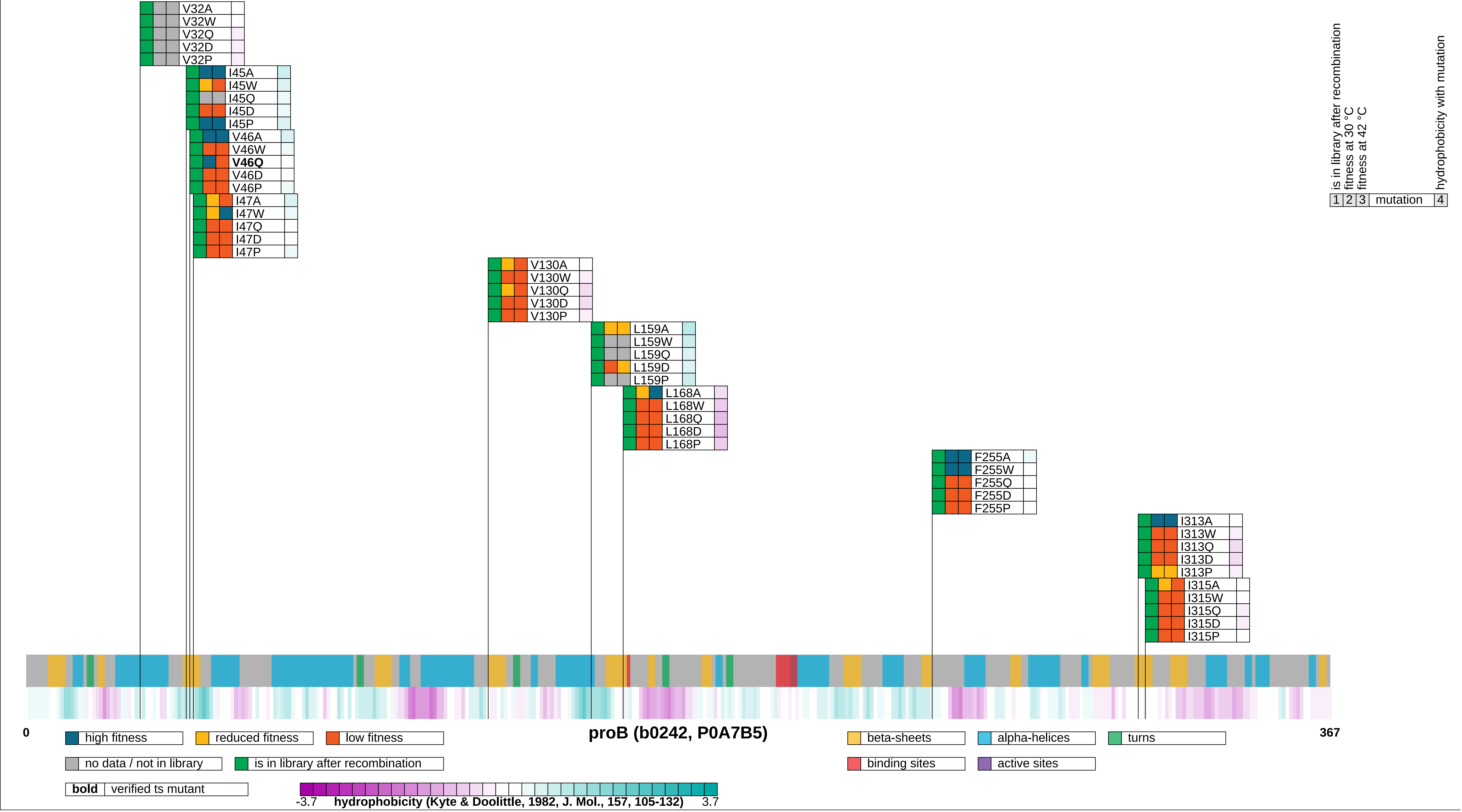
V32A
V32W
V32Q
V32D
V32P
I45A
I45W
I45Q
I45D
I45P
V46A
V46W
V46Q
V46D
V46P
I47A
I47W
I47Q
I47D
I47P
V130A
V130W
V130Q
V130D
V130P
L159A
L159W
L159Q
L159D
L159P
L168A
L168W
L168Q
L168D
L168P
F255A
F255W
F255Q
F255D
F255P
I313A
I313W
I313Q
I313D
I313P
I315A
I315W
I315Q
I315D
I315P
is in library after recombination
fitness at 30 °C
fitness at 42 °C
hydrophobicity with mutation
1 2 3 mutation 4
0
proB (b0242, P0A7B5)
367
high fitness
reduced fitness
low fitness
beta-sheets
alpha-helices
turns
no data / not in library
is in library after recombination
binding sites
active sites
bold verified ts mutant
-3.7 hydrophobicity (Kyte & Doolittle, 1982, J. Mol., 157, 105-132) 3.7

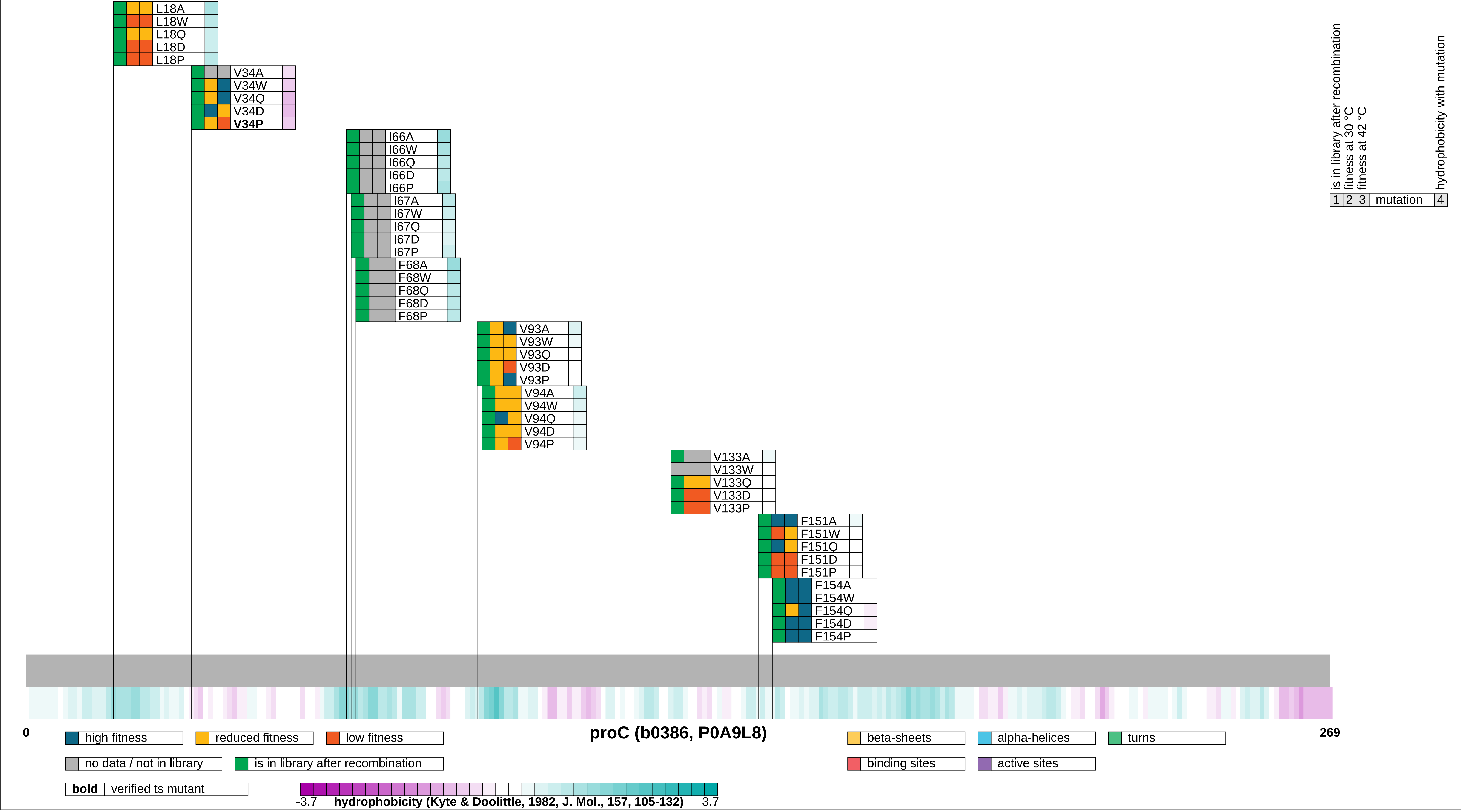
L18A
L18W
L18Q
L18D
L18P
V34A
V34W
V34Q
V34D
V34P
I66A
I66W
I66Q
I66D
I66P
I67A
I67W
I67Q
I67D
I67P
F68A
F68W
F68Q
F68D
F68P
V93A
V93W
V93Q
V93D
V93P
V94A
V94W
V94Q
V94D
V94P
V133A
V133W
V133Q
V133D
V133P
F151A
F151W
F151Q
F151D
F151P
F154A
F154W
F154Q
F154D
F154P
is in library after recombination
fitness at 30 °C
fitness at 42 °C
hydrophobicity with mutation
1 2 3 mutation 4
0
proC (b0386, P0A9L8)
269
high fitness
reduced fitness
low fitness
beta-sheets
alpha-helices
turns
no data / not in library
is in library after recombination
binding sites
active sites
bold verified ts mutant
-3.7
hydrophobicity (Kyte & Doolittle, 1982, J. Mol., 157, 105-132)
3.7

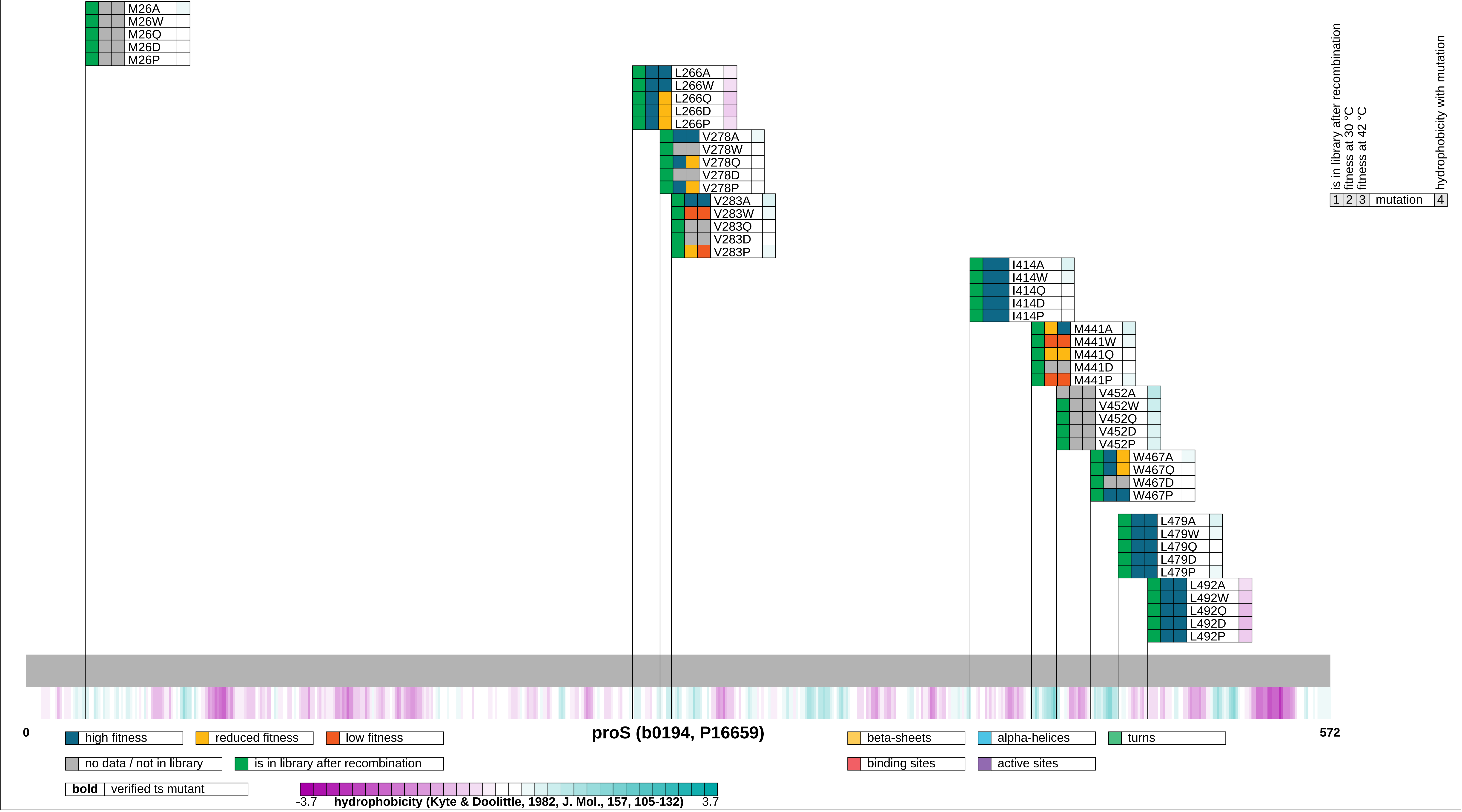
M26A
M26W
M26Q
M26D
M26P
L266A
L266W
L266Q
L266D
L266P
V278A
V278W
V278Q
V278D
V278P
V283A
V283W
V283Q
V283D
V283P
I414A
I414W
I414Q
I414D
I414P
M441A
M441W
M441Q
M441D
M441P
V452A
V452W
V452Q
V452D
V452P
W467A
W467Q
W467D
W467P
L479A
L479W
L479Q
L479D
L479P
L492A
L492W
L492Q
L492D
L492P
is in library after recombination
fitness at 30 °C
fitness at 42 °C
hydrophobicity with mutation
1 2 3 mutation 4
0
proS (b0194, P16659)
572
high fitness
reduced fitness
low fitness
no data / not in library
is in library after recombination
bold verified ts mutant
beta-sheets
alpha-helices
turns
binding sites
active sites
-3.7
hydrophobicity (Kyte & Doolittle, 1982, J. Mol., 157, 105-132)
3.7

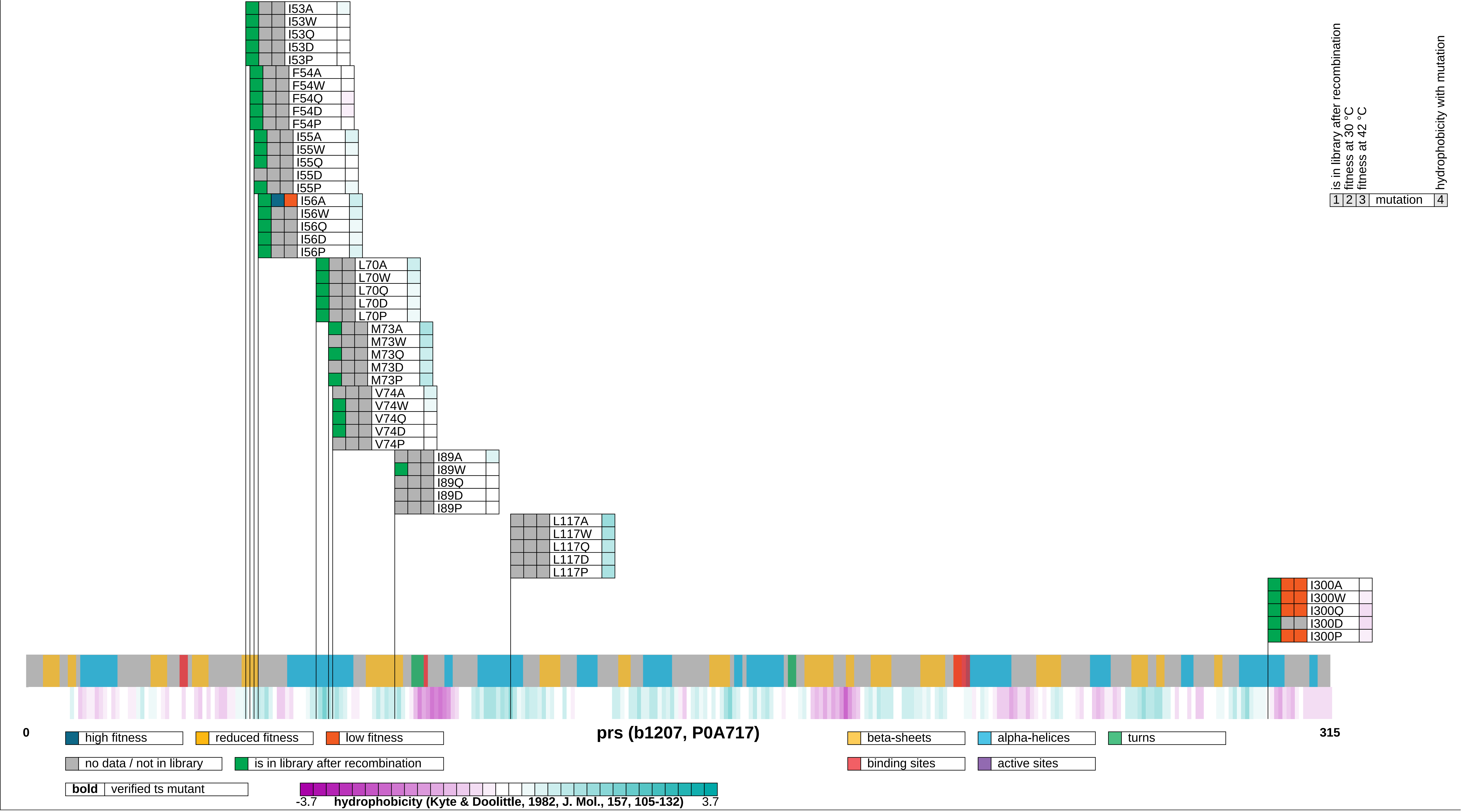
I53A
I53W
I53Q
I53D
I53P
F54A
F54W
F54Q
F54D
F54P
I55A
I55W
I55Q
I55D
I55P
I56A
I56W
I56Q
I56D
I56P
L70A
L70W
L70Q
L70D
L70P
M73A
M73W
M73Q
M73D
M73P
V74A
V74W
V74Q
V74D
V74P
I89A
I89W
I89Q
I89D
I89P
L117A
L117W
L117Q
L117D
L117P
I300A
I300W
I300Q
I300D
I300P
is in library after recombination
fitness at 30 °C
fitness at 42 °C
hydrophobicity with mutation
1 2 3 mutation 4
0
prs (b1207, P0A717)
315
high fitness
reduced fitness
low fitness
beta-sheets
alpha-helices
turns
no data / not in library
is in library after recombination
binding sites
active sites
bold verified ts mutant
-3.7 hydrophobicity (Kyte & Doolittle, 1982, J. Mol., 157, 105-132) 3.7

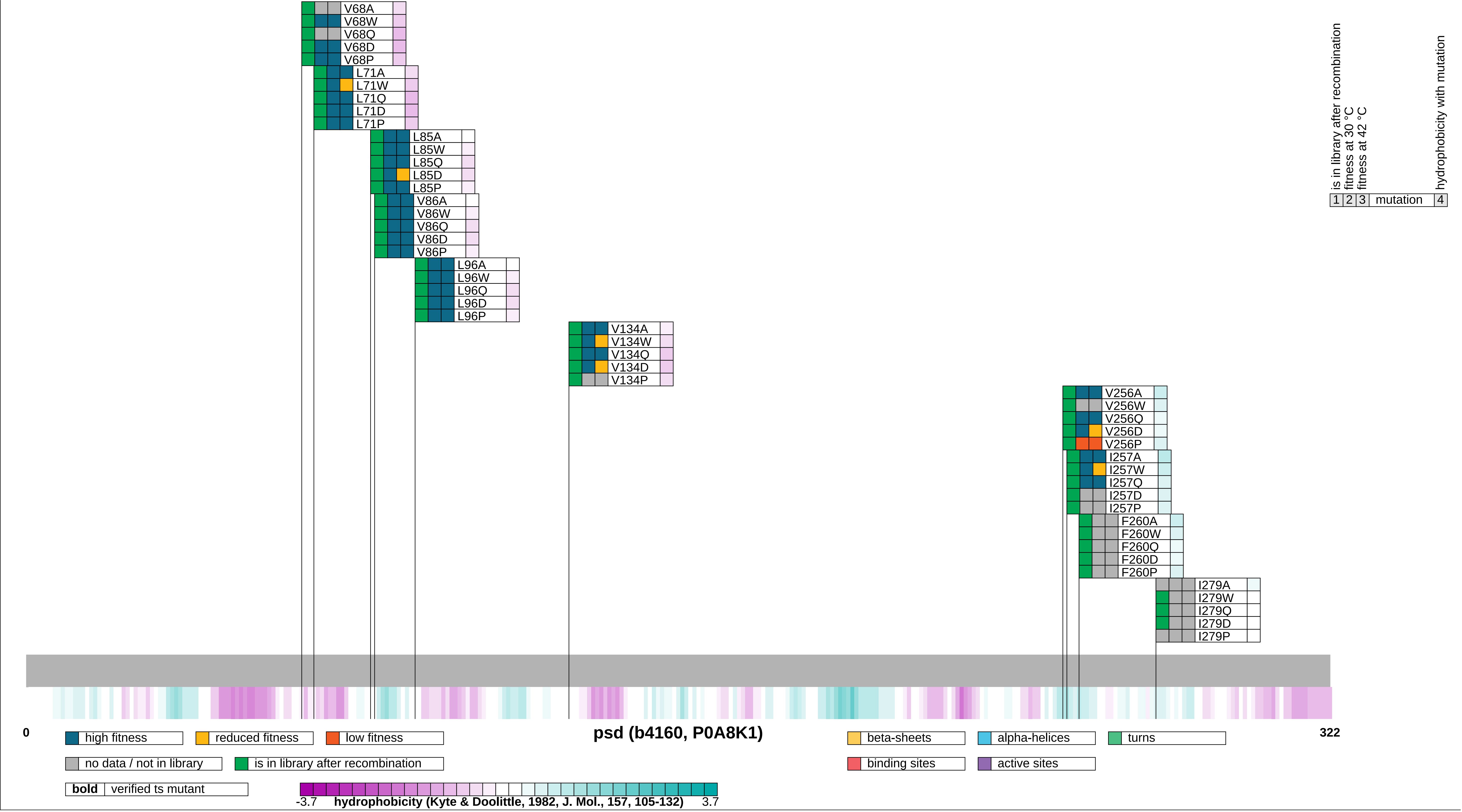

is in library after recombination
fitness at 30 °C
fitness at 42 °C
hydrophobicity with mutation
1 2 3 mutation 4
V68A
V68W
V68Q
V68D
V68P
L71A
L71W
L71Q
L71D
L71P
L85A
L85W
L85Q
L85D
L85P
V86A
V86W
V86Q
V86D
V86P
L96A
L96W
L96Q
L96D
L96P
V134A
V134W
V134Q
V134D
V134P
V256A
V256W
V256Q
V256D
V256P
I257A
I257W
I257Q
I257D
I257P
F260A
F260W
F260Q
F260D
F260P
I279A
I279W
I279Q
I279D
I279P
0
psd (b4160, P0A8K1)
322
high fitness
reduced fitness
low fitness
no data / not in library
is in library after recombination
bold verified ts mutant
beta-sheets
alpha-helices
turns
binding sites
active sites
-3.7 hydrophobicity (Kyte & Doolittle, 1982, J. Mol., 157, 105-132) 3.7

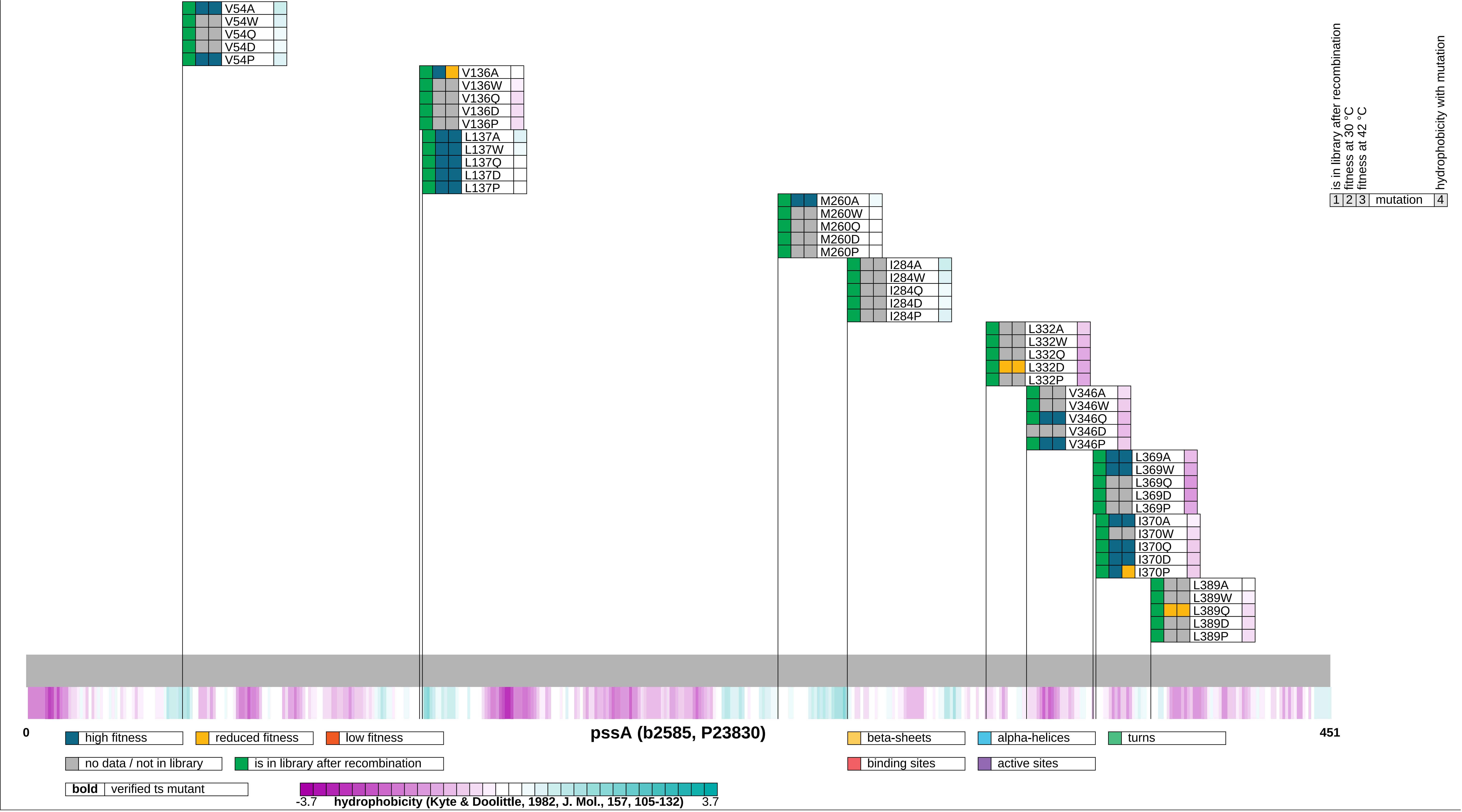

V54A
V54W
V54Q
V54D
V54P
V136A
V136W
V136Q
V136D
V136P
L137A
L137W
L137Q
L137D
L137P
M260A
M260W
M260Q
M260D
M260P
I284A
I284W
I284Q
I284D
I284P
L332A
L332W
L332Q
L332D
L332P
V346A
V346W
V346Q
V346D
V346P
L369A
L369W
L369Q
L369D
L369P
I370A
I370W
I370Q
I370D
I370P
L389A
L389W
L389Q
L389D
L389P
is in library after recombination
fitness at 30 °C
fitness at 42 °C
hydrophobicity with mutation
1 2 3 mutation 4
0
pssA (b2585, P23830)
451
high fitness
reduced fitness
low fitness
no data / not in library
is in library after recombination
bold verified ts mutant
beta-sheets
alpha-helices
turns
binding sites
active sites
-3.7
hydrophobicity (Kyte & Doolittle, 1982, J. Mol., 157, 105-132)
3.7

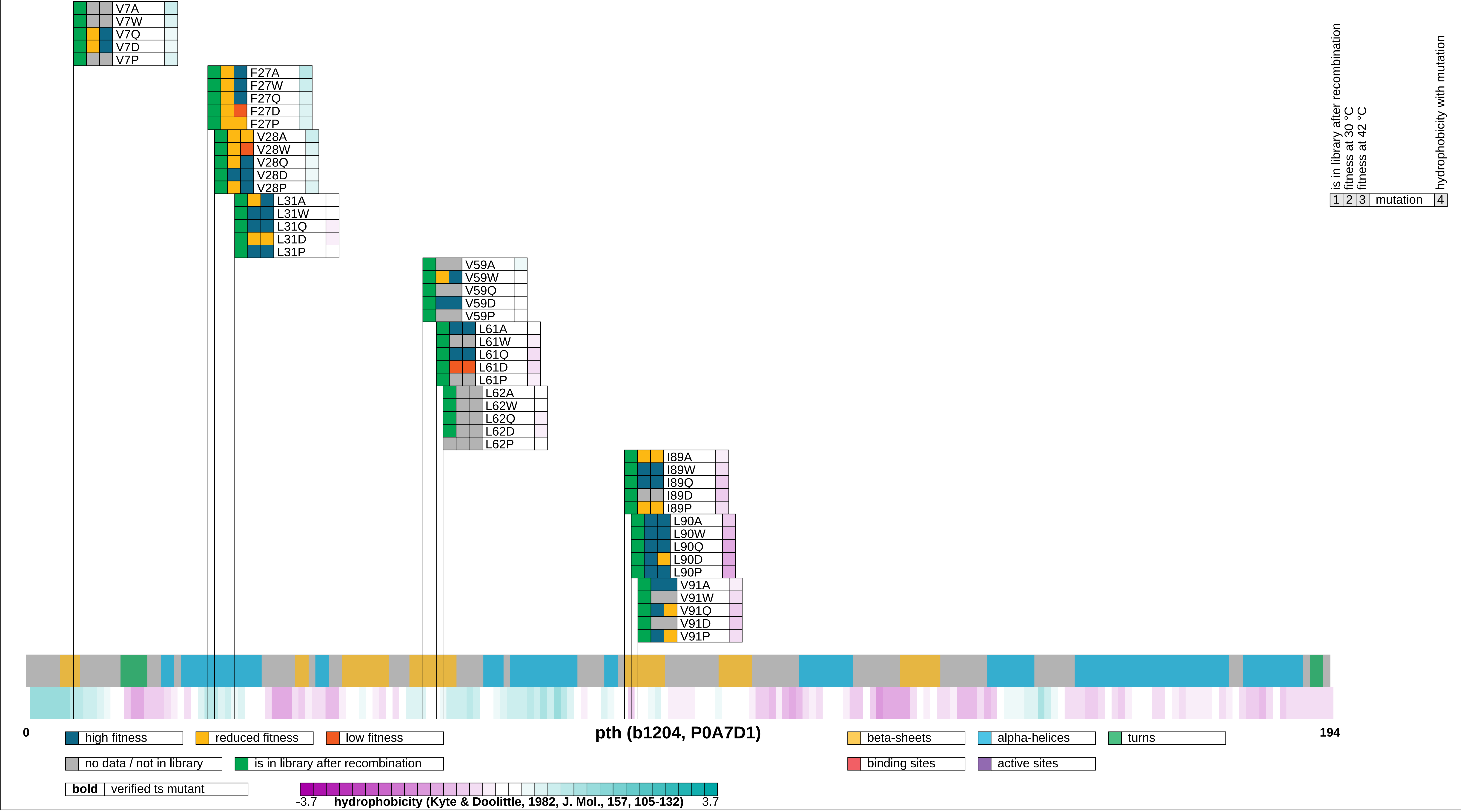
V7A
V7W
V7Q
V7D
V7P
F27A
F27W
F27Q
F27D
F27P
V28A
V28W
V28Q
V28D
V28P
L31A
L31W
L31Q
L31D
L31P
V59A
V59W
V59Q
V59D
V59P
L61A
L61W
L61Q
L61D
L61P
L62A
L62W
L62Q
L62D
L62P
I89A
I89W
I89Q
I89D
I89P
L90A
L90W
L90Q
L90D
L90P
V91A
V91W
V91Q
V91D
V91P
is in library after recombination
fitness at 30 °C
fitness at 42 °C
hydrophobicity with mutation
1
2
3
mutation
4
0
pth (b1204, P0A7D1)
194
high fitness
reduced fitness
low fitness
beta-sheets
alpha-helices
turns
no data / not in library
is in library after recombination
binding sites
active sites
bold
verified ts mutant
-3.7
hydrophobicity (Kyte & Doolittle, 1982, J. Mol., 157, 105-132)
3.7

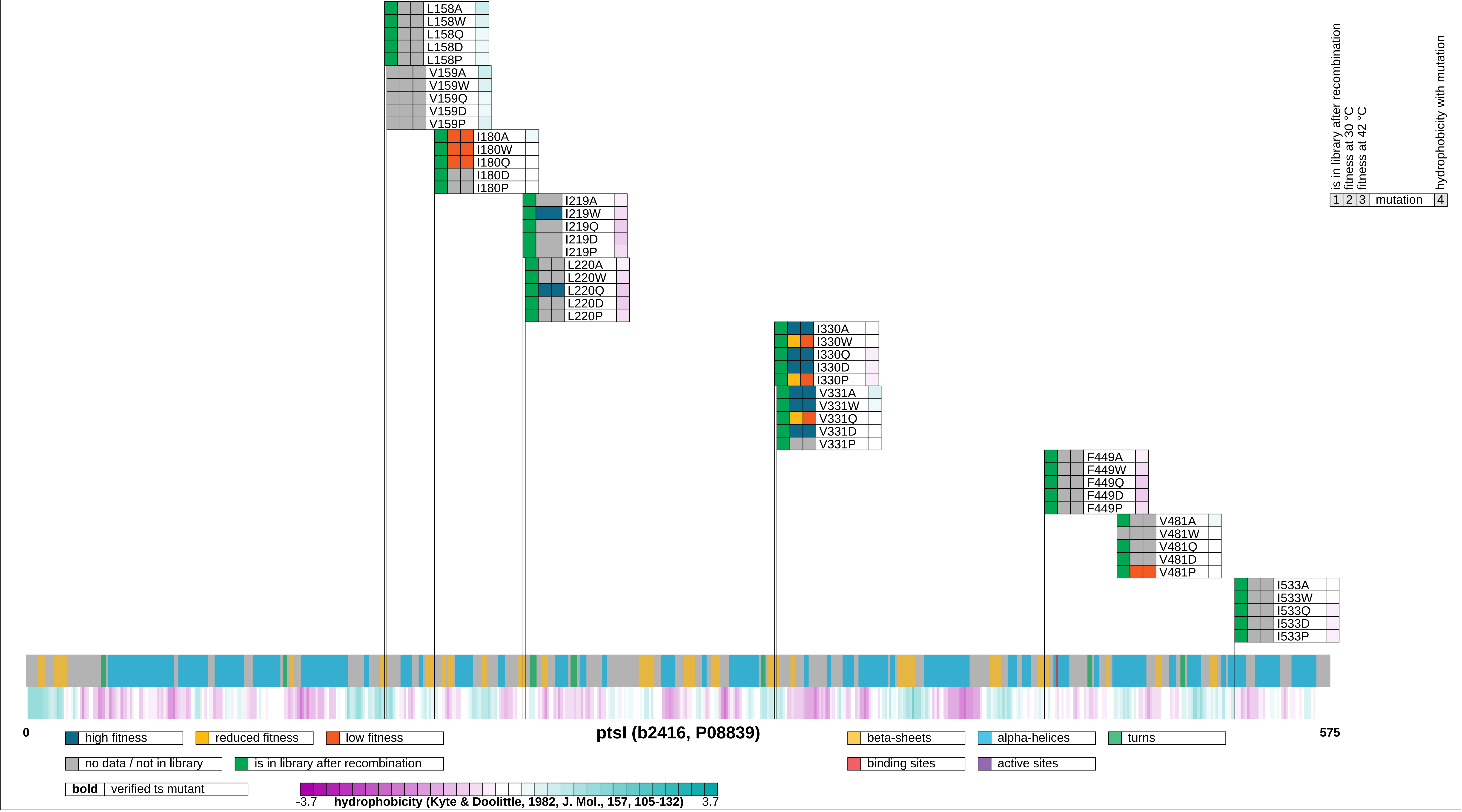

L158A
L158W
L158Q
L158D
L158P
V159A
V159W
V159Q
V159D
V159P
I180A
I180W
I180Q
I180D
I180P
I219A
I219W
I219Q
I219D
I219P
L220A
L220W
L220Q
L220D
L220P
I330A
I330W
I330Q
I330D
I330P
V331A
V331W
V331Q
V331D
V331P
F449A
F449W
F449Q
F449D
F449P
V481A
V481W
V481Q
V481D
V481P
I533A
I533W
I533Q
I533D
I533P
is in library after recombination
fitness at 30 °C
fitness at 42 °C
hydrophobicity with mutation
1 2 3 mutation 4
0
575
ptsI (b2416, P08839)
high fitness
reduced fitness
low fitness
beta-sheets
alpha-helices
turns
no data / not in library
is in library after recombination
binding sites
active sites
bold verified ts mutant
-3.7
hydrophobicity (Kyte & Doolittle, 1982, J. Mol., 157, 105-132)
3.7

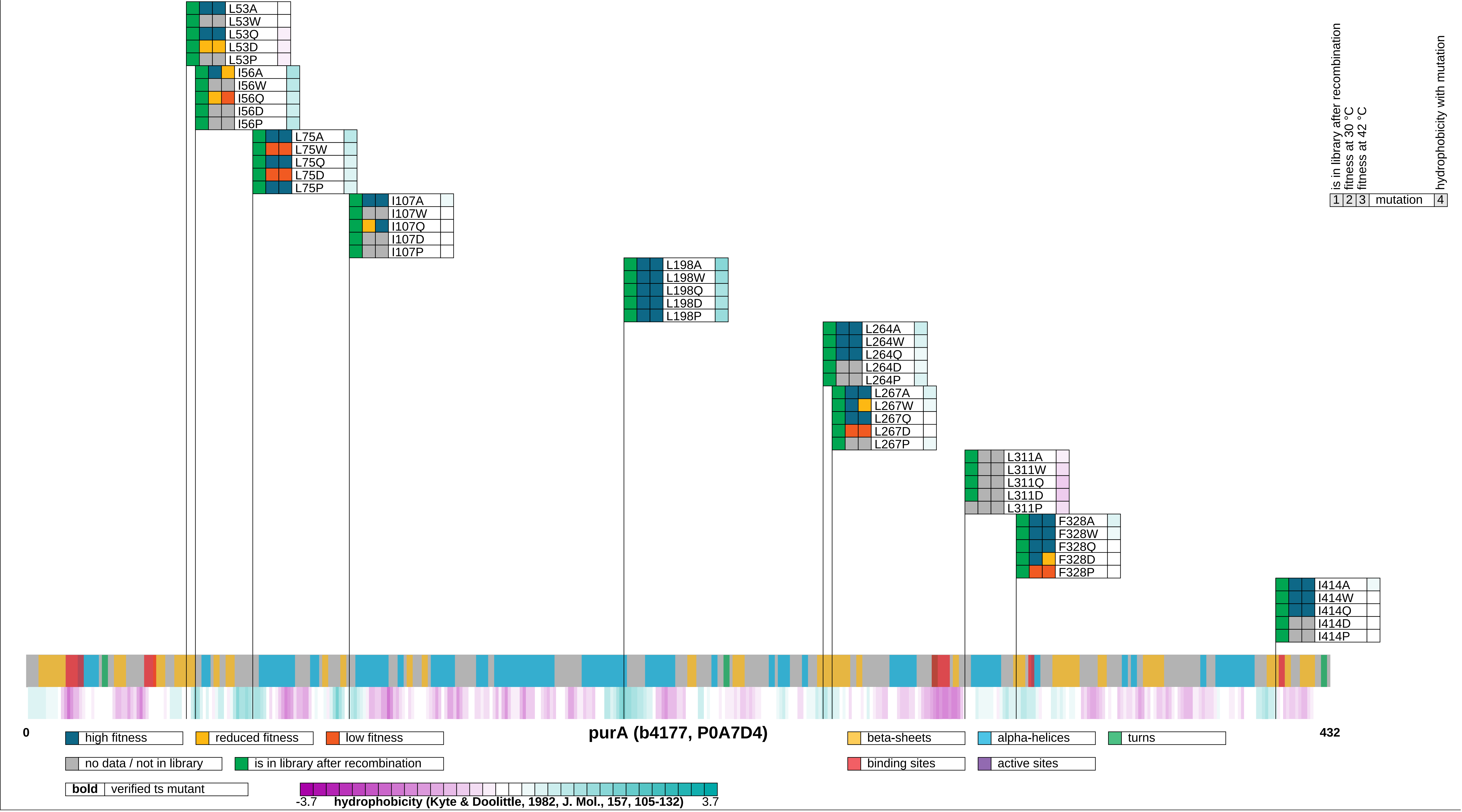
L53A
L53W
L53Q
L53D
L53P
I56A
I56W
I56Q
I56D
I56P
L75A
L75W
L75Q
L75D
L75P
I107A
I107W
I107Q
I107D
I107P
L198A
L198W
L198Q
L198D
L198P
L264A
L264W
L264Q
L264D
L264P
L267A
L267W
L267Q
L267D
L267P
L311A
L311W
L311Q
L311D
L311P
F328A
F328W
F328Q
F328D
F328P
I414A
I414W
I414Q
I414D
I414P
is in library after recombination
fitness at 30 °C
fitness at 42 °C
hydrophobicity with mutation
1 2 3 mutation 4
0
432
purA (b4177, P0A7D4)
high fitness
reduced fitness
low fitness
no data / not in library
is in library after recombination
bold verified ts mutant
beta-sheets
alpha-helices
turns
binding sites
active sites
-3.7
hydrophobicity (Kyte & Doolittle, 1982, J. Mol., 157, 105-132)
3.7

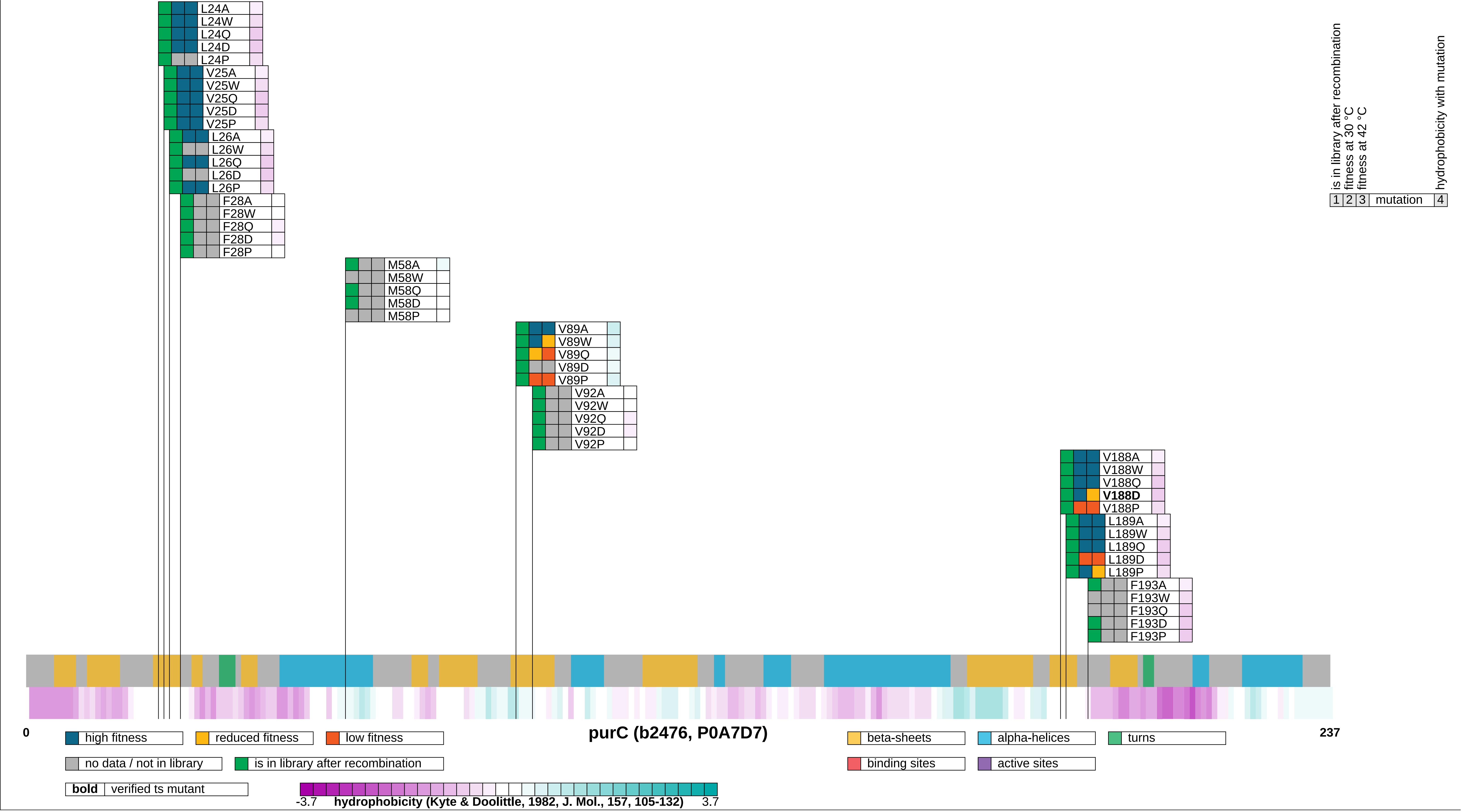

L24A
L24W
L24Q
L24D
L24P
V25A
V25W
V25Q
V25D
V25P
L26A
L26W
L26Q
L26D
L26P
F28A
F28W
F28Q
F28D
F28P
M58A
M58W
M58Q
M58D
M58P
V89A
V89W
V89Q
V89D
V89P
V92A
V92W
V92Q
V92D
V92P
V188A
V188W
V188Q
V188D
V188P
L189A
L189W
L189Q
L189D
L189P
F193A
F193W
F193Q
F193D
F193P
is in library after recombination
fitness at 30 °C
fitness at 42 °C
hydrophobicity with mutation
1
2
3
mutation
4
0
237
purC (b2476, P0A7D7)
high fitness
reduced fitness
low fitness
beta-sheets
alpha-helices
turns
no data / not in library
is in library after recombination
binding sites
active sites
bold
verified ts mutant
-3.7
hydrophobicity (Kyte & Doolittle, 1982, J. Mol., 157, 105-132)
3.7

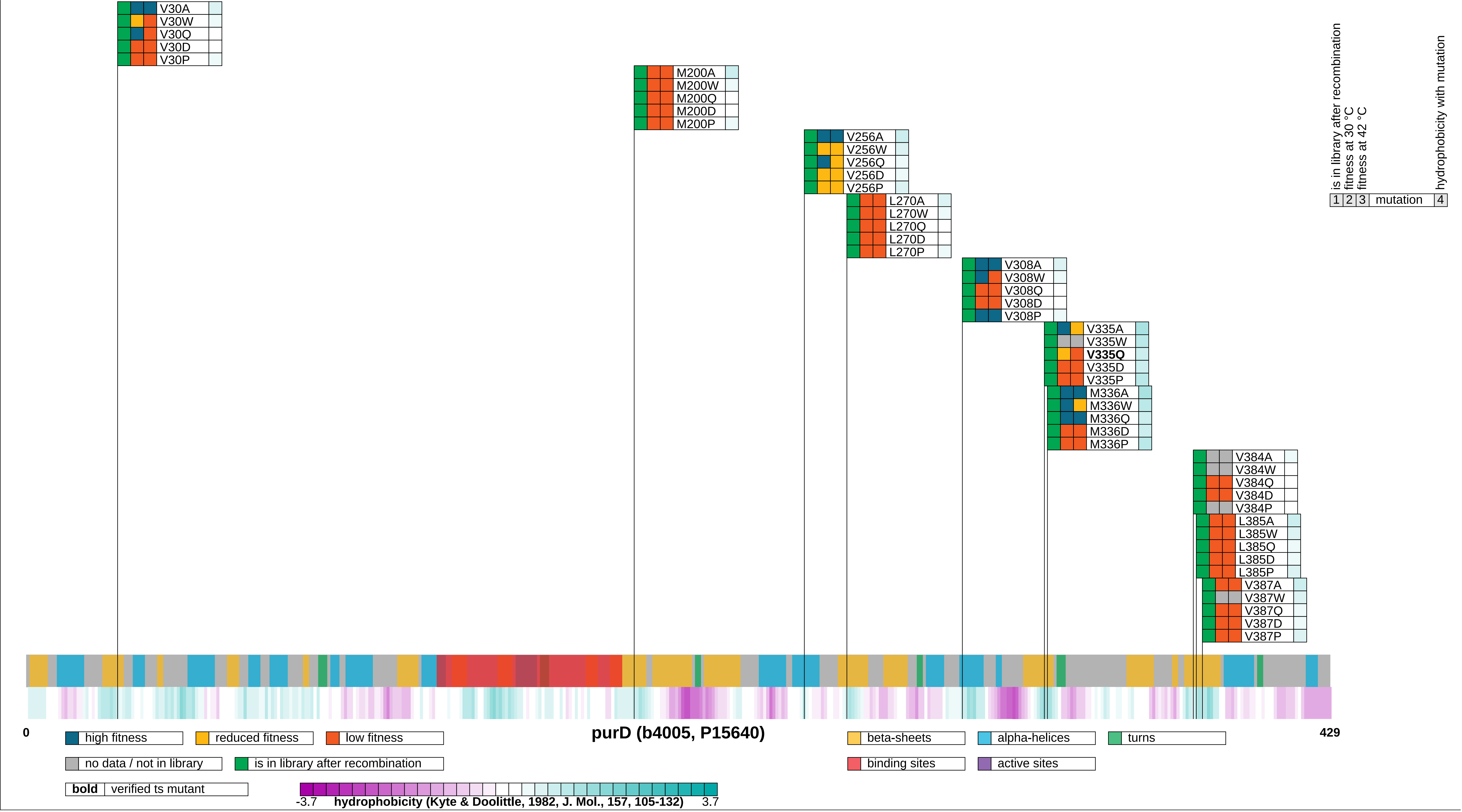

V30A
V30W
V30Q
V30D
V30P
M200A
M200W
M200Q
M200D
M200P
V256A
V256W
V256Q
V256D
V256P
L270A
L270W
L270Q
L270D
L270P
V308A
V308W
V308Q
V308D
V308P
V335A
V335W
V335Q
V335D
V335P
M336A
M336W
M336Q
M336D
M336P
V384A
V384W
V384Q
V384D
V384P
L385A
L385W
L385Q
L385D
L385P
V387A
V387W
V387Q
V387D
V387P
is in library after recombination
fitness at 30 °C
fitness at 42 °C
hydrophobicity with mutation
1 2 3 mutation 4
0
429
purD (b4005, P15640)
high fitness
reduced fitness
low fitness
no data / not in library
is in library after recombination
bold verified ts mutant
beta-sheets
alpha-helices
turns
binding sites
active sites
-3.7
hydrophobicity (Kyte & Doolittle, 1982, J. Mol., 157, 105-132)
3.7

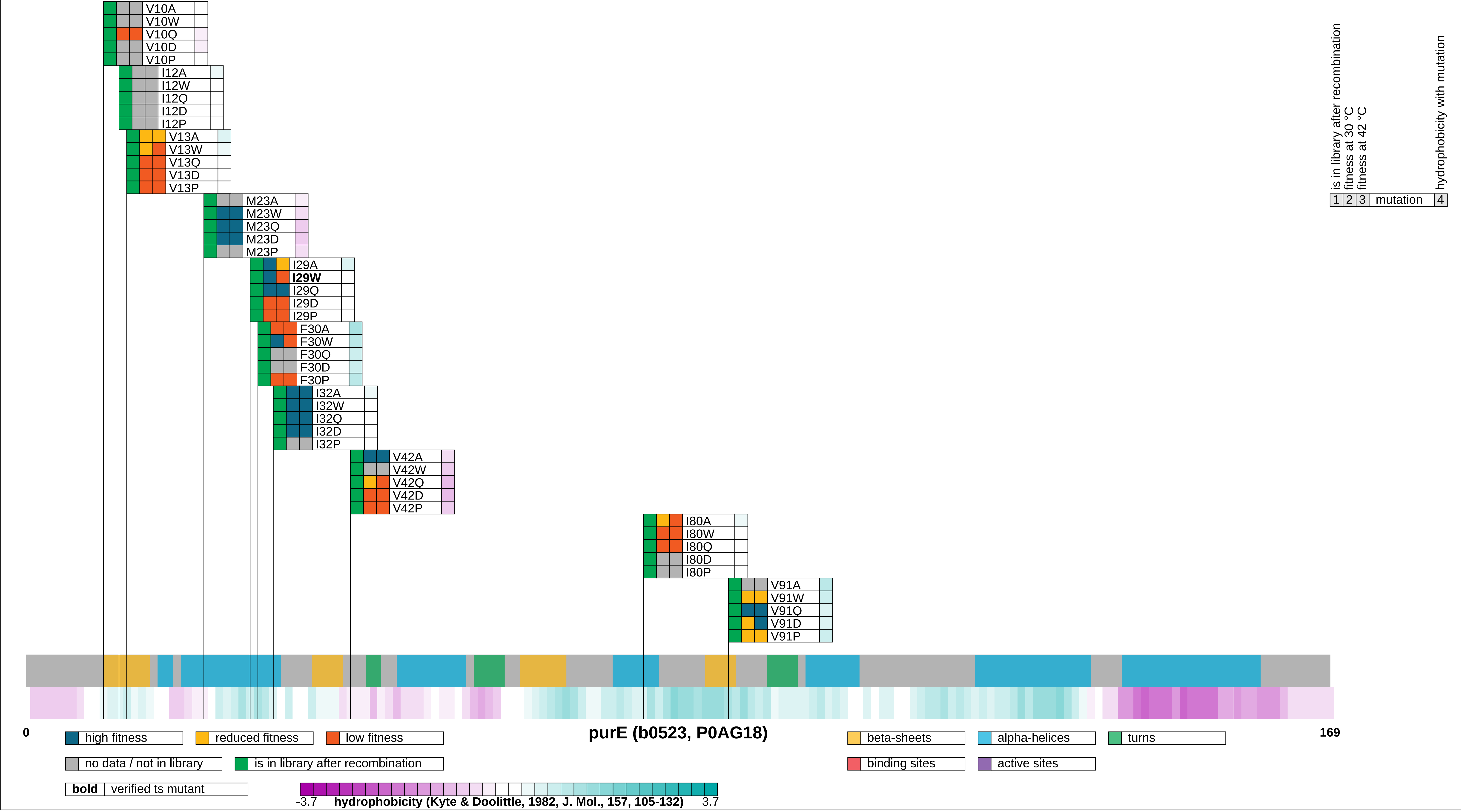

V10A
V10W
V10Q
V10D
V10P
I12A
I12W
I12Q
I12D
I12P
V13A
V13W
V13Q
V13D
V13P
M23A
M23W
M23Q
M23D
M23P
I29A
I29W
I29Q
I29D
I29P
F30A
F30W
F30Q
F30D
F30P
I32A
I32W
I32Q
I32D
I32P
V42A
V42W
V42Q
V42D
V42P
I80A
I80W
I80Q
I80D
I80P
V91A
V91W
V91Q
V91D
V91P
is in library after recombination
fitness at 30 °C
fitness at 42 °C
hydrophobicity with mutation
1
2
3
mutation
4
0
purE (b0523, P0AG18)
169
high fitness
reduced fitness
low fitness
beta-sheets
alpha-helices
turns
no data / not in library
is in library after recombination
binding sites
active sites
bold
verified ts mutant
-3.7
hydrophobicity (Kyte & Doolittle, 1982, J. Mol., 157, 105-132)
3.7

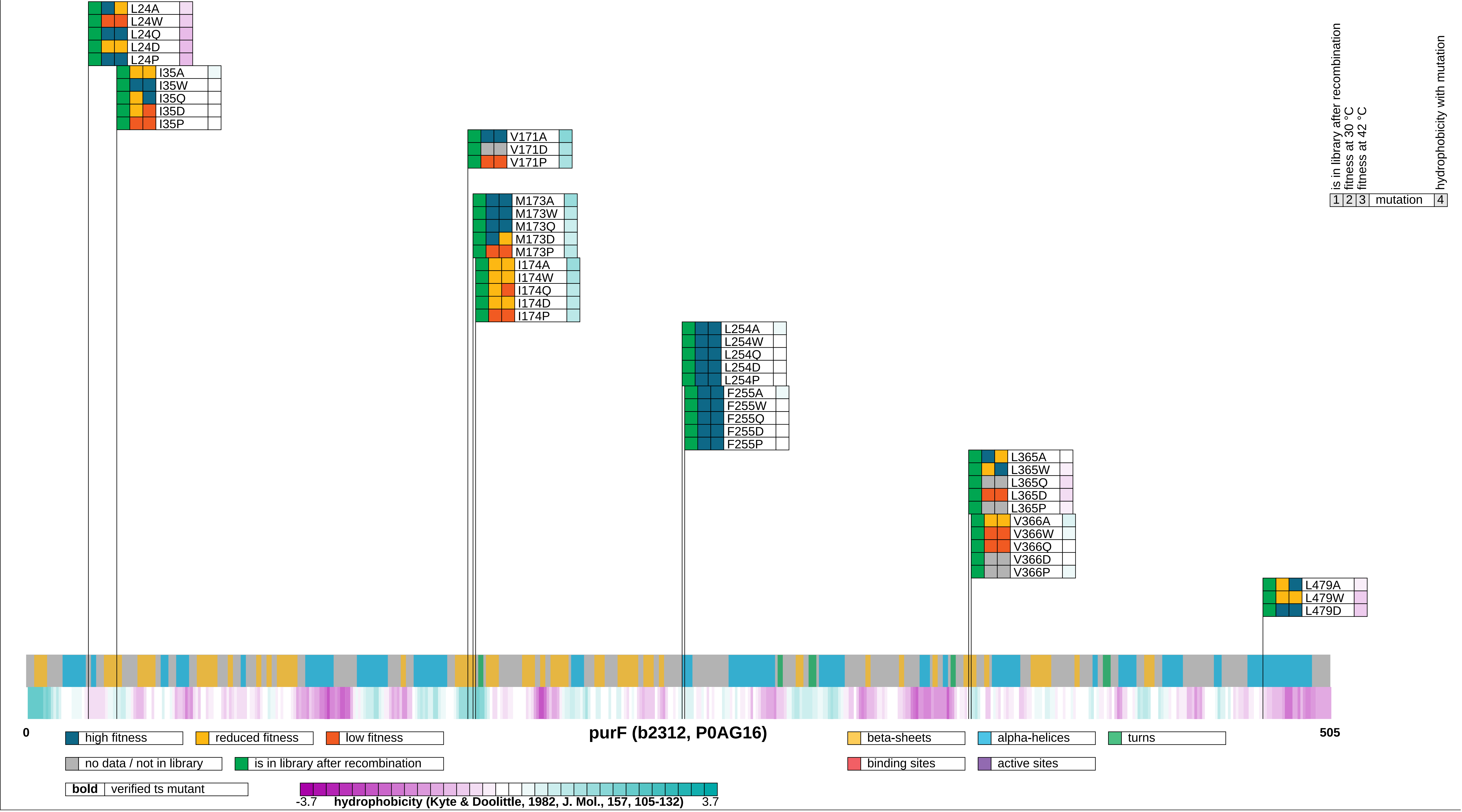
L24A
L24W
L24Q
L24D
L24P
I35A
I35W
I35Q
I35D
I35P
V171A
V171D
V171P
M173A
M173W
M173Q
M173D
M173P
I174A
I174W
I174Q
I174D
I174P
L254A
L254W
L254Q
L254D
L254P
F255A
F255W
F255Q
F255D
F255P
L365A
L365W
L365Q
L365D
L365P
V366A
V366W
V366Q
V366D
V366P
L479A
L479W
L479D
is in library after recombination
fitness at 30 °C
fitness at 42 °C
hydrophobicity with mutation
1 2 3 mutation 4
0
505
purF (b2312, P0AG16)
high fitness
reduced fitness
low fitness
no data / not in library
is in library after recombination
bold verified ts mutant
beta-sheets
alpha-helices
turns
binding sites
active sites
-3.7
hydrophobicity (Kyte & Doolittle, 1982, J. Mol., 157, 105-132)
3.7

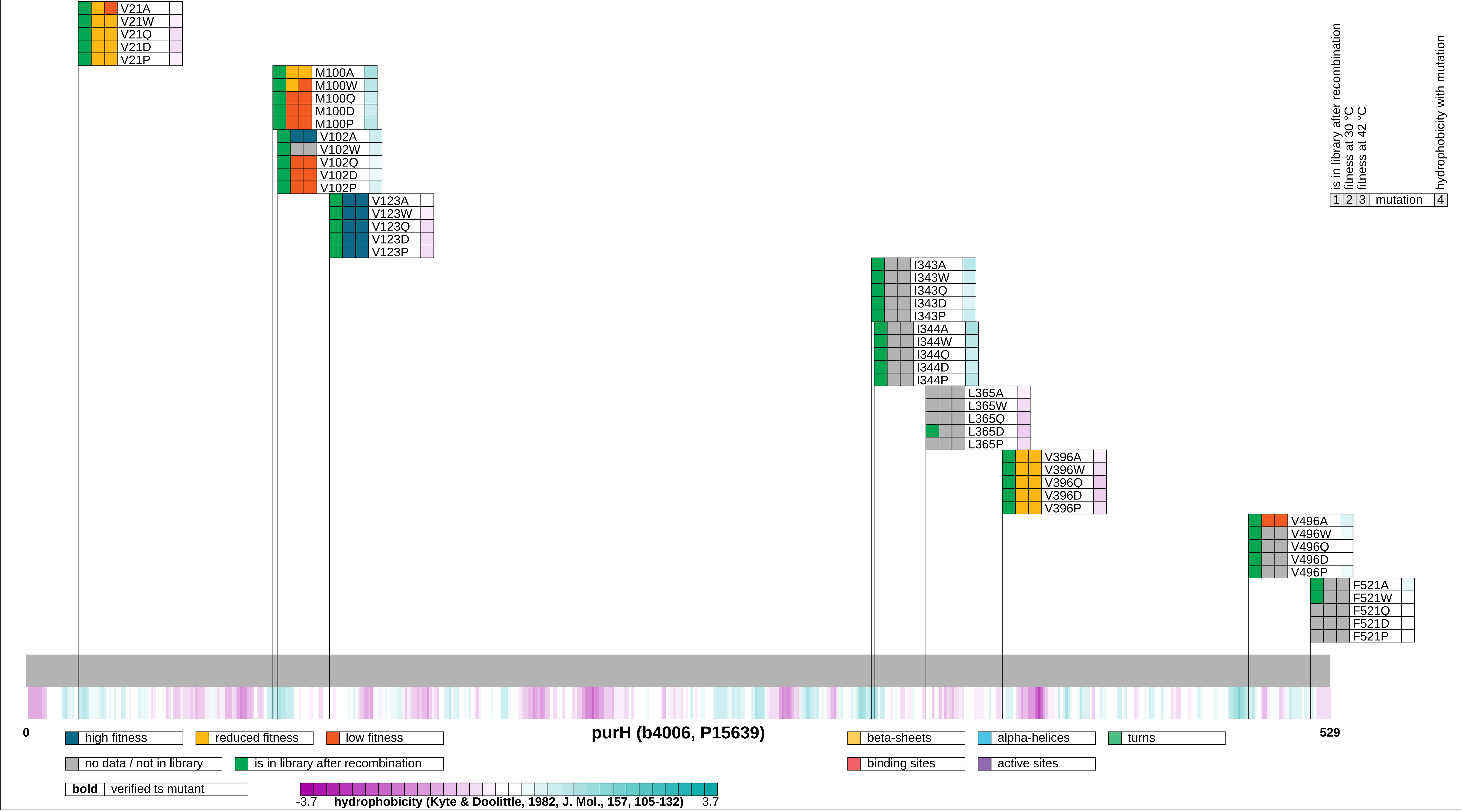

V21A
V21W
V21Q
V21D
V21P
M100A
M100W
M100Q
M100D
M100P
V102A
V102W
V102Q
V102D
V102P
V123A
V123W
V123Q
V123D
V123P
I343A
I343W
I343Q
I343D
I343P
I344A
I344W
I344Q
I344D
I344P
L365A
L365W
L365Q
L365D
L365P
V396A
V396W
V396Q
V396D
V396P
V496A
V496W
V496Q
V496D
V496P
F521A
F521W
F521Q
F521D
F521P
is in library after recombination
fitness at 30 °C
fitness at 42 °C
hydrophobicity with mutation
1
2
3
mutation
4
0
529
purH (b4006, P15639)
high fitness
reduced fitness
low fitness
no data / not in library
is in library after recombination
bold
verified ts mutant
beta-sheets
alpha-helices
turns
binding sites
active sites
-3.7
hydrophobicity (Kyte & Doolittle, 1982, J. Mol., 157, 105-132)
3.7

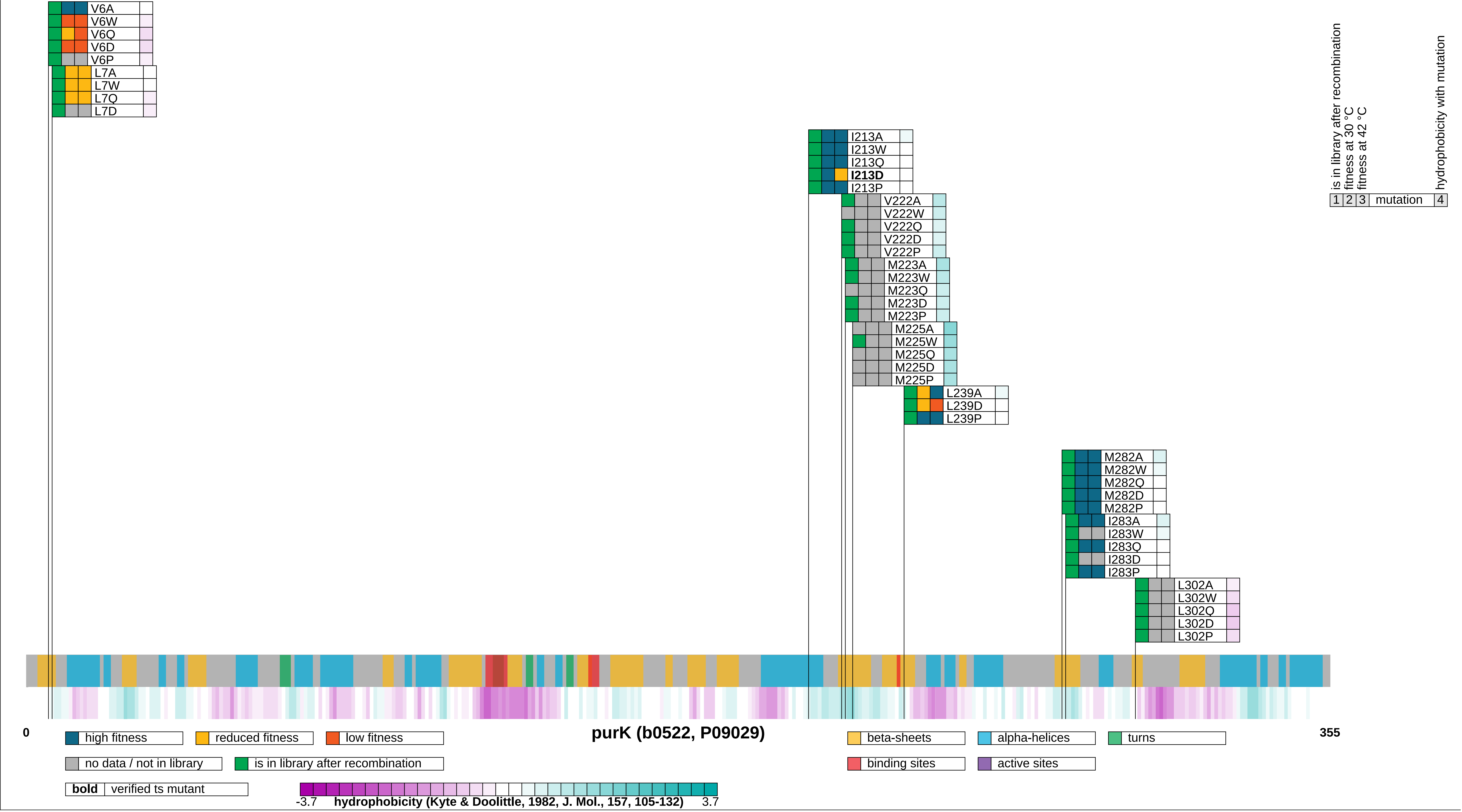
V6A
V6W
V6Q
V6D
V6P
L7A
L7W
L7Q
L7D
I213A
I213W
I213Q
I213D
I213P
V222A
V222W
V222Q
V222D
V222P
M223A
M223W
M223Q
M223D
M223P
M225A
M225W
M225Q
M225D
M225P
L239A
L239D
L239P
M282A
M282W
M282Q
M282D
M282P
I283A
I283W
I283Q
I283D
I283P
L302A
L302W
L302Q
L302D
L302P
is in library after recombination
fitness at 30 °C
fitness at 42 °C
hydrophobicity with mutation
1 2 3 mutation 4
0
355
purK (b0522, P09029)
high fitness
reduced fitness
low fitness
no data / not in library
is in library after recombination
bold verified ts mutant
beta-sheets
alpha-helices
turns
binding sites
active sites
-3.7 hydrophobicity (Kyte & Doolittle, 1982, J. Mol., 157, 105-132) 3.7

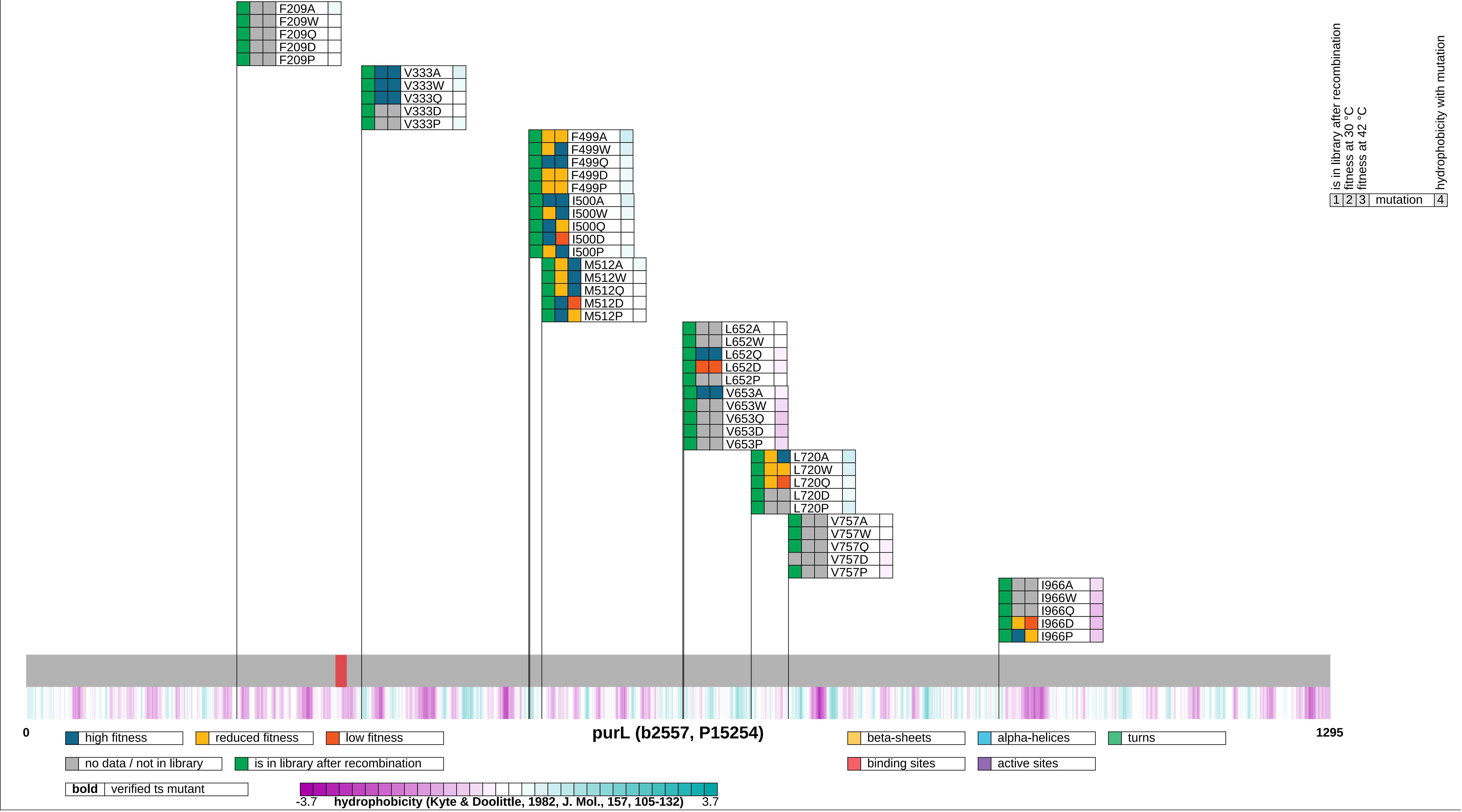

F209A
F209W
F209Q
F209D
F209P
V333A
V333W
V333Q
V333D
V333P
F499A
F499W
F499Q
F499D
F499P
I500A
I500W
I500Q
I500D
I500P
M512A
M512W
M512Q
M512D
M512P
L652A
L652W
L652Q
L652D
L652P
V653A
V653W
V653Q
V653D
V653P
L720A
L720W
L720Q
L720D
L720P
V757A
V757W
V757Q
V757D
V757P
I966A
I966W
I966Q
I966D
I966P
is in library after recombination
fitness at 30 °C
fitness at 42 °C
hydrophobicity with mutation
1 2 3 mutation 4
0
purL (b2557, P15254)
1295
high fitness
reduced fitness
low fitness
beta-sheets
alpha-helices
turns
no data / not in library
is in library after recombination
binding sites
active sites
bold verified ts mutant
-3.7 hydrophobicity (Kyte & Doolittle, 1982, J. Mol., 157, 105-132) 3.7

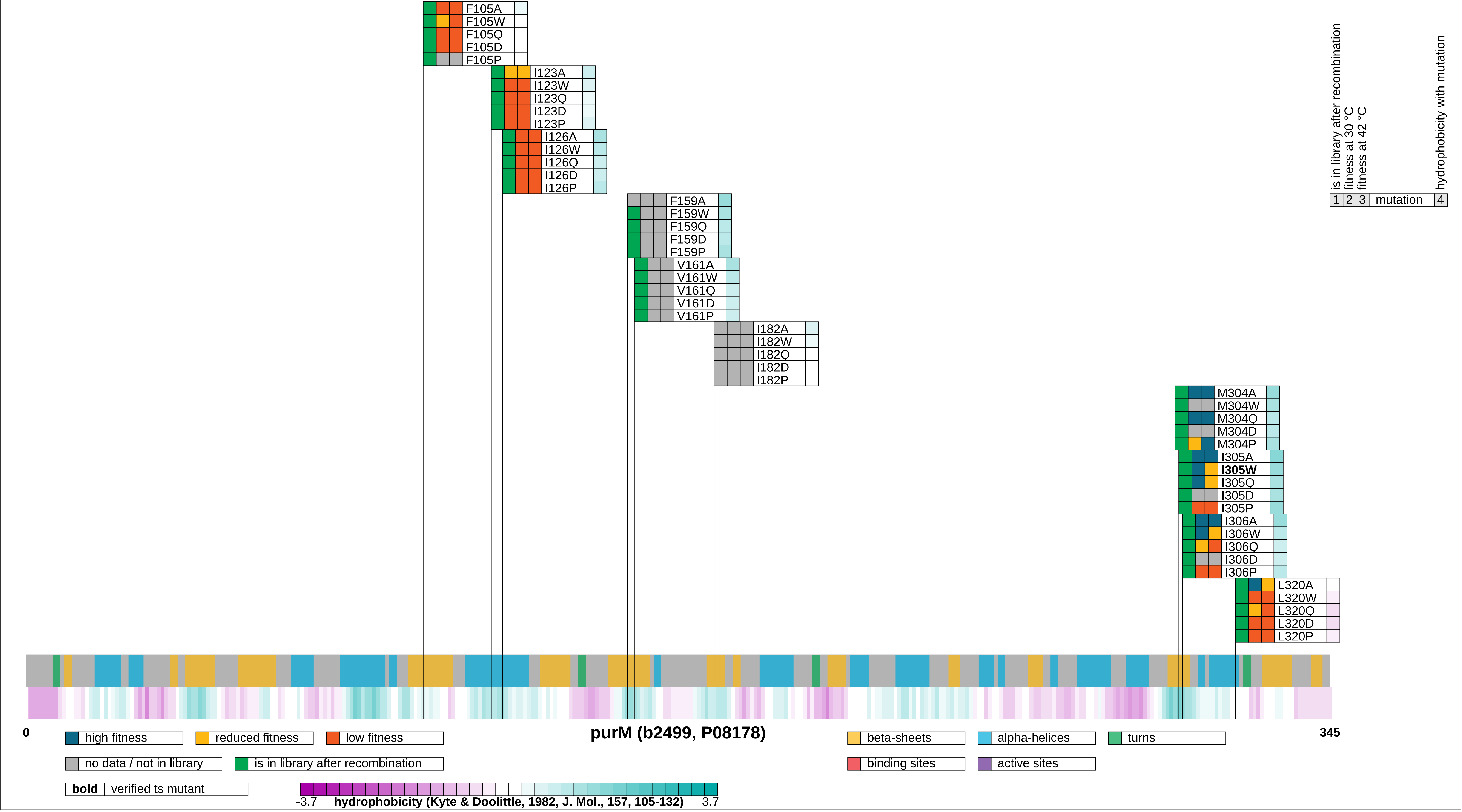
F105A
F105W
F105Q
F105D
F105P
I123A
I123W
I123Q
I123D
I123P
I126A
I126W
I126Q
I126D
I126P
F159A
F159W
F159Q
F159D
F159P
V161A
V161W
V161Q
V161D
V161P
I182A
I182W
I182Q
I182D
I182P
M304A
M304W
M304Q
M304D
M304P
I305A
I305W
I305Q
I305D
I305P
I306A
I306W
I306Q
I306D
I306P
L320A
L320W
L320Q
L320D
L320P
is in library after recombination
fitness at 30 °C
fitness at 42 °C
hydrophobicity with mutation
1 2 3 mutation 4
0
345
purM (b2499, P08178)
high fitness
reduced fitness
low fitness
no data / not in library
is in library after recombination
bold verified ts mutant
beta-sheets
alpha-helices
turns
binding sites
active sites
-3.7
hydrophobicity (Kyte & Doolittle, 1982, J. Mol., 157, 105-132)
3.7

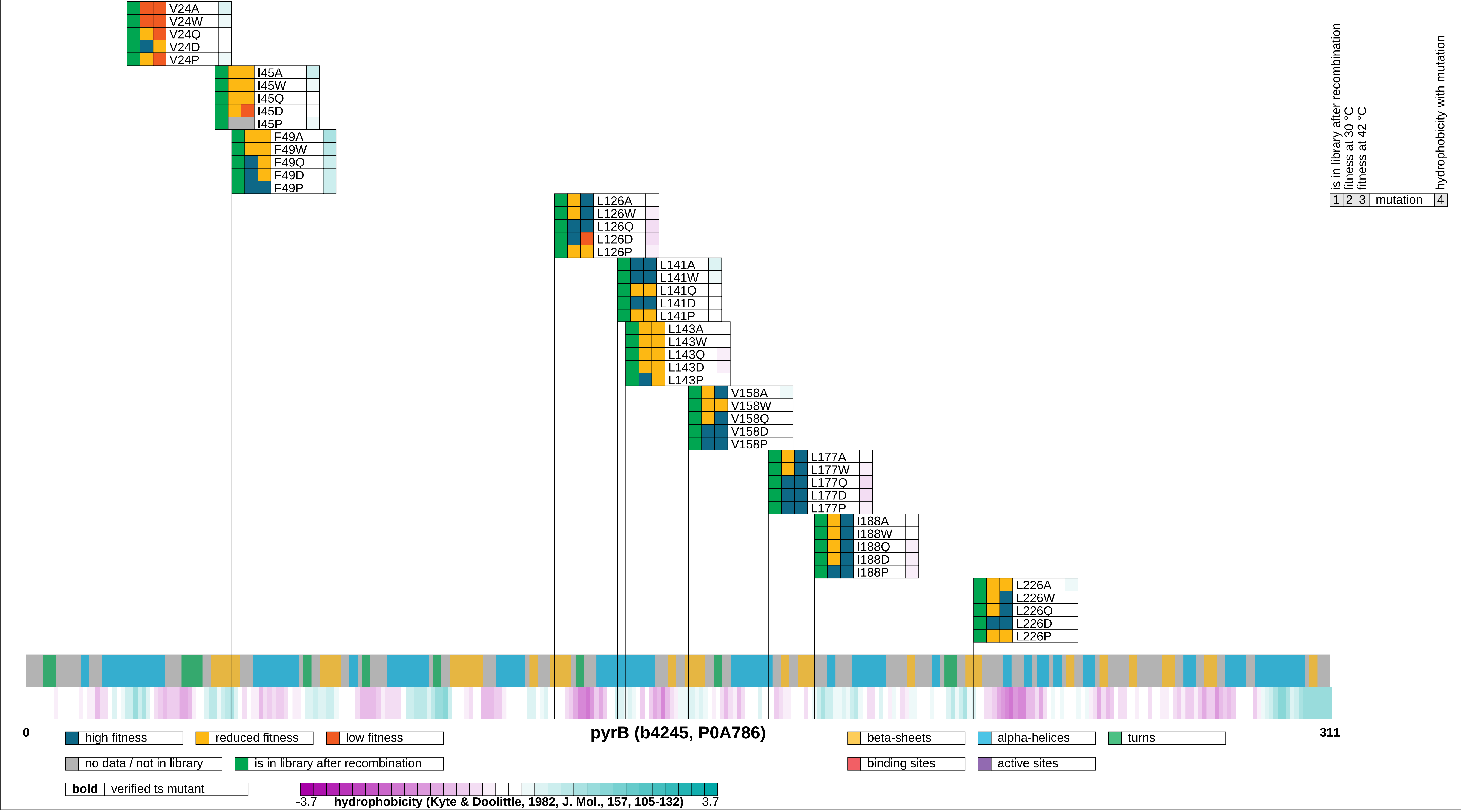

V24A
V24W
V24Q
V24D
V24P
I45A
I45W
I45Q
I45D
I45P
F49A
F49W
F49Q
F49D
F49P
L126A
L126W
L126Q
L126D
L126P
L141A
L141W
L141Q
L141D
L141P
L143A
L143W
L143Q
L143D
L143P
V158A
V158W
V158Q
V158D
V158P
L177A
L177W
L177Q
L177D
L177P
I188A
I188W
I188Q
I188D
I188P
L226A
L226W
L226Q
L226D
L226P
is in library after recombination
fitness at 30 °C
fitness at 42 °C
hydrophobicity with mutation
1 2 3 mutation 4
0
311
pyrB (b4245, P0A786)
high fitness
reduced fitness
low fitness
beta-sheets
alpha-helices
turns
no data / not in library
is in library after recombination
binding sites
active sites
bold verified ts mutant
-3.7
hydrophobicity (Kyte & Doolittle, 1982, J. Mol., 157, 105-132)
3.7

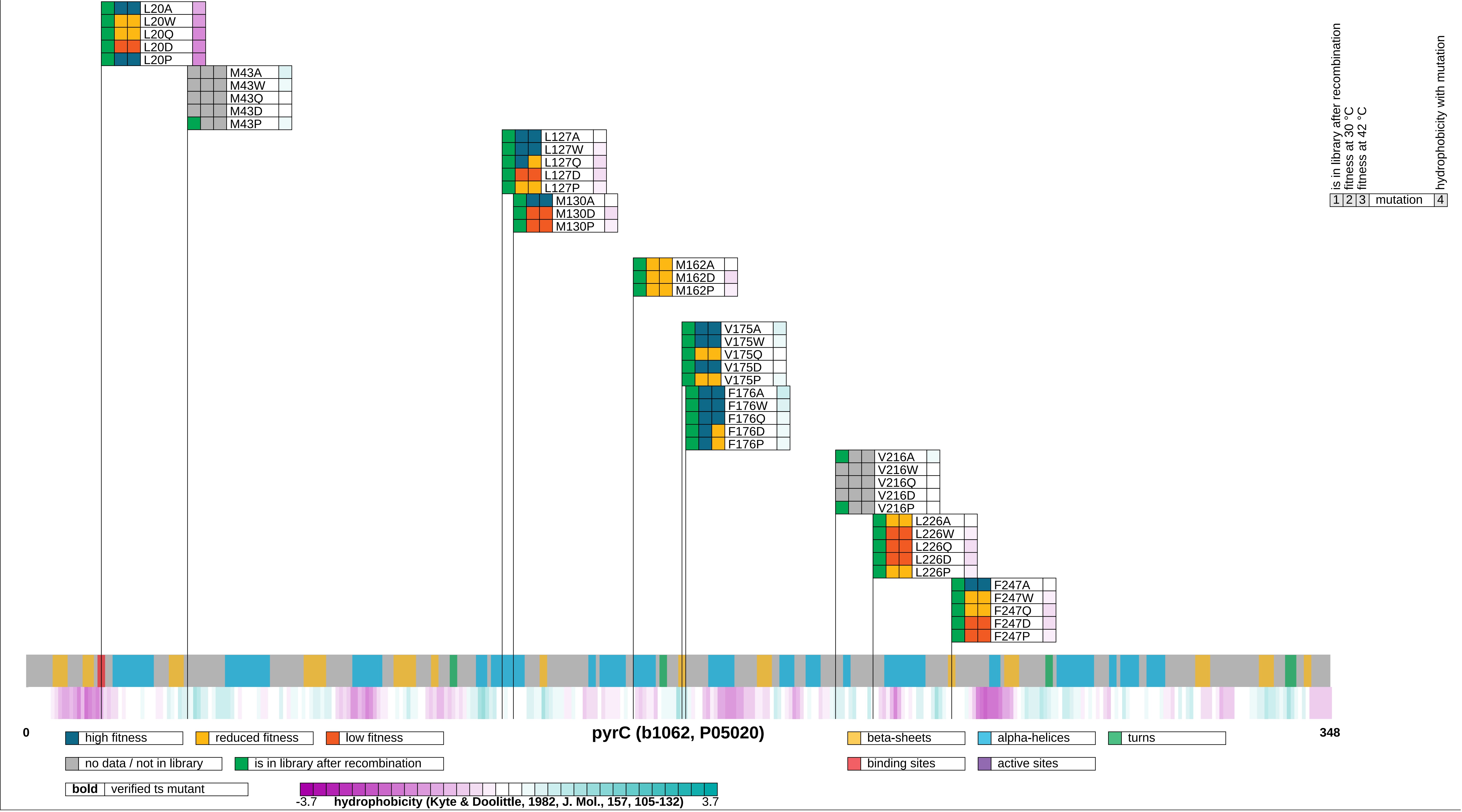
L20A
L20W
L20Q
L20D
L20P
M43A
M43W
M43Q
M43D
M43P
L127A
L127W
L127Q
L127D
L127P
M130A
M130D
M130P
M162A
M162D
M162P
V175A
V175W
V175Q
V175D
V175P
F176A
F176W
F176Q
F176D
F176P
V216A
V216W
V216Q
V216D
V216P
L226A
L226W
L226Q
L226D
L226P
F247A
F247W
F247Q
F247D
F247P
is in library after recombination
fitness at 30 °C
fitness at 42 °C
hydrophobicity with mutation
1 2 3 mutation 4
0
pyrC (b1062, P05020)
348
high fitness
reduced fitness
low fitness
beta-sheets
alpha-helices
turns
no data / not in library
is in library after recombination
binding sites
active sites
bold verified ts mutant
-3.7 hydrophobicity (Kyte & Doolittle, 1982, J. Mol., 157, 105-132) 3.7

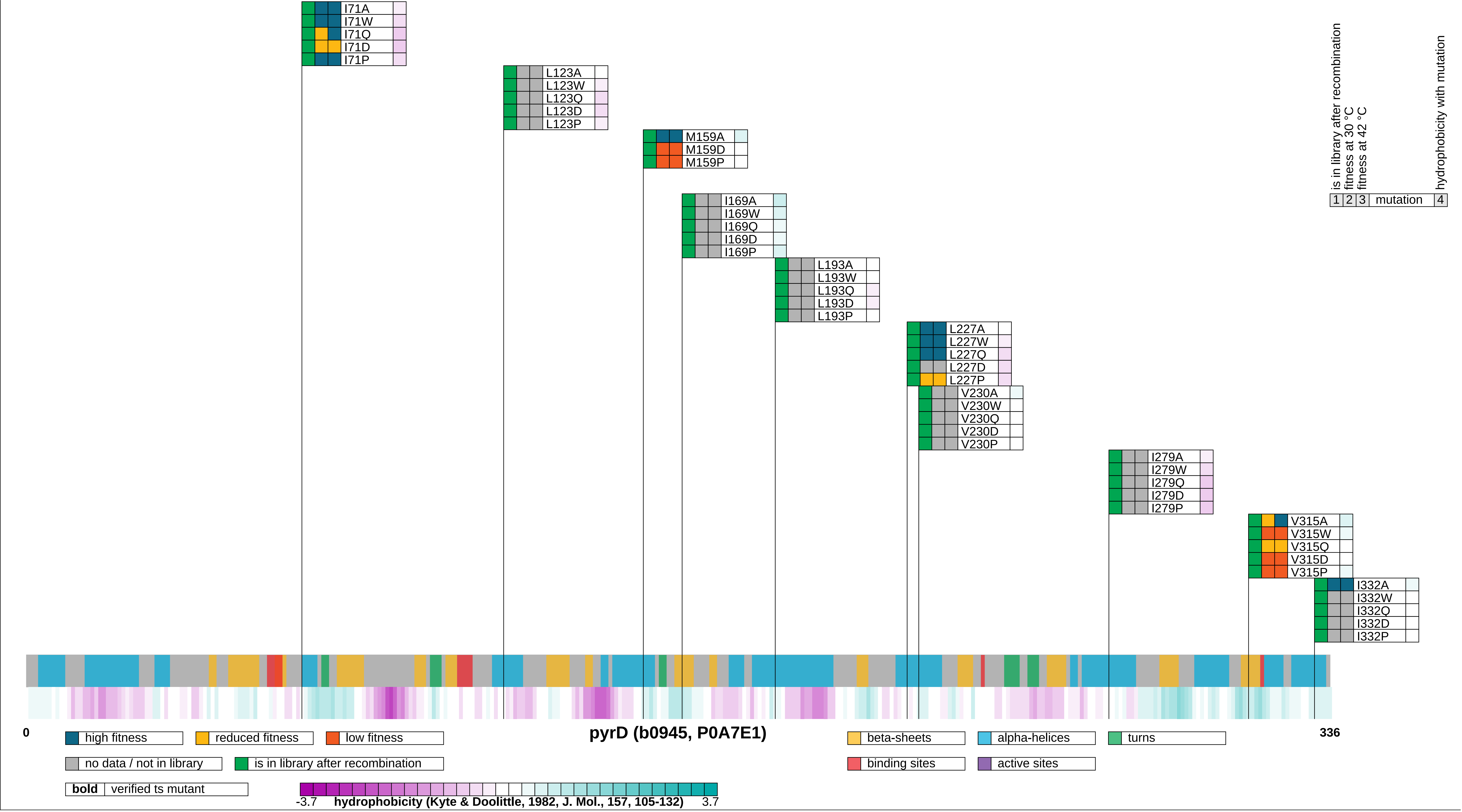
I71A
I71W
I71Q
I71D
I71P
L123A
L123W
L123Q
L123D
L123P
M159A
M159D
M159P
I169A
I169W
I169Q
I169D
I169P
L193A
L193W
L193Q
L193D
L193P
L227A
L227W
L227Q
L227D
L227P
V230A
V230W
V230Q
V230D
V230P
I279A
I279W
I279Q
I279D
I279P
V315A
V315W
V315Q
V315D
V315P
I332A
I332W
I332Q
I332D
I332P
is in library after recombination
fitness at 30 °C
fitness at 42 °C
hydrophobicity with mutation
1 2 3 mutation 4
0
pyrD (b0945, P0A7E1)
336
high fitness
reduced fitness
low fitness
beta-sheets
alpha-helices
turns
no data / not in library
is in library after recombination
binding sites
active sites
bold verified ts mutant
-3.7
hydrophobicity (Kyte & Doolittle, 1982, J. Mol., 157, 105-132)
3.7

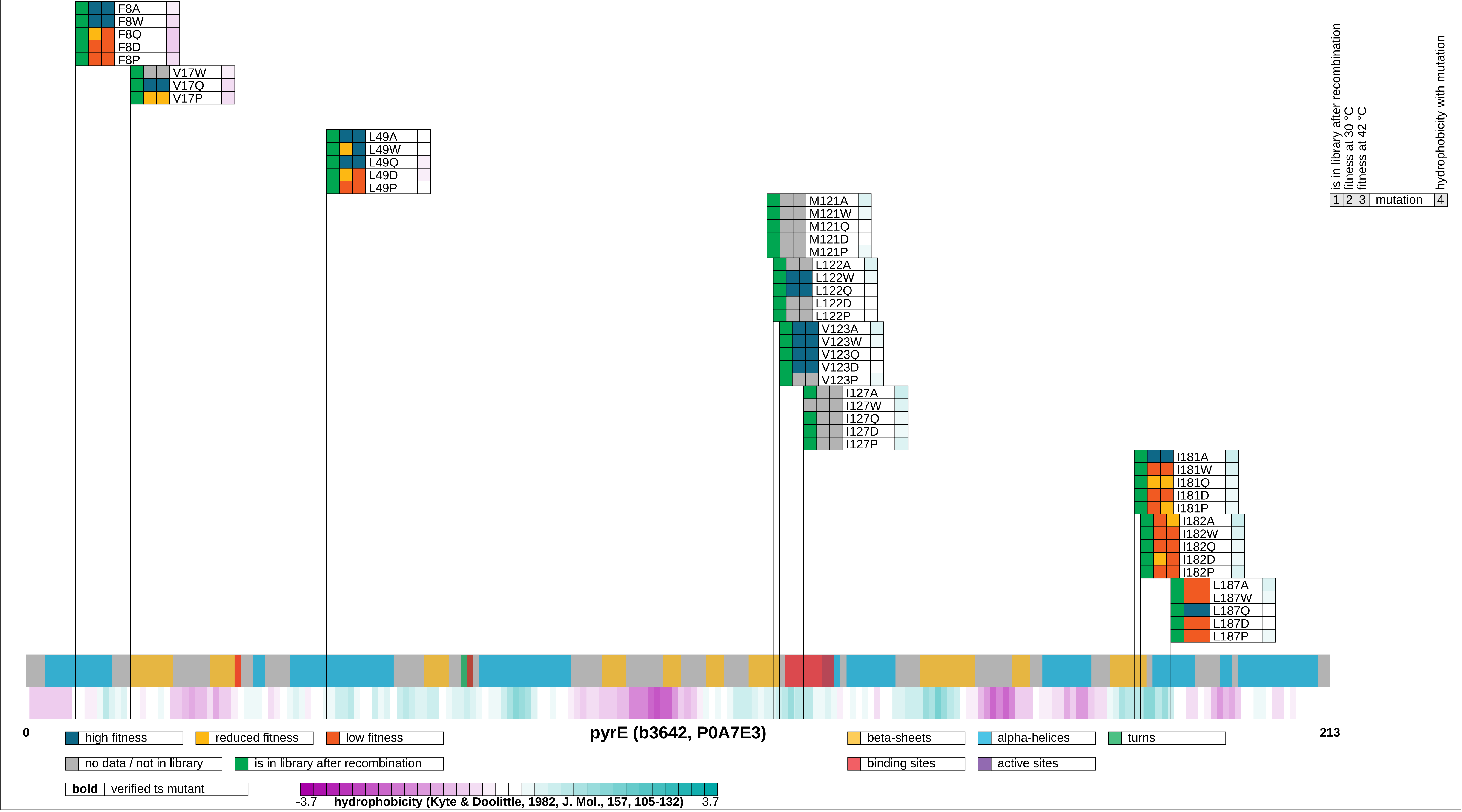
F8A
F8W
F8Q
F8D
F8P
V17W
V17Q
V17P
L49A
L49W
L49Q
L49D
L49P
M121A
M121W
M121Q
M121D
M121P
L122A
L122W
L122Q
L122D
L122P
V123A
V123W
V123Q
V123D
V123P
I127A
I127W
I127Q
I127D
I127P
I181A
I181W
I181Q
I181D
I181P
I182A
I182W
I182Q
I182D
I182P
L187A
L187W
L187Q
L187D
L187P
is in library after recombination
fitness at 30 °C
fitness at 42 °C
hydrophobicity with mutation
1 2 3 mutation 4
0
pyrE (b3642, P0A7E3)
213
high fitness
reduced fitness
low fitness
beta-sheets
alpha-helices
turns
no data / not in library
is in library after recombination
binding sites
active sites
bold verified ts mutant
-3.7 hydrophobicity (Kyte & Doolittle, 1982, J. Mol., 157, 105-132) 3.7

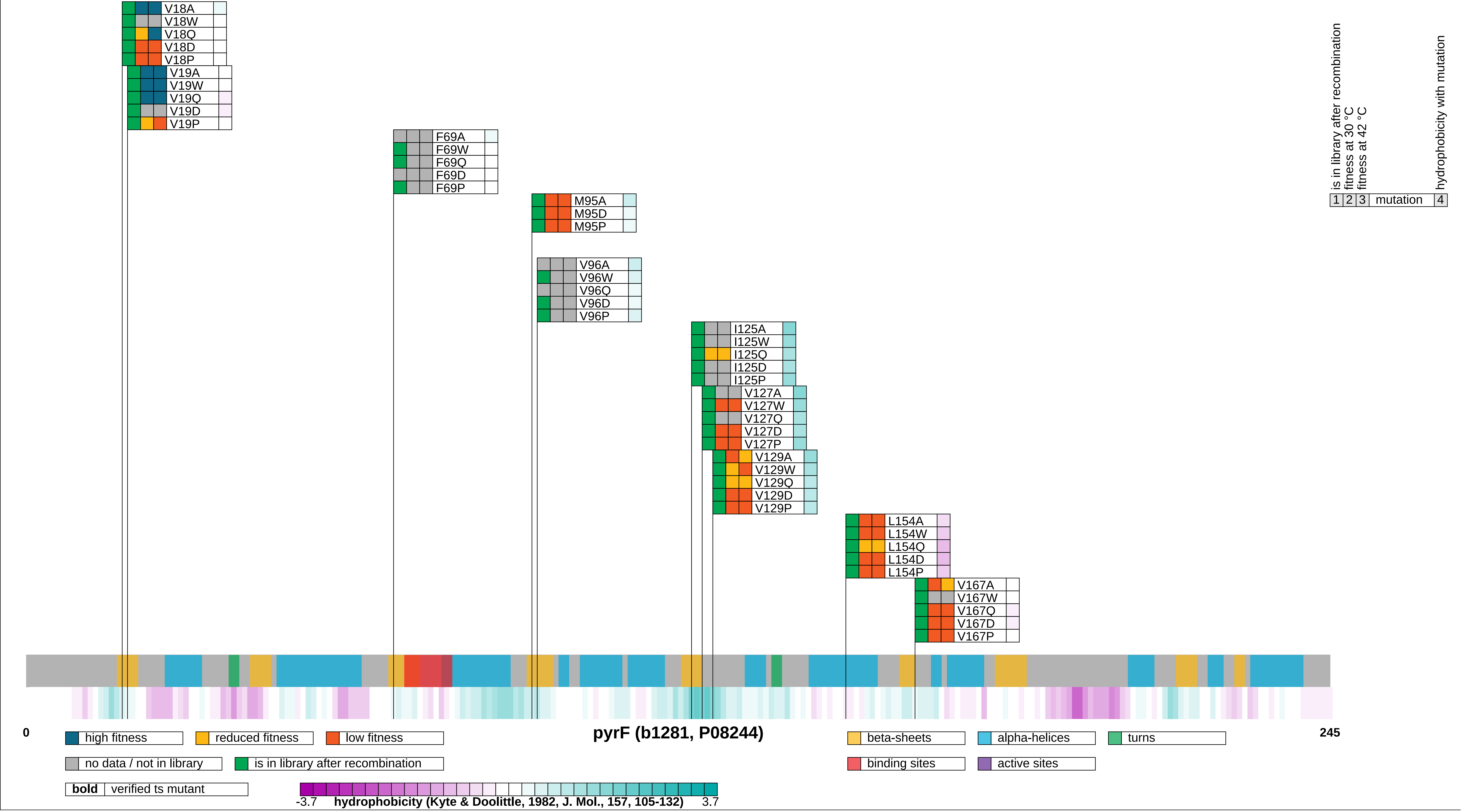
V18A
V18W
V18Q
V18D
V18P
V19A
V19W
V19Q
V19D
V19P
F69A
F69W
F69Q
F69D
F69P
M95A
M95D
M95P
V96A
V96W
V96Q
V96D
V96P
I125A
I125W
I125Q
I125D
I125P
V127A
V127W
V127Q
V127D
V127P
V129A
V129W
V129Q
V129D
V129P
L154A
L154W
L154Q
L154D
L154P
V167A
V167W
V167Q
V167D
V167P
is in library after recombination
fitness at 30 °C
fitness at 42 °C
hydrophobicity with mutation
1 2 3 mutation 4
0
pyrF (b1281, P08244)
245
high fitness
reduced fitness
low fitness
no data / not in library
is in library after recombination
bold verified ts mutant
beta-sheets
alpha-helices
turns
binding sites
active sites
-3.7 hydrophobicity (Kyte & Doolittle, 1982, J. Mol., 157, 105-132) 3.7

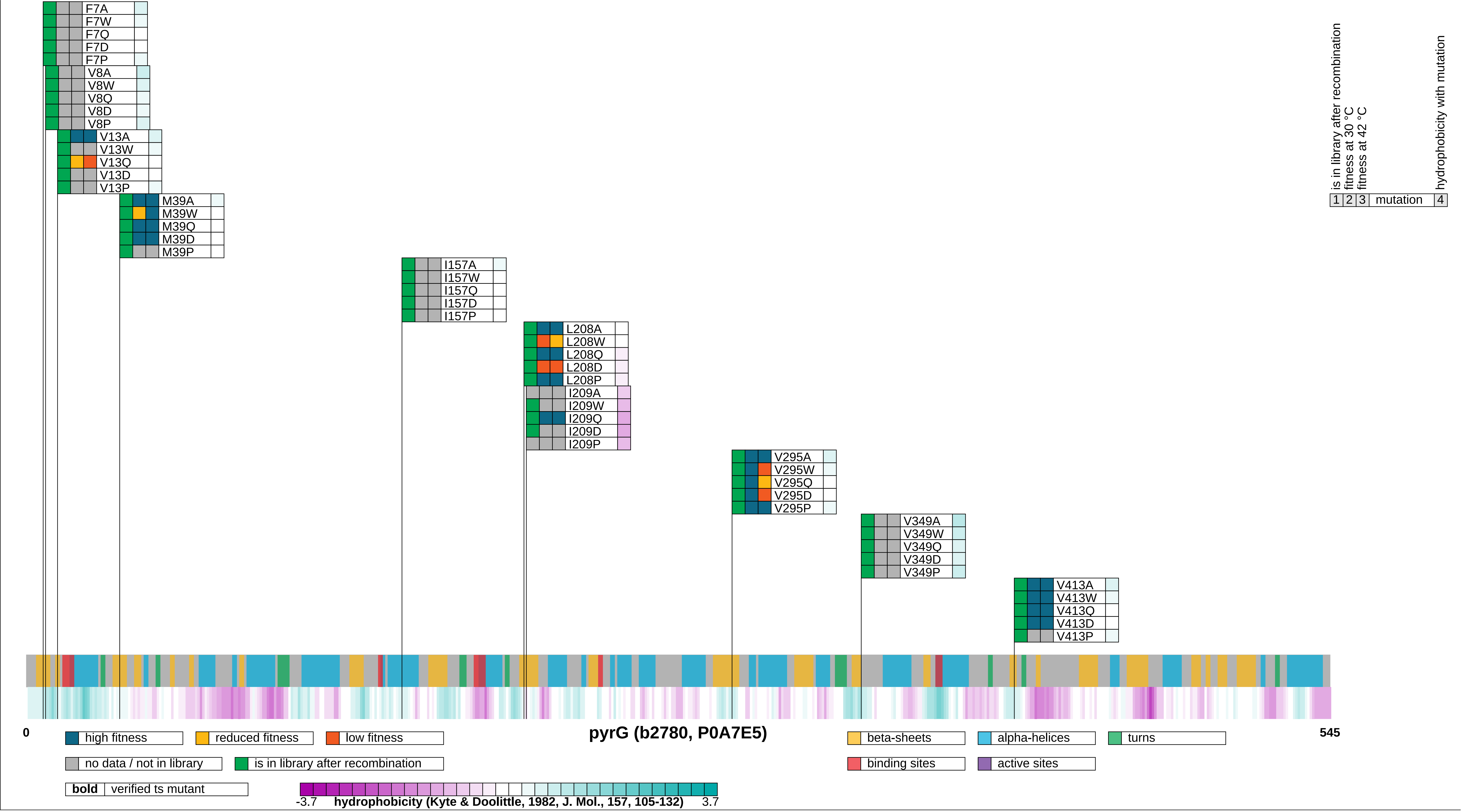
F7A
F7W
F7Q
F7D
F7P
V8A
V8W
V8Q
V8D
V8P
V13A
V13W
V13Q
V13D
V13P
M39A
M39W
M39Q
M39D
M39P
I157A
I157W
I157Q
I157D
I157P
L208A
L208W
L208Q
L208D
L208P
I209A
I209W
I209Q
I209D
I209P
V295A
V295W
V295Q
V295D
V295P
V349A
V349W
V349Q
V349D
V349P
V413A
V413W
V413Q
V413D
V413P
is in library after recombination
fitness at 30 °C
fitness at 42 °C
hydrophobicity with mutation
1
2
3
mutation
4
0
pyrG (b2780, P0A7E5)
545
high fitness
reduced fitness
low fitness
beta-sheets
alpha-helices
turns
no data / not in library
is in library after recombination
binding sites
active sites
bold
verified ts mutant
-3.7
hydrophobicity (Kyte & Doolittle, 1982, J. Mol., 157, 105-132)
3.7

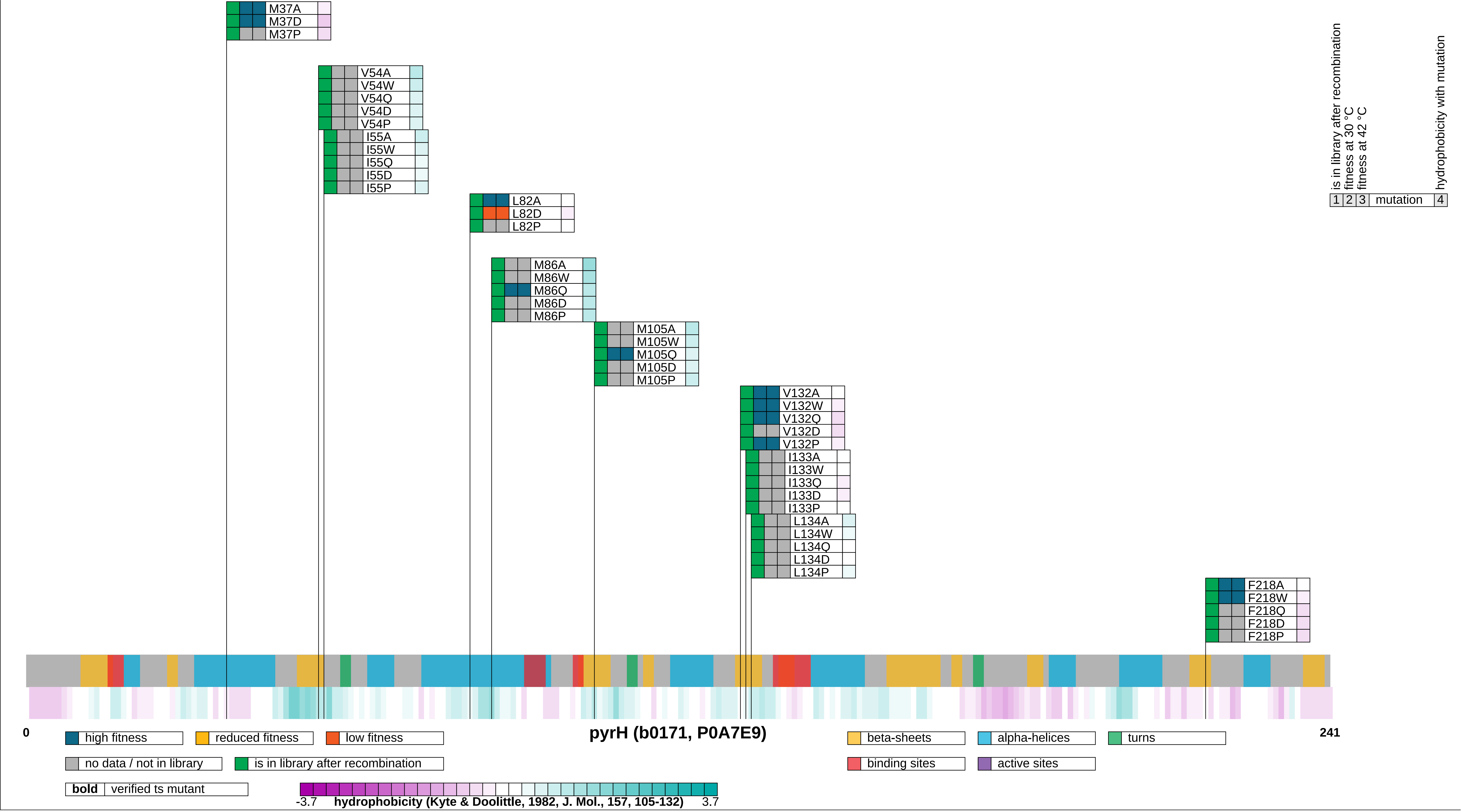

M37A
M37D
M37P
V54A
V54W
V54Q
V54D
V54P
I55A
I55W
I55Q
I55D
I55P
L82A
L82D
L82P
M86A
M86W
M86Q
M86D
M86P
M105A
M105W
M105Q
M105D
M105P
V132A
V132W
V132Q
V132D
V132P
I133A
I133W
I133Q
I133D
I133P
L134A
L134W
L134Q
L134D
L134P
F218A
F218W
F218Q
F218D
F218P
is in library after recombination
fitness at 30 °C
fitness at 42 °C
hydrophobicity with mutation
1 2 3 mutation 4
0
pyrH (b0171, P0A7E9)
241
high fitness
reduced fitness
low fitness
beta-sheets
alpha-helices
turns
no data / not in library
is in library after recombination
binding sites
active sites
bold verified ts mutant
-3.7
hydrophobicity (Kyte & Doolittle, 1982, J. Mol., 157, 105-132)
3.7

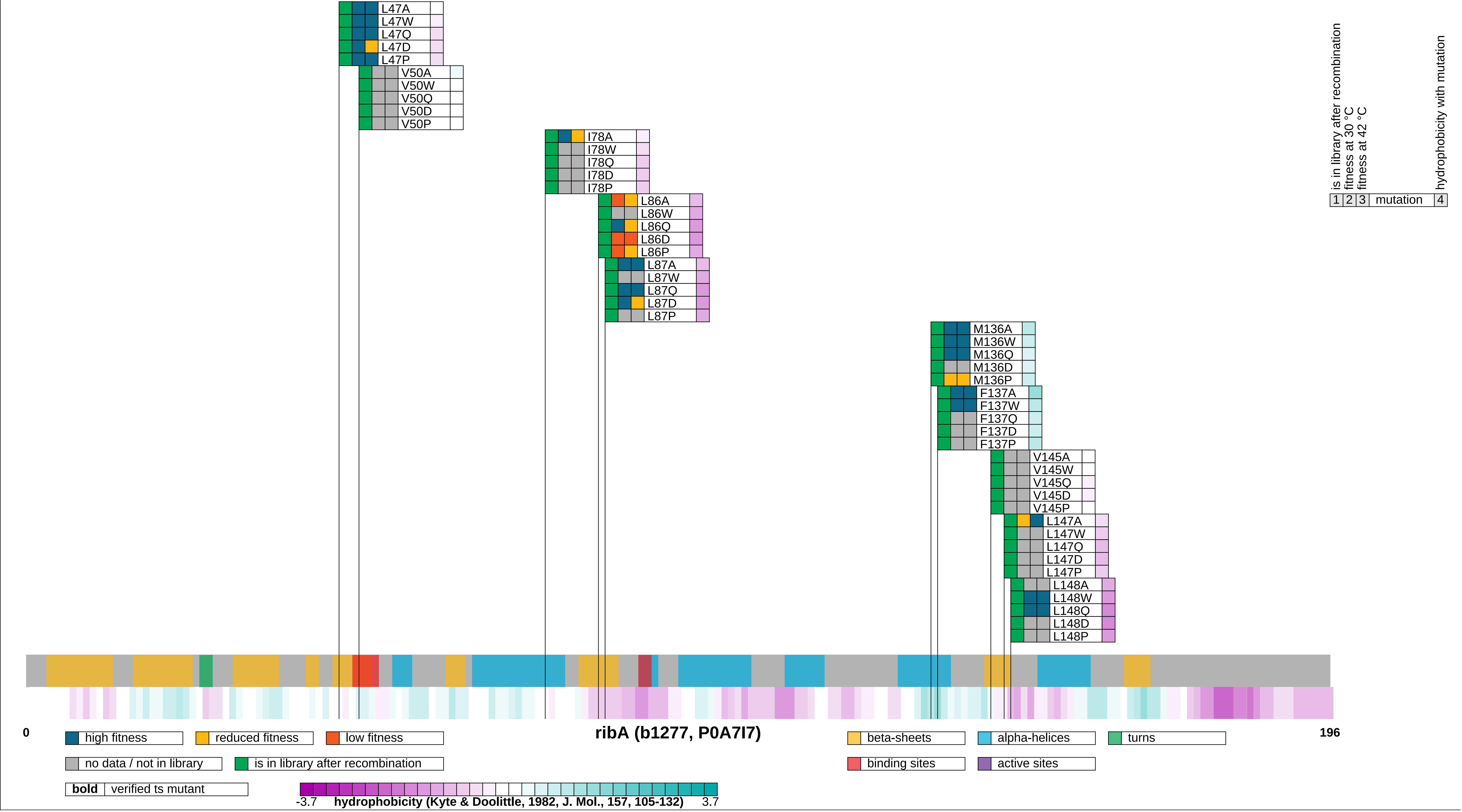

L47A
L47W
L47Q
L47D
L47P
V50A
V50W
V50Q
V50D
V50P
I78A
I78W
I78Q
I78D
I78P
L86A
L86W
L86Q
L86D
L86P
L87A
L87W
L87Q
L87D
L87P
M136A
M136W
M136Q
M136D
M136P
F137A
F137W
F137Q
F137D
F137P
V145A
V145W
V145Q
V145D
V145P
L147A
L147W
L147Q
L147D
L147P
L148A
L148W
L148Q
L148D
L148P
is in library after recombination
fitness at 30 °C
fitness at 42 °C
hydrophobicity with mutation
1 2 3 mutation 4
0
196
ribA (b1277, P0A7I7)
high fitness
reduced fitness
low fitness
no data / not in library
is in library after recombination
bold verified ts mutant
beta-sheets
alpha-helices
turns
binding sites
active sites
-3.7
hydrophobicity (Kyte & Doolittle, 1982, J. Mol., 157, 105-132)
3.7

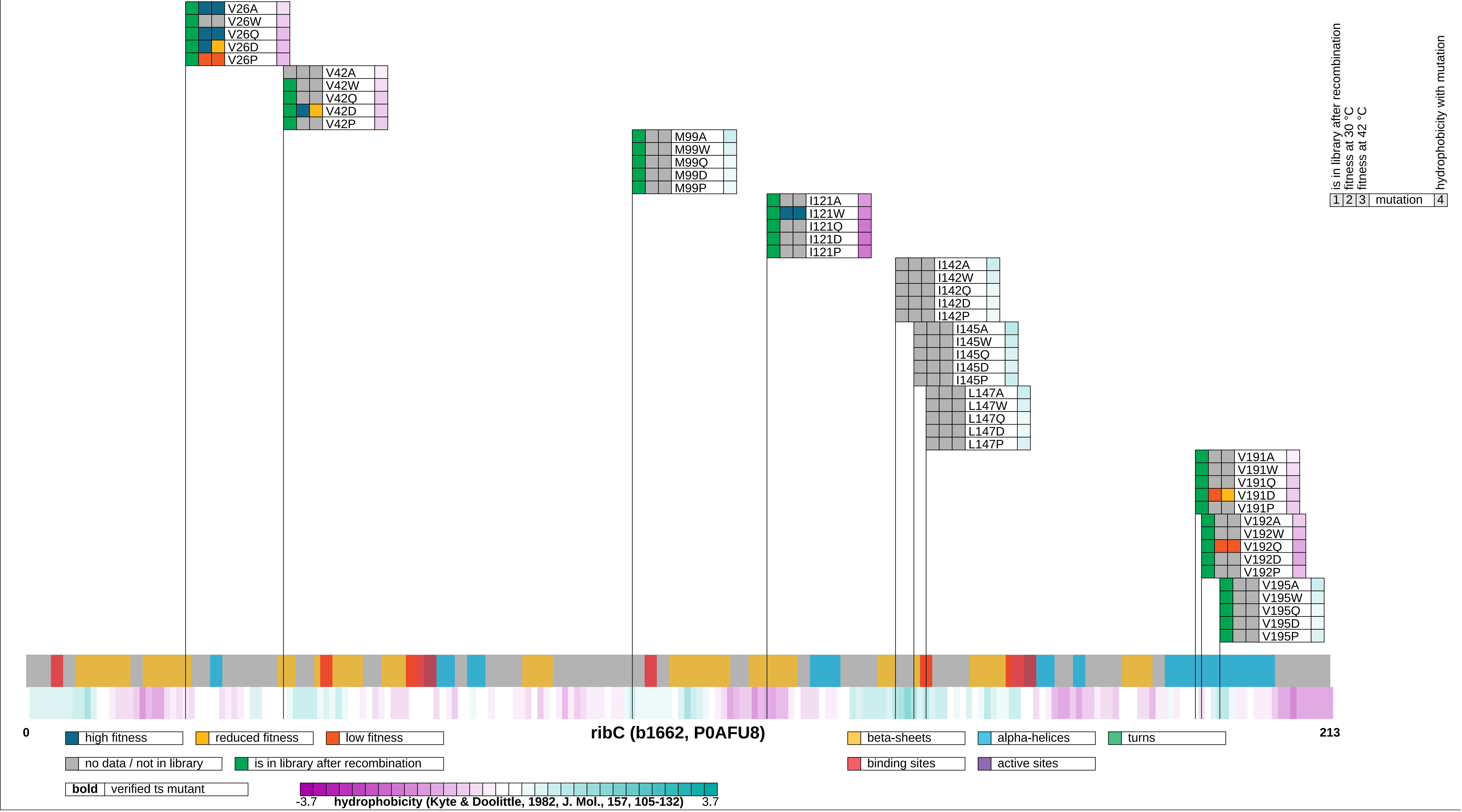

V26A
V26W
V26Q
V26D
V26P
V42A
V42W
V42Q
V42D
V42P
M99A
M99W
M99Q
M99D
M99P
I121A
I121W
I121Q
I121D
I121P
I142A
I142W
I142Q
I142D
I142P
I145A
I145W
I145Q
I145D
I145P
L147A
L147W
L147Q
L147D
L147P
V191A
V191W
V191Q
V191D
V191P
V192A
V192W
V192Q
V192D
V192P
V195A
V195W
V195Q
V195D
V195P
is in library after recombination
fitness at 30 °C
fitness at 42 °C
hydrophobicity with mutation
1 2 3 mutation 4
0
ribC (b1662, P0AFU8)
213
high fitness
reduced fitness
low fitness
beta-sheets
alpha-helices
turns
no data / not in library
is in library after recombination
binding sites
active sites
bold verified ts mutant
-3.7
hydrophobicity (Kyte & Doolittle, 1982, J. Mol., 157, 105-132)
3.7

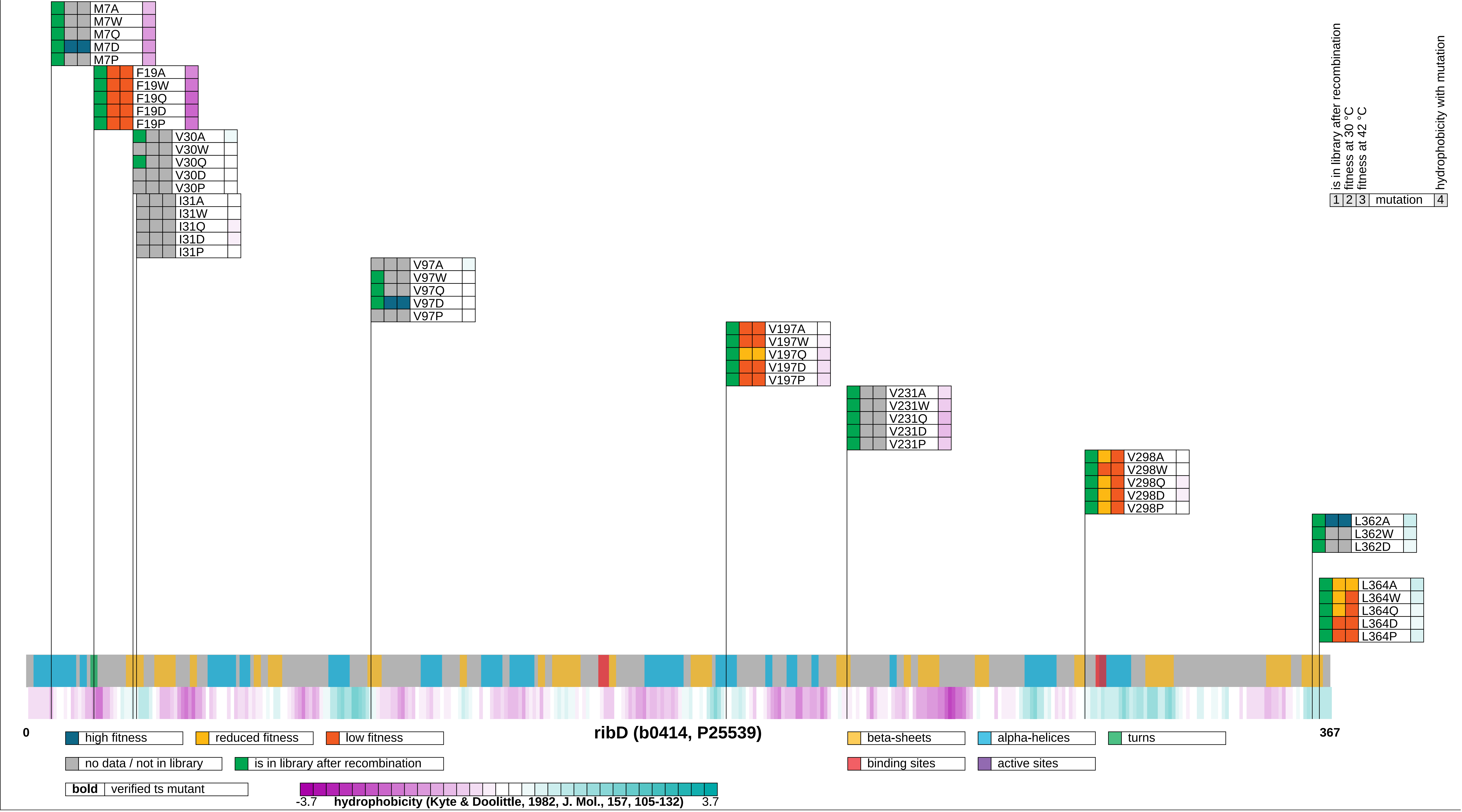

M7A
M7W
M7Q
M7D
M7P
F19A
F19W
F19Q
F19D
F19P
V30A
V30W
V30Q
V30D
V30P
I31A
I31W
I31Q
I31D
I31P
V97A
V97W
V97Q
V97D
V97P
V197A
V197W
V197Q
V197D
V197P
V231A
V231W
V231Q
V231D
V231P
V298A
V298W
V298Q
V298D
V298P
L362A
L362W
L362D
L364A
L364W
L364Q
L364D
L364P
is in library after recombination
fitness at 30 °C
fitness at 42 °C
hydrophobicity with mutation
1 2 3 mutation 4
0
ribD (b0414, P25539)
367
high fitness
reduced fitness
low fitness
beta-sheets
alpha-helices
turns
no data / not in library
is in library after recombination
binding sites
active sites
bold verified ts mutant
-3.7 hydrophobicity (Kyte & Doolittle, 1982, J. Mol., 157, 105-132) 3.7

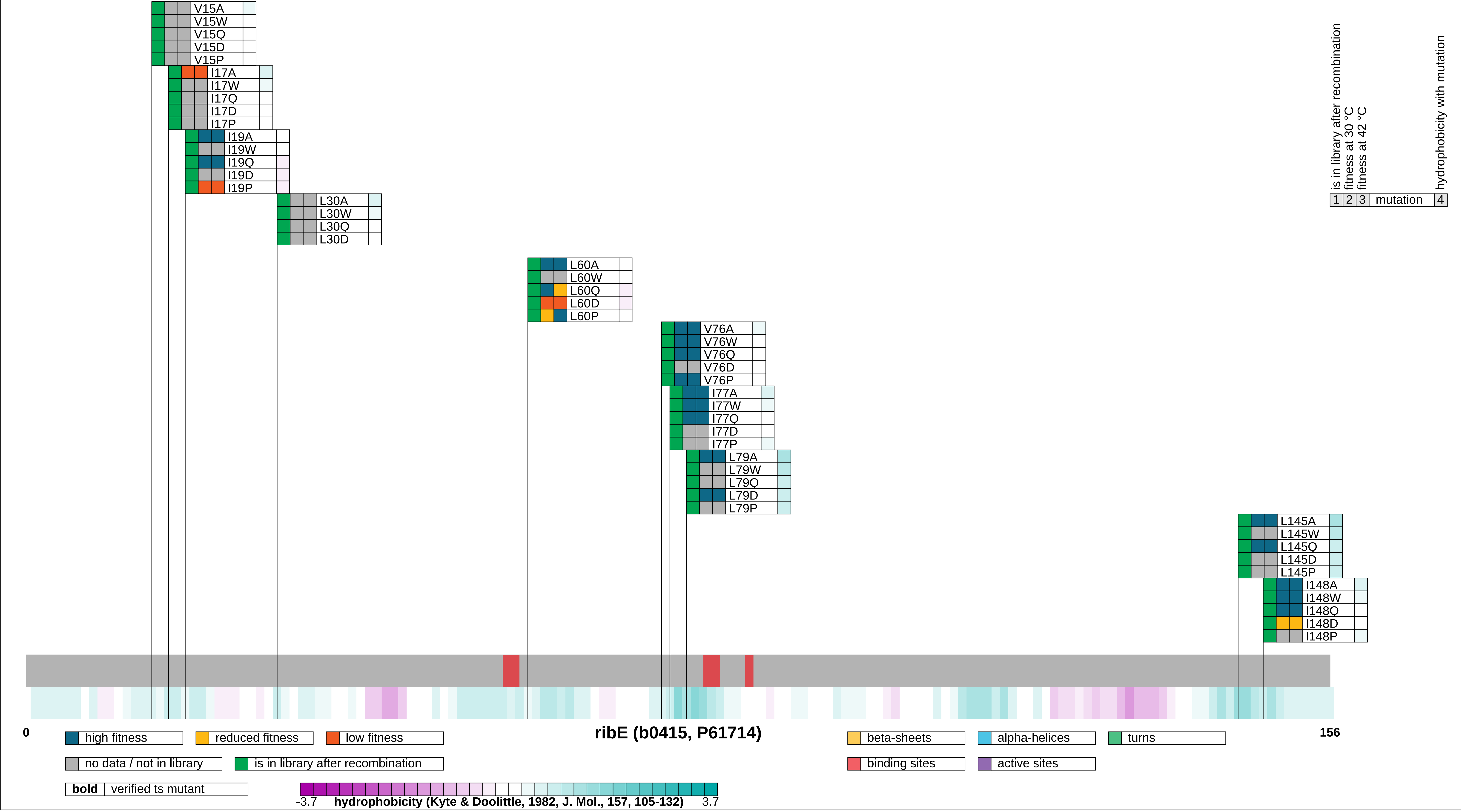
V15A
V15W
V15Q
V15D
V15P
I17A
I17W
I17Q
I17D
I17P
I19A
I19W
I19Q
I19D
I19P
L30A
L30W
L30Q
L30D
L60A
L60W
L60Q
L60D
L60P
V76A
V76W
V76Q
V76D
V76P
I77A
I77W
I77Q
I77D
I77P
L79A
L79W
L79Q
L79D
L79P
L145A
L145W
L145Q
L145D
L145P
I148A
I148W
I148Q
I148D
I148P
is in library after recombination
fitness at 30 °C
fitness at 42 °C
hydrophobicity with mutation
1 2 3 mutation 4
0
ribE (b0415, P61714)
156
high fitness
reduced fitness
low fitness
no data / not in library
is in library after recombination
bold verified ts mutant
beta-sheets
alpha-helices
turns
binding sites
active sites
-3.7 hydrophobicity (Kyte & Doolittle, 1982, J. Mol., 157, 105-132) 3.7

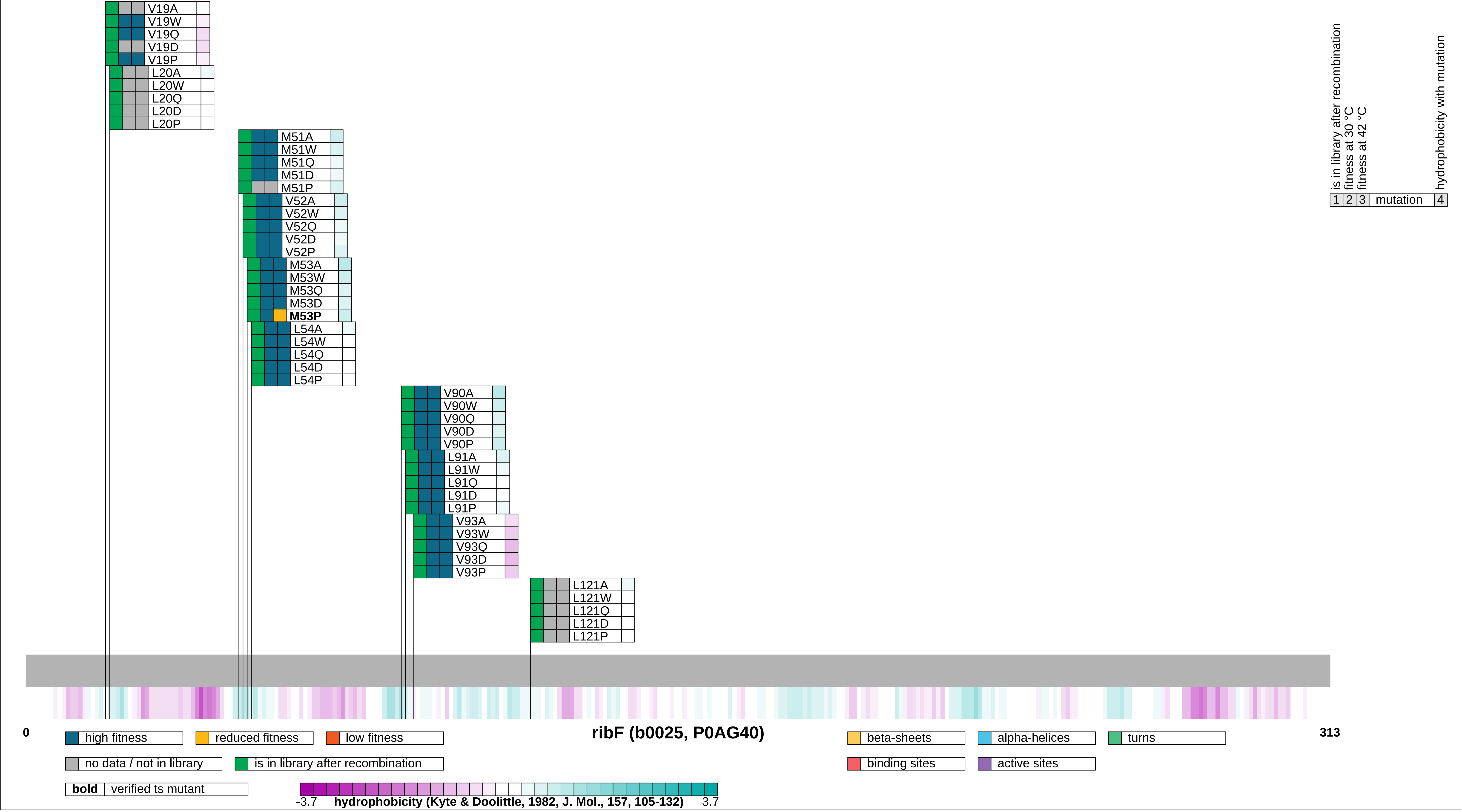
V19A
V19W
V19Q
V19D
V19P
L20A
L20W
L20Q
L20D
L20P
M51A
M51W
M51Q
M51D
M51P
V52A
V52W
V52Q
V52D
V52P
M53A
M53W
M53Q
M53D
M53P
L54A
L54W
L54Q
L54D
L54P
V90A
V90W
V90Q
V90D
V90P
L91A
L91W
L91Q
L91D
L91P
V93A
V93W
V93Q
V93D
V93P
L121A
L121W
L121Q
L121D
L121P
is in library after recombination
fitness at 30 °C
fitness at 42 °C
hydrophobicity with mutation
1 2 3 mutation 4
0
ribF (b0025, P0AG40)
313
high fitness
reduced fitness
low fitness
beta-sheets
alpha-helices
turns
no data / not in library
is in library after recombination
binding sites
active sites
bold verified ts mutant
-3.7 hydrophobicity (Kyte & Doolittle, 1982, J. Mol., 157, 105-132) 3.7

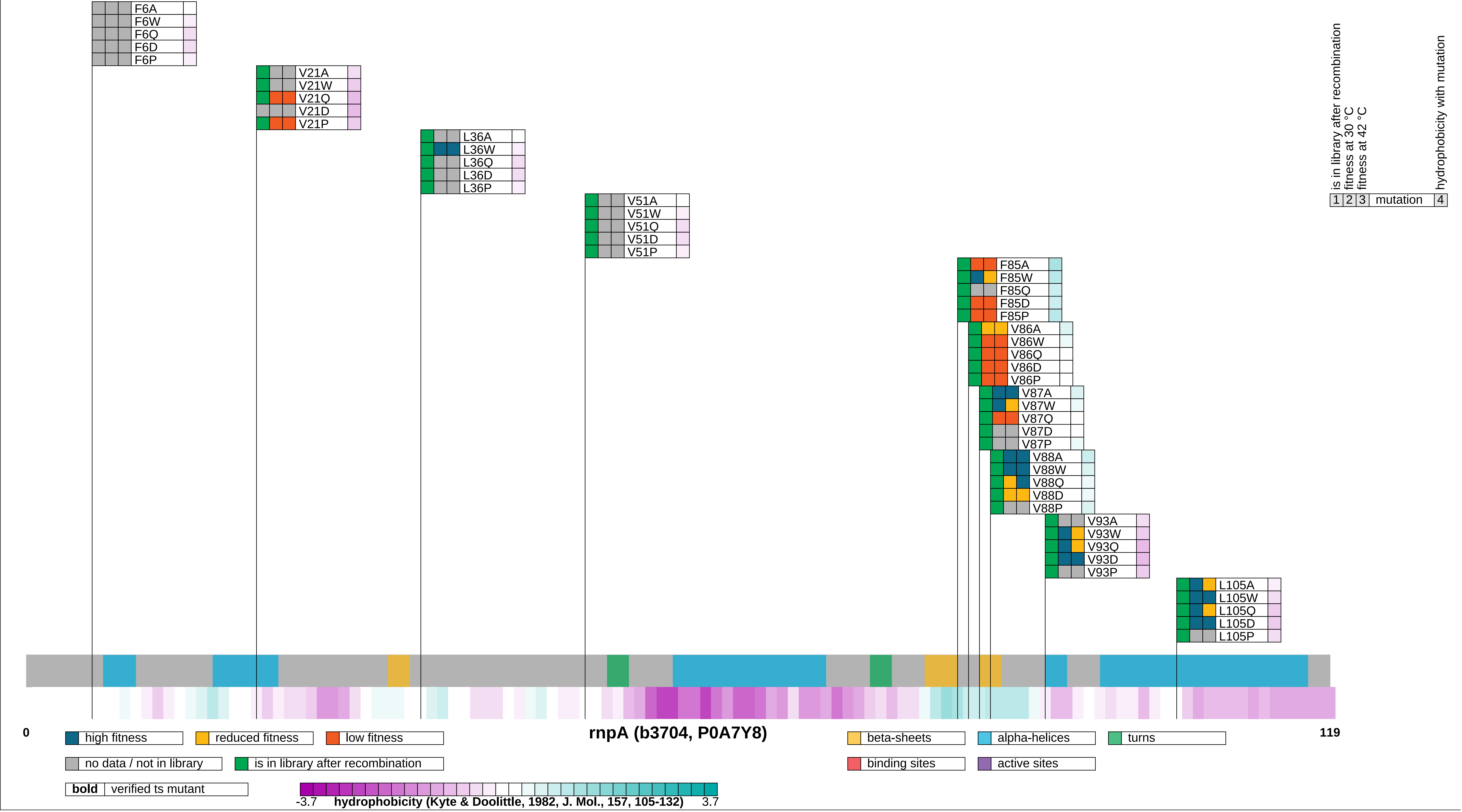
F6A
F6W
F6Q
F6D
F6P
V21A
V21W
V21Q
V21D
V21P
L36A
L36W
L36Q
L36D
L36P
V51A
V51W
V51Q
V51D
V51P
F85A
F85W
F85Q
F85D
F85P
V86A
V86W
V86Q
V86D
V86P
V87A
V87W
V87Q
V87D
V87P
V88A
V88W
V88Q
V88D
V88P
V93A
V93W
V93Q
V93D
V93P
L105A
L105W
L105Q
L105D
L105P
is in library after recombination
fitness at 30 °C
fitness at 42 °C
hydrophobicity with mutation
1 2 3 mutation 4
0
rnpA (b3704, P0A7Y8)
119
high fitness
reduced fitness
low fitness
beta-sheets
alpha-helices
turns
no data / not in library
is in library after recombination
binding sites
active sites
bold verified ts mutant
-3.7 hydrophobicity (Kyte & Doolittle, 1982, J. Mol., 157, 105-132) 3.7

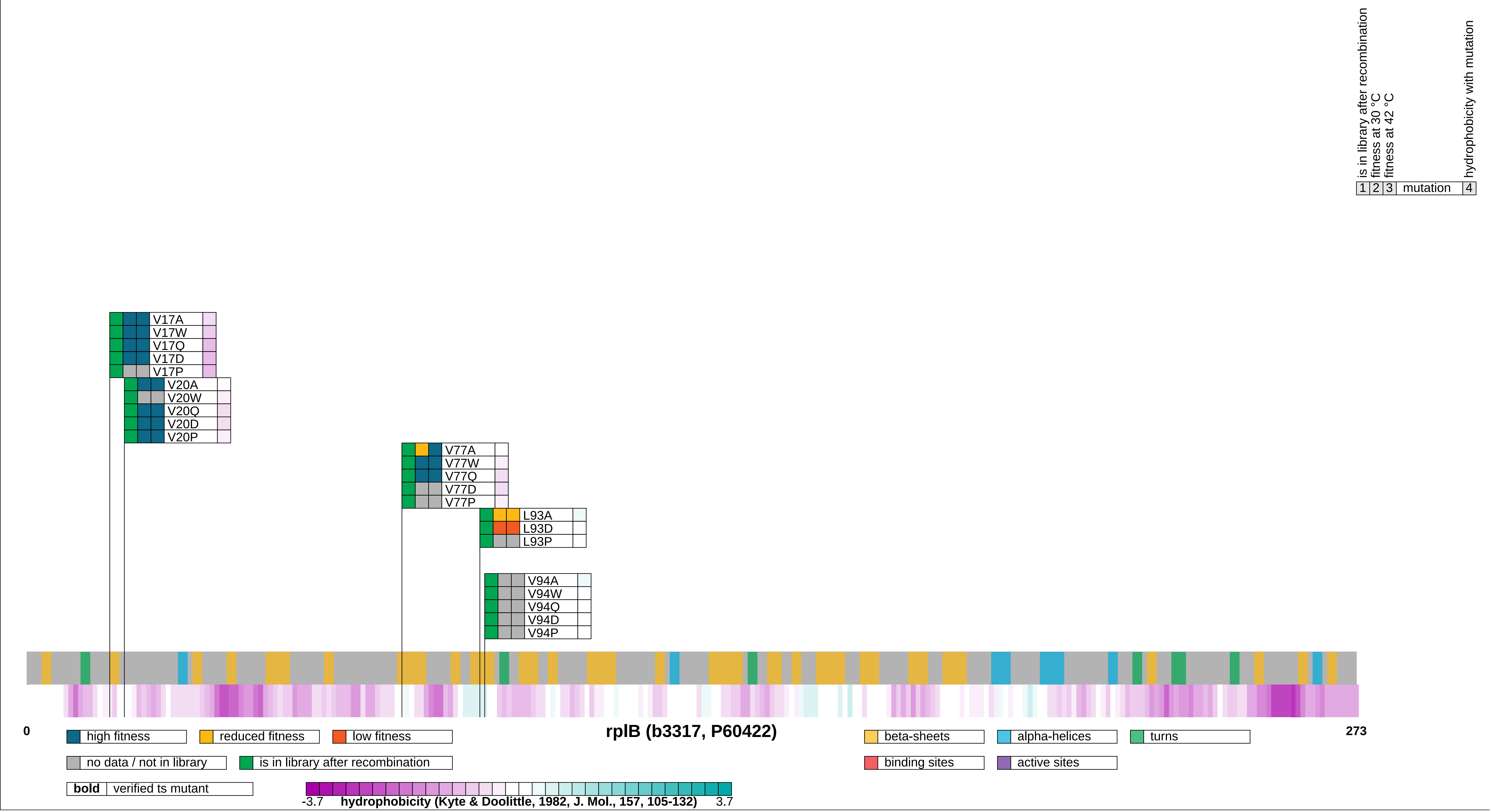

is in library after recombination
fitness at 30 °C
fitness at 42 °C
hydrophobicity with mutation
1
2
3
mutation
4
V17A
V17W
V17Q
V17D
V17P
V20A
V20W
V20Q
V20D
V20P
V77A
V77W
V77Q
V77D
V77P
L93A
L93D
L93P
V94A
V94W
V94Q
V94D
V94P
0
rplB (b3317, P60422)
273
high fitness
reduced fitness
low fitness
beta-sheets
alpha-helices
turns
no data / not in library
is in library after recombination
binding sites
active sites
bold
verified ts mutant
-3.7
hydrophobicity (Kyte & Doolittle, 1982, J. Mol., 157, 105-132)
3.7

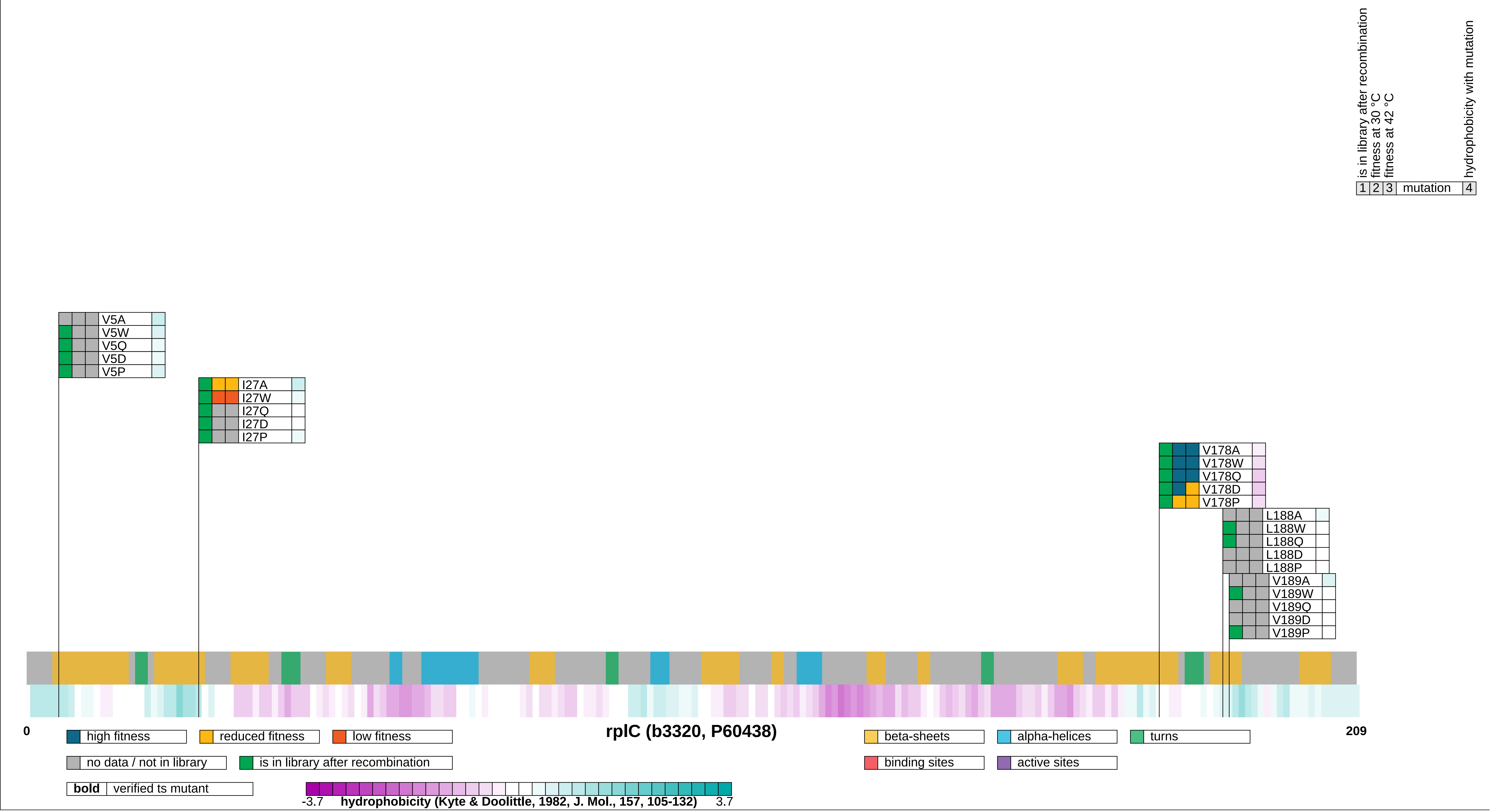

is in library after recombination
fitness at 30 °C
fitness at 42 °C
hydrophobicity with mutation
1 2 3 mutation 4
V5A
V5W
V5Q
V5D
V5P
I27A
I27W
I27Q
I27D
I27P
V178A
V178W
V178Q
V178D
V178P
L188A
L188W
L188Q
L188D
L188P
V189A
V189W
V189Q
V189D
V189P
0
rplC (b3320, P60438)
209
high fitness
reduced fitness
low fitness
beta-sheets
alpha-helices
turns
no data / not in library
is in library after recombination
binding sites
active sites
bold verified ts mutant
-3.7
hydrophobicity (Kyte & Doolittle, 1982, J. Mol., 157, 105-132)
3.7

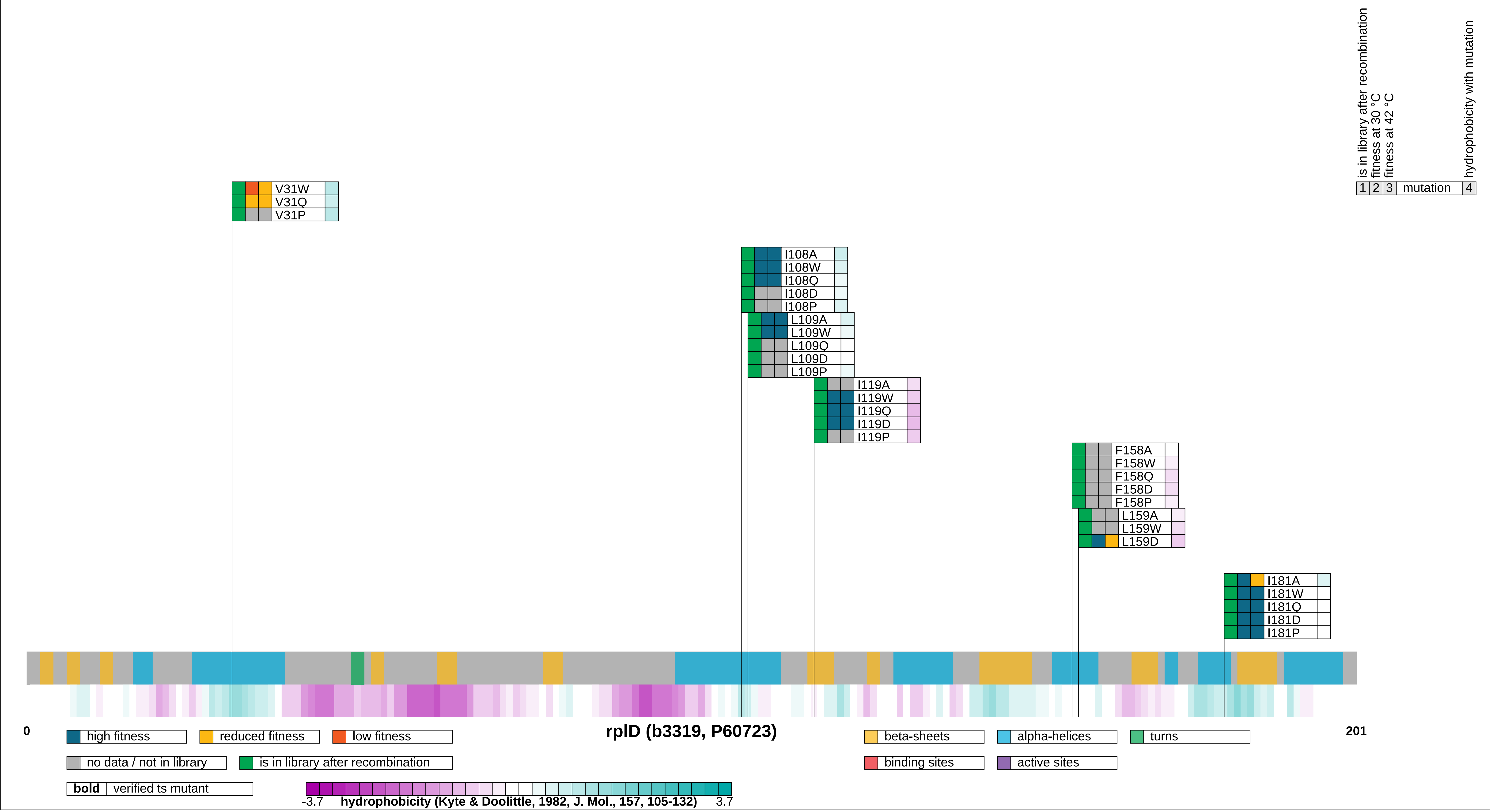

is in library after recombination
fitness at 30 °C
fitness at 42 °C
hydrophobicity with mutation
1 2 3 mutation 4
V31W
V31Q
V31P
I108A
I108W
I108Q
I108D
I108P
L109A
L109W
L109Q
L109D
L109P
I119A
I119W
I119Q
I119D
I119P
F158A
F158W
F158Q
F158D
F158P
L159A
L159W
L159D
I181A
I181W
I181Q
I181D
I181P
0
rplD (b3319, P60723)
201
high fitness
reduced fitness
low fitness
beta-sheets
alpha-helices
turns
no data / not in library
is in library after recombination
binding sites
active sites
bold verified ts mutant
-3.7 hydrophobicity (Kyte & Doolittle, 1982, J. Mol., 157, 105-132) 3.7

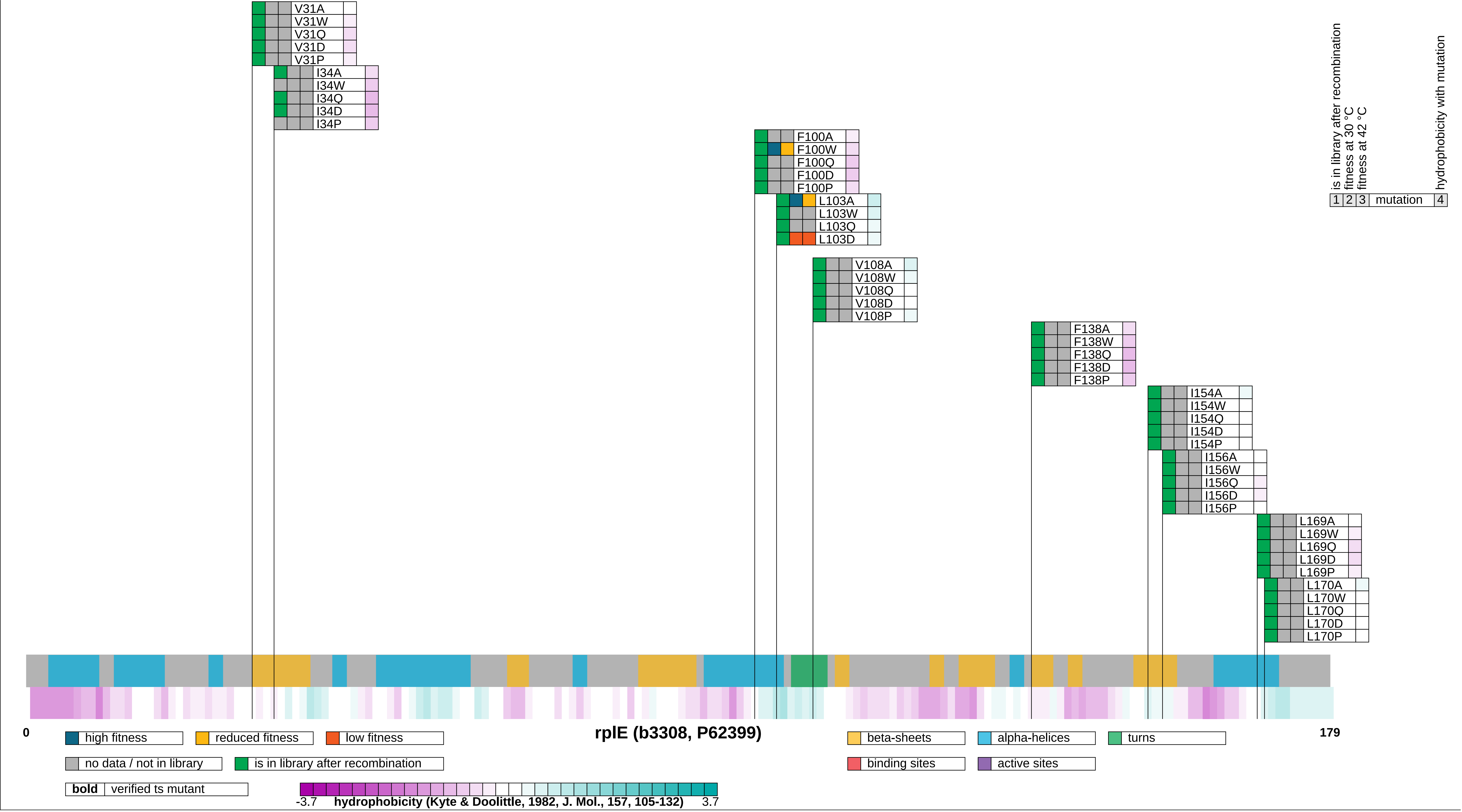

V31A
V31W
V31Q
V31D
V31P
I34A
I34W
I34Q
I34D
I34P
F100A
F100W
F100Q
F100D
F100P
L103A
L103W
L103Q
L103D
V108A
V108W
V108Q
V108D
V108P
F138A
F138W
F138Q
F138D
F138P
I154A
I154W
I154Q
I154D
I154P
I156A
I156W
I156Q
I156D
I156P
L169A
L169W
L169Q
L169D
L169P
L170A
L170W
L170Q
L170D
L170P
is in library after recombination
fitness at 30 °C
fitness at 42 °C
hydrophobicity with mutation
1 2 3 mutation 4
0
rplE (b3308, P62399)
179
high fitness
reduced fitness
low fitness
no data / not in library
is in library after recombination
bold verified ts mutant
beta-sheets
alpha-helices
turns
binding sites
active sites
-3.7 hydrophobicity (Kyte & Doolittle, 1982, J. Mol., 157, 105-132) 3.7

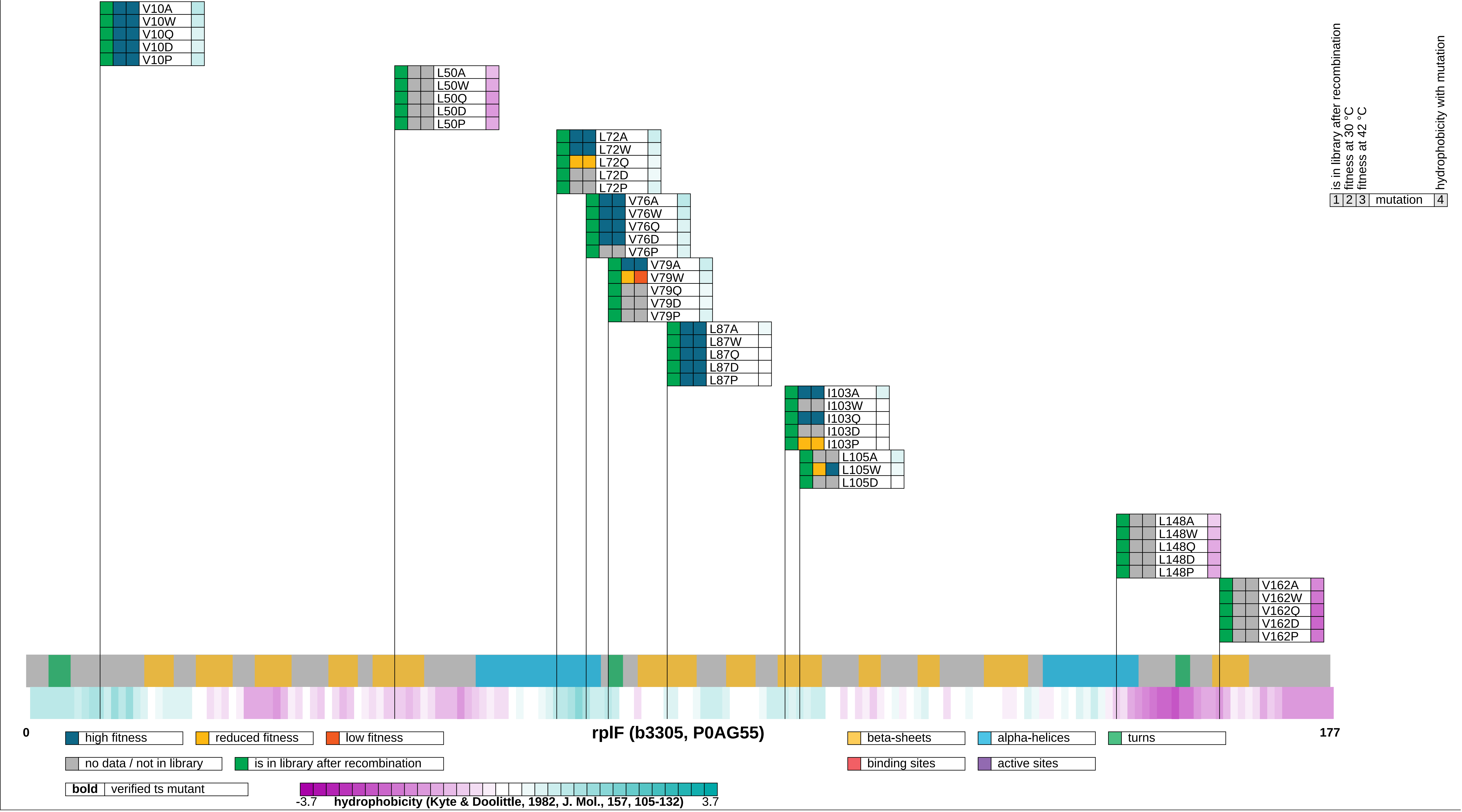

V10A
V10W
V10Q
V10D
V10P
L50A
L50W
L50Q
L50D
L50P
L72A
L72W
L72Q
L72D
L72P
V76A
V76W
V76Q
V76D
V76P
V79A
V79W
V79Q
V79D
V79P
L87A
L87W
L87Q
L87D
L87P
I103A
I103W
I103Q
I103D
I103P
L105A
L105W
L105D
L148A
L148W
L148Q
L148D
L148P
V162A
V162W
V162Q
V162D
V162P
is in library after recombination
fitness at 30 °C
fitness at 42 °C
hydrophobicity with mutation
1 2 3 mutation 4
0
rplF (b3305, P0AG55)
177
high fitness
reduced fitness
low fitness
no data / not in library
is in library after recombination
bold verified ts mutant
beta-sheets
alpha-helices
turns
binding sites
active sites
-3.7 hydrophobicity (Kyte & Doolittle, 1982, J. Mol., 157, 105-132) 3.7

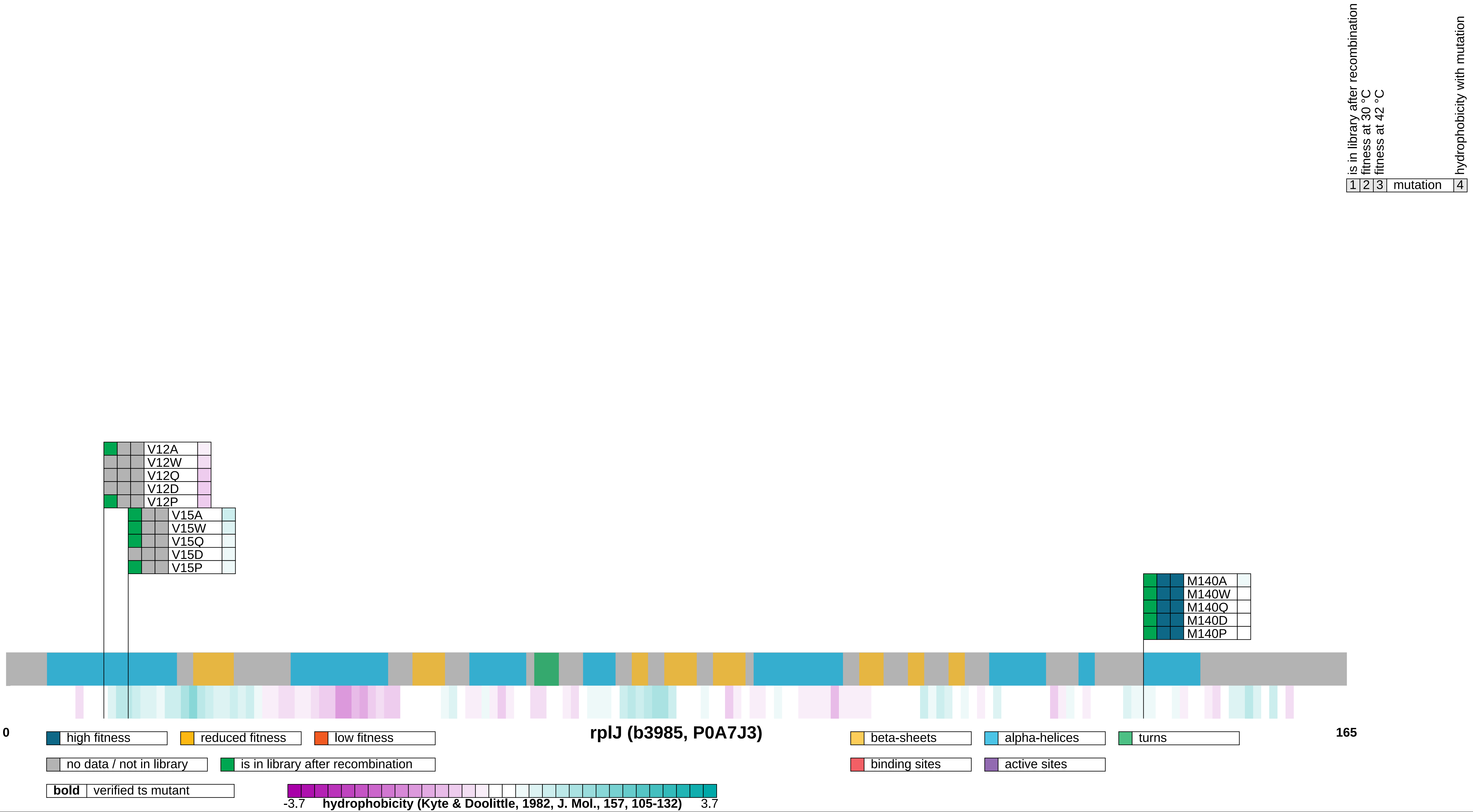
is in library after recombination
fitness at 30 °C
fitness at 42 °C
hydrophobicity with mutation
1
2
3
mutation
4
V12A
V12W
V12Q
V12D
V12P
V15A
V15W
V15Q
V15D
V15P
M140A
M140W
M140Q
M140D
M140P
0
rplJ (b3985, P0A7J3)
165
high fitness
reduced fitness
low fitness
beta-sheets
alpha-helices
turns
no data / not in library
is in library after recombination
binding sites
active sites
bold
verified ts mutant
-3.7
hydrophobicity (Kyte & Doolittle, 1982, J. Mol., 157, 105-132)
3.7

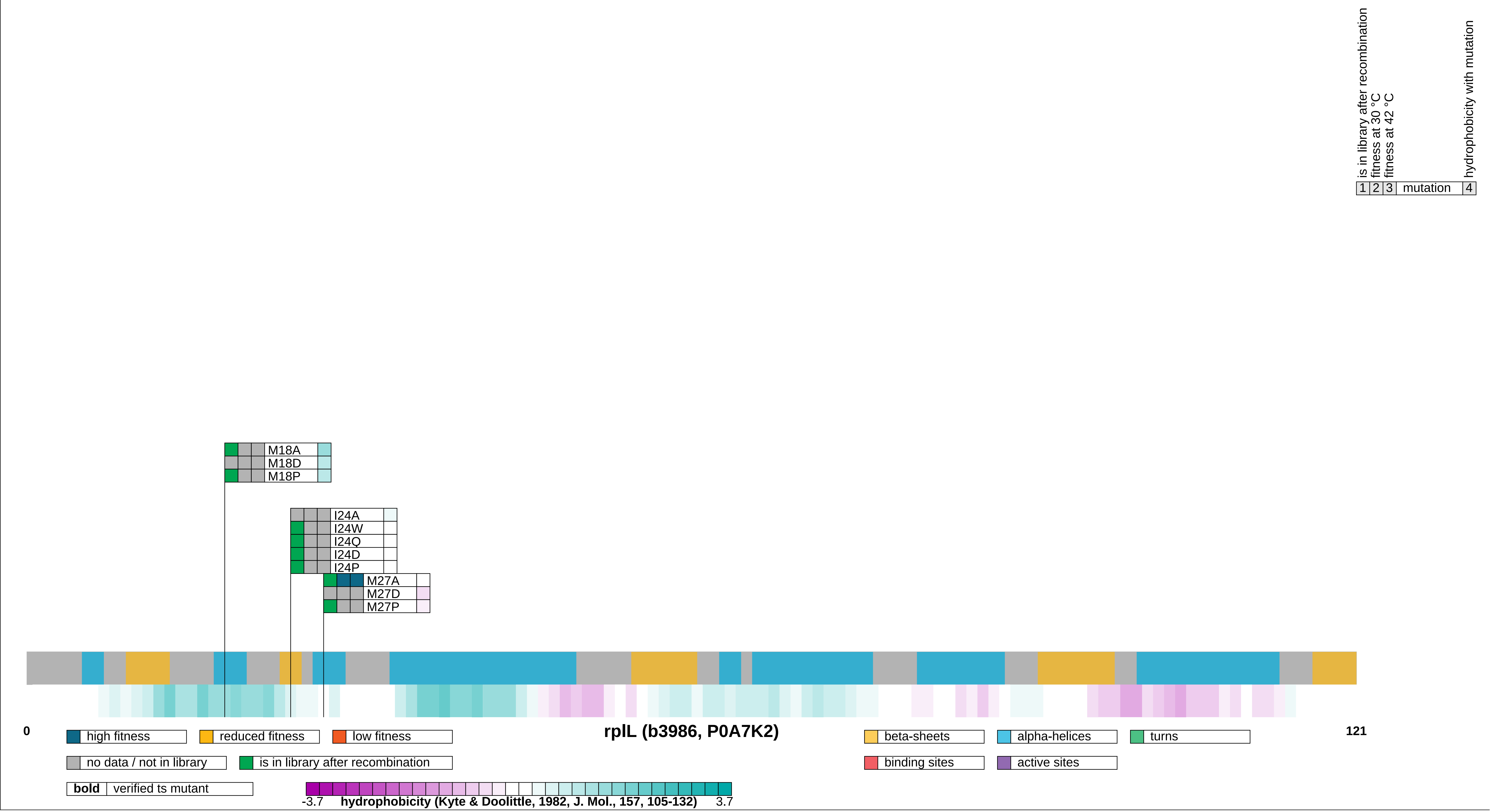

is in library after recombination
fitness at 30 °C
fitness at 42 °C
hydrophobicity with mutation
1 2 3 mutation 4
M18A
M18D
M18P
I24A
I24W
I24Q
I24D
I24P
M27A
M27D
M27P
0
rplL (b3986, P0A7K2)
121
high fitness
reduced fitness
low fitness
beta-sheets
alpha-helices
turns
no data / not in library
is in library after recombination
binding sites
active sites
bold verified ts mutant
-3.7
hydrophobicity (Kyte & Doolittle, 1982, J. Mol., 157, 105-132)
3.7

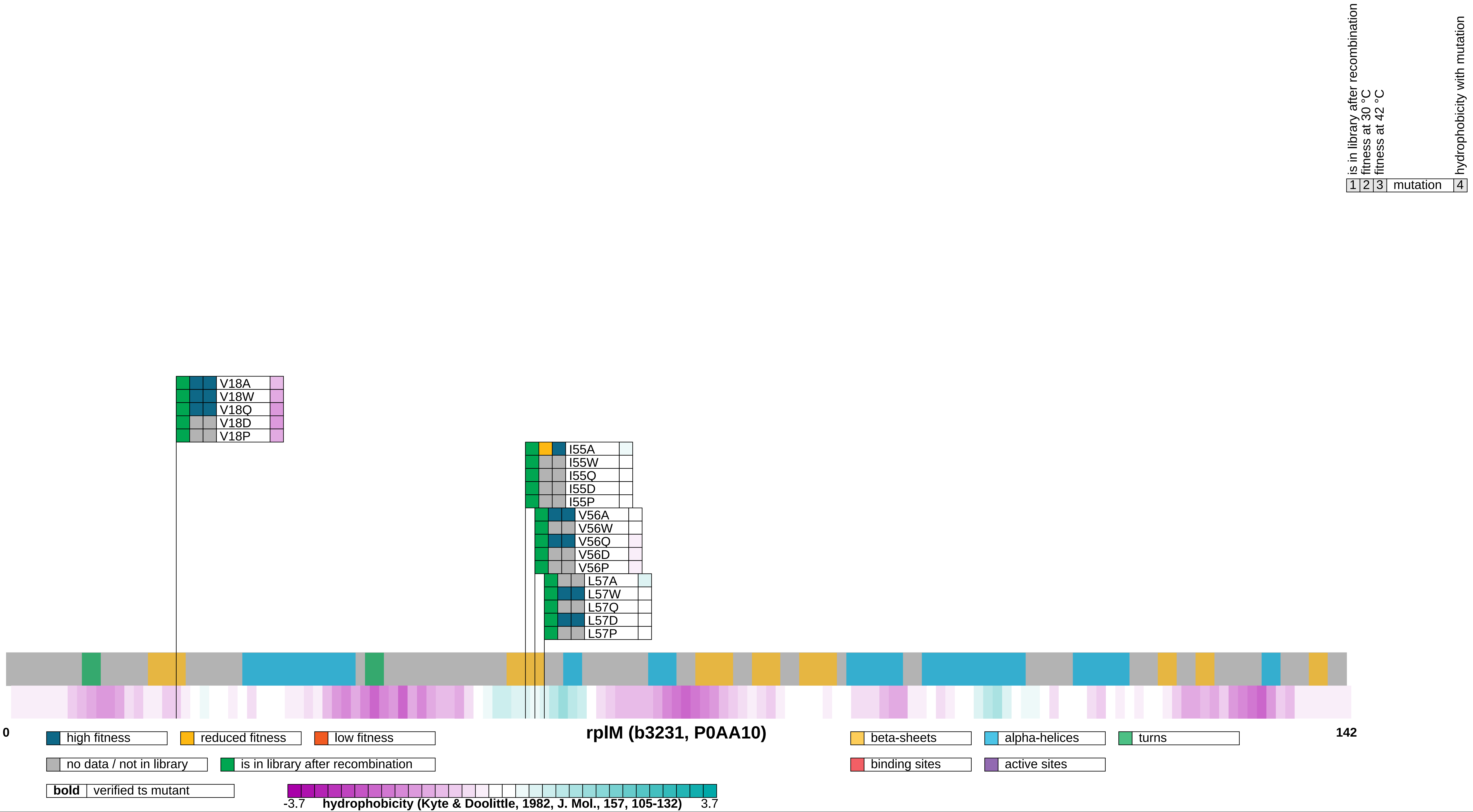
is in library after recombination
fitness at 30 °C
fitness at 42 °C
hydrophobicity with mutation
1 2 3 mutation 4
V18A
V18W
V18Q
V18D
V18P
I55A
I55W
I55Q
I55D
I55P
V56A
V56W
V56Q
V56D
V56P
L57A
L57W
L57Q
L57D
L57P
0
rplM (b3231, P0AA10)
142
high fitness
reduced fitness
low fitness
beta-sheets
alpha-helices
turns
no data / not in library
is in library after recombination
binding sites
active sites
bold verified ts mutant
-3.7
hydrophobicity (Kyte & Doolittle, 1982, J. Mol., 157, 105-132)
3.7

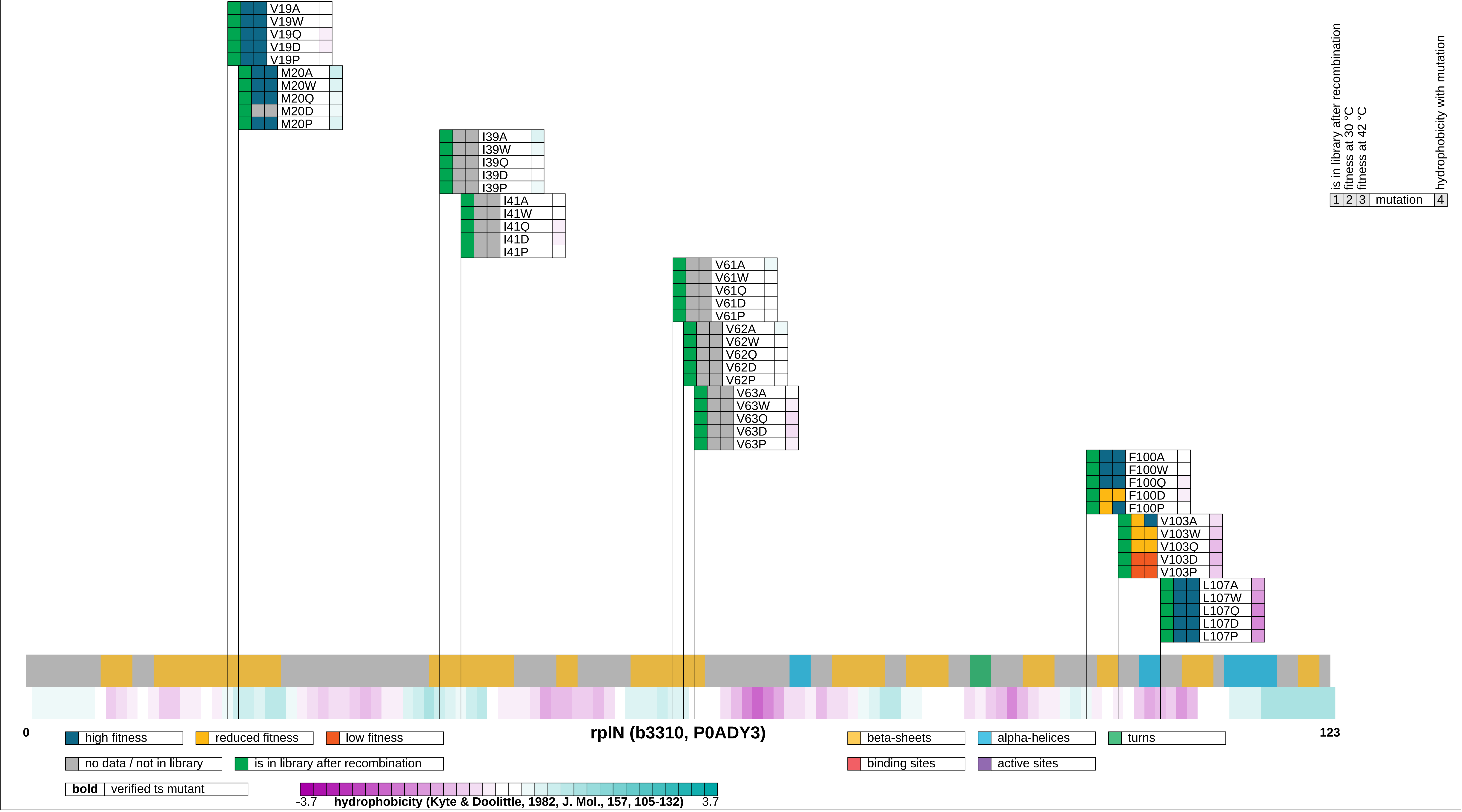

is in library after recombination
fitness at 30 °C
fitness at 42 °C
hydrophobicity with mutation
1 2 3 mutation 4
V19A
V19W
V19Q
V19D
V19P
M20A
M20W
M20Q
M20D
M20P
I39A
I39W
I39Q
I39D
I39P
I41A
I41W
I41Q
I41D
I41P
V61A
V61W
V61Q
V61D
V61P
V62A
V62W
V62Q
V62D
V62P
V63A
V63W
V63Q
V63D
V63P
F100A
F100W
F100Q
F100D
F100P
V103A
V103W
V103Q
V103D
V103P
L107A
L107W
L107Q
L107D
L107P
0
rplN (b3310, P0ADY3)
123
high fitness
reduced fitness
low fitness
beta-sheets
alpha-helices
turns
no data / not in library
is in library after recombination
binding sites
active sites
bold verified ts mutant
-3.7 hydrophobicity (Kyte & Doolittle, 1982, J. Mol., 157, 105-132) 3.7

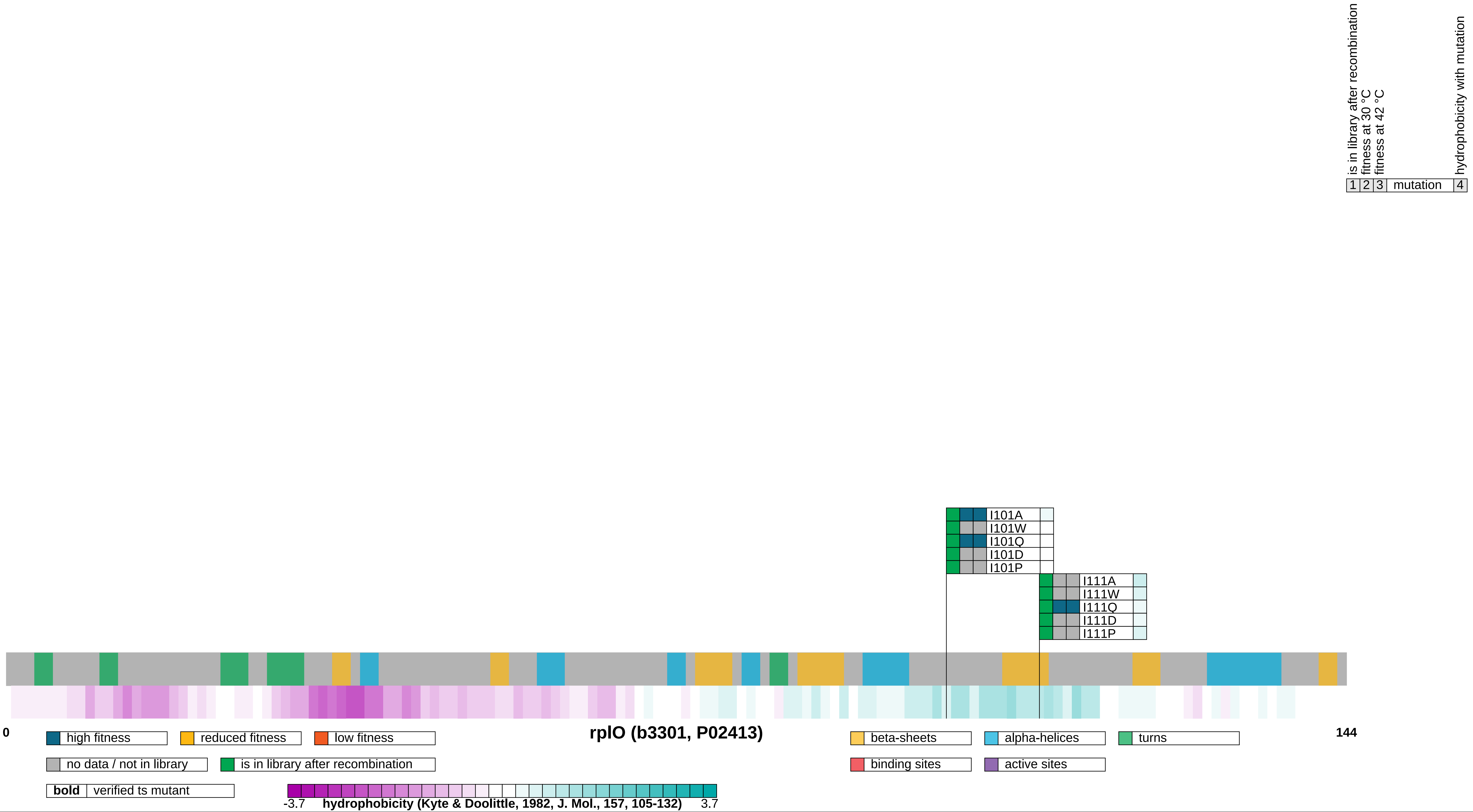

is in library after recombination
fitness at 30 °C
fitness at 42 °C
hydrophobicity with mutation
1
2
3
mutation
4
I101A
I101W
I101Q
I101D
I101P
I111A
I111W
I111Q
I111D
I111P
0
144
rplO (b3301, P02413)
high fitness
reduced fitness
low fitness
no data / not in library
is in library after recombination
bold
verified ts mutant
beta-sheets
alpha-helices
turns
binding sites
active sites
-3.7
hydrophobicity (Kyte & Doolittle, 1982, J. Mol., 157, 105-132)
3.7

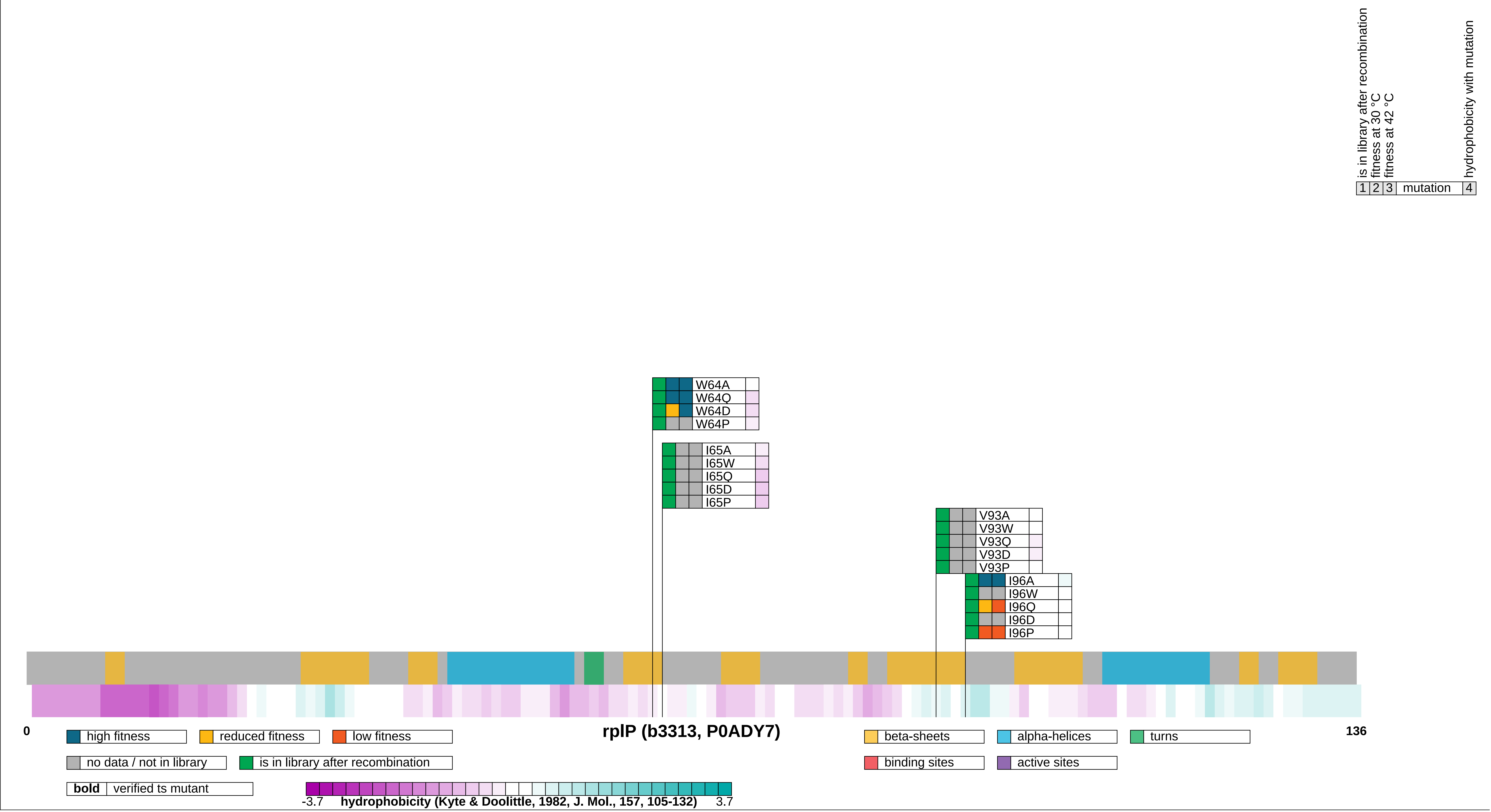
is in library after recombination
fitness at 30 °C
fitness at 42 °C
hydrophobicity with mutation
1 2 3 mutation 4
W64A
W64Q
W64D
W64P
I65A
I65W
I65Q
I65D
I65P
V93A
V93W
V93Q
V93D
V93P
I96A
I96W
I96Q
I96D
I96P
0
rplP (b3313, P0ADY7)
136
high fitness
reduced fitness
low fitness
no data / not in library
is in library after recombination
bold verified ts mutant
beta-sheets
alpha-helices
turns
binding sites
active sites
-3.7
hydrophobicity (Kyte & Doolittle, 1982, J. Mol., 157, 105-132)
3.7

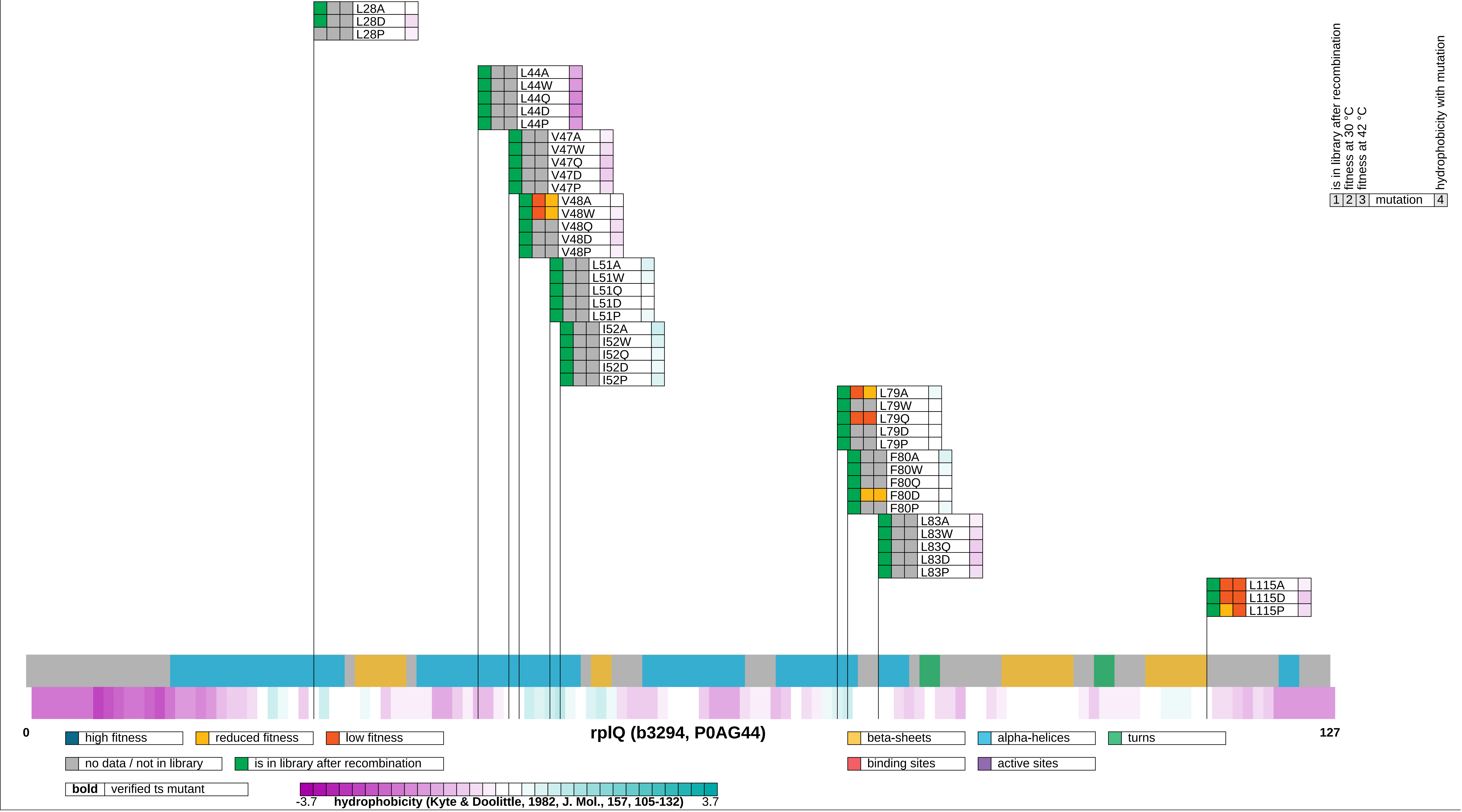
L28A
L28D
L28P
L44A
L44W
L44Q
L44D
L44P
V47A
V47W
V47Q
V47D
V47P
V48A
V48W
V48Q
V48D
V48P
L51A
L51W
L51Q
L51D
L51P
I52A
I52W
I52Q
I52D
I52P
L79A
L79W
L79Q
L79D
L79P
F80A
F80W
F80Q
F80D
F80P
L83A
L83W
L83Q
L83D
L83P
L115A
L115D
L115P
is in library after recombination
fitness at 30 °C
fitness at 42 °C
hydrophobicity with mutation
1 2 3 mutation 4
0
rplQ (b3294, P0AG44)
127
high fitness
reduced fitness
low fitness
beta-sheets
alpha-helices
turns
no data / not in library
is in library after recombination
binding sites
active sites
bold verified ts mutant
-3.7 hydrophobicity (Kyte & Doolittle, 1982, J. Mol., 157, 105-132) 3.7

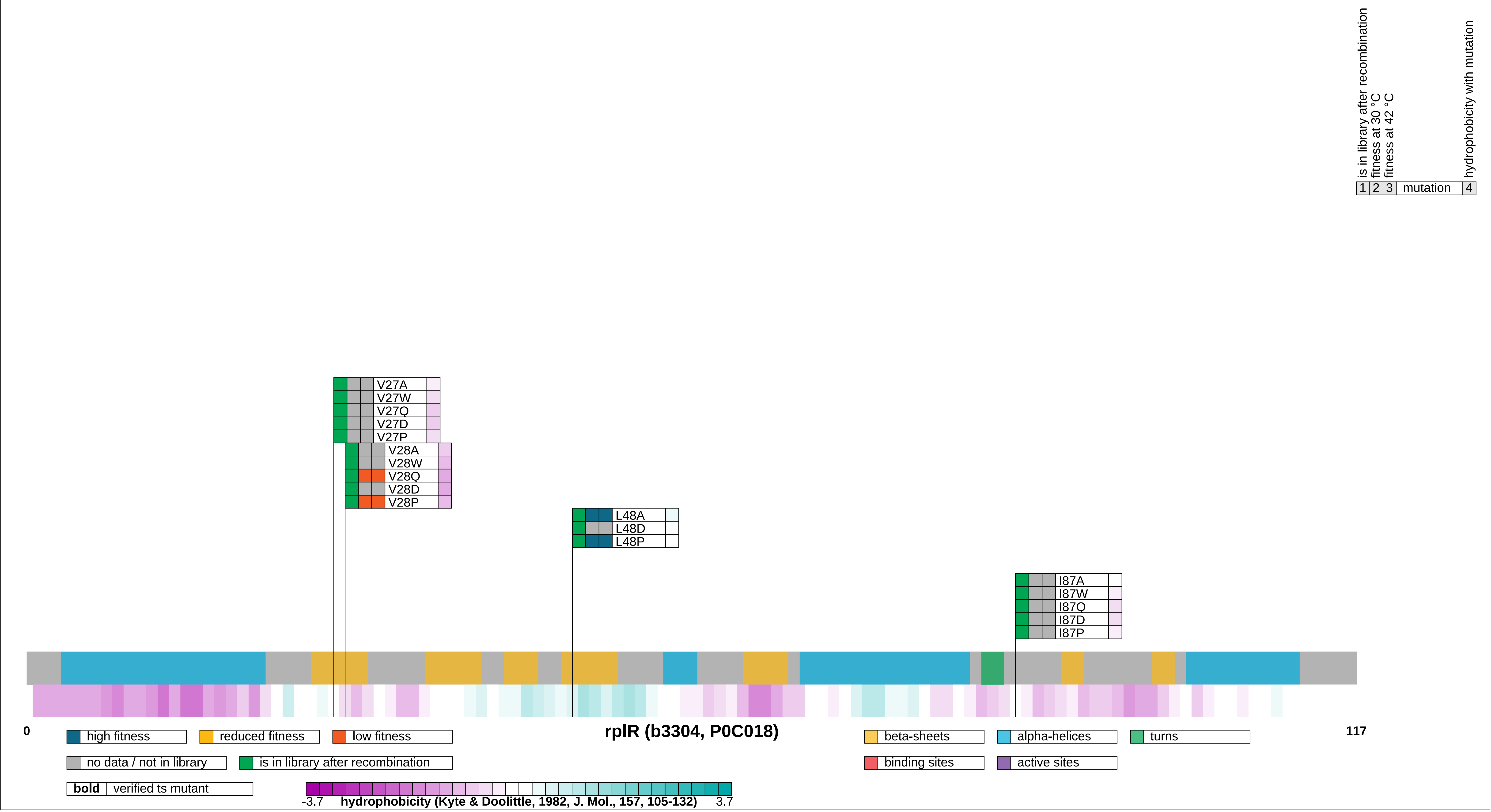

is in library after recombination
fitness at 30 °C
fitness at 42 °C
hydrophobicity with mutation
1 2 3 mutation 4
V27A
V27W
V27Q
V27D
V27P
V28A
V28W
V28Q
V28D
V28P
L48A
L48D
L48P
I87A
I87W
I87Q
I87D
I87P
0
rplR (b3304, P0C018)
117
high fitness
reduced fitness
low fitness
beta-sheets
alpha-helices
turns
no data / not in library
is in library after recombination
binding sites
active sites
bold verified ts mutant
-3.7 hydrophobicity (Kyte & Doolittle, 1982, J. Mol., 157, 105-132) 3.7

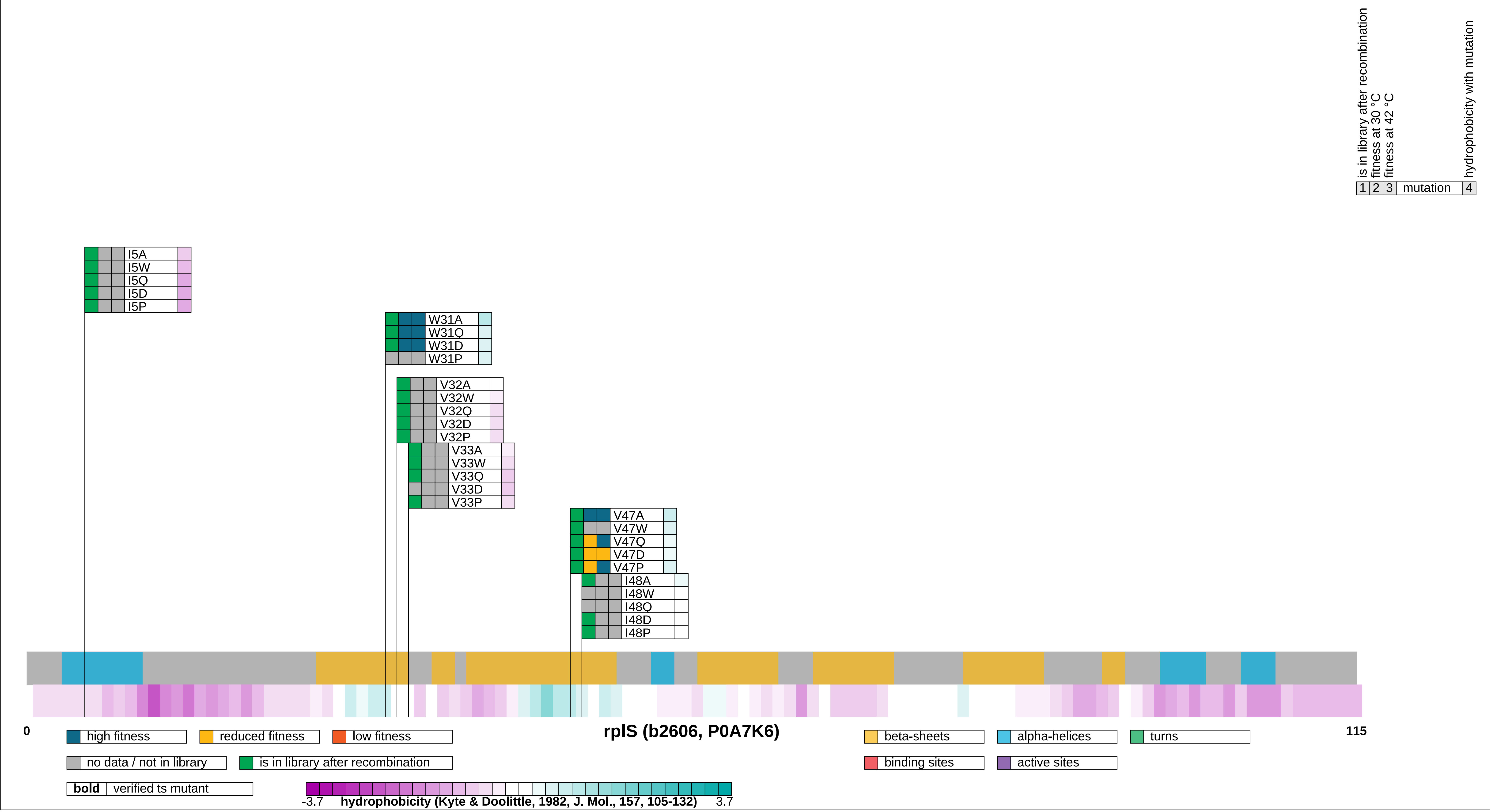
is in library after recombination
fitness at 30 °C
fitness at 42 °C
hydrophobicity with mutation
1 2 3 mutation 4
I5A
I5W
I5Q
I5D
I5P
W31A
W31Q
W31D
W31P
V32A
V32W
V32Q
V32D
V32P
V33A
V33W
V33Q
V33D
V33P
V47A
V47W
V47Q
V47D
V47P
I48A
I48W
I48Q
I48D
I48P
0
rplS (b2606, P0A7K6)
115
high fitness
reduced fitness
low fitness
beta-sheets
alpha-helices
turns
no data / not in library
is in library after recombination
binding sites
active sites
bold verified ts mutant
-3.7
hydrophobicity (Kyte & Doolittle, 1982, J. Mol., 157, 105-132)
3.7

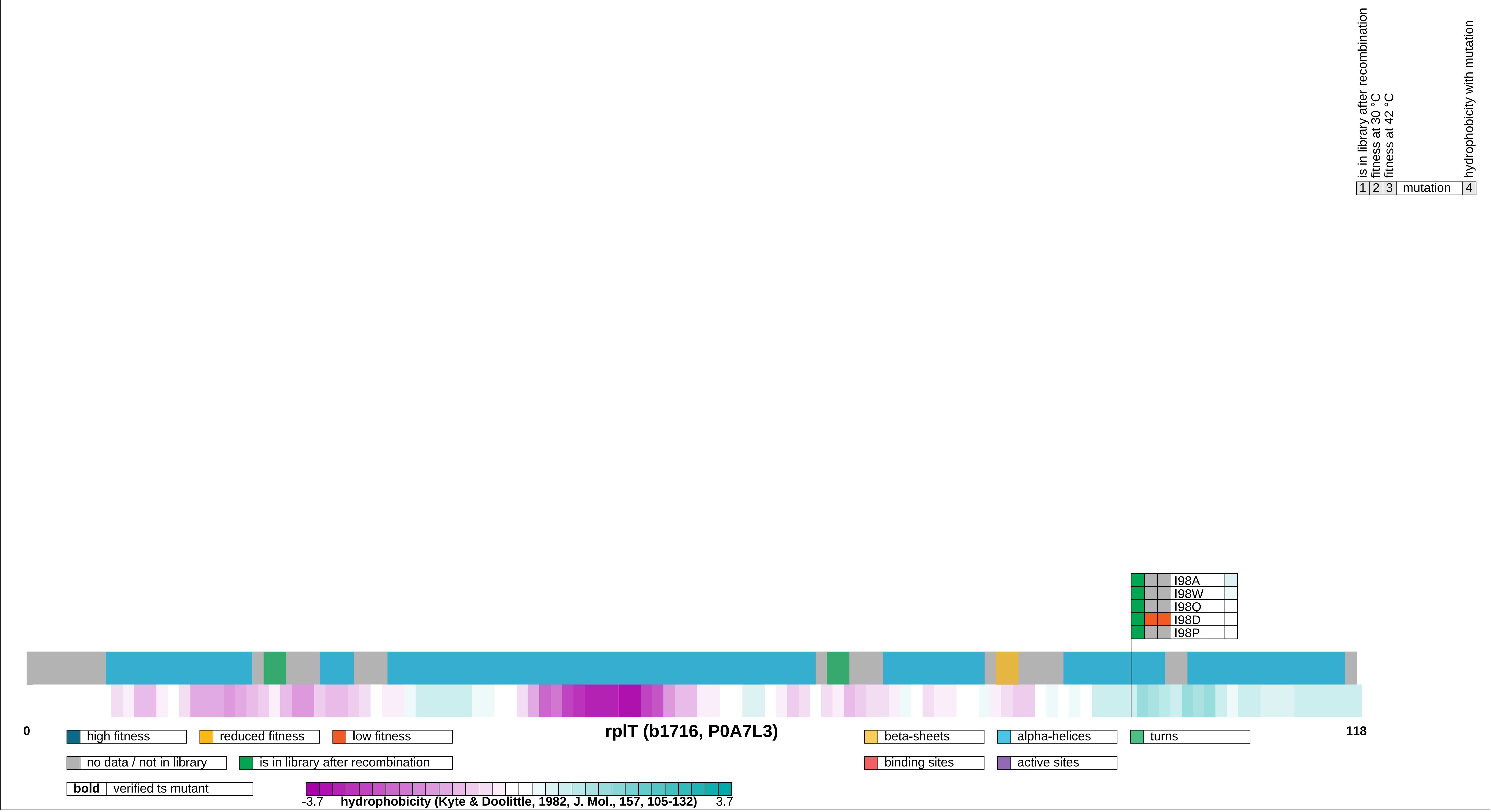

is in library after recombination
fitness at 30 °C
fitness at 42 °C
hydrophobicity with mutation
1
2
3
mutation
4
I98A
I98W
I98Q
I98D
I98P
0
rplT (b1716, P0A7L3)
118
high fitness
reduced fitness
low fitness
beta-sheets
alpha-helices
turns
no data / not in library
is in library after recombination
binding sites
active sites
bold
verified ts mutant
-3.7
hydrophobicity (Kyte & Doolittle, 1982, J. Mol., 157, 105-132)
3.7

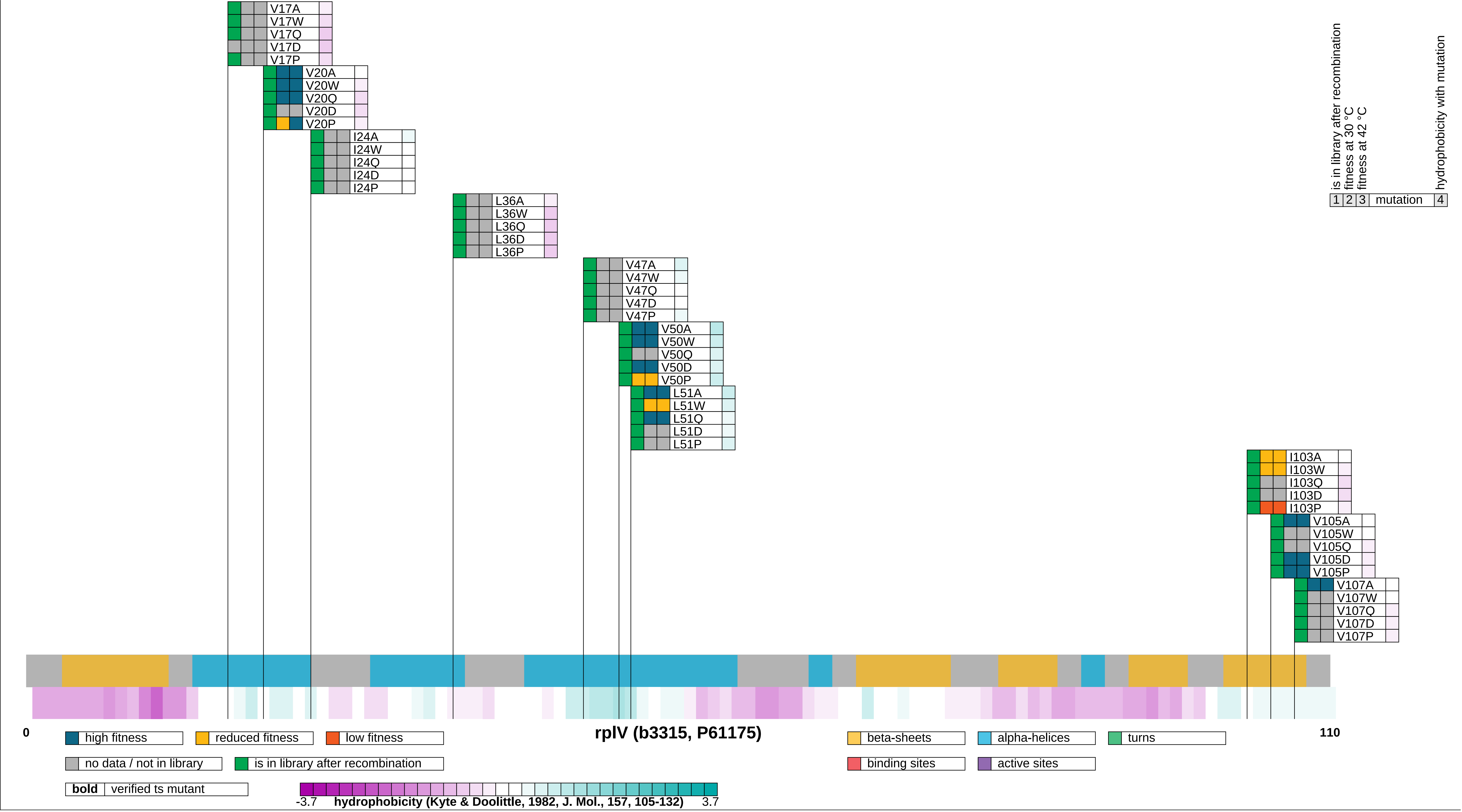
V17A
V17W
V17Q
V17D
V17P
V20A
V20W
V20Q
V20D
V20P
I24A
I24W
I24Q
I24D
I24P
L36A
L36W
L36Q
L36D
L36P
V47A
V47W
V47Q
V47D
V47P
V50A
V50W
V50Q
V50D
V50P
L51A
L51W
L51Q
L51D
L51P
I103A
I103W
I103Q
I103D
I103P
V105A
V105W
V105Q
V105D
V105P
V107A
V107W
V107Q
V107D
V107P
is in library after recombination
fitness at 30 °C
fitness at 42 °C
hydrophobicity with mutation
1 2 3 mutation 4
0
110
rplV (b3315, P61175)
high fitness
reduced fitness
low fitness
no data / not in library
is in library after recombination
bold verified ts mutant
beta-sheets
alpha-helices
turns
binding sites
active sites
-3.7
hydrophobicity (Kyte & Doolittle, 1982, J. Mol., 157, 105-132)
3.7

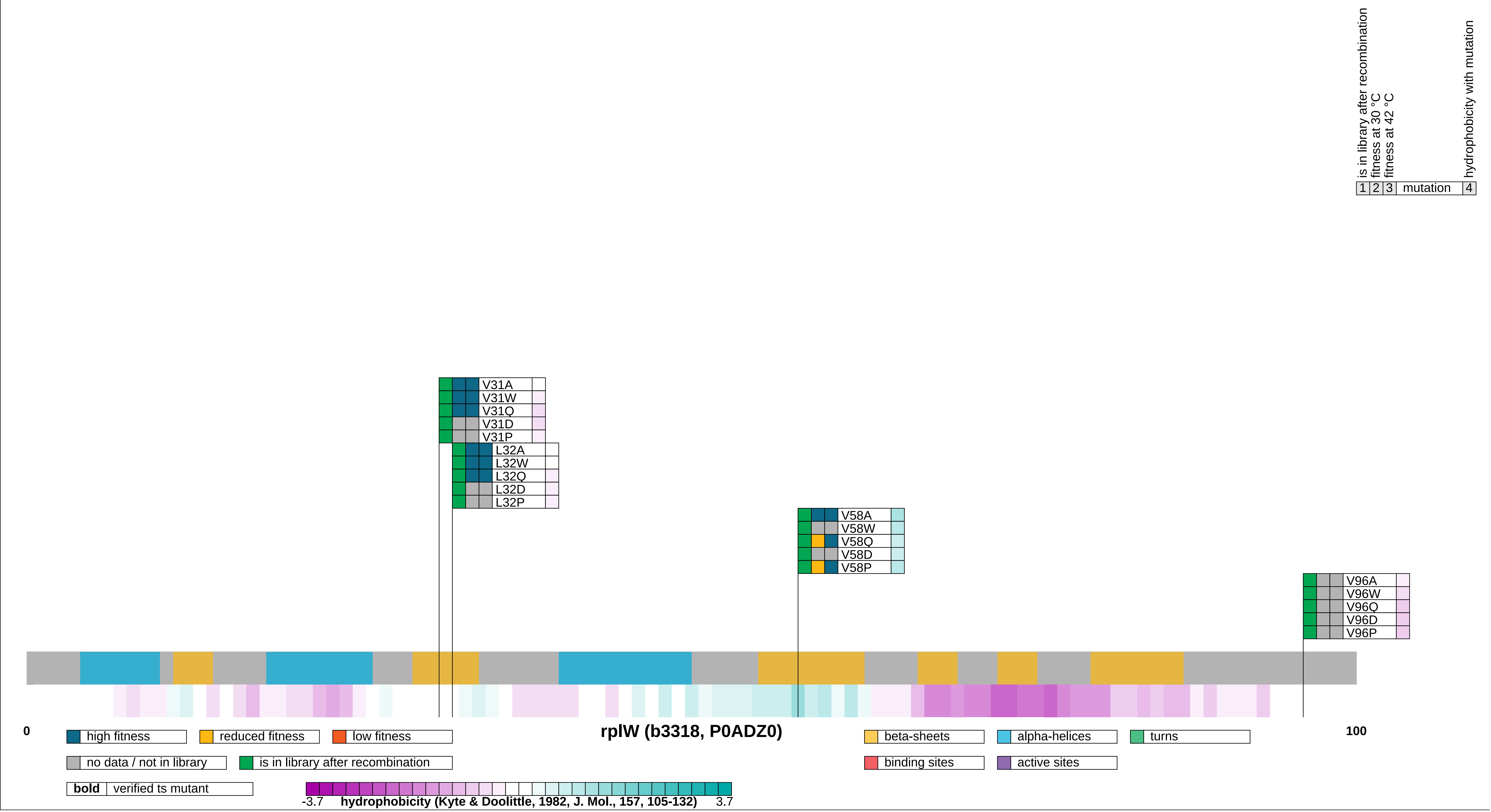
is in library after recombination
fitness at 30 °C
fitness at 42 °C
hydrophobicity with mutation
1 2 3 mutation 4
V31A
V31W
V31Q
V31D
V31P
L32A
L32W
L32Q
L32D
L32P
V58A
V58W
V58Q
V58D
V58P
V96A
V96W
V96Q
V96D
V96P
0
rplW (b3318, P0ADZ0)
100
high fitness
reduced fitness
low fitness
beta-sheets
alpha-helices
turns
no data / not in library
is in library after recombination
binding sites
active sites
bold verified ts mutant
-3.7
hydrophobicity (Kyte & Doolittle, 1982, J. Mol., 157, 105-132)
3.7

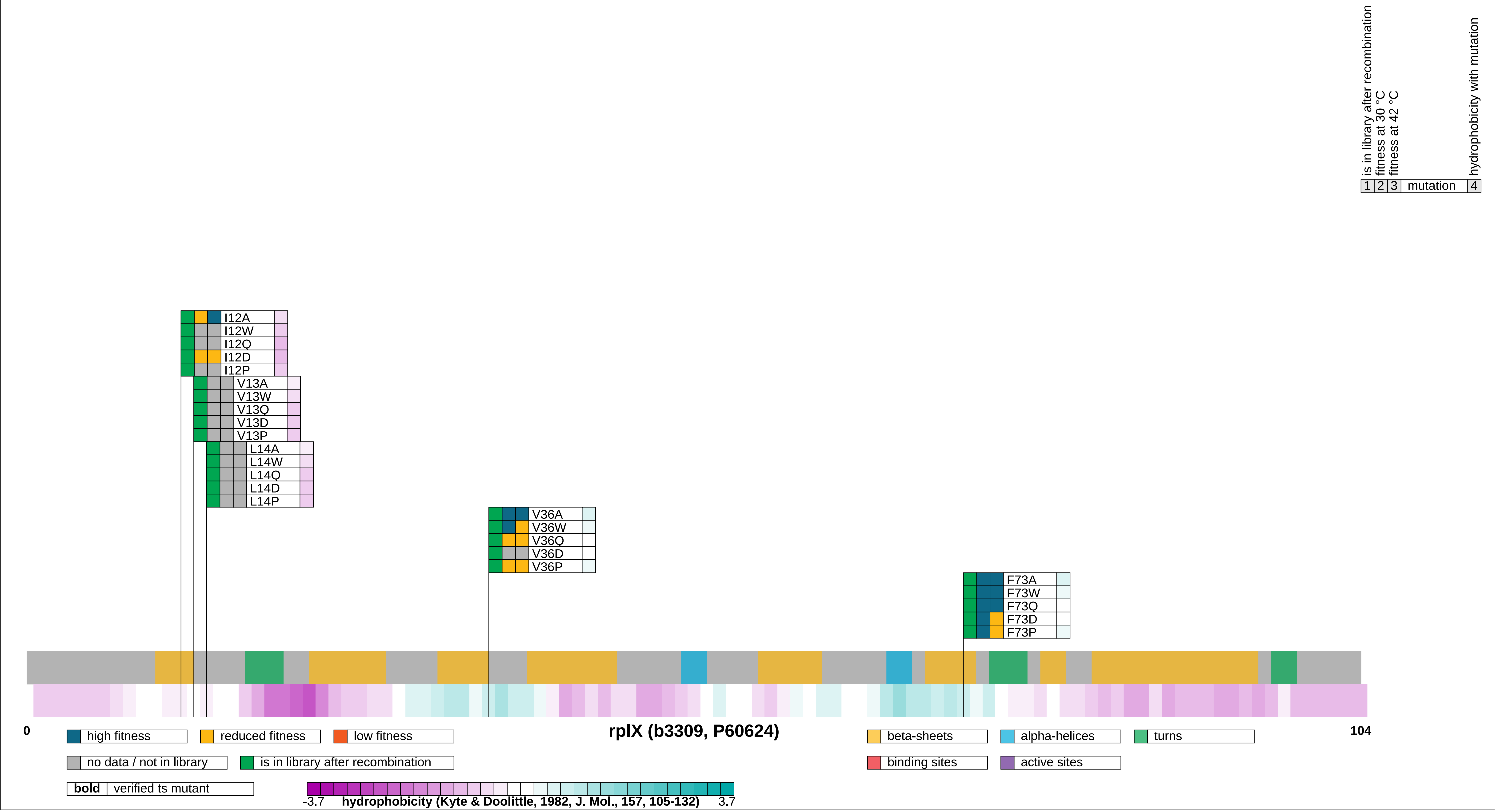
is in library after recombination
fitness at 30 °C
fitness at 42 °C
hydrophobicity with mutation
1 2 3 mutation 4
I12A
I12W
I12Q
I12D
I12P
V13A
V13W
V13Q
V13D
V13P
L14A
L14W
L14Q
L14D
L14P
V36A
V36W
V36Q
V36D
V36P
F73A
F73W
F73Q
F73D
F73P
0
rplX (b3309, P60624)
104
high fitness
reduced fitness
low fitness
beta-sheets
alpha-helices
turns
no data / not in library
is in library after recombination
binding sites
active sites
bold verified ts mutant
-3.7 hydrophobicity (Kyte & Doolittle, 1982, J. Mol., 157, 105-132) 3.7

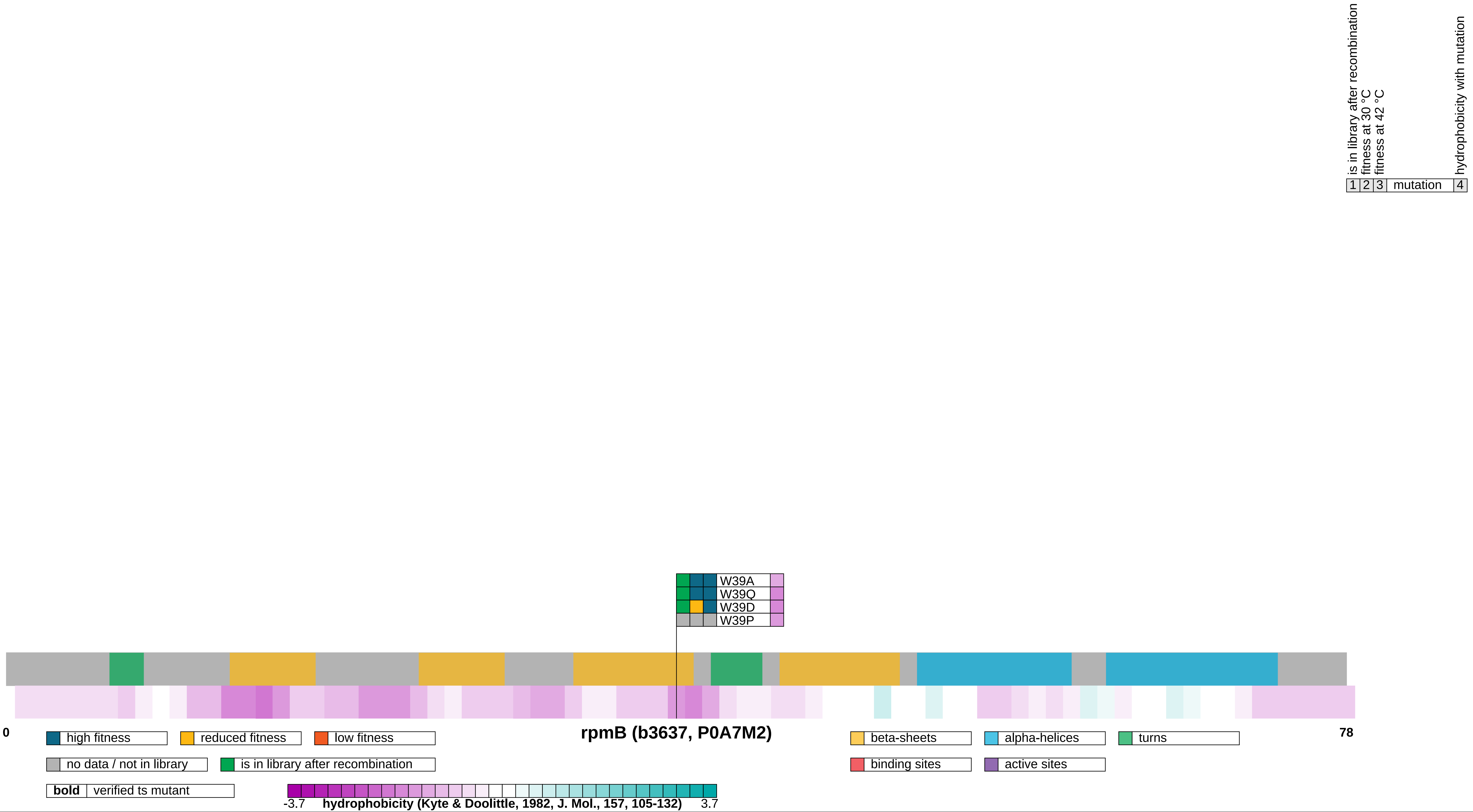

is in library after recombination
fitness at 30 °C
fitness at 42 °C
hydrophobicity with mutation
1
2
3
mutation
4
W39A
W39Q
W39D
W39P
0
rpmB (b3637, P0A7M2)
78
high fitness
reduced fitness
low fitness
beta-sheets
alpha-helices
turns
no data / not in library
is in library after recombination
binding sites
active sites
bold
verified ts mutant
-3.7
hydrophobicity (Kyte & Doolittle, 1982, J. Mol., 157, 105-132)
3.7

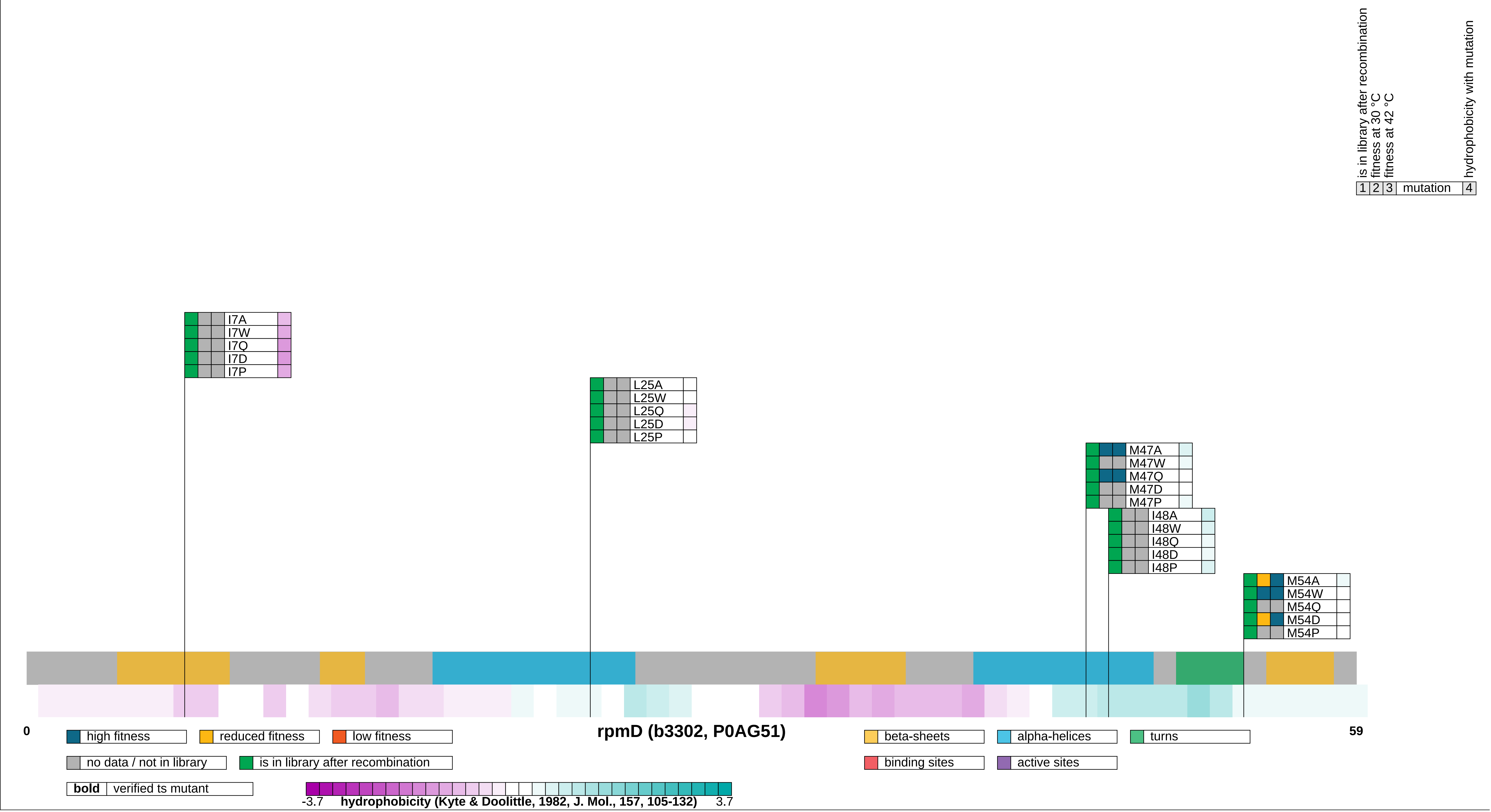

is in library after recombination
fitness at 30 °C
fitness at 42 °C
hydrophobicity with mutation
1 2 3 mutation 4
I7A
I7W
I7Q
I7D
I7P
L25A
L25W
L25Q
L25D
L25P
M47A
M47W
M47Q
M47D
M47P
I48A
I48W
I48Q
I48D
I48P
M54A
M54W
M54Q
M54D
M54P
0
rpmD (b3302, P0AG51)
59
high fitness
reduced fitness
low fitness
beta-sheets
alpha-helices
turns
no data / not in library
is in library after recombination
binding sites
active sites
bold verified ts mutant
-3.7 hydrophobicity (Kyte & Doolittle, 1982, J. Mol., 157, 105-132) 3.7

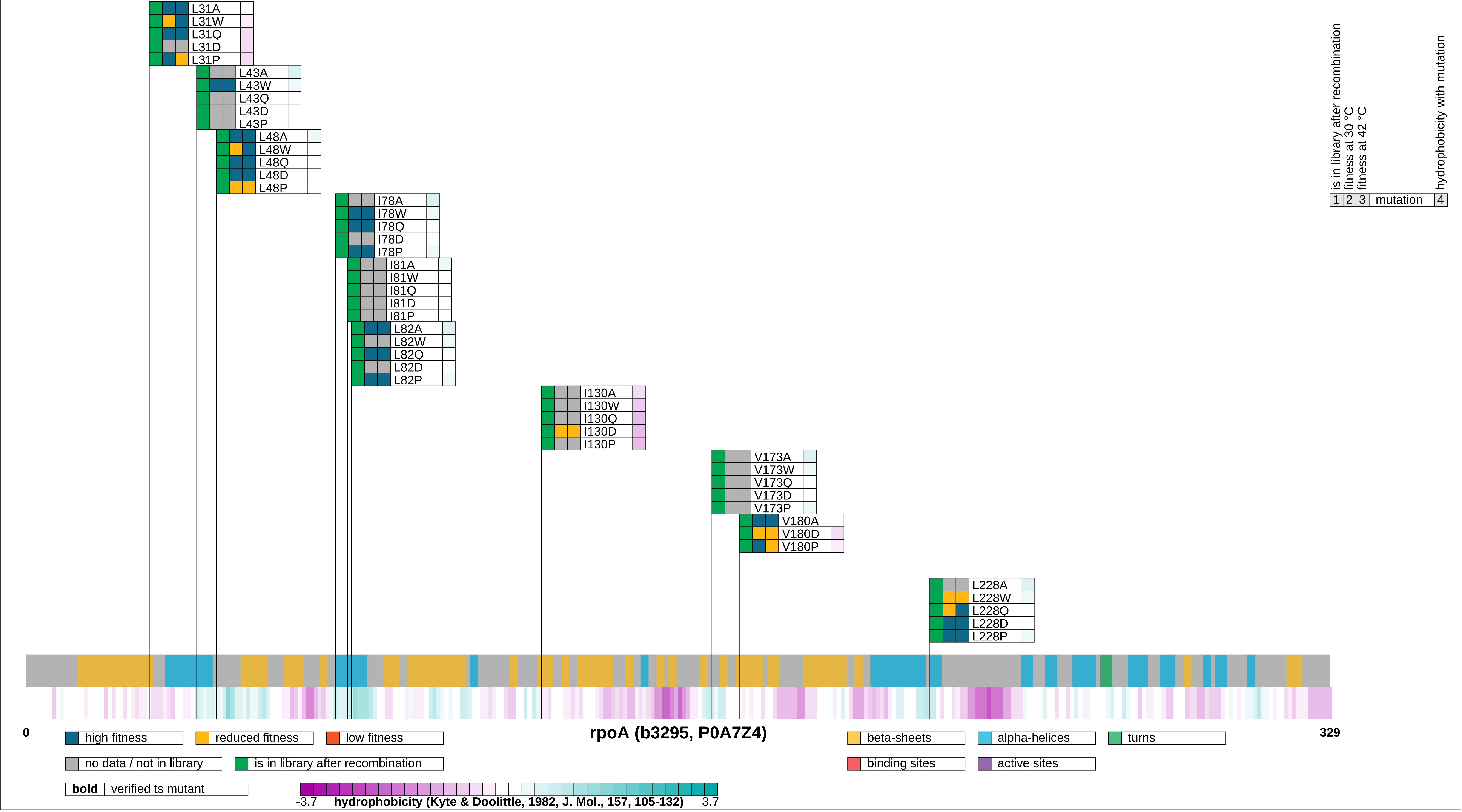
L31A
L31W
L31Q
L31D
L31P
L43A
L43W
L43Q
L43D
L43P
L48A
L48W
L48Q
L48D
L48P
I78A
I78W
I78Q
I78D
I78P
I81A
I81W
I81Q
I81D
I81P
L82A
L82W
L82Q
L82D
L82P
I130A
I130W
I130Q
I130D
I130P
V173A
V173W
V173Q
V173D
V173P
V180A
V180D
V180P
L228A
L228W
L228Q
L228D
L228P
is in library after recombination
fitness at 30 °C
fitness at 42 °C
hydrophobicity with mutation
1 2 3 mutation 4
0
rpoA (b3295, P0A7Z4)
329
high fitness
reduced fitness
low fitness
beta-sheets
alpha-helices
turns
no data / not in library
is in library after recombination
binding sites
active sites
bold verified ts mutant
-3.7
hydrophobicity (Kyte & Doolittle, 1982, J. Mol., 157, 105-132)
3.7

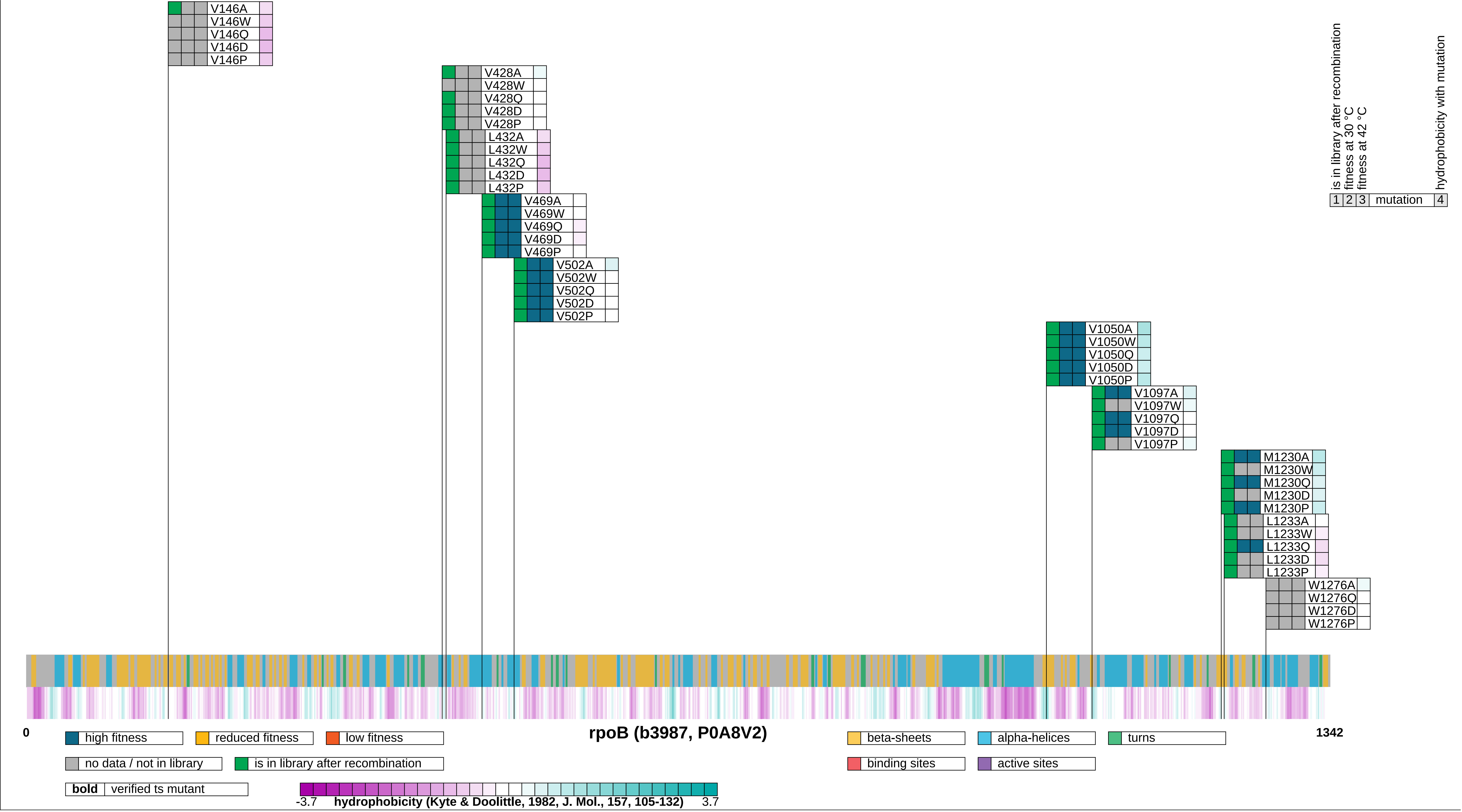
V146A
V146W
V146Q
V146D
V146P
V428A
V428W
V428Q
V428D
V428P
L432A
L432W
L432Q
L432D
L432P
V469A
V469W
V469Q
V469D
V469P
V502A
V502W
V502Q
V502D
V502P
V1050A
V1050W
V1050Q
V1050D
V1050P
V1097A
V1097W
V1097Q
V1097D
V1097P
M1230A
M1230W
M1230Q
M1230D
M1230P
L1233A
L1233W
L1233Q
L1233D
L1233P
W1276A
W1276Q
W1276D
W1276P
is in library after recombination
fitness at 30 °C
fitness at 42 °C
hydrophobicity with mutation
1 2 3 mutation 4
0
rpoB (b3987, P0A8V2)
1342
high fitness
reduced fitness
low fitness
no data / not in library
is in library after recombination
bold verified ts mutant
beta-sheets
alpha-helices
turns
binding sites
active sites
-3.7 hydrophobicity (Kyte & Doolittle, 1982, J. Mol., 157, 105-132) 3.7

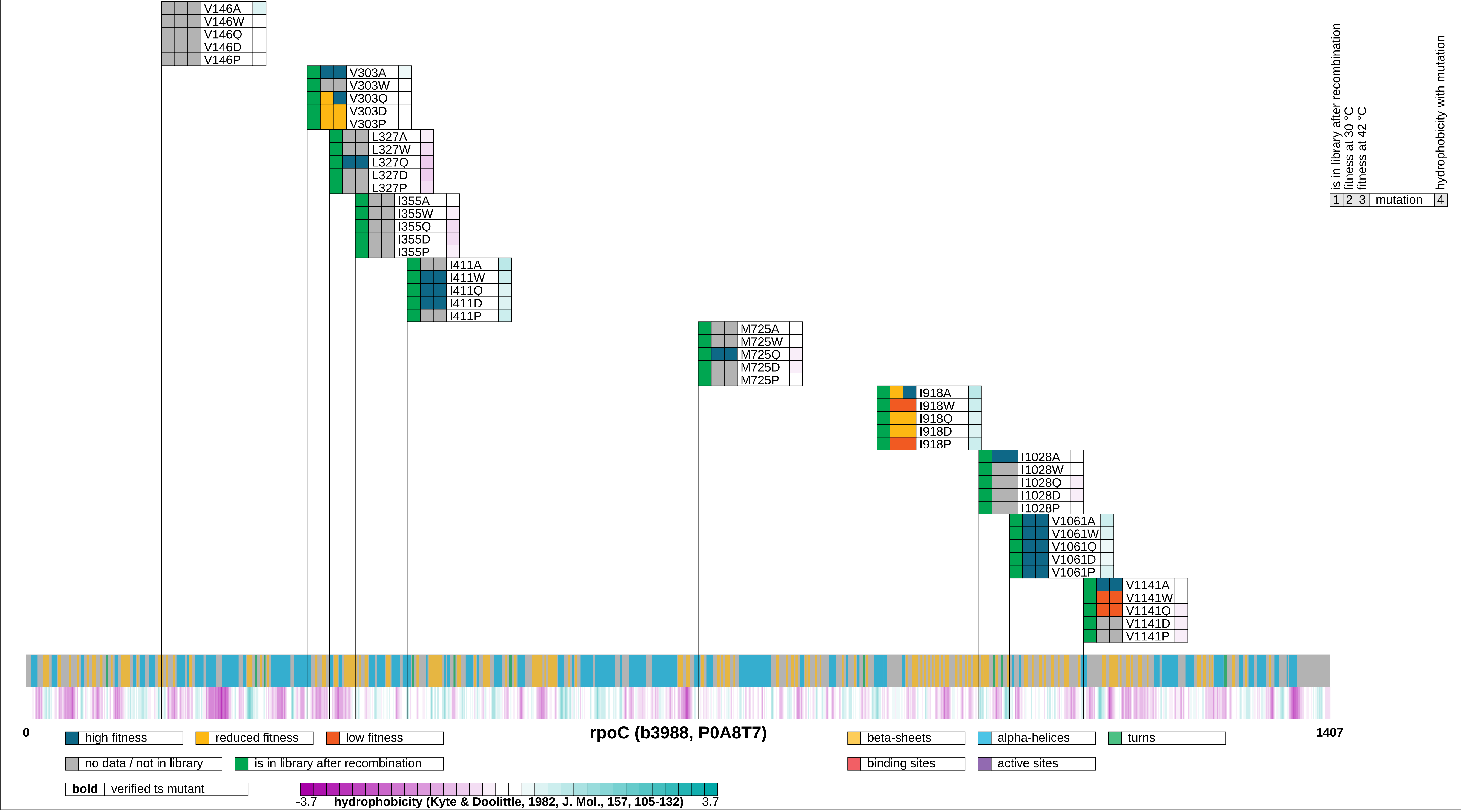

V146A
V146W
V146Q
V146D
V146P
V303A
V303W
V303Q
V303D
V303P
L327A
L327W
L327Q
L327D
L327P
I355A
I355W
I355Q
I355D
I355P
I411A
I411W
I411Q
I411D
I411P
M725A
M725W
M725Q
M725D
M725P
I918A
I918W
I918Q
I918D
I918P
I1028A
I1028W
I1028Q
I1028D
I1028P
V1061A
V1061W
V1061Q
V1061D
V1061P
V1141A
V1141W
V1141Q
V1141D
V1141P
is in library after recombination
fitness at 30 °C
fitness at 42 °C
hydrophobicity with mutation
1 2 3 mutation 4
0
rpoC (b3988, P0A8T7)
1407
high fitness
reduced fitness
low fitness
beta-sheets
alpha-helices
turns
no data / not in library
is in library after recombination
binding sites
active sites
bold verified ts mutant
-3.7 hydrophobicity (Kyte & Doolittle, 1982, J. Mol., 157, 105-132) 3.7

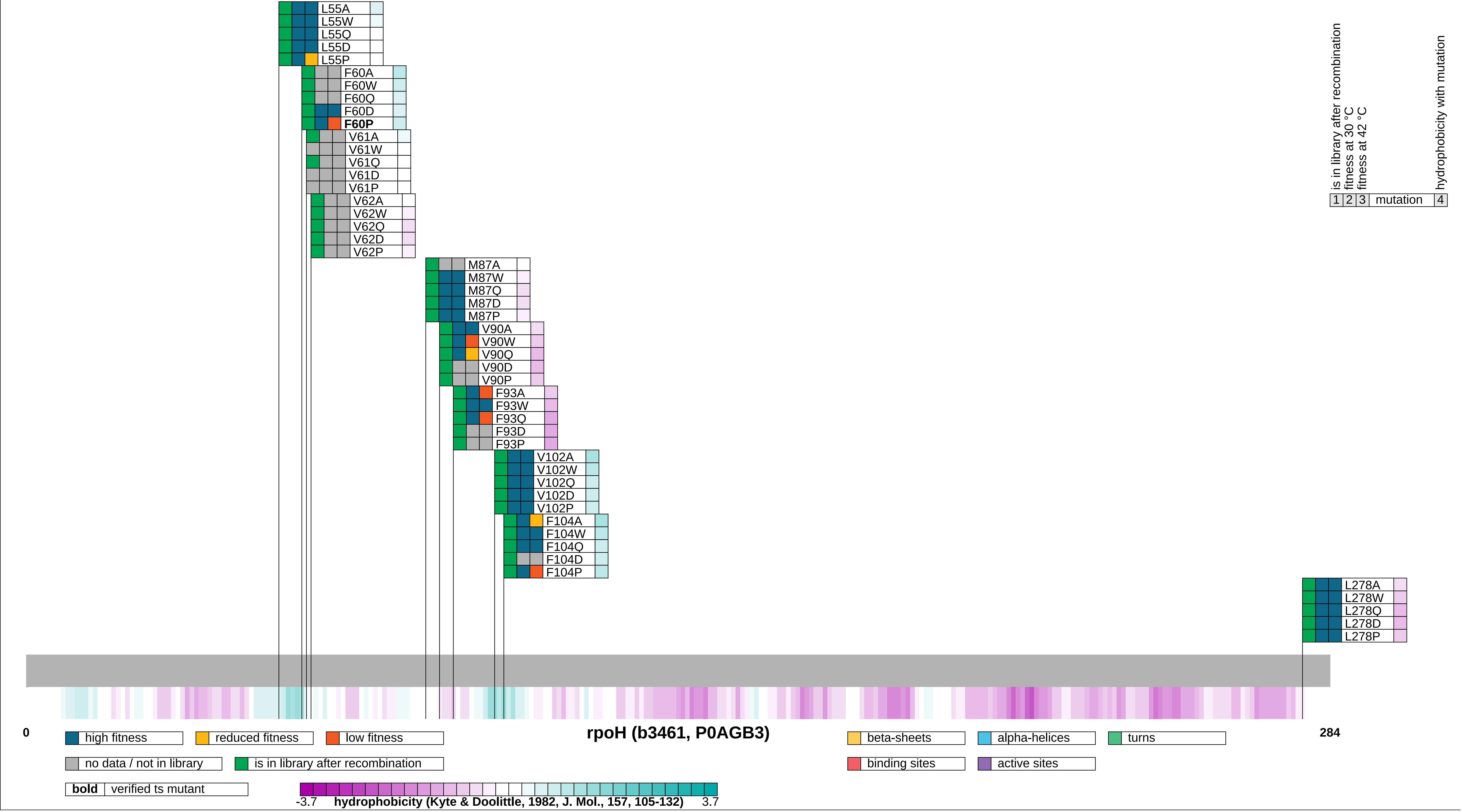

is in library after recombination
fitness at 30 °C
fitness at 42 °C
hydrophobicity with mutation
1 2 3 mutation 4
L55A
L55W
L55Q
L55D
L55P
F60A
F60W
F60Q
F60D
F60P
V61A
V61W
V61Q
V61D
V61P
V62A
V62W
V62Q
V62D
V62P
M87A
M87W
M87Q
M87D
M87P
V90A
V90W
V90Q
V90D
V90P
F93A
F93W
F93Q
F93D
F93P
V102A
V102W
V102Q
V102D
V102P
F104A
F104W
F104Q
F104D
F104P
L278A
L278W
L278Q
L278D
L278P
0
rpoH (b3461, P0AGB3)
284
high fitness
reduced fitness
low fitness
beta-sheets
alpha-helices
turns
no data / not in library
is in library after recombination
binding sites
active sites
bold verified ts mutant
-3.7 hydrophobicity (Kyte & Doolittle, 1982, J. Mol., 157, 105-132) 3.7

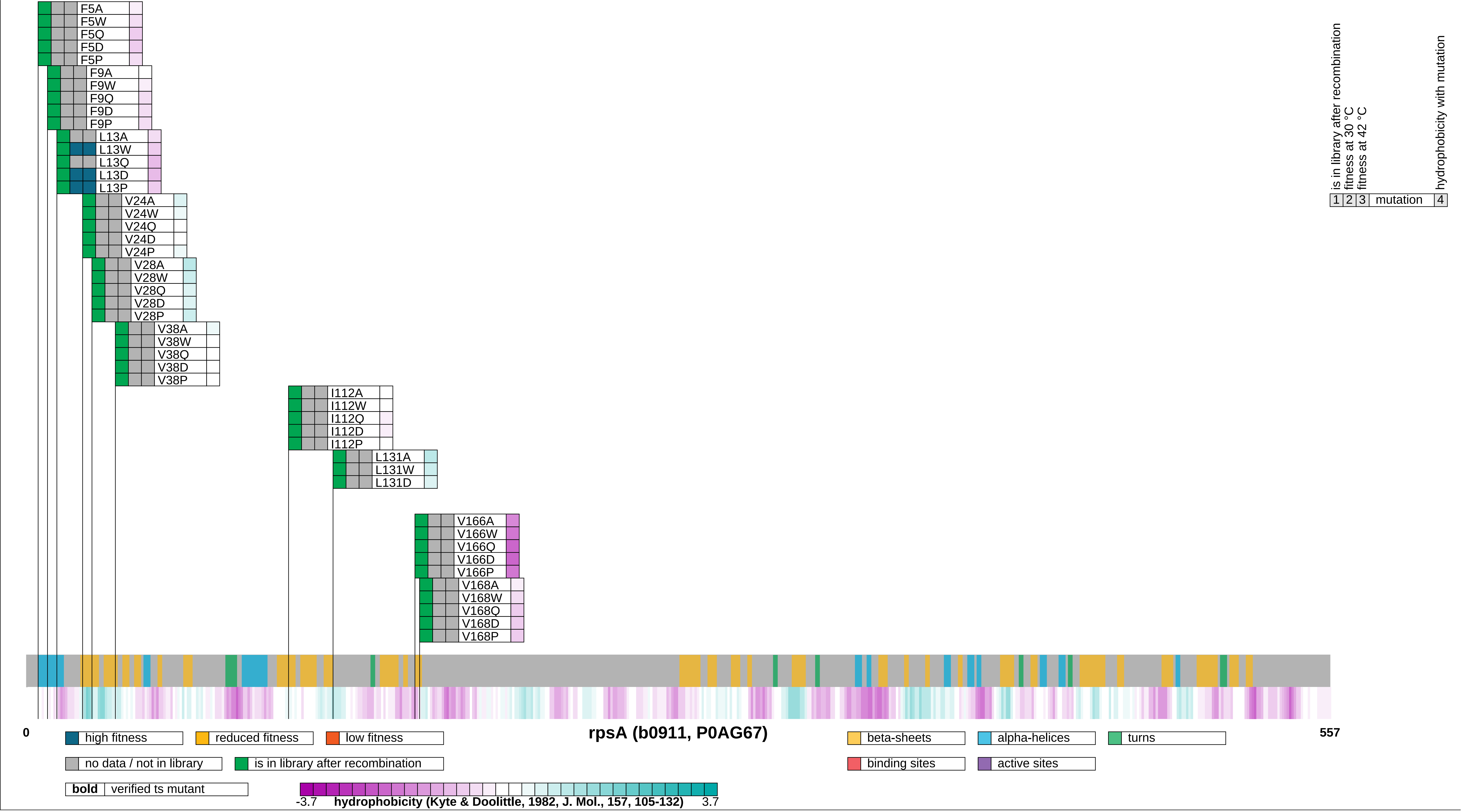

F5A
F5W
F5Q
F5D
F5P
F9A
F9W
F9Q
F9D
F9P
L13A
L13W
L13Q
L13D
L13P
V24A
V24W
V24Q
V24D
V24P
V28A
V28W
V28Q
V28D
V28P
V38A
V38W
V38Q
V38D
V38P
I112A
I112W
I112Q
I112D
I112P
L131A
L131W
L131D
V166A
V166W
V166Q
V166D
V166P
V168A
V168W
V168Q
V168D
V168P
is in library after recombination
fitness at 30 °C
fitness at 42 °C
hydrophobicity with mutation
1
2
3
mutation
4
0
rpsA (b0911, P0AG67)
557
high fitness
reduced fitness
low fitness
beta-sheets
alpha-helices
turns
no data / not in library
is in library after recombination
binding sites
active sites
bold
verified ts mutant
-3.7
hydrophobicity (Kyte & Doolittle, 1982, J. Mol., 157, 105-132)
3.7

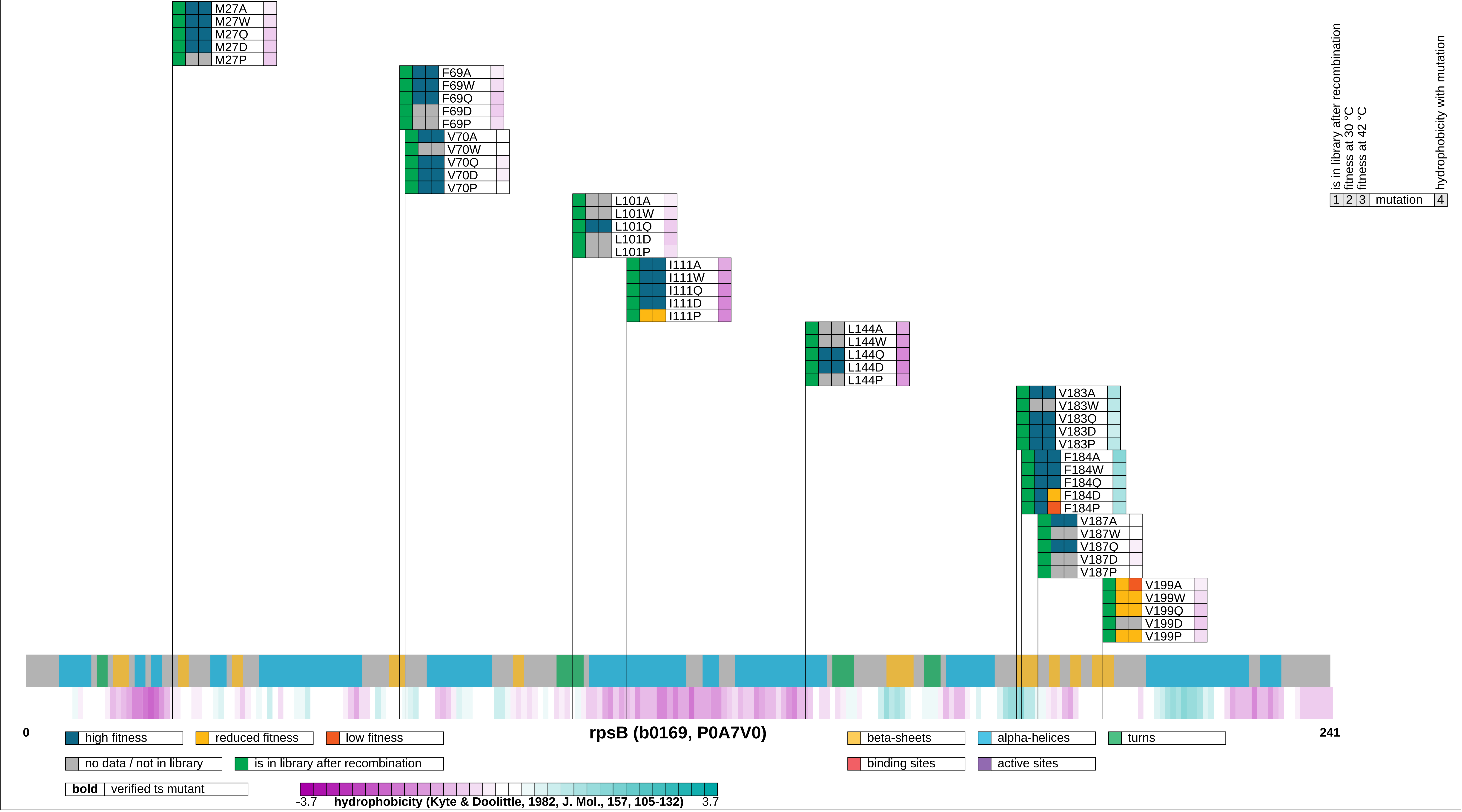

M27A
M27W
M27Q
M27D
M27P
F69A
F69W
F69Q
F69D
F69P
V70A
V70W
V70Q
V70D
V70P
L101A
L101W
L101Q
L101D
L101P
I111A
I111W
I111Q
I111D
I111P
L144A
L144W
L144Q
L144D
L144P
V183A
V183W
V183Q
V183D
V183P
F184A
F184W
F184Q
F184D
F184P
V187A
V187W
V187Q
V187D
V187P
V199A
V199W
V199Q
V199D
V199P
is in library after recombination
fitness at 30 °C
fitness at 42 °C
hydrophobicity with mutation
1 2 3 mutation 4
0
rpsB (b0169, P0A7V0)
241
high fitness
reduced fitness
low fitness
beta-sheets
alpha-helices
turns
no data / not in library
is in library after recombination
binding sites
active sites
bold verified ts mutant
-3.7
hydrophobicity (Kyte & Doolittle, 1982, J. Mol., 157, 105-132)
3.7

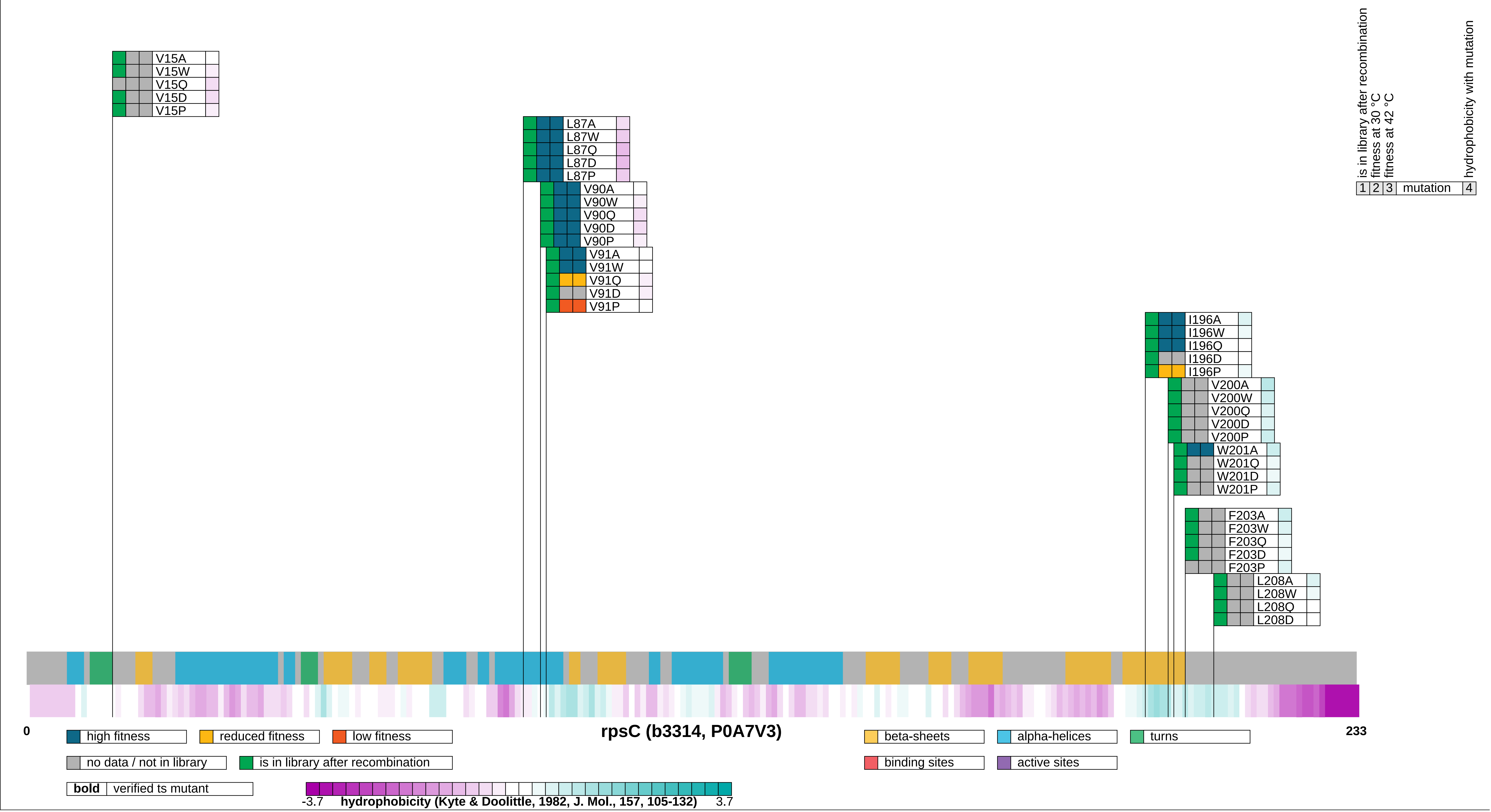
V15A
V15W
V15Q
V15D
V15P
L87A
L87W
L87Q
L87D
L87P
V90A
V90W
V90Q
V90D
V90P
V91A
V91W
V91Q
V91D
V91P
I196A
I196W
I196Q
I196D
I196P
V200A
V200W
V200Q
V200D
V200P
W201A
W201Q
W201D
W201P
F203A
F203W
F203Q
F203D
F203P
L208A
L208W
L208Q
L208D
is in library after recombination
fitness at 30 °C
fitness at 42 °C
hydrophobicity with mutation
1 2 3 mutation 4
0
rpsC (b3314, P0A7V3)
233
high fitness
reduced fitness
low fitness
no data / not in library
is in library after recombination
bold verified ts mutant
beta-sheets
alpha-helices
turns
binding sites
active sites
-3.7 hydrophobicity (Kyte & Doolittle, 1982, J. Mol., 157, 105-132) 3.7

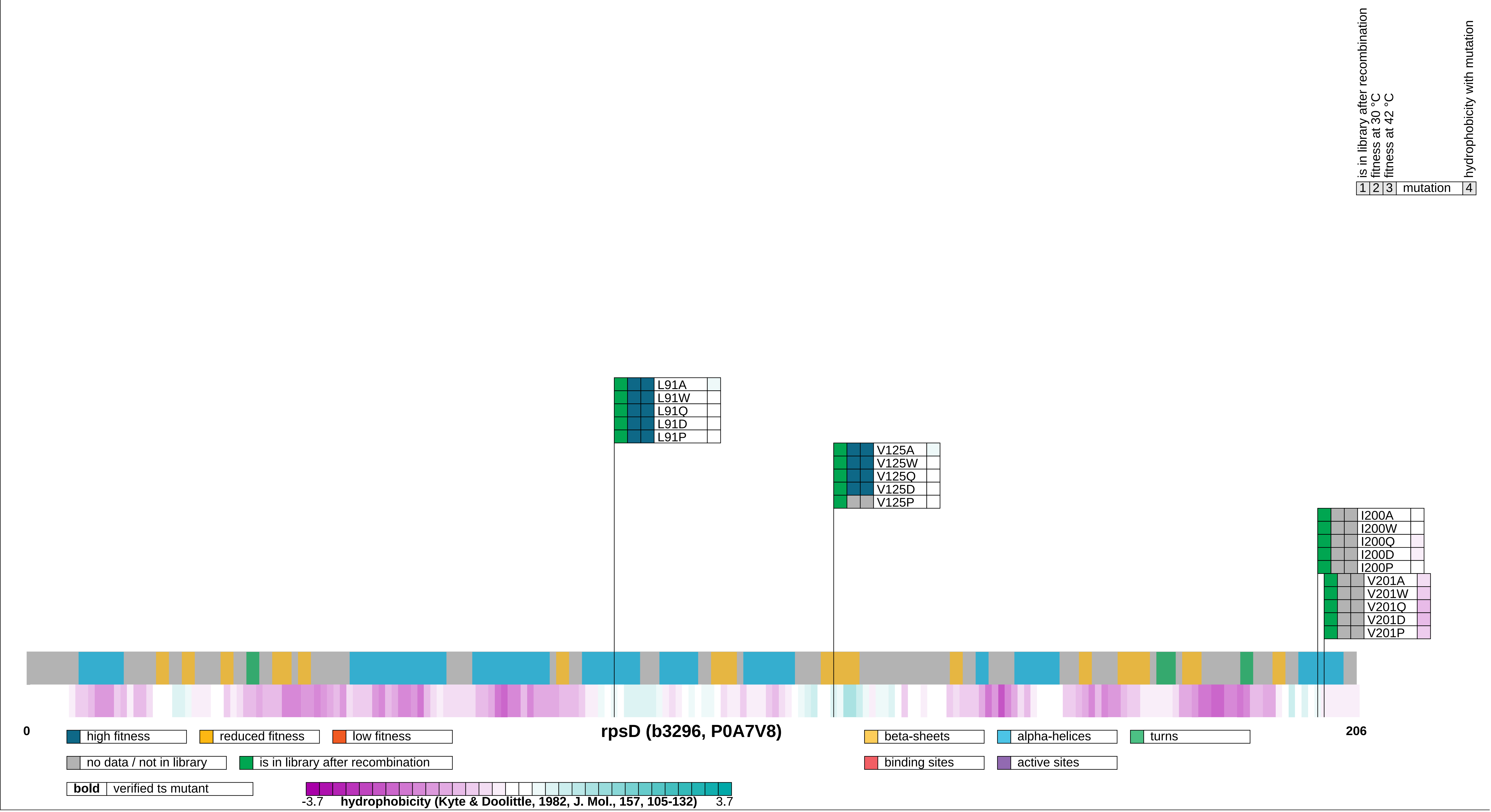

is in library after recombination
fitness at 30 °C
fitness at 42 °C
hydrophobicity with mutation
1 2 3 mutation 4
L91A
L91W
L91Q
L91D
L91P
V125A
V125W
V125Q
V125D
V125P
I200A
I200W
I200Q
I200D
I200P
V201A
V201W
V201Q
V201D
V201P
0
rpsD (b3296, P0A7V8)
206
high fitness
reduced fitness
low fitness
beta-sheets
alpha-helices
turns
no data / not in library
is in library after recombination
binding sites
active sites
bold verified ts mutant
-3.7 hydrophobicity (Kyte & Doolittle, 1982, J. Mol., 157, 105-132) 3.7

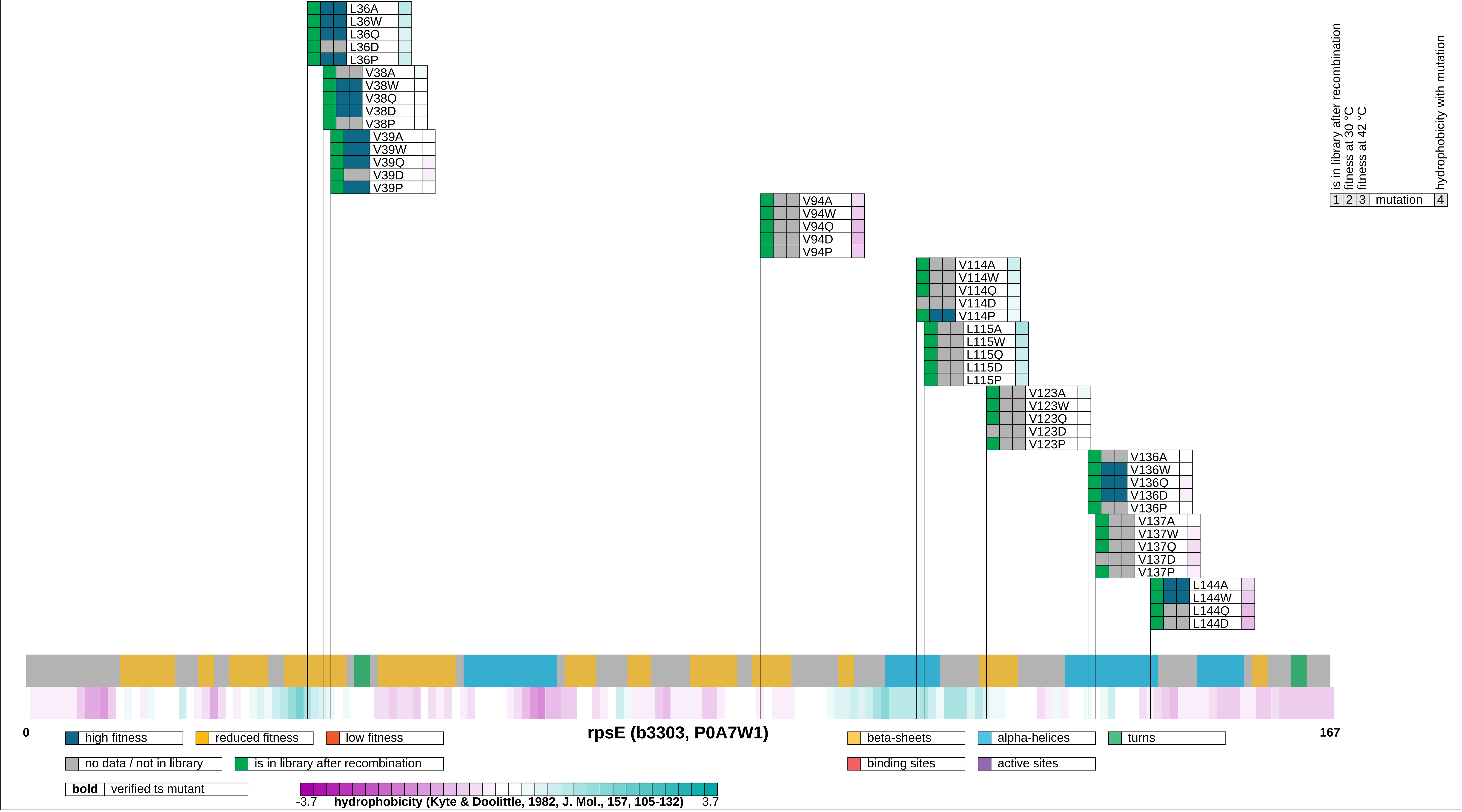

L36A
L36W
L36Q
L36D
L36P
V38A
V38W
V38Q
V38D
V38P
V39A
V39W
V39Q
V39D
V39P
V94A
V94W
V94Q
V94D
V94P
V114A
V114W
V114Q
V114D
V114P
L115A
L115W
L115Q
L115D
L115P
V123A
V123W
V123Q
V123D
V123P
V136A
V136W
V136Q
V136D
V136P
V137A
V137W
V137Q
V137D
V137P
L144A
L144W
L144Q
L144D
is in library after recombination
fitness at 30 °C
fitness at 42 °C
hydrophobicity with mutation
1 2 3 mutation 4
0
rpsE (b3303, P0A7W1)
167
high fitness
reduced fitness
low fitness
beta-sheets
alpha-helices
turns
no data / not in library
is in library after recombination
binding sites
active sites
bold verified ts mutant
-3.7 hydrophobicity (Kyte & Doolittle, 1982, J. Mol., 157, 105-132) 3.7

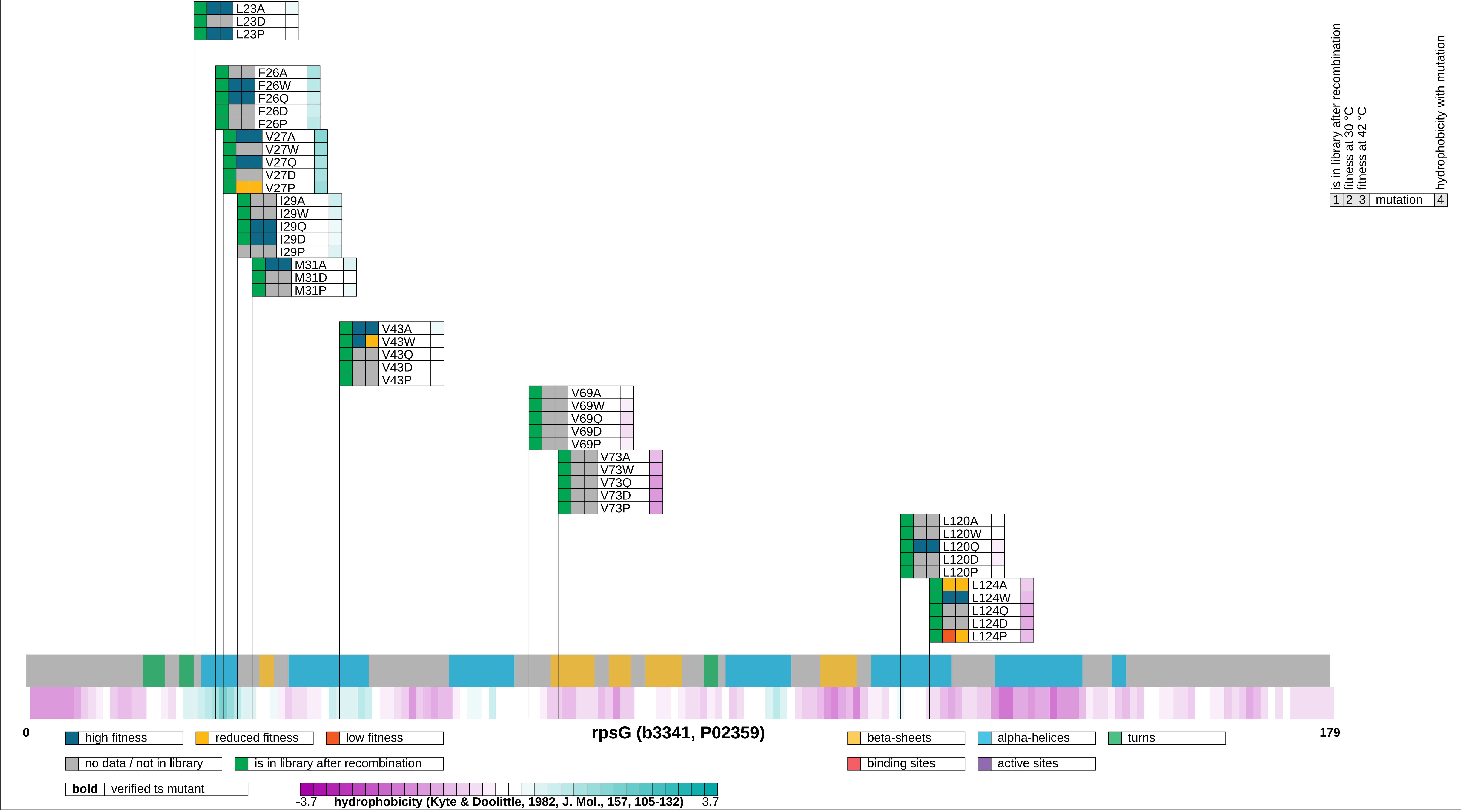
L23A
L23D
L23P
F26A
F26W
F26Q
F26D
F26P
V27A
V27W
V27Q
V27D
V27P
I29A
I29W
I29Q
I29D
I29P
M31A
M31D
M31P
V43A
V43W
V43Q
V43D
V43P
V69A
V69W
V69Q
V69D
V69P
V73A
V73W
V73Q
V73D
V73P
L120A
L120W
L120Q
L120D
L120P
L124A
L124W
L124Q
L124D
L124P
is in library after recombination
fitness at 30 °C
fitness at 42 °C
hydrophobicity with mutation
1 2 3 mutation 4
0
179
rpsG (b3341, P02359)
high fitness
reduced fitness
low fitness
no data / not in library
is in library after recombination
bold verified ts mutant
beta-sheets
alpha-helices
turns
binding sites
active sites
-3.7
hydrophobicity (Kyte & Doolittle, 1982, J. Mol., 157, 105-132)
3.7

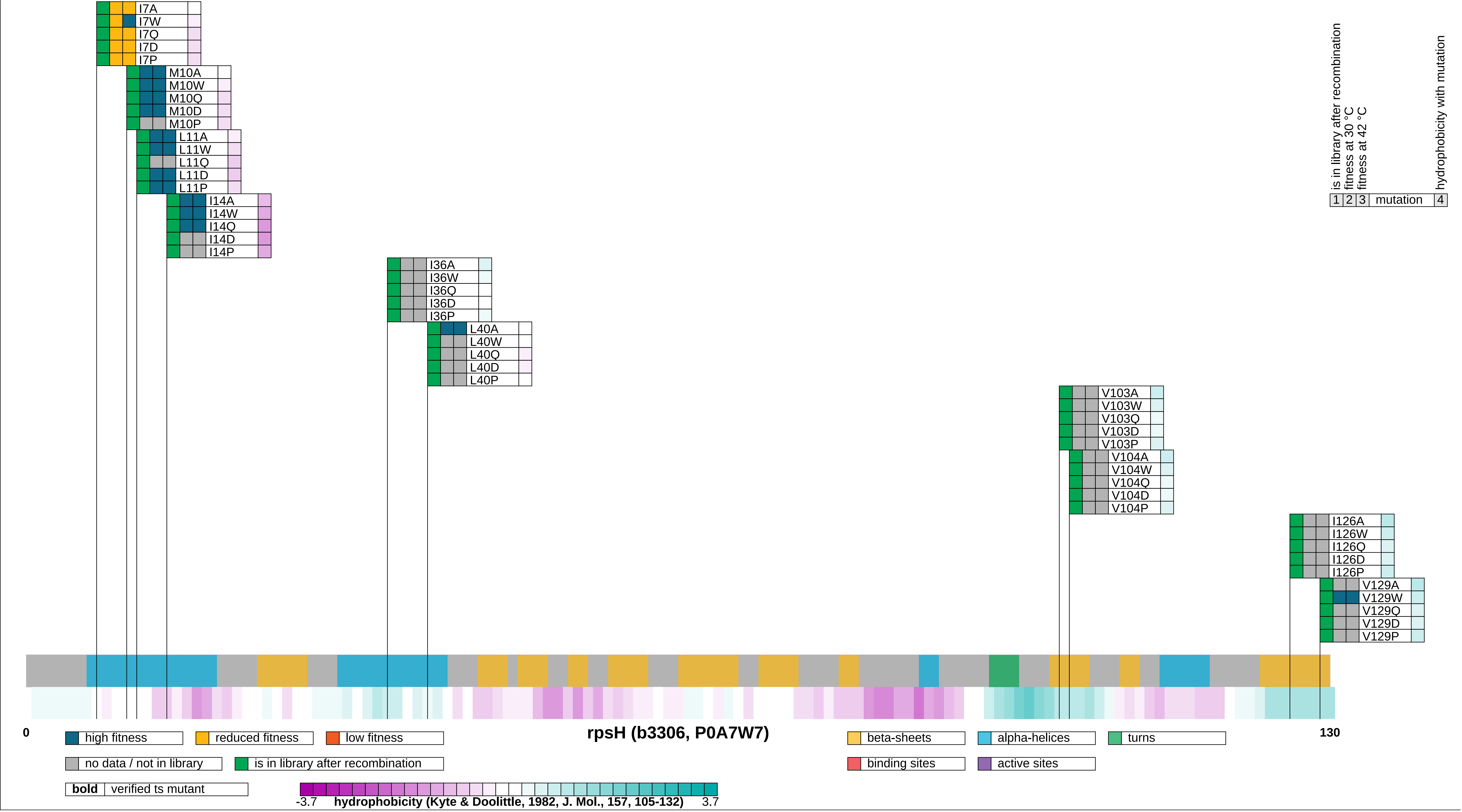

I7A
I7W
I7Q
I7D
I7P
M10A
M10W
M10Q
M10D
M10P
L11A
L11W
L11Q
L11D
L11P
I14A
I14W
I14Q
I14D
I14P
I36A
I36W
I36Q
I36D
I36P
L40A
L40W
L40Q
L40D
L40P
V103A
V103W
V103Q
V103D
V103P
V104A
V104W
V104Q
V104D
V104P
I126A
I126W
I126Q
I126D
I126P
V129A
V129W
V129Q
V129D
V129P
is in library after recombination
fitness at 30 °C
fitness at 42 °C
hydrophobicity with mutation
1
2
3
mutation
4
0
rpsH (b3306, P0A7W7)
130
high fitness
reduced fitness
low fitness
beta-sheets
alpha-helices
turns
no data / not in library
is in library after recombination
binding sites
active sites
bold
verified ts mutant
-3.7
hydrophobicity (Kyte & Doolittle, 1982, J. Mol., 157, 105-132)
3.7

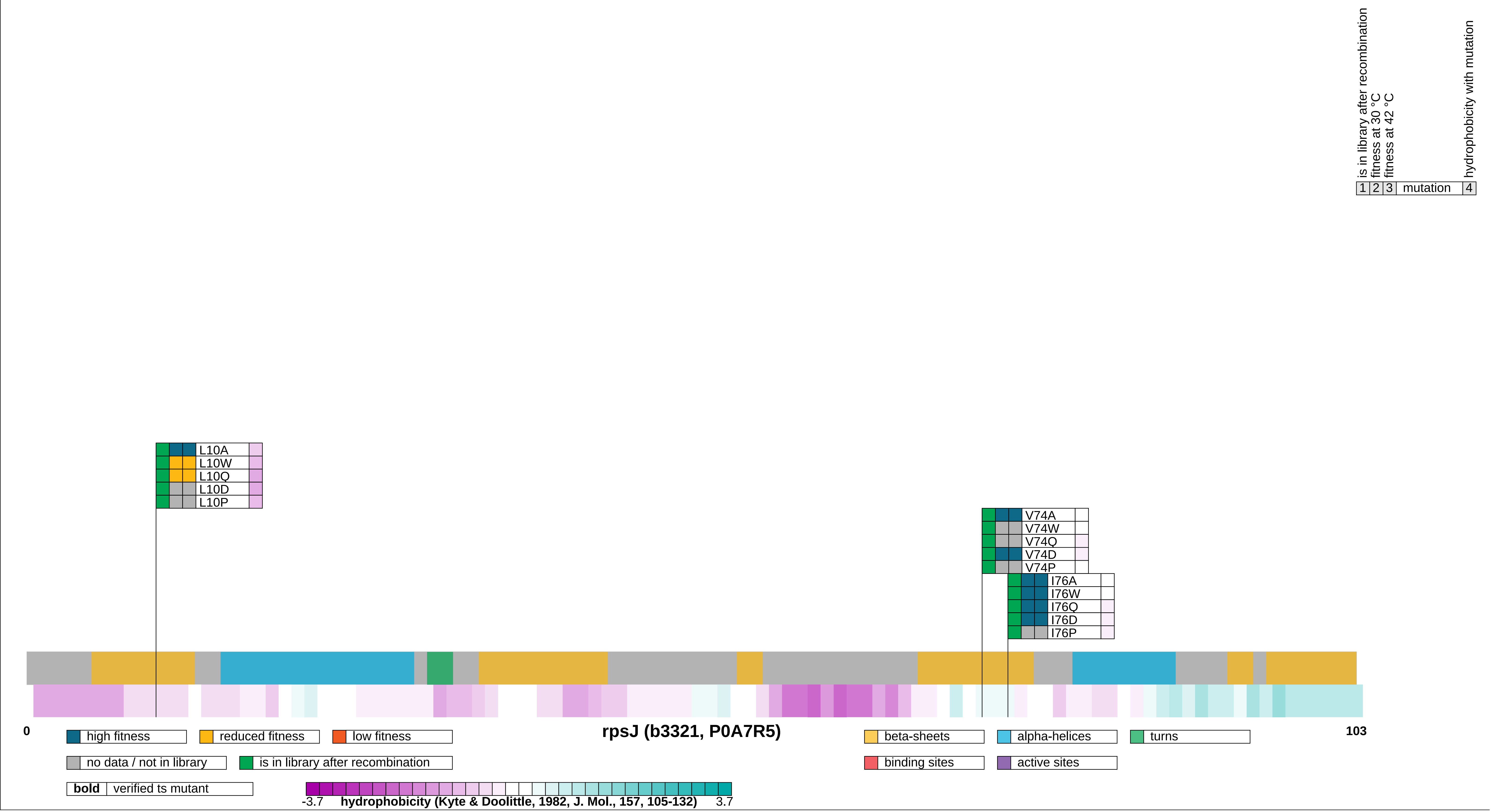
is in library after recombination
fitness at 30 °C
fitness at 42 °C
hydrophobicity with mutation
1 2 3 mutation 4
L10A
L10W
L10Q
L10D
L10P
V74A
V74W
V74Q
V74D
V74P
I76A
I76W
I76Q
I76D
I76P
0
rpsJ (b3321, P0A7R5)
103
high fitness
reduced fitness
low fitness
beta-sheets
alpha-helices
turns
no data / not in library
is in library after recombination
binding sites
active sites
bold verified ts mutant
-3.7 hydrophobicity (Kyte & Doolittle, 1982, J. Mol., 157, 105-132) 3.7

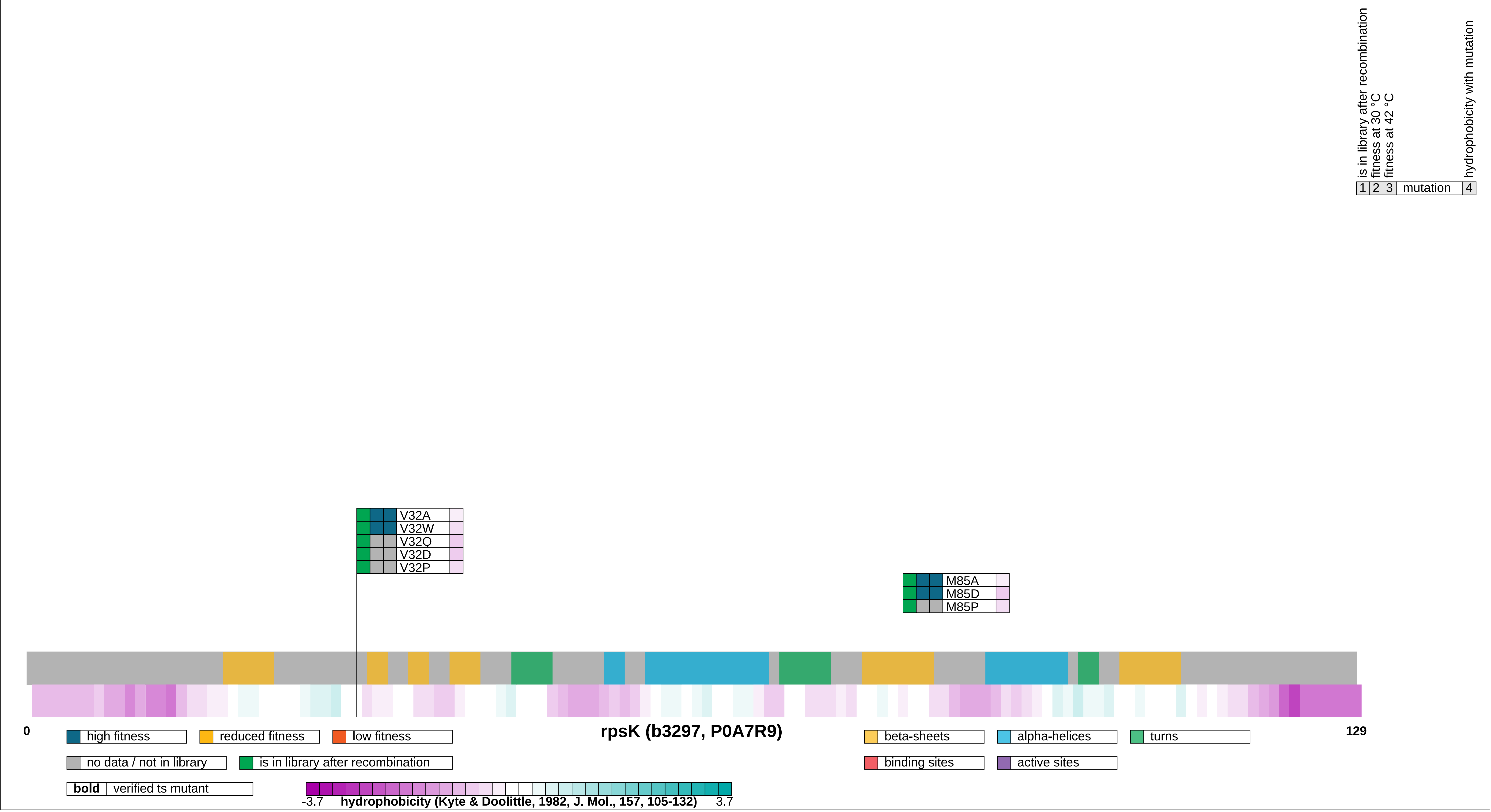
is in library after recombination
fitness at 30 °C
fitness at 42 °C
hydrophobicity with mutation
1 2 3 mutation 4
V32A
V32W
V32Q
V32D
V32P
M85A
M85D
M85P
0
rpsK (b3297, P0A7R9)
129
high fitness
reduced fitness
low fitness
beta-sheets
alpha-helices
turns
no data / not in library
is in library after recombination
binding sites
active sites
bold verified ts mutant
-3.7 hydrophobicity (Kyte & Doolittle, 1982, J. Mol., 157, 105-132) 3.7

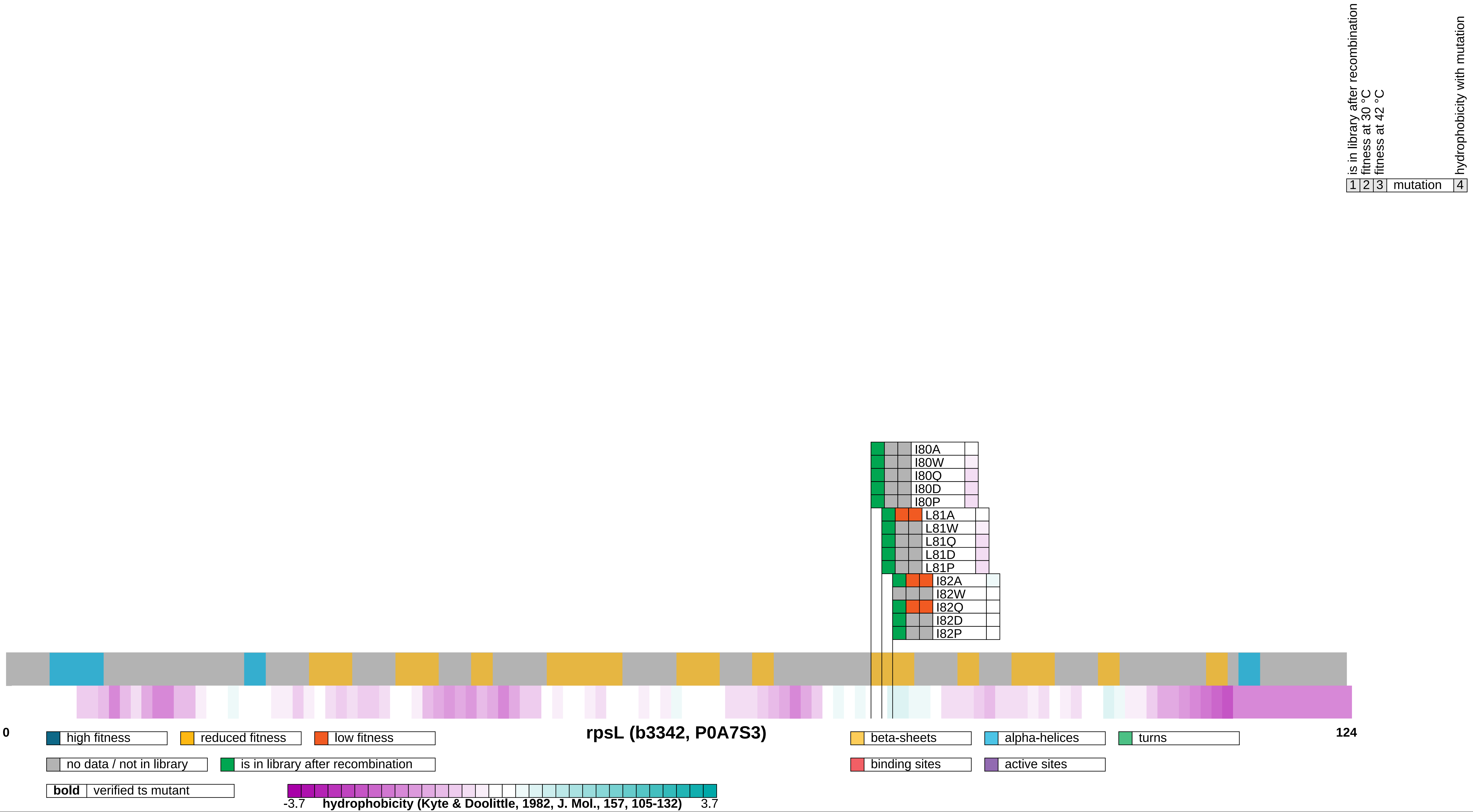
is in library after recombination
fitness at 30 °C
fitness at 42 °C
hydrophobicity with mutation
1 2 3 mutation 4
I80A
I80W
I80Q
I80D
I80P
L81A
L81W
L81Q
L81D
L81P
I82A
I82W
I82Q
I82D
I82P
0
rpsL (b3342, P0A7S3)
124
high fitness
reduced fitness
low fitness
beta-sheets
alpha-helices
turns
no data / not in library
is in library after recombination
binding sites
active sites
bold verified ts mutant
-3.7
hydrophobicity (Kyte & Doolittle, 1982, J. Mol., 157, 105-132)
3.7

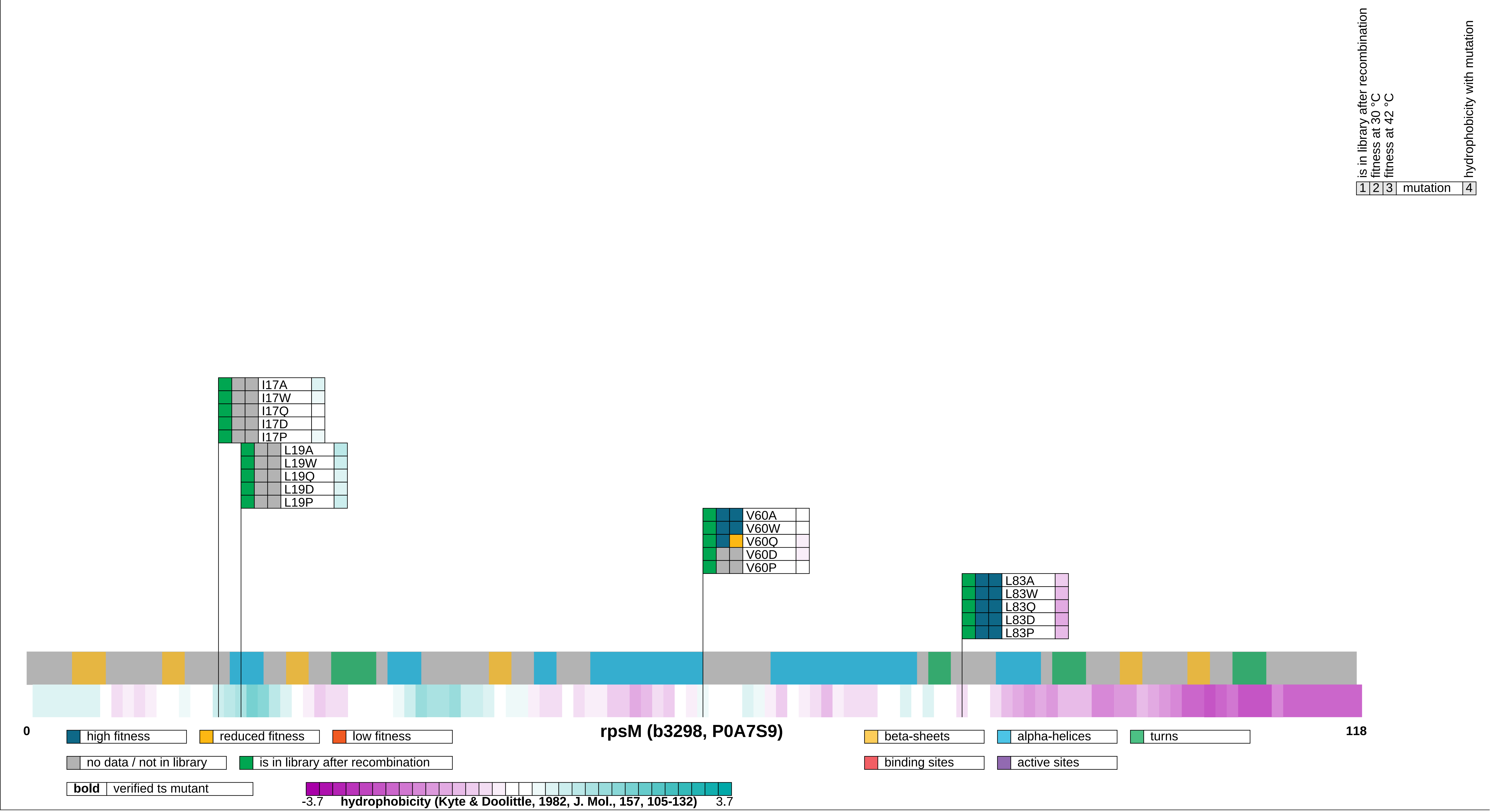

is in library after recombination
fitness at 30 °C
fitness at 42 °C
hydrophobicity with mutation
1 2 3 mutation 4
I17A
I17W
I17Q
I17D
I17P
L19A
L19W
L19Q
L19D
L19P
V60A
V60W
V60Q
V60D
V60P
L83A
L83W
L83Q
L83D
L83P
0
rpsM (b3298, P0A7S9)
118
high fitness
reduced fitness
low fitness
beta-sheets
alpha-helices
turns
no data / not in library
is in library after recombination
binding sites
active sites
bold verified ts mutant
-3.7
hydrophobicity (Kyte & Doolittle, 1982, J. Mol., 157, 105-132)
3.7

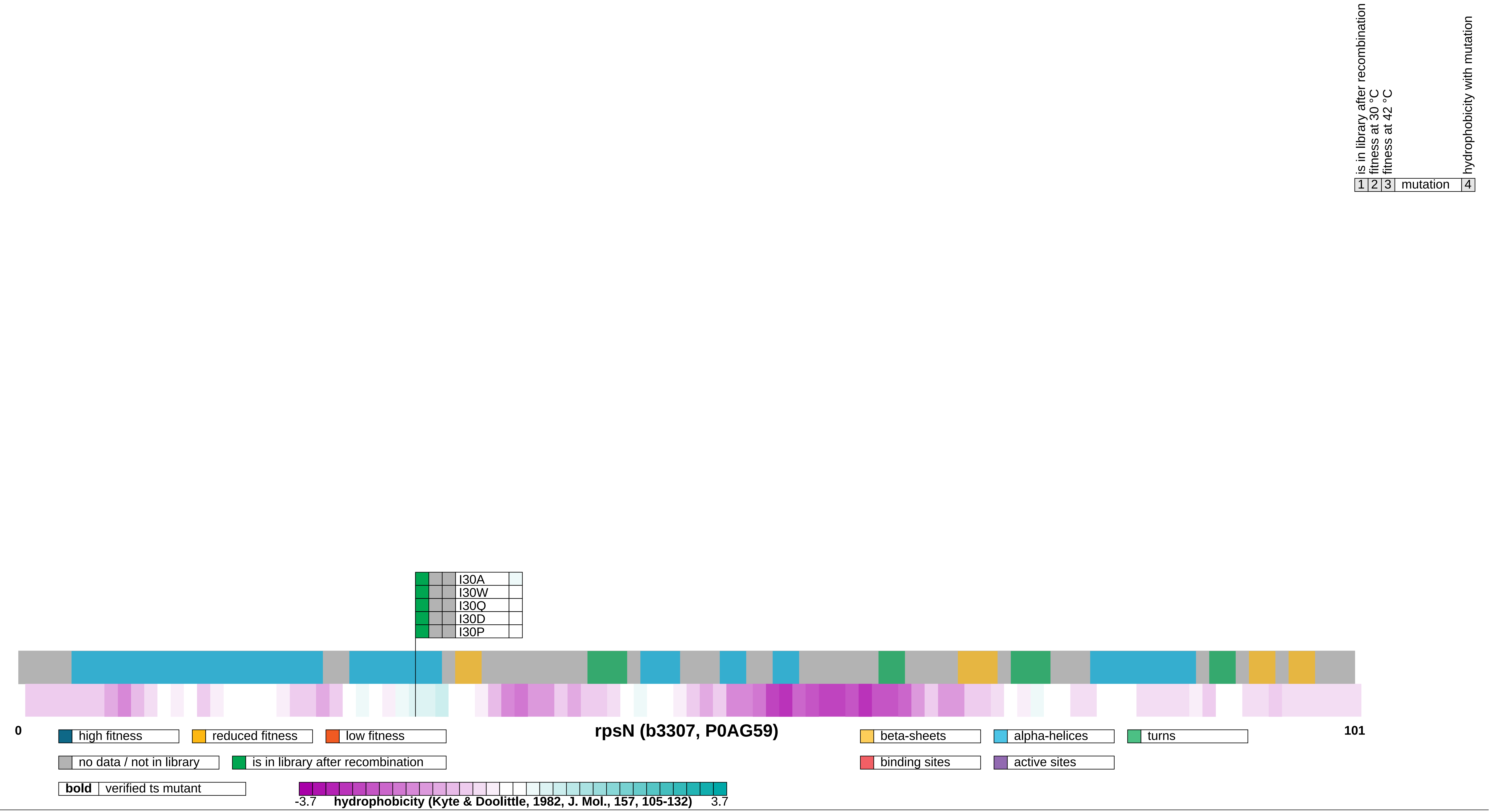
is in library after recombination
fitness at 30 °C
fitness at 42 °C
hydrophobicity with mutation
1 2 3 mutation 4
I30A
I30W
I30Q
I30D
I30P
0
rpsN (b3307, P0AG59)
101
high fitness
reduced fitness
low fitness
no data / not in library
is in library after recombination
bold verified ts mutant
beta-sheets
alpha-helices
turns
binding sites
active sites
-3.7
hydrophobicity (Kyte & Doolittle, 1982, J. Mol., 157, 105-132)
3.7

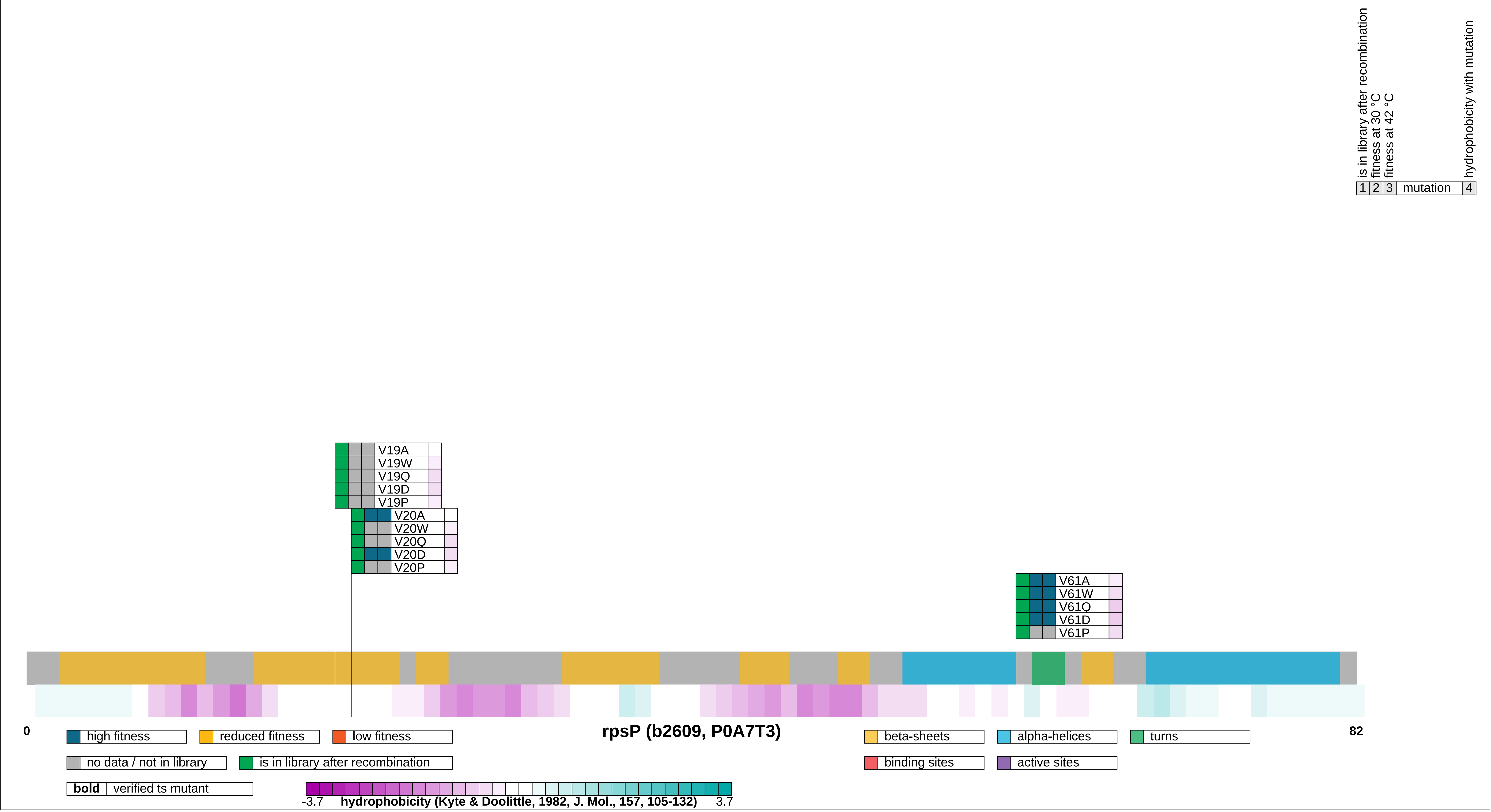

is in library after recombination
fitness at 30 °C
fitness at 42 °C
hydrophobicity with mutation
1 2 3 mutation 4
V19A
V19W
V19Q
V19D
V19P
V20A
V20W
V20Q
V20D
V20P
V61A
V61W
V61Q
V61D
V61P
0
rpsP (b2609, P0A7T3)
82
high fitness
reduced fitness
low fitness
beta-sheets
alpha-helices
turns
no data / not in library
is in library after recombination
binding sites
active sites
bold verified ts mutant
-3.7 hydrophobicity (Kyte & Doolittle, 1982, J. Mol., 157, 105-132) 3.7

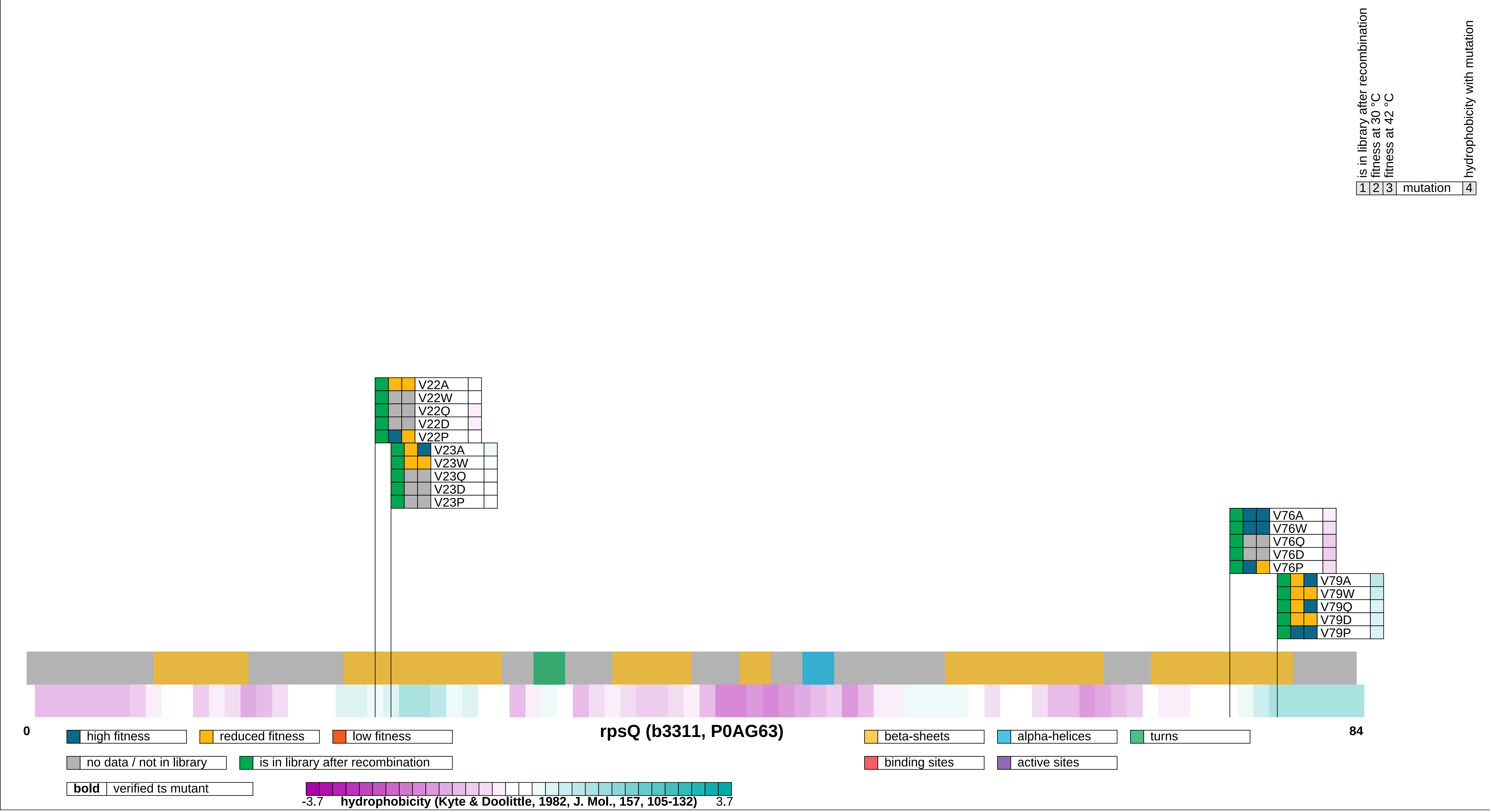

is in library after recombination
fitness at 30 °C
fitness at 42 °C
hydrophobicity with mutation
1 2 3 mutation 4
V22A
V22W
V22Q
V22D
V22P
V23A
V23W
V23Q
V23D
V23P
V76A
V76W
V76Q
V76D
V76P
V79A
V79W
V79Q
V79D
V79P
0
rpsQ (b3311, P0AG63)
84
high fitness
reduced fitness
low fitness
beta-sheets
alpha-helices
turns
no data / not in library
is in library after recombination
binding sites
active sites
bold verified ts mutant
-3.7 hydrophobicity (Kyte & Doolittle, 1982, J. Mol., 157, 105-132) 3.7

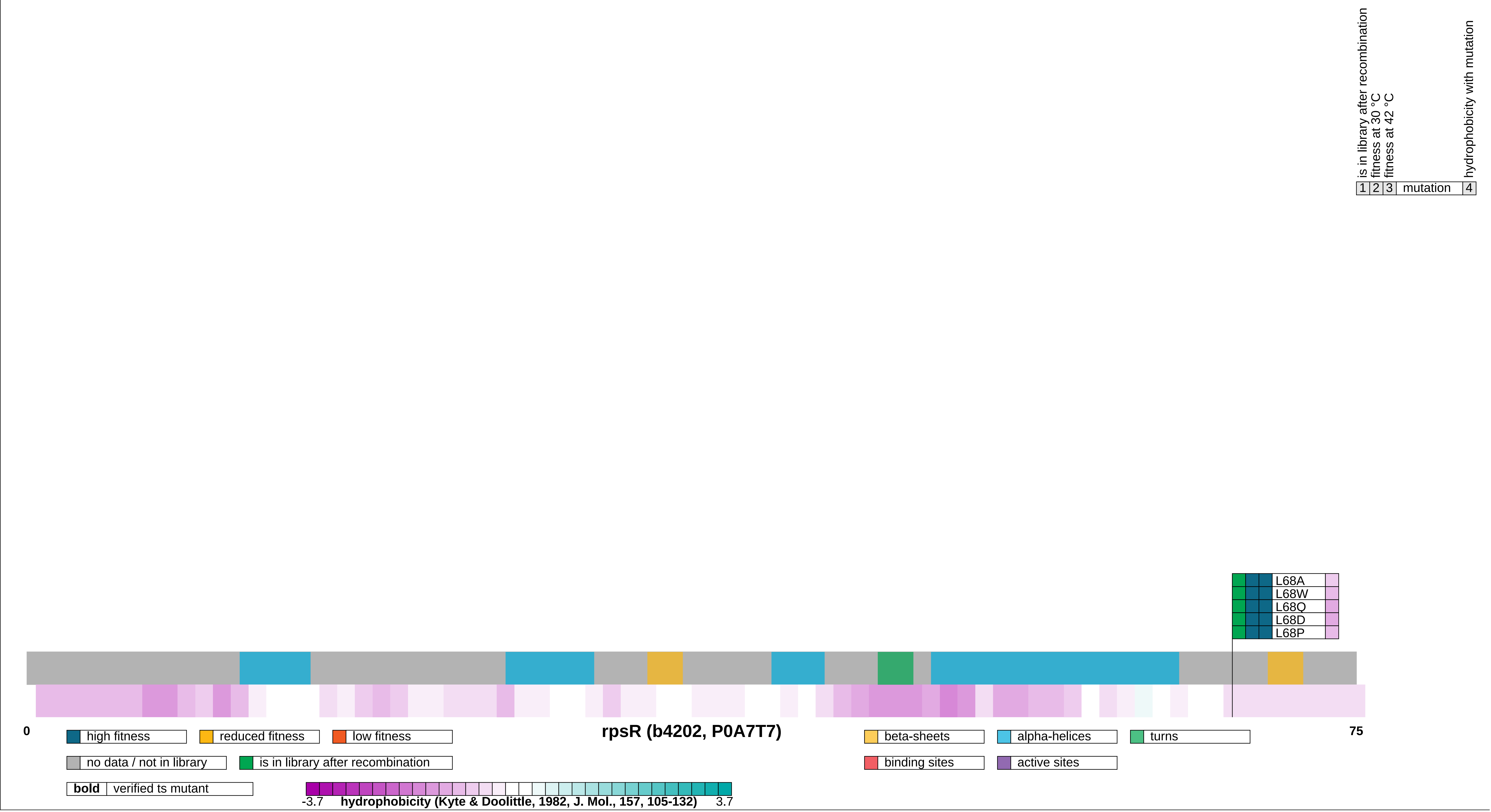

is in library after recombination
fitness at 30 °C
fitness at 42 °C
hydrophobicity with mutation
1 2 3 mutation 4
L68A
L68W
L68Q
L68D
L68P
0
rpsR (b4202, P0A7T7)
75
high fitness
reduced fitness
low fitness
beta-sheets
alpha-helices
turns
no data / not in library
is in library after recombination
binding sites
active sites
bold verified ts mutant
-3.7
hydrophobicity (Kyte & Doolittle, 1982, J. Mol., 157, 105-132)
3.7

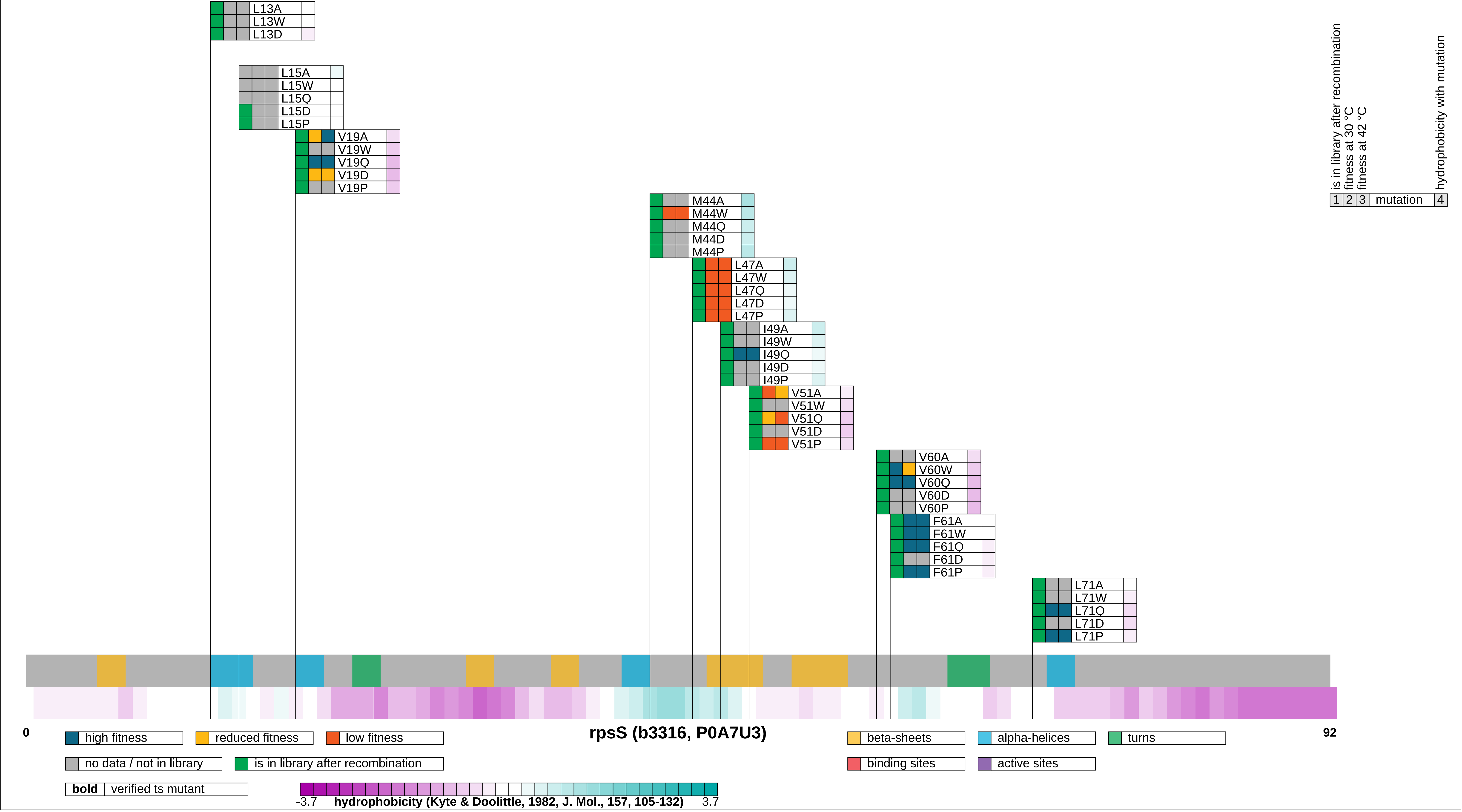

L13A
L13W
L13D
L15A
L15W
L15Q
L15D
L15P
V19A
V19W
V19Q
V19D
V19P
M44A
M44W
M44Q
M44D
M44P
L47A
L47W
L47Q
L47D
L47P
I49A
I49W
I49Q
I49D
I49P
V51A
V51W
V51Q
V51D
V51P
V60A
V60W
V60Q
V60D
V60P
F61A
F61W
F61Q
F61D
F61P
L71A
L71W
L71Q
L71D
L71P
is in library after recombination
fitness at 30 °C
fitness at 42 °C
hydrophobicity with mutation
1
2
3
mutation
4
0
rpsS (b3316, P0A7U3)
92
high fitness
reduced fitness
low fitness
beta-sheets
alpha-helices
turns
no data / not in library
is in library after recombination
binding sites
active sites
bold
verified ts mutant
-3.7
hydrophobicity (Kyte & Doolittle, 1982, J. Mol., 157, 105-132)
3.7

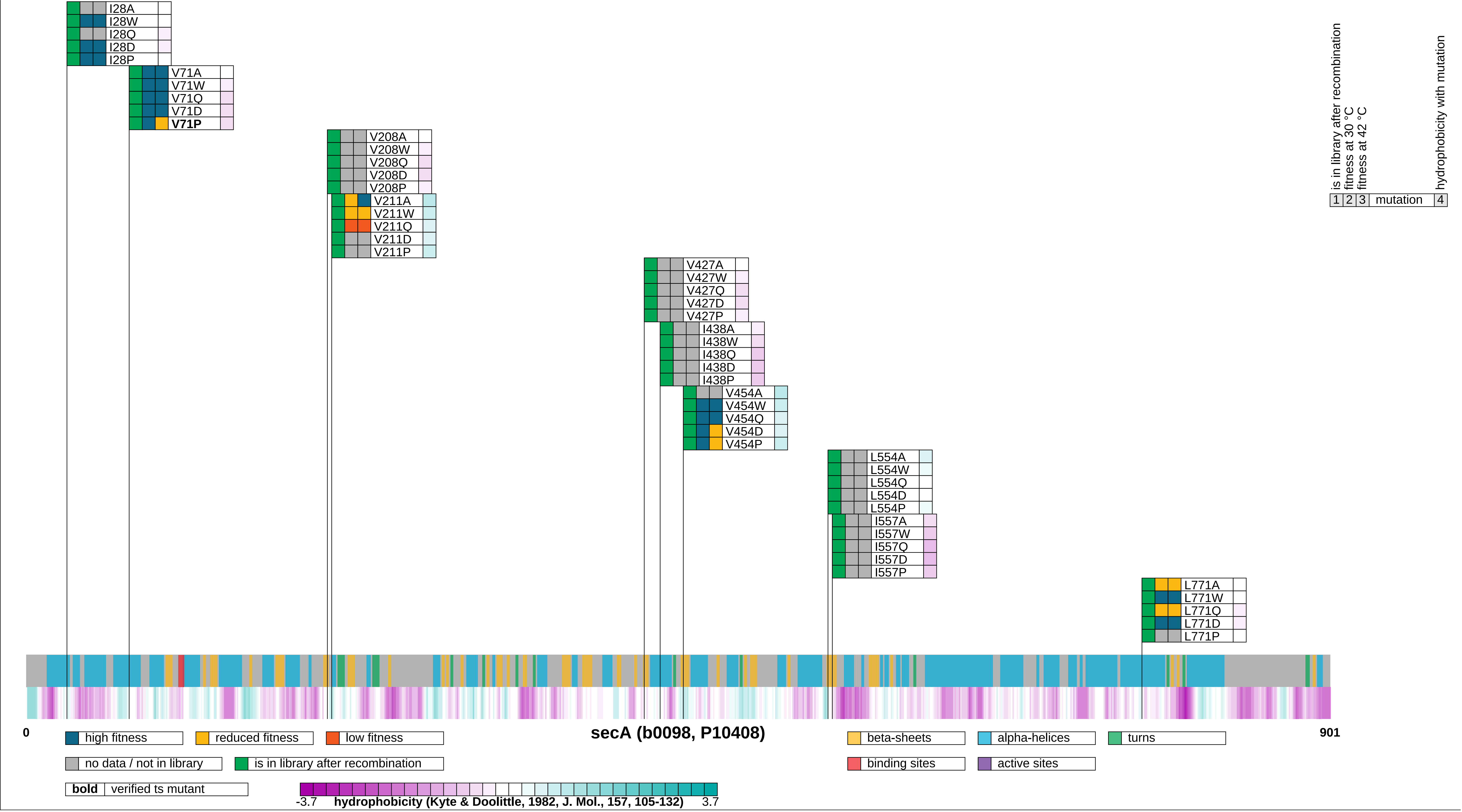
I28A
I28W
I28Q
I28D
I28P
V71A
V71W
V71Q
V71D
V71P
V208A
V208W
V208Q
V208D
V208P
V211A
V211W
V211Q
V211D
V211P
V427A
V427W
V427Q
V427D
V427P
I438A
I438W
I438Q
I438D
I438P
V454A
V454W
V454Q
V454D
V454P
L554A
L554W
L554Q
L554D
L554P
I557A
I557W
I557Q
I557D
I557P
L771A
L771W
L771Q
L771D
L771P
is in library after recombination
fitness at 30 °C
fitness at 42 °C
hydrophobicity with mutation
1 2 3 mutation 4
0
secA (b0098, P10408)
901
high fitness
reduced fitness
low fitness
beta-sheets
alpha-helices
turns
no data / not in library
is in library after recombination
binding sites
active sites
bold verified ts mutant
-3.7 hydrophobicity (Kyte & Doolittle, 1982, J. Mol., 157, 105-132) 3.7

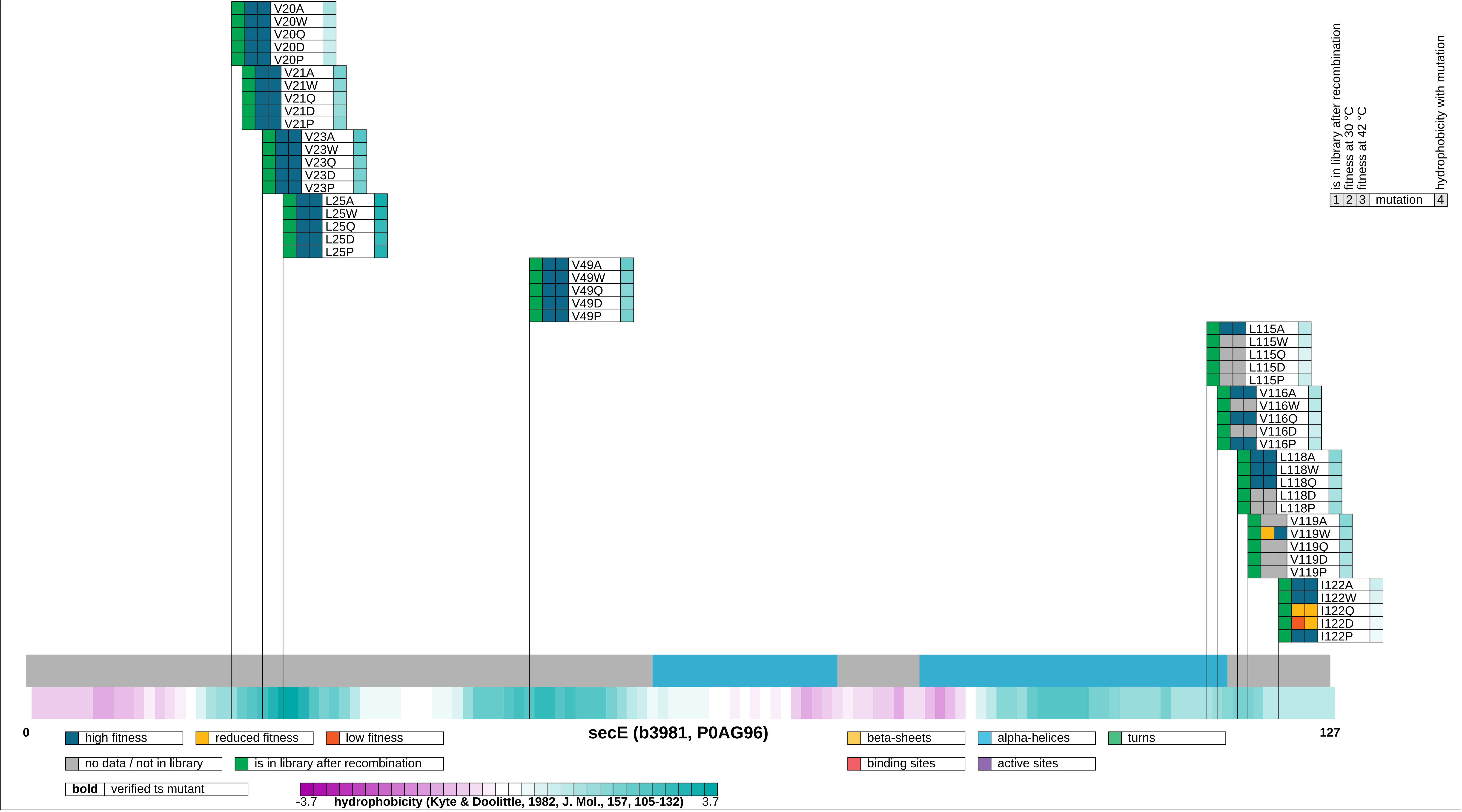

V20A
V20W
V20Q
V20D
V20P
V21A
V21W
V21Q
V21D
V21P
V23A
V23W
V23Q
V23D
V23P
L25A
L25W
L25Q
L25D
L25P
V49A
V49W
V49Q
V49D
V49P
L115A
L115W
L115Q
L115D
L115P
V116A
V116W
V116Q
V116D
V116P
L118A
L118W
L118Q
L118D
L118P
V119A
V119W
V119Q
V119D
V119P
I122A
I122W
I122Q
I122D
I122P
is in library after recombination
fitness at 30 °C
fitness at 42 °C
hydrophobicity with mutation
1 2 3 mutation 4
0
secE (b3981, P0AG96)
127
high fitness
reduced fitness
low fitness
beta-sheets
alpha-helices
turns
no data / not in library
is in library after recombination
binding sites
active sites
bold verified ts mutant
-3.7
hydrophobicity (Kyte & Doolittle, 1982, J. Mol., 157, 105-132)
3.7

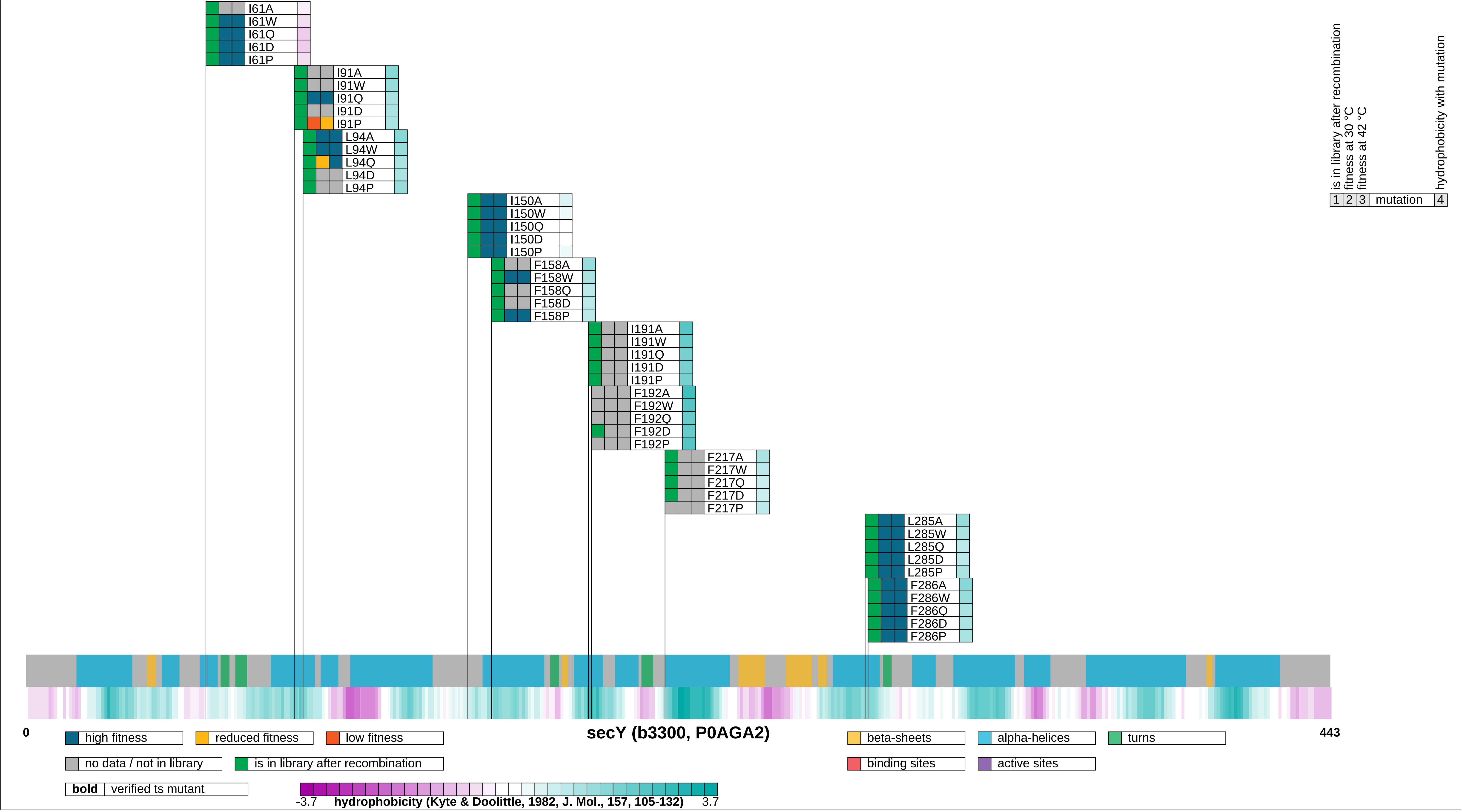

I61A
I61W
I61Q
I61D
I61P
I91A
I91W
I91Q
I91D
I91P
L94A
L94W
L94Q
L94D
L94P
I150A
I150W
I150Q
I150D
I150P
F158A
F158W
F158Q
F158D
F158P
I191A
I191W
I191Q
I191D
I191P
F192A
F192W
F192Q
F192D
F192P
F217A
F217W
F217Q
F217D
F217P
L285A
L285W
L285Q
L285D
L285P
F286A
F286W
F286Q
F286D
F286P
is in library after recombination
fitness at 30 °C
fitness at 42 °C
hydrophobicity with mutation
1 2 3 mutation 4
0
secY (b3300, P0AGA2)
443
high fitness
reduced fitness
low fitness
beta-sheets
alpha-helices
turns
no data / not in library
is in library after recombination
binding sites
active sites
bold verified ts mutant
-3.7 hydrophobicity (Kyte & Doolittle, 1982, J. Mol., 157, 105-132) 3.7

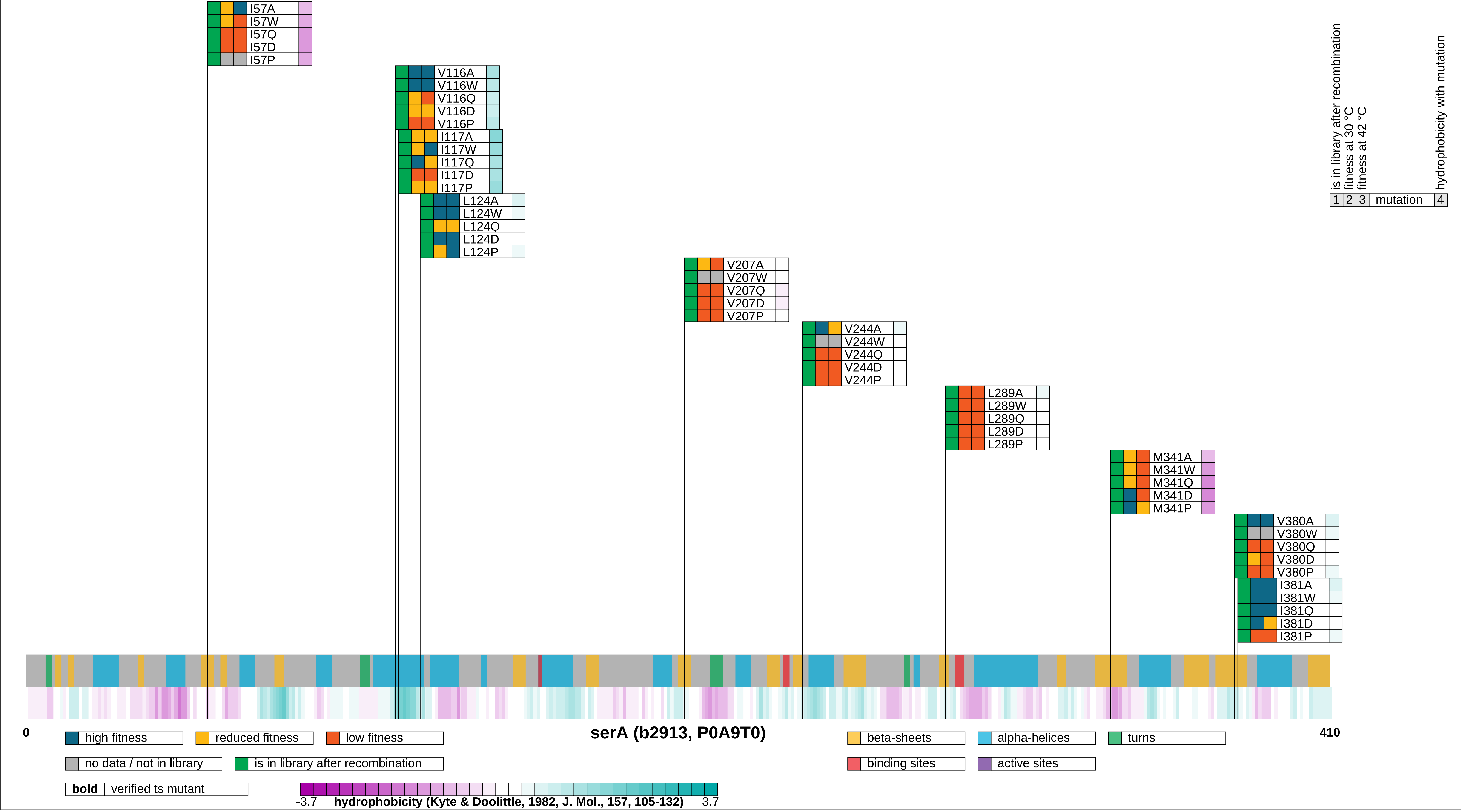
I57A
I57W
I57Q
I57D
I57P
V116A
V116W
V116Q
V116D
V116P
I117A
I117W
I117Q
I117D
I117P
L124A
L124W
L124Q
L124D
L124P
V207A
V207W
V207Q
V207D
V207P
V244A
V244W
V244Q
V244D
V244P
L289A
L289W
L289Q
L289D
L289P
M341A
M341W
M341Q
M341D
M341P
V380A
V380W
V380Q
V380D
V380P
I381A
I381W
I381Q
I381D
I381P
is in library after recombination
fitness at 30 °C
fitness at 42 °C
hydrophobicity with mutation
1 2 3 mutation 4
0
serA (b2913, P0A9T0)
410
high fitness
reduced fitness
low fitness
no data / not in library
is in library after recombination
bold verified ts mutant
beta-sheets
alpha-helices
turns
binding sites
active sites
-3.7
hydrophobicity (Kyte & Doolittle, 1982, J. Mol., 157, 105-132)
3.7

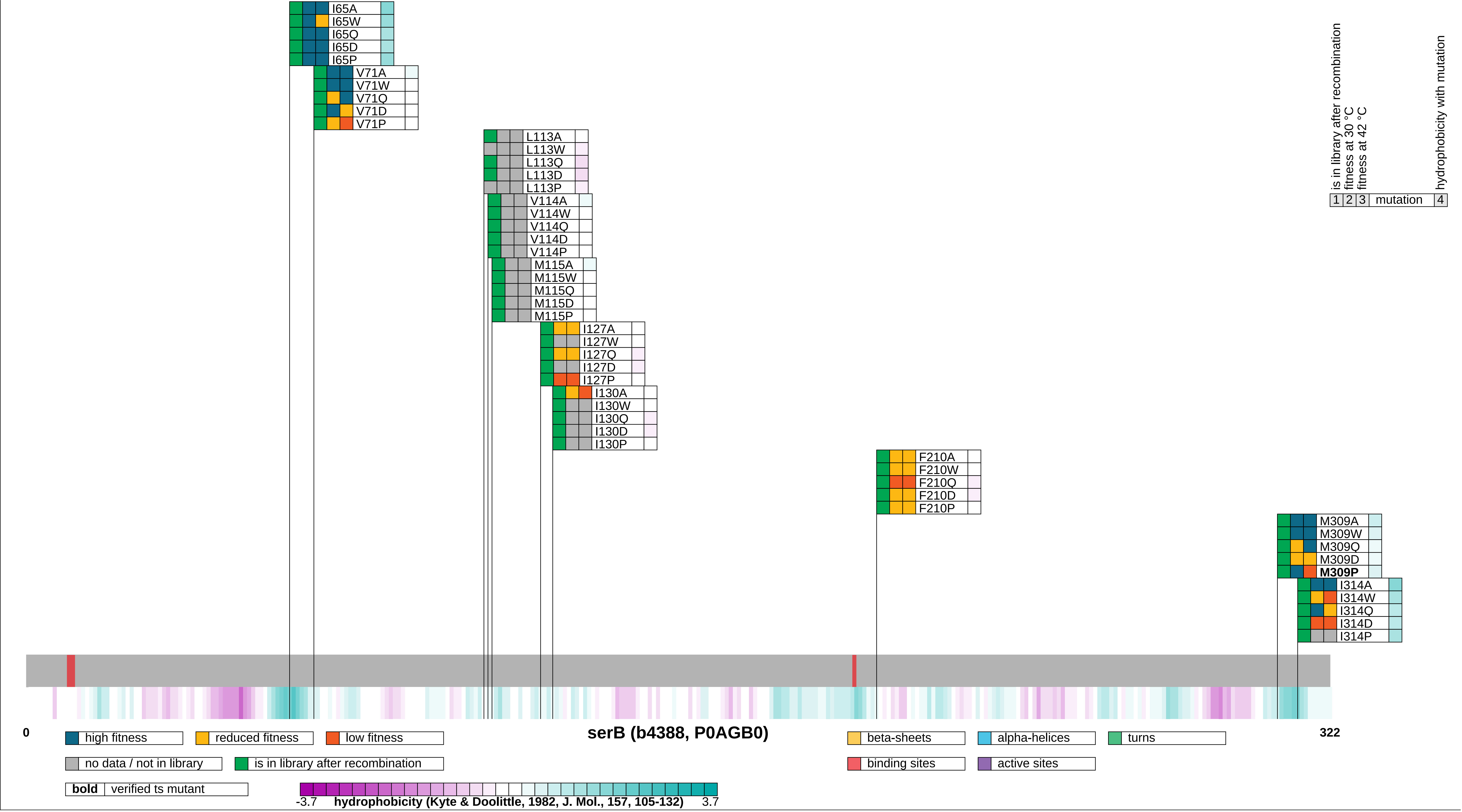

I65A
I65W
I65Q
I65D
I65P
V71A
V71W
V71Q
V71D
V71P
L113A
L113W
L113Q
L113D
L113P
V114A
V114W
V114Q
V114D
V114P
M115A
M115W
M115Q
M115D
M115P
I127A
I127W
I127Q
I127D
I127P
I130A
I130W
I130Q
I130D
I130P
F210A
F210W
F210Q
F210D
F210P
M309A
M309W
M309Q
M309D
M309P
I314A
I314W
I314Q
I314D
I314P
is in library after recombination
fitness at 30 °C
fitness at 42 °C
hydrophobicity with mutation
1 2 3 mutation 4
0
serB (b4388, P0AGB0)
322
high fitness
reduced fitness
low fitness
beta-sheets
alpha-helices
turns
no data / not in library
is in library after recombination
binding sites
active sites
bold verified ts mutant
-3.7 hydrophobicity (Kyte & Doolittle, 1982, J. Mol., 157, 105-132) 3.7

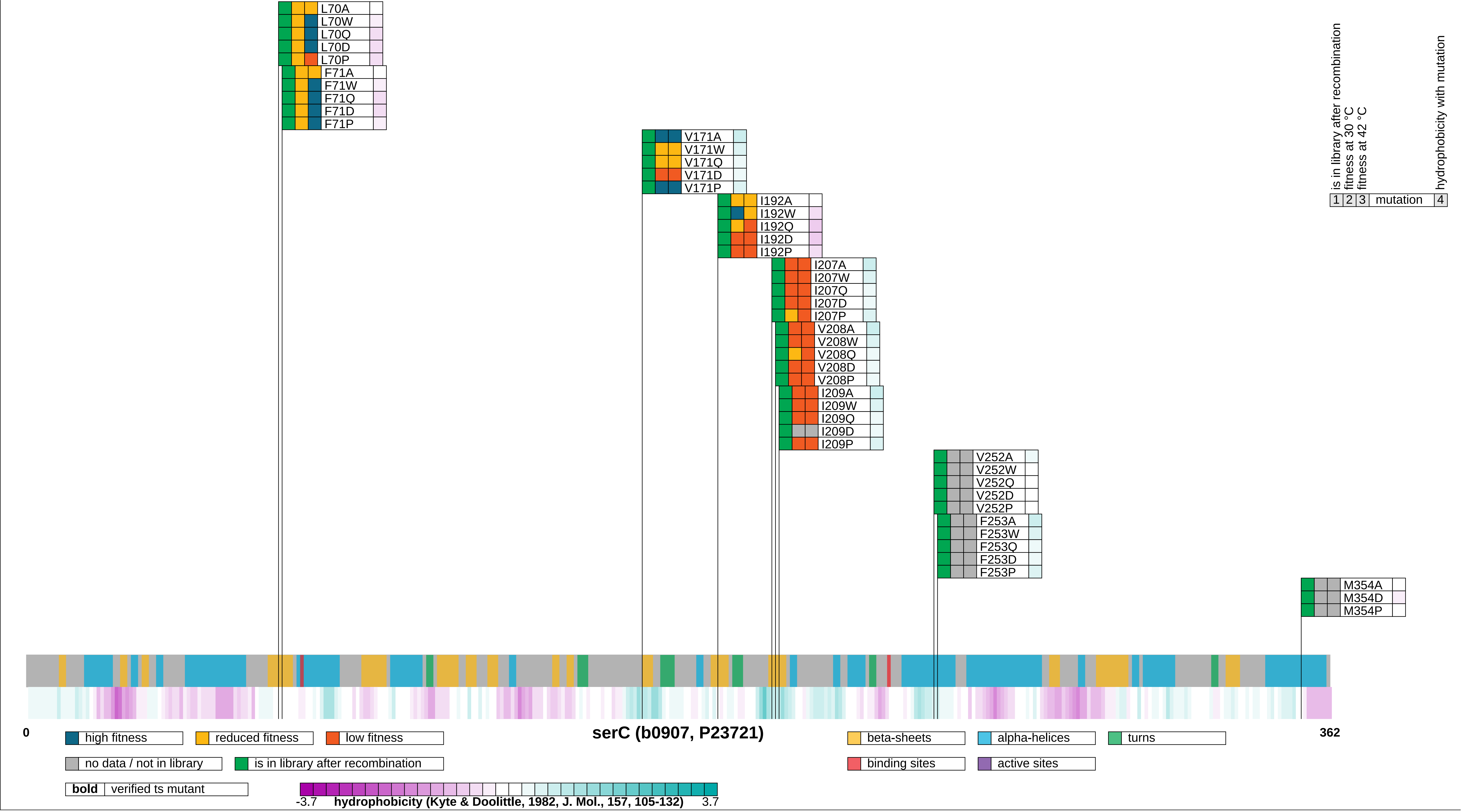
L70A
L70W
L70Q
L70D
L70P
F71A
F71W
F71Q
F71D
F71P
V171A
V171W
V171Q
V171D
V171P
I192A
I192W
I192Q
I192D
I192P
I207A
I207W
I207Q
I207D
I207P
V208A
V208W
V208Q
V208D
V208P
I209A
I209W
I209Q
I209D
I209P
V252A
V252W
V252Q
V252D
V252P
F253A
F253W
F253Q
F253D
F253P
M354A
M354D
M354P
is in library after recombination
fitness at 30 °C
fitness at 42 °C
hydrophobicity with mutation
1
2
3
mutation
4
0
serC (b0907, P23721)
362
high fitness
reduced fitness
low fitness
beta-sheets
alpha-helices
turns
no data / not in library
is in library after recombination
binding sites
active sites
bold
verified ts mutant
-3.7
hydrophobicity (Kyte & Doolittle, 1982, J. Mol., 157, 105-132)
3.7

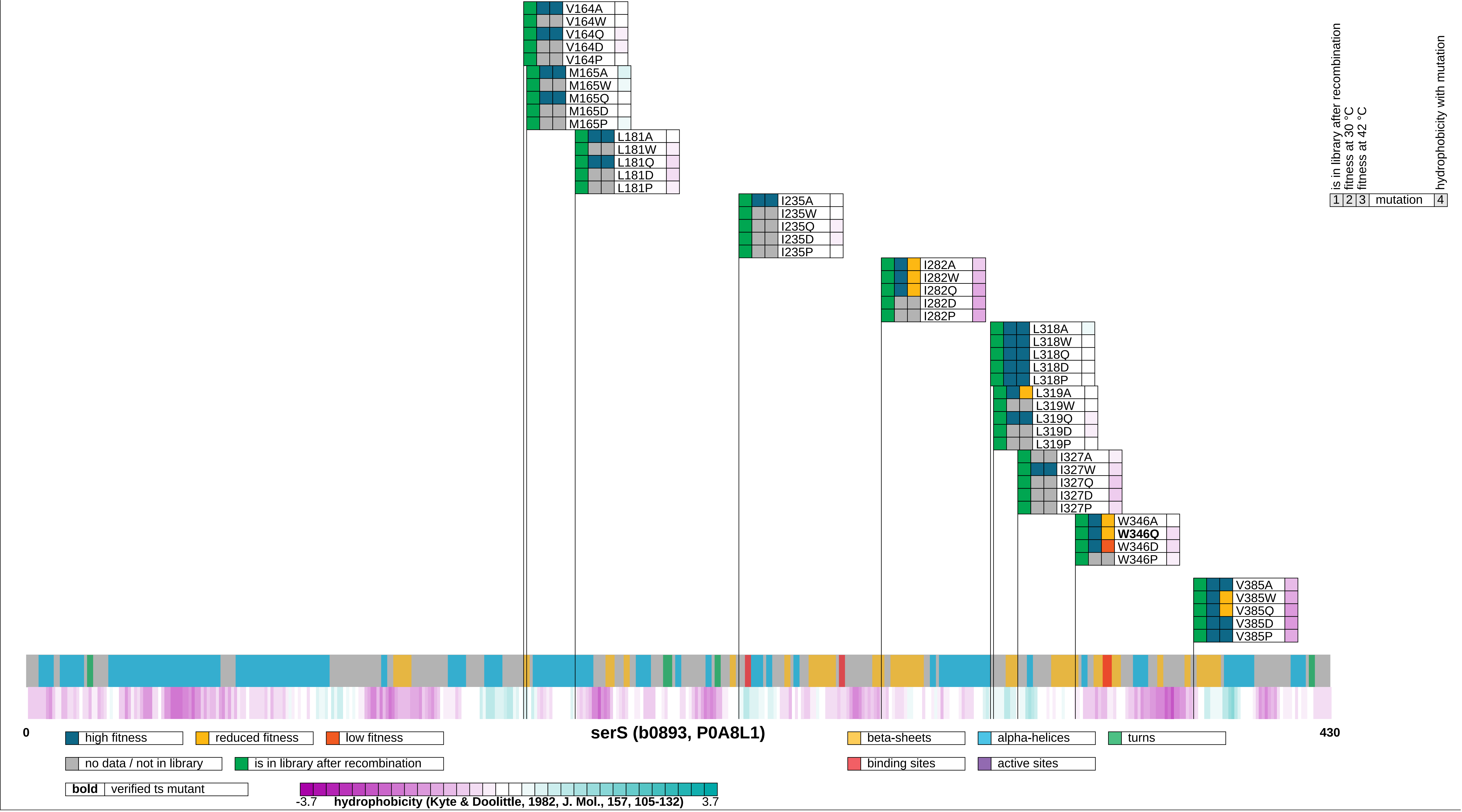
V164A
V164W
V164Q
V164D
V164P
M165A
M165W
M165Q
M165D
M165P
L181A
L181W
L181Q
L181D
L181P
I235A
I235W
I235Q
I235D
I235P
I282A
I282W
I282Q
I282D
I282P
L318A
L318W
L318Q
L318D
L318P
L319A
L319W
L319Q
L319D
L319P
I327A
I327W
I327Q
I327D
I327P
W346A
W346Q
W346D
W346P
V385A
V385W
V385Q
V385D
V385P
is in library after recombination
fitness at 30 °C
fitness at 42 °C
hydrophobicity with mutation
1 2 3 mutation 4
0
serS (b0893, P0A8L1)
430
high fitness
reduced fitness
low fitness
beta-sheets
alpha-helices
turns
no data / not in library
is in library after recombination
binding sites
active sites
bold verified ts mutant
-3.7
hydrophobicity (Kyte & Doolittle, 1982, J. Mol., 157, 105-132)
3.7

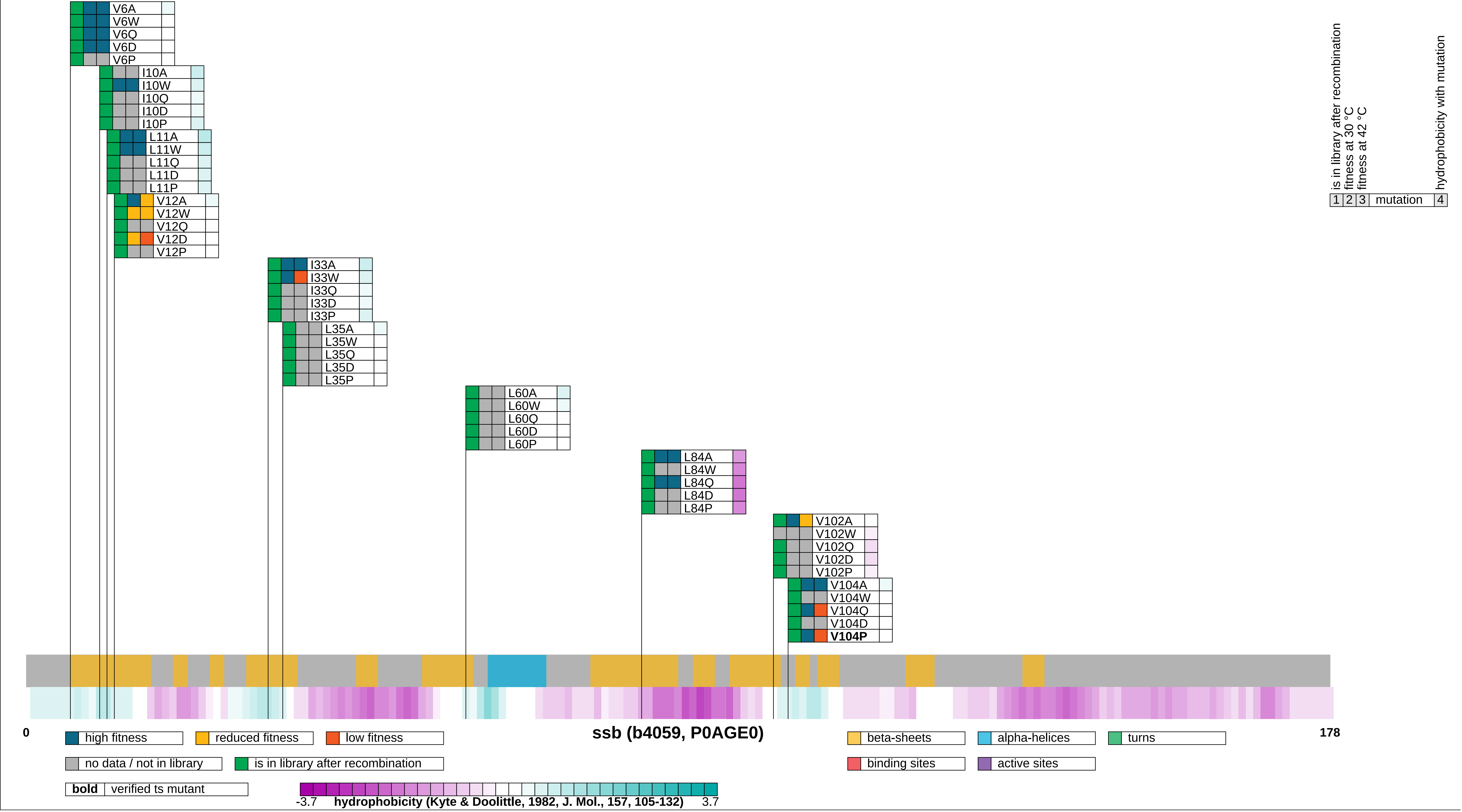

V6A
V6W
V6Q
V6D
V6P
I10A
I10W
I10Q
I10D
I10P
L11A
L11W
L11Q
L11D
L11P
V12A
V12W
V12Q
V12D
V12P
I33A
I33W
I33Q
I33D
I33P
L35A
L35W
L35Q
L35D
L35P
L60A
L60W
L60Q
L60D
L60P
L84A
L84W
L84Q
L84D
L84P
V102A
V102W
V102Q
V102D
V102P
V104A
V104W
V104Q
V104D
V104P
is in library after recombination
fitness at 30 °C
fitness at 42 °C
hydrophobicity with mutation
1 2 3 mutation 4
0
ssb (b4059, P0AGE0)
178
high fitness
reduced fitness
low fitness
beta-sheets
alpha-helices
turns
no data / not in library
is in library after recombination
binding sites
active sites
bold verified ts mutant
-3.7
hydrophobicity (Kyte & Doolittle, 1982, J. Mol., 157, 105-132)
3.7

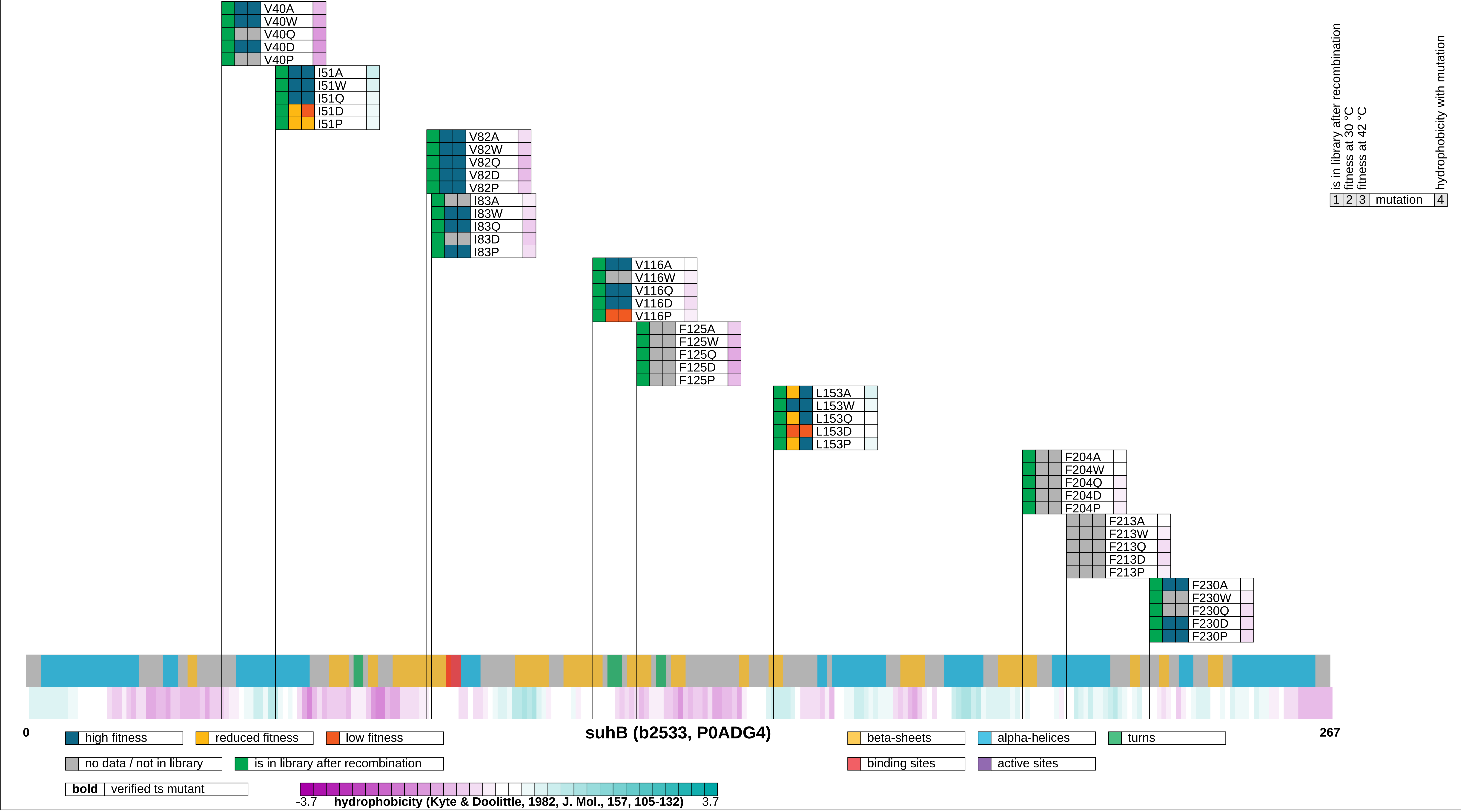
V40A
V40W
V40Q
V40D
V40P
I51A
I51W
I51Q
I51D
I51P
V82A
V82W
V82Q
V82D
V82P
I83A
I83W
I83Q
I83D
I83P
V116A
V116W
V116Q
V116D
V116P
F125A
F125W
F125Q
F125D
F125P
L153A
L153W
L153Q
L153D
L153P
F204A
F204W
F204Q
F204D
F204P
F213A
F213W
F213Q
F213D
F213P
F230A
F230W
F230Q
F230D
F230P
is in library after recombination
fitness at 30 °C
fitness at 42 °C
hydrophobicity with mutation
1 2 3 mutation 4
0
suhB (b2533, P0ADG4)
267
high fitness
reduced fitness
low fitness
no data / not in library
is in library after recombination
bold verified ts mutant
beta-sheets
alpha-helices
turns
binding sites
active sites
-3.7 hydrophobicity (Kyte & Doolittle, 1982, J. Mol., 157, 105-132) 3.7

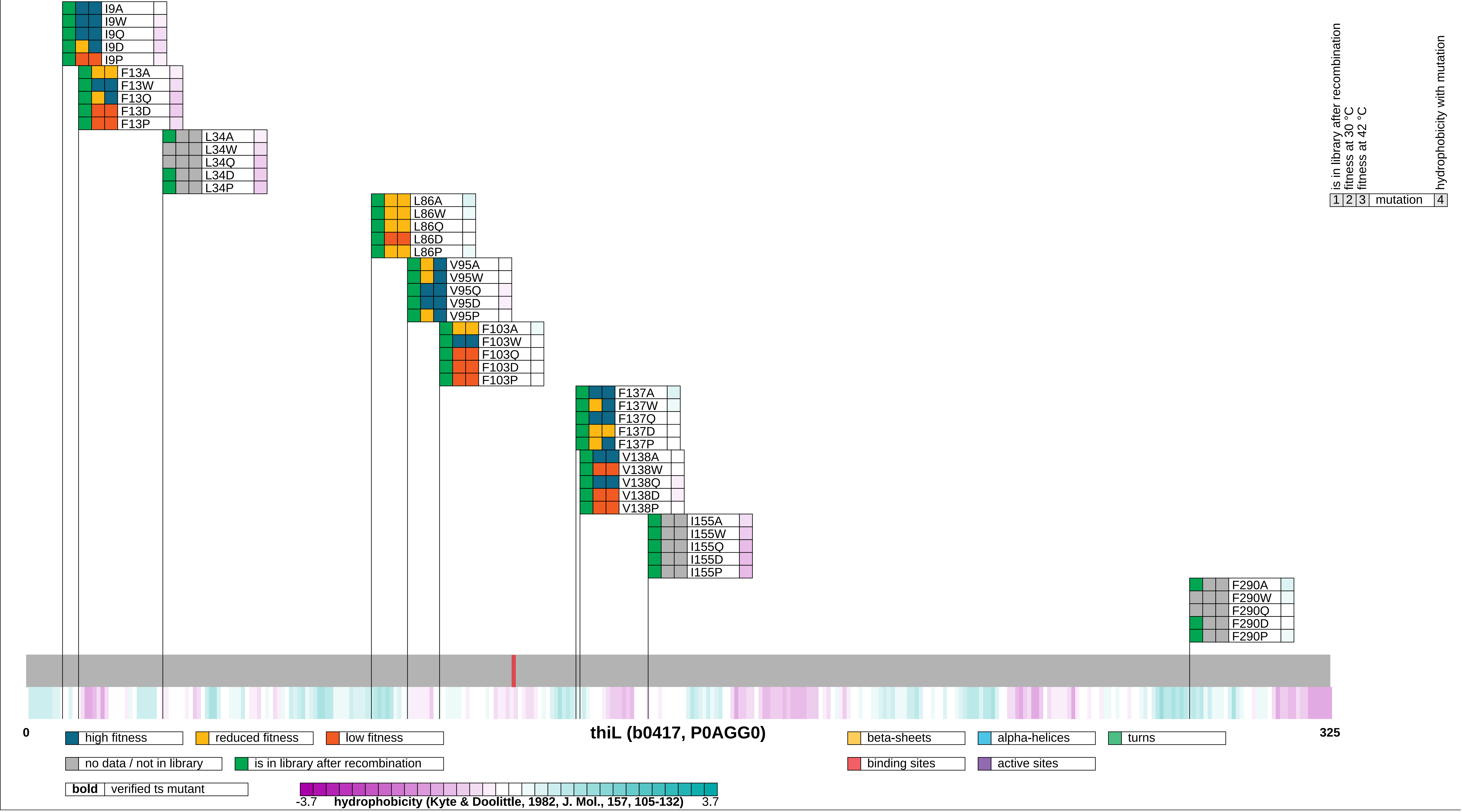
I9A
I9W
I9Q
I9D
I9P
F13A
F13W
F13Q
F13D
F13P
L34A
L34W
L34Q
L34D
L34P
L86A
L86W
L86Q
L86D
L86P
V95A
V95W
V95Q
V95D
V95P
F103A
F103W
F103Q
F103D
F103P
F137A
F137W
F137Q
F137D
F137P
V138A
V138W
V138Q
V138D
V138P
I155A
I155W
I155Q
I155D
I155P
F290A
F290W
F290Q
F290D
F290P
is in library after recombination
fitness at 30 °C
fitness at 42 °C
hydrophobicity with mutation
1 2 3 mutation 4
0
thiL (b0417, P0AGG0)
325
high fitness
reduced fitness
low fitness
beta-sheets
alpha-helices
turns
no data / not in library
is in library after recombination
binding sites
active sites
bold verified ts mutant
-3.7 hydrophobicity (Kyte & Doolittle, 1982, J. Mol., 157, 105-132) 3.7

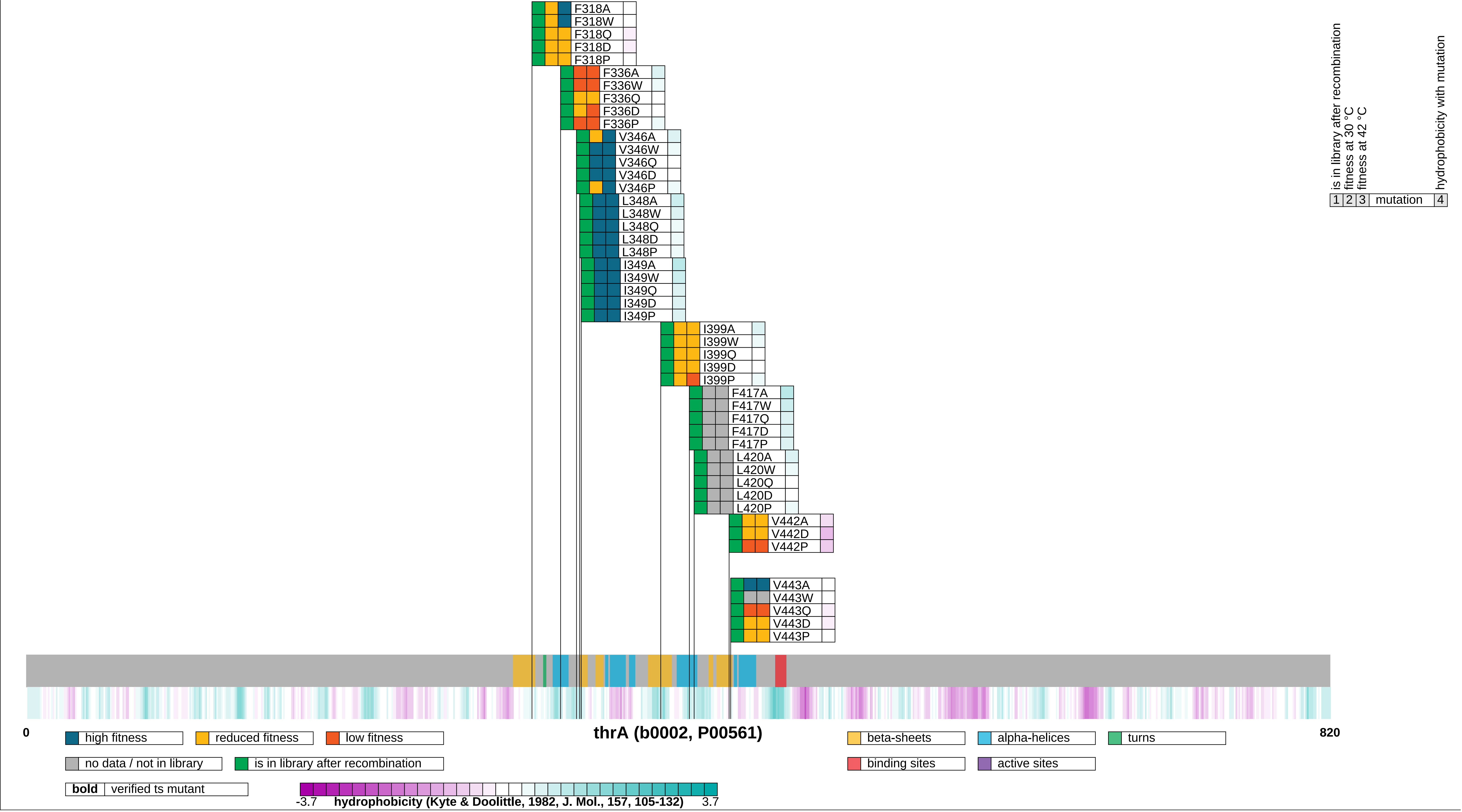
F318A
F318W
F318Q
F318D
F318P
F336A
F336W
F336Q
F336D
F336P
V346A
V346W
V346Q
V346D
V346P
L348A
L348W
L348Q
L348D
L348P
I349A
I349W
I349Q
I349D
I349P
I399A
I399W
I399Q
I399D
I399P
F417A
F417W
F417Q
F417D
F417P
L420A
L420W
L420Q
L420D
L420P
V442A
V442D
V442P
V443A
V443W
V443Q
V443D
V443P
is in library after recombination
fitness at 30 °C
fitness at 42 °C
hydrophobicity with mutation
1 2 3 mutation 4
0
820
thrA (b0002, P00561)
high fitness
reduced fitness
low fitness
no data / not in library
is in library after recombination
bold verified ts mutant
beta-sheets
alpha-helices
turns
binding sites
active sites
-3.7
hydrophobicity (Kyte & Doolittle, 1982, J. Mol., 157, 105-132)
3.7

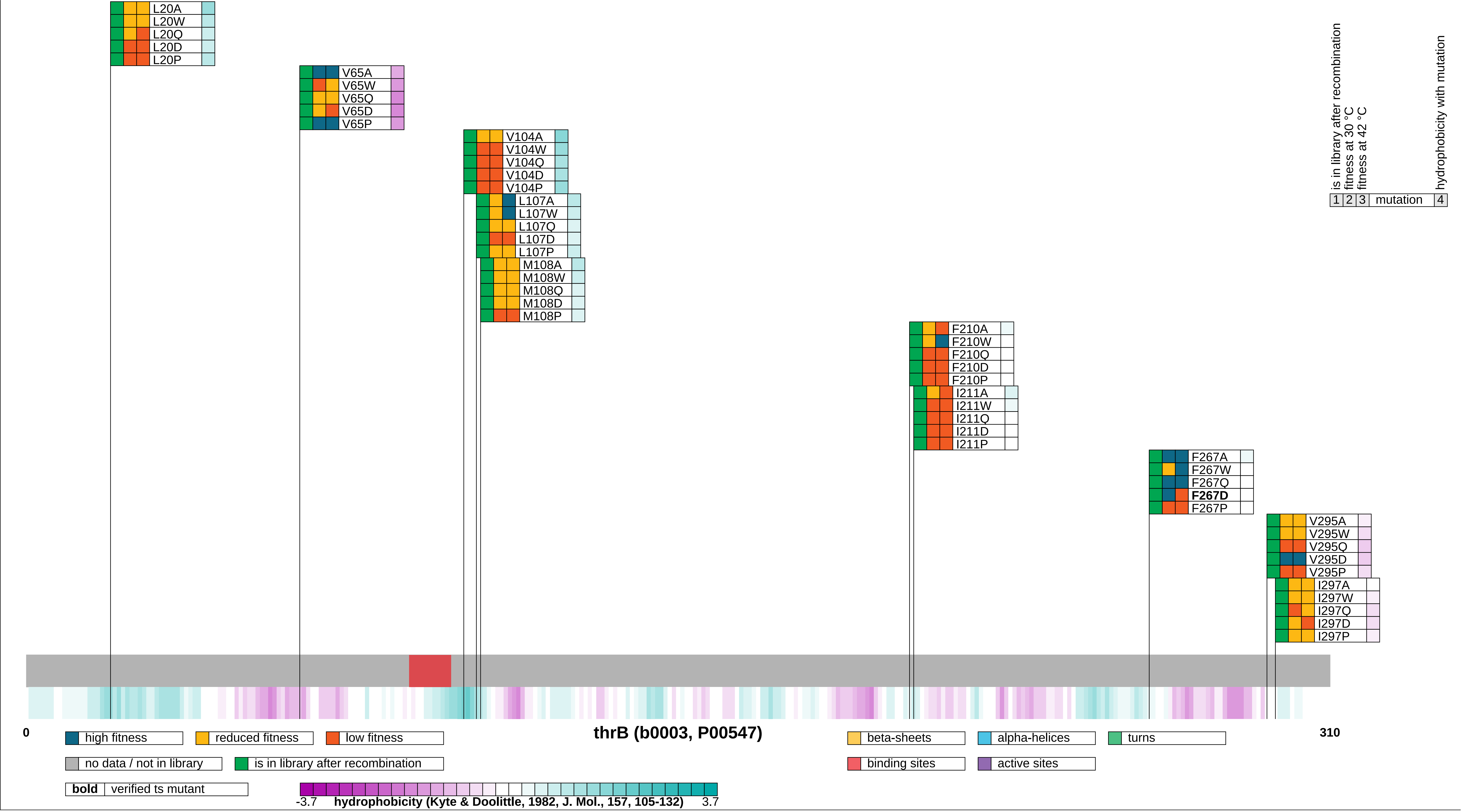
L20A
L20W
L20Q
L20D
L20P
V65A
V65W
V65Q
V65D
V65P
V104A
V104W
V104Q
V104D
V104P
L107A
L107W
L107Q
L107D
L107P
M108A
M108W
M108Q
M108D
M108P
F210A
F210W
F210Q
F210D
F210P
I211A
I211W
I211Q
I211D
I211P
F267A
F267W
F267Q
F267D
F267P
V295A
V295W
V295Q
V295D
V295P
I297A
I297W
I297Q
I297D
I297P
is in library after recombination
fitness at 30 °C
fitness at 42 °C
hydrophobicity with mutation
1
2
3
mutation
4
0
thrB (b0003, P00547)
310
high fitness
reduced fitness
low fitness
beta-sheets
alpha-helices
turns
no data / not in library
is in library after recombination
binding sites
active sites
bold
verified ts mutant
-3.7
hydrophobicity (Kyte & Doolittle, 1982, J. Mol., 157, 105-132)
3.7

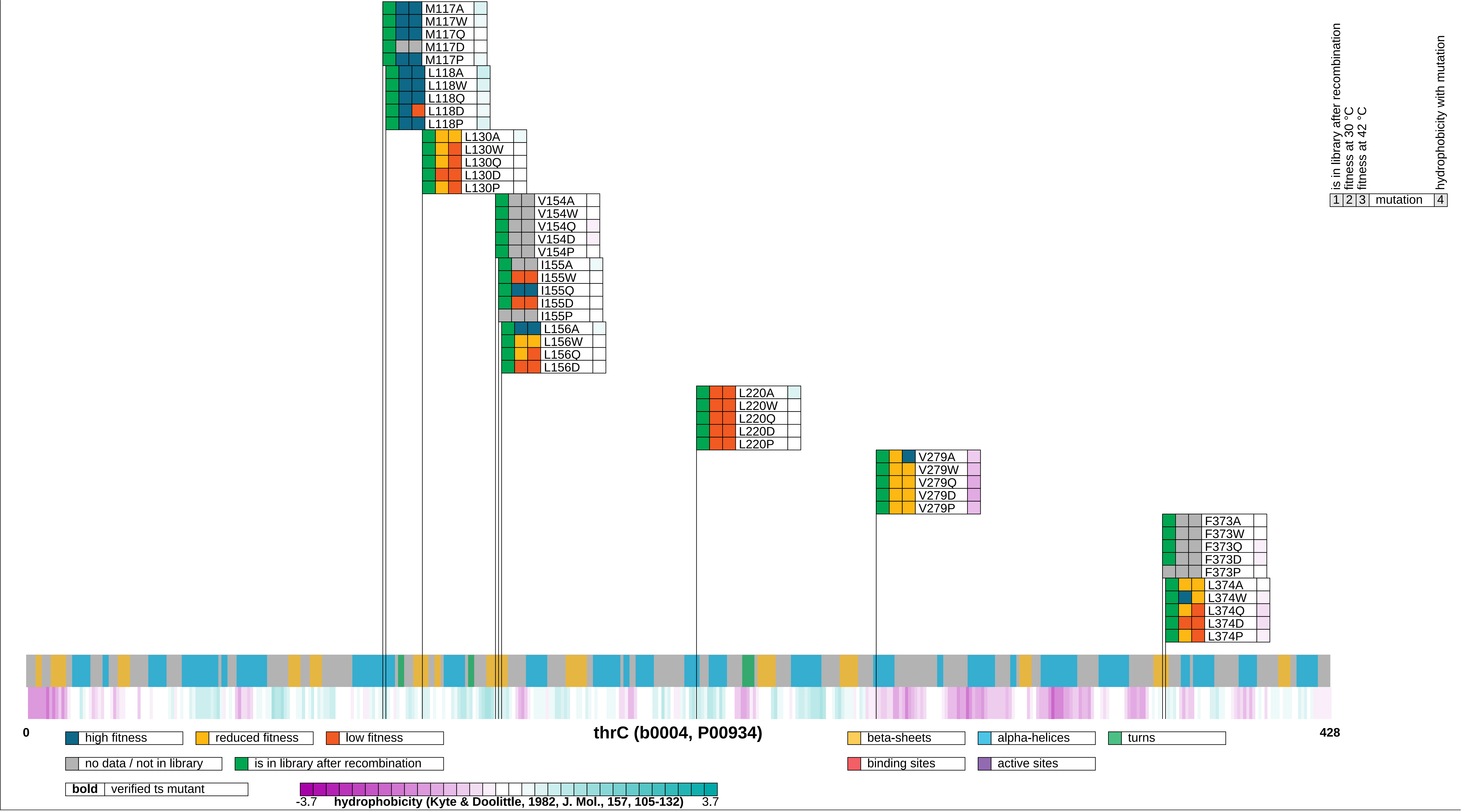

is in library after recombination
fitness at 30 °C
fitness at 42 °C
hydrophobicity with mutation
1
2
3
mutation
4
M117A
M117W
M117Q
M117D
M117P
L118A
L118W
L118Q
L118D
L118P
L130A
L130W
L130Q
L130D
L130P
V154A
V154W
V154Q
V154D
V154P
I155A
I155W
I155Q
I155D
I155P
L156A
L156W
L156Q
L156D
L220A
L220W
L220Q
L220D
L220P
V279A
V279W
V279Q
V279D
V279P
F373A
F373W
F373Q
F373D
F373P
L374A
L374W
L374Q
L374D
L374P
0
428
thrC (b0004, P00934)
high fitness
reduced fitness
low fitness
beta-sheets
alpha-helices
turns
no data / not in library
is in library after recombination
binding sites
active sites
bold
verified ts mutant
-3.7
hydrophobicity (Kyte & Doolittle, 1982, J. Mol., 157, 105-132)
3.7

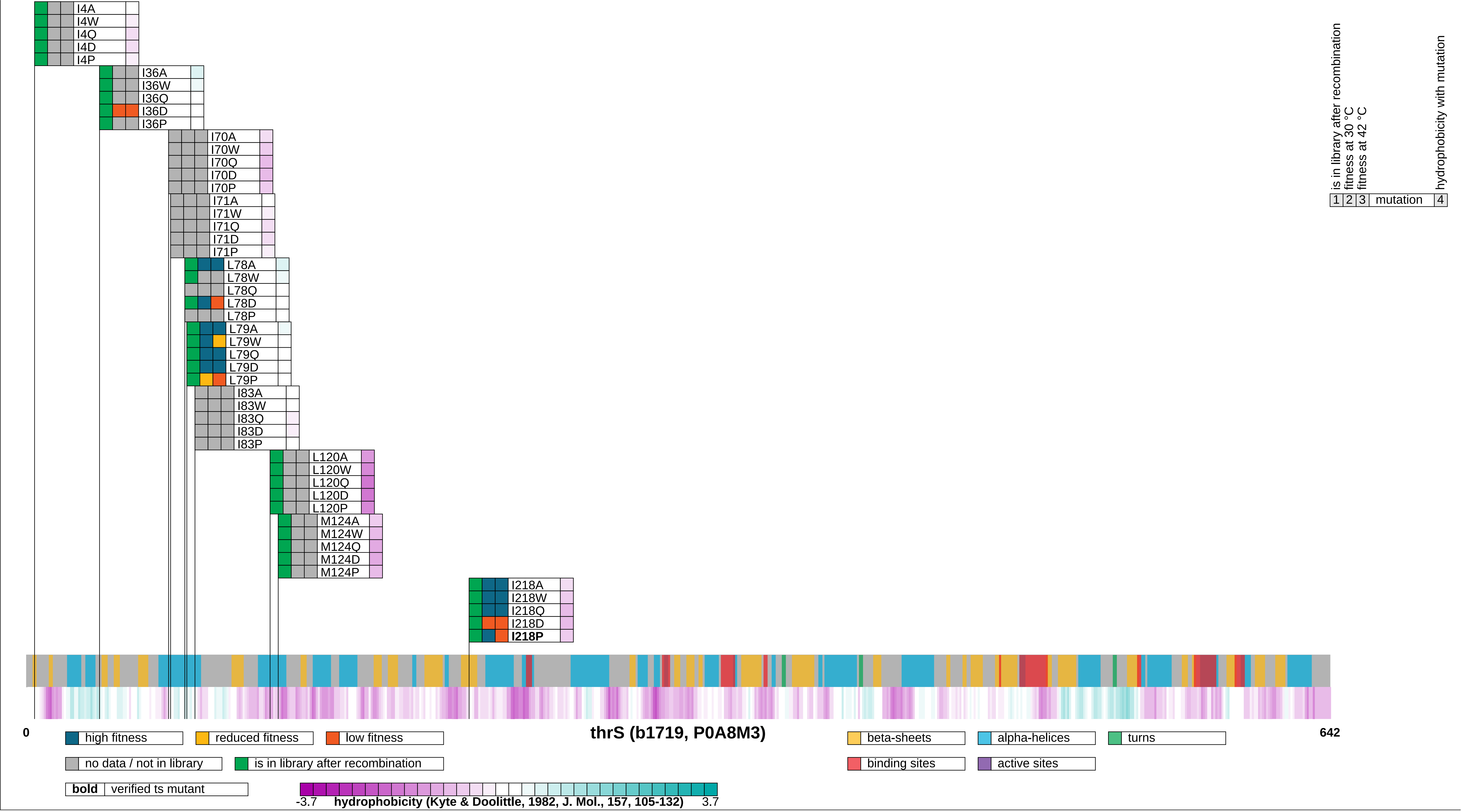

I4A
I4W
I4Q
I4D
I4P
I36A
I36W
I36Q
I36D
I36P
I70A
I70W
I70Q
I70D
I70P
I71A
I71W
I71Q
I71D
I71P
L78A
L78W
L78Q
L78D
L78P
L79A
L79W
L79Q
L79D
L79P
I83A
I83W
I83Q
I83D
I83P
L120A
L120W
L120Q
L120D
L120P
M124A
M124W
M124Q
M124D
M124P
I218A
I218W
I218Q
I218D
I218P
is in library after recombination
fitness at 30 °C
fitness at 42 °C
hydrophobicity with mutation
1
2
3
mutation
4
0
642
thrS (b1719, P0A8M3)
high fitness
reduced fitness
low fitness
no data / not in library
is in library after recombination
bold verified ts mutant
beta-sheets
alpha-helices
turns
binding sites
active sites
-3.7
hydrophobicity (Kyte & Doolittle, 1982, J. Mol., 157, 105-132)
3.7

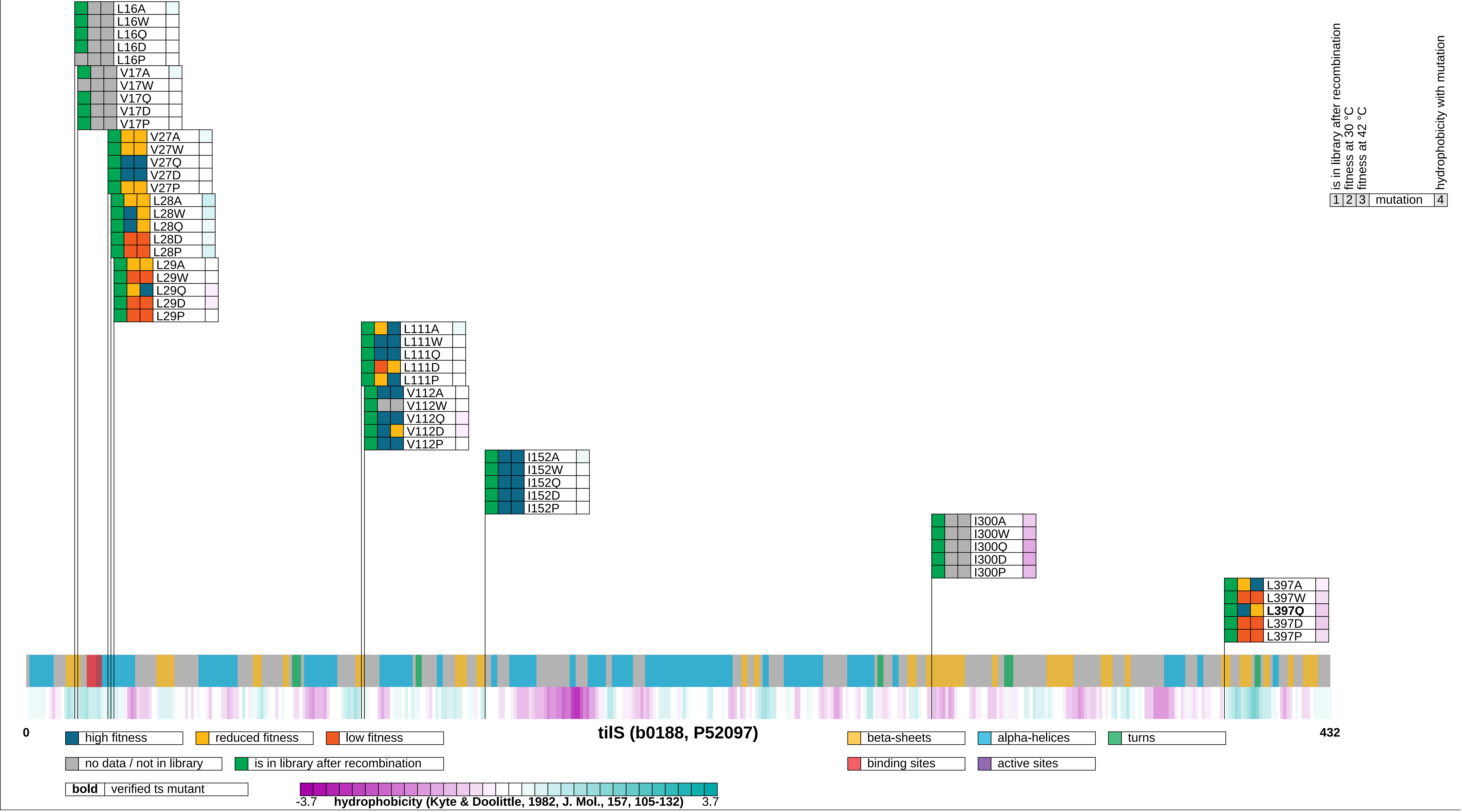

L16A
L16W
L16Q
L16D
L16P
V17A
V17W
V17Q
V17D
V17P
V27A
V27W
V27Q
V27D
V27P
L28A
L28W
L28Q
L28D
L28P
L29A
L29W
L29Q
L29D
L29P
L111A
L111W
L111Q
L111D
L111P
V112A
V112W
V112Q
V112D
V112P
I152A
I152W
I152Q
I152D
I152P
I300A
I300W
I300Q
I300D
I300P
L397A
L397W
L397Q
L397D
L397P
is in library after recombination
fitness at 30 °C
fitness at 42 °C
hydrophobicity with mutation
1 2 3 mutation 4
0
432
tilS (b0188, P52097)
high fitness
reduced fitness
low fitness
no data / not in library
is in library after recombination
bold verified ts mutant
beta-sheets
alpha-helices
turns
binding sites
active sites
-3.7 hydrophobicity (Kyte & Doolittle, 1982, J. Mol., 157, 105-132) 3.7

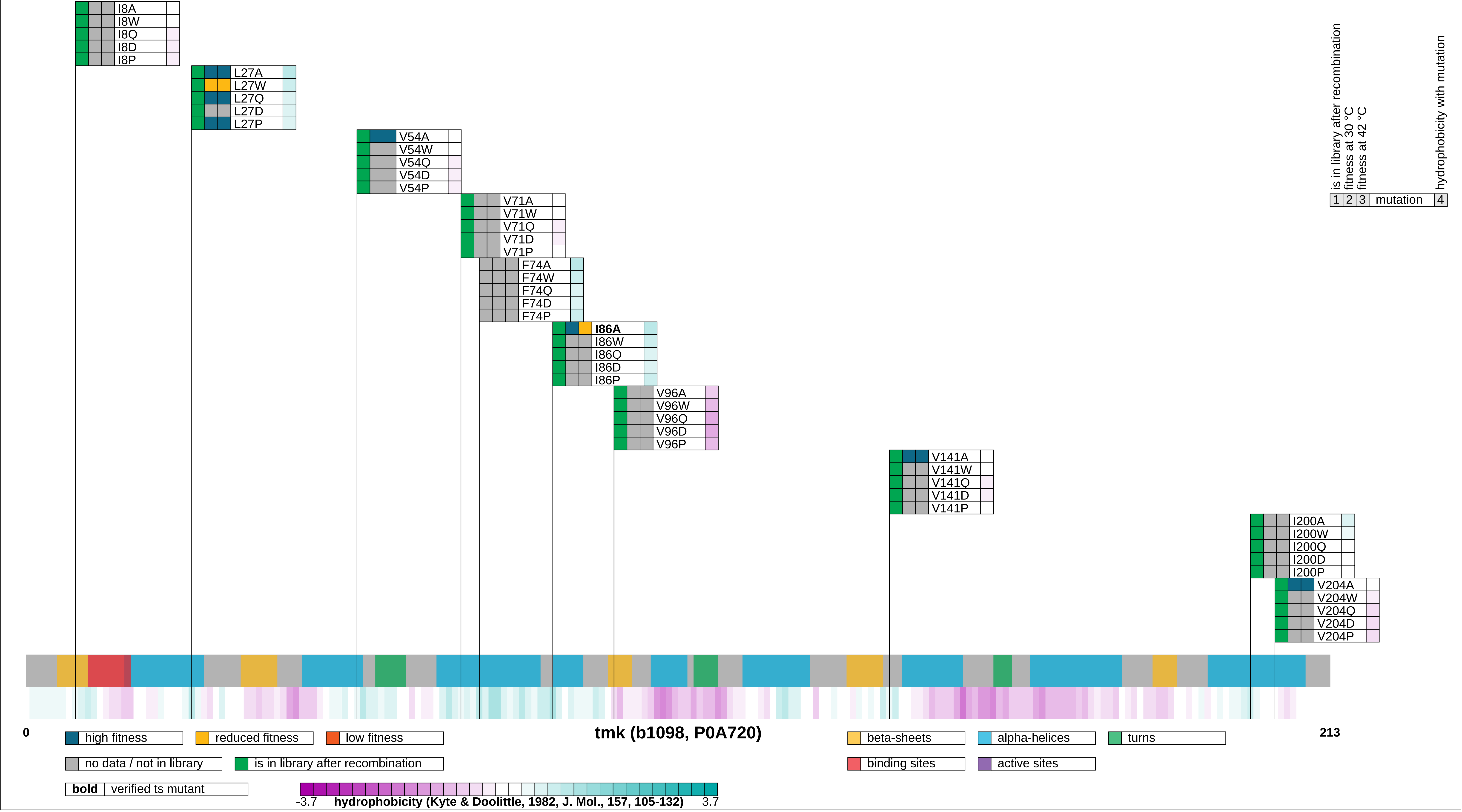

I8A
I8W
I8Q
I8D
I8P
L27A
L27W
L27Q
L27D
L27P
V54A
V54W
V54Q
V54D
V54P
V71A
V71W
V71Q
V71D
V71P
F74A
F74W
F74Q
F74D
F74P
I86A
I86W
I86Q
I86D
I86P
V96A
V96W
V96Q
V96D
V96P
V141A
V141W
V141Q
V141D
V141P
I200A
I200W
I200Q
I200D
I200P
V204A
V204W
V204Q
V204D
V204P
is in library after recombination
fitness at 30 °C
fitness at 42 °C
hydrophobicity with mutation
1 2 3 mutation 4
0
tmk (b1098, P0A720)
213
high fitness
reduced fitness
low fitness
beta-sheets
alpha-helices
turns
no data / not in library
is in library after recombination
binding sites
active sites
bold verified ts mutant
-3.7
hydrophobicity (Kyte & Doolittle, 1982, J. Mol., 157, 105-132)
3.7

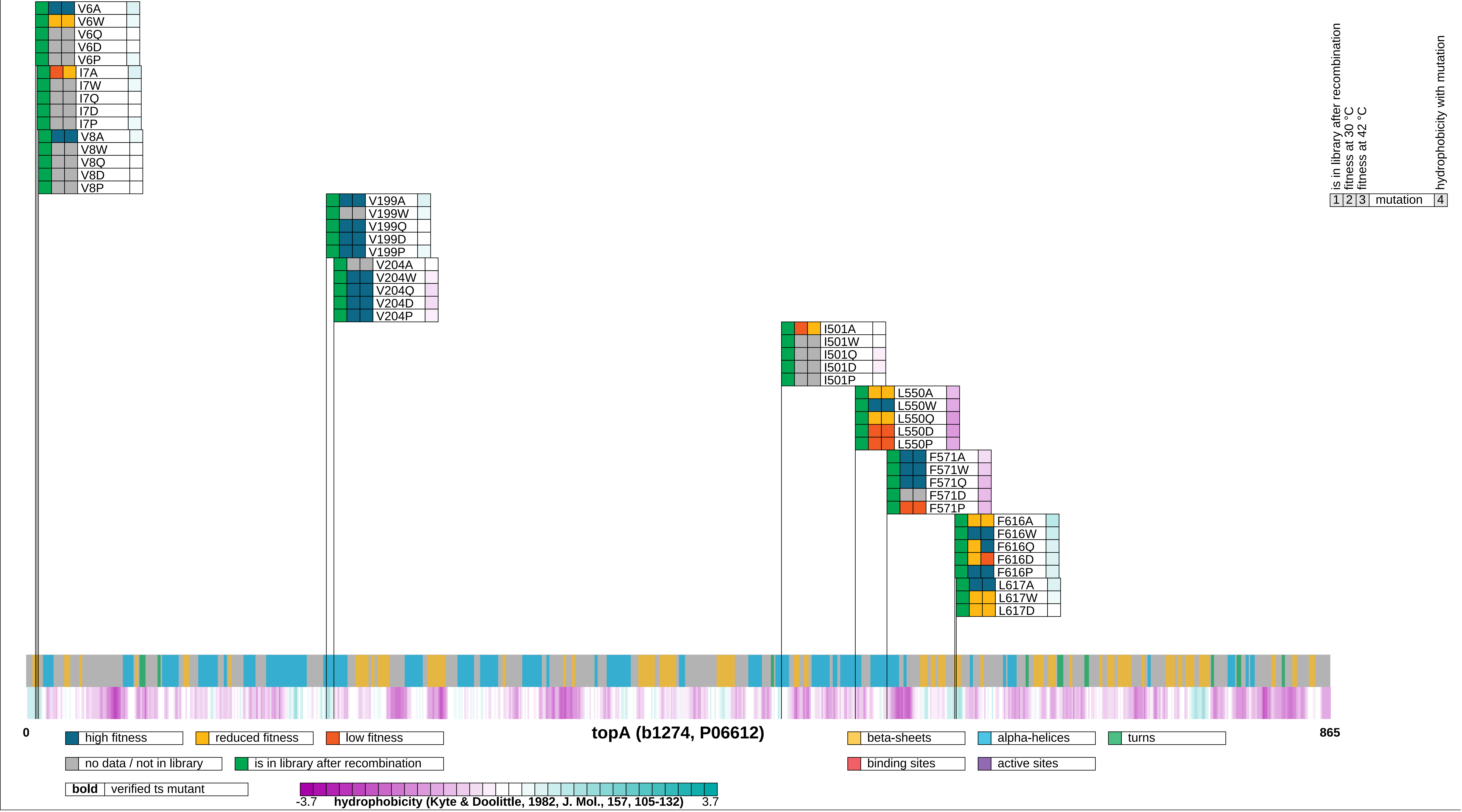
V6A
V6W
V6Q
V6D
V6P
I7A
I7W
I7Q
I7D
I7P
V8A
V8W
V8Q
V8D
V8P
V199A
V199W
V199Q
V199D
V199P
V204A
V204W
V204Q
V204D
V204P
I501A
I501W
I501Q
I501D
I501P
L550A
L550W
L550Q
L550D
L550P
F571A
F571W
F571Q
F571D
F571P
F616A
F616W
F616Q
F616D
F616P
L617A
L617W
L617D
is in library after recombination
fitness at 30 °C
fitness at 42 °C
hydrophobicity with mutation
1 2 3 mutation 4
0
topA (b1274, P06612)
865
high fitness
reduced fitness
low fitness
beta-sheets
alpha-helices
turns
no data / not in library
is in library after recombination
binding sites
active sites
bold verified ts mutant
-3.7
hydrophobicity (Kyte & Doolittle, 1982, J. Mol., 157, 105-132)
3.7

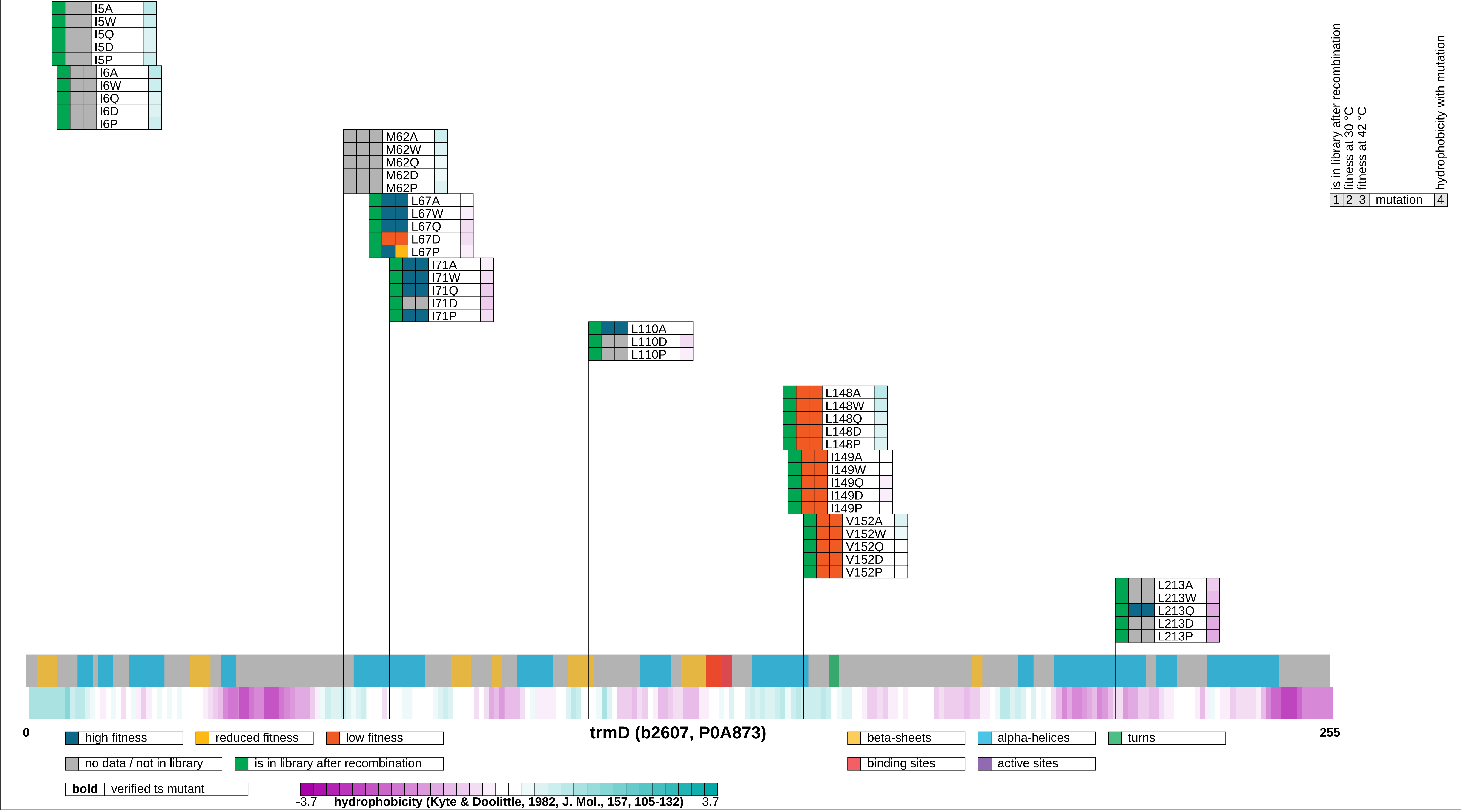

I5A
I5W
I5Q
I5D
I5P
I6A
I6W
I6Q
I6D
I6P
M62A
M62W
M62Q
M62D
M62P
L67A
L67W
L67Q
L67D
L67P
I71A
I71W
I71Q
I71D
I71P
L110A
L110D
L110P
L148A
L148W
L148Q
L148D
L148P
I149A
I149W
I149Q
I149D
I149P
V152A
V152W
V152Q
V152D
V152P
L213A
L213W
L213Q
L213D
L213P
is in library after recombination
fitness at 30 °C
fitness at 42 °C
hydrophobicity with mutation
1 2 3 mutation 4
0
trmD (b2607, P0A873)
255
high fitness
reduced fitness
low fitness
no data / not in library
is in library after recombination
bold verified ts mutant
beta-sheets
alpha-helices
turns
binding sites
active sites
-3.7
hydrophobicity (Kyte & Doolittle, 1982, J. Mol., 157, 105-132)
3.7

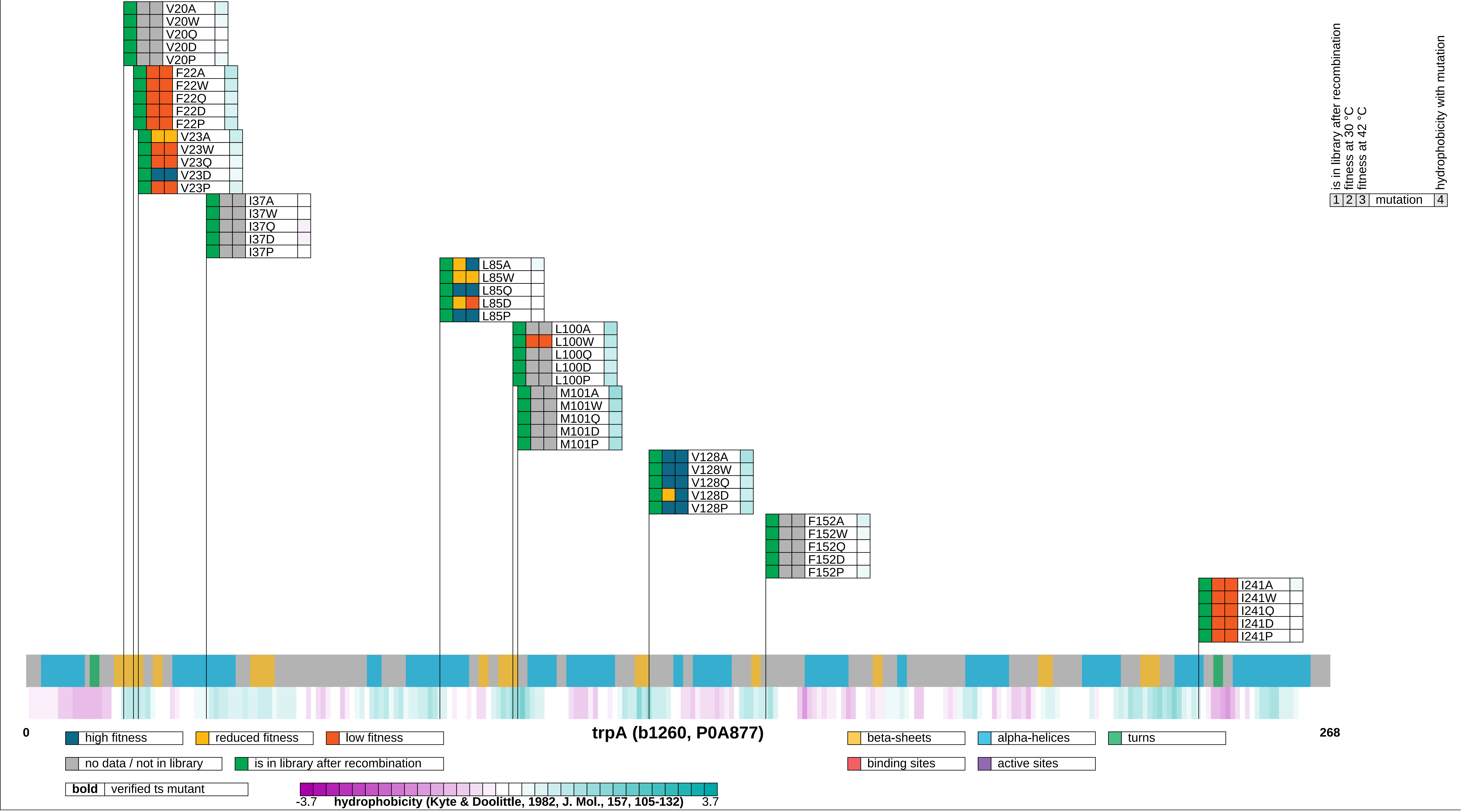

V20A
V20W
V20Q
V20D
V20P
F22A
F22W
F22Q
F22D
F22P
V23A
V23W
V23Q
V23D
V23P
I37A
I37W
I37Q
I37D
I37P
L85A
L85W
L85Q
L85D
L85P
L100A
L100W
L100Q
L100D
L100P
M101A
M101W
M101Q
M101D
M101P
V128A
V128W
V128Q
V128D
V128P
F152A
F152W
F152Q
F152D
F152P
I241A
I241W
I241Q
I241D
I241P
is in library after recombination
fitness at 30 °C
fitness at 42 °C
hydrophobicity with mutation
1
2
3
mutation
4
0
trpA (b1260, P0A877)
268
high fitness
reduced fitness
low fitness
beta-sheets
alpha-helices
turns
no data / not in library
is in library after recombination
binding sites
active sites
bold
verified ts mutant
-3.7
hydrophobicity (Kyte & Doolittle, 1982, J. Mol., 157, 105-132)
3.7

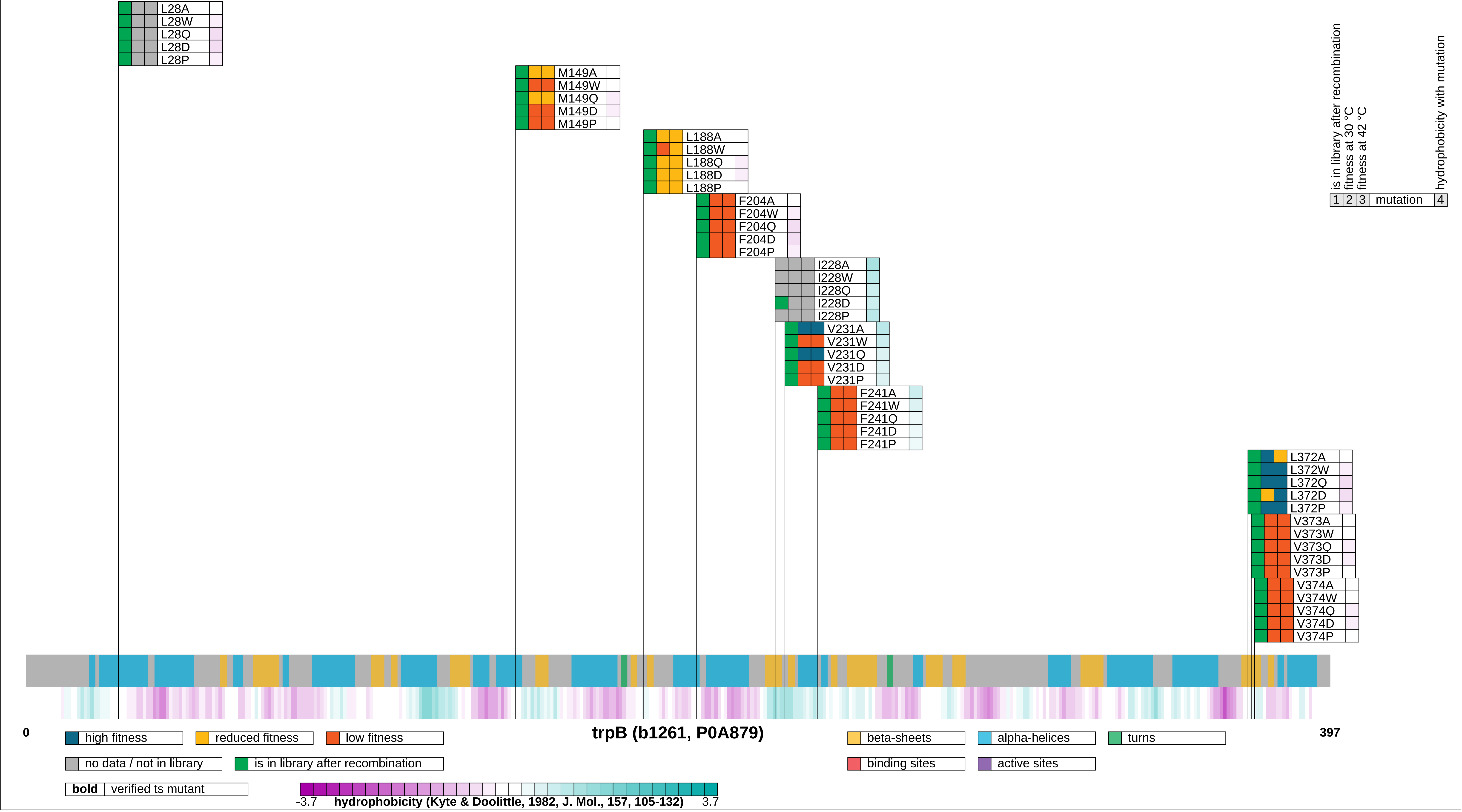
L28A
L28W
L28Q
L28D
L28P
M149A
M149W
M149Q
M149D
M149P
L188A
L188W
L188Q
L188D
L188P
F204A
F204W
F204Q
F204D
F204P
I228A
I228W
I228Q
I228D
I228P
V231A
V231W
V231Q
V231D
V231P
F241A
F241W
F241Q
F241D
F241P
L372A
L372W
L372Q
L372D
L372P
V373A
V373W
V373Q
V373D
V373P
V374A
V374W
V374Q
V374D
V374P
is in library after recombination
fitness at 30 °C
fitness at 42 °C
hydrophobicity with mutation
1 2 3 mutation 4
0
trpB (b1261, P0A879)
397
high fitness
reduced fitness
low fitness
beta-sheets
alpha-helices
turns
no data / not in library
is in library after recombination
binding sites
active sites
bold verified ts mutant
-3.7
hydrophobicity (Kyte & Doolittle, 1982, J. Mol., 157, 105-132)
3.7

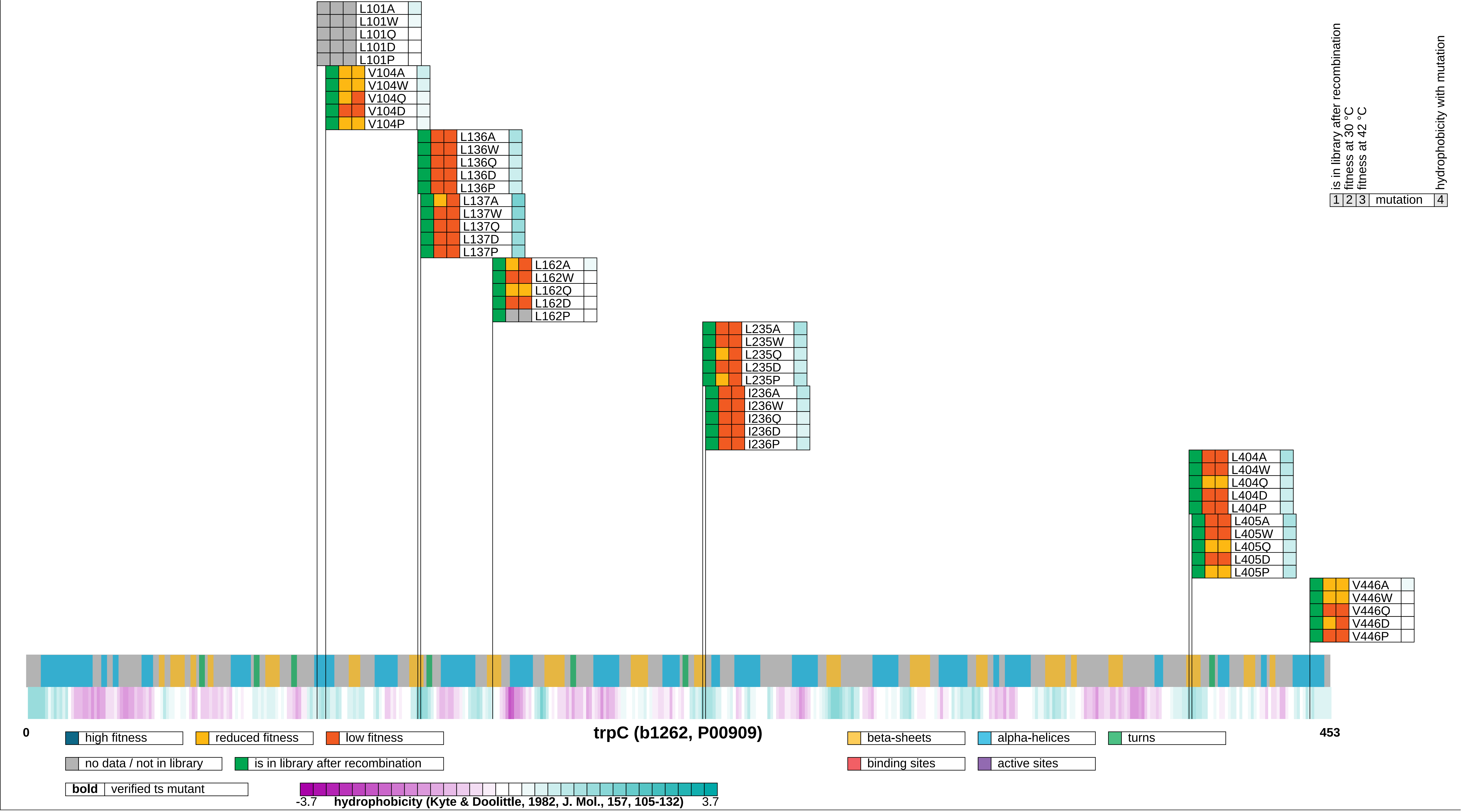
L101A
L101W
L101Q
L101D
L101P
V104A
V104W
V104Q
V104D
V104P
L136A
L136W
L136Q
L136D
L136P
L137A
L137W
L137Q
L137D
L137P
L162A
L162W
L162Q
L162D
L162P
L235A
L235W
L235Q
L235D
L235P
I236A
I236W
I236Q
I236D
I236P
L404A
L404W
L404Q
L404D
L404P
L405A
L405W
L405Q
L405D
L405P
V446A
V446W
V446Q
V446D
V446P
is in library after recombination
fitness at 30 °C
fitness at 42 °C
hydrophobicity with mutation
1
2
3
mutation
4
0
trpC (b1262, P00909)
453
high fitness
reduced fitness
low fitness
no data / not in library
is in library after recombination
bold
verified ts mutant
beta-sheets
alpha-helices
turns
binding sites
active sites
-3.7
hydrophobicity (Kyte & Doolittle, 1982, J. Mol., 157, 105-132)
3.7

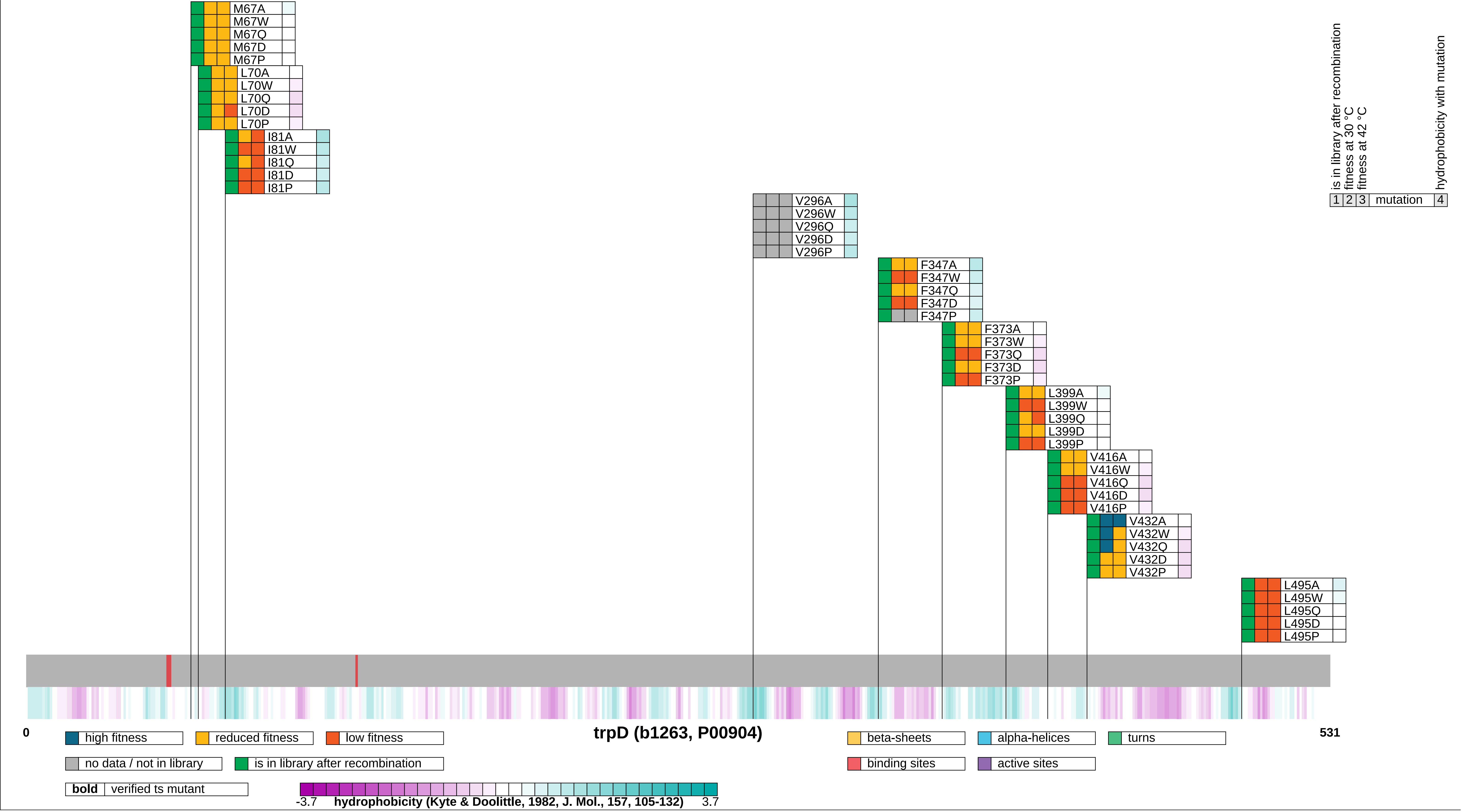

is in library after recombination
fitness at 30 °C
fitness at 42 °C
hydrophobicity with mutation
1
2
3
mutation
4
M67A
M67W
M67Q
M67D
M67P
L70A
L70W
L70Q
L70D
L70P
I81A
I81W
I81Q
I81D
I81P
V296A
V296W
V296Q
V296D
V296P
F347A
F347W
F347Q
F347D
F347P
F373A
F373W
F373Q
F373D
F373P
L399A
L399W
L399Q
L399D
L399P
V416A
V416W
V416Q
V416D
V416P
V432A
V432W
V432Q
V432D
V432P
L495A
L495W
L495Q
L495D
L495P
0
trpD (b1263, P00904)
531
high fitness
reduced fitness
low fitness
beta-sheets
alpha-helices
turns
no data / not in library
is in library after recombination
binding sites
active sites
bold verified ts mutant
-3.7
hydrophobicity (Kyte & Doolittle, 1982, J. Mol., 157, 105-132)
3.7

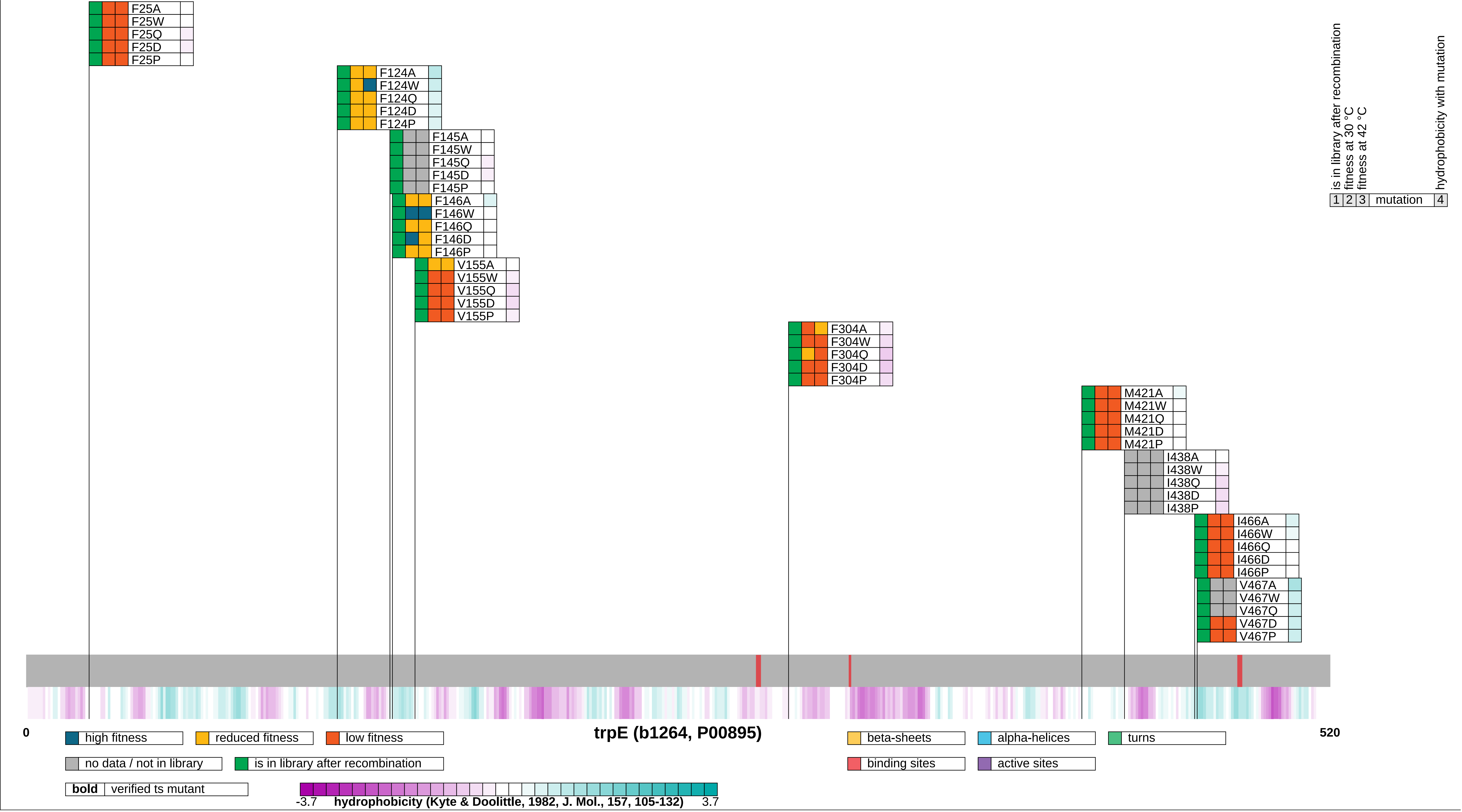
is in library after recombination
fitness at 30 °C
fitness at 42 °C
hydrophobicity with mutation
1 2 3 mutation 4
F25A
F25W
F25Q
F25D
F25P
F124A
F124W
F124Q
F124D
F124P
F145A
F145W
F145Q
F145D
F145P
F146A
F146W
F146Q
F146D
F146P
V155A
V155W
V155Q
V155D
V155P
F304A
F304W
F304Q
F304D
F304P
M421A
M421W
M421Q
M421D
M421P
I438A
I438W
I438Q
I438D
I438P
I466A
I466W
I466Q
I466D
I466P
V467A
V467W
V467Q
V467D
V467P
0
trpE (b1264, P00895)
520
high fitness
reduced fitness
low fitness
beta-sheets
alpha-helices
turns
no data / not in library
is in library after recombination
binding sites
active sites
bold verified ts mutant
-3.7 hydrophobicity (Kyte & Doolittle, 1982, J. Mol., 157, 105-132) 3.7

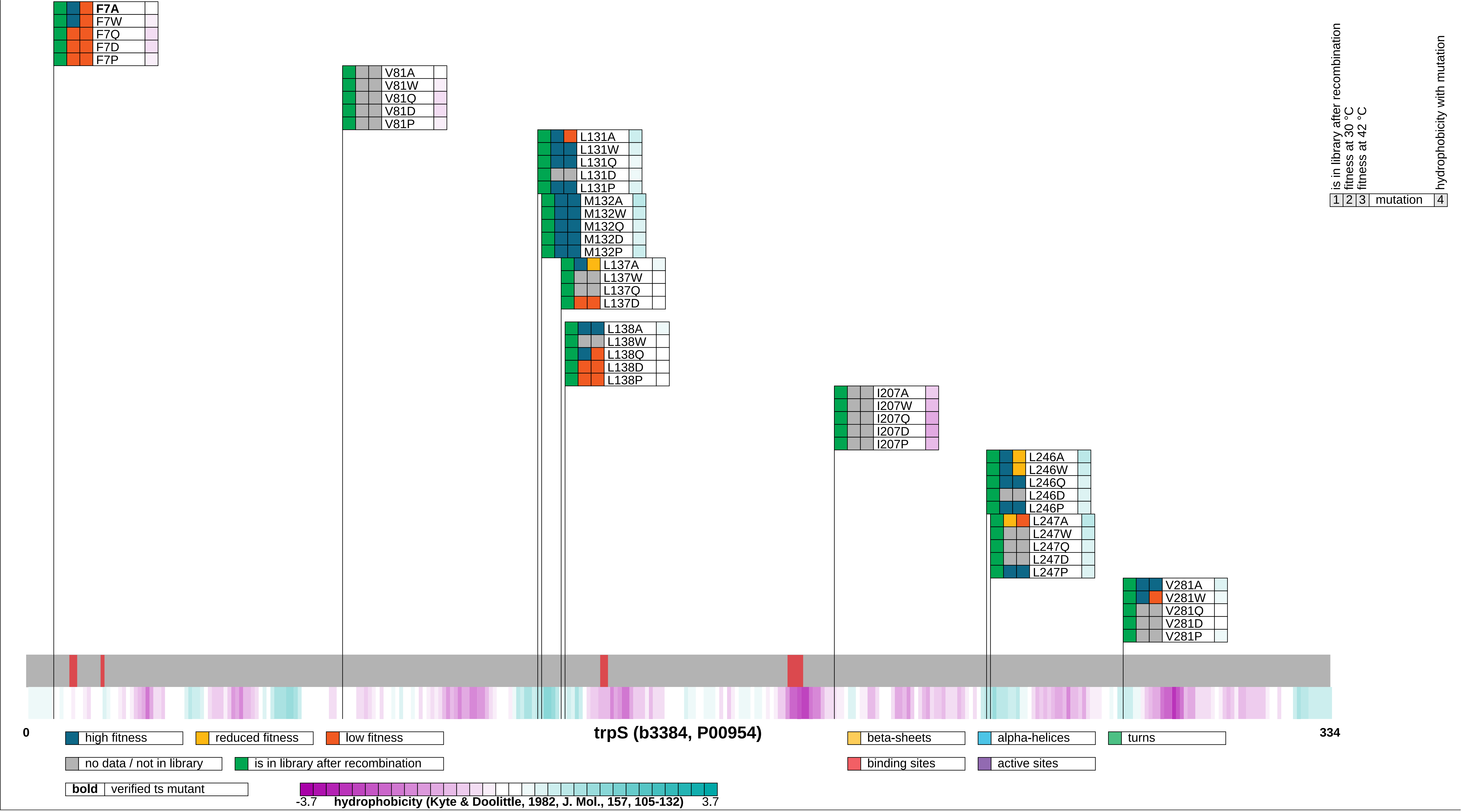

F7A
F7W
F7Q
F7D
F7P
V81A
V81W
V81Q
V81D
V81P
L131A
L131W
L131Q
L131D
L131P
M132A
M132W
M132Q
M132D
M132P
L137A
L137W
L137Q
L137D
L138A
L138W
L138Q
L138D
L138P
I207A
I207W
I207Q
I207D
I207P
L246A
L246W
L246Q
L246D
L246P
L247A
L247W
L247Q
L247D
L247P
V281A
V281W
V281Q
V281D
V281P
is in library after recombination
fitness at 30 °C
fitness at 42 °C
hydrophobicity with mutation
1
2
3
mutation
4
0
trpS (b3384, P00954)
334
high fitness
reduced fitness
low fitness
beta-sheets
alpha-helices
turns
no data / not in library
is in library after recombination
binding sites
active sites
bold
verified ts mutant
-3.7
hydrophobicity (Kyte & Doolittle, 1982, J. Mol., 157, 105-132)
3.7

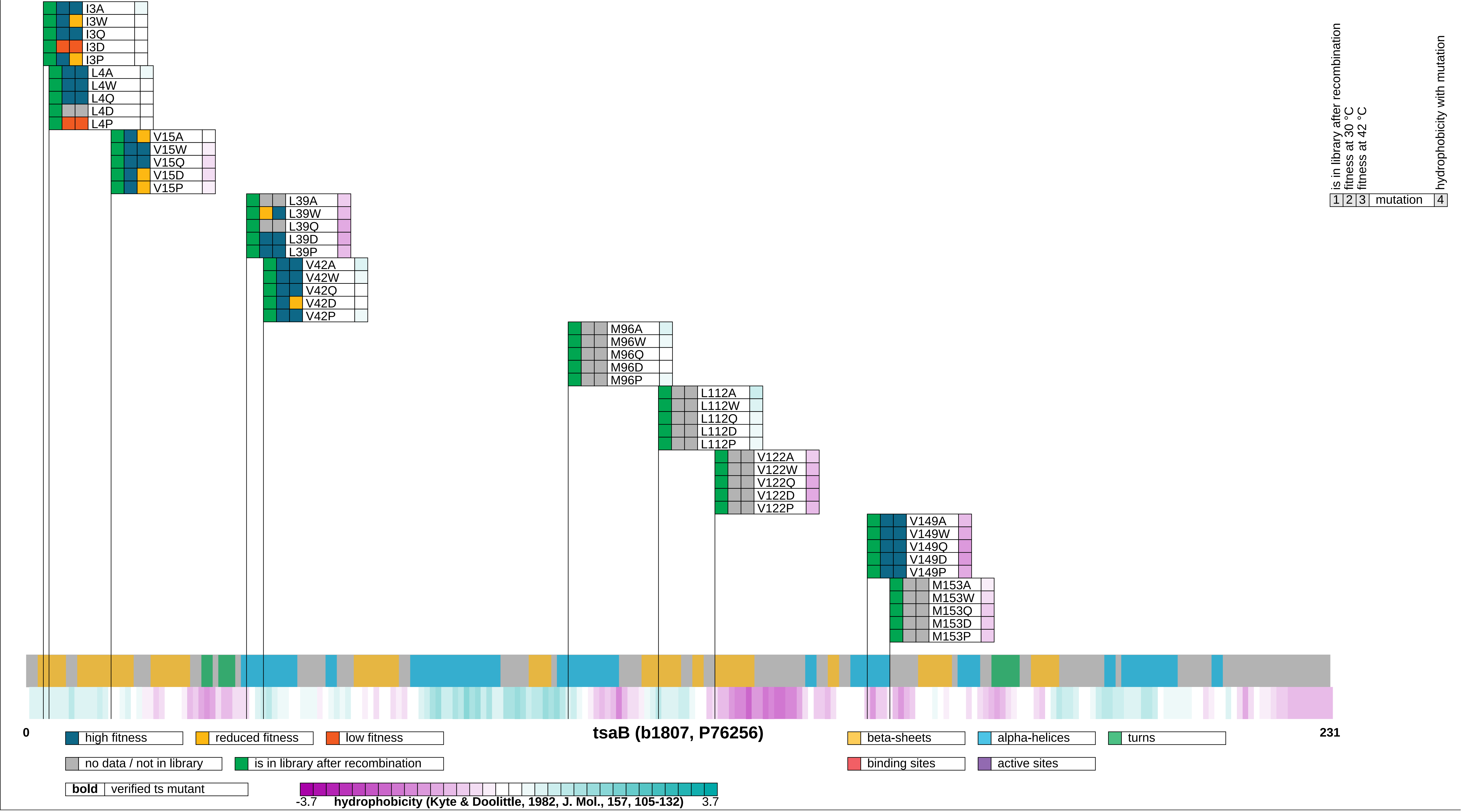

I3A
I3W
I3Q
I3D
I3P
L4A
L4W
L4Q
L4D
L4P
V15A
V15W
V15Q
V15D
V15P
L39A
L39W
L39Q
L39D
L39P
V42A
V42W
V42Q
V42D
V42P
M96A
M96W
M96Q
M96D
M96P
L112A
L112W
L112Q
L112D
L112P
V122A
V122W
V122Q
V122D
V122P
V149A
V149W
V149Q
V149D
V149P
M153A
M153W
M153Q
M153D
M153P
is in library after recombination
fitness at 30 °C
fitness at 42 °C
hydrophobicity with mutation
1
2
3
mutation
4
0
tsaB (b1807, P76256)
231
high fitness
reduced fitness
low fitness
beta-sheets
alpha-helices
turns
no data / not in library
is in library after recombination
binding sites
active sites
bold
verified ts mutant
-3.7
hydrophobicity (Kyte & Doolittle, 1982, J. Mol., 157, 105-132)
3.7

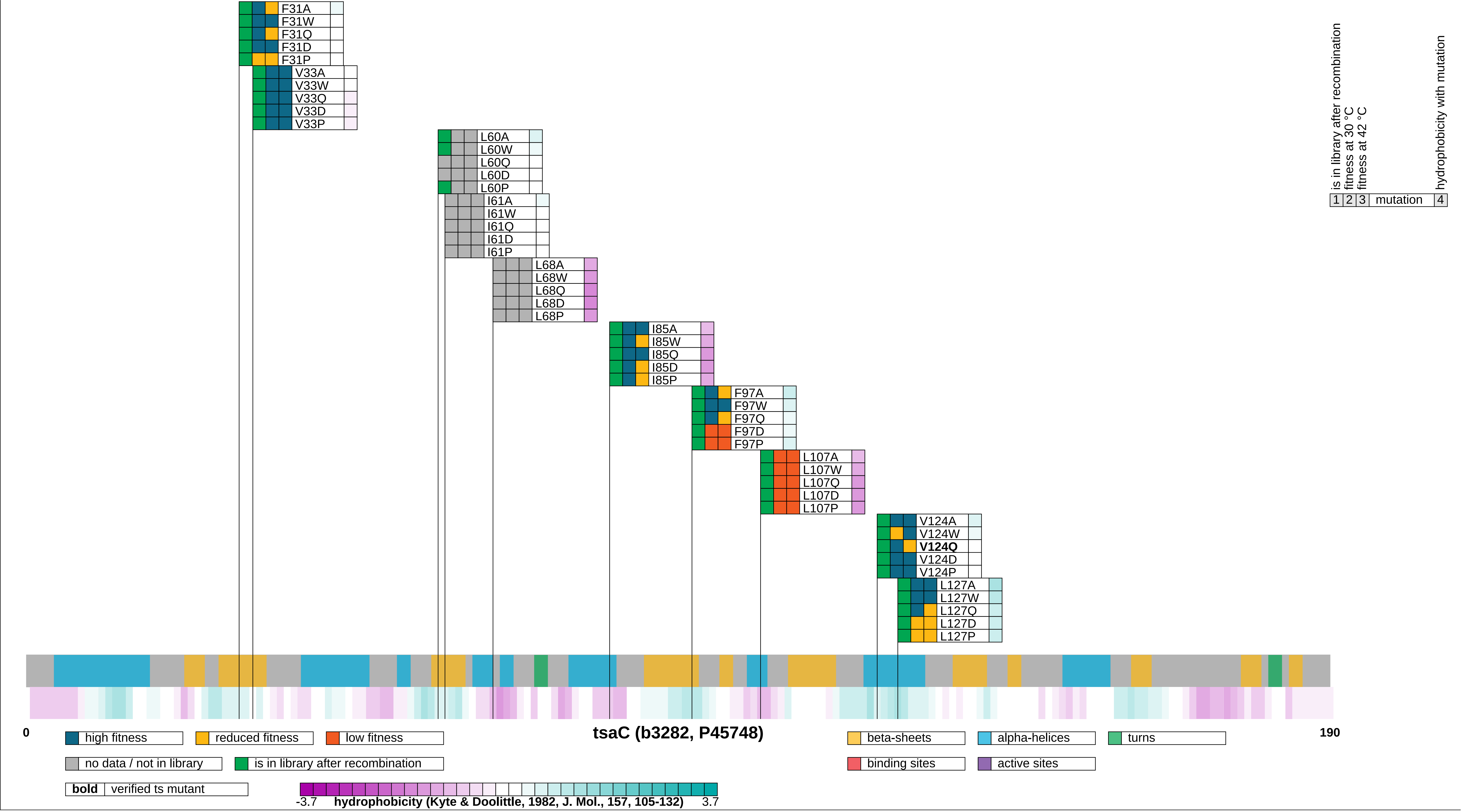

F31A
F31W
F31Q
F31D
F31P
V33A
V33W
V33Q
V33D
V33P
L60A
L60W
L60Q
L60D
L60P
I61A
I61W
I61Q
I61D
I61P
L68A
L68W
L68Q
L68D
L68P
I85A
I85W
I85Q
I85D
I85P
F97A
F97W
F97Q
F97D
F97P
L107A
L107W
L107Q
L107D
L107P
V124A
V124W
V124Q
V124D
V124P
L127A
L127W
L127Q
L127D
L127P
is in library after recombination
fitness at 30 °C
fitness at 42 °C
hydrophobicity with mutation
1 2 3 mutation 4
0
tsaC (b3282, P45748)
190
high fitness
reduced fitness
low fitness
beta-sheets
alpha-helices
turns
no data / not in library
is in library after recombination
binding sites
active sites
bold verified ts mutant
-3.7 hydrophobicity (Kyte & Doolittle, 1982, J. Mol., 157, 105-132) 3.7

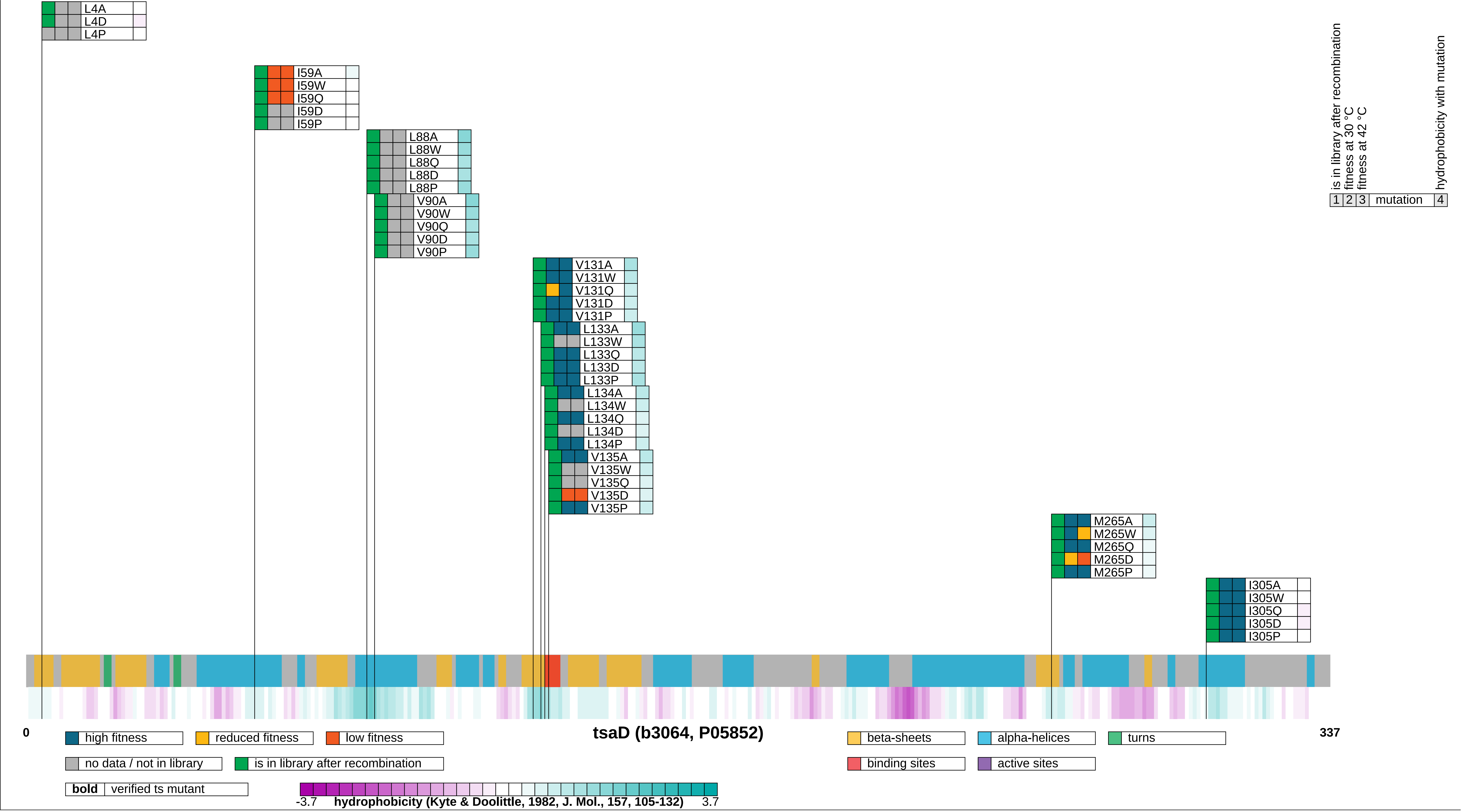

L4A
L4D
L4P
I59A
I59W
I59Q
I59D
I59P
L88A
L88W
L88Q
L88D
L88P
V90A
V90W
V90Q
V90D
V90P
V131A
V131W
V131Q
V131D
V131P
L133A
L133W
L133Q
L133D
L133P
L134A
L134W
L134Q
L134D
L134P
V135A
V135W
V135Q
V135D
V135P
M265A
M265W
M265Q
M265D
M265P
I305A
I305W
I305Q
I305D
I305P
is in library after recombination
fitness at 30 °C
fitness at 42 °C
hydrophobicity with mutation
1 2 3 mutation 4
0
tsaD (b3064, P05852)
337
high fitness
reduced fitness
low fitness
no data / not in library
is in library after recombination
bold verified ts mutant
beta-sheets
alpha-helices
turns
binding sites
active sites
-3.7 hydrophobicity (Kyte & Doolittle, 1982, J. Mol., 157, 105-132) 3.7

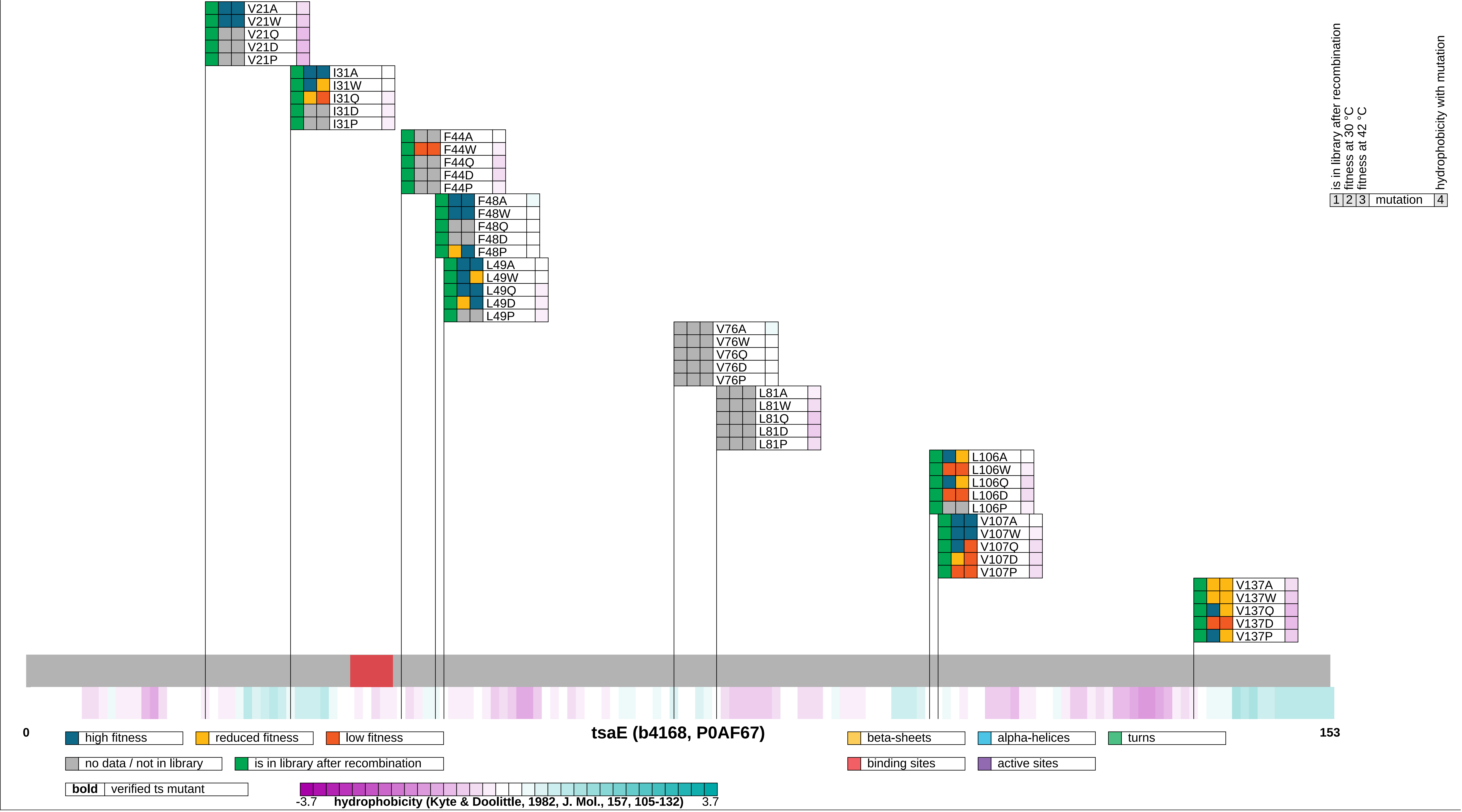

V21A
V21W
V21Q
V21D
V21P
I31A
I31W
I31Q
I31D
I31P
F44A
F44W
F44Q
F44D
F44P
F48A
F48W
F48Q
F48D
F48P
L49A
L49W
L49Q
L49D
L49P
V76A
V76W
V76Q
V76D
V76P
L81A
L81W
L81Q
L81D
L81P
L106A
L106W
L106Q
L106D
L106P
V107A
V107W
V107Q
V107D
V107P
V137A
V137W
V137Q
V137D
V137P
is in library after recombination
fitness at 30 °C
fitness at 42 °C
hydrophobicity with mutation
1
2
3
mutation
4
0
tsaE (b4168, P0AF67)
153
high fitness
reduced fitness
low fitness
beta-sheets
alpha-helices
turns
no data / not in library
is in library after recombination
binding sites
active sites
bold
verified ts mutant
-3.7
hydrophobicity (Kyte & Doolittle, 1982, J. Mol., 157, 105-132)
3.7

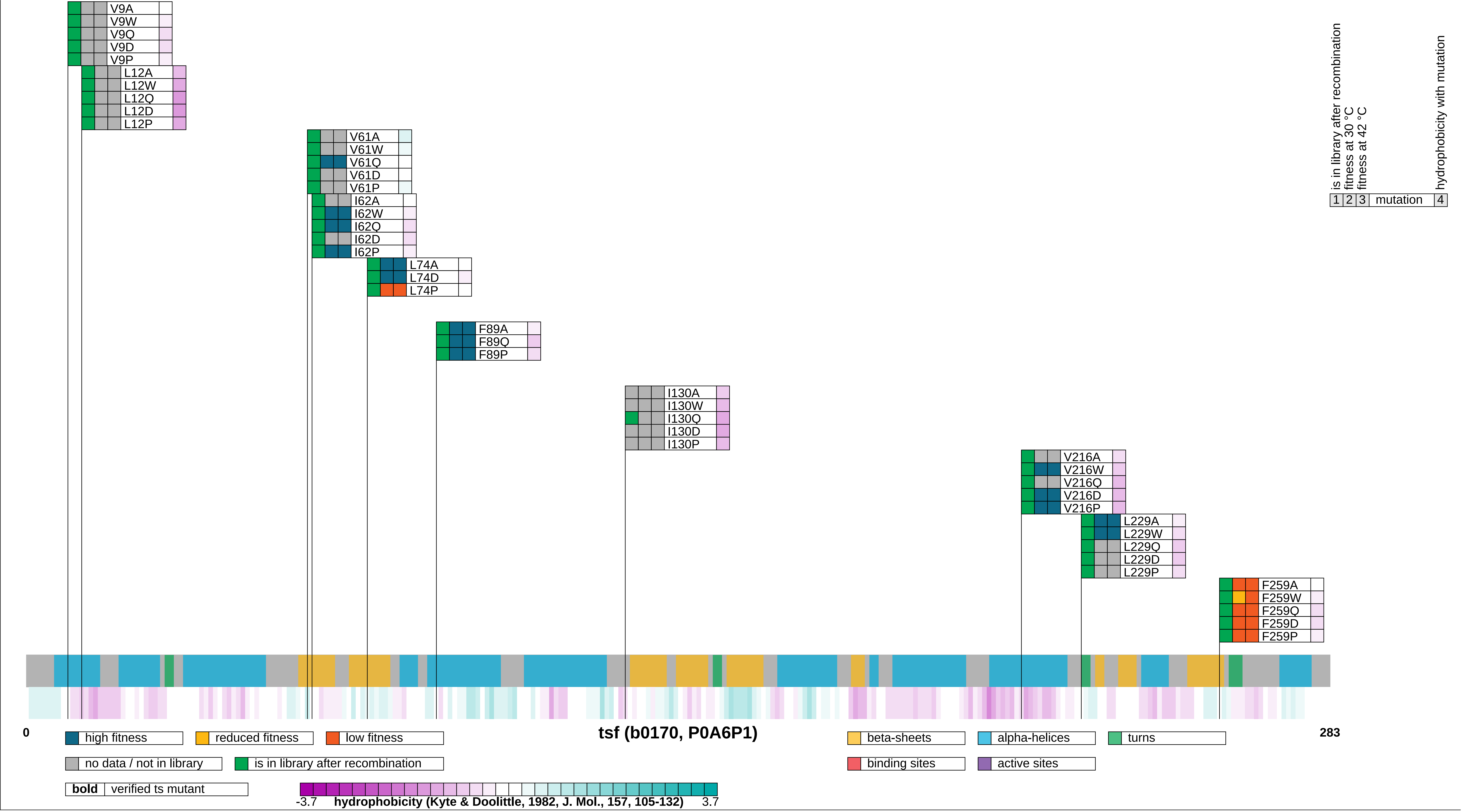

V9A
V9W
V9Q
V9D
V9P
L12A
L12W
L12Q
L12D
L12P
V61A
V61W
V61Q
V61D
V61P
I62A
I62W
I62Q
I62D
I62P
L74A
L74D
L74P
F89A
F89Q
F89P
I130A
I130W
I130Q
I130D
I130P
V216A
V216W
V216Q
V216D
V216P
L229A
L229W
L229Q
L229D
L229P
F259A
F259W
F259Q
F259D
F259P
is in library after recombination
fitness at 30 °C
fitness at 42 °C
hydrophobicity with mutation
1 2 3 mutation 4
0
283
tsf (b0170, P0A6P1)
high fitness
reduced fitness
low fitness
no data / not in library
is in library after recombination
bold verified ts mutant
beta-sheets
alpha-helices
turns
binding sites
active sites
-3.7
hydrophobicity (Kyte & Doolittle, 1982, J. Mol., 157, 105-132)
3.7

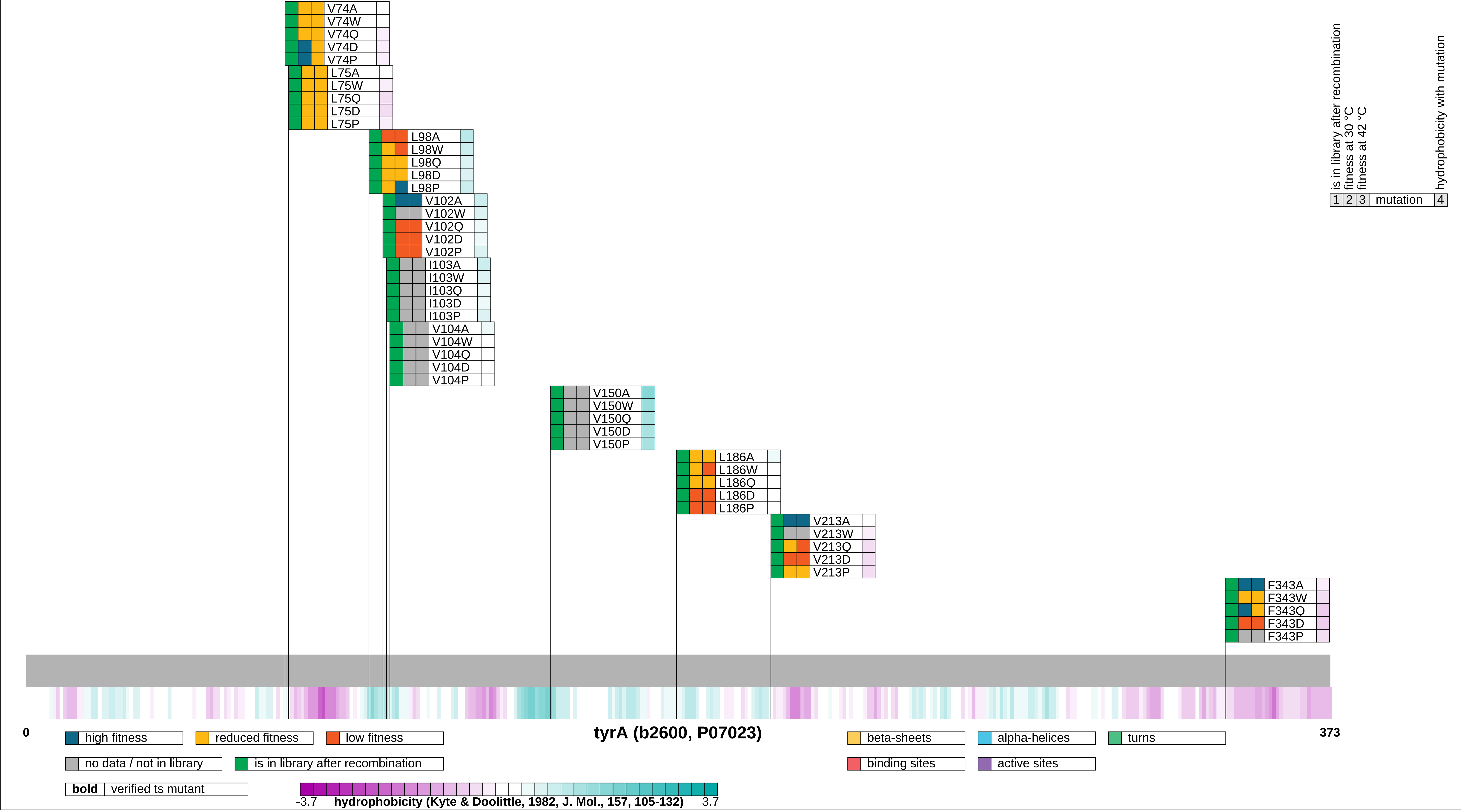
V74A
V74W
V74Q
V74D
V74P
L75A
L75W
L75Q
L75D
L75P
L98A
L98W
L98Q
L98D
L98P
V102A
V102W
V102Q
V102D
V102P
I103A
I103W
I103Q
I103D
I103P
V104A
V104W
V104Q
V104D
V104P
V150A
V150W
V150Q
V150D
V150P
L186A
L186W
L186Q
L186D
L186P
V213A
V213W
V213Q
V213D
V213P
F343A
F343W
F343Q
F343D
F343P
is in library after recombination
fitness at 30 °C
fitness at 42 °C
hydrophobicity with mutation
1 2 3 mutation 4
0
tyrA (b2600, P07023)
373
high fitness
reduced fitness
low fitness
beta-sheets
alpha-helices
turns
no data / not in library
is in library after recombination
binding sites
active sites
bold verified ts mutant
-3.7
hydrophobicity (Kyte & Doolittle, 1982, J. Mol., 157, 105-132)
3.7

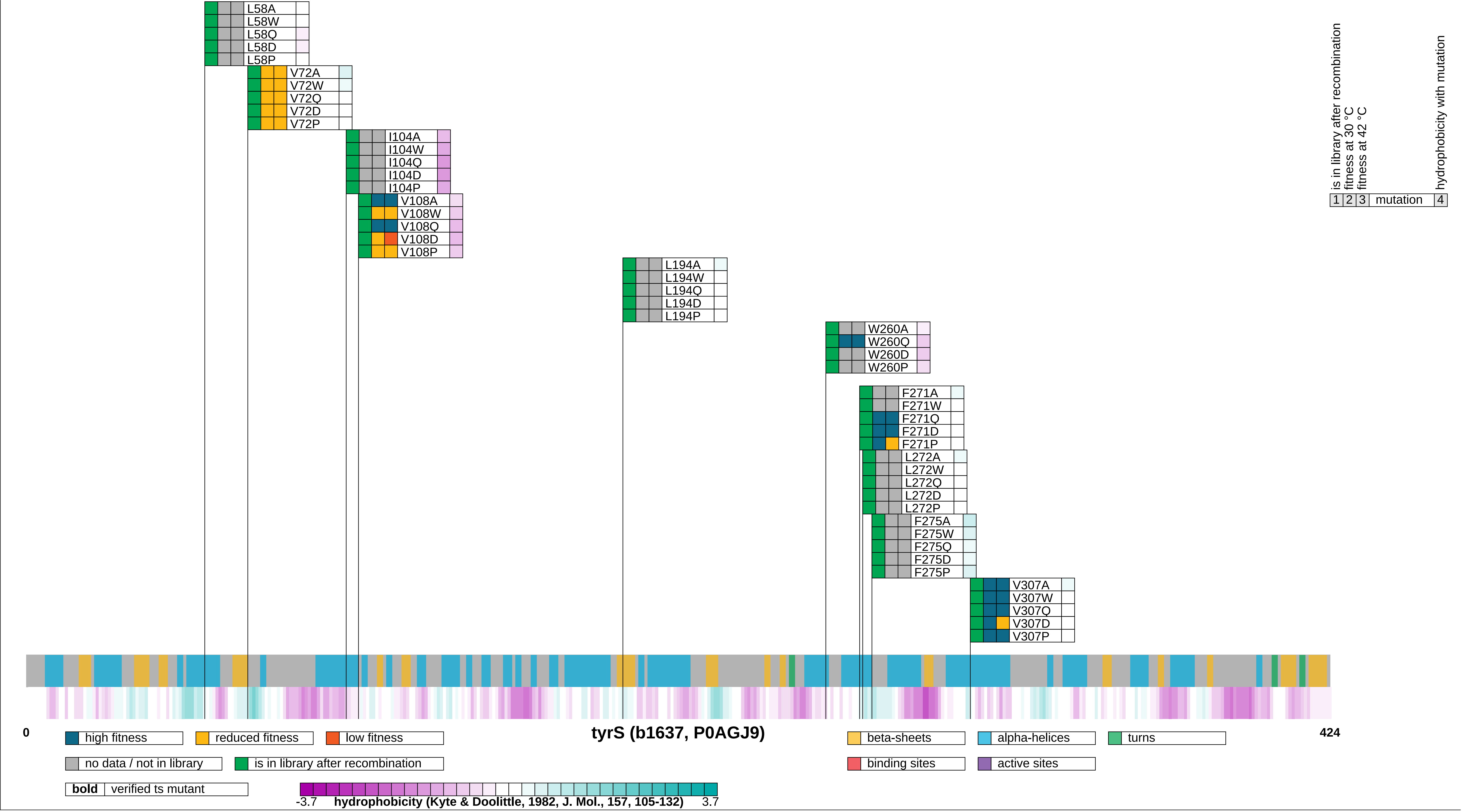

is in library after recombination
fitness at 30 °C
fitness at 42 °C
hydrophobicity with mutation
1 2 3 mutation 4
L58A
L58W
L58Q
L58D
L58P
V72A
V72W
V72Q
V72D
V72P
I104A
I104W
I104Q
I104D
I104P
V108A
V108W
V108Q
V108D
V108P
L194A
L194W
L194Q
L194D
L194P
W260A
W260Q
W260D
W260P
F271A
F271W
F271Q
F271D
F271P
L272A
L272W
L272Q
L272D
L272P
F275A
F275W
F275Q
F275D
F275P
V307A
V307W
V307Q
V307D
V307P
0
tyrS (b1637, P0AGJ9)
424
high fitness
reduced fitness
low fitness
beta-sheets
alpha-helices
turns
no data / not in library
is in library after recombination
binding sites
active sites
bold verified ts mutant
-3.7
hydrophobicity (Kyte & Doolittle, 1982, J. Mol., 157, 105-132)
3.7

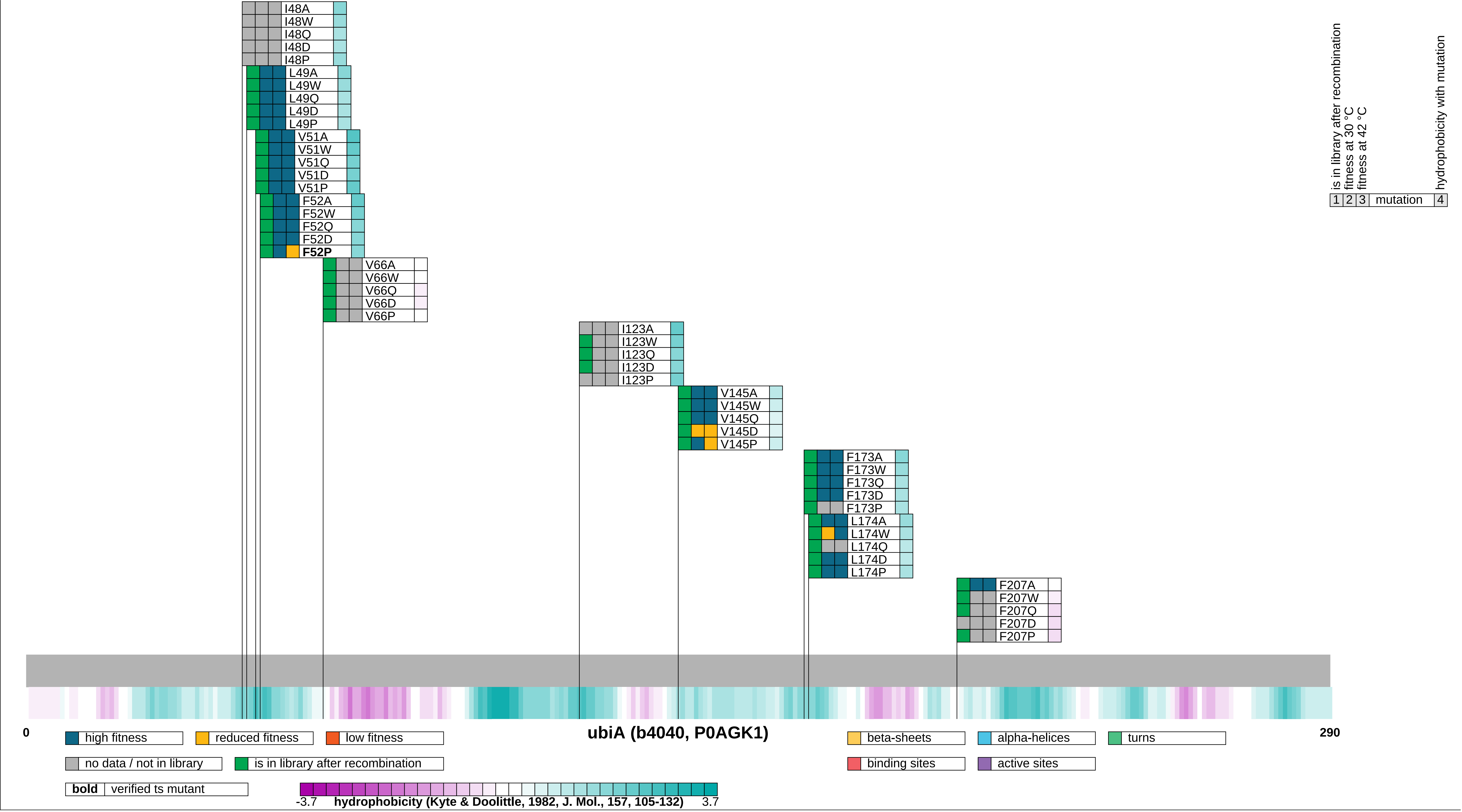

I48A
I48W
I48Q
I48D
I48P
L49A
L49W
L49Q
L49D
L49P
V51A
V51W
V51Q
V51D
V51P
F52A
F52W
F52Q
F52D
F52P
V66A
V66W
V66Q
V66D
V66P
I123A
I123W
I123Q
I123D
I123P
V145A
V145W
V145Q
V145D
V145P
F173A
F173W
F173Q
F173D
F173P
L174A
L174W
L174Q
L174D
L174P
F207A
F207W
F207Q
F207D
F207P
is in library after recombination
fitness at 30 °C
fitness at 42 °C
hydrophobicity with mutation
1 2 3 mutation 4
0
290
ubiA (b4040, P0AGK1)
high fitness
reduced fitness
low fitness
beta-sheets
alpha-helices
turns
no data / not in library
is in library after recombination
binding sites
active sites
bold verified ts mutant
-3.7
hydrophobicity (Kyte & Doolittle, 1982, J. Mol., 157, 105-132)
3.7

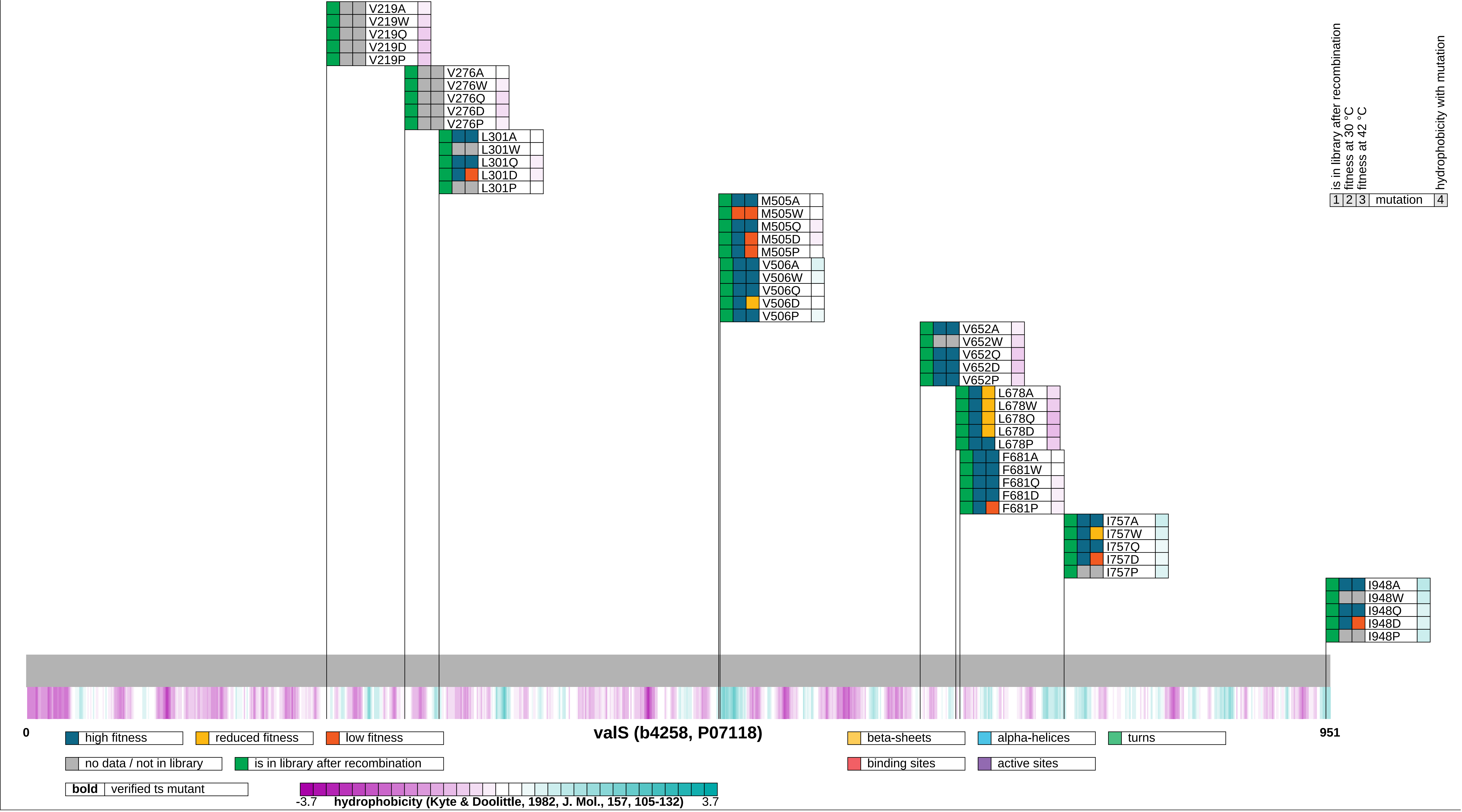
V219A
V219W
V219Q
V219D
V219P
V276A
V276W
V276Q
V276D
V276P
L301A
L301W
L301Q
L301D
L301P
M505A
M505W
M505Q
M505D
M505P
V506A
V506W
V506Q
V506D
V506P
V652A
V652W
V652Q
V652D
V652P
L678A
L678W
L678Q
L678D
L678P
F681A
F681W
F681Q
F681D
F681P
I757A
I757W
I757Q
I757D
I757P
I948A
I948W
I948Q
I948D
I948P
is in library after recombination
fitness at 30 °C
fitness at 42 °C
hydrophobicity with mutation
1 2 3 mutation 4
0
valS (b4258, P07118)
951
high fitness
reduced fitness
low fitness
no data / not in library
is in library after recombination
bold verified ts mutant
beta-sheets
alpha-helices
turns
binding sites
active sites
-3.7
hydrophobicity (Kyte & Doolittle, 1982, J. Mol., 157, 105-132)
3.7

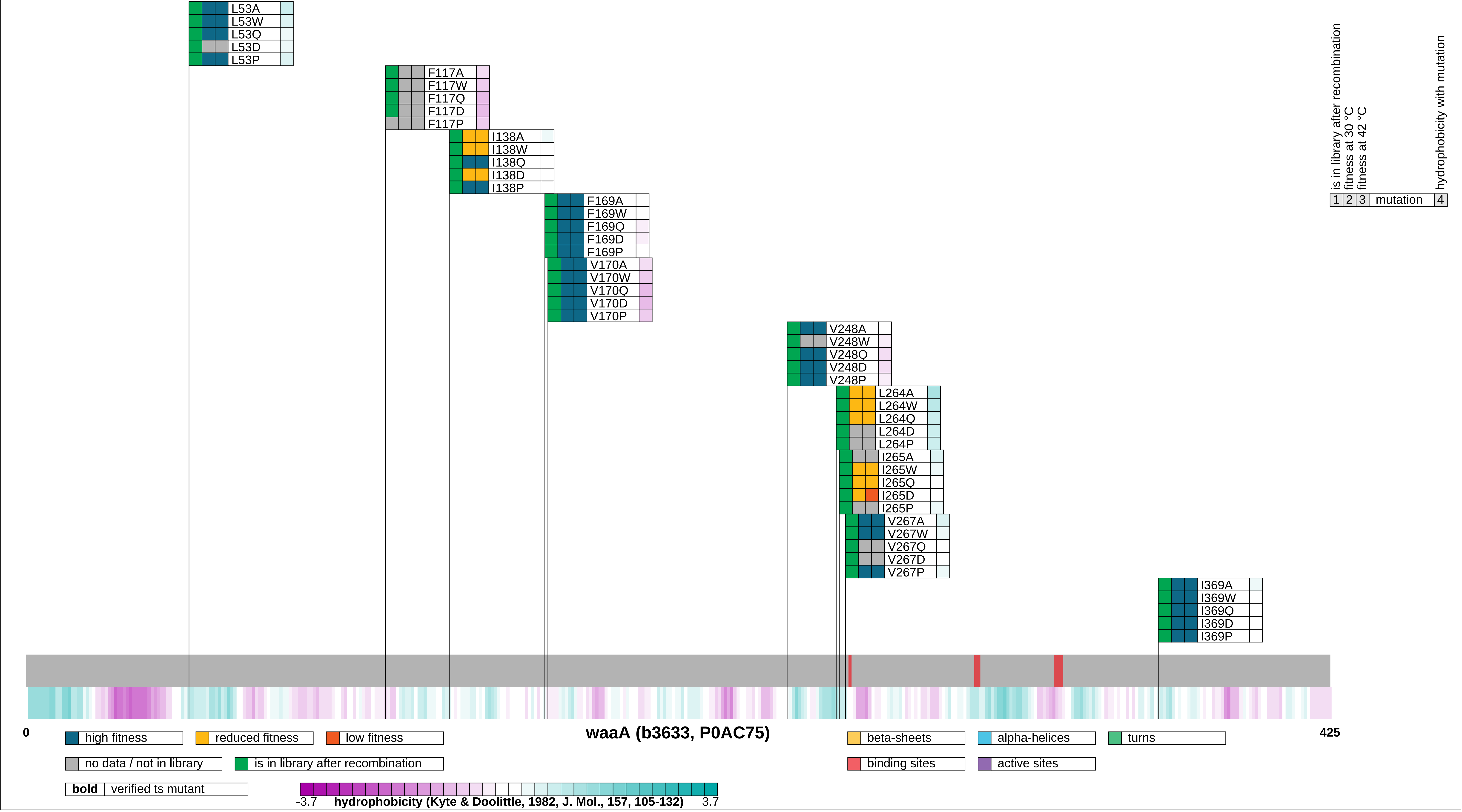

L53A
L53W
L53Q
L53D
L53P
F117A
F117W
F117Q
F117D
F117P
I138A
I138W
I138Q
I138D
I138P
F169A
F169W
F169Q
F169D
F169P
V170A
V170W
V170Q
V170D
V170P
V248A
V248W
V248Q
V248D
V248P
L264A
L264W
L264Q
L264D
L264P
I265A
I265W
I265Q
I265D
I265P
V267A
V267W
V267Q
V267D
V267P
I369A
I369W
I369Q
I369D
I369P
is in library after recombination
fitness at 30 °C
fitness at 42 °C
hydrophobicity with mutation
1 2 3 mutation 4
0
waaA (b3633, P0AC75)
425
high fitness
reduced fitness
low fitness
beta-sheets
alpha-helices
turns
no data / not in library
is in library after recombination
binding sites
active sites
bold verified ts mutant
-3.7 hydrophobicity (Kyte & Doolittle, 1982, J. Mol., 157, 105-132) 3.7

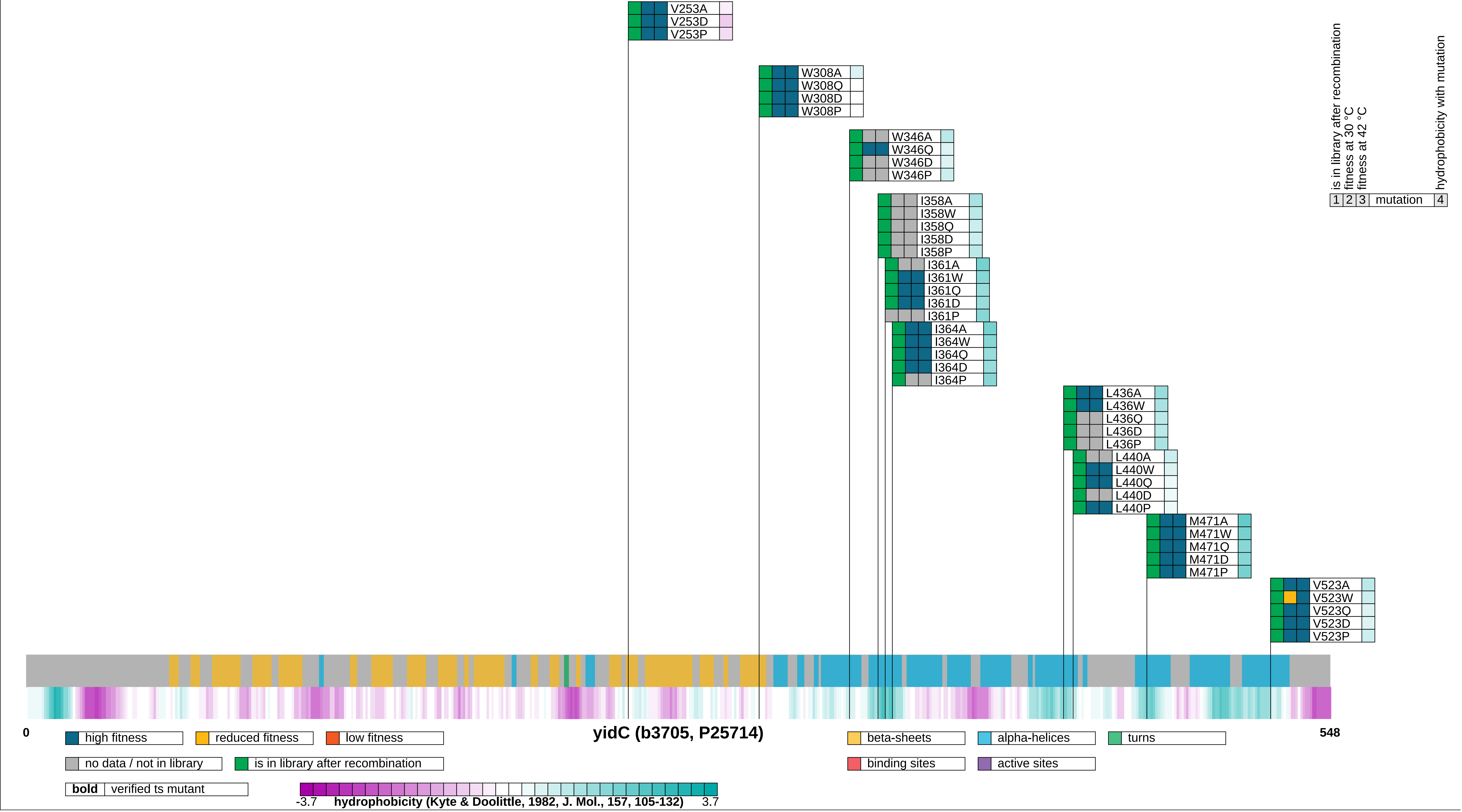
V253A
V253D
V253P
W308A
W308Q
W308D
W308P
W346A
W346Q
W346D
W346P
I358A
I358W
I358Q
I358D
I358P
I361A
I361W
I361Q
I361D
I361P
I364A
I364W
I364Q
I364D
I364P
L436A
L436W
L436Q
L436D
L436P
L440A
L440W
L440Q
L440D
L440P
M471A
M471W
M471Q
M471D
M471P
V523A
V523W
V523Q
V523D
V523P
is in library after recombination
fitness at 30 °C
fitness at 42 °C
hydrophobicity with mutation
1 2 3 mutation 4
0
yidC (b3705, P25714)
548
high fitness
reduced fitness
low fitness
beta-sheets
alpha-helices
turns
no data / not in library
is in library after recombination
binding sites
active sites
bold verified ts mutant
-3.7 hydrophobicity (Kyte & Doolittle, 1982, J. Mol., 157, 105-132) 3.7

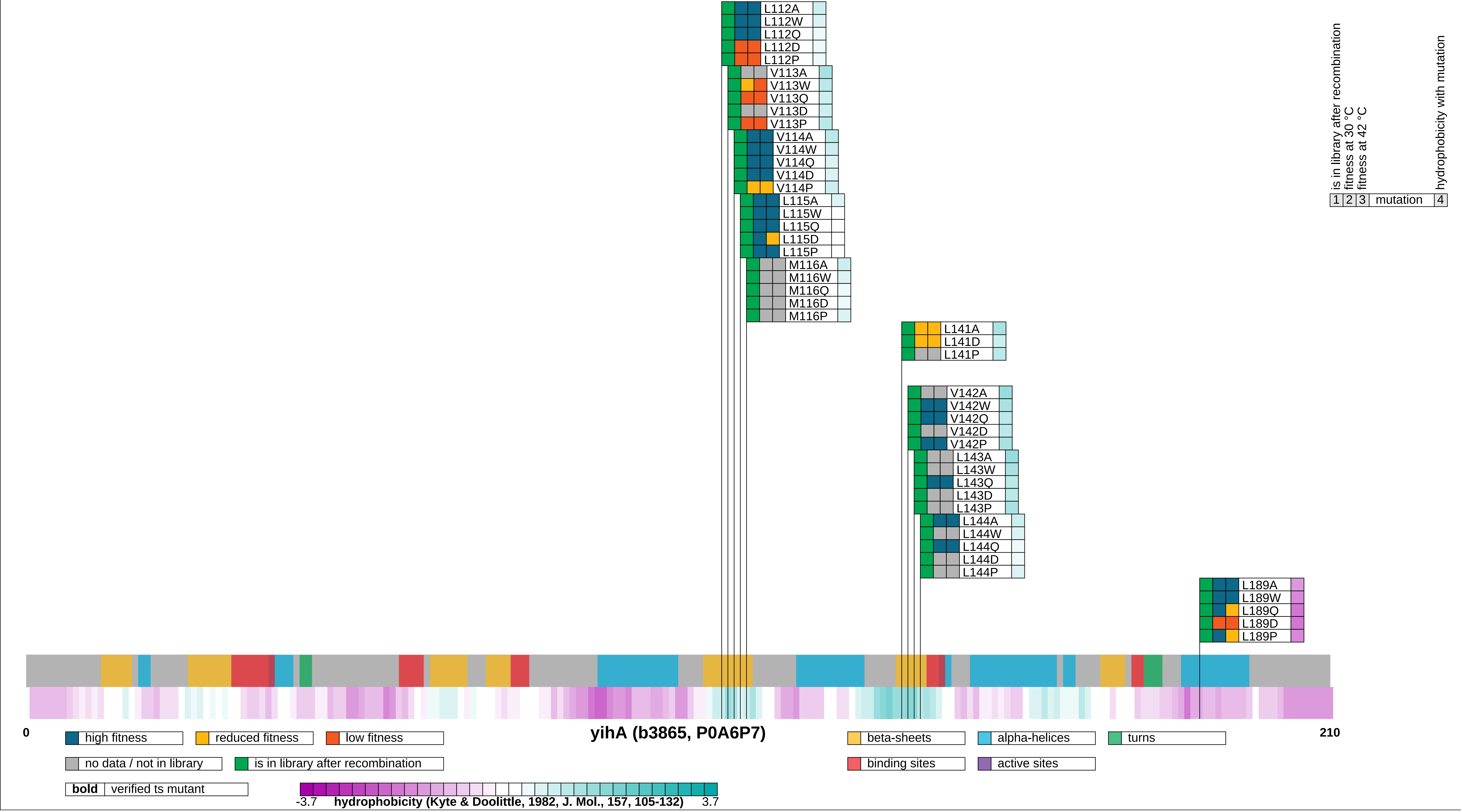

L112A
L112W
L112Q
L112D
L112P
V113A
V113W
V113Q
V113D
V113P
V114A
V114W
V114Q
V114D
V114P
L115A
L115W
L115Q
L115D
L115P
M116A
M116W
M116Q
M116D
M116P
L141A
L141D
L141P
V142A
V142W
V142Q
V142D
V142P
L143A
L143W
L143Q
L143D
L143P
L144A
L144W
L144Q
L144D
L144P
L189A
L189W
L189Q
L189D
L189P
is in library after recombination
fitness at 30 °C
fitness at 42 °C
hydrophobicity with mutation
1 2 3 mutation 4
0
yihA (b3865, P0A6P7)
210
high fitness
reduced fitness
low fitness
no data / not in library
is in library after recombination
bold verified ts mutant
beta-sheets
alpha-helices
turns
binding sites
active sites
-3.7 hydrophobicity (Kyte & Doolittle, 1982, J. Mol., 157, 105-132) 3.7

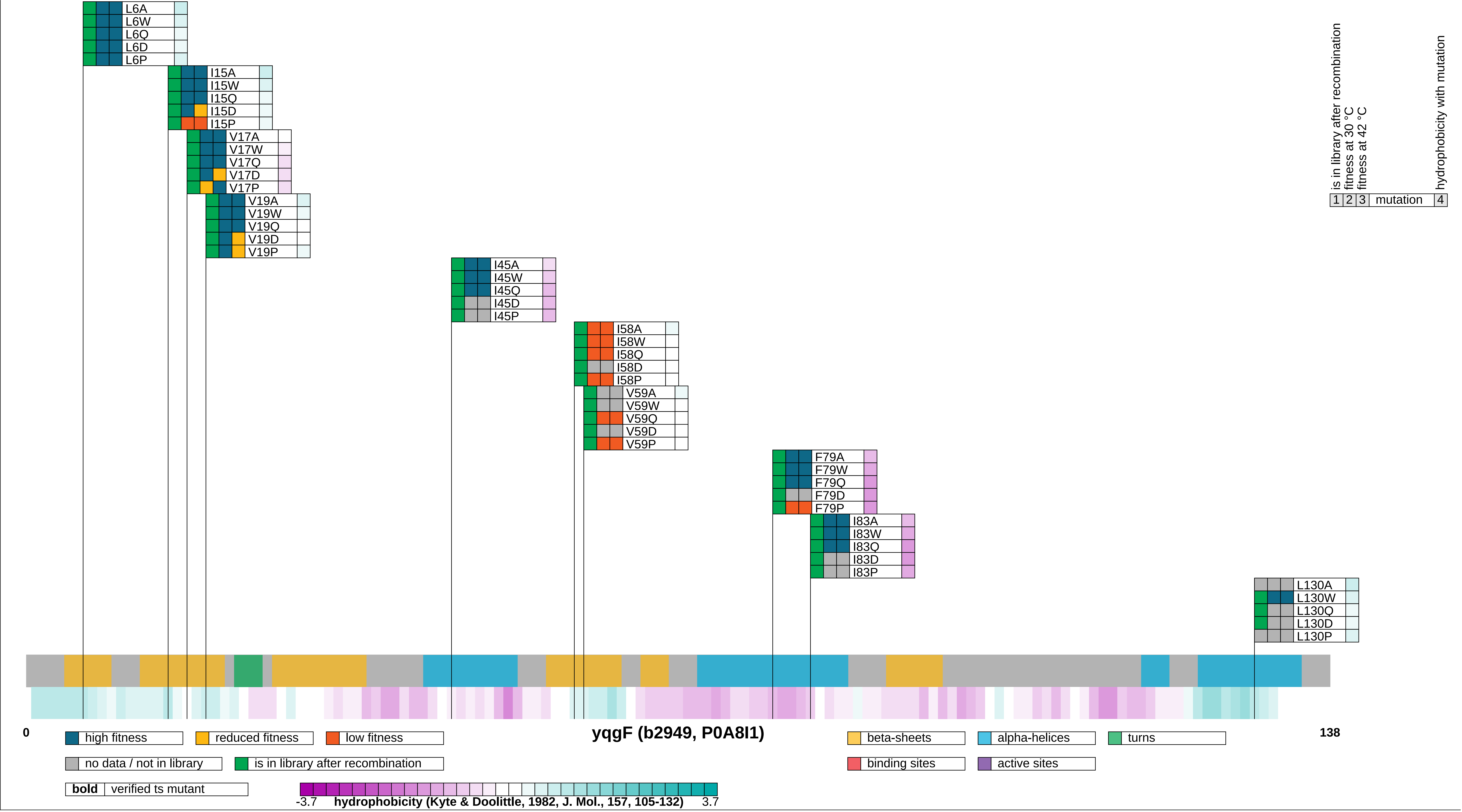

L6A
L6W
L6Q
L6D
L6P
I15A
I15W
I15Q
I15D
I15P
V17A
V17W
V17Q
V17D
V17P
V19A
V19W
V19Q
V19D
V19P
I45A
I45W
I45Q
I45D
I45P
I58A
I58W
I58Q
I58D
I58P
V59A
V59W
V59Q
V59D
V59P
F79A
F79W
F79Q
F79D
F79P
I83A
I83W
I83Q
I83D
I83P
L130A
L130W
L130Q
L130D
L130P
is in library after recombination
fitness at 30 °C
fitness at 42 °C
hydrophobicity with mutation
1 2 3 mutation 4
0
yqgF (b2949, P0A8I1)
138
high fitness
reduced fitness
low fitness
beta-sheets
alpha-helices
turns
no data / not in library
is in library after recombination
binding sites
active sites
bold verified ts mutant
-3.7 hydrophobicity (Kyte & Doolittle, 1982, J. Mol., 157, 105-132) 3.7

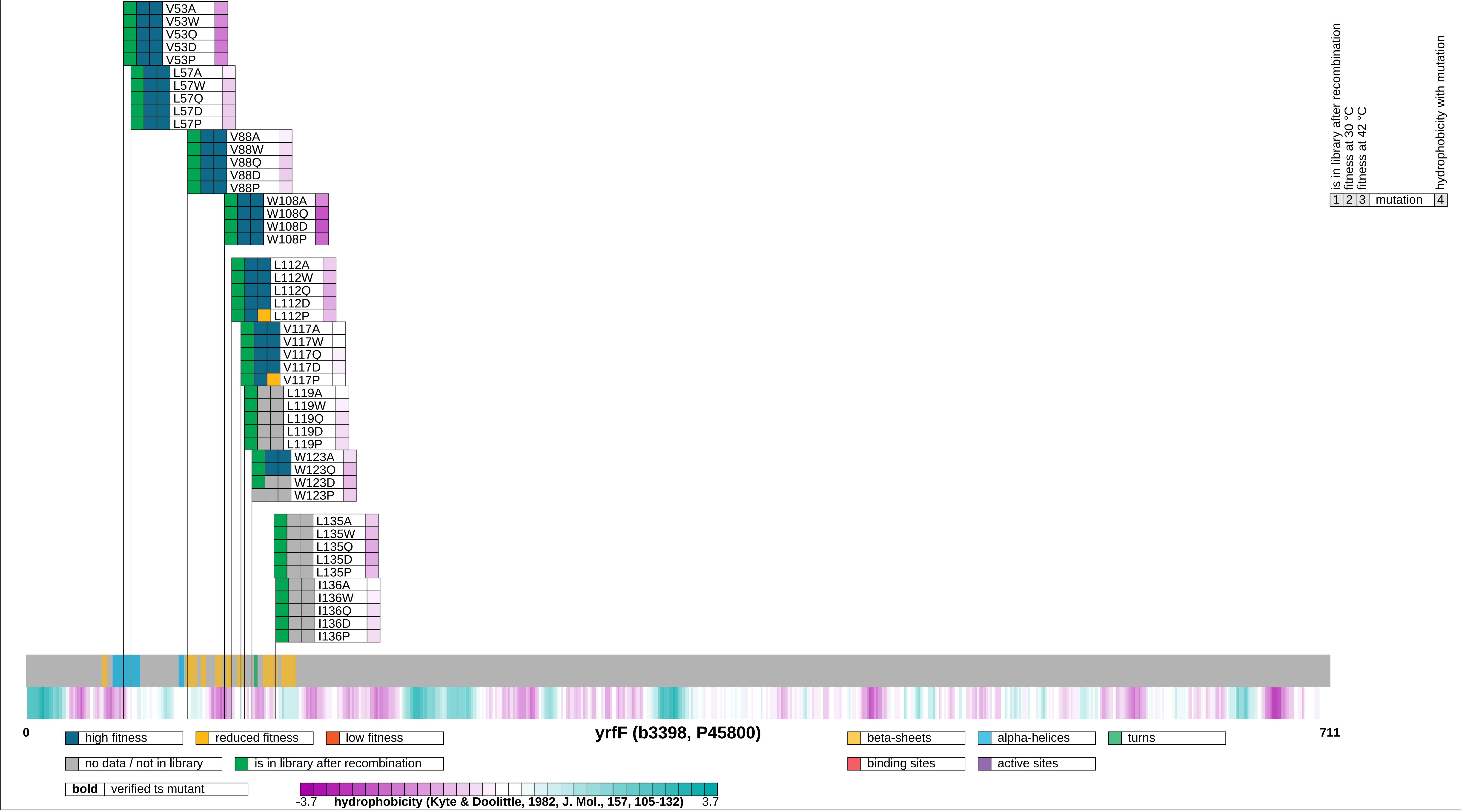
V53A
V53W
V53Q
V53D
V53P
L57A
L57W
L57Q
L57D
L57P
V88A
V88W
V88Q
V88D
V88P
W108A
W108Q
W108D
W108P
L112A
L112W
L112Q
L112D
L112P
V117A
V117W
V117Q
V117D
V117P
L119A
L119W
L119Q
L119D
L119P
W123A
W123Q
W123D
W123P
L135A
L135W
L135Q
L135D
L135P
I136A
I136W
I136Q
I136D
I136P
is in library after recombination
fitness at 30 °C
fitness at 42 °C
hydrophobicity with mutation
1 2 3 mutation 4
0
yrfF (b3398, P45800)
711
high fitness
reduced fitness
low fitness
beta-sheets
alpha-helices
turns
no data / not in library
is in library after recombination
binding sites
active sites
bold verified ts mutant
-3.7
hydrophobicity (Kyte & Doolittle, 1982, J. Mol., 157, 105-132)
3.7

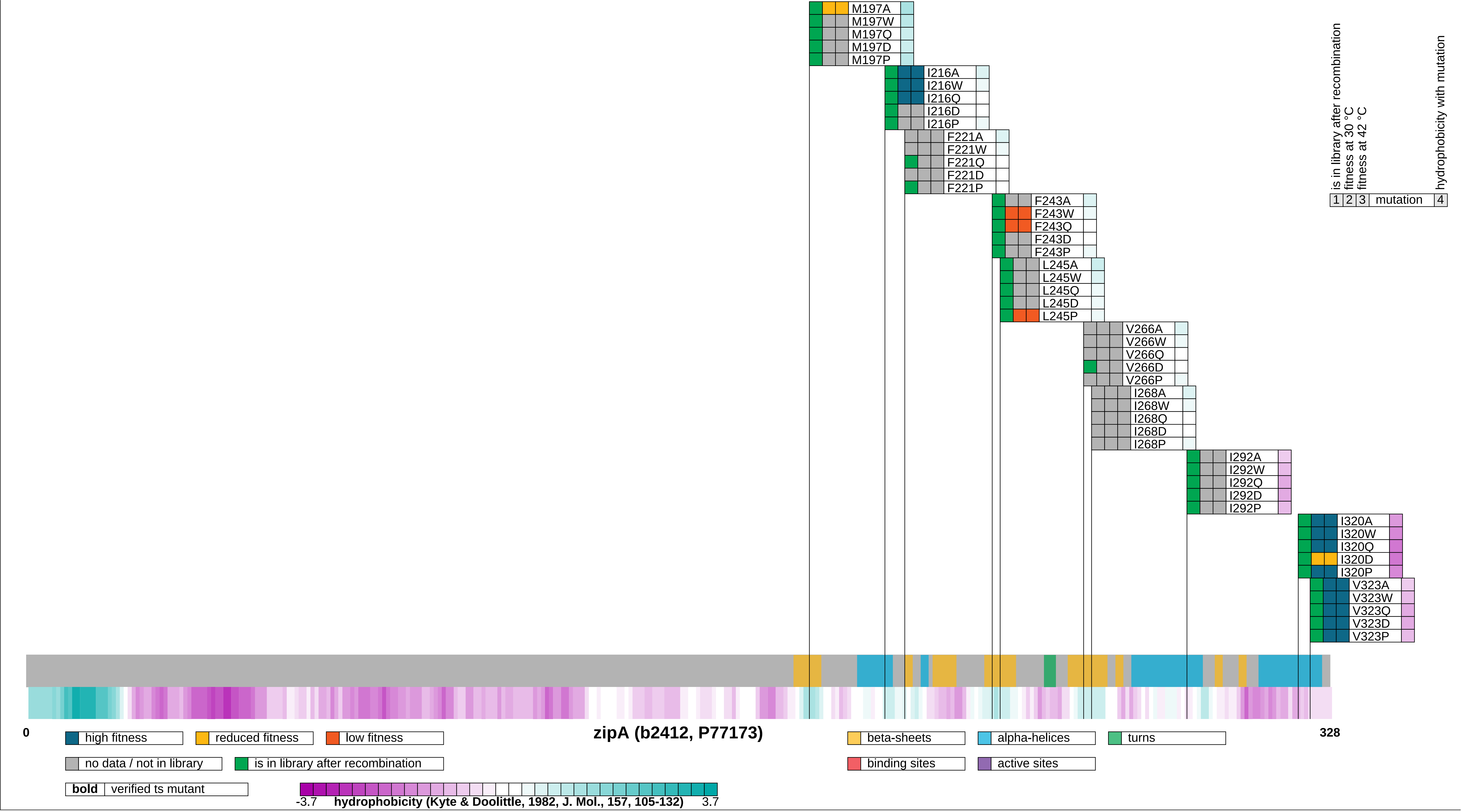

M197A
M197W
M197Q
M197D
M197P
I216A
I216W
I216Q
I216D
I216P
F221A
F221W
F221Q
F221D
F221P
F243A
F243W
F243Q
F243D
F243P
L245A
L245W
L245Q
L245D
L245P
V266A
V266W
V266Q
V266D
V266P
I268A
I268W
I268Q
I268D
I268P
I292A
I292W
I292Q
I292D
I292P
I320A
I320W
I320Q
I320D
I320P
V323A
V323W
V323Q
V323D
V323P
is in library after recombination
fitness at 30 °C
fitness at 42 °C
hydrophobicity with mutation
1 2 3 mutation 4
0
zipA (b2412, P77173)
328
high fitness
reduced fitness
low fitness
beta-sheets
alpha-helices
turns
no data / not in library
is in library after recombination
binding sites
active sites
bold verified ts mutant
-3.7 hydrophobicity (Kyte & Doolittle, 1982, J. Mol., 157, 105-132) 3.7
