# Supplementary material for: Mapping temperature‐sensitive mutations at a genome scale to engineer growth switches in Escherichia coli
Source: Mol Syst Biol. 2023 Aug 29;19(10):e11596. doi: 10.15252/msb.202311596 (PMC10568205; doi:10.15252/msb.202311596)
Supplement: Supplementary file 20 [file MSB-19-e11596-s003.pdf]

molecular systems biology

Mapping temperature-sensitive mutations at a genome-scale to engineer growth-switches in E. coli

Thorben Schramm, Paul Lubrano, Vanessa Pahl, Amelie Stadelmann, Andreas Verhuelsdonk, and Hannes Link
DOI: 10.15252/msb.202311596

Corresponding author(s): Hannes Link (hannes.link@uni-tuebingen.de)

Review Timeline:

Submission Date:	14th Feb 23
Editorial Decision:	11th Apr 23
Revision Received:	18th Jul 23
Editorial Decision:	8th Aug 23
Revision Received:	14th Aug 23
Accepted:	14th Aug 23

Editor: Maria Polychronidou

Transaction Report:

(Note: With the exception of the correction of typographical or spelling errors that could be a source of ambiguity, letters and reports are not edited. Depending on transfer agreements, referee reports obtained elsewhere may or may not be included in this compilation. Referee reports are anonymous unless the Referee chooses to sign their reports.)

11th Apr 2023

RE: MSB-2023-11596, Mapping temperature-sensitive mutations at a genome-scale to engineer growth-switches in E. coli

Dear Hannes,

Thank you again for submitting your work to Molecular Systems Biology. I apologise for the delay in sending you a decision. Unfortunately, despite multiple reminders, we have not received a report from reviewer #2. We have now heard back from two of the three reviewers who agreed to evaluate your study. In the interest of time and given that the recommendations of the two reviewers are similar, we have decided to proceed with making a decision based on these two reports. As you will see below, the reviewers appreciate that the study represents a relevant contribution to the field. However, they raise a series of concerns, which we would ask you to address in a revision.

The reviewers' recommendations are quite clear and I therefore see no need to repeat any of them here. All issues raised would need to be satisfactorily addressed. Please let me know in case you would like to discuss in further detail any of the issues raised, I would be happy to schedule a call.

On a more editorial level, we would ask you to address the following points:

- Please provide a .doc version of the manuscript text (including legends for main figures) and individual production quality figure files for the main Figures (one file per figure).

- We have replaced Supplementary Information by the Expanded View (EV format). In this case, all additional figures can be included in a PDF called Appendix. Appendix figures should be labeled and called out as: "Appendix Figure S1, Appendix Figure S2... Appendix Table S1..." etc. Each legend should be below the corresponding Figure/Table in the Appendix. Please include a Table of Contents in the beginning of the Appendix. For detailed instructions regarding expanded view please refer to our Author Guidelines: .

- Supplementary Tables S1-S6 should be provided as EV Datasets. Please provide one file per dataset. In each file, a description of the dataset should be provided in a separate tab.

- Please provide a "standfirst text" summarizing the study in one or two sentences (approximately 250 characters), three to four "bullet points" highlighting the main findings and a "synopsis image" (550px width and max 400px height, jpeg format) to highlight the paper on our homepage.

- All Materials and Methods need to be described in the main text. We would encourage you to use 'Structured Methods', our new Materials and Methods format. According to this format, the Material and Methods section should include a Reagents and Tools Table (listing key reagents, experimental models, software and relevant equipment and including their sources and relevant identifiers) followed by a Methods and Protocols section in which we encourage the authors to describe their methods using a step-by-step protocol format with bullet points, to facilitate the adoption of the methodologies across labs. More information on how to adhere to this format as well as downloadable templates (.doc or .xls) for the Reagents and Tools Table can be found in our author guidelines: . An example of a Method paper with Structured Methods can be found here:

- Please include a Data availability section describing how the data and code have been made available. This section needs to be formatted according to the example below:
The datasets and computer code produced in this study are available in the following databases:
- Chip-Seq data: Gene Expression Omnibus GSE46748 (https://www.ncbi.nlm.nih.gov/geo/query/acc.cgi?acc=GSE46748)
- Modeling computer scripts: GitHub (https://github.com/SysBioChalmers/GECKO/releases/tag/v1.0)
- [data type]: [full name of the resource] [accession number/identifier] ([doi or URL or identifiers.org/DATABASE:ACCESSION])

- For data quantification: please specify the name of the statistical test used to generate error bars and P values, the number (n) of independent experiments (specify technical or biological replicates) underlying each data point and the test used to calculate p-values in each figure legend. The figure legends should contain a basic description of n, P and the test applied. Graphs must include a description of the bars and the error bars (s.d., s.e.m.).

- Please include a "Disclosure & Competing Interests Statement" in the main text.

- When you resubmit your manuscript, please download our CHECKLIST (https://bit.ly/EMBOPressAuthorChecklist) and include the completed form in your submission.
Please note that the Author Checklist will be published alongside the paper as part of the transparent process (https://www.embopress.org/page/journal/17444292/authorguide#transparentprocess).

If you feel you can satisfactorily deal with these points and those listed by the referees, you may wish to submit a revised version of your manuscript. Please attach a covering letter giving details of the way in which you have handled each of the points raised by the referees. A revised manuscript will be once again subject to review and you probably understand that we can give you no guarantee at this stage that the eventual outcome will be favorable.

Best wishes,

Maria

Maria Polychronidou, PhD
Senior Editor
Molecular Systems Biology

We realize that it is difficult to revise to a specific deadline. In the interest of protecting the conceptual advance provided by the work, we recommend a revision within 3 months (10th Jul 2023). Please discuss the revision progress ahead of this time with the editor if you require more time to complete the revisions. Use the link below to submit your revision:

https://msb.msubmit.net/cgi-bin/main.plex

IMPORTANT: When you send your revision, we will require the following items:
1. the manuscript text in LaTeX, RTF or MS Word format
2. a letter with a detailed description of the changes made in response to the referees. Please specify clearly the exact places in the text (pages and paragraphs) where each change has been made in response to each specific comment given
3. three to four 'bullet points' highlighting the main findings of your study
4. a short 'blurb' text summarizing in two sentences the study (max. 250 characters)
5. a 'thumbnail image' (550px width and max 400px height, Illustrator, PowerPoint or jpeg format), which can be used as 'visual title' for the synopsis section of your paper.
6. Please include an author contributions statement after the Acknowledgements section (see https://www.embopress.org/page/journal/17444292/authorguide)
7. Please complete the CHECKLIST available at (https://bit.ly/EMBOPressAuthorChecklist).
Please note that the Author Checklist will be published alongside the paper as part of the transparent process (https://www.embopress.org/page/journal/17444292/authorguide#transparentprocess).
8. When assembling figures, please refer to our figure preparation guideline in order to ensure proper formatting and readability in print as well as on screen:
https://bit.ly/EMBOPressFigurePreparationGuideline
See also figure legend guidelines: https://www.embopress.org/page/journal/17444292/authorguide#figureformat
9. Please note that corresponding authors are required to supply an ORCID ID for their name upon submission of a revised manuscript (EMBO Press signed a joint statement to encourage ORCID adoption).
(https://www.embopress.org/page/journal/17444292/authorguide#editorialprocess)
Currently, our records indicate that there is no ORCID associated with your account.

Please click the link below to provide an ORCID:
Link Not Available

10. At EMBO Press we ask authors to provide source data for the main manuscript figures. Our source data coordinator will contact you to discuss which figure panels we would need source data for and will also provide you with helpful tips on how to upload and organize the files.

The system will prompt you to fill in your funding and payment information. This will allow Wiley to send you a quote for the article processing charge (APC) in case of acceptance. This quote takes into account any reduction or fee waivers that you may be eligible for. Authors do not need to pay any fees before their manuscript is accepted and transferred to the publisher.

EMBO Press participates in many Publish and Read agreements that allow authors to publish Open Access with reduced/no publication charges. Check your eligibility: https://authorservices.wiley.com/author-resources/Journal-Authors/open-access/affiliation-policies-payments/index.html

As a matter of course, please make sure that you have correctly followed the instructions for authors as given on the submission website.

*** PLEASE NOTE *** As part of the EMBO Press transparent editorial process initiative (see our Editorial at

https://dx.doi.org/10.1038/msb.2010.72), Molecular Systems Biology publishes online a Review Process File with each accepted manuscripts. This file will be published in conjunction with your paper and will include the anonymous referee reports, your point-by-point response and all pertinent correspondence relating to the manuscript. If you do NOT want this File to be published, please inform the editorial office at msb@embo.org within 14 days upon receipt of the present letter.

Reviewer #1:

The manuscript by Schramm et al presents a large CRISPR base-editing screen for temperature sensitive alleles of genes essential for E. coli growth on minimal media. Beginning with >15000 mutants, the authors create a set of 94 validated ts-alleles. This resource significantly expands the number of curated E. coli ts-alleles available. They also go on to show several examples of the usefulness of these strains for metabolic studies. Overall, this is a strong manuscript which makes important technical contributions and creates new resources. Below are some suggestions for improvement.

1. Going from 250 in the sublibrary to the 94 randomly selection strains, seems like an important step. How many of the directly tested clones failed to reproduce temperature sensitivity when tested one-by-one? If none, why not isolate the majority of all 250 strains?

2. How many of the 94 strains hit the same gene? Comparison's of allelic series for ts-alleles would be useful at an early stage of the manuscript if the authors bothered to isolate multiple mutant alleles of ts-residues and found different behavior.

3. What does the high drop-out rate at 30C tell you about TSPred? Could you use the dataset to make better TS predictions? I think showing some value in the data on the hits and misses from the ts-experiment would add tremendously to the paper. (see my comment above about a potential allelic series). Basically, does this big study teach us anything about how to more efficiently predict and engineer ts-alleles in proteins.

4. The value of the arginine strain experiments in Figure 5 needs to be better articulated. Producing a strain that is 32% of maximum does not seem that great? I understand the value of non-growing strains producing metabolites but it is not well articulated in the manuscript. Can you provide a reference or rationale for the interest in non-growing bacteria to help readers put this in context.

5. Are many of the ts-alleles simple auxotrophs? Have the authors compared growth on rich or defined/supplemented media to confirm this or not? It seems plausible that some would be simple auxotrophs while others might not be. This could be a simple assay that would add to characterization of the collection (e.g. spot dilution or liquid growth assays of the alleles on minimal or rich media at different temperatures).

6. Statistical comparisons are not mentioned in the paper. While I understand differences may be large in some assays, a proper statistical treatment of the data needs to be highlighted in the Figures/legends.

7. For some data, especially in Figure 4/5, use of a mock CRISPR edited control (e.g. editing with a WT template) would be better than simply a non-edited strain.

8. For Figure 4, a control strain should be used for each temperature

9. For Figure 5, are the authors concerned about modeling a linear increase between 6 and 24 hours? Perhaps the arginine concentration plateaus after 12 or 16 hours? Is there a way to present the data that doesn't simply join these datapoints with a line?

Reviewer #3:

General Comments
Schramm et al describe a high-throughput method for constructing temperature-sensitive(TS) alleles of E. coli essential genes. While TS alleles represent a valuable genetic tool for exploring essential gene function, there exist few generalizable and scalable approaches for constructing such conditional alleles. This study describes a highly useful strategy that should be applicable to other systems and thus it is of interest to general research community. The manuscript is written clearly and the figures are highly informative. TS alleles can be incredibly powerful reagents for functional analysis and this study clearly highlights the potential for applying CRISPR-based approaches to generate these valuable reagents.

Specific Comments
• The authors do an excellent job of highlighting specific examples through the manuscript. However, in some cases it is unclear if these are examples are anecdotal or if they reflect more general trends.

• The authors constructed ~15,000 mutant alleles corresponding to 346 essential genes. A subset of ~6000 mutants were inviable at permissive temperature, while the remaining ~9000 mutants were viable. It would be useful to comment on the nature of these mutations. Did they observe any trends related to structural and/or biophysical properties of the essential proteins, which can be used to improve prediction of TS mutations. A systematic analysis of this kind would be incredibly valuable for similar essential gene analyses in other systems (ie human cell lines) that are more difficult to study at scale

• In total, the original screen 1269 putative TS mutants that grew normally at permissive temperature but showed a significant growth defect at high temperature representing a high confidence set of TS mutants. The vast majority of mutants that exhibited a growth defect at 30oC also showed a growth defect at 42oC. Did most of these mutants show a more severe fitness defect at 42oC relative to their growth at permissive temperature? These would also represent TS mutants and would impact conclusions related to the prevalence of conditional essential gene mutations and the efficacy of the approach used to predict such mutations. Related to the previous comment, did the authors look for/identify specific gene or protein features that are related to the strength of the TS phenotype?

• For each gene, how many different alleles (on average) were identified? What was the maximal number of unique alleles identified for any given gene? Did different alleles of the same gene have similar growth kinetics (e.g. switch vs gradual growth) or did they differ?

• This work includes some interesting metabolomics analysis. In total, 94 TS alleles were reconstructed and subjected to metabolomics analysis. While most mutants had distinct metabolic profiles, 29 pairs of functionally related genes shared similar metabolic profiles. I would like to see a little more analysis of the gene set that includes these 94 TS alleles. How many of the 94 TS mutants encode functionally related gene pairs (i.e. what is the background rate of functionally related genes?). What is the functional breadth of the 94 genes examined?

• Given the background set of functionally related gene pairs, is 29 functionally related pairs that share similar metabolic profiles significantly enriched?

• Did the authors test if different TS alleles in the same essential gene yield similar metabolic profiles?

• Was there a relationship between the strength and/or growth kinetics associated with metabolic profile. For example, did the 24 strains that showed an increase in direct substrate metabolites tend to have stronger TS phenotypes or particular growth-temperature relationship?

• Was there anything particularly interesting about genes and corresponding TS mutants that affect a large number of metabolic pathways? For example, based on Fig 3D, several TS strains affected more than 30 pathways

We thank both reviewers for their constructive comments, on which we based new experiments and revisions of the manuscript. A point-by-point response is below, and the most important changes are:

- **Allelic series:** We re-constructed 14 TS mutants for *cysS* and measured growth at 6 temperatures (new figure “Fig EV2” and “Table EV4”).
- **Mock edit controls:** We constructed two mock edit controls (MetA and ThrB), in which we inserted a wild-type sequence including the silent PAM mutation and compared growth of the mock edit controls to the non-edited control (new figure “Appendix Fig S8”).
- **TS mutants in rich medium:** We cultured the 94 TS mutants in rich medium to identify auxotrophic strains (new figure “Appendix Fig S12” and “Table EV8”).
- **Re-analysis of the CRISPR screen:** We re-analyzed the data from the CRISPR screen to identify protein structure related properties and enriched mutations in two groups: (1) the putative TS mutations and (2) mutants with strong fitness defects (new figure Fig “EV1” and “Table EV3”).
- **Statistics:** The statistical treatment was improved throughout the entire manuscript.
- **Re-analysis of the metabolome data:** We re-analyzed the metabolome data using a random forest (RF) classifier. The RF model predicts growth phenotypes at 42°C from the metabolome data with a precision of ca. 70% (new figure “Appendix Fig S15”).

Response to Reviewer #1:

The manuscript by Schramm et al presents a large CRISPR base-editing screen for temperature sensitive alleles of genes essential for E. coli growth on minimal media. Beginning with >15000 mutants, the authors create a set of 94 validated ts-alleles. This resource significantly expands the number of curated E. coli ts-alleles available. They also go on to show several examples of the usefulness of these strains for metabolic studies. Overall, this is a strong manuscript which makes important technical contributions and creates new resources. Below are some suggestions for improvement.

1. Going from 250 in the sublibrary to the 94 randomly selection strains, seems like an important step. How many of the directly tested clones failed to reproduce temperature sensitivity when tested one-by-one? If none, why not isolate the majority of all 250 strains?

We agree with the reviewer that this is an important point, because it validates the results from the pooled screen. However, because we randomly isolated clones from the pool of 250 strains, it is difficult to determine how many strains failed to reproduce the TS phenotype. Therefore, we performed new experiments and validated the results from the pooled screen with an allelic series of *cysS*. Additionally, we better describe the results with the pool of 250 strains in the revised manuscript:

Line 441, Discussion: *"To validate that the CRISPR screen identified genuine TS mutants, we focused on the allelic series of cysS. To this end, we reconstructed 14 TS mutants of cysS, all of which exhibited temperature-dependent growth in plate reader cultivations. This confirmed that the CRISPR screen effectively identified authentic TS mutants. Additionally, we reconstructed a pool of 250 putative TS mutants and successfully confirmed a TS phenotype in 94 of them. However, for the remaining 156 strains, it has proven to be a challenge to determine whether they possess the TS phenotype or not. A conservative estimate is that only 94 out of the initial 250 strains are true TS mutants. However, it is important to note that the abundance of the 250 strains varied significantly, and we observed a bias towards highly abundant mutants during isolation of the strains. Therefore the 2016 random isolates have likely missed a significant portion of the 250 strains. Furthermore, measurements of TS phenotypes in plate readers were limited to a ca. 6-hour exponential phase. In contrast, the CRISPR screen allowed us to monitor TS phenotypes for a duration of 14 hours, because we back-diluted the cultures. Therefore, it is likely that the plate reader screening method may have missed some delayed TS phenotypes that have been captured in the extended time frame of the CRISPR screen. These factors suggest that the number of true TS mutants in the sub-library of 250 strains could potentially be higher than our conservative estimate of 94."*

Line 201, Main text: *"We then randomly isolated 2016 clones from the sub-library and tested them for temperature-sensitivity in 96-well plates. In total, 456 clones (22%) showed temperature sensitive growth and sequencing of the single clones identified 123 unique strains (Table EV6). Out of the 123 strains, we selected 94 strains for further analysis. Notably, the group of 94 strains was biased towards high abundant strains in the pool of 250 strains (p = 1.2e-06, Wilcoxon rank sum test, two-tailed, Appendix Fig S7A), which could explain why we picked them over others."*

Line 650, Methods section: *"The sub-library of 250 mutants (Table EV5) was constructed following the same protocol as for the large CRISPR library (also see M.3.1). After plating the pooled library onto LB agar plates with kanamycin and chloramphenicol, 2016 colonies were picked, cultivated in LB with kanamycin and chloramphenicol in 96-well plates at 30°C overnight, and cryo stocks prepared. Subsequently, growth was measured in plate reader cultivations at 30 °C and 42 °C. 456 isolates were selected for sequencing, out of which 123 were unique (Table EV6) and 94 selected for further work."*

2. How many of the 94 strains hit the same gene? Comparison's of allelic series for ts-alleles would be useful at an early stage of the manuscript if the authors bothered to isolate multiple mutant alleles of ts-residues and found different behavior.

We thank the reviewer for this suggestion and included comparison of allelic series at an early stage of the revised manuscript.

First, we re-analyzed the results of the CRISPR Screen and provide the information on how many TS mutations hit the same gene in the revised text and a new figure "Appendix Fig S5":

Line 180: *"The 1,269 putative TS alleles occurred in 267 of the 346 genes. On average, the CRISPR screen identified 3.7 TS alleles per gene, and 5 genes had more than 16 TS alleles: panM (30), panB (25), panC (17), cysN (17), and cysS (17) (Appendix Fig S5)."*

We also performed additional experiments, and reconstructed **14 TS mutants for a single gene (*cysS*)**:

Line 182: *"To validate some of the putative TS mutations, we focused on cysS, which encodes cysteine-tRNA ligase. The CRISPR screen identified 17 TS alleles for cysS, and we re-constructed 14 of these cysS mutants (Fig EV2). All the 14 cysS mutants that we tested showed significant TS phenotypes when we cultured them one-by-one in 96-well plates at 6 different temperatures (two-sample t-test, two-tailed, α = 0.05, n = 3, mutant vs. control at 42 °C, Fig EV2, Table EV4)."*

3. What does the high drop-out rate at 30C tell you about TSPred? Could you use the dataset to make better TS predictions? I think showing some value in the data on the hits and misses from the ts-experiment would add tremendously to the paper. (see my comment above about a potential allelic series). Basically, does this big study teach us anything about how to more efficiently predict and engineer ts-alleles in proteins.

We thank the reviewer for this suggestion. To address this point, we re-analyzed the data from the CRISPR screen by searching for enrichments in two groups of mutations:

- 1st group: 1269 TS mutation
- 2nd group: 8290 low-fitness mutations

We examined if these mutations preferentially occurred in alpha helices, beta sheets, turns, binding sites, or active sites. Further, we tested if specific amino acid changes are enriched and included the results in a new section in the main text:

Line 154: *"Having identified 8,290 mutations that strongly reduce fitness (low-fitness mutations) as well as 1,269 putative TS mutations (Fig EV1A), we tested if the mutations preferentially occur in alpha helices, beta sheets, active and binding sites, or turns (Fig EV1B). Further, we tested if certain amino acid changes are enriched in the two groups (Fig EV1C). Low-fitness mutants showed an enrichment for mutations in beta sheets (p = 7.8e-04, Fisher's exact test, one-tailed, Fig EV1B, Table EV3) and turns (p = 0.036). Moreover, low-fitness mutations were enriched in mutations that changed hydrophobic amino acids (Ile, Leu, Phe, Val, Met, Trp) into proline or aspartate (Fig EV1C).*

The putative TS mutations were not enriched in structure related features (Fig EV1B). However, putative TS mutations showed an enrichment of five mutations: Val-Gln, Phe-Gln, Iso-Gln, Val-Pro and Trp-Gln (p-values < 0.05, Fisher's exact test, one-tailed, Fig EV1C). Thus, although we had a limited search space of maximal 50 mutations per gene, each predicted with TSpred, we could identify simple design rules that may help to improve predictions of TS mutations."

These findings indicate that the CRISPR screen can indeed derive design rules for TS alleles. Therefore, future studies should exploit this to generate predictive models (e.g. with machine learning methods), ideally using a saturation mutagenesis. We describe this in the revised discussion:

Line 436: *"Despite the limited search space (max. 50 mutations per gene), we found that distinct amino acid changes were enriched in TS mutants. Thus, future studies could scale-up the approach presented here and search all TS alleles of essential E. coli proteins. Such a dataset may have the potential to train machine learning models that predict TS mutations in E. coli proteins and then transfer this knowledge to other organisms."*

4. The value of the arginine strain experiments in Figure 5 needs to be better articulated. Producing a strain that is 32% of maximum does not seem that great? I understand the value of non-growing strains producing metabolites but it is not well articulated in the manuscript. Can you provide a reference or rationale for the interest in non-growing bacteria to help readers put this in context.

In the revised manuscript, we better explain the advantages of two-stage bioprocesses and why thermo-switches are attractive for metabolic engineering. (Also referring to question 9, we now calculate the production rate for the initial 6 hours, which was 1.35 mmol $g_{DW}^{-1}h^{-1}$.)

Line 409, Main text: "*Despite the lower arginine production rate compared to growing cells (59%), producing arginine with non-growing E. coli has several advantages for process performance. The main advantage is a higher yield, because non-growing bacteria convert the feedstock mainly towards product synthesis. Another advantage is that non-growing bacteria are more stable and less likely to undergo mutations that lead to a decline in product formation. Finally, two-stage processes with non-growing cells simplify process control, because there is no need to control growth-related parameters like feeding rates."*

Line 61, Introduction: *"Thermal control of growth is especially important to realize two-stage bioprocesses, which separate a growth phase from a production phase with non-growing cells. Unlike processes with growing cells that require regulation of growth-related parameters, two-stage bioprocesses eliminate the need for controlling biomass levels via the supply of growth limiting nutrient. This allows for continuous processes without the additional complexity of growth control."*

Line 485, Discussion: "*It is likely that the choice of TS mutant has a significant impact on the longevity of (non-growing) producer cells and thus on the productivity of a bioprocess. However, more work is required to understand which TS mutants can improve the production of a particular bio-based product (Jang et al, 2023). Nevertheless, we expect that TS-based growth control create new possibilities to increase robustness and productivity of two-stage bioprocesses."*

5. Are many of the ts-alleles simple auxotrophs? Have the authors compared growth on rich or defined/supplemented media to confirm this or not? It seems plausible that some would be simple auxotrophs while others might not be. This could be a simple assay that would add to characterization of the collection (e.g. spot dilution or liquid growth assays of the alleles on minimal or rich media at different temperatures).

We thank the reviewer for this idea and performed the proposed experiment. For this purpose, we cultured the 94 TS mutants in rich medium (LB medium). Indeed, 49 out of 94 strains are conditional auxotrophs, because they lost their TS phenotype and grew like the control on rich medium (at 42°C). We included this in the revised manuscript:

Line 255*, "To test which TS mutants are conditionally auxotrophic, we cultured the TS mutants in rich medium (LB). 49 out of the 94 TS mutants indeed grew like the control strain at 42°C in LB medium (two-sample t-test, two-tailed, n = 3, α = 0.05, Appendix Fig S12, Table EV8). This is in sharp contrast to the significant growth defect of these 49 strains in minimal medium at 42°C (Fig 2B, Table EV7). These data indicate that the 49 TS mutants are conditionally auxotrophic and that supplementation of the missing nutrient restores growth. For example, 28 out of 31 TS mutants of genes in amino acid biosynthesis pathways lost their TS phenotype in rich LB medium, because de novo amino acid synthesis is not active in the presence of external amino acids."*

6. Statistical comparisons are not mentioned in the paper. While I understand differences may be large in some assays, a proper statistical treatment of the data needs to be highlighted in the Figures/legends.

We agree with this point and performed additional statistical analyses:

Growth assays with the 94 TS mutants, the *cysS* allelic series, and the mock edit controls were performed with n = 3 biological replicates, and we used a two-sample t-test (two-sided) between each mutant and the control strains at each temperature (new table "**Table EV4**", "**Table EV7**", and "**Table EV8**"). We further improved "**Fig 2B**", display standard deviations, and indicate the upper boundaries for the observed p-values for mutants vs. the control strain at 42 °C (all p-values were < 0.044) and 43 °C (all p-values were < 0.002), which indeed confirms that all TS mutants have a significantly lower growth rate than the control strain at these two non-permissive temperatures.

Metabolomics was performed with n = 3 biological replicates, and we calculated mod. z-scores based on the mean values of the 3 replicates. We used error propagation to estimate the standard deviations of the mod. z-score values based on the three replicates and show them in revised Figures "**Fig 3B**" and "**Appendix Fig S14**". Additionally, we provide background for selecting a mod. z-score cut-off of 3 for strong metabolite increases in the manuscript:

Line 277, *"We chose this mod. z-score value of 3 considering the distributions of standard deviations between replicates (n = 3) and mod. z-score values (Appendix Fig S13)."*

In brief, we confirmed that the data followed a normal distribution by a Kolmogorov-Smirnov test (p = 0, **Appendix Fig S13**). This means that a mod. z-score of 1 is equivalent to one standard deviation over the 95 measured strains. We then determined the distribution of standard deviations from the replicates and compared it to the mod. z-score distribution. This analysis shows that differences from replicates are, for the vast majority, smaller than a mod. z-score of 3.

Similarities of metabolic profiles: we improved our analysis of metabolite profile similarities using a Wilcoxon rank sum test (two-sided), which revealed that mutants within pathways and enzyme complexes have more similar profiles than other mutants. We included these results including p-values in a revised figure "**Fig EV4**".

Isolation bias of abundant mutants: Using a Wilcoxon rank sum test (two-sided), we show that the 94 isolated TS mutants were overly abundant in the sub-library of 250 mutants. The results are given with the new "**Appendix Fig S7A**".

Enrichment analyses were mostly based on Fisher's exact test (one-tailed), which helped identifying features of TS mutations and mutations with strong fitness defects (new "**Fig. EV1**"). We report the resulting p-values in the new "**Table EV3**".

7. For some data, especially in Figure 4/5, use of a mock CRISPR edited control (e.g. editing with a WT template) would be better than simply a non-edited strain.

We agree that a mock control is important and therefore we constructed two mock edited control strains, in which we only mutated the PAM site and re-introduced the wild-type sequence. We compared the growth of the mock-controls with the non-edited control strain at 6 different temperatures (new "**Appendix Fig S8**"). The results are included in the revised manuscript in two paragraphs:

Line 211: *"We also used two mock CRISPR edited controls to confirm that the editing itself has no effect on growth. In these mock edit controls we re-introduced a wild type sequence of thrA and metA together with a silent PAM mutation. Both the thrA-control and metA-control grew like the non-edited control strain (Appendix Fig S8, Table EV4)"*

Given the similarity between the non-edited control strain and the new mock controls, we decided to keep un-edited control in Figure 2 and also in Figure 4 (see also answer to next point).

8. For Figure 4, a control strain should be used for each temperature

We agree and performed the experiments with the non-edited control strain at all temperatures. These data are included in a **new "Figure 4"**.

9. For Figure 5, are the authors concerned about modeling a linear increase between 6 and 24 hours? Perhaps the arginine concentration plateaus after 12 or 16 hours? Is there a way to present the data that doesn't simply join these datapoints with a line?

We agree that the assumption of a linear increase between 6 and 24 is not justified. To avoid a false interpretation, we changed Figure 5 and used a linear fit only between 0 and 6 hours (results in a higher production rate). The 23 h and 24 h time point are shown separately. We also included this in the main text:

"During the initial 6 hours of the growth arrest, the arginine production rate was constant at 1.35 mmol g_{DW}^{-1} h^{-1}, which is 59% of the production rate (2.3 mmol g_{DW}^{-1} h^{-1})."

In the old Figure EV5, we had a very similar representation for the other metabolites, which we corrected in the new revised figure "Fig EV5".

Response to Reviewer #3:

General Comments

Schramm et al describe a high-throughput method for constructing temperature-sensitive(TS) alleles of E. coli essential genes. While TS alleles represent a valuable genetic tool for exploring essential gene function, there exist few generalizable and scalable approaches for constructing such conditional alleles. This study describes a highly useful strategy that should be applicable to other systems and thus it is of interest to general research community. The manuscript is written clearly and the figures are highly informative. TS alleles can be incredibly powerful reagents for functional analysis and this study clearly highlights the potential for applying CRISPR-based approaches to generate these valuable reagents.

Specific Comments

1. The authors do an excellent job of highlighting specific examples through the manuscript. However, in some cases it is unclear if these are examples are anecdotal or if they reflect more general trends.

We agree with the reviewer that this point was not always clear. The examples were used to illustrate general trends. In the revised manuscript, we combine the examples with cluster analyses to show the general trends:

Figure 1: MurE^{W381Q} and FtsQ^{I74Q}. These examples illustrate the different dynamics of TS mutants. In a new analysis, we clustered the time profiles of the 1,269 TS mutants and show the four general trends (e.g. strong TS phenotype and weaker TS phenotypes) in a new "Appendix Fig S4". We further included this analysis in the main text:

Line 169: *"To classify the strength of the TS mutations, we clustered the 1,269 TS mutants into 4 groups using k-means clustering (Appendix Fig S4). A group of 64 TS mutants had strong TS phenotypes, because these strains grew well at 30°C and disappeared fast from the library at 42°C (first cluster in Appendix Fig S4). The MurE^{W381Q} mutant is an example of a strain with such a strong TS phenotype (Fig 1D). Another group of 284 mutants disappeared with a time-delay from the 42°C cultures and the FtsQ^{I74Q} strain is an example for the delayed TS phenotypes (Fig 1D)."*

Figure 2: AccD^{V120W} and FolA^{M29P}. These are examples for a "switch-like response" and a "gradual response" of the growth rate towards changes in temperature. We integrate these examples better with the k-means clustering of all 94 strains in "Appendix Fig S10":

Line 237: *"K-mean clustering of the temperature-growth relationship identified 52 TS mutant with a switch-like response, and 42 TS mutants with a gradual response (Appendix Fig S10).*

The AccD^{V120W} mutant is an example for a switch-like response (Fig 2C), because a small change in temperature (38°C to 40°C) had a large effect on growth rates (0.81 h^{-1} to 0.02 h^{-1}). In contrast, the FolA^{M29P} mutant is an example for a gradual temperature-growth relationship (Fig 2C).“

Figure 2: HisC and MurE. These two strains showed a fast response in the pooled screen.

“Therefore, we selected two TS mutants from our panel of 94 strains that showed a fast response in the pooled competition assay: the MurE^{W381Q} mutant (Fig 1D) and the HisC^{I336D} mutant (Appendix Fig S11).“

2. The authors constructed ~15,000 mutant alleles corresponding to 346 essential genes. A subset of ~6000 mutants were inviable at permissive temperature, while the remaining ~9000 mutants were viable. It would be useful to comment on the nature of these mutations. Did they observe any trends related to structural and/or biophysical properties of the essential proteins, which can be used to improve prediction of TS mutations. A systematic analysis of this kind would be incredibly valuable for similar essential gene analyses in other systems (ie human cell lines) that are more difficult to study at scale

We agree and thank the reviewer for the suggestions. To address this, we re-analyzed the data from the CRISPR screen and examined two groups of mutations: (1) the TS mutations (1269 mutations) and (2) mutations that cause a strong fitness defect (8290 mutations). We indeed found enrichments in both groups and included the findings in a new paragraph in the main text as well as a new figure (“Fig EV1”):

Line 154: *“Having identified 8,290 mutations that strongly reduce fitness (low-fitness mutations) as well as 1,269 putative TS mutations (Fig EV1A), we tested if the mutations preferentially occur in alpha helices, beta sheets, active and binding sites, or turns (Fig EV1B). Further, we tested if certain amino acid changes are enriched in the two groups (Fig EV1C). Low-fitness mutants showed an enrichment for mutations in beta sheets (p = 7.8e-04, Fisher’s exact test, one-tailed, Fig EV1B, Table EV3) and turns (p = 0.036). Moreover, low-fitness mutations were enriched in mutations that changed hydrophobic amino acids (Ile, Leu, Phe, Val, Met, Trp) into proline or aspartate (Fig EV1C).*

The putative TS mutations were not enriched in structure related features (Fig EV1B). However, putative TS mutations showed an enrichment of five mutations: Val-Gln, Phe-Gln, Iso-Gln, Val-Pro and Trp-Gln (p-values < 0.05, Fisher’s exact test, one-tailed, Fig EV1C). Thus, although we had a limited search space of maximal 50 mutations per gene, each predicted with TSpred, we could identify simple design rules that may help to improve predictions of TS mutations.”

3. In total, the original screen 1269 putative TS mutants that grew normally at permissive temperature but showed a significant growth defect at high temperature representing a high

confidence set of TS mutants. The vast majority of mutants that exhibited a growth defect at 30oC also showed a growth defect at 42oC. Did most of these mutants show a more severe fitness defect at 42oC relative to their growth at permissive temperature? These would also represent TS mutants and would impact conclusions related to the prevalence of conditional essential gene mutations and the efficacy of the approach used to predict such mutations.

We agree with the reviewer that such stains are also TS mutants. Our analysis by k-means clustering included these strains (strains with reduced fitness at 30°C and low fitness at 42°C). In the revised manuscript, we describe this better using k-means clustering of only the 1,269 TS mutants (also see our answer to question 1):

Line 175: *"… The other two clusters captured 459 strains with mild TS phenotypes and 462 strains with weak TS phenotypes. These strains showed a growth defect at 30°C and a more severe growth defect at 42°C."*

Related to the previous comment, did the authors look for/identify specific gene or protein features that are related to the strength of the TS phenotype?

We found no relation between gene function and the strength of the TS phenotype. We illustrate this in a plot with one page per gene that shows the effect and location of each mutation. The according pdf-file is included as "File EV1". We included this in the main text:

Line 177: *"Thus, the growth phenotypes of the 1,269 putative TS mutants exhibit distinct characteristics, and it seems that they are primarily determined by the specific nature of each mutation rather than the overall gene function (File EV1)."*

4. For each gene, how many different alleles (on average) were identified? What was the maximal number of unique alleles identified for any given gene?

We added this information in the revised manuscript:

"The 1,269 putative TS alleles occurred in 267 of the 346 genes. On average, the CRISPR screen identified 3.7 TS alleles per gene, and 5 genes had more than 16 TS alleles: panM (30), panB (25), panC (17), cysN (17), and cysS (17) (Appendix Fig S5)."

Did different alleles of the same gene have similar growth kinetics (e.g. switch vs gradual growth) or did they differ?

We cloned an allelic series of 14 TS mutants of *cysS.* Their growth at 6 different temperatures shows that TS alleles of the same gene can have very different growth kinetics (Fig EV2). These differences even occurred at the same mutation site with different amino acid substitutions, like for example M96D and M96Q or V45W and V46Q. Although our pooled fitness assay covered only two temperatures and, thus, cannot recall full growth kinetics, our clustering approach revealed a large variety of fitness levels among single mutation sites and alleles of the same gene. We present this in a large, new figure that

provides an overview of all the different alleles and their fitness as well as protein structural properties for each gene individually (File EV1).

5. This work includes some interesting metabolomics analysis. In total, 94 TS alleles were reconstructed and subjected to metabolomics analysis. While most mutants had distinct metabolic profiles, 29 pairs of functionally related genes shared similar metabolic profiles. I would like to see a little more analysis of the gene set that includes these 94 TS alleles.

We thank the reviewer for the suggestions. In the revised manuscript, we performed additional analyses, in which we analyzed the similarity more systematically.

For this purpose, we removed the previous analysis based on the cut-off (PCC >0.7). Instead we inspected the PCC of **all genes** in 2 groups:

(1) all genes that are in the same enzyme complex

(2) all genes within the same metabolic pathway

Gene pairs with high similarity were clearly enriched in both groups. We include this in the revised text:

Line 287: *"However, the similarity was significantly higher between functionally related genes, which were genes in the same metabolic pathway and genes that belong to the same protein complex ($p < 0.002$, Wilcoxon rank sum test, two-tailed, Fig EV4B, Table EV9)."*

How many of the 94 TS mutants encode functionally related gene pairs (i.e. what is the background rate of functionally related genes?). What is the functional breadth of the 94 genes examined?

We included information about the functional breadth of the 94 genes in a new figure "*Appendix Fig S7*".

6. Given the background set of functionally related gene pairs, is 29 functionally related pairs that share similar metabolic profiles significantly enriched?

Functionally related genes had a significantly higher correlation. We show this in a new "Fig EV4B" (see also our answer to question 5). We removed the previous analysis with the 29 high correlating genes (based on PCC > 0.7 cut-off) and instead we inspected the PCC of all functionally related genes. We think this is a more systematic analysis and not biased by the choice of the cutoff.

7. Did the authors test if different TS alleles in the same essential gene yield similar metabolic profiles?

The 94 TS mutants in Figure 3 hit different genes. When we measured the metabolome of the CysS allelic series, we observed stronger metabolome changes (zscore >3) in 4 TS mutants (Figure R1). Three of these TS strains had strong growth defects at 38°C (Figure R1). Therefore, we assumed that the metabolome of CysS is dominated by growth rate effects, and we decided not to include this into the manuscript. We added information about growth effects into the analysis of the 94 TS mutants (see answer to the next point).

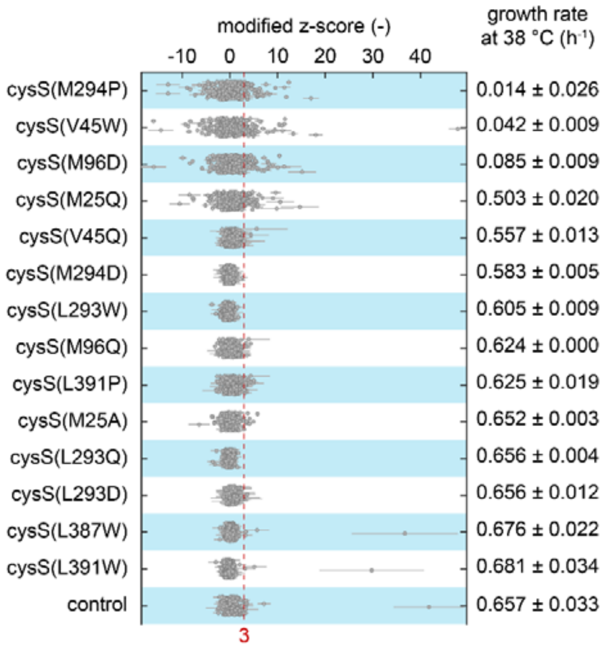

Figure R1. Metabolome of the 14 CysS mutants after 16 hours at 42°C. Shown are also growth rates at 38°C.

8. Was there a relationship between the strength and/or growth kinetics associated with metabolic profile. For example, did the 24 strains that showed an increase in direct substrate metabolites tend to have stronger TS phenotypes or particular growth-temperature relationship?

We found indeed a relationship between growth rates at 42°C and the metabolome, and included this new analysis in the revised manuscript:

Line 346: *"Moreover, a random forest model predicted non-growing strains based on their metabolome data with an accuracy of up to 70% (FigX). Together, these results suggest that the metabolome changes are influenced at two levels: i) by global growth effects and ii) by local effects in the perturbed metabolic pathways. Next, we inspected the accumulation of substrate metabolites."*

9. Was there anything particularly interesting about genes and corresponding TS mutants that affect a large number of metabolic pathways? For example, based on Fig 3D, several TS strains affected more than 30 pathways.

Strains that affect many metabolites/pathways were indeed related to the strength of growth inhibition (see also answer to question 8):

Line 342: *"Despite the gene specific response of the metabolome, we also noticed a growth-rate effect in non-growing strains (growth rate < 0.1 h-1), which had more increased metabolites than growing strains (growth rate >= 0.1 h-1, Appendix Fig S15)."*

8th Aug 2023

Manuscript Number: MSB-2023-11596R
Title: Mapping temperature-sensitive mutations at a genome-scale to engineer growth-switches in E. coli

Dear Hannes,

Thank you for sending us your revised manuscript. We have now heard back from the two reviewers who were asked to evaluate your revised study. As you will see below, they are satisfied with the performed revisions and support publication. I am therefore glad to inform you that we can soon accept your manuscript for publication pending some minor editorial issues listed below:

- Our data editors have noticed some unclear or missing information in the figure legends, please see the attached .doc file. Please make all requested text changes using the attached file and *keeping the "track changes" mode* so that we can easily access the edits made.

- All EV Tables (Table EV1-EV10) should be renamed as EV Datasets. Please make sure that the callouts in the text are updated accordingly.

- Please provide a "standfirst text" summarizing the study in one or two sentences (approximately 250 characters), three to four "bullet points" highlighting the main findings and a "synopsis image" (exactly 550px width and max 400px height, jpeg format) to highlight the paper on our homepage.

- File EV1 should be renamed as Dataset EV11. Please include a description of the Dataset in the first page of the PDF.

- The source data need to be reorganized: please provide one file per main figure (e.g. SD for Fig. 1B and 1D should be combined into a single Excel file named Source Data for Figure 1).

Please resubmit your revised manuscript online **within one month** and ideally as soon as possible. If we do not receive the revised manuscript within this time period, the file might be closed and any subsequent resubmission would be treated as a new manuscript. Please use the Manuscript Number (above) in all correspondence.

Click on the link below to submit your revised paper.

https://msb.msubmit.net/cgi-bin/main.plex

Thank you for submitting this paper to Molecular Systems Biology.

Kind regards,

Maria

Maria Polychronidou, PhD
Senior Editor
Molecular Systems Biology

If you do choose to resubmit, please click on the link below to submit the revision online before 7th Sep 2023.

https://msb.msubmit.net/cgi-bin/main.plex

IMPORTANT: Please note that corresponding authors are required to supply an ORCID ID for their name upon submission of a revised manuscript (EMBO Press signed a joint statement to encourage ORCID adoption).
(https://www.embopress.org/page/journal/17444292/authorguide#editorialprocess)
Currently, our records indicate that the ORCID for your account is 0000-0002-6677-555X.

Please click the link below to modify this ORCID:
Link Not Available

The system will prompt you to fill in your funding and payment information. This will allow Wiley to send you a quote for the article processing charge (APC) in case of acceptance. This quote takes into account any reduction or fee waivers that you may

be eligible for. Authors do not need to pay any fees before their manuscript is accepted and transferred to the publisher.

As a matter of course, please make sure that you have correctly followed the instructions for authors as given on the submission website.

*** PLEASE NOTE *** As part of the EMBO Press transparent editorial process initiative (see our Editorial at https://dx.doi.org/10.1038/msb.2010.72 , Molecular Systems Biology will publish online a Review Process File to accompany accepted manuscripts. When preparing your letter of response, please be aware that in the event of acceptance, your cover letter/point-by-point document will be included as part of this File, which will be available to the scientific community. More information about this initiative is available in our Instructions to Authors. If you have any questions about this initiative, please contact the editorial office (msb@embo.org).

Reviewer #1:

The authors satisfactorily responded to all of my queries and suggestions. I think the manuscript is much improved and amounts to a substantive and innovative contribution to the field. I have no further suggestions.

Reviewer #3:

The authors have addressed my concerns very well. The resulting manuscript makes a valuable contribution to a number of different fields, including genetics and metabolomics.

All editorial and formatting issues were resolved by the authors.

14th Aug 2023

Manuscript number: MSB-2023-11596RR
Title: Mapping temperature-sensitive mutations at a genome-scale to engineer growth-switches in E. coli

Dear Hannes,

Thank you again for sending us your revised manuscript. We are now satisfied with the modifications made and I am pleased to inform you that your paper has been accepted for publication.

*** PLEASE NOTE *** As part of the EMBO Publications transparent editorial process initiative (see our Editorial at https://dx.doi.org/10.103/msb.2010.72), Molecular Systems Biology publishes online a Review Process File with each accepted manuscripts. This file will be published in conjunction with your paper and will include the anonymous referee reports, your point- by-point response and all pertinent correspondence relating to the manuscript. If you do NOT want this File to be published, please inform the editorial office at msb@embo.org within 14 days upon receipt of the present letter.

LICENSE AND PAYMENT:
All articles published in Molecular Systems Biology are fully open access: immediately and freely available to read, download and share.

Molecular Systems Biology charges an article processing charge (APC) to cover the publication costs. You, as the corresponding author for this manuscript, should have already received a quote with the article processing fee separately. Please let us know in case this quote has not been received.

Once your article is at Wiley for editorial production, you will receive an email from Wiley's Author Services system, which will ask you to log in and will present you with the publication license form for completion. Within the same system the publication fee can be paid by credit card, an invoice or pro forma can be requested.

Payment of the publication charge and the signed Open Access Agreement form must be received before the article can be published online.

Upon acceptance it is mandatory for you to return the completed payment form. Failure to send back the form may result in a delay in the publication of your paper.

Molecular Systems Biology articles are published under the Creative Commons licence CC BY, which facilitates the sharing of scientific information by reducing legal barriers, while mandating attribution of the source in accordance to standard scholarly practice.

Proofs will be forwarded to you within the next 2-3 weeks.

Thank you very much for submitting your work to Molecular Systems Biology.

Kind regards,

Maria

Maria Polychronidou, PhD
Senior Editor
Molecular Systems Biology

EMBO Press Author Checklist

Corresponding Author Name: Hannes Link	
Journal Submitted to: MSB	
Manuscript Number: MSB-2023-11596	

USEFUL LINKS FOR COMPLETING THIS FORM
The EMBO Journal - Author Guidelines
EMBO Reports - Author Guidelines
Molecular Systems Biology - Author Guidelines
EMBO Molecular Medicine - Author Guidelines

Reporting Checklist for Life Science Articles (updated January
This checklist is adapted from Materials Design Analysis Reporting (MDAR) Checklist for Authors. MDAR establishes a minimum set of requirements in transparent reporting in the life sciences (see Statement of Task: 10.31222/osf.io/9sm4x). Please follow the journal's guidelines in preparing your
Please note that a copy of this checklist will be published alongside your article.

Abridged guidelines for figures
1. Data
The data shown in figures should satisfy the following conditions:
- the data were obtained and processed according to the field's best practice and are presented to reflect the results of the experiments in an accurate and unbiased manner.
- ideally, figure panels should include only measurements that are directly comparable to each other and obtained with the same assay.
- plots include clearly labeled error bars for independent experiments and sample sizes. Unless justified, error bars should not be shown for technical
- if n<5, the individual data points from each experiment should be plotted. Any statistical test employed should be justified.
- Source Data should be included to report the data underlying figures according to the guidelines set out in the authorship guidelines on Data

2. Captions
Each figure caption should contain the following information, for each panel where they are relevant:
- a specification of the experimental system investigated (eg cell line, species name).
- the assay(s) and method(s) used to carry out the reported observations and measurements.
- an explicit mention of the biological and chemical entity(ies) that are being measured.
- an explicit mention of the biological and chemical entity(ies) that are altered/varied/perturbed in a controlled manner.
- the exact sample size (n) for each experimental group/condition, given as a number, not a range;
- a description of the sample collection allowing the reader to understand whether the samples represent technical or biological replicates (including how many animals, litters, cultures, etc.).
- a statement of how many times the experiment shown was independently replicated in the laboratory.
- definitions of statistical methods and measures:
  - common tests, such as t-test (please specify whether paired vs. unpaired), simple χ2 tests, Wilcoxon and Mann-Whitney tests, can be unambiguously identified by name only, but more complex techniques should be described in the methods section;
  - are tests one-sided or two-sided?
  - are there adjustments for multiple comparisons?
  - exact statistical test results, e.g., P values = x but not P values < x;
  - definition of 'center values' as median or average;
  - definition of error bars as s.d. or s.e.m.

Please complete ALL of the questions below.
Select "Not Applicable" only when the requested information is not relevant for your study.

Materials

Newly Created Materials	Information included in the manuscript?	In which section is the information available? (Reagents and Tools Table, Materials and Methods, Figures, Data Availability Section)
New materials and reagents need to be available; do any restrictions apply?	Not Applicable	

Antibodies	Information included in the manuscript?	In which section is the information available? (Reagents and Tools Table, Materials and Methods, Figures, Data Availability Section)
For **antibodies** provide the following information:<br>- Commercial antibodies: RRID (if possible) or supplier name, catalogue number and or/clone number<br>- Non-commercial: RRID or citation	Not Applicable	

DNA and RNA sequences	Information included in the manuscript?	In which section is the information available? (Reagents and Tools Table, Materials and Methods, Figures, Data Availability Section)
**Short novel DNA or RNA including primers, probes**: provide the sequences.	Yes	

Cell materials	Information included in the manuscript?	In which section is the information available? (Reagents and Tools Table, Materials and Methods, Figures, Data Availability Section)
**Cell lines:** Provide species information, strain. Provide accession number in repository **OR** supplier name, catalog number, clone number, and/**OR** RRID.	Yes	Reagents and Tools Table
**Primary cultures:** Provide species, strain, sex of origin, genetic modification status.	Yes	Reagents and Tools Table
Report if the cell lines were recently **authenticated** (e.g., by STR profiling) and tested for mycoplasma contamination.	Not Applicable	

Experimental animals	Information included in the manuscript?	In which section is the information available? (Reagents and Tools Table, Materials and Methods, Figures, Data Availability Section)
**Laboratory animals or Model organisms:** Provide species, strain, sex, age, genetic modification status. Provide accession number in repository **OR** supplier name, catalog number, clone number, **OR** RRID.	Not Applicable	
**Animal observed in or captured from the field:** Provide species, sex, and age where possible**.**	Not Applicable	
Please detail **housing and husbandry conditions**.	Not Applicable	

Plants and microbes	Information included in the manuscript?	In which section is the information available? (Reagents and Tools Table, Materials and Methods, Figures, Data Availability Section)
**Plants:** provide species and strain, ecotype and cultivar where relevant, unique accession number if available, and source (including location for collected wild specimens).	Not Applicable	
**Microbes:** provide species and strain, unique accession number if available, and source.	Yes	Reagents and Tools Table

Human research participants	Information included in the manuscript?	In which section is the information available? (Reagents and Tools Table, Materials and Methods, Figures, Data Availability Section)
If collected and within the bounds of privacy constraints report on age, sex and gender or ethnicity for all study participants.	Not Applicable	

Core facilities	Information included in the manuscript?	In which section is the information available? (Reagents and Tools Table, Materials and Methods, Figures, Data Availability Section)
If your work benefited from core facilities, was their service mentioned in the acknowledgments section?	Yes	Acknowledgements

Design

Study protocol	Information included in the manuscript?	In which section is the information available? (Reagents and Tools Table, Materials and Methods, Figures, Data Availability Section)
If study protocol has been **pre-registered, provide DOI in the manuscript**. For clinical trials, provide the trial registration number **OR** cite DOI.	Not Applicable	
Report the **clinical trial registration number** (at ClinicalTrials.gov or equivalent), where applicable.	Not Applicable	

Laboratory protocol	Information included in the manuscript?	In which section is the information available? (Reagents and Tools Table, Materials and Methods, Figures, Data Availability Section)
Provide DOI OR other citation details if **external detailed step-by-step protocols** are available.	Not Applicable	

Experimental study design and statistics	Information included in the manuscript?	In which section is the information available? (Reagents and Tools Table, Materials and Methods, Figures, Data Availability Section)
Include a statement about **sample size** estimate even if no statistical methods were used.	Yes	
Were any steps taken to minimize the effects of subjective bias when allocating animals/samples to treatment (e.g. **randomization procedure**)? If yes, have they been described?	Not Applicable	
Include a statement about **blinding** even if no blinding was done.	Not Applicable	
Describe **inclusion/exclusion criteria** if samples or animals were excluded from the analysis. Were the criteria pre-established?<br><br>If sample or data points were omitted from analysis, report if this was due to attrition or intentional exclusion and provide justification.	Not Applicable	
For every figure, are **statistical tests** justified as appropriate? Do the data meet the assumptions of the tests (e.g., normal distribution)? Describe any methods used to assess it. Is there an estimate of variation within each group of data? Is the variance similar between the groups that are being statistically compared?	Yes	

Sample definition and in-laboratory replication	Information included in the manuscript?	In which section is the information available? (Reagents and Tools Table, Materials and Methods, Figures, Data Availability Section)
In the figure legends: state number of times the experiment was **replicated** in laboratory.	Yes	
In the figure legends: define whether data describe **technical or biological replicates**.	Yes	

Ethics

Ethics	Information included in the manuscript?	In which section is the information available? (Reagents and Tools Table, Materials and Methods, Figures, Data Availability Section)
Studies involving **human participants**: State details of **authority granting ethics approval** (IRB or equivalent committee(s), provide reference number for approval.	Not Applicable	
Studies involving **human participants**: Include a statement confirming that **informed consent** was obtained from all subjects and that the experiments conformed to the principles set out in the WMA Declaration of Helsinki and the Department of Health and Human Services Belmont Report.	Not Applicable	
Studies involving **human participants:** For publication of **patient photos**, include a statement confirming that consent to publish was obtained.	Not Applicable	
Studies involving experimental **animals**: State details of **authority granting ethics approval** (IRB or equivalent committee(s), provide reference number for approval. Include a statement of compliance with ethical regulations.	Not Applicable	
Studies involving **specimen and field samples:** State if relevant **permits** obtained, provide details of authority approving study; if none were required, explain why.	Not Applicable	

Dual Use Research of Concern (DURC)	Information included in the manuscript?	In which section is the information available? (Reagents and Tools Table, Materials and Methods, Figures, Data Availability Section)
Could your study fall under dual use research restrictions? Please check biosecurity documents and list of **select agents and toxins** (CDC): https://www.selectagents.gov/sat/list.htm	Not Applicable	
If you used a select agent, is the security level of the lab appropriate and reported in the manuscript?	Not Applicable	
If a study is subject to dual use research of concern regulations, is the name of the **authority granting approval and reference number** for the regulatory approval provided in the manuscript?	Not Applicable	

Reporting

The MDAR framework recommends adoption of discipline-specific guidelines, established and endorsed through community initiatives. Journals have their own policy about requiring specific guidelines and recommendations to complement MDAR.

Adherence to community standards	Information included in the manuscript?	In which section is the information available? (Reagents and Tools Table, Materials and Methods, Figures, Data Availability Section)
State if relevant guidelines or checklists (e.g., **ICMJE, MIBBI, ARRIVE, PRISMA**) have been followed or provided.	Not Applicable	
For **tumor marker prognostic studies,** we recommend that you follow the **REMARK** reporting guidelines (see link list at top right). See author guidelines, under 'Reporting Guidelines'. Please confirm you have followed these guidelines.	Not Applicable	
For **phase II and III randomized controlled trials**, please refer to the **CONSORT** flow diagram (see link list at top right) and submit the CONSORT checklist (see link list at top right) with your submission. See author guidelines, under 'Reporting Guidelines'. Please confirm you have submitted this list.	Not Applicable	

Data Availability

Data availability	Information included in the manuscript?	In which section is the information available? (Reagents and Tools Table, Materials and Methods, Figures, Data Availability Section)
Have **primary datasets** been deposited according to the journal's guidelines (see 'Data Deposition' section) and the respective accession numbers provided in the Data Availability Section?	Yes	Data Availabity Statement
Were **human clinical and genomic datasets** deposited in a public access-controlled repository in accordance to ethical obligations to the patients and to the applicable consent agreement?	Not Applicable	
Are **computational models** that are central and integral to a study available without restrictions in a machine-readable form? Were the relevant accession numbers or links provided?	Not Applicable	
If publicly available data were reused, provide the respective **data citations in the reference list**.	Not Applicable	
